# Comparison of soil sampling and analytical methods for asbestos at the Sumas Mountain Asbestos Site—Working towards a toolbox for better assessment

**DOI:** 10.1371/journal.pone.0180210

**Published:** 2017-07-31

**Authors:** Julie Wroble, Timothy Frederick, Alicia Frame, Daniel Vallero

**Affiliations:** 1 Office of Environmental Review and Assessment (OERA), United States Environmental Protection Agency Region 10, Seattle, Washington, United States of America; 2 Scientific Support Section, Superfund Division, United States Environmental Protection Agency Region 4, Atlanta, Georgia, United States of America; 3 Assessment and Remedy Division, Office of Superfund Remediation and Technology Innovation, Office of Land and Emergency Management (OLEM), Arlington, Virginia, United States of America; 4 Research Physical Scientist, National Exposure Research Laboratory (NERL), Research Triangle Park, North Carolina, United States of America; The Education University of Hong Kong, HONG KONG

## Abstract

Established soil sampling methods for asbestos are inadequate to support risk assessment and risk-based decision making at Superfund sites due to difficulties in detecting asbestos at low concentrations and difficulty in extrapolating soil concentrations to air concentrations. Environmental Protection Agency (EPA)’s Office of Land and Emergency Management (OLEM) currently recommends the rigorous process of Activity Based Sampling (ABS) to characterize site exposures. The purpose of this study was to compare three soil analytical methods and two soil sampling methods to determine whether one method, or combination of methods, would yield more reliable soil asbestos data than other methods. Samples were collected using both traditional discrete (“grab”) samples and incremental sampling methodology (ISM). Analyses were conducted using polarized light microscopy (PLM), transmission electron microscopy (TEM) methods or a combination of these two methods. Data show that the fluidized bed asbestos segregator (FBAS) followed by TEM analysis could detect asbestos at locations that were not detected using other analytical methods; however, this method exhibited high relative standard deviations, indicating the results may be more variable than other soil asbestos methods. The comparison of samples collected using ISM versus discrete techniques for asbestos resulted in no clear conclusions regarding preferred sampling method. However, analytical results for metals clearly showed that measured concentrations in ISM samples were less variable than discrete samples.

## Background

As part of the Superfund program, the Environmental Protection Agency (EPA) continues to clean up legacy sites contaminated with asbestos. Asbestos is consistently listed as a top 10 contaminant of concern for time-critical removals at Superfund removal sites (Information from the Superfund Enterprise Management System (SEMS) as of May 2, 2016). Many sites have asbestos contamination from building materials that were not removed or disposed in accordance with EPA regulations. Other sites, such as the Sumas Mountain Asbestos site, have naturally-occurring asbestos that has been disturbed by human activity and/or natural processes.

EPA has been working to identify better methods for investigating soil at asbestos-contaminated sites since the 1990s. Office of Land and Emergency Management’s (OLEM’s) Technical Review Workgroup (TRW) for Asbestos, which formed in 2005, is a formal working group tasked with improving consistency in investigation and remediation at asbestos-contaminated sites. The TRW developed the Framework for Investigating Asbestos-Contaminated Superfund Sites (Framework), “for investigating and characterizing the potential for human exposure from asbestos contamination in outdoor soil and indoor dust at Superfund removal and remedial sites [1].” Currently, the Framework recommends that soil sampling be performed to determine if sites are so contaminated they should progress directly to cleanup. Soil sampling is not recommended to determine that sites need no further characterization because most soil methods lack adequate sensitivity to identify low levels of asbestos or the amount of material sampled is so small that it may not represent a large area of the site. Activity-based sampling (ABS) is used to determine if asbestos released from soil to air poses a risk to human receptors, but this technique is time- and labor-intensive.

In recent years, EPA has attempted to advance the state-of-the-science related to asbestos and other elongated mineral particles, especially as it relates to the process and mechanisms to determine mobility of asbestos in sediment and soil, and its release to air. A key finding from this and other recent research is that in certain situations, the exposures resulting from releases of asbestos contaminated sediments and soils can lead to elevated human health risks [2, 3]. Because of the risks to human health, it is important to be able to accurately detect and quantify the presence of asbestos in soils and sediments.

Generating reproducible results for detection and quantitation of asbestos in soil is difficult. Analytical methods for detecting asbestos in soil generally have relatively low precision at levels of potential health concern. Although a variety of analytical methods are available, many are focused on asbestos containing materials or bulk aggregate, which may not apply to individual asbestos fibers in soil. An important analytical complication is the soil matrix itself. Unlike detecting fibers in fluids (i.e., air and water), asbestos in soil is comprised of a solid-within-a-solid matrix. The solid asbestos particles do not disperse well in the solid matrix, and this matrix heterogeneity hinders identification [4]. The analytical community has also found variability among labs and between analysts within labs such that results often are not reproducible [5]. Finally, EPA’s experience at asbestos-contaminated sites has demonstrated that it is difficult to extrapolate between asbestos measurements in soil and concentrations of asbestos released to air. Yet, assessments of potential exposure and human health are based upon the inhalation pathway [6].

The Sumas Mountain Asbestos site was selected as the study location for this project because there was extensive information about asbestos concentrations at this site and it was near EPA’s Region 10 facilities. The Sumas Mountain site is impacted by naturally occurring asbestos. Landslides originating on Sumas Mountain annually deposit asbestos-containing rubble into the Swift Creek and Sumas River [7]. Flooding and human-mediated movement of sediments can result in human exposures when asbestos contaminated sediments are distributed to upland areas and dry out, resulting in release to the air.

An advantage of conducting this study at the Sumas Mountain Asbestos Site is its naturally occurring chrysotile asbestos, studied by EPA and others, and the potential applicability of this investigation to other sites with naturally occurring asbestos. Most of the asbestos detected at this site is chrysotile; however, a small percentage of actinolite fibers are found in some samples. EPA has been investigating this site since 2006 and has a growing understanding of the mechanisms that distribute asbestos from the sediments around this community. Because the EPA Framework has been applied at the Sumas Mountain site, researchers can investigate the sampling methods evaluated in this effort, especially how improved methods may be incorporated into updates of the Framework [1].

The lack of an analytical methodology with adequate accuracy at low sensitivities to characterize asbestos in soils for site characterization and establishment of clean-up levels is a fundamental technological gap that affects site investigations. As such there are several ongoing research efforts to improve and more reliably quantify asbestos levels in soil [8].

Over the past 10 years of EPA’s involvement at the site, EPA has pursued more cost-effective methods of determining where Sumas Mountain slide materials are located. Schreier [9] noted that certain metals were co-located with asbestos-containing sediments and used metals as an indicator of these sediments. Specifically, chromium, cobalt, manganese and nickel were associated with asbestos [9, 10]. EPA has collected metals in many sampling events, including the current study, and aims to show statistically which metals could best be used to indicate the presence of asbestos or materials originating from the slide area.

### Analytical techniques for quantifying asbestos in soil

Polarized light microscopy (PLM) has traditionally been used to identify asbestos in soil. EPA Method EPA/600/R-93/116 [11] is a PLM method developed for the identification of asbestos in building materials, which typically contain relatively high concentrations of asbestos in a homogenous matrix. Soil, by contrast, typically contains relatively low levels of asbestos in a highly heterogeneous matrix. Therefore, the EPA PLM method as typically used, without employing special preparation steps such as gravimetric reduction and milling, is typically inadequate to produce reliable data for asbestos in soil, and was not used for this research project.

The California Air Resources Board (CARB) Method 435 is a PLM method that prescribes milling soil to reduce heterogeneity [12]. Milling reduces particle size so that a single aliquot of sample of similar particle size can be placed on a slide; however, even milled samples can be heterogeneous due to different particle densities. Turbula mixers were used to homogenize just prior to sample preparation and quantitation. Milled samples are analyzed by PLM using a point-counting method to generate an estimate of percent (i.e., the percentage of points occupied by asbestos divided by the total points counted).

The American Society of Testing and Materials (ASTM) D7521-13 [8], Standard Test Method for Determination of Asbestos in Soil, is a relatively new method for assessment of asbestos in soil that requires sieving soil samples into prescribed size fractions so that each fraction can be analyzed independently. Both PLM and TEM (finest fraction only) are used to quantify the amount of asbestos in soil.

The CARB and ASTM methods both quantify the amount of asbestos in each soil sample. It is beyond the scope of either method to provide data that can be used directly to estimate exposure via the inhalation pathway of interest. Asbestos concentration data obtained via these methods is useful for determining the presence of asbestos in soil that may contribute to potential health risks.

The fluidized bed asbestos segregator (FBAS) is a benchtop instrument used for determining the concentration of mineral fibers that can become airborne if the soil is disturbed. The FBAS utilizes air elutriation to separate fibrous mineral structures from heavier matrix particles and deposits them onto a filter that is analyzed by transmission electron microscopy (TEM) or other appropriate microscopic techniques. Research conducted by EPA has demonstrated there is an approximate linear relationship between the concentration of asbestos in performance evaluation standards (as mass percent) and the mean concentration estimated by TEM analysis following preparation by FBAS, expressed as asbestos structures captured on a filter per gram of test material [13]. The FBAS method detection limits range from 0.002% to 0.005% by weight.

### Sample collection for asbestos in soil

How samples are physically collected in the field can also impact the reliability and reproducibility of sample results. Discrete (grab) sampling methods are commonly used to investigate asbestos-contaminated soils, but may not adequately control for soil heterogeneity [7, 14, 15]. Incremental Sampling Methodology (ISM) techniques are designed to collect samples that control sampling error. ISM techniques have not been widely used for investigation of asbestos-contaminated sites (S1 Text. Borrow Evaluation for DSL Lands), even though the working draft of the updated CARB 435 method encourage the use of incremental sampling as one means of obtaining a more representative soil sample [16].

Superfund site investigations use an estimate of the mean concentration of contaminants of interest to represent the exposure point concentration used in human health risk assessment calculations [6]. Since ISM techniques result in an unbiased estimate of the mean over a given area, these sampling methods are well-suited for use in risk assessments. A primary goal of this study is to investigate the efficacy of ISM for asbestos-contaminated sites. Ultimately, if the ISM soil sampling results better capture and represent the mean soil fiber concentration over an area, then ISM techniques could lead to more reliable benchmarks for comparison to fiber concentrations in the air.

The Framework acknowledges the limitations of existing analytical methods for asbestos in soil and recommends the use of ABS for evaluating potential exposure to asbestos-contaminated soil. ABS requires that workers perform a soil-disturbing activity and the air in their breathing zone is monitored for asbestos. This is a labor intensive and time-consuming method, but the resulting air concentration data, in units of fibers per cubic centimeter of air (f/cc) can be directly input into exposure assessments and used to evaluate the potential human health risks at asbestos-contaminated sites.

A possible complimentary method to ABS under development by EPA and others is the FBAS; i.e., a soil preparation method used to separate releasable/respirable asbestos particles from soil samples into the air within a designed sampling chamber, whereupon fibers can be captured on a membrane filter which is analyzed using an appropriate microscopy technique. FBAS allows for faster sample throughput with less chance for cross contamination between samples than comparable methods since all components that contact the sample other than the glass chamber itself, are one-time use, disposable parts. The reusable glass chambers are more easily decontaminated by wet washing [13]. The resulting data are reported in units of releasable/respirable fibers/gram of soil (f/g). Additional work is needed to understand how these data can be translated into units that can be used to assess potential human exposure, which is typically expressed as fibers (or structures) per cubic centimeter of air.

## Research objectives

This study was designed to meet the following research objectives:

1. Evaluate and compare three analytical methods for detecting asbestos using soils collected from a site where asbestos is the primary contaminant of concern. The analytical methods to be compared include: a PLM method (i.e., CARB 435) [12, 16], a method combining PLM and TEM for the finest fraction (i.e., ASTM D7521) [8], and FBAS followed by TEM [14, 17].
  If possible, establish a hierarchy of analytical methods for reliably characterizing asbestos levels in site soils. The criteria for method comparison includes: sensitivity (ability of the method to identify low concentrations of asbestos) and reproducibility/representativeness (as determined by evaluating variability between samples from the same area, as measured by the relative standard deviation,).
2. Evaluate and compare variability in measured asbestos concentrations in samples collected by ISM and discrete sampling techniques (as determined by evaluating variability between samples). Samples were also submitted for metals analysis (EPA Method 200.2/200.7) to measure how well sampling and analytical error for metals may have been controlled by the different sampling and analytical methods. While metals and asbestos concentrations are not directly comparable, the comparison of metals and asbestos variability in this study may provide insight into potential sources of sampling and/or analytical error.
3. Determine whether metals concentrations or ratios between certain inorganic compounds can be used as an indicator of the types of soils that may contain asbestos [9, 10, 18].
4. Compare soil concentrations to air measurements collected via ABS.

## Methods

### Sample collection

Samples were collected at a property adjacent to Swift Creek, owned by Whatcom County that also included a public right-of-way. Permission to sample was given to EPA by Frank Abart, Director of Whatcom County Public Works, and Roland Storme with Washington State Department of Transportation, Mount Baker Area. Asbestos contaminated sediments dredged from Swift Creek have been placed on the banks of Swift Creek on this parcel and on other parcels in the area. This location is being used by the county as a base for maintenance dredging operations. Sediments dredged from under bridges that cross Swift Creek also are stockpiled here. The home and sheds formerly present on the property were demolished, but the large barn remains and served as the command post for the field event.

**Fig 1:**
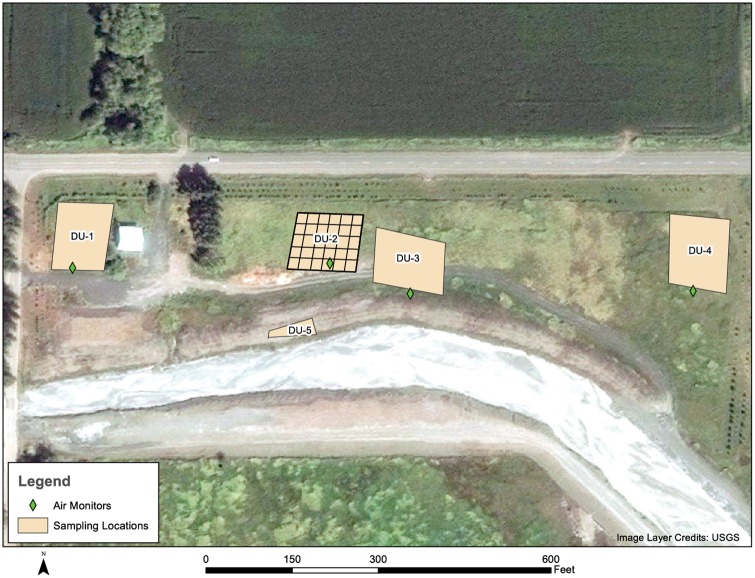
Five site decision units and how the grids were established for one decision unit.

Applying a judgmental sampling approach, potential decision units (DUs) were identified during a field reconnaissance visit on September 4, 2014. The Interstate Technology and Regulatory Council (ITRC) defines a DU as “the smallest volume of soil for which a decision will be made [14].” DUs were selected to represent a range of expected soil levels. Some areas appeared to have been relatively unaffected by flood events or dredging activities. Other areas were selected on the expectation of having higher concentrations of asbestos. The range of expected asbestos concentrations was informed by previous environmental sampling results, the location of past sediment deposition from flood events, human-mediated movement of contaminated material, windblown dust, and irrigation. Specifically, five DUs on a single property (Fig 1) were selected because they were expected to vary in concentrations of asbestos based on their proximity to Swift Creek, historical flooding, and placement of dredged materials.

Table 1 provides the latitude and longitude for the four corners of each decision unit.

**Table 1:** Latitude and longitude of decision units.

Decision Unit Corners	Latitude	Longitude
DU-1	48.92006999960	-122.30361000000
DU-1	48.91974900010	-122.30367200000
DU-1	48.91975000020	-122.30405400000
DU-1	48.92007199970	-122.30401500000
DU-2	48.92003725710	-122.30180941000
DU-2	48.91976600000	-122.30185400000
DU-2	48.91977599960	-122.30234600000
DU-2	48.92004100010	-122.30228200000
DU-3	48.91991299960	-122.30120900000
DU-3	48.91966299970	-122.30121800000
DU-3	48.91971899960	-122.30172800000
DU-3	48.91998200010	-122.30171100000
DU-4	48.92004999960	-122.29915400000
DU-4	48.91969700040	-122.29917000000
DU-4	48.91974199980	-122.29958900000
DU-4	48.92007199970	-122.29959000000
DU-5	48.91947790760	-122.30247120900
DU-5	48.91954273160	-122.30216264700
DU-5	48.91946482150	-122.30212736400
DU-5	48.91944740620	-122.30247609900

Field work occurred during the week of September 29, 2014. A field crew of nine EPA staff mobilized to the site. At four of the five DU locations, an area of approximately one quarter acre (120 feet by 100 feet) was measured with a field tape and staked out with wooden survey stakes. The field sampling team used rope to set up a 20-foot by 20-foot grid for a total of 30 grid cells within each decision unit. In the fifth DU, which was located on a berm of dredged material intended to represent relatively higher asbestos concentrations, a sample grid of about 100 feet by about 25 feet was created with each grid cell measuring approximately 6 feet by 12 feet. The smaller footprint for this DU was necessary to contain the sampling area on the flat top of the bermed material. Four of five of the sampling locations (DUs 1–4) had significant vegetative cover including very tall grass in some areas. Conducting the incremental sample collection efficiently required that this vegetation be cleared using a string trimmer.

Soil sampling was conducted in Level C personal protective equipment, including Tyvek coveralls, full face respirators with HEPA (P-100) filter cartridges, gloves, and protective footwear.

### Incremental sampling methodology

**Fig 2:**
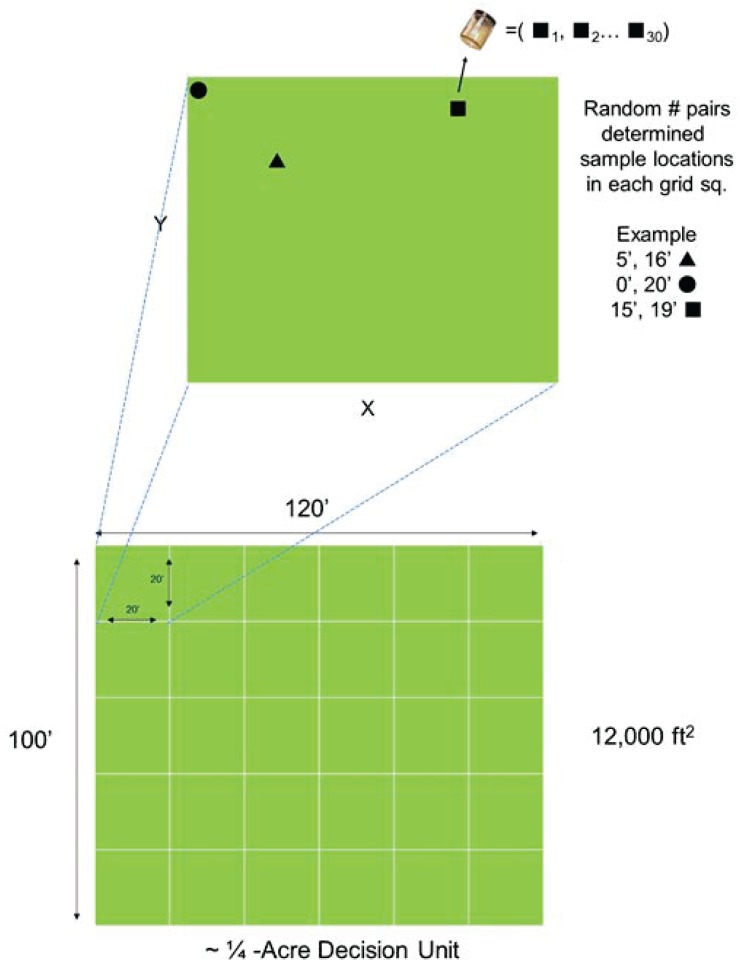
This figure shows how increments from each grid are combined to create each incremental sample. Note that three separate incremental samples consisting of increments from each of 30 locations are used to represent each decision unit.

Incremental samples were collected in accordance with the ITRC guidance document *Incremental Sampling Methodology ISM-1* [14]. For each incremental sample, a set of random coordinates determined the increment location in the first grid cell of each DU. Subsequent increments of that sample were collected from the same coordinates in each of 29 remaining grid cells of that DU. This process of random placement (within the first grid cell) for the first increment of an individual incremental sample was repeated three times per DU to produce three independent replicate incremental samples from the same DU. Fig 1 illustrates how ISM samples were collected in each grid. Fig 2 shows how increments from each grid were collected across all grids to generate an incremental sample.

**Fig 3:**
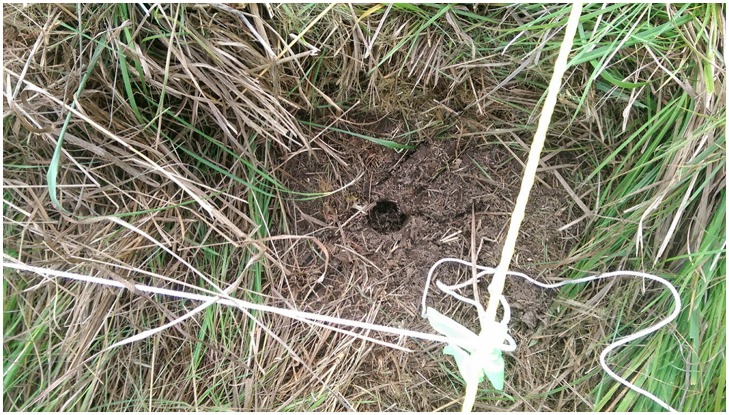
View of typical cleared sample location, following use of specialized ISM sampling tool.

After the locations within the cells had been determined, the field team then collected increments from the same coordinates within each grid cell of the respective decision unit to yield one incremental sample. Three separate incremental samples (called replicates in the ITRC guidance document) were collected in each grid to yield three, thirty-increment samples for each decision unit. At each increment location within each grid, a field team member pushed the sampling tool (EVC Incremental Sampler, Field Environmental Instruments, Inc., Woodinville, WA) into the ground, stepped on the tool to push it down, rocked the tool to loosen the soil, then pulled up the sample and ejected the collected core into a plastic bag. The sampling tool collected a cylindrical soil boring 6.35 centimeters in length and 4.13 centimeters in diameter (a volume of 85 cubic centimeters per increment location). Thirty incremental soil samples yielded a total volume of approximately 2.6 liters of soil per incremental sample. Fig 3 shows a typical increment collection location.

**Fig 4:**
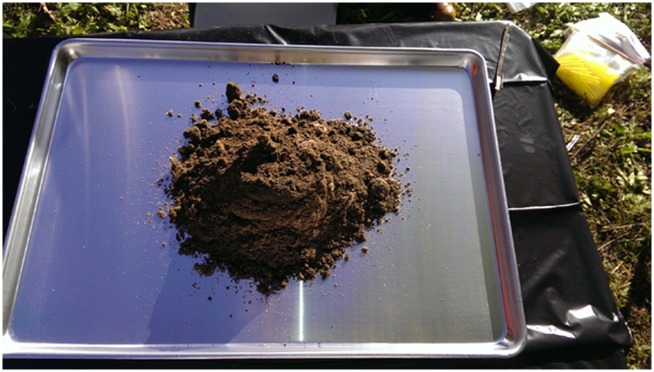
ISM soil sample spread out on aluminum baking tray.

ISM-collected samples required further processing and subsampling to meet the study objectives. ISM samples were transported by the field personnel to the designated subsampling area for processing. First, the bag containing the ISM-collected sample was opened and the material spread out on a clean stainless steel baking tray (Fig 4). Next, clumps and clods of soil were manually disaggregated and vegetation was removed by hand to the extent possible. The material was then spread evenly over the baking tray to form a two-dimensional slabcake for subsampling in accordance with procedures described in the ITRC guidance [14].

**Fig 5:**
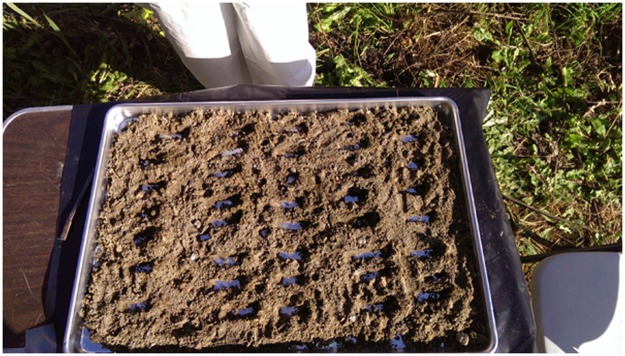
Soil was subsampled using ISM techniques in the field to obtain a representative sample of the larger area.

Because the metals analysis required that precisely 10 grams of sample be extracted, subsampling for this sample was performed first. A clean glass jar was tared using a Sartorus field balance. Next, a fine subsampling spatula was used to place approximately evenly spaced subsamples from across the soil sample spread on the baking tray into the tared glass jar. Once 10 grams of soil was collected, this container was capped, bagged, labeled, and placed in the sample collection cooler for storage and eventual transport to the laboratory. Additional systematic subsampling was performed on the remaining soil to prepare samples for the other analyses, specifically analysis for asbestos. Quantities of soil required for the other analyses were greater, but with larger tolerances of precision; however, the intent was to subsample the ISM-collected sample so that the material in each sample container was representative of material from across the baking tray (Fig 5). A variety of subsampling tools were available, and the choice of tool depended on the subsample volume needed for analysis and soil properties.

### Discrete soil sampling

Field methods used to collect, mix, and prepare discrete samples were intended to represent typical soil sampling methods used in asbestos investigations. For each of the 5 discrete (i.e., grab) sample locations (each of 4 corners and center of the five selected decision units, i.e., a quincunx pattern), the sampler cleared vegetation as needed and then used a pre-cleaned trowel to loosen and sample the soil from the surface to a depth of up to 6 inches in a one-foot diameter area. Soil was sampled until the disposable bowl was nearly full, avoiding the inclusion of vegetation to the extent possible. Once sufficient volume was collected, the samples were well mixed before being placed in the respective sample jars as required for analysis. No additional subsampling was conducted for discrete samples.

From the outset, the investigators were aware of the need to account for uncertainties. The soil preparation techniques were not consistent for all subsamples, depending on the type of soil sample collected (ISM versus discrete) and the requirements of the analytical method. These different preparation methods may have introduced differing amounts of sampling error and/or variability. Some, but not all, analytical methods required additional processing in the laboratory.

### Activity-based sampling

ABS was performed to determine asbestos levels in the breathing zone air of workers conducting the field activities. Soil preparation, soil sampling and soil subsampling activities served as the activities for ABS. This varied slightly from the project plan; however, the field team agreed that these activities would result in exposures to any dust and fibers released from soil. For each of the five sample areas described above, ABS samples were collected in accordance with EPA-recommended methods [1].

**Fig 6:**
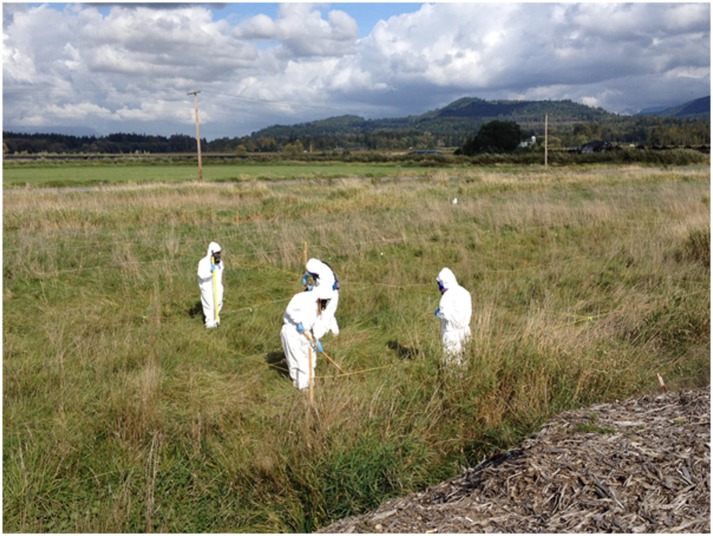
Grid clearing prior to sampling; note that one of the field crew is wearing a portable pump and air monitoring cassette.

**Fig 7:**
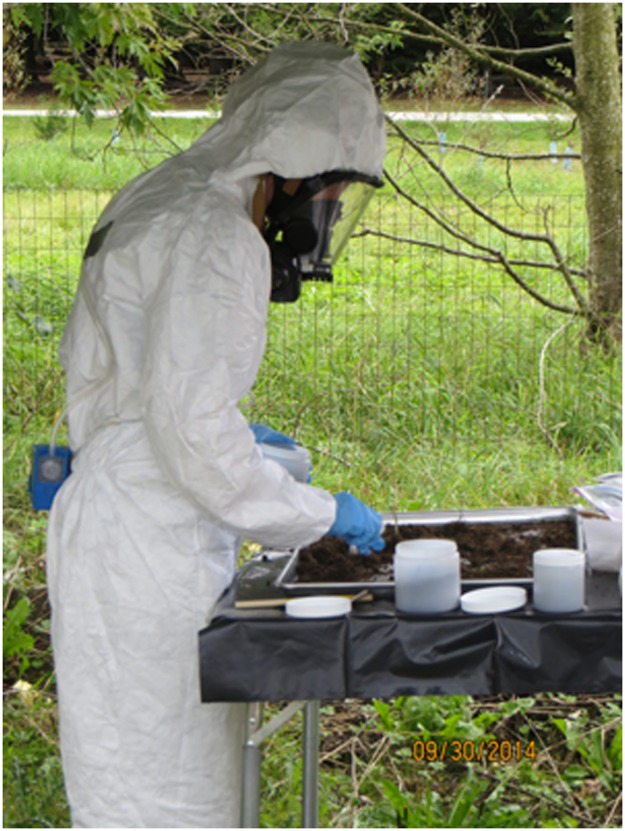
Breathing zone sample being collected during ISM subsampling.

Figs 6 and 7 show soil preparation, soil sampling, and soil subsampling activities being performed by EPA personnel. The field crew that conducted the soil sampling had the appropriate training, personal protective equipment, and occupational health clearance to wear respirators. Breathing zone air samples were collected on 25-mm mixed cellulose acetate, 10-μm pore size filter cassettes attached to sampling pumps with a flow rate of about 2.5 liters per minute (LPM). Perimeter stationary air samples were collected on 25-mm filter cassettes as described for ABS samples but with flow rates of about 4.5 LPM.

### Laboratory analysis of soil samples

Following collection and subsampling (as needed), the discrete and ISM-collected samples were transported to EPA Region 10’s Manchester Environmental Laboratory (MEL). A subset of samples was prepared for analysis by MEL using the FBAS. Filters from this preparation and soil samples were sent to a commercial asbestos laboratory for analysis by CARB 435, ASTM D7521-13, and ISO 10312 for FBAS soils only. Samples also were analyzed by MEL for metals (USEPA Method 200.2/200.7). Table 2 summarizes the analyses performed for soil, indicates the number of samples analyzed by each method, and summarizes the quality assurance analyses that were performed.

**Table 2:** Analytical method summary.

Location		DU1		DU2		DU3		DU4		DU5	
Analysis	Volume Submitted for Analysis	ISM	Discrete	ISM	Discrete	ISM	Discrete	ISM	Discrete	ISM	Discrete
CARB 435 (PLM)	16 oz. HDPE bottle	5*	6**	3	5	3	6**	3	5	3	6**
ASTM D7521 (PLM/TEM)	16 oz. HDPE bottle	5*	6**	3	5	3	6**	3	5	3	6**
FBAS/ISO 10312 (TEM)	250 ml HDPE jar	5*	6**	3	5	3	6**	3	5	4***	6**
EPA 200.2/200.7 (Metals)	4 oz. glass jar		6**		5		6**		5		6**
EPA 200.2/200.7 (Metals)	2 oz. glass jar (10 g of soil exactly)	5*		3		3		3		3	
Percent Solids	2 oz. glass jar	5*	6**	3	5	3	6**	3	5	3	6**

Key:

DU—decision unit

ISM—incremental sampling methodology

TEM—transmission electron microscopy

*Includes ISM triplicates

**Includes field duplicate

***Includes laboratory duplicate

Soil samples analyzed by CARB 435 were reported as percent asbestos (based on a 400-point count procedure) or no asbestos detected (NAD). Soil samples analyzed by ASTM D7521 were reported as visual estimated percent for each grain size fraction. If the fine fraction showed less than 1%, then a 400-point count on that fraction was performed. Any NAD for each grain size was followed with TEM analysis on the smallest size fraction, with the results reported as both structures per gram and as a weight percent. Each soil sample was also analyzed for metals using Method 200.2/200.7.

For samples analyzed by CARB 435 [12], additional sample processing proceeded in accordance with method requirements and as specified by the project statement of work (S2 Text. QAPP). Samples were transported to the laboratory by EPA and then were processed by the laboratory in spaces with controlled air flows. All drying, milling, and sieving was performed in a fume hood or within glove boxes. Next, the samples were adequately dried using convection oven or muffle furnace. Samples were then pulverized using milling to produce a material with particle size for analysis of about 250 microns in size. PLM point counting was then performed on 8 separate preparations of the milled samples.

For samples analyzed by ASTM D7521 [8], samples were first dried until weights were stable. Next, samples were dry sieved using a shaker apparatus into 3 size fractions (sieve sizes 19 millimeter (mm), 2 mm, and 106 microns), each fraction was weighed and then analyzed using stereomicroscopy with PLM to identify asbestos using visual estimation techniques. If the fine fraction was less than 1% asbestos, then a 400-point count of this fraction was performed. If no asbestos was found in the fine fraction using PLM techniques, then the fine fraction was analyzed using TEM to determine a weight percent.

Samples were prepared by FBAS [19] first collecting about 250 cc of representative soil, drying it, and sieving it through an 850-um sieve. An aliquot (0.5 to 1-gram) of this sieved material is added to a vial containing the appropriate amount of laboratory-grade Ottawa 20/30 sand such that the total weight of the combined material equaled 20 grams (S3 Text. Sumas Asbestos Methods FBAS). The combined material was placed into the bottom of a glass fluidization chamber. Air was passed through the chamber for three minutes as a flow rate of approximately 19 liters per minute resulting in the sample material in the bottom of the chamber forming a spouted fluidized bed. As the sample material circulates, smaller light particles including asbestos fibers elutriate to the top of the glass chamber and enter an isokinetic flow splitter. A portion of material split from the flow is deposited onto a MCE filter at a flow rate of approximately 240 cubic centimeters per minute. The prepared filters were submitted for analysis by TEM using ISO 10312 [17].

### Laboratory analysis of filters

Air samples (both stationary and ABS) were analyzed for asbestos fibers by method ISO 10312-Annex-E recording total and phase-contrast microscope equivalent (PCME) fibers. The resulting data were reported as structures per cubic centimeter (s/cc) of air. PCME fibers are those structures having length greater than 5 microns, width between 0.25 and 3 microns, inclusive, and a 3-to-1 aspect ratio. PCME fibers are important to capture in air samples as the toxicity value used to assess health risks associated with asbestos in EPA’s Integrated Risk Information System database is based on fibers measured using phase contrast microscopy (PCM). PCME measurements are assumed to approximate what would have been observed using PCM.

Soil samples processed using the FBAS produced filters that were sent to Lab/Cor, Inc., for analysis by ISO 10312; results were reported as structures per gram (s/g). None of the air samples or FBAS filters required analysis using indirect methods, indicating that filters were not overloaded with particulate.

### Data analysis

Laboratory results were received as National Asbestos Data Entry Spreadsheets (NADES) [20] reports for asbestos data and the EPA R10/CLP Universal EDD format for metals and collated into Excel. All project data (field collection/measurements, locational, final validated laboratory) will be archived to EPA Scribe.net for data warehousing using the R10 Scribe template [21]. Statistical analyses were performed with Microsoft Excel 2013 Data Analysis ToolPak and R [22], version 3.2.3, and the corrplot [23] and NADA [24] libraries. To determine whether significant correlations between metals and asbestos concentrations were present, we calculated the Kendall’s Tau correlation coefficient. Asbestos data from the FBAS-prepared samples was used for the comparison, because it was the only method that identified asbestos in every sample.

## Results

We found that there was no significant difference in variability in asbestos concentrations between ISM & discrete samples, although ISM resulted in significantly lower relative standard deviations (RSDs) for metals. We found that FBAS with TEM was the most sensitive analytical method, but concluded that different analytical methods cannot easily be compared. Finally, we found that metal concentrations did correlate with asbestos concentrations, which supports the use of metals as indicators on a site-specific basis.

### Asbestos concentrations in soil

The asbestos data were collected for each of five decision units utilizing two different sampling methods (Table 3), and three different analytical methods. In each decision unit, five discrete samples and three ISM samples were collected (exclusive of QA duplicate samples). For each set of samples, mean, standard deviation (SD), and RSD were calculated.

**Table 3:** Summary of variability in soil data for asbestos.

Analytical Method	Reporting Information	Sample type	Decision Units														
			DU1			DU2			DU3			DU4			DU5		
			Mean	SD	RSD	Mean	SD	RSD	Mean	SD	RSD	Mean	SD	RSD	Mean	SD	RSD
EPA FBAS/ISO 10312	Total Asbestos (s/g)	ISM	9E+05	8E+05	***0*.*87***	1E+06	8E+05	***0*.*77***	2E+06	4E+06	1.64	1E+08	7E+07	***0*.*62***	6E+07	6E+07	1.02
		DISCRETE	1E+06	1E+06	1.21	1E+06	1E+06	0.94	6E+06	5E+06	***0*.*89***	2E+08	1E+08	0.69	4E+07	3E+07	***0*.*79***
	PCME (s/g)	ISM	2E+04	3E+04	***1*.*73***	4E+04	7E+04	1.73	8E+04	1E+05	1.73	3E+06	2E+06	***0*.*54***	2E+06	2E+06	1.02
		DISCRETE	7E+04	2E+05	2.23	3E+04	5E+04	1.73	2E+05	2E+05	***0*.*93***	4E+06	3E+06	0.87	1E+06	5E+05	***0*.*45***
CARB 435	PML (%)	ISM	<0.13	NAD	NAD	<0.083	NAD	NAD	<0.25	NAD	NAD	1E+01	2E+00	***0*.*16***	1E+01	1E-02	***0*.*01***
		DISCRETE	<0.05	NAD	NAD	<0.2	NAD	NAD	1E+00	NAD	NAD	2E+00	8E-01	0.33	1E+01	5E-01	0.05
ASTM D7521	Fine (%)	ISM	NAD	NAD	NAD	NAD	NAD	NAD	NAD	NAD	NAD	3E+00	2E+00	0.46	3E+00	0E+00	***0***
		DISCRETE	NAD	NAD	NAD	NAD	NAD	NAD	NAD	NAD	NAD	5E+00	9E-01	***0*.*19***	3E+00	5E-01	0.17
	Medium (%)	ISM	NAD	NAD	NAD	NAD	NAD	NAD	NAD	NAD	NAD	4E+00	1E+00	0.25	3E+00	6E-01	***0*.*22***
		DISCRETE	NAD	NAD	NAD	NAD	NAD	NAD	NAD	NAD	NAD	5E+00	9E-01	***0*.*17***	3E+00	1E+00	0.37
	Coarse (%)	ISM	NAD	NAD	NAD	NAD	NAD	NAD	NAD	NAD	NAD	4E+00	2E+00	0.35	1E+00	6E-01	0.43
		DISCRETE	NAD	NAD	NAD	NAD	NAD	NAD	NAD	NAD	NAD	6E+00	1E+00	***0*.*22***	1E+00	5E-01	***0*.*37***
	TEM-Fine (%)	ISM	3E-04	5E-04	***1*.*73***	1E-05	1E-05	***0*.*93***	1E-02	2E-02	***1*.*63***	N/A	N/A	N/A	N/A	N/A	N/A
		DISCRETE	4E-03	9E-03	2.24	7E-04	2E-05	2.23	8E-04	1E-03	1.85	N/A	N/A	N/A	N/A	N/A	N/A

Key:

DU—decision unit

ISM—incremental sampling methodology

N/A—not analyzed

NAD—no asbestos detected

RSD—relative standard deviation

SD—standard deviation

s/g—structures per gram

Table 3 presents a summary of soil data and associated variability. RSDs were calculated to determine data set variability [14]. A lower RSD generally indicates that results may be more reproducible.

Asbestos results from the five DUs indicate that three of the DUs exhibited low concentrations of asbestos (DUs 1–3) while 2 of the DUs had elevated concentrations of asbestos (DUs 4 and 5). DU5’s highest concentrations of asbestos were expected given this DU was placed on stockpiled, dredged sediments.

CARB 435 results for asbestos in soils indicated mostly no asbestos detected or trace levels in samples from DU1, DU2, and DU3, regardless of whether samples were collected as ISM or discrete samples (Table 3). Asbestos was detected in samples from DU4 and DU5. At DU4, the mean concentration from the ISM samples was about 4–5 times higher than the corresponding mean concentration from discrete samples. Concentrations from ISM and discrete samples at DU5 were similar with the highest concentrations measured in any DU using this method. Statistical analysis of these results indicated that ISM samples showed lower RSDs than discrete samples for DUs 4 and 5; the DUs where asbestos was consistently detected. This suggests that the combination of ISM and CARB 435 may provide more reproducible results than either FBAS followed by TEM or ASTM D7521 when asbestos is detected at concentrations greater than about 1%. Direct comparisons were not possible at lower concentrations due to large number of non-detects.

ASTM D7521 results for asbestos in soils indicated no asbestos was detected in DU1, DU2 and DU3 when only PLM was used (Table 3). TEM analysis of the fine fraction from these samples indicated very low levels of asbestos (0.00001% to 0.01%). ASTM D7521 identified asbestos in all fractions from DUs 4 and 5, so TEM analysis of the fine fraction in samples from these DUs was not performed. Concentrations of asbestos found in DUs 4 and 5 were within an order of magnitude for FBAS/ISO and within about 10% for the other soil asbestos methods for both ISM and discrete samples. There is no clear pattern for which sampling method provided more reproducible results for D7521 analyzed samples. In 4 of 6 comparisons, the RSDs for discrete samples were lower than the RSDs for ISM samples.

ISO10312 results from filters prepared using the FBAS indicated asbestos was detected in nearly every sample (Table 3). Results are presented for both total asbestos fibers and PCME fibers as the latter category of fiber type is often used in exposure assessments for risk assessments at EPA. For both total and PCME asbestos, concentrations in DU1, DU2 and DU3 were 2–3 orders of magnitude smaller than concentrations in DUs 4 and 5. The RSDs for FBAS-prepared samples were larger than PLM-analyzed samples, but about the same or lower than other TEM-analyzed samples. For total asbestos, variability (as indicated by RSD) was lower for ISM samples compared to discrete samples for 3 of the 5 decision units. For PCME asbestos, variability was lower for ISM samples compared to discrete samples for 2 of the 5 decision units. Variability was lower for discrete samples for 2 of the 5 decision units and variability was the same for one decision unit. Compared to other soil analytical methods, FBAS-prepared samples showed the greatest variability; however, this method also identified asbestos in every sample where other methods did not.

### Asbestos in air

**Fig 8:**
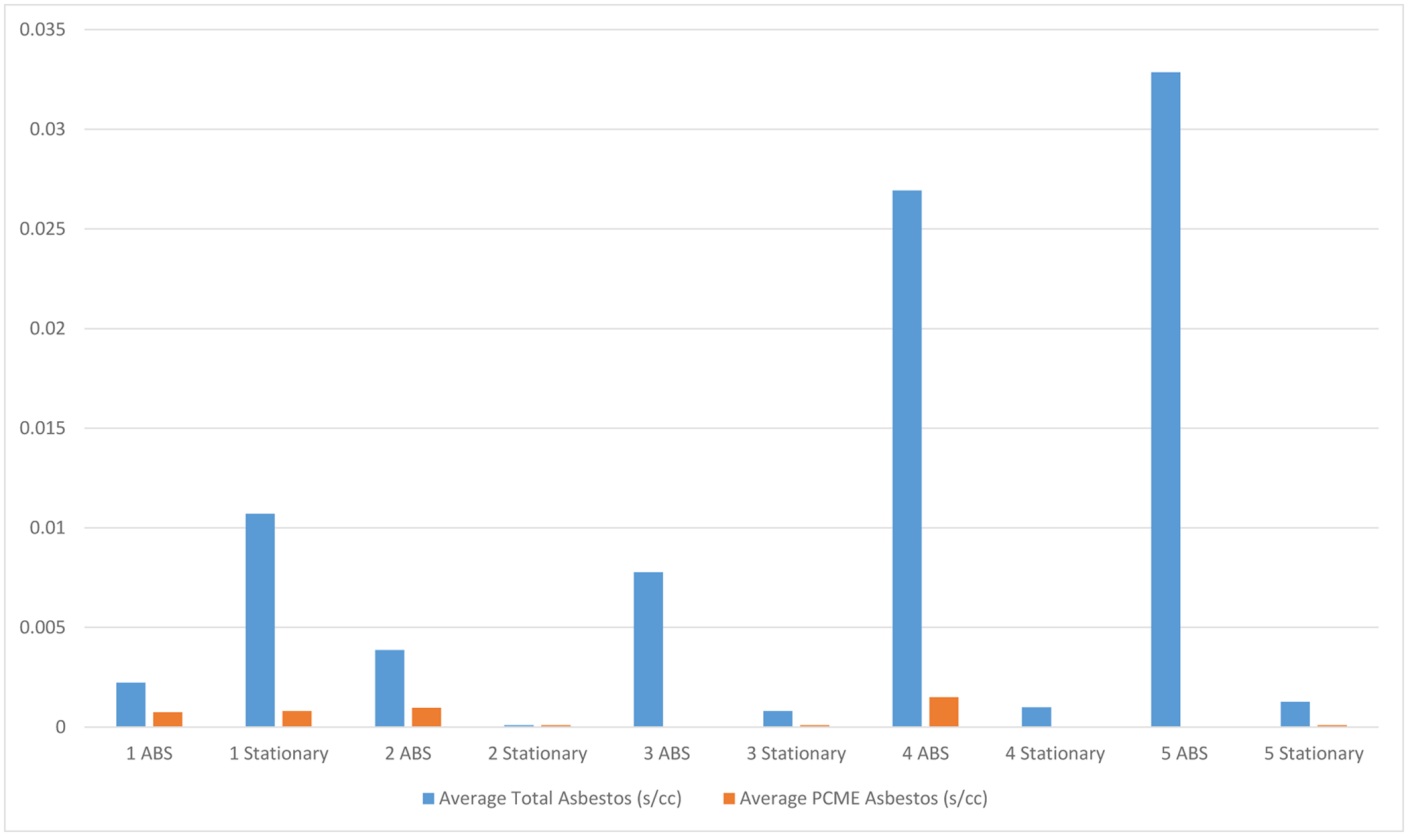
Plot of ABS and stationary air concentrations of asbestos at each decision unit.

Air samples for asbestos (both ABS and stationary) exhibited many non-detect results which may have been the result of damp and humid conditions during the week of sampling. Further, there was more vegetation on site than expected and vegetation may limit the release of fibers from the soil matrix. Relative humidity increased during the field event from about 59% on September 30 to 64% on October 1 and to 70% on October 2. Fig 8 summarizes the stationary and ABS air sampling results. As expected, based on the soil sampling results, the highest air concentrations of asbestos were found in samples collected from DUs 4 and 5, where soil concentrations of asbestos were highest. Fig 8 presents mean concentrations for both total and PCME asbestos fiber populations. PCME concentrations are always lower as PCME represents a size fraction that is a subset of the total asbestos fibers. These air samples were collected to relate soil concentrations to ABS air PCME concentrations; however, given the large number of non-detect results for PCME for ABS (75%) and the limited detections of asbestos in several DUs, the ability to draw relationships between soil asbestos concentrations and PCME ABS concentrations is limited.

### Metals in soil

Metals data were collected as part of this sampling effort to serve as a benchmark for the underlying heterogeneity of the soil matrix and to provide a relative guide to how well sample error was controlled. The potential use of the metals data as a predictor of asbestos content in the soil also could be investigated from this data set. As with the asbestos soil samples, the metals data were collected from each of the five decision units utilizing two different sampling methods (Table 4). In each decision unit, five discrete samples and three ISM samples were collected (exclusive of QA duplicate samples). For each set of samples, mean, SD, and RSD (the standard deviation for a given data set divided by the mean of that data set) were calculated for each of the 12 metals and each of asbestos analytical categories (Table 2). A lower RSD generally indicates that results are more reproducible than samples with a higher RSD.

**Table 4:** Summary of variability in soil data for metals.

Analyte	Sample type	Decision Units														
		DU1			DU2			DU3			DU4			DU5		
		Mean	SD	RSD	Mean	SD	RSD	Mean	SD	RSD	Mean	SD	RSD	Mean	SD	RSD
Aluminum	ISM	19766.7	1422.4	***0*.*07***	24000.0	360.6	***0*.*02***	23266.7	472.6	***0*.*02***	11366.7	472.6	***0*.*04***	6860.0	513.9	***0*.*07***
	DISCRETE	11572.0	4409.5	0.38	14160.0	873.5	0.06	13020.0	3505.3	0.27	10052.0	472.6	0.05	4162.0	366.9	0.09
Barium	ISM	161.7	7.2	***0*.*04***	144.7	1.2	***0*.*01***	140.3	6.7	***0*.*05***	89.8	4.0	***0*.*04***	37.7	5.5	***0*.*15***
	DISCRETE	128.1	35.3	0.27	113.6	7.9	0.07	94.8	26.8	0.28	85.2	18.1	0.21	38.0	12.3	0.32
Calcium	ISM	7433.3	251.7	***0*.*03***	7000.0	173.2	***0*.*02***	6900.0	173.2	***0*.*02***	3766.7	115.5	***0*.*03***	3433.3	665.8	0.19
	DISCRETE	4180.0	734.9	0.18	3190.0	214.8	0.07	2826.0	793.9	0.28	2614.0	564.5	0.22	1822.0	304.4	***0*.*17***
Chromium	ISM	56.0	0.9	***0*.*02***	55.4	0.7	***0*.*01***	70.9	3.8	***0*.*05***	227.0	13.8	***0*.*06***	294.3	30.6	0.1
	DISCRETE	59.1	31.9	0.54	48.1	4.2	0.09	79.7	62.9	0.79	214.4	36.0	0.17	231.2	23.6	***0*.*1***
Cobalt	ISM	20.0	1.0	***0*.*05***	19.0	0.0	***0*.*0***	24.7	0.6	***0*.*02***	81.7	5.1	***0*.*06***	95.3	0.6	***0*.*01***
	DISCRETE	23.8	14.1	0.59	17.6	1.3	0.08	29.4	22.8	0.77	80.6	11.4	0.14	87.5	3.3	0.04
Copper	ISM	26.6	0.5	***0*.*02***	23.7	0.6	***0*.*02***	24.7	0.6	***0*.*02***	19.7	1.2	***0*.*06***	8.6	0.8	***0*.*09***
	DISCRETE	25.8	6.2	0.24	23.2	1.8	0.08	21.8	3.1	0.14	24.1	1.5	0.06	10.0	0.9	0.09
Iron	ISM	31333.3	1721.4	***0*.*06***	31900.0	458.3	***0*.*01***	33733.3	251.7	***0*.*01***	45566.7	1266.2	***0*.*03***	47633.3	568.6	***0*.*01***
	DISCRETE	27520.0	7123.7	0.26	26920.0	1302.7	0.05	30760.0	4224.1	0.14	43520.0	1702.1	0.04	37180.0	2147.6	0.06
Magnesium	ISM	16833.3	2059.9	***0*.*12***	16800.0	264/58	***0*.*02***	27800.0	2193.2	***0*.*08***	144333.3	7371.1	***0*.*05***	184000.0	4000.0	***0*.*02***
	DISCRETE	25126.0	29528.5	1.2	15560.0	1331.5	0.09	41860.0	50651.8	1.2	134180.0	29138.7	0.2	157200.0	6496.2	0.04
Manganese	ISM	583.3	22.0	***0*.*04***	533.7	8.1	***0*.*02***	583.0	16.1	***0*.*03***	882.0	23.4	***0*.*03***	770.0	9.5	***0*.*01***
	DISCRETE	554.4	126.7	0.23	499.3	53.0	0.11	547.8	70.5	0.13	778.2	59.9	0.08	667.4	26.8	0.04
Nickel	ISM	163.3	15.3	***0*.*09***	140.0	0.0	***0*.*0***	243.3	23.1	***0*.*1***	1433.3	115.5	***0*.*08***	2000.0	0.0	***0*.*0***
	DISCRETE	280.0	352.5	1.3	132.0	13.0	0.1	404.2	541.7	1.3	1380.8	296.9	0.21	1762.0	72.3	0.04
Vanadium	ISM	53.3	4.8	***0*.*09***	61.9	2.0	***0*.*03***	59.7	2.0	***0*.*03***	31.3	1.5	***0*.*05***	24.7	1.2	***0*.*05***
	DISCRETE	37.7	14.8	0.39	44.4	1.5	0.03	41.6	8.8	0.21	28.8	4.6	0.16	15.2	1.3	0.09
Zinc	ISM	100.8	3.9	***0*.*04***	77.0	0.7	***0*.*01***	77.6	1.5	***0*.*02***	54.2	3.2	***0*.*06***	35.4	1.5	***0*.*04***
	DISCRETE	90.5	12.3	0.14	66.2	4.0	0.06	60.0	15.0	0.25	53.0	15.0	0.28	29.7	1.4	0.05

Concentrations are reported in units of mg/kg.

Overall, the ISM samples had lower RSDs than the discrete samples. There were 60 sample sets comprised of 12 metals across 5 DUs. Only two out of the sixty sample sets (3.3%) collected had a lower RSD for the discrete sample than the ISM samples (Table 4). Some discrete samples (magnesium and nickel at DU1 and DU4) had RSDs greater than 100%. Even after recalculation of mean, SD, and RSD of these four occurrences, the ISM samples still had lower RSD than the discrete samples, which indicates less sampling variability and the likelihood of improved reproducibility compared to existing methods.

Paired t-tests were performed on a DU-by-DU basis to determine whether there was a significant difference between discrete and incremental samples with respect to how much variability occurred in the metals data. The degree of variability was measured using the RSD. A significant difference is found if the p-value of the test is less than 0.05. The p-values for these tests for metals are summarized on a DU-by-DU basis (S1 Table). In general, data that are more variable (e.g., discrete samples) are less reliable for supporting cleanup decisions than less variable data.

We also used the metals results to test whether the concentration of metals predicted asbestos concentrations. Using R 3.2.3, and the corrplot [23], and nada [24] libraries, we calculated the Kendall’s tau correlation coefficient between the concentration of each metal and the FBAS total asbestos concentration for each sample collected, separately for ISM samples and discrete samples. Asbestos data from the FBAS-prepared samples was used for the comparison, because it was the only method that identified asbestos in every sample. The other soil asbestos methods had too many non-detects to make a useful comparison to metals data.

**Fig 9:**
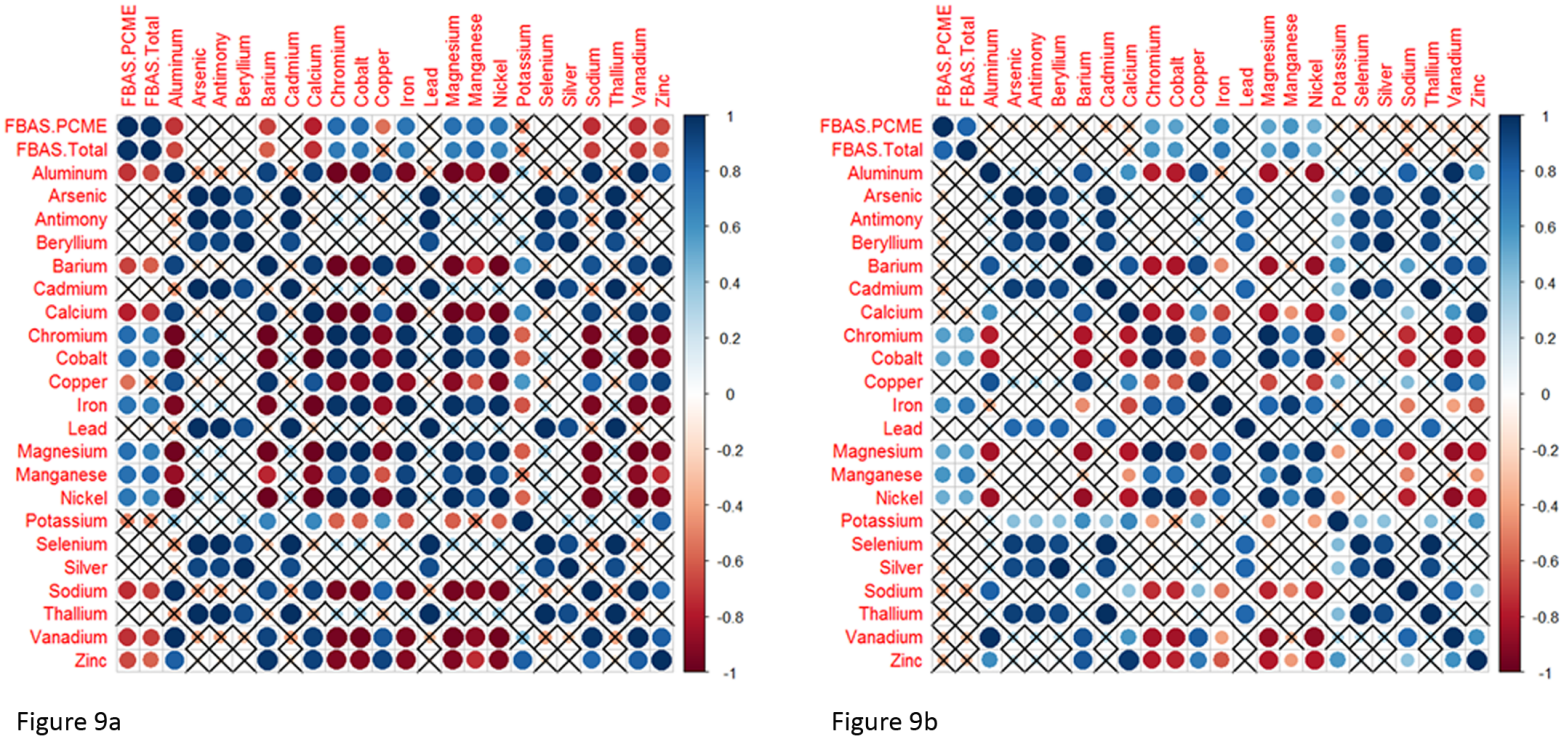
Correlation plot for asbestos versus metals (measured concentrations from ISM samples (9a) and discrete samples (9b)). Correlations were calculated using Kendall’s Tau. Non-significant correlations are indicated by an X (p = 0.05). Dark blue = strong positive correlation; Dark red = strong negative correlation.

Fig 9a shows a plot of metals correlated with the FBAS total asbestos concentrations and FBAS PCME asbestos concentrations for ISM samples while Fig 9b shows the same plot for discrete samples. This analysis revealed positive correlations between asbestos and chromium, cobalt, iron, magnesium, manganese and nickel; and negative correlations between asbestos and aluminum, barium, calcium, sodium, and zinc. Our data is consistent with observations made by Schreier et al [9] of Ca:Mg ratios of around 0.02 in Swift Creek sediments. Our data suggests that when Ca:Mg is between 0.01 and 0.03, then asbestos concentrations are greater than 1%. Table 5 shows the Ca:Mg ratios for all DUs compared to CARB 435 asbestos concentrations.

**Table 5:** Comparison of asbestos concentration to calcium: Magnesium ratio.

Decision Unit/Sample Type	Mean Percent Asbestos (CARB 435)	Mean Magnesium Concentration (mg/kg)	Mean Calcium Concentration (mg/kg)	Calcium:Magnesium
1 ISM	0.13	16833	7433	0.44
1 Discrete	0.05	25126	4180	0.17
2 ISM	0.083	16800	7000	0.42
2 Discrete	0.2	15560	3190	0.21
3 ISM	0.25	27800	6900	0.25
3 Discrete	0.95	41860	2826	0.068
4 ISM	10.42	144333	3767	0.026
4 Discrete	2.3	134180	2614	0.019
5 ISM	9.92	184000	3433	0.019
5 Discrete	10.35	157200	1822	0.012

mg/kg—milligrams per kilogram

### Quality assurance/quality control

EPA performed quality assurance/quality control review of analytical data. This review involved examining the asbestos laboratory’s calibration procedures, bench sheets and supporting documentation [25]. This validation revealed some discrepancies that resulted in qualifying some of the results as estimated; however, the data were still considered valid for the purposes of our study. Data validation packages are included (S4 through S11 Text). For FBAS samples, it appears as if sampling error was responsible for elevated RSDs as laboratory replicates showed good agreement. The data for other analytical methods did not lend itself to this approach due to the high frequency of nondetects in the asbestos data set.

## Discussion

This project supports the need for better, more accurate methods for determining asbestos levels in soils. Variability occurs during sample collection and analysis, depending on the methodologies chosen. Collection of triplicate ISM samples for each DU allowed for calculation of RSDs for metals and asbestos. Relatively low RSDs were calculated for ISM metals data compared to the RSDs for asbestos samples by either sampling method (ISM or discrete). While the low variability of the metals in discrete samples suggests a relatively homogenous underlying soil matrix, the variability of metals in ISM samples was even lower. The high variability of asbestos in soil samples regardless of sampling method suggests that either asbestos behaves differently from metals in the soil, or that the available laboratory preparation and analytical methods provide less reliable and more variable estimates of soil concentrations.

Asbestos soil analytical methods may not be reporting accurate concentrations due to inherent heterogeneity of asbestos in soil, the analytical methods may not be not sensitive enough, or there may be some fundamental property of asbestos, or the analytical methods used to measure it, which creates special challenges for reliably determining asbestos concentrations in soil. Counting asbestos particles that meet specified morphological and mineralogical criteria may also introduce heterogeneity that isn’t present in metals analysis. It is also possible that collection of 30 increments for each ISM sample was insufficient to account for the inherent variability of the asbestos in the soil samples. The ITRC’s ISM guidance document suggests 50–100 increments when high heterogeneity is expected; however, collecting as many as 100 increments for each ISM sample would have been difficult given the site conditions encountered (high grass) for this sampling effort.

Metals analysis methods for ISM samples account for the increased sample volume that is submitted to the lab following slabcake subsampling and generation of the 10-gram sample. This increased volume is extracted so that the analysis result better reflects what was sampled using ISM techniques. Asbestos analytical laboratories have not yet, but are in the process of, improving their sample processing techniques so that they too can better represent in the analysis what comes to the laboratory in the sample jar. For example, recognition that sieving samples into different particle size fractions and analyzing these to generate an estimate of asbestos in the original sample is one technique to better represent a large sample. Alternatively, using riffle splitters, Turbula mixers, or other homogenization techniques to allow for the smaller sample that is analyzed in the lab to better represent what was placed into the sample jar will aid in determining a better estimate of the mean of the area sampled—the ultimate goal of environmental sampling for risk-based decision making.

One research objective was to establish a hierarchy of analytical methods for reliably characterizing asbestos levels in site soils. Our intent was to be able to rank these methods in terms of their relative sensitivity, but given the limitations of the study, we can only conclude that the FBAS/TEM approach appears to be more sensitive at finding asbestos than other methods. There were many non-detects using the CARB 435 and ASTM D7521 methods. An added complication to comparing the methods is the variety of units reported by different methods, which are not necessarily interconvertible. As a result, our ability to compare these methods quantitatively is limited.

Recent research into asbestos exposure in Libby, MT, found that disturbance of soils with less than 1% asbestos could result in airborne concentrations of asbestos that are a potential health concern [26]. These findings highlight the public health need to be able to reliably identify asbestos in soil at low concentrations. Unfortunately, analytical methods have not yet caught up to this need. EPA’s PLM method for asbestos in bulk materials (not soil) was developed with a threshold of 1% because that was deemed sufficient to find asbestos in products to which asbestos was intentionally added; CARB 435, with a sensitivity of 0.25%, was developed for testing aggregate in California for use in construction projects. Neither the EPA PLM method nor CARB 435 method was developed for measuring asbestos in soil; EPA asbestos investigations have helped to demonstrate that the 1% threshold for soil is insufficient for making risk-based decisions [1].

CARB is developing guidance to improve the sample preparation techniques associated with Method 435 [16]. First, stereromicroscopic evaluation of samples at lower magnification is done to identify any fibers apparent in the sample prior to any processing. Further recommendations are focused on use of 3D mixers, pulverization techniques to achieve the most consistent material without overgrinding/loss of fibers, and selecting portions of the prepared sample for analysis to maximize sample representativeness.

Similarly, ASTM D7521 improves earlier soil methods by accounting for the mass of each size fraction of samples and determining asbestos concentrations in each. The resulting concentration may better represent the original sample submitted to the lab by factoring in the total mass of the sample, instead of using a portion to represent the whole.

Newer methodologies appear to be more promising at detecting low levels of asbestos in soil. Soil samples prepared using the FBAS and subsequently analyzed by ISO 10312 had the greatest sensitivity (asbestos was detected in nearly every sample); however, these results also had the greatest variability. Some reasons for this may include the following:

- The small subsample mass used to generate the analytical result analyzed may account for some of the variability. Unlike metals samples where the exact amount needed in analysis is generated using ISM subsampling techniques, a small aliquot of the total sample is used in FBAS sample preparation. Soil samples are collected in the field, transported to the laboratory, and a subsample of these is mixed with laboratory-grade Ottawa sand as a fluidization matrix amendment to prepare a filter for analysis by TEM [19]. This could lead to subsampling error. The material deposited on the filter only represents what becomes airborne from the sample during the FBAS processing which is typically 3-minutes in duration. So, it is representative of the fraction fine enough to become airborne. This is by design as it helps segregate the light particles like asbestos from the larger course particles that serve as interferences during bulk analysis like PLM (CARB 435 or ASTM D7521, for example).
- The FBAS operator often adjusts the mass of the soil subsample used to optimize the best combination of soil versus sand. For this project, the FBAS operator did two test sample checks to try to get particulate loading on the filter to range between 5 and 20%. Any more than 20% and the filter would have been overloaded. Only about 0.5 to 1 gram of soil was used to load the filters due to the fine soil texture and large amount of root fibers contained in the samples. This is consistent with the FBAS SOP [19].
- Fiber types (e.g., certain amphiboles) not associated with the site were found in samples with lower concentrations of asbestos (e.g., those from DUs 1, 2, and 3). This could indicate that contamination is being introduced at some step of the sample preparation procedure or could result from TEM analytical laboratory contamination. However, the TEM laboratory ran 3 filter lot blanks, 5 laboratory blanks, and 2 sand blanks. Asbestos wasn’t found in any of these samples, ruling out potential laboratory contamination.
- The sensitivity of the FBAS prepared samples is much better than other analytical methods for soil. There may be greater variability at low levels. However, this doesn’t seem supported by the high RSDs also observed in samples from DUs 4 and 5.

While additional research is needed to better refine FBAS methodologies and understand the drivers of variability, our research has shown that FBAS with ISO 10312 is the most promising at detecting the presence of asbestos at low concentrations.

Testing whether there was a correlation between metals and asbestos was an ancillary effort to this research project. If the presence of specific metals, at a given location, correlated with asbestos concentrations, then real time detection methods available for metals could be used to quickly and cheaply identify areas for further asbestos testing. Schreier [9, 18] notes that some metals, especially magnesium, nickel and chromium, are associated with the Sumas Mountain slide material. Table 4 clearly shows that concentrations of these metals and several others (cobalt, iron, manganese) are elevated in the DUs that have higher asbestos concentrations. Similarly, the calcium to magnesium ratio (Ca:Mg) is between 0.01 and 0.03, indicating the presence of serpentinite soils [9, 27, 28, 29]. The mix of mineral species occurring in the Sumas/Swift Creek sediments are variable and have some very similar or almost the same chemistry as chrysotile, but differ in crystal structure and morphology. It is difficult to distinguish natural soil values from that of asbestos in these areas given the quantity of asbestos in these soils. Altered serpentinite formations in the landslide contribute discrete sources of chrysotile, lizardite, the hydroxy minerals (brucite, coalingite and pyroaurite), chlorite, and magnetite to the Swift Creek drainage (S12 Text. Trip Report and X-Ray Diffraction Analysis). Over the past decade, EPA’s sampling at the site has indicated when the Ca:Mg ratio is less than about 0.1 then asbestos concentrations in soils are often elevated. Similarly, other data show that when magnesium concentrations are greater than about 100,000 mg/kg then asbestos is likely to be present at concentrations greater than trace levels. Both Ca:Mg ratio and elevated magnesium were noted in our data at DUs 4 and 5. These findings are intriguing, and, if similar findings are reported at other sites, metals data may eventually prove to be useful in helping to guide identification of serpentinite soils containing asbestos in soil investigations.

The rate at which asbestos in soil becomes disturbed and distributed into the air is an important driver of risk at sites like Sumas Mountain. Unfortunately, potentially due to high humidity during sampling and abundant ground cover, asbestos in the air was not detected in most ABS samples. While limited by the low amount of asbestos detected in air in the present study, a qualitative comparison of air to soil results shows some trends. Air concentrations of asbestos were markedly lower for DUs 1, 2, and 3 than for DUs 4 and 5. This is consistent with the soil findings that show higher asbestos concentrations in the soil of DUs 4 and 5. ABS concentrations were higher than stationary air samples at each DU, except DU 1. Total asbestos concentrations were always higher than PCME concentrations which is expected since PCME is a subset of total asbestos fibers. Since ABS has been considered to be the “gold-standard” for use in risk assessments, the inability to link asbestos releases from soil to air introduced uncertainty in human exposure estimates [1]. Repeating this study in areas with high concentrations of asbestos in dry conditions may result in a more robust dataset for addressing this question. For example, at El Dorado, California, ABS with disturbed soils at schools and recreation areas showed the presence of asbestos at elevated levels in air at breathing heights for children and adults (S13 Text. El Dorado Hills Naturally Occurring Asbestos Multimedia Exposure Assessment). Similarly, EPA Region 6 conducted ABS at sites with similar asbestos levels in soils in Louisiana and New Mexico and found higher asbestos concentrations in air when the environment was more arid [26].

## Recommendations for future research

This study did not achieve its goal of identifying a combination of sampling, preparation, and analytical methods that would result in reproducible sample data for asbestos in soil, although we did find that FBAS/ISO 10312 was the most sensitive method available. Future study is needed to ascertain whether statistical error is due to field sampling and/or preparation, analytical preparation and/or analysis. Field sampling error could be addressed by increasing the number of increments (50–100) which may adequately address heterogeneity of asbestos in soils and allow for more reproducible sample results. Field preparation error could be addressed by having subsampling performed more consistently, perhaps in a laboratory instead of in the field. Analytical preparation error could be addressed by improving preparation of samples such as using a Turbula mixer to more thoroughly mix soils in the laboratory. Analytical error might be addressed through analysis of a larger sample aliquot or through improvements in analytical methods [14]. Gy discusses systematic methods for resolving sampling error [30, 31]. Additional lines of inquiry should also include determining whether there is a difference in data reproducibility between fiber types (chrysotile vs amphibole fibers) and continued exploration of metals ratios as a predictor of asbestos content in soil.

## Supporting information

S1 Table Summary of p-values for metals. P-value calculations for decision units.
(XLSX)

S1 Text Borrow evaluation for DSL lands. (PDF)

S2 Text QAPP. This is the Quality Assurance Project Plan for the sampling and analysis effort.
(PDF)

S3 Text Sumas asbestos methods FBAS. Case narrative for Technical Support—Fluidized Bed Asbestos Segregator.
(PDF)

S4 Text 141013 asbestos validation report. (PDF)

S5 Text 141014 asbestos validation report. (PDF)

S6 Text 142958 asbestos validation report. (PDF)

S7 Text 142959 asbestos validation report. (PDF)

S8 Text 142960 asbestos validation report. (PDF)

S9 Text 142961 asbestos validation report. (PDF)

S10 Text 150099 asbestos validation report. (PDF)

S11 Text QA SFP-078 Sumas Mt metals. Quality Assurance Review of the Sumas Mountain Asbestos Site Regional Methods Study for Metals.
(PDF)

S12 Text Trip report and X-ray diffraction analysis. Trip report and X-ray diffraction of samples collected April 6, 2006.
(PDF)

S13 Text El dorado hills naturally occurring asbestos. El Dorado Hills Naturally Occurring Asbestos Multimedia Exposure Study.
(PDF)
