# Supplementary material for: Comparison of soil sampling and analytical methods for asbestos at the Sumas Mountain Asbestos Site—Working towards a toolbox for better assessment
Source: PLoS One. 2017 Jul 31;12(7):e0180210. doi: 10.1371/journal.pone.0180210 (PMC5536314; doi:10.1371/journal.pone.0180210)
Supplement: S1 Text — (PDF) [file pone.0180210.s002.pdf]

UNITED STATES ENVIRONMENTAL PROTECTION AGENCY
REGION 10
1200 Sixth Avenue, Suite 900
Seattle, WA 98101-3140

OFFICE OF
ENVIRONMENTAL ASSESSMENT

September 4, 2013

MEMORANDUM

SUBJECT: Borrow Evaluation for DSL Lands, North Ridge Estate Site, Klamath Falls, Oregon

FROM: Julie Wroble, Region 10 Toxicologist

TO: Denise Baker, Remedial Project Manager

The United States Environmental Protection Agency (EPA) collected surface and subsurface samples at four borrow source locations (study area) at the Oregon Department of State Lands (DSL) to the northwest of the North Ridge Estates Site (see Figure 1 [CDM Smith 2013]). These samples were collected and analyzed using a variety of techniques to characterize and assess the concentration of metals, pesticides, polychlorinated biphenyls (PCBs), herbicides, total petroleum hydrocarbons (TPH), semi-volatile organic compounds (SVOC), and asbestos in soil from potential borrow sources. A description of the work that was performed is included in the Final Oregon Department of State Land Borrow Source Investigation, North Ridge Estates Site, Klamath County, Oregon, Operable Unit 1 (CDM Smith 2013). This memo will focus on the results of the asbestos sampling and analysis.

Sampling of DSL lands was conducted in October of 2012 to identify suitable borrow sources to use as capping material on site. Samples were collected and analyzed for asbestos using three different methods: 1) Soil samples were analyzed by polarized light microscopy (PLM); 2) Soil samples were prepared using a fluidized bed asbestos segregator (FBAS) and resultant filters analyzed by transmission electronic microscopy (TEM); and, 3) activity-based sampling (ABS) was conducted and the resulting air filters were analyzed by TEM (see Table 1). Each of these methods has benefits and limitations, but together they provide a robust dataset for determining the presence of asbestos contamination at potential borrow source locations.

Sample Collection and Analysis

Polarized Light Microscope Analysis of Soil Samples

All samples were collected using Incremental Sampling Methodology (ISM) to better represent spatial distribution of contaminant levels within each borrow source decision unit (DU) and also to reduce sampling variability (ITRC 2012). Thirty increments were collected from each DU at the soil surface and from excavated materials to determine the concentrations at depth. Three replicates were taken from surface and subsurface soils at four potential borrow source DUs, resulting in 24 PLM results[1]. Asbestos was not detected by PLM in any of the potential borrow source samples. The analytical sensitivity achieved by PLM was 0.25%; however, no fibers were

[1] Three replicates at two depth intervals (surface and subsurface) and four locations = 24

Printed on Recycled Paper

observed in the field of view during scans of the prepared mounts so the practical analytical sensitivity for this method was somewhat lower than 0.25%.

Fluidized Bed Asbestos Segregator Analysis of Soils (FBAS)
The FBAS is a tool currently under development by EPA to assess the presence of releasable asbestos fibers from a soil matrix. The FBAS is a bench top apparatus used to elutriate small particles (such as asbestos fibers) from an unconsolidated matrix, such as soil. FBAS is useful for determining low levels of asbestos in soils and was used to determine whether asbestos was present in soils from DSL borrow sources. Using the samples collected as described above, a small amount of soil from the ISM collected soils (e.g., a few grams) was placed in the FBAS with laboratory grade Ottawa 20/30 sand, which is used as a fluidization catalyst. The FBAS is used to prepare an air filter for analysis by TEM. The resulting soil concentration is expressed as asbestos structures per gram (s/g) of soil. Of the 24 samples collected on the DSL lands, asbestos was detected in 5 samples. Two FBAS filters were overloaded with particulates and were not able to be analyzed. Chrysotile was the fiber type detected in the 5 samples, but in each case, the detected fibers were smaller than phase contrast microscopy-equivalent (PCME) dimensions[2]. In surface soil, one sample from one DU had a single chrysotile fiber detected. This sample had triplicate FBAS filters prepared (for quality assurance purposes) and chrysotile was detected in only 1 of 3 triplicates. In subsurface soil, a single chrysotile fiber was detected in 3 samples and two chrysotile fibers were detected in one sample. No amphibole asbestos was reported in any sample.

Detected concentrations of chrysotile asbestos in soil ranged from 1.4E+04 to 2.8E+04 s/g. Using the regression equation for chrysotile in soil provided in Januch et al. (2013), this concentrations would equate to a soil level of about 0.001 percent by mass. While these results should not be considered definitive, they provide another line of evidence that potential borrow soils are mostly free from asbestos, consistent with an interpretation that these soils have not been affected by any release from the site, and low level detections represent either natural conditions or limited anthropogenic impacts.

Activity-Based Sampling (ABS) of Surface Soil and Excavated Material
ABS is routinely used for determining potential risks posed by asbestos present in soil at contaminated sites. This sampling method is used because the exposure medium of interest for asbestos is air and soil sampling results cannot be easily related to air concentrations within a person's breathing zone. ABS samples for assessing asbestos concentrations in surface and subsurface soils were collected while a member of the sample team raked soils – simulating a common soil-disturbing activity that a homeowner might perform. Surface soils were raked before any excavation was conducted in the study area. This sampling represented exposures to possible surface contamination in the study area. Then, the study area was excavated, the materials were stockpiled on plastic sheets, and the materials on the plastic sheets were raked again. The raking activity conducted on the excavated soils stockpile represented exposures to subsurface soils. During the raking activity, the sampler wore two air monitoring pumps – one set to a high flow rate and one set to a low flow rate, resulting in the collection of a high volume (HV) filter and a low volume (LV) filter over the same sampling interval. The HV filter was

[2] PCME structures are defined as being longer than 5 micrometers (µm), with a width of between 0.25and 3 µm, and an aspect ratio (length:width) of 3:1 or greater.

Printed on Recycled Paper

analyzed in preference to the LV filter; but the LV filter was analyzed if the HV filter was overloaded with particulates. If both filters were deemed to be overloaded, no analysis was performed. Of the 24 ABS samples collected from the DSL lands, two were overloaded with particulate and not analyzed. Actinolite asbestos was detected in one of three replicates from one surface sample location at a concentration of 3.3E-04 PCME structures per cubic centimeter (s/cc).

The impact of a single actinolite fiber in a single ABS sample can be assessed by pooling the data. Pooling the data is a common technique employed at asbestos-contaminated sites to generate an exposure point concentration from a group of exchangeable sample results and was used to determine average ambient air concentrations in the baseline risk assessment for the North Ridge Estates Site (CDM 2010). When pooling results, it is assumed that all of the ABS samples collected on potential borrow soils are exchangeable (i.e., interchangeable). The pooled concentration is calculated as follows:

$$Cpooled = \sum N_i / \sum (1/S_i)$$

where:

Cpooled = the pooled air concentration (s/cc)
N_i = the total number of structures observed in analysis 'i' (s)
$1/S_i$ = the reciprocal of the achieved sensitivity in analysis 'i' (cc)

For the DSL lands data set for ABS, there are 22 results available for which data can be pooled.[3] The analytical sensitivities ranged from 3.2E-04 fibers per cubic centimeter (f/cc) to 3.4E-04 f/cc. Table 1 shows the calculations described above. If these samples are assumed to be exchangeable (which they are since they all represent borrow sources), then the data can be pooled. The single fiber detected is assumed to be found over the total volume of air collected for these samples, resulting in a pooled concentration of 1.5E-5 PCME s/cc (see Table 2).

Sampling for organics and inorganic substances also was conducted on the DSL lands. Oregon DEQ concluded that the results of this sampling indicated that this soil was suitable as fill and elevated levels of arsenic and chromium were consistent with background concentrations of these metals (ODEQ 2013).

Risk Evaluation of ABS Data

The ABS sample results for DSL lands soils can be assessed using the same risk assessment approach as was used in the baseline risk assessment for the North Ridge Estates Site (CDM 2010). If borrow area soils are used on site as a capping material in the future, then future on-site residents could be assumed to perform soil disturbing activities (such as raking their yards) on these soils at the same frequency as was assumed in the risk assessment. Therefore, a time-weighting factor (TWF) can be determined based on 5 hours of exposure a day, 100 days a year, for 30 years. The resulting TWF is 0.024. The pooled mean asbestos concentration for borrow soils (i.e., 1.5E-05 PCME s/cc) can be multiplied by the TWF and the inhalation unit risk for

[3] Two overloaded filters could not be analyzed and are excluded from the pooled results.

Printed on Recycled Paper

asbestos (i.e., 0.23 per f/cc) and the resulting risk is 8E-08, which is well below EPA's and DEQ's acceptable risk ranges.

In the event that borrow material is taken from a larger area than the study area, then pooling data for both borrow and background and assessing the potential risk is appropriate
The FBAS results further support the determination that asbestos levels in potential borrow source areas is very low.

Recommendations
In conclusion, the soils from the DSL lands can be considered suitable as borrow material for on-site uses. Fibers may be found occasionally in these samples, as evidenced by the sample results summarized above; however, asbestos concentrations are expected to be sufficiently low so that risks should not exceed EPA's or ODEQ's acceptable risk level of 1E-06.

Printed on Recycled Paper

REFERENCES

CDM Smith, 2013. Final Oregon Department of State Land Borrow Source Investigation, North Ridge Estates Site, Klamath County, Oregon, Operable Unit 1. September 2013.

CDM Federal Programs Corporation (CDM). 2010. Final Remedial Investigation Report, North Ridge Estates Site, Klamath County, Oregon. January.

ITRC (Interstate Technology & Regulatory Council). 2012. Incremental Sampling Methodology. ISM-1. Washington, D.C.: Interstate Technology & Regulatory Council, Incremental Sampling Methodology Team. www.itrcweb.org.

Januch, J., et al. 2013. "Evaluation of a fluidized bed asbestos segregator preparation method for the analysis of low-levels of asbestos in soil and other solid media." Analytical Methods **5**(7): 1658-1668.

ODEQ 2013. Department of State Lands Borrow Soil Evaluation. Memorandum from Susan Turnblom and Paul Seidel, ODEQ to Denise- Baker, U.S. Environmental Protection Agency, December 2, 2013.

Printed on Recycled Paper

Table 1

		PLM-CARB 435		ABS-TEM		FBAS-TEM	
		Surface	Sub-surface	Surface	Sub-surface	Surface	Sub-surface
**Borrow Locations (DSL Land)**							
DU1	Rep 1	ND	ND	ND	ND	ND	--
	Rep 2	ND	ND	---	ND	--	ND
	Rep 3	ND	ND	---	ND	ND	ND
DU2	Rep 1	ND	ND	ND	ND	ND	1.4E+04 s/g [CH]
	Rep 2	ND	ND	ND	ND	ND	1.4E+04 s/g [CH]
	Rep 3	ND	ND	ND	ND	ND	ND
DU3	Rep 1	ND	ND	0.00033 s/cc [AC]	ND	ND	ND
	Rep 2	ND	ND	ND	ND	1.5E+04 s/g [CH]*	2.8E+04 s/g [CH]**
	Rep 3	ND	ND	ND	ND	ND	ND
DU4	Rep 1	ND	ND	ND	ND	ND	ND
	Rep 2	ND	ND	ND	ND	ND	ND
	Rep 3	ND	ND	ND	ND	ND	1.4E+04 s/g [CH]

ND = non-detect

--- = overloaded; TEM analysis not performed

$^{+}$ Based on PCME

*Chrysotile was detected in one of three ***replicates*** collected at this location.

** Two chrysotile fibers were detected in the FBAS reprep of this sample.

Notes:

PLM-CARB 435 analytical sensitivity was 0.25% as a point count. Also, no structures were detected in any fields of view in any sample.

ABS-TEM analytical sensitivity was between 0.00032 and 0.00034 s/cc for all samples.

Actinolite fiber detected in ABS sample meets PCME size requirements.

Chrysotile fibers detected in FBAS samples were all too small to be considered PCME fibers.

Table 2

			ABS-TEM			
			**Sample ID**	**Senstivity (cc)$^{-1}$**	**Number of Fibers**	**Reciprocal**
**Borrow Locations (DSL Land)**						
DU1	Surface	Rep 1	DU1-SS1_LV	3.4E-04	0	2.9E+03
		Rep 2	DU1-SS2-LV	---		
		Rep 3	DU1-SS3-LV	---		
	Sub-surface	Rep 1	DU1-SUB1-HV	3.2E-04	0	3.1E+03
		Rep 2	DU1-SUB2-HV	3.4E-04	0	2.9E+03
		Rep 3	DU1-SUB3-HV	3.4E-04	0	2.9E+03
DU2	Surface	Rep 1	DU2-SS1-LV	3.4E-04	0	2.9E+03
		Rep 2	DU2-SS2-HV	3.4E-04	0	2.9E+03
		Rep 3	DU2-SS3-LV	3.4E-04	0	2.9E+03
	Sub-surface	Rep 1	DU2-SUB1-HV	3.4E-04	0	2.9E+03
		Rep 2	DU2-SUB2-HV	3.3E-04	0	3.0E+03
		Rep 3	DU2-SUB3-HV	3.2E-04	0	3.1E+03
DU3	Surface	Rep 1	DU3-SS1-LV	3.3E-04	1	3.0E+03
		Rep 2	DU3-SS2-LV	3.2E-04	0	3.1E+03
		Rep 3	DU3-SS3-HV	3.4E-04	0	2.9E+03
	Sub-surface	Rep 1	DU3-SUB1-HV	3.4E-04	0	2.9E+03
		Rep 2	DU3-SUB2-HV	3.4E-04	0	2.9E+03
		Rep 3	DU3-SUB3-HV	3.3E-04	0	3.0E+03
DU4	Surface	Rep 1	DU4-SS1-HV	3.4E-04	0	2.9E+03
		Rep 2	DU4-SS2-HV	3.4E-04	0	2.9E+03
		Rep 3	DU4-SS3-HV	3.3E-04	0	3.0E+03
	Sub-surface	Rep 1	DU4-SUB1-HV	3.3E-04	0	3.0E+03
		Rep 2	DU4-SUB2-HV	3.3E-04	0	3.0E+03
		Rep 3	DU4-SUB3-HV	3.3E-04	0	3.0E+03
					**Sum of Recicprocals**	6.6E+04
					**Reciprocal of the Sum**	1.5E-05
