# Supplementary material for: Comparison of soil sampling and analytical methods for asbestos at the Sumas Mountain Asbestos Site—Working towards a toolbox for better assessment
Source: PLoS One. 2017 Jul 31;12(7):e0180210. doi: 10.1371/journal.pone.0180210 (PMC5536314; doi:10.1371/journal.pone.0180210)
Supplement: S2 Text — This is the Quality Assurance Project Plan for the sampling and analysis effort. (PDF) [file pone.0180210.s003.pdf]

QUALITY ASSURANCE PROJECT PLAN (QAPP)

FOR

Regional Methods Project: Comparison of Soil Sampling Methods for Asbestos at the Sumas Mountain Asbestos Site, Whatcom County Washington

Date: September 2014
Revision: 2.2

APPROVAL OF QAPP:

__________________________________ Date: 9/25/14
Julie Wroble, Project Manager
Risk Evaluation Unit
Office of Environmental Assessment

__________________________________ Date: 9/26/2014
Sheila Fleming, Manager
Risk Evaluation Unit
Office of Environmental Assessment

__________________________________ Date: 9/25/14
Ginna Grepo-Grove, Regional QA Manager
Office of Environmental Assessment (OEA)

TABLE OF CONTENTS

A. PROJECT MANAGEMENT 4
A3 – DISTRIBUTION LIST 4
A4 – PROJECT/ TASK ORGANIZATION 4
A5 – PROBLEM DEFINITION/BACKGROUND 6
Location *7*
Problem Definition .... ***Error! Bookmark not defined.***
A6 – PROJECT TASK DESCRIPTION 7
Table 1 – Schedule of Tasks *8*
Health and Safety *8*
A7 – QUALITY OBJECTIVES AND CRITERIA 8
A8 – SPECIAL TRAINING AND CERTIFICATION 14
A9 – DOCUMENTS AND RECORDS 14
B. DATA GENERATION AND ACQUISITION 15
B1 – SAMPLING PROCESS DESIGN (EXPERIMENTAL DESIGN) 15
B2 – SAMPLING METHODS 15
B3 – SAMPLE HANDLING AND CUSTODY 17
B5 – QUALITY CONTROL 18
B6 – INSTRUMENT/EQUIPMENT TESTING, INSPECTION, AND MAINTENANCE 20
B7 – INSTRUMENT/EQUIPMENT CALIBRATION AND FREQUENCY 21
B8 – INSPECTION/ACCEPTANCE OF CONSUMABLE SUPPLIES 21
B9 – NON-DIRECT MEASUREMENTS 21
B10 – DATA MANAGEMENT 21
C. ASSESSMENT AND OVERSIGHT 22
C1 – ASSESSMENTS AND RESPONSE ACTIONS 22
C2 – REPORTS TO MANAGEMENT 22
D. DATA VALIDATION AND USABILITY 22
D1 – DATA REVIEW, VERIFICATION, AND VALIDATION 22
D2 – VERIFICATION AND VALIDATION METHODS 23
D3 – RECONCILIATION WITH USER REQUIREMENTS 24
TABLE 2 AND 3 – DATA QUALITY OBJECTIVES SUMMARIES 25
ATTACHMENT 1 – SAMPLE ALTERATION FORM .... ERROR! BOOKMARK NOT DEFINED.
ATTACHMENT 2 – CORRECTIVE ACTION FORM 27

Appendix A – Sampling and Analysis Plan
Appendix B – Standard Operating Procedures
Appendix C – Site Safety Plan
Appendix D – Users Manuals
Appendix E – Field Data Sheets, Chain of Custody Forms
Appendix F – Statements of Work
Appendix G - NADES

ACRONYMS

ABS – Activity-Based Sampling
AHERA – Asbestos Hazard Emergency Response Act
ASTM – American Society for Testing and Materials
BL – Procedural Blank
CARB – California Air Resources Board
CLP – Contract Laboratory Program
FBAS - Fluidized Bed Asbestos Segregator
HDPE – High-density Polyethylene
ISM – Incremental Sampling Methodology
ISO – International Organization for Standardization
LCS – Laboratory Control Sample
MEL - Manchester Environmental Laboratory
MRL – Method reporting limit
NIST – National Institute of Standards and Technology
NVLAP – National Voluntary Laboratory Accreditation Program
ORD – Office of Research and Development
OSHA – Occupational Safety and Health Administration
PARCC – Precision, accuracy, representativeness, completeness, and comparability
PCM – Phase Contrast Microscopy
PDF – Portable Document File
PLM – Polarized Light Microscopy
PPE – Personal Protection Equipment
QA – Quality Assurance
QAO – Quality Assurance Officer
QAPP - Quality Assurance Project Plan
QC – Quality Control
RPD – Relative Percent Difference
RPM – Regional project manager
RSCC – Regional Sample Control Coordinator
S4VE/S4VEM – Stage 4 validation, manually performed
SAF – Sample Alteration Form
SAP – Sampling and Analysis Plan
SOP – Standard Operating Procedures
SOW – Statement of Work
SRM – Standard Reference Material
TEM – Transmission Electron Microscopy
TNI – The NELAC Institute
TRW – Technical Review Workgroup

A. Project Management

A3 – Distribution List

Copies of the completed/signed QA project plan should be distributed to:

Julie Wroble – Regional Methods Project Manager	OEA-140
DanVallero – Contracting Officer Representative	ORD
Jed Januch – Field Team Leader	LAB
Tim Frederick – Environmental Unit Leader	Region 4
Sheila Fleming – Risk Evaluation Unit Manager	OEA-140
Ginna Grepo-Grove – QA Manager	OEA-140
Jennifer Crawford – QA Chemist / RSCC	OEA-140
Don Matheny – QA Chemist / Scribe Project Manager	OEA-140
Grady Maxwell – Site Safety Officer	OEA-140
Sandra Brozusky – Field Team Member	OCE-184
Raymond Wu – Field Team Member	OEA-140
Doc Thompson – Field Support	LAB

At the conclusion of analysis and data validation, electronic laboratory data should be provided to:

Julie Wroble, Regional Methods Project Manager	OEA-140
Jed Januch, Field Team Leader	LAB
Tim Frederick, Environmental Unit Leader	Region 4

A4 – Project/ Task Organization

The following individuals are EPA staff with responsibility for the design and implementation of this project, and will be the primary data users and decision makers:

- **Julie Wroble,** (206) 553-1079 (primary contact), Toxicologist responsible for preparing the QAPP and the sampling and analysis plan, review and analysis of data, data verification for asbestos, and final report preparation.

- **Dan Vallero**, (919) 541-3306, Contracting Officer Representative serving as the primary point of contact for the Asbestos analytical laboratory.

- **Tim Frederick**, (404) 562-8958, Toxicologist responsible for assisting with preparation of the QAPP, overseeing incremental sampling activities, and assisting with data review and report preparation.

- **Jed Januch**, (360) 871-8731, Investigator responsible for ordering and organizing field supplies and equipment. Establish procedures to ensure integration of sampling data and analytical results; and arrange for use of special equipment.

- **Ginna Grepo-Grove,** (206) 553-1632, Regional QA Manager responsible for assisting the Investigator in the development of the QA Project Plan (QAPP), subsequent revisions, and amendments and for reviewing and approving the QAPP and SAP prior to field mobilization. A R10 QA Chemist with delegated authority will review and approve the QA Documents in lieu of the RQAM.

- **Jennifer Crawford,** (206) 553-6261, Regional Sample Control Coordinator (RSCC) and QA Chemist residing in the OEA ESU, coordinates sample analyses performed by the EPA R10 Laboratory (MEL) and the Superfund Contract Laboratory Program (CLP). The RSCC will provide sample numbers and a unique project code. Jennifer will also assist with field sampling activities.

- **Don Matheny**, (206) 553-2599, QA Chemist and Scribe Project Manager for this event. Responsible for the Scribe software project management to include: pre-field setup and generation of labels; in-field documentation of sample collection, locational data, monitoring data, and COC generation; final data import of validated results into Scribe for upload and archival to Scribe.net. All these activities are in accordance with the R10 Data Management Plan (2014).

- **Grady Maxwell**, (206) 553-0241, Site safety officer ensuring that field activities are performed in accordance with the Site Health and Safety Plan.

- Other team members will assist with collection of environmental samples at the project site in accordance with the procedures specified in this document along with the Sampling and Analysis Plan for the event.

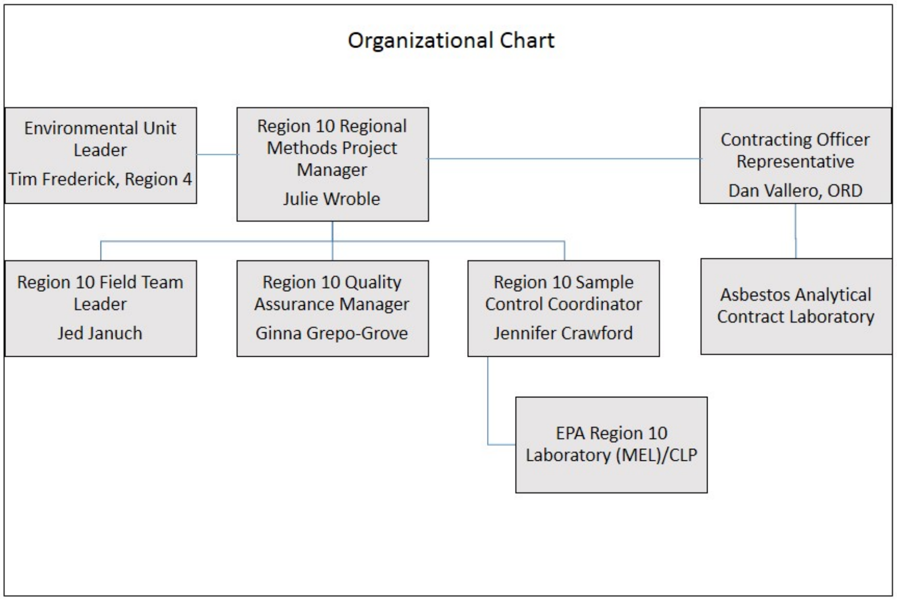

A5 – Problem Definition/Background

A variety of sampling and analysis tools for asbestos in soil are available. However, these tools differ in their ability to detect asbestos and some that are less expensive and most readily available may not be adequate to determine whether asbestos poses a health risk at a given site. A comparison of several sampling and analysis methods is needed to better understand their respective utility.

SITE HISTORY/CONDITIONS

An earth-flow type of landslide on the west side of the Sumas Mountain initiated around 1940 resulted in significant deposition of sediments into Swift Creek during periods of rain and snow melt. Sampling and analysis conducted by EPA over the past several years has confirmed the presence of chrysotile and actinolite asbestos in dredged materials from Swift Creek, material taken from the dredge piles and placed elsewhere, Swift Creek and Sumas River bank sediments, Swift Creek and Sumas River surface water, and upland areas where flood events deposited sediments. The concentration of asbestos in dredged materials, as measured by PLM, ranged from 0.1 % to 4.4%, with an average concentration of 1.6%. The concentration of asbestos in sediments and upland flood deposits, as measured by PLM, ranged from 0.5 % to 27%. EPA also conducted activity-based sampling (ABS) in 2006 to determine whether disturbance of dredged materials would result in breathing zone concentrations of asbestos that potentially pose a risk to human health. The results of this testing indicated that breathing zone concentrations of asbestos during soil disturbing activities resulted in risks at the range that EPA generally considers to be acceptable. Recognizing these risk levels and that other exposures in the community are possible due to historical use of the dredge materials as fill in the community, EPA restricted dredging of Swift Creek sediments. Appropriate disposal of these sediments is not feasible because the costs associated with transport are extraordinarily high. Flooding in 2009 resulted in deposition of sediments onto upland areas at several locations along Swift Creek and the Sumas River. Sampling of these deposits indicated that concentrations were higher than what was found in the dredged materials along Swift Creek and follow up activity-based sampling indicated that exposures to individuals who disturb these materials could result in elevated risks (EPA 2009a, 2011).

SITE LOCATION

The sample locations will be on publicly-owned properties located near Everson along Swift Creek in Whatcom County, Washington.

PROJECT DESCRIPTION

The Environmental question being asked is: How do various soil sampling methods compare and how do these compare with ABS results from the same locations? EPA wants to compare various methods of sampling and analysis of asbestos in soil and air at the Sumas Mountain Asbestos Site to identify a hierarchy of approaches to use for screening and more detailed assessment. The sensitivity, effectiveness, and reproducibility of each method will be evaluated and compared with other methods.

Location

The slide area is located on the west side of Sumas Mountain in Whatcom County (Latitude 48° 54' 801" North, Longitude 122° 14' 099" West). Swift Creek flows in a westerly direction from the slide area to the Sumas River. The sampling locations are located along Swift Creek. The location of the study area for this project will be properties located adjacent to Swift Creek which have been impacted directly by flooding or indirectly via placement of asbestos-containing fill materials or suspected windblown dust. Figure 1 shows these proposed sampling locations.

A6 – Project Task Description

The goal of this sampling event is 1) to compare a variety of soil sampling and analytical techniques for asbestos; 2) collect information on metals content of these soils for comparative analysis; and 3) conduct activity-based sampling to determine airborne concentrations in the soil sample areas. Stationary air samples also will be collected to measure asbestos concentrations in air adjacent to where sampling activities are being performed. The basic field and analytical tasks required to achieve the objectives of this project are listed below:

1) Collect discrete (grab) soil samples for analysis of asbestos and metals (see Table 2).
2) Collect ISM soil samples for analysis of asbestos and metals.
3) Collect personal air monitoring samples on filters during activity based sampling.
4) Collect stationary air monitoring samples on filters during site activities.
5) Screen ABS filters by PCM.
6) Collect meteorological data during activities.
7) Samples will be sent off-site for asbestos and metals analysis.

The quality assurance (QA) requirements described in this document that are critical to the success of this project are derived from EPA QA/R-5 EPA Requirements for Quality Assurance Project Plans (March 2001).

Table 1 includes a schedule for conducting tasks related to this project. It is intended as a guideline only as it is possible that unforeseen circumstances and conditions will require adjustment to some or all of the following dates that have been proposed.

Table 1 – Schedule of Tasks

Activity	Estimated Start Date	Estimated Completion Date	Comments
Asbestos Site-Specific QAPP Review/Approval	August 11, 2014	September 25, 2014	
Sample Collection and Screening Filters by PCM	September 29, 2014	October 3, 2014	
Laboratory Receipt of Samples	October 6, 2014	November 1, 2014	FBAS samples require preparation at MEL and will arrive at the asbestos laboratory later than other samples.
Laboratory Analysis	October 6, 2014	January 31, 2015	
Data Validation/Verification	January 1, 2015	February 28, 2015	

<u>Health and Safety</u>

When working with potentially hazardous materials, investigators are to follow U.S. EPA and site specific health and safety procedures in compliance with Occupational Safety and Health Administration (OSHA) standards. The level of personal protection equipment (PPE) indicated for sampling activities at this site based on the most current information is **Level C**.

A7 – Quality Objectives and Criteria

Overall project objectives include:

Comparing a variety of sampling and analysis techniques for asbestos in soil to determine effectiveness of various methods especially with respect to sensitivity and relative cost.

1) Establish a hierarchy of analytical methods for characterizing asbestos levels in site soils. The criteria for method comparison includes: reproducibility, representativeness, precision, accuracy and sensitivity (see Table 2). [Soil methods compared include: CARB 435, ASTM D7521-13, and FBAS followed by TEM (ISO 10312). Activity-based sampling during sampling will provide an estimate of the level of asbestos in the breathing zone of workers performing these activities.]. Asbestos sampling and analytical combinations will be ordered from most to least sensitive.

2) Evaluate the cost effectiveness of the field and laboratory methods as screening tools to evaluate site conditions and determine if additional site characterization is warranted.

3) Determine the utility of the ISM sample collection approach at asbestos-contaminated sites.

4) A final objective is to determine if there are associations between asbestos and metals concentrations or if the ratios between certain inorganic elements (e.g., Ca:Mg) can be used as an

indicator for the presence of asbestos. Metals data, which have established methods that are reliably utilized at Superfund sites, will also be used to compare the reproducibility of results.

Step 1: State the Problem – A variety of sampling and analysis tools for asbestos in soil are available. However, these tools differ in their ability to detect asbestos and certain less expensive techniques the most readily available may not be adequate to determine whether asbestos does not pose a health risk at a given site. A comparison of several sampling and analysis methods is needed to better understand their respective utility.

Step 2: Identify the Goal of the Study
EPA wants to compare various methods of sampling and analysis of asbestos in soil and air at the Sumas Mountain Asbestos site to determine if there is a clear hierarchy of approaches to be used that will work for screening and more detailed assessment. The sensitivity, effectiveness, and reproducibility of each method will be evaluated and compared with other methods (see Step 6 below). Metals concentrations may be a good indicator of reproducibility of results at a given location.

Step 3: Identify Information Inputs
The information inputs are summarized in the table below. Each of these sets of data will be collected at each of 5 locations.

**Sample Type**	**ISM**	**Discrete**	**Activity-Based**
Number of samples	3	5	3
FBAS/ISO 10312 TEM	Yes	Yes	No
CARB 435	Yes	Yes	No
ASTM D7521	Yes	Yes	No
Metals	Yes	Yes	No
ISO 10312/TEM	No	No	Yes

Step 4: Define the Boundaries of the Study
The physical boundaries of the study site are areas impacted by Sumas Mountain asbestos-containing sediments. These could include areas where flooding has deposited material onto soil; areas where dredged materials were placed as fill; areas where agricultural activities (e.g., irrigation, fill placement) may have resulted in asbestos being present in soil; and areas where windblown dust from the dredged materials has resulted in asbestos being present in soil. The areas selected for study are known to contain asbestos at varying concentrations based upon existing data. A background sample is not being collected, but at least one or two locations are expected to have very low levels of asbestos based on work performed by EPA (2013).

Step 5: Develop the Analytical Approach
The various methods to be used at this site have been used to some degree in the past at EPA-led site investigations with the exception of the ASTM D7521 soil method for asbestos. Each method has advantages and disadvantages as described below.

- The Fluidized Bed Asbestos Segregator (FBAS) is a device that is used to determine the amount of asbestos present in a soil sample in terms of fibers per gram of soil. This device has been

shown to be helpful in detecting very low levels of asbestos in soil (see Januch et al 2013), but requires unique equipment, specialized preparation by a trained person, and expensive ISO 10312 analysis.

- The CARB 435 PLM method for asbestos in soil has been widely used, is relatively inexpensive, but may not be sensitive enough to see levels of asbestos in soil that pose a risk to human health. Therefore, this method may be useful to screen sites "in" for further evaluation, but cannot (without further site-specific evaluation) be used to declare that a site poses no risk to human health.
- The ASTM D7521 method for asbestos in soil may be somewhat more sensitive at achieving lower detection limits than the CARB 435 PLM method, but the ability to see low levels of asbestos in soil may depend on the site conditions, soil type, and asbestos type. This method has not yet been used to any great extent by EPA, but could prove useful as a screening tool.
- Metals analysis will be used to look for a select group of metals. Hans Schreier (2007) has indicated that certain metals ratios are altered in serpentinite soils and we have seen evidence of this in prior investigations at the Sumas Mountain Asbestos site. For example, magnesium concentrations appear to be elevated in samples that contain asbestos concentrations greater than about 1%. In the current study, concentrations of several metals will be evaluated to see if any one indicator or ratio stands out as being well correlated with the presence of serpentinite soils (both chrysotile and lizardite [non-asbestos] are present in Sumas Mountain sediments). Metals analysis can also be used as a surrogate measure for determining reproducibility within a decision unit that can be compared to asbestos methods.
- Activity-based sampling (ABS) has been considered to be the gold standard for collecting data for risk assessment of asbestos-contaminated soils. ABS followed by TEM analysis results in air concentrations of asbestos that can be used to assess risk. The downside of this approach is that data collection efforts are very involved and the analysis of the loaded filters can be expensive. Further, meteorological factors may impact sample results. Stationary samples will be used to assess ambient air conditions during sampling.
- A final aspect of the data collection effort is comparison of sampling techniques. Incremental Sampling Methodology (ISM) will be used to collect soil samples in attempt to better represent the exposure areas of interest. The ISM samples should correlate directly to areas where ABS is performed. In contrast, 5 discrete samples from each of these areas will be collected in a manner similar to what has traditionally been done at asbestos sites. These samples can be evaluated individually and a mean (and other appropriate statistics) for each set of discrete samples can be determined and compared with the ISM data sets.

The data collected as part of this study may not be sufficiently robust to rigorously compare the various methods, but it is typical of the numbers of samples routinely used for decision making and may inform future research. Visualization of the data using box and whisker plots (and other graphical analysis) will be done along with basic statistical analysis. If the project statistician (if needed) determines that the data set is robust enough for more detailed statistical comparisons to be completed, then a more rigorous and complete statistical analysis will be completed.

Step 6: Specify Performance Acceptance Criteria

For each location, there will be 8 samples for each of the following analyses: FBAS/TEM, CARB 435, ASTM D7521, and Metals. 3 of these will be ISM samples and 5 will be discrete grab samples. The ISM samples may need a bit more preparation before analysis because they are larger samples by design and need to be subsampled properly prior to analysis. Subsampling for ISM samples will be performed in the field. In addition, there will be 3 ABS samples for each location that will be analyzed using ISO 10312. Reproducibility will be measured by calculating a relative standard deviation for samples of a similar type. For each location, the method that provides the most reproducible (least variable) data can be determined.

We also want to know which methods are more sensitive for others. So at a given site, perhaps some methods will not detect asbestos in every sample while others will. One limitation in comparing data across various methods is that the analytical units are not the same in each case. As a result, relative comparisons will need to be made from one type of analysis to the next and across sites. Box-whisker plots may be helpful for visualizing data. An example data set for FBAS results across 5 sample locations is shown below.

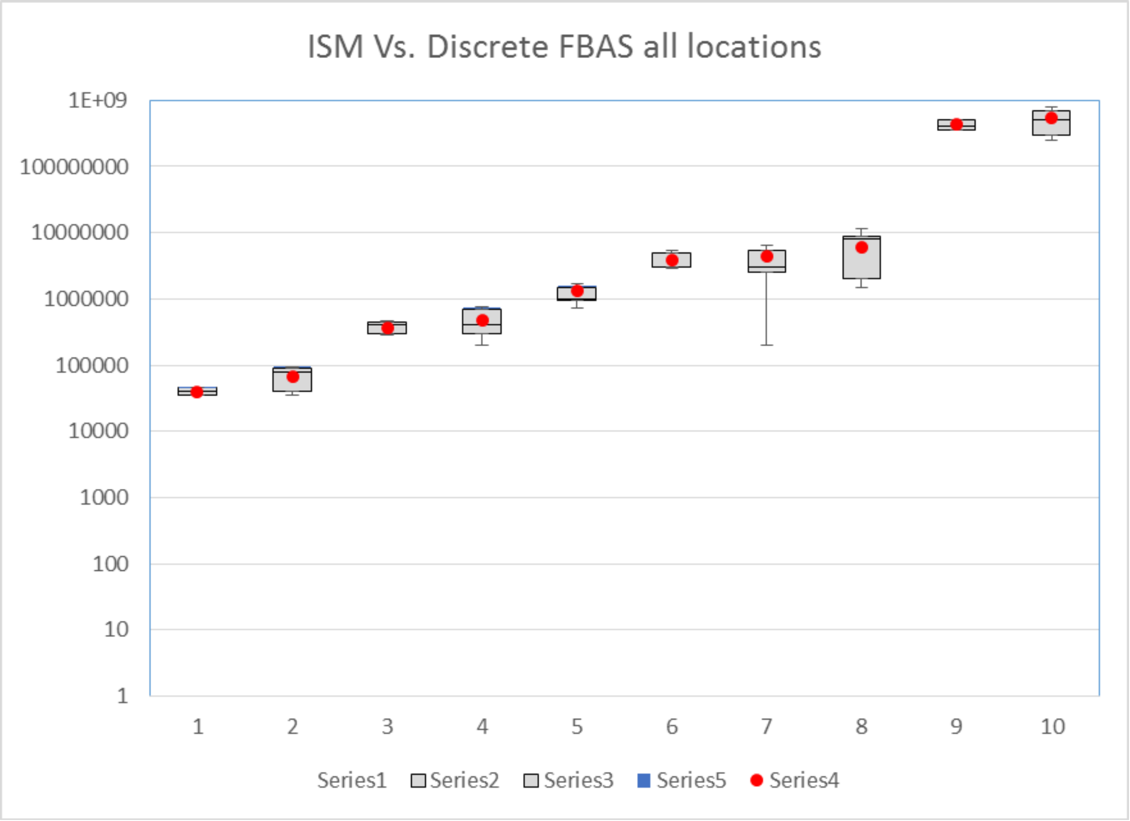

Relationships between asbestos soil concentrations, individual inorganics concentrations and ABS air sample concentrations will be developed for this site. As mentioned previously, ABS is considered the “gold standard” for assessing potential risks associated with asbestos in soil, so it forms the basis for method comparability. At this stage, the connection between ABS and ISM data will be evaluated on a

preliminary basis. Informative data may be used to direct future research. Additional details on sample preparation are included in the Sampling and Analysis Plan (Appendix A).

Since each method relies upon visual quantification, effectiveness will be evaluated based upon the ability of each method to "see" asbestos where it is expected to be present. It will be noted if particular methods "see" asbestos more reliably and at lower detection limits than others. How will we measure effectiveness? Do we see asbestos in each method when we expect to? Given the sensitivity of the method, do we "see" asbestos when we should? Are some methods better at detecting asbestos than others? We expect that ABS and FBAS following by TEM analysis will be the most representative and sensitive techniques.

Step 7: Develop the Plan for Obtaining Data

Step 3 includes a table that summarizes the data to be obtained. Sample locations will be determined in consultation with the RPM for the site. Other stakeholders (Washington Dept. of Ecology, Whatcom County) also may have input in sampling locations. Ideally, areas that have been sampled previously and areas with potentially lower concentrations of asbestos should be targeted because one purpose is to see which methods are most sensitive.

The QAPP includes details on the size of the area to be sampled, the grid spacing used for the ISM samples, the number of increments (i.e., 30) to be included in each ISM sample, and a set of rules for obtaining the 5 discrete samples within that area.

Site Samples

Five locations where we expect to find asbestos have been identified (see Figure 1). At least one of the location is expected to exhibit fairly low levels of asbestos which is beneficial for testing some of the more sensitive techniques (e.g., FBAS followed by ISO 10312).

Background Samples

A background sample will not be collected at this time as limited resources are available and we want to compare asbestos concentrations across all 5 sampling locations.

Who will use the data?

Data will be used by EPA Regions 4 and 10, the Asbestos TRW, and ORD to better understand sampling and analysis tools available for asbestos in soil and air.

What will the data be used for?

The data will be used to understand the comparability between different sampling and analysis methods for asbestos and also to determine a possible hierarchy in approach. The data could be used to determine if more cost effective approaches are appropriate for some sites. The data may also help us understand if ISM is an appropriate and helpful tool for understanding sites with naturally occurring asbestos.

What types of data are needed?

Sampling type and matrix: Soil, Air
Type of Data: Definitive

Analytical Techniques: PCM (for field screening samples) PLM (CARB 435 and ASTM D7521), TEM (soil by ASTM D7521, FBAS, ABS, and stationary samples), FBAS preparation of soil samples, Metals by EPA 200.2/200.7 (or current SOW) for soil samples.

Parameters: e.g., Asbestos-PLM, TEM, Metals

Type of sampling equipment: Field Data Sheets (e.g., time on, time off, initial pressure/flow, final pressure/flow), GPS unit with digital camera, safety utility knife, hand-sprayers and tap-sprayers, re-sealable plastic bags, polyethylene sheeting, generators, air sampling pumps (high-volume and/or low-volume), flow meter (high-volume and/or low/, medium-volume), sample cassettes, disposal plastic scoops, bowls or large plastic bags for soil homogenization, baking trays for ISM subsampling, soil moisture monitor, wooden stakes, yellow warning tape, rope for setting up ISM grid, sample jars, backpacks or belts for wearing personal air sampling pumps, laptop with printer, ABS-specific equipment (*e.g.*, rakes,), PPE (up to Level C), and decontamination supplies, ISM sampling tools (to rent), ISM subsampling scoops.

Access Agreement, if applicable: Yes

Sampling locations: On-site/Off-site

<u>How much data are needed?</u>

The number of samples needed for each analytical group, matrix, and concentration level. At each of 5 sample locations, 3 ISM and 5 discrete samples will be collected (see Table 2). Also, 3 ABS samples will be collected at each location. Field duplicates will be collected at a frequency of 1 in 10 for all sample types. For ISM samples, a triplicate of the triplicate will be collected at one location.

<u>How "good" does the data need to be in order to support the environmental decision?</u>

The data in this study are being used for research purposes only. The data should meet the requirements of the analytical procedures and meet basic validation criteria. R-flagged data may not be appropriate for use in the data evaluation. Establish project sampling/analytical measurement performance criteria for PARCC parameters. Refer to Tables 2 and 3.

<u>Where, when, and how should the data be collected/generated?</u>

Samples will be collected from publicly- and privately-owned properties near Everson, WA (see Figures 1 and 2). Signed access agreements have been obtained by EPA. Samples will be collected the week of September 29, 2014 and data will be available no later than January 31, 2015. All locational data, sample collection information, field monitoring data, shipment/custody documentation, and final validated laboratory data will be maintained in Scribe and published to Scribe.net for archival use in an EPA database according to requirements and valid values specified in the R10 DMP (2014). A backup file of the Scribe project file will be provided to R10 RSCC and the R10 GIS staff within OEA for archival and future use as well.

<u>Who will collect and generate the data?</u>

An asbestos analytical contract laboratory will generate the TEM and PLM data for soil and air samples. MEL/CLP will generate inorganics data for soil samples. Data collection and review will be done by EPA Region 10 staff with assistance from EPA Region 4 staff.

How will the data be reported?

The data will be reported by the field personnel in field logbooks and data sheets. All field locational and monitoring equipment will be set to log/record data where applicable so that file export and subsequent Scribe import may be possible. Final laboratory data will be produced in the EPA Region 10 Standard Lab EDD format (DMP 2014) wherever possible and applicable. The analytical laboratory data will be provided to the Regional Methods Project Manager and Scribe Project Manager. The SOWs contain additional information on project reporting requirements for asbestos data. NADES or equivalent will be used to report the asbestos data (see Appendices F and G).

How will the data be archived?

The Project Manager will provide final, validated data to the Scribe Project Manager for import and archival in the Scribe database for publishing to Scribe.net for assurance of security and archival of all data. Asbestos data will be reported using NADES or equivalent (for ASTM D7521). NADES data does not easily export into Scribe, but Region 10 will determine how best to import the final results into Scribe. The EPA R10 laboratory and/or CLP data package will include all raw data and log book documentation sufficient to perform a Stage 4 validation, manually performed (S4VE/S4VEM) data validation as defined in EPA's 2009 Guidance for Labeling Externally Validated Laboratory Data for Superfund Use (EPA-540-R-08-005, EPA 2009b). These data packages will be archived in the FRC according to the required retention schedule. Final asbestos data and supporting documentation will be provided to EPA and maintained by the project manager.

A8 – Special Training and Certification

The field staff conducting sampling for this study have completed at minimum the 40-hour training in Basic Health and Safety (HAZWOPER), completed respirator fit testing, and are enrolled in medical monitoring. In addition, they have completed an 8-hour health and safety training refresher course within the last year and at least 3 members of the team will also have training in first aid and CPR. Field support staff do not need to meet these training and health and safety requirements.

The laboratory performing the analysis of air samples by TEM will be accredited by the National Voluntary Laboratory Accreditation Program (NVLAP) as sponsored by the National Institute of Standards and Technology (NIST) or equivalent accreditation in accordance with the EPA FEM Laboratory Competency Policy (2009) such as The NELAC Institute (TNI) or the Superfund Contract Laboratory Program (CLP). Additional requirements for the analytical laboratories are specifically described in the Statements of Work (SOWs) for Soil and Air Analysis for Asbestos.

A9 – Documents and Records

It will be the responsibility of the Project Manager to ensure that appropriate project personnel have the most current approved version of the QAPP including updates. The final version of the QAPP and any updates will be distributed in a signed portable document file (PDF) format.

Field documentation may include, but is not limited to, field notes and field sheets, photographs, monitoring and locational data, and sample collection data, and Scribe chain-of-custody forms. Laboratory documentation may include, but is not limited to, raw data, sample preparation and analysis logs, and

results of calibration and quality control (QC) checks. EPA's Scribe software will be used for project sample management.

The field and laboratory documentation will be kept in a case file and submitted to ORD with the final report. The following documents will be archived at the contracted asbestos laboratory: (1) signed hard copies of sampling and chain-of-custody records (2) electronic and hard copies of analytical data. Documentation that is required for validation must be maintained as described in the attached SOW (Appendix F). The laboratory will store all sample receipt, sample login, and laboratory instrument documentation for a minimum of seven years.

B. Data Generation and Acquisition

The elements in Sections B1-B10 are designed to ensure that appropriate methods for sampling, measurement and analysis, data collection, data handling, and quality control (QC) activities are employed and documented:

B1 – Sampling Process Design (Experimental Design)

The types of samples collected during this project will consist of soil samples collected as discrete grabs or as ISM samples and personal air monitoring samples collected during the activity based sampling that will be conducted on properties along Swift Creek and the Sumas River. The measurement parameter of interest is asbestos (primarily chrysotile and actinolite). The proposed activities and sampling locations include the following:

Activity	Study Location	Number of Samples	Duration
Soil Sampling, Raking, Personal Air Sampling	ABS will be performed at each of 5 sampling locations	3 personal	180 minutes
Soil Sampling	Incremental Samples will be collected in triplicate at each of 5 sampling locations	15	NA
	5 Discrete/Grab Samples will be collected at each of 5 locations	25	NA
Ambient Air Monitoring	4 samples will be collected daily during field activities. 2 downwind, 1 upwind, and 1 at the command post	16	8 hours

B2 – Sampling Methods

The collection of air monitoring samples for this project will be done in accordance with applicable EPA standard operating procedures (SOP's) and the sections of appropriate analytical methods that address sample collection. Specifically air sampling will be conducted using the EPA - Emergency Response Team SOPs #2015 (Asbestos Air Sampling) and #2084 (Activity-Based Air Sampling for Asbestos) and the relevant sampling guidance provided in analytical methods ISO 10312 and NIOSH 7400 (modified). Additional specific information related to the collection of samples for this project is included in the sampling and analysis plan (SAP).

<u>Personal and Stationary Air Monitors</u>

Remove the top cover of a sample cassette and attach the cassette with flexible tubing to the sampling pump. For personal air monitoring, fasten the sample cassette to the worker's lapel and orient the face of the cassette in a downward direction. Stationary monitoring should be done using a rigid stand that allows the cassette to be oriented downward approximately 5 feet above the surface of the ground.

Prior to sampling the pump should be calibrated according to standard operating procedures. The flow rate of the air sampling pump should be adjusted to 0.5 liters per minute (L/min) or greater when collecting air samples for analysis. The maximum flow rate for personal monitoring air sampling pumps is generally 2 - 4 L/min. The maximum flow rate for stationary sampling pumps is generally 5 - 6 L/min.

Adjust the sampling flow rate Q (L/min), and time, t (min) to produce a fiber density, E, of 100 to 1,300 fibers per square millimeter (mm^2) derived from [3.85×10^4 to 5×10^5 fibers per 25-mm filter with a collection area, $A_c = 385\ mm^2$] for optimum accuracy. These variables are related to the action level (one-half the standard), L (fibers/cc), of the fibrous aerosol being sampled by:

$$t = \frac{(A_c)\ (E)}{(Q)\ (L)\ (10^3)}$$

At the end of sampling, replace the top cover and end plugs of the sample cassette. Samples should be shipped and stored in such a manner as to avoid electrostatic forces which may cause fiber loss from the sample filter. Filters will be placed inside a cardboard box with non-static material such as bubble wrap for cushioning.

Incremental sample collection - Where possible, incremental samples will be collected consistent with multi-increment sampling techniques described in the ITRC Tech Reg (ITRC date). Ideally, the location to be sampled will be roughly 100 by 120 feet and will be divided into 30 grids measuring 20 feet on each side. There will be a minimum of 30 increments collected to make up each individual ISM sample. The specimens will be collected using an incremental sampling tool in order to ensure each increment is represented equally in the final sample volume. Each sample forming part of the triplicate for each location will be collected with a unique tool. The tools will be thoroughly washed and rinsed between locations. Subsampling will be conducted in the field in order to split the sample into representative portions for each of the analyses to be performed. One portion will be placed into a clean HDPE container for analysis of asbestos by PLM. A second portion will be placed into a clean HDPE container for analysis of asbestos by ASTM D7521-13. About 10 grams will be placed into a clean glass jar for analysis of metals and this whole quantity will be digested; there will be no residual. A duplicate will be collected for each ISM metals sample as a backup in case there are problems with the initial sample. A final portion of material will be placed into a 250 ml HDPE container for fluidized bed preparation for asbestos TEM analysis.

Grab samples – Grab soil samples will be collected from 5 discrete locations on each property using new/clean stainless steel spoons from the top 2 inches below the surface of the ground. At each location, rough homogenization in the field will be done prior to placing soil into sampling containers for each type of analysis as described above. The same size containers as used for ISM samples will be used for discrete samples to be sent for asbestos analysis. For metals analysis, an entire 4 oz. glass jar will be filled with soil and sent to the lab. The asbestos samples will be analyzed the same as the ISM soil samples described

above. Metals analysis will proceed in a routine fashion where only a small amount of the total sample is used for analysis.

Moisture Content (Soil / Rock) - Moisture content (i.e., percent solids) will be determined as part of the inorganics analysis from ISM and discrete samples. An aliquot of soil will be placed into a 2-oz. jar to determine moisture content. The jar for moisture content should be full.

GPS Locational Information – GPS waypoints will be collected on each of four corners for the ISM sampling areas in addition to the individual soil grab and stationary air monitoring locations. Waypoints will be averaged over a 3-minute timeframe and consist of latitude and longitude in decimal degree format in the WGS84 datum. A GPS field worksheet will be used to record the location ID and/or description, date, time, latitude and longitude. For the ISM areas, a geographic centroid will be calculated and entered into Scribe. All waypoints will be entered into Scribe for the grab and stationary air monitoring locations.

Meteorological Monitoring - Data on meteorological conditions during the activity based sampling will be collected with a Coastal Environmental Systems WeatherPak® 2000 portable weather station equipped with an ultrasonic wind sensor. Data will be collected on wind direction and velocity, relative humidity, and air temperature. Monitoring will be done according to the instructions provided in the WeatherPak® 2000 user manual. The WeatherPak® 2000 will be sited in an exposed location free of obstructions and disturbing metal or other magnetic materials.

B3 – Sample Handling and Custody

Each sample will be identified with a unique sample number, project code, and container ID code assigned by the RSCC. EPA Region 10 chain-of-custody procedures and Scribe will be used (EPA DMP 2014). Custody seals will be placed on all sample containers during transit to the laboratory (2 external/1 internal when shipping samples vs hand delivery). Samples for analysis by TEM will be hand-delivered or shipped to the contract Laboratory.

B4 – Analytical Methods

Air

Personal and Stationary Air Monitors
A sub-set of samples will be screened with a phase contrast microscope (PCM) by method NIOSH 7400 (modified). The screening is intended to determine the degree of filter loading during ABS. If overloaded filters are detected by PCM, the sample flow rate and duration can be altered to help avoid overloading of filters intended for costly TEM analysis.

Most of the air samples will be analyzed by TEM by method ISO 10312 for determination of asbestos fibers by direct transfer transmission electron microscopy. For TEM analysis, the microscopist will record the size (length and width) and the type (chrysotile, amphibole, or non-asbestos) of the fiber. The counting rules are specified in the analytical method. The analytical sensitivity and detection limit is a function of the volume of air drawn through the filter and the number of grid openings counted. The target analytical

sensitivity for activity-based air samples is 0.001 s/cc. The target analytical sensitivity for stationary air samples for this project is 0.0001 s/cc. If samples are overloaded, then they may be analyzed using an indirect method (ISO 13794). Note that EPA requires that samples having less than 4 structures have concentrations reported as the number of fibers observed multiplied by the analytical sensitivity.

Soil

Sample Preparation

The asbestos soil samples to be analyzed by CARB 435 will be crushed to produce material with a nominal size less than 8 centimeters (cm). Before crushing the sample will be dried in a lab oven at a sufficient temperature that will not alter the optical properties of chrysotile asbestos (60 - 105° C for 2-6 hours). ASTM method C-702-80 will be used to reduce the size of the crushed grab sample to a one-pint aliquot. The one-pint aliquot will be further crushed using a BICO mill or equivalent to produce a material which is less than 200 Tyler mesh (0.074 millimeters). For samples analyzed using ASTM D7521, sieving will occur at the laboratory, so no preparation is needed beyond what is required in the method.

The soil samples that will be run through the fluidized bed will be prepared as described in the SOP (see Appendix B).

The metals soil samples will be prepared according to the laboratory SOP and the analytical method specified in Table 2. A 10-gram aliquot will be generated in the field. Include reference to the SAP section that discusses this.

Analytical Requirements

The soil samples will be analyzed for asbestos by stereo and polarized light microscopy (PLM) using the California Air Resources Board (CARB) method 435. Samples will be analyzed for asbestos by PLM using 400 point counts per CARB method 435. The analyst should report results as trace (<0.25%) when asbestos does not occupy any of the points counted, but is observed in the field of view. Samples also will be analyzed using ASTM D7521 which uses a combination of PLM and TEM. Results are reported down to about 0.1% asbestos. Soil samples also will be prepared using the FBAS for subsequent analysis by ISO 10312. The soil samples also will be analyzed for metals by EPA methods 200.2/200.7 (or current SOW).

Moisture Content (Soil)

Moisture content of the soils will be analyzed in accordance with the metals analytical method requirements for moisture correction to dry-weight for laboratory soil results.

B5 – Quality Control

The following QC activities will be performed by the laboratory performing analytical services in support of this project.

Air Samples Analysis by TEM and Screening by PCM

Blank and QC Determinations - A minimum of two unused filters from each filter lot used for this

project will be analyzed to determine the mean asbestos structure count. If the mean count of all types of asbestos structures is found to be more than 10 structures per square millimeter (s/mm^2) or if the mean count for asbestos fibers and/or bundles longer than 5 microns (µm) is greater than 0.1 s/mm^2, the filter lot should be rejected. Two filter lot blanks will be analyzed both for air and dust samples.

The analysis of blanks shall be performed in such a manner as to achieve equivalent analytical sensitivity (number of grid openings counted) as to be comparable to those of the sample set.

Negative Controls - A minimum of two laboratory QA blank filters will be prepared and analyzed by TEM for this project. The laboratory blank filters shall be left uncovered during preparation of the sample sets and wedges from the blank filters shall be prepared alongside the wedges of sample filters.

Precision and Accuracy - For TEM analysis, the precision and accuracy should be measured according to the procedures in Section 10.3 of method ISO 10312. Screening samples by PCM will be done primarily to determine the degree of filter loading with particulates in order to help avoid overloading of filters that require in-direct preparation for TEM analysis.

Samples Analyzed by PLM

Negative Controls – A blank slide must be prepared before analysis of each set of samples. A sample of isotropic non-asbestos material such as fiberglass (standard reference material (SRM) 1866a) should be mounted in a drop of refractive index liquid on a clean slide. Preparation tools including forceps and dissecting needle should be rubbed in the drop of liquid and a clean cover slip should be placed on the drop. The entire area under the cover slip should be scanned by PLM to detect asbestos fiber contamination. If asbestos fibers are detected, the test should be repeated using a clean slide and cleaned preparation tools. If asbestos fibers are still found, have the refractive index fluid checked and replace if needed.

Duplicate Analysis (intra- & inter-analyst precision) – The analyst will perform 2 independent measurements at a frequency of 1 out of every 10 samples to determine the ability of the analyst to repeat a measurement. At least 1 out of every 10 samples should be re-analyzed by another analyst. In addition, the lab will send up to 3 prepared samples for PLM analysis to EPA Region 10's MEL for interlaboratory verification and comparison.

Reference Sample Analysis – The analyst will perform analysis of a material with a known concentration of asbestos from SRM provided by the National Institute of Standards and Technology (NIST) SRM 1866b for common commercial asbestos such as chrysotile and SRM 1867a for uncommon commercial asbestos such as actinolite.

Samples Analyzed by TEM

The quality control checks required for this method are described in the Statements of Work (SOWs) included in Appendix F.

Samples Analyzed for Metals

Standard required lab QC for metals analysis will be performed in accordance with the analytical method and includes:

PROCEDURAL BLANK
One Procedural Blank (BL) is required for each analytical batch. This blank consists of the reagents used in sample preparation, taken through the entire sample preparation and analysis procedure. The purpose of this blank is to determine the extent of contamination introduced by sample preparation and analysis. The absolute value of the procedural blank shall be < MRL.

MATRIX SPIKES
One set of Matrix Spike/Matrix Spike Duplicate samples is required for each analytical batch of 20 samples, provided that there is adequate sample available. A matrix spike is a sample to which a spike solution of a known concentration of metals is added prior to digestion. Percent recoveries must be within 75 - 125% of the true value of the spike. If the concentration of the native sample is greater than 4 times the matrix spike amount, then spike recovery limits do not apply

DUPLICATE
One Duplicate sample is required for each analytical batch, provided there is adequate sample available. The duplicate sample is prepared in the same manner as the native sample, and is used to assess the reproducibility of the sample preparation procedure. For results greater than five times the MRL, the Relative Percent Difference (RPD) between the native sample and the duplicate sample shall be ≤20%. A Laboratory Control Sample (LCS) is required for each analytical batch. This standard is from a separate source from the calibration standards. This sample is carried through the entire digestion procedure. The percent recoveries must be within 80 - 120%.

B6 – Instrument/Equipment Testing, Inspection, and Maintenance

Field Instruments

The personal air monitors and Gilian soap-bubble calibration standard to be used for this project are serviced annually (most recent service August 15, 2013). The equipment service records shall be maintained by the Manchester Environmental Laboratory.

The PCM used for screening ABS samples will be maintained according to the manufacturers' instructions and the laboratory SOP's. Records for equipment service shall be maintained by the Manchester Environmental Laboratory.

The WeatherPak® 2000 was serviced on November 11, 2013.

The Garmin Montana with Camera does not require regular servicing.

B7 – Instrument/Equipment Calibration and Frequency

Field Instruments

The Gilian soap-bubble calibration standard to be used for this project was serviced and verified accurate on August 15, 2013). Certification of the calibration standard shall be maintained by the Manchester Environmental Laboratory.

The Weatherpak 2000 was calibrated on November 11, 2013.

The PCM used for screening activity based monitoring samples will be calibrated according to the Manchester Environmental Laboratory SOP (ASB-002) Standard Operating Procedure for Analysis of Asbestos and Other Fibers by Phase Contrast Microscopy. Records for calibration shall be maintained by the Manchester Environmental Laboratory. Other laboratory equipment such as drying ovens and balances will be calibrated according to the manufacturers' instructions and laboratory SOP's.

Laboratory Instruments

Laboratory equipment will be calibrated using the method and frequency specified in the laboratory SOP's and the analytical method. Records on calibration of laboratory equipment shall be maintained by the laboratory and be made available to EPA upon request.

B8 – Inspection/Acceptance of Consumable Supplies

Consumable supplies in the field will consist primarily of air monitoring filter cassettes, glass and/or plastic jars, ziplock bags, drum liner bags, labels, paper, and PPE. Consumable supplies in the laboratory will consist of reagents, acid, and standards. The quality of standards and other consumable supplies used for this project should be documented by the supplier and certificates included in the raw data package to be made available to EPA on request. EPA Region 10 field staff use only QC class sample containers.

B9 – Non-Direct Measurements

Non-direct measurements may be acquired through review of previous sampling and analysis activities conducted by EPA Region 10, the State of Washington, Northwest Washington Air Pollution Control Authority, contractors, or researchers currently or previously involved with analysis of samples from this site. These types of data may be used for planning purposes for this project. The data acquired through non-direct measurement should be reviewed by the EPA QA office to ensure it meets minimum standards and acceptance criteria.

B10 – Data Management

All data management will be completed in accordance with the R10 Data Management Plan (2014). This includes all field sample collection, locational, monitoring, custody, and final lab result data. A field log notebook, photos, and the Field Sampling sheets and Scribe will be used to document the sampling activities. The Field Samples will have at a minimum the following information: R10 sample number,

container ID, project code, station location, sample type (ISM, Discrete, or ABS), date and time of each sample collection, and the sampler's name or initials.

All data generated during this project will be processed, stored, and distributed according to laboratory's SOPs and EPA's SOWs for asbestos analysis (see Appendix F).

C. Assessment and Oversight

C1 – Assessments and Response Actions

Quality assurance (QA) assessments may be conducted during the course of this project. Given the short time frame, only one assessment may occur before completion of the project. The quality assurance assessment performed during this project may include the following:

1) Oversight of field sampling activities.
2) Oversight of sample handling and chain-of-custody procedures.
3) Laboratory Inspections.

Quality assurance assessments may be conducted by the EPA Region 10 quality assurance manager or QA staff delegated by the manager to conduct assessments. QA staff participating in the field sampling will not be performing QA oversight.

Laboratories routinely perform performance checks using different program specific blind and double blind check PE standards. Each asbestos method of analysis requires specific QA/QC runs that must be complied with by the laboratory performing the analysis. An internal assessment of the data and results are also routinely conducted by the appropriate supervisors and the Laboratory QA Coordinator. No additional audits will be performed on the laboratory for this project.

Corrective action procedures that might be implemented from QA results or detection of unacceptable data will be developed if required and documented in Attachment 2.

C2 – Reports to Management

If, for any reason, the schedules or procedures above cannot be followed, the project manager shall complete Attachment 2 – Sample Alteration Form (SAF). The SAF should be reviewed and approved by the QAO. The laboratory should be given a copy of the QAO approved SAF for reference and project file.

D. Data Validation and Usability

D1 – Data Review, Verification, and Validation

<u>Data Verification</u>

Data verification is a consistent and systematic process that determines whether the data have been collected in accordance with the QAPP.

Data Validation

Data validation is an evaluation of the technical usability of the verified data with respect to the planned objectives of the project. Data validation is performed external to the laboratory by applying a defined set of performance criteria to the body of data in the evaluation process. For MEL, data will be 100% verified as equivalent to a S4VM (EPA 2009b).

D2 – Verification and Validation Methods

Data Verification

Data verification will include a review of the findings of all QA assessment activities including:

1) Field Collection Procedures
2) Sample Labeling Methods.
3) Chain-of-Custody Procedures.
4) Assessments of analytical data collection, recording, and reporting.

If any deviations are identified, the potential impact of those deviations on the reliability of the data will be assessed, and the information will be provided to the project manager.

Data Validation

Data validation consists of evaluation of all individual samples collected and analyzed to determine if the results are within acceptable limits. Quantitative or qualitative limits of acceptability are defined for precision, accuracy, representativeness, comparability, and completeness. Project specific criteria for these Data Quality Indicators are provided in Table 2. For the total metals analysis, data validation will be consistent with either Region 10 Manchester internal data review procedures or the National Functional Guidelines for Inorganic Superfund Data Review. For asbestos, a minimum of Stage 2b data review will be performed by Julie Wroble or another EPA staff member, to be determined.

1) Precision: It is defined as the agreement between a set of replicate measurements without assumption and knowledge of the true value. Agreement is expressed as either the relative percent difference (RPD) for duplicate measurements or the range and standard deviation for larger numbers of replicates. Data on precision are obtained by analyzing duplicate and replicate samples.

2) Accuracy: Accuracy is a measure of the closeness of a sample analysis result to the “true” value. Accuracy will be determined primarily by an evaluation of the agreement between repeat analyses, both within and between laboratories.

3) Representativeness: Is defined as the degree to which data accurately and precisely represents characteristics of a population, parameter variations at a sampling point, or an environmental condition. For this project, representativeness will be ensured by selection of sampling locations in accordance with the sampling design requirements in this QAPP.

4) Comparability: Data are comparable if collection techniques, measurement procedures, methods, and reporting units are equivalent for the samples within a sample set. Comparable data for this project will be obtained by specifying standard units for physical measurements and standard procedures for sample collection, processing and analysis.

5) Completeness: Data are complete when a prescribed percentage of the total intended measurements and samples are obtained. Analytical completeness is defined as the percentage of valid analytical results requested. For this project, acceptable completeness should be > 90%.

6) Sensitivity: The capability of a method or instrument to discriminate between measurement responses representing different levels of the variable of interest (see Table 2).

D3 – Reconciliation with User Requirements

Data that have passed validation will be collated into a data report package. Further evaluation of the validated data will be performed in order to determine if the level of sensitivity for each respective method has been achieved. Upon completion, data determined to be suitable for use will be compiled as part of the final report. Data excluded from the report for either validation or suitability reasons will be documented in a data exclusion section.

Table 2 – Analytical - Data Quality Objectives Summary

Analytical Group	Number of Samples	# of QA Samples: Reference Samples	Matrix	Method	Method Detection Limits	Accuracy	Precision (RPD)	Complete-Ness	Volume, Container	Holding Time (days)
					**Laboratory Measurements**					
Asbestos TEM Personal	15	2	Air Filter	ISO 10312	3.0 structures/liter	N/A	+/- 25%	> 90%	Filter Cassette	None
Asbestos TEM Stationary	12	2	Air Filter	ISO 10312	0.3 structures/liter	N/A	+/- 25%	> 90%	Filter Cassette	None
Asbestos PLM	40	5	Soil	CARB 435	0.25% plus field of view	Variable	Variable	> 90%	16 oz. HDPE bottle	None
Asbestos PLM/TEM	40	5	Soil	ASTM 7521-13	0.1%	Variable	Variable	>90%	16 oz. HDPE bottle	None
Asbestos TEM	40	5	Soil (fluidized bed)	ISO 10312	0.0001 f/cc	Variable	Variable	>90%	250 ml HDPE jar	None
Discrete Metals (not including Hg)	25	3	Soil	EPA 200.2/200.7	Per MEL MRL or CLP CRQL	+/- 25%	+/- 20%	>90%	4 oz glass jar	180 days
ISM Metals (not including Hg)	15	2	Soil	EPA 200.2/200.7	Per MEL MRL or CLP CRQL	+/- 25%	+/- 20%	>90%	2 oz glass jar	180 days
Percent Solids	40	5	Soil	Per MEL	Per MEL MRL or CLP CRQL	+/- 25%	+/- 20%	>90%	2 oz glass jar	180 days
Fiber Count by PCM (screening)	15	2	Air Filter	NIOSH 7400	< 0.1 fibers/cc	N/A	N/A	> 90%	Filter Cassette	None

Table 3 – Field Measurement - Data Quality Objectives Summary

Meteorological Conditions	Instrument	Range	Resolution	Accuracy	Sample Duration
Wind Speed	Gill Ultrasonic Wind Sensor	0-60 meters/second	0.01 meters/second	+/- 2%	The weather monitoring will be conducted between 0800 and 1800 daily during the activity based monitoring
Wind Direction	Gill Ultrasonic Wind Sensor	0-360°	1°	+/- 3°	Same
Relative Humidity	Vaisala Humitter	0-100% RH	Not applicable	+/- 3% at 20° C	Same
Temperature	WeatherPak 2000	-50 ° to + 50 ° C	Not applicable	+/- 0.2 ° C	Same

Attachment 1 – Sample Alteration Form
(QAPP Addendum – SPAF # NN)

QAPP Title, Author (company), Revision, and Approval Date of standing 'parent' QAPP:

Project Name and assigned Region 10 Project Code:

Material to be Sampled:

Measurement Parameters:

Standard Procedure for Field Collection and Laboratory Analysis (cite references):

Reason for Change in Field Procedure or Analytical Variation:

Variation from Field or Analytical Procedure (reference specific QAPP sections):

Special Equipment, Materials, or Personnel Required:

CONTACT	APPROVAL SIGNATURE	DATE
**Initiator:**<br>First and Last Name, Title		
**Other Approval:**<br>First and Last Name, Title		
**EPA Project Manager:**<br>First and Last Name, Title		
**EPA QA Manager:**<br>First and Last Name, Title		

Attachment 2 – Corrective Action Form

QAPP Title, Author (company), Revision, and Approval Date of standing 'parent' QAPP:

Project Name and assigned Region 10 Project Code:

Sample Dates Involved:

Measurement Parameters:

Acceptable Data Range:

Problem Areas Requiring Corrective Action:

Measures Required to Correct Problem:

Means of Detecting Problems and Verifying Correction:

CONTACT	APPROVAL SIGNATURE	DATE
**Initiator:**<br>First and Last Name, Title		
**Contractor Project Manager:**<br>First and Last Name, Title		
**EPA Project Manager:**<br>First and Last Name, Title		
**EPA QA Manager:**<br>First and Last Name		

References:

Interstate Technology Regulatory Council (ITRC), February 2012, Incremental Sampling Methodology, Technical and Regulatory Guidance, http://www.itrcweb.org/ism-1/pdfs/ISM-1_021512_Final.pdf.

Januch, J., et al. (2013). "Evaluation of a fluidized bed asbestos segregator preparation method for the analysis of low-levels of asbestos in soil and other solid media." Analytical Methods **5**(7): 1658-1668.

Smith, I., K. Hall, L.M. Lavkulich, and H. Schreier. **2007**. Trace metal concentrations in an intensively used agricultural watershed in B.C. Journ. American Water Res. Assoc. Vol 43(6) 1455-1467.

U.S. EPA Region 10, October 13, 2009a, Soil, Sediment and Surface Water Sampling, Sumas Mountain Naturally-Occurring Asbestos Site, Whatcom County, Washington, prepared by the Office of Environmental Assessment, Seattle, Washington, http://www.epa.gov/region10/pdf/sites/sumasmountain/soil_sediment_surfacewater_sampling_finalreport_sumas_2009.pdf.

U.S. EPA, January 2009b, Guidance for Labeling Externally Validated Laboratory Analytical Data for Superfund Use, OSWER No. 9200.1-85, EPA 540-R-08-005.

U.S. EPA Region 10, April 2011, Environmental Monitoring for Asbestos: Sumas Mountain Asbestos Site Selected Residential Properties, Bulk Sampling and Analysis Activity Based Sampling Surface Water Sampling, Whatcom County, Washington, prepared by the Office of Environmental Assessment, http://www.epa.gov/region10/pdf/sites/sumasmountain/asbestos_monitoring_report_april2011.pdf.

U.S. EPA Region 10, November 19, 2013, Soil Sampling Report, Sumas Mountain Asbestos Site, Whatcom County, Washington, Office of Environmental Assessment, Seattle, Washington.

SAMPLE PLAN ALTERATION FORM
(QAPP Addendum – SPAF # 01)

QAPP Title, Author (company), Revision, and Approval Date of standing 'parent' QAPP:

Quality Assurance Project Plan for Regional Methods Project: Comparison of Soil Sampling Methods for Asbestos at the Sumas Mountain Asbestos Site, Whatcom County, Washington
Approval Date – 9/25/2014

Project Name and assigned Region 10 Project Code:

Sumas Mtn Asbestos Regional Methods Study, SFP-078A

Material to be Sampled:

Cobbles from Driveway at Oat Coles/South Pass Road, Everson, WA

Measurement Parameters:

Asbestos by XRD, SEM, PLM

Standard Procedure for Field Collection and Laboratory Analysis (cite references):

Method XRD-QL for Compound Identification by X-ray Diffraction Analysis (U.S. EPA Region 10 Laboratory)
ASB_004 Standard Operating Procedure for Asbestos Analysis by SEM/EDS
EPA 600/R-93/116 – Method for Determination of Asbestos in Bulk Building Materials

Reason for Change in Field Procedure or Analytical Variation:

This material was identified as a potential confounder during the field event. EPA wants to determine the type of asbestos, if any, present in the cobbles on the driveway at the property where samples were collected.

Variation from Field or Analytical Procedure (reference specific QAPP sections):

Special Equipment, Materials, or Personnel Required:

Jed Januch / MEL can do these analytical methods.

CONTACT	APPROVAL SIGNATURE	DATE
**Initiator:** Julie Wroble, Regional Methods Project Manager	[signature]	10/9/14
**EPA REU Manager:** Sheila Fleming	[signature]	10/9/2014
**EPA Project Manager:** Julie Wroble, Regional Methods Project Manager	[signature]	10/9/14
**EPA QA Manager:** Ginna Grepo-Grove	[signature]	10/9/2014

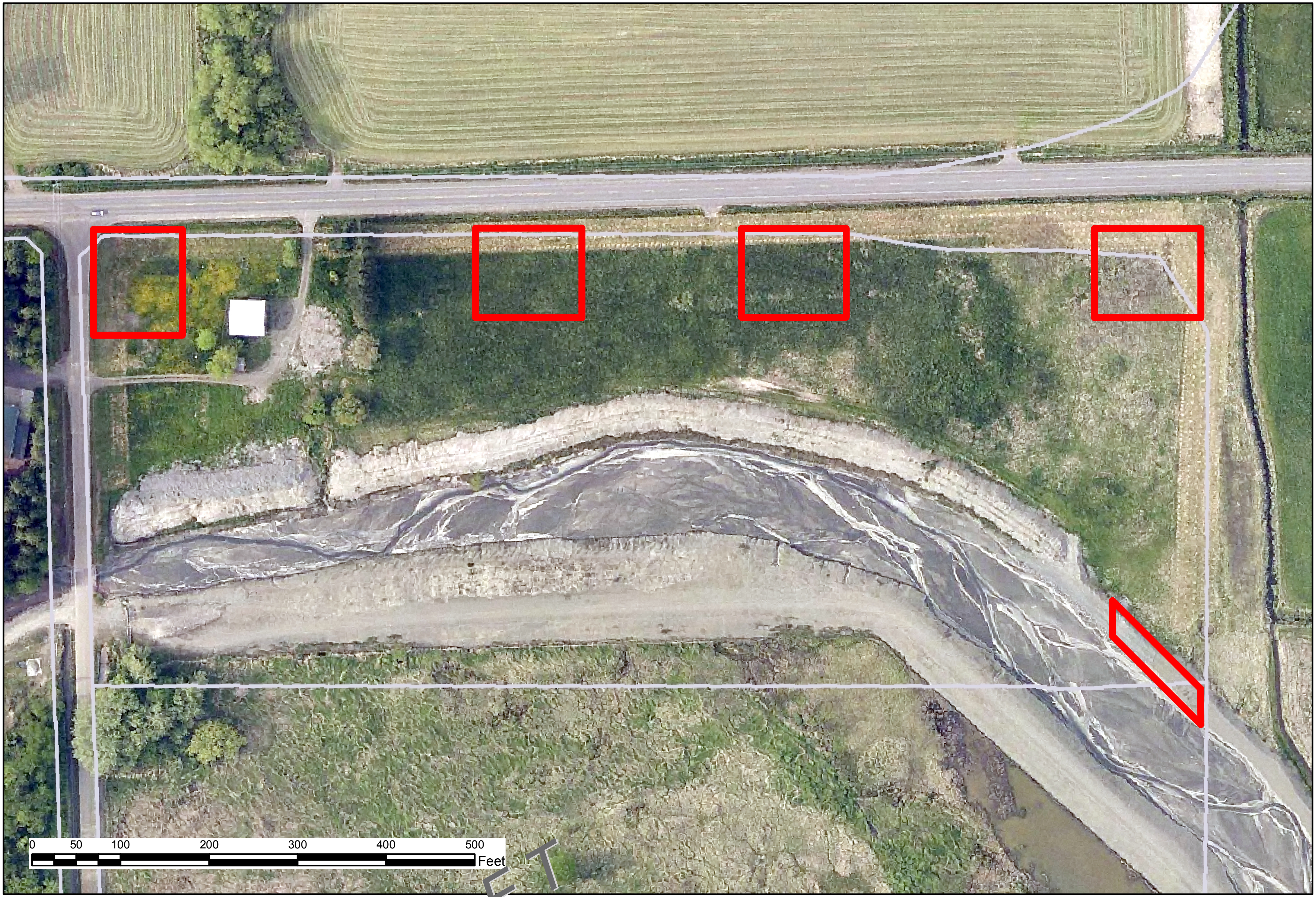

Figure 1
Proposed Sampling Locations,
Oat Coles/South Pass Road
Sumas Mountain Asbestos Site

EPA Region 10 GIS Team

Image Data from Whatcom County Administrative Services
Image Date 2013, pixel resolutinon 9"
Map Created 09/25/2014

To see all the details that are visible on the screen, use the "Print" link next to the map.

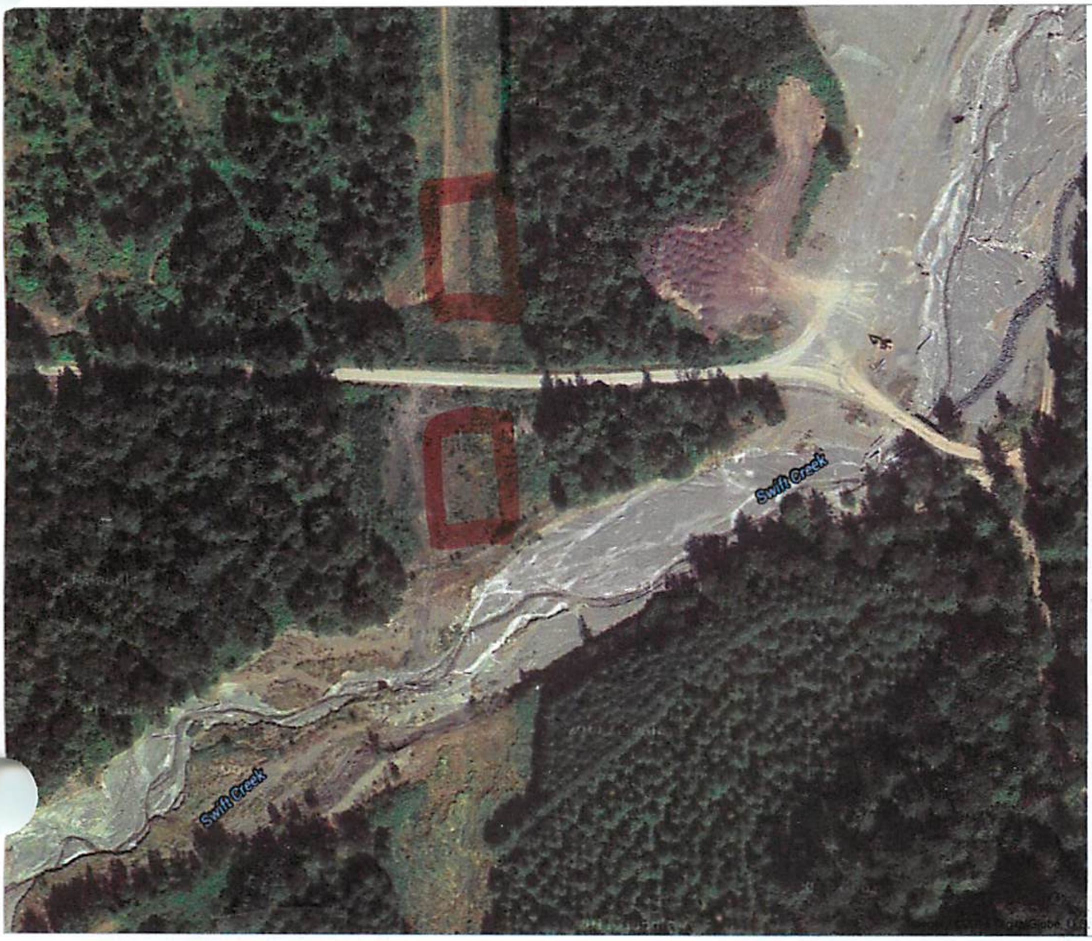

Geological Survey, Map data ©2014 Google -

Figure 2
Proposed Sampling Locations
Williams Pipeline Right of Way
Sumas Mountain Asbestos Site

APPENDIX A

SAMPLING AND ANALYSIS PLAN

Appendix A
Sampling and Analysis Plan
Comparison of Soil Sampling Methods
Sumas Mountain Asbestos Site

Project Background

An earth flow type of landslide on the west side of the Sumas Mountain initiated around 1940 resulted in significant deposition of sediments into Swift Creek during periods of rain and snow melt. Sampling and analysis conducted by EPA over the past several years has confirmed the presence of chrysotile and actinolite asbestos in dredged materials from Swift Creek, material taken from the dredge piles and placed elsewhere, Swift Creek and Sumas River bank sediments, Swift Creek and Sumas River surface water, and upland areas where flood events deposited sediments. The concentration of asbestos in dredged materials, as measured by PLM, ranged from 0.1 % to 4.4%, with an average concentration of 1.6%. The concentration of asbestos in sediments and upland flood deposits, as measured by PLM, ranged from 0.5 % to 27%. EPA also conducted activity-based sampling (ABS) in 2006 to determine whether disturbance of dredged materials would result in breathing zone concentrations of asbestos that potentially pose a risk to human health. The results of this testing indicated that breathing zone concentrations of asbestos during soil disturbing activities resulted in risks at the high end of the range that EPA generally considers to be acceptable. Recognizing these risk levels and because other exposures in the community are possible due to historical use of the dredge materials as fill in the community, EPA restricted dredging of Swift Creek sediments. Appropriate disposal of these sediments is not feasible because the costs associated with transport are extraordinarily high. Flooding in 2009 resulted in deposition of sediments onto upland areas at several locations along Swift Creek and the Sumas River. Sampling of these deposits indicated that concentrations were higher than what was found in the dredged materials along Swift Creek and follow up activity-based sampling indicated that exposures to individuals who disturb these materials could result in elevated risks (EPA 2009, 2011).

Incremental and Grab Sampling of Soils, Activity Based Sampling

The Comparison of Soil Sampling Methods for Asbestos at the Sumas Mountain Asbestos Site Quality Assurance Project Plan describes the rationale for conducting the sampling activities. Briefly, the purpose of this project is to compare a variety of soil sampling and analysis techniques for asbestos. In addition, activity-based sampling (ABS) and metals analysis in these same areas will provide additional data for comparison.

Because we are trying to compare methods across several locations, the sampling will be conducted in the same manner as much as possible at each location. One difference will be meteorological conditions in the day of sampling. The hope is that weather conditions are sufficiently similar across sampling days so that this is not a big factor. However, for ABS changes in relative humidity, wind speed and direction, and other meteorological factors can impact results. The activity that workers doing ABS will perform is clearing vegetation in the

area of sampling, soil sampling (both ISM and discrete sampling), and if needed, a more generic raking activity across the sampling location. Weed trimming and mowing may be needed to remove thick vegetation in some areas. If vegetation prevents easy collection of a soil sample, then weed trimming may be performed at the location prior to performing soil sampling. The decision about whether weed trimming will be done at each location will be made at each location on the day of sampling.

The following activities will be conducted for this project:

Activity 1 (ABS 1) – Collecting ISM samples at location, subsampling soils
Activity 2 (ABS 2) – Collecting discrete samples at location
Activity 3 (ABS 3) - Raking activity
Activity 4 (ABS 4) – Weed Trimming at a location (optional)

Field Team

The following is a list of EPA staff working as a field team for this project and the functions they will perform.

OEA Staff	Function	Phone Number
Julie Wroble	Project Manager	(206) 553-1079
Jed Januch	Equip. Calibration/Analysis	(360) 871-8731
Raymond Wu	ABS	(206) 553-1413
Jennifer Crawford	RSCC, ABS	(206) 553-6261
Don Matheny	Scribe	(206) 553-2599
Doc Thompson	Field Support	(360) 871-8721
Grady Maxwell	Site Safety Officer	(206) 553-0241

Other EPA Staff	Function	Phone Number
Dan Vallero*	Contracting Officer Representative	(919) 541-3306
Tim Frederick	ISM Expert	(404) 562-8958
Sandra Brozusky	ABS	(206) 553-5317

*-Will not participate in field activities, but is a critical member of project team.

Proposed Schedule of Activities

**Activity**	**Estimated Start Date**	**Estimated Completion Date**	**Comments**
Asbestos Site-Specific QAPP Review/Approval	9/15/14	9/26/14	
Sample Collection	9/29/14	10/2/14	9/29/14 travel and pick up equipment<br>10/3/14 equipment decontamination and travel
Laboratory Receipt of Samples	10/3/14	10/8/14	Samples will be hand-delivered or shipped via commercial delivery service (UPS).
Laboratory Analysis	10/6/14	1/31/15	Lab turnaround may vary.
Data Verification/Validation	2/1/15	3/30/15	

			OEA staff (Januch, Wroble, and MEL Chemists) will do this.

Detailed Itinerary – This is intended as a guideline and may change based on site conditions, final composition of the field team, and other factors.

Monday 9/29 –
Mobilize to the site (all)
Set up study areas and command center (all)
Set up weather station (Januch)
Safety Briefing (all – Wroble, Brozusky lead)
Calibrate stationary and personal monitors (Januch)
Deploy Stationary monitors (Maxwell and Thompson)
Collect discrete and ISM soil samples, Location 1 (all)
Perform ABS raking at Location 1 (all)
Collect GPS coordinates and associated photos for documentation (Matheny)
Scribe Sample Management; prepare samples for shipping (Wroble, Matheny)

Tuesday 9/30 –
Set up weather station (Januch)
Safety Briefing (all)
Calibrate stationary and personal monitors (Januch)
Deploy Stationary monitors (Maxwell and Thompson)
Collect discrete and ISM soil samples, Location 2 (all)
Perform ABS raking at Location 2, 0900 – 1200 (all)
Collect discrete and ISM soil samples, Location 3 (all)
Perform ABS raking at Location 3, 1300 – 1600 (all)
Collect GPS coordinates and associated photos for documentation (Matheny)
Scribe Sample Management; prepare samples for shipping (Wroble, Matheny)

Wednesday 10/1 -
Set up weather station (Januch)
Safety Briefing (all)
Calibrate personal monitors (Januch)
Deploy Stationary monitors (Maxwell and Thompson)
Collect discrete and ISM soil samples, Location 4 (all)
Perform ABS raking at Location 4, 0900 – 1200 (all)
Collect discrete and ISM soil samples, Location 5 (all)
Perform ABS raking at Location 5, 1300 – 1600 (all)
Collect GPS coordinates and associated photos for documentation (Matheny)
Scribe Sample Management; prepare samples for shipping (Wroble, Matheny)

Thursday 10/2 -
Set up weather station (Januch)
Safety Briefing (all - Wroble lead)
Deploy Stationary monitors (Maxwell and Thompson)
Calibrate personal monitors (Januch)
Collect any remaining soil samples (Wu & Crawford)
Perform ABS at any locations not completed earlier in week
Collect GPS coordinates and associated photos for documentation (Matheny)
Scribe Sample Management; prepare samples for shipping (Wroble, Matheny)

Friday 10/3 - Decontaminate equipment (all)
Return rental equipment –water tank, ISM sampling tools (Wroble, Januch)
Deliver samples to MEL (Januch)
Deliver samples to UPS or contract laboratory (Crawford)
Scribe Sample Management; prepare samples for shipping (Wroble, Matheny)
Return to Manchester Lab/home

Weather Forecast

The following weather forecast information is from weather.com:

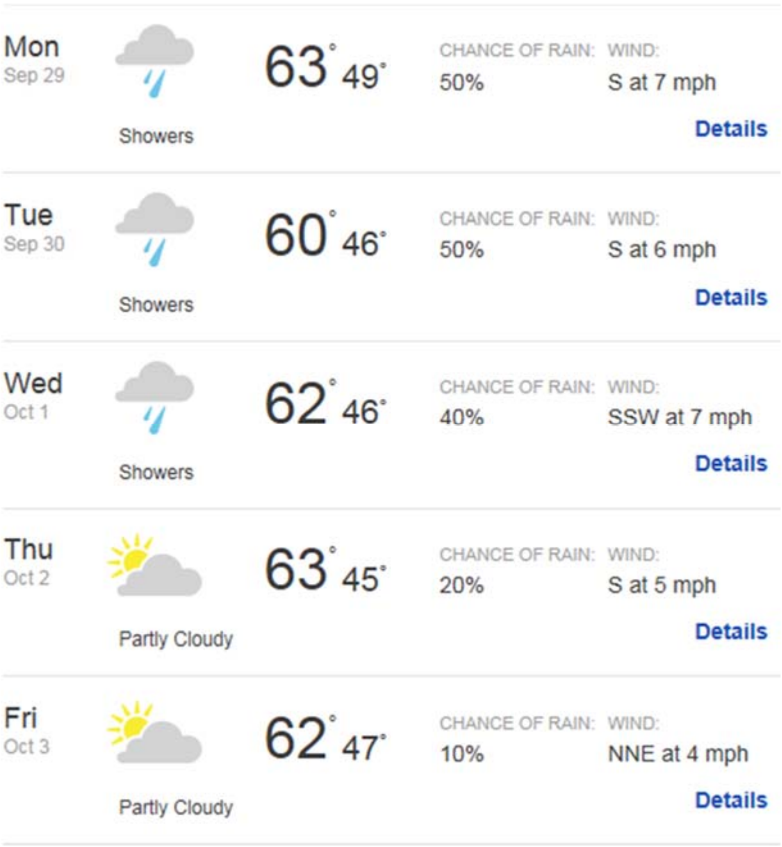

Mon Sep 29	Showers	63° 49°	CHANCE OF RAIN: 50%	WIND: S at 7 mph	Details
Tue Sep 30	Showers	60° 46°	CHANCE OF RAIN: 50%	WIND: S at 6 mph	Details
Wed Oct 1	Showers	62° 46°	CHANCE OF RAIN: 40%	WIND: SSW at 7 mph	Details
Thu Oct 2	Partly Cloudy	63° 45°	CHANCE OF RAIN: 20%	WIND: S at 5 mph	Details
Fri Oct 3	Partly Cloudy	62° 47°	CHANCE OF RAIN: 10%	WIND: NNE at 4 mph	Details

Location

The map in Figure 1 shows the parcel where field activities will be focused. Activity-based sampling and associated soil sampling will be conducted at this parcel. Note that exact sample locations will be determined in the field and reported in the final report on field activities.

Sample Collection

Personal Air Samples- will be collected during all of the activities described in the following sections. The procedure for sampling is specifically described in the EPA standard operating procedures (EPA - Emergency Response Team SOPs #2015 – Asbestos Air Sampling and #2084 – Activity-based Sampling) included in Appendix B. The individuals participating in the activities will wear a sampling pump that will be connected with Tygon® tubing to a sampling

cassette that housed a 0.8-micron (µm), 25-millimeter (mm), mixed cellulose ester (MCE) filter. The top cover from the cowl extension on the sampling cassette shall be removed ("open-face") and the cassette oriented face down within the breathing zone (approximately 6-9 inches around the face). Personal air monitoring samples will be collected at a target flow rate of 2 to 4 liters per minute (L/min) for approximately three hours for a total target volume of >500 liters (L) of air.

During activity-based sampling the participants performing the activities will wear Level C personnel protective equipment (PPE) consisting of Tyvek® coveralls with an elastic band hood and boot covers (optional), latex gloves, and a full-face respirator with high efficiency particle arrestance (HEPA) filter cartridges (P-100 or equivalent). Participants can opt not to wear boot covers if they have rubber boots that can be easily decontaminated. Additional information on PPE requirements and health and safety considerations associated with this project are covered in the health and safety plan (HASP) included in Appendix C.

Workers will wear one of more personal pumps during activities. A utility belt will be used for carrying individual personal sampling pumps. Multiple pumps will be carried inside disposable back packs.

Equipment/Supply Requirements

**Type**	**Description**	**Quantity Required**	**Source**	**Estimated Cost/Rental GOV rate ***
Pump Calibrator	Gilian electronic soap film meter	1	OEA will provide	None
Personal Sampling Pumps	Gilian Hi Volume pumps	6	OEA will provide	None
Stationary Air Sampling Equipment	Allegro High Volume sampling pumps	3	OEA will provide	None
Stationary air monitors	Air metrics	4	OEA will provide	None
Filter Cassettes	Zefon PCM filter cassettes 0.8 µm MCE.	1 case (72 filters)	OEA will provide	None
PPE	Tyvek suits, latex gloves, respirators and P-100 filters	- 30 L Tyvek<br>- 10 XL Tyvek<br>- 10 3XL Tyvek<br>- 3 boxes each of gloves M, L, XL<br>- 4 boxes of P-100 respirator cartridges	OEA will provide	None

For activities that generate a large quantity of dust (i.e., particulates), sample flow rates should be lowered accordingly. For example, sampling pumps for raking should use a flow rate of approximately 3 L/min. Using these sample collection parameters will provide a sensitivity limit of less than or equal to 0.003 structures per cubic centimeter. The activity based sampling program is contingent on dry weather. If the weather patterns are not conducive to air sampling, the event will be postponed. In addition, four Air Metrics® stationary air monitors will be deployed each day. Two will be downwind of the activity area, one will be upwind, and one will be located adjacent to the command post. The filter cassettes for the stationary monitors will be mounted on stands five feet above the ground surface and will be oriented downward and

operated open face during the activity.

It is expected that participants will switch out from the activity. When needed, a backup participant will be suited and ready prior to the personnel exchange. The participant will stop the activity, remove the backpack or belt, and pass it to the relief participant similar to the transfer of a baton in a relay race. The original participant will assist the relief participant with donning and adjusting the backpack or belt. The exchange is anticipated to take less than 60 seconds, therefore the sampling pumps and event time clock will not be halted during the exchange. If the exchange requires more than 60 seconds, the pump and event clock will be stopped until activity is re-initiated.

Collecting ISM Samples (all locations)

This activity is required at each location as part of the project involves collection of ISM samples in triplicate at each location. Three participants will walk across the property collecting an ISM sample. This involves stopping at each of 30 locations and collecting a plug of soil from the ground surface and placing this in the sample container. A rake should be used to pull back vegetation to the extent possible. The sampling tool has a variety of tips. We expect to use a tip that is 1 or 1.25 inches in diameter and the plug is expected to be about 2 inches deep. It is anticipated that one person can do this activity alone; however, the project manager may decide to have participants work in pairs. At each location, rope will be used to lay out the grid system in which ISM samples will be collected.

Equipment/Supply Requirements

**Type**	**Description**	**Quantity Required**	**Source**	**Estimated Cost/Rental GOV rate ***
Stakes, tape, rope, measuring wheel	To be used to delineate area and sample locations	4-8 stakes	OEA will provide	None
Sampling Tool	Incremental Sampling Tool designed to get consistent plug of soil at each location	3	Field Environmental Instruments, Inc.	$180 per week each<br>$540 total
Plastic bags	ISM samples are placed into 1-gallon baggies during sample collection	20	OEA will provide	None
Stationary air monitors	Air metrics	4	OEA will provide	None

The anticipated maximum duration of this activity will be 180 minutes.

Collecting Discrete Soil Samples

This activity is required at each location as part of this project requires collecting 5 discrete soil samples at each location. The discrete soil sample locations will be marked with a stake. Using a rake to remove vegetation and a hand trowel to collect soil, two participants will collect sufficient volume of soil for the required analyses (about 48 oz. of soil). Soil will be placed in a bowl and mixed by hand, vegetation will be removed by hand, and soil will be placed into sampling containers. Soil sampling will be conducted in accordance with EPA ERT SOP #2012

(http://www.ert.org/products/2006.PDF, see Appendix B). A portable moisture content sampler will be used to determine percent moisture at each location and this will be noted on the field data sheet, but results from the fixed laboratory will be used for formal reporting.

Equipment/Supply Requirements

**Type**	**Description**	**Quantity**	**Source**	**Estimated Cost/Rental GOV rate ***
Hand trowels	Flat or spade	2	OEA will provide	None
Bowls	Stainless steel mixing bowls	25	OEA will provide	None

Subsampling of ISM Samples

In this activity two participants will subsample the three ISM samples from each location. This involves spreading out the collected soil for each ISM sample onto a disposable baking tray. Any visible vegetation will be removed by hand. Then, soil will be divided into 30 sections, then each section will be subsampled and material will be placed into the sample containers for the 4 types of analyses to be done. This process needs to be done systematically so that each sample represents the entire area that has been sampled. The ISM samples will be collected with a specialized coring device, placed in a plastic baggie, and then spread onto a disposal baking tray for subsampling. A specialized scoop with square side, or large diameter straw will be used to do the subsampling. One increment from each section of the pan will be placed into each sample container. For example, the sample container could be divided into 30 grids and the sample should contain the same amount of material from each grid. Using the specialized scoop, 10 grams of soil will be placed into 2-oz. glass jars for metals analysis and plastic bottles for asbestos analysis.

Equipment/Supply Requirements

**Type**	**Description**	**Quantity**	**Source**	**Estimated Cost/Rental GOV rate ***
Baking Trays	Aluminum or stainless steel baking trays	20	OEA will provide	None
Sampling Tool/Straws	A Square sided sampling tool or straws are needed to subsample the soil	20	TBD	TBD

Incremental Sampling Activities

At each location, 3 ABS samples will be collected. The total sample collection time for each ABS sample should be about 180 minutes. At some locations, a string trimmer may need to be used to clear vegetation before soil samples are collected. After trimming vegetation, a hand rake will be used to move trimmed vegetation to one side. This will be done only at points where soil will be collected as the vegetation is currently providing a protective barrier for exposures to

asbestos in soil. Field personnel will do these activities as part of the ABS for that location. Similarly, if there is time remaining after the ISM and discrete samples are collected, raking of soil will be done for the remainder of the sampling period, so only a single filter is used at each location. A steel garden rake or leaf rake with a width of approximately 15-24 inches will be used to remove debris such as rocks, leaves, thatch, and weeds. In addition a hand rake may be used to clear vegetation from sampling areas.

Equipment/Supply Requirements

**Type**	**Description**	**Quantity**	**Source**	**Estimated Cost/Rental GOV rate ***
String Trimmer	String Trimmer and Extra String	1	OEA will provide	None
Rakes	15-24-inch wide rake and Hand rake	2	OEA will provide	TBD

Each participant will wear at least one personal air monitor. Because 2 4QA samples are needed, two personal pumps will be used on the same person during the same time frame in two instances to collect field duplicate samples for activity-based sampling.

Additional Environmental Measurements

A portable weather station will be used to measure wind speed, wind direction, relative humidity, temperature, and barometric pressure, proximal to the study area. The weather station will be placed in a location free of obstructions that may interfere with accurate readings. Operating procedures for the Coastal Weatherpak® weather station are included in Appendix D. Soil moisture will be tested in the field and recorded at each location on the day of sampling. In addition, samples of soil and/or dredged material at each location will be collected for analysis of moisture content. These samples will be collected with stainless steel spoons and placed in 2 oz. glass jars.

Equipment/Supply Requirements

**Type**	**Description**	**Quantity**	**Source**	**Estimated Cost/Rental GOV rate ***
Weather Station	Coastal Weatherpak®	1	OEA will provide	None
Moisture Tests	2 oz. sample jars	45	OEA will provide	None
Zipper-top baggies	Pint/sandwich sized baggies	45	OEA	None
Bubble wrap	Bubble wrap baggies are used to cushion glass sampling jars	120	OEA	None

Soil Sample Collection

Three ISM soil samples will be collected from each location as described above. We anticipate using a 1 or 1.25 inch-diameter sampling tool to collect a 2-inch deep sample from each of 30 ISM locations. In addition, 5 discrete soil samples will be collected from each location. The field logbook will note which type of samples are collected. Sampling locations for ISM samples will

be noted by marking the 4 corners of the rectangular area with a GPS. These locations will be documented in the field logbook, staked and photographed against the local landmarks. Exact sampling locations for discrete samples will be determined in the field and marked using a GPS.

Soil sampling will be conducted in accordance with EPA ERT SOP #2012 (http://www.ert.org/products/2006.PDF, see Appendix B). Samples of soil from the study locations will be analyzed for asbestos by PLM (CARB 435), asbestos by PLM and TEM (ASTM D7521-13), and metals (EPA 200.2/200.7 http://www.epa.gov/superfund/programs/clp/ism1.htm). In addition, an aliquot of each soil sample will be processed using the FBAS followed by TEM analysis. These methods are included in Appendix B. PLM analysis will be performed by a contract laboratory; however, quality assurance on a subset of samples will be performed at EPA Region 10's Manchester Environmental Laboratory (MEL). Metals analysis for both scaled-up ISM prep samples and standard discrete samples will be performed at MEL. The discrete samples will be collected with stainless steel spoons, placed in a plastic mixing bowl, and then placed into 4-oz. plastic bottles for metals analysis and plastic bottles for asbestos analysis. The ISM samples require special processing that will be done in the field as part of the ABS activity.

Equipment/Supply Requirements

Type	Description	Quantity	Source	Cost est.
Asbestos/Metals Analysis in Soil	16 oz. HDPE Bottles (Asbestos in soil)	90	OEA	None
	Square-sided sampling tools/scoopulas	17	OEA	TBD
	Stainless Steel Spoons	45	OEA	None
	2 oz. Glass or Plastic Jars (ISM metals, 2 for each sample)	34	OEA	None
	2 oz. Glass or Plastic Jars (Lab QC)	8	OEA	None
	4 oz. Glass or Plastic Jars (discrete metals)	28	OEA	None
Fluidized Bed Samples	250 ml HDPE Bottles	45	OEA	None
Sieve and brushes	No. 14? Sieve for removing clumps and vegetation for ISM samples and brushes for decon	1	OEA	None
Zipper-top baggies	Quart sized baggies<br>Pint/sandwich sized baggies	90<br>115	OEA	None
Garbage Bags	Regular waste<br>Asbestos debris	1 box<br>5 bags	OEA	None
Field Printer	HP Field printer with extra cartridges and paper	1		
Analytical Balance	Sartorus field balance	1		
Weight set – for balance calibration verification	Single 10 gram weight would be adequate	1		

* - each participant is responsible for providing their own respirator.

Asbestos analysis will be performed by TBD. QA samples for asbestos in soil (CARB 435) will be analyzed at a designated QC laboratory (TBD). MEL will perform scanning electron

microscopy (SEM) to collect images and energy dispersive spectroscopy (EDS) to confirm mineralogy. The samples will be collected with stainless steel spoons or ISM scoops/straws and placed in plastic bottles. Fluidized bed samples will be processed at MEL (see SOP in Appendix B). The filters generated from the fluidized bed will be shipped to TBD for analysis by TEM.

Quality Assurance Samples

The quality control for air sampling will consist of 4 lot blanks that will be submitted and analyzed per ISO 10312 at two contract laboratories (2 filters each) prior to conducting field work. Field duplicate ABS air monitoring samples will be collected twice or one per every 10 samples. Additional quality assurance and quality control details are included in the QAPP.

Triplicate ISM samples will be collected at each location. This is part of the routine procedure for collection of ISM samples and provides an estimate of the total uncertainty inherent in the sample results. In addition, a QA triplicate sample for ISM is collected at one location to determine the variability contributed by subsampling. The quality control for discrete soil sampling is described in the Quality Assurance Project Plan and will consist of field and laboratory duplicates, same analyst recounts, and different analyst recounts. A field duplicate soil sample will be collected at two locations. An additional aliquot of the duplicate sample will be send to MEL for interlaboratory comparison after the samples is processed in the primary laboratory. Additional quality assurance and quality control details are included in the QAPP.

Equipment Decontamination and Dust Suppression

Equipment decontamination will be conducted in accordance with EPA ERT SOP #2006 (http://www.ert.org/products/2006.PDF, see Appendix B). Clean water will be needed for decontamination of workers and equipment. EPA staff will bring clean water to the site. Water will be transported to study sites in a pressurized water tank and garden sprayers. If the sieve is used for ISM samples, it will need to be washed with water and wiped down in between samples.

Respirator cartridges used by participants will be changed out after each day's activities. Tyvek® Suits will be changed when the sampler leaves each sampling location. When the participants cross the perimeter of the study location, they will be rinsed with amended water before removing PPE. Waste PPE (Tyvek® suits, respirator cartridges, etc.) will be collected in asbestos debris bags and transported back to the Manchester Environmental Laboratory for temporary storage and disposal.

Equipment/Supply Requirements

**Type**	**Description**	**Quantity**	**Source**	**Estimated Cost/Rental GOV rate ***
Water tank	40 gallon pressurized	1	OEA will provide	None
	Decon Trailer	1	OEA will provide	None
Respirator wipes	Alcohol free	2 boxes	OEA will provide	None
Decontamination kit	Buckets, brushes, soap, DI water, Alconox (or other detergent)	2 or each	OEA will provide	None
Restroom	Portable rental type	1	Baker Septic - Ferndale	< $100.00/wk

Numbers of Samples

The number of samples for this project is estimated to be 15 personal monitoring samples, 15 ISM soil samples and 25 discrete soil samples plus QA samples. These estimates represent the maximum number of samples expected. The table below summarizes the number and type of samples requiring analysis for this project:

Activity	Sample Type	Number of Samples	Analytical Method	Estimated Flow Rate liters/min	Duration	Estimated volume of air (L)	Analytical Sensitivity Structures/cc
ISM and discrete soil sampling, subsampling, raking	Personal 3 samples/location	15	ISO 10312	2-4	3 hours	540	0.003
	Stationary	16	ISO 10312	5-10	6-8 hours	3,000 – 5000	0.0003
QA (Personal)	-------------	2	ISO 10312	2-4	3 hours	540	0.003
QA (Stationary)		1		5-10	6-8 hours	3,000 – 5,000	0.0003
Totals		34					

Activity	Sample Type	Number of samples	Analytical Sensitivity
Soil Sampling	ISM – Asbestos by PLM (15 x 2 + 1 QA triplicate)	17	0.25%
	ISM – Asbestos by PLM/TEM (15 x 1 + 2 QA triplicate)	17	0.1%
	ISM – Metals (Scaled up 10g) (15 x 1 + 6 QA triplicate)	17	Per method
Soil Sampling	Discrete – Asbestos by PLM (25 x 1 + 3 QA duplicates)	28	0.25%
	Discrete – Asbestos (25 x 1 + 3 QA duplicates)	28	0.1%
	Discrete – Metals (25 x 1 + 3 QA duplicates)	28	Per method
Fluidized Bed	ISM – Asbestos by TEM following processing (15 x 1 + 2 QA triplicate)	17	0.1% or less
	Discrete – Asbestos by TEM following processing (25 x 1 + 3 QA duplicates)	28	0.1% or less

Sample Documentation, Packaging and Shipping

Sample information, chain of custody documentation and sample labels will be managed and/or printed on-site using the Scribe software. Shipping notifications are provided to the RSCC for forwarding to the R10 EPA Manchester Lab and CLP Sample Management Office on the day of sample shipment. Air sampling cassettes will be placed inside the rigid cardboard container in an upright position. The cardboard container will be wrapped in clean bubble pack and placed in a rigid container such as a cooler. Air sampling cassettes will be sent to the laboratory following

standard EPA chain of custody procedures. The EPA Adhesive EPA custody seals will be applied on two sides of the lid of the shipping cooler. Signed chain of custody forms will be placed in sealable bags and taped to the inside lid of each cooler. Examples of the custody seals, EPA Region 10 chain of custody form, and FADES sample work sheets and data entry instructions are included in Appendix E.

Analysis of Samples

Both the personal and stationary air monitoring samples will be analyzed for asbestos fibers using the International Organization for Standardization (ISO) 10312 entitled method Ambient Air – Determination of Asbestos Fibers: Direct-Transfer Transmission Electron Microscopy Method. The analytical sensitivity and detection limits for analysis are stated in the QAPP. The analytical data will be managed using the National Asbestos Data Entry Spreadsheet (NADES) which is compatible with ISO 10312. A copy of the NADES is included in Appendix G.

Soil samples will be analyzed for asbestos using CARB 435 – Determination of Asbestos Content in Serpentine Aggregate and ASTM D7521-13 – Standard Test Method for Determination of Asbestos in Soil. Additionally, dredged materials and soils will be analyzed for metals using Method EPA 200.2/200.7. The analytical sensitivity and detection limits for the analysis are stated in the QAPP.

Soil samples also will be processed at MEL using the fluidized bed (SOP is included in Appendix B). This device is used to entrain fine particulates into the air inside the device, where they can be captured on a membrane filter. The filter is subsequently analyzed by TEM using ISO 10312.

Rinsate samples will be analyzed for asbestos using EPA Method 100.2 – Determination of Asbestos Structures over 10 um in Length in Drinking Water. The analytical sensitivity and detection limits for the analysis are stated in the QAPP.

Acronyms:

ABS – Activity-Based Sampling
CLP – Contract Laboratory Program
EDS – Energy Dispersive Spectroscopy
ERT – Environmental Response Team
FABS – Fluidized Bed Asbestos Segregator
FADES – Field Asbestos Data Entry Sheet
HASP – Health and Safety Plan
HEPA – High Efficiency Particle Arrestance
ISM – Incremental Sampling Methodology
ISO – International Organization for Standardization
MCE – Mixed Cellulose Ester
MEL – Manchester Environmental Laboratory
NADES – National Asbestos Data Entry Spreadsheet
PLM – Polarized Light Microscopy
PPE – Personnel Protective Equipment

SEM – Scanning Electron Microscopy
SOP – Standard Operating Procedures
TBD – To be determined
TEM – Transmission electron microscopy
QA – Quality Assurance
QAPP – Quality Assurance Project Plan

References:

U.S. EPA Region 10, October 13, 2009, Soil, Sediment and Surface Water Sampling, Sumas Mountain Naturally-Occurring Asbestos Site, Whatcom County, Washington, prepared by the Office of Environmental Assessment, Seattle, Washington, http://www.epa.gov/region10/pdf/sites/sumasmountain/soil_sediment_surfacewater_sampling_finalreport_sumas_2009.pdf.

U.S. EPA Region 10, April 2011, Environmental Monitoring for Asbestos: Sumas Mountain Asbestos Site Selected Residential Properties, Bulk Sampling and Analysis Activity Based Sampling Surface Water Sampling, Whatcom County, Washington, prepared by the Office of Environmental Assessment, http://www.epa.gov/region10/pdf/sites/sumasmountain/asbestos_monitoring_report_april2011.pdf.

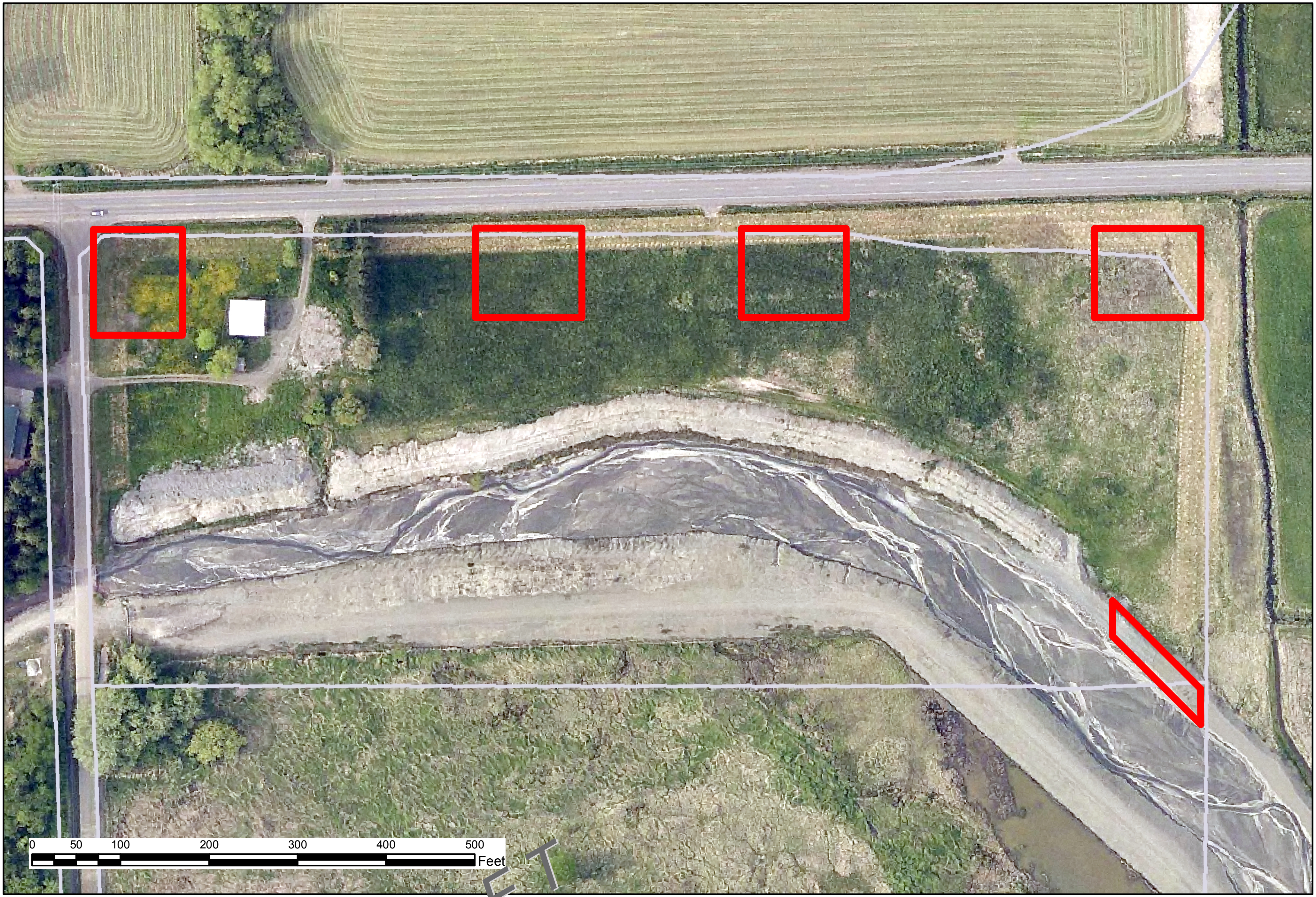

N

Figure 1
Proposed Sampling Locations,
Oat Coles/South Pass Road
Sumas Mountain Asbestos Site

EPA Region 10 GIS Team

Image Data from Whatcom County Administrative Services
Image Date 2013, pixel resolutinon 9"
Map Created 09/25/2014

APPENDIX B

STANDARD OPERATING PROCEDURES

STANDARD OPERATING PROCEDURES

ACTIVITY-BASED AIR SAMPLING FOR ASBESTOS

CONTENTS

1.0 SCOPE AND APPLICATION

2.0 METHOD SUMMARY

3.0 SAMPLE PRESERVATION, CONTAINERS, HANDLING AND STORAGE

3.1 Sample Preservation
3.2 Sample Handling, Container and Storage Procedures

4.0 INTERFERENCES AND POTENTIAL PROBLEMS

4.1 Area Selection
4.2 Flow Rate Considerations
4.3 Transmission Electron Microscopy (TEM) Specimen Preparation Methods

4.3.1 Direct-Transfer TEM Specimen Preparation Methods
4.3.2 Indirect TEM Specimen Preparation Methods

4.4 Sampling Cassette Orientation

5.0 EQUIPMENT/APPARATUS

6.0 REAGENTS

7.0 PROCEDURES

7.1 Pre-Site Sampling Preparation
7.2 Calibration Procedures

7.2.1 Calibrating a Personal Sampling Pump with a Rotameter
7.2.2 Calibrating a Personal Sampling Pump with an Electronic Calibrator

7.3 Meteorology
7.4 General Sampling Information
7.5 Generic Activity-Based Sampling Scenario/Raking

CONTENTS (cont'd)

7.6 Site-Specific Activity-Based Sampling Scenarios

7.6.1 ATV Riding
7.6.2 Child Playing in the Dirt
7.6.3 Gardening/Rototilling
7.6.4 Weed Whacking/Cutting
7.6.5 Digging
7.6.6 Lawn Mowing
7.6.7 Walker with Stroller
7.6.8 Jogging
7.6.9 Two Bicycles
7.6.10 Basketball Scenario

7.7 Cumulative Exposure Scenario
7.8 Background/Reference Sampling
7.9 Perimeter Sampling
7.10 Soil Sampling

8.0 CALCULATIONS

9.0 QUALITY ASSURANCE/QUALITY CONTROL

10.0 DATA VALIDATION

11.0 HEALTH AND SAFETY

12.0 REFERENCES

13.0 APPENDICES

A - Tables

1.0 SCOPE AND APPLICATION

As a result of a directive issued by the United States Environmental Protection Agency (U.S. EPA) Office of Solid Waste and Emergency Response (OSWER Directive 9345.4), estimating asbestos exposures resulting from suspension of soils is an area of increased importance to the Superfund Program. Such exposures may be estimated via monitoring and/or modeling methods. At present, models are not available to accurately estimate asbestos exposure associated with the disturbance of contaminated soil. Therefore, personal monitoring in the form of activity-based sampling (ABS) is the most appropriate technique to estimate exposure. Personal exposure is influenced by the activities performed, the duration of the activity and the site-specific soils of interest.

At a number of diverse sites across the county (Clear Creek Management Area, San Benito County, California (CA), El Dorado Schools, North Ridge Estates, Klamath Falls, Oregon, Slodusty Road, Garden Valley CA, Ambler Alaska), the U.S. EPA has demonstrated that disturbance of soil with low levels of asbestos (including soil concentrations less than 1.0 percent (%) as measured by Polarized Light Microscopy) can potentially result in significant concentrations (>0.1 structures per cubic centimeter) of respirable asbestos fibers in the breathing zone of individuals engaged in various physical activities. This may result in a cancer risk in excess of Comprehensive Environmental Response, Compensation and Liability Act (CERCLA) remedial objectives.

Since personal monitoring is more representative of actual exposure than samples obtained from a fixed downwind location (McBride 1999, Rodes 1995, Hildemann 2005), personal monitoring results are generally most relevant to CERCLA risk characterizations. Thus the best measure of actual exposure to an individual would be through the collection of personal air samples over the exposure period of interest (NIOSH 1977). However, at CERCLA sites, it is neither always possible nor practical to do so. EPA has thus developed a sampling procedure called ABS, designed to mimic the activities of a potential receptor.

As part of ABS, U.S. EPA or contractor personnel trained in hazard recognition and mitigation, serve as surrogates for the potentially exposed populace of interest. ABS simulates routine activities in order to mimic and evaluate or predict personal exposures from disturbance of materials potentially contaminated with asbestos. Similar sampling approaches have been used to assess exposures to pesticides and lead (U.S. EPA 2000) and this technique has long been a cornerstone of industrial hygiene wherein workplace exposures are routinely assessed via personal exposure monitoring.

This document provides guidance for ABS for a particular set of activities or scenarios. Personal monitoring may be conducted during various activities such as raking, All-Terrain Vehicle (ATV) riding, rototilling, digging, a child playing in the dirt, weed whacking, lawn mowing, walking with a stroller, bicycling, and playing basketball.

These are standard (i.e., typically applicable) operating procedures which may be varied or changed as required, dependent upon site conditions, equipment limitations or limitations imposed by the procedure. In all instances, the ultimate procedures employed should be documented and associated with the final report.

This document is not intended to be used as a substitute for a site-specific Quality Assurance Project Plan (QAPP) or a detailed Sampling and Analysis Plan (SAP). This document is intended to be used as a reference for developing site-specific QAPPs and SAPs.

Mention of trade names or commercial products does not constitute U.S. EPA endorsement or recommendation for use.

2.0 METHOD SUMMARY

There are two types of ABS that can be employed in the field: generic ABS and site-specific ABS. Generic ABS can be used with potentially contaminated soil and utilizes a rake to disturb the soil over a known area in conjunction with the collection of air samples to characterize potential exposure. Site-specific ABS is also used with contaminated soil; however, it utilizes site-specific activities to disturb the soil, such as riding ATVs, jogging or riding bikes. Although site-specific ABS provides a more realistic measure of fiber release, it can also be more resource intensive and it is recommended to be used after the generic ABS, if results deem necessary.

For all ABS events, asbestos samples should be collected from the breathing zones of the subjects at an appropriate flow rate. Special consideration should be given to characterizing exposure to children as it has been hypothesized that children are more prone to exposure than adults (U.S. EPA 2000) because they tend to be closer to the source. Sample flow rates, duration and final volume will need to be weighed against the number of grid openings that must be counted (cost factor) to obtain the needed sensitivity. Sampling periods should be of sufficient durations (averaging time) to facilitate collection of a representative sample and achieving the required level of sensitivity.

3.0 SAMPLE PRESERVATION, CONTAINERS, HANDLING AND STORAGE

3.1 Sample Preservation

No preservation is required for asbestos samples.

3.2 Sample Handling, Container, and Storage Procedures

1. Place a sample label on the cassette indicating a unique sampling number. Do not put sampling cassettes in shirt or coat pockets as the filter can pick up fibers or a static charge that could disturb the dust deposited on the filter media.

2. Samples must be handled gently with the filter inlet facing upward to avoid disturbing the particulate deposited on the filter and to minimize the potential of imparting a static charge to the cassette, which might alter the particulate deposition on the filter media.

3. Place the cassette individually in a manila-type envelope. Each envelope should be marked with the sample identification number, total volume, and date.

4. To the best extent possible, the sampling cassettes in the manila envelopes should be placed right side up so that the cassette inlet cap is on top and cassette base is on bottom. Place samples into a shipping container and use enough packing material to prevent jostling or damage. Samples must be handled gently so as not to disturb the dust deposited on the filter media. Do not use vermiculite or any other type of fibrous packing material for samples. If possible, hand carry to lab.

5. Provide appropriate documentation with samples (i.e., chain of custody and requested analytical methodology).

4.0 INTERFERENCES AND POTENTIAL PROBLEMS

4.1 Area Selection

When selecting areas for ABS, consideration should be given to the potential for off-site migration of contaminants and possible exposure of the public. Within the constraints of ABS, to the degree practical, particulate generation migration off-site should be minimized, and constraints or mitigation protocols established to eliminate public exposure. These constraints/mitigation protocols may include conducting the ABS in remote areas of the site, dust suppression using water mist, building a containment structure, etc. Air sampling should be conducted to document the airborne concentration of asbestos at the site perimeter during activities.

4.2 Flow Rate Considerations

For activities that generate a large quantity of dust (i.e., particulates), sample flow rates may need to be reduced accordingly to avoid overloading the filters. For example, a sampling pump flow rate of approximately 3.0 liters per minute (L/min) was found most effective at one site for monitoring for asbestos while riding ATVs on dusty soils while high soil moisture and reduced particulate generation at another site permitted a 5.0 L/min flow rate.

High flow rates may result in filter damage due to failure of its physical support associated with increased pressure drop, leakage of air around the filter mount so that the filter is bypassed or damage to the asbestos structures (breakup of bundles and clusters) due to increased impact velocities (ISO 10312). High flow rates can also tear the filters during initial pump startup due to the shock load placed on the filter when the pump is first started.

Sampling larger volumes of air and analyzing greater areas of the filter media can theoretically lower the limit of detection indefinitely. In practice, the total suspended particulate (TSP) concentration limits the volume of air that can be filtered as TSP can obscure asbestos fibers. The International Organization for Standardization (ISO) Method 10312 states that the direct analytical method cannot be used if the general particulate loading exceeds approximately 10% coverage of the collection filter. An airborne concentration of approximately 10 micrograms per cubic meter

(µg/m^3), corresponding to clean rural air, results in approximately 10% coverage of the filter media based on a 4000-L sample.

The following formula from ISO 10132 may be used to calculate the analytical sensitivity:

$$S = \frac{A_t}{KA_gV}$$

Where:

S = Analytical sensitivity expressed in structures per liter

A_t = Active area in square millimeters of the collection media or filter

A_g = Mean area in square millimeters (mm^2) of the grid openings examined,

K = Number of grid openings examined

V = Volume of air sampled, in liters

NOTE: 25-millimeter (mm) cassettes have an effective filter area of 385 mm^2 and 37-mm cassettes have an effective filter area of 855 mm^2. The typical grid opening is 0.0057 mm^2. Note: Grid size will vary between laboratories and dimensions should be verified prior to calculating the number of grid openings that must be counted to achieve a particular level of sensitivity.

Table 1 provides an example of the minimum number of grid openings that must be counted in order to achieve various sensitivity and detection limits.

It is frequently more efficient to employ co-located samplers to collect a high and low volume of air. This increases the likelihood of at least one of the two samples being readable using the direct analytical method (ISO 10312) than to lose the sample due to overloading or having to analyze by the indirect method (ISO 13794).

4.3 Transmission Electron Microscopy (TEM) Specimen Preparation Methods

It can be argued that direct methods yield an under-estimate of the asbestos structure concentration because other particulate material with which they are associated conceals many of the asbestos fibers present. Conversely, indirect methods can be considered to yield an over-estimate because some types of complex asbestos structures disintegrate during the preparation, resulting in an increase in the numbers of structures counted.

4.3.1 Direct-Transfer TEM Specimen Preparation Methods

Direct-transfer preparation methods are intended to retain all particles in the same relative positions with respect to each other on the final TEM grids as on the original filter. The membrane filter, or a portion of it, is placed on a microscope slide with the sample face upward, and then collapsed by exposure to acetone vapor. The cleared filter is then etched in a low-temperature plasma asher, subsequently coated with carbon in a sputtering device and then peeled from the glass slide. A portion of the collapsed, etched and carbon-coated filter is then transferred to an electron microscope grid and then extracted with dimethylformamide, glacial acetic acid and water to remove the filter. Once the process is complete, the particles originally collected on the filter are bound in the carbon film and the grids can be observed on a transmission electron microscope (ISO 1995). Direct-transfer TEM specimen preparation methods have the following significant interferences:

- The particulate density on the filter, which in turn is controlled by the sampled air volume and the total suspended particulate concentration in the atmosphere being sampled, restricts the achievable detection limit.
- The precision of the result is dependent on the uniformity of the deposit of asbestos structures on the sample collection filter.
- Air samples must be collected so that they have particulate and fiber loadings within narrow ranges. If too high a particulate loading occurs on the filter, it is not possible to prepare satisfactory TEM specimens by a direct-transfer method. If too high a fiber loading occurs on the filter, even if satisfactory TEM specimens can be prepared, accurate fiber counting may not be possible.

4.3.2 Indirect TEM Specimen Preparation Methods

In the indirect preparation method the membrane filter, or a portion thereof, is placed on a microscope slide, sample face downward, and ashed in a low temperature asher until complete calcination of the filter is achieved. The ash is then recovered in distilled water and the solution then filtered on a polycarbonate filter. The indirect transfer method re-distributes the particulate on a new membrane filter.

Indirect TEM specimen preparation methods have the following interferences:

- The size distribution of asbestos structures is modified (clusters, matrices bundles, etc. may be broken up during sample preparation).
- There is increased opportunity for fiber loss or introduction of extraneous contamination from laboratory glassware, process water, etc.

- When sample collection filters are ashed, any fiber contamination in the filter medium is concentrated on the TEM specimen grid.

The direct analytical method (ISO 10312) is the preferred method and every reasonable effort should be made to prevent overloading of the filter, which would necessitate use of the indirect method. Samples that are overloaded may, at the discretion of the project management team, be analyzed by ISO Method 13794 "Ambient air – Determination of asbestos fibres – Indirect-transfer transmission electron microscopy method" (ISO 1999). Results of the ISO 13794 analysis should be reviewed discrete of the ISO 10312 samples and a decision made regarding combining the two data sets.

4.4 Sampling Cassette Orientation

Air sampling cassettes must be oriented with the open face pointing down to preclude large non-respirable particles from falling or settling onto the filter media.

5.0 EQUIPMENT/APPARATUS

- Personal sampling pumps, providing a flow rate from 0.020 L/min up to 4.0 L/min, battery powered
- High flow sampling pumps (i.e., Quik Take 30 or AirCon II), capable of providing a flow rate from 4.0 to 12 L/min, battery or alternating current (AC)
- Mixed cellulose ester (MCE) filter cassettes, 0.45 or 0.8 micrometer (µm), 25-mm diameter, purchased from a certified vendor with appropriate documentation (low filter background counts, consistent filter area, certified leak-free cassettes)
- Sampling setups, Tygon® tubing with Luer type adaptor
- Backpacks
- Sampling stands, for perimeter sampling
- Duct tape
- Tools, miscellaneous (e.g., screwdrivers, pliers, cutting tool, etc.)
- Envelopes, manila-type
- Whirlpak® bags
- Sample labels

- Chain of custody (COC) records
- Logbook and/or sampling worksheets
- Precision rotameter or primary flow standard appropriate for sampling flow rate
- Personal protective equipment (PPE), including but not limited to respirators, boots, gloves, eye protection, hard hat, to be determined based on type of activity and possible exposure
- Decon equipment (Plastic sheeting, liquinox®, buckets, brushes, water, Hudson sprayers, garbage bags, etc.)
- Power sources, e.g., line power, solar recharging batteries, power inverters, generators, etc.

6.0 REAGENTS

Reagents are not required for the preservation of asbestos samples.

7.0 PROCEDURES

7.1 Pre-Site Sampling Preparation

1. Determine the extent of the sampling effort (number of locations, repetitions, number of samples, etc.), the sampling methods to be employed, and the types and amounts of equipment and supplies needed.
2. Obtain necessary sampling equipment and ensure it is in working order and fully charged (if necessary).
3. Perform a general site survey prior to site entry in accordance with the site-specific Health and Safety Plan (HASP).
4. Once on-site the calibration is performed in the clean zone. The calibration procedures are listed in Section 7.2.
5. After calibrating the sampling pump, mobilize to the sampling location.

7.2 Calibration Procedures

To determine if a sampling pump is measuring the flow rate or volume of air correctly, it is necessary to calibrate the equipment. Sampling pumps should be calibrated on a routine basis and prior to use.

A rotameter can be used provided it has been calibrated with a primary calibrator. Typically rotameters are calibrated on a yearly basis. Sampling pumps can be calibrated prior to coming on-site in order to expedite on-site calibration. However, calibration must be verified on-site prior to use.

7.2.1 Calibrating a Personal Sampling Pump with a Rotameter

1. Refer to the manufacturer's manual for the Rotameter Operational Instructions.

2. Set up the calibration train using a rotameter, sampling pump and the sampling cassette that will be used during the sampling event. This train may be set up prior to field mobilization and will be checked in the field again prior to use.

3. To set up the calibration train, attach one end of the polyvinyl chloride (PVC) tubing (approx. 2 ft) to the cassette base; attach the other end of the tubing to the inlet plug on the pump. Another piece of tubing is attached from the cassette cap to the rotameter. Insure that the tubing and rotameter used to calibrate the pump do not restrict the airflow.

4. Assemble the base of the flow meter with the screw provided and tighten in place. The flow meter should be mounted within 6 degrees (^{o}) of vertical (Omega 1987).

5. Turn the sampling pump on.

6. Turn the flow adjust screw (or knob) on the personal sampling pump until the float ball on the rotameter is lined up with the pre-calibrated flow rate value on the rotameter. Note: rotameters should be marked with the previous calibration date and corresponding flow rates and scale.

7. A verification of calibration is generally performed on-site in the clean zone immediately prior to the sampling.

7.2.2 Calibrating a Personal Sampling Pump with an Electronic Calibrator

1. Refer to the manufacturer's manual for operational instructions.

2. Set up the calibration train using a sampling pump, electronic calibrator, and the actual sampling cassette or a representative filter cassette. The same lot of cassettes used for sampling should also be used for calibration.

3. To set up the calibration train, attach one end of the PVC tubing (approx. 2 foot) to the cassette base; attach the other end of the tubing to the inlet plug on the pump. Another piece of tubing is attached from the cassette cap to the electronic calibrator.

4. Turn the electronic calibrator and sampling pump on. Select a flow rate to calibrate.

5. Turn the flow-adjust screw or knob on the pump until the desired flow rate is attained on the rotameter.

6. Using the primary calibrator, obtain approximately 10 readings three times until the flow rate of ± 5% of the required flow is attained.

7.3. Meteorology

It is recommended that an onsite, portable, 3-meter meteorological station be established. If possible, sample after two to three days of dry weather and when wind conditions are representative for the climatology of the location based on month and time of day. Historical hourly wind speed and wind direction data should be analyzed before mobilization. Wind speed, wind direction, temperature, and station pressure should be recorded on the meteorological station data logger and real-time data should be available for review on the station display panel. Suggested meteorological station specifications can be found in Table 2, Appendix A or ERT SOP #2129, *Met One Remote Meteorological Station.* Alternatively, a nearby representative meteorological station, as determined by a meteorologist, may be used to acquire the necessary data.

7.4 General Sampling Information

For all activity-based sampling events, except as noted otherwise, asbestos samples will be collected from the breathing zones of the event participants. The breathing zone can be visualized as a hemisphere approximately 6 to 9 inches around an individual's face. Breathing zone samples provide the best approximation of the concentration of contaminants in the air that an individual is

actually breathing. Specific breathing zone heights should be determined on a project-by-project basis based on the anthropometrics for the study population and the participants' positions during the performance of each task.

If it is necessary to relieve a participant from the activity, another sample collector should be suited and ready to participate in the ABS prior to the personnel exchange. The participant will stop the activity, remove the backpack or belt, and pass it to the relief participant similar to the transfer of a baton in a relay race. The original participant will assist the relief participant with donning and adjusting the backpack or belt. The exchange is anticipated to take less than 60 seconds, therefore the sampling pumps and event time clock will not be halted during the exchange. If the exchange requires more than 60 seconds, the pump and event clock will be stopped until activity is re-initiated.

Sample volumes and detection/quantification limits should be specified in the site-specific QAPP with flow rates and sampling periods adjusted accordingly. Typical sensitivity limits that have been employed for risk assessment have been approximately 0.001 S/cc for ABS samples and 0.0001 S/cc for background or reference samples. Based on ISO 10312 Table 1, a sensitivity limit of 0.001 S/cc would require a sample volume of greater than 500 liters to keep the number of grid openings to be counted below 100. Similarly, a sample volume greater than 5000 L would be required to reach 0.0001 S/cc and count fewer than 100 grid openings. For all asbestos sampling, an asbestos sampling train consisting of 0.8-µm, 25-mm mixed cellulose ester (MCE) filter connected to a personal sampling pump will be used. The top cover from the cowl extension on the sampling cassette shall be removed ("open-face") and the cassette oriented face down for all asbestos filters. All samples should be collected open-faced unless a specific requirement for sampling closed-faced exists.

For activity based sampling, a personal sampling pump (or equivalent) or SKC Quick Take 30 will be calibrated to collect between 2 and 12 L/min of air through the filter depending on the capacity of the pump. The flow rate will be based upon the duration of time required to collect a minimum target volume of 560 L and provide a sensitivity limit of 0.001 S/cc.

Generally each activity based sampling event should be repeated a minimum of three times in an area to expose trends. This can be accomplished by a single participant repeating the activity three or more times or by having a single simulation with three or more participants. If soil moisture or seasonal variability is a concern, then three events for each different season or meteorological conditions may be appropriate.

The sampling pumps used should provide non-fluctuating airflows through the filter, and should maintain the initial volume flow rate to within ± 10% throughout the sampling period. A constant flow or critical orifice controlled pump typically meets these requirements. If the flow rate changes by more than 5% during the sampling period, the average of the pre- and post-sampling

rates will be used to calculate the total sample volume. If at any time the measurement indicates that the flow-rate has decreased by more than 30%, sampling should be terminated. Depending on the type of sampling pump used, it may be possible to salvage the sample if sufficient volume was collected; however, it may not be representative of the time it takes for the actual activity to be completed. Depending on the type of sampling pump used, the actual sampling time in hours and minutes before the sampling fault may be displayed and an actual sample volume calculated. If the fault was due to battery failure, it may be possible to check the post-sampling flow.

During certain ABS activities, participants may be fitted with two sampling pumps to collect a high-flow or volume and a low-flow or volume sample. Co-located samples are collected to sample a high and low volume of air to increase the likelihood of at least one of the two samples being readable using the direct analytical method (ISO 10312). Approximately 560 L (40 CFR 763) is collected for the low-flow samples and up to 4000 L for the high-flow samples. The targeted high volume is typically 1200 L, which permits counting approximately 54 grid openings for a sensitivity level of 0.001 S/cc.

7.5 Generic Activity-Based Sampling Scenario / Raking

The raking scenario, also referred to as the generic scenario, is appropriate for all sites with soils potentially contaminated with asbestos. Generic ABS should be employed in a grid pattern to evaluate the potential for fiber release from soil over a portion of the site. If the analytical results are above the criteria that were derived for the site, then remediation or institutional controls should be implemented or additional site-specific ABS should be undertaken. If the analytical results are below the criteria that were derived, then no further action may be necessary.

In this activity or simulation a participant will rake a lawn or garden area to remove debris such as rocks, leaves, thatch and weeds using a leaf rake with a rake width of approximately 20 to 28 inches. Participants should strive to disturb the top half-inch of soil with an aggressive raking motion. This depth will vary based on the objective of the scenario.

Each raking participant donning appropriate PPE will be fitted with a personal sampling pump contained in a backpack with the cassette secured to the shoulder straps near the operator's lapels in the breathing zone. Personnel will rake a lawn or garden area to remove debris for a minimum of 1 to 2 hours (flow rate and sensitivity level dependent). Raking will occur in a measured area with vegetation, soil or rocks/gravel and will occur in an arched motion raking from the left of the participant to the right. The participants will rake the debris towards themselves facing one side of the square for 15 minutes then the participant will turn 90 degrees clockwise and begin a new side. Participants will continue to rake each side of the square and rotate 90 degrees. Once several small piles of debris have been made, the participant shall pick up the debris and place it in a trashcan. The sequence of raking, rotating and picking up debris shall be repeated for the duration of the sampling period. The participant should stay in the same plot for the entire sampling period.

7.6 Site-Specific Activity-Based Sampling Scenarios

If site-specific ABS is undertaken, the number and types of activities as well as the types of scenarios should be based on current and potential land use. Reference to current and currently planned future land use and the effectiveness of institutional or legal controls placed on the future use of the land should be evaluated. Probable land use should be selected based on zoning and the existing land use of the site and adjacent areas.

Land use assumptions should be based on a factual understanding of site-specific conditions and reasonably anticipated use. The land use evaluated for the assessment should be based on a residential exposure scenario (i.e., the default worst-case) unless residential land use is not plausible for the site. Future land use assumptions should be consistent with reasonably anticipated future land use based on input from planning boards, appropriate officials, and the public.

7.6.1 ATV Riding

This scenario might be appropriate for recreational areas or other areas where ATVs are typically ridden where asbestos contamination is present. This activity is designed to be representative of two or more ATV participants riding on a course or trail. Riders should maintain their relative position (lead, middle, tail) throughout the activity.

Each ATV rider wearing appropriate PPE will be fitted with two personal sampling pumps set at two distinct flow rates, to collect approximately 560 and 1200 liters of air, because of filter overloading concerns. The cassettes for the personal sampling pumps will be attached to the shoulder straps of the backpack proximal to the riders' lapels in the breathing zone. It may be beneficial to attach a dust monitor (e.g., DataRAM) to the tail ATV to record dust levels and gauge dust loading. The sampling pumps will be carried in a backpack while the dust monitor, if used, will be mounted to the ATV.

Personnel will ride the ATVs around a course at the same time until a sufficient volume of air has been collected to achieve the required sensitivity limit of 0.001 S/cc of air. The riders, one lead rider and one following rider, will vary the vehicle speed between 5 and 30 miles per hour (mph). Riders will strive for an average speed of 10 mph. The average speed is a target speed only; vehicle speeds will be adjusted to meet track conditions. Vehicles will be equipped with a speedometer and odometer to record speeds and distance traveled. ATV riding and sampling should be conducted for 30 to 120 minutes in duration, depending on dust loading and required detection limits.

ATVs and ATV tires should be selected as appropriate for the area being studied. Specifically, the size (i.e., weight, horsepower, etc.) of the ATV should be appropriate for

the study area. The vehicle tires should have a tread pattern that is representative of those typically used in the area. Local ATV shops or ATV clubs should be consulted for guidance.

7.6.2 Child Playing in the Dirt

This scenario might be appropriate for sites where schools, playgrounds, parks or residential areas, etc. are contaminated with asbestos; the overarching criteria being areas where a child might be expected to play or dig in the dirt. This scenario was designed to be representative of a child playing in the dirt with a shovel and pail.

The event participant wearing appropriate PPE will be fitted with a personal sampling pump; the inlet to the filter will be at a height of approximately 1 to 3 feet above the ground to simulate a child's breathing zone. The actual pump unit should be secured in a backpack or on a belt.

A participant should sit on the ground while digging or scraping the top 2 to 6 inches of surface soil, placing it in a small bucket or pail and dumping it back on the ground. The activity will be paced such that soil will be placed in the bucket and dumped approximately every two to five minutes, regardless of the amount of material in the bucket. The bucket should be emptied rapidly from a height of approximately 12 inches, based on observations of two to four-year-olds playing in a sandbox.

A sampling period and flow rate to collect a sufficient volume of air will be determined as to achieve the project-specific detection/quantification limit. The sampling period will be divided into equal sub-periods to facilitate having the participant face each compass direction for an equal amount of time during the activity. This approach is designed to mitigate the effect of wind direction on potential exposure. Random head and body movement during the activity should further mitigate the impact of wind direction on exposure. Ideally, the participants will face each compass direction at least twice during the sampling event. For example, during a two-hour or 120-minute event, the participant might face North for 15 minutes, rotate to the East for 15 minutes, then South for 15 minutes, then West for 15 minutes and return to the North to repeat the cycle. Participants should move to a fresh patch of soil after the completion of each cycle (360 degree rotation).

7.6.3 Gardening/Rototilling

This scenario might be appropriate for sites where gardening or surface disturbance to a depth of approximately one foot is anticipated. This activity is designed to be representative of individuals participating in gardening activities using a rototiller.

Each rototilling participant donning appropriate PPE will be fitted with a personal sampling pump. The actual pump unit will be contained in a backpack with the cassette secured to the shoulder straps near the operator's lapels in the breathing zone.

Personnel will operate a rototiller for a minimum of two hours to loosen soil in the yard to a depth of approximately 12 inches. The depth chosen is area-specific and will need to be determined on a case-by-case basis. A rear tine rototiller in the six to eight horsepower range will be selected. Other types or sizes of tillers may be appropriate based on the soil conditions and type of gardening being conducted.

A 100 to 720-square-foot plot of land will be selected to till. The average size of a community garden in New Jersey was 720 square feet based on a survey conducted by Rutgers University in 1991 (Patel 1991). The edges will be delineated. Square plots are preferred. The rototiller operator will conduct typical associated activities such as removing rocks and debris from the tilled area. To account for the effects of varying wind direction on potential exposure, the operator will till the soil back and forth towards each side of the square continuously for 10 minutes, shut down the machine or place it in neutral, and rake or sort through the material for five minutes. The operator will then turn 90 degrees in a clockwise direction and repeat the previous 15-minute procedure. The operator will continue to rotate 90 degrees clockwise every 15 minutes until the two-hour sampling period is complete. The participant should stay in the same plot for the entire sampling period.

7.6.4 Weed Whacking/Cutting

This scenario might be appropriate for sites where lawn maintenance might be conducted such as in residential and commercial areas. This activity is designed to simulate a person trimming weeds and grasses.

Each weed-whacking participant will be fitted with a personal sampling pump. The actual pump unit will be contained in a backpack with the cassette secured to the shoulder straps near the operator's lapels in the breathing zone. Personnel wearing appropriate PPE will operate a gas or electric-powered string trimmer. A 25 to 35-cc gas or electric-powered trimmer with a 16 to 18- inch cutting swath will be selected. Trimming and edging will occur in a measured area with thick vegetation (typically 100 to 720-square feet, based on a typical residential garden) (Patel 1991). Trimming will be done using a side to side sweeping motion with the operator moving in a series of straight lines back and forth towards one side of the selected area for 10 minutes, resting five minutes, and turning 90 degrees in a clockwise direction before repeating this 15-minute procedure for the

duration of the sampling period. The participant should stay in the same plot for the entire sampling period.

7.6.5 Digging

Digging might be appropriate for sites where construction projects are likely to occur or where plants might be planted. Digging will occur in a measured area with vegetation, soil or rocks/gravel.

Each digger participant donning appropriate PPE will be fitted with a personal sampling pump contained in a backpack with the cassette secured to the shoulder straps near the operator's lapels in the breathing zone. The participants will dig a hole to approximately two feet deep and two feet (representative of planting a small shrub or digging a fencepost; site-specific dimensions should be specified in the QAPP/SAP) in diameter (Vodak 2004) and will place the soil next to the hole. The participants will then refill the hole with the soil that had been removed. Participants will then rotate 90 degrees in a clockwise direction and continue to dig and refill additional holes until the sampling period is complete. The sequence of digging, filling and rotating shall be repeated for the duration of the sampling period.

7.6.6 Lawn Mowing

Lawn mowing might be appropriate for sites where lawn maintenance might be conducted such as residential and commercial areas.

Each lawn-mowing participant will be fitted with a personal sampling pump contained in a backpack with the cassette secured to the shoulder straps near the operator's lapels in the breathing zone. Personnel wearing appropriate PPE will operate a gas-powered lawn mower. Mowing will occur in a measured area with thick vegetation and will occur in a shrinking square pattern. Participants will divide the area into a number of squares that decrease in size towards the center of the square by the width of the mower swath. Mower blades will be set at approximately 2 to 2.5 inches. A bag-less side discharge 3- to 5-horsepower lawn mower will be used for this exercise.

7.6.7 Walker with Stroller

This scenario might be appropriate for sites such as parks, paths or open-space. The actual pump unit will be secured in a backpack. The cassette for the personal sampling pump will be attached to the shoulder straps of the backpack proximal to the walker's lapel in the breathing zone. A second pump will be placed in the stroller at a child's breathing zone height.

During these events, walkers wearing appropriate PPE pushing a stroller will walk back and forth along a portion of a path until a sufficient volume of air has been collected to achieve the required detection limit. The walkers will vary their speed between 1.5 and 4 mph. Walkers will strive for an average speed of 2 mph. The average speed is a target speed only; speeds will be adjusted to meet trail conditions. Walkers should be equipped with a global positioning system (GPS) unit to estimate average speed and distance traveled.

7.6.8 Jogging

This scenario might be appropriate for sites such as parks, paths or open-space. The actual pump unit will be secured in a backpack. The cassette for the personal sampling pump will be attached to the shoulder straps of the backpack proximal to the jogger's lapel in the breathing zone.

During these events, joggers wearing appropriate PPE will run/jog back and forth along a portion of a path until a sufficient volume of air has been collected to achieve the required detection limit. The joggers will vary their speed between 2.5 and 5 mph. Joggers will strive for an average speed of 4 mph. The average speed is a target speed only; speeds will be adjusted to meet trail conditions. Joggers should be equipped with a GPS unit to estimate average speed and distance traveled.

Two or more joggers can participate in this activity. When multiple joggers participate, they should maintain their relative position throughout the event (lead, middle, tail). Joggers should be spaced five feet apart.

7.6.9 Two Bicycles

Bicycling might be appropriate for sites such as parks, paths or open-space. Two bicyclists wearing appropriate PPE will ride back and forth with one leading and one following along the length of the site portion of a path or ride around a site (no trail) until a sufficient volume of air has been collected to achieve the required detection limit.

The bicycling participants will each be fitted with personal sampling pumps. The actual pump units will be contained in backpacks with the cassettes secured to the shoulder straps near the cyclists' lapels in the breathing zone.

During these events, the bicycle riders will vary their speed between 3 and 15 mph. Riders will strive for an average speed of 8 mph. The average speed is a target speed only; bicycle speeds will be adjusted to meet trail conditions. Bicycles will be equipped

with a GPS to estimate average speed and distance traveled. Riders should maintain their relative position (lead, tail) throughout the activity.

7.6.10 Basketball Scenario

This scenario might be appropriate for sites where basketball courts are present. The basketball scenario was developed to simulate a group of recreational basketball players gathering to play a casual game of basketball for 120 minutes on an outdoor concrete or macadam court. Between four and 10 players wearing appropriate PPE can participate in this exercise.

- From 0 to 15 minutes, two of the players will sweep court with push brooms from the perimeter of the court to the center. While these two people are sweeping the court, the remaining personnel should mill about under the basket and take a few shots.

- From 15 to 30 minutes, shot practice participants stand around the key as for a free throw, with the exception that one of the participants is positioned under the basket to retrieve the ball after each shot. The player closest to the basket on the left side (facing the basket) takes two shots and the ball/shooter rotates counter clockwise after those two shots. Each person shoots consecutively until everyone has taken two shots. The entire group then rotates clockwise. This sequence should be repeated until time expires. Ideally, each player should shoot from each key position and take a turn retrieving the ball under the basket.

- From 30 to 45 minutes, each player takes turns practicing lay-ups. All players line up on the left side of the basket (facing the basket) and shoot one after another. The first person shoots then retrieves the ball for next person in line and so on. Players should use two basketballs with the second person bouncing the ball outside of the key as the first person shoots. Players should run a full cycle from left then a full cycle from right; repeating the left, right cycles until the interval time is up.

- From 45 to 60 minutes, shot practice as described in the 15 to 30 minute interval above will be conducted.

- From 60 to 75 minutes, a half-court game will be played to the degree practical.

- From 75 to 100 minutes, shot practice as described in the 15 to 30-minute interval above will be conducted.

- From 100 to 120 minutes, a lay-up drill as described in the 30 to 45 minute interval above will be conducted.

7.7 Cumulative Exposure Scenario

A cumulative exposure study might be appropriate for sites where individuals move about a site during the course of a day, with varying levels of exposure at multiple indoor and outdoor locations. The objective is to estimate aggregate and cumulative exposure to asbestos over the course of a day. Cumulative exposure studies should be conducted in order to increase understanding of linkages between sources of asbestos and subsequent exposure and dose to humans for use in mitigating risk and reducing exposure and disease.

Over periods of weeks, years or decades, exposures to environmental agents such as asbestos occur intermittently rather than continuously. Yet long-term health effects, such as cancer, are routinely projected based on an average dose over the period of interest (typically years), rather than as a series of intermittent exposures. Consequently, long-term doses are usually estimated by summing doses across discrete exposure episodes and then calculating an average dose for the period of interest (e.g., year, lifetime).

For the cumulative exposure studies, representative members of the population of interest should be selected for 24 hour sampling. The volunteers should be instructed to go about their day as usual. That is, they should not modify their schedule or activities just because they will be wearing a sampling pump.

A minimal description of exposure for a particular route must include exposure concentration and the duration. This is the method of choice to describe and estimate short-term doses, where integration times are of the order of minutes, hours or days. When projecting long term exposures, on the order of years or a lifetime, since it is typically impractical to sample for the entire exposure period, short-term exposure estimates are assumed to be representative of long-term periods and are integrated to estimate long-term exposures, typically with a safety factor to account for variability.

Observations of activities should be recorded throughout each cumulative exposure study, together with the other relevant factors including locations and activities during the study.

Samples will be collected using a personal air pump with a flow rate of approximately 3.5 L/min. Samples shall be collected open-faced with the inlet facing downward at a personal breathing zone height of 4 to 6 feet for 24 hours. Because the battery life for a personal monitor is typically eight to10 hours, the pump shall be changed out at approximately 8-hour intervals (keeping the same filter cassette). Each pump shall be pre-calibrated to 3.5 L/min prior to use. Each monitor shall be worn at normal breathing height during all waking hours. During sleep, the monitor will be placed

in the same room as the sleeping individual. The sampling cassette will be placed proximal to the breathing zone of the reclined participant.

Should a study subject participate in a high dust generating activity such as riding an ATV, the 24 hour sampling cassette event should be paused and a short term exposure sample should be collected on a separate cassette with an appropriately calibrated sampling pump. Once the high dust activity has been terminated, the original 24-hour cassette and pump should be resumed for the remainder of the sampling period. Results of the 2 or more samples, depending on the number of high dust generating events should be summed to derive the total 24-hour exposure data.

7.8 Background/Reference Sampling

Background/reference samples should be collected for all sampling events. A background or reference sample is defined as a sample collected upwind at a distance sufficient to prevent being influenced by the simulated activities and outside the site perimeter. To the degree practical, the area selected for background or reference sampling should be free of known asbestos contamination. The background level should reflect the concentration of asbestos in air for the environmental setting on or near a site or activity location and can be used to evaluate whether or not a release from the site or activity has occurred. Background level does not necessarily represent pre-release conditions or conditions in the absence of influence from source at the site. A background level may or may not be less than the detection limit, but if it is greater than the detection limit, it should account for variability in local concentrations. Background or reference samples should be collected concurrent with ABS using stationary sampling pumps. Sampling and analytical parameters (sample volume grid opening count, etc.) should be prescribed to permit a detection limit approximately an order of magnitude below that of the ABS detection limit.

An Aircon II sampling pump (or equivalent) will be calibrated to collect 10 L/min for on-site and off-site air samples through the filter. The flow rate will allow a minimum target volume of 4000 L and will provide a sensitivity limit of 0.0001 S/cc. Lower volume air samples will be collected concurrently at the ambient air sampling locations. Personal sampling pumps will be utilized in the same manner with the same media at a flow rate between 2- and 3- L/min in order to collect a sample volume of approximately 1000 L. The target sensitivity of these samples is also 0.0001 S/cc when additional grids are counted in accordance with the method. Co-located samples are collected to sample a high and low volume of air to increase the likelihood of at least one of the two samples being readable using the direct analytical method (ISO 10312).

7.9 Perimeter Sampling

Perimeter samples are defined as samples collected upwind, downwind or crosswind of a specific activity. When selecting areas for ABS, consideration should be given to the potential for off-site migration of contaminants and possible exposure of the public. Within the constraints of ABS, to

the degree practical, particulate generation migration off-site should be minimized, and constraints or mitigation protocols established to eliminate public exposure. These constraints/mitigation protocols may include conducting the ABS in remote areas of the site, dust suppression using water mist, building a containment structure, etc. Air sampling should be conducted to document the airborne concentration of asbestos at the site perimeter during activities. Perimeter air monitoring should be conducted to:

- Document air quality during ABS and establish background or upwind levels of asbestos during site activities
- Monitor and document air quality during site activities near sensitive receptors
- Provide risk management information and address public confidence
- Reduce possible liabilities associated with ABS

Perimeter air sampling should be performed to ensure that ABS activities do not result in excessive airborne asbestos emissions from the site. Air samples should be collected and analyzed to determine the concentrations of asbestos at the site perimeter.

An Aircon II sampling pump (or equivalent) will be calibrated to collect 10 L/min for on-site and off-site air samples through the filter. The flow rate will allow a target volume of 4000 L and will provide a sensitivity limit of 0.0001 S/cc. Lower volume air samples will be collected concurrently at the perimeter sampling locations using personal sampling pumps, if loading is an issue. These pumps will be utilized in the same manner with the same media at a flow rate between 2- and 3-L/min in order to collect a sample volume of approximately 1000 L. The target sensitivity of these samples is also 0.0001 S/cc when additional grids are counted in accordance with the method. Co-located samples are collected to sample a high and low volume of air to increase the likelihood of at least one of the two samples being readable using the direct analytical method (ISO 10312).

7.10 Soil Sampling

A sufficient number of soil samples should be collected to characterize the study area. Since particulates are expected to be released from the entire study area, the primary objective of the soil sampling is to estimate the populations mean concentration. Composite samples are appropriate for characterizing study areas and a sampling design program such as Visual Sampling Plan is recommended for calculating the number and location of samples with the appropriate confidence intervals. Soil sampling should be conducted in accordance with ERT SOP #2012, *Soil Sampling.*

Soil characteristics should be documented in conjunction with the activity-based personal exposure monitoring using American Society of Testing and Materials (ASTM), Method D2488 - 00: *Description and Identification of Soils (Visual-Manual Procedure)*, soil moisture by ASTM Method D2216-05*: Standard Test Methods for Laboratory Determination of Water (Moisture)*

Content of Soil and Rock by Mass and grain size by ASTM Method D6913-04e1: *Standard Test Methods for Particle-Size Distribution (Gradation) of Soils Using Sieve Analysis* or Method D422-63 (2002): *Standard Test Method for Particle-Size Analysis of Soils.*

Soil samples should be representative of the soil. Table 3 provides examples of soil sampling depths, which may be disturbed by the activity being performed.

The relationship between the concentration of asbestos in a source material (typically soil) and the concentration of fibers in air that results when the source is disturbed is very complex, depending on a wide range of variables. To date, no method has been found that reliably predicts the concentration of asbestos in air given the concentration of asbestos in the source. Because of this limitation, this SOP emphasizes an empiric approach, where concentrations of asbestos in air at the location of a source disturbance are measured rather than predicted.

8.0 CALCULATIONS

The sample volume is calculated from the average flow rate of the pump multiplied by the number of minutes the pump was running (volume = flow rate X time in minutes). The sample volume should be submitted to the laboratory and identified on the chain of custody for each sample (zero for lot, and field blanks).

The concentration result is calculated by dividing the number of asbestos structures reported after the application of the cluster and matrix counting criteria by the sample volume (concentration = number of asbestos structures / sample volume).

9.0 QUALITY ASSURANCE/QUALITY CONTROL (QA/QC)

The following general QA procedures apply:

1. All data must be documented on field data sheets or within site logbooks. Record the following: date, time, location, sample identification number, pump number, flow rate, and cumulative time.

2. All instruments/equipment must be operated in accordance with operating instructions as supplied by the manufacturer, unless otherwise specified in the work plan. Equipment checkout and calibration activities must occur prior to sampling/operation and they must be documented.

3. Field blanks should be collected at a rate of one per twenty samples or one per sampling event, whichever is greater

4. Lot blanks should be collected at a rate of at least two per lot

5. Collocated samples should be collected at the frequency of one per sampling event

For TEM analysis, the following QC procedures apply:

1. Examine lot blanks to determine the background asbestos structure concentration.

2. Examine field blanks to determine whether there is contamination by extraneous asbestos structures during specimen preparation or handling.

3. Examine laboratory blanks to determine if contamination is being introduced during critical phases of the laboratory program.

4. To determine if the laboratory can satisfactorily analyze samples of known asbestos structure concentrations, reference filters shall be examined. Reference filters should be maintained as part of the laboratory's Quality Assurance program.

5. To minimize subjective effects, some specimens should be recounted by a different microscopist.

6. Asbestos laboratories shall be accredited by the National Voluntary Laboratory Accreditation Program.

7. At this time, performance evaluation samples for asbestos in air are not commonly available for Removal Program Activities; however, they should be considered on a case-by-case basis.

10.0 DATA VALIDATION

Results of QC samples will be evaluated for contamination. This information will be utilized to qualify the environmental sample results accordingly with the project's data quality objectives.

11.0 HEALTH AND SAFETY

When working with potentially hazardous materials, follow U.S. EPA, OSHA, and corporate health and safety procedures. More specifically, when entering an unknown situation involving asbestos, a powered air-purifying respirator (PAPR) (full face-piece) is necessary in conjunction with high-efficiency particulate air (HEPA) filter cartridges. See applicable regulations for action levels, permissible exposure levels (PEL) and threshold limit values (TLV). If previous sampling indicates asbestos concentrations are below personal health and safety levels, then Level D personal protection is adequate.

For all ABS, appropriate PPE, including Tyvek coveralls, protective gloves and foot wear, and a respirator with HEPA filter cartridges (P-100 or equivalent) should be worn to protect participants. Details regarding PPE and other protective measures should be specified in the site-specific Health and Safety Plan. Special

consideration should be given to the physical safety of the event participants as well as heat stress associated with performing vigorous activities in impermeable clothing.

12.0 REFERENCES

40 CFR Part 763 Asbestos Worker Protection

ERT SOP #2015, *Asbestos Sampling*.

Berman, Mark, Anthony Kolk, 2000. DRAFT: Modified Elutriator Method for the Determination of Asbestos in Soil and Bulk Materials, Revision 1.

Hildemann, L. 2005. Major Sources of Personal Exposure to Airborne Particulate Matter, Seminar at EPA Region IX, March 15, 2005.

International Organization for Standardization. 1995. *Ambient air - Determination of asbestos fibres - Direct-transfer transmission electron microscopy method,* Method 10312.

International Organization for Standardization. 1999. *Ambient air - Determination of asbestos fibres - Indirect-transfer transmission electron microscopy method,* Method 13794.

Januch, Jed. 2005. EPA Region 10 Standard Operating Procedure 10-1EU-001, Standard Operating Procedure for Sampling Airborne Asbestos Fibers in a Laboratory Enclosure- a Qualitative Procedure.

McBride, SJ et al. 1999. Investigations of the proximity effect for pollutants in the indoor environment. J Expo Anal Environ Epidemiol. 1999 9(6): 602–621. Nov–Dec.

National Institute for Occupational Safety and Health. 1977. Occupational Exposure Sampling Strategy Manual, Publication No. 77-173, January 1977.

Omega. 1987. FL-1600 Series Rotameters, http://www.omega.com/Manuals/manualpdf/M0379.pdf accessed February 2007.

Patel, Ishwarbhai, "Gardening's Socioeconomic Impacts", Journal of Extension, Volume 29, Number 4, Winter 1991, http://www.joe.org/joe/1991 winter/a1.html, accessed January 2004.

Rodes, CE., Kamens, RM and Wiener, RW. 1995. Experimental considerations for the study of contaminant dispersion near the body. Am Ind Hyg Assoc J; 56: 535–45.

U.S. EPA. 2000. *Strategy for Research on Environmental Risks to Children*, EPA/600/R-00/068, Office of Research and Development.

U.S. EPA. 2004. *Clarifying Cleanup Goals and Identification of New Assessment Tools for Evaluating Asbestos at Superfund Cleanups*, OSWER Directive 9345.4-05, August 10, 2004.

Vodak, Mark C., Arthur J. Vrecenak, 2004. Transplanting Trees and Shrubs. New Jersey Agricultural Experimentation Center, Rutgers Cooperative Research & Extension, Agricultural Experiment Station, Rutgers, The State University of New Jersey, Fact Sheet 376.

13.0 APPENDICES

TABLE 1. Minimum Number of Grid Openings Required To Be Counted to Achieve a Given Analytical Sensitivity and Detection Limit. (Adapted from ISO 10312)

TABLE 2. Suggested Meteorological Station Specifications

TABLE 3. Soil Sampling Depth Based on Activities Performed

TABLE 1. Minimum Number of Grid Openings Required To Be Counted to Achieve a Given Analytical Sensitivity and Detection Limit. (Adapted from ISO 10312)

Analytical Sensitivity Structures/cc	Limit of Detection Structures/cc	Volume of Air Sampled (Liters)					
		500	1000	2000	3000	4000	5000
0.0001	0.0003	1066	533	267	178	134	107
0.0002	0.0006	533	267	134	89	67	54
0.0003	0.0009	358	178	89	60	45	36
0.0004	0.0012	267	134	67	45	34	27
0.0005	0.0015	214	107	54	36	27	22
0.0007	0.0021	153	77	39	26	20	16
0.001	0.003	107	54	27	18	14	11
0.002	0.006	54	27	14	9	7	6
0.003	0.009	36	18	9	6	5	4
0.004	0.012	27	14	7	5	4	4
0.005	0.015	22	11	6	4	4	4
0.007	0.021	16	8	4	4	4	4
0.01	0.030	11	6	4	4	4	4

TABLE 2. Suggested Meteorological Station Specifications

Variable	Accuracy	Resolution
Wind Speed (horizontal and vertical)	± (0.2 m/s + 5% of observed)	0.1 m/s
Wind Direction (azimuth and elevation)	± 5 degrees	1.0 degrees
Ambient Temperature	± 0.5" C	0.1" C
Precipitation	± 10% of observed or ± 0.5 mm	0.3 mm
Pressure	± 3 mb (0.3 kPa)	0.5 mb
Solar Radiation	± 5% of observed	10 W/m^2

m/s = meters per second
"C = degrees Centigrade
mm = millimeters
mb = millibar
W/m^2 = watts per square meter
kPa = kilopascal

TABLE 3. Soil Sampling Depth Based on Activities Performed

**Activity Based Sampling Scenario**	**Soil Sampling Depth**
Raking (metal garden rake)	Surface to 3 inches
Raking (leaf rake)	Surface to 2 inch
ATV riding	Surface to 2 inch
Rototilling	Surface to 12 inches
Digging	Surface to depth of excavation
Child Playing in the dirt	Surface to 3 inches
Weed Whacking	Surface to 2 inches
Lawn Mowing	Surface to 2 inch
Walking with Stroller	Surface to 2 inch
Two Bicycles	Surface to 2 inch
Activities on solid surfaces such as asphalt or concrete	Microvacuum ASTM D 5755

ASBESTOS SAMPLING

SOP#: 2015
DATE: 11/17/94
REV. #: 0.0

1.0 SCOPE AND APPLICATION

Asbestos has been used in many commercial products including building materials such as flooring tiles and sheet goods, paints and coatings, insulation, and roofing asphalts. These products and others may be found at hazardous waste sites hanging on overhead pipes, contained in drums, abandoned in piles, or as part of a structure. Asbestos tailing piles from mining operations can also be a source of ambient asbestos fibers. Asbestos is a known carcinogen and requires air sampling to assess airborne exposure to human health. This Standard Operating Procedure (SOP) provides procedures for asbestos air sampling by drawing a known volume of air through a mixed cellulose ester (MCE) filter. The filter is then sent to a laboratory for analysis. The U.S. Environmental Protection Agency/Environmental Response Team (U.S. EPA/ERT) uses one of four analytical methods for determining asbestos in air. These include: U.S. EPA's Environmental Asbestos Assessment Manual, Superfund Method for the Determination of Asbestos in Ambient Air for Transmission Electron Microscopy (TEM)[1]; U.S. EPA's Modified Yamate Method for TEM[2]; National Institute for Occupational Safety and Health (NIOSH) Method 7402 (direct method only) for TEM; and NIOSH Method 7400 for Phase Contrast Microscopy (PCM)[3]. Each method has specific sampling and analytical requirements (i.e., sample volume and flow rate) for determining asbestos in air.

The U.S. EPA/ERT typically follows procedures outlined in the TEM methods for determining mineralogical types of asbestos in air and for distinguishing asbestos from non-asbestos minerals. The Phase Contrast Microscopy (PCM) method is used by U.S. EPA/ERT as a screening tool since it is less costly than TEM. PCM cannot distinguish asbestos from non-asbestos fibers, therefore the TEM method may be necessary to confirm analytical results. For example, if an action level for the presence of fibers has been set and PCM analysis indicates that the action level has been exceeded, then TEM analysis can be used to quantify and identify asbestos structures through examination of their morphology crystal structures (through electron diffraction), and elemental composition (through energy dispersive X-ray analysis). In this instance samples should be collected for both analyses in side by side sampling trains (some laboratories are able to perform PCM and TEM analysis from the same filter). The Superfund method is designed specifically to provide results suitable for supporting risk assessments at Superfund sites, it is applicable to a wide range of ambient air situations at hazardous waste sites. U.S. EPA's Modified Yamate Method for TEM is also used for ambient air sampling due to high volume requirements. The PCM and TEM NIOSH analytical methods require lower sample volumes and are typically used indoors; however, ERT will increase the volume requirement for outdoor application.

Other Regulations pertaining to asbestos have been promulgated by U.S. EPA and OSHA. U.S. EPA's National Emission Standards for Hazardous Air Pollutants (NESHAP) regulates asbestos-containing waste materials. NESHAP establishes management practices and standards for the handling of asbestos and emissions from waste disposal operations (40 CFR Part 61, Subparts A and M). U.S. EPA's 40 CFR 763 (July 1, 1987)[4] and its addendum 40 CFR 763 (October 30, 1987)[4] provide comprehensive rules for the asbestos abatement industry. State and local regulations on these issues vary and may be more stringent than federal requirements. The OSHA regulations in 29 CFR 1910.1001 and 29 CFR 1926.58 specify work practices and safety equipment such as respiratory protection and protective clothing when handling asbestos. The OSHA standard for an 8-hour, time-weighted average (TWA) is 0.2 fibers/cubic centimeters of air. This standard pertains to fibers with a length-to-width ratio of 3 to 1 with a fiber length >5 µm[5,6]. An action level of 0.1 fiber/cc (one-half the OSHA standard) is the level U.S. EPA has established in which employers must initiate such activities as air monitoring, employee training, and

medical surveillance[5,6].

These are standard (i.e., typically applicable) operating procedures which may be varied or changed as required, dependent upon site conditions, equipment limitations or limitations imposed by the procedure. In all instances, the ultimate procedures employed should be documented and associated with the final report.

Mention of trade names or commercial products does not constitute U.S. EPA endorsement or recommendation for use.

2.0 METHOD SUMMARY

Prior to sampling, the site should be characterized by identifying on-site as well as off-site sources of airborne asbestos. The array of sampling locations and the schedule for sample collection, is critical to the success of an investigation. Generally, sampling strategies to characterize a single point source are fairly straightforward, while multiple point sources and area sources increase the complexity of the sampling strategy. It is not within the scope of this SOP to provide a generic asbestos air sampling plan. Experience, objectives, and site characteristics will dictate the sampling strategy.

During a site investigation, sampling stations should be arranged to distinguish spatial trends in airborne asbestos concentrations. Sampling schedules should be fashioned to establish temporal trends. The sampling strategy typically requires that the concentration of asbestos at the source (worst case) or area of concern (downwind), crosswind, as well as background (upwind) contributions be quantified. See Table 1 (Appendix A) for U.S. EPA/ERT recommended sampling set up for ambient air. Indoor asbestos sampling requires a different type of strategy which is identified in Table 2 (Appendix A). It is important to establish background levels of contaminants in order to develop a reference point from which to evaluate the source data. Field blanks and lot blanks can be utilized to determine other sources.

Much information can be derived from each analytical method previously mentioned. Each analytical method has specific sampling requirements and produce results which may or may not be applicable to a specific sampling effort. The site sampling objectives should be carefully identified so as to select the most appropriate analytical method. Additionally, some preparation (i.e., lot blanks results) prior to site sampling may be required, these requirements are specified in the analytical methods.

3.0 SAMPLE PRESERVATION, CONTAINERS, HANDLING, AND STORAGE

3.1 Sample Preservation

No preservation is required for asbestos samples.

3.2 Sample Handling, Container and Storage Procedures

1. Place a sample label on the cassette indicating a unique sampling number. Do not put sampling cassettes in shirt or coat pockets as the filter can pick up fibers. The original cassette box is used to hold the samples.

2. Wrap the cassette individually in a plastic sample bag. Each bag should be marked indicating sample identification number, total volume, and date.

3. The wrapped sampling cassettes should be placed upright in a rigid container so that the cassette cap is on top and cassette base is on bottom. Use enough packing material to prevent jostling or damage. Do not use vermiculite as packing material for samples. If possible, hand carry to lab.

4. Provide appropriate documentation with samples (i.e., chain of custody and requested analytical methodology).

4.0 INTERFERENCES AND POTENTIAL PROBLEMS

Flow rates exceeding 16 liters/minute (L/min) which could result in filter destruction due to (a) failure of its physical support under force from the increased pressure drop; (b) leakage of air around the filter mount so that the filter is bypassed, or (c) damage to the asbestos structures due to increased impact velocities.

4.1 U.S. EPA's Superfund Method

4.1.1 Direct-transfer TEM Specimen Preparation Methods

Direct-Transfer TEM specimen preparation methods have the following significant interferences:

- The achievable detection limit is restricted by the particulate density on the filter, which in turn is controlled by the sampled air volume and the total suspended particulate concentration in the atmosphere being sampled.

- The precision of the result is dependent on the uniformity of the deposit of asbestos structures on the sample collection filter.

- Air samples must be collected so that they have particulate and fiber loadings within narrow ranges. If too high a particulate loading occurs on the filter, it is not possible to prepare satisfactory TEM specimens by a direct-transfer method. If too high a fiber loading occurs on the filter, even if satisfactory TEM specimens can be prepared, accurate fiber counting will not be possible.

4.1.2 Indirect TEM Specimen Preparation Methods

Indirect TEM specimen preparation methods have the following interferences:

- The size distribution of asbestos structures is modified.

- There is increased opportunity for fiber loss or introduction of extraneous contamination.

- When sample collection filters are ashed, any fiber contamination in the filter medium is concentrated on the TEM specimen grid.

It can be argued that direct methods yield an under-estimate of the asbestos structure concentration because many of the asbestos fibers present are concealed by other particulate material with which they are associated. Conversely, indirect methods can be considered to yield an over-estimate because some types of complex asbestos structures disintegrate during the preparation, resulting in an increase in the numbers of structures counted.

4.2 U.S. EPA's Modified Yamate Method for TEM

High concentrations of background dust interfere with fiber identification.

4.3 NIOSH Method for TEM

Other amphibole particles that have aspect ratios greater than 3:1 and elemental compositions similar to the asbestos minerals may interfere in the TEM analysis. Some non-amphibole minerals may give electron diffraction patterns similar to amphiboles. High concentrations of background dust interfere with fiber identification.

4.4 NIOSH Method for PCM

PCM cannot distinguish asbestos from non-asbestos fibers; therefore, all particles meeting the counting criteria are counted as total asbestos fibers. Fiber less than 0.25 um in length will not be detected by this method. High levels of non-fibrous dust particles may obscure fibers in the field of view and increase the detection limit.

5.0 EQUIPMENT/MATERIALS

5.1 Sampling Pump

The constant flow or critical orifice controlled sampling pump should be capable of a flow-rate and pumping time sufficient to achieve the desired volume of air sampled.

The lower flow personal sampling pumps generally provide a flow rate of 20 cubic centimeters/minute (cc/min) to 4 L/min. These pumps are usually battery powered. High flow pumps are utilized when flow rates between 2 L/min to 20 L/min are required. High flow pumps are used for short sampling periods so as to obtain the desired sample volume. High flow pumps usually run on AC power and can be plugged into a nearby outlet. If an outlet is not available then a generator should be obtained. The generator should be positioned downwind from the sampling pump. Additional voltage may be required if more than one pump is plugged into the same generator. Several

electrical extension cords may be required if sampling locations are remote.

The recommended volume for the Superfund method (Phase I) requires approximately 20 hours to collect. Such pumps typically draw 6 amps at full power so that 2 lead/acid batteries should provide sufficient power to collect a full sample. The use of line voltage, where available, eliminates the difficulties associated with transporting stored electrical energy.

A stand should be used to hold the filter cassette at the desired height for sampling and the filter cassette shall be isolated from the vibrations of the pump.

5.2 Filter Cassette

The cassettes are purchased with the required filters in position, or can be assembled in a laminar flow hood or clean area. When the filters are in position, a shrink cellulose band or adhesive tape should be applied to cassette joints to prevent air leakage.

5.2.1 TEM Cassette Requirements

Commercially available field monitors, comprising 25 mm diameter three-piece cassettes, with conductive extension cowls shall be used for sample collection. The cassette must be new and not previously used. The cassette shall be loaded with an MCE filter of pore size 0.45 µm, and supplied from a lot number which has been qualified as low background for asbestos determination. The cowls should be constructed of electrically conducting material to minimize electrostatic effects. The filter shall be backed by a 5 µm pore size MCE filter (Figure 1, Appendix B).

5.2.2 PCM Cassette Requirements

NIOSH Method 7400, PCM involves using a 0.8 to 1.2 µm mixed cellulose ester membrane, 25 mm diameter, 50 mm conductive cowl on cassette (Figure 2, Appendix B). Some labs are able to perform PCM and TEM analysis on the same filter; however, this should be discussed with the laboratory prior to sampling.

5.3 Other Equipment

C Inert tubing with glass cyclone and hose barb
C Whirlbags (plastic bags) for cassettes
C Tools - small screw drivers
C Container - to keep samples upright
C Generator or electrical outlet (may not be required)
C Extension cords (may not be required)
C Multiple plug outlet
C Sample labels
C Air data sheets
C Chain of Custody records

6.0 REAGENTS

Reagents are not required for the preservation of asbestos samples.

7.0 PROCEDURES

7.1 Air Volumes and Flow Rates

Sampling volumes are determined on the basis of how many fibers need to be collected for reliable measurements. Therefore, one must estimate how many airborne fibers may be in the sampling location.

Since the concentration of airborne aerosol contaminants will have some effect on the sample, the following is a suggested criteria to assist in selecting a flow rate based on real-time aerosol monitor (RAM) readings in milligrams/cubic meter (mg/m^3).

	Concentration	Flow Rate
C Low RAM readings:	<6.0 mg/m^3	11-15. L/min
C Medium RAM readings:	>6.0 mg/m_3	7.5 L/min
C High RAM readings:	>10. mg/m^3	2.5 L/min

In practice, pumps that are available for environmental sampling at remote locations operate under a maximum load of approximately 12 L/min.

7.1.1 U.S. EPA's Superfund Method

The Superfund Method incorporates an indirect preparation procedure to provide flexibility in the amount of deposit that be can be tolerated on the sample filter and to allow for the selective concentration of asbestos prior to analysis. To minimize contributions to background contamination from asbestos present in the plastic matrices of membrane filters while allowing for sufficient quantities of asbestos to be collected, this method also requires the collection of a larger volume of air per unit area of filter than has traditionally been collected

for asbestos analysis. Due to the need to collect large volumes of air, higher sampling flow rates are recommended in this method than have generally been employed for asbestos sampling in the past. As an alternative, samples may be collected over longer time intervals. However, this restricts the flexibility required to allow samples to be collected while uniform meteorological conditions prevail.

The sampling rate and the period of sampling should be selected to yield as high a sampled volume as possible, which will minimize the influence of filter contamination. Wherever possible, a volume of 15 cubic meters (15,000 L) shall be sampled for those samples intended for analysis only by the indirect TEM preparation method (Phase 1 samples). For those samples to be prepared by both the indirect and the direct specimen preparation methods (Phase 2 samples), the volumes must be adjusted so as to provide a suitably-loaded filter for the direct TEM preparation method. One option is to collect filters at several loadings to bracket the estimated optimum loading for a particular site. Such filters can be screened in the laboratory so that only those filters closest to optimal loading are analyzed. It has been found that the volume cannot normally exceed 5 cubic meters (5000 L) in an urban or agricultural area, and 10 cubic meters (10,000 L) in a rural area for samples collected on a 25 mm filter and prepared by a direct-transfer technique.

An upper limit to the range of acceptable flow rates for this method is 15 L/min. At many locations, wind patterns exhibit strong diurnal variations. Therefore, intermittent sampling (sampling over a fixed time interval repeated over several days) may be necessary to accumulate 20 hours of sampling time over constant wind conditions. Other sampling objectives also may necessitate intermittent sampling. The objective is to design a sampling schedule so that samples are collected under uniform conditions throughout the sampling interval. This method provides for such options. Air volumes collected on Phase I samples are maximized (<16 L/min). Air volumes collected on Phase 2 samples are limited to provide optimum loading for filters to be prepared by a direct-transfer procedure.

7.1.2 U.S. EPA's Modified Yamate Method for TEM

U.S. EPA's TEM method requires a minimum volume of 560 L and a maximum volume of 3,800 L in order to obtain an analytical sensitivity of 0.005 structures/cc. The optimal volume for TEM is 1200 L to 1800 L. These volumes are determined using a 200 mesh EM grid opening with a 25-mm filter cassette. Changes in volume would be necessary if a 37-mm filter cassette is used since the effective area of a 25 mm (385 sq mm) and 37 mm (855 sq m) differ.

7.1.3 NIOSH Method for TEM and PCM

The minimum recommended volume for TEM and PCM is 400 L at 0.1 fiber/cc. Sampling time is adjusted to obtain optimum fiber loading on the filter. A sampling rate of 1 to 4 L/min for eight hours (700 to 2800 L) is appropriate in non-dusty atmospheres containing 0.1 fiber/cc. Dusty atmospheres i.e., areas with high levels of asbestos, require smaller sample volumes (<400 L) to obtain countable samples.

In such cases, take short, consecutive samples and average the results over the total collection time. For documenting episodic exposures, use high flow rates (7 to 16 L/min) over shorter sampling times. In relatively clean atmospheres where targeted fiber concentrations are much less than 0.1 fiber/cc, use larger sample volumes (3,000 to 10,000 L) to achieve quantifiable loadings. Take care, however, not to overload the filter with background dust. If > 50% of the filter surface is covered with particles, the filter may be too overloaded to count and will bias the measured fiber concentration. Do not exceed 0.5 mg total dust loading on the filter.

7.2 Calibration Procedures

In order to determine if a sampling pump is measuring the flow rate or volume of air correctly, it is necessary to calibrate the instrument. Sampling pumps should be calibrated immediately before and after each use. Preliminary calibration should be conducted using a primary calibrator such as a soap bubble type calibrator, (e.g., a Buck Calibrator, Gilibrator, or equivalent primary calibrator) with a representative filter cassette installed between the pump and the calibrator. The representative sampling cassette can be reused for calibrating other pumps that will be used for asbestos sampling. The same cassette lot used for sampling should also be used for the calibration. A sticker should be affixed to the outside of the extension cowl marked "Calibration Cassette."

A rotameter can be used provided it has been recently precalibrated with a primary calibrator. Three separate constant flow calibration readings should be obtained both before sampling and after sampling. Should the flow rate change by more than 5% during the sampling period, the average of the pre- and post-calibration rates will be used to calculate the total sample volume. The sampling pump used shall provide a non-fluctuating air-flow through the filter, and shall maintain the initial volume flow-rate to within ± 10% throughout the sampling period. The mean value of these flow-rate measurements shall be used to calculate the total air volume sampled. A constant flow or critical orifice controlled pump meets these requirements. If at any time the measurement indicates that the flow-rate has decreased by more than 30%, the sampling shall be terminated. Flexible tubing is used to connect the filter cassette to the sampling pump.Sampling pumps can be calibrated prior to coming on-site so that time is saved when performing on-site calibration.

7.2.1 Calibrating a Personal Sampling Pump with an Electronic Calibrator

1. See Manufacturer's manual for operational instructions.

2. Set up the calibration train as shown in (Figure 3, Appendix B) using a sampling pump, electronic calibrator, and a representative filter cassette. The same lot sampling cassette used for sampling should also be used for calibrating.

3. To set up the calibration train, attach one end of the PVC tubing (approx. 2 foot) to the cassette base; attach the other end of the tubing to the inlet plug on the pump. Another piece of tubing is attached from the cassette cap to the electronic calibrator.

4. Turn the electronic calibrator and sampling pump on. Create a bubble at the bottom of the flow chamber by pressing the bubble initiate button. The bubble should rise to the top of the flow chamber. After the bubble runs its course, the flow rate is shown on the LED display.

5. Turn the flow adjust screw or knob on the pump until the desired flow rate is attained.

6. Perform the calibration three times until the desired flow rate of ± 5% is attained.

7.2.2 Calibrating a Rotameter with an Electronic Calibrator

1. See manufacturer's manual for operational instructions.

2. Set up the calibration train as shown in (Figure 4, Appendix B) using a sampling pump, rotameter, and electronic calibrator.

3. Assemble the base of the flow meter with the screw provided and tighten in place. The flow meter should be mounted within 6° vertical.

4. Turn the electronic calibrator and sampling pump on.

5. Create a bubble at the bottom of the flow chamber by pressing the bubble initiate button. The bubble should rise to the top of the flow chamber. After the bubble runs its course, the flow rate is shown on the LED display.

6. Turn the flow adjust screw or knob on the pump until the desired flow rate is attained.

7. Record the electronic calibrator flow rate reading and the corresponding rotameter reading. Indicate these values on the rotameter (sticker). The rotameter should be able to work within the desired flow range. Readings can also be calibrated for 10 cm^3 increments for Low Flow rotameters, 500 cm^3 increments for medium flow rotameters and 1 liter increments for high flow rotameters.

8. Perform the calibration three times until the desired flow rate of ± 5% is attained. Once on site, a secondary calibrator, i.e., rotameter may be used to calibrate sampling pumps.

7.2.3 Calibrating a Personal Sampling Pump with a Rotameter

1. See manufacturer's manual for Rotameter's Operational Instructions.

2. Set up the calibration train as shown in (Figure 5, Appendix B) using a rotameter, sampling pump, and a representative sampling cassette.

3. To set up the calibration train, attach one end of the PVC tubing (approx. 2 ft) to the cassette base; attach the other end of the tubing to the inlet plug on the pump. Another piece of tubing is attached from the cassette cap to the rotameter.

4. Assemble the base of the flow meter with the screw provided and tighten in place. The flow meter should be mounted within 6° vertical.

5. Turn the sampling pump on.

6. Turn the flow adjust screw (or knob) on the personal sampling pump until the float ball on the rotameter is lined up with the precalibrated flow rate value. A sticker on the rotameter should indicate this value.

7. A verification of calibration is generally performed on-site in the clean zone immediately prior to the sampling.

7.3. Meteorology

It is recommended that a meteorological station be established. If possible, sample after two to three days of dry weather and when the wind conditions are at 10 mph or greater. Record wind speed, wind direction, temperature, and pressure in a field logbook. Wind direction is particularly important when monitoring for asbestos downwind from a fixed source.

7.4 Ambient Sampling Procedures

7.4.1 Pre-site Sampling Preparation

1. Determine the extent of the sampling effort, the sampling methods to be employed, and the types and amounts of equipment and supplies needed.

2. Obtain necessary sampling equipment and ensure it is in working order and fully charged (if necessary).

3. Perform a general site survey prior to site entry in accordance with the site specific Health and Safety plan.

4. Once on-site the calibration is performed in the clean zone. The calibration procedures are listed in Section 7.2.

5. After calibrating the sampling pump, mobilize to the sampling location.

7.4.2 Site Sampling

1. To set up the sampling train, attach the air intake hose to the cassette base. Remove the cassette cap (Figure 6 and 7, Appendix B). The cassette should be positioned downward, perpendicular to the wind

2. If AC or DC electricity is required then turn it on. If used, the generator should be placed 10 ft. downwind from the sampling pump.

3. Record the following in a field logbook: date, time, location, sample identification number, pump number, flow rate, and cumulative time.

4. Turn the pump on. Should intermittent sampling be required, sampling filters must be covered between active periods of sampling. To cover the sample filter: turn the cassette to face upward, place the cassette cap on the cassette, remove the inlet plug from the cassette cap, attach a rotameter to the inlet opening of the cassette cap to measure the flow rate, turn off the sampling pump, place the inlet plug into the inlet opening on the cassette cap. To resume sampling: remove the inlet plug, turn on the sampling pump, attach a rotameter to measure the flow rate, remove the cassette cap, replace the inlet plug in the cassette cap and invert the cassette, face downward and perpendicular to the wind.

5. Check the pump at sampling midpoint if sampling is longer than 4 hours. The generators may need to be regased depending on tank size. If a filter darkens in appearance or if loose dust is seen in the filter, a second sample should be started.

6. At the end of the sampling period, orient the cassette up, turn the pump off.

7. Check the flow rate as shown in Section 7.2.3. When sampling open-faced, the sampling cap should be replaced before post calibrating. Use the same cassette used for sampling for post calibration (increase dust/fiber loading may have altered the flow rate.

8. Record the post flow rate.

9. Record the cumulative time or run.

10. Remove the tubing from the sampling cassette. Still holding the cassette upright, replace the inlet plug on the cassette cap and the outlet plug on the cassette base.

7.4.3. Post Site Sampling

1. Follow handling procedures in Section 3.2, steps 1-4.

2. Obtain an electronic or hard copy of meteorological data which occurred during the sampling event. Record weather: wind speed, ambient temperature, wind direction, and precipitation. Obtaining weather data several days prior to the sampling event can also be useful.

7.5 Indoor Sampling Procedures

PCM analysis is used for indoor air samples. When analysis shows total fiber count above the OSHA action level 0.1 f/cc then TEM (U.S. EPA's Modified Yamate Method) is used to identify asbestos from non-asbestos fibers.

Sampling pumps should be placed four to five feet above ground level away from obstructions that may influence air flow. The pump can be placed on a table or counter. Refer to Table 2 (Appendix A) for a summary of indoor sampling locations and rationale for selection.

Indoor sampling utilizes high flow rates to increased sample volumes (2000 L for PCM and 2800 to 4200 L for TEM) in order to obtain lower detection limits below the standard, (i.e., 0.01 f/cc or lower [PCM] and 0.005 structures/cc or lower [TEM]).

7.5.1 Aggressive Sampling Procedures

Sampling equipment at fixed locations may fail to detect the presence of asbestos fibers. Due to limited air movement, many fibers may settle out of the air onto the floor and other surfaces and may not be captured on the filter. In the past, an 8-hour sampling period was recommended to cover various air circulation conditions. A quicker and more effective way to capture asbestos fibers is to circulate the air artificially so that the fibers remain airborne during sampling. The results from this sampling option typifies worst case condition. This is referred to as aggressive air sampling for asbestos. Refer to Table 2 for sample station locations.

1. Before starting the sampling pumps, direct forced air (such as a 1-horsepower leaf blower or large fan) against walls, ceilings, floors, ledges, and other surfaces in the room to initially dislodge fibers from surfaces. This should take at least 5 minutes per 1000 sq. ft. of floor.

2. Place a 20-inch fan in the center of the room. (Use one fan per 10,000 cubic feet of room space.) Place the fan on slow speed and point it toward the ceiling.

3. Follow procedures in Section 7.4.1 and 7.4.2 (Turn off the pump and then the fan(s) when sampling is complete.).

4. Follow handling procedures in Section 3.2, steps 1-4.

8.0 CALCULATIONS

The sample volume is calculated from the average flow rate of the pump multiplied by the number of minutes the pump was running (volume = flow rate X time in minutes). The sample volume should be submitted to the laboratory and identified on the chain of custody for each sample (zero for lot, field and trip blanks).

The concentration result is calculated using the sample volume and the numbers of asbestos structures reported after the application of the cluster and matrix counting criteria.

9.0 QUALITY ASSURANCE/ QUALITY CONTROL

Follow all QA/QC requirements from the laboratories as well as the analytical methods.

9.1 TEM Requirements

1. Examine lot blanks to determine the background asbestos structure concentration.

2. Examine field blanks to determine whether there is contamination by extraneous asbestos structures during specimen preparation.

3. Examine of laboratory blanks to determine if contamination is being introduced during critical phases of the laboratory program.

4. To determine if the laboratory can satisfactorily analyze samples of known asbestos structure concentrations, reference filters shall be examined. Reference filters should be maintained as part of the laboratory's Quality Assurance program.

5. To minimize subjective effects, some specimens should be recounted by a different microscopist.

6. Asbestos laboratories shall be accredited by the National Voluntary Laboratory Accreditation Program.

7. At this time, performance evaluation samples for asbestos in air are not available for Removal Program Activities.

9.2 PCM Requirements

1. Examine reference slides of known concentration to determine the analyst's ability to satisfactorily count fibers. Reference slides should be maintained as part of the laboratory's quality assurance program.

2. Examine field blanks to determine if there is contamination by extraneous structures during sample handling.

3. Some samples should be relabeled then submitted for counting by the same analyst to determine possible bias by the analyst.

4. Participation in a proficiency testing program such as the AIHA-NIOSH proficiency analytical testing (PAT) program.

10.0 DATA VALIDATION

Results of quality control samples will be evaluated for contamination. This information will be utilized to qualify the environmental sample results accordingly with the project's data quality objectives.

11.0 HEALTH AND SAFETY

When working with potentially hazardous materials, follow U.S. EPA, OSHA, and corporate health and safety procedures. More specifically, when entering an unknown situation involving asbestos, a powered air purifying respirator (PAPR) (full face-piece) is necessary in conjunction with HEPA filter cartridges. See applicable regulations for action level, PEL, TLV, etc. If previous sampling indicates asbestos concentrations are below personal health and safety levels, then Level D personal protection is adequate.

12.0 REFERENCES

(1) Environmental Asbestos Assessment Manual, Superfund Method for the Determination of Asbestos in Ambient Air, Part 1: Method, EPA/540/2-90/005a, May 1990, and Part 2: Technical Background Document, EPA/540/2-90/005b, May 1990.

(2) Methodology for the Measurement of Airborne Asbestos by Electron Microscopy, EPA's Report No. 68-02-3266, 1984, G. Yamate, S.C. Agarwal, and R. D. Gibbons.

(3) National Institute for Occupational Safety and Health. NIOSH Manual of Analytical Method. Third Edition. 1987.

(4) U.S. Environmental Protection Agency. Code of Federal Regulations 40 CFR 763. July 1, 1987. Code of Federal Regulations 40 CFR 763 Addendum. October 30, 1987.

(5) U.S. Environmental Protection Agency. Asbestos-Containing Materials in Schools; Final Rule and Notice. 52 FR 41826.

(6) Occupational Safety and Health Administration. Code of Federal Regulations 29 CFR 1910.1001. Washington, D.C. 1987.

APPENDIX A

Tables

TABLE 1.<br><br>SAMPLE STATIONS FOR OUTDOOR SAMPLING		
Sample Station Location	Sample Numbers	Rationale
Upwind/Background[1]	Collect a minimum of two simultaneous upwind/background samples 30° apart from the prevailing windlines.	Establishes background fiber levels.
Downwind	Deploy a minimum of 3 sampling stations in a 180 degree arc downwind from the source.	Indicates if asbestos is leaving the site.
Site Representative and/or Worst Case	Obtain one site representative sample which shows average condition on-site or obtain worst case sample (optional).	Verify and continually confirm and document selection of proper levels of worker protection.

(1) More than one background station may be required if the asbestos originates from different sources.

APPENDIX A (Cont'd)

Tables

TABLE 2<br><br>SAMPLE STATIONS FOR INDOOR SAMPLING		
Sample Station Location	Sample Numbers	Rationale
Indoor Sampling	If a work site is a single room, disperse 5 samplers throughout the room.<br><br>If the work site contains up to 5 rooms, place at least one sampler in each room.<br><br>If the work site contains more than 5 rooms, select a representative sample of the rooms.	Establishes representative samples from a homogeneous area.
Upwind/Background	If outside sources are suspected, deploy a minimum of two simultaneous upwind/background samples 30° apart from the prevailing windlines.	Establish whether indoor asbestos concentrations are coming from an outside source.
Worst Case	Obtain one worst case sample, i.e., aggressive sampling (optional).	Verify and continually confirm and document selection of proper levels of worker protection.

APPENDIX B

Figures

FIGURE 1. Transmission Electron Microscopy Filter Cassette

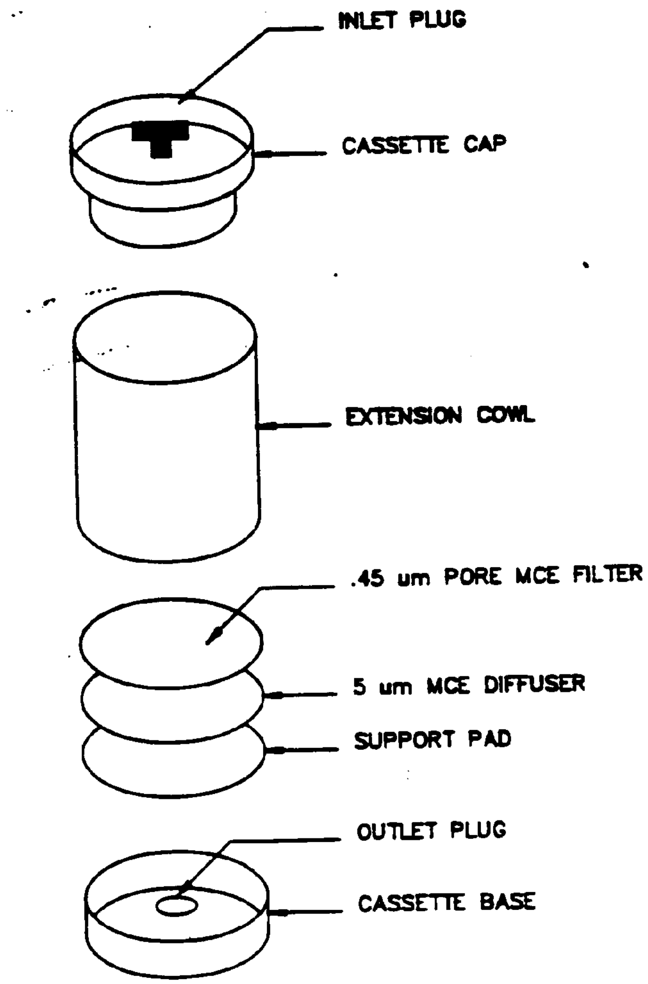

APPENDIX B (Cont'd)

Figures

FIGURE 2. Phase Contrast Microscopy Filter Cassette

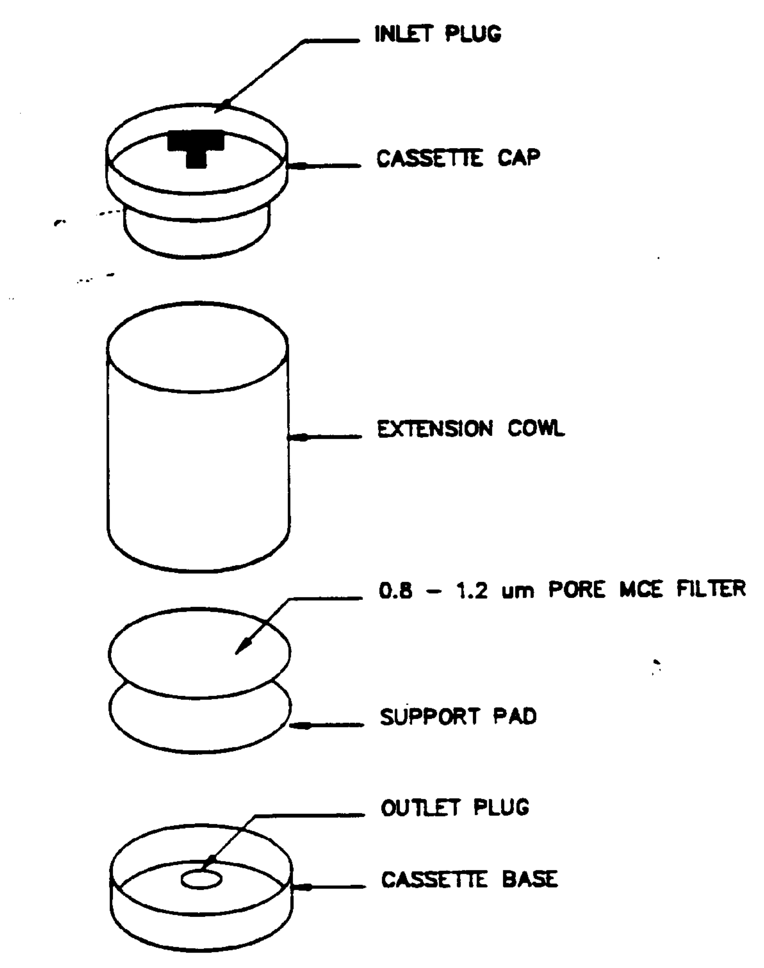

APPENDIX B (Cont'd)

Figures

FIGURE 3. Calibrating a Personal Sampling Pump with a Bubble Meter

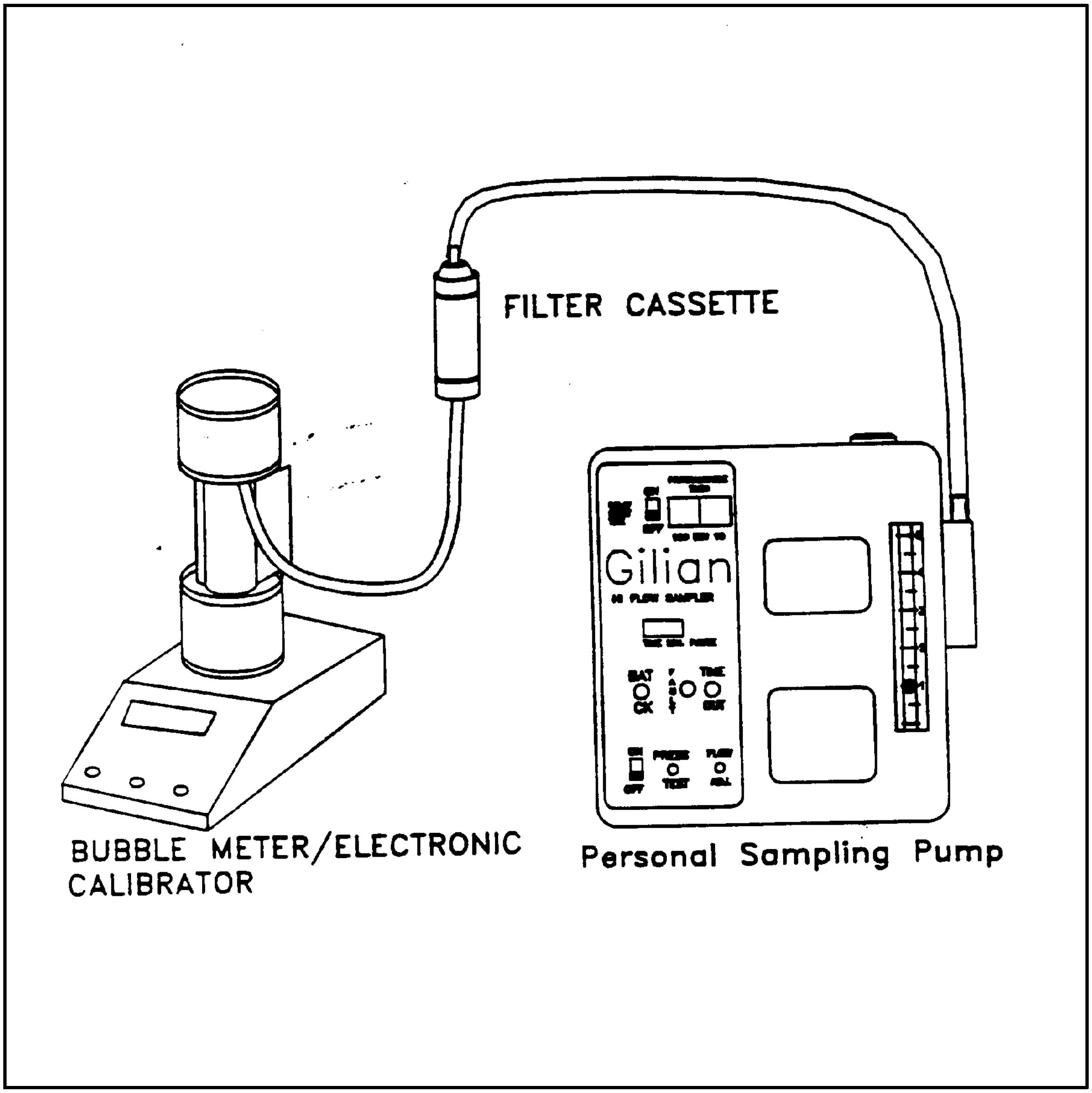

APPENDIX B (Cont'd)

Figures

FIGURE 4. Calibrating a Rotameter with a Bubble Meter

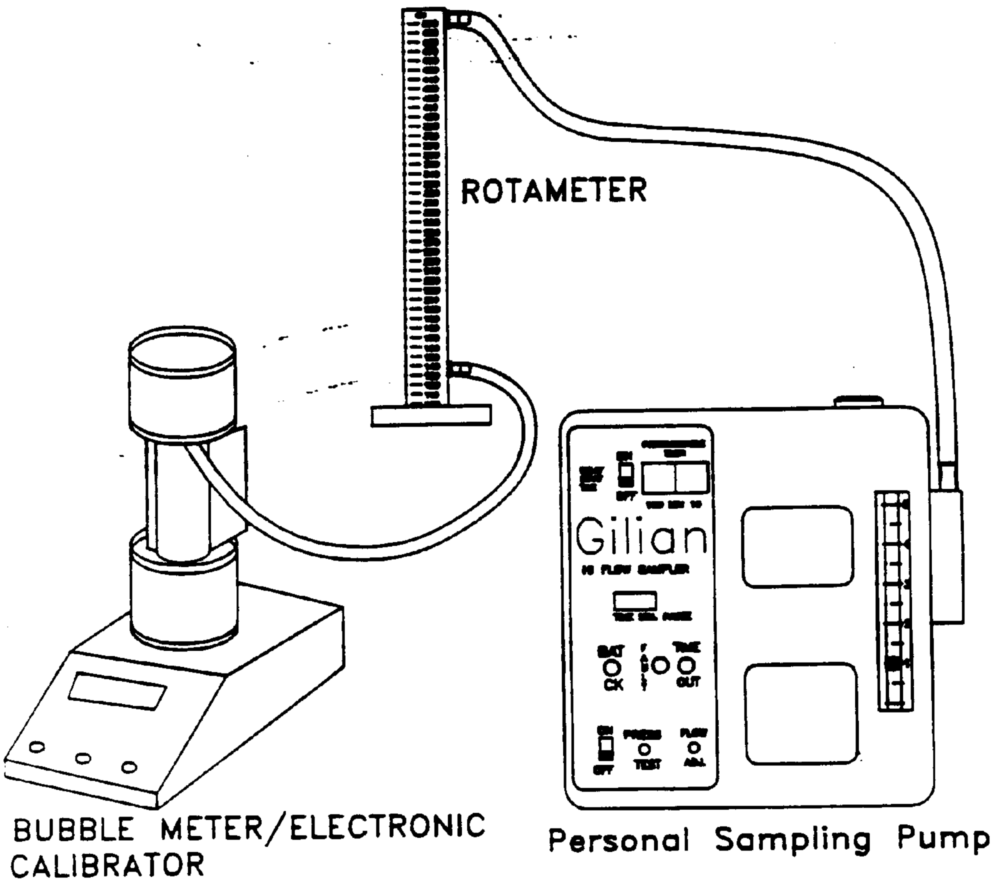

APPENDIX B (Cont'd)

Figures

FIGURE 5. Calibrating a Sampling Pump with a Rotameter

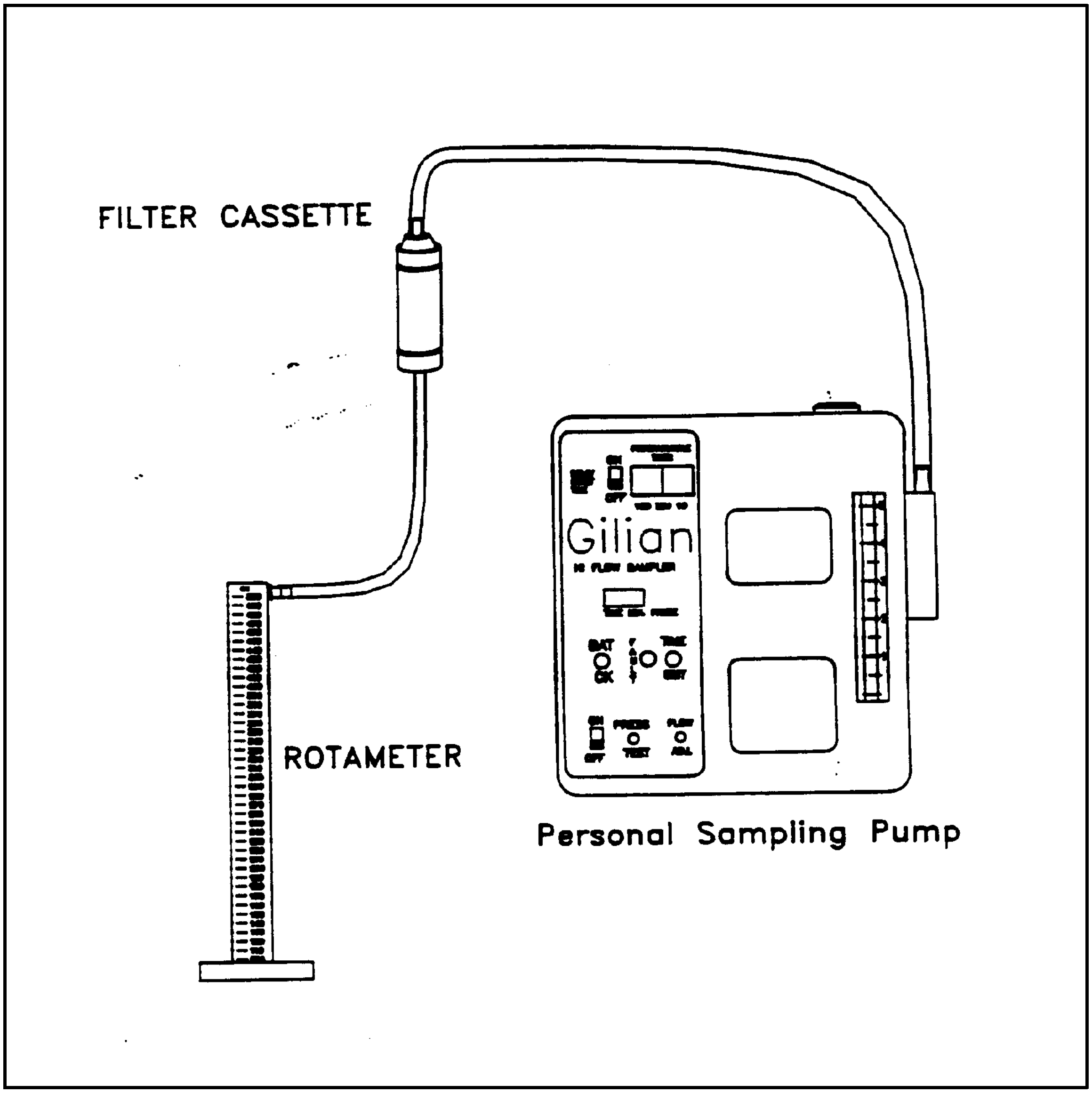

APPENDIX B (Cont'd)

Figures

FIGURE 6. Personal Sampling Train for Asbestos

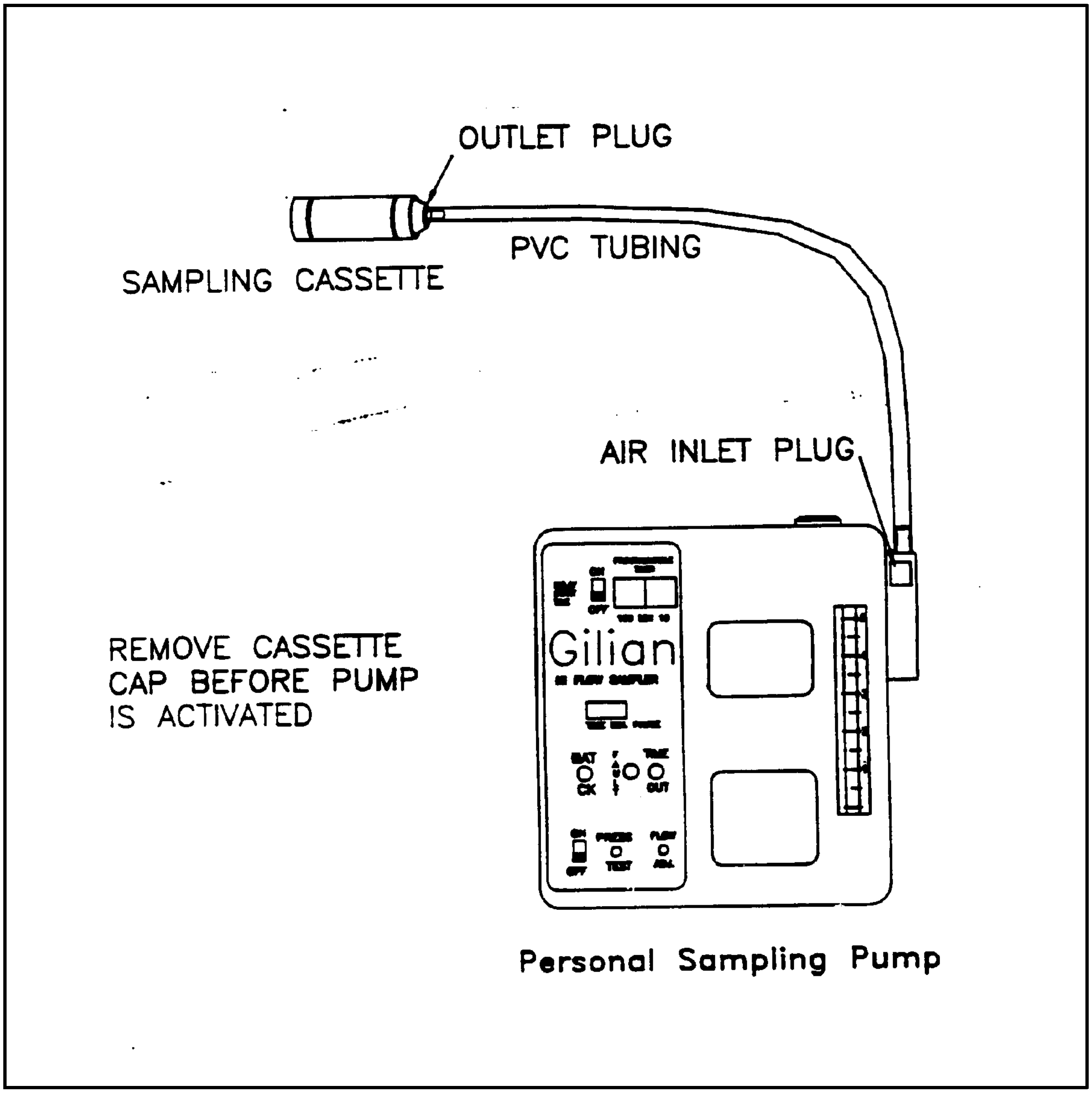

APPENDIX B (Cont'd)

Figures

FIGURE 7. High Flow Sampling Train for Asbestos

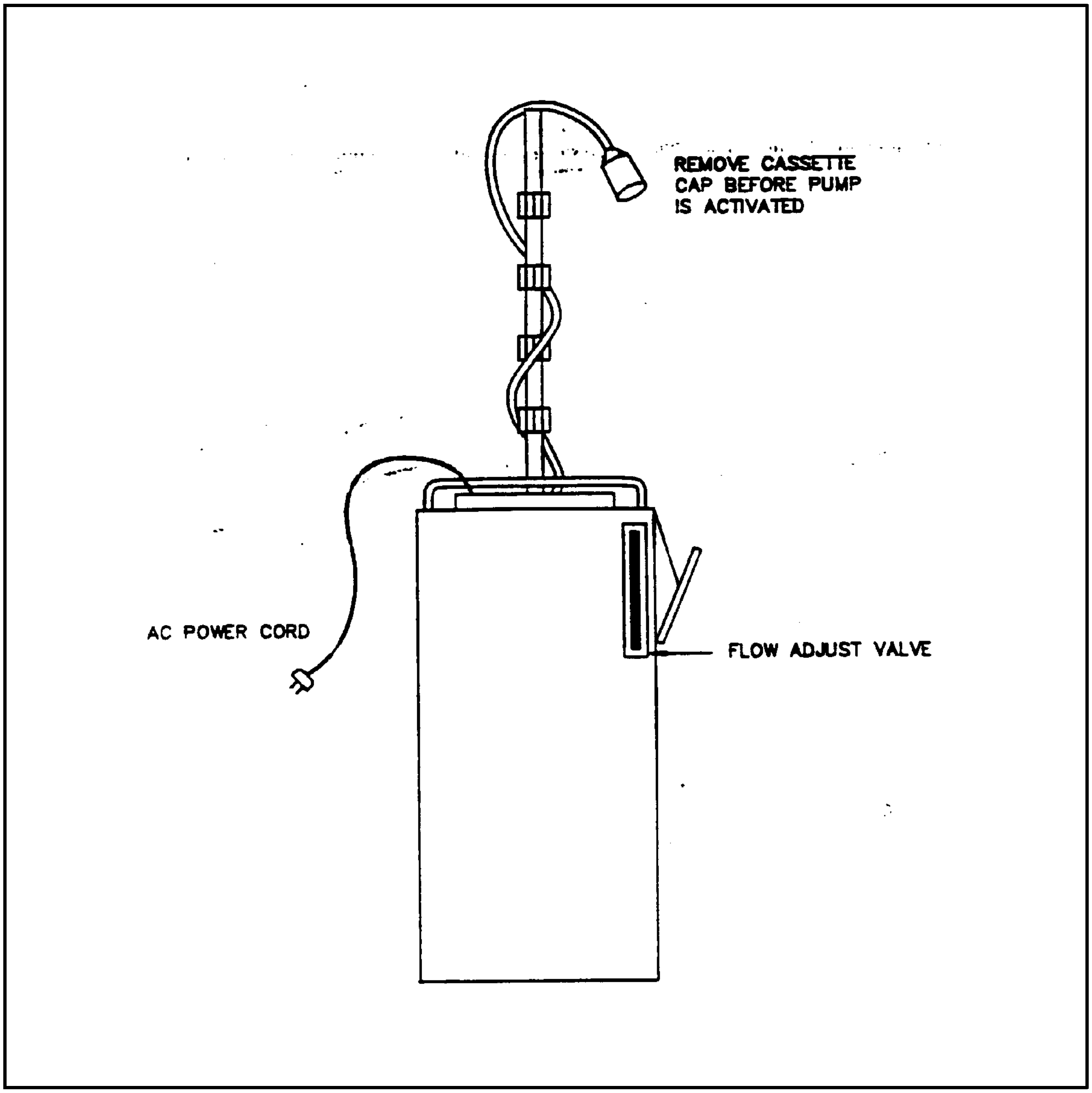

U. S. EPA ENVIRONMENTAL RESPONSE TEAM

STANDARD OPERATING PROCEDURES

SOIL SAMPLING

CONTENTS

1.0 SCOPE AND APPLICATION

2.0 METHOD SUMMARY

3.0 SAMPLE PRESERVATION, CONTAINERS, HANDLING, AND STORAGE

4.0 POTENTIAL PROBLEMS

5.0 EQUIPMENT

6.0 REAGENTS

7.0 PROCEDURES

- 7.1 Preparation
- 7.2 Sample Collection
  - 7.2.1 Surface Soil Samples
  - 7.2.2 Sampling at Depth with Augers and Thin Wall Tube Samplers
  - 7.2.3 Sampling at Depth with a Trier
  - 7.2.4 Sampling at Depth with a Split Spoon (Barrel) Sampler
  - 7.2.5 Test Pit/Trench Excavation

8.0 CALCULATIONS

9.0 QUALITY ASSURANCE/QUALITY CONTROL

10.0 DATA VALIDATION

11.0 HEALTH AND SAFETY

12.0 REFERENCES

13.0 APPENDIX
Figures

SUPERCEDES: SOP #2012; Revision 0.0; 11/16/94; U.S. EPA Contract 68-C4-0022.

1.0 SCOPE AND APPLICATION

The purpose of this standard operating procedure (SOP) is to describe the procedures for the collection of representative soil samples. Sampling depths are assumed to be those that can be reached without the use of a drill rig, direct-push, or other mechanized equipment (except for a back-hoe). Analysis of soil samples may determine whether concentrations of specific pollutants exceed established action levels, or if the concentrations of pollutants present a risk to public health, welfare, or the environment.

These are standard (i.e., typically applicable) operating procedures which may be varied or changed as required, dependent upon site conditions, equipment limitations or limitations imposed by the procedure. In all instances, the actual procedures used should be documented and described in an appropriate site report.

Mention of trade names or commercial products does not constitute U.S. Environmental Protection Agency (EPA) endorsement or recommendation for use.

2.0 METHOD SUMMARY

Soil samples may be collected using a variety of methods and equipment depending on the depth of the desired sample, the type of sample required (disturbed vs. undisturbed), and the soil type. Near-surface soils may be easily sampled using a spade, trowel, and scoop. Sampling at greater depths may be performed using a hand auger, continuous flight auger, a trier, a split-spoon, or, if required, a backhoe.

3.0 SAMPLE PRESERVATION, CONTAINERS, HANDLING, AND STORAGE

Chemical preservation of solids is not generally recommended. Samples should, however, be cooled and protected from sunlight to minimize any potential reaction. The amount of sample to be collected and proper sample container type are discussed in ERT/REAC SOP #2003 Rev. 0.0 08/11/94, *Sample Storage, Preservation and Handling*.

4.0 INTERFERENCES AND POTENTIAL PROBLEMS

There are two primary potential problems associated with soil sampling - cross contamination of samples and improper sample collection. Cross contamination problems can be eliminated or minimized through the use of dedicated sampling equipment. If this is not possible or practical, then decontamination of sampling equipment is necessary. Improper sample collection can involve using contaminated equipment, disturbance of the matrix resulting in compaction of the sample, or inadequate homogenization of the samples where required, resulting in variable, non-representative results.

5.0 EQUIPMENT

Soil sampling equipment includes the following:

- Maps/plot plan
- Safety equipment, as specified in the site-specific Health and Safety Plan
- Survey equipment or global positioning system (GPS) to locate sampling points
- Tape measure
- Survey stakes or flags
- Camera and film
- Stainless steel, plastic, or other appropriate homogenization bucket, bowl or pan
- Appropriate size sample containers
- Ziplock plastic bags
- Logbook
- Labels
- Chain of Custody records and custody seals
- Field data sheets and sample labels
- Cooler(s)
- Ice
- Vermiculite
- Decontamination supplies/equipment
- Canvas or plastic sheet
- Spade or shovel
- Spatula
- Scoop
- Plastic or stainless steel spoons
- Trowel(s)
- Continuous flight (screw) auger
- Bucket auger
- Post hole auger
- Extension rods
- T-handle
- Sampling trier
- Thin wall tube sampler
- Split spoons
- Vehimeyer soil sampler outfit
  - Tubes
  - Points
  - Drive head
  - Drop hammer
  - Puller jack and grip
- Backhoe

6.0 REAGENTS

Reagents are not used for the preservation of soil samples. Decontamination solutions are specified in ERT/REAC SOP #2006 Rev. 0.0 08/11/94, *Sampling Equipment Decontamination*, and the site specific work plan.

7.0 PROCEDURES

7.1 Preparation

1. Determine the extent of the sampling effort, the sampling methods to be employed, and the types and amounts of equipment and supplies required.

2. Obtain necessary sampling and monitoring equipment.

3. Decontaminate or pre-clean equipment, and ensure that it is in working order.

4. Prepare schedules and coordinate with staff, client, and regulatory agencies, if appropriate.

5. Perform a general site survey prior to site entry in accordance with the site specific Health and Safety Plan.

6. Use stakes, flagging, or buoys to identify and mark all sampling locations. Specific site factors, including extent and nature of contaminant, should be considered when selecting sample location. If required, the proposed locations may be adjusted based on site access, property boundaries, and surface obstructions. All staked locations should be utility-cleared by the property owner or the On-Scene-Coordinator (OSC) prior to soil sampling; and utility clearance should always be confirmed before beginning work.

7.2 Sample Collection

7.2.1 Surface Soil Samples

Collection of samples from near-surface soil can be accomplished with tools such as spades, shovels, trowels, and scoops. Surface material is removed to the required depth and a stainless steel or plastic scoop is then used to collect the sample.

This method can be used in most soil types but is limited to sampling at or near the ground surface. Accurate, representative samples can be collected with this procedure depending on the care and precision demonstrated by the sample team member. A flat, pointed mason trowel to cut a block of the desired soil is helpful when undisturbed profiles are required. Tools plated with chrome or other materials should not be used. Plating is particularly common with garden implements such as potting trowels.

The following procedure is used to collect surface soil samples:

1. Carefully remove the top layer of soil or debris to the desired sample depth with a pre-cleaned spade.

2. Using a pre-cleaned, stainless steel scoop, plastic spoon, or trowel, remove and discard a thin layer of soil from the area which came in contact with the spade.

3. If volatile organic analysis is to be performed, transfer the sample directly into an appropriate, labeled sample container with a stainless steel lab spoon, or equivalent and secure the cap tightly. Place the remainder of the sample into a stainless steel, plastic, or other appropriate homogenization container, and mix thoroughly to obtain a homogenous sample representative of the entire sampling interval. Then, either place the sample into appropriate, labeled containers and secure the caps tightly; or, if composite samples are to be collected, place a sample from another sampling interval or location into the homogenization container and mix thoroughly. When compositing is complete, place the sample into appropriate, labeled containers and secure the caps tightly.

7.2.2 Sampling at Depth with Augers and Thin Wall Tube Samplers

This system consists of an auger, or a thin-wall tube sampler, a series of extensions, and a "T" handle (Figure 1, Appendix A). The auger is used to bore a hole to a desired sampling depth, and is then withdrawn. The sample may be collected directly from the auger. If a core sample is to be collected, the auger tip is then replaced with a thin wall tube sampler. The system is then lowered down the borehole, and driven into the soil to the completion depth. The system is withdrawn and the core is collected from the thin wall tube sampler.

Several types of augers are available; these include: bucket type, continuous flight (screw), and post-hole augers. Bucket type augers are better for direct sample recovery because they provide a large volume of sample in a short time. When continuous flight augers are used, the sample can be collected directly from the flights. The continuous flight augers are satisfactory when a composite of the complete soil column is desired. Post-hole augers have limited utility for sample collection as they are designed to cut through fibrous, rooted, swampy soil and cannot be used below a depth of approximately three feet.

The following procedure is used for collecting soil samples with the auger:

1. Attach the auger bit to a drill rod extension, and attach the "T" handle to the drill rod.

2. Clear the area to be sampled of any surface debris (e.g., twigs, rocks, litter). It may be advisable to remove the first three to six inches of surface soil for an area approximately six inches in radius around the drilling location.

3. Begin augering, periodically removing and depositing accumulated soils onto a plastic sheet spread near the hole. This prevents accidental brushing of loose material back down the borehole when removing the auger or adding drill rods. It also facilitates refilling the hole, and avoids possible contamination of the surrounding area.

4. After reaching the desired depth, slowly and carefully remove the auger from the hole. When sampling directly from the auger, collect the sample after the auger is removed from the hole and proceed to Step 10.

5. Remove auger tip from the extension rods and replace with a pre-cleaned thin wall tube sampler. Install the proper cutting tip.

6. Carefully lower the tube sampler down the borehole. Gradually force the tube sampler into the soil. Do not scrape the borehole sides. Avoid hammering the rods as the vibrations may cause the boring walls to collapse.

7. Remove the tube sampler, and unscrew the drill rods.

8. Remove the cutting tip and the core from the device.

9. Discard the top of the core (approximately 1 inch), as this possibly represents material collected before penetration of the layer of concern. Place the remaining core into the appropriate labeled sample container. Sample homogenization is not required.

10. If volatile organic analysis is to be performed, transfer the sample into an appropriate, labeled sample container with a stainless steel lab spoon, or equivalent and secure the cap tightly. Place the remainder of the sample into a stainless steel, plastic, or other appropriate homogenization container, and mix thoroughly to obtain a homogenous sample representative of the entire sampling interval. Then, either place the sample into appropriate, labeled containers and secure the caps tightly; or, if composite samples are to be collected, place a sample from another sampling interval into the homogenization container and mix thoroughly.

    When compositing is complete, place the sample into appropriate, labeled containers and secure the caps tightly.

11. If another sample is to be collected in the same hole, but at a greater depth, reattach the auger bit to the drill and assembly, and follow steps 3 through 11, making sure to decontaminate the auger and tube sampler between samples.

12. Abandon the hole according to applicable state regulations. Generally, shallow holes can simply be backfilled with the removed soil material.

7.2.3 Sampling with a Trier

The system consists of a trier, and a "T" handle. The auger is driven into the soil to be sampled and used to extract a core sample from the appropriate depth.

The following procedure is used to collect soil samples with a sampling trier:

1. Insert the trier (Figure 2, Appendix A) into the material to be sampled at a 0° to 45° angle from horizontal. This orientation minimizes the spillage of sample.

2. Rotate the trier once or twice to cut a core of material.

3. Slowly withdraw the trier, making sure that the slot is facing upward.

4. If volatile organic analyses are required, transfer the sample into an appropriate, labeled sample container with a stainless steel lab spoon, or equivalent and secure the cap tightly. Place the remainder of the sample into a stainless steel, plastic, or other appropriate homogenization container, and mix thoroughly to obtain a homogenous sample representative of the entire sampling interval. Then, either place the sample into appropriate, labeled containers and secure the caps tightly; or, if composite samples are to be collected, place a sample from another sampling interval into the homogenization container and mix thoroughly. When compositing is complete, place the sample into appropriate, labeled containers and secure the caps tightly.

7.2.4 Sampling at Depth with a Split Spoon (Barrel) Sampler

Split spoon sampling is generally used to collect undisturbed soil cores of 18 or 24 inches in length. A series of consecutive cores may be extracted with a split spoon sampler to give a complete soil column profile, or an auger may be used to drill down to the desired depth for sampling. The split spoon is then driven to its sampling depth through the bottom of the augured hole and the core extracted.

When split spoon sampling is performed to gain geologic information, all work should

be performed in accordance with ASTM D1586-98, "Standard Test Method for Penetration Test and Split-Barrel Sampling of Soils".

The following procedures are used for collecting soil samples with a split spoon:

1. Assemble the sampler by aligning both sides of barrel and then screwing the drive shoe on the bottom and the head piece on top.

2. Place the sampler in a perpendicular position on the sample material.

3. Using a well ring, drive the tube. Do not drive past the bottom of the head piece or compression of the sample will result.

4. Record in the site logbook or on field data sheets the length of the tube used to penetrate the material being sampled, and the number of blows required to obtain this depth.

5. Withdraw the sampler, and open by unscrewing the bit and head and splitting the barrel. The amount of recovery and soil type should be recorded on the boring log. If a split sample is desired, a cleaned, stainless steel knife should be used to divide the tube contents in half, longitudinally. This sampler is typically available in 2 and 3 1/2 inch diameters. A larger barrel may be necessary to obtain the required sample volume.

6. Without disturbing the core, transfer it to appropriate labeled sample container(s) and seal tightly.

7.2.5 Test Pit/Trench Excavation

A backhoe can be used to remove sections of soil, when detailed examination of soil characteristics are required. This is probably the most expensive sampling method because of the relatively high cost of backhoe operation.

The following procedures are used for collecting soil samples from test pits or trenches:

1. Prior to any excavation with a backhoe, it is important to ensure that all sampling locations are clear of overhead and buried utilities.

2. Review the site specific Health & Safety plan and ensure that all safety precautions including appropriate monitoring equipment are installed as required.

3. Using the backhoe, excavate a trench approximately three feet wide and approximately one foot deep below the cleared sampling location. Place excavated soils on plastic sheets. Trenches greater than five feet deep must be sloped or protected by a shoring system, as required by OSHA regulations.

4. A shovel is used to remove a one to two inch layer of soil from the vertical face of the pit where sampling is to be done.

5. Samples are taken using a trowel, scoop, or coring device at the desired intervals. Be sure to scrape the vertical face at the point of sampling to remove any soil that may have fallen from above, and to expose fresh soil for sampling. In many instances, samples can be collected directly from the backhoe bucket.

6. If volatile organic analyses are required, transfer the sample into an appropriate, labeled sample container with a stainless steel lab spoon, or equivalent and secure the cap tightly. Place the remainder of the sample into a stainless steel, plastic, or other appropriate homogenization container, and mix thoroughly to obtain a homogenous sample representative of the entire sampling interval. Then, either place the sample into appropriate, labeled containers and secure the caps tightly; or, if composite samples are to be collected, place a sample from another sampling interval into the homogenization container and mix thoroughly. When compositing is complete, place the sample into appropriate, labeled containers and secure the caps tightly.

7. Abandon the pit or excavation according to applicable state regulations. Generally, shallow excavations can simply be backfilled with the removed soil material.

8.0 CALCULATIONS

This section is not applicable to this SOP.

9.0 QUALITY ASSURANCE/QUALITY CONTROL

There are no specific quality assurance (QA) activities which apply to the implementation of these procedures. However, the following QA procedures apply:

1. All data must be documented on field data sheets or within site logbooks.

2. All instrumentation must be operated in accordance with operating instructions as supplied by the manufacturer, unless otherwise specified in the work plan. Equipment checkout and calibration

activities must occur prior to sampling/operation, and they must be documented.

10.0 DATA VALIDATION

This section is not applicable to this SOP.

11.0 HEALTH AND SAFETY

When working with potentially hazardous materials, follow U.S. EPA, OHSA and corporate health and safety procedures, in addition to the procedures specified in the site specific Health & Safety Plan..

12.0 REFERENCES

Mason, B.J. 1983. Preparation of Soil Sampling Protocol: Technique and Strategies. EPA-600/4-83-020.

Barth, D.S. and B.J. Mason. 1984. Soil Sampling Quality Assurance User's Guide. EPA-600/4-84-043.

U.S. Environmental Protection Agency. 1984 Characterization of Hazardous Waste Sites - A Methods Manual: Volume II. Available Sampling Methods, Second Edition. EPA-600/4-84-076.

de Vera, E.R., B.P. Simmons, R.D. Stephen, and D.L. Storm. 1980. Samplers and Sampling Procedures for Hazardous Waste Streams. EPA-600/2-80-018.

ASTM D 1586-98, ASTM Committee on Standards, Philadelphia, PA.

APPENDIX A
Figures
SOP #2012
February 2000

FIGURE 1. Sampling Augers

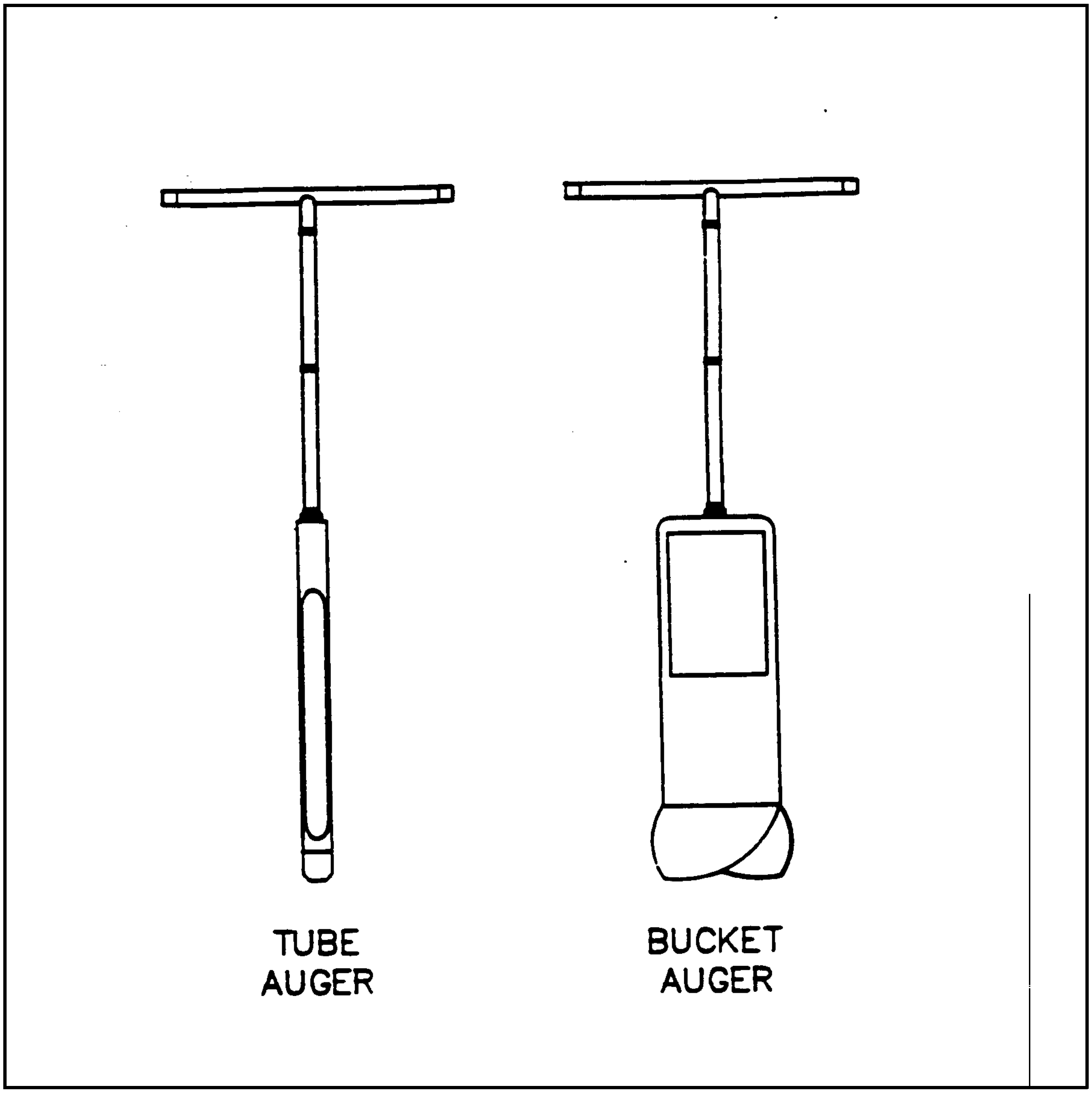

FIGURE 2. Sampling Trier

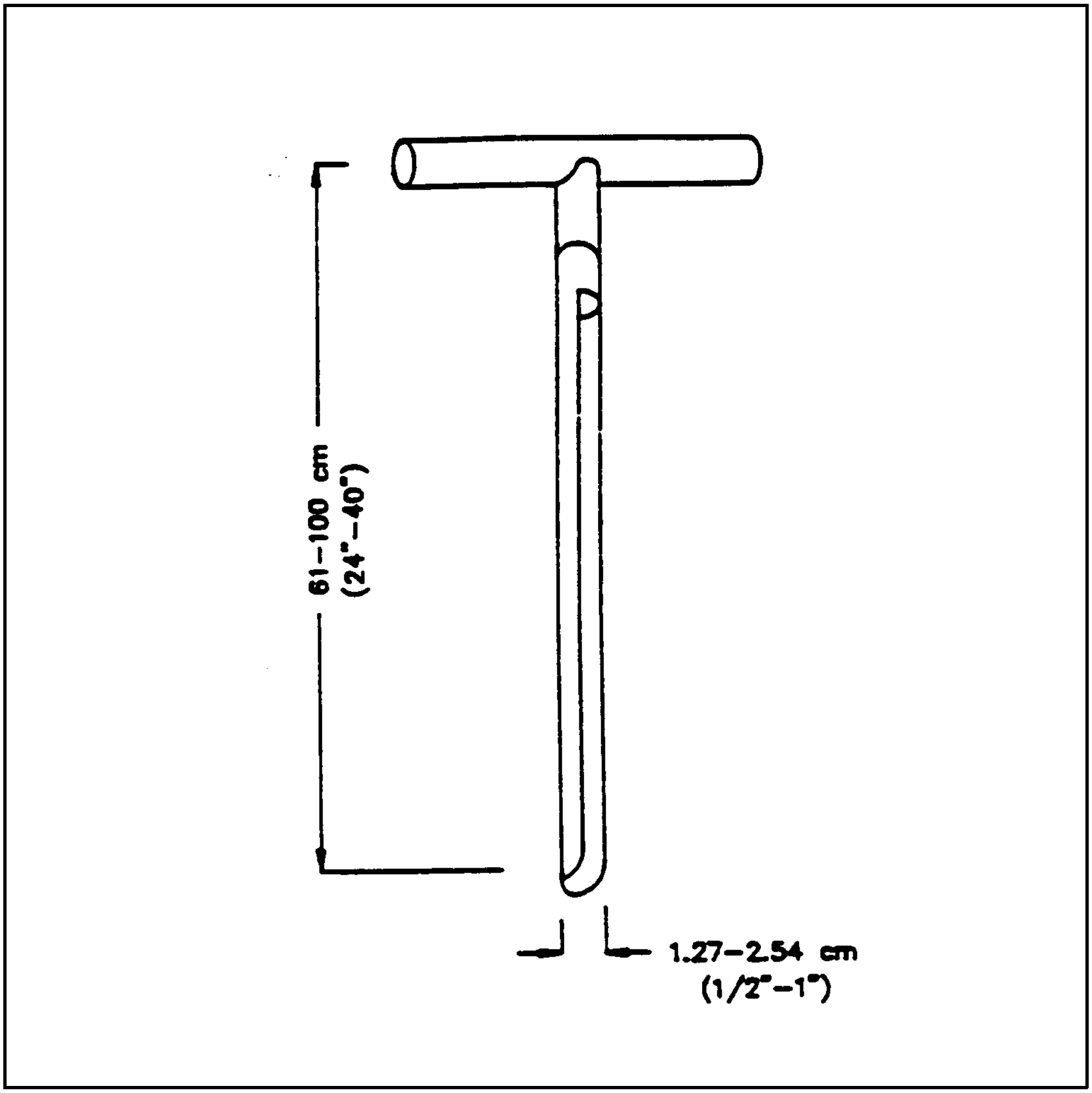

ASBESTOS SAMPLING

SOP#: 2015
DATE: 11/17/94
REV. #: 0.0

1.0 SCOPE AND APPLICATION

Asbestos has been used in many commercial products including building materials such as flooring tiles and sheet goods, paints and coatings, insulation, and roofing asphalts. These products and others may be found at hazardous waste sites hanging on overhead pipes, contained in drums, abandoned in piles, or as part of a structure. Asbestos tailing piles from mining operations can also be a source of ambient asbestos fibers. Asbestos is a known carcinogen and requires air sampling to assess airborne exposure to human health. This Standard Operating Procedure (SOP) provides procedures for asbestos air sampling by drawing a known volume of air through a mixed cellulose ester (MCE) filter. The filter is then sent to a laboratory for analysis. The U.S. Environmental Protection Agency/Environmental Response Team (U.S. EPA/ERT) uses one of four analytical methods for determining asbestos in air. These include: U.S. EPA's Environmental Asbestos Assessment Manual, Superfund Method for the Determination of Asbestos in Ambient Air for Transmission Electron Microscopy (TEM)[(1)]; U.S. EPA's Modified Yamate Method for TEM[(2)]; National Institute for Occupational Safety and Health (NIOSH) Method 7402 (direct method only) for TEM; and NIOSH Method 7400 for Phase Contrast Microscopy (PCM)[(3)]. Each method has specific sampling and analytical requirements (i.e., sample volume and flow rate) for determining asbestos in air.

The U.S. EPA/ERT typically follows procedures outlined in the TEM methods for determining mineralogical types of asbestos in air and for distinguishing asbestos from non-asbestos minerals. The Phase Contrast Microscopy (PCM) method is used by U.S. EPA/ERT as a screening tool since it is less costly than TEM. PCM cannot distinguish asbestos from non-asbestos fibers, therefore the TEM method may be necessary to confirm analytical results. For example, if an action level for the presence of fibers has been set and PCM analysis indicates that the action level has been exceeded, then TEM analysis can be used to quantify and identify asbestos structures through examination of their morphology crystal structures (through electron diffraction), and elemental composition (through energy dispersive X-ray analysis). In this instance samples should be collected for both analyses in side by side sampling trains (some laboratories are able to perform PCM and TEM analysis from the same filter). The Superfund method is designed specifically to provide results suitable for supporting risk assessments at Superfund sites, it is applicable to a wide range of ambient air situations at hazardous waste sites. U.S. EPA's Modified Yamate Method for TEM is also used for ambient air sampling due to high volume requirements. The PCM and TEM NIOSH analytical methods require lower sample volumes and are typically used indoors; however, ERT will increase the volume requirement for outdoor application.

Other Regulations pertaining to asbestos have been promulgated by U.S. EPA and OSHA. U.S. EPA's National Emission Standards for Hazardous Air Pollutants (NESHAP) regulates asbestos-containing waste materials. NESHAP establishes management practices and standards for the handling of asbestos and emissions from waste disposal operations (40 CFR Part 61, Subparts A and M). U.S. EPA's 40 CFR 763 (July 1, 1987)[(4)] and its addendum 40 CFR 763 (October 30, 1987)[(4)] provide comprehensive rules for the asbestos abatement industry. State and local regulations on these issues vary and may be more stringent than federal requirements. The OSHA regulations in 29 CFR 1910.1001 and 29 CFR 1926.58 specify work practices and safety equipment such as respiratory protection and protective clothing when handling asbestos. The OSHA standard for an 8-hour, time-weighted average (TWA) is 0.2 fibers/cubic centimeters of air. This standard pertains to fibers with a length-to-width ratio of 3 to 1 with a fiber length >5 µm[(5,6)]. An action level of 0.1 fiber/cc (one-half the OSHA standard) is the level U.S. EPA has established in which employers must initiate such activities as air monitoring, employee training, and

medical surveillance[5,6].

These are standard (i.e., typically applicable) operating procedures which may be varied or changed as required, dependent upon site conditions, equipment limitations or limitations imposed by the procedure. In all instances, the ultimate procedures employed should be documented and associated with the final report.

Mention of trade names or commercial products does not constitute U.S. EPA endorsement or recommendation for use.

2.0 METHOD SUMMARY

Prior to sampling, the site should be characterized by identifying on-site as well as off-site sources of airborne asbestos. The array of sampling locations and the schedule for sample collection, is critical to the success of an investigation. Generally, sampling strategies to characterize a single point source are fairly straightforward, while multiple point sources and area sources increase the complexity of the sampling strategy. It is not within the scope of this SOP to provide a generic asbestos air sampling plan. Experience, objectives, and site characteristics will dictate the sampling strategy.

During a site investigation, sampling stations should be arranged to distinguish spatial trends in airborne asbestos concentrations. Sampling schedules should be fashioned to establish temporal trends. The sampling strategy typically requires that the concentration of asbestos at the source (worst case) or area of concern (downwind), crosswind, as well as background (upwind) contributions be quantified. See Table 1 (Appendix A) for U.S. EPA/ERT recommended sampling set up for ambient air. Indoor asbestos sampling requires a different type of strategy which is identified in Table 2 (Appendix A). It is important to establish background levels of contaminants in order to develop a reference point from which to evaluate the source data. Field blanks and lot blanks can be utilized to determine other sources.

Much information can be derived from each analytical method previously mentioned. Each analytical method has specific sampling requirements and produce results which may or may not be applicable to a specific sampling effort. The site sampling objectives should be carefully identified so as to select the most appropriate analytical method. Additionally, some preparation (i.e., lot blanks results) prior to site sampling may be required, these requirements are specified in the analytical methods.

3.0 SAMPLE PRESERVATION, CONTAINERS, HANDLING, AND STORAGE

3.1 Sample Preservation

No preservation is required for asbestos samples.

3.2 Sample Handling, Container and Storage Procedures

1. Place a sample label on the cassette indicating a unique sampling number. Do not put sampling cassettes in shirt or coat pockets as the filter can pick up fibers. The original cassette box is used to hold the samples.

2. Wrap the cassette individually in a plastic sample bag. Each bag should be marked indicating sample identification number, total volume, and date.

3. The wrapped sampling cassettes should be placed upright in a rigid container so that the cassette cap is on top and cassette base is on bottom. Use enough packing material to prevent jostling or damage. Do not use vermiculite as packing material for samples. If possible, hand carry to lab.

4. Provide appropriate documentation with samples (i.e., chain of custody and requested analytical methodology).

4.0 INTERFERENCES AND POTENTIAL PROBLEMS

Flow rates exceeding 16 liters/minute (L/min) which could result in filter destruction due to (a) failure of its physical support under force from the increased pressure drop; (b) leakage of air around the filter mount so that the filter is bypassed, or (c) damage to the asbestos structures due to increased impact velocities.

4.1 U.S. EPA's Superfund Method

4.1.1 Direct-transfer TEM Specimen Preparation Methods

Direct-Transfer TEM specimen preparation methods have the following significant interferences:

- C The achievable detection limit is restricted by the particulate density on the filter, which in turn is controlled by the sampled air volume and the total suspended particulate concentration in the atmosphere being sampled.

- C The precision of the result is dependent on the uniformity of the deposit of asbestos structures on the sample collection filter.

- C Air samples must be collected so that they have particulate and fiber loadings within narrow ranges. If too high a particulate loading occurs on the filter, it is not possible to prepare satisfactory TEM specimens by a direct-transfer method. If too high a fiber loading occurs on the filter, even if satisfactory TEM specimens can be prepared, accurate fiber counting will not be possible.

4.1.2 Indirect TEM Specimen Preparation Methods

Indirect TEM specimen preparation methods have the following interferences:

- C The size distribution of asbestos structures is modified.

- C There is increased opportunity for fiber loss or introduction of extraneous contamination.

- C When sample collection filters are ashed, any fiber contamination in the filter medium is concentrated on the TEM specimen grid.

It can be argued that direct methods yield an under-estimate of the asbestos structure concentration because many of the asbestos fibers present are concealed by other particulate material with which they are associated. Conversely, indirect methods can be considered to yield an over-estimate because some types of complex asbestos structures disintegrate during the preparation, resulting in an increase in the numbers of structures counted.

4.2 U.S. EPA's Modified Yamate Method for TEM

High concentrations of background dust interfere with fiber identification.

4.3 NIOSH Method for TEM

Other amphibole particles that have aspect ratios greater than 3:1 and elemental compositions similar to the asbestos minerals may interfere in the TEM analysis. Some non-amphibole minerals may give electron diffraction patterns similar to amphiboles. High concentrations of background dust interfere with fiber identification.

4.4 NIOSH Method for PCM

PCM cannot distinguish asbestos from non-asbestos fibers; therefore, all particles meeting the counting criteria are counted as total asbestos fibers. Fiber less than 0.25 um in length will not be detected by this method. High levels of non-fibrous dust particles may obscure fibers in the field of view and increase the detection limit.

5.0 EQUIPMENT/MATERIALS

5.1 Sampling Pump

The constant flow or critical orifice controlled sampling pump should be capable of a flow-rate and pumping time sufficient to achieve the desired volume of air sampled.

The lower flow personal sampling pumps generally provide a flow rate of 20 cubic centimeters/minute (cc/min) to 4 L/min. These pumps are usually battery powered. High flow pumps are utilized when flow rates between 2 L/min to 20 L/min are required. High flow pumps are used for short sampling periods so as to obtain the desired sample volume. High flow pumps usually run on AC power and can be plugged into a nearby outlet. If an outlet is not available then a generator should be obtained. The generator should be positioned downwind from the sampling pump. Additional voltage may be required if more than one pump is plugged into the same generator. Several

electrical extension cords may be required if sampling locations are remote.

The recommended volume for the Superfund method (Phase I) requires approximately 20 hours to collect. Such pumps typically draw 6 amps at full power so that 2 lead/acid batteries should provide sufficient power to collect a full sample. The use of line voltage, where available, eliminates the difficulties associated with transporting stored electrical energy.

A stand should be used to hold the filter cassette at the desired height for sampling and the filter cassette shall be isolated from the vibrations of the pump.

5.2 Filter Cassette

The cassettes are purchased with the required filters in position, or can be assembled in a laminar flow hood or clean area. When the filters are in position, a shrink cellulose band or adhesive tape should be applied to cassette joints to prevent air leakage.

5.2.1 TEM Cassette Requirements

Commercially available field monitors, comprising 25 mm diameter three-piece cassettes, with conductive extension cowls shall be used for sample collection. The cassette must be new and not previously used. The cassette shall be loaded with an MCE filter of pore size 0.45 µm, and supplied from a lot number which has been qualified as low background for asbestos determination. The cowls should be constructed of electrically conducting material to minimize electrostatic effects. The filter shall be backed by a 5 µm pore size MCE filter (Figure 1, Appendix B).

5.2.2 PCM Cassette Requirements

NIOSH Method 7400, PCM involves using a 0.8 to 1.2 µm mixed cellulose ester membrane, 25 mm diameter, 50 mm conductive cowl on cassette (Figure 2, Appendix B). Some labs are able to perform PCM and TEM analysis on the same filter; however, this should be discussed with the laboratory prior to sampling.

5.3 Other Equipment

C Inert tubing with glass cyclone and hose barb
C Whirlbags (plastic bags) for cassettes
C Tools - small screw drivers
C Container - to keep samples upright
C Generator or electrical outlet (may not be required)
C Extension cords (may not be required)
C Multiple plug outlet
C Sample labels
C Air data sheets
C Chain of Custody records

6.0 REAGENTS

Reagents are not required for the preservation of asbestos samples.

7.0 PROCEDURES

7.1 Air Volumes and Flow Rates

Sampling volumes are determined on the basis of how many fibers need to be collected for reliable measurements. Therefore, one must estimate how many airborne fibers may be in the sampling location.

Since the concentration of airborne aerosol contaminants will have some effect on the sample, the following is a suggested criteria to assist in selecting a flow rate based on real-time aerosol monitor (RAM) readings in milligrams/cubic meter (mg/m^3).

	Concentration	Flow Rate
C Low RAM readings:	<6.0 mg/m^3	11-15. L/min
C Medium RAM readings:	>6.0 mg/m_3	7.5 L/min
C High RAM readings:	>10. mg/m^3	2.5 L/min

In practice, pumps that are available for environmental sampling at remote locations operate under a maximum load of approximately 12 L/min.

7.1.1 U.S. EPA's Superfund Method

The Superfund Method incorporates an indirect preparation procedure to provide flexibility in the amount of deposit that be can be tolerated on the sample filter and to allow for the selective concentration of asbestos prior to analysis. To minimize contributions to background contamination from asbestos present in the plastic matrices of membrane filters while allowing for sufficient quantities of asbestos to be collected, this method also requires the collection of a larger volume of air per unit area of filter than has traditionally been collected

for asbestos analysis. Due to the need to collect large volumes of air, higher sampling flow rates are recommended in this method than have generally been employed for asbestos sampling in the past. As an alternative, samples may be collected over longer time intervals. However, this restricts the flexibility required to allow samples to be collected while uniform meteorological conditions prevail.

The sampling rate and the period of sampling should be selected to yield as high a sampled volume as possible, which will minimize the influence of filter contamination. Wherever possible, a volume of 15 cubic meters (15,000 L) shall be sampled for those samples intended for analysis only by the indirect TEM preparation method (Phase 1 samples). For those samples to be prepared by both the indirect and the direct specimen preparation methods (Phase 2 samples), the volumes must be adjusted so as to provide a suitably-loaded filter for the direct TEM preparation method. One option is to collect filters at several loadings to bracket the estimated optimum loading for a particular site. Such filters can be screened in the laboratory so that only those filters closest to optimal loading are analyzed. It has been found that the volume cannot normally exceed 5 cubic meters (5000 L) in an urban or agricultural area, and 10 cubic meters (10,000 L) in a rural area for samples collected on a 25 mm filter and prepared by a direct-transfer technique.

An upper limit to the range of acceptable flow rates for this method is 15 L/min. At many locations, wind patterns exhibit strong diurnal variations. Therefore, intermittent sampling (sampling over a fixed time interval repeated over several days) may be necessary to accumulate 20 hours of sampling time over constant wind conditions. Other sampling objectives also may necessitate intermittent sampling. The objective is to design a sampling schedule so that samples are collected under uniform conditions throughout the sampling interval. This method provides for such options. Air volumes collected on Phase I samples are maximized (<16 L/min). Air volumes collected on Phase 2 samples are limited to provide optimum loading for filters to be prepared by a direct-transfer procedure.

7.1.2 U.S. EPA's Modified Yamate Method for TEM

U.S. EPA's TEM method requires a minimum volume of 560 L and a maximum volume of 3,800 L in order to obtain an analytical sensitivity of 0.005 structures/cc. The optimal volume for TEM is 1200 L to 1800 L. These volumes are determined using a 200 mesh EM grid opening with a 25-mm filter cassette. Changes in volume would be necessary if a 37-mm filter cassette is used since the effective area of a 25 mm (385 sq mm) and 37 mm (855 sq m) differ.

7.1.3 NIOSH Method for TEM and PCM

The minimum recommended volume for TEM and PCM is 400 L at 0.1 fiber/cc. Sampling time is adjusted to obtain optimum fiber loading on the filter. A sampling rate of 1 to 4 L/min for eight hours (700 to 2800 L) is appropriate in non-dusty atmospheres containing 0.1 fiber/cc. Dusty atmospheres i.e., areas with high levels of asbestos, require smaller sample volumes (<400 L) to obtain countable samples.

In such cases, take short, consecutive samples and average the results over the total collection time. For documenting episodic exposures, use high flow rates (7 to 16 L/min) over shorter sampling times. In relatively clean atmospheres where targeted fiber concentrations are much less than 0.1 fiber/cc, use larger sample volumes (3,000 to 10,000 L) to achieve quantifiable loadings. Take care, however, not to overload the filter with background dust. If > 50% of the filter surface is covered with particles, the filter may be too overloaded to count and will bias the measured fiber concentration. Do not exceed 0.5 mg total dust loading on the filter.

7.2 Calibration Procedures

In order to determine if a sampling pump is measuring the flow rate or volume of air correctly, it is necessary to calibrate the instrument. Sampling pumps should be calibrated immediately before and after each use. Preliminary calibration should be conducted using a primary calibrator such as a soap bubble type calibrator, (e.g., a Buck Calibrator, Gilibrator, or equivalent primary calibrator) with a representative filter cassette installed between the pump and the calibrator. The representative sampling cassette can be reused for calibrating other pumps that will be used for asbestos sampling. The same cassette lot used for sampling should also be used for the calibration. A sticker should be affixed to the outside of the extension cowl marked "Calibration Cassette."

A rotameter can be used provided it has been recently precalibrated with a primary calibrator. Three separate constant flow calibration readings should be obtained both before sampling and after sampling. Should the flow rate change by more than 5% during the sampling period, the average of the pre- and post-calibration rates will be used to calculate the total sample volume. The sampling pump used shall provide a non-fluctuating air-flow through the filter, and shall maintain the initial volume flow-rate to within ± 10% throughout the sampling period. The mean value of these flow-rate measurements shall be used to calculate the total air volume sampled. A constant flow or critical orifice controlled pump meets these requirements. If at any time the measurement indicates that the flow-rate has decreased by more than 30%, the sampling shall be terminated. Flexible tubing is used to connect the filter cassette to the sampling pump.Sampling pumps can be calibrated prior to coming on-site so that time is saved when performing on-site calibration.

7.2.1 Calibrating a Personal Sampling Pump with an Electronic Calibrator

1. See Manufacturer's manual for operational instructions.

2. Set up the calibration train as shown in (Figure 3, Appendix B) using a sampling pump, electronic calibrator, and a representative filter cassette. The same lot sampling cassette used for sampling should also be used for calibrating.

3. To set up the calibration train, attach one end of the PVC tubing (approx. 2 foot) to the cassette base; attach the other end of the tubing to the inlet plug on the pump. Another piece of tubing is attached from the cassette cap to the electronic calibrator.

4. Turn the electronic calibrator and sampling pump on. Create a bubble at the bottom of the flow chamber by pressing the bubble initiate button. The bubble should rise to the top of the flow chamber. After the bubble runs its course, the flow rate is shown on the LED display.

5. Turn the flow adjust screw or knob on the pump until the desired flow rate is attained.

6. Perform the calibration three times until the desired flow rate of ± 5% is attained.

7.2.2 Calibrating a Rotameter with an Electronic Calibrator

1. See manufacturer's manual for operational instructions.

2. Set up the calibration train as shown in (Figure 4, Appendix B) using a sampling pump, rotameter, and electronic calibrator.

3. Assemble the base of the flow meter with the screw provided and tighten in place. The flow meter should be mounted within 6° vertical.

4. Turn the electronic calibrator and sampling pump on.

5. Create a bubble at the bottom of the flow chamber by pressing the bubble initiate button. The bubble should rise to the top of the flow chamber. After the bubble runs its course, the flow rate is shown on the LED display.

6. Turn the flow adjust screw or knob on the pump until the desired flow rate is attained.

7. Record the electronic calibrator flow rate reading and the corresponding rotameter reading. Indicate these values on the rotameter (sticker). The rotameter should be able to work within the desired flow range. Readings can also be calibrated for 10 cm^3 increments for Low Flow rotameters, 500 cm^3 increments for medium flow rotameters and 1 liter increments for high flow rotameters.

8. Perform the calibration three times until the desired flow rate of ± 5% is attained. Once on site, a secondary calibrator, i.e., rotameter may be used to calibrate sampling pumps.

7.2.3 Calibrating a Personal Sampling Pump with a Rotameter

1. See manufacturer's manual for Rotameter's Operational Instructions.

2. Set up the calibration train as shown in (Figure 5, Appendix B) using a rotameter, sampling pump, and a representative sampling cassette.

3. To set up the calibration train, attach one end of the PVC tubing (approx. 2 ft) to the cassette base; attach the other end of the tubing to the inlet plug on the pump. Another piece of tubing is attached from the cassette cap to the rotameter.

4. Assemble the base of the flow meter with the screw provided and tighten in place. The flow meter should be mounted within 6° vertical.

5. Turn the sampling pump on.

6. Turn the flow adjust screw (or knob) on the personal sampling pump until the float ball on the rotameter is lined up with the precalibrated flow rate value. A sticker on the rotameter should indicate this value.

7. A verification of calibration is generally performed on-site in the clean zone immediately prior to the sampling.

7.3. Meteorology

It is recommended that a meteorological station be established. If possible, sample after two to three days of dry weather and when the wind conditions are at 10 mph or greater. Record wind speed, wind direction, temperature, and pressure in a field logbook. Wind direction is particularly important when monitoring for asbestos downwind from a fixed source.

7.4 Ambient Sampling Procedures

7.4.1 Pre-site Sampling Preparation

1. Determine the extent of the sampling effort, the sampling methods to be employed, and the types and amounts of equipment and supplies needed.

2. Obtain necessary sampling equipment and ensure it is in working order and fully charged (if necessary).

3. Perform a general site survey prior to site entry in accordance with the site specific Health and Safety plan.

4. Once on-site the calibration is performed in the clean zone. The calibration procedures are listed in Section 7.2.

5. After calibrating the sampling pump, mobilize to the sampling location.

7.4.2 Site Sampling

1. To set up the sampling train, attach the air intake hose to the cassette base. Remove the cassette cap (Figure 6 and 7, Appendix B). The cassette should be positioned downward, perpendicular to the wind

2. If AC or DC electricity is required then turn it on. If used, the generator should be placed 10 ft. downwind from the sampling pump.

3. Record the following in a field logbook: date, time, location, sample identification number, pump number, flow rate, and cumulative time.

4. Turn the pump on. Should intermittent sampling be required, sampling filters must be covered between active periods of sampling. To cover the sample filter: turn the cassette to face upward, place the cassette cap on the cassette, remove the inlet plug from the cassette cap, attach a rotameter to the inlet opening of the cassette cap to measure the flow rate, turn off the sampling pump, place the inlet plug into the inlet opening on the cassette cap. To resume sampling: remove the inlet plug, turn on the sampling pump, attach a rotameter to measure the flow rate, remove the cassette cap, replace the inlet plug in the cassette cap and invert the cassette, face downward and perpendicular to the wind.

5. Check the pump at sampling midpoint if sampling is longer than 4 hours. The generators may need to be regased depending on tank size. If a filter darkens in appearance or if loose dust is seen in the filter, a second sample should be started.

6. At the end of the sampling period, orient the cassette up, turn the pump off.

7. Check the flow rate as shown in Section 7.2.3. When sampling open-faced, the sampling cap should be replaced before post calibrating. Use the same cassette used for sampling for post calibration (increase dust/fiber loading may have altered the flow rate.

8. Record the post flow rate.

9. Record the cumulative time or run.

10. Remove the tubing from the sampling cassette. Still holding the cassette upright, replace the inlet plug on the cassette cap and the outlet plug on the cassette base.

7.4.3. Post Site Sampling

1. Follow handling procedures in Section 3.2, steps 1-4.

2. Obtain an electronic or hard copy of meteorological data which occurred during the sampling event. Record weather: wind speed, ambient temperature, wind direction, and precipitation. Obtaining weather data several days prior to the sampling event can also be useful.

7.5 Indoor Sampling Procedures

PCM analysis is used for indoor air samples. When analysis shows total fiber count above the OSHA action level 0.1 f/cc then TEM (U.S. EPA's Modified Yamate Method) is used to identify asbestos from non-asbestos fibers.

Sampling pumps should be placed four to five feet above ground level away from obstructions that may influence air flow. The pump can be placed on a table or counter. Refer to Table 2 (Appendix A) for a summary of indoor sampling locations and rationale for selection.

Indoor sampling utilizes high flow rates to increased sample volumes (2000 L for PCM and 2800 to 4200 L for TEM) in order to obtain lower detection limits below the standard, (i.e., 0.01 f/cc or lower [PCM] and 0.005 structures/cc or lower [TEM]).

7.5.1 Aggressive Sampling Procedures

Sampling equipment at fixed locations may fail to detect the presence of asbestos fibers. Due to limited air movement, many fibers may settle out of the air onto the floor and other surfaces and may not be captured on the filter. In the past, an 8-hour sampling period was recommended to cover various air circulation conditions. A quicker and more effective way to capture asbestos fibers is to circulate the air artificially so that the fibers remain airborne during sampling. The results from this sampling option typifies worst case condition. This is referred to as aggressive air sampling for asbestos. Refer to Table 2 for sample station locations.

1. Before starting the sampling pumps, direct forced air (such as a 1-horsepower leaf blower or large fan) against walls, ceilings, floors, ledges, and other surfaces in the room to initially dislodge fibers from surfaces. This should take at least 5 minutes per 1000 sq. ft. of floor.

2. Place a 20-inch fan in the center of the room. (Use one fan per 10,000 cubic feet of room space.) Place the fan on slow speed and point it toward the ceiling.

3. Follow procedures in Section 7.4.1 and 7.4.2 (Turn off the pump and then the fan(s) when sampling is complete.).

4. Follow handling procedures in Section 3.2, steps 1-4.

8.0 CALCULATIONS

The sample volume is calculated from the average flow rate of the pump multiplied by the number of minutes the pump was running (volume = flow rate X time in minutes). The sample volume should be submitted to the laboratory and identified on the chain of custody for each sample (zero for lot, field and trip blanks).

The concentration result is calculated using the sample volume and the numbers of asbestos structures reported after the application of the cluster and matrix counting criteria.

9.0 QUALITY ASSURANCE/ QUALITY CONTROL

Follow all QA/QC requirements from the laboratories as well as the analytical methods.

9.1 TEM Requirements

1. Examine lot blanks to determine the background asbestos structure concentration.

2. Examine field blanks to determine whether there is contamination by extraneous asbestos structures during specimen preparation.

3. Examine of laboratory blanks to determine if contamination is being introduced during critical phases of the laboratory program.

4. To determine if the laboratory can satisfactorily analyze samples of known asbestos structure concentrations, reference filters shall be examined. Reference filters should be maintained as part of the laboratory's Quality Assurance program.

5. To minimize subjective effects, some specimens should be recounted by a different microscopist.

6. Asbestos laboratories shall be accredited by the National Voluntary Laboratory Accreditation Program.

7. At this time, performance evaluation samples for asbestos in air are not available for Removal Program Activities.

9.2 PCM Requirements

1. Examine reference slides of known concentration to determine the analyst's ability to satisfactorily count fibers. Reference slides should be maintained as part of the laboratory's quality assurance program.

2. Examine field blanks to determine if there is contamination by extraneous structures during sample handling.

3. Some samples should be relabeled then submitted for counting by the same analyst to determine possible bias by the analyst.

4. Participation in a proficiency testing program such as the AIHA-NIOSH proficiency analytical testing (PAT) program.

10.0 DATA VALIDATION

Results of quality control samples will be evaluated for contamination. This information will be utilized to qualify the environmental sample results accordingly with the project's data quality objectives.

11.0 HEALTH AND SAFETY

When working with potentially hazardous materials, follow U.S. EPA, OSHA, and corporate health and safety procedures. More specifically, when entering an unknown situation involving asbestos, a powered air purifying respirator (PAPR) (full face-piece) is necessary in conjunction with HEPA filter cartridges. See applicable regulations for action level, PEL, TLV, etc. If previous sampling indicates asbestos concentrations are below personal health and safety levels, then Level D personal protection is adequate.

12.0 REFERENCES

(1) Environmental Asbestos Assessment Manual, Superfund Method for the Determination of Asbestos in Ambient Air, Part 1: Method, EPA/540/2-90/005a, May 1990, and Part 2: Technical Background Document, EPA/540/2-90/005b, May 1990.

(2) Methodology for the Measurement of Airborne Asbestos by Electron Microscopy, EPA's Report No. 68-02-3266, 1984, G. Yamate, S.C. Agarwal, and R. D. Gibbons.

(3) National Institute for Occupational Safety and Health. NIOSH Manual of Analytical Method. Third Edition. 1987.

(4) U.S. Environmental Protection Agency. Code of Federal Regulations 40 CFR 763. July 1, 1987. Code of Federal Regulations 40 CFR 763 Addendum. October 30, 1987.

(5) U.S. Environmental Protection Agency. Asbestos-Containing Materials in Schools; Final Rule and Notice. 52 FR 41826.

(6) Occupational Safety and Health Administration. Code of Federal Regulations 29 CFR 1910.1001. Washington, D.C. 1987.

APPENDIX A

Tables

TABLE 1.<br><br>SAMPLE STATIONS FOR OUTDOOR SAMPLING		
Sample Station Location	Sample Numbers	Rationale
Upwind/Background[1]	Collect a minimum of two simultaneous upwind/background samples 30º apart from the prevailing windlines.	Establishes background fiber levels.
Downwind	Deploy a minimum of 3 sampling stations in a 180 degree arc downwind from the source.	Indicates if asbestos is leaving the site.
Site Representative and/or Worst Case	Obtain one site representative sample which shows average condition on-site or obtain worst case sample (optional).	Verify and continually confirm and document selection of proper levels of worker protection.

[1] More than one background station may be required if the asbestos originates from different sources.

APPENDIX A (Cont'd)

Tables

TABLE 2<br><br>SAMPLE STATIONS FOR INDOOR SAMPLING		
Sample Station Location	Sample Numbers	Rationale
Indoor Sampling	If a work site is a single room, disperse 5 samplers throughout the room.<br><br>If the work site contains up to 5 rooms, place at least one sampler in each room.<br><br>If the work site contains more than 5 rooms, select a representative sample of the rooms.	Establishes representative samples from a homogeneous area.
Upwind/Background	If outside sources are suspected, deploy a minimum of two simultaneous upwind/background samples 30° apart from the prevailing windlines.	Establish whether indoor asbestos concentrations are coming from an outside source.
Worst Case	Obtain one worst case sample, i.e., aggressive sampling (optional).	Verify and continually confirm and document selection of proper levels of worker protection.

APPENDIX B

Figures

FIGURE 1. Transmission Electron Microscopy Filter Cassette

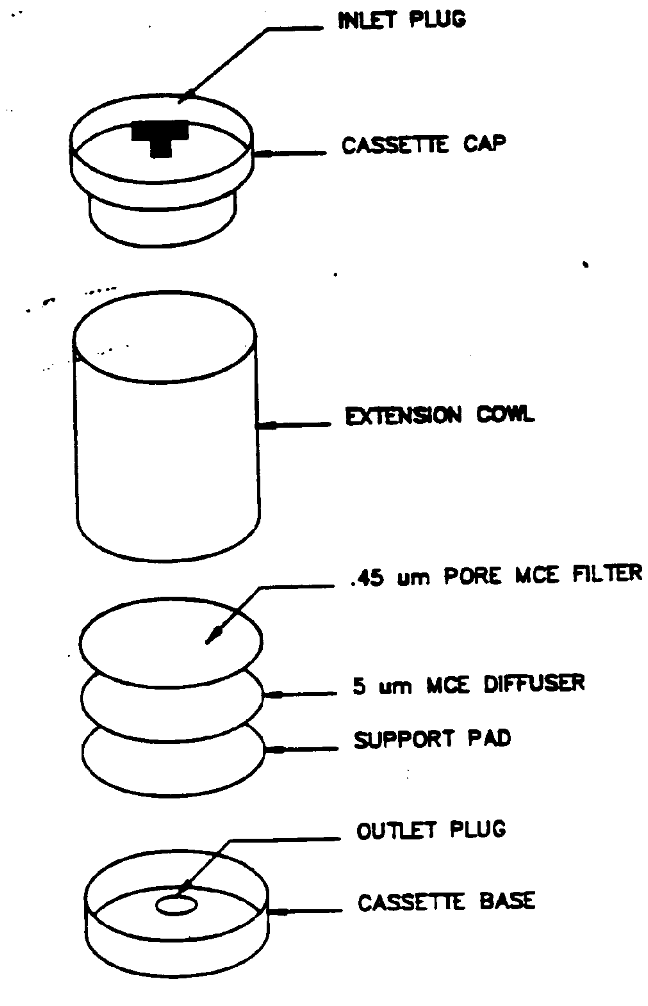

APPENDIX B (Cont'd)

Figures

FIGURE 2. Phase Contrast Microscopy Filter Cassette

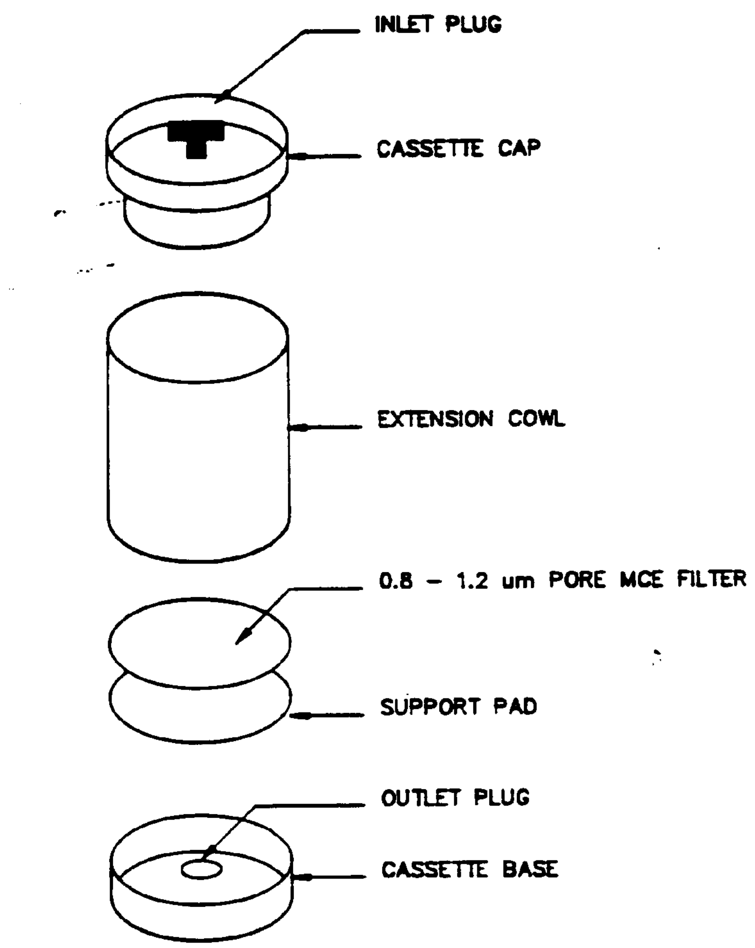

APPENDIX B (Cont'd)

Figures

FIGURE 3. Calibrating a Personal Sampling Pump with a Bubble Meter

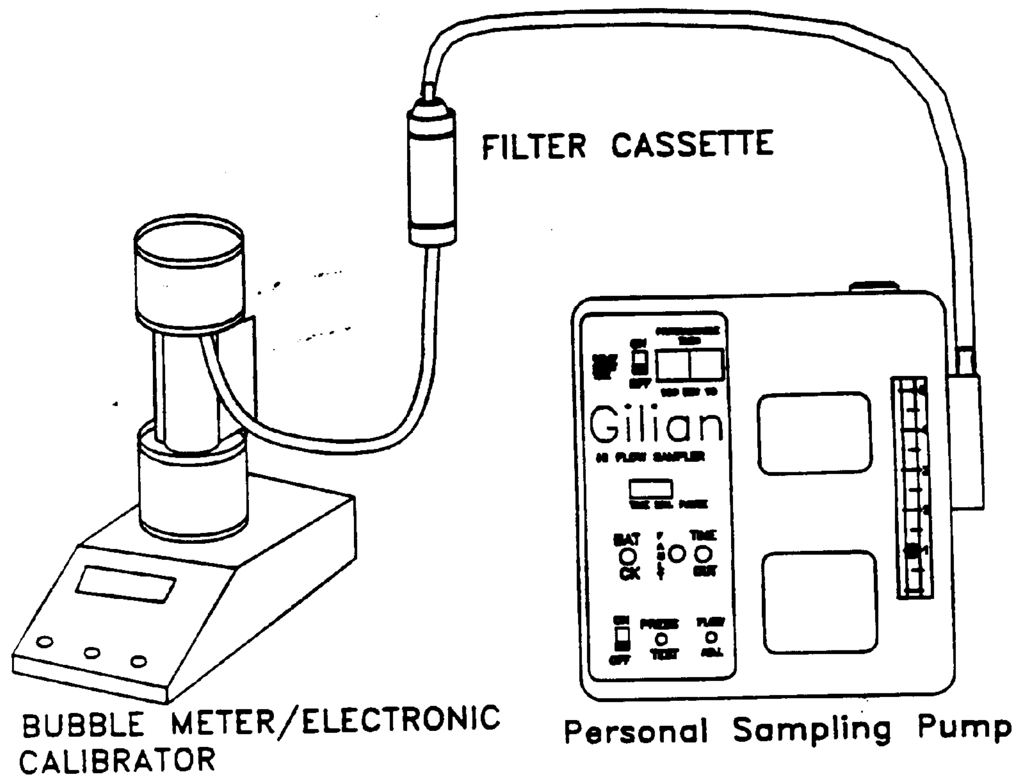

APPENDIX B (Cont'd)

Figures

FIGURE 4. Calibrating a Rotameter with a Bubble Meter

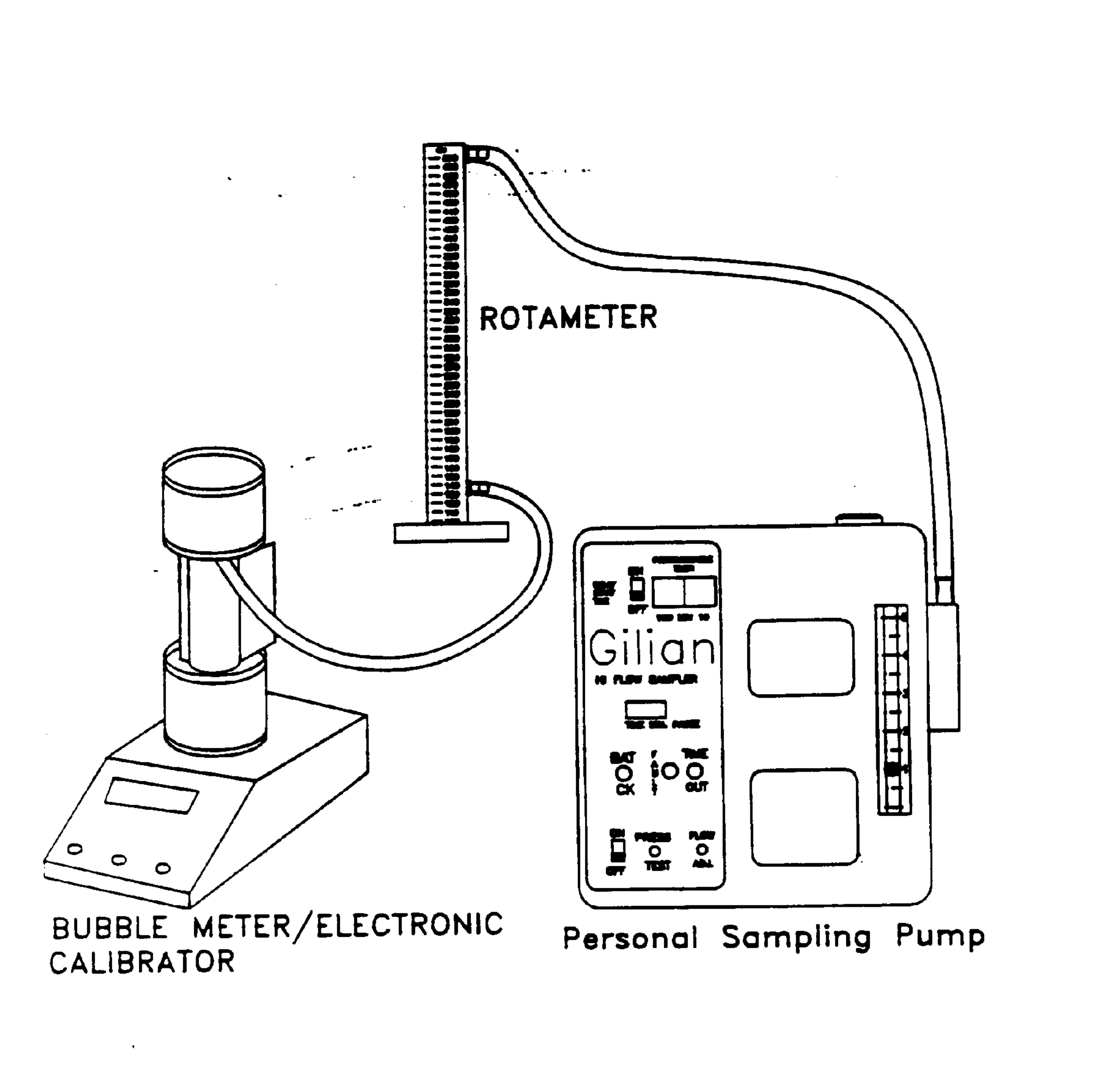

APPENDIX B (Cont'd)

Figures

FIGURE 5. Calibrating a Sampling Pump with a Rotameter

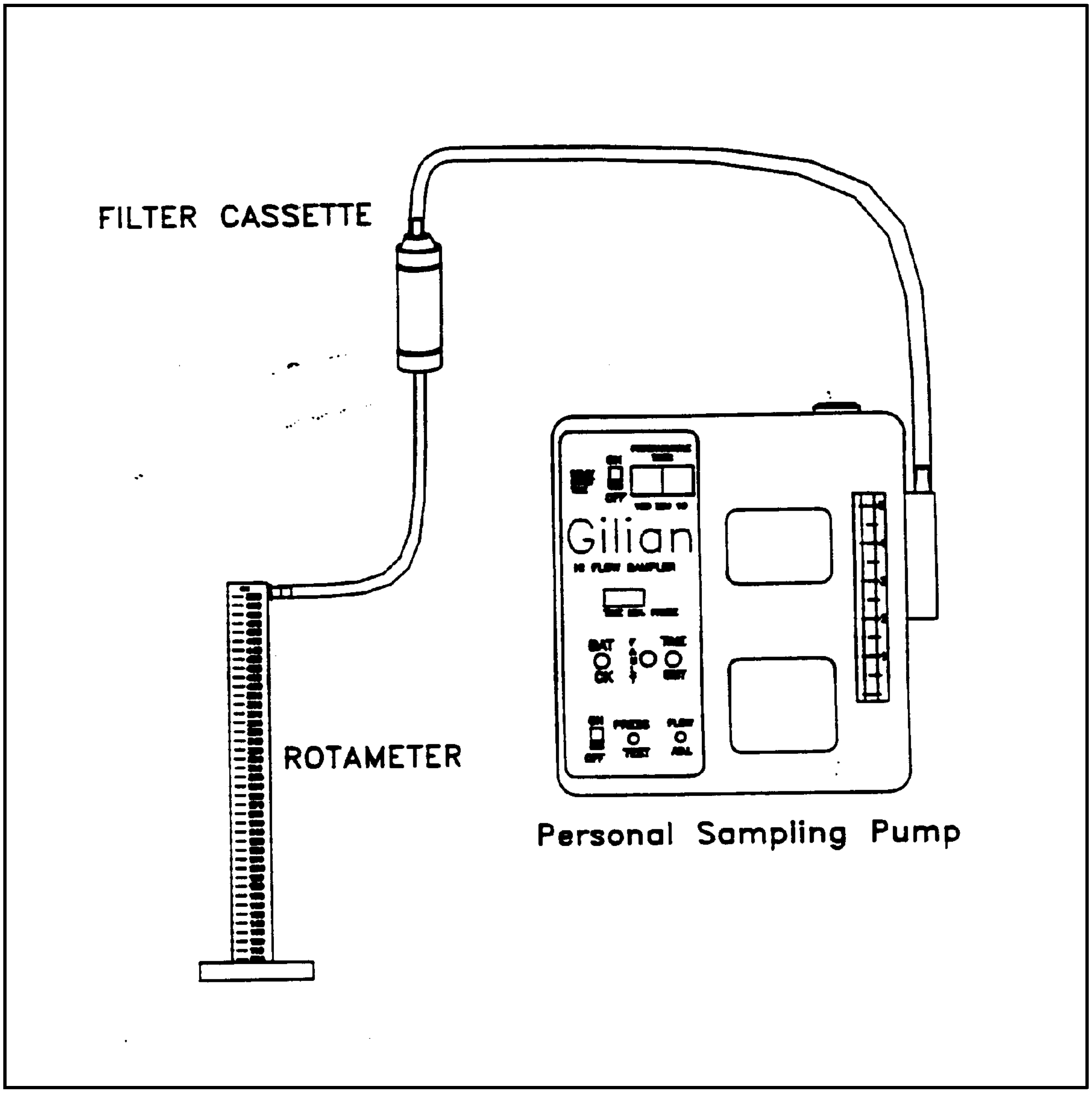

APPENDIX B (Cont'd)

Figures

FIGURE 6. Personal Sampling Train for Asbestos

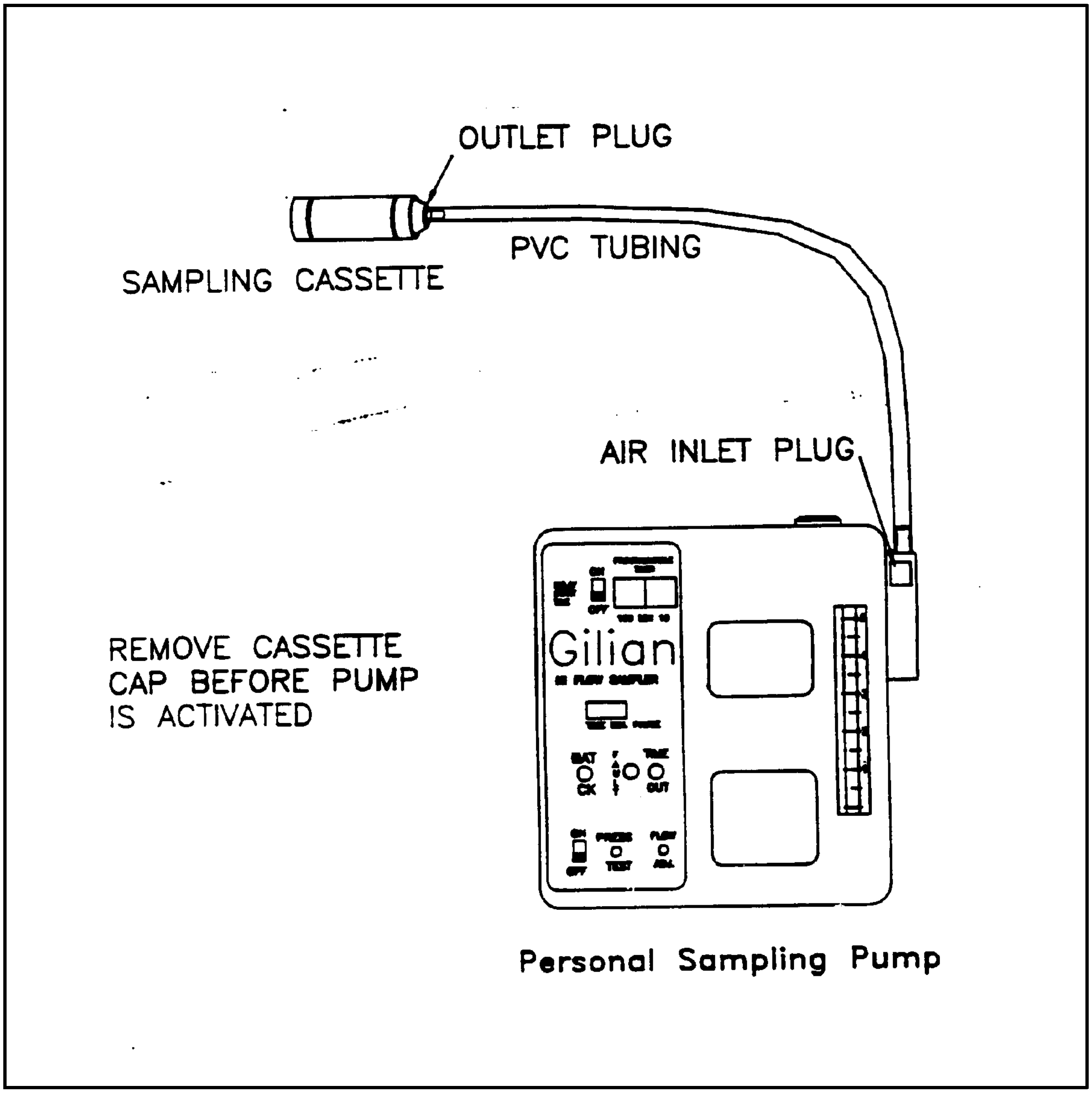

APPENDIX B (Cont'd)

Figures

FIGURE 7. High Flow Sampling Train for Asbestos

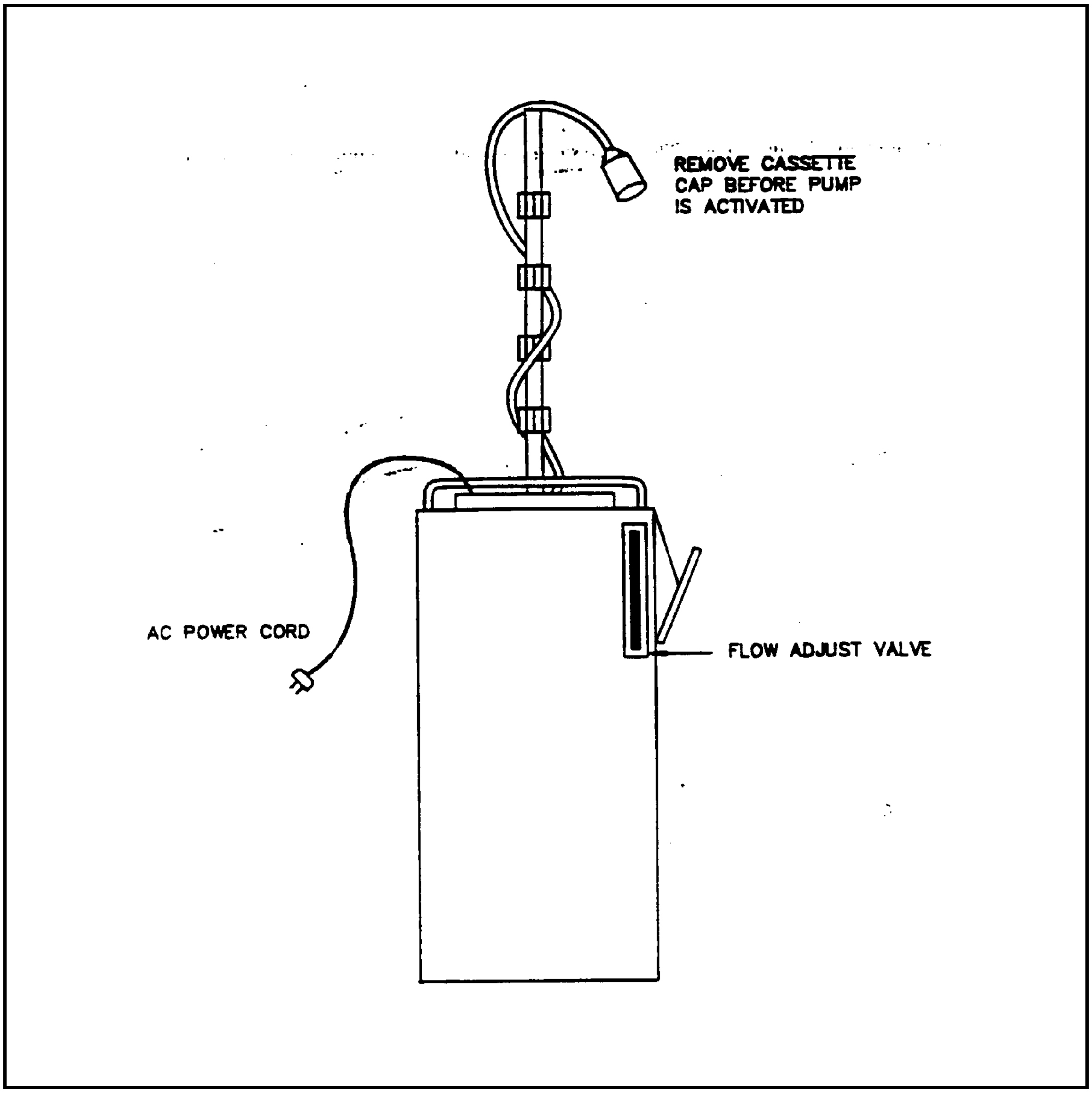

STANDARD OPERATING PROCEDURES

ACTIVITY-BASED AIR SAMPLING FOR ASBESTOS

CONTENTS

1.0 SCOPE AND APPLICATION

2.0 METHOD SUMMARY

3.0 SAMPLE PRESERVATION, CONTAINERS, HANDLING AND STORAGE

3.1 Sample Preservation
3.2 Sample Handling, Container and Storage Procedures

4.0 INTERFERENCES AND POTENTIAL PROBLEMS

4.1 Area Selection
4.2 Flow Rate Considerations
4.3 Transmission Electron Microscopy (TEM) Specimen Preparation Methods

4.3.1 Direct-Transfer TEM Specimen Preparation Methods
4.3.2 Indirect TEM Specimen Preparation Methods

4.4 Sampling Cassette Orientation

5.0 EQUIPMENT/APPARATUS

6.0 REAGENTS

7.0 PROCEDURES

7.1 Pre-Site Sampling Preparation
7.2 Calibration Procedures

7.2.1 Calibrating a Personal Sampling Pump with a Rotameter
7.2.2 Calibrating a Personal Sampling Pump with an Electronic Calibrator

7.3 Meteorology
7.4 General Sampling Information
7.5 Generic Activity-Based Sampling Scenario/Raking

CONTENTS (cont'd)

7.6 Site-Specific Activity-Based Sampling Scenarios

7.6.1 ATV Riding
7.6.2 Child Playing in the Dirt
7.6.3 Gardening/Rototilling
7.6.4 Weed Whacking/Cutting
7.6.5 Digging
7.6.6 Lawn Mowing
7.6.7 Walker with Stroller
7.6.8 Jogging
7.6.9 Two Bicycles
7.6.10 Basketball Scenario

7.7 Cumulative Exposure Scenario
7.8 Background/Reference Sampling
7.9 Perimeter Sampling
7.10 Soil Sampling

8.0 CALCULATIONS

9.0 QUALITY ASSURANCE/QUALITY CONTROL

10.0 DATA VALIDATION

11.0 HEALTH AND SAFETY

12.0 REFERENCES

13.0 APPENDICES

A - Tables

1.0 SCOPE AND APPLICATION

As a result of a directive issued by the United States Environmental Protection Agency (U.S. EPA) Office of Solid Waste and Emergency Response (OSWER Directive 9345.4), estimating asbestos exposures resulting from suspension of soils is an area of increased importance to the Superfund Program. Such exposures may be estimated via monitoring and/or modeling methods. At present, models are not available to accurately estimate asbestos exposure associated with the disturbance of contaminated soil. Therefore, personal monitoring in the form of activity-based sampling (ABS) is the most appropriate technique to estimate exposure. Personal exposure is influenced by the activities performed, the duration of the activity and the site-specific soils of interest.

At a number of diverse sites across the county (Clear Creek Management Area, San Benito County, California (CA), El Dorado Schools, North Ridge Estates, Klamath Falls, Oregon, Slodusty Road, Garden Valley CA, Ambler Alaska), the U.S. EPA has demonstrated that disturbance of soil with low levels of asbestos (including soil concentrations less than 1.0 percent (%) as measured by Polarized Light Microscopy) can potentially result in significant concentrations (>0.1 structures per cubic centimeter) of respirable asbestos fibers in the breathing zone of individuals engaged in various physical activities. This may result in a cancer risk in excess of Comprehensive Environmental Response, Compensation and Liability Act (CERCLA) remedial objectives.

Since personal monitoring is more representative of actual exposure than samples obtained from a fixed downwind location (McBride 1999, Rodes 1995, Hildemann 2005), personal monitoring results are generally most relevant to CERCLA risk characterizations. Thus the best measure of actual exposure to an individual would be through the collection of personal air samples over the exposure period of interest (NIOSH 1977). However, at CERCLA sites, it is neither always possible nor practical to do so. EPA has thus developed a sampling procedure called ABS, designed to mimic the activities of a potential receptor.

As part of ABS, U.S. EPA or contractor personnel trained in hazard recognition and mitigation, serve as surrogates for the potentially exposed populace of interest. ABS simulates routine activities in order to mimic and evaluate or predict personal exposures from disturbance of materials potentially contaminated with asbestos. Similar sampling approaches have been used to assess exposures to pesticides and lead (U.S. EPA 2000) and this technique has long been a cornerstone of industrial hygiene wherein workplace exposures are routinely assessed via personal exposure monitoring.

This document provides guidance for ABS for a particular set of activities or scenarios. Personal monitoring may be conducted during various activities such as raking, All-Terrain Vehicle (ATV) riding, rototilling, digging, a child playing in the dirt, weed whacking, lawn mowing, walking with a stroller, bicycling, and playing basketball.

These are standard (i.e., typically applicable) operating procedures which may be varied or changed as required, dependent upon site conditions, equipment limitations or limitations imposed by the procedure. In all instances, the ultimate procedures employed should be documented and associated with the final report.

This document is not intended to be used as a substitute for a site-specific Quality Assurance Project Plan (QAPP) or a detailed Sampling and Analysis Plan (SAP). This document is intended to be used as a reference for developing site-specific QAPPs and SAPs.

Mention of trade names or commercial products does not constitute U.S. EPA endorsement or recommendation for use.

2.0 METHOD SUMMARY

There are two types of ABS that can be employed in the field: generic ABS and site-specific ABS. Generic ABS can be used with potentially contaminated soil and utilizes a rake to disturb the soil over a known area in conjunction with the collection of air samples to characterize potential exposure. Site-specific ABS is also used with contaminated soil; however, it utilizes site-specific activities to disturb the soil, such as riding ATVs, jogging or riding bikes. Although site-specific ABS provides a more realistic measure of fiber release, it can also be more resource intensive and it is recommended to be used after the generic ABS, if results deem necessary.

For all ABS events, asbestos samples should be collected from the breathing zones of the subjects at an appropriate flow rate. Special consideration should be given to characterizing exposure to children as it has been hypothesized that children are more prone to exposure than adults (U.S. EPA 2000) because they tend to be closer to the source. Sample flow rates, duration and final volume will need to be weighed against the number of grid openings that must be counted (cost factor) to obtain the needed sensitivity. Sampling periods should be of sufficient durations (averaging time) to facilitate collection of a representative sample and achieving the required level of sensitivity.

3.0 SAMPLE PRESERVATION, CONTAINERS, HANDLING AND STORAGE

3.1 Sample Preservation

No preservation is required for asbestos samples.

3.2 Sample Handling, Container, and Storage Procedures

1. Place a sample label on the cassette indicating a unique sampling number. Do not put sampling cassettes in shirt or coat pockets as the filter can pick up fibers or a static charge that could disturb the dust deposited on the filter media.

2. Samples must be handled gently with the filter inlet facing upward to avoid disturbing the particulate deposited on the filter and to minimize the potential of imparting a static charge to the cassette, which might alter the particulate deposition on the filter media.

3. Place the cassette individually in a manila-type envelope. Each envelope should be marked with the sample identification number, total volume, and date.

4. To the best extent possible, the sampling cassettes in the manila envelopes should be placed right side up so that the cassette inlet cap is on top and cassette base is on bottom. Place samples into a shipping container and use enough packing material to prevent jostling or damage. Samples must be handled gently so as not to disturb the dust deposited on the filter media. Do not use vermiculite or any other type of fibrous packing material for samples. If possible, hand carry to lab.

5. Provide appropriate documentation with samples (i.e., chain of custody and requested analytical methodology).

4.0 INTERFERENCES AND POTENTIAL PROBLEMS

4.1 Area Selection

When selecting areas for ABS, consideration should be given to the potential for off-site migration of contaminants and possible exposure of the public. Within the constraints of ABS, to the degree practical, particulate generation migration off-site should be minimized, and constraints or mitigation protocols established to eliminate public exposure. These constraints/mitigation protocols may include conducting the ABS in remote areas of the site, dust suppression using water mist, building a containment structure, etc. Air sampling should be conducted to document the airborne concentration of asbestos at the site perimeter during activities.

4.2 Flow Rate Considerations

For activities that generate a large quantity of dust (i.e., particulates), sample flow rates may need to be reduced accordingly to avoid overloading the filters. For example, a sampling pump flow rate of approximately 3.0 liters per minute (L/min) was found most effective at one site for monitoring for asbestos while riding ATVs on dusty soils while high soil moisture and reduced particulate generation at another site permitted a 5.0 L/min flow rate.

High flow rates may result in filter damage due to failure of its physical support associated with increased pressure drop, leakage of air around the filter mount so that the filter is bypassed or damage to the asbestos structures (breakup of bundles and clusters) due to increased impact velocities (ISO 10312). High flow rates can also tear the filters during initial pump startup due to the shock load placed on the filter when the pump is first started.

Sampling larger volumes of air and analyzing greater areas of the filter media can theoretically lower the limit of detection indefinitely. In practice, the total suspended particulate (TSP) concentration limits the volume of air that can be filtered as TSP can obscure asbestos fibers. The International Organization for Standardization (ISO) Method 10312 states that the direct analytical method cannot be used if the general particulate loading exceeds approximately 10% coverage of the collection filter. An airborne concentration of approximately 10 micrograms per cubic meter

(µg/m³), corresponding to clean rural air, results in approximately 10% coverage of the filter media based on a 4000-L sample.

The following formula from ISO 10132 may be used to calculate the analytical sensitivity:

$$S = \frac{A_t}{K A_g V}$$

Where:

S = Analytical sensitivity expressed in structures per liter

A_t = Active area in square millimeters of the collection media or filter

A_g = Mean area in square millimeters (mm^2) of the grid openings examined,

K = Number of grid openings examined

V = Volume of air sampled, in liters

NOTE: 25-millimeter (mm) cassettes have an effective filter area of 385 mm^2 and 37-mm cassettes have an effective filter area of 855 mm^2. The typical grid opening is 0.0057 mm^2. Note: Grid size will vary between laboratories and dimensions should be verified prior to calculating the number of grid openings that must be counted to achieve a particular level of sensitivity.

Table 1 provides an example of the minimum number of grid openings that must be counted in order to achieve various sensitivity and detection limits.

It is frequently more efficient to employ co-located samplers to collect a high and low volume of air. This increases the likelihood of at least one of the two samples being readable using the direct analytical method (ISO 10312) than to lose the sample due to overloading or having to analyze by the indirect method (ISO 13794).

4.3 Transmission Electron Microscopy (TEM) Specimen Preparation Methods

It can be argued that direct methods yield an under-estimate of the asbestos structure concentration because other particulate material with which they are associated conceals many of the asbestos fibers present. Conversely, indirect methods can be considered to yield an over-estimate because some types of complex asbestos structures disintegrate during the preparation, resulting in an increase in the numbers of structures counted.

4.3.1 Direct-Transfer TEM Specimen Preparation Methods

Direct-transfer preparation methods are intended to retain all particles in the same relative positions with respect to each other on the final TEM grids as on the original filter. The membrane filter, or a portion of it, is placed on a microscope slide with the sample face upward, and then collapsed by exposure to acetone vapor. The cleared filter is then etched in a low-temperature plasma asher, subsequently coated with carbon in a sputtering device and then peeled from the glass slide. A portion of the collapsed, etched and carbon-coated filter is then transferred to an electron microscope grid and then extracted with dimethylformamide, glacial acetic acid and water to remove the filter. Once the process is complete, the particles originally collected on the filter are bound in the carbon film and the grids can be observed on a transmission electron microscope (ISO 1995). Direct-transfer TEM specimen preparation methods have the following significant interferences:

- The particulate density on the filter, which in turn is controlled by the sampled air volume and the total suspended particulate concentration in the atmosphere being sampled, restricts the achievable detection limit.

- The precision of the result is dependent on the uniformity of the deposit of asbestos structures on the sample collection filter.

- Air samples must be collected so that they have particulate and fiber loadings within narrow ranges. If too high a particulate loading occurs on the filter, it is not possible to prepare satisfactory TEM specimens by a direct-transfer method. If too high a fiber loading occurs on the filter, even if satisfactory TEM specimens can be prepared, accurate fiber counting may not be possible.

4.3.2 Indirect TEM Specimen Preparation Methods

In the indirect preparation method the membrane filter, or a portion thereof, is placed on a microscope slide, sample face downward, and ashed in a low temperature asher until complete calcination of the filter is achieved. The ash is then recovered in distilled water and the solution then filtered on a polycarbonate filter. The indirect transfer method re-distributes the particulate on a new membrane filter.

Indirect TEM specimen preparation methods have the following interferences:

- The size distribution of asbestos structures is modified (clusters, matrices bundles, etc. may be broken up during sample preparation).

- There is increased opportunity for fiber loss or introduction of extraneous contamination from laboratory glassware, process water, etc.

- When sample collection filters are ashed, any fiber contamination in the filter medium is concentrated on the TEM specimen grid.

The direct analytical method (ISO 10312) is the preferred method and every reasonable effort should be made to prevent overloading of the filter, which would necessitate use of the indirect method. Samples that are overloaded may, at the discretion of the project management team, be analyzed by ISO Method 13794 "Ambient air – Determination of asbestos fibres – Indirect-transfer transmission electron microscopy method" (ISO 1999). Results of the ISO 13794 analysis should be reviewed discrete of the ISO 10312 samples and a decision made regarding combining the two data sets.

4.4 Sampling Cassette Orientation

Air sampling cassettes must be oriented with the open face pointing down to preclude large non-respirable particles from falling or settling onto the filter media.

5.0 EQUIPMENT/APPARATUS

- Personal sampling pumps, providing a flow rate from 0.020 L/min up to 4.0 L/min, battery powered
- High flow sampling pumps (i.e., Quik Take 30 or AirCon II), capable of providing a flow rate from 4.0 to 12 L/min, battery or alternating current (AC)
- Mixed cellulose ester (MCE) filter cassettes, 0.45 or 0.8 micrometer (µm), 25-mm diameter, purchased from a certified vendor with appropriate documentation (low filter background counts, consistent filter area, certified leak-free cassettes)
- Sampling setups, Tygon® tubing with Luer type adaptor
- Backpacks
- Sampling stands, for perimeter sampling
- Duct tape
- Tools, miscellaneous (e.g., screwdrivers, pliers, cutting tool, etc.)
- Envelopes, manila-type
- Whirlpak® bags
- Sample labels

- Chain of custody (COC) records
- Logbook and/or sampling worksheets
- Precision rotameter or primary flow standard appropriate for sampling flow rate
- Personal protective equipment (PPE), including but not limited to respirators, boots, gloves, eye protection, hard hat, to be determined based on type of activity and possible exposure
- Decon equipment (Plastic sheeting, liquinox®, buckets, brushes, water, Hudson sprayers, garbage bags, etc.)
- Power sources, e.g., line power, solar recharging batteries, power inverters, generators, etc.

6.0 REAGENTS

Reagents are not required for the preservation of asbestos samples.

7.0 PROCEDURES

7.1 Pre-Site Sampling Preparation

1. Determine the extent of the sampling effort (number of locations, repetitions, number of samples, etc.), the sampling methods to be employed, and the types and amounts of equipment and supplies needed.
2. Obtain necessary sampling equipment and ensure it is in working order and fully charged (if necessary).
3. Perform a general site survey prior to site entry in accordance with the site-specific Health and Safety Plan (HASP).
4. Once on-site the calibration is performed in the clean zone. The calibration procedures are listed in Section 7.2.
5. After calibrating the sampling pump, mobilize to the sampling location.

7.2 Calibration Procedures

To determine if a sampling pump is measuring the flow rate or volume of air correctly, it is necessary to calibrate the equipment. Sampling pumps should be calibrated on a routine basis and prior to use.

A rotameter can be used provided it has been calibrated with a primary calibrator. Typically rotameters are calibrated on a yearly basis. Sampling pumps can be calibrated prior to coming on-site in order to expedite on-site calibration. However, calibration must be verified on-site prior to use.

7.2.1 Calibrating a Personal Sampling Pump with a Rotameter

1. Refer to the manufacturer's manual for the Rotameter Operational Instructions.

2. Set up the calibration train using a rotameter, sampling pump and the sampling cassette that will be used during the sampling event. This train may be set up prior to field mobilization and will be checked in the field again prior to use.

3. To set up the calibration train, attach one end of the polyvinyl chloride (PVC) tubing (approx. 2 ft) to the cassette base; attach the other end of the tubing to the inlet plug on the pump. Another piece of tubing is attached from the cassette cap to the rotameter. Insure that the tubing and rotameter used to calibrate the pump do not restrict the airflow.

4. Assemble the base of the flow meter with the screw provided and tighten in place. The flow meter should be mounted within 6 degrees (^{o}) of vertical (Omega 1987).

5. Turn the sampling pump on.

6. Turn the flow adjust screw (or knob) on the personal sampling pump until the float ball on the rotameter is lined up with the pre-calibrated flow rate value on the rotameter. Note: rotameters should be marked with the previous calibration date and corresponding flow rates and scale.

7. A verification of calibration is generally performed on-site in the clean zone immediately prior to the sampling.

7.2.2 Calibrating a Personal Sampling Pump with an Electronic Calibrator

1. Refer to the manufacturer's manual for operational instructions.

2. Set up the calibration train using a sampling pump, electronic calibrator, and the actual sampling cassette or a representative filter cassette. The same lot of cassettes used for sampling should also be used for calibration.

3. To set up the calibration train, attach one end of the PVC tubing (approx. 2 foot) to the cassette base; attach the other end of the tubing to the inlet plug on the pump. Another piece of tubing is attached from the cassette cap to the electronic calibrator.

4. Turn the electronic calibrator and sampling pump on. Select a flow rate to calibrate.

5. Turn the flow-adjust screw or knob on the pump until the desired flow rate is attained on the rotameter.

6. Using the primary calibrator, obtain approximately 10 readings three times until the flow rate of ± 5% of the required flow is attained.

7.3. Meteorology

It is recommended that an onsite, portable, 3-meter meteorological station be established. If possible, sample after two to three days of dry weather and when wind conditions are representative for the climatology of the location based on month and time of day. Historical hourly wind speed and wind direction data should be analyzed before mobilization. Wind speed, wind direction, temperature, and station pressure should be recorded on the meteorological station data logger and real-time data should be available for review on the station display panel. Suggested meteorological station specifications can be found in Table 2, Appendix A or ERT SOP #2129, *Met One Remote Meteorological Station.* Alternatively, a nearby representative meteorological station, as determined by a meteorologist, may be used to acquire the necessary data.

7.4 General Sampling Information

For all activity-based sampling events, except as noted otherwise, asbestos samples will be collected from the breathing zones of the event participants. The breathing zone can be visualized as a hemisphere approximately 6 to 9 inches around an individual's face. Breathing zone samples provide the best approximation of the concentration of contaminants in the air that an individual is

actually breathing. Specific breathing zone heights should be determined on a project-by-project basis based on the anthropometrics for the study population and the participants' positions during the performance of each task.

If it is necessary to relieve a participant from the activity, another sample collector should be suited and ready to participate in the ABS prior to the personnel exchange. The participant will stop the activity, remove the backpack or belt, and pass it to the relief participant similar to the transfer of a baton in a relay race. The original participant will assist the relief participant with donning and adjusting the backpack or belt. The exchange is anticipated to take less than 60 seconds, therefore the sampling pumps and event time clock will not be halted during the exchange. If the exchange requires more than 60 seconds, the pump and event clock will be stopped until activity is re-initiated.

Sample volumes and detection/quantification limits should be specified in the site-specific QAPP with flow rates and sampling periods adjusted accordingly. Typical sensitivity limits that have been employed for risk assessment have been approximately 0.001 S/cc for ABS samples and 0.0001 S/cc for background or reference samples. Based on ISO 10312 Table 1, a sensitivity limit of 0.001 S/cc would require a sample volume of greater than 500 liters to keep the number of grid openings to be counted below 100. Similarly, a sample volume greater than 5000 L would be required to reach 0.0001 S/cc and count fewer than 100 grid openings. For all asbestos sampling, an asbestos sampling train consisting of 0.8-µm, 25-mm mixed cellulose ester (MCE) filter connected to a personal sampling pump will be used. The top cover from the cowl extension on the sampling cassette shall be removed ("open-face") and the cassette oriented face down for all asbestos filters. All samples should be collected open-faced unless a specific requirement for sampling closed-faced exists.

For activity based sampling, a personal sampling pump (or equivalent) or SKC Quick Take 30 will be calibrated to collect between 2 and 12 L/min of air through the filter depending on the capacity of the pump. The flow rate will be based upon the duration of time required to collect a minimum target volume of 560 L and provide a sensitivity limit of 0.001 S/cc.

Generally each activity based sampling event should be repeated a minimum of three times in an area to expose trends. This can be accomplished by a single participant repeating the activity three or more times or by having a single simulation with three or more participants. If soil moisture or seasonal variability is a concern, then three events for each different season or meteorological conditions may be appropriate.

The sampling pumps used should provide non-fluctuating airflows through the filter, and should maintain the initial volume flow rate to within ± 10% throughout the sampling period. A constant flow or critical orifice controlled pump typically meets these requirements. If the flow rate changes by more than 5% during the sampling period, the average of the pre- and post-sampling

rates will be used to calculate the total sample volume. If at any time the measurement indicates that the flow-rate has decreased by more than 30%, sampling should be terminated. Depending on the type of sampling pump used, it may be possible to salvage the sample if sufficient volume was collected; however, it may not be representative of the time it takes for the actual activity to be completed. Depending on the type of sampling pump used, the actual sampling time in hours and minutes before the sampling fault may be displayed and an actual sample volume calculated. If the fault was due to battery failure, it may be possible to check the post-sampling flow.

During certain ABS activities, participants may be fitted with two sampling pumps to collect a high-flow or volume and a low-flow or volume sample. Co-located samples are collected to sample a high and low volume of air to increase the likelihood of at least one of the two samples being readable using the direct analytical method (ISO 10312). Approximately 560 L (40 CFR 763) is collected for the low-flow samples and up to 4000 L for the high-flow samples. The targeted high volume is typically 1200 L, which permits counting approximately 54 grid openings for a sensitivity level of 0.001 S/cc.

7.5 Generic Activity-Based Sampling Scenario / Raking

The raking scenario, also referred to as the generic scenario, is appropriate for all sites with soils potentially contaminated with asbestos. Generic ABS should be employed in a grid pattern to evaluate the potential for fiber release from soil over a portion of the site. If the analytical results are above the criteria that were derived for the site, then remediation or institutional controls should be implemented or additional site-specific ABS should be undertaken. If the analytical results are below the criteria that were derived, then no further action may be necessary.

In this activity or simulation a participant will rake a lawn or garden area to remove debris such as rocks, leaves, thatch and weeds using a leaf rake with a rake width of approximately 20 to 28 inches. Participants should strive to disturb the top half-inch of soil with an aggressive raking motion. This depth will vary based on the objective of the scenario.

Each raking participant donning appropriate PPE will be fitted with a personal sampling pump contained in a backpack with the cassette secured to the shoulder straps near the operator's lapels in the breathing zone. Personnel will rake a lawn or garden area to remove debris for a minimum of 1 to 2 hours (flow rate and sensitivity level dependent). Raking will occur in a measured area with vegetation, soil or rocks/gravel and will occur in an arched motion raking from the left of the participant to the right. The participants will rake the debris towards themselves facing one side of the square for 15 minutes then the participant will turn 90 degrees clockwise and begin a new side. Participants will continue to rake each side of the square and rotate 90 degrees. Once several small piles of debris have been made, the participant shall pick up the debris and place it in a trashcan. The sequence of raking, rotating and picking up debris shall be repeated for the duration of the sampling period. The participant should stay in the same plot for the entire sampling period.

7.6 Site-Specific Activity-Based Sampling Scenarios

If site-specific ABS is undertaken, the number and types of activities as well as the types of scenarios should be based on current and potential land use. Reference to current and currently planned future land use and the effectiveness of institutional or legal controls placed on the future use of the land should be evaluated. Probable land use should be selected based on zoning and the existing land use of the site and adjacent areas.

Land use assumptions should be based on a factual understanding of site-specific conditions and reasonably anticipated use. The land use evaluated for the assessment should be based on a residential exposure scenario (i.e., the default worst-case) unless residential land use is not plausible for the site. Future land use assumptions should be consistent with reasonably anticipated future land use based on input from planning boards, appropriate officials, and the public.

7.6.1 ATV Riding

This scenario might be appropriate for recreational areas or other areas where ATVs are typically ridden where asbestos contamination is present. This activity is designed to be representative of two or more ATV participants riding on a course or trail. Riders should maintain their relative position (lead, middle, tail) throughout the activity.

Each ATV rider wearing appropriate PPE will be fitted with two personal sampling pumps set at two distinct flow rates, to collect approximately 560 and 1200 liters of air, because of filter overloading concerns. The cassettes for the personal sampling pumps will be attached to the shoulder straps of the backpack proximal to the riders' lapels in the breathing zone. It may be beneficial to attach a dust monitor (e.g., DataRAM) to the tail ATV to record dust levels and gauge dust loading. The sampling pumps will be carried in a backpack while the dust monitor, if used, will be mounted to the ATV.

Personnel will ride the ATVs around a course at the same time until a sufficient volume of air has been collected to achieve the required sensitivity limit of 0.001 S/cc of air. The riders, one lead rider and one following rider, will vary the vehicle speed between 5 and 30 miles per hour (mph). Riders will strive for an average speed of 10 mph. The average speed is a target speed only; vehicle speeds will be adjusted to meet track conditions. Vehicles will be equipped with a speedometer and odometer to record speeds and distance traveled. ATV riding and sampling should be conducted for 30 to 120 minutes in duration, depending on dust loading and required detection limits.

ATVs and ATV tires should be selected as appropriate for the area being studied. Specifically, the size (i.e., weight, horsepower, etc.) of the ATV should be appropriate for

the study area. The vehicle tires should have a tread pattern that is representative of those typically used in the area. Local ATV shops or ATV clubs should be consulted for guidance.

7.6.2 Child Playing in the Dirt

This scenario might be appropriate for sites where schools, playgrounds, parks or residential areas, etc. are contaminated with asbestos; the overarching criteria being areas where a child might be expected to play or dig in the dirt. This scenario was designed to be representative of a child playing in the dirt with a shovel and pail.

The event participant wearing appropriate PPE will be fitted with a personal sampling pump; the inlet to the filter will be at a height of approximately 1 to 3 feet above the ground to simulate a child's breathing zone. The actual pump unit should be secured in a backpack or on a belt.

A participant should sit on the ground while digging or scraping the top 2 to 6 inches of surface soil, placing it in a small bucket or pail and dumping it back on the ground. The activity will be paced such that soil will be placed in the bucket and dumped approximately every two to five minutes, regardless of the amount of material in the bucket. The bucket should be emptied rapidly from a height of approximately 12 inches, based on observations of two to four-year-olds playing in a sandbox.

A sampling period and flow rate to collect a sufficient volume of air will be determined as to achieve the project-specific detection/quantification limit. The sampling period will be divided into equal sub-periods to facilitate having the participant face each compass direction for an equal amount of time during the activity. This approach is designed to mitigate the effect of wind direction on potential exposure. Random head and body movement during the activity should further mitigate the impact of wind direction on exposure. Ideally, the participants will face each compass direction at least twice during the sampling event. For example, during a two-hour or 120-minute event, the participant might face North for 15 minutes, rotate to the East for 15 minutes, then South for 15 minutes, then West for 15 minutes and return to the North to repeat the cycle. Participants should move to a fresh patch of soil after the completion of each cycle (360 degree rotation).

7.6.3 Gardening/Rototilling

This scenario might be appropriate for sites where gardening or surface disturbance to a depth of approximately one foot is anticipated. This activity is designed to be representative of individuals participating in gardening activities using a rototiller.

Each rototilling participant donning appropriate PPE will be fitted with a personal sampling pump. The actual pump unit will be contained in a backpack with the cassette secured to the shoulder straps near the operator's lapels in the breathing zone.

Personnel will operate a rototiller for a minimum of two hours to loosen soil in the yard to a depth of approximately 12 inches. The depth chosen is area-specific and will need to be determined on a case-by-case basis. A rear tine rototiller in the six to eight horsepower range will be selected. Other types or sizes of tillers may be appropriate based on the soil conditions and type of gardening being conducted.

A 100 to 720-square-foot plot of land will be selected to till. The average size of a community garden in New Jersey was 720 square feet based on a survey conducted by Rutgers University in 1991 (Patel 1991). The edges will be delineated. Square plots are preferred. The rototiller operator will conduct typical associated activities such as removing rocks and debris from the tilled area. To account for the effects of varying wind direction on potential exposure, the operator will till the soil back and forth towards each side of the square continuously for 10 minutes, shut down the machine or place it in neutral, and rake or sort through the material for five minutes. The operator will then turn 90 degrees in a clockwise direction and repeat the previous 15-minute procedure. The operator will continue to rotate 90 degrees clockwise every 15 minutes until the two-hour sampling period is complete. The participant should stay in the same plot for the entire sampling period.

7.6.4 Weed Whacking/Cutting

This scenario might be appropriate for sites where lawn maintenance might be conducted such as in residential and commercial areas. This activity is designed to simulate a person trimming weeds and grasses.

Each weed-whacking participant will be fitted with a personal sampling pump. The actual pump unit will be contained in a backpack with the cassette secured to the shoulder straps near the operator's lapels in the breathing zone. Personnel wearing appropriate PPE will operate a gas or electric-powered string trimmer. A 25 to 35-cc gas or electric-powered trimmer with a 16 to 18- inch cutting swath will be selected. Trimming and edging will occur in a measured area with thick vegetation (typically 100 to 720-square feet, based on a typical residential garden) (Patel 1991). Trimming will be done using a side to side sweeping motion with the operator moving in a series of straight lines back and forth towards one side of the selected area for 10 minutes, resting five minutes, and turning 90 degrees in a clockwise direction before repeating this 15-minute procedure for the

duration of the sampling period. The participant should stay in the same plot for the entire sampling period.

7.6.5 Digging

Digging might be appropriate for sites where construction projects are likely to occur or where plants might be planted. Digging will occur in a measured area with vegetation, soil or rocks/gravel.

Each digger participant donning appropriate PPE will be fitted with a personal sampling pump contained in a backpack with the cassette secured to the shoulder straps near the operator's lapels in the breathing zone. The participants will dig a hole to approximately two feet deep and two feet (representative of planting a small shrub or digging a fencepost; site-specific dimensions should be specified in the QAPP/SAP) in diameter (Vodak 2004) and will place the soil next to the hole. The participants will then refill the hole with the soil that had been removed. Participants will then rotate 90 degrees in a clockwise direction and continue to dig and refill additional holes until the sampling period is complete. The sequence of digging, filling and rotating shall be repeated for the duration of the sampling period.

7.6.6 Lawn Mowing

Lawn mowing might be appropriate for sites where lawn maintenance might be conducted such as residential and commercial areas.

Each lawn-mowing participant will be fitted with a personal sampling pump contained in a backpack with the cassette secured to the shoulder straps near the operator's lapels in the breathing zone. Personnel wearing appropriate PPE will operate a gas-powered lawn mower. Mowing will occur in a measured area with thick vegetation and will occur in a shrinking square pattern. Participants will divide the area into a number of squares that decrease in size towards the center of the square by the width of the mower swath. Mower blades will be set at approximately 2 to 2.5 inches. A bag-less side discharge 3- to 5-horsepower lawn mower will be used for this exercise.

7.6.7 Walker with Stroller

This scenario might be appropriate for sites such as parks, paths or open-space. The actual pump unit will be secured in a backpack. The cassette for the personal sampling pump will be attached to the shoulder straps of the backpack proximal to the walker's lapel in the breathing zone. A second pump will be placed in the stroller at a child's breathing zone height.

During these events, walkers wearing appropriate PPE pushing a stroller will walk back and forth along a portion of a path until a sufficient volume of air has been collected to achieve the required detection limit. The walkers will vary their speed between 1.5 and 4 mph. Walkers will strive for an average speed of 2 mph. The average speed is a target speed only; speeds will be adjusted to meet trail conditions. Walkers should be equipped with a global positioning system (GPS) unit to estimate average speed and distance traveled.

7.6.8 Jogging

This scenario might be appropriate for sites such as parks, paths or open-space. The actual pump unit will be secured in a backpack. The cassette for the personal sampling pump will be attached to the shoulder straps of the backpack proximal to the jogger's lapel in the breathing zone.

During these events, joggers wearing appropriate PPE will run/jog back and forth along a portion of a path until a sufficient volume of air has been collected to achieve the required detection limit. The joggers will vary their speed between 2.5 and 5 mph. Joggers will strive for an average speed of 4 mph. The average speed is a target speed only; speeds will be adjusted to meet trail conditions. Joggers should be equipped with a GPS unit to estimate average speed and distance traveled.

Two or more joggers can participate in this activity. When multiple joggers participate, they should maintain their relative position throughout the event (lead, middle, tail). Joggers should be spaced five feet apart.

7.6.9 Two Bicycles

Bicycling might be appropriate for sites such as parks, paths or open-space. Two bicyclists wearing appropriate PPE will ride back and forth with one leading and one following along the length of the site portion of a path or ride around a site (no trail) until a sufficient volume of air has been collected to achieve the required detection limit.

The bicycling participants will each be fitted with personal sampling pumps. The actual pump units will be contained in backpacks with the cassettes secured to the shoulder straps near the cyclists' lapels in the breathing zone.

During these events, the bicycle riders will vary their speed between 3 and 15 mph. Riders will strive for an average speed of 8 mph. The average speed is a target speed only; bicycle speeds will be adjusted to meet trail conditions. Bicycles will be equipped

with a GPS to estimate average speed and distance traveled. Riders should maintain their relative position (lead, tail) throughout the activity.

7.6.10 Basketball Scenario

This scenario might be appropriate for sites where basketball courts are present. The basketball scenario was developed to simulate a group of recreational basketball players gathering to play a casual game of basketball for 120 minutes on an outdoor concrete or macadam court. Between four and 10 players wearing appropriate PPE can participate in this exercise.

- From 0 to 15 minutes, two of the players will sweep court with push brooms from the perimeter of the court to the center. While these two people are sweeping the court, the remaining personnel should mill about under the basket and take a few shots.

- From 15 to 30 minutes, shot practice participants stand around the key as for a free throw, with the exception that one of the participants is positioned under the basket to retrieve the ball after each shot. The player closest to the basket on the left side (facing the basket) takes two shots and the ball/shooter rotates counter clockwise after those two shots. Each person shoots consecutively until everyone has taken two shots. The entire group then rotates clockwise. This sequence should be repeated until time expires. Ideally, each player should shoot from each key position and take a turn retrieving the ball under the basket.

- From 30 to 45 minutes, each player takes turns practicing lay-ups. All players line up on the left side of the basket (facing the basket) and shoot one after another. The first person shoots then retrieves the ball for next person in line and so on. Players should use two basketballs with the second person bouncing the ball outside of the key as the first person shoots. Players should run a full cycle from left then a full cycle from right; repeating the left, right cycles until the interval time is up.

- From 45 to 60 minutes, shot practice as described in the 15 to 30 minute interval above will be conducted.

- From 60 to 75 minutes, a half-court game will be played to the degree practical.

- From 75 to 100 minutes, shot practice as described in the 15 to 30-minute interval above will be conducted.

- From 100 to 120 minutes, a lay-up drill as described in the 30 to 45 minute interval above will be conducted.

7.7 Cumulative Exposure Scenario

A cumulative exposure study might be appropriate for sites where individuals move about a site during the course of a day, with varying levels of exposure at multiple indoor and outdoor locations. The objective is to estimate aggregate and cumulative exposure to asbestos over the course of a day. Cumulative exposure studies should be conducted in order to increase understanding of linkages between sources of asbestos and subsequent exposure and dose to humans for use in mitigating risk and reducing exposure and disease.

Over periods of weeks, years or decades, exposures to environmental agents such as asbestos occur intermittently rather than continuously. Yet long-term health effects, such as cancer, are routinely projected based on an average dose over the period of interest (typically years), rather than as a series of intermittent exposures. Consequently, long-term doses are usually estimated by summing doses across discrete exposure episodes and then calculating an average dose for the period of interest (e.g., year, lifetime).

For the cumulative exposure studies, representative members of the population of interest should be selected for 24 hour sampling. The volunteers should be instructed to go about their day as usual. That is, they should not modify their schedule or activities just because they will be wearing a sampling pump.

A minimal description of exposure for a particular route must include exposure concentration and the duration. This is the method of choice to describe and estimate short-term doses, where integration times are of the order of minutes, hours or days. When projecting long term exposures, on the order of years or a lifetime, since it is typically impractical to sample for the entire exposure period, short-term exposure estimates are assumed to be representative of long-term periods and are integrated to estimate long-term exposures, typically with a safety factor to account for variability.

Observations of activities should be recorded throughout each cumulative exposure study, together with the other relevant factors including locations and activities during the study.

Samples will be collected using a personal air pump with a flow rate of approximately 3.5 L/min. Samples shall be collected open-faced with the inlet facing downward at a personal breathing zone height of 4 to 6 feet for 24 hours. Because the battery life for a personal monitor is typically eight to10 hours, the pump shall be changed out at approximately 8-hour intervals (keeping the same filter cassette). Each pump shall be pre-calibrated to 3.5 L/min prior to use. Each monitor shall be worn at normal breathing height during all waking hours. During sleep, the monitor will be placed

in the same room as the sleeping individual. The sampling cassette will be placed proximal to the breathing zone of the reclined participant.

Should a study subject participate in a high dust generating activity such as riding an ATV, the 24 hour sampling cassette event should be paused and a short term exposure sample should be collected on a separate cassette with an appropriately calibrated sampling pump. Once the high dust activity has been terminated, the original 24-hour cassette and pump should be resumed for the remainder of the sampling period. Results of the 2 or more samples, depending on the number of high dust generating events should be summed to derive the total 24-hour exposure data.

7.8 Background/Reference Sampling

Background/reference samples should be collected for all sampling events. A background or reference sample is defined as a sample collected upwind at a distance sufficient to prevent being influenced by the simulated activities and outside the site perimeter. To the degree practical, the area selected for background or reference sampling should be free of known asbestos contamination. The background level should reflect the concentration of asbestos in air for the environmental setting on or near a site or activity location and can be used to evaluate whether or not a release from the site or activity has occurred. Background level does not necessarily represent pre-release conditions or conditions in the absence of influence from source at the site. A background level may or may not be less than the detection limit, but if it is greater than the detection limit, it should account for variability in local concentrations. Background or reference samples should be collected concurrent with ABS using stationary sampling pumps. Sampling and analytical parameters (sample volume grid opening count, etc.) should be prescribed to permit a detection limit approximately an order of magnitude below that of the ABS detection limit.

An Aircon II sampling pump (or equivalent) will be calibrated to collect 10 L/min for on-site and off-site air samples through the filter. The flow rate will allow a minimum target volume of 4000 L and will provide a sensitivity limit of 0.0001 S/cc. Lower volume air samples will be collected concurrently at the ambient air sampling locations. Personal sampling pumps will be utilized in the same manner with the same media at a flow rate between 2- and 3- L/min in order to collect a sample volume of approximately 1000 L. The target sensitivity of these samples is also 0.0001 S/cc when additional grids are counted in accordance with the method. Co-located samples are collected to sample a high and low volume of air to increase the likelihood of at least one of the two samples being readable using the direct analytical method (ISO 10312).

7.9 Perimeter Sampling

Perimeter samples are defined as samples collected upwind, downwind or crosswind of a specific activity. When selecting areas for ABS, consideration should be given to the potential for off-site migration of contaminants and possible exposure of the public. Within the constraints of ABS, to

the degree practical, particulate generation migration off-site should be minimized, and constraints or mitigation protocols established to eliminate public exposure. These constraints/mitigation protocols may include conducting the ABS in remote areas of the site, dust suppression using water mist, building a containment structure, etc. Air sampling should be conducted to document the airborne concentration of asbestos at the site perimeter during activities. Perimeter air monitoring should be conducted to:

- Document air quality during ABS and establish background or upwind levels of asbestos during site activities
- Monitor and document air quality during site activities near sensitive receptors
- Provide risk management information and address public confidence
- Reduce possible liabilities associated with ABS

Perimeter air sampling should be performed to ensure that ABS activities do not result in excessive airborne asbestos emissions from the site. Air samples should be collected and analyzed to determine the concentrations of asbestos at the site perimeter.

An Aircon II sampling pump (or equivalent) will be calibrated to collect 10 L/min for on-site and off-site air samples through the filter. The flow rate will allow a target volume of 4000 L and will provide a sensitivity limit of 0.0001 S/cc. Lower volume air samples will be collected concurrently at the perimeter sampling locations using personal sampling pumps, if loading is an issue. These pumps will be utilized in the same manner with the same media at a flow rate between 2- and 3-L/min in order to collect a sample volume of approximately 1000 L. The target sensitivity of these samples is also 0.0001 S/cc when additional grids are counted in accordance with the method. Co-located samples are collected to sample a high and low volume of air to increase the likelihood of at least one of the two samples being readable using the direct analytical method (ISO 10312).

7.10 Soil Sampling

A sufficient number of soil samples should be collected to characterize the study area. Since particulates are expected to be released from the entire study area, the primary objective of the soil sampling is to estimate the populations mean concentration. Composite samples are appropriate for characterizing study areas and a sampling design program such as Visual Sampling Plan is recommended for calculating the number and location of samples with the appropriate confidence intervals. Soil sampling should be conducted in accordance with ERT SOP #2012, *Soil Sampling.*

Soil characteristics should be documented in conjunction with the activity-based personal exposure monitoring using American Society of Testing and Materials (ASTM), Method D2488 - 00: *Description and Identification of Soils (Visual-Manual Procedure)*, soil moisture by ASTM Method D2216-05*: Standard Test Methods for Laboratory Determination of Water (Moisture)*

Content of Soil and Rock by Mass and grain size by ASTM Method D6913-04e1: *Standard Test Methods for Particle-Size Distribution (Gradation) of Soils Using Sieve Analysis* or Method D422-63 (2002): *Standard Test Method for Particle-Size Analysis of Soils.*

Soil samples should be representative of the soil. Table 3 provides examples of soil sampling depths, which may be disturbed by the activity being performed.

The relationship between the concentration of asbestos in a source material (typically soil) and the concentration of fibers in air that results when the source is disturbed is very complex, depending on a wide range of variables. To date, no method has been found that reliably predicts the concentration of asbestos in air given the concentration of asbestos in the source. Because of this limitation, this SOP emphasizes an empiric approach, where concentrations of asbestos in air at the location of a source disturbance are measured rather than predicted.

8.0 CALCULATIONS

The sample volume is calculated from the average flow rate of the pump multiplied by the number of minutes the pump was running (volume = flow rate X time in minutes). The sample volume should be submitted to the laboratory and identified on the chain of custody for each sample (zero for lot, and field blanks).

The concentration result is calculated by dividing the number of asbestos structures reported after the application of the cluster and matrix counting criteria by the sample volume (concentration = number of asbestos structures / sample volume).

9.0 QUALITY ASSURANCE/QUALITY CONTROL (QA/QC)

The following general QA procedures apply:

1. All data must be documented on field data sheets or within site logbooks. Record the following: date, time, location, sample identification number, pump number, flow rate, and cumulative time.

2. All instruments/equipment must be operated in accordance with operating instructions as supplied by the manufacturer, unless otherwise specified in the work plan. Equipment checkout and calibration activities must occur prior to sampling/operation and they must be documented.

3. Field blanks should be collected at a rate of one per twenty samples or one per sampling event, whichever is greater

4. Lot blanks should be collected at a rate of at least two per lot

5. Collocated samples should be collected at the frequency of one per sampling event

For TEM analysis, the following QC procedures apply:

1. Examine lot blanks to determine the background asbestos structure concentration.

2. Examine field blanks to determine whether there is contamination by extraneous asbestos structures during specimen preparation or handling.

3. Examine laboratory blanks to determine if contamination is being introduced during critical phases of the laboratory program.

4. To determine if the laboratory can satisfactorily analyze samples of known asbestos structure concentrations, reference filters shall be examined. Reference filters should be maintained as part of the laboratory's Quality Assurance program.

5. To minimize subjective effects, some specimens should be recounted by a different microscopist.

6. Asbestos laboratories shall be accredited by the National Voluntary Laboratory Accreditation Program.

7. At this time, performance evaluation samples for asbestos in air are not commonly available for Removal Program Activities; however, they should be considered on a case-by-case basis.

10.0 DATA VALIDATION

Results of QC samples will be evaluated for contamination. This information will be utilized to qualify the environmental sample results accordingly with the project's data quality objectives.

11.0 HEALTH AND SAFETY

When working with potentially hazardous materials, follow U.S. EPA, OSHA, and corporate health and safety procedures. More specifically, when entering an unknown situation involving asbestos, a powered air-purifying respirator (PAPR) (full face-piece) is necessary in conjunction with high-efficiency particulate air (HEPA) filter cartridges. See applicable regulations for action levels, permissible exposure levels (PEL) and threshold limit values (TLV). If previous sampling indicates asbestos concentrations are below personal health and safety levels, then Level D personal protection is adequate.

For all ABS, appropriate PPE, including Tyvek coveralls, protective gloves and foot wear, and a respirator with HEPA filter cartridges (P-100 or equivalent) should be worn to protect participants. Details regarding PPE and other protective measures should be specified in the site-specific Health and Safety Plan. Special

consideration should be given to the physical safety of the event participants as well as heat stress associated with performing vigorous activities in impermeable clothing.

12.0 REFERENCES

40 CFR Part 763 Asbestos Worker Protection

ERT SOP #2015, *Asbestos Sampling*.

Berman, Mark, Anthony Kolk, 2000. DRAFT: Modified Elutriator Method for the Determination of Asbestos in Soil and Bulk Materials, Revision 1.

Hildemann, L. 2005. Major Sources of Personal Exposure to Airborne Particulate Matter, Seminar at EPA Region IX, March 15, 2005.

International Organization for Standardization. 1995. *Ambient air - Determination of asbestos fibres - Direct-transfer transmission electron microscopy method,* Method 10312.

International Organization for Standardization. 1999. *Ambient air - Determination of asbestos fibres - Indirect-transfer transmission electron microscopy method,* Method 13794.

Januch, Jed. 2005. EPA Region 10 Standard Operating Procedure 10-1EU-001, Standard Operating Procedure for Sampling Airborne Asbestos Fibers in a Laboratory Enclosure- a Qualitative Procedure.

McBride, SJ et al. 1999. Investigations of the proximity effect for pollutants in the indoor environment. J Expo Anal Environ Epidemiol. 1999 9(6): 602–621. Nov–Dec.

National Institute for Occupational Safety and Health. 1977. Occupational Exposure Sampling Strategy Manual, Publication No. 77-173, January 1977.

Omega. 1987. FL-1600 Series Rotameters, http://www.omega.com/Manuals/manualpdf/M0379.pdf accessed February 2007.

Patel, Ishwarbhai, "Gardening's Socioeconomic Impacts", Journal of Extension, Volume 29, Number 4, Winter 1991, http://www.joe.org/joe/1991 winter/a1.html, accessed January 2004.

Rodes, CE., Kamens, RM and Wiener, RW. 1995. Experimental considerations for the study of contaminant dispersion near the body. Am Ind Hyg Assoc J; 56: 535–45.

U.S. EPA. 2000. *Strategy for Research on Environmental Risks to Children*, EPA/600/R-00/068, Office of Research and Development.

U.S. EPA. 2004. *Clarifying Cleanup Goals and Identification of New Assessment Tools for Evaluating Asbestos at Superfund Cleanups*, OSWER Directive 9345.4-05, August 10, 2004.

Vodak, Mark C., Arthur J. Vrecenak, 2004. Transplanting Trees and Shrubs. New Jersey Agricultural Experimentation Center, Rutgers Cooperative Research & Extension, Agricultural Experiment Station, Rutgers, The State University of New Jersey, Fact Sheet 376.

13.0 APPENDICES

TABLE 1. Minimum Number of Grid Openings Required To Be Counted to Achieve a Given Analytical Sensitivity and Detection Limit. (Adapted from ISO 10312)

TABLE 2. Suggested Meteorological Station Specifications

TABLE 3. Soil Sampling Depth Based on Activities Performed

TABLE 1. Minimum Number of Grid Openings Required To Be Counted to Achieve a Given Analytical Sensitivity and Detection Limit. (Adapted from ISO 10312)

Analytical Sensitivity Structures/cc	Limit of Detection Structures/cc	Volume of Air Sampled (Liters)					
		500	1000	2000	3000	4000	5000
0.0001	0.0003	1066	533	267	178	134	107
0.0002	0.0006	533	267	134	89	67	54
0.0003	0.0009	358	178	89	60	45	36
0.0004	0.0012	267	134	67	45	34	27
0.0005	0.0015	214	107	54	36	27	22
0.0007	0.0021	153	77	39	26	20	16
0.001	0.003	107	54	27	18	14	11
0.002	0.006	54	27	14	9	7	6
0.003	0.009	36	18	9	6	5	4
0.004	0.012	27	14	7	5	4	4
0.005	0.015	22	11	6	4	4	4
0.007	0.021	16	8	4	4	4	4
0.01	0.030	11	6	4	4	4	4

TABLE 2. Suggested Meteorological Station Specifications

Variable	Accuracy	Resolution
Wind Speed (horizontal and vertical)	± (0.2 m/s + 5% of observed)	0.1 m/s
Wind Direction (azimuth and elevation)	± 5 degrees	1.0 degrees
Ambient Temperature	± 0.5" C	0.1" C
Precipitation	± 10% of observed or ± 0.5 mm	0.3 mm
Pressure	± 3 mb (0.3 kPa)	0.5 mb
Solar Radiation	± 5% of observed	10 W/m^2

m/s = meters per second
"C = degrees Centigrade
mm = millimeters
mb = millibar
W/m^2 = watts per square meter
kPa = kilopascal

TABLE 3. Soil Sampling Depth Based on Activities Performed

**Activity Based Sampling Scenario**	**Soil Sampling Depth**
Raking (metal garden rake)	Surface to 3 inches
Raking (leaf rake)	Surface to 2 inch
ATV riding	Surface to 2 inch
Rototilling	Surface to 12 inches
Digging	Surface to depth of excavation
Child Playing in the dirt	Surface to 3 inches
Weed Whacking	Surface to 2 inches
Lawn Mowing	Surface to 2 inch
Walking with Stroller	Surface to 2 inch
Two Bicycles	Surface to 2 inch
Activities on solid surfaces such as asphalt or concrete	Microvacuum ASTM D 5755

U.S. EPA REGION 10 Office of Environmental Assessment
Field Standard Operating Procedure

Title: Sampling, Sample Preparation and Operation of the Fluidized Bed Asbestos Segregator

Effective Date: August 19, 2011

Document Number: OEAFIELDSOP-102 (Revision 1.0)

Review and Approvals

Signature__________ Date 8/15/11
Prepared by: Jed Januch, Environmental Protection Specialist
Office of Environmental Assessment

Signature__________ Date 8/18/11
Peer Review: Don Matheny, Senior Chemist
Office of Environmental Assessment

Signature__________ Date 8/22/11
QA Manager: Gina Grepo-Grove
Office of Environmental Assessment

Signature__________ Date 8/22/11
Environmental Services Unit Manager: Mark Filippini
Office of Environmental Assessment

Table of Contents

1.0 Purpose....3
2.0 Applicability....3
3.0 Summary....3
4.0 Definitions....4
5.0 Responsibilities....4
6.0 Interferences....4
7.0 Safety....4
8.0 Equipment and Supplies....5
9.0 Reagents and Standards....8
10.0 Procedures....8
10.1 Set up the Segregator, Glass Vessel, and Sampling Assembly....8
10.2 Sampling Considerations....10
10.3 Obtain a Test Sample....10
10.4 Decontamination of the Fluidized bed Asbestos Segregator....11
10.5 Collecting a Sample....12
10.6 Post Sampling Decontamination....13
11.0 Record Keeping, Chain of Custody, and Shipping....13
12.0 Calculations and Reporting Results....13
13.0 QA/QC Evaluation....15
13.1 Negative Controls....15
13.2 Performance Testing Samples....15
13.3 Calibration....15
14.0 Pollution Prevention....16
15.0 Waste Management....16
16.0 Change History....16

Figure 1 – Image of a fully assembled fluidized bed asbestos segregator....3
Figure 2 – Diagram of glass vessel....6
Figure 3 – Diagram of isokinetic splitter....7
Figure 4 – Isokinetic splitter attached to a check valve....8
Figure 5 – Screened stopper connected to check valve....9
Figure 6 – Stacked conductive filter cowls connected to isokinetic splitter....10
Figure 7 – Cyclone Attachment connected to the isokinetic splitter....12

1.0 Purpose

The purpose of this standard operating procedure (SOP) is to describe how to collect and process samples in the fluidized bed asbestos segregator (FBAS). An image of the fluidized bed asbestos segregator is included in Figure 1.

Figure 1 – Image of a fully assembled fluidized bed asbestos segregator.

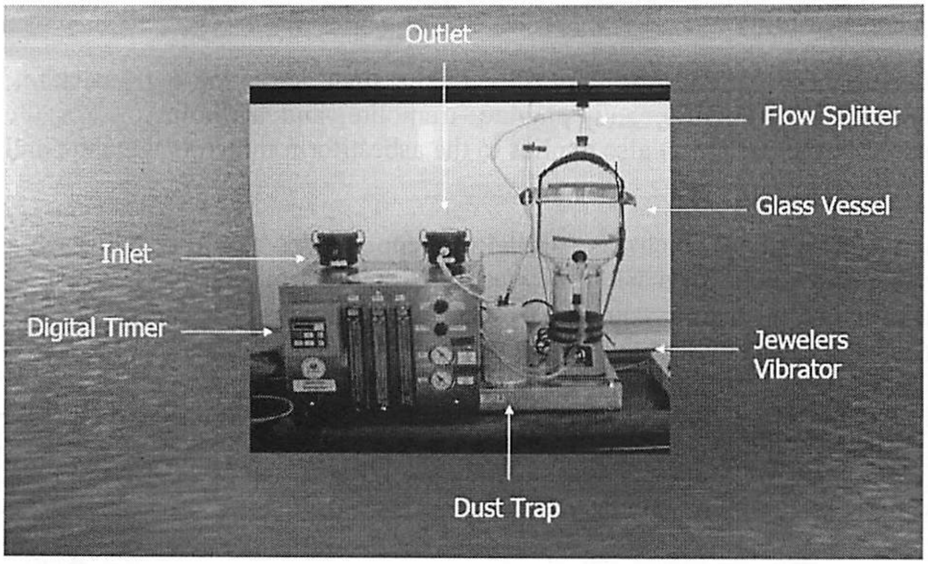

2.0 Applicability

This SOP applies to the preparation/collection of samples performed by or for the U.S. Environmental Protection Agency (EPA). It should be used as a sample preparation technique that segregates asbestos fibers from solid media, such as soil, and deposits these fibers onto an air filter, allowing an analysis of the filter to determine releasable asbestos content of the soil. Initial performance evaluation studies have shown that analyses of filters prepared using the FBAS technique are able to detect asbestos at levels that are much lower than most standard analytical methods for the analysis of asbestos in bulk materials. In addition, preliminary field-based testing suggests that results for soils prepared using the FBAS technique may correlate with activity-based air sampling results. Limitations of this technique are that it has only undergone limited inter-laboratory and field-based testing and development.

3.0 Summary

Soil, or other solid media, is collected from an area of interest. It is size-segregated by sieving and the fine fraction is then homogenized and fluidized in the FBAS. Small particles, nominally 10 micrometer (μm) aerodynamic diameter and smaller, are elutriated from the bulk material and collected on a filter. The filter is then analyzed by transmission electron microscopy (TEM) for asbestos fibers. Fibers are counted using the rules specified in the analytical method (typically ISO 10312:1995(E) or ISO 13794:1999(E)). The concentration of asbestos in the soil can be expressed as either asbestos structures per gram (g) of soil or as mass percent (g of asbestos per 100 g of soil).

4.0 Definitions

Aerodynamic Diameter - The expression of a particle's behavior in air as if it were a perfect sphere with unit-density (1 g per cubic centimeter) - that is - the diameter of spherical water droplet having the same terminal velocity as the fiber or fiber bundle.

Asbestiform – A specific type of mineral form in which fibers and/or fibrils possess high tensile strength and flexibility.

Asbestos – Asbestiform varieties of chrysotile (serpentine), crocidolite (riebeckite), amosite (cummingtonite-grunerite), anthophyllite, tremolite, and actinolite.[1] For the purposes of this SOP, the definition also applies to the asbestiform minerals winchite and richterite.

Fiber - An elongated particle which has parallel or stepped sides. A fiber will have a length greater than 0.5 µm, with a width between 0.25 and 3 µm, and have an aspect (length: width) ratio greater than or equal to 3:1. Fibers with a density of 3 g/cc, a width of 3.1 µm and an aspect ratio of 3:1 have an "aerodynamic diameter" of about 10 µm.

Fiber Bundle - A structure composed of parallel, smaller diameter fibers attached along their lengths. A fiber bundle may exhibit diverging fibers at one or both ends.

Structure - A single fiber or fiber bundle.

5.0 Responsibilities

It is the responsibility of the operator of the FBAS to collect samples in accordance with this SOP and the specifications provided in a site or project specific quality assurance project plan (QAPP). The operator will keep records concerning use of the FBAS in a bound notebook.

6.0 Interferences

High levels of non-asbestos dust particles may overload the surface of the filter and obscure asbestos fibers in the microscope field of view. Overloading can be minimized by monitoring the particulate load on the filter by screening the filter with a phase contrast microscope (PCM) - see Section 10.3 - and adjusting the sample amount placed in the FBAS and sampling duration accordingly to achieve optimum particulate load on the filter which is typically between 10% and 25%.

7.0 Safety

Inhalation of asbestos fibers will increase the risk of lung cancer, mesothelioma, and non-malignant lung and pleural disorders including asbestosis, pleural plaques, pleural thickening, and pleural effusions.[2] When handling materials suspected of containing asbestos, precautions should be taken to avoid inhalation exposures. Conduct work with

---

[1] Title 40 of the Code of Federal Regulations (CFR) Part 763.83

[2] ATSDR Web Site http://www.atsdr.cdc.gov/asbestos/asbestos_effects.html

materials suspected of containing asbestos in a safety hood equipped with a negative pressure high-efficiency particulate arrestance (HEPA) filter system.

Engineered safety features of the FBAS include: 1) a vacuum pump to maintain the system under negative pressure and contain asbestos contamination within the system, 2) the sample filter and a HEPA filter upstream of the vacuum pump to remove asbestos prior to the pump exhaust, 3) a diaphragm-style check valve to prevent the asbestos-containing materials (ACM) from draining out of the dust collector bottle, and 4) most of the components and tubing (with the exception of the glass vessel and cyclone attachment - if used) that contact asbestos are disposable to minimize handling and cleaning for contaminated equipment.

8.0 Equipment and Supplies

Sample Collection and Preparation

- Negative flow HEPA workstation or proper personal protective equipment, including respirators for handling unconfined material suspected of containing asbestos.
- 25- millimeter (mm) mixed cellulose ester (MCE) filter composed of mixed cellulose ester having a pore size equal to 0.8 µm (such as part number 225-231 from SKC)
- Notebook for recording sampling information.
- Small flat-bottomed metal scoop (do not use a rounded-bottom scoop)
- 250 milliliter (ml) or larger beaker with 50 ml (or finer) gradations
- 8 inch sieves (brass or steel); 6.3 mm opening and 0.85 mm opening (U.S. Standard Sieve No. 20), receiver pan and lid.
- Balance capable of weighing 1-20 g to ± 0.01 g
- U.S. Silica ASTM 20/30 sand
- Sample containers (e.g., 250 ml glass bottles with caps)
- Metal spatula
- Drying oven capable of operating at 60 ° C ± 5° C
- (Optional) Phase Contrast Microscope (PCM) and sample preparation kit

Fluidized Bed Segregator Unit Parts

- Inlet HEPA filter (such as part number 5169K72 (housing) and 9179K14 (HEPA) from McMaster Carr)
- Bypass HEPA filter (such as part number 5169K72 (housing) and 9179K14 (HEPA) from McMaster Carr)
- Polyvinyl chloride (PVC) tubing, 1/4 inch inner diameter (ID) (such as McMaster-Carr part number 5231K161)
- PVC tubing, 3/8 inch ID (such as VWR part number 60985-544)32 ounce (oz) Multipurpose calibration jar, polypropylene - used for oversize dust collection (SKC part no. 225-111)
- Jewelers Vibrator (such as Sunburst Investment Vibrator 110 volt AC, 60 Hertz [Hz], 0.7 amp (A))
- Allegro Industries vacuum pump, Part Number 9804-88, oil-less rotary vane, 1/10 horsepower (HP) 115 volt AC, 60 Hz.

Sample Processing

- Calibrated barometer and thermometer to measure local barometric pressure and temperature.
- Fluidized bed chamber assembly:
    - o Glass vessel (per diagram in Figure 2) – Replacements available through Precision Glassblowing of Colorado, 14775 East Hinsdale Avenue – Centennial, Colorado 80112-4243, phone (303) 693-7329.

Figure 2 – Diagram of glass vessel.

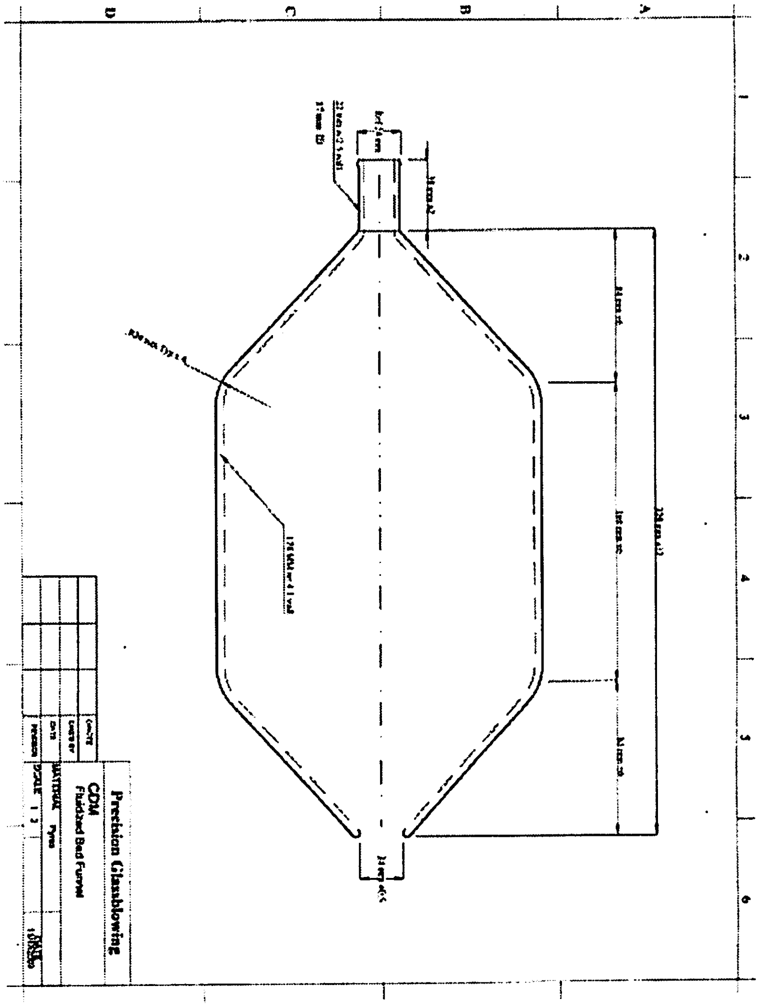

- o Two check valves (plastic diaphragm, 5/16 inch tube ID, such as McMaster-Carr part number 47245K24)
- o No. 2 or No. 3 black rubber stopper with standard ¼ inch hole, with nylon mesh (20 µm to 45 µm) covering hole (Use No. 3 stopper if stopper is to be removed after use, otherwise, use No. 2 stopper, such as McMaster-Carr part number 9545K27).
- o Nylon elbow connectors, 1/4 inch ID (Such as Cole–Parmer Part# L0-4NN)
- o Isokinetic sample flow splitter assembly (per diagram in Figure 3) – replacements available through Cascade Plastics Corporation, Inc. 7009-45th Street Ct. East – Fife, Washington 98424, phone 1 (800) 699-3460.

Figure 3 – Diagram of isokinetic splitter.

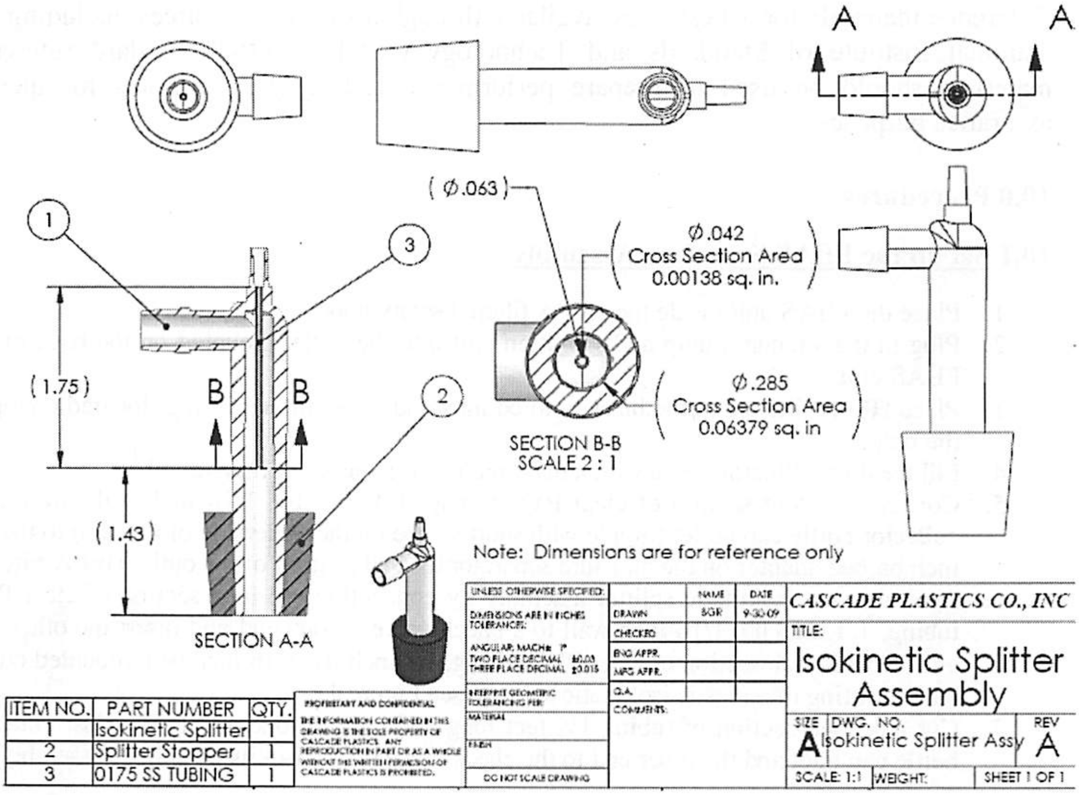

- MCE filter cassettes with 25- mm, 0.8 µm pore size (such as part number 225-231 from SKC).

- Washed (3X in deionized water) and dried (12 hours at 60°C) ASTM 20/30 quartz sand (e.g., U.S. Silica brand or Restek Ottawa Sand), 15-19 g per sample depending on the results from the test sample described in Section 10.3 of this SOP.
- Nilfisk (or equivalent) HEPA vacuum with hose and brush attachment.
- Digital timer

9.0 Reagents and Standards

Reference materials for asbestos are available through a variety of sources, including the National Institute of Standards and Technology (NIST). NIST standard reference materials should be used to prepare performance testing (PT) samples for quality assurance purposes.

10.0 Procedures

10.1 Set up the FBAS Sampling Assembly

1. Place the FBAS unit inside the HEPA filtered safety hood.
2. Plug in the vacuum pump and vibration unit into the outlet mounted on the back of the FBAS unit.
3. Place HEPA filters into the black colored inlet and outlet filter housings located on top of the unit.
4. Fill the dust collector bottle with a maximum of 1-2 inches of mineral oil.
5. Connect a 1-foot section of clear PVC tubing, 1/4 inch ID, 1/16 inch wall, from dust collector bottle cap outlet (nipple with shorter tube on the underside of the cap) to the 1/4 inch barbed adapter on the moisture separator located in front of the outlet HEPA filter.
6. Assemble the isokinetic splitter assembly by connecting a 6-inch section of clear PVC tubing, 1/4 inch ID, 1/16 inch wall to a check valve on one end and insert the other free end into a 2-inch section of clear PVC tubing, 3/8 inch ID, 1/16 inch wall mounted on the barbed fitting of a plastic isokinetic splitter (see Figure 4).
7. Cut a second section of tubing 1½ feet long and connect one end to the dust collector bottle cap inlet and the other end to the check valve on the isokinetic splitter assembly.

Figure 4 – Isokinetic splitter attached to a check valve.

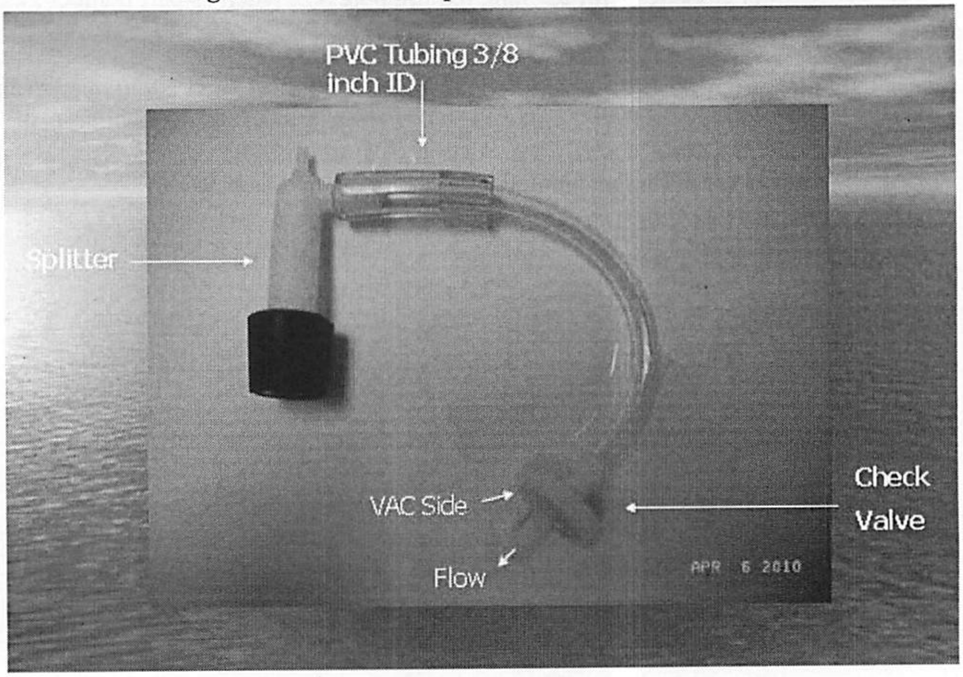

Caution – If you are replacing a previously used HEPA filter or tubing, you should assume that these items, as well as the contents of the dust collector bottle and the inlet filter housing, are contaminated with asbestos.

Note – Make certain the check valves connected to the isokinetic splitter assembly and meshed stopper assembly are facing the right direction in relation to the direction of air flow. The abbreviation for vacuum "VAC" will face in a direction going away from the splitter and toward the screened stopper.

7. Connect one end of a 6-inch section of PVC tubing to the bottom check valve and the other end to a Nylon barbed elbow connector, 1/4 inch ID, that's been inserted into a number 2 screened bottom stopper (see Figure 5).

Figure 5 – Screened stopper connected to check valve.

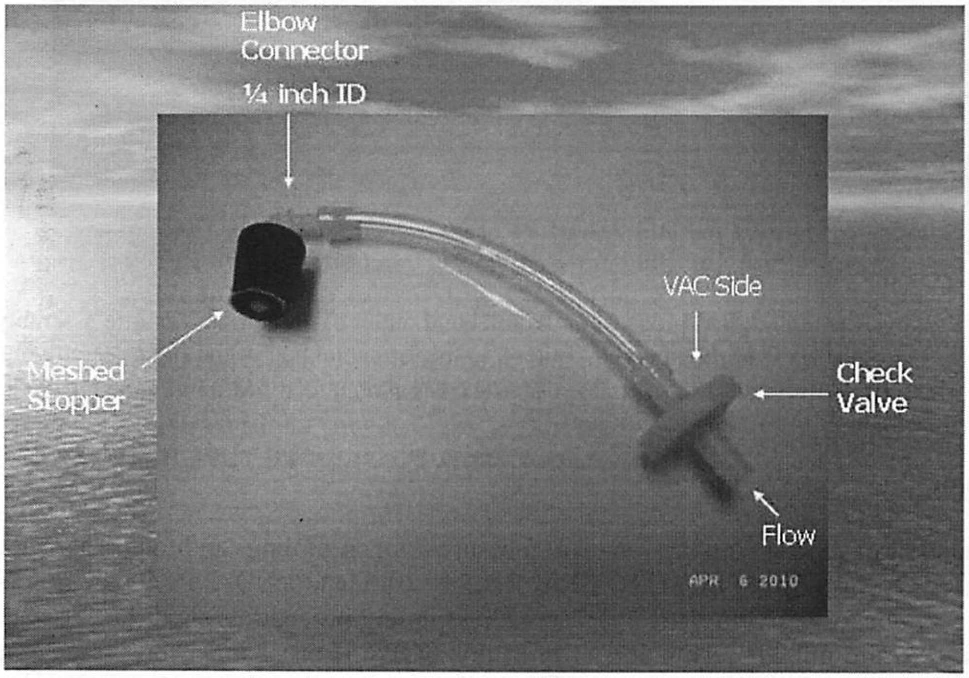

8. Push the stopper into the bottom opening of the glass vessel.
9. Mount a clean glass vessel onto the plastic holder connected to the top surface of the vibrator unit. Fasten the safety belt and the front elastic cord to secure the glass vessel.
10. Seat the isokinetic splitter stopper into the top opening of the glass vessel so the outlet peg is facing up.
11. Remove the cap on the bottom of the conductive cowl of a filter cassette and insert an additional cowl (stack one cowl on top of the other) and cap the bottom of the additional cowl (see Figure 6).

Figure 6 – Stacked conductive filter cowls connected to isokinetic splitter.

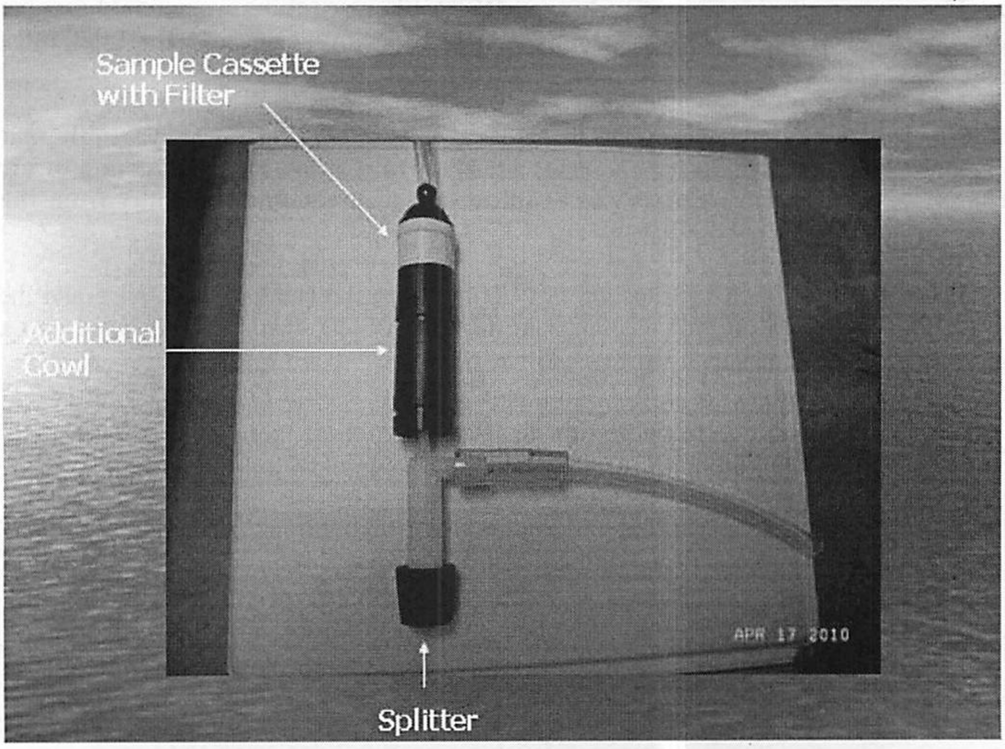

12. Remove the red colored plugs from the inlet and outlet of the sample cassette assembly.
13. Connect the PVC tubing marked "Filter", to the top outlet peg of the filter cassette outlet.
14. Seat the bottom inlet peg of the filter cassette assembly to the top outlet peg of the isokinetic splitter.

10.2 Sampling Considerations

Quality assurance and quality control measures for sampling and analysis will be performed according to the U.S. EPA Region 10 Generic QAPP or an approved site/project-specific QAPP. However, specific sampling procedures should be performed according to the guidance contained within this SOP. If procedures to obtain samples differ from this SOP, the changes should be well documented in the project notebook.

Samples should be examined during test sampling and periodically during regular sample collecting to determine the degree of filter loading. Filters can be examined visually, or with the aid of a PCM (preferable). If a PCM is used, follow the sample preparation and analytical instructions found in the National Institute of Occupational Safety and Health (NIOSH) Method 7400, Issue 2.

10.3 Obtain a Test Sample

The purpose of the test sample is to determine how much soil sample mass is necessary to properly load the collection filter on the FBAS.

1. Use the flat-bottomed scoop to collect about 250 ml of representative soil sample. Collect the sample from the uppermost 1 inch of surface soil or as otherwise specified in the QAPP. Take the entire soil column (rectangular cross-section for entire linear scoop). Move the scoop slowly (<2 cm/s) to reduce segregation at the cutter edges.

NOTE: Proper sub-sampling technique is critical to obtain representative samples of particulate solids. Do not use a rounded-bottom scoop for soil sampling.

2. If the soil is moist and does not disaggregate into individual grains easily, it should be dried before proceeding. Dry in a drying oven at 60° C for 12 hours.
3. Assemble the sieves so that the largest opening (6.3 mm) sieve is on top of the 0.85 mm sieve and the collection pan is on the bottom.
4. Put the sample on the top sieve of the sieve stack. Place the lid on the top sieve and shake the sieves by hand back and forth for 2-5 minutes. Alternatively, an automatic sieve shaker can be used to increase shaking time and improve sorting.
5. Use the material collected in the bottom pan for the test sample.
6. Weigh out at least three replicate 1-5 g portions of sample and 15-19 g portions of sand to help determine the optimum sample/sand combination that results in optimum filter loading. An average estimate of loading between the three replicate tests per combination will ensure that optimum filter loading has been achieved.
7. Combine the sample and the sand so that the combined weigh of the mixture equals 20 g.
8. Record the weight of the sample and the weight of the sand in the notebook.
9. Remove the isokinetic splitter stopper and place a combined mixture of sample/sand into the glass vessel, then replace the isokinetic splitter stopper.
10. Set the digital timer for a minimum of three minutes.
11. Depress the "on" button on the digital timer which will engage the vacuum pump and the vibration unit. The pump and vibration unit will shut off after sample time (typically 3 minutes) has elapsed.
12. Examine the filter using PCM (preferable) or visually when the segregator completes the sample. If the segregator did not clog and the filter is optimally loaded (between 10% and 25% particulate load), run the remaining test samples. Use a new filter cassette assembly and a clean glass vessel for each sample.
13. Repeat steps 9-12 with the different sample/sand combinations, increasing or decreasing the soil mass as appropriate. Compare the filters to determine which will give the optimum filter loading. Try different amounts of soil but be sure to use a balance of sand so that the sample is 20 g.

When the optimum soil sample mass for the FBAS has been determined, make a note in the notebook as to the proper mass and continue with formal sample collection (Section 10.5).

10.4 Decontamination of the FBAS

1. Remove the glass vessel from the vibration unit and lay it on its side in front of the FBAS.
2. Remove the isokinetic splitter and the meshed stopper from the ends of the glass vessel and discard in the asbestos equipment waste container.
3. Pour the spent sample/sand material into the asbestos sample waste container.
4. Use the Nilfisk HEPA vacuum to evacuate fibers by placing the HEPA vacuum hose over one of the ends of the glass vessel to extract the airborne fines and visible spent sample material off the inner surface of the vessel. This should only take 15-30 seconds.
5. Remove the HEPA vacuum hose.

6. Place the wand of a garden sprayer (filled with amended –soapy water) into the glass vessel and wet the inside of the vessel. Seal the openings of the vessel with tape (either lab tape or duct tape).
7. Remove the vessel from the HEPA hood and clean with soap and water in a wash basin. Use a flask brush to scrub the inside of the vessel.
8. Use warm tap water to rinse the soap out of the interior and off the outer surface of the glass vessel. Thoroughly rinse the interior and exterior surfaces of the glass vessel with deionized water.
9. The glass vessel can either be air dried on a drying rack or can be placed inside a drying oven and dried at 60° C for approximately 1 hour.
10. Remove the glass vessel from the drying oven and allow it to cool. Then seal all openings with tape to avoid contamination before next use.
11. Between sample sets, use a HEPA vacuum to clean the outer surface of the FBAS frame, including the pump, vibrator, time, and flow meters.

10.5 Collecting a Sample

1. Combine the appropriate amount of sample/sand, based on the test sample findings from Section 10.3, and place it inside a clean glass vessel that has been mounted on the segregator as described in Section 10.1.
2. Insert the isokinetic splitter stopper into the top opening of the glass vessel.
3. Remove the red plugs from the inlet and outlet of the filter cassette.
4. Attach a filter cassette to the top post of the isokinetic splitter.
5. Attach the PVC tubing from the bulkhead barb labeled "from sample filter" on the top of the segregator to the top outlet peg on top of the filter cassette.
6. Set valves to achieve the desired flow rate. There are two flow setting options. The low-flow setting accommodates collecting samples at a flow rate of approximately 200 cubic centimeters of air per minute (cc/min) onto a standard 25-mm MCE filter. The high-flow setting accommodates collecting samples using a cyclone attachment (Figure 7) placed in the sample train between the isokinetic splitter and a 37-mm MCE filter. Samples collected with the cyclone attachment are run at a flow rate of approximately 2 liters per minute (L/min). Before collecting a low-flow sample, ensure that the low-flow valve is fully open and the high-flow valve is fully closed. The adjustment knob on the bottom of the rotometer should be set accordingly to achieve a flow rate or 200 cc/min. Before collecting a high-flow sample, ensure that the high-flow valve is fully open and the low-flow valve is fully closed. The adjustment knob on the bottom of the high-flow rotometer should be set accordingly to achieve a flow rate of 2 L/min.

7. Set the digital timer for a minimum of three minutes.
8. Depress the "on" button on the digital timer, which will engage the vacuum pump and the vibration unit.
9. Monitor the flow and vacuum gauges and adjust as necessary to maintain settings. If the system is operating properly, readjustments will not be necessary. Record instrument readings in the notebook.
10. After the vacuum pump and vibrator unit are shut off, disconnect the filter cassette, remove the additional cowl, and replace the cap and end plugs of the sample cassette.

Figure 7 – Cyclone Attachment connected to the isokinetic splitter.

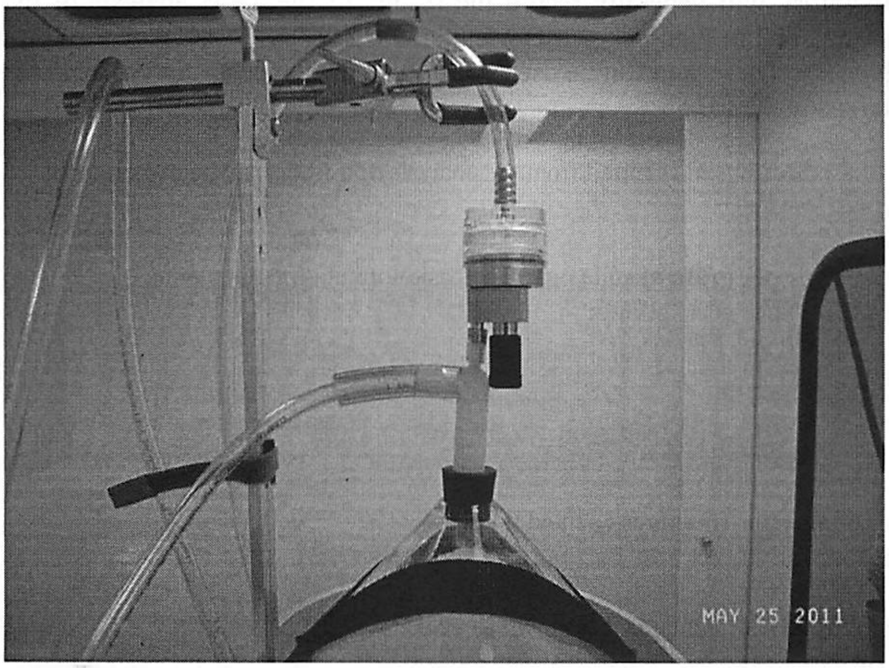

10.6 Post-Sampling Decontamination

1. Repeat steps in Section 10.4.
2. In addition, if mineral oil in the dust collector needs to be replaced (oil will appear dark with sediment settled on the bottom), empty the contents of into a rigid disposable container and dispose of it as asbestos waste material.

11.0 Record Keeping, Chain of Custody, and Shipping

Sampling records should be maintained in a logbook in the format equivalent to the example provided in Attachment 1. To correct errors in the logbook, make a one-line mark through the error and initial and date the change.

Use the FBAS Chain of Custody (COC) Form or equivalent COC form to record sample information and analysis required for the laboratory. An example of the form is included in Attachment 2. Containers used to transport/ship sample cassettes should be secured with a chain of custody seal, or equivalent. An example of the seal is included in Attachment 3.

Ship air sampling cassettes in a rigid container with cassettes in an upright position (filter facing upward) with the cowl attached. Use packing material to prevent jostling or damage during shipment. Do not use untreated polystyrene foam as packing because electrostatic forces may cause fiber loss from the filter.

The condition of samples and the custody seals should be noted on the COC form upon arrival at the laboratory.

12.0 Calculations and Reporting Results

The analytical laboratory will generally record the results of the TEM analysis of the filter in the format included in Figure 4 of ISO Method10312:1995(E) or Figure 5 of ISO Method 13794:1999(E). These data can be used to estimate the concentration of asbestos in the soil prepared using the FBAS.

The concentration of asbestos in soil reported from the analysis of a filter may be expressed in two alternate ways:

1. **Structures of asbestos per gram of soil (s/g).** The basic formula for calculating concentration in these units is as follows:

$$C_{soil}\ (s/g) = N \cdot S$$

where:

N = number of asbestos structures counted (s)
S = analytical sensitivity (g^{-1})

The analytical sensitivity (S) is calculated as follows:

$$S\ (g^{-1}) = EFA\ /\ (GOx \cdot Ago \cdot M \cdot Q_R)$$

where:

EFA = effective filter area (square millimeters [mm^2])
Q_R = flow ratio; this is the fraction of air passed through the soil sample (V_{total}) that is captured on the air filter (V_{filter}), and is calculated as:

$$Q_R = V_{filter}/V_{total}$$

GOx = Number of grid openings evaluated
Ago = Area of one grid opening (mm^2)
M = mass of asbestos-containing soil placed in the FBAS (g); does not include the mass of sand

2. **Mass percent (grams of asbestos per 100 grams of soil).** In order to express soil concentration as mass percent, the mass of each asbestos structure observed is estimated from its dimensions. In the absence of detailed data on the true geometry of each particle, the mass is approximated by assuming a simple rectangular solid shape, as follows:

$$m_i = l_i \cdot w_i^2 \cdot \delta \cdot 1E\text{-}12$$

where:

l_i = length of structure *i* (um)
w_i = width of structure *i* (um)
δ = density of asbestos (e.g., LA = 3.1 g/cm^3, chrysotile = 2.6 g/cm^3)
1E-12 = conversation factor (cm^3 per um^3)

The concentration, expressed as mass percent (grams of asbestos per 100 grams of soil), is then calculated as follows:

$$C_{soil}\,(\text{mass percent}) = \Sigma m_i \cdot S \cdot 100$$

13.0 QA/QC Evaluation

Accuracy, precision, and detection limit have not been completely evaluated for this SOP.

13.1 Negative Controls

Lot Blanks

A minimum of two lot blanks from each filter lot used will be analyzed to determine the mean asbestos structure loading. If the mean count of all types of asbestos structures is > 10 s/mm^2 or if the mean count of asbestos fibers and/or bundles longer than 5 µm is > 0.1 s/mm^2, the filter lot should be rejected. The analysis of blanks shall be performed in such a manner as to achieve an equivalent number of grids counted as to be comparable to those of the sample set.

Preparation Blanks

Preparation blanks shall be submitted for analysis at a frequency specified in a site/project specific QAPP and will be evaluated according to acceptance criteria specified in the QAPP. A preparation blank is a filter that is left uncovered on the bench top inside the FBAS hood during processing of soil samples with the FBAS. It is a measure of general laboratory cleanliness.

Sand Blanks

A sand blank shall be submitted for analysis at a frequency specified in a site/project specific QAPP and will be evaluated according to acceptance criteria specified in the QAPP. A sand blank consists of a filter generated from operating the FBAS as described in Section 10.5 with clean sand added to a clean glass vessel but without adding a soil sample.

13.2 Performance Testing Samples

Soil samples prepared as PT samples may be spiked with known concentrations of NIST SRM or equivalent. Samples should be collected in accordance with this SOP. Sampling conditions such as flow rate, sampling duration, sample size, etc. should be documented in the project notebook – per Section 5.0.

13.3 Calibration

The balance used to weigh samples should be checked daily with a set of metric test weights to verify accuracy. The flow meters on the FBAS should be checked with a rotometer calibrated to a primary standard at least once a week. Replace the inlet and

bypass HEPA filters whenever they are observed to be excessively discolored or the total flow diminishes appreciably. Always change the HEPA filters before total flow approaches 15 liters per minute; the total flow should not be allowed to fall below 15 liters per minute. Replace both check valves at the same time that the HEPA filters are changed. The check valves should also be replaced if the FBAS between sample preparation projects from different sites.

14.0 Pollution Prevention

Environmental factors, as defined by the EPA Region 10 Laboratory Environmental Policy Statement, will be considered in acquisition, use, and disposal decisions supporting asbestos analysis activities.

15.0 Waste Management

Waste materials potentially contaminated with asbestos, including used flow splitters, meshed stoppers, spent sample, spent sand, mineral oil from the dust collector, and HEPA filters, should be placed in double zip-top plastic bags and labeled with an asbestos warning labels. Asbestos waste material is disposed of at the Kitsap County Landfill per local regulations.

16.0 Change History

Revision 1.0 of this SOP includes corrected or clarified information. It also includes the following:

Section 10.5 – addition of instructions for use of a cyclone attachment and an image of the attachment in Figure 7.

Section 11 – replacement of the Region 10 Sample Custody and Analysis Required Form with a specific FBAS Chain of Custody Record.

Section 12 – includes revised calculations for results expressed as fibers per gram and mass percent.

EPA Region 10 - FBAS Sample Log Sheet **Page____ of ____**
OEAFIELDSOP-102 (Revision 1.0)

Project Name: ____

Technician(s):____

Sample Date: ____________________ Temperature: ________ Humidity: ________ Q_R: __________

Flow Rate: FI-1______l/m FI-2_______cc/m FI-3________l/m Balance Calibration: Pass____ Fail___

Sample Time	Sample ID	Sample Type Check One					Total Sample Weight (g)		FBAS Sample Weight (g)		Comments
		Field sample	Sand Blank	Lot Blank	Preparation Blank	FBAS Replicate	>850 µm fraction	<850 µm fraction	Soil	Sand	

EPA Region 10 - FBAS Sample Custody Seal
OEAFIELDSOP-102 (Revision 1.0)

UNITED STATES ENVIRONMENTAL PROTECTION AGENCY REGION 10

CUSTODY SEAL

Date:
Signature:

UNITED STATES ENVIRONMENTAL PROTECTION AGENCY REGION 10

EPA Region 10 - FBAS CHAIN OF CUSTODY RECORD
OEAFIELDSOP-102 (Revision 1.0)

COC No.: ____________________
[FBAS-YYMMDD###]

COC entered by (signature): ____________________ **COC Page** ______ **of** ______

Project Manager: ____________________ **Project Identifier:** ____________________

Method of Shipment: ______________ **Shipment Tracking No.:** ______________ **Ship Date:** ______________

Shipment Destination: ____________________ **Shipment Origin:** ____________________

Sample Information		Sample Preparation Request				Filter Analysis Request				FBAS Information			Sample Remarks
Sample ID (FE Standard ID or Replicate Filter ID)	Medium [S for soil; A for air filter]	OEAFIELDSOP-102 (Rev 1)*	with cyclone	without cyclone		TEM, ISO 10312:1995(E)**				Blank? [mark 'x' if yes]	FBAS Flow Ratio (Q_R)	FBAS Soil Mass (grams, dry weight)	

RELINQUISHED BY:		RELINQUISHED TO:	
NAME, AFFILIATION	DATE, TIME	NAME, AFFILIATION	DATE, TIME

COMMENTS/CONDITION OF SAMPLES:

* Prepare replicate filters using fluidized bed asbestos segregator in accordance with SOP.

** Specific recording and stopping rules are specified in a site/project specific QAPP. As needed, grids may be prepared indirectly in accordance with ISO 13794:1999(E).

APPENDIX C

SITE SAFETY PLAN

EPA Region 10 Site Safety Plan
Environmental Services Unit

1.0 General Information

1.1 Project Logistics and Responsibilities Include:

Site: Sumas Mountain Asbestos Site

Location: The Sumas Mountain Asbestos Site is located near Nooksack, Washington, along Swift Creek and the Sumas River.

Investigation Dates: September 29 – October 3, 2014

Plan Prepared By: ______________
Julie Wroble
Region 10 Toxicologist
Phone Number: (206) 553-1079
Cell Number: (206) 335-0971

Plan Reviewed By: ______________
Grady Maxwell
Site Safety Officer
Phone Number: (206) 553-0241
Cell Number: (206) 399-9394

Plan Approved By: ______________
Carol Haynes or Grady Maxwell
EPA Region 10 Laboratory Health and Safety Manager
Phone Number: (360) 871-8878 or (206) 553-0241

Field Team Members

Name	Office	Phone Number	Mobile Number
Jed Januch*	OEA	(360) 871-8731	(206) 465-8257
Doc Thompson*	OEA	(360) 871-8721	(206) 947-8638
Julie Wroble	OEA	(206) 553-1079	(206) 335-0971
Tim Frederick	EPA R4	(404) 562-8598	(404) 422-7026
Jennifer Crawford	OEA	(206) 553-6261	(253) 208-8322
Grady Maxwell*	OEA	(206) 553-0241	(206) 399-9394
Raymond Wu	OEA	(206) 553-1413	(678) 852-7925
Sandra Brozusky	OCE	(206) 553-5317	(360) 441-5470
Don Matheny*	OEA	(206) 553-2599	(425) 501-1978
Elly Hale*	ECL	(206) 553-1215	(206) 679-0935

*These individuals are not anticipated to need respirators during the field work because they are not conducting any actual sampling activities; however, they will be providing field support, such as getting bottles ready for sampling and handling sample documentation, including photographs and logbooks.

Note: Other agency personnel present on site will abide by their own applicable health and safety requirements. These individuals are welcome to attend EPA's health and safety briefings and will have access to the contents of this plan upon request.

1.2 Site Activities: Activity-Based Sampling
Soil Sampling

1.3 Project Description:

This project will involve activity-based sampling at several publicly-owned properties located near Swift Creek and the Sumas River in the vicinity of Nooksack, Washington. Asbestos is a known contaminant of concern at the Sumas Mountain Asbestos Site. The presence of asbestos has been confirmed at one of the two properties which are proposed for investigation during this sampling event. The activities for this project will include soil sampling, soil sample preparation, weed trimming, and raking.

2.0 Emergency Contacts

In case of any emergency,
1) an experienced and certified responder will apply necessary first aid, if applicable;
2) call 911 and/or other appropriate emergency numbers listed below;
3) once the situation is under control, notify the appropriate EPA Supervisor;
4) notify the EPA Region 10 Health and Safety Officer or his acting designee.

The field team must ensure they have the proper emergency information for the designated site before they start working on the project. Applicable emergency information will be presented at site safety briefings and is listed below.

-Police, Fire and Medical Aid –dial 911
This site does fall within the 911 response area. However, cell phone coverage for the site may not be reliable for all locations.

-EPA Health and Safety Staff: Grady Maxwell (206) 553-0241,
cell: (206) 399-9394
Carol Haines (360) 871-8878

-Occupational Health Physicians: Dr. Christopher Holland (301) 594-0272
Lekeeta Carr, PSC, LCDR (202) 564-3389

-Hospital Nearest Project Site

St. Joseph Hospital
2901 Squalicum Parkway
Bellingham, WA 98225
(360)734-5400

1. Start out going NORTH on OAT COLES RD toward S PASS RD.
2. Turn LEFT onto S PASS RD.
3. Turn LEFT onto NOOKSACK RD/WA-9. Continue to follow WA-9.
4. Turn RIGHT onto WA-542/MT BAKER HWY. Continue to follow WA-542.
5. WA-542 becomes E SUNSET DR.
6. Turn RIGHT onto ELLIS ST.
7. Turn LEFT onto SQUALICUM PKWY.
8. End at 2901 Squalicum Pkwy Bellingham, WA 98225-1851

Figure 2 – Hospital Driving Directions

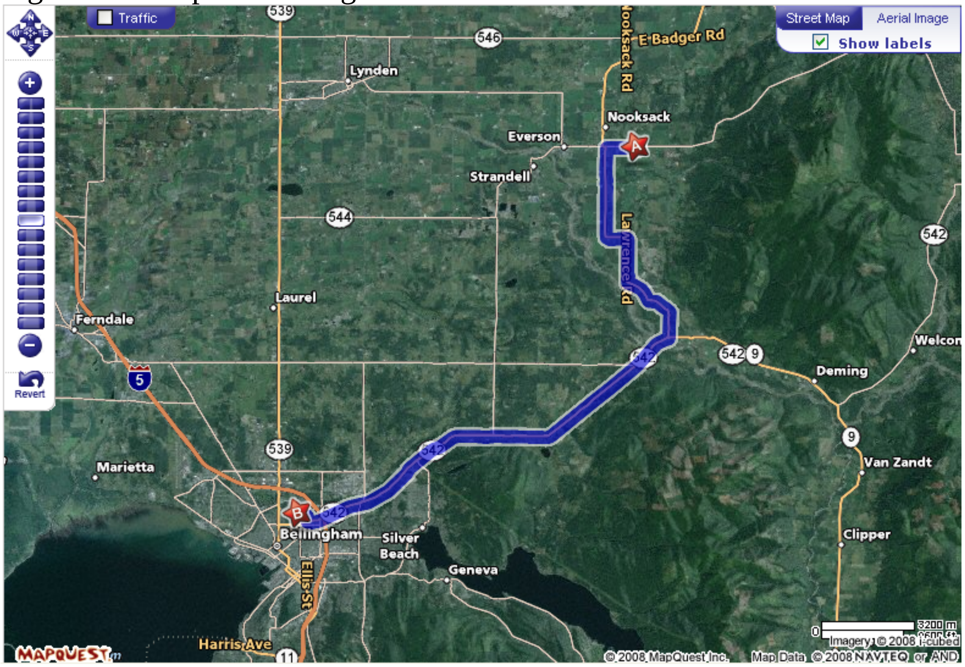

-Other Emergency Numbers:

Sumas Fire Department	(360) 988-9563
Whatcom County Fire District #1	(360) 966-5452
Everson/Deming/Nooksack	
Everson Police Department	(360) 966-4212

3.0 Site Hazards

Possible site hazards are identified for this site.

3.1 Hazards of Concern

Asbestos, primarily chrysotile, is the primary hazard of concern for this site. 29 CFR 1910.1001(c) defines the permissible 8-hour time-weighted average limit (TWA) to be an airborne concentration of no more than 0.1 fibers per cubic centimeter of air (f/cc). A phase contrast microscopy equivalent (PCME) asbestos fiber is a structure greater than 5 micrometers (µm) in length with aspect ratio (length to width) of 3:1. With regard to the excursion limit, no field team member shall be exposed in excess of 1.0 f/cc as averaged over a sampling period of 30 minutes.

Asbestos is a known human carcinogen; however no level immediately dangerous to life and health (IDLH) has yet been determined. The primary exposure route for asbestos is through inhalation. Other potentially minor exposure routes include ingestion and skin and/or eye contact.

3.2 Physical and Other Environmental Hazards

Hazards may include moving vehicles, tripping in holes or rocks, temperature concerns with use of PPE (heat exhaustion or stroke), sunburn and dusty conditions. Wildlife hazards include but are not limited to rodents and insects (bees, spiders, ticks).

3.3 Prevention and Protection

The field team will walk through the site or the sampling area to be addressed that day to determine the layout and potential hazards. Each day before conducting the sampling, the field team will attend a safety briefing to identify and discuss hazards and hazard controls.

Use of personal protective equipment (PPE) during sampling operations may result in heat-related injury. The field team will take frequent breaks in a shaded location in fresh air. EPA will provide bottled drinking water and/or sports drinks, sun block lotion, and cooling vests. The field team will not work in PPE for a period exceeding 1 hour without a break when temperatures exceed 75 degrees Fahrenheit (^{o}F).

At least one observer will be present and in direct eye contact of individuals performing the activity-based sampling within the hot zone. Individuals leaving the hot zone will be decontaminated with clean rinse water from either a pressurized water tank or garden sprayers.

3.4 Hazardous Duty

The field team participating in the activity-based sampling or working a supporting role and entering the hot zone(s) will meet the requirements specified in Title 5, CFR, Part 550, Subpart I, Appendix A, Exposure to Hazardous Agents, working with or in close proximity to Asbestos. These workers will be eligible for a hazard pay differential of 8% based on the requirements of EPA order 3100.3A. These workers will complete the REQUEST FOR APPROVAL OF A HAZARD PAY DIFFERENTIAL (AND INCORPORATED EMPLOYEE EXPOSURE RECORD) Form included in Attachment 1 and submit it for approval by their manager, the EPA Region 10 Health and Safety Officer and the Human Resources Office.

4.0 Personal Protective Equipment

The following personal protective equipment will be used during this project.

-Leather Safety Boots (steel toe and shank),
-Gloves (Nitrile),
-Tyvek Suit (with hood),
-Full Face Respirator (with P-100 HEPA cartridges),
- Level of Protection (C) listed in the ABS Plan

The following safety equipment will be available on site:

-First Aid Kit (restocked September 2014),
-Portable Eye Wash,
-Fire Extinguisher,
-Air Horn,
-Portable Phone – at least two cell phones.

5.0 Training

All field team members that participate in sampling activities will have satisfied all EPA and OSHA training requirements for the work being performed. The field team members have had 40-hour Health and Safety Training and will be current on the annual refresher (8-hr) requirements, respirator fit-testing, first aid and CPR. In addition, all field team personnel will

-review the site safety plan prior to beginning field work and
-attend site-specific safety briefings.

6.0 Medical Surveillance Program

EPA personnel performing sampling activities on this site must be enrolled and current in the Occupational Medical Surveillance Program.

7.0 Monitoring Program

Air monitoring, as described in section 3.1, will be performed during the activity-based sampling. The site-specific monitoring program will consist of collecting personal air samples. Sampling pumps will be calibrated pre and post sampling. Air sampling filter cartridges will be 0.8 µm MCE type. Air samples will be analyzed by transmission electron microscopy (TEM) method ISO 10312. Additional information

on the air monitoring program is included in the site specific sampling and analysis plan and the quality assurance project.

8.0 Site Control Measures

8.1 Work Zones will be established including a control zone, decontamination zone, and an exclusion zone. These zones will be demarcated with yellow tape to ensure adequate site control.

8.2 Site Map – see Figure 1.

8.3 Communications will be maintained with cell phones (if cell coverage is available) and an air horn.

9.0 Decontamination

Field team members leaving the hot zone will be decontaminated with clean rinse water from either a pressurized water tank or garden sprayers. The field team will dispose of used PPE (Tyvek, gloves and used respirator cartridges) in asbestos debris bags. Respirators will be wiped down with damp towels after each use and washed with soap and water after each daily use. Rinse water will be disposed of at the site. Waste PPE will be bagged and removed from the site for proper disposal. All disposable of used PPE will be the responsibility of the Field Team Leader.

REQUEST FOR APPROVAL OF A HAZARD PAY DIFFERENTIAL (AND INCORPORATED EMPLOYEE EXPOSURE RECORD)

Date:______________________________

I. EMPLOYEE

Name: ________________________________

Title & Grade: __________________________ **Work Phone:** ______________

Organization:____

II. SITE OR LOCATION OF HAZARDOUS DUTY PERFORMED

____ **Site ID Number:** __________

III. APPLICABLE HAZARDOUS CONDITIONS (CHECK ALL THAT APPLY)

[ ] Hazardous Weather [ ] Hazardous Terrain [ ] Extreme Temperature
[ ] Explosive Materials [ ] Incendiary Materials [ ] Toxic Chemicals
[ ] Virulent Biologicals [ ] Asbestos [ ] Fire
[ ] Underwater Duty [ ] Sea Duty–Sampling [ ] Limited Control Flight
[ ] Height Work [ ] Unsafe Structure Work [ ] Fuel Storage Tank Inspect
[ ] Other (please explain)

____

IV. LEVEL OF PROTECTION (A, B, C, D etc.)

Level A:

Level B: Suit: [ ] Saran [ ] Tyvek [ ] Acid/Rain [ ]
Other
Gloves: Boots:

Level C:

Canister Type/#:______________; Cartridge Type/#:_______________________;
APR:_______________________

Level D—(Justify):

V. TYPE OF AIR MONITORING

[ ] Radiation [ ] O2/LEL [ ] PID/FID [ ] RAM [ ] Draeger
[ ] Personal [ ] Area [ ]Other:______________________________________

VI. DESCRIPTION OF ACTIVITY AND JUSTIFICATION FOR REQUEST

____
____
____
____
____
____

(supervisor: attach employee comments, if any, and any relevant sampling and monitoring data):
Applicable 5 Code of Federal Regulation 550, Subpart I, Appendix A Citation:

VII. HOURS IN PAY STATUS DURING DAY (INCLUDING CONTINUATION OF TOUR IN SUCCEEDING CALENDAR DAY) IN WHICH THE HAZARDOUS DUTY WAS WORKED OR THE PHYSICAL HARDSHIP WAS EXPERIENCED (A separate form must be used for each pay period in which hazardous duty was performed or physical hardship was experienced—do not enter hours for days for which a HPD is not claimed.)
Pay Period:_____________________, ending _____________________________
(enter number of hours in pay status for each day hazard pay differential is warranted).

Su	M	T	W	Th	F	Sa	Su	M	T	W	Th	F	Sa
Date:													
Hours in Pay:													
Status (incl. regularly scheduled tour of duty, overtime and paid leave)													

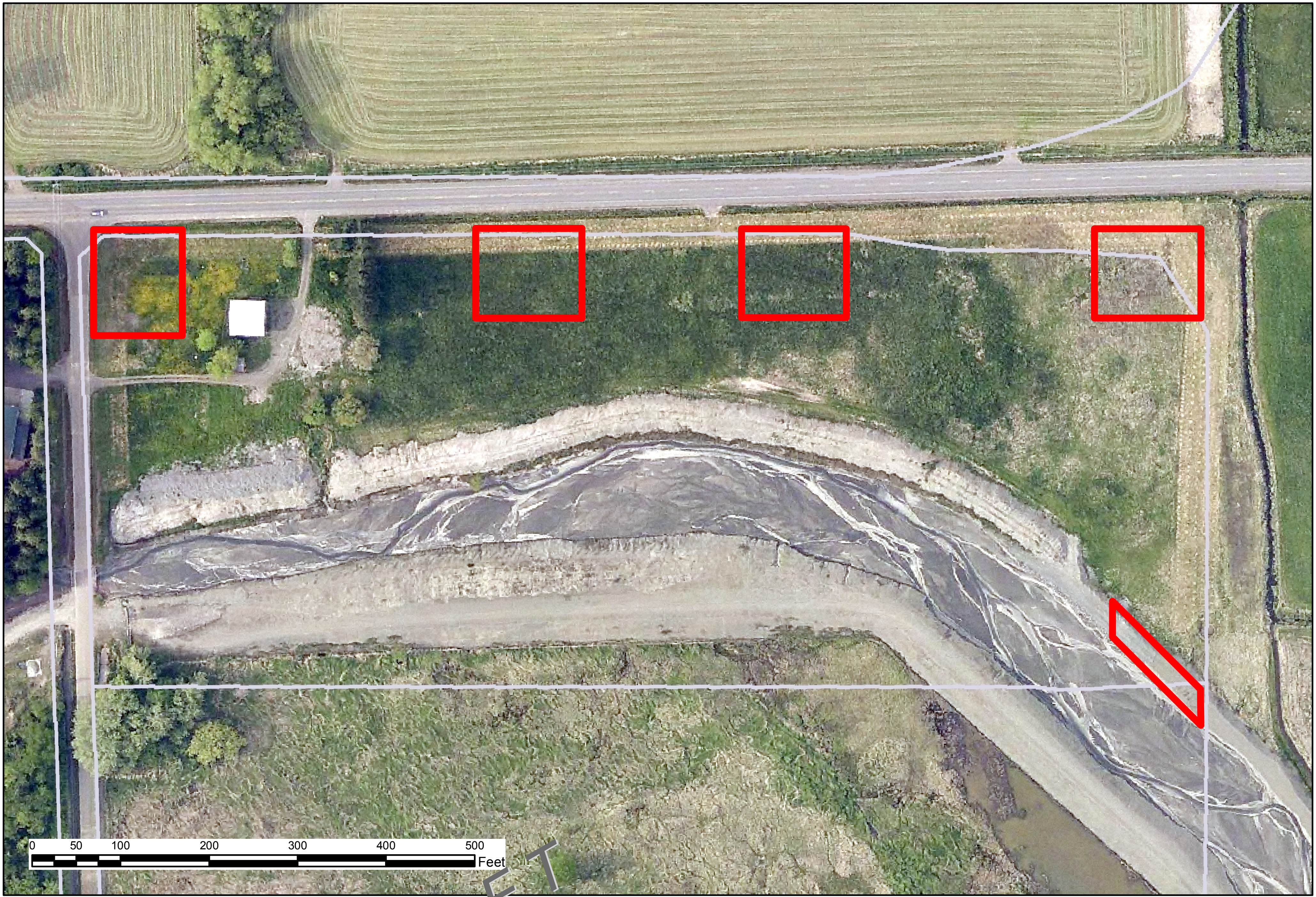

N

DRAFT

Figure 1
Proposed Sampling Locations,
Oat Coles/South Pass Road
Sumas Mountain Asbestos Site

EPA Region 10 GIS Team

Image Data from Whatcom County Administrative Services
Image Date 2013, pixel resolutinon 9"
Map Created 09/25/2014

APPENDIX D

USER’S MANUALS FOR EQUIPMENT

WEATHERPAK® -2000

User Manual

November 1, 1999

COASTAL ENVIRONMENTAL SYSTEMS

820 First Avenue South • Seattle, WA 98134
(206) 682-6048 (800) 488-8291 Fax: (206) 682-5658

www.coastalenvironmental.com

TABLE OF CONTENTS

IMPORTANT NOTES 1
INSTALLATION 2
Erecting a Tower 2
Quick Release Mount 2
Cables and Connectors 2
Compass 2
COMMUNICATION SETUP 3
Introduction 3
Connecting the Terminal 3
Powering up the WEATHERPAK 3
Automatic Bootup Sequence 3
WEATHERPAK®-2000 USER MODE 4
Introduction 4
Changing and saving settings 4
Help facilities 4
Communications settings 4
Changing System settings 5
Change Item n to Value m 5
System date and time 6
Internal Temperature Calibration 6
Other commands 6
Changing the data collection schedule 7
Inspecting Raw Data 8
Exiting the User Interface Mode. 8
GLOSSARY 10
MATHEMATICS OF WIND MEASUREMENTS 11
WEATHERPAK-2000 for HAZMAT RESPONSE 13
Installation 13
Tripod Mount Setup 16
Van Mount Setup 17
Receiver Display Box 18
Low Power Indicators 19
Plume Modeling Software 20
TROUBLESHOOTING 23
MAINTENANCE 24
SYSTEM DESCRIPTION, SCHEMATICS and CONFIGURATION 24

IMPORTANT NOTES

DO CALL/FAX US IF YOU HAVE QUESTIONS OR PROBLEMS
Call Coastal Environmental Systems if any troubles arise or if you have any questions pertaining to the equipment. Our telephone number is 206-682-6048 and the FAX number is 206-682-5658. Refer to the model number (WEATHERPAK® -2000) and the serial number of the WEATHERPAK® which can be found by looking under the radiation shield on top of the unit.

DO WASH OFF CHEMICALS OR DEBRIS WHILE THE WEATHERPAK® IS ASSEMBLED to prevent getting water in the connectors. The WEATHERPAK® is water-resistant when assembled.

DO FOLLOW THE MAINTENANCE SCHEDULE.

INCORRECT WIND SPEED WILL RESULT IF THE PROPELLER NUT IS NOT TIGHT.

DO NOT OPEN THE WEATHERPAK®. It is purged with desiccant bags to seal out all the moisture. Opening it may allow moisture to foul the electronics and will invalidate the warranty.

DO NOT PICK UP THE WEATHERPAK® BY THE WIND VANE. This is a durable but sensitive wind sensor and it can be broken if it is used to lift the entire unit.

DO NOT REMOVE THE ANTENNA FROM THE WEATHERPAK®. If the WEATHERPAK® is activated without the antenna, the radio can be damaged.

The WEATHERPAK is designed to be completely automatic. When power is applied, if no terminal intervention occurs, the system will automatically initialize itself and begin sampling according to its predetermined schedule. The WEATHERPAK has a hardware clock accurate to about 5 seconds per day and an EEPROM portion of memory where sampling variables and system parameters are stored. Data are time marked with date/time.

All transient protection sensor signals and power lines are protected from transients and EMI by circuitry inside the WEATHERPAK. EMI filters are single pole RC filters (100 ohm and 0.1 microfarads). Transient protection is by 18 volt tranzorb diodes. If for any reason -- lightning, RF interference, power surge -- the computer program is corrupted and the sampling loop is broken, then a watchdog timer will reset the entire system and the WEATHERPAK will go into its autoboot routine.

DEPENDING ON YOUR CONFIGURATION THIS USER MANUAL CONTAINS INFORMATION THAT MAY NOT APPLY TO YOUR SPECIFIC WEATHERPAK.

INSTALLATION

This section will offer generalized advice on the siting and installation of a WEATHERPAK and its associated sensors. All installations are unique with their own set of problems, so care and attention are required. Coastal Environmental Systems will be happy to provide special advice for your application.

Select an exposed site, removed from obstructions and more than 3 meters above the ground or the prevailing terrain. The wind pattern around a building or other obstacle is highly disturbed for a considerable distance, so the site should be as. A rule of thumb is that the sensors should be placed at a height 1½ times the nearby obstacles or that they are placed at a distance away of ten times the height.

Erecting a Tower

The WEATHERPAK mounts on the quick release mount which is threaded on the bottom with a 1½ inch NPT male pipe thread. The quick release mount must be securely mounted to withstand wind loading and vibration at the installation site. Standard 1½ inch NPT pipe fittings can be used. We recommend that fittings be assembled with an anti-seizing compound to facilitate disassembly. If you use a free standing tower we recommend that you also use guy wires to support the WEATHERPAK in high winds. The guy wires attach just below the quick release mount.

Quick Release Mount

The quick release mount can be threaded into the tower and fully secured before the tower is erected. ***Keep the protective PVC cap in the quick release whenever the WEATHERPAK is not connected.*** This prevents sand or water from getting onto electrical contacts. Keep the cap in a safe place and always replace it if the WEATHERPAK is removed for maintenance. When removing the PVC cap, be sure the quick release is clean and dry. During the removal, any water or dirt that has collected on top of the cap can fall into the mount.

Cables and Connectors

Problems with cables and connectors cause at least 40% of all system failures. Our recommendation is "do it right the first time." Take special care of external cables and connectors. Air pressure differences from wind or differential heating of instruments can cause cables to vent air into sensors and connectors; wind can force sand or moisture into the most insignificant interstices; and solar radiation is destructive to plastics and paint.
Below is a list of considerations:

1. ***Protect external connectors.*** Molded neoprene oceanographic connectors are probably the only connectors that do not need extra protection. Any other types, including MS-style environmental connectors, must be protected. We recommend that you wrap them in self-vulcanizing rubber tape followed by an outer wrap of black electrical tape (environmental grade). Use only the highest quality wrapping tape -- tape is cheap insurance.
2. ***Consider conduit.*** Conduit is good insurance. It protects cables from sunlight and abrasion. Animals love to chew on cables. Human beings may also harm cables.
3. ***Dorn fittings.*** Dorn fittings (also called compression fittings) are cable pass-through fittings, which use a rubber stopper to squeeze the cable. The Dorn fitting should be the proper size to snugly squeeze the cable -- when it is tight, you cannot pull the cable through the fitting. A small bit of silicone grease on the cable in the stopper is added protection. Dorn fittings are an excellent means of getting watertight security at a reasonable price, but they are not perfect. As we said earlier, cables "breathe", so a Dorn fitting is no more than a straw into your electronic enclosure unless care is taken to block the cable. Alternatively, breather holes are often provided to allow the pressure difference to equilibrate.
4. ***Oceanographic connectors.*** For the best and most reliable system, use molded oceanographic connectors. With these expensive connectors, you will have perfect seals for indefinite periods.

Compass

Observe the following cautions:

- Make sure there is no disturbing metal or other magnetic material around.
- Re-check the site routinely for new magnetic contamination. On a buoy, ice, or other remote site this is rare, but at an industrial or urban site you can expect problems.

In a semi-permanent or permanent fixed site, we advise that you do not use the compass, but instead survey in the WEATHERPAK to the proper alignment. In doing so, bear in mind that the alignment pin on the quick release and the alignment box on the wind sensor are at ***relative South.***

COMMUNICATION SETUP

Introduction

The WEATHERPAK has a set of menus which are used to change the data collection schedule, upload logged data, display and change communications parameters, and display and change system parameters and values. You can reach the menus connecting a terminal (test set) directly to the user interface communications port on the front of the WEATHERPAK; any terminal or computer with RS232 input will do.

A PC or AT running a terminal program such as CROSSTALK or MIRROR is ideal. You can also use the Microsoft WINDOWS® terminal program to communicate with the WEATHERPAK; or even a dumb terminal with an RS-232 serial port. Coastal Environmental Systems can provide terminal software for this task.

Most terminal emulation programs have a "capture" mode that lets you save the session on a disk file. This practice is highly recommended as it provides a reference if any questions about operation or setup arise.

Connecting the Terminal

To communicate with the WEATHERPAK, your terminal must be connected to the user interface communications port on the front of the WEATHERPAK.

Set the terminal as follows:

- Baud rate = 9600 (you can change and save this) (Radio Base Stations use 1200 Baud)
- Start bits = 1
- Stop bits = 1
- Data bits = 8
- Parity = none

Powering up the WEATHERPAK

Power is supplied via the umbilical cable. The system requires 11-18 VDC. The mean current drain is 20 mA and the peak current required is 80 mA.

Automatic Bootup Sequence

The WEATHERPAK commences operation when power is applied to it. The program commences by setting all default parameters. The parameter EEPROM memory is read into RAM. This memory contains parameters such as sampling rates, calibration coefficients, and state flags.

If a terminal is connected while the power is applied you will see something similar to the following message after about 5 seconds:

```
Watchdog Reset
Zeno 3200 V1.50 Nov 27 1994 14:36:30
Copyright (C) Coastal Environmental Systems, 1994.
System Time = 94/11/28 12:26:50
Initializing (approximately 8 seconds) . . ./
Sampling . . . Type 'U' To Enter The User Menus.
```

WEATHERPAK®-2000 USER MODE

Introduction

The WEATHERPAK®-2000 User Mode lets you:

- change communications settings;
- change system settings such as the clock time;
- change the data collection schedule;
- upload logged data.

Type U to enter the User Menu. The WEATHERPAK will display the following menu structure.

```
USER MENU
(C) Communications Menu          (T) Test Menu
(F) System Functions Menu        (Z) Zeno Program Menus
(S) Sample Period Menu           (H) Help
(D) Data Retrieval Menu          (Q) Quit
```

Changing and saving settings

The configuration of your WEATHERPAK is held in EEPROM. This means that the settings are retained, even when the system powered down. When you power up the WEATHERPAK, the settings are read from EEPROM into RAM; the WEATHERPAK operates according to the configuration in its RAM.

When you change any settings in the User Mode, the new configuration is held in RAM, and takes effect immediately. However, if the system is powered down, the settings in RAM will be lost -- the old configuration will take effect the next time the system boots. To save settings to EEPROM -- making them permanent until the next change -- you need to enter E from any of the sub-menus.

Type E within any sub-menu to save all settings to EEPROM.
You will lose any unsaved settings when the system powers down.

The WEATHERPAK continues data collection while you are in the User Mode. If there is no terminal activity for approximately three minutes, the WEATHERPAK will exit the User Mode. You should save any settings you wish to keep to EEPROM before quitting the User Mode -- you will lose any unsaved settings when the system powers down.

Help facilities

Context-sensitive on-line help is available through two commands. By entering Hn, where n is a number, you obtain help on line item n. For example, in the System Functions Menu, H5 gives you help on line item 5 -- Add Compass To Vane. Remember to type <RETURN> or <ENTER> after any command.

By entering HX, where X is a command, you obtain help on that command. For example, entering HQ in the User Menu gives you information on the Quit option.

Communications settings

The communications parameters for your WEATHERPAK have been factory set. We do not recommend that they be changed. If you do wish to alter these settings (through the Communications Menu), you should be aware that the WEATHERPAK COM3 port is the one connected to your terminal.

Changing System settings

The System Functions Menu covers a number of miscellaneous functions, such as:

- setting the system clock;
- calibrating the internal thermistor;
- finding your software version number;
- adding the compass reading to the measured direction when doing a wind vector average.

Type F, followed by <RETURN> or <ENTER>, from the main menu to enter the system FUNCTIONS menu. The WEATHERPAK user interface is not case sensitive – f will also work. Once you are within the user interface, you must type <Enter> or <Return> at every command

```
SYSTEM FUNCTIONS MENU
(Cn/m) Change Item n to Value m    (E) Save Parameters To EEPROM
(D)    Display System Clock        (U) User Menu
(S)    Set System Clock            (Q) Quit
(V)    Program Version             (H) Help
(T)    Calibrate Internal Temperature

Item 1: 0   (Primary Unit/Experiment ID, 0 to 9999)
Item 2: 0   (Secondary Unit/Experiment ID, 0 to 9999)
Item 3: 1   (Data Dump Format)
Item 4: 1   (Real Time Output Format)
Item 5: 0   (Add Compass to Vane, 0 = NO, 1 = YES)
Item 6: 0   (Compass Offset in degrees, -180 to 180)
Item 7: 0   (Barometer Elevation in meters, 0 to 5000)
```

Change Item n to Value m

Enter Cn/m to **CHANGE** the value of line item n to value m. For example, type **C1/3**, followed by **<RETURN>** or **<ENTER>**, to set the primary unit/experiment ID number to 3. If you wish to make this change permanent, you should then enter E to save the setting to EEPROM.

The different line items have the following meaning.
Primary Unit/Experiment ID: This item affects the real time output messages output by the WEATHERPAK during data collection. You may have seen these messages on the terminal before entering the User Menu. The values of this unit ID number, and unit ID number 2 are attached to these messages, to let you identify the source of the message. For example, they let you identify a single WEATHERPAK in a multi-site system; or they can be used to define the current system configuration.
Secondary Unit/Experiment ID: This is a second unit identifier, used together with the primary unit/experiment ID.
Data dump format: The format of the data messages you receive from the WEATHERPAK at the terminal when using the Data Retrieval menu. The formats are defined in the table overleaf.
Real time output format: This item affects the messages transmitted by the WEATHERPAK during normal data collection.
Add compass to vane: Controls whether or not the WEATHERPAK adds the compass value to the measured wind direction (the vane) when doing a wind vector average. This is described in more detail in Appendix 0.
Compass offset: This value is added to the compass measurement, to compensate for local variations in magnetic North. If you have no compass, this option has no effect. More details are given in Appendix 0.
Barometer elevation: The elevation above sea level. This quantity is used to correct the barometric pressure reading to sea level.

System settings

Line	Quantity	Values available
1	Primary Unit/Experiment ID	0 to 9999
2	Secondary Unit/Experiment ID	0 to 9999
3	Data dump format	1 Space delimited<br>2 Comma delimited<br>3 Match the real time output format
4	Real time output format	0 None (output message suppressed)<br>1 ASCII characters, width dependent on data values, comma separated<br>2 ASCII characters, width as factory configured, no automatic separators
5	Add compass to vane	0 No<br>1 Yes
6	Compass offset (degrees)	-180 to +180
7	Barometer elevation (meters)	0 to 5000

System date and time

Options D and S respectively let you DISPLAY and SET the current date (format YY/MM/DD) and time (format HH:MM:SS) on the system clock to the nearest second. If you set the clock, the new setting is saved immediately: you do not have to type E to save the new setting, since it is not held in EEPROM. Be sure to use the "/" format for dates, and ":" for time.

Internal Temperature Calibration

Option T lets you calibrate the internal Temperature. You will be prompted to enter the current ambient air temperature -- measured with an accurate temperature probe placed near the WEATHERPAK electronics. If you move the WEATHERPAK to the probe, rather than the probe to the WEATHERPAK, you must give the internal thermistor time to equilibrate at the new temperature -- 15 minutes is usually adequate for this. The temperature can be specified in units of FAHRENHEIT (example input: 72.5F), CENTIGRADE (23.2C), or KELVIN (295.6K). If you recalibrate the thermistor, the new calibration is saved immediately to EEPROM: you do not have to type E to save the new calibration.

Changes to the system clock and internal thermistor are saved immediately -- without typing E

Other commands

Option V gives you the current software VERSION number.

Please note the software version before calling Coastal's customer service

Option E, as already described, saves changes to EEPROM.
Option U returns you to the USER Menu.
Option Q lets you QUIT directly from the current menu. Any changes made will remain in RAM, but will not be saved to EEPROM. You can return to the previous configuration by powering down and then re-powering your WEATHERPAK.

Changing the data collection schedule

A wide range of sensor sampling schemes are available from the Weatherpak. Examples are:

A. the sensors can be sampled continuously, with no gaps;

B. the Weatherpak can take samples from ten past until half past each hour.

The following time line illustrates these schemes -- a period of 3 hours is shown. The light grey areas indicate that sensors are being sampled.

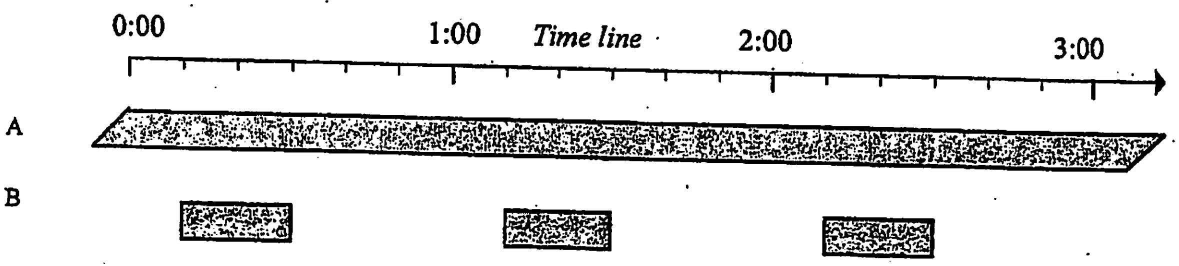

The Weatherpak uses the same data collection schedule for all of the sensors. For the two cases listed above, you would set up the following schemes within the Sample Period Menu:

A. start a sampling period each hour, minute or second, and let that period last one hour, minute or second;

B. start a sampling period each hour, at 10 minutes past the hour, and let that period last 20 minutes.

Enter S from the User Menu to enter the **SAMPLE** Period Menu.

```
SAMPLE PERIOD MENU
(Cn/m)  Change Item n To Value m            (Q) Quit
(E)     Save Settings To EEPROM             (H) Help
(U)     User Menu

Item  1:  3600  (Sample Interval Time in seconds)
Item  2:  1200  (Sample Duration Time in seconds)
Item  3:  600   (Sample Time Offset in seconds)
```

The three variables are as follows:

- the sample interval time is the time elapsing between the start of successive sampling periods (i.e., one hour or 3600 seconds in case B above);
- the sample duration time is the time for which the sampling actually takes place within each sample interval (i.e., 20 minutes or 1200 seconds in case B above);
- the sample time offset defines the time at which the sampling begins, relative to a round number of sampling periods -- 10 minutes in case B above.

Sample period settings

Line	Quantity	Values available
1	Sample interval T_{Int} (seconds)	1 to 86400 (24 hours)
2	Sample duration time T_{dur} (seconds)	1 to T_{Int}
3	Sample time offset T_{off}	0 to T_{Int} - T_{dur}

The following time line illustrates the three variables, with case B in mind. Sampling takes place within the time periods marked gray.

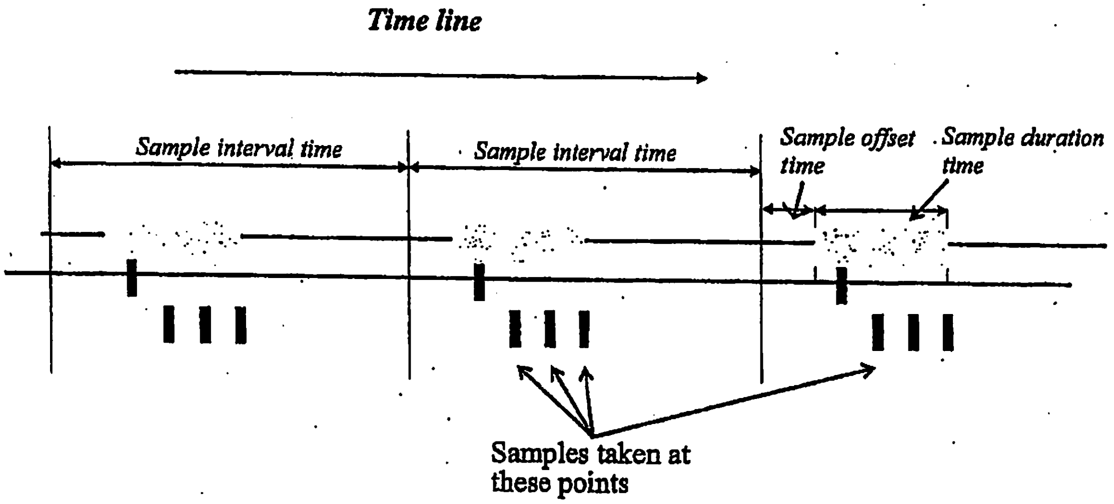

The sample interval always starts, as far as possible, at an even time -- for example, if the sample interval time is one hour then the sample intervals start on the hour. The sensor outputs are sampled throughout the sample duration time.

A wide range of sensor sampling schemes are available from the WEATHERPAK. Examples are: the sensors can be sampled continuously, with no gaps; the WEATHERPAK can take samples from ten past until half past each hour.

Inspecting Raw Data

The Test Menu lets you inspect raw data as it is measured -- without any averaging or other processing. The data can be inspected either before or after conversion to engineering units. Enter `T` from the user menu to reach the `TEST` Menu.

```
TEST MENU
(R) Display Raw Sensor Data         (H) Help
(S) Display Scaled Sensor Data      (U) User Menu
(Q) Quit
```

You cannot change anything from the Test Menu.

R Display **RAW** sensor data. The terminal will display, as it is measured, raw data from each sensor. The values are displayed before conversion to engineering units -- for example, volts for analog sensor inputs.

S Display **SCALED** sensor data. The terminal will display, as it is measured, scaled data from each sensor -- in engineering units.

Exiting the User Interface Mode.

The WEATHERPAK continues to collect data while you are in the User Interface mode. If there is no terminal activity for approximately five minutes, the WEATHERPAK will time-out, exit the User Interface mode, and resume displaying the data sample lines. To manually exit the User Interface Mode, select `Q` to `QUIT` from any menu.

Uploading logged data from the WEATHERPAK

The Data Retrieval Menu lets you upload and inspect data being collected by the WEATHERPAK. Type D from the user menu to enter the DATA retrieval menu. The WEATHERPAK stores sensor data as a set of data records: each data record consists of a time stamp and one or more processed sensor data values. You may have seen the processed sensor data values being output to the terminal before entering the User Menu.

```
DATA RETRIEVAL MENU
(A)  Show Records AFTER Specified Time   (C) Compute Data Logging Capacity
(B)  Show Records BETWEEN TimeSpan       (D) Delete All Data Records
(Ln) Show Last n Records                 (N) Show Number of Records Logged
(*)  Show All Data Records               (U) User Menu
(@n) Show n Unmarked Records             (Q) Quit
(M)  Mark Recently Shown Data            (H) Help
```

You cannot change anything from the data retrieval menu. The following data retrieval commands are available.

A Show records AFTER specified time. This option lets you examine a fixed number of records, starting at a given time. For example, you may wish to examine the next 3 records logged after 3 p.m. You will be prompted to enter the start time, and the number of records to be displayed. Be sure to enter the start and end times in the format "YY/MM/DD HH:MM:SS"; for example, 94/11/28 14:45:00. Hit the space bar to halt the display; you will be prompted for confirmation.

B Show all records BETWEEN timespan. This option lets you examine all records logged between a defined start and end time -- you will be prompted for the start and end time if you make this choice. Be sure to enter the start and end times in the format "YY/MM/DD HH:MM:SS"; for example, 94/11/28 14:45:00. Hit the space bar to halt the display; you will be prompted for confirmation.

C Compute data logging CAPACITY. The terminal will show the total memory available for data storage, the total number of data records that can be stored, and the total time taken to fill the data logging memory.

N Show NUMBER of records logged. The terminal will show the number of data records that have been collected and stored in memory. This value must be less than or equal to the data logging capacity.

D DELETE all data records from the data logging memory. You will be prompted for confirmation.

Ln Show the LAST n data records. For each data record, the terminal will show the date and time stamp, followed by the logged, processed, sensor data values. The display will look something like this (assuming relative humidity and air temperature are being measured). The detailed format of the display (e.g., whether commas or spaces separate the data values) can be changed from the System Functions menu (choose S from the user menu). Hit the space bar to halt the display; you will be prompted for confirmation.

```
> L3
Hit The Space Bar To Halt The Log Data Output.
 DATE     TIME    RH  AT
94/04/15 4:43:38 86.4 78.6
94/04/15 4:43:40 86.4 78.6
94/04/15 4:43:42 86.4 78.6
```

@n Show n unmarked records. The data retrieval menu lets you "mark" logged records as already having been read. The first time you enter @n , the WEATHERPAK will display the oldest n records. If you subsequently enter M, these records will be marked as read. You will no longer be able to access these records using the @n command, although they will be accessible using the L (show last records), J (show records between specified times), and K (show records after a specified time) commands. The next time you enter @n , the WEATHERPAK will display the oldest unmarked n records.

M MARK recently shown data. This command marks as read the last set of records displayed using the @n command. This command will work irrespective of when the last @n command was used -- even if you have left and re-entered the user interface in the mean time.

* Show all data records. You will see all the records logged to date sequentially, in the same format as the above. Hit the space bar to halt the display; you will be prompted for confirmation.

GLOSSARY

ALOHA: Aerial Location of Hazardous Atmospheres

ASCII: American Standard Code for Information Interchange.

Autoboot; Automatic bootup.

Base Mount: A shortened name for the "quick-release base mount", the device which holds the WEATHERPAK and provides electrical interface.

Boot or Bootup: When a computer initializes itself and automatically starts operation of a program when the power is turned on or after a warm reset.

Byte: A group of eight bits of computer information.

CAMEO: Computer Aided Management of Emergency Operations

Declination: See Variation.

Dorn Fittings: Compression fittings that allow an electronic cable to penetrate a casing wall and maintain a hermetic seal.

EEPROM: Electronically Erasable Programmable Read-Only Memory— allow programmable setting of system parameters that is preserved when all power is removed.

Magnetic declination: See Variation.

Magnetic variation: See Variation.

RAM: Random Access Memory -- memory for data storage and program operation. RAM can be written to and read by addressing from the microprocessor.

RC Filter: An analog low-pass filter made of resistors and capacitors.

Resolution: The smallest amount of change in a measured quantity that can be discerned in a measurement. Unless stated otherwise, resolution implies the "exported resolution" which is the least resolvable change in the final datum. As an example, temperature might be reported to 0.1□C but the actual measurement and internal processing might maintain 0.01□C resolution. In this case, the resolution would be 0.1□C. (sometimes written ±0.1□C).

RFI: Radio Frequency Interference -- any noise that enters into a system from external sources.

RS232: The most common computer serial interface standard.

Tranzorb: A special diode semiconductor device that will acts as a short to high-voltage transients. It is installed on electrical input or output connections to protect a device from corona or electrical transients such as lightning.

Variation: Also magnetic variation and deviation. At most points on the Earth, the direction to true north varies from the direction to magnetic north by some number of degrees. This difference is called the **magnetic declination**. When the local magnetic anomalies are added to the declination, the result is called the **magnetic variation** or simply the **variation**. Either term, declination or variation, is correct although surveyors generally prefer the term declination and navigators the term variation.

Aeronautical and marine charts give variation as isogonic lines, or lines of constant magnetic variation. Variation changes with time, and the charts usually give the rate of change. In some areas, such as the Arctic, the charts note that compass measurements are unreliable. For the highest accuracy, it is important to use up-to-date charts. When a compass needle points east of true north it is due to **easterly variation**. This is the case for most of the continental U.S.A. The correction from magnetic to true direction for an easterly variation is a positive correction. As an example, in Seattle WA the variation is approximately 22° and a compass reading of 0° Magnetic corresponds to a true direction of 22° True. Alternatively, a **western variation** results in a negative correction.

Vector Averaging: The technique for averaging a wind or ocean current by converting each measured speed and direction pair to a corresponding pair of x-y coordinates for averaging. The coordinates are summed over an averaging interval and their final average is computed at the end of the averaging time. Vector averaging removes the errors that occur when direction cycles from 359° to 0°.

Warm Reset: A system reset whereby the computer is reset and restarted but all peripherals remain powered.

Watchdog Timer: A special countdown timer whose function is to cold or warm reset a system if its computer program stops operating. Often during lightning or intense RFI a computer RAM will be corrupted and the program will "crash".

MATHEMATICS OF WIND MEASUREMENTS

Wind direction is the compass angle from which the wind is blowing

Many different wind statistics are in common use. Vector averaging of some sort is necessary for reliable wind direction estimates. You cannot simply average the vane measurement directly without risking grave errors. As an example if the wind blew half the time from 359° and the other half from 1°, the simple vane average would yield a mean direction of 180°!

The compass bearing can be added to the vane measurement for each sample. If the vane direction measurement relative to the sensor is θ_s and the measured corrected compass angle is α, the instantaneous wind direction relative to true north is $\theta_i = \theta_s + \alpha$. If you know the magnetic declination δ at your site, you should input it to the WEATHERPAK, using the System Functions Menu (section 0). However, if there are contaminating magnetic objects in the vicinity of your WEATHERPAK, or if the compass is not installed, then the wind monitor must be aligned as closely as possible to true north. The flag Add Compass To Vane, also set in the System Functions Menu, should be set to 0 (false) in this case.

The different wind statistics available are described below. They are all based upon the same measurements: the wind speed s and direction θ (including the compass bearing) are measured typically once per second (the rate can be factory set). From these, the WEATHERPAK calculates the following quantities for each measurement i:

$$x_i = \sin\theta_i;$$
$$y_i = \cos\theta_i;$$
$$u_i = s_i x_i;$$
$$v_i = s_i y_i;$$

giving x_i and y_i, the unit vector components corresponding to east and north respectively, and u_i and v_i, the wind speed vector components. The vector components are summed over the averaging period, and the vector averages are computed at the end of the averaging interval:

$$X = \frac{1}{N}\sum_{i=1}^{N} x_i;$$
$$Y = \frac{1}{N}\sum_{i=1}^{N} y_i;$$
$$U = \frac{1}{N}\sum_{i=1}^{N} u_i;$$
$$V = \frac{1}{N}\sum_{i=1}^{N} v_i.$$

Vector Average Wind Speed and Direction

The U and V terms are used to compute the vector average wind speed S_V and direction θ_V:

$$S_V = \sqrt{U^2 + V^2};$$
$$\theta_V = \arctan(U, V).$$

The arctangent notation (U,V) means that the signs of U and V are used to determine the exact angle in the range of 0-360°.

Scalar Average Wind Speed

The mean speed S_S is computed from the speed measurements alone:

$$S_S = \frac{1}{N}\sum_{i=1}^{N} s_i.$$

Scalar wind is a measure of the wind regardless of its direction. Yamartino showed[1] that wind gustiness and direction fluctuation will lead to a scalar wind that is always larger than the vector average wind. S_S is usually only a few percent greater than S_V and it serves as a backup measurement if the compass or vane fails and S_V is invalidated.

Standard Deviation of Wind Direction, Sigma-Theta

The standard deviation σ_θ of the wind direction is based on the time series of wind direction measurements, θ_i, and the average wind direction, θ_V. A formal definition of σ_θ is:

$$\sigma_\theta^2 = \frac{1}{N}\sum_{i=1}^{N}\Delta_i^2 - \left[\frac{1}{N}\sum_{i=1}^{N}\Delta_i\right]^2,$$

where all measurements are in radians: Δ_i is defined such that each $|\Delta_i|$ is the smaller of $|\theta_i-\theta_V|$ and $2\pi-|\theta_i-\theta_V|$. Calculating this exactly requires two passes over the data: first to calculate θ_V, and then to find σ_θ.

Coastal Environmental Systems uses a single pass method because data can be processed in real time without storing all the wind samples in memory. Yamartino compared several single pass computations and showed that the most accurate equation -- used in Coastal Environmental System analysis -- is:

$$\sigma_\theta = \arcsin \varepsilon\left[1 + b\varepsilon^3\right],$$

where:

$$\varepsilon^3 = 1-\left(X^2 - Y^2\right);$$

$$b = \frac{2}{\sqrt{3}} = 0.1547.$$

Maximum Wind Speed

Maximum winds (gusts) are determined during each individual averaging period. Gust information is important for risk studies of damage to constructions and buildings. The duration of the gust is an important consideration and is often related to the intended application. Some applications require information about extreme values of the shortest gusts (1 second duration), while in other cases, the damaging gusts are those that engulf the entire structure (5-10 seconds duration). Fortunately, with little error, there is a technique for relating extreme gust measurements taken for one duration to extreme gusts of other durations[2]. The gust duration D in the WEATHERPAK can be factory set to values between 1 and 5 seconds.

In the Coastal Environmental Systems wind algorithm, a D-second running average of the speed measurements is applied to the data as it is collected. At the time of each measurement the average of the circular buffer is computed and compared against the maximum value up to that time. When the current "gust" measurement exceeds the stored maximum, the maximum value is updated.

---

[1] Yamartino, R.J. (1984) A Comparison of Several "Single-Pass" Estimators of the Standard Deviation of Wind Direction. Journal of Climate and Applied Metéorology, 23, 1362-1366.

[2] Beljaars, A.C.M. (1987) The Influence of Sampling and Filtering on Measured Wind Gusts. Journal of Atmospheric and Oceanic Technology, 6, 613-626.

WEATHERPAK-2000 FOR HAZMAT RESPONSE

The WEATHERPAK® -2000 can be configured for HazMat Spill Response. In this configuration the system measures the air temperature and the speed, direction and stability class of the wind. As an option, it can measure barometric pressure and relative humidity. Information is sampled every 2 seconds and then computed into a 5-minute running average. The data is then transmitted to you every 30 seconds for an updated line of data.

A WEATHERPAK® -2000 is suitable for extremely portable use when mounted on a van or a portable tower. It can also be mounted at a fixed site, such as an industrial plant.

The WEATHERPAK® was designed with the following unique features for use in hazardous materials response:

- A built-in electronic compass allows the WEATHERPAK® to be set up in any orientation – it will automatically determine true North and give you true wind direction. [1]
- Set-up time is under two minutes.
- When the unit is assembled, there are no electrical connections that can spark.
- The housing is constructed of 6061-T6 aluminum that is non-corrosive and will not spark if dropped or struck.
- The housing is sealed and dried with desiccant to protect the electronics against moisture. This means that the WEATHERPAK® can easily withstand decontamination procedures.
- A beeper in the tower sounds when battery replacement is required because of low voltage in the battery pack. [2]
- All of the electronics are grounded at a single point to protect the WEATHERPAK® against unexpected large voltages and radio interference.

[1] The variation of true North from magnetic North is entered into your WEATHERPAK® for your location. It is easily changed, but should not be done so without first consulting Coastal Environmental Systems.
[2] The WEATHERPAK® must be installed for the beeper to function.

Installation

Siting Considerations

a) **Compass Measurements** – Remember that the WEATHERPAK® contains an electronic fluxgate compass for automatic North alignment. Like any compass, it can make an error if located too close (laterally) to large amounts of steel or other magnetic material. (In other words, on top of a van is OK, but next to it is not an ideal location.) Try to place the WEATHERPAK® at least 30 meters from large vans, busses, cranes, etc.

b) **Wind Measurements** – Select as exposed a site as is possible. The wind pattern around a building or other obstacle is disturbed for a considerable distance. If the WEATHERPAK® is placed immediately North of your vehicle and the wind is coming from the South, an erroneous wind direction and speed reading will result.

c) **Radio Transmissions** – Locate the WEATHERPAK® as close as safety permits within 2miles of the receiving unit. Do not attempt to transmit through structures containing steel, or through hills. Reception range will vary depending on radio signal path.

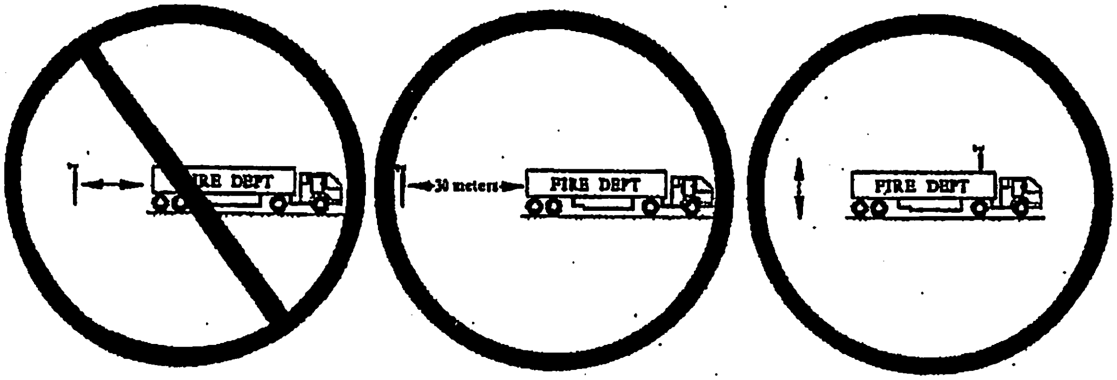

Placement on top of vehicles is okay

Tripod Mount

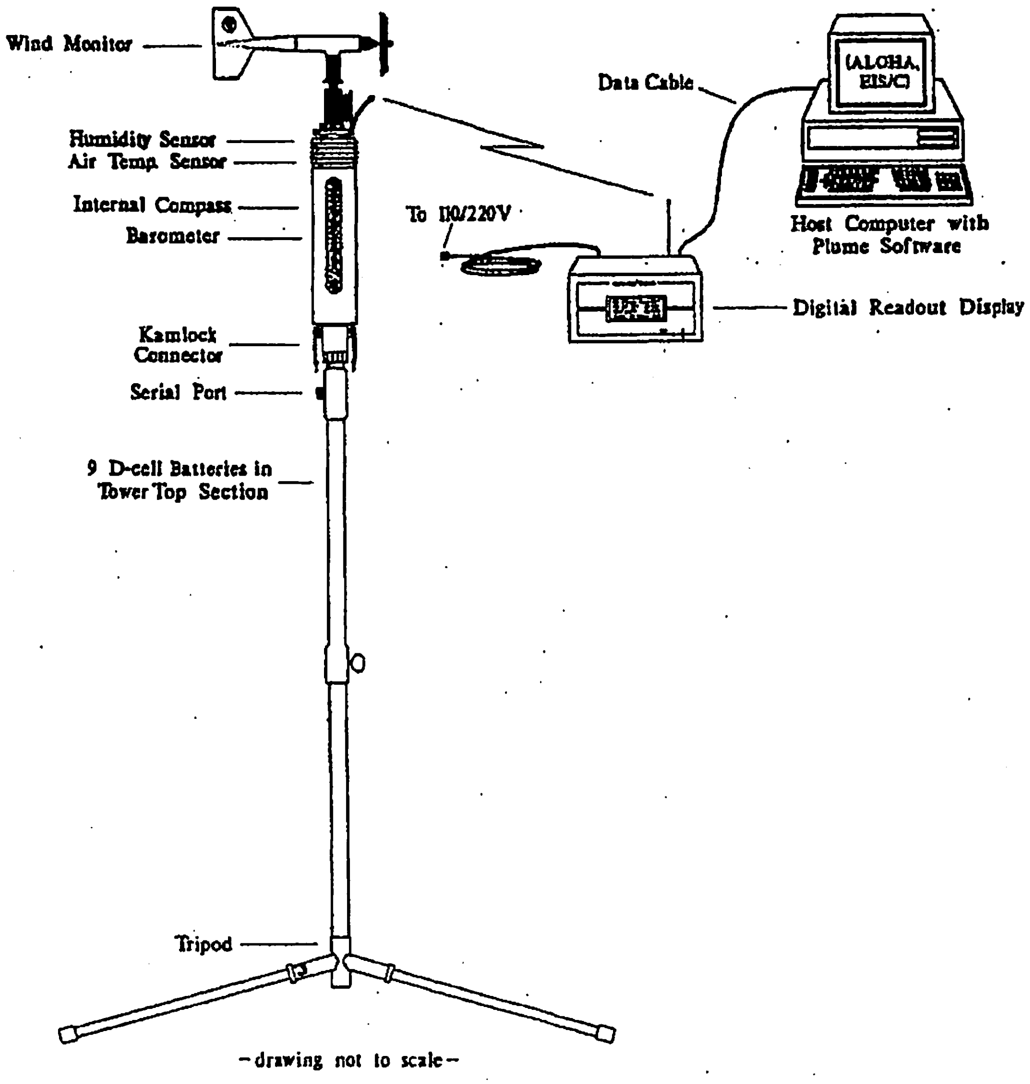

The WEATHERPAK® -2000TRx with tower

Tripod Mount Setup

1) Lock the legs on to the bottom section of the tower forming the tower tripod base.

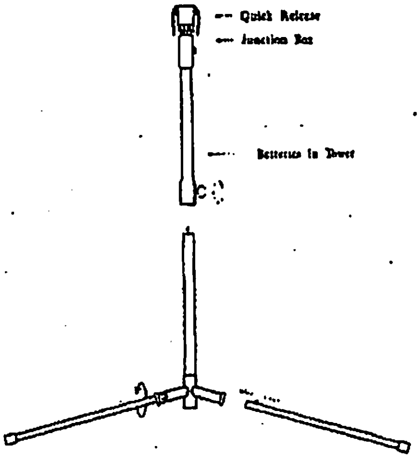

2) Remove the wind vane from the case. Place the propeller on the front shaft screw the nut into place on the propeller shaft to secure the propeller.

3) Plug the wind vane into the top of the WEATHERPAK®. There is an alignment pin and slot inside the connector to assure a proper and aligned fit.
Be certain that the wind vane is plugged all the way in.
DO NOT rotate the wind vane when installing or removing.

4) Align the slot on the WEATHERPAK® with the guide pin on the quick release and push straight in. (DO NOT "screw" the WEATHERPAK® onto the quick release.) This is a good sealed fit and may require an extra push - then **push the arms of the clamp down** to assure a tight fit.
DO NOT rotate the WEATHERPAK® or tower when installing or removing.

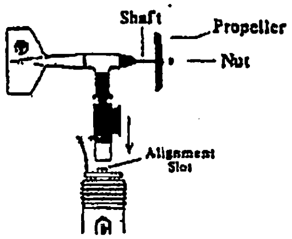

5) Place the entire unit -- tower top and WEATHERPAK® -- onto the tripod and securely tighten the tower locking knob.

6) The WEATHERPAK® is now running and is sampling. When the WEATHERPAK® is removed from the Quick Release, it will stop sampling and shut itself off.

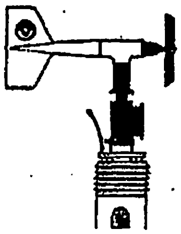

Van Mount Setup

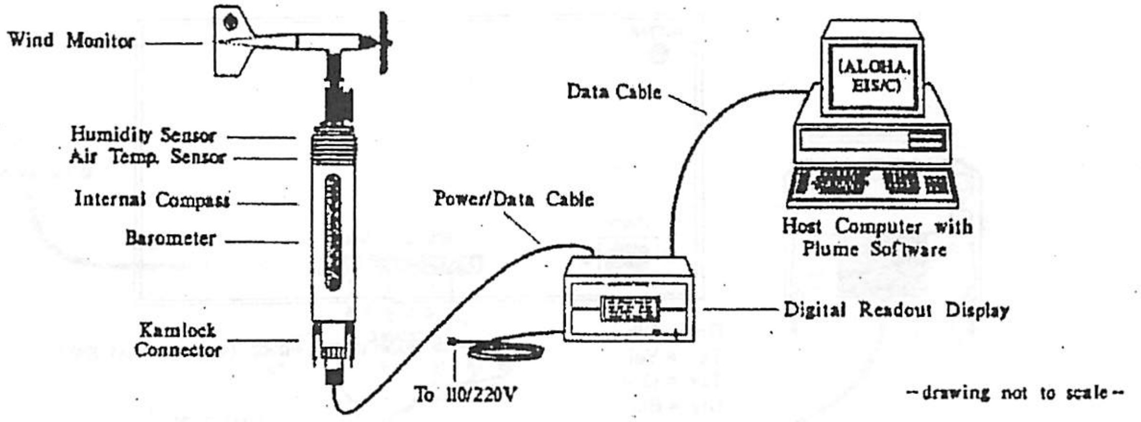

1) Remove the wind vane from the case. Place the propeller on the front shaft screw the nut into place on the propeller shaft to secure the propeller.
2) Plug the wind vane into the top of the WEATHERPAK®. There is an alignment pin and slot inside the connector to assure a proper and aligned fit. **Be certain that the wind vane is plugged all the way in. DO NOT rotate the wind vane when installing or removing.**
3) Align the slot on the WEATHERPAK® with the guide pin on the quick release and push straight in. (DO NOT "screw" the WEATHERPAK® onto the quick release.) This is a good sealed fit and may require an extra push - then **push the arms of the clamp down** to assure a tight fit. DO NOT rotate the WEATHERPAK® or tower when installing or removing.
4) The WEATHERPAK® is now running and is sampling. When the WEATHERPAK® is removed from the Quick Release, it will stop sampling and shut itself off.
5) The Van Mount Quick Release is permanently mounted on a vehicle. Coastal supplies a 1-½" female SPT.

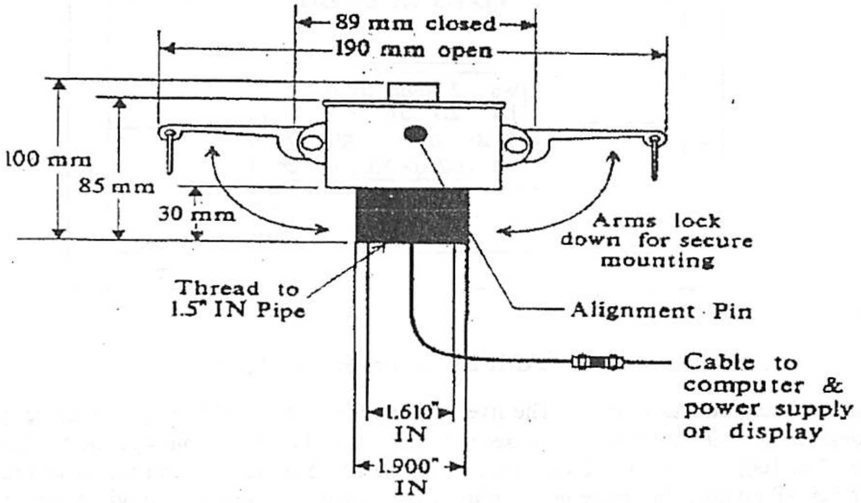

Van Mount Quick Release

Receiver Display Box

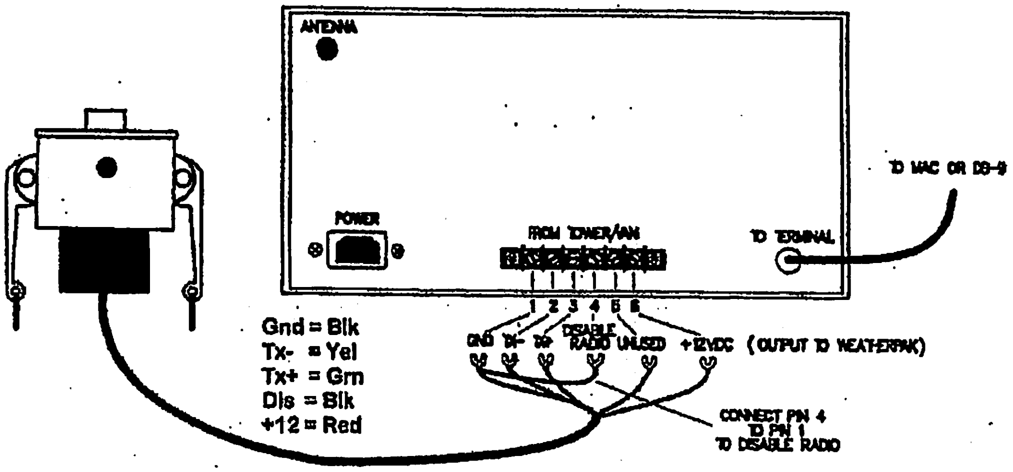

Receiver box rear panel connections.

Internal Switches

The following internal switches change the functions of the RX Box Display only. Serial data will not be affected by switch settings. **IMPORTANT NOTE:** <u>**DO NOT**</u> open the receiver/display box without permission from Coastal Environmental Systems Customer Service, as doing so may void your warranty. Always disconnect the A/C power cord before opening the box. The receiver box contains many static sensitive parts which can be inadvertently damaged by improper handling. **DS1** Unused (remains off); **DS2** Standard = off / Metric = on, **DS3** Degrees = off / Ordinal points = on, **DS4** Unused (remains off), **DS5** English = off / French = on

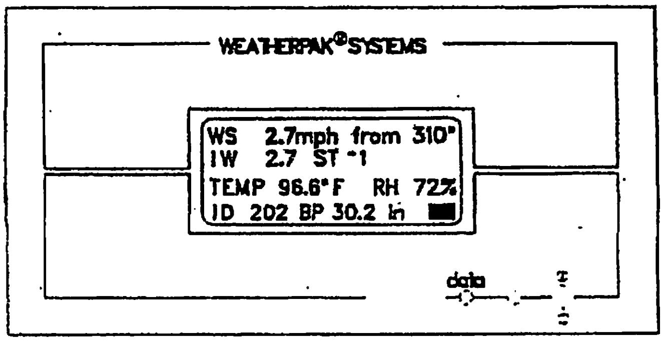

EXAMPLE Receiver front panel

The example is interpreted as follows: The five minute average wind (WS) is 2.7 miles per hour and is coming from 310 degrees. The instantaneous – one second wind speed (IW) is 2.7 miles per hour. The stability (ST) will range from −1 to 100. The −1 is a default reading for the first 5 minutes. After that it will range from 1 to 100. The higher the ST number, the more unstable the wind is with a corresponding wider area of concern. The air temperature (TEMP) is 96.6 degrees Fahrenheit. The relative humidity (RH) is an option. The ID# is unique to your WEATHERPAK®. The barometric pressure (BP) in inches is an option.

Low Power Indicators

1. A beeper will sound in the tower. The WEATHERPAK® must be installed. *Dead batteries will not activate the beeper.*
2. A "T" will flash on the Receiver front panel
3. The battery voltage reads below 11.0 on the "Processed Sam Data: screen

There are low power warning indicators which will flash in the lower right corner of the display. A **flashing "R"** indicates that the Receiver batteries are low. R warning flash starts ≅ 11.5 VDC. Receiver lights go out ≅ 10.5 VDC. If the receiver lights go out, plug the receiver into a wall outlet, then reset the receiver by flipping the power switch off then back on. This will recharge the batteries. Batteries last = approximately 2 hours. Recharge time = approximately 1 hour. A **flashing "T"** indicates that the batteries in the tower are low and need replacing.

Replacing tower batteries and fuse

The WEATHERPAK® has nine alkaline D cell batteries which are located in the top section of the tower, as shown in the figure below. The batteries can be replaced with standard alkaline batteries. Make sure that the replacement batteries are of the same type. There is an in-line fuse located in the tower junction box (see figure below.)

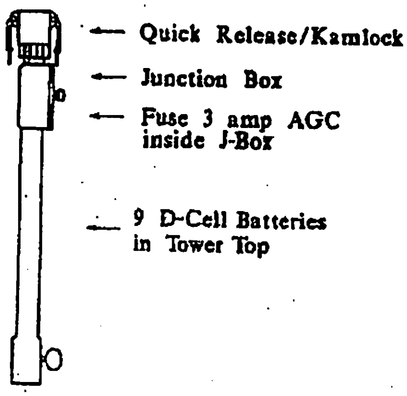

Replacing the batteries

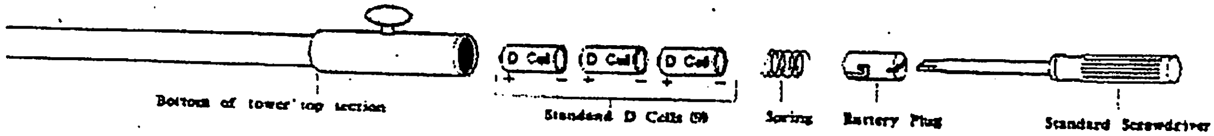

Figure 11. Replacing the batteries (2)

You will need a large screwdriver to replace the batteries. The battery "plug" is slotted. With the screwdriver, **PUSH** (the plug is held in place by a spring) and rotate the plug clockwise. The plug will come out followed by a spring and the batteries. Slide the new batteries in (positive end first) and replace the spring and plug.

To check the new voltage, set up the WEATHERPAK® and get the ALOHA plume model running. Then pull down the [MISC] menu to "Processed Sam data". One of the items shown is battery voltage (see figure 10 on page 14.) It should read about 13 to 15 volts. The tower low voltage beeper will go off when the battery voltage reaches 11 volts.

Plume Modeling Software

With the WEATHERPAK set-up and operational and the Receiver/Display box connected to your computer, real-time data is available to run air dispersion plume modeling software.

IMPORTANT NOTE: Some versions of ALOHA (including 5.2.1) need to be upgraded in order to work with a Station for Atmospheric Measurement (SAM); the WEATHERPAK is a SAM. The US EPA provides CAMEO/ALOHA software support and information at their website http://www.epa.gov/ceppo/cameo/support.htm. To upgrade, go to Updates and Utilities which exits to http://www.nsc.org/ehc/cam/fixitlib.cfm scroll down to ALOHA UPDATES 5.2.2. These addresses change periodically – we'd appreciate knowing any changes you encounter.

The following is a brief outline on using the system to produce an ALOHA plume model on a PC operating Windows 95. There is a general assumption that the user is familiar with ALOHA and that the program is properly loaded on the user's computer. Please consult the ALOHA user's manual for additional details and program limitations.

1. Open ALOHA by clicking on its desktop icon, or selecting it from the Programs menu
2. A series of dialogue boxes will appear including important notes on program limitations.
3. A Text Summary window will appear with information summarizing the event.
4. Confirm that your Site Data information is correct. If required, use the SiteData pull-down menu to change it.
5. Using the pull-down menu SetUp/Chemical select the chemical (chlorine, for example).
6. Using the pull-down menu SetUp/Atmospheric/SAM Station a series of dialogue boxes will appear requiring user observations or assumptions. Relative humidity data is not captured automatically and can be hand-entered using data from the Receiver/Display box.
7. Using the pull-down menu SAMOptions select Processed Data. The WEATHERPAK delivers data in the proper format for ALOHA to use. A Processed SAM Data window will appear. If the WEATHERPAK has been collecting data for less than five minutes a warning message will be displayed in both the Text Summary and Processed SAM Data windows.
8. Before allowing selection of the source of the leak (tank, pipe, direct etc.), ALOHA requires the SAM station to record five minutes worth of data. Using the pull-down menu SetUp/Source select the source of the leak (tank, for example); a series of dialogue boxes will appear requiring user observations or assumptions.
9. Use the Display/Footprint pull-down menu to show the plume footprint. A footprint is required before plotting the plume onto a street map. Refer to the ALOHA manual for interpretations and explanations on selecting locations within the plume footprint.
10. Optional graphs: Use the Display pull-down menu to produce graphs for source strength release rate, concentration, and dose.
11. Use the Display/Tile Windows to show multiple windows on the same screen; reposition and re-size as needed. Display/Stack will organize and stack the windows for quick access.
12. To plot the plume onto a street map, use the Sharing pull-down menu. As weather conditions change the plume size and position will change shortly after the WEATHERPAK provides updated data. Note: some software versions require that the ALOHA window overlay the map window in order for the map-plume to update automatically.

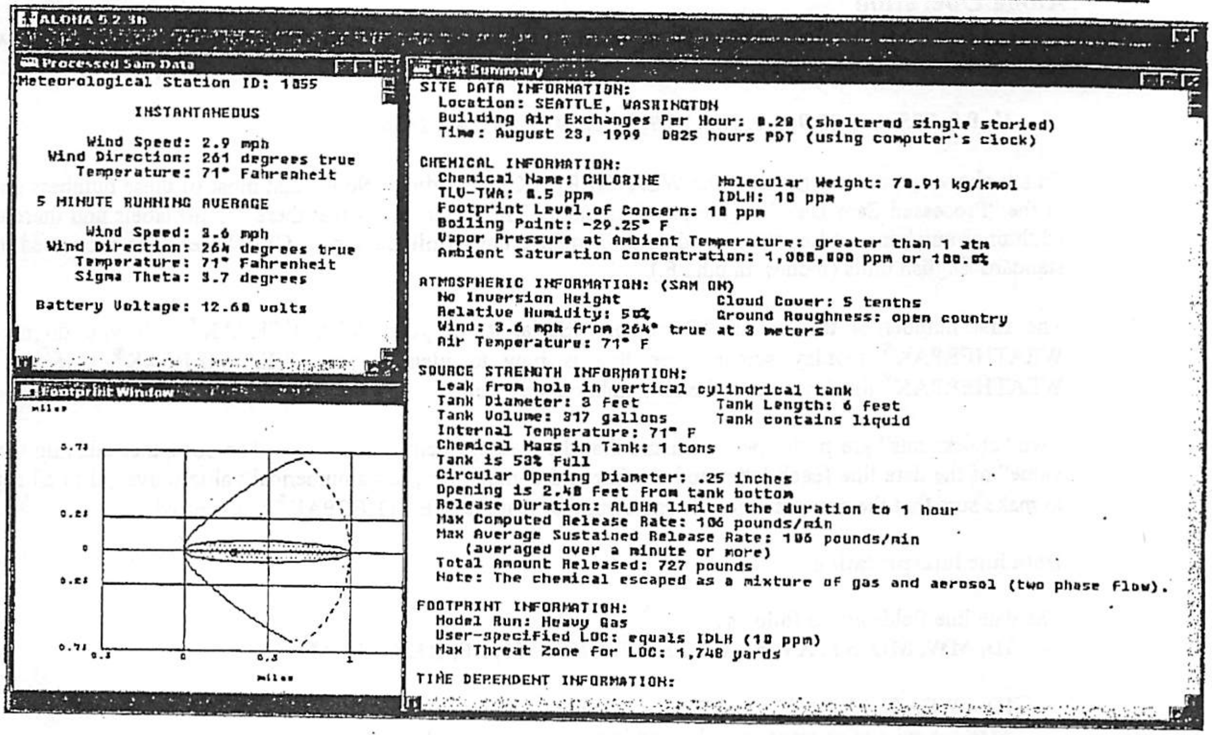

The WEATHERPAK takes a sensor sample approximately every 1-2 seconds and calculates a five minute running average and sends updated data approximately every thirty seconds. "INSTANTANEOUS" data is captured just prior to the thirty-second update. By comparing the "5 MINUTE RUNNING AVERAGE" and "INSTANTANEOUS" data, the user can get a good idea of changing conditions. The WEATHERPAK calculates "Sigma Theta", or air mixing, and produces a Stability Class value used by the plume model. The battery voltage is also transmitted. Note: If voltage is below 11.0 the batteries in the WEATHERPAK tower should be replaced.

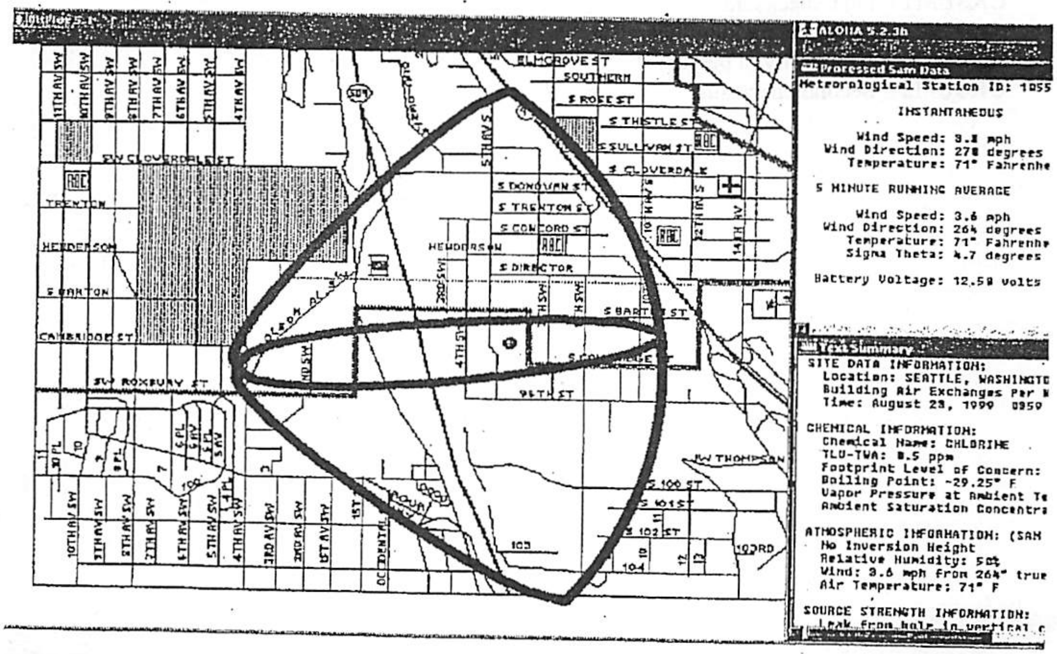

Aloha Operation

If you pull down the "SAM Options" from the Main menu and select "Raw data", something like the following line of data will appear:

421, 0.9, 225, 1.0, 23.9, 1.0. 226, 23.9, 14,0, 1917, 999, 46, 2536

This is showing you exactly what the WEATHERPAK® is sending. Notice that most of these numbers are present in the "Processed Sam Data" on the previous page. The difference is that there are no labels and there are some additional numbers. Also, this raw data is in metric units (millibars, m/s, C), whereas the processed data is in standard English units (inches, m.p.h., F.)

The first number is the unique ID number (Serial #) of your WEATHERPAK®. If you do not have a WEATHERPAK® display screen, then this is how to identify your WEATHERPAK®. If you have a WEATHERPAK® display screen, the ID number appears on it.

Two "checksums" are performed to ensure that the message sent was correct. The computer adds up the "ASCII value" of the data line (each letter and number and comma, etc., has a numerical value universal to all computers) to make sure that the computer got the same number that the WEATHERPAK® transmitted.

Data line interpretation

The data line fields are as follows:

ID, MW, MD, ST, AT, SI, DI, TI, BV, CKSUM1, BP, RH CKSUM2

ID – WEATHERPAK® identification number
MW – 5 minute averaged wind speed in meters per second
MD – 5 minute averaged wind direction in degrees
ST – Stability class in degrees
AT – 5 minute averaged air temperature in degrees Centigrade
SI – Instantaneous wind speed in meters per second
DI – Instantaneous wind direction in degrees
TI – Instantaneous air temperature in degrees Centigrade
BV – Battery voltage in volts
CKSUM1 – First checksum
BP – Barometric pressure in millibars
RH – Relative humidity in percent
CKSUM2 – Second checksum

TROUBLESHOOTING

Do not take the WEATHERPAK® or the Receiver box apart; this will void the warranty. If the procedures below do not solve the problem, call Coastal Environmental Systems.

Is the WEATHERPAK® on?

Once the WEATHERPAK® is set up, it turns itself on, and starts sampling and transmits data every 30 seconds. If it does not:

- Check that the WEATHERPAK® is properly secured in the quick release – reseat firmly.
- Check the power connection to the WEATHERPAK®, or
- Check the tower batteries and fuse.

Is the Receiver/Display Box on?

The display light should be on and the display characters visible. If this does not occur, do the following:

- Be sure the receiver unit is plugged in and turned on.
- Check the power light on the front panel. It should be lit.
- If it is not lit, check for power at the outlet the RX Box is plugged into.
- Check the cable connections from the WEATHERPAK® to the receiver,

Receiver/Display Box Data light not flashing?

Every 30 seconds (approximately), the data light will flash signifying that the WEATHERPAK® is updating the display. If the light does not flash, then do the following:

- Be sure the receiver unit is plugged in and turned on.
- Check the power light on the front panel, it should be lit.
- Check to see if the WEATHERPAK® is "line of sight" (less than 2 miles, and not transmitting hrough hills, or steel walls, etc.)
- Be sure you are not trying to transmit through structures containing lots of steel.
- Check to see if both antennas are connected (WEATHERPAK® and receiver.)
- Unclamp, remove, wait 10 seconds, then replace the WEATHERPAK® on the tower (this resets it.)
- Check to see if the low battery beeper in the tower is beeping.
- Check that the batteries in the tower are properly aligned and are the correct voltage.
- Check the power connection to the WEATHERPAK®.
- Check the cable connections from the WEATHERPAK® to the receiver

Wind speed off?

- Check that the propeller is turning. There is a minimum threshold of about 2.5 MPH.

Propeller is turning, wind speed reads zero?

- Make sure that the wind monitor has been pushed down all the way on to the WEATHERPAK®.

Wind direction off?

For this job you need a partner. Have your partner go to the wind monitor and hold the vane so the propeller points in a known direction. Does the display read correctly? If not:

- Make sure that the wind monitor has been pushed down all the way on to the WEATHERPAK®. There is an alignment pin and slot inside the connector to assure a proper and aligned fit; make sure that the pin is in the slot. See Tower Mount Setup.

Other sensor readings off?

- Contact Coastal Environmental Systems. The WEATHERPAK® may be broken or require maintenance.

There is data at the display but there is either no data or erroneous data at the computer?

- Check all the connections from the receiver to the computer.

Error messages while running plume modeling?

Typically, these are not related to the WEATHERPAK®; they are coming from the plume modeling software.

- ALOHA version 5.2.1 will not work with weather stations, free upgrades are available
- Consult your CAMEO/ALOHA manual.
- Contact the US EPA for help with CAMEO/ALOHA software.
- Coastal is NOT an authorized CAMEO/ALOHA representative; however, we may be able to help.

MAINTENANCE

Routine maintenance is required on the WEATHERPAK® every 12 months. This maintenance is to ensure that the overall system and its sensors are working and performing to specifications. The actual service varies, based on the sensors installed in your model of WEATHERPAK®. For example, a WEATHERPAK® -2000 should have the wind, compass, air temperature (and, optionally, the barometric pressure and relative humidity) sensors tested to their stated specifications. In addition, the entire WEATHERPAK® should be examined for any wear, damage or other non-conforming variances.

SYSTEM DESCRIPTION, SCHEMATICS AND CONFIGURATION

Your WEATHERPAK®-2000 Weather Station is made up of the following major components:

WIND MONITOR		S1104W	RM Young 05103
		S1145W	RM Young AQ
			Handar Sonic
AIR TEMPERATURE		S1074W	YSI 44203
			YSI 44304
RELATIVE HUMIDITY			VAISALA 50U – 3%
			Hygrometrics – 4%
			VAISALA Hmm20D – 2%
BAROMETER		S1081W	IC Sensor 1220A
			Setra 270
PYRANOMETER		S1114	Licor 200 SZ
			Eppley 8-40
			Eppley PIR
DATA STORAGE MEMORY			1 MB
OPERATING SOFTWARE		S1288	ZENOSOFT version 1.966
		S1329	MET EXPERT
DATA COLLECTION SOFTWARE		S1341	INTERCEPT Network 3.34B
		S1137	INTERCEPT 3.34B
		S1140	INTERCEPT Format Editor 2
TOWER		S1125W	3 Meter Tripod Tower
			3 Meter Universal Tower
		S1118W	10 Meter Universal Tower

RADIO		S1064W	2 Watt 467.8
RADIO RECEIVER			
RADIO RECEIVER/DISPLAY BOX		S1016	
RECEIVER/DISPLAY BOX		S1011	
CABLE LENGTH			

VAISALA

100 Commerce Way, Woburn, MA 01801
Tel: 1-888-VAISALA (824-7252)
Fax: (781) 933-8029
Email: incsales@vaisala.com
www.vaisala.com

Humidity Modules for OEM Applications

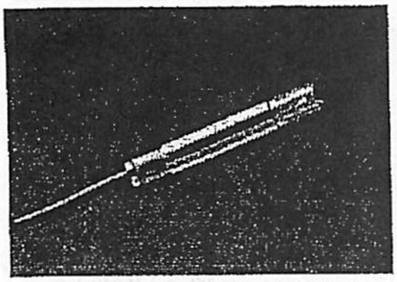

The Vaisala INTERCAP® Humidity Probes HUMITTER50 are a low cost humidity and temperature probes for OEM applications. It features excellent long-term stability, low hysteresis and repeatability.

CE

HUMITTER50

- 0...100 %RH measurement
- -10...+60 °C (+14...+140 °F) temperature measurement range (HUMITTER 50Y and 50YX)
- ±3 %RH accuracy with better than ±1 %RH stability per year
- No recalibration
- Fully interchangeable Vaisala INTERCAP® Sensor is easy to replace
- Low cost
- Suitable for OEM applications in HVAC, environmental monitoring, food transportation and cabinets, and humidifiers and dehumidifiers

Vaisala developed the HUMITTER50 to meet the demand for a simple and cost effective humidity transmitter, which is suitable for volume OEM applications or integration into other manufacturers' equipment.

Dimensions

Dimensions in mm.
HUMMITTER50

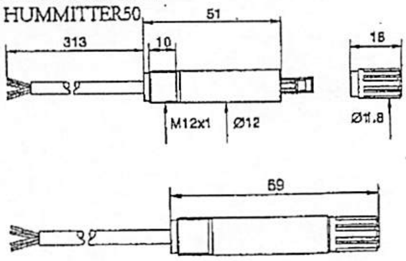

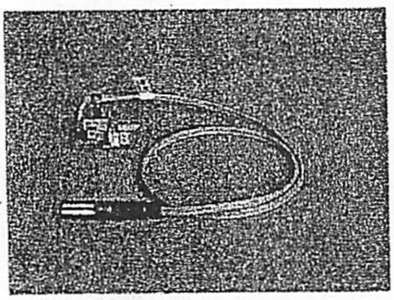

The Vaisala HUMICAP® Humidity Module HMM22D is ideal for use in incubators.

HMM22D

- Vaisala HUMICAP® Sensor for excellent accuracy and long-term stability, negligible hysteresis and resistance to dust and most chemicals.
- NIST traceable (certificate included)

HMM22D
- 4 to 20 mA output
- Temperature range -40...+176 °F (-40...+80 °C)
- Cable length 25.6 in (65 cm)

The HMM22D and the HMM30C are two examples of the diverse products that Vaisala designed to meet specific customer requirements.

Dimensions

Dimensions in mm.
HMM22D

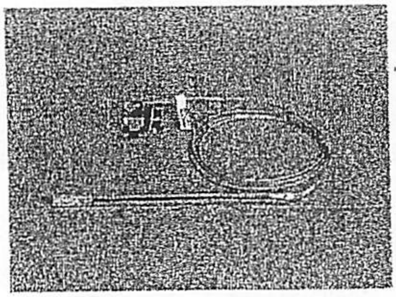

The Vaisala HUMICAP® Humidity Module HMM30C is ideal for use in high temperature environmental chambers.

HMM30C

- Vaisala HUMICAP® Sensor for excellent accuracy and long-term stability, negligible hysteresis and resistance to dust and most chemicals.
- NIST traceable (certificate included)

HMM30C
- High temperature applications
- Temperature range -40 ...356 °F (-40...+180°C)
- Cable length 5 ft (1.6 m)

The HMM30C was designed to for high temperature, high performance environmental chambers, such as those used for accelerated aging tests.

Dimensions

Dimensions in mm.
HMM30C

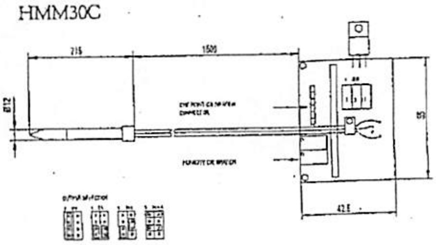

Humidity Modules for Environmental Chamber OEM Applications

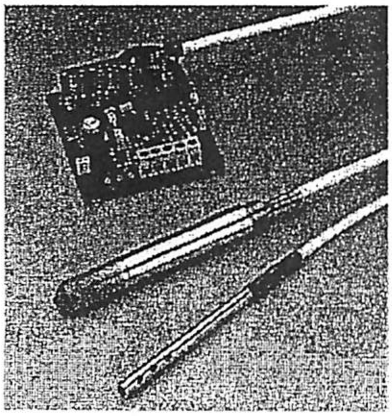

The Vaisala HUMICAP® Humidity and Temperature Module Series HMM210 provide fast real-time measurement in a wide temperature range. The modules are especially suitable for demanding OEM applications, environmental chambers and incubators.

HMM210 series

- Three probe configurations
- Relative humidity (RH) plus temperature (T) probe.
- Dewpoint probe features Vaisala's unique Composite Sensor, which remains heated a few degrees above ambient to prevent dew formation (condensation) on the sensor.
- Dewpoint probe described above, together with a temperature probe for obtaining relative humidity and temperature outputs.
- Three module configurations
- Different probe and cable lengths
- Chemical purge option maintains high measurement performance in demanding chemical conditions.
- All HMM210 series modules have excellent EMC characteristics.
- NIST traceable (certificate included)

The Vaisala HUMICAP® Humidity and Temperature Module Series HMM210 are designed for OEM type applications needing humidity or dewpoint measurement in demanding environments, environmental chambers growth chambers and incubators.

NOTE:

The HUMITTER50, HMM22C, HMM30C and the HMM210 Series are OEM products. Please call for pricing.

Dimensions

Dimensions in mm.
HMM210 Series

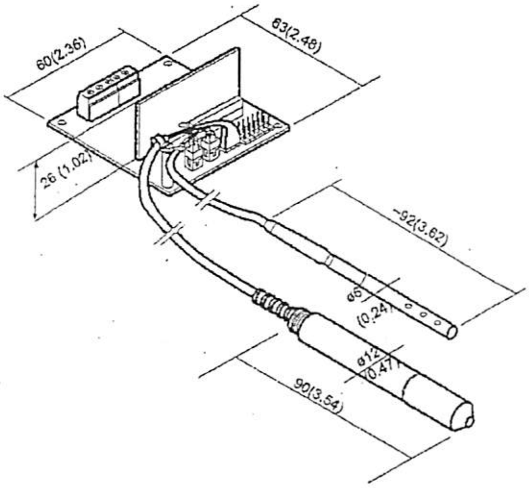

Test Tech:	Date	Part Number	S/N
KM	12 MAY 2010	2930-000-299	041845

Applies to p/ns: 2930000292, 2930000299, 2930000321

Test Equipment Required

Device	S/N	Cal Due Date	Remarks	Pass/Fail
Power Supply w/ current limit	N/A	N/A		
Terminal	N/A	N/A		
Host system (Zeno or Weatherpak)	N/A	N/A		

Procedure

1 Bench test. Couple the WindSonic to the host system and power supply, using a known working test cable. Use the terminal and record the configuration by going into Configuration mode and using the D3 command. See Manual Section 10.3 *Checking the configuration* for more information. A typical configuration looks like:

```
D3
M2,U2,O2,L1,P2,B3,F1,H1,NQ,E3,T1,S3,C1,
```

1a. Configuration	M2,U1,O1,L1,P2,B3,F1,H1,NQ,E3,T1,S3,C1,

1b. Check for normal output data, and that the Status Code is OK – 00 (or A for NMEA format). If the status code is other than 00, refer to Manual Section 12.5 *Status (error) codes* for more information.

Item	Value	Limit	Remarks	Pass/Fail
1b. Status Code	00	OK		PASS

1c. Use an office fan or similar to check that the unit is sensing wind, turning the unit to simulate changing wind direction and to check that both axes are functioning. Note that this a quick functional test. There are no calibration adjustments; the unit is designed NOT to require re-calibration within its lifetime.

Item	Observation	Limit	Remarks	Pass/Fail
1c. Changing wind direction	YES	Direction must change		PASS

2 Self-Test (Still Air). This test checks Alignment, Gain and Checksums. **Alignment tests :** The unit performs a transducer geometry check and compares the result with its factory setting. **Gain tests :** The unit performs a check of its operating gain against its factory setting. **Checksum tests :** The unit performs a check of its program and data memory. *Important This test is a stringent laboratory test which will only be passed if carried out under still air conditions at room temperature (17-23°C).*

Use the original packing box (inner and outer) to enclose the unit. (The packaging was designed as a zero wind enclosure). Go into Configuration Mode * ENTER . Carry out the Self-test by entering D 6 ENTER. A message similar to that shown below in the table will be generated. For each of the Alignment and Gain tests a Pass or Refer to Manual message is generated. For each of the Checksum tests a Pass or Fail message is generated (except the first message "Alignment Limit:")

Item	Value	Limit	Remarks	Pass/Fail
2a. ALIGNMENT LIMITS:0D59,0CF5	N/A	PASS		PASS
2b. ALIGNMENT U:OD15 *PASS*		PASS		PASS
2c. ALIGNMENT V:OD16 *PASS*		PASS		PASS
2d. GAIN 0:0001 *PASS*		PASS		PASS
2e. GAIN 1:0001 *PASS*		PASS		PASS
2f. GAIN 2:0001 *PASS*		PASS		PASS
2g. GAIN 3:0001 *PASS*		PASS		PASS
2h. CHECKSUM ROM:AB7D AB7D *PASS*		PASS		PASS
2i. CHECKSUM FAC:04F4 04F4 *PASS*		PASS		PASS
2j. CHECKSUM ENG:082A 082A *PASS*		PASS		PASS
2k. CHECKSUM CAL:A9C1 A9C1 *PASS*		PASS		PASS

If any of the tests fail, contact your supplier. If a "refer to manual" message appears please *see Section 12.3 Fault Finding. Note that it will only pass if the specified temperature and zero wind conditions are met.* Check that there are no visible obstructions or damage to the unit before contacting Gill or your authorized distributor for further advice.

Evaluation

All steps must be completed and all tests must be passed.

QA:	Pass /Fail	Date:	Remarks
P. GARNER	PASS	5/12/10	

Rev	Date	By	Description
0	5/12/05	SJN	New Test

0308000073Rev0 Test Form, WPAK, Service and Repair FAT 20 Oct 2006

Test Tech:	Date:	Sales Order	WP Model #	WP Serial #
KM	12 MAY 2010	SO-8637	WP2000	1822

Test Equipment Required

Device	S/N	Cal Due Date	Remarks	Pass/Fail
Fluke DVM	US 36138891	4 JAN 2011		PASS
AT RH ref sensor	W2120019	25 FEB 2011		PASS
Barometric pressure ref	7C3976	6-30-10		PASS
Wind speed calibrator	NA	NA		NA
Wind direction table	NA	NA		NA
Compass table	NA	NA		NA
RF wattmeter w/10W 400-1000 MHz element	NA	NA		NA
Configuration file: 18224178.txt.TXT	ZENO3200 (ZENOSOFT) Software version, date and C.S. V2.02 Sep 10 2002 11:29:41 CS B97B			

1. HIGH CURRENT DRAIN TEST (ALL SENSORS AND RADIO CONNECTED, *NO TERMINAL*)

Current W/Radio	Limit	Current No Radio	Limit	Pass/Fail	Remarks
N/A	750ma to 1000ma	140 mA	<240 mA Average	PASS	

2. WIND SPEED TEST For 2a or 2b attach wind test to back of test form.
2c. Speed in measured in units of: m/s mph kts

Calibrator RPM	Wind Speed	Limit RMY 05103 RMY 05203	Limit RMY 05305	Pass/fail	Remarks
200	N/A	0.0 - 1.2 m/s<br>0.0 - 2.6 mph<br>0.0 - 2.3 kts	0.8 - 1.2 m/s<br>1.8 - 2.8 mph<br>1.5 - 2.3 kts	N/A	GILL
6400		30.7 - 32.0 m/s<br>68.8 - 71.6 mph<br>59.7 - 62.2 kts	32.0 - 33.3 m/s<br>71.6 - 74.5 mph<br>62.2 - 64.7 kts		

Wind Speed Threshold

Wind Monitor	Torque	Limit gm-cm	Pass/Fail	Remarks
5103	N/A	<2.4	N/A	GILL
5305 (AQ)		<0.3		
5701 (RE)		<0.3		

3. VANE TESTS

Table CW	Measured	Table CCW	Measured	Limit	Pass/Fail	Remarks
0	N/A	0	N/A	357-3	N/A	GILL
45		45		42-48		
90		90		87-93		
135		135		132-138		
180		180		177-183		
225		225		222-228		
270		270		267-273		
315		315		312-318		
355		355		352-1		

Wind Direction Threshold

Wind Monitor	Torque measured	Limit Torque gm-cm	Pass/Fail	Remarks
5103	N/A	30	N/A	GILL
5305(AQ)		9		
5701(RE)		7		

4. COMPASS TEST

TABLE	CLOCKWISE	Counter CW	LIMIT	Pass/Fail	Remarks
0	1	0	357 - 3	PASS	
60	61	SKIP THIS	57 - 63	PASS	
120	122	121	117 - 123	PASS	
180	181	SKIP THIS	177 - 183	PASS	
240	241	241	237 - 243	PASS	
300	300	SKIP THIS	297 - 303	PASS	
360	2	1	357 - 3	PASS	

5. Air Temperature

	LAB	WP	LIMIT	Pass/Fail	Remarks
WP400			±0.2°C		
WP2000	22.8	22.8	±0.2°C	PASS	

6. RELATIVE HUMIDITY

Lab	Wpak	Delta	Limit	Pass/Fail	Remarks
36	36	0%	S1057: 2% (Vaisala HMM22D)<br>S1112: 2% (Vaisala HMP45D)<br>~~S1276: 3% (Vaisala Humitter)~~<br>~~S1113: 4% (Humirel HTM2500)~~<br>S1276: 3% (Vaisala HMP50)	PASS	

7. BAROMETER

LAB	WP	DELTA	Type of pressure sensor	Limit mB	Pass/Fail	Remarks
			S1233 Honeywell PPT (serial i/f)	±0.3		
			S1233 Honeywell PPT (analog i/f)	±0.6		
			S1079 Setra 270 w' option 623 (12V)	±0.3		
			S1080 Setra 270 w' options 623, 703	±0.3		
			S1394 Setra 270 w' options 623, 707	±0.2		
1024	1024	0	S1081 3C 1220A-015A-3L	±4.0	PASS	
			(N/A) Vaisala PTB 200	±0.2		
			S1082 Paroscientific 6016-B	±0.1		

8. RADIO TEST

Forward Power	Limit	Reflected Power	Limit	Pass/Fail	Remarks
N/A	1.0 - 2.0 W	N/A	< 0.1W	N/A	

9. RECIEVER AND TOWER TESTS

	Value	Limit	Pass/Fail	Remarks
9.a Rcvr software Ver.			NA	
9.b Display operation	NA	Data Present on Display	NA	
9.c Tower Battery Voltage	NA	13.0 - 13.9Vdc	NA	
9.d Buzzer operation	NA	Buzzer sounds	NA	
9.e Diode check	NA	14.5 - 15.0Vdc	NA	
9.f Up-light Test On	NA	Light on w/ wpak connected	NA	
9.g Up-light Test Off	NA	Light off w/ wpak removed	NA	

PARAMETERS AS SHIPPED

System OK?	Decals OK?	Clean?	BP Offset	Compass Offset	Unit ID
YES	YES	YES	547.9	0	2

FINAL QUALIFICATION

QA by:	Date:	Pass/Fail	Remarks
P. GARNER	5/12/10	PASS	

SENSIDYNE, INC.

CALIBRATION CERTIFICATE

CELL S/N: 002598-S DATE: 07 - 23 - 2010

This is to certify that the above referenced Gilibrator Flow Cell was calibrated using film flowmeter MCS-102-A, which has been calibrated by instruments directly traceable to the National Institute of Standards and Technology. NIST Report 8361604.

Results:

REFERENCE MCS-102-A cc/min	S/N 002598-S cc/min	RELATIVE DIFF. cc/min	PERCENT DIFF.
2021	2021	0	0.0
2020	2022	2	0.1
2020	2022	2	0.1
2022	2021	-1	-0.05
2020	2024	4	0.2
2022	2021	-1	-0.05
2022	2024	2	0.1
2024	2021	-3	-0.15
2024	2022	-2	-0.1
2024	2022	-2	-0.1
MAX		4	0.2
MEAN 2021.9	2022		

CALIBRATED BY Agnes Bayfield DATE: 07 - 23 - 2010

CODE 000

© 2004 GOES 441CN

SENSIDYNE, INC.

CALIBRATION CERTIFICATE

CELL S/N: 0208000-H DATE: 07 - 23 - 2010

This is to certify that the above referenced Gilibrator Flow Cell was calibrated using film flowmeter MCH-101-A, which has been calibrated by instruments directly traceable to the National Institute of Standards and Technology. NIST Report 8361604.

Results:

	REFERENCE MCH-101-A LPM	S/N 0208000-H LPM	RELATIVE DIFF. LPM	PERCENT DIFF.
	5.03	5.03	0	0.0
	5.018	5.011	-.007	-0.14
	5.032	5.033	.001	0.02
	5.029	5.03	.001	0.02
	5.03	5.034	.004	0.08
	5.039	5.038	-.001	-0.02
	5.032	5.039	.007	0.14
	5.027	5.027	0	0.0
	5.026	5.032	.006	0.12
	5.032	5.034	.002	0.04
MAX			-.007	-0.14
MEAN	5.03	5.031		

CALIBRATED BY *Agnes Banfield* DATE: 07 - 23 - 2010

CODE 100

LITHO. IN U.S.A.

© 2004 GOES 441CN

APPENDIX E

EXAMPLE FIELD DATA SHEETS

CHAIN-OF-CUSTODY SHEETS

Air Sampling Data Sheet

Site: Sumas Mtn. Asbestos Methods Comparison Study

Project Code: SFP-078A

Date:

Sample ID	Location ID	Pump ID No. / Name*	Flow Rate (L/min)			Time		Duration (minutes)	Volume (liters)
			Start	Stop	Average	Start	Stop		
					#DIV/0!			0	#DIV/0!
					#DIV/0!			0	#DIV/0!
					#DIV/0!			0	#DIV/0!
					#DIV/0!			0	#DIV/0!
					#DIV/0!			0	#DIV/0!
					#DIV/0!			0	#DIV/0!
					#DIV/0!			0	#DIV/0!
					#DIV/0!			0	#DIV/0!

Entries by: **Date:**

* For Air Monitoring Stations, enter "Stationary" otherwise enter the persons Name for personal air monitor.

Sumas Mtn Asbestos Site - Region Methods Comparison Study

GPS Coordinate Field Sheet

Datum: WGS84 **GeoMethod:** GPS **Instrument:** Garmin Montana / SN#: ______________

Location	Latitude	Longitude	Date	Time	Collected by
**ISM Sampling Area:** OCSP-01-ISM					
**NW Corner**					
**NE Corner**					
**SW Corner**					
**SE Corner**					
**Centroid (Calc.)**			NA	NA	NA
**Soil Sample Grab Locations**					
**D-01**					
**D-02**					
**D-03**					
**D-04**					
**D-05**					

EPA Region 10, 1200 Sixth Avenue, Seattle, WA 98101

Sample Custody & Analysis Required Form

EPA Manchester Laboratory, 7411 Beach Drive East, Port Orchard, WA 98366, 360-871-8700

Form Effective Date: January 2005

Revision 0

Project Name	*Project Code*	*Method of Shipment/carrier*	*Airbill Number (if known prior to sealing):*
Account Code	*EPA Project Manager/phone number*	*Check all that apply* ❑ Enforce/Custody ❑ Possible Toxic/Hazardous ❑ Data Confidential	

Sampler Names (Print & Sign). *Mark* (R) *after name of principal recorder.*

If applicable, circle the set of selected metals:

Al	Sb	As	Ba	Be
B	Cd	Ca	Cr	Co
Cu	Fe	Pb	Mg	Mn
Mo	Ni	K	Se	Ag
Na	Sn	Tl	V	Zn

(see reverse for more to add/circle)

① Matrix Codes:
- 10 Water/Liquid (Total)
- 20 Water/Liquid (Filtered)
- 40 Sediment/Soil/Solid/Bulk
- 70 Tissue
- 80 Oil/Solvent
- 44 Air filter
- 42 Wipe/Swab[1]
- 00 ________________

[1] PCB wipe is to be 10cm x 10cm (100 cm^2)

#C ② enter the number of containers for each preservative type followed by the appropriate preservation code **P ③:**

- A - HCl
- B - HNO_3
- C - NaOH
- D - H_2SO_4
- E - $Na_2S_2O_3$
- F - ascorbic acid[2], then HCl
- G - $Na_2S_2O_3$+EDTA
- H - EDTA
- N - No chemical preservation
- P - Bottles pre-preserved at lab
- T - To be preserved at the lab

[2] *$Na_2S_2O_3$ if required by plan.*

W - ________________________

❑ Check here if the cooler is iced

Sampler's comments for the laboratory:

Laboratory: *see the applicable QAPP, SOW and/or Analytical Support Request for specific methods and detection, reporting, and/or quantitation limits*

EPA Sample number: Yr	Wk	Sequence	Sampling Date & Time: Yr	Mo	Day	Time	Matrix ①	#C ②	P ③	#C ②	P ③	#C ②	P ③	#C ②	P ③	Sampler Initials	Sample/Station Description/Field Measurements	**Organics** *(see reverse)*: VOA	BNA	Pest	PCB	PAH	**Metals** *(see reverse)*: CLP	Selected	Mercury	**Micro** *(see reverse)*: E. Coli	F. Coliform	T. Coliform	**General Chemistry** *(see reverse)*: TSS	TDS	BOD 5	NO2+NO3	Oil & Grease	Asbestos	**Additional Write in Analyses** *(see reverse)*			

Chain of Custody Record

Relinquished by *(Signature)*	Date	Time	Received by *(Signature)*	Date	Time
Relinquished by *(Signature)*	Date	Time	Received by *(Signature)*	Date	Time
Relinquished by *(Signature)*	Date	Time	Received by **Mobile Lab** for Field Analysis *(Signature)*	Date	Time
**Shipped** by *(Signature)*	Date	Time	Received for **lab** by *(Signature)*	Date	Time

Receiving Laboratory Information Condition of Samples upon Receipt at Lab:

Custody Seals Intact: ❑ yes ❑ no ❑ none

Distribution: White - Laboratory Copy;
Yellow - Regional Sample Control Center (RSCC) Copy; Pink - Field or Office Copy

Additional Matrix Codes: 30 Leachate 50 Sludge 60 Air

Matrix codes: these are the codes in use at the EPA Region 10 Laboratory. Pick the matrix code that best matches the sample matrix. If in the opinion of the sampler, the sample matrix needs to be specially described, select 00 and write in a matrix description. Remember, tissue can be animal or vegetable in nature.

If the write in area becomes filled, cross out one of the pre-printed analyses and write in what is needed. Try to use the bolded analyte symbol/abbreviation (some analyses are not abbreviated).

Organics pre-printed on the form:

PAH Polynuclear Aromatic Hydrocarbons (these are a subset of the compounds reported from GC-MS analyses for BNA - PAH by HPLC or SIM-GC/MS methods are usually requested in order to get low reporting limits). **Pest** Organochlorine Pesticides **PCB** Polychlorinated Biphenyls aka Aroclors **VOA** (aka VOC) - volatile organic compounds **BNA** (aka SVOC or SVOA) - semivolatile organic compounds

Organics that can be written in:

AED scan (detects chlorinated or brominated hydrocarbons) **Butyltins** Butyltins (mono, di, tri, tetra substituted) **CB Con** - Chlorinated Biphenyl Congener analysis **Chlor Hyd**. Chlorinated Hydrocarbons **Chlorophenols** **Gua/Cat** Guaiacols/Catechols scan **Herb** Herbicides **OP Pest** Organophosphorous Pesticides **PBDE** Polybrominated diphenylethers **Resin Acids** **TPH-Dx** Total Petroleum Hydrocarbons, diesel range **TPH-Dx-ext** Total Petroleum Hydrocarbons, diesel range extended to motor oil **TPH-Gx** Total Petroleum Hydrocarbons, gasoline range **TPH-HCID** Total Petroleum Hydrocarbons, identification **THMs** Trihalomethanes

Metals pre-printed on the form *(underlined = 'CLP metals' - mercury must be separately requested)*:

Al aluminum **Sb** antimony **As** arsenic **Ba** barium **Be** beryllium **B** boron **Cd** cadmium **Ca** calcium **Cr** chromium **Co** cobalt **Cu** copper **Fe** iron **Pb** lead **Mg** magnesium **Mn** manganese **Hg** mercury **Ni** nickel **K** potassium **Se** selenium **Ag** silver **Na** sodium **Sn** tin **Tl** thallium **V** vanadium **Zn** zinc

Metals that can be written in and then circled under the box used for designating selected metals:

Au gold **Cr+6** hexavalent chromium **Mo** molybdenum **Sr** strontium **Ti** titanium **W** tungsten **Zr** zirconium

Note: some metals may not be analyzed for on matrices other than soil/sed or water.

Microbiology Analyses pre-printed on the form:

E. Coli Escherichia coli **F. Coliform** Fecal Coliform **T. Coliform** Total Coliform

Microbiology Analyses that can be written in:

Enterococci **MPA** Microscopic Particulate Analysis for Determining GWUDI **G/C** Giardia/Cryptosporidium **Coliphage** **Staph a** Staphylococcus aureus

Toxicity Characteristic Leaching Procedure (TCLP) write in analyses[3]:

TCLP BNA **TCLP Herb** TCLP Herbicides **TCLP met+Hg** TCLP metals including mercury **TCLP met** TCLP metals not including mercury **TCLP Hg** TCLP mercury **TCLP Pest** TCLP Pesticides **TCLP VOA**

[3] *Analyses are normally only conducted for analytes with a TCLP regulatory criteria.*

General analyses pre-printed on the form:

BOD Biochemical Oxygen Demand **NO_2+NO_3** Nitrite plus Nitrate **Oil & Grease** **TDS** Total Dissolved Solids **TSS** Total Suspended Solids

General analyses that can be written in:

Acidity **Alk** Alkalinity **TNH3** Ammonia **HCO_3** Bicarbonate **Br** Bromide **CO_3** Carbonate **COD** Chemical Oxygen Demand **Cl** Chloride **Color** Color **Cond** Conductivity **CN** Cyanide **CN-W&D** Cyanide, weak & dissociable **Flash** Flash Point **F** Fluoride **Grn Siz** Grain Size **Hard** Hardness **NO_2** Nitrite **NO_3** Nitrate **TNVS** Non-Volatile Solids **NVSS** Non-Volatile Suspended Solids **CLO_4** Perchlorate **pH** **Phenol** Phenolics **SiO_3** Silica - dissolved **SO_4** Sulfate **S** Sulfide **TOC** Total Organic Carbon **TS** Total Solids **% V Slds** % Volatile Solids **TVS** Volatile Solids **TVSS** Volatile Suspended Solids **SetSlds** Settleable Solids **% Tot** % Total Solids **TKN** Total Kjeldahl Nitrogen **T-Phos** Total Phosphorous **D-Phos** Dissolved Phosphorous **O-Phos** Ortho Phosphrous **D-O-Phos** Dissolved Ortho Phosphrous **Turb** Turbidity

Container guidance.

Note: this is general information only - consult the QA Project Plan on appropriate containers and preservatives for each project. Modifying methods may require modifying the number/type of containers. Freezing samples for one or more analyses may require collection of individual containers. Contact the laboratory for minimum sample volumes in situations where sample material is limited. Minimum volumes required for analysis will depend on the analysis and required reporting limits.

Containers for soil/sediment:

Metals/cyanide/mercury: 1, wide mouth 8 ounce glass or HDPE.
Extractable organics: 1, 8 ounce wide mouth amber glass, for one or two analyte groups
Inorganics and organics: 1, sixteen ounce wide mouth amber glass.
VOAs/purgeables: Contact the laboratory for the proper number/type of special Closed-System sample containers.

Containers/chemical preservatives for water[4]:

Metals/regular mercury: 1, one liter HDPE, HNO3 to pH<2
Mercury by method 1631: HCl and 250 mL containers provided by MEL
Cyanide: 1, 250 mL or larger HDPE, remove sulfides and/or residual chlorine then add NaOH to pH>12
Extractable organics (BNA, Pest, PCP, PAH etc.): two, one liter amber glass containers **for each analysis** - if more than one liter will be extracted for the project, it is advisable that the container size match (but not exceed) the volume to be extracted. Two separate volumes are usually collected for each analysis to allow for re-extraction if needed.
VOAs/purgeables: 3, zero headspace 40 mL amber glass vials with Teflon Septa, remove residual chlorine then add HCl to pH<2
Alkalinity: 1, 250 mL or larger HDPE, no extra volume for lab QC
Ammonia: 1, 250 mL or larger HDPE, H2SO4 to pH<2, no extra volume for lab QC
BOD 5: 1, one gallon HDPE, no extra volume for lab QC
TSS: 1, one liter or larger HDPE, no extra volume for lab QC
TDS: 1, 250 mL or larger HDPE, no extra volume for lab QC
Oil & Grease: 1, one liter clear glass, HCl to pH<2, submit 4 **separate** containers for the lab QC sample
NO2+NO3: 1, 250 mL or larger HDPE, H2SO4 to pH<2, no extra volume for lab QC
Br, Cl, F, SO4, CLO4: for analysis by ion chromatography, 1, 100 mL or larger HDPE, no extra volume for lab QC

[4] *Water samples to be designated for lab QC should have double volume submitted for metals, triple volume for organics. In general, extra volume is usually not required for lab QC for soil/sediment.*

APPENDIX F

STATEMENTS OF WORK FOR ASBESTOS ANALYSIS

STATEMENT OF WORK TEMPLATE
ASBESTOS ANALYTICAL SERVICES FOR AIR

Technical Representative: Julie Wroble

Date of Request: TBD

1. General information or description of analytical service requested:

This request is for Service #: 1

Service 1: Analysis of Air Samples for All ISO 10312 Asbestos Structures by Transmission Electron Microscopy (TEM) at 20,000x Magnification with a modified aspect ratio of 3:1.

Service 2: Analysis of Air Samples for PCMe Asbestos Structures Only by Transmission Electron Microscopy (TEM) at Low (at least 5000x) Magnification. PCMe Asbestos Structures are defined in Attachment 3, which modifies the PCMe language in ISO 10312.

Service 3: Analysis of Air Samples for All ISO 10312 Asbestos Structures by Transmission Electron Microscopy (TEM) at 20,000x Magnification with a modified aspect ratio of 3:1 to establish fiber size distribution, then switching to reading of PCMe Asbestos Structures Only by Transmission Electron Microscopy (TEM) at Low (at least 5000x) Magnification. PCMe Asbestos Structures are defined in Attachment 3, which modifies the PCMe language in ISO 10312.

2. Number, type of work units, and expected concentrations: Concentrations are unknown.

Item 1: Analysis of Air Samples for Asbestos to 0.003 s/cc sensitivity
Item 2: Analysis of Air Samples for Asbestos to 0.0003 s/cc sensitivity
Item 3: Analysis of FBAS samples for Asbestos to 6.3E+03 s/g sensitivity

<u>Item 1</u>
Base Quantity: 15
Option Quantity 1: 2
Option Quantity 2: 5

<u>Item 2</u>
Base Quantity: 16
Option Quantity 1: 1
Option Quantity 2: 5

Item 3

Base Quantity: 46

Option Quantity 1: 3

Option Quantity 2: 5

3. Supplies: Does this request require the laboratory to provide the supplies, consisting of 0.8 micrometer pore size Mixed Cellulose Ester (MCE) filter cassettes?

☐ Yes – include filters in pricing   X No – do not include filters in pricing

4. Program or Division: Region 10 Office of Environmental Assessment

5. Estimated date(s) of collection (base quantities): September 29 – October 3, 2014

6. Estimated date(s) of shipment (base quantities): October 3, 2014

7. Name of sampling/shipping contacts: Julie Wroble, Jed Januch

8. Number of days that data, including all supporting data in Attachment 1, is required after receipt of samples: 90

9. Analytical protocols required:

Method ISO 10312:1995 "Ambient air – Determination of asbestos fibres – Direct transfer transmission electron microscopy method". (See note.)

> Note: ISO method is copyrighted, so copies cannot be provided with this solicitation. If a method is needed, it may be purchased on-line at the following addresses:
>
> ISO: **http://webstore.ansi.org/ansidocstore/iso.asp**
>
> It should be noted that offerors will be expected to provide pre-award materials substantiating prior experience with the methods, such that qualified offerors are expected to already have copies of the methods. See Section 13 of this request and Section I of Attachment 1.

Special technical instructions:

a. Filter Overload, Low Volume Backup Filters, and Indirect Analysis:

Filter overload will be set at 25%. This is a modification to ISO 10312, Section 9.5.b.

a.1. Use of low volume backup filter – billable as one unit: Low volume filters may be collected as backup for some sample locations. The low volume filter is only analyzed if the matching high volume filter is overloaded. For each filter overloaded where a low volume backup filter is available, the laboratory will document the overload issue in the case narrative, substitute the low volume filter, and proceed with analysis. **In this case, only the one low volume filter analysis is billable.**

a.2. Indirect analysis – billable as two units: If the low volume filter is not available or is also overloaded, the Contractor shall contact the Technical Representative before proceeding. The Technical Representative will then direct the Contractor to either discard the impacted filter (non-billable) or proceed with the indirect method ISO 13794. The ISO 13794 method can also be obtained at the ISO web link above. **In this case, the analysis will constitute two billable units to account for the preparation of the high and/or low filters plus the preparation and analysis by the indirect method.**

b. Grids Required:

As required by ISO 10312, the laboratory shall prepare and analyze at least two separate grid preparations from a single air filter.

c. Grid Openings Required:

The laboratory shall read a minimum of 10 grid openings, which are compiled of grid openings from both grid preparations. (The number grid openings read per preparation is at lab discretion provided each grid has at least one grid opening read.) The minimum size of each of the 10 grid openings is 0.0057 mm^2. After 10 grid openings have been read, continue to count structures until the required analytical sensitivity has been reached, based on the sample volume and the number of grid openings counted. The count may be terminated upon completion of the grid opening containing the 100th structure, regardless of whether or not the target analytical sensitivity has been reached. (See Section 9 below for estimated grid openings based on analytical sensitivity.)

d. Counting/recording:

Each individual Asbestos structure shall be fully documented on the National Asbestos Data Entry Spreadsheet (NADES) spreadsheet in accordance with Attachment 2 (Spreadsheet Template including Data Entry Instructions). ISO 10312, Section 3.22 is modified to change the aspect ratio from “equal to or greater than 5:1” to “equal to or greater than 3:1”.

e. Mineral identification procedures:

Include mineral identification for all chrysotile and asbestiform amphibole structures meeting the size criteria including fibers that the analyst believes could potentially be cleavage fragments, and transition fibers. If a structure, traditionally defined as a “cleavage fragment” (structure which may not meet the approximately parallel sides cited in the ISO 10312 & 13794 methods), is ≥ 0.5µm in length and has an aspect ratio ≥ 3:1, it shall be counted as a fiber. The fiber shall

be included in the ISO structure tally and a note placed in the comments column that it might be considered a cleavage fragment.

The mineral species to be recorded are: Actinolite, Amosite, Anthophyllite, Chrysotile, Crocidolite, Tremolite, WRTA (Winchite/Richterite/Tremolite/Actinolite), Other Amphibole, Solid Solution Series (Amosite, Cummingtonite-Grunerite), Solid Solution Series (Tremolite-Actinolite).

Energy Dispersive X-ray Analysis (EDXA) and Selected Area Electron Diffraction (SAED) analysis are required to specifically identify structure/fiber minerals. The level of documentation needed for this order is Option #: 2

Option 1: Provide a photograph or sketch of each structure recorded in NADES. Additionally, perform, record, and provide documentation for EDXA and SAED for each structure recorded in NADES.

Option 2: For each sample, provide a photograph or sketch of a minimum of one structure of each type recorded in NADES. Additionally, perform, record, and provide documentation for EDXA and SAED for a minimum of one structure of each type recorded in NADES.

f. All photographs, sketches, spectra, and/or diffraction patterns generated must be cross-referenced in Data Entry 2 comments on the NADES spreadsheet, such that a given structure can be matched to its accompanying documentation within the data package. If the EDXA or SAED of a given structure could not be generated as required, the problem encountered must be documented in the case narrative.

10. Sensitivity requirements and pricing on a "per sample" basis:

Pricing for each Item should be a single unit price on a "per sample" basis, not on a "grid openings counted" basis. Instructions below differ based upon the requested sensitivity.

Item/Matrix	Detection Limit*	Sensitivity Limit	Estimated Volume	Estimated # Grid Openings**
Item 1: High Volume Air	0.009 s/cc	0.003 s/cc	480 L	20
Item 1: Low Volume Backup	0.009 s/cc	0.003 s/cc	240 L	40
Item 2: High Volume Air	0.0009 s/cc	0.0003 s/cc	4,000 L	20
Item 3: FBAS Samples	1E+04 s/g	6.3E+03 s/g	NA	50

* Detection limit defined as upper 95% confidence limit of Poisson distribution for a count of 0 structures (equivalent to 2.99 times the sensitivity level)

** Based upon an average grid opening size of 0.013 mm^2, a minimum of 10 grid openings for all samples, and a maximum of 100 grid openings for a low volume backup.

Item 1: For samples requiring 0.003 structures/cc sensitivity, volumes are estimated and the sensitivity must be achieved regardless of volume actually received. "Per sample" pricing should account for the fact that there are potential variations and a lower volume may be received. This includes samples for which the low volume backup must be analyzed in place of an overloaded high volume.

Item 2: For samples requiring 0.0003 structures/cc sensitivity, volumes are estimated and the sensitivity must be achieved so long as at least 1000 L volume is received.

Item 3: Stopping rules for FBAS samples should be 100 grid openings or 50 structures.

11. QC Requirements: QC submitted from the field (lot blanks and field blanks) are billable. All other QC required by the Statement of Work, including the referenced methods, must be performed and results reported to the client. These QC analyses are non-billable. These include, but are not limited to: Re-analysis Same Analyst, Re-analysis Different Analyst, and Preparation Blanks at the frequency below. The Re-analysis Same Analyst and Re-analysis Different Analyst will be performed on an intra-laboratory basis (within the same laboratory). The costs of these efforts shall be incorporated into the unit price for the samples.

Audits Required	Frequency of Collection	Limits
Lot Blanks	2 per Lot	< 10 asbestos structures /mm^2<br>< 0.1 fibers or bundles/mm^2 for >5 µm
Field Blanks	1 per Sampling Event or 1 per 20 samples if Event is >20 samples	<10 asbestos structures/mm^2<br>< 0.1 fibers or bundles/mm^2 for >5 µm
Re-analysis Same Analyst*	1 per Sampling Event or 1 per 20 samples if Event is >20 samples	Confirms mineral type for each structure Exact for ≤10 structures in Grid Opening, 10 % for >10 Structures In Grid Opening
Re-analysis Different Analyst* Event is >20 samples	1 per Sampling Event or 1 per 20 samples if	Confirms mineral type for each structure Exact for ≤10 structures in Grid Opening, 10 % for >10 Structures In Grid Opening
Preparation Blanks**	1 per preparation day	<10 asbestos structures/mm^2<br>< 0.1 fibers or bundles/mm^2 for >5 µm

*The sample with the highest number of countable structures will be used for the re-analysis. A non-detect sample will be used for the re-analysis only when all samples in the event or the group of 20 are non-detect. All of the same grid openings originally counted will be recounted in the re-analysis.

**** Preparation blanks shall be analyzed and results submitted, regardless of whether field sample results are positive. Where both direct and indirect preparations were performed, blanks specific to each of these procedures shall be analyzed and results submitted.**

12. Analytical results required:

a. Electronic Data Deliverable: Analysis documentation shall be provided by the laboratory in the National Asbestos Data Entry Spreadsheet (NADES) format. See Attachment 2. Concentrations will be reported to the sensitivity level, rather than the detection level.

b. Hardcopy and pdf Data Package: All supporting original hardcopy documentation shall be provided as described in Attachment 1. This documentation shall include the original Chain-of-Custody and Airbill documents received by the laboratory. All hardcopy documentation will also be provided as a pdf. A case narrative detailing any analytical problems encountered during routine laboratory operations shall be included with each data package submitted to the client. Concentrations will be reported to the sensitivity level, rather than the detection level. All preparation logs and calibration data for the dates of preparation and analysis and for all instrument(s) used shall be provided <u>with each data package</u>. Calibration data is expected to include: Plasma Asher Calibration (quarterly), Grid Measurement/Calibration (per lot), TEM Log (daily), Daily Microscope Alignment Check, Cu/Al Calibration (daily), Magnification Calibration at 20,000x (monthly), Magnification Calibration at 10,000x (monthly), Camera Constant (monthly), Spot Size (quarterly), Detector Resolution Check – Mn (semi-annual), Na Sensitivity (quarterly), Mg/Si Sensitivity (quarterly), Beam Dose Calibration for Chrysotile (quarterly), k-factors (semi-annual), and copies of spectra or other documentation for the standards used for reference. All quality control analyses performed for the samples shall be provided with each data package.

13. Data deliverables:

a. Each individual project within the order must have a stand-alone data package delivered to the address below. Individual projects will be distinguished by Project Number and not by delivery date of the samples, as multiple projects may deliver samples to the laboratory at the same time.

b. Submit all contract deliverables (hardcopy and CD/writable DVD) to the following mailing address:

Julie Wroble
EPA Region 10
1200 6th Ave., Suite 900, OEA-140
Seattle, WA 98101

c. Submit NADES and pdf contract deliverables (e-mail) to the following e-mail address:

wroble.julie@epa.gov

All deliverables provided by e-mail must also be provided on the CD/writable DVD.

14. Laboratory shall archive all samples, including those prepared for analysis, until retrieved by the client up until a period of 18 months from receipt of the samples. After the 18 month period, the laboratory shall contact the client for disposition instructions which may include, but not be limited to, disposal or return of samples to client or designee under chain of custody.

The costs of preparing and labeling the samples for shipment will be included in the per sample price. However, the client will provide a courier account to cover the cost of the actual shipment. Samples should be returned organized by project and sealed in their original individual envelopes or anti-static bags. Each project of samples should be wrapped in a bubble material or in a material that will cushion or absorb vibrations/impacts and placed into a labeled anti-static bag, which is then wrapped in a bubble material or in a material that will cushion or absorb vibrations/impacts and then placed into a box. The Chain-of-Custody and sample collection information will be included inside of the first box for each project, but not inside of the batch sample bag. (If sample collection information was not provided with the original shipment to the laboratory, the client will provide it to the laboratory at the time of sample return, and it will be placed with the Chain-of-Custody.) That box is then wrapped in a bubble material or in a material that will cushion or absorb vibrations/impacts and placed into an outer box for shipping.

15. **Pre-award requirements: Information which will be requested prior to award as part of the Responsibility Determination.**

Information Needed from the Laboratory Prior to Award

1. Copy of most recent **NVLAP Audit**, **NVLAP Certificate (**National Institute of Standards and Technology National Voluntary Laboratory Accreditation Program) **and Certificate Scope** (for Airborne Asbestos Fiber Analysis) –or- Copy of NELAP Certificate (National Environmental Laboratory Accreditation Program) and Certificate Scope (for Analyte Asbestos)

2. Brief statement describing **experience** with ISO 10312 and ISO 13794. The statement will include control charts or results for inter-laboratory analyses which have been conducted within the preceding 12 months.

3. **Calibration records** for plasma etcher and for each TEM instrument.

4. A copy of the **current laboratory Quality Management Plan, which is usually known as the Quality Manual**. The Quality Manual will be evaluated for the presence or absence of elements described in Section V of Attachment 1.

5. Identify potential conflicts of interest within the last 5 years.

16. For orders placed directly by EPA, invoices must be submitted to the Finance address specified on the order and must contain the order number, as well as the EPA-assigned Project number applicable to the invoice. Invoices included with the data packages cannot be processed for payment.

Attachments (2)

1. General Analytical Services Scope of Work
2. NADES Spreadsheet Template

ATTACHMENT 1

GENERAL ANALYTICAL SERVICES SCOPE OF WORK

BACKGROUND

Analytical services (laboratory analyses) are needed to support investigations, studies, and various projects to collect environmental data. These analytical services are required to gather data which are needed to make decisions about extent of contamination, threats to human health and the environment, or to develop baseline information. In order for the analytical data to support the intended use of the data, the Contractor shall adhere to the required analytical methods and quality control procedures detailed in this Scope of Work.

The Contractor shall furnish the necessary personnel, material, equipment, services and facilities to perform the analyses of environmental samples utilizing approved analytical methods, following strict quality assurance/quality control procedures, and submitting analytical results in a standardized format, as described in this SOW or applicable attachments.

I. Facilities (Equipment/Personnel/Materials Specification)

The Contractor shall have personnel experienced in the preparation and analysis of environmental samples, and shall be experienced in the timely, accurate, and precise analysis of environmental samples as demonstrated by documentation for sample handling, logistics and preparation, methods, procedures, extractions and/or digestions, concentration, standards preparation, instrument repair, automated and/or manual report generation, and quality assurance/quality control.

The Contractor shall have installed and operating, at a minimum, the specified type and number of instruments and apparatus required to perform analyses as specified. The Contractor shall be responsible for all maintenance of this equipment.

The Contractor shall provide personnel, facilities and equipment for performance of sample analyses and data reporting. The sample preparation specialist(s) and analysts assigned to each project shall have experience in the specified preparation technique(s) and analytical procedures. Analysts shall also be experienced in the interpretation of analytical results from environmental samples by the instrumentation required to perform analyses as stated in the attached methods.

The Contractor shall furnish the necessary calibration standards for each project and must have them in-house at the time of sample receipt.

II. Sample Documentation

Because of the nature of the data being collected, the custody of the samples must be traceable from the time of collection until the time of sample disposal. A sample is physical evidence collected from a facility or the environment. Controlling evidence is an essential part of the

hazardous waste investigation effort. To accomplish this, the following sample identification, chain-of-custody (COC), sample receiving, and sample tracking procedures have been established. These procedures shall be followed in all areas of the laboratory where client's samples are prepared and analyzed.

A. Sample Delivery Group and Identification

A Sample Delivery Group (SDG) is a unit within a single sampling event that is used to identify a group of samples upon delivery. An SDG is a group of 20 or fewer field samples within a shipment, received over a period of up to 7 calendar days. Data from all samples in an SDG are due concurrently. A Sample Delivery Group is defined by one of the following, whichever occurs first:

All samples within a sampling event; or

Every set of 20 field samples within a sampling event; or

All samples received within a 7-day calendar period.

Samples may be assigned to Sample Delivery Groups by matrix (i.e., all soil samples in one SDG, all water samples in another), at the discretion of the laboratory.

The Contractor shall have a specified method for maintaining identification of samples throughout the laboratory to assure traceability of samples while in possession of the Contractor. Each sample and sample preparation container shall be labeled with a unique laboratory identifier. The unique laboratory identifier shall be cross-referenced to the sample identification information provided by the sampling organization.

The Contractor shall have and follow written SOPs for receiving and logging in samples as well as the assignment of unique laboratory identifiers, including a description of the method used to assign the unique laboratory identifier and cross reference of the EPA sample number.

The Contractor shall have and follow written SOPs on the assigning of prefixes or suffixes in addition to sample identification numbers (if this procedure is used by the laboratory).

B. Chain-of-Custody Procedures

The Contractor shall have procedures ensuring that EPA sample custody is maintained and documented. A sample is under custody if the following applies:

1. It is in your possession, or
2. It is in your view after being in your possession, or
3. It was in your possession and you locked it up, or

4. It is in a designated secure area (accessible to authorized personnel only).

The Contractor shall have and follow written SOPs for maintaining identification of EPA samples throughout the laboratory.

The Contractor shall have and follow written SOPs describing the method by which the laboratory maintains samples under custody.

C. Sample Receiving Procedures

The Contractor shall designate a sample custodian responsible for receiving all samples.

The Contractor shall designate a representative to receive samples in the event that the sample custodian is not available.

The Contractor shall have the sample custodian or designated representative inspect the condition of the sample bottles on receipt by the laboratory.

The Contractor shall have the sample custodian or designated representative check for the presence or absence of shipping documents accompanying the sample shipment.

The Contractor shall require the sample custodian or designated representative to sign and date all shipping documents accompanying the samples.

The Contractor shall require the sample custodian or designated representative to note and document problems such as absent documents, conflicting information, broken custody seals, and unsatisfactory sample condition (e.g. leaking sample bottles).

The Contractor shall have and follow written SOPs for sample receipt. The SOP shall address, but are not limited to, the following information:

- Presence or absence of COC forms.
- Presence or absence of airbills.
- Presence or absence of traffic reports or packing lists.
- Presence or absence of custody seals on shipping and/or sample containers and their condition.
- Presence or absence of sample tags.
- Sample tag ID numbers.
- Type and condition of shipping container and sample bottles.
- Verification of agreement of or non-agreement of information on shipping documents and/or COC with sample containers.
- Resolution of problems or discrepancies with the sampling organization.
- An explanation of any terms used to describe sample conditions upon receipt (e.g. good, fine, OK, etc.).

D. Sample Storage

The Contractor shall have written SOPs describing all storage areas for samples in the laboratory. The SOPs shall include a list of authorized personnel who have access or keys to secure storage areas. The SOPs shall include instructions for monitoring temperatures in temperature controlled sample storage areas, as well as provide appropriate corrective actions if temperature tolerances are exceeded.

E. Tracking of Sample Analysis

The Contractor shall have written SOPs for tracking work performed on any particular sample. The tracking SOP shall include:

- A description of the documents used to record sample receipt, sample storage, sample transfers, sample analysis and sample disposal.

- A description of the documents used to record calibration and QA/QC laboratory work.

- Examples of document formats and laboratory documents used in the sample receipt, sample storage, sample transfer and sample analysis.

- A narrative step-by-step description of how documents are used to track samples.

III. Analytical Methods

The Contractor shall utilize the methods specified in the order.

IV. Deliverables

All documents produced by the Contractor which are related to the receipt, preparation, and analysis of the samples are considered as deliverables. These records shall be submitted to the client as originals; however, copies will be acceptable where the document is part of a bound laboratory logbook or notebook.

The following are included to specify and emphasize general documentation requirements and are not intended to supersede or change the requirements of the referenced methods. The sample data package shall include:

A. Case Narrative describing the analyses and any unusual problems with the project.

B. Final tabulated results of sample analyses showing analytes, methods used, analyte concentrations, units of quantitation, dates of analysis, sample receipt date, client sample number, laboratory sample number, type of matrix.

C. Instrument raw data and analyst bench records describing dilutions, weighing, sample size prepared, and final volumes such that an independent data reviewer may recreate the calculations.

D. Example calculations of sample results and quantitation limits.

E. All calibration pertaining to the project analysis dates for all instruments used for the project samples or for associated quality control samples.

F. Blank and reanalysis results.

G. A Complete SDG File (CSF) will consist of the following **original** documents in addition to the **original** documents in the Sample Data Package. The contents of the CSF will be consecutively numbered. No copies will be placed in the CSF unless the originals are bound in a logbook that is maintained by Contractor.

1. Contractor shall supply one CSF to the client within the number of calendar days designated.

2. The contents of the CSF are:

2.1 Sample Data Package

2.2 All **original** shipping documents, including, but not limited to, the following documents:

Chain of Custody Record(s)
Airbills
Sample Tags (if present) sealed in plastic bags

2.3 All **original** receiving documents, including, but not limited to, the following documents:

Sample Log-In Sheet
Other receiving forms or copies of receiving logbooks
Cover Sheet for Chain-Of-Custody(s).

2.4 All **original** laboratory records, not submitted elsewhere in the Sample Data Package, of sample transfer, preparation and analysis, including, but not limited to, the following documents:

Original preparation and analysis forms or copies of preparation and analysis logbook pages.
Internal sample and sample extract transfer chain-of-custody records.
Screening records.

All instrument output, including strip charts from screening activities.

2.5 All other **original** Request-specific documents in the possession of the Contractor, including, but not limited to, the following documents:

Telephone contact logs
Copies of personal logbook pages
All handwritten Request-specific notes
Any other Request-specific documents not covered by the above.

Submit all contract deliverables to the address specified in the Statement of Work.

V. <u>QA/QC Requirements</u>

General Requirements:

The Contractor shall follow the quality control (QC) requirements described in each project specific method and Region 10 Request for Analytical Services.

In addition, the project **<u>may</u>** include blind quality control samples. These may consist of blanks and/or spikes. Successful performance on the spike shall be defined as proper identification and quantitation of the target analyte(s) within the established quantitative acceptance windows. Successful performance for the blank shall be defined as no contaminants present that interfere with the analytical integrity of the target analytes.

In the event of unacceptable performance, client shall have the option of rejecting all or part of a data package. Performance as specified by the contract will serve as a basis of acceptance. The Agency shall have 30 days from the receipt of the data package for review and inspection. No payment shall be made for rejected data.

The Contractor shall establish and implement a comprehensive quality assurance (QA) program in order to define the reliability of the analytical results for analyses performed under this SOW. This program shall incorporate the quality control (QC) procedures, any necessary corrective action, and all documentation required during data collection as well as the quality assessment measures performed by management to ensure reliable data production. This program shall include the use of standard reference materials. Reliable sources of standard reference materials include historical NIST materials (no longer available from NIST), UICC materials, or other materials evaluated by a geologist using multiple analytical methods. Such a QA program shall be documented in a written Quality Manual.

The Contractor’s written Quality Manual shall present the policies, organization, objectives, functional guidelines, and specific QA and QC activities designed to achieve the data quality

requirements in this SOW or its attachments. Where applicable, SOPs pertaining to each element listed below shall be included or referenced as part of this Quality Manual. The Contractor's written Quality Manual must be available to the client during any on-site audits and must be submitted to the client upon request.

The Contractor's Quality Manual must describe the procedures which have been implemented to achieve the following:

- Maintain data integrity, validity and usability.

- Ensure that analytical measurement systems are maintained in an acceptable state of accuracy, stability and reproducibility.

- Detect problems through quality control indicators and establish corrective action procedures which keep all analytical processes reliable.

- Document all aspect of the measurement process in order to provide data which are technically sound and legally defensible.

The Contractor's Quality Manual must address the following elements:

A. Organization and Personnel

1. QA Policy and Objectives

2. QA Management

   a. Organization
   b. Assignment of QA and QC Responsibilities
   c. Reporting Relationship between QA and Management
   d. QA Document Control Procedures
   e. QA Program Assessment Procedures

3. Personnel

   a. Resumes
   b. Education and Experience
   c. Training Goals

B. Facilities and Equipment

1. Instrumentation and Backup Alternatives
2. Maintenance Activities and Schedules

C. Document Control

1. Laboratory Notebook Policy
2. Sample Tracking/Custody Procedures
3. Logbook Maintenance and Archiving Procedures
4. Project File Organization, Preparation and Review Process
5. Procedures for Preparation, Review, Revision and Distribution of SOPs
6. Process for Revision of Technical or Documentation Procedures

D. Analytical Methodology

1. Receipt and Review of Analysis Request
2. Calibration Procedure and Frequency
3. Sample Preparation/Extraction Procedures
4. Sample Analysis Procedures
5. Standards Preparation Procedures
6. Decision Processes, Procedures, and Responsibility for Initiation of Corrective Action

E. Data Generation

1. Data Collection Procedures
2. Data Reduction Procedures
3. Data Validation Procedures
4. Data Reporting and Authorization Procedures

F. Quality Control

1. Solvent, Reagent, and Adsorbent Check Analysis
2. Reference Material Analysis
3. Internal Quality Control Checks
4. Determination of QC Acceptance Limit Procedures
5. Determination of Corrective Action Procedures
6. Responsibility Designation

G. Quality Assurance

1. Data Quality Assurance
2. Systems/Internal Audits
3. Performance/External Audits
4. Corrective Action Procedures
5. Quality Assurance Reporting Procedures
6. Responsibility Designation

VI. <u>Document Control Procedures</u>

The Contractor shall provide reports and other deliverables as specified. In addition, the laboratory shall follow document control procedures. The goal of the laboratory document control program is to assure that all documents for a specified project will be accounted for when the project is complete. Accountable documents used by the Contractor shall include, but are not limited to, logbooks, chain-of-custody records, sample work sheets, sample run logs, instrument raw data, bench sheets, sample preparation records and other documents relating to the sample analysis.

Because the Contractor may be required to provide copies of sample analysis documents to the client, the Contractor may exercise the option of using only laboratory identification numbers in the documents rather than clients names to preserve the confidentiality of other clients. The existence of client names in the documents does not excuse the laboratory from providing the required documents to the client. The Contractor shall provide a cross-reference for internal sample identification versus sampling organization's sample identification.

All original documentation not provided to the client with the data package related to the preparation and analysis of the samples shall be kept on file for a minimum of five years. If at the end of the five year period, the Contractor desires to dispose of the original documents, the Contractor should first contact the client for permission to dispose of the documents. If directed by the client, the laboratory shall ship all project documents to the client rather than disposing of the documents.

VII. Sample Handling/Disposal

The Contractor shall be responsible for all handling or processing required for receipt of samples.

Because of the potential hazards associated with the handling and analyses of these samples, the Contractor shall be responsible for taking all necessary measures to ensure the health and safety of its employees.

The Contractor shall dispose of unused sample volume and used sample bottles/containers no earlier than 18 months following complete submission of analytical data, unless otherwise instructed by the client. Sample/extract disposal and disposal of unused sample bottles/containers shall be done pursuant to all applicable laws and regulations governing disposal of such materials. The Contractor shall be responsible for long term sample storage regardless of disposal capacity/availability.

VIII. Standard Operating Procedures

The Contractor shall have and follow written Standard Operating Procedures for all laboratory operations. These procedures are necessary to ensure that analytical data produced under this SOW are of known quality and are defensible. Copies of the Contractor's written SOPs shall be provided to EPA upon request. A Standard Operating Procedure (SOP) is defined as a written step-by-step description of Laboratory operating procedures including examples of laboratory

documents. The SOPs shall accurately describe the actual procedures used by the laboratory, and copies of the written SOPs shall be available to the appropriate laboratory personnel.

The Contractor shall have and follow written SOPs which describe how the following operations are conducted in the laboratory:

1. Preventing sample contamination.

2. Security for laboratory and samples.

3. Standards purity/preparation.

4. Maintaining instrument records and logbooks.

5. Sample analysis and data control systems, i.e., laboratory information management systems (computerized or manual).

6. Glassware cleaning.

7. Technical and managerial review of laboratory operation and data report/data package generation.

8. Internal review of contractually required QA/QC data for each project.

9. Sample analysis, data handling and reporting.

10. Laboratory data validation/laboratory self-inspection system.

    a. Data flow and chain of command for data review.

    b. Procedures for measuring precision and accuracy.

    c. Evaluation parameters for identifying systematic errors.

    d. Demonstration of internal QA inspection procedures (demonstrated by supervisory sign-off on personal notebooks, internal PE samples, etc.).

    e. Frequency and type of internal audits (e.g. random, quarterly, spot checks, perceived trouble areas).

    f. Demonstration of problem identification/corrective action and resumption of analytical processing.

    g. Documentation of audit reports (internal and external), response, corrective action, etc.

11. Data Management and Handing

a. Procedures for controlling and estimating data entry errors.

b. Procedures for reviewing changes to data and deliverables. Procedures for ensuring traceability of updates.

c. Procedures for testing, modifying, and implementing changes to existing computing systems including hardware, software, and installing new systems.

d. Database security, backup, and archival procedures including recovery from system failures.

e. Data system maintenance procedures and response time.

f. Individuals responsible for data system operation, maintenance, data integrity and security.

g. Specification for staff training procedures.

h. List of signatures, initials and typed name of laboratory personnel.

ATTACHMENT 2

INSTRUCTIONS

National Asbestos Data Entry Spreadsheet (NADES) for Air & Dust Analysis by Superfund TEM

This spreadsheet is designed to record the raw fiber counts for air and dust samples analyzed by Superfund TEM

This is NADES version 13-DRAFT

Raw Data Recording

Raw data are to be recorded by the analyst in hard copy using Lab Sheet 1 (Sample) and as many Lab Sheet 2 (Structure) pages as needed.

Electronic Data Entry

Data on the hard copy lab sheets are to be entered electronically on Data Entry 1 and 2

Areas for data entry are highlighted in **YELLOW**

OR are indicated by a **PULL-DOWN MENU**

Cells that are shaded gray do not require any data input

Cells that are shaded red either require data input or contain an apparent data inconsistency

Do not enter data in any other location!

Avoid drag & drop and cut & paste methods of data entry.

Enter all values individually

File Save

After entering all data on "Data Entry 1" (or any time thereafter), SAVE THE FILE by clicking on the macro button located on "Data Entry 1" or "Data Entry 2".

The file name is generated automatically by concatinating information provided in Data Entry 1, including:

a) Site/Project Identifier Code, b) Lab Name, c) Field Sample ID, d) Analysis Date, e) Lab Job ID f) Prep method, and g) QA Type.

Note that the directory where the macro will save the file depends on how the template file is opened:

If you open EXCEL and then open this spreadsheet, the new file will be saved in the same directory as the spreadsheet.

If you open this spreadsheet from Windows Explorer, then the file will be saved in you default directory for Excel (usually this is C:\Documents and Settings\My Documents).

STATEMENT OF WORK TEMPLATE
ASBESTOS ANALYTICAL SERVICES FOR SOIL/BULK

Technical Representative: Julie Wroble

Date of Request: TBD

1. General information or description of analytical service requested:
Analysis of Soil for Asbestos

Item 1: Analysis of Soil/Bulk Samples for Asbestos by PLM
Item 2: Analysis of Soil/Bulk for Asbestos by PLM/TEM

2. Number, type of work units, and expected concentrations: Concentrations are unknown.

Item 1 (Soil/Bulk):
Base Quantity: 46
Option Quantity 1: 3
Option Quantity 2:

Items 2 (Soil/Bulk):
Base Quantity: 46
Option Quantity 1: 3
Option Quantity 2:

3. Program or Division: Region 10 Office of Environmental Assessment

4. Estimated date(s) of collection (base quantities): 9/29/14

5. Estimated date(s) of shipment (base quantities): 10/3/14

6. Name of sampling/shipping contacts: Julie Wroble, Jed Januch

7. Number of days data, including all supporting data in Attachment 1, is required after receipt of samples: 90

8. <u>Analytical protocols required:</u>

Item 1 (Asbestos): CARB 435
The CARB Method may be found at http://www.epa.gov/ttn/emc/ctm.html. This method is modified as described in Section 8 below.

Item 2 (Asbestos): ASTM D7521-13
Standards are available for purchase at www.astm.org.

Special technical instructions for Item 1:

a. Applicable Sections of CARB 435:
The CARB 435 method covers tasks which are not required under this Statement of Work. The method is modified for this Statement of Work as follows: Sections 1, 2, 7, 8, and 9 will apply to this Statement of Work. Sections 3-6 and 10 will not apply to this Statement of Work.

b. Modification to CARB 435 for Particle Size:
Samples are transported to the laboratory by EPA and then processed by the laboratory in spaces with controlled air flows. All drying, pulverizing, and sieving is to be performed in a fume hood or within glove boxes. Before crushing, the samples must be adequately dried using convection oven or muffle furnace. (If required, the sample shall be crushed to produce a material with a nominal size of less than three-eighths of an inch. ASTM method C-702-80, which is incorporated herein by reference, shall be used to reduce the crush sample to a one pint aliquot. It is unlikely that the samples for this order will require this step.)

The sample shall then be crushed using milling to produce a material with particle size for analysis of about 250 um in size. The material should pass through the 250 um (60 mesh) and be mostly retained on the 75 um (200 mesh).

c. Modification to CARB 435 for Counting:
The laboratory will perform Option #: 2

Option 1: Both (1) point counting and (2) visual estimate.
The point counting will be performed first. If structures are quantitatively detected by point count, then the visual estimate is not required. For samples where structures are visible but do not cross a point, the point count result is reported as "Trace" and is not quantitative. If non-detect or "Trace" by point count, the laboratory will proceed with visual estimate, in addition to the point count. Two separate results for point count and visual estimate results will be reported. The laboratory will perform the visual estimate according to EPA/600/R-93/116, July 1993, which indicates that "Visual Area Estimation is a semi-quantitative method that must relate back to calibration materials." The laboratory will also perform the visual estimate according to EPA/600/R-93/116, July 1993 instructions as follows: "Visual area estimates should be made by comparison of the sample to calibration materials that have similar textures and fiber abundance (see Section 2.1.6 and Appendix C). A minimum of three slide mounts should be examined to determine the asbestos content by visual area estimation. Each slide should be scanned in its entirety and the relative proportions of asbestos and non-asbestos noted. It is suggested that the ratio of asbestos to non-asbestos material be recorded for several fields for each slide and the results be compared to data derived from the analysis of calibration materials having similar textures and asbestos content." If the visual estimate is detected at

less than the lowest calibration standard available, the result is reported as "Trace". The lowest calibration standard available must be no more than 1%.

Option 2: Both (1) point counting and (2) field of view.
The point counting will be performed first. If structures are quantitatively detected by point count, then the field of view is not required. For samples where structures are visible but do not cross a point, the point count result is reported as "Trace" and is not quantitative. If non-detect or "Trace" by point count, the laboratory will proceed with field of view, in addition to the point count. Two separate results for point count and field of view results will be reported. The field of view will consist of reporting a specific count of fibers observed and documenting the number of fields of view observed to determine this count. For example, the analyst will report "x fibers observed in y fields of view". Two separate results should be reported. All the fields of view used for the point count will also be used to count the number of structures for the field of view result.

Special technical instructions for Item 2:

a. Applicable Sections of ASTM D7521-13: NA

9. Sensitivity requirement for Item 1: 0.25%
Sensitivity requirement for Item 2: 0.1%

10. QC Requirements: QC submitted from the field (field duplicates) are billable. All other QC required by the Statement of Work, including the referenced methods, must be performed and results reported to the client. These QC analyses are non-billable. These include, but are not limited to, Re-analysis Same Analyst, Re-analysis Different Analyst, Laboratory Blanks, and Reference Sample Analysis at the frequency below. The Re-analysis Same Analyst and Re-analysis Different Analyst will be performed on an intra-laboratory basis (within the same laboratory). The costs of these efforts shall be incorporated into the unit price for the samples.

Audits Required	Frequency of Collection	Limits
Re-analysis Same Analyst	1 per sampling event or 1 per 10 samples if Event is >10 samples	Confirms presence/absence Confirms predominant mineral type
Re-analysis Different Analyst	1 per sampling event or 1 per 10 samples if Event is >10 samples	Confirms presence/absence Confirms predominant mineral type
Laboratory Blanks*	Before analysis of each set of samples	Confirms absence of Asbestos
Reference Sample Analysis	1 per sampling event	Confirms predominant mineral type

***** A sample of isotropic non-asbestos material such as fiberglass (SRM 1866a) should be mounted in a drop of refractive index liquid on a clean slide. Preparation tools including forceps and dissecting needle should be rubbed in the drop of liquid and a clean cover slip should be placed on the drop. The entire area under the cover slip should be scanned by PLM to detect asbestos fiber contamination. If asbestos fibers are detected, the test should be repeated using a clean slide and cleaned preparation tools. If asbestos fibers are still found, have the refractive index fluid checked and replace if needed.

11. Analytical results required:

a. Electronic Data Deliverable:

For Item 1, analysis documentation shall be provided by the laboratory in the National Asbestos Data Entry Spreadsheet (NADES) format. See Attachment 2.

For Item 2, analysis documentation shall be provided by the laboratory in a format consistent with NADES.

b. Hardcopy and pdf Data Package: All supporting original hardcopy documentation shall be provided as described in Attachment 1. This documentation shall include the original Chain-of-Custody and Airbill documents received by the laboratory. All hardcopy documentation will also be provided as a pdf. A case narrative detailing any analytical problems encountered during routine laboratory operations shall be included with each data package submitted to the client. All preparation logs and calibration data for the dates of preparation and analysis and for all instrument(s) used shall be provided with each data package. Calibration data is expected to include: Microscope Alignment Checks (daily), Reference Analysis (1 per project), Refractive Index Liquids (semi-annual), Laboratory Temperature monitored during calibration and general use of Refractive Index Liquids, Laboratory Oven Temperature (Moisture Content), and copies of spectra or other documentation for the standards used for reference. All quality control analyses performed for the samples shall be provided with each data package.

12. Data deliverables:

a. Each individual project within the order must have a stand-alone data package delivered to the address below. Individual projects will be distinguished by Project Number and not by delivery date of the samples, as multiple projects may deliver samples to the laboratory at the same time.

b. Submit all contract deliverables (hardcopy and CD/writable DVD) to the following mailing address:

Julie Wroble
US EPA Region 10
1200 6th Ave., Suite 900, OEA-140
Seattle, WA 98101

Submit NADES and pdf contract deliverables (e-mail) to the following e-mail address:

wroble.julie@epa.gov

All deliverables provided by e-mail must also be provided on the CD/writable DVD.

13. Laboratory shall archive all samples, including those prepared for analysis, until retrieved by the client up until a period of 18 months from receipt of the samples. After the 18 month period, the laboratory shall contact the client for disposition instructions which may include, but not be limited to, disposal or return of samples to client or designee under chain of custody. The costs of preparing and labeling the samples for shipment will be included in the per sample price. However, the client will provide a courier account to cover the cost of the actual shipment.

14. **Pre-award requirements: Information which will be requested prior to award as part of the Responsibility Determination.**

Information Needed from the Laboratory Prior to Award

1. Copy of most recent **NVLAP Audit**, **NVLAP Certificate (**National Institute of Standards and Technology National Voluntary Laboratory Accreditation Program) **and Certificate Scope** (Asbestos Fiber Analysis –PLM Test Method).

2. Brief statement describing **experience** with Polarized Light Microscopy by CARB 435 and ASTM D5755. The statement will include control charts or results for inter-laboratory analyses which have been conducted within the preceding 12 months.

3**.** A copy of the **current laboratory Quality Management Plan, which is usually known as the Quality Manual**. The Quality Manual will be evaluated for the presence or absence of elements described in Section V of Attachment 1.

4. Identify potential conflicts of interest within the last 5 years.

15. For orders placed directly by EPA, invoices must be submitted to the Finance address specified on the order and must contain the order number, as well as the EPA-assigned Project number applicable to the invoice. Invoices included with the data packages cannot be processed for payment.

Attachments (2):
1. General Analytical Services Scope of Work
2. NADES Spreadsheet Template

ATTACHMENT 1

GENERAL ANALYTICAL SERVICES SCOPE OF WORK

BACKGROUND

Analytical services (laboratory analyses) are needed to support investigations, studies, and various projects to collect environmental data. These analytical services are required to gather data which are needed to make decisions about extent of contamination, threats to human health and the environment, or to develop baseline information. In order for the analytical data to support the intended use of the data, the Contractor shall adhere to the required analytical methods and quality control procedures detailed in this Scope of Work.

The Contractor shall furnish the necessary personnel, material, equipment, services and facilities to perform the analyses of environmental samples utilizing approved analytical methods, following strict quality assurance/quality control procedures, and submitting analytical results in a standardized format, as described in this SOW or applicable attachments.

I. Facilities (Equipment/Personnel/Materials Specification)

The Contractor shall have personnel experienced in the preparation and analysis of environmental samples, and shall be experienced in the timely, accurate, and precise analysis of environmental samples as demonstrated by documentation for sample handling, logistics and preparation, methods, procedures, extractions and/or digestions, concentration, standards preparation, instrument repair, automated and/or manual report generation, and quality assurance/quality control.

The Contractor shall have installed and operating, at a minimum, the specified type and number of instruments and apparatus required to perform analyses as specified. The Contractor shall be responsible for all maintenance of this equipment.

The Contractor shall provide personnel, facilities and equipment for performance of sample analyses and data reporting. The sample preparation specialist(s) and analysts assigned to each project shall have experience in the specified preparation technique(s) and analytical procedures. Analysts shall also be experienced in the interpretation of analytical results from environmental samples by the instrumentation required to perform analyses as stated in the attached methods.

The Contractor shall furnish the necessary calibration standards for each project and must have them in-house at the time of sample receipt.

II. Sample Documentation

Because of the nature of the data being collected, the custody of the samples must be traceable from the time of collection until the time of sample disposal. A sample is physical evidence collected from a facility or the environment. Controlling evidence is an essential part of the

hazardous waste investigation effort. To accomplish this, the following sample identification, chain-of-custody (COC), sample receiving, and sample tracking procedures have been established. These procedures shall be followed in all areas of the laboratory where client's samples are prepared and analyzed.

A. Sample Delivery Group and Identification

A Sample Delivery Group (SDG) is a unit within a single sampling event that is used to identify a group of samples upon delivery. An SDG is a group of 20 or fewer field samples within a shipment, received over a period of up to 7 calendar days. Data from all samples in an SDG are due concurrently. A Sample Delivery Group is defined by one of the following, whichever occurs first:

All samples within a sampling event; or

Every set of 20 field samples within a sampling event; or

All samples received within a 7-day calendar period.

Samples may be assigned to Sample Delivery Groups by matrix (i.e., all soil samples in one SDG, all water samples in another), at the discretion of the laboratory.

The Contractor shall have a specified method for maintaining identification of samples throughout the laboratory to assure traceability of samples while in possession of the Contractor. Each sample and sample preparation container shall be labeled with a unique laboratory identifier. The unique laboratory identifier shall be cross-referenced to the sample identification information provided by the sampling organization.

The Contractor shall have and follow written SOPs for receiving and logging in samples as well as the assignment of unique laboratory identifiers, including a description of the method used to assign the unique laboratory identifier and cross reference of the client sample number.

The Contractor shall have and follow written SOPs on the assigning of prefixes or suffixes in addition to sample identification numbers (if this procedure is used by the laboratory).

B. Chain-of-Custody Procedures

The Contractor shall have procedures ensuring that EPA sample custody is maintained and documented. A sample is under custody if the following applies:

1. It is in your possession, or
2. It is in your view after being in your possession, or
3. It was in your possession and you locked it up, or

4. It is in a designated secure area (accessible to authorized personnel only).

The Contractor shall have and follow written SOPs for maintaining identification of client samples throughout the laboratory.

The Contractor shall have and follow written SOPs describing the method by which the laboratory maintains samples under custody.

C. <u>Sample Receiving Procedures</u>

The Contractor shall designate a sample custodian responsible for receiving all samples.

The Contractor shall designate a representative to receive samples in the event that the sample custodian is not available.

The Contractor shall have the sample custodian or designated representative inspect the condition of the sample bottles on receipt by the laboratory.

The Contractor shall have the sample custodian or designated representative check for the presence or absence of shipping documents accompanying the sample shipment.

The Contractor shall require the sample custodian or designated representative to sign and date all shipping documents accompanying the samples.

The Contractor shall require the sample custodian or designated representative to note and document problems such as absent documents, conflicting information, broken custody seals, and unsatisfactory sample condition (e.g. leaking sample bottles).

The Contractor shall have and follow written SOPs for sample receipt. The SOP shall address, but are not limited to, the following information:

- Presence or absence of COC forms.
- Presence or absence of airbills.
- Presence or absence of traffic reports or packing lists.
- Presence or absence of custody seals on shipping and/or sample containers and their condition.
- Presence or absence of sample tags.
- Sample tag ID numbers.
- Type and condition of shipping container and sample containers.
- Verification of agreement of or non-agreement of information on shipping documents and/or COC with sample containers.
- Resolution of problems or discrepancies with the sampling organization.
- An explanation of any terms used to describe sample conditions upon receipt (e.g. good, fine, OK, etc.).

D. <u>Sample Storage</u>

The Contractor shall have written SOPs describing all storage areas for samples in the laboratory. The SOPs shall include a list of authorized personnel who have access or keys to secure storage areas. The SOPs shall include instructions for monitoring temperatures in temperature controlled sample storage areas, as well as provide appropriate corrective actions if temperature tolerances are exceeded.

E. Tracking of Sample Analysis

The Contractor shall have written SOPs for tracking work performed on any particular sample. The tracking SOP shall include:

- A description of the documents used to record sample receipt, sample storage, sample transfers, sample analysis and sample disposal.

- A description of the documents used to record calibration and QA/QC laboratory work.

- Examples of document formats and laboratory documents used in the sample receipt, sample storage, sample transfer and sample analysis.

- A narrative step-by-step description of how documents are used to track samples.

III. Analytical Methods

The Contractor shall utilize the methods specified in the order.

IV. Deliverables

All documents produced by the Contractor which are related to the receipt, preparation, and analysis of the samples are considered as deliverables. These records shall be submitted to the client as originals; however, copies will be acceptable where the document is part of a bound laboratory logbook or notebook.

The following are included to specify and emphasize general documentation requirements and are not intended to supersede or change the requirements of the referenced methods. The sample data package shall include:

A. Case Narrative describing the analyses and any unusual problems with the project.

B. Final tabulated results of sample analyses showing analytes, methods used, analyte concentrations, units of quantitation, dates of analysis, sample receipt date, client sample number, laboratory sample number, and type of matrix.

C. Instrument raw data and analyst bench records describing weighing, sample size prepared, and final volumes, such that an independent data reviewer may recreate the calculations.

D. Example calculations of sample results and quantitation limits.

E. All calibration pertaining to the project analysis dates for all instruments used for the project samples or for associated quality control samples.

F. Blank and re-analysis results.

G. A Complete SDG File (CSF) will consist of the following **original** documents in addition to the **original** documents in the Sample Data Package. The contents of the CSF will be consecutively numbered. No copies will be placed in the CSF unless the originals are bound in a logbook that is maintained by Contractor.

1. Contractor shall supply one CSF to the client within the number of calendar days designated.

2. The contents of the CSF are:

2.1 Sample Data Package

2.2 All **original** shipping documents, including, but not limited to, the following documents:

Chain of Custody Record(s)
Airbills
Sample Tags (if present) sealed in plastic bags

2.3 All **original** receiving documents, including, but not limited to, the following documents:

Sample Log-In Sheet
Other receiving forms or copies of receiving logbooks
Cover Sheet for Chain-Of-Custody(s).

2.4 All **original** laboratory records, not submitted elsewhere in the Sample Data Package, of sample transfer, preparation and analysis, including, but not limited to, the following documents:

Original preparation and analysis forms or copies of preparation and analysis logbook pages.
Internal sample transfer chain-of-custody records.
Screening records.

All instrument output, including those from screening activities.

2.5 All other **original** Request-specific documents in the possession of the Contractor, including, but not limited to, the following documents:

Telephone contact logs
Copies of personal logbook pages
All handwritten Request-specific notes
Any other Request-specific documents not covered by the above.

Submit all contract deliverables to the address specified in the Statement of Work.

V. <u>QA/QC Requirements</u>

General Requirements:

The Contractor shall follow the quality control (QC) requirements described in the Statement of Work, including the referenced specific methods.

In addition, the project **<u>may</u>** include blind quality control (Performance Evaluation) samples. These may consist of blanks and/or spikes. Successful performance on the spike shall be defined as proper identification and quantitation of the target analyte(s) within the established quantitative acceptance windows. Successful performance for the blank shall be defined as no contaminants present that interfere with the analytical integrity of the target analytes.

In the event of unacceptable performance, client shall have the option of rejecting all or part of a data package. Performance as specified by the contract will serve as a basis of acceptance. The client shall have 30 days from the receipt of the data package for review and inspection. No payment shall be made for rejected data.

The Contractor shall establish and implement a comprehensive quality assurance (QA) program in order to define the reliability of the analytical results for analyses performed under this SOW. This program shall incorporate the quality control (QC) procedures, any necessary corrective action, and all documentation required during data collection as well as the quality assessment measures performed by management to ensure reliable data production. This program shall include the use of standard reference materials. Reliable sources of standard reference materials include historical NIST materials (no longer available from NIST), UICC materials, or other materials evaluated by a geologist using multiple analytical methods. Such a QA program shall be documented in a written Quality Manual.

The Contractor’s written Quality Manual shall present the policies, organization, objectives, functional guidelines, and specific QA and QC activities designed to achieve the data quality

requirements in this SOW or its attachments. Where applicable, SOPs pertaining to each element listed below shall be included or referenced as part of this Quality Manual. The Contractor's written Quality Manual must be available to the client during any on-site audits and must be submitted to the client upon request.

The Contractor's Quality Manual must describe the procedures which have been implemented to achieve the following:

- Maintain data integrity, validity and usability.

- Ensure that analytical measurement systems are maintained in an acceptable state of accuracy, stability and reproducibility.

- Detect problems through quality control indicators and establish corrective action procedures which keep all analytical processes reliable.

- Document all aspect of the measurement process in order to provide data which are technically sound and legally defensible.

The Contractor's Quality Manual must address the following elements:

A. Organization and Personnel

1. QA Policy and Objectives

2. QA Management

    a. Organization
    b. Assignment of QA and QC Responsibilities
    c. Reporting Relationship between QA and Management
    d. QA Document Control Procedures
    e. QA Program Assessment Procedures

3. Personnel

    a. Resumes
    b. Education and Experience
    c. Training Goals

B. Facilities and Equipment

1. Instrumentation and Backup Alternatives
2. Maintenance Activities and Schedules

C. Document Control

1. Laboratory Notebook Policy
2. Sample Tracking/Custody Procedures
3. Logbook Maintenance and Archiving Procedures
4. Project File Organization, Preparation and Review Process
5. Procedures for Preparation, Review, Revision and Distribution of SOPs
6. Process for Revision of Technical or Documentation Procedures

D. Analytical Methodology

1. Receipt and Review of Analysis Request
2. Calibration Procedure and Frequency
3. Sample Preparation/Extraction Procedures
4. Sample Analysis Procedures
5. Standards Preparation Procedures
6. Decision Processes, Procedures, and Responsibility for Initiation of Corrective Action

E. Data Generation

1. Data Collection Procedures
2. Data Reduction Procedures
3. Data Validation Procedures
4. Data Reporting and Authorization Procedures

F. Quality Control

1. Solvent, Reagent, and Adsorbent Check Analysis
2. Reference Material Analysis
3. Internal Quality Control Checks
4. Determination of QC Acceptance Limit Procedures
5. Determination of Corrective Action Procedures
6. Responsibility Designation

G. Quality Assurance

1. Data Quality Assurance
2. Systems/Internal Audits
3. Performance/External Audits
4. Corrective Action Procedures
5. Quality Assurance Reporting Procedures
6. Responsibility Designation

VI. <u>Document Control Procedures</u>

The Contractor shall provide reports and other deliverables as specified. In addition, the laboratory shall follow document control procedures. The goal of the laboratory document control program is to assure that all documents for a specified project will be accounted for when the project is complete. Accountable documents used by The Contractor shall include, but are not limited to, logbooks, chain-of-custody records, sample work sheets, sample run logs, instrument raw data, bench sheets, sample preparation records and other documents relating to the sample analysis.

Because the Contractor may be required to provide copies of sample analysis documents to the client, the Contractor may exercise the option of using only laboratory identification numbers in the documents rather than clients names to preserve the confidentiality of other clients. The existence of other client names in the documents does not excuse the laboratory from providing the required documents. The Contractor shall provide a cross-reference for internal sample identification versus sampling organization's sample identification.

All original documentation not provided to the client with the data package related to the preparation and analysis of the samples shall be kept on file for a minimum of five years. If at the end of the five year period, the Contractor desires to dispose of the original documents, the Contractor should first contact the client for permission to dispose of the documents. If directed by the client, the laboratory shall ship all project documents to the client rather than disposing of the documents.

VII. Sample Handling/Disposal

The Contractor shall be responsible for all handling or processing required for receipt of samples.

Because of the potential hazards associated with the handling and analyses of these samples, the Contractor shall be responsible for taking all necessary measures to ensure the health and safety of its employees.

The Contractor shall dispose of unused sample volume and used sample bottles/containers no earlier than 18 months following complete submission of analytical data, unless otherwise instructed by the client. Sample/extract disposal and disposal of unused sample bottles/containers shall be done pursuant to all applicable laws and regulations governing disposal of such materials. The Contractor shall be responsible for long term sample storage regardless of disposal capacity/availability.

VIII. Standard Operating Procedures

The Contractor shall have and follow written Standard Operating Procedures for all laboratory operations. These procedures are necessary to ensure that analytical data produced under this SOW are of known quality and are defensible. Copies of the Contractor's written SOPs shall be provided to the client upon request. A Standard Operating Procedure (SOP) is defined as a written step-by-step description of Laboratory operating procedures including examples of

laboratory documents. The SOPs shall accurately describe the actual procedures used by the laboratory, and copies of the written SOPs shall be available to the appropriate laboratory personnel.

The Contractor shall have and follow written SOPs which describe how the following operations are conducted in the laboratory:

1. Preventing sample contamination.

2. Security for laboratory and samples.

3. Standards purity/preparation.

4. Maintaining instrument records and logbooks.

5. Sample analysis and data control systems, i.e., laboratory information management systems (computerized or manual).

6. Glassware cleaning.

7. Technical and managerial review of laboratory operation and data report/data package generation.

8. Internal review of contractually required QA/QC data for each project.

9. Sample analysis, data handling and reporting.

10. Laboratory data validation/laboratory self-inspection system.

    a. Data flow and chain of command for data review.

    b. Procedures for measuring precision and accuracy.

    c. Evaluation parameters for identifying systematic errors.

    d. Demonstration of internal QA inspection procedures (demonstrated by supervisory sign-off on personal notebooks, internal PE samples, etc.).

    e. Frequency and type of internal audits (e.g. random, quarterly, spot checks, perceived trouble areas).

    f. Demonstration of problem identification/corrective action and resumption of analytical processing.

g. Documentation of audit reports (internal and external), response, corrective action, etc.

11. Data Management and Handing

a. Procedures for controlling and estimating data entry errors.

b. Procedures for reviewing changes to data and deliverables. Procedures for ensuring traceability of updates.

c. Procedures for testing, modifying, and implementing changes to existing computing systems including hardware, software, and installing new systems.

d. Database security, backup, and archival procedures including recovery from system failures.

e. Data system maintenance procedures and response time.

f. Individuals responsible for data system operation, maintenance, data integrity and security.

g. Specification for staff training procedures.

h. List of signatures, initials and typed name of laboratory personnel.

ATTACHMENT 2

INSTRUCTIONS

National Asbestos Data Entry Spreadsheet (NADES) for Bulk & Soil Analysis by PLM

This spreadsheet is designed to record the raw fiber counts for bulk and soil samples analyzed by PLM
This is NADES version 10-DRAFT

Raw Data Recording

Raw data are to be recorded by the analyst in hard copy using VE Lab Sheet for visual area estimation approach and PC Lab Sheet for a point count approach.

Electronic Data Entry

Data on the hard copy lab sheets are to be entered electronically on Visual Data Entry and Point Count Data Entry

Enter data on only one sheet (either VE or PC) DO NOT enter mixed data (some one sheet, some on the other)

Areas for data entry are highlighted in **YELLOW**

OR are indicated by a **PULL-DOWN MENU**

Cells that are shaded gray do not require any data input

Cells that are shaded red either require data input or contain an apparent data inconsistency

Do not enter data in any other location!

Avoid drag & drop and cut & paste methods of data entry.

Enter all values individually

File Save

After entering all data on "General Data Entry" (or any time thereafter), SAVE THE FILE by clicking on the macro button located on "General Data Entry" or "Visual Data Entry1" or "Visual Data Entry2" or "Point Count Data Entry".

The file name is generated automatically by concatinating information provided in General Data Entry, including:

a) Site/Project Identifier Code, b) Lab Name, c) Lab Job Number, d) Analysis Type, and e) Method (VE or PC).

Note that the directory where the macro will save the file depends on how the template file is opened:

If you open EXCEL and then open this spreadsheet, the new file will be saved in the same directory as the spreadsheet.

If you open this spreadsheet from Windows Explorer, then the file will be saved in you default directory for Excel (usually this is C:\Documents and Settings\My Documents)

APPENDIX G

NATIONAL ASBESTOS DATA ENTRY SHEET

INSTRUCTIONS

ATTACHMENT 2

INSTRUCTIONS

National Asbestos Data Entry Spreadsheet (NADES) for Air & Dust Analysis by Superfund TEM

This spreadsheet is designed to record the raw fiber counts for air and dust samples analyzed by Superfund TEM
This is NADES version 13-DRAFT

Raw Data Recording

Raw data are to be recorded by the analyst in hard copy using Lab Sheet 1 (Sample) and as many Lab Sheet 2 (Structure) pages as needed.

Electronic Data Entry

Data on the hard copy lab sheets are to be entered electronically on Data Entry 1 and 2

Areas for data entry are highlighted in **YELLOW**

OR are indicated by a **PULL-DOWN MENU**

Cells that are shaded gray do not require any data input

Cells that are shaded red either require data input or contain an apparent data inconsistency

Do not enter data in any other location!

Avoid drag & drop and cut & paste methods of data entry.
Enter all values individually

File Save

After entering all data on "Data Entry 1" (or any time thereafter), SAVE THE FILE by clicking on the macro button located on "Data Entry 1" or "Data Entry 2".

The file name is generated automatically by concatinating information provided in Data Entry 1, including:

a) Site/Project Identifier Code, b) Lab Name, c) Field Sample ID, d) Analysis Date, e) Lab Job ID f) Prep method, and g) QA Type.

Note that the directory where the macro will save the file depends on how the template file is opened:

If you open EXCEL and then open this spreadsheet, the new file will be saved in the same directory as the spreadsheet.
If you open this spreadsheet from Windows Explorer, then the file will be saved in you default directory for Excel (usually this is C:\Documents and Settings\My Documents)

ATTACHMENT 2

INSTRUCTIONS

National Asbestos Data Entry Spreadsheet (NADES) for Bulk & Soil Analysis by PLM

This spreadsheet is designed to record the raw fiber counts for bulk and soil samples analyzed by PLM

This is NADES version 10-DRAFT

Raw Data Recording

Raw data are to be recorded by the analyst in hard copy using VE Lab Sheet for visual area estimation approach and PC Lab Sheet for a point count approach.

Electronic Data Entry

Data on the hard copy lab sheets are to be entered electronically on Visual Data Entry and Point Count Data Entry

Enter data on only one sheet (either VE or PC) DO NOT enter mixed data (some one sheet, some on the other)

Areas for data entry are highlighted in **YELLOW**

OR are indicated by a **PULL-DOWN MENU**

Cells that are shaded gray do not require any data input

Cells that are shaded red either require data input or contain an apparent data inconsistency

Do not enter data in any other location!

Avoid drag & drop and cut & paste methods of data entry.

Enter all values individually

File Save

After entering all data on "General Data Entry" (or any time thereafter), SAVE THE FILE by clicking on the macro button located on "General Data Entry" or "Visual Data Entry1" or "Visual Data Entry2" or "Point Count Data Entry".

The file name is generated automatically by concatinating information provided in General Data Entry, including:

a) Site/Project Identifier Code, b) Lab Name, c) Lab Job Number, d) Analysis Type, and e) Method (VE or PC).

Note that the directory where the macro will save the file depends on how the template file is opened:

If you open EXCEL and then open this spreadsheet, the new file will be saved in the same directory as the spreadsheet.
If you open this spreadsheet from Windows Explorer, then the file will be saved in you default directory for Excel (usually this is C:\Documents and Settings\My Documents)
