# Supplementary material for: Comparison of soil sampling and analytical methods for asbestos at the Sumas Mountain Asbestos Site—Working towards a toolbox for better assessment
Source: PLoS One. 2017 Jul 31;12(7):e0180210. doi: 10.1371/journal.pone.0180210 (PMC5536314; doi:10.1371/journal.pone.0180210)
Supplement: S3 Text — Case narrative for Technical Support—Fluidized Bed Asbestos Segregator. (PDF) [file pone.0180210.s004.pdf]

UNITED STATES ENVIRONMENTAL PROTECTION AGENCY
REGION 10 LABORATORY
7411 Beach Dr. East
PortOrchard, Washington 98366

MAR 0 5 2015

MEMORANDUM

TO: Julie Wroble, Toxicologist
Risk Evaluation Unit
Office of Environmental Assessment
U.S. EPA Region 10

FROM: Jed Januch, Environmental Protection Specialist
Environmental Services Unit
Office of Environmental Assessment
U.S. EPA Region 10

SUBJECT: Case narrative for Technical Support – Fluidized Bed Asbestos Segregator
Sumas Mountain Asbestos Methods Study
Project Code: SFP-078A

Introduction

This memorandum documents sampling of soil materials conducted by staff from the U.S. EPA Region 10 Office of Environmental Assessment (OEA) using a fluidized bed asbestos segregator (FBAS). Forty-five specimens of soil were submitted for sampling in the FBAS. The sampling was done between January 26, 2015, and February 5, 2015, by Jed Januch, U.S. EPA Region 10.

Soil Preparation

The forty-five specimens of soil were dried in a laboratory oven at 60° centigrade (C) for 24 hours. After drying, the samples were sieved through a Number 20, USA Standard Testing Sieve with 850 micrometer (μm) mesh. The fraction greater than 850 μm was examined with the aid of a Wild M5 stereomicroscope to assess whether suspected asbestos structures were present. There were suspected asbestos structures detected in the fractions greater than 850 μm for some of the samples containing grey colored sand/sediment. The factions less than 850 μm were tested in the FBAS.

Sampling Procedure

Sampling was done according to the U.S. EPA Region 10 standard operating procedure (SOP) OEAFIELDSOP-102 for Sampling, Sample Preparation and Operation of the Fluidized Bed Asbestos Segregator, Revision 1.0, dated August 19, 2011. To begin with, aliquots of each specimen (approximately 0.5 -1.0 grams) were combined with laboratory grade Ottawa 20/30 sand (approximately 19.0-19.5 grams). The sample/sand mixture (bed) was placed inside a clean glass vessel and air was drawn through the specimens for a sampling duration of 3 minutes. When the air flow is increased to where the pressure drop through the bed equals the weight of the bed, the solid material begins to circulate and act as a fluid. Small particles elutriate from the bed and are drawn with the air flow through the top of the glass vessel where a portion of the

air/particulate mixture is split off at an average flow rate of 237 cubic centimeters per minute (cc/m) and drawn through a 0.8 μm pore size mixed cellulose ester (MCE) filter. The particulates captured on the filter will be analyzed by transmission electron microscope (TEM) using the counting rules of the ISO10312 method. A summary of the sampling information is included in Table 1.

Table 1 – Sample information summary

Fluidized Bed Asbestos Segregator - Sampling Record
Project: Sumas Mtn. Asbestos Methods Study
Project Code: SFP-078A

Date	Time	Sample ID	Grams Sample	Grams Sand	Comments
1/26/2015	1600	14394100	0.555	19.50	Light brown soil - PMC/SEM/EDS duplicate
1/26/2015	1619	14394101	0.501	19.50	Light brown soil
1/26/2015	1627	14394102	0.500	19.50	Dark brown soil - vegetation
1/26/2015	1715	14394103	0.509	19.50	Dark brown soil - vegetation
1/26/2015	1730	14394104	0.510	19.50	Dark brown soil - vegetation
1/28/2015	1545	14394105	0.495	19.50	Dark brown soil - vegetation
1/28/2015	1610	14394106	0.500	19.50	Dark brown soil - vegetation
1/28/2015	1630	14394107	0.508	19.50	Light brown soil
1/28/2015	1647	14394108	0.499	19.50	Light brown soil
1/28/2015	1655	14394109	0.501	19.50	Light brown soil
1/29/2015	1102	14394110	0.506	19.50	Light brown soil - vegetation
1/29/2015	1153	14394111	0.514	19.50	Light brown soil - vegetation
1/29/2015	1400	14394112	0.511	19.50	Light brown soil - vegetation
1/29/2015	1430	14394113	0.520	19.50	Light brown soil - vegetation
1/29/2015	1530	14394114	0.504	19.50	Light brown soil - vegetation
1/29/2015	1549	14394115	1.001	19.00	Light brown soil - vegetation
1/29/2015	1630	14394116	1.049	19.00	Light brown soil - vegetation
1/29/2015	1649	14394117	1.003	19.00	Light brown soil
1/29/2015	1700	14394118	1.006	19.00	Light brown soil - vegetation
1/29/2015	1715	14394119	1.005	19.00	Light brown soil
2/2/2015	1330	14394120	1.005	19.00	Light brown soil
2/2/2015	1500	14394121	1.009	19.00	Light brown soil
2/2/2015	1647	14394122	1.008	19.00	Light brown soil
2/2/2015	1714	14394123	1.001	19.00	Light brown soil
2/2/2015	1724	14394124	1.009	19.00	Light brown soil
2/3/2015	0916	14394125	1.008	19.00	Light brown soil
2/3/2015	0927	14394126	1.004	19.00	Light brown soil
2/3/2015	0942	14394127	1.003	19.00	Light brown soil - vegetation
2/3/2015	1348	14394128	1.000	19.00	Light brown soil
2/3/2015	1425	14394129	1.000	19.00	Light brown/grey soil
2/3/2015	1536	14394130	1.004	19.00	Light brown/grey soil
2/3/2015	1629	14394131	1.008	19.00	grey sand/sediment - PCM/SEM/EDS duplicate

2/3/2015	1643	14394132	0.998	19.00	grey sand/sediment
2/3/2015	1658	14394133	1.003	19.00	grey sand/sediment
2/4/2015	1336	14394134	1.009	19.00	grey sand/sediment
2/4/2015	1447	14394135	1.003	19.00	grey sand/sediment
2/4/2015	1502	14394136	0.999	19.00	grey sand/sediment
2/4/2015	1617	14394137	1.004	19.00	grey sand/sediment
2/4/2015	1630	14394138	1.000	19.00	grey sand/sediment
2/4/2015	1434	14394139	1.003	19.00	grey sand/sediment
2/5/2015	1529	14394140	1.001	19.00	grey sand/sediment
2/5/2015	1549	14394141	1.005	19.00	grey sand/sediment
2/5/2015	1559	14394142	1.002	19.00	grey sand/sediment
2/5/2015	1610	14394143	0.999	19.00	grey sand/sediment
2/5/2015	1723	14394143	1.000	19.00	grey sand/sediment - QA Replicate
2/5/2015	1627	14394144	1.004	19.00	grey sand/sediment

Quality Assurance and Quality Control

Sand blanks and process blanks were collected during the course of the project. Analysis of the sand blanks and the process blanks will help determine if cross contamination is occurring, and if so, the degree to which it occurs. In addition, two filter lot blanks from SKC Lot Number 14723-7DE-036 were submitted for analysis. A copy of the quality certification for the MCE filters is appended to this report. Table 2 provides a summary of the quality control (QC) samples prepared for this project.

Table 2 – QC sample summary

Fluidized Bed Asbestos Segregator - Quality Control Sample Summary
Project: Sumas Mtn. Asbestos Methods Study
Project Code: SFP-078A

Date	Sample ID	QC Sample Type	Q_r	Comments
12/9/2014	14394189	Lot Blank	n/a	SKC Lot No. 14723-7DE-036
12/9/2014	14394190	Lot Blank	n/a	SKC Lot No. 14723-7DE-036
12/9/2014	14394191	Sand Blank	0.0125	ResTek Ottawa Sand Lot No. 904101-AE
1/29/2015	14394192	Process Blank	n/a	Sample open face in HEPA workstation
2/2/2015	14394193	Sand Blank	0.0122	ResTek Ottawa Sand Lot No. 904101-AE
2/3/2015	14394194	Process Blank	n/a	Sample open face in HEPA workstation
2/5/2015	14394195	Sand Blank	0.0122	ResTek Ottawa Sand Lot No. 904101-AE

The flow rate of air passing through the FBAS was calibrated with a Gilian Primary Flow standard, serial number 003507, equipped with a high-flow cell, serial number 0208000-H for the total flow rate in liters per minute, and a low-flow cell, serial number 002598-S for filter calibration in cc/m. The calibrator base was serviced and the flow cells were calibrated by the manufacturer December 9-10, 2014. Copies of the calibration certificates for the flow cells are appended to this report.

The specimens of soil used for this project were weighed inside a HEPA hood with a Mettler PM400 balance, serial number M73584. The balance was checked daily with the aid of a Troemner ASTM Class 1 metric weight set, serial number 41766. The balance was accurate within +/- 0.001 grams. The sand was weighed outside the HEPA hood with a Mettler Toledo B502-S balance serial number 1119200622. It was also checked with the Troemner metric weight set daily and found to be accurate within +/- 0.01 grams.

The degree of filter loading on two samples was screened with the aid of an Olympus CX21 phase contrast microscope (PCM) at 400x magnification. Images of random locations on the filters were collected using a Carl Zeiss Axioskop 40 microscope (set up for PCM) equipped with a Carl Zeiss AxioCam. In addition, two replicate samples were prepared in polycarbonate filters for analysis by scanning electron microscope (SEM) and energy dispersive spectroscopy (EDS). Photomicrographs by PCM and SEM as well as EDS spectra are appended to this report.

Sample Shipping and Chain of Custody

Samples were hand delivered by EPA staff on February 9, 2015, to Lab/Cor, Inc., located at 7619 6th Avenue NW, Seattle, WA 98117 and were received for Lab/Cor by Kate March. The original chain of custody forms are appended to this report.

Appendices

1. Quality Certificate – MCE filters
2. Flow Cell Calibration Certificates
3. Photomicrographs
4. Chain of Custody Forms

Appendix 1

CERTIFICATION

SKC Omega Specialty Division is committed to manufacturing the highest quality cassettes for asbestos sampling. This commitment is backed by the BestChek™ Seal of Approval which assures a higher standard in cassette reliability. Our BestChek™ approved cassettes are assembled in a full HEPA filtered clean environment. Our cassettes comply with both the NIOSH 7400 method which allows up to 5 fibers/100 fields and OSHA ID160 regulations which allow 4 fibers/100 fields. Always look for the BestChek™ Seal of Approval when purchasing air monitoring cassettes.

Cassette Category:	25mm, 3 Piece Conductive Cassette
Product Code:	225-326
Lot Number:	14723-7DE-036
Compliance:	NIOSH 7400, OSHA ID-160
Average Background Count:	1.75 fibers/100 Fields
PCM Analysis:	Passed
Clearing, Acetone Vapor:	Passed
Clearing, DP/DO (Monitoring Solution):	Passed
Plastic Conductivity by ASTM D-257:	2.3 x 10 ohms-cm
Percent Carbon by ASTM D-1603:	19.05
Cowl Conductivity by Resistance Meter:	10-5008 avg/ (x1000)
Filter Material:	MCE
Pore Size:	0.8 µm
Pad Material:	Cellulosic
Effective Filter Area:	385 mm^2
Cowl Length:	2 Inch
Analyst:	PH

Please keep for your records

SKC

1-800-752-8472

Appendix 2

Sensidyne, LP

CALIBRATION CERTIFICATE

Cell S/N: 0208000-H Date: December 10, 2014

This is to certify that the above referenced Gilibrator Flow Cell was calibrated using film flowmeter MCH-101-A, which has been calibrated by instruments directly traceable to the National Institute of Standards and Technology. NIST Report 8361604.

Results:

	REFERENCE MCH-101-A l/min	S/N 0208000-H l/min	RELATIVE DIFF. l/min	PERCENT DIFF.
	5.036	5.032	-0.004	-0.08
	5.031	5.031	0.000	0.00
	5.038	5.040	0.002	0.04
	5.036	5.037	0.001	0.02
	5.040	5.034	-0.006	-0.12
	5.039	5.031	-0.008	-0.16
	5.038	5.038	0.000	0.00
	5.036	5.032	-0.004	-0.08
	5.039	5.036	-0.003	-0.06
	5.037	5.029	-0.008	-0.16
MAX			-0.008	-0.160
MEAN	5.037	5.034		

Calibrated by *Agnes Banfield* Date: December 10, 2014

CODE 000

Sensidyne, LP

CALIBRATION CERTIFICATE

Cell S/N: 002598-S Date: December 10, 2014

This is to certify that the above referenced Gilibrator Flow Cell was calibrated using film flowmeter MCS-102-A, which has been calibrated by instruments directly traceable to the National Institute of Standards and Technology. NIST Report 8361604.

Results:

	REFERENCE MCS-102-A cc/min	S/N 002598-S cc/min	RELATIVE DIFF. cc/min	PERCENT DIFF.
	2017	2018	1	0.05
	2016	2018	2	0.10
	2018	2018	0	0.00
	2016	2020	4	0.20
	2021	2017	-4	-0.20
	2020	2018	-2	-0.10
	2018	2018	0	0.00
	2020	2018	-2	-0.10
	2022	2018	-4	-0.20
	2020	2020	0	0.00
MAX			4.0	0.2
MEAN	2018.8	2018.3		

Calibrated by ______________ Date: December 10, 2014

CODE 100

Appendix 3

Sumas Mountain Asbestos Methods
FBAS Test Samples
Project Code: SFP-078A

Analyst

Jed Januch

Equipment

Stereomicroscope – Wild M5 with Nikon CoolPix camera

PLM – Carl Zeiss Axioskop 40 with AxioCam MRc imaging system

SEM – JEOL JSM6510 LV

EDS – EDAX Genesis System

Sumas Mountain Asbestos Methods
FBAS Test Samples
Project Code: SFP-078A

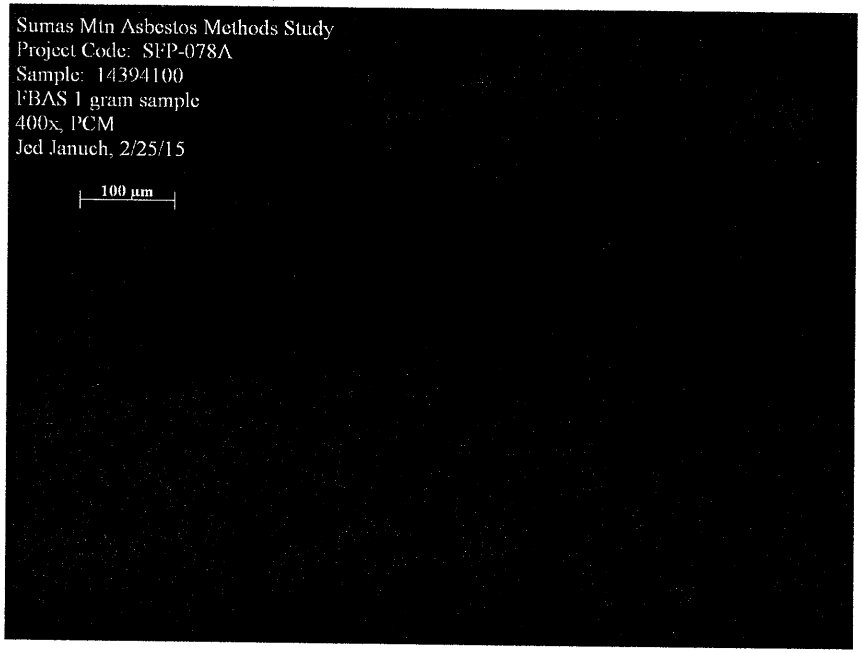

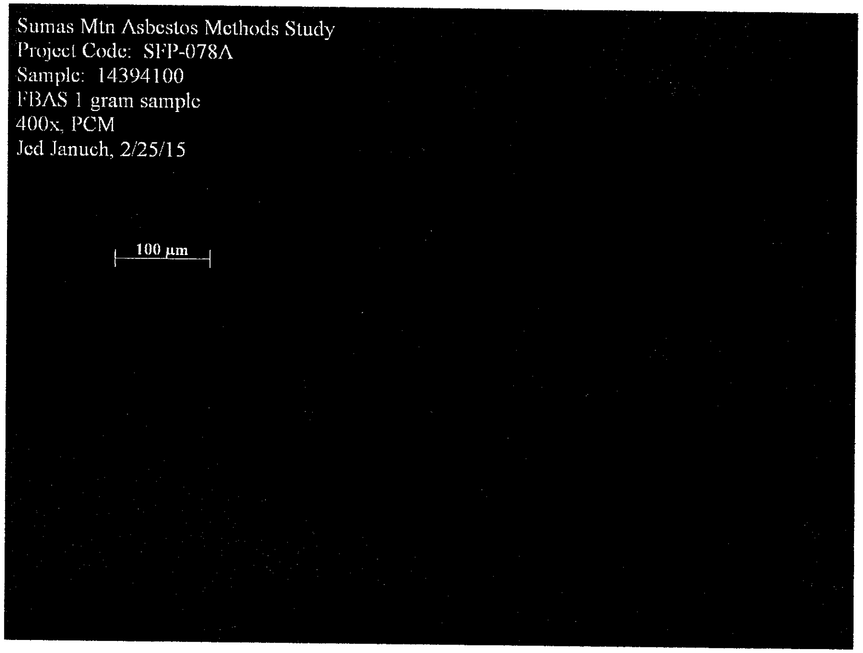

Sumas Mountain Asbestos Methods
FBAS Test Samples
Project Code: SFP-078A

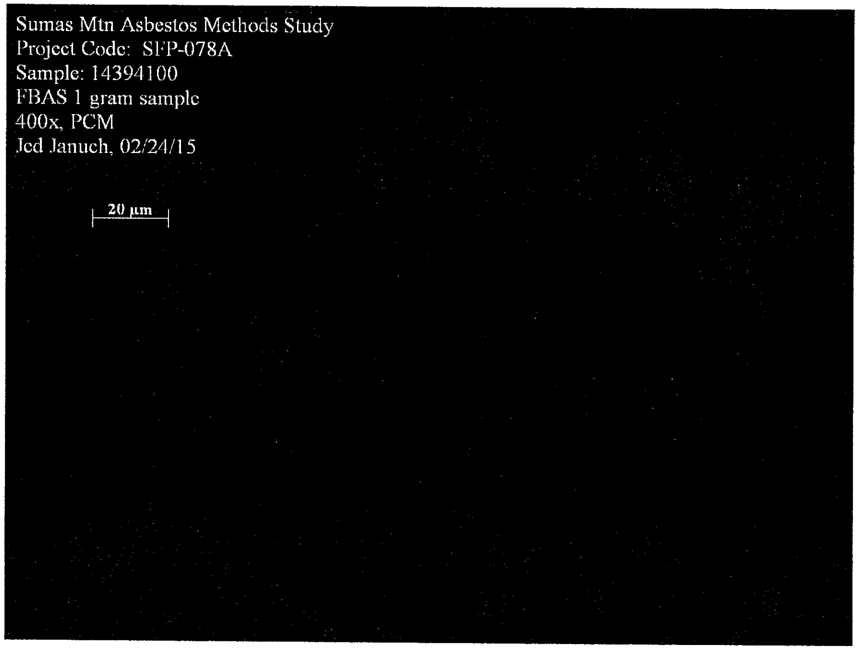

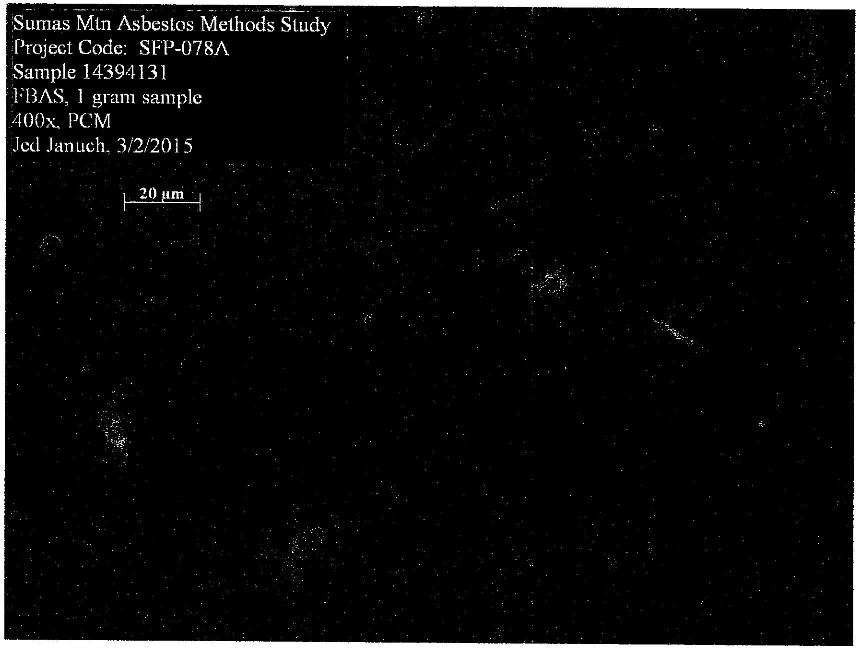

Sumas Mountain Asbestos Methods
FBAS Test Samples
Project Code: SFP-078A

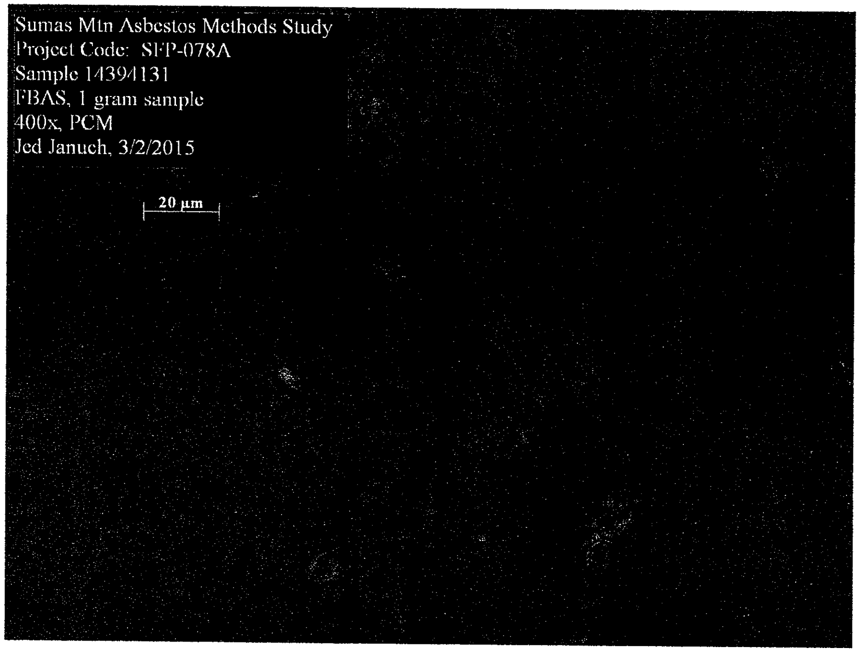

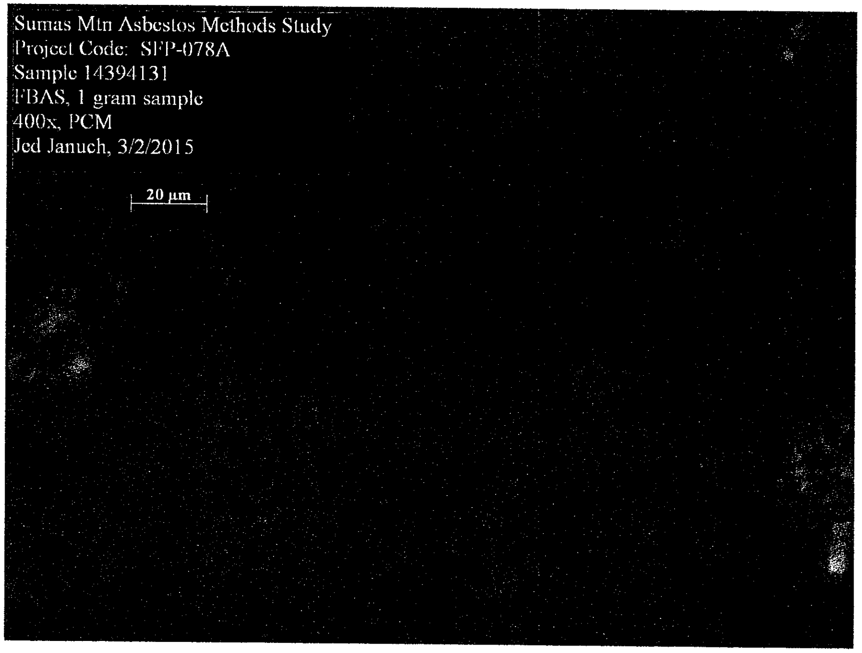

Sumas Mountain Asbestos Methods
FBAS Test Samples
Project Code: SFP-078A

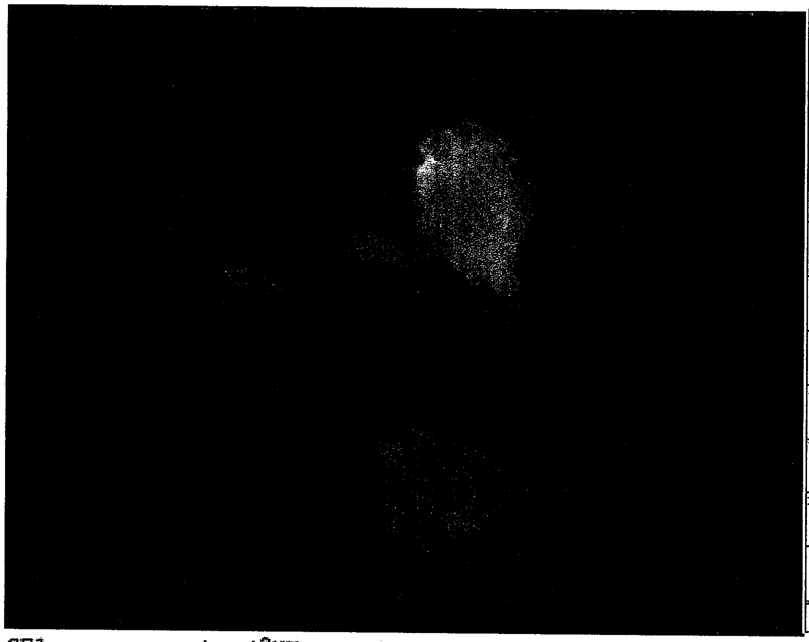

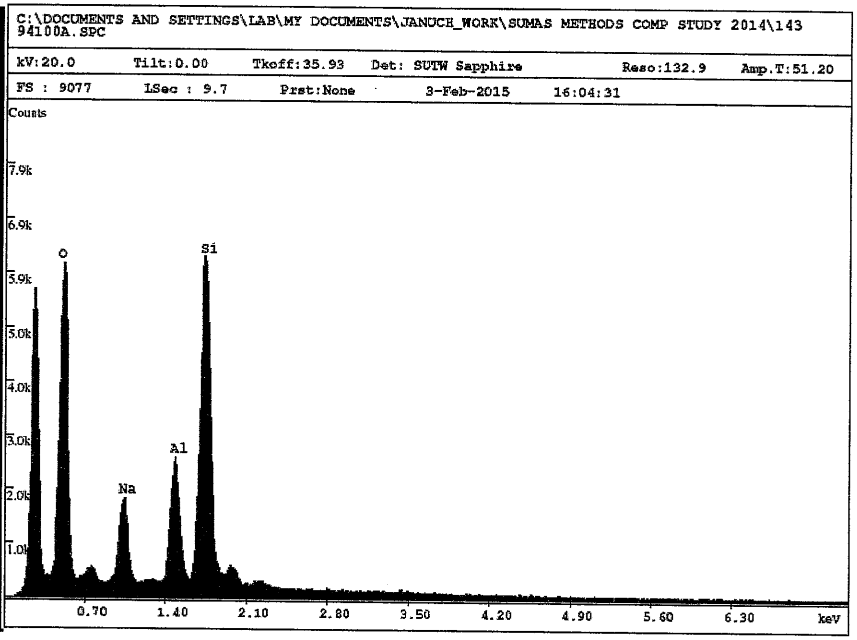

Sumas Mountain Asbestos Methods
FBAS Test Samples
Project Code: SFP-078A

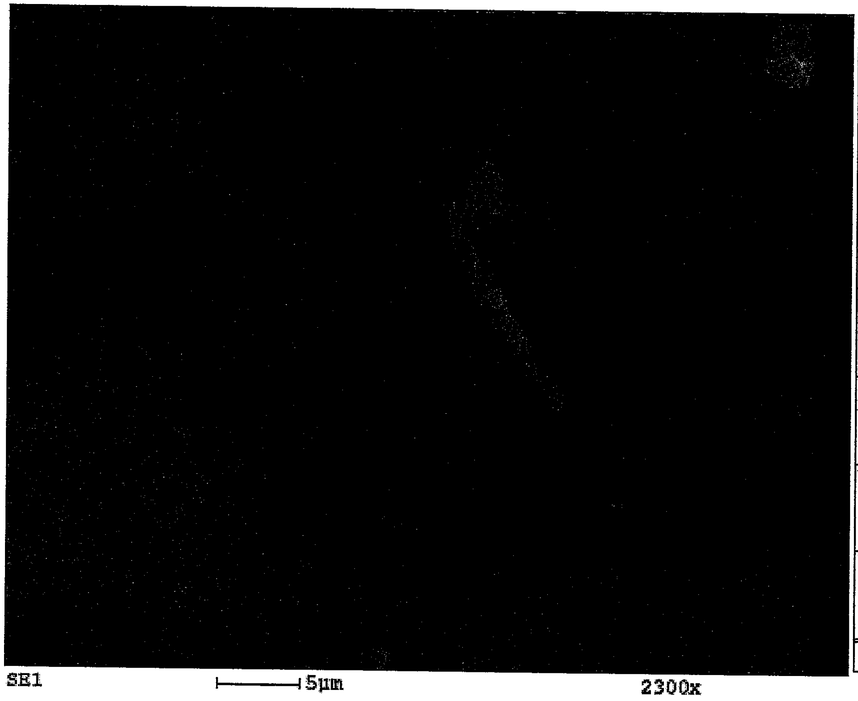

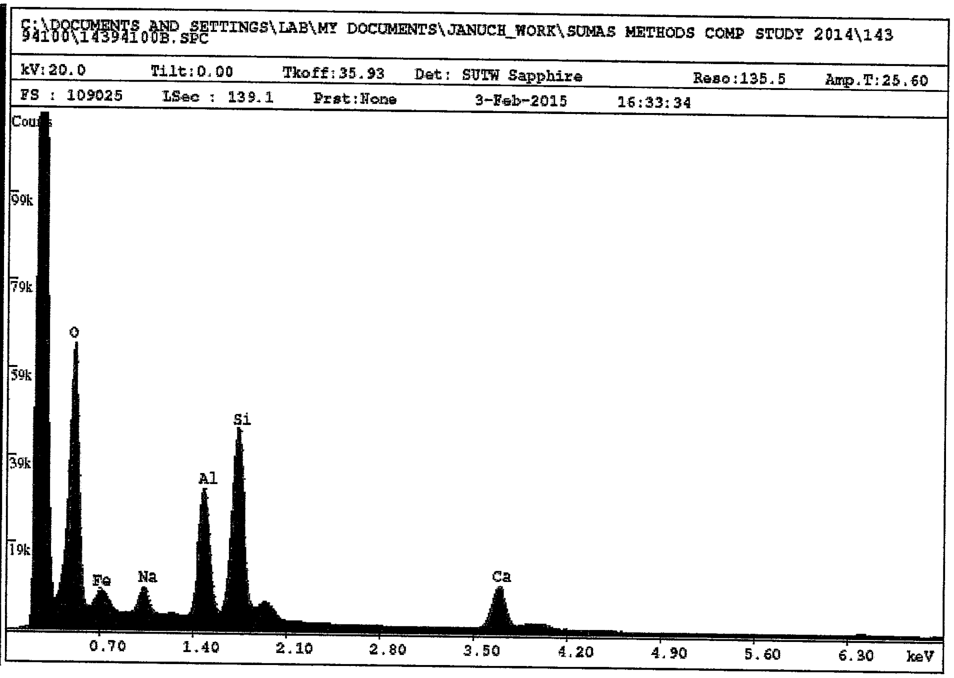

Sumas Mountain Asbestos Methods
FBAS Test Samples
Project Code: SFP-078A

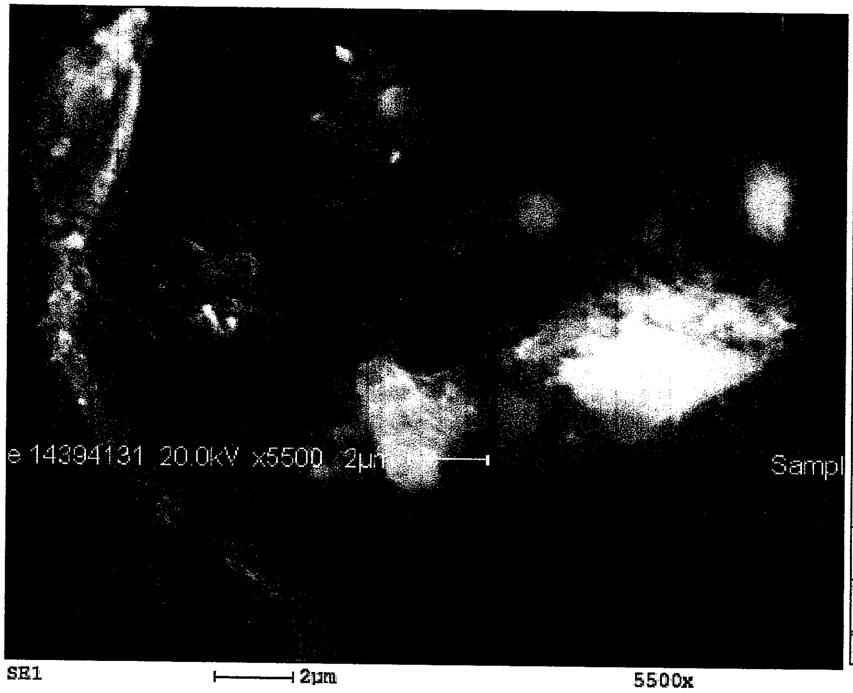

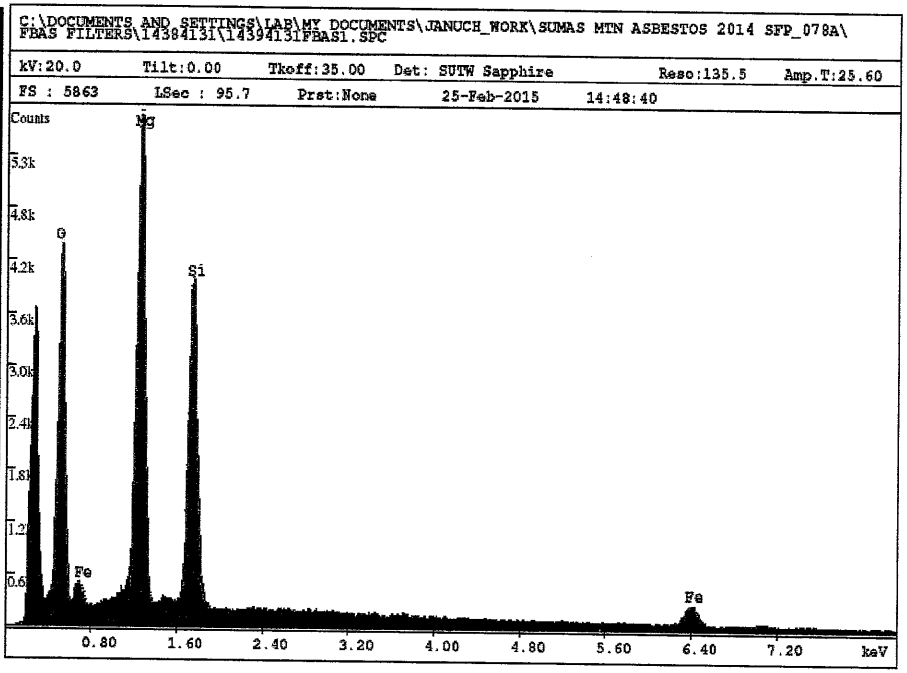

Sumas Mountain Asbestos Methods
FBAS Test Samples
Project Code: SFP-078A

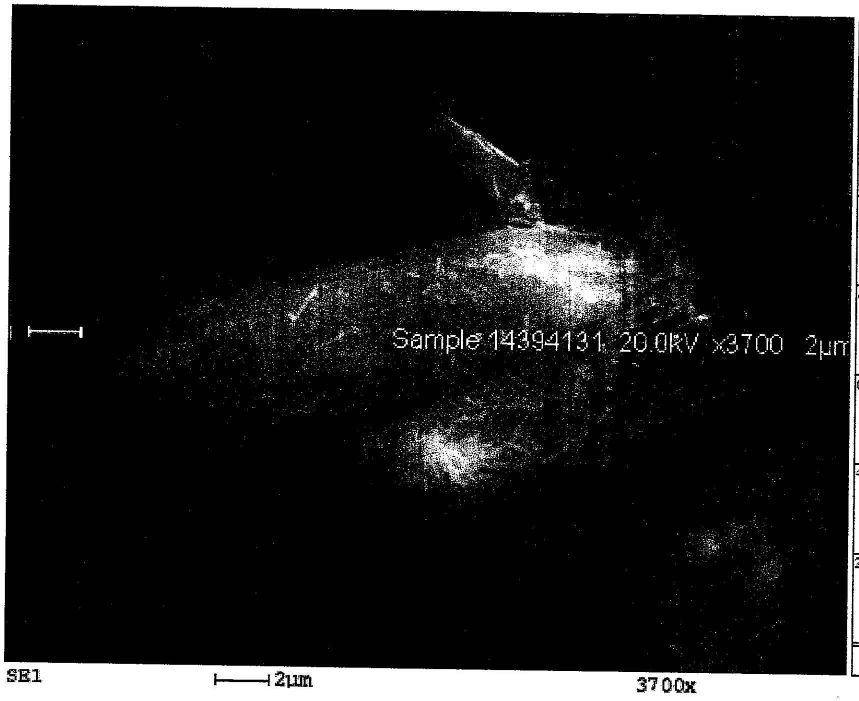

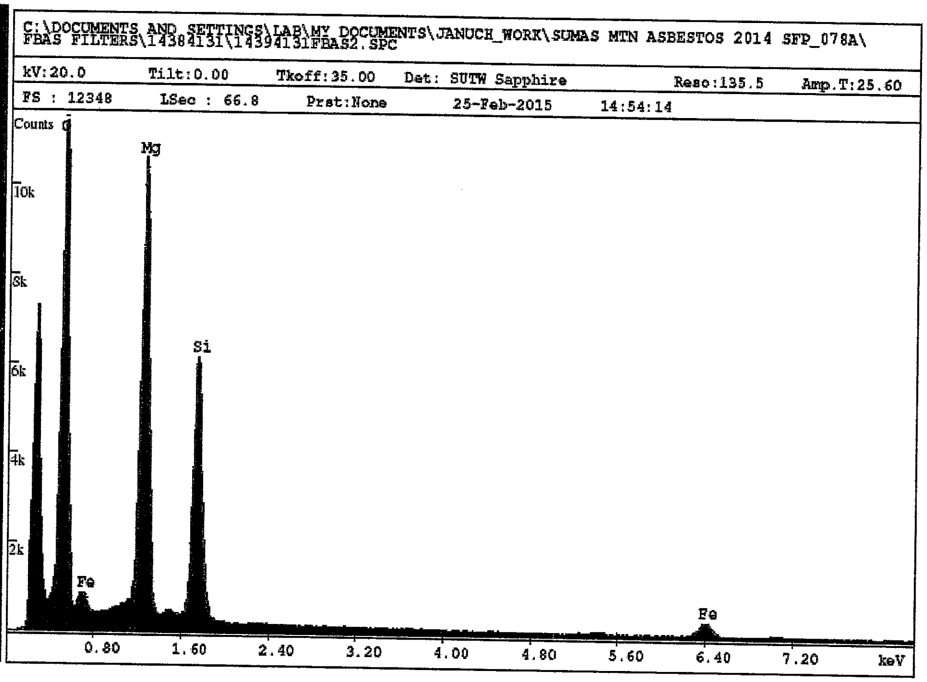

Sumas Mountain Asbestos Methods
FBAS Test Samples
Project Code: SFP-078A

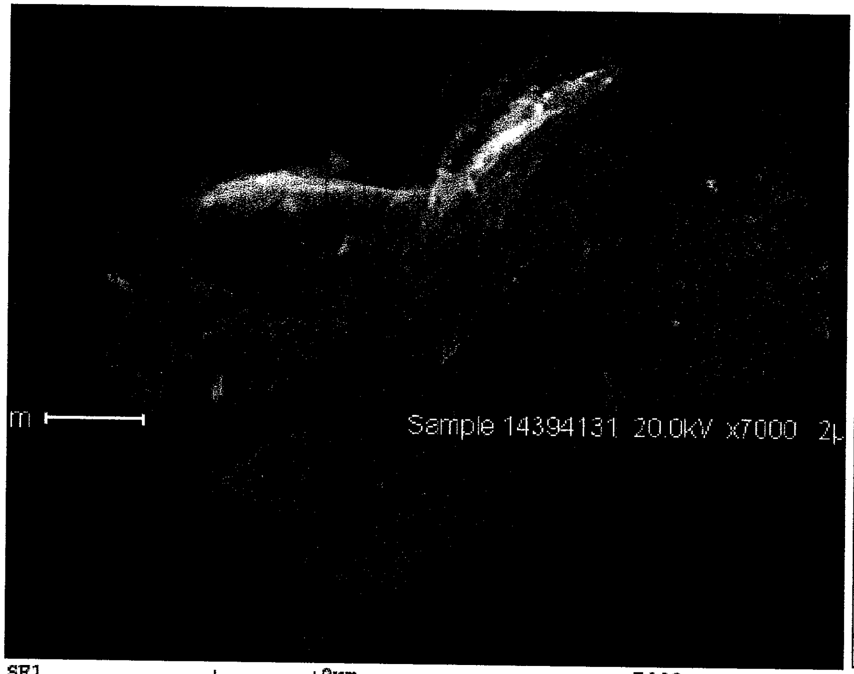

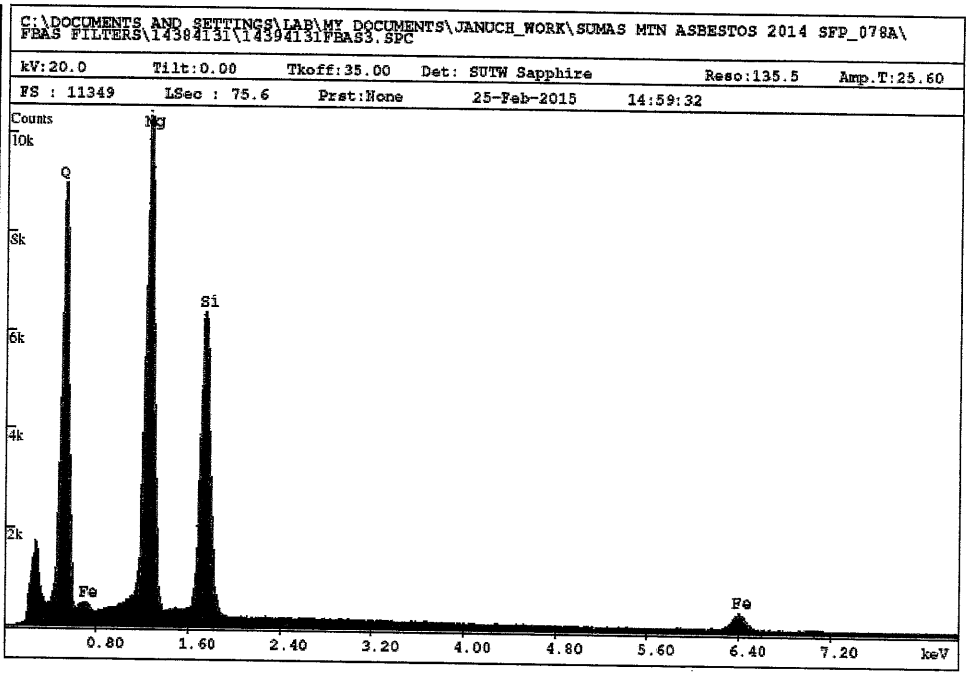

Sumas Mountain Asbestos Methods
FBAS Test Samples
Project Code: SFP-078A

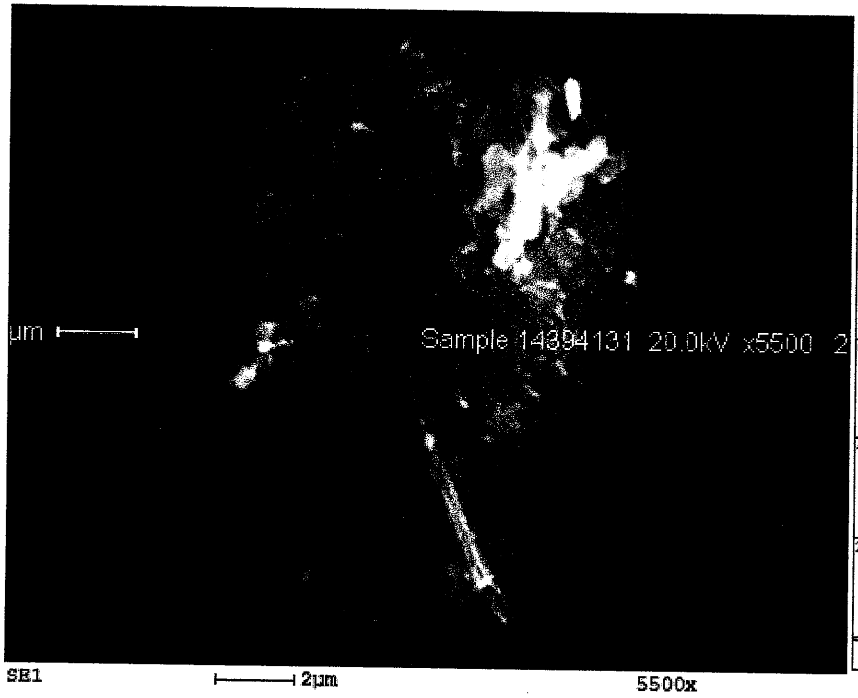

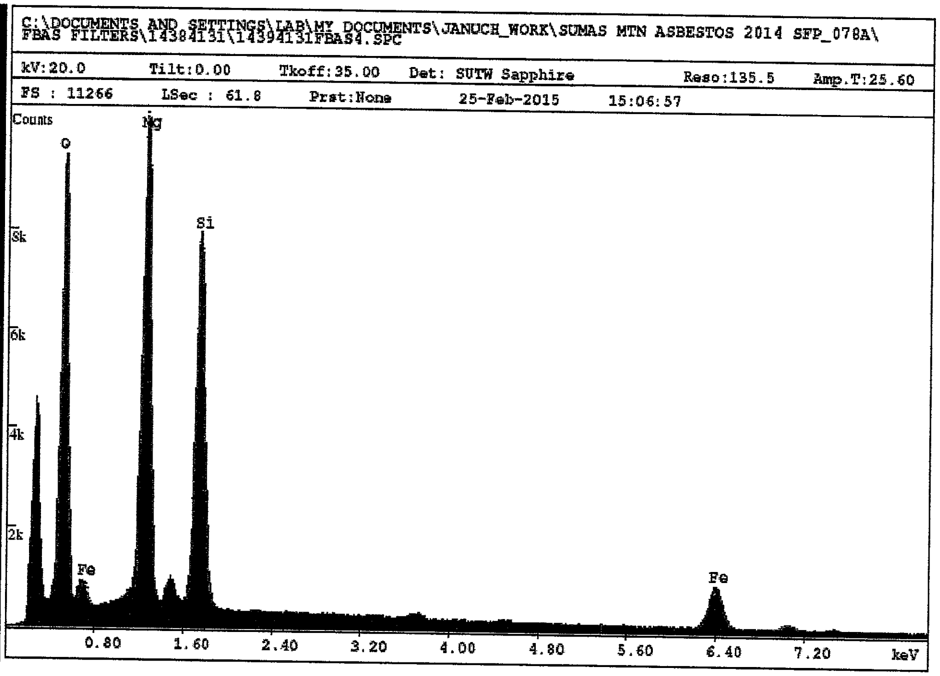

Sumas Mountain Asbestos Methods
FBAS Test Samples
Project Code: SFP-078A

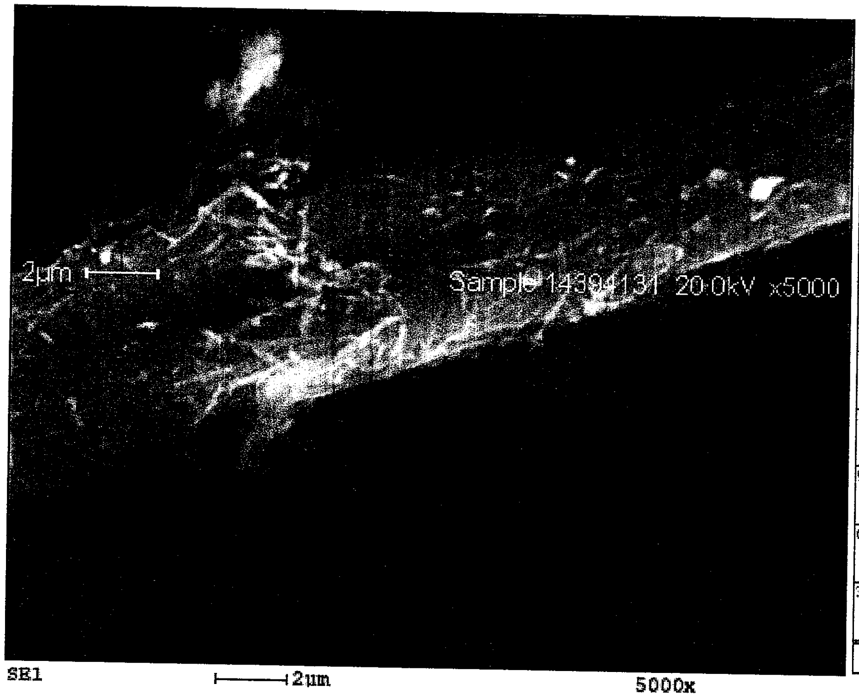

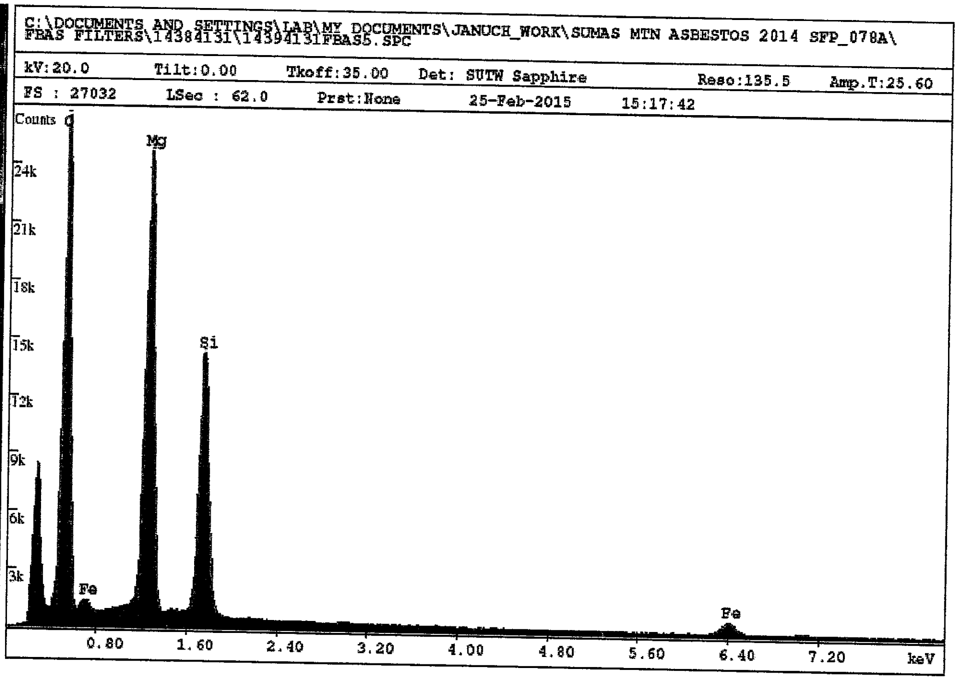

Appendix 4

EPA Region 10 - FBAS CHAIN OF CUSTODY RECORD
OEAFIELDSOP-102 (Revision 1.0)

COC No.: ____________ [FBAS-YYMMDD###]

COC entered by (signature): [signature]

COC Page 1 of 1

Project Manager: Julie Wroble

Project Identifier: Sumas Mtn. Asbestos - FBAS

Method of Shipment: EPA - deliver

Shipment Tracking No.: ____________

Ship Date: ____________

Shipment Destination: Lab/Cor, Inc. Seattle, WA

Shipment Origin: EPA Region 10 Laboratory Port Orchard, WA

Sample Information		Sample Preparation Request				Filter Analysis Request				FBAS Information			
Sample ID (PE Standard ID or Replicate Filter ID)	Medium [S for soil; A for air filter]	OEAFIELDSOP-102 (Rev 1)++	with cyclone	without cyclone		TEM, ISO 10312:1995(E)**				Blank? [mark 'x' if yes]	FBAS Flow Ratio (Q_R)	FBAS Soil Mass (grams, dry weight)	Sample Remarks
14394189	A	X				X				X		N/A	
14394190	A	X				X				X		"	
14394191	A	X		X		X				X	0.0125	"	

RELINQUISHED BY:		RELINQUISHED TO:	
NAME, AFFILIATION	DATE, TIME	NAME, AFFILIATION	DATE, TIME
[signature], EPA	12/11/14 0930	M. V. Thompson	12-11-14 0930

COMMENTS/CONDITION OF SAMPLES:

++ Prepare replicate filters using fluidized bed asbestos segregator in accordance with SOP.

** Specific recording and stopping rules are specified in a site/project specific QAPP. As needed, grids may be prepared indirectly in accordance with ISO 13794:1999(E).

FBAS CHAIN OF CUSTODY RECORD

COC No.: FBAS-150209001
[FBAS-YYMMDD###]
COC Page 1 of 3

COC entered by (signature): [signature]

Project Manager: Julie Wroble   Project Identifier: Sumas Mtn Asbestos Methods - FBAS

Method of Shipment: Hand Deliver   Shipment Tracking No.: N/A   Ship Date: 2/9/15

Shipment Origin: EPA Region 10 Laboratory, Port Orchard, WA   Shipment Destination: LabCor, Inc., Seattle, WA

Sample Information		Sample Preparation Request				Filter Analysis Request				FBAS Information			
Sample ID (PE Standard ID or Replicate Filter ID)	Medium [S for soil; A for air filter]	OEAFIELDSOP-102 (Rev 1)++	with cyclone	without cyclone		TEM, ISO 10312:1995(E)**				Blank [LT for lot; PB for prep; SB for sand]	FBAS Flow Ratio (Q_R)	FBAS Soil Mass (grams, dry weight)	Sample Remarks
14394100	A	X		X		X					0.0122	0.555 g	1/27/15
14394101	A	X		X		X					0.0122	0.501 g	1/27/15
14394102	A	X		X		X					0.0122	0.500 g	1/27/15
14394103	A	X		X		X					0.0122	0.509 g	1/27/15
14394104	A	X		X		X					0.0122	0.510 g	1/27/15
14394105	A	X		X		X					0.0122	0.495 g	1/28/15
14394106	A	X		X		X					0.0122	0.500 g	1/28/15
14394107	A	X		X		X					0.0122	0.508 g	1/28/15
14394108	A	X		X		X					0.0122	0.499 g	1/28/15
14394109	A	X		X		X					0.0122	0.561 g	1/28/15
14394110	A	X		X		X					0.0122	0.506 g	1/29/15
14394111	A	X		X		X					0.0122	0.514 g	1/29/15
14394112	A	X		X		X					0.0122	0.511 g	1/29/15
14394113	A	X		X		X					0.0122	0.520 g	1/29/15
14394114	A	X		X		X					0.0122	0.504 g	1/29/15
14394115	A	X		X		X					0.0122	1.001 g	1/29/15
14394116	A	X		X		X					0.0122	1.049 g	1/29/15
14394117	A	X		X		X					0.0122	1.003 g	1/29/15
14394118	A	X		X		X					0.0122	1.006 g	1/29/15
14394119	A	X		X		X					0.0122	1.005 g	1/29/15
14394120	A	X		X		X					0.0122	1.005 g	2/2/15

RELINQUISHED BY: NAME, AFFILIATION	DATE, TIME	RELINQUISHED TO: NAME, AFFILIATION	DATE, TIME
[signature] U.S. EPA	2/9/15 1330	[signature] LC	2/9/15 1330

COMMENTS/CONDITION OF SAMPLES:

++ Prepare replicate filters using fluidized bed asbestos segregator in accordance with SOP.

** Specific recording and stopping rules are specified in the study QAPP. As needed, grids may be prepared indirectly in accordance with ISO 13794:1999(E).

FBAS CHAIN OF CUSTODY RECORD

COC No.: FBAS-150209002
[FBAS-YYMMDD###]
COC Page 2 of 3

COC entered by (signature): [signature]

Project Manager: Julie Wroble

Project Identifier: Sumas Mtn Asbestos Methods - FBAS

Method of Shipment: Hand Deliver

Shipment Tracking No.: N/A

Ship Date: 2/9/15

Shipment Origin: EPA Region 10 Laboratory Port Orchard, WA

Shipment Destination: LAB/COR, Inc. Seattle, WA

Sample Information		Sample Preparation Request				Filter Analysis Request				FBAS Information			
Sample ID (PE Standard ID or Replicate Filter ID)	Medium [S for soil; A for air filter]	OEAFIELDSOP-102 (Rev 1)++	with cyclone	without cyclone		TEM, ISO 10312:1995(E)**				Blank [LT for lot;PB for prep; SB for sand]	FBAS Flow Ratio (Q_R)	FBAS Soil Mass (grams, dry weight)	Sample Remarks
14394121	A	X		X		X					0.0122	1.009 g	2/2/15
14394122	A	X		X		X					0.0122	1.008 g	2/2/15
14394123	A	X		X		X					0.0122	1.001 g	2/2/15
14394124	A	X		X		X					0.0122	1.009 g	2/2/15
14394125	A	X		X		X					0.0122	1.008 g	2/3/15
14394126	A	X		X		X					0.0122	1.004 g	2/3/15
14394127	A	X		X		X					0.0122	1.003 g	2/3/15
14394128	A	X		X		X					0.0122	1.000 g	2/3/15
14394129	A	X		X		X					0.0122	1.000 g	2/3/15
14394130	A	X		X		X					0.0122	1.004 g	2/3/15
14394131	A	X		X		X					0.0122	1.008 g	2/3/15
14394132	A	X		X		X					0.0122	0.998 g	2/3/15
14394133	A	X		X		X					0.0122	1.003 g	2/3/15
14394134	A	X		X		X					0.0122	1.009 g	2/4/15
14394135	A	X		X		X					0.0122	1.003 g	2/4/15
14394136	A	X		X		X					0.0122	0.999 g	2/4/15
14394137	A	X		X		X					0.0122	1.004 g	2/4/15
14394138	A	X		X		X					0.0122	1.000 g	2/4/15
14394139	A	X		X		X					0.0122	1.003 g	2/4/15
14394140	A	X		X		X					0.0122	1.001 g	2/5/15
14394141	A	X		X		X					0.0122	1.005 g	2/5/15

RELINQUISHED BY: NAME, AFFILIATION	DATE, TIME	RELINQUISHED TO: NAME, AFFILIATION	DATE, TIME
[signature] U.S. EPA	2/9/15 1330	KWaller L/C	2/9/15 1330

COMMENTS/CONDITION OF SAMPLES:

++ Prepare replicate filters using fluidized bed asbestos segregator in accordance with SOP.

** Specific recording and stopping rules are specified in the study QAPP. As needed, grids may be prepared indirectly in accordance with ISO 13794:1999(E).

FBAS CHAIN OF CUSTODY RECORD

COC No.: FBAS-150209003
[FBAS-YYMMDD###]
COC Page 3 of 3

COC entered by (signature): [signature]

Project Manager: Julie Wroble

Project Identifier: Sumas Mtn Asbestos Methods - FBAS

Method of Shipment: Hand Deliver

Shipment Tracking No.: N/A

Ship Date: 2/9/15

Shipment Origin: EPA Region 10 Laboratory Port Orchard, WA

Shipment Destination: LAB/COR, Inc. Seattle, WA

Sample Information		Sample Preparation Request				Filter Analysis Request				FBAS Information			
Sample ID (PE Standard ID or Replicate Filter ID)	Medium [S for soil; A for air filter]	OEAFIELDSOP-102 (Rev 1)++	with cyclone	without cyclone		TEM, ISO 10312:1995(E)**				Blank [LT for lot;PB for prep; SB for sand]	FBAS Flow Ratio (Q_R)	FBAS Soil Mass (grams, dry weight)	Sample Remarks
14394142	A	X		X		X					0.0122	1.002 g	2/5/15
14394143	A	X		X		X					0.0122	0.999 g	2/5/15
14394143 dup	A	X		X		X					0.0122	1.000 g	2/5/15
14394144	A	X		X		X					0.0122	1.004 g	2/5/15
14394192	A	X		X		X				PB	—	—	1/29/15
14394193	A	X		X		X				SB	0.0122	Ø	2/2/15
14394194	A	X		X		X				PB	—	—	2/3/15
14394195	A	X		X		X				SB	0.0122	Ø	2/5/15

RELINQUISHED BY:		RELINQUISHED TO:	
NAME, AFFILIATION	DATE, TIME	NAME, AFFILIATION	DATE, TIME
[signature] U.S. EPA	2/9/15 1330	[signature] L/C	2/9/15 1330

COMMENTS/CONDITION OF SAMPLES:

++ Prepare replicate filters using fluidized bed asbestos segregator in accordance with SOP.

** Specific recording and stopping rules are specified in the study QAPP. As needed, grids may be prepared indirectly in accordance with ISO 13794:1999(E).
