# Supplementary material for: Comparison of soil sampling and analytical methods for asbestos at the Sumas Mountain Asbestos Site—Working towards a toolbox for better assessment
Source: PLoS One. 2017 Jul 31;12(7):e0180210. doi: 10.1371/journal.pone.0180210 (PMC5536314; doi:10.1371/journal.pone.0180210)
Supplement: S4 Text — (PDF) [file pone.0180210.s005.pdf]

CB&I
2700 Chandler Avenue, Building C
Las Vegas, NV 89120
Tel: +1 702 795 0515
Fax: +1 702 795 8210
www.CBI.com

July 10, 2015

Julie Wroble
EPA Site Manager Region 10
EPA Region 10
1200 6th Ave, Suite 900
Seattle, WA 98101

Document ID #: 3015-07102015-4

Dear Ms. Wroble:

EPA CONTRACT NUMBER EP-W-10-033
TASK ORDER NUMBER 3015
ASBESTOS QA SUPPORT

Enclosed please find the Release of Validated Data Report for the validation of Transmission Electron Microscopy (TEM) air sample data, Laboratory Job Number 141013. The three (3) FBAS air samples associated with these data were analyzed by Lab/Cor Portland, Inc., Portland, Oregon for the Sumas Mountain Asbestos Soil Project. This report and accompanying appendices are deliverables under Task 10 of the subject Task Order.

If you have any questions, please feel free to contact me.

Sincerely,

Lyndsay Gensler
Task Leader, QATS Program
CB&I Federal Services LLC
Phone: 702.895.8730
E-Mail Address: lyndsay.gensler@cbifederalservices.com

cc: Shari Myer, EPA-ASB, QATS Task Order Project Officer
Administrative Contracting Officer (letter only)

RELEASE OF VALIDATED DATA

DATE: July 10, 2015

SUBJECT: Review of Data for Laboratory Job Number: 141013

LABORATORY: Lab/Cor Portland, Inc., Portland, Oregon

FROM: Quality Assurance Technical Support (QATS) Program, Las Vegas, NV
CB&I Federal Services LLC

TO: Julie Wroble, Environmental Protection Agency

QATS reviewed the data for the following case:

Applicable SAP: NA

Chain-of-Custody Number: NA

Method: Transmission Electron Microscopy (TEM) ISO 10312.

Applicable Laboratory Modification(s): NA

Number and Type of Samples: 3 FBAS Air Samples

EPA Sample Numbers: 14394189, 14394190, and 14394191.

VALIDATION SUMMARY

Three (3) FBAS air samples from Laboratory Job Number 141013, were collected and hand-delivered to Lab/Cor Portland, Inc. in Portland, OR for preparation by FBAS SOP OEAFIELDSOP-102 (Rev. 1) and analyzed by TEM ISO 10312. The samples were received at the laboratory intact on 12/11/2014, and were analyzed on 01/07/2015.

Listed below are the Data Qualification Summary Table, EDD/Bench Sheet Discrepancy Table, Data Qualifier Table, and Reason Code Table.

DATA QUALIFICATION SUMMARY TABLE

Criteria Exceeded	EPA Sample ID	Validation Qualifier	Reason Code
None			

EDD/BENCH SHEET DISCREPANCY TABLE

EPA Sample ID	C# *	Method/Matrix	Lab. Job No.	Analysis Date	Discrepancy
14394189<br>14394190<br>14394191	0	ISO 10312/Air	141013	01/07/2015	EDD indicates magnification as 15,941, whereas, the 01/03/2015 calibration indicates magnification as 16,074 for the H-7000FA instrument.

'*' The EDD correction number in column 2. (i.e., C0, C1, C2, etc..)

DATA QUALIFIER TABLE

Qualifier	Definition
**J**	The result is estimated. The associated numerical value is an approximation.
**UJ**	The non-detect result may be inaccurate or imprecise due to the quality of the data generated because certain QC criteria were not met.
**R**	The sample results are rejected due to serious deficiencies.
**X**	Validator defined.

TEM REASON CODE TABLE

Reason Code	Definition
**MC**	Structure/fiber counts and recorded structure dimensions may be inaccurate due to improper or infrequent scope alignment and/or magnification calibrations.
**IC**	Identification by elemental composition or diffraction pattern may be inaccurate due to improper or infrequent EDXA or camera constant calibration.
**PA**	Structure/fiber counts and reported concentrations may be inaccurate due to improper or infrequent calibration of the plasma asher.
**SC**	The reported concentration may be inaccurate due to the condition of samples upon receipt at the laboratory.
**DL**	The area analyzed, structures counted, or AS do not meet the requirements specified in the applicable SAP Analytical Summary.
**ID**	The asbestos identification and concentrations may be inaccurate because the recorded structure types are not consistent with those described in the applicable TEM Method and/or laboratory modification(s).

VALIDATION PROCESS

The date the samples for Laboratory Job Number 141013 were collected from the subject site was not indicated on the chain-of-custody record. All samples were prepared by the FBAS SOP OEAFIELDSOP-102 (Rev. 1) and analyzed in accordance with TEM ISO 10312. CB&I's Quality Assurance Technical Support (QATS) Program performed validation and a transcription check in accordance with method-specific data validation SOPs. QATS preparation of this report and appendices was performed under Technical Direction 03, Task 10, of Task Order 3015.

The sample results on bench sheets and other supporting documents provided in the hardcopy deliverables were compared to the entries in the associated laboratory method-specific EDDs (where applicable) to ensure that the reported results are complete, compliant with the specified methodology, and accurate. Additional support information provided in this data validation report include the QATS Data Review Checklist used to document the data validation process (see Appendix A); and the sample results as reported by the laboratory, with qualifiers as applicable (see Appendix B).

TEM VALIDATION SUMMARY

1. **DATA PACKAGE INVENTORY AND SAMPLE RECEIPT:** The data package included a narrative, Chain-of-Custody (COC) record, EDD files, raw data (bench sheets), and QC samples. The samples were properly packaged, sealed, undamaged, and labeled upon receipt at the laboratory. The COC record was reviewed and found to be acceptable, though the sample collection data was not indicated.

2. **SAMPLE PREPARATION:** The appropriate preparation documents were provided.

3. **EQUIPMENT CALIBRATION AND PERFORMANCE CHECKS (i.e., daily microscope alignment, screen magnification, EDS calibration, and sensitivity checks):** The equipment alignment and calibration documentation provided separately were performed at the correct frequency, indicating that the instruments were in proper working order during the time of sample analyses. However, the EDD indicates magnification as 15,941, whereas, the 01/03/2015 calibration indicates magnification as 16,074 for the H-7000FA instrument.

4. **ANALYTICAL SENSITIVITY:** A sufficient number of grid openings have been analyzed to achieve the required analytical sensitivity and/or the appropriate stopping rule was invoked.

5. **STRUCTURE RECORDING AND ASBESTOS IDENTIFICATION:** The structure recording and asbestos identification were found to be acceptable, with no structures found.

6. **BLANK ANALYSIS:** The three blanks analyzed and reported with this sample set comprise the entire lab job.

7. **ANALYTICAL VARIABILITY:** No QC samples were reported with this sample set.

8. **LABORATORY MODIFICATIONS:** NA

9. **OVERALL ASSESSMENT OF DATA:** With the exception of the magnification discrepancy described above, the deliverable was found to be complete and accurate. No structures were found in the samples. No qualification of the data is necessary.

REVIEWED BY: Lyndsay Gensler **DATE:** 06/16/2015

Appendix A

Data Review Checklist

Data Review Checklist for the Verification and Validation of Transmission Electron Microscopy (TEM) Data Deliverables

**Project Name: Sumas Mtn Asbestos Project**	**Case or Sample Set ID: 141013**
**Number of Samples/Matrix: 3 Air Samples**	**COC Number: NA**
**TEM Analytical Method: ISO 10312, OEAFIELDSOP-102 (Rev. 1)**	**Level of Validation (Circle one): 1 2 [3] Other**

**1.0 Data Package Inventory**	**Yes**	**No**	**Comments**
1.1 Were the project-specific requirements (i.e. acceptance criteria & analytical sensitivities) provided by the client prior to the initiation of validation activities?	☐	☒	A request was made to the laboratory on 06/04/2015 for the laboratory's QAPP and/or SOPs documenting the frequency of the different TEM calibrations. The documents were received on 06/18/2015.
1.2 Did the received hard copy deliverables contain all the necessary components:			
1.2.1 Case Narrative (Level 1, 2 & 3)?	☒	☐	
1.2.2 Chain-of-Custody (Level 1, 2 & 3)?	☐	☒	
1.2.3 Form I or equivalent (Level 1, 2 & 3)?	☒	☐	
1.2.4 Raw Data - Count Sheets (Level 1, 2 & 3)?	☒	☐	
1.2.5 QC Sample Data (Level 2 & 3):	☒	☐	
1.2.5.1 Blank(s)?	☒	☐	All three samples were blanks with two indicated as Lot Blanks for EPA Sample Nos. 14394189 and 14394190 and one as a blank for EPA Sample No. 14394191 on the Analysis Report and Direct Raw Data sheets. Note that the EDD's for all three samples indicate sample type as "Lot Blank".
1.2.5.2 Replicate(s)?	☐	☒	
1.2.5.3 Duplicate(s)?	☐	☒	
1.2.5.4 Verified Analysis?	☐	☒	
1.2.6 Calibration Data (Level 3)?	☒	☐	
1.2.7 Communication Records (Level 1, 2 & 3)?	☐	☒	
1.2.8 Miscellaneous?	☐	☐	
1.3 Were the necessary components received to perform the requested level of validation?	☒	☐	
**2.0 Chain-of-Custody Information Verification (Level 1, 2 & 3)**			
2.1 Were the following information recorded in the hard copy electronic deliverables (if applicable) consistent with the information recorded on the COC:			
2.1.1 COC Number?	☐	☒	No COC No. indicated on the EPA Region 10 – FBAS Chain of Custody Record.
2.1.2 Case or Sample Set Number?	☒	☐	
2.1.3 EPA Sample ID?	☒	☐	
2.1.4 Date/Time Collected?	☐	☐	NA
2.1.5 Sample Volume?	☐	☐	NA
2.1.6 Sample Matrix?	☒	☐	
2.1.7 Analyses (Method)?	☒	☐	
2.1.8 Date/Time Received?	☒	☐	
2.1.9 Other (describe)?	☐	☐	
2.2 Were the COC records signed and dated upon receipt?	☒	☐	

Additional Comments:

Data Review Checklist for the Verification and Validation of Transmission Electron Microscopy (TEM) Data Deliverables

3.0 Sample Result Verification & Validation (Level 1, 2 & 3)	Yes	No	Comments
3.1 Is the sample preparation method documented and final sample volume recorded?	☒	☐	
3.2 Is the correct number of grid openings used to achieve the specified analytical sensitivity?	☒	☐	
3.3 Verify that the following information from the laboratory's bench sheets have been transcribed correctly:			
3.3.1.1 Grid identification?	☒	☐	
3.3.1.2 Grid opening?	☒	☐	
3.3.1.3 Structure type?	☒	☐	
3.3.1.4 Number of primary and secondary structures?	☒	☐	
3.3.1.5 Length and width dimensions?	☒	☐	
3.3.1.6 Structure identification?	☒	☐	
3.3.1.7 Mineral type?	☒	☐	
3.4 Are overloaded samples correctly reported to the specified percent obscuration (i.e. 10%, 25%)?	☐	☐	NA
3.5 If overloading occurs, are samples prepared by an alternate method (i.e. indirect preparation)?	☐	☐	NA
3.6 Verify that the following information is documented correctly:			
3.6.1 Magnification?	☒	☐	
3.6.2 Field or QC sample type?	☒	☐	
3.6.3 Number of grids prepared?	☒	☐	
3.6.4 Filter area in (mm^2)?	☒	☐	
3.6.5 Analysis date?	☒	☐	
3.7 Verify the totals reported on the count sheets for the various types of structures. These may include:			No structures were detected in any of the samples.
3.7.1 Total EPA Structures	☐	☐	NA
3.7.2 PCMe Structures	☐	☐	NA
3.7.3 AHERA Structures	☐	☐	NA
3.7.4 Berman Crump Structures	☐	☐	NA
3.8 Are the required spectra included for all hits reported (i.e. ED, EDXA, SAED)?	☐	☐	NA
3.9 Recalculate the reported concentration on at least 10% of the results reported.			
3.9.1 Are the recalculated concentrations consistent with those reported?	☐	☐	NA

Additional Comments:

Data Review Checklist for the Verification and Validation of Transmission Electron Microscopy (TEM) Data Deliverables

4.0 Quality Control Verification & Validation (Level 2 and 3)	Yes	No	Comments
4.1 Blanks			
4.1.1 Are laboratory blanks (direct, indirect) prepared and analyzed at the required frequency?	☒	☐	The laboratory performed three blank analyses.
4.1.2 Are laboratory blank results within the specified criteria?	☒	☐	
4.1.2.1 If "no" then qualify the associated results in accordance with the Blank Result table in SOP QATS-70-091.			
4.2 Replicate Analyses			
4.2.1 Are replicate (second analyst on the same grids but different grid openings) sample analyses performed at the required frequency?	☐	☐	NA
4.2.2 Are replicate sample results within the specified acceptance limits?	☐	☐	NA
4.2.2.1 If "no" then qualify the associated results in accordance with the Analytical Variability Results table in SOP QATS-70-091.			
4.3 Duplicate Analyses			
4.3.1 Are duplicates (analysis of a second sample preparation obtained from the final filter) prepared and analyzed at the required frequency?	☐	☐	NA
4.3.2 Are duplicate sample results within the specified acceptance limits?	☐	☐	NA
4.3.2.1 If "no" then qualify the associated results in accordance with the Analytical Variability Results table in SOP QATS-70-091.			
4.4 Verified Analyses			
4.4.1 Are verified analyses (second analysis on same grids and grid openings) at the required frequency?	☐	☐	NA
4.4.2 Are sample verification results within the specified acceptance limits?	☐	☐	NA
4.4.2.1 If "no" then qualify the associated results in accordance with the Analytical Variability Results table in SOP QATS-70-091.			

Additional Comments:

Data Review Checklist for the Verification and Validation of Transmission Electron Microscopy (TEM) Data Deliverables

5.0 Calibration & Microscope Alignment Validation (Level 3)	Yes	No	Comments
5.1 Is evidence of the calibration of TEM Screen Magnification provided for all sample analyses?	☒	☐	Daily calibration on the H-7000FA instrument was not received affecting all three EPA Samples in Lab Job 141013.
5.1.1 Camera Constant Calibration?	☒	☐	
5.1.2 Calibration of the EDXA System?	☒	☐	
5.1.3 k-Factors?	☒	☐	
5.2 Are the calibration checks listed above performed at the required frequencies?	☐	☒	
5.3 Are the calibration checks within the specified criteria?	☒	☐	
5.4 Are all calibration checks traceable to the associated samples analyses?	☒	☐	
5.5 If required, are the following additional system checks provided:			
5.5.1 Beam Dose Check?	☒	☐	
5.5.2 Spot Size Check?	☒	☐	
5.5.3 Detector Resolution Check?	☒	☐	
5.5.4 Resolvable Na, Mg, and Si Peaks?	☒	☐	
5.5.5 If "no" then qualify the associated results in accordance with the Calibration Results table in SOP QATS-70-091.			
**6.0 Case Narrative Validation (Levels 2 & 3)**			
6.1 Does the data package narrative include descriptions of the following:			
6.1.1 Samples received (matrix/method)?	☒	☐	
6.1.2 Method/project requirement deviations?	☒	☐	
6.1.3 Example sample calculation?	☐	☒	
6.1.4 Laboratory blank contamination?	☐	☐	NA
6.1.5 Quality control analyses outside specified criteria?	☐	☒	
6.1.6 Any problems encountered and subsequent corrective action?	☐	☐	NA

Additional Comments:

Validator's Signature Lyndsay Gensler **Date** 06/16/2015

QA Review Shellee McGrath **Date** 06/19/2015

Appendix B

Qualified Result Forms

version 13-DRAFT

Sumas Mtn Asbestos [Sumas Mtn Asbestos]

National Asbestos Data Entry Spreadsheet (NADES) for Air & Dust Analysis by Superfund TEM

ANALYTICAL REPORT

FILE NAME: Sumas Mtn Asbestos_LabCor Inc._14394189_01-07-15_141013_TEM_D.xls

SAMPLE/ANALYSIS INFORMATION			
**Field Sample Number**	14394189	**Lab Sample Number**	141013-S1
**Media**	N/A	**Preparation**	Direct
**Sample Type**	Lot Blank	**Sample Status**	Analyzed
		**Analysis Date**	1/7/2015
**QA Sample Type**	Not QC	**Method SOP**	0
**Stopping Rule(s):**	Max Area = , Structures = , Sensitivity = 0.00E+00		
**Recording Rule(s):**	Min Aspect Ratio = , Min Length = µm, Min Width = µm		

* Chrysotile was not counted for all grid openings evaluated; this field is utilized only for the Libby site.

ANALYSIS PARAMETERS	
**Effective filter area (mm2)**	385
**F-factor**	1.00E+00
**Grid opening area (mm2)**	0.0101
**# GOs counted High Magnification**	10
**# GOs counted Low Magnification**	0
**Sensitivity (1/cc)**	
**Total Asbestos**	blank
**PCME**	blank
**Maximum Area Examined**	
**High Magnification**	1.0E-01
**Low Magnification**	0.0E+00

Number of Structures with Fatal Data Entry Errors 0 *(Structures with fatal errors are excluded from calculations below)*

Desired Confidence Interval (%):

Mineral Class	Number of Structures (a)	Loading on Filter (b) (s/mm^2)		% Poisson Confidence Interval for this Sample
**Total TEM Structures**				
Total Asbestos	0	0.0E+00	blank	blank - blank
Total Chrysotile (CH)	0	0.0E+00	blank	blank - blank
Total Amphibole	0	0.0E+00	blank	blank - blank
actinolite (AC)	0	0.0E+00	blank	blank - blank
amosite (AM)	0	0.0E+00	blank	blank - blank
anthophyllite (AN)	0	0.0E+00	blank	blank - blank
crocidolite (CR)	0	0.0E+00	blank	blank - blank
tremolite (TR)	0	0.0E+00	blank	blank - blank
winchite/richterite/tremolite /actinolite (WRTA)	0	0.0E+00	blank	blank - blank
other amphibole (OA)	0	0.0E+00	blank	blank - blank
Solid Soln: Amosite	0	0.0E+00	blank	blank - blank
Solid Soln: Trem-Act	0	0.0E+00	blank	blank - blank
other mineral class (OM)	0	0.0E+00	blank	blank - blank
**PCM Equivalent Structures (PCME)**				
Total Asbestos	0	0.0E+00	blank	blank - blank
Total Chrysotile (CH)	0	0.0E+00	blank	blank - blank
Total Amphibole	0	0.0E+00	blank	blank - blank
actinolite (AC)	0	0.0E+00	blank	blank - blank
amosite (AM)	0	0.0E+00	blank	blank - blank
anthophyllite (AN)	0	0.0E+00	blank	blank - blank
crocidolite (CR)	0	0.0E+00	blank	blank - blank
tremolite (TR)	0	0.0E+00	blank	blank - blank
winchite/richterite/tremolite /actinolite (WRTA)	0	0.0E+00	blank	blank - blank
other amphibole (OA)	0	0.0E+00	blank	blank - blank
Solid Soln: Amosite	0	0.0E+00	blank	blank - blank
Solid Soln: Trem-Act	0	0.0E+00	blank	blank - blank
other mineral class (OM)	0	0.0E+00	blank	blank - blank

Binning Rule Description:

Apply to fibers (F) only:
L ≥ 0.5µm, AR ≥ 3

No restrictions for other structure types.

ChiSq test for even filter loading for Total TE
(see Annex F2 in ISO 10312)

Filter loading is OK

Binning Rule Description:

Apply to all structures where Total column > 0

L > 5µm, W ≥ 0.25µm and W ≤ 3µm, AR ≥ 3

ChiSq test for even filter loading for PCMe
(see Annex F2 in ISO 10312)

[redacted]

(a) Based on countable structures only.
(b) Loading on Filter (s/mm^2) = N structures / (GOs Counted * GO Area). Results for indirect samples are based on the secondary filter.
(c) Air Concentration (s/cc) = (N structures * EFA) / (GOs Counted * GO Area * F-factor * Air Volume * 1000).
Dust Loading (s/cm2) = (N structures * EFA) / (GOs Counted * GO Area * F-factor * Dust Collection Area).

version 13-DRAFT

Sumas Mtn Asbestos [Sumas Mtn Asbestos]

National Asbestos Data Entry Spreadsheet (NADES) for Air & Dust Analysis by Superfund TEM
ANALYTICAL REPORT
FILE NAME: Sumas Mtn Asbestos_LabCor Inc._14394190_01-07-15_141013_TEM_D.xls

SAMPLE/ANALYSIS INFORMATION			
**Field Sample Number**	14394190	**Lab Sample Number**	141013-S2
**Media**	N/A	**Preparation**	Direct
**Sample Type**	Lot Blank	**Sample Status**	Analyzed
		**Analysis Date**	1/7/2015
**QA Sample Type**	Not QC	**Method SOP**	0
**Stopping Rule(s):**	Max Area = , Structures = , Sensitivity = 0.00E+00		
**Recording Rule(s):**	Min Aspect Ratio = , Min Length = µm, Min Width = µm		

* Chrysotile was not counted for all grid openings evaluated; this field is utilized only for the Libby site.

ANALYSIS PARAMETERS	
**Effective filter area (mm2)**	385
**F-factor**	1.00E+00
**Grid opening area (mm2)**	0.0101
**# GOs counted High Magnification**	10
**# GOs counted Low Magnification**	0
**Sensitivity (1/cc)**	
**Total Asbestos**	blank
**PCME**	blank
**Maximum Area Examined**	
**High Magnification**	1.0E-01
**Low Magnification**	0.0E+00

Number of Structures with Fatal Data Entry Errors 0 *(Structures with fatal errors are excluded from calculations below)*

Desired Confidence Interval (%):

Mineral Class	Number of Structures (a)	Loading on Filter (b) (s/mm^2)		% Poisson Confidence Interval for this Sample
**Total TEM Structures**				
Total Asbestos	0	0.0E+00	blank	blank - blank
Total Chrysotile (CH)	0	0.0E+00	blank	blank - blank
Total Amphibole	0	0.0E+00	blank	blank - blank
actinolite (AC)	0	0.0E+00	blank	blank - blank
amosite (AM)	0	0.0E+00	blank	blank - blank
anthophyllite (AN)	0	0.0E+00	blank	blank - blank
crocidolite (CR)	0	0.0E+00	blank	blank - blank
tremolite (TR)	0	0.0E+00	blank	blank - blank
winchite/richterite/tremolite /actinolite (WRTA)	0	0.0E+00	blank	blank - blank
other amphibole (OA)	0	0.0E+00	blank	blank - blank
Solid Soln: Amosite	0	0.0E+00	blank	blank - blank
Solid Soln: Trem-Act	0	0.0E+00	blank	blank - blank
other mineral class (OM)	0	0.0E+00	blank	blank - blank
**PCM Equivalent Structures (PCME)**				
Total Asbestos	0	0.0E+00	blank	blank - blank
Total Chrysotile (CH)	0	0.0E+00	blank	blank - blank
Total Amphibole	0	0.0E+00	blank	blank - blank
actinolite (AC)	0	0.0E+00	blank	blank - blank
amosite (AM)	0	0.0E+00	blank	blank - blank
anthophyllite (AN)	0	0.0E+00	blank	blank - blank
crocidolite (CR)	0	0.0E+00	blank	blank - blank
tremolite (TR)	0	0.0E+00	blank	blank - blank
winchite/richterite/tremolite /actinolite (WRTA)	0	0.0E+00	blank	blank - blank
other amphibole (OA)	0	0.0E+00	blank	blank - blank
Solid Soln: Amosite	0	0.0E+00	blank	blank - blank
Solid Soln: Trem-Act	0	0.0E+00	blank	blank - blank
other mineral class (OM)	0	0.0E+00	blank	blank - blank

Binning Rule Description:

Apply to fibers (F) only:
L ≥ 0.5µm, AR ≥ 3

No restrictions for other structure types.

ChiSq test for even filter loading for Total TE
(see Annex F2 in ISO 10312)
Filter loading is OK

Binning Rule Description:

Apply to all structures where Total column > 0

L > 5µm, W ≥ 0.25µm and W ≤ 3µm, AR ≥ 3

ChiSq test for even filter loading for PCMe
(see Annex F2 in ISO 10312)

[redacted]

(a) Based on countable structures only.
(b) Loading on Filter (s/mm^2) = N structures / (GOs Counted * GO Area). Results for indirect samples are based on the secondary filter.
(c) Air Concentration (s/cc) = (N structures * EFA) / (GOs Counted * GO Area * F-factor * Air Volume * 1000).
Dust Loading (s/cm2) = (N structures * EFA) / (GOs Counted * GO Area * F-factor * Dust Collection Area).

version 13-DRAFT

Sumas Mtn Asbestos [Sumas Mtn Asbestos]

National Asbestos Data Entry Spreadsheet (NADES) for Air & Dust Analysis by Superfund TEM

ANALYTICAL REPORT

FILE NAME: Sumas Mtn Asbestos_LabCor Inc._14394191_01-07-15_141013_TEM_D.xls

SAMPLE/ANALYSIS INFORMATION			
**Field Sample Number**	14394191	**Lab Sample Number**	141013-S3
**Media**	N/A	**Preparation**	Direct
**Sample Type**	Lot Blank	**Sample Status**	Analyzed
		**Analysis Date**	1/7/2015
**QA Sample Type**	Not QC	**Method SOP**	0
**Stopping Rule(s):**	Max Area = , Structures = , Sensitivity = 0.00E+00		
**Recording Rule(s):**	Min Aspect Ratio = , Min Length = µm, Min Width = µm		

* Chrysotile was not counted for all grid openings evaluated; this field is utilized only for the Libby site.

ANALYSIS PARAMETERS	
**Effective filter area (mm2)**	385
**F-factor**	1.00E+00
**Grid opening area (mm2)**	0.0101
**# GOs counted High Magnification**	10
**# GOs counted Low Magnification**	0
**Sensitivity (1/cc)**	
**Total Asbestos**	blank
**PCME**	blank
**Maximum Area Examined**	
**High Magnification**	1.0E-01
**Low Magnification**	0.0E+00

Number of Structures with Fatal Data Entry Errors 0 *(Structures with fatal errors are excluded from calculations below)*

Desired Confidence Interval (%):

Mineral Class	Number of Structures (a)	Loading on Filter (b) (s/mm^2)		% Poisson Confidence Interval for this Sample
**Total TEM Structures**				
Total Asbestos	0	0.0E+00	blank	blank - blank
Total Chrysotile (CH)	0	0.0E+00	blank	blank - blank
Total Amphibole	0	0.0E+00	blank	blank - blank
actinolite (AC)	0	0.0E+00	blank	blank - blank
amosite (AM)	0	0.0E+00	blank	blank - blank
anthophyllite (AN)	0	0.0E+00	blank	blank - blank
crocidolite (CR)	0	0.0E+00	blank	blank - blank
tremolite (TR)	0	0.0E+00	blank	blank - blank
winchite/richterite/tremolite /actinolite (WRTA)	0	0.0E+00	blank	blank - blank
other amphibole (OA)	0	0.0E+00	blank	blank - blank
Solid Soln: Amosite	0	0.0E+00	blank	blank - blank
Solid Soln: Trem-Act	0	0.0E+00	blank	blank - blank
other mineral class (OM)	0	0.0E+00	blank	blank - blank
**PCM Equivalent Structures (PCME)**				
Total Asbestos	0	0.0E+00	blank	blank - blank
Total Chrysotile (CH)	0	0.0E+00	blank	blank - blank
Total Amphibole	0	0.0E+00	blank	blank - blank
actinolite (AC)	0	0.0E+00	blank	blank - blank
amosite (AM)	0	0.0E+00	blank	blank - blank
anthophyllite (AN)	0	0.0E+00	blank	blank - blank
crocidolite (CR)	0	0.0E+00	blank	blank - blank
tremolite (TR)	0	0.0E+00	blank	blank - blank
winchite/richterite/tremolite /actinolite (WRTA)	0	0.0E+00	blank	blank - blank
other amphibole (OA)	0	0.0E+00	blank	blank - blank
Solid Soln: Amosite	0	0.0E+00	blank	blank - blank
Solid Soln: Trem-Act	0	0.0E+00	blank	blank - blank
other mineral class (OM)	0	0.0E+00	blank	blank - blank

Binning Rule Description:

Apply to fibers (F) only:
L ≥ 0.5µm, AR ≥ 3

No restrictions for other structure types.

ChiSq test for even filter loading for Total TE (see Annex F2 in ISO 10312)

Filter loading is OK

Binning Rule Description:

Apply to all structures where Total column > 0

L > 5µm, W ≥ 0.25µm and W ≤ 3µm, AR ≥ 3

ChiSq test for even filter loading for PCMe (see Annex F2 in ISO 10312)

[redacted]

(a) Based on countable structures only.

(b) Loading on Filter (s/mm^2) = N structures / (GOs Counted * GO Area). Results for indirect samples are based on the secondary filter.

(c) Air Concentration (s/cc) = (N structures * EFA) / (GOs Counted * GO Area * F-factor * Air Volume * 1000).

Dust Loading (s/cm2) = (N structures * EFA) / (GOs Counted * GO Area * F-factor * Dust Collection Area).
