# Supplementary material for: Comparison of soil sampling and analytical methods for asbestos at the Sumas Mountain Asbestos Site—Working towards a toolbox for better assessment
Source: PLoS One. 2017 Jul 31;12(7):e0180210. doi: 10.1371/journal.pone.0180210 (PMC5536314; doi:10.1371/journal.pone.0180210)
Supplement: S5 Text — (PDF) [file pone.0180210.s006.pdf]

CB&I
2700 Chandler Avenue, Building C
Las Vegas, NV 89120
Tel: +1 702 795 0515
Fax: +1 702 795 8210
www.CBI.com

July 10, 2015

Julie Wroble
EPA Site Manager Region 10
EPA Region 10
1200 6th Ave, Suite 900
Seattle, WA 98101

Document ID #: 3015-07102015-1

Dear Ms. Wroble:

EPA CONTRACT NUMBER EP-W-10-033
TASK ORDER NUMBER 3015
ASBESTOS QA SUPPORT

Enclosed please find the Release of Validated Data Report for the validation of Transmission Electron Microscopy (TEM) air sample data, Laboratory Job Number 141014. The thirty-two (32) air samples associated with these data were analyzed by Lab/Cor Portland, Inc., Portland, Oregon for the Sumas Mountain Asbestos Soil Project. This report and accompanying appendices are deliverables under Task 10 of the subject Task Order.

If you have any questions, please feel free to contact me.

Sincerely,

Lyndsay Gensler
Task Leader, QATS Program
CB&I Federal Services LLC
Phone: 702.895.8730
E-Mail Address: lyndsay.gensler@cbifederalservices.com

cc: Shari Myer, EPA-ASB, QATS Task Order Project Officer
Administrative Contracting Officer (letter only)

RELEASE OF VALIDATED DATA

DATE: July 10, 2015

SUBJECT: Review of Data for Laboratory Job Number: 141014

LABORATORY: Lab/Cor Portland, Inc., Portland, Oregon

FROM: Quality Assurance Technical Support (QATS) Program, Las Vegas, NV
CB&I Federal Services LLC

TO: Julie Wroble, Environmental Protection Agency

QATS reviewed the data for the following case:

Applicable SAP: NA

Chain-of-Custody Number: 10-100614-135155-0009

Method: Transmission Electron Microscopy (TEM) ISO 10312

Applicable Laboratory Modification(s): NA

Number and Type of Samples: 32 Air Samples

EPA Sample Numbers: 14394145, 14394146, 14394147, 14394148, 14394150, 14394151, 14394153, 14394154, 14394156, 14394157, 14394158, 14394161, 14394162, 14394163, 14394164, 14394165, 14394169, 14394170, 14394171, 14394173, 14394174, 14394175, 14394176, 14394178, 14394179, 14394180, 14394181, 14394182, 14394183, 14394186, 14394187, 14394188.

Note that sample 14394152 was not analyzed as it was rejected by the laboratory due to issues related to the backing pad; therefore, it is not included in this validated data summary.

VALIDATION SUMMARY

Thirty-two (32) air samples from Laboratory Job Number 141014 were collected on 09/30/2014, 10/01/2014, and 10/02/2014 and hand-delivered to Lab/Cor Portland, Inc. in Portland, OR for TEM analysis by ISO 10312. The samples were received at the laboratory intact on 12/11/2014, and were analyzed between 01/15/2015 and 02/24/2015.

Listed below are the Data Qualification Summary Table, EDD/Bench Sheet Discrepancy Table, Data Qualifier Table, and Reason Code Table.

DATA QUALIFICATION SUMMARY TABLE

Criteria Exceeded	EPA Sample ID	Validation Qualifier	Reason Code
No daily calibration for instrument H-7000 on 01/16/2015	14394154<br>14394156	J/UJ	MC
Daily calibration exceeded Al peak upper acceptance limit for instrument H-7000 on 01/21/2015	14394186<br>14394187	J	MC
k-Factors were not performed at the required frequency (last on 01/24/2014) for instrument H-7000	14394154<br>14394165<br>14394154 RS	J	IC
Component structure was not properly recorded.	14394176	J	ID
Only one (1) grid was evaluated	14394150 (field blank)	UJ	DL
RD QC value outside of lower 95% confidence limit.	14394181<br>14394181 RD	J	DR

EDD/BENCH SHEET DISCREPANCY TABLE

EPA Sample ID	C# *	Method/Matrix	Lab. Job No.	Analysis Date	Discrepancy
14394153	02	ISO 10312/Air	1414014	01/15/2015	Analysis date incorrectly listed as 01/16/2015 on Direct Summary Data Final Report. Correct date is 01/15/2015 as listed on NADES.<br>Photo IDs in comments incorrectly listed as "H39…" (The correct IDs are "H38…").
14394169	02	ISO 10312/Air	1414014	02/18/2015	Photo ID in comments incorrectly listed as H38527 (The correct ID is H38723).
14394187	02	ISO 10312/Air	1414014	01/21-22/2015	Analyst/Date #2 listed as 01/22/2014 on NADES. The correct date is 01/22/2015.
14394154	02	ISO 10312/Air	1414014	01/16/2015	The structure type indicated on the NADES worksheet (CR10) and the ISO 10312 – Direct Raw Data Report (CR5-0) differ for total structure #54.
14394176	02	ISO 10312/Air	1414014	02/01/2015	The NADES worksheet is missing an MR structure at Grid 1, Grid Opening 10, as recorded on the ISO 10312 – Direct Raw Data Report as total structure #10. As a result, the NADES worksheet lists 12 total structures while the raw data identifies 13.
14394153 RD<br>14394154 RS<br>14394181 RD	02	ISO 10312/Air	1414014	02/24/2015,<br>02/23/2015,<br>02/24/2015	The EDD indicates magnification as 15,941, whereas the 02/20/2015 calibration indicates a magnification of 16,379 for the H-7000 instrument.

'*' The EDD correction number in column 2. (i.e., C0, C1, C2, etc..)

DATA QUALIFIER TABLE

Qualifier	Definition
**J**	The result is estimated. The associated numerical value is an approximation.
**UJ**	The non-detect result may be inaccurate or imprecise due to the quality of the data generated because certain QC criteria were not met.
**R**	The sample results are rejected due to serious deficiencies.
**X**	Validator defined.

TEM REASON CODE TABLE

Reason Code	Definition
**MC**	Structure/fiber counts and recorded structure dimensions may be inaccurate due to improper or infrequent scope alignment and/or magnification calibrations.
**IC**	Identification by elemental composition or diffraction pattern may be inaccurate due to improper or infrequent EDXA or camera constant calibration.
**PA**	Structure/fiber counts and reported concentrations may be inaccurate due to improper or infrequent calibration of the plasma asher.
**DR**	The reported concentrations or structure/fiber identification may be inaccurate due to infrequent or discordant intra- and/or inter-analyst, laboratory duplicate, and/or reference material analyses.
**SC**	The reported concentration may be inaccurate due to the condition of samples upon receipt at the laboratory.
**DL**	The area analyzed, structures counted, or AS do not meet the requirements specified in the applicable SAP Analytical Summary.
**ID**	The asbestos identification and concentrations may be inaccurate because the recorded structure types are not consistent with those described in the applicable TEM Method and/or laboratory modification(s).

VALIDATION PROCESS

The samples for Laboratory Job Number 141014 were collected from the subject site on 09/30/2014, 10/01/2014, and 10/02/2014. All samples were prepared and analyzed in accordance with TEM ISO 10312. CB&I's Quality Assurance Technical Support (QATS) Program performed validation and a transcription check in accordance with method-specific data validation SOPs. QATS preparation of this report was performed under Technical Direction 03, Task 10, of Task Order 3015.

The sample results on bench sheets and other supporting documents provided in the hardcopy deliverables were compared to the entries in the associated laboratory method-specific EDDs (where applicable) to ensure that the reported results are complete, compliant with the specified methodology, and accurate. Additional support information provided in this data validation report include the QATS Data Review Checklist used to document the data validation process (see Appendix A); and the sample results as reported by the laboratory, with qualifiers as applicable (see Appendix B).

TEM VALIDATION SUMMARY

1. **DATA PACKAGE INVENTORY AND SAMPLE RECEIPT:** The data package included a narrative, Chain-of-Custody (COC) record, EDD files, raw data (bench sheets), and QC samples. The samples were properly packaged, sealed, undamaged, and labeled upon receipt at the laboratory. The COC record was reviewed and found to be acceptable.

2. **SAMPLE PREPARATION:** The appropriate preparation documents were provided.

3. **EQUIPMENT CALIBRATION AND PERFORMANCE CHECKS (i.e., daily microscope alignment, screen magnification, EDS calibration, and sensitivity checks):** The equipment alignment and calibration documentation provided show that in general, calibrations were performed at the correct frequency, indicating that the instruments were in proper working order during the time of sample analyses. There were three exceptions: The k-factors provided by the laboratory were performed on 01/24/2014 for instrument (scope) H-7000 and on 07/25/2014 for instrument (scope) JOEL 1200, more than six months prior to the analyses of the samples in this SDG. The laboratory's QAPP states for k-factors, "Calibration is performed on a biyearly basis, the first week of January and the first week of July." QATS requested the January 2015 k-factors from the laboratory; however, k-factors calculated on 03/30/2015 for Scope H-7000 and 05/21/2015 for Scope JOEL 1200 were received. As a result of the described calibration deficiencies, three TEM amphibole results reported in this SDG are qualified "J" due to the k-factor calibration. Additionally, the daily calibration exceeded the Al peak upper acceptance limit for instrument H-7000 on 01/21/2015, and no daily calibration was performed for instrument H-7000 on 01/16/2015 or for instrument JOEL 1200 on 03/29/2015 resulting in qualification of the results for samples analyzed on those dates.

4. **ANALYTICAL SENSITIVITY:** For all but one sample, a sufficient number of grid openings were analyzed to achieve the required analytical sensitivity and/or the appropriate stopping rule was invoked. The exception is EPA Sample No. 14394150, a field blank, in which only one grid was evaluated. As a result, the reported result for this field blank is qualified "UJ".

5. **STRUCTURE RECORDING AND ASBESTOS IDENTIFICATION:** The structure recording and asbestos identification were found to be acceptable, with the exception of EPA Sample No. 14394176 which excluded an "MR" component structure on the NADES worksheet though indicated on the ISO 10312 – Direct Raw Data report. As a result, the NADES worksheet reported one less total structure than was recorded in the raw data and caused this sample result to be qualified "J".

6. **BLANK ANALYSIS:** The laboratory performed and reported three blank analyses with this sample set. There were no structures observed.

7. **ANALYTICAL VARIABILITY:** The laboratory performed two recount different (RD) analyses on EPA Sample Nos. 14394153 and 14394181 and one recount same (RS) analysis on EPA Sample No. 14394154. All QC samples passed the established QC criteria with the exception of the RD analysis on EPA Sample No. 14394181, for which the value was outside the lower 95% confidence limit. EPA Sample No. 14394181 and 14394181 RD are qualified "J".

8. **LABORATORY MODIFICATIONS:** NA

9. **OVERALL ASSESSMENT OF DATA:** For 25 of the 32 samples, the reported results were acceptable with no qualification applied. Qualifiers were assigned to several sample results due to daily calibration and k-factor discrepancies, QC samples failing requirements, and other factors, as detailed in the Data Qualification Summary Table. EDD/Bench Sheet discrepancies were also identified in several samples, as detailed in the EDD/Bench Sheet Discrepancy Table.

REVIEWED BY: Lyndsay Gensler DATE: 07/08/2015

Appendix A

Data Review Checklist

Data Review Checklist for the Verification and Validation of Transmission Electron Microscopy (TEM) Data Deliverables

**Project Name: Sumas Mtn Asbestos Project**	**Case or Sample Set ID: 141014**
**Number of Samples/Matrix: 32 Air Samples**	**COC Number: 10-100614-135155-0009**
**TEM Analytical Method: ISO 10312**	**Level of Validation (Circle one): 1 2 [3] Other**

**1.0 Data Package Inventory**	**Yes**	**No**	**Comments**
1.1 Were the project-specific requirements (i.e. acceptance criteria & analytical sensitivities) provided by the client prior to the initiation of validation activities?	☐	☒	A request was made to the laboratory on 06/04/2015 for the laboratory's QAPP and/or SOPs documenting the frequency of the different TEM calibrations. The documents were received on 06/18/2015.
1.2 Did the received hard copy deliverables contain all the necessary components:			
1.2.1 Case Narrative (Level 1, 2 & 3)?	☒	☐	
1.2.2 Chain-of-Custody (Level 1, 2 & 3)?	☒	☐	
1.2.3 Form I or equivalent (Level 1, 2 & 3)?	☒	☐	
1.2.4 Raw Data - Count Sheets (Level 1, 2 & 3)?	☒	☐	
1.2.5 QC Sample Data (Level 2 & 3):	☒	☐	The laboratory performed three laboratory blank (LB) analyses, two recount different (RD) analyses on EPA Sample Nos. 14394153 and 14394181 and one recount same (RS) analysis on EPA Sample No. 14394154.
1.2.5.1 Blank(s)?	☒	☐	
1.2.5.2 Replicate(s)?	☒	☐	
1.2.5.3 Duplicate(s)?	☒	☐	
1.2.5.4 Verified Analysis?	☐	☒	
1.2.6 Calibration Data (Level 3)?	☒	☐	
1.2.7 Communication Records (Level 1, 2 & 3)?	☐	☒	
1.2.8 Miscellaneous?	☐	☐	NA
1.3 Were the necessary components received to perform the requested level of validation?	☒	☐	
**2.0 Chain-of-Custody Information Verification (Level 1, 2 & 3)**			
2.1 Were the following information recorded in the hard copy electronic deliverables (if applicable) consistent with the information recorded on the COC:			One sample (EPA Sample No. 14394152) was received though not analyzed as it was rejected by the laboratory per the Analysis Notes on the Analysis Report Cover Final Report.
2.1.1 COC Number?	☒	☐	
2.1.2 Case or Sample Set Number?	☒	☐	
2.1.3 EPA Sample ID?	☒	☐	
2.1.4 Date/Time Collected?	☒	☐	
2.1.5 Sample Volume?	☒	☐	
2.1.6 Sample Matrix?	☒	☐	
2.1.7 Analyses (Method)?	☒	☐	
2.1.8 Date/Time Received?	☒	☐	
2.1.9 Other (describe)?	☐	☐	
2.2 Were the COC records signed and dated upon receipt?	☒	☐	

Additional Comments:

Data Review Checklist for the Verification and Validation of Transmission Electron Microscopy (TEM) Data Deliverables

3.0 Sample Result Verification & Validation (Level 1, 2 & 3)	Yes	No	Comments
3.1 Is the sample preparation method documented and final sample volume recorded?	☒	☐	
3.2 Is the correct number of grid openings used to achieve the specified analytical sensitivity?	☐	☒	For EPA Sample No. 1434150, a field blank, only one grid (G1) was evaluated.
3.3 Verify that the following information from the laboratory's bench sheets have been transcribed correctly:			EPA Sample No. 14394176 excluded an “MR” component structure on the NADES worksheet as indicated on the ISO 10312 – Direct Raw Data report as total structure #10. As a result, the NADES worksheet reported one less total structure than was recorded in the raw data.
3.3.1.1 Grid identification?	☐	☒	
3.3.1.2 Grid opening?	☐	☒	
3.3.1.3 Structure type?	☐	☒	
3.3.1.4 Number of primary and secondary structures?	☐	☒	
3.3.1.5 Length and width dimensions?	☐	☒	
3.3.1.6 Structure identification?	☐	☒	
3.3.1.7 Mineral type?	☐	☒	
3.4 Are overloaded samples correctly reported to the specified percent obscuration (i.e. 10%, 25%)?	☐	☐	NA
3.5 If overloading occurs, are samples prepared by an alternate method (i.e. indirect preparation)?	☐	☐	NA
3.6 Verify that the following information is documented correctly:			
3.6.1 Magnification?	☒	☐	
3.6.2 Field or QC sample type?	☒	☐	
3.6.3 Number of grids prepared?	☒	☐	
3.6.4 Filter area in (mm^2)?	☒	☐	
3.6.5 Analysis date?	☒	☐	
3.7 Verify the totals reported on the count sheets for the various types of structures. These may include:			
3.7.1 Total EPA Structures	☒	☐	See checklist item 3.3 above.
3.7.2 PCMe Structures	☒	☐	
3.7.3 AHERA Structures	☐	☐	NA
3.7.4 Berman Crump Structures	☐	☐	NA
3.8 Are the required spectra included for all hits reported (i.e. ED, EDXA, SAED)?	☒	☐	
3.9 Recalculate the reported concentration on at least 10% of the results reported.			
3.9.1 Are the recalculated concentrations consistent with those reported?	☒	☐	

Additional Comments:

Data Review Checklist for the Verification and Validation of Transmission Electron Microscopy (TEM) Data Deliverables

4.0 Quality Control Verification & Validation (Level 2 and 3)	Yes	No	Comments
4.1 Blanks			
4.1.1 Are laboratory blanks (direct, indirect) prepared and analyzed at the required frequency?	☒	☐	The laboratory performed three laboratory blank (LB) analyses.
4.1.2 Are laboratory blank results within the specified criteria?	☒	☐	
4.1.2.1 If "no" then qualify the associated results in accordance with the Blank Result table in SOP QATS-70-091.			
4.2 Replicate Analyses			
4.2.1 Are replicate (second analyst on the same grids but different grid openings) sample analyses performed at the required frequency?	☒	☐	The laboratory performed one RS analysis on EPA Sample No. 14394154.
4.2.2 Are replicate sample results within the specified acceptance limits?	☒	☐	
4.2.2.1 If "no" then qualify the associated results in accordance with the Analytical Variability Results table in SOP QATS-70-091.			
4.3 Duplicate Analyses			
4.3.1 Are duplicates (analysis of a second sample preparation obtained from the final filter) prepared and analyzed at the required frequency?	☒	☐	The laboratory performed two RD analyses on EPA Sample Nos. 14394153 and 14394181.
4.3.2 Are duplicate sample results within the specified acceptance limits?	☐	☒	The QC value for the RD analysis for EPA Sample No. 14394181 was outside of lower 95% confidence limit.
4.3.2.1 If "no" then qualify the associated results in accordance with the Analytical Variability Results table in SOP QATS-70-091.			
4.4 Verified Analyses			
4.4.1 Are verified analyses (second analysis on same grids and grid openings) at the required frequency?	☐	☐	NA
4.4.2 Are sample verification results within the specified acceptance limits?	☐	☐	NA
4.4.2.1 If "no" then qualify the associated results in accordance with the Analytical Variability Results table in SOP QATS-70-091.			

Additional Comments:

Data Review Checklist for the Verification and Validation of Transmission Electron Microscopy (TEM) Data Deliverables

5.0 Calibration & Microscope Alignment Validation (Level 3)	Yes	No	Comments
5.1 Is evidence of the calibration of TEM Screen Magnification provided for all sample analyses?	☒	☐	Daily calibration on instrument H-7000 instrument was not performed on 01/16/2015 affecting EPA Sample Nos. 14394154 and 14394156. Daily calibration on instrument JOEL 1200 was not performed on 03/29/2015 affecting the LB samples.
5.1.1 Camera Constant Calibration?	☒	☐	
5.1.2 Calibration of the EDXA System?	☒	☐	
5.1.3 k-Factors?	☒	☐	
5.2 Are the calibration checks listed above performed at the required frequencies?	☐	☒	
5.3 Are the calibration checks within the specified criteria?	☐	☒	The k-Factors provided by the laboratory were performed on 01/24/2014) for instrument H-7000 and 07/25/2014 for instrument JOEL 1200, more than six months prior to the analyses of the samples in this SDG. The laboratory's QAPP states for k-factors "Calibration is performed on a biyearly basis, the first week of January and the first week of July." Amphibole results for three samples were affected.
5.4 Are all calibration checks traceable to the associated samples analyses?	☒	☐	
5.5 If required, are the following additional system checks provided:			
5.5.1 Beam Dose Check?	☒	☐	
5.5.2 Spot Size Check?	☒	☐	
5.5.3 Detector Resolution Check?	☒	☐	
5.5.4 Resolvable Na, Mg, and Si Peaks?	☒	☐	
5.5.5 If "no" then qualify the associated results in accordance with the Calibration Results table in SOP QATS-70-091.			Daily calibration exceeded Al peak upper acceptance limit for instrument H-7000 on 01/21/2015 affecting EPA Sample Nos. 14394186 and 14394187.<br><br>As a result of all the above listed calibration discrepancies, qualifiers were assigned.
**6.0 Case Narrative Validation (Levels 2 & 3)**			
6.1 Does the data package narrative include descriptions of the following:			
6.1.1 Samples received (matrix/method)?	☒	☐	
6.1.2 Method/project requirement deviations?	☒	☐	
6.1.3 Example sample calculation?	☐	☒	
6.1.4 Laboratory blank contamination?	☐	☐	NA
6.1.5 Quality control analyses outside specified criteria?	☐	☒	
6.1.6 Any problems encountered and subsequent corrective action?	☒	☐	
**Additional Comments:**			

Validator's Signature Lyndsay Gensler **Date** 06/16/2015

QA Review Shellee McGrath **Date** 07/03/2015

Appendix B

Qualified Result Forms

version 13-DRAFT

Sumas Mtn Asbestos Soil [SFP-078A]

National Asbestos Data Entry Spreadsheet (NADES) for Air & Dust Analysis by Superfund TEM

ANALYTICAL REPORT

FILE NAME: SFP-078A_LabCor Inc._14394145_01-15-15_141014_TEM_D.xls

SAMPLE/ANALYSIS INFORMATION			
**Field Sample Number**	14394145	**Lab Sample Number**	141014-S1
**Media**	Air	**Preparation**	Direct
**Sample Type**	Field Sample	**Sample Status**	Analyzed
**Air Volume (L)**	365.885	**Analysis Date**	1/15/2015
**QA Sample Type**	Not QC	**Method SOP**	0
**Stopping Rule(s):**	Max Area = , Structures = 100, Sensitivity = 3.00E-03		
**Recording Rule(s):**	Min Aspect Ratio = , Min Length = 0.5µm, Min Width = µm		

* Chrysotile was not counted for all grid openings evaluated; this field is utilized only for the Libby site.

ANALYSIS PARAMETERS	
**Effective filter area (mm2)**	385
**F-factor**	1.00E+00
**Grid opening area (mm2)**	0.0104
**# GOs counted High Magnification**	34
**# GOs counted Low Magnification**	0
**Sensitivity (1/cc)**	
**Total Asbestos**	0.0029701
**PCME**	0.0029701
**Maximum Area Examined**	
**High Magnification**	3.5E-01
**Low Magnification**	0.0E+00

Number of Structures with Fatal Data Entry Errors 0 *(Structures with fatal errors are excluded from calculations below)*

Desired Confidence Interval (%):

Mineral Class	Number of Structures (a)	Loading on Filter (b) (s/mm²)	Air Conc (c) (s/cc)	% Poisson Confidence Interval for this Sample
**Total TEM Structures**				
Total Asbestos	0	0.0E+00	0.0E+00	0.0E+00 - 2.1E-03
Total Chrysotile (CH)	0	0.0E+00	0.0E+00	0.0E+00 - 2.1E-03
Total Amphibole	0	0.0E+00	0.0E+00	0.0E+00 - 2.1E-03
actinolite (AC)	0	0.0E+00	0.0E+00	0.0E+00 - 2.1E-03
amosite (AM)	0	0.0E+00	0.0E+00	0.0E+00 - 2.1E-03
anthophyllite (AN)	0	0.0E+00	0.0E+00	0.0E+00 - 2.1E-03
crocidolite (CR)	0	0.0E+00	0.0E+00	0.0E+00 - 2.1E-03
tremolite (TR)	0	0.0E+00	0.0E+00	0.0E+00 - 2.1E-03
winchite/richterite/tremolite /actinolite (WRTA)	0	0.0E+00	0.0E+00	0.0E+00 - 2.1E-03
other amphibole (OA)	0	0.0E+00	0.0E+00	0.0E+00 - 2.1E-03
Solid Soln: Amosite	0	0.0E+00	0.0E+00	0.0E+00 - 2.1E-03
Solid Soln: Trem-Act	0	0.0E+00	0.0E+00	0.0E+00 - 2.1E-03
other mineral class (OM)	0	0.0E+00	0.0E+00	0.0E+00 - 2.1E-03
**PCM Equivalent Structures (PCME)**				
Total Asbestos	0	0.0E+00	0.0E+00	0.0E+00 - 2.1E-03
Total Chrysotile (CH)	0	0.0E+00	0.0E+00	0.0E+00 - 2.1E-03
Total Amphibole	0	0.0E+00	0.0E+00	0.0E+00 - 2.1E-03
actinolite (AC)	0	0.0E+00	0.0E+00	0.0E+00 - 2.1E-03
amosite (AM)	0	0.0E+00	0.0E+00	0.0E+00 - 2.1E-03
anthophyllite (AN)	0	0.0E+00	0.0E+00	0.0E+00 - 2.1E-03
crocidolite (CR)	0	0.0E+00	0.0E+00	0.0E+00 - 2.1E-03
tremolite (TR)	0	0.0E+00	0.0E+00	0.0E+00 - 2.1E-03
winchite/richterite/tremolite /actinolite (WRTA)	0	0.0E+00	0.0E+00	0.0E+00 - 2.1E-03
other amphibole (OA)	0	0.0E+00	0.0E+00	0.0E+00 - 2.1E-03
Solid Soln: Amosite	0	0.0E+00	0.0E+00	0.0E+00 - 2.1E-03
Solid Soln: Trem-Act	0	0.0E+00	0.0E+00	0.0E+00 - 2.1E-03
other mineral class (OM)	0	0.0E+00	0.0E+00	0.0E+00 - 2.1E-03

Binning Rule Description:

Apply to fibers (F) only:
L ≥ 0.5µm, AR ≥ 3

No restrictions for other structure types.

ChiSq test for even filter loading for Total TE (see Annex F2 in ISO 10312)

Filter loading is OK

Binning Rule Description:

Apply to all structures where Total column > 0

L > 5µm, W ≥ 0.25µm and W ≤ 3µm, AR ≥ 3

ChiSq test for even filter loading for PCMe (see Annex F2 in ISO 10312)

(a) Based on countable structures only.
(b) Loading on Filter (s/mm²) = N structures / (GOs Counted * GO Area). Results for indirect samples are based on the secondary filter.
(c) Air Concentration (s/cc) = (N structures * EFA) / (GOs Counted * GO Area * F-factor * Air Volume * 1000).
Dust Loading (s/cm2) = (N structures * EFA) / (GOs Counted * GO Area * F-factor * Dust Collection Area).

version 13-DRAFT

Sumas Mtn Asbestos Soil [SFP-078A]

National Asbestos Data Entry Spreadsheet (NADES) for Air & Dust Analysis by Superfund TEM

ANALYTICAL REPORT

FILE NAME: SFP-078A_LabCor Inc._14394146_01-15-15_141014_TEM_D.xls

SAMPLE/ANALYSIS INFORMATION

**Field Sample Number**	14394146	**Lab Sample Number**	141014-S2
**Media**	Air	**Preparation**	Direct
**Sample Type**	Field Sample	**Sample Status**	Analyzed
**Air Volume (L)**	231.34	**Analysis Date**	1/15/2015
**QA Sample Type**	Not QC	**Method SOP**	0

Stopping Rule(s): Max Area = , Structures = 100, Sensitivity = 3.00E-03

Recording Rule(s): Min Aspect Ratio = , Min Length = 0.5µm, Min Width = µm

* Chrysotile was not counted for all grid openings evaluated; this field is utilized only for the Libby site.

ANALYSIS PARAMETERS

**Effective filter area (mm2)**	385
**F-factor**	1.00E+00
**Grid opening area (mm2)**	0.0104
**# GOs counted High Magnification**	54
**# GOs counted Low Magnification**	0
**Sensitivity (1/cc)**	
**Total Asbestos**	0.0029577
**PCME**	0.0029577
**Maximum Area Examined**	
**High Magnification**	5.6E-01
**Low Magnification**	0.0E+00

Number of Structures with Fatal Data Entry Errors 0 *(Structures with fatal errors are excluded from calculations below)*

Desired Confidence Interval (%):

Mineral Class	Number of Structures (a)	Loading on Filter (b) (s/mm^2)	Air Conc (c) (s/cc)	% Poisson Confidence Interval for this Sample
**Total TEM Structures**				
Total Asbestos	0	0.0E+00	0.0E+00	0.0E+00 - 2.1E-03
Total Chrysotile (CH)	0	0.0E+00	0.0E+00	0.0E+00 - 2.1E-03
Total Amphibole	0	0.0E+00	0.0E+00	0.0E+00 - 2.1E-03
actinolite (AC)	0	0.0E+00	0.0E+00	0.0E+00 - 2.1E-03
amosite (AM)	0	0.0E+00	0.0E+00	0.0E+00 - 2.1E-03
anthophyllite (AN)	0	0.0E+00	0.0E+00	0.0E+00 - 2.1E-03
crocidolite (CR)	0	0.0E+00	0.0E+00	0.0E+00 - 2.1E-03
tremolite (TR)	0	0.0E+00	0.0E+00	0.0E+00 - 2.1E-03
winchite/richterite/tremolite /actinolite (WRTA)	0	0.0E+00	0.0E+00	0.0E+00 - 2.1E-03
other amphibole (OA)	0	0.0E+00	0.0E+00	0.0E+00 - 2.1E-03
Solid Soln: Amosite	0	0.0E+00	0.0E+00	0.0E+00 - 2.1E-03
Solid Soln: Trem-Act	0	0.0E+00	0.0E+00	0.0E+00 - 2.1E-03
other mineral class (OM)	0	0.0E+00	0.0E+00	0.0E+00 - 2.1E-03
**PCM Equivalent Structures (PCME)**				
Total Asbestos	0	0.0E+00	0.0E+00	0.0E+00 - 2.1E-03
Total Chrysotile (CH)	0	0.0E+00	0.0E+00	0.0E+00 - 2.1E-03
Total Amphibole	0	0.0E+00	0.0E+00	0.0E+00 - 2.1E-03
actinolite (AC)	0	0.0E+00	0.0E+00	0.0E+00 - 2.1E-03
amosite (AM)	0	0.0E+00	0.0E+00	0.0E+00 - 2.1E-03
anthophyllite (AN)	0	0.0E+00	0.0E+00	0.0E+00 - 2.1E-03
crocidolite (CR)	0	0.0E+00	0.0E+00	0.0E+00 - 2.1E-03
tremolite (TR)	0	0.0E+00	0.0E+00	0.0E+00 - 2.1E-03
winchite/richterite/tremolite /actinolite (WRTA)	0	0.0E+00	0.0E+00	0.0E+00 - 2.1E-03
other amphibole (OA)	0	0.0E+00	0.0E+00	0.0E+00 - 2.1E-03
Solid Soln: Amosite	0	0.0E+00	0.0E+00	0.0E+00 - 2.1E-03
Solid Soln: Trem-Act	0	0.0E+00	0.0E+00	0.0E+00 - 2.1E-03
other mineral class (OM)	0	0.0E+00	0.0E+00	0.0E+00 - 2.1E-03

Binning Rule Description: (Total TEM Structures)

Apply to fibers (F) only:
L ≥ 0.5µm, AR ≥ 3

No restrictions for other structure types.

ChiSq test for even filter loading for Total TE
(see Annex F2 in ISO 10312)

Filter loading is OK

Binning Rule Description: (PCME)

Apply to all structures where Total column > 0

L > 5µm, W ≥ 0.25µm and W ≤ 3µm, AR ≥ 3

ChiSq test for even filter loading for PCMe
(see Annex F2 in ISO 10312)

[redacted]

(a) Based on countable structures only.

(b) Loading on Filter (s/mm^2) = N structures / (GOs Counted * GO Area). Results for indirect samples are based on the secondary filter.

(c) Air Concentration (s/cc) = (N structures * EFA) / (GOs Counted * GO Area * F-factor * Air Volume * 1000).
Dust Loading (s/cm2) = (N structures * EFA) / (GOs Counted * GO Area * F-factor * Dust Collection Area).

version 13-DRAFT

Sumas Mtn Asbestos Soil [SFP-078A]

National Asbestos Data Entry Spreadsheet (NADES) for Air & Dust Analysis by Superfund TEM

ANALYTICAL REPORT

FILE NAME: SFP-078A_LabCor Inc._14394147_01-15-15_141014_TEM_D.xls

SAMPLE/ANALYSIS INFORMATION			
**Field Sample Number**	14394147	**Lab Sample Number**	141014-S3
**Media**	Air	**Preparation**	Direct
**Sample Type**	Field Sample	**Sample Status**	Analyzed
**Air Volume (L)**	202.02	**Analysis Date**	1/15/2015
**QA Sample Type**	Not QC	**Method SOP**	0
**Stopping Rule(s):**	Max Area = n/a, Structures = 100, Sensitivity = 3.00E-03		
**Recording Rule(s):**	Min Aspect Ratio = , Min Length = 0.5µm, Min Width = µm		

* Chrysotile was not counted for all grid openings evaluated; this field is utilized only for the Libby site.

ANALYSIS PARAMETERS	
**Effective filter area (mm2)**	385
**F-factor**	1.00E+00
**Grid opening area (mm2)**	0.0104
**# GOs counted High Magnification**	61
**# GOs counted Low Magnification**	0
**Sensitivity (1/cc)**	
**Total Asbestos**	0.0029983
**PCME**	0.0029983
**Maximum Area Examined**	
**High Magnification**	6.4E-01
**Low Magnification**	0.0E+00

Number of Structures with Fatal Data Entry Errors 0 *(Structures with fatal errors are excluded from calculations below)*

Desired Confidence Interval (%):

Mineral Class	Number of Structures (a)	Loading on Filter (b) (s/mm^2)	Air Conc (c) (s/cc)	% Poisson Confidence Interval for this Sample
**Total TEM Structures**				
Total Asbestos	0	0.0E+00	0.0E+00	0.0E+00 - 2.1E-03
Total Chrysotile (CH)	0	0.0E+00	0.0E+00	0.0E+00 - 2.1E-03
Total Amphibole	0	0.0E+00	0.0E+00	0.0E+00 - 2.1E-03
actinolite (AC)	0	0.0E+00	0.0E+00	0.0E+00 - 2.1E-03
amosite (AM)	0	0.0E+00	0.0E+00	0.0E+00 - 2.1E-03
anthophyllite (AN)	0	0.0E+00	0.0E+00	0.0E+00 - 2.1E-03
crocidolite (CR)	0	0.0E+00	0.0E+00	0.0E+00 - 2.1E-03
tremolite (TR)	0	0.0E+00	0.0E+00	0.0E+00 - 2.1E-03
winchite/richterite/tremolite /actinolite (WRTA)	0	0.0E+00	0.0E+00	0.0E+00 - 2.1E-03
other amphibole (OA)	0	0.0E+00	0.0E+00	0.0E+00 - 2.1E-03
Solid Soln: Amosite	0	0.0E+00	0.0E+00	0.0E+00 - 2.1E-03
Solid Soln: Trem-Act	0	0.0E+00	0.0E+00	0.0E+00 - 2.1E-03
other mineral class (OM)	0	0.0E+00	0.0E+00	0.0E+00 - 2.1E-03
**PCM Equivalent Structures (PCME)**				
Total Asbestos	0	0.0E+00	0.0E+00	0.0E+00 - 2.1E-03
Total Chrysotile (CH)	0	0.0E+00	0.0E+00	0.0E+00 - 2.1E-03
Total Amphibole	0	0.0E+00	0.0E+00	0.0E+00 - 2.1E-03
actinolite (AC)	0	0.0E+00	0.0E+00	0.0E+00 - 2.1E-03
amosite (AM)	0	0.0E+00	0.0E+00	0.0E+00 - 2.1E-03
anthophyllite (AN)	0	0.0E+00	0.0E+00	0.0E+00 - 2.1E-03
crocidolite (CR)	0	0.0E+00	0.0E+00	0.0E+00 - 2.1E-03
tremolite (TR)	0	0.0E+00	0.0E+00	0.0E+00 - 2.1E-03
winchite/richterite/tremolite /actinolite (WRTA)	0	0.0E+00	0.0E+00	0.0E+00 - 2.1E-03
other amphibole (OA)	0	0.0E+00	0.0E+00	0.0E+00 - 2.1E-03
Solid Soln: Amosite	0	0.0E+00	0.0E+00	0.0E+00 - 2.1E-03
Solid Soln: Trem-Act	0	0.0E+00	0.0E+00	0.0E+00 - 2.1E-03
other mineral class (OM)	0	0.0E+00	0.0E+00	0.0E+00 - 2.1E-03

Binning Rule Description:

Apply to fibers (F) only:
L ≥ 0.5µm, AR ≥ 3

No restrictions for other structure types.

ChiSq test for even filter loading for Total TE
(see Annex F2 in ISO 10312)

Filter loading is OK

Binning Rule Description:

Apply to all structures where Total column > 0

L > 5µm, W ≥ 0.25µm and W ≤ 3µm, AR ≥ 3

ChiSq test for even filter loading for PCMe
(see Annex F2 in ISO 10312)

[redacted]

(a) Based on countable structures only.

(b) Loading on Filter (s/mm^2) = N structures / (GOs Counted * GO Area). Results for indirect samples are based on the secondary filter

(c) Air Concentration (s/cc) = (N structures * EFA) / (GOs Counted * GO Area * F-factor * Air Volume * 1000).
Dust Loading (s/cm2) = (N structures * EFA) / (GOs Counted * GO Area * F-factor * Dust Collection Area).

version 13-DRAFT

Sumas Mtn Asbestos Soil [SFP-078A]

National Asbestos Data Entry Spreadsheet (NADES) for Air & Dust Analysis by Superfund TEM

ANALYTICAL REPORT

FILE NAME: SFP-078A_LabCor Inc._14394148_01-15-15_141014_TEM_D.xls

SAMPLE/ANALYSIS INFORMATION			
**Field Sample Number**	14394148	**Lab Sample Number**	141014-S4
**Media**	Air	**Preparation**	Direct
**Sample Type**	Field Sample	**Sample Status**	Analyzed
**Air Volume (L)**	288.99	**Analysis Date**	1/15/2015
**QA Sample Type**	Not QC	**Method SOP**	0
**Stopping Rule(s):**	Max Area = n/a, Structures = 100, Sensitivity = 3.00E-03		
**Recording Rule(s):**	Min Aspect Ratio = , Min Length = 0.5µm, Min Width = µm		

* Chrysotile was not counted for all grid openings evaluated; this field is utilized only for the Libby site.

ANALYSIS PARAMETERS	
**Effective filter area (mm2)**	385
**F-factor**	1.00E+00
**Grid opening area (mm2)**	0.0104
**# GOs counted High Magnification**	43
**# GOs counted Low Magnification**	0
**Sensitivity (1/cc)**	
**Total Asbestos**	0.0029733
**PCME**	0.0029733
**Maximum Area Examined**	
**High Magnification**	4.5E-01
**Low Magnification**	0.0E+00

Number of Structures with Fatal Data Entry Errors 0 *(Structures with fatal errors are excluded from calculations below)*

Desired Confidence Interval (%):

Mineral Class	Number of Structures (a)	Loading on Filter (b) (s/mm^2)	Air Conc (c) (s/cc)	% Poisson Confidence Interval for this Sample
**Total TEM Structures**				
Total Asbestos	3	6.7E+00	8.9E-03	8.0E-03 - 1.1E-02
Total Chrysotile (CH)	3	6.7E+00	8.9E-03	8.0E-03 - 1.1E-02
Total Amphibole	0	0.0E+00	0.0E+00	0.0E+00 - 2.1E-03
actinolite (AC)	0	0.0E+00	0.0E+00	0.0E+00 - 2.1E-03
amosite (AM)	0	0.0E+00	0.0E+00	0.0E+00 - 2.1E-03
anthophyllite (AN)	0	0.0E+00	0.0E+00	0.0E+00 - 2.1E-03
crocidolite (CR)	0	0.0E+00	0.0E+00	0.0E+00 - 2.1E-03
tremolite (TR)	0	0.0E+00	0.0E+00	0.0E+00 - 2.1E-03
winchite/richterite/tremolite /actinolite (WRTA)	0	0.0E+00	0.0E+00	0.0E+00 - 2.1E-03
other amphibole (OA)	0	0.0E+00	0.0E+00	0.0E+00 - 2.1E-03
Solid Soln: Amosite	0	0.0E+00	0.0E+00	0.0E+00 - 2.1E-03
Solid Soln: Trem-Act	0	0.0E+00	0.0E+00	0.0E+00 - 2.1E-03
other mineral class (OM)	0	0.0E+00	0.0E+00	0.0E+00 - 2.1E-03
**PCM Equivalent Structures (PCME)**				
Total Asbestos	1	2.2E+00	3.0E-03	2.1E-03 - 5.0E-03
Total Chrysotile (CH)	1	2.2E+00	3.0E-03	2.1E-03 - 5.0E-03
Total Amphibole	0	0.0E+00	0.0E+00	0.0E+00 - 2.1E-03
actinolite (AC)	0	0.0E+00	0.0E+00	0.0E+00 - 2.1E-03
amosite (AM)	0	0.0E+00	0.0E+00	0.0E+00 - 2.1E-03
anthophyllite (AN)	0	0.0E+00	0.0E+00	0.0E+00 - 2.1E-03
crocidolite (CR)	0	0.0E+00	0.0E+00	0.0E+00 - 2.1E-03
tremolite (TR)	0	0.0E+00	0.0E+00	0.0E+00 - 2.1E-03
winchite/richterite/tremolite /actinolite (WRTA)	0	0.0E+00	0.0E+00	0.0E+00 - 2.1E-03
other amphibole (OA)	0	0.0E+00	0.0E+00	0.0E+00 - 2.1E-03
Solid Soln: Amosite	0	0.0E+00	0.0E+00	0.0E+00 - 2.1E-03
Solid Soln: Trem-Act	0	0.0E+00	0.0E+00	0.0E+00 - 2.1E-03
other mineral class (OM)	0	0.0E+00	0.0E+00	0.0E+00 - 2.1E-03

Binning Rule Description:

Apply to fibers (F) only:
L ≥ 0.5µm, AR ≥ 3

No restrictions for other structure types.

ChiSq test for even filter loading for Total TE
(see Annex F2 in ISO 10312)

Filter loading is OK

Binning Rule Description:

Apply to all structures where Total column > 0

L > 5µm, W ≥ 0.25µm and W ≤ 3µm, AR ≥ 3

ChiSq test for even filter loading for PCMe
(see Annex F2 in ISO 10312)

Filter loading is OK

[redacted]

(a) Based on countable structures only.
(b) Loading on Filter (s/mm^2) = N structures / (GOs Counted * GO Area). Results for indirect samples are based on the secondary filter.
(c) Air Concentration (s/cc) = (N structures * EFA) / (GOs Counted * GO Area * F-factor * Air Volume * 1000).
Dust Loading (s/cm2) = (N structures * EFA) / (GOs Counted * GO Area * F-factor * Dust Collection Area).

version 13-DRAFT

Sumas Mtn Asbestos Soil [SFP-078A]

National Asbestos Data Entry Spreadsheet (NADES) for Air & Dust Analysis by Superfund TEM

ANALYTICAL REPORT

FILE NAME: SFP-078A_LabCor Inc._14394150_01-15-15_141014_TEM_D.xls

SAMPLE/ANALYSIS INFORMATION			
**Field Sample Number**	14394150	**Lab Sample Number**	141014-S5
**Media**	Air	**Preparation**	Direct
**Sample Type**	Field Blank	**Sample Status**	Analyzed
**Air Volume (L)**		**Analysis Date**	1/15/2015
**QA Sample Type**	Not QC	**Method SOP**	0
**Stopping Rule(s):**	Max Area = n/a, Structures = 100, Sensitivity = 3.00E-03		
**Recording Rule(s):**	Min Aspect Ratio = , Min Length = 0.5µm, Min Width = µm		

* Chrysotile was not counted for all grid openings evaluated; this field is utilized only for the Libby site.

ANALYSIS PARAMETERS	
**Effective filter area (mm2)**	385
**F-factor**	1.00E+00
**Grid opening area (mm2)**	0.0104
**# GOs counted High Magnification**	10
**# GOs counted Low Magnification**	0
**Sensitivity (1/cc)**	
**Total Asbestos**	blank
**PCME**	blank
**Maximum Area Examined**	
**High Magnification**	1.0E-01
**Low Magnification**	0.0E+00

Number of Structures with Fatal Data Entry Errors 0 *(Structures with fatal errors are excluded from calculations below)*

Desired Confidence Interval (%):

Mineral Class	Number of Structures (a)	Loading on Filter (b) (s/mm²)	Air Conc (c) (s/cc)	% Poisson Confidence Interval for this Sample
**Total TEM Structures**				
Total Asbestos	0 UJ	0.0E+00	blank RC =	blank - blank
Total Chrysotile (CH)	0	0.0E+00	blank	blank - blank
Total Amphibole	0	0.0E+00	blank DL	blank - blank
actinolite (AC)	0	0.0E+00	blank	blank - blank
amosite (AM)	0	0.0E+00	blank	blank - blank
anthophyllite (AN)	0	0.0E+00	blank	blank - blank
crocidolite (CR)	0	0.0E+00	blank	blank - blank
tremolite (TR)	0	0.0E+00	blank	blank - blank
winchite/richterite/tremolite /actinolite (WRTA)	0	0.0E+00	blank	blank - blank
other amphibole (OA)	0	0.0E+00	blank	blank - blank
Solid Soln: Amosite	0	0.0E+00	blank	blank - blank
Solid Soln: Trem-Act	0	0.0E+00	blank	blank - blank
other mineral class (OM)	0	0.0E+00	blank	blank - blank
**PCM Equivalent Structures (PCME)**				
Total Asbestos	0	0.0E+00	blank	blank - blank
Total Chrysotile (CH)	0	0.0E+00	blank	blank - blank
Total Amphibole	0	0.0E+00	blank	blank - blank
actinolite (AC)	0	0.0E+00	blank	blank - blank
amosite (AM)	0	0.0E+00	blank	blank - blank
anthophyllite (AN)	0	0.0E+00	blank	blank - blank
crocidolite (CR)	0	0.0E+00	blank	blank - blank
tremolite (TR)	0	0.0E+00	blank	blank - blank
winchite/richterite/tremolite /actinolite (WRTA)	0	0.0E+00	blank	blank - blank
other amphibole (OA)	0	0.0E+00	blank	blank - blank
Solid Soln: Amosite	0	0.0E+00	blank	blank - blank
Solid Soln: Trem-Act	0	0.0E+00	blank	blank - blank
other mineral class (OM)	0	0.0E+00	blank	blank - blank

Binning Rule Description:

Apply to fibers (F) only:
L ≥ 0.5µm, AR ≥ 3

No restrictions for other structure types.

ChiSq test for even filter loading for Total TE (see Annex F2 in ISO 10312)

Filter loading is OK

Binning Rule Description:

Apply to all structures where Total column > 0

L > 5µm, W ≥ 0.25µm and W ≤ 3µm, AR ≥ 3

ChiSq test for even filter loading for PCMe (see Annex F2 in ISO 10312)

[redacted]

(a) Based on countable structures only.
(b) Loading on Filter (s/mm²) = N structures / (GOs Counted * GO Area). Results for indirect samples are based on the secondary filter.
(c) Air Concentration (s/cc) = (N structures * EFA) / (GOs Counted * GO Area * F-factor * Air Volume * 1000).
Dust Loading (s/cm2) = (N structures * EFA) / (GOs Counted * GO Area * F-factor * Dust Collection Area).

version 13-DRAFT

Sumas Mtn Asbestos Soil [SFP-078A]

National Asbestos Data Entry Spreadsheet (NADES) for Air & Dust Analysis by Superfund TEM
ANALYTICAL REPORT
FILE NAME: SFP-078A_LabCor Inc._14394151_01-15-15_141014_TEM_D.xls

SAMPLE/ANALYSIS INFORMATION			
**Field Sample Number**	14394151	**Lab Sample Number**	141014-S6
**Media**	Air	**Preparation**	Direct
**Sample Type**	Field Blank	**Sample Status**	Analyzed
**Air Volume (L)**		**Analysis Date**	1/15/2015
**QA Sample Type**	Not QC	**Method SOP**	0
**Stopping Rule(s):**	Max Area = n/a, Structures = 100, Sensitivity = 3.00E-03		
**Recording Rule(s):**	Min Aspect Ratio = , Min Length = 0.5µm, Min Width = µm		

* Chrysotile was not counted for all grid openings evaluated; this field is utilized only for the Libby site.

ANALYSIS PARAMETERS	
**Effective filter area (mm2)**	385
**F-factor**	1.00E+00
**Grid opening area (mm2)**	0.0104
**# GOs counted High Magnification**	10
**# GOs counted Low Magnification**	0
**Sensitivity (1/cc)**	
**Total Asbestos**	blank
**PCME**	blank
**Maximum Area Examined**	
**High Magnification**	1.0E-01
**Low Magnification**	0.0E+00

Number of Structures with Fatal Data Entry Errors 0 *(Structures with fatal errors are excluded from calculations below)*

Desired Confidence Interval (%):

Mineral Class	Number of Structures (a)	Loading on Filter (b) (s/mm²)	Air Conc (c) (s/cc)	% Poisson Confidence Interval for this Sample
**Total TEM Structures**				
Total Asbestos	0	0.0E+00	blank	blank - blank
Total Chrysotile (CH)	0	0.0E+00	blank	blank - blank
Total Amphibole	0	0.0E+00	blank	blank - blank
actinolite (AC)	0	0.0E+00	blank	blank - blank
amosite (AM)	0	0.0E+00	blank	blank - blank
anthophyllite (AN)	0	0.0E+00	blank	blank - blank
crocidolite (CR)	0	0.0E+00	blank	blank - blank
tremolite (TR)	0	0.0E+00	blank	blank - blank
winchite/richterite/tremolite /actinolite (WRTA)	0	0.0E+00	blank	blank - blank
other amphibole (OA)	0	0.0E+00	blank	blank - blank
Solid Soln: Amosite	0	0.0E+00	blank	blank - blank
Solid Soln: Trem-Act	0	0.0E+00	blank	blank - blank
other mineral class (OM)	0	0.0E+00	blank	blank - blank
**PCM Equivalent Structures (PCME)**				
Total Asbestos	0	0.0E+00	blank	blank - blank
Total Chrysotile (CH)	0	0.0E+00	blank	blank - blank
Total Amphibole	0	0.0E+00	blank	blank - blank
actinolite (AC)	0	0.0E+00	blank	blank - blank
amosite (AM)	0	0.0E+00	blank	blank - blank
anthophyllite (AN)	0	0.0E+00	blank	blank - blank
crocidolite (CR)	0	0.0E+00	blank	blank - blank
tremolite (TR)	0	0.0E+00	blank	blank - blank
winchite/richterite/tremolite /actinolite (WRTA)	0	0.0E+00	blank	blank - blank
other amphibole (OA)	0	0.0E+00	blank	blank - blank
Solid Soln: Amosite	0	0.0E+00	blank	blank - blank
Solid Soln: Trem-Act	0	0.0E+00	blank	blank - blank
other mineral class (OM)	0	0.0E+00	blank	blank - blank

Binning Rule Description:

Apply to fibers (F) only:
L ≥ 0.5µm, AR ≥ 3

No restrictions for other structure types.

ChiSq test for even filter loading for Total TE (see Annex F2 in ISO 10312)

Filter loading is OK

Binning Rule Description:

Apply to all structures where Total column > 0

L > 5µm, W ≥ 0.25µm and W ≤ 3µm, AR ≥ 3

ChiSq test for even filter loading for PCMe (see Annex F2 in ISO 10312)

[redacted]

(a) Based on countable structures only.
(b) Loading on Filter (s/mm²) = N structures / (GOs Counted * GO Area). Results for indirect samples are based on the secondary filter.
(c) Air Concentration (s/cc) = (N structures * EFA) / (GOs Counted * GO Area * F-factor * Air Volume * 1000).
Dust Loading (s/cm2) = (N structures * EFA) / (GOs Counted * GO Area * F-factor * Dust Collection Area)

version 13-DRAFT

Sumas Mtn Asbestos Soil [SFP-078A]

National Asbestos Data Entry Spreadsheet (NADES) for Air & Dust Analysis by Superfund TEM

ANALYTICAL REPORT

FILE NAME: SFP-078A_LabCor Inc._14394153_01-15-15_141014_TEM_D.xls

SAMPLE/ANALYSIS INFORMATION			
**Field Sample Number**	14394153	**Lab Sample Number**	141014-S8
**Media**	Air	**Preparation**	Direct
**Sample Type**	Field Sample	**Sample Status**	Analyzed
**Air Volume (L)**	2277.6	**Analysis Date**	1/15/2015 - 2/4/2015
**QA Sample Type**	Not QC	**Method SOP**	0
**Stopping Rule(s):**	Max Area = n/a, Structures = 100, Sensitivity = 3.00E-04		
**Recording Rule(s):**	Min Aspect Ratio = , Min Length = 0.5µm, Min Width = µm		

* Chrysotile was not counted for all grid openings evaluated; this field is utilized only for the Libby site.

ANALYSIS PARAMETERS	
**Effective filter area (mm2)**	385
**F-factor**	1.00E+00
**Grid opening area (mm2)**	0.0104
**# GOs counted High Magnification**	55
**# GOs counted Low Magnification**	0
**Sensitivity (1/cc)**	
**Total Asbestos**	0.000295
**PCME**	0.000295
**Maximum Area Examined**	
**High Magnification**	5.7E-01
**Low Magnification**	0.0E+00

Number of Structures with Fatal Data Entry Errors 0 *(Structures with fatal errors are excluded from calculations below)*

Desired Confidence Interval (%):

Mineral Class	Number of Structures (a)	Loading on Filter (b) (s/mm²)	Air Conc (c) (s/cc)	% Poisson Confidence Interval for this Sample
**Total TEM Structures**				
Total Asbestos	33	5.8E+01	9.7E-03	9.6E-03 - 9.9E-03
Total Chrysotile (CH)	33	5.8E+01	9.7E-03	9.6E-03 - 9.9E-03
Total Amphibole	0	0.0E+00	0.0E+00	0.0E+00 - 2.0E-04
actinolite (AC)	0	0.0E+00	0.0E+00	0.0E+00 - 2.0E-04
amosite (AM)	0	0.0E+00	0.0E+00	0.0E+00 - 2.0E-04
anthophyllite (AN)	0	0.0E+00	0.0E+00	0.0E+00 - 2.0E-04
crocidolite (CR)	0	0.0E+00	0.0E+00	0.0E+00 - 2.0E-04
tremolite (TR)	0	0.0E+00	0.0E+00	0.0E+00 - 2.0E-04
winchite/richterite/tremolite /actinolite (WRTA)	0	0.0E+00	0.0E+00	0.0E+00 - 2.0E-04
other amphibole (OA)	0	0.0E+00	0.0E+00	0.0E+00 - 2.0E-04
Solid Soln: Amosite	0	0.0E+00	0.0E+00	0.0E+00 - 2.0E-04
Solid Soln: Trem-Act	0	0.0E+00	0.0E+00	0.0E+00 - 2.0E-04
other mineral class (OM)	0	0.0E+00	0.0E+00	0.0E+00 - 2.0E-04
**PCM Equivalent Structures (PCME)**				
Total Asbestos	3	5.2E+00	8.8E-04	7.9E-04 - 1.1E-03
Total Chrysotile (CH)	3	5.2E+00	8.8E-04	7.9E-04 - 1.1E-03
Total Amphibole	0	0.0E+00	0.0E+00	0.0E+00 - 2.0E-04
actinolite (AC)	0	0.0E+00	0.0E+00	0.0E+00 - 2.0E-04
amosite (AM)	0	0.0E+00	0.0E+00	0.0E+00 - 2.0E-04
anthophyllite (AN)	0	0.0E+00	0.0E+00	0.0E+00 - 2.0E-04
crocidolite (CR)	0	0.0E+00	0.0E+00	0.0E+00 - 2.0E-04
tremolite (TR)	0	0.0E+00	0.0E+00	0.0E+00 - 2.0E-04
winchite/richterite/tremolite /actinolite (WRTA)	0	0.0E+00	0.0E+00	0.0E+00 - 2.0E-04
other amphibole (OA)	0	0.0E+00	0.0E+00	0.0E+00 - 2.0E-04
Solid Soln: Amosite	0	0.0E+00	0.0E+00	0.0E+00 - 2.0E-04
Solid Soln: Trem-Act	0	0.0E+00	0.0E+00	0.0E+00 - 2.0E-04
other mineral class (OM)	0	0.0E+00	0.0E+00	0.0E+00 - 2.0E-04

Binning Rule Description:

Apply to fibers (F) only:
L ≥ 0.5µm, AR ≥ 3

No restrictions for other structure types.

ChiSq test for even filter loading for Total TE
(see Annex F2 in ISO 10312)

Filter loading appears uneven. Results may be uncertain.

Binning Rule Description:

Apply to all structures where Total column > 0

L > 5µm, W ≥ 0.25µm and W ≤ 3µm, AR ≥ 3

ChiSq test for even filter loading for PCMe
(see Annex F2 in ISO 10312)

Filter loading is OK

[redacted]

(a) Based on countable structures only.
(b) Loading on Filter (s/mm²) = N structures / (GOs Counted * GO Area). Results for indirect samples are based on the secondary filter.
(c) Air Concentration (s/cc) = (N structures * EFA) / (GOs Counted * GO Area * F-factor * Air Volume * 1000)
Dust Loading (s/cm2) = (N structures * EFA) / (GOs Counted * GO Area * F-factor * Dust Collection Area)

version 13-DRAFT

Sumas Mtn Asbestos Soil [SFP-078A]

National Asbestos Data Entry Spreadsheet (NADES) for Air & Dust Analysis by Superfund TEM

ANALYTICAL REPORT

FILE NAME: SFP-078A_LabCor Inc._14394154_01-16-15_141014_TEM_D.xls

SAMPLE/ANALYSIS INFORMATION			
**Field Sample Number**	14394154	**Lab Sample Number**	141014-S9
**Media**	Air	**Preparation**	Direct
**Sample Type**	Field Sample	**Sample Status**	Analyzed
**Air Volume (L)**	2172	**Analysis Date**	1/16/2015
**QA Sample Type**	Not QC	**Method SOP**	0
**Stopping Rule(s):**	Max Area = n/a, Structures = 100, Sensitivity = 3.00E-04		
**Recording Rule(s):**	Min Aspect Ratio = , Min Length = 0.5µm, Min Width = µm		

* Chrysotile was not counted for all grid openings evaluated; this field is utilized only for the Libby site.

ANALYSIS PARAMETERS	
**Effective filter area (mm2)**	385
**F-factor**	1.00E+00
**Grid opening area (mm2)**	0.0104
**# GOs counted High Magnification**	57
**# GOs counted Low Magnification**	0
**Sensitivity (1/cc)**	
**Total Asbestos**	0.0002984
**PCME**	0.0002984
**Maximum Area Examined**	
**High Magnification**	5.9E-01
**Low Magnification**	0.0E+00

Number of Structures with Fatal Data Entry Errors 0 *(Structures with fatal errors are excluded from calculations below)*

Desired Confidence Interval (%):

Mineral Class	Number of Structures (a)	Loading on Filter (b) (s/mm^2)	Air Conc (c) (s/cc)	% Poisson Confidence Interval for this Sample
**Total TEM Structures**				
Total Asbestos	75	1.3E+02	2.2E-02	2.2E-02 - 2.3E-02
Total Chrysotile (CH)	74	1.2E+02	2.2E-02	2.2E-02 - 2.2E-02
Total Amphibole	1	1.7E+00	3.0E-04	2.1E-04 - 5.0E-04
actinolite (AC)	0	0.0E+00	0.0E+00	0.0E+00 - 2.1E-04
amosite (AM)	0	0.0E+00	0.0E+00	0.0E+00 - 2.1E-04
anthophyllite (AN)	0	0.0E+00	0.0E+00	0.0E+00 - 2.1E-04
crocidolite (CR)	0	0.0E+00	0.0E+00	0.0E+00 - 2.1E-04
tremolite (TR)	0	0.0E+00	0.0E+00	0.0E+00 - 2.1E-04
winchite/richterite/tremolite /actinolite (WRTA)	0	0.0E+00	0.0E+00	0.0E+00 - 2.1E-04
other amphibole (OA)	1	1.7E+00	3.0E-04	2.1E-04 - 5.0E-04
Solid Soln: Amosite	0	0.0E+00	0.0E+00	0.0E+00 - 2.1E-04
Solid Soln: Trem-Act	0	0.0E+00	0.0E+00	0.0E+00 - 2.1E-04
other mineral class (OM)	0	0.0E+00	0.0E+00	0.0E+00 - 2.1E-04
**PCM Equivalent Structures (PCME)**				
Total Asbestos	5	8.4E+00	1.5E-03	1.4E-03 - 1.7E-03
Total Chrysotile (CH)	5	8.4E+00	1.5E-03	1.4E-03 - 1.7E-03
Total Amphibole	0	0.0E+00	0.0E+00	0.0E+00 - 2.1E-04
actinolite (AC)	0	0.0E+00	0.0E+00	0.0E+00 - 2.1E-04
amosite (AM)	0	0.0E+00	0.0E+00	0.0E+00 - 2.1E-04
anthophyllite (AN)	0	0.0E+00	0.0E+00	0.0E+00 - 2.1E-04
crocidolite (CR)	0	0.0E+00	0.0E+00	0.0E+00 - 2.1E-04
tremolite (TR)	0	0.0E+00	0.0E+00	0.0E+00 - 2.1E-04
winchite/richterite/tremolite /actinolite (WRTA)	0	0.0E+00	0.0E+00	0.0E+00 - 2.1E-04
other amphibole (OA)	0	0.0E+00	0.0E+00	0.0E+00 - 2.1E-04
Solid Soln: Amosite	0	0.0E+00	0.0E+00	0.0E+00 - 2.1E-04
Solid Soln: Trem-Act	0	0.0E+00	0.0E+00	0.0E+00 - 2.1E-04
other mineral class (OM)	0	0.0E+00	0.0E+00	0.0E+00 - 2.1E-04

Binning Rule Description:

Apply to fibers (F) only:
L ≥ 0.5µm, AR ≥ 3

No restrictions for other structure types.

ChiSq test for even filter loading for Total TE (see Annex F2 in ISO 10312)

Filter loading appears uneven. Results may be uncertain.

Binning Rule Description:

Apply to all structures where Total column > 0

L > 5µm, W ≥ 0.25µm and W ≤ 3µm, AR ≥ 3

ChiSq test for even filter loading for PCMe (see Annex F2 in ISO 10312)

Filter loading is OK

[redacted]

(a) Based on countable structures only.
(b) Loading on Filter (s/mm^2) = N structures / (GOs Counted * GO Area). Results for indirect samples are based on the secondary filter.
(c) Air Concentration (s/cc) = (N structures * EFA) / (GOs Counted * GO Area * F-factor * Air Volume * 1000).
Dust Loading (s/cm2) = (N structures * EFA) / (GOs Counted * GO Area * F-factor * Dust Collection Area).

version 13-DRAFT

Sumas Mtn Asbestos Soil [SFP-078A]

National Asbestos Data Entry Spreadsheet (NADES) for Air & Dust Analysis by Superfund TEM

ANALYTICAL REPORT

FILE NAME: SFP-078A_LabCor Inc._14394156_01-16-15_141014_TEM_D.xls

SAMPLE/ANALYSIS INFORMATION			
**Field Sample Number**	14394156	**Lab Sample Number**	141014-S10
**Media**	Air	**Preparation**	Direct
**Sample Type**	Field Sample	**Sample Status**	Analyzed
**Air Volume (L)**	244.335	**Analysis Date**	1/16/2015
**QA Sample Type**	Not QC	**Method SOP**	0
**Stopping Rule(s):**	Max Area = n/a, Structures = 100, Sensitivity = 3.00E-03		
**Recording Rule(s):**	Min Aspect Ratio = , Min Length = 0.5µm, Min Width = µm		

* Chrysotile was not counted for all grid openings evaluated; this field is utilized only for the Libby site.

ANALYSIS PARAMETERS	
**Effective filter area (mm2)**	385
**F-factor**	1.00E+00
**Grid opening area (mm2)**	0.0104
**# GOs counted High Magnification**	51
**# GOs counted Low Magnification**	0
**Sensitivity (1/cc)**	
**Total Asbestos**	0.0029651
**PCME**	0.0029651
**Maximum Area Examined**	
**High Magnification**	5.3E-01
**Low Magnification**	0.0E+00

Number of Structures with Fatal Data Entry Errors 0 *(Structures with fatal errors are excluded from calculations below)*

Desired Confidence Interval (%):

Mineral Class	Number of Structures (a)	Loading on Filter (b) (s/mm^2)	Air Conc (c) (s/cc)	% Poisson Confidence Interval for this Sample
**Total TEM Structures**				
Total Asbestos	0	0.0E+00	0.0E+00	0.0E+00 - 2.1E-03
Total Chrysotile (CH)	0	0.0E+00	0.0E+00	0.0E+00 - 2.1E-03
Total Amphibole	0	0.0E+00	0.0E+00	0.0E+00 - 2.1E-03
actinolite (AC)	0	0.0E+00	0.0E+00	0.0E+00 - 2.1E-03
amosite (AM)	0	0.0E+00	0.0E+00	0.0E+00 - 2.1E-03
anthophyllite (AN)	0	0.0E+00	0.0E+00	0.0E+00 - 2.1E-03
crocidolite (CR)	0	0.0E+00	0.0E+00	0.0E+00 - 2.1E-03
tremolite (TR)	0	0.0E+00	0.0E+00	0.0E+00 - 2.1E-03
winchite/richterite/tremolite /actinolite (WRTA)	0	0.0E+00	0.0E+00	0.0E+00 - 2.1E-03
other amphibole (OA)	0	0.0E+00	0.0E+00	0.0E+00 - 2.1E-03
Solid Soln: Amosite	0	0.0E+00	0.0E+00	0.0E+00 - 2.1E-03
Solid Soln: Trem-Act	0	0.0E+00	0.0E+00	0.0E+00 - 2.1E-03
other mineral class (OM)	0	0.0E+00	0.0E+00	0.0E+00 - 2.1E-03
**PCM Equivalent Structures (PCME)**				
Total Asbestos	0	0.0E+00	0.0E+00	0.0E+00 - 2.1E-03
Total Chrysotile (CH)	0	0.0E+00	0.0E+00	0.0E+00 - 2.1E-03
Total Amphibole	0	0.0E+00	0.0E+00	0.0E+00 - 2.1E-03
actinolite (AC)	0	0.0E+00	0.0E+00	0.0E+00 - 2.1E-03
amosite (AM)	0	0.0E+00	0.0E+00	0.0E+00 - 2.1E-03
anthophyllite (AN)	0	0.0E+00	0.0E+00	0.0E+00 - 2.1E-03
crocidolite (CR)	0	0.0E+00	0.0E+00	0.0E+00 - 2.1E-03
tremolite (TR)	0	0.0E+00	0.0E+00	0.0E+00 - 2.1E-03
winchite/richterite/tremolite /actinolite (WRTA)	0	0.0E+00	0.0E+00	0.0E+00 - 2.1E-03
other amphibole (OA)	0	0.0E+00	0.0E+00	0.0E+00 - 2.1E-03
Solid Soln: Amosite	0	0.0E+00	0.0E+00	0.0E+00 - 2.1E-03
Solid Soln: Trem-Act	0	0.0E+00	0.0E+00	0.0E+00 - 2.1E-03
other mineral class (OM)	0	0.0E+00	0.0E+00	0.0E+00 - 2.1E-03

Binning Rule Description:

Apply to fibers (F) only:
L ≥ 0.5µm, AR ≥ 3

No restrictions for other structure types.

ChiSq test for even filter loading for Total TE
(see Annex F2 in ISO 10312)

Filter loading is OK

Binning Rule Description:

Apply to all structures where Total column > 0

L > 5µm, W ≥ 0.25µm and W ≤ 3µm, AR ≥ 3

ChiSq test for even filter loading for PCMe
(see Annex F2 in ISO 10312)

[redacted]

(a) Based on countable structures only.
(b) Loading on Filter (s/mm^2) = N structures / (GOs Counted * GO Area). Results for indirect samples are based on the secondary filter.
(c) Air Concentration (s/cc) = (N structures * EFA) / (GOs Counted * GO Area * F-factor * Air Volume * 1000).
Dust Loading (s/cm2) = (N structures * EFA) / (GOs Counted * GO Area * F-factor * Dust Collection Area).

version 13-DRAFT

Sumas Mtn Asbestos Soil [SFP-078A]

National Asbestos Data Entry Spreadsheet (NADES) for Air & Dust Analysis by Superfund TEM

ANALYTICAL REPORT

FILE NAME: SFP-078A_LabCor Inc._14394157_01-29-15_141014_TEM_D.xls

SAMPLE/ANALYSIS INFORMATION			
**Field Sample Number**	14394157	**Lab Sample Number**	141014-S11
**Media**	Air	**Preparation**	Direct
**Sample Type**	Field Sample	**Sample Status**	Analyzed
**Air Volume (L)**	97.47	**Analysis Date**	1/29/2015
**QA Sample Type**	Not QC	**Method SOP**	0
**Stopping Rule(s):**	Max Area = n/a, Structures = 100, Sensitivity = 3.00E-03		
**Recording Rule(s):**	Min Aspect Ratio = , Min Length = 0.5µm, Min Width = µm		

* Chrysotile was not counted for all grid openings evaluated; this field is utilized only for the Libby site.

ANALYSIS PARAMETERS	
**Effective filter area (mm2)**	385
**F-factor**	1.00E+00
**Grid opening area (mm2)**	0.0104
**# GOs counted High Magnification**	127
**# GOs counted Low Magnification**	0
**Sensitivity (1/cc)**	
**Total Asbestos**	0.0029848
**PCME**	0.0029848
**Maximum Area Examined**	
**High Magnification**	1.3E+00
**Low Magnification**	0.0E+00

Number of Structures with Fatal Data Entry Errors 0 *(Structures with fatal errors are excluded from calculations below)*

Desired Confidence Interval (%):

Mineral Class	Number of Structures (a)	Loading on Filter (b) (s/mm^2)	Air Conc (c) (s/cc)	% Poisson Confidence Interval for this Sample
**Total TEM Structures**				
Total Asbestos	1	7.6E-01	3.0E-03	2.1E-03 - 5.0E-03
Total Chrysotile (CH)	1	7.6E-01	3.0E-03	2.1E-03 - 5.0E-03
Total Amphibole	0	0.0E+00	0.0E+00	0.0E+00 - 2.1E-03
actinolite (AC)	0	0.0E+00	0.0E+00	0.0E+00 - 2.1E-03
amosite (AM)	0	0.0E+00	0.0E+00	0.0E+00 - 2.1E-03
anthophyllite (AN)	0	0.0E+00	0.0E+00	0.0E+00 - 2.1E-03
crocidolite (CR)	0	0.0E+00	0.0E+00	0.0E+00 - 2.1E-03
tremolite (TR)	0	0.0E+00	0.0E+00	0.0E+00 - 2.1E-03
winchite/richterite/tremolite /actinolite (WRTA)	0	0.0E+00	0.0E+00	0.0E+00 - 2.1E-03
other amphibole (OA)	0	0.0E+00	0.0E+00	0.0E+00 - 2.1E-03
Solid Soln: Amosite	0	0.0E+00	0.0E+00	0.0E+00 - 2.1E-03
Solid Soln: Trem-Act	0	0.0E+00	0.0E+00	0.0E+00 - 2.1E-03
other mineral class (OM)	0	0.0E+00	0.0E+00	0.0E+00 - 2.1E-03
**PCM Equivalent Structures (PCME)**				
Total Asbestos	0	0.0E+00	0.0E+00	0.0E+00 - 2.1E-03
Total Chrysotile (CH)	0	0.0E+00	0.0E+00	0.0E+00 - 2.1E-03
Total Amphibole	0	0.0E+00	0.0E+00	0.0E+00 - 2.1E-03
actinolite (AC)	0	0.0E+00	0.0E+00	0.0E+00 - 2.1E-03
amosite (AM)	0	0.0E+00	0.0E+00	0.0E+00 - 2.1E-03
anthophyllite (AN)	0	0.0E+00	0.0E+00	0.0E+00 - 2.1E-03
crocidolite (CR)	0	0.0E+00	0.0E+00	0.0E+00 - 2.1E-03
tremolite (TR)	0	0.0E+00	0.0E+00	0.0E+00 - 2.1E-03
winchite/richterite/tremolite /actinolite (WRTA)	0	0.0E+00	0.0E+00	0.0E+00 - 2.1E-03
other amphibole (OA)	0	0.0E+00	0.0E+00	0.0E+00 - 2.1E-03
Solid Soln: Amosite	0	0.0E+00	0.0E+00	0.0E+00 - 2.1E-03
Solid Soln: Trem-Act	0	0.0E+00	0.0E+00	0.0E+00 - 2.1E-03
other mineral class (OM)	0	0.0E+00	0.0E+00	0.0E+00 - 2.1E-03

Binning Rule Description:

Apply to fibers (F) only:
L ≥ 0.5µm, AR ≥ 3

No restrictions for other structure types.

ChiSq test for even filter loading for Total TE
(see Annex F2 in ISO 10312)

Filter loading appears uneven. Results may be uncertain.

Binning Rule Description:

Apply to all structures where Total column > 0

L > 5µm, W ≥ 0.25µm and W ≤ 3µm, AR ≥ 3

ChiSq test for even filter loading for PCMe
(see Annex F2 in ISO 10312)

[redacted]

(a) Based on countable structures only.
(b) Loading on Filter (s/mm^2) = N structures / (GOs Counted * GO Area). Results for indirect samples are based on the secondary filter.
(c) Air Concentration (s/cc) = (N structures * EFA) / (GOs Counted * GO Area * F-factor * Air Volume * 1000).
Dust Loading (s/cm2) = (N structures * EFA) / (GOs Counted * GO Area * F-factor * Dust Collection Area).

version 13-DRAFT

Sumas Mtn Asbestos Soil [SFP-078A]

National Asbestos Data Entry Spreadsheet (NADES) for Air & Dust Analysis by Superfund TEM

ANALYTICAL REPORT

FILE NAME: SFP-078A_LabCor Inc._14394158_01-30-15_141014_TEM_D.xls

SAMPLE/ANALYSIS INFORMATION			
**Field Sample Number**	14394158	**Lab Sample Number**	141014-S12
**Media**	Air	**Preparation**	Direct
**Sample Type**	Field Sample	**Sample Status**	Analyzed
**Air Volume (L)**	678.6	**Analysis Date**	1/30/2015
**QA Sample Type**	Not QC	**Method SOP**	0
**Stopping Rule(s):**	Max Area = n/a, Structures = 100, Sensitivity = 3.00E-03		
**Recording Rule(s):**	Min Aspect Ratio = , Min Length = 0.5µm, Min Width = µm		

* Chrysotile was not counted for all grid openings evaluated; this field is utilized only for the Libby site.

ANALYSIS PARAMETERS	
**Effective filter area (mm2)**	385
**F-factor**	1.00E+00
**Grid opening area (mm2)**	0.0104
**# GOs counted High Magnification**	19
**# GOs counted Low Magnification**	0
**Sensitivity (1/cc)**	
**Total Asbestos**	0.0028657
**PCME**	0.0028657
**Maximum Area Examined**	
**High Magnification**	2.0E-01
**Low Magnification**	0.0E+00

Number of Structures with Fatal Data Entry Errors 0 *(Structures with fatal errors are excluded from calculations below)*

Desired Confidence Interval (%):

Mineral Class	Number of Structures (a)	Loading on Filter (b) (s/mm^2)	Air Conc (c) (s/cc)	% Poisson Confidence Interval for this Sample
**Total TEM Structures**				
Total Asbestos	3	1.5E+01	8.6E-03	7.7E-03 - 1.1E-02
Total Chrysotile (CH)	3	1.5E+01	8.6E-03	7.7E-03 - 1.1E-02
Total Amphibole	0	0.0E+00	0.0E+00	0.0E+00 - 2.0E-03
actinolite (AC)	0	0.0E+00	0.0E+00	0.0E+00 - 2.0E-03
amosite (AM)	0	0.0E+00	0.0E+00	0.0E+00 - 2.0E-03
anthophyllite (AN)	0	0.0E+00	0.0E+00	0.0E+00 - 2.0E-03
crocidolite (CR)	0	0.0E+00	0.0E+00	0.0E+00 - 2.0E-03
tremolite (TR)	0	0.0E+00	0.0E+00	0.0E+00 - 2.0E-03
winchite/richterite/tremolite /actinolite (WRTA)	0	0.0E+00	0.0E+00	0.0E+00 - 2.0E-03
other amphibole (OA)	0	0.0E+00	0.0E+00	0.0E+00 - 2.0E-03
Solid Soln: Amosite	0	0.0E+00	0.0E+00	0.0E+00 - 2.0E-03
Solid Soln: Trem-Act	0	0.0E+00	0.0E+00	0.0E+00 - 2.0E-03
other mineral class (OM)	0	0.0E+00	0.0E+00	0.0E+00 - 2.0E-03
**PCM Equivalent Structures (PCME)**				
Total Asbestos	1	5.1E+00	2.9E-03	2.0E-03 - 4.8E-03
Total Chrysotile (CH)	1	5.1E+00	2.9E-03	2.0E-03 - 4.8E-03
Total Amphibole	0	0.0E+00	0.0E+00	0.0E+00 - 2.0E-03
actinolite (AC)	0	0.0E+00	0.0E+00	0.0E+00 - 2.0E-03
amosite (AM)	0	0.0E+00	0.0E+00	0.0E+00 - 2.0E-03
anthophyllite (AN)	0	0.0E+00	0.0E+00	0.0E+00 - 2.0E-03
crocidolite (CR)	0	0.0E+00	0.0E+00	0.0E+00 - 2.0E-03
tremolite (TR)	0	0.0E+00	0.0E+00	0.0E+00 - 2.0E-03
winchite/richterite/tremolite /actinolite (WRTA)	0	0.0E+00	0.0E+00	0.0E+00 - 2.0E-03
other amphibole (OA)	0	0.0E+00	0.0E+00	0.0E+00 - 2.0E-03
Solid Soln: Amosite	0	0.0E+00	0.0E+00	0.0E+00 - 2.0E-03
Solid Soln: Trem-Act	0	0.0E+00	0.0E+00	0.0E+00 - 2.0E-03
other mineral class (OM)	0	0.0E+00	0.0E+00	0.0E+00 - 2.0E-03

Binning Rule Description:

Apply to fibers (F) only:
L ≥ 0.5µm, AR ≥ 3

No restrictions for other structure types.

ChiSq test for even filter loading for Total TE (see Annex F2 in ISO 10312)

Filter loading appears uneven. Results may be uncertain.

Binning Rule Description:

Apply to all structures where Total column > 0

L > 5µm, W ≥ 0.25µm and W ≤ 3µm, AR ≥ 3

ChiSq test for even filter loading for PCMe (see Annex F2 in ISO 10312)

Filter loading is OK

[redacted]

(a) Based on countable structures only.

(b) Loading on Filter (s/mm^2) = N structures / (GOs Counted * GO Area). Results for indirect samples are based on the secondary filter

(c) Air Concentration (s/cc) = (N structures * EFA) / (GOs Counted * GO Area * F-factor * Air Volume * 1000)
Dust Loading (s/cm2) = (N structures * EFA) / (GOs Counted * GO Area * F-factor * Dust Collection Area).

version 13-DRAFT

Sumas Mtn Asbestos Soil [SFP-078A]

National Asbestos Data Entry Spreadsheet (NADES) for Air & Dust Analysis by Superfund TEM

ANALYTICAL REPORT

FILE NAME: SFP-078A_LabCor Inc._14394161_02-05-15_141014_TEM_D.xls

SAMPLE/ANALYSIS INFORMATION			
**Field Sample Number**	14394161	**Lab Sample Number**	141014-S13
**Media**	Air	**Preparation**	Direct
**Sample Type**	Field Sample	**Sample Status**	Analyzed
**Air Volume (L)**	2212.8	**Analysis Date**	2/5/2015
**QA Sample Type**	Not QC	**Method SOP**	0
**Stopping Rule(s):**	Max Area = n/a, Structures = 100, Sensitivity = 3.00E-04		
**Recording Rule(s):**	Min Aspect Ratio = , Min Length = 0.5µm, Min Width = µm		

* Chrysotile was not counted for all grid openings evaluated; this field is utilized only for the Libby site.

ANALYSIS PARAMETERS	
**Effective filter area (mm2)**	385
**F-factor**	1.00E+00
**Grid opening area (mm2)**	0.0104
**# GOs counted High Magnification**	57
**# GOs counted Low Magnification**	0
**Sensitivity (1/cc)**	
**Total Asbestos**	0.0002929
**PCME**	0.0002929
**Maximum Area Examined**	
**High Magnification**	5.9E-01
**Low Magnification**	0.0E+00

Number of Structures with Fatal Data Entry Errors 0 *(Structures with fatal errors are excluded from calculations below)*

Desired Confidence Interval (%):

Mineral Class	Number of Structures (a)	Loading on Filter (b) (s/mm^2)	Air Conc (c) (s/cc)	% Poisson Confidence Interval for this Sample
**Total TEM Structures**				
Total Asbestos	0	0.0E+00	0.0E+00	0.0E+00 - 2.0E-04
Total Chrysotile (CH)	0	0.0E+00	0.0E+00	0.0E+00 - 2.0E-04
Total Amphibole	0	0.0E+00	0.0E+00	0.0E+00 - 2.0E-04
actinolite (AC)	0	0.0E+00	0.0E+00	0.0E+00 - 2.0E-04
amosite (AM)	0	0.0E+00	0.0E+00	0.0E+00 - 2.0E-04
anthophyllite (AN)	0	0.0E+00	0.0E+00	0.0E+00 - 2.0E-04
crocidolite (CR)	0	0.0E+00	0.0E+00	0.0E+00 - 2.0E-04
tremolite (TR)	0	0.0E+00	0.0E+00	0.0E+00 - 2.0E-04
winchite/richterite/tremolite /actinolite (WRTA)	0	0.0E+00	0.0E+00	0.0E+00 - 2.0E-04
other amphibole (OA)	0	0.0E+00	0.0E+00	0.0E+00 - 2.0E-04
Solid Soln: Amosite	0	0.0E+00	0.0E+00	0.0E+00 - 2.0E-04
Solid Soln: Trem-Act	0	0.0E+00	0.0E+00	0.0E+00 - 2.0E-04
other mineral class (OM)	0	0.0E+00	0.0E+00	0.0E+00 - 2.0E-04
**PCM Equivalent Structures (PCME)**				
Total Asbestos	0	0.0E+00	0.0E+00	0.0E+00 - 2.0E-04
Total Chrysotile (CH)	0	0.0E+00	0.0E+00	0.0E+00 - 2.0E-04
Total Amphibole	0	0.0E+00	0.0E+00	0.0E+00 - 2.0E-04
actinolite (AC)	0	0.0E+00	0.0E+00	0.0E+00 - 2.0E-04
amosite (AM)	0	0.0E+00	0.0E+00	0.0E+00 - 2.0E-04
anthophyllite (AN)	0	0.0E+00	0.0E+00	0.0E+00 - 2.0E-04
crocidolite (CR)	0	0.0E+00	0.0E+00	0.0E+00 - 2.0E-04
tremolite (TR)	0	0.0E+00	0.0E+00	0.0E+00 - 2.0E-04
winchite/richterite/tremolite /actinolite (WRTA)	0	0.0E+00	0.0E+00	0.0E+00 - 2.0E-04
other amphibole (OA)	0	0.0E+00	0.0E+00	0.0E+00 - 2.0E-04
Solid Soln: Amosite	0	0.0E+00	0.0E+00	0.0E+00 - 2.0E-04
Solid Soln: Trem-Act	0	0.0E+00	0.0E+00	0.0E+00 - 2.0E-04
other mineral class (OM)	0	0.0E+00	0.0E+00	0.0E+00 - 2.0E-04

Binning Rule Description:

Apply to fibers (F) only:
L ≥ 0.5µm, AR ≥ 3

No restrictions for other structure types.

ChiSq test for even filter loading for Total TE
(see Annex F2 in ISO 10312)

Filter loading is OK

Binning Rule Description:

Apply to all structures where Total column > 0

L > 5µm, W ≥ 0.25µm and W ≤ 3µm, AR ≥ 3

ChiSq test for even filter loading for PCMe
(see Annex F2 in ISO 10312)

[redacted]

(a) Based on countable structures only.

(b) Loading on Filter (s/mm^2) = N structures / (GOs Counted * GO Area). Results for indirect samples are based on the secondary filter.

(c) Air Concentration (s/cc) = (N structures * EFA) / (GOs Counted * GO Area * F-factor * Air Volume * 1000).
Dust Loading (s/cm2) = (N structures * EFA) / (GOs Counted * GO Area * F-factor * Dust Collection Area).

version 13-DRAFT

Sumas Mtn Asbestos Soil [SFP-078A]

National Asbestos Data Entry Spreadsheet (NADES) for Air & Dust Analysis by Superfund TEM

ANALYTICAL REPORT

FILE NAME: SFP-078A_LabCor Inc._14394162_02-05-15_141014_TEM_D.xls

SAMPLE/ANALYSIS INFORMATION			
**Field Sample Number**	14394162	**Lab Sample Number**	141014-S14
**Media**	Air	**Preparation**	Direct
**Sample Type**	Field Sample	**Sample Status**	Analyzed
**Air Volume (L)**	2186.4	**Analysis Date**	2/5/2015
**QA Sample Type**	Not QC	**Method SOP**	0
**Stopping Rule(s):**	Max Area = n/a, Structures = 100, Sensitivity = 3.00E-04		
**Recording Rule(s):**	Min Aspect Ratio = , Min Length = 0.5µm, Min Width = µm		

* Chrysotile was not counted for all grid openings evaluated; this field is utilized only for the Libby site.

ANALYSIS PARAMETERS	
**Effective filter area (mm2)**	385
**F-factor**	1.00E+00
**Grid opening area (mm2)**	0.0104
**# GOs counted High Magnification**	57
**# GOs counted Low Magnification**	0
**Sensitivity (1/cc)**	
**Total Asbestos**	0.0002965
**PCME**	0.0002965
**Maximum Area Examined**	
**High Magnification**	5.9E-01
**Low Magnification**	0.0E+00

Number of Structures with Fatal Data Entry Errors 0 *(Structures with fatal errors are excluded from calculations below)*

Desired Confidence Interval (%):

Mineral Class	Number of Structures (a)	Loading on Filter (b) (s/mm^2)	Air Conc (c) (s/cc)	% Poisson Confidence Interval for this Sample
**Total TEM Structures**				
Total Asbestos	1	1.7E+00	3.0E-04	2.1E-04 - 5.0E-04
Total Chrysotile (CH)	1	1.7E+00	3.0E-04	2.1E-04 - 5.0E-04
Total Amphibole	0	0.0E+00	0.0E+00	0.0E+00 - 2.1E-04
actinolite (AC)	0	0.0E+00	0.0E+00	0.0E+00 - 2.1E-04
amosite (AM)	0	0.0E+00	0.0E+00	0.0E+00 - 2.1E-04
anthophyllite (AN)	0	0.0E+00	0.0E+00	0.0E+00 - 2.1E-04
crocidolite (CR)	0	0.0E+00	0.0E+00	0.0E+00 - 2.1E-04
tremolite (TR)	0	0.0E+00	0.0E+00	0.0E+00 - 2.1E-04
winchite/richterite/tremolite /actinolite (WRTA)	0	0.0E+00	0.0E+00	0.0E+00 - 2.1E-04
other amphibole (OA)	0	0.0E+00	0.0E+00	0.0E+00 - 2.1E-04
Solid Soln: Amosite	0	0.0E+00	0.0E+00	0.0E+00 - 2.1E-04
Solid Soln: Trem-Act	0	0.0E+00	0.0E+00	0.0E+00 - 2.1E-04
other mineral class (OM)	0	0.0E+00	0.0E+00	0.0E+00 - 2.1E-04
**PCM Equivalent Structures (PCME)**				
Total Asbestos	1	1.7E+00	3.0E-04	2.1E-04 - 5.0E-04
Total Chrysotile (CH)	1	1.7E+00	3.0E-04	2.1E-04 - 5.0E-04
Total Amphibole	0	0.0E+00	0.0E+00	0.0E+00 - 2.1E-04
actinolite (AC)	0	0.0E+00	0.0E+00	0.0E+00 - 2.1E-04
amosite (AM)	0	0.0E+00	0.0E+00	0.0E+00 - 2.1E-04
anthophyllite (AN)	0	0.0E+00	0.0E+00	0.0E+00 - 2.1E-04
crocidolite (CR)	0	0.0E+00	0.0E+00	0.0E+00 - 2.1E-04
tremolite (TR)	0	0.0E+00	0.0E+00	0.0E+00 - 2.1E-04
winchite/richterite/tremolite /actinolite (WRTA)	0	0.0E+00	0.0E+00	0.0E+00 - 2.1E-04
other amphibole (OA)	0	0.0E+00	0.0E+00	0.0E+00 - 2.1E-04
Solid Soln: Amosite	0	0.0E+00	0.0E+00	0.0E+00 - 2.1E-04
Solid Soln: Trem-Act	0	0.0E+00	0.0E+00	0.0E+00 - 2.1E-04
other mineral class (OM)	0	0.0E+00	0.0E+00	0.0E+00 - 2.1E-04

Binning Rule Description: (Total TEM Structures)

Apply to fibers (F) only:
L ≥ 0.5µm, AR ≥ 3

No restrictions for other structure types.

ChiSq test for even filter loading for Total TE
(see Annex F2 in ISO 10312)

Filter loading appears uneven. Results may be uncertain.

Binning Rule Description: (PCME)

Apply to all structures where Total column > 0

L > 5µm, W ≥ 0.25µm and W ≤ 3µm, AR ≥ 3

ChiSq test for even filter loading for PCMe
(see Annex F2 in ISO 10312)

Filter loading is OK

[redacted]

(a) Based on countable structures only.
(b) Loading on Filter (s/mm^2) = N structures / (GOs Counted * GO Area). Results for indirect samples are based on the secondary filter.
(c) Air Concentration (s/cc) = (N structures * EFA) / (GOs Counted * GO Area * F-factor * Air Volume * 1000).
Dust Loading (s/cm2) = (N structures * EFA) / (GOs Counted * GO Area * F-factor * Dust Collection Area).

version 13-DRAFT

Sumas Mtn Asbestos Soil [SFP-078A]

National Asbestos Data Entry Spreadsheet (NADES) for Air & Dust Analysis by Superfund TEM

ANALYTICAL REPORT

FILE NAME: SFP-078A_LabCor Inc._14394163_02-18-15_141014_TEM_D.xls

SAMPLE/ANALYSIS INFORMATION			
**Field Sample Number**	14394163	**Lab Sample Number**	141014-S15
**Media**	Air	**Preparation**	Direct
**Sample Type**	Field Sample	**Sample Status**	Analyzed
**Air Volume (L)**	2174.4	**Analysis Date**	2/18/2015
**QA Sample Type**	Not QC	**Method SOP**	0
**Stopping Rule(s):**	Max Area = n/a, Structures = 100, Sensitivity = 3.00E-04		
**Recording Rule(s):**	Min Aspect Ratio = , Min Length = 0.5µm, Min Width = µm		

* Chrysotile was not counted for all grid openings evaluated; this field is utilized only for the Libby site.

ANALYSIS PARAMETERS	
**Effective filter area (mm2)**	385
**F-factor**	1.00E+00
**Grid opening area (mm2)**	0.0104
**# GOs counted High Magnification**	57
**# GOs counted Low Magnification**	0
**Sensitivity (1/cc)**	
**Total Asbestos**	0.0002981
**PCME**	0.0002981
**Maximum Area Examined**	
**High Magnification**	5.9E-01
**Low Magnification**	0.0E+00

Number of Structures with Fatal Data Entry Errors 0 *(Structures with fatal errors are excluded from calculations below)*

Desired Confidence Interval (%):

Mineral Class	Number of Structures (a)	Loading on Filter (b) (s/mm^2)	Air Conc (c) (s/cc)	% Poisson Confidence Interval for this Sample
**Total TEM Structures**				
Total Asbestos	0	0.0E+00	0.0E+00	0.0E+00 - 2.1E-04
Total Chrysotile (CH)	0	0.0E+00	0.0E+00	0.0E+00 - 2.1E-04
Total Amphibole	0	0.0E+00	0.0E+00	0.0E+00 - 2.1E-04
actinolite (AC)	0	0.0E+00	0.0E+00	0.0E+00 - 2.1E-04
amosite (AM)	0	0.0E+00	0.0E+00	0.0E+00 - 2.1E-04
anthophyllite (AN)	0	0.0E+00	0.0E+00	0.0E+00 - 2.1E-04
crocidolite (CR)	0	0.0E+00	0.0E+00	0.0E+00 - 2.1E-04
tremolite (TR)	0	0.0E+00	0.0E+00	0.0E+00 - 2.1E-04
winchite/richterite/tremolite /actinolite (WRTA)	0	0.0E+00	0.0E+00	0.0E+00 - 2.1E-04
other amphibole (OA)	0	0.0E+00	0.0E+00	0.0E+00 - 2.1E-04
Solid Soln: Amosite	0	0.0E+00	0.0E+00	0.0E+00 - 2.1E-04
Solid Soln: Trem-Act	0	0.0E+00	0.0E+00	0.0E+00 - 2.1E-04
other mineral class (OM)	0	0.0E+00	0.0E+00	0.0E+00 - 2.1E-04
**PCM Equivalent Structures (PCME)**				
Total Asbestos	0	0.0E+00	0.0E+00	0.0E+00 - 2.1E-04
Total Chrysotile (CH)	0	0.0E+00	0.0E+00	0.0E+00 - 2.1E-04
Total Amphibole	0	0.0E+00	0.0E+00	0.0E+00 - 2.1E-04
actinolite (AC)	0	0.0E+00	0.0E+00	0.0E+00 - 2.1E-04
amosite (AM)	0	0.0E+00	0.0E+00	0.0E+00 - 2.1E-04
anthophyllite (AN)	0	0.0E+00	0.0E+00	0.0E+00 - 2.1E-04
crocidolite (CR)	0	0.0E+00	0.0E+00	0.0E+00 - 2.1E-04
tremolite (TR)	0	0.0E+00	0.0E+00	0.0E+00 - 2.1E-04
winchite/richterite/tremolite /actinolite (WRTA)	0	0.0E+00	0.0E+00	0.0E+00 - 2.1E-04
other amphibole (OA)	0	0.0E+00	0.0E+00	0.0E+00 - 2.1E-04
Solid Soln: Amosite	0	0.0E+00	0.0E+00	0.0E+00 - 2.1E-04
Solid Soln: Trem-Act	0	0.0E+00	0.0E+00	0.0E+00 - 2.1E-04
other mineral class (OM)	0	0.0E+00	0.0E+00	0.0E+00 - 2.1E-04

Binning Rule Description:

Apply to fibers (F) only:
L ≥ 0.5µm, AR ≥ 3

No restrictions for other structure types.

ChiSq test for even filter loading for Total TE
(see Annex F2 in ISO 10312)

Filter loading is OK

Binning Rule Description:

Apply to all structures where Total column > 0

L > 5µm, W ≥ 0.25µm and W ≤ 3µm, AR ≥ 3

ChiSq test for even filter loading for PCMe
(see Annex F2 in ISO 10312)

[redacted]

(a) Based on countable structures only.
(b) Loading on Filter (s/mm^2) = N structures / (GOs Counted * GO Area). Results for indirect samples are based on the secondary filter.
(c) Air Concentration (s/cc) = (N structures * EFA) / (GOs Counted * GO Area * F-factor * Air Volume * 1000).
Dust Loading (s/cm2) = (N structures * EFA) / (GOs Counted * GO Area * F-factor * Dust Collection Area).

version 13-DRAFT

Sumas Mtn Asbestos Soil [SFP-078A]

National Asbestos Data Entry Spreadsheet (NADES) for Air & Dust Analysis by Superfund TEM

ANALYTICAL REPORT

FILE NAME: SFP-078A_LabCor Inc._14394164_01-30-15_141014_TEM_D.xls

SAMPLE/ANALYSIS INFORMATION			
**Field Sample Number**	14394164	**Lab Sample Number**	141014-S16
**Media**	Air	**Preparation**	Direct
**Sample Type**	Field Sample	**Sample Status**	Analyzed
**Air Volume (L)**	191.345	**Analysis Date**	1/30/2015
**QA Sample Type**	Not QC	**Method SOP**	0
**Stopping Rule(s):**	Max Area = n/a, Structures = 100, Sensitivity = 3.00E-03		
**Recording Rule(s):**	Min Aspect Ratio = , Min Length = 0.5µm, Min Width = µm		

* Chrysotile was not counted for all grid openings evaluated; this field is utilized only for the Libby site.

ANALYSIS PARAMETERS	
**Effective filter area (mm2)**	385
**F-factor**	1.00E+00
**Grid opening area (mm2)**	0.0104
**# GOs counted High Magnification**	65
**# GOs counted Low Magnification**	0
**Sensitivity (1/cc)**	
**Total Asbestos**	0.0029707
**PCME**	0.0029707
**Maximum Area Examined**	
**High Magnification**	6.8E-01
**Low Magnification**	0.0E+00

Number of Structures with Fatal Data Entry Errors 0 *(Structures with fatal errors are excluded from calculations below)*

Desired Confidence Interval (%):

Mineral Class	Number of Structures (a)	Loading on Filter (b) (s/mm^2)	Air Conc (c) (s/cc)	% Poisson Confidence Interval for this Sample
**Total TEM Structures**				
Total Asbestos	3	4.4E+00	8.9E-03	7.9E-03 - 1.1E-02
Total Chrysotile (CH)	3	4.4E+00	8.9E-03	7.9E-03 - 1.1E-02
Total Amphibole	0	0.0E+00	0.0E+00	0.0E+00 - 2.1E-03
actinolite (AC)	0	0.0E+00	0.0E+00	0.0E+00 - 2.1E-03
amosite (AM)	0	0.0E+00	0.0E+00	0.0E+00 - 2.1E-03
anthophyllite (AN)	0	0.0E+00	0.0E+00	0.0E+00 - 2.1E-03
crocidolite (CR)	0	0.0E+00	0.0E+00	0.0E+00 - 2.1E-03
tremolite (TR)	0	0.0E+00	0.0E+00	0.0E+00 - 2.1E-03
winchite/richterite/tremolite /actinolite (WRTA)	0	0.0E+00	0.0E+00	0.0E+00 - 2.1E-03
other amphibole (OA)	0	0.0E+00	0.0E+00	0.0E+00 - 2.1E-03
Solid Soln: Amosite	0	0.0E+00	0.0E+00	0.0E+00 - 2.1E-03
Solid Soln: Trem-Act	0	0.0E+00	0.0E+00	0.0E+00 - 2.1E-03
other mineral class (OM)	0	0.0E+00	0.0E+00	0.0E+00 - 2.1E-03
**PCM Equivalent Structures (PCME)**				
Total Asbestos	0	0.0E+00	0.0E+00	0.0E+00 - 2.1E-03
Total Chrysotile (CH)	0	0.0E+00	0.0E+00	0.0E+00 - 2.1E-03
Total Amphibole	0	0.0E+00	0.0E+00	0.0E+00 - 2.1E-03
actinolite (AC)	0	0.0E+00	0.0E+00	0.0E+00 - 2.1E-03
amosite (AM)	0	0.0E+00	0.0E+00	0.0E+00 - 2.1E-03
anthophyllite (AN)	0	0.0E+00	0.0E+00	0.0E+00 - 2.1E-03
crocidolite (CR)	0	0.0E+00	0.0E+00	0.0E+00 - 2.1E-03
tremolite (TR)	0	0.0E+00	0.0E+00	0.0E+00 - 2.1E-03
winchite/richterite/tremolite /actinolite (WRTA)	0	0.0E+00	0.0E+00	0.0E+00 - 2.1E-03
other amphibole (OA)	0	0.0E+00	0.0E+00	0.0E+00 - 2.1E-03
Solid Soln: Amosite	0	0.0E+00	0.0E+00	0.0E+00 - 2.1E-03
Solid Soln: Trem-Act	0	0.0E+00	0.0E+00	0.0E+00 - 2.1E-03
other mineral class (OM)	0	0.0E+00	0.0E+00	0.0E+00 - 2.1E-03

Binning Rule Description:

Apply to fibers (F) only:
L ≥ 0.5µm, AR ≥ 3

No restrictions for other structure types.

ChiSq test for even filter loading for Total TE
(see Annex F2 in ISO 10312)

Filter loading appears uneven. Results may be uncertain.

Binning Rule Description:

Apply to all structures where Total column > 0

L > 5µm, W ≥ 0.25µm and W ≤ 3µm, AR ≥ 3

ChiSq test for even filter loading for PCMe
(see Annex F2 in ISO 10312)

[redacted]

(a) Based on countable structures only.
(b) Loading on Filter (s/mm^2) = N structures / (GOs Counted * GO Area). Results for indirect samples are based on the secondary filter
(c) Air Concentration (s/cc) = (N structures * EFA) / (GOs Counted * GO Area * F-factor * Air Volume * 1000)
Dust Loading (s/cm2) = (N structures * EFA) / (GOs Counted * GO Area * F-factor * Dust Collection Area).

version 13-DRAFT

Sumas Mtn Asbestos Soil [SFP-078A]

National Asbestos Data Entry Spreadsheet (NADES) for Air & Dust Analysis by Superfund TEM

ANALYTICAL REPORT

FILE NAME: SFP-078A_LabCor Inc._14394165_01-30-15_141014_TEM_D.xls

SAMPLE/ANALYSIS INFORMATION			
**Field Sample Number**	14394165	**Lab Sample Number**	141014-S17
**Media**	Air	**Preparation**	Direct
**Sample Type**	Field Sample	**Sample Status**	Analyzed
**Air Volume (L)**	189.035	**Analysis Date**	1/30/2015
**QA Sample Type**	Not QC	**Method SOP**	0
**Stopping Rule(s):**	Max Area = , Structures = 100, Sensitivity = 3.00E-03		
**Recording Rule(s):**	Min Aspect Ratio = , Min Length = 0.5µm, Min Width = µm		

* Chrysotile was not counted for all grid openings evaluated; this field is utilized only for the Libby site.

ANALYSIS PARAMETERS	
**Effective filter area (mm2)**	385
**F-factor**	1.00E+00
**Grid opening area (mm2)**	0.0104
**# GOs counted High Magnification**	68
**# GOs counted Low Magnification**	0
**Sensitivity (1/cc)**	
**Total Asbestos**	0.0028744
**PCME**	0.0028744
**Maximum Area Examined**	
**High Magnification**	7.1E-01
**Low Magnification**	0.0E+00

Number of Structures with Fatal Data Entry Errors 0 *(Structures with fatal errors are excluded from calculations below)*

Desired Confidence Interval (%):

Mineral Class	Number of Structures (a)	Loading on Filter (b) (s/mm^2)	Air Conc (c) (s/cc)	% Poisson Confidence Interval for this Sample
**Total TEM Structures**			RC	
Total Asbestos	8	1.1E+01	2.3E-02	2.2E-02 - 2.5E-02
Total Chrysotile (CH)	7	9.9E+00	2.0E-02	1.9E-02 - 2.2E-02
Total Amphibole	1 J	1.4E+00	2.9E-03 IC	2.0E-03 - 4.8E-03
actinolite (AC)	0	0.0E+00	0.0E+00	0.0E+00 - 2.0E-03
amosite (AM)	0	0.0E+00	0.0E+00	0.0E+00 - 2.0E-03
anthophyllite (AN)	1 J	1.4E+00	2.9E-03 IC	2.0E-03 - 4.8E-03
crocidolite (CR)	0	0.0E+00	0.0E+00	0.0E+00 - 2.0E-03
tremolite (TR)	0	0.0E+00	0.0E+00	0.0E+00 - 2.0E-03
winchite/richterite/tremolite /actinolite (WRTA)	0	0.0E+00	0.0E+00	0.0E+00 - 2.0E-03
other amphibole (OA)	0	0.0E+00	0.0E+00	0.0E+00 - 2.0E-03
Solid Soln: Amosite	0	0.0E+00	0.0E+00	0.0E+00 - 2.0E-03
Solid Soln: Trem-Act	0	0.0E+00	0.0E+00	0.0E+00 - 2.0E-03
other mineral class (OM)	0	0.0E+00	0.0E+00	0.0E+00 - 2.0E-03
**PCM Equivalent Structures (PCME)**				
Total Asbestos	0	0.0E+00	0.0E+00	0.0E+00 - 2.0E-03
Total Chrysotile (CH)	0	0.0E+00	0.0E+00	0.0E+00 - 2.0E-03
Total Amphibole	0	0.0E+00	0.0E+00	0.0E+00 - 2.0E-03
actinolite (AC)	0	0.0E+00	0.0E+00	0.0E+00 - 2.0E-03
amosite (AM)	0	0.0E+00	0.0E+00	0.0E+00 - 2.0E-03
anthophyllite (AN)	0	0.0E+00	0.0E+00	0.0E+00 - 2.0E-03
crocidolite (CR)	0	0.0E+00	0.0E+00	0.0E+00 - 2.0E-03
tremolite (TR)	0	0.0E+00	0.0E+00	0.0E+00 - 2.0E-03
winchite/richterite/tremolite /actinolite (WRTA)	0	0.0E+00	0.0E+00	0.0E+00 - 2.0E-03
other amphibole (OA)	0	0.0E+00	0.0E+00	0.0E+00 - 2.0E-03
Solid Soln: Amosite	0	0.0E+00	0.0E+00	0.0E+00 - 2.0E-03
Solid Soln: Trem-Act	0	0.0E+00	0.0E+00	0.0E+00 - 2.0E-03
other mineral class (OM)	0	0.0E+00	0.0E+00	0.0E+00 - 2.0E-03

Binning Rule Description:

Apply to fibers (F) only:
L ≥ 0.5µm, AR ≥ 3

No restrictions for other structure types.

ChiSq test for even filter loading for Total TE (see Annex F2 in ISO 10312)

Filter loading appears uneven. Results may be uncertain.

Binning Rule Description:

Apply to all structures where Total column > 0

L > 5µm, W ≥ 0.25µm and W ≤ 3µm, AR ≥ 3

ChiSq test for even filter loading for PCMe (see Annex F2 in ISO 10312)

[redacted]

(a) Based on countable structures only

(b) Loading on Filter (s/mm^2) = N structures / (GOs Counted * GO Area) Results for indirect samples are based on the secondary filter

(c) Air Concentration (s/cc) = (N structures * EFA) / (GOs Counted * GO Area * F-factor * Air Volume * 1000).
Dust Loading (s/cm2) = (N structures * EFA) / (GOs Counted * GO Area * F-factor * Dust Collection Area).

version 13-DRAFT

Sumas Mtn Asbestos Soil [SFP-078A]

National Asbestos Data Entry Spreadsheet (NADES) for Air & Dust Analysis by Superfund TEM

ANALYTICAL REPORT

FILE NAME: SFP-078A_LabCor Inc._14394169_02-18-15_141014_TEM_D.xls

SAMPLE/ANALYSIS INFORMATION			
**Field Sample Number**	14394169	**Lab Sample Number**	141014-S18
**Media**	Air	**Preparation**	Direct
**Sample Type**	Field Sample	**Sample Status**	Analyzed
**Air Volume (L)**	1702.8	**Analysis Date**	2/18/2015
**QA Sample Type**	Not QC	**Method SOP**	0
**Stopping Rule(s):**	Max Area = n/a, Structures = 100, Sensitivity = 3.00E-04		
**Recording Rule(s):**	Min Aspect Ratio = , Min Length = 0.5µm, Min Width = µm		

* Chrysotile was not counted for all grid openings evaluated; this field is utilized only for the Libby site.

ANALYSIS PARAMETERS	
**Effective filter area (mm2)**	385
**F-factor**	1.00E+00
**Grid opening area (mm2)**	0.0104
**# GOs counted High Magnification**	75
**# GOs counted Low Magnification**	0
**Sensitivity (1/cc)**	
**Total Asbestos**	0.0002893
**PCME**	0.0002893
**Maximum Area Examined**	
**High Magnification**	7.8E-01
**Low Magnification**	0.0E+00

Number of Structures with Fatal Data Entry Errors 0 *(Structures with fatal errors are excluded from calculations below)*

Desired Confidence Interval (%):

Mineral Class	Number of Structures (a)	Loading on Filter (b) (s/mm^2)	Air Conc (c) (s/cc)	% Poisson Confidence Interval for this Sample
**Total TEM Structures**				
Total Asbestos	1	1.3E+00	2.9E-04	2.0E-04 - 4.9E-04
Total Chrysotile (CH)	1	1.3E+00	2.9E-04	2.0E-04 - 4.9E-04
Total Amphibole	0	0.0E+00	0.0E+00	0.0E+00 - 2.0E-04
actinolite (AC)	0	0.0E+00	0.0E+00	0.0E+00 - 2.0E-04
amosite (AM)	0	0.0E+00	0.0E+00	0.0E+00 - 2.0E-04
anthophyllite (AN)	0	0.0E+00	0.0E+00	0.0E+00 - 2.0E-04
crocidolite (CR)	0	0.0E+00	0.0E+00	0.0E+00 - 2.0E-04
tremolite (TR)	0	0.0E+00	0.0E+00	0.0E+00 - 2.0E-04
winchite/richterite/tremolite /actinolite (WRTA)	0	0.0E+00	0.0E+00	0.0E+00 - 2.0E-04
other amphibole (OA)	0	0.0E+00	0.0E+00	0.0E+00 - 2.0E-04
Solid Soln: Amosite	0	0.0E+00	0.0E+00	0.0E+00 - 2.0E-04
Solid Soln: Trem-Act	0	0.0E+00	0.0E+00	0.0E+00 - 2.0E-04
other mineral class (OM)	0	0.0E+00	0.0E+00	0.0E+00 - 2.0E-04
**PCM Equivalent Structures (PCME)**				
Total Asbestos	0	0.0E+00	0.0E+00	0.0E+00 - 2.0E-04
Total Chrysotile (CH)	0	0.0E+00	0.0E+00	0.0E+00 - 2.0E-04
Total Amphibole	0	0.0E+00	0.0E+00	0.0E+00 - 2.0E-04
actinolite (AC)	0	0.0E+00	0.0E+00	0.0E+00 - 2.0E-04
amosite (AM)	0	0.0E+00	0.0E+00	0.0E+00 - 2.0E-04
anthophyllite (AN)	0	0.0E+00	0.0E+00	0.0E+00 - 2.0E-04
crocidolite (CR)	0	0.0E+00	0.0E+00	0.0E+00 - 2.0E-04
tremolite (TR)	0	0.0E+00	0.0E+00	0.0E+00 - 2.0E-04
winchite/richterite/tremolite /actinolite (WRTA)	0	0.0E+00	0.0E+00	0.0E+00 - 2.0E-04
other amphibole (OA)	0	0.0E+00	0.0E+00	0.0E+00 - 2.0E-04
Solid Soln: Amosite	0	0.0E+00	0.0E+00	0.0E+00 - 2.0E-04
Solid Soln: Trem-Act	0	0.0E+00	0.0E+00	0.0E+00 - 2.0E-04
other mineral class (OM)	0	0.0E+00	0.0E+00	0.0E+00 - 2.0E-04

Binning Rule Description:

Apply to fibers (F) only:
L ≥ 0.5µm, AR ≥ 3

No restrictions for other structure types.

ChiSq test for even filter loading for Total TE
(see Annex F2 in ISO 10312)

Filter loading appears uneven. Results may be uncertain.

Binning Rule Description:

Apply to all structures where Total column > 0

L > 5µm, W ≥ 0.25µm and W ≤ 3µm, AR ≥ 3

ChiSq test for even filter loading for PCMe
(see Annex F2 in ISO 10312)

[redacted]

(a) Based on countable structures only

(b) Loading on Filter (s/mm^2) = N structures / (GOs Counted * GO Area). Results for indirect samples are based on the secondary filter.

(c) Air Concentration (s/cc) = (N structures * EFA) / (GOs Counted * GO Area * F-factor * Air Volume * 1000).
Dust Loading (s/cm2) = (N structures * EFA) / (GOs Counted * GO Area * F-factor * Dust Collection Area).

version 13-DRAFT

Sumas Mtn Asbestos Soil [SFP-078A]

National Asbestos Data Entry Spreadsheet (NADES) for Air & Dust Analysis by Superfund TEM

ANALYTICAL REPORT

FILE NAME: SFP-078A_LabCor Inc._14394170_02-18-15_141014_TEM_D.xls

SAMPLE/ANALYSIS INFORMATION			
**Field Sample Number**	14394170	**Lab Sample Number**	141014-S19
**Media**	Air	**Preparation**	Direct
**Sample Type**	Field Sample	**Sample Status**	Analyzed
**Air Volume (L)**	1657.8	**Analysis Date**	2/18/2015
**QA Sample Type**	Not QC	**Method SOP**	0
**Stopping Rule(s):**	Max Area = n/a, Structures = 100, Sensitivity = 3.00E-04		
**Recording Rule(s):**	Min Aspect Ratio = , Min Length = 0.5µm, Min Width = µm		

* Chrysotile was not counted for all grid openings evaluated; this field is utilized only for the Libby site.

ANALYSIS PARAMETERS	
**Effective filter area (mm2)**	385
**F-factor**	1.00E+00
**Grid opening area (mm2)**	0.0104
**# GOs counted High Magnification**	75
**# GOs counted Low Magnification**	0
**Sensitivity (1/cc)**	
**Total Asbestos**	0.0002972
**PCME**	0.0002972
**Maximum Area Examined**	
**High Magnification**	7.8E-01
**Low Magnification**	0.0E+00

Number of Structures with Fatal Data Entry Errors 0 *(Structures with fatal errors are excluded from calculations below)*

Desired Confidence Interval (%):

Mineral Class	Number of Structures (a)	Loading on Filter (b) (s/mm^2)	Air Conc (c) (s/cc)	% Poisson Confidence Interval for this Sample
**Total TEM Structures**				
Total Asbestos	4	5.1E+00	1.2E-03	1.1E-03 - 1.4E-03
Total Chrysotile (CH)	4	5.1E+00	1.2E-03	1.1E-03 - 1.4E-03
Total Amphibole	0	0.0E+00	0.0E+00	0.0E+00 - 2.1E-04
actinolite (AC)	0	0.0E+00	0.0E+00	0.0E+00 - 2.1E-04
amosite (AM)	0	0.0E+00	0.0E+00	0.0E+00 - 2.1E-04
anthophyllite (AN)	0	0.0E+00	0.0E+00	0.0E+00 - 2.1E-04
crocidolite (CR)	0	0.0E+00	0.0E+00	0.0E+00 - 2.1E-04
tremolite (TR)	0	0.0E+00	0.0E+00	0.0E+00 - 2.1E-04
winchite/richterite/tremolite /actinolite (WRTA)	0	0.0E+00	0.0E+00	0.0E+00 - 2.1E-04
other amphibole (OA)	0	0.0E+00	0.0E+00	0.0E+00 - 2.1E-04
Solid Soln: Amosite	0	0.0E+00	0.0E+00	0.0E+00 - 2.1E-04
Solid Soln: Trem-Act	0	0.0E+00	0.0E+00	0.0E+00 - 2.1E-04
other mineral class (OM)	0	0.0E+00	0.0E+00	0.0E+00 - 2.1E-04
**PCM Equivalent Structures (PCME)**				
Total Asbestos	0	0.0E+00	0.0E+00	0.0E+00 - 2.1E-04
Total Chrysotile (CH)	0	0.0E+00	0.0E+00	0.0E+00 - 2.1E-04
Total Amphibole	0	0.0E+00	0.0E+00	0.0E+00 - 2.1E-04
actinolite (AC)	0	0.0E+00	0.0E+00	0.0E+00 - 2.1E-04
amosite (AM)	0	0.0E+00	0.0E+00	0.0E+00 - 2.1E-04
anthophyllite (AN)	0	0.0E+00	0.0E+00	0.0E+00 - 2.1E-04
crocidolite (CR)	0	0.0E+00	0.0E+00	0.0E+00 - 2.1E-04
tremolite (TR)	0	0.0E+00	0.0E+00	0.0E+00 - 2.1E-04
winchite/richterite/tremolite /actinolite (WRTA)	0	0.0E+00	0.0E+00	0.0E+00 - 2.1E-04
other amphibole (OA)	0	0.0E+00	0.0E+00	0.0E+00 - 2.1E-04
Solid Soln: Amosite	0	0.0E+00	0.0E+00	0.0E+00 - 2.1E-04
Solid Soln: Trem-Act	0	0.0E+00	0.0E+00	0.0E+00 - 2.1E-04
other mineral class (OM)	0	0.0E+00	0.0E+00	0.0E+00 - 2.1E-04

Binning Rule Description:

Apply to fibers (F) only:
L ≥ 0.5µm, AR ≥ 3

No restrictions for other structure types.

ChiSq test for even filter loading for Total TE (see Annex F2 in ISO 10312)

Filter loading appears uneven. Results may be uncertain.

Binning Rule Description:

Apply to all structures where Total column > 0

L > 5µm, W ≥ 0.25µm and W ≤ 3µm, AR ≥ 3

ChiSq test for even filter loading for PCMe (see Annex F2 in ISO 10312)

[redacted]

(a) Based on countable structures only.

(b) Loading on Filter (s/mm^2) = N structures / (GOs Counted * GO Area). Results for indirect samples are based on the secondary filter.

(c) Air Concentration (s/cc) = (N structures * EFA) / (GOs Counted * GO Area * F-factor * Air Volume * 1000).
Dust Loading (s/cm2) = (N structures * EFA) / (GOs Counted * GO Area * F-factor * Dust Collection Area).

version 13-DRAFT

Sumas Mtn Asbestos Soil [SFP-078A]

National Asbestos Data Entry Spreadsheet (NADES) for Air & Dust Analysis by Superfund TEM

ANALYTICAL REPORT

FILE NAME: SFP-078A_LabCor Inc._14394171_02-18-15_141014_TEM_D.xls

SAMPLE/ANALYSIS INFORMATION			
**Field Sample Number**	14394171	**Lab Sample Number**	141014-S20
**Media**	Air	**Preparation**	Direct
**Sample Type**	Field Sample	**Sample Status**	Analyzed
**Air Volume (L)**	1647	**Analysis Date**	2/18/2015
**QA Sample Type**	Not QC	**Method SOP**	0
**Stopping Rule(s):**	Max Area = n/a, Structures = 100, Sensitivity = 3.00E-04		
**Recording Rule(s):**	Min Aspect Ratio = , Min Length = 0.5µm, Min Width = µm		

* Chrysotile was not counted for all grid openings evaluated; this field is utilized only for the Libby site.

ANALYSIS PARAMETERS	
**Effective filter area (mm2)**	385
**F-factor**	1.00E+00
**Grid opening area (mm2)**	0.0104
**# GOs counted High Magnification**	75
**# GOs counted Low Magnification**	0
**Sensitivity (1/cc)**	
**Total Asbestos**	0.0002991
**PCME**	0.0002991
**Maximum Area Examined**	
**High Magnification**	7.8E-01
**Low Magnification**	0.0E+00

Number of Structures with Fatal Data Entry Errors 0 *(Structures with fatal errors are excluded from calculations below)*

Desired Confidence Interval (%):

Mineral Class	Number of Structures (a)	Loading on Filter (b) (s/mm^2)	Air Conc (c) (s/cc)	% Poisson Confidence Interval for this Sample
**Total TEM Structures**				
Total Asbestos	3	3.8E+00	9.0E-04	8.0E-04 - 1.1E-03
Total Chrysotile (CH)	3	3.8E+00	9.0E-04	8.0E-04 - 1.1E-03
Total Amphibole	0	0.0E+00	0.0E+00	0.0E+00 - 2.1E-04
actinolite (AC)	0	0.0E+00	0.0E+00	0.0E+00 - 2.1E-04
amosite (AM)	0	0.0E+00	0.0E+00	0.0E+00 - 2.1E-04
anthophyllite (AN)	0	0.0E+00	0.0E+00	0.0E+00 - 2.1E-04
crocidolite (CR)	0	0.0E+00	0.0E+00	0.0E+00 - 2.1E-04
tremolite (TR)	0	0.0E+00	0.0E+00	0.0E+00 - 2.1E-04
winchite/richterite/tremolite /actinolite (WRTA)	0	0.0E+00	0.0E+00	0.0E+00 - 2.1E-04
other amphibole (OA)	0	0.0E+00	0.0E+00	0.0E+00 - 2.1E-04
Solid Soln: Amosite	0	0.0E+00	0.0E+00	0.0E+00 - 2.1E-04
Solid Soln: Trem-Act	0	0.0E+00	0.0E+00	0.0E+00 - 2.1E-04
other mineral class (OM)	0	0.0E+00	0.0E+00	0.0E+00 - 2.1E-04
**PCM Equivalent Structures (PCME)**				
Total Asbestos	1	1.3E+00	3.0E-04	2.1E-04 - 5.0E-04
Total Chrysotile (CH)	1	1.3E+00	3.0E-04	2.1E-04 - 5.0E-04
Total Amphibole	0	0.0E+00	0.0E+00	0.0E+00 - 2.1E-04
actinolite (AC)	0	0.0E+00	0.0E+00	0.0E+00 - 2.1E-04
amosite (AM)	0	0.0E+00	0.0E+00	0.0E+00 - 2.1E-04
anthophyllite (AN)	0	0.0E+00	0.0E+00	0.0E+00 - 2.1E-04
crocidolite (CR)	0	0.0E+00	0.0E+00	0.0E+00 - 2.1E-04
tremolite (TR)	0	0.0E+00	0.0E+00	0.0E+00 - 2.1E-04
winchite/richterite/tremolite /actinolite (WRTA)	0	0.0E+00	0.0E+00	0.0E+00 - 2.1E-04
other amphibole (OA)	0	0.0E+00	0.0E+00	0.0E+00 - 2.1E-04
Solid Soln: Amosite	0	0.0E+00	0.0E+00	0.0E+00 - 2.1E-04
Solid Soln: Trem-Act	0	0.0E+00	0.0E+00	0.0E+00 - 2.1E-04
other mineral class (OM)	0	0.0E+00	0.0E+00	0.0E+00 - 2.1E-04

Binning Rule Description:

Apply to fibers (F) only:
L ≥ 0.5µm, AR ≥ 3

No restrictions for other structure types.

ChiSq test for even filter loading for Total TE (see Annex F2 in ISO 10312)

Filter loading appears uneven. Results may be uncertain.

Binning Rule Description:

Apply to all structures where Total column > 0

L > 5µm, W ≥ 0.25µm and W ≤ 3µm, AR ≥ 3

ChiSq test for even filter loading for PCMe (see Annex F2 in ISO 10312)

Filter loading is OK

[redacted]

(a) Based on countable structures only.

(b) Loading on Filter (s/mm^2) = N structures / (GOs Counted * GO Area). Results for indirect samples are based on the secondary filter.

(c) Air Concentration (s/cc) = (N structures * EFA) / (GOs Counted * GO Area * F-factor * Air Volume * 1000)

Dust Loading (s/cm2) = (N structures * EFA) / (GOs Counted * GO Area * F-factor * Dust Collection Area)

version 13-DRAFT

Sumas Mtn Asbestos Soil [SFP-078A]

National Asbestos Data Entry Spreadsheet (NADES) for Air & Dust Analysis by Superfund TEM

ANALYTICAL REPORT

FILE NAME: SFP-078A_LabCor Inc._14394173_02-01-15_141014_TEM_D.xls

SAMPLE/ANALYSIS INFORMATION			
**Field Sample Number**	14394173	**Lab Sample Number**	141014-S21
**Media**	Air	**Preparation**	Direct
**Sample Type**	Field Blank	**Sample Status**	Analyzed
**Air Volume (L)**		**Analysis Date**	2/1/2015
**QA Sample Type**	Not QC	**Method SOP**	0
**Stopping Rule(s):**	Max Area = n/a, Structures = 100, Sensitivity = 3.00E-03		
**Recording Rule(s):**	Min Aspect Ratio = , Min Length = 0.5µm, Min Width = µm		

* Chrysotile was not counted for all grid openings evaluated; this field is utilized only for the Libby site

ANALYSIS PARAMETERS	
**Effective filter area (mm2)**	385
**F-factor**	1.00E+00
**Grid opening area (mm2)**	0.0104
**# GOs counted High Magnification**	10
**# GOs counted Low Magnification**	0
**Sensitivity (1/cc)**	
**Total Asbestos**	blank
**PCME**	blank
**Maximum Area Examined**	
**High Magnification**	1.0E-01
**Low Magnification**	0.0E+00

Number of Structures with Fatal Data Entry Errors 0 *(Structures with fatal errors are excluded from calculations below)*

Desired Confidence Interval (%):

Mineral Class	Number of Structures (a)	Loading on Filter (b) (s/mm^2)	Air Conc (c) (s/cc)	% Poisson Confidence Interval for this Sample
**Total TEM Structures**				
Total Asbestos	0	0.0E+00	blank	blank - blank
Total Chrysotile (CH)	0	0.0E+00	blank	blank - blank
Total Amphibole	0	0.0E+00	blank	blank - blank
actinolite (AC)	0	0.0E+00	blank	blank - blank
amosite (AM)	0	0.0E+00	blank	blank - blank
anthophyllite (AN)	0	0.0E+00	blank	blank - blank
crocidolite (CR)	0	0.0E+00	blank	blank - blank
tremolite (TR)	0	0.0E+00	blank	blank - blank
winchite/richterite/tremolite /actinolite (WRTA)	0	0.0E+00	blank	blank - blank
other amphibole (OA)	0	0.0E+00	blank	blank - blank
Solid Soln: Amosite	0	0.0E+00	blank	blank - blank
Solid Soln: Trem-Act	0	0.0E+00	blank	blank - blank
other mineral class (OM)	0	0.0E+00	blank	blank - blank
**PCM Equivalent Structures (PCME)**				
Total Asbestos	0	0.0E+00	blank	blank - blank
Total Chrysotile (CH)	0	0.0E+00	blank	blank - blank
Total Amphibole	0	0.0E+00	blank	blank - blank
actinolite (AC)	0	0.0E+00	blank	blank - blank
amosite (AM)	0	0.0E+00	blank	blank - blank
anthophyllite (AN)	0	0.0E+00	blank	blank - blank
crocidolite (CR)	0	0.0E+00	blank	blank - blank
tremolite (TR)	0	0.0E+00	blank	blank - blank
winchite/richterite/tremolite /actinolite (WRTA)	0	0.0E+00	blank	blank - blank
other amphibole (OA)	0	0.0E+00	blank	blank - blank
Solid Soln: Amosite	0	0.0E+00	blank	blank - blank
Solid Soln: Trem-Act	0	0.0E+00	blank	blank - blank
other mineral class (OM)	0	0.0E+00	blank	blank - blank

Binning Rule Description: (Total TEM Structures)

Apply to fibers (F) only:
L ≥ 0.5µm, AR ≥ 3

No restrictions for other structure types.

ChiSq test for even filter loading for Total TE (see Annex F2 in ISO 10312)

Filter loading is OK

Binning Rule Description: (PCME)

Apply to all structures where Total column > 0

L > 5µm, W ≥ 0.25µm and W ≤ 3µm, AR ≥ 3

ChiSq test for even filter loading for PCMe (see Annex F2 in ISO 10312)

[redacted]

(a) Based on countable structures only.
(b) Loading on Filter (s/mm^2) = N structures / (GOs Counted * GO Area). Results for indirect samples are based on the secondary filter.
(c) Air Concentration (s/cc) = (N structures * EFA) / (GOs Counted * GO Area * F-factor * Air Volume * 1000)
Dust Loading (s/cm2) = (N structures * EFA) / (GOs Counted * GO Area * F-factor * Dust Collection Area).

version 13-DRAFT

Sumas Mtn Asbestos Soil [SFP-078A]

National Asbestos Data Entry Spreadsheet (NADES) for Air & Dust Analysis by Superfund TEM

ANALYTICAL REPORT

FILE NAME: SFP-078A_LabCor Inc._14394174_02-01-15_141014_TEM_D.xls

SAMPLE/ANALYSIS INFORMATION				
**Field Sample Number**	14394174	**Lab Sample Number**	141014-S22	
**Media**	Air	**Preparation**	Direct	
**Sample Type**	Field Sample	**Sample Status**	Analyzed	
**Air Volume (L)**	103	**Analysis Date**	2/1/2015 -	2/2/2015
**QA Sample Type**	Not QC	**Method SOP**	0	
**Stopping Rule(s):**	Max Area = n/a, Structures = 100, Sensitivity = 3.00E-03			
**Recording Rule(s):**	Min Aspect Ratio = , Min Length = 0.5µm, Min Width = µm			

* Chrysotile was not counted for all grid openings evaluated; this field is utilized only for the Libby site.

ANALYSIS PARAMETERS	
**Effective filter area (mm2)**	385
**F-factor**	1.00E+00
**Grid opening area (mm2)**	0.0104
**# GOs counted High Magnification**	120
**# GOs counted Low Magnification**	0
**Sensitivity (1/cc)**	
**Total Asbestos**	0.0029893
**PCME**	0.0029893
**Maximum Area Examined**	
**High Magnification**	1.3E+00
**Low Magnification**	0.0E+00

Number of Structures with Fatal Data Entry Errors 0 *(Structures with fatal errors are excluded from calculations below)*

Desired Confidence Interval (%):

Mineral Class	Number of Structures (a)	Loading on Filter (b) (s/mm^2)	Air Conc (c) (s/cc)	% Poisson Confidence Interval for this Sample
**Total TEM Structures**				
Total Asbestos	21	1.7E+01	6.3E-02	6.2E-02 - 6.5E-02
Total Chrysotile (CH)	21	1.7E+01	6.3E-02	6.2E-02 - 6.5E-02
Total Amphibole	0	0.0E+00	0.0E+00	0.0E+00 - 2.1E-03
actinolite (AC)	0	0.0E+00	0.0E+00	0.0E+00 - 2.1E-03
amosite (AM)	0	0.0E+00	0.0E+00	0.0E+00 - 2.1E-03
anthophyllite (AN)	0	0.0E+00	0.0E+00	0.0E+00 - 2.1E-03
crocidolite (CR)	0	0.0E+00	0.0E+00	0.0E+00 - 2.1E-03
tremolite (TR)	0	0.0E+00	0.0E+00	0.0E+00 - 2.1E-03
winchite/richterite/tremolite /actinolite (WRTA)	0	0.0E+00	0.0E+00	0.0E+00 - 2.1E-03
other amphibole (OA)	0	0.0E+00	0.0E+00	0.0E+00 - 2.1E-03
Solid Soln: Amosite	0	0.0E+00	0.0E+00	0.0E+00 - 2.1E-03
Solid Soln: Trem-Act	0	0.0E+00	0.0E+00	0.0E+00 - 2.1E-03
other mineral class (OM)	0	0.0E+00	0.0E+00	0.0E+00 - 2.1E-03
**PCM Equivalent Structures (PCME)**				
Total Asbestos	1	8.0E-01	3.0E-03	2.1E-03 - 5.0E-03
Total Chrysotile (CH)	1	8.0E-01	3.0E-03	2.1E-03 - 5.0E-03
Total Amphibole	0	0.0E+00	0.0E+00	0.0E+00 - 2.1E-03
actinolite (AC)	0	0.0E+00	0.0E+00	0.0E+00 - 2.1E-03
amosite (AM)	0	0.0E+00	0.0E+00	0.0E+00 - 2.1E-03
anthophyllite (AN)	0	0.0E+00	0.0E+00	0.0E+00 - 2.1E-03
crocidolite (CR)	0	0.0E+00	0.0E+00	0.0E+00 - 2.1E-03
tremolite (TR)	0	0.0E+00	0.0E+00	0.0E+00 - 2.1E-03
winchite/richterite/tremolite /actinolite (WRTA)	0	0.0E+00	0.0E+00	0.0E+00 - 2.1E-03
other amphibole (OA)	0	0.0E+00	0.0E+00	0.0E+00 - 2.1E-03
Solid Soln: Amosite	0	0.0E+00	0.0E+00	0.0E+00 - 2.1E-03
Solid Soln: Trem-Act	0	0.0E+00	0.0E+00	0.0E+00 - 2.1E-03
other mineral class (OM)	0	0.0E+00	0.0E+00	0.0E+00 - 2.1E-03

Binning Rule Description:

Apply to fibers (F) only:
L ≥ 0.5µm, AR ≥ 3

No restrictions for other structure types.

ChiSq test for even filter loading for Total TE
(see Annex F2 in ISO 10312)

Filter loading appears uneven. Results may be uncertain.

Binning Rule Description:

Apply to all structures where Total column > 0

L > 5µm, W ≥ 0.25µm and W ≤ 3µm, AR ≥ 3

ChiSq test for even filter loading for PCMe
(see Annex F2 in ISO 10312)

Filter loading is OK

[redacted]

(a) Based on countable structures only
(b) Loading on Filter (s/mm^2) = N structures / (GOs Counted * GO Area) Results for indirect samples are based on the secondary filter.
(c) Air Concentration (s/cc) = (N structures * EFA) / (GOs Counted * GO Area * F-factor * Air Volume * 1000).
Dust Loading (s/cm2) = (N structures * EFA) / (GOs Counted * GO Area * F-factor * Dust Collection Area).

version 13-DRAFT

Sumas Mtn Asbestos Soil [SFP-078A]

National Asbestos Data Entry Spreadsheet (NADES) for Air & Dust Analysis by Superfund TEM

ANALYTICAL REPORT

FILE NAME: SFP-078A_LabCor Inc._14394175_02-03-15_141014_TEM_D.xls

SAMPLE/ANALYSIS INFORMATION			
**Field Sample Number**	14394175	**Lab Sample Number**	141014-S23
**Media**	Air	**Preparation**	Direct
**Sample Type**	Field Sample	**Sample Status**	Analyzed
**Air Volume (L)**	107.6	**Analysis Date**	2/3/2015
**QA Sample Type**	Not QC	**Method SOP**	0
**Stopping Rule(s):**	Max Area = n/a, Structures = 100, Sensitivity = 3.00E-03		
**Recording Rule(s):**	Min Aspect Ratio = , Min Length = 0.5µm, Min Width = µm		

* Chrysotile was not counted for all grid openings evaluated; this field is utilized only for the Libby site.

ANALYSIS PARAMETERS	
**Effective filter area (mm2)**	385
**F-factor**	1.00E+00
**Grid opening area (mm2)**	0.0104
**# GOs counted High Magnification**	115
**# GOs counted Low Magnification**	0
**Sensitivity (1/cc)**	
**Total Asbestos**	0.002986
**PCME**	0.002986
**Maximum Area Examined**	
**High Magnification**	1.2E+00
**Low Magnification**	0.0E+00

Number of Structures with Fatal Data Entry Errors 0 *(Structures with fatal errors are excluded from calculations below)*

Desired Confidence Interval (%):

Mineral Class	Number of Structures (a)	Loading on Filter (b) (s/mm^2)	Air Conc (c) (s/cc)	% Poisson Confidence Interval for this Sample
**Total TEM Structures**				
Total Asbestos	2	1.7E+00	6.0E-03	5.0E-03 - 8.0E-03
Total Chrysotile (CH)	2	1.7E+00	6.0E-03	5.0E-03 - 8.0E-03
Total Amphibole	0	0.0E+00	0.0E+00	0.0E+00 - 2.1E-03
actinolite (AC)	0	0.0E+00	0.0E+00	0.0E+00 - 2.1E-03
amosite (AM)	0	0.0E+00	0.0E+00	0.0E+00 - 2.1E-03
anthophyllite (AN)	0	0.0E+00	0.0E+00	0.0E+00 - 2.1E-03
crocidolite (CR)	0	0.0E+00	0.0E+00	0.0E+00 - 2.1E-03
tremolite (TR)	0	0.0E+00	0.0E+00	0.0E+00 - 2.1E-03
winchite/richterite/tremolite /actinolite (WRTA)	0	0.0E+00	0.0E+00	0.0E+00 - 2.1E-03
other amphibole (OA)	0	0.0E+00	0.0E+00	0.0E+00 - 2.1E-03
Solid Soln: Amosite	0	0.0E+00	0.0E+00	0.0E+00 - 2.1E-03
Solid Soln: Trem-Act	0	0.0E+00	0.0E+00	0.0E+00 - 2.1E-03
other mineral class (OM)	0	0.0E+00	0.0E+00	0.0E+00 - 2.1E-03
**PCM Equivalent Structures (PCME)**				
Total Asbestos	1	8.3E-01	3.0E-03	2.1E-03 - 5.0E-03
Total Chrysotile (CH)	1	8.3E-01	3.0E-03	2.1E-03 - 5.0E-03
Total Amphibole	0	0.0E+00	0.0E+00	0.0E+00 - 2.1E-03
actinolite (AC)	0	0.0E+00	0.0E+00	0.0E+00 - 2.1E-03
amosite (AM)	0	0.0E+00	0.0E+00	0.0E+00 - 2.1E-03
anthophyllite (AN)	0	0.0E+00	0.0E+00	0.0E+00 - 2.1E-03
crocidolite (CR)	0	0.0E+00	0.0E+00	0.0E+00 - 2.1E-03
tremolite (TR)	0	0.0E+00	0.0E+00	0.0E+00 - 2.1E-03
winchite/richterite/tremolite /actinolite (WRTA)	0	0.0E+00	0.0E+00	0.0E+00 - 2.1E-03
other amphibole (OA)	0	0.0E+00	0.0E+00	0.0E+00 - 2.1E-03
Solid Soln: Amosite	0	0.0E+00	0.0E+00	0.0E+00 - 2.1E-03
Solid Soln: Trem-Act	0	0.0E+00	0.0E+00	0.0E+00 - 2.1E-03
other mineral class (OM)	0	0.0E+00	0.0E+00	0.0E+00 - 2.1E-03

Binning Rule Description:

Apply to fibers (F) only:
L ≥ 0.5µm, AR ≥ 3

No restrictions for other structure types.

ChiSq test for even filter loading for Total TE (see Annex F2 in ISO 10312)

Filter loading appears uneven. Results may be uncertain.

Binning Rule Description:

Apply to all structures where Total column > 0

L > 5µm, W ≥ 0.25µm and W ≤ 3µm, AR ≥ 3

ChiSq test for even filter loading for PCMe (see Annex F2 in ISO 10312)

Filter loading is OK

[redacted]

(a) Based on countable structures only

(b) Loading on Filter (s/mm^2) = N structures / (GOs Counted * GO Area) Results for indirect samples are based on the secondary filter

(c) Air Concentration (s/cc) = (N structures * EFA) / (GOs Counted * GO Area * F-factor * Air Volume * 1000).
Dust Loading (s/cm2) = (N structures * EFA) / (GOs Counted * GO Area * F-factor * Dust Collection Area)

version 13-DRAFT

Sumas Mtn Asbestos Soil [SFP-078A]

National Asbestos Data Entry Spreadsheet (NADES) for Air & Dust Analysis by Superfund TEM

ANALYTICAL REPORT

FILE NAME: SFP-078A_LabCor Inc._14394176_02-01-15_141014_TEM_D.xls

SAMPLE/ANALYSIS INFORMATION			
**Field Sample Number**	14394176	**Lab Sample Number**	141014-S24
**Media**	Air	**Preparation**	Direct
**Sample Type**	Field Sample	**Sample Status**	Analyzed
**Air Volume (L)**	493.455	**Analysis Date**	2/1/2015
**QA Sample Type**	Not QC	**Method SOP**	0
**Stopping Rule(s):**	Max Area = n/a, Structures = 100, Sensitivity = 3.00E-03		
**Recording Rule(s):**	Min Aspect Ratio = , Min Length = 0.5µm, Min Width = µm		

* Chrysotile was not counted for all grid openings evaluated; this field is utilized only for the Libby site.

ANALYSIS PARAMETERS	
**Effective filter area (mm2)**	385
**F-factor**	1.00E+00
**Grid opening area (mm2)**	0.0104
**# GOs counted High Magnification**	25
**# GOs counted Low Magnification**	0
**Sensitivity (1/cc)**	
**Total Asbestos**	0.0029951
**PCME**	0.0029951
**Maximum Area Examined**	
**High Magnification**	2.6E-01
**Low Magnification**	0.0E+00

Number of Structures with Fatal Data Entry Errors 0 *(Structures with fatal errors are excluded from calculations below)*

Desired Confidence Interval (%):

Mineral Class	Number of Structures (a)	Loading on Filter (b) (s/mm^2)	Air Conc (c) (s/cc)	% Poisson Confidence Interval for this Sample
**Total TEM Structures**				
Total Asbestos	12	4.6E+01	3.6E-02	3.5E-02 - 3.8E-02
Total Chrysotile (CH)	12	4.6E+01	3.6E-02	3.5E-02 - 3.8E-02
Total Amphibole	0	0.0E+00	0.0E+00	0.0E+00 - 2.1E-03
actinolite (AC)	0	0.0E+00	0.0E+00	0.0E+00 - 2.1E-03
amosite (AM)	0	0.0E+00	0.0E+00	0.0E+00 - 2.1E-03
anthophyllite (AN)	0	0.0E+00	0.0E+00	0.0E+00 - 2.1E-03
crocidolite (CR)	0	0.0E+00	0.0E+00	0.0E+00 - 2.1E-03
tremolite (TR)	0	0.0E+00	0.0E+00	0.0E+00 - 2.1E-03
winchite/richterite/tremolite /actinolite (WRTA)	0	0.0E+00	0.0E+00	0.0E+00 - 2.1E-03
other amphibole (OA)	0	0.0E+00	0.0E+00	0.0E+00 - 2.1E-03
Solid Soln: Amosite	0	0.0E+00	0.0E+00	0.0E+00 - 2.1E-03
Solid Soln: Trem-Act	0	0.0E+00	0.0E+00	0.0E+00 - 2.1E-03
other mineral class (OM)	0	0.0E+00	0.0E+00	0.0E+00 - 2.1E-03
**PCM Equivalent Structures (PCME)**				
Total Asbestos	0	0.0E+00	0.0E+00	0.0E+00 - 2.1E-03
Total Chrysotile (CH)	0	0.0E+00	0.0E+00	0.0E+00 - 2.1E-03
Total Amphibole	0	0.0E+00	0.0E+00	0.0E+00 - 2.1E-03
actinolite (AC)	0	0.0E+00	0.0E+00	0.0E+00 - 2.1E-03
amosite (AM)	0	0.0E+00	0.0E+00	0.0E+00 - 2.1E-03
anthophyllite (AN)	0	0.0E+00	0.0E+00	0.0E+00 - 2.1E-03
crocidolite (CR)	0	0.0E+00	0.0E+00	0.0E+00 - 2.1E-03
tremolite (TR)	0	0.0E+00	0.0E+00	0.0E+00 - 2.1E-03
winchite/richterite/tremolite /actinolite (WRTA)	0	0.0E+00	0.0E+00	0.0E+00 - 2.1E-03
other amphibole (OA)	0	0.0E+00	0.0E+00	0.0E+00 - 2.1E-03
Solid Soln: Amosite	0	0.0E+00	0.0E+00	0.0E+00 - 2.1E-03
Solid Soln: Trem-Act	0	0.0E+00	0.0E+00	0.0E+00 - 2.1E-03
other mineral class (OM)	0	0.0E+00	0.0E+00	0.0E+00 - 2.1E-03

Binning Rule Description:

Apply to fibers (F) only:
L ≥ 0.5µm, AR ≥ 3

No restrictions for other structure types.

ChiSq test for even filter loading for Total TE (see Annex F2 in ISO 10312)

Filter loading appears uneven. Results may be uncertain.

Binning Rule Description:

Apply to all structures where Total column > 0

L > 5µm, W ≥ 0.25µm and W ≤ 3µm, AR ≥ 3

ChiSq test for even filter loading for PCMe (see Annex F2 in ISO 10312)

[redacted]

(a) Based on countable structures only.
(b) Loading on Filter (s/mm^2) = N structures / (GOs Counted * GO Area). Results for indirect samples are based on the secondary filter
(c) Air Concentration (s/cc) = (N structures * EFA) / (GOs Counted * GO Area * F-factor * Air Volume * 1000).
Dust Loading (s/cm2) = (N structures * EFA) / (GOs Counted * GO Area * F-factor * Dust Collection Area).

version 13-DRAFT

Sumas Mtn Asbestos Soil [SFP-078A]

National Asbestos Data Entry Spreadsheet (NADES) for Air & Dust Analysis by Superfund TEM

ANALYTICAL REPORT

FILE NAME: SFP-078A_LabCor Inc._14394178_01-19-15_141014_TEM_D.xls

SAMPLE/ANALYSIS INFORMATION			
**Field Sample Number**	14394178	**Lab Sample Number**	141014-S25
**Media**	Air	**Preparation**	Direct
**Sample Type**	Field Sample	**Sample Status**	Analyzed
**Air Volume (L)**	2203.2	**Analysis Date**	1/19/2015
**QA Sample Type**	Not QC	**Method SOP**	0
**Stopping Rule(s):**	Max Area = n/a, Structures = 100, Sensitivity = 3.00E-04		
**Recording Rule(s):**	Min Aspect Ratio = , Min Length = 0.5µm, Min Width = µm		

* Chrysotile was not counted for all grid openings evaluated, this field is utilized only for the Libby site.

ANALYSIS PARAMETERS	
**Effective filter area (mm2)**	385
**F-factor**	1.00E+00
**Grid opening area (mm2)**	0.0104
**# GOs counted High Magnification**	56
**# GOs counted Low Magnification**	0
**Sensitivity (1/cc)**	
**Total Asbestos**	0.0002995
**PCME**	0.0002995
**Maximum Area Examined**	
**High Magnification**	5.8E-01
**Low Magnification**	0.0E+00

Number of Structures with Fatal Data Entry Errors 0 *(Structures with fatal errors are excluded from calculations below)*

Desired Confidence Interval (%):

Mineral Class	Number of Structures (a)	Loading on Filter (b) (s/mm^2)	Air Conc (c) (s/cc)	% Poisson Confidence Interval for this Sample
**Total TEM Structures**				
Total Asbestos	9	1.5E+01	2.7E-03	2.6E-03 - 2.9E-03
Total Chrysotile (CH)	9	1.5E+01	2.7E-03	2.6E-03 - 2.9E-03
Total Amphibole	0	0.0E+00	0.0E+00	0.0E+00 - 2.1E-04
actinolite (AC)	0	0.0E+00	0.0E+00	0.0E+00 - 2.1E-04
amosite (AM)	0	0.0E+00	0.0E+00	0.0E+00 - 2.1E-04
anthophyllite (AN)	0	0.0E+00	0.0E+00	0.0E+00 - 2.1E-04
crocidolite (CR)	0	0.0E+00	0.0E+00	0.0E+00 - 2.1E-04
tremolite (TR)	0	0.0E+00	0.0E+00	0.0E+00 - 2.1E-04
winchite/richterite/tremolite /actinolite (WRTA)	0	0.0E+00	0.0E+00	0.0E+00 - 2.1E-04
other amphibole (OA)	0	0.0E+00	0.0E+00	0.0E+00 - 2.1E-04
Solid Soln: Amosite	0	0.0E+00	0.0E+00	0.0E+00 - 2.1E-04
Solid Soln: Trem-Act	0	0.0E+00	0.0E+00	0.0E+00 - 2.1E-04
other mineral class (OM)	0	0.0E+00	0.0E+00	0.0E+00 - 2.1E-04
**PCM Equivalent Structures (PCME)**				
Total Asbestos	0	0.0E+00	0.0E+00	0.0E+00 - 2.1E-04
Total Chrysotile (CH)	0	0.0E+00	0.0E+00	0.0E+00 - 2.1E-04
Total Amphibole	0	0.0E+00	0.0E+00	0.0E+00 - 2.1E-04
actinolite (AC)	0	0.0E+00	0.0E+00	0.0E+00 - 2.1E-04
amosite (AM)	0	0.0E+00	0.0E+00	0.0E+00 - 2.1E-04
anthophyllite (AN)	0	0.0E+00	0.0E+00	0.0E+00 - 2.1E-04
crocidolite (CR)	0	0.0E+00	0.0E+00	0.0E+00 - 2.1E-04
tremolite (TR)	0	0.0E+00	0.0E+00	0.0E+00 - 2.1E-04
winchite/richterite/tremolite /actinolite (WRTA)	0	0.0E+00	0.0E+00	0.0E+00 - 2.1E-04
other amphibole (OA)	0	0.0E+00	0.0E+00	0.0E+00 - 2.1E-04
Solid Soln: Amosite	0	0.0E+00	0.0E+00	0.0E+00 - 2.1E-04
Solid Soln: Trem-Act	0	0.0E+00	0.0E+00	0.0E+00 - 2.1E-04
other mineral class (OM)	0	0.0E+00	0.0E+00	0.0E+00 - 2.1E-04

Binning Rule Description:

Apply to fibers (F) only:
L ≥ 0.5µm, AR ≥ 3

No restrictions for other structure types.

ChiSq test for even filter loading for Total TE (see Annex F2 in ISO 10312)

Filter loading appears uneven. Results may be uncertain.

Binning Rule Description:

Apply to all structures where Total column > 0

L > 5µm, W ≥ 0.25µm and W ≤ 3µm, AR ≥ 3

ChiSq test for even filter loading for PCMe (see Annex F2 in ISO 10312)

[redacted]

(a) Based on countable structures only.

(b) Loading on Filter (s/mm^2) = N structures / (GOs Counted * GO Area). Results for indirect samples are based on the secondary filter.

(c) Air Concentration (s/cc) = (N structures * EFA) / (GOs Counted * GO Area * F-factor * Air Volume * 1000).
Dust Loading (s/cm2) = (N structures * EFA) / (GOs Counted * GO Area * F-factor * Dust Collection Area).

version 13-DRAFT

Sumas Mtn Asbestos Soil [SFP-078A]

National Asbestos Data Entry Spreadsheet (NADES) for Air & Dust Analysis by Superfund TEM

ANALYTICAL REPORT

FILE NAME: SFP-078A_LabCor Inc._14394179_01-20-15_141014_TEM_D.xls

SAMPLE/ANALYSIS INFORMATION			
Field Sample Number	14394179	Lab Sample Number	141014-S26
Media	Air	Preparation	Direct
Sample Type	Field Sample	Sample Status	Analyzed
Air Volume (L)	2200.8	Analysis Date	1/20/2015
QA Sample Type	Not QC	Method SOP	0
Stopping Rule(s):	Max Area = n/a, Structures = 100, Sensitivity = 3.00E-04		
Recording Rule(s):	Min Aspect Ratio = , Min Length = 0.5µm, Min Width = µm		

* Chrysotile was not counted for all grid openings evaluated; this field is utilized only for the Libby site.

ANALYSIS PARAMETERS	
Effective filter area (mm2)	385
F-factor	1.00E+00
Grid opening area (mm2)	0.0104
# GOs counted High Magnification	58
# GOs counted Low Magnification	0
Sensitivity (1/cc)	
Total Asbestos	0.0002895
PCME	0.0002895
Maximum Area Examined	
High Magnification	6.0E-01
Low Magnification	0.0E+00

Number of Structures with Fatal Data Entry Errors 0 *(Structures with fatal errors are excluded from calculations below)*

Desired Confidence Interval (%):

Mineral Class	Number of Structures (a)	Loading on Filter (b) (s/mm²)	Air Conc (c) (s/cc)	% Poisson Confidence Interval for this Sample
**Total TEM Structures**				
Total Asbestos	0	0.0E+00	0.0E+00	0.0E+00 - 2.0E-04
Total Chrysotile (CH)	0	0.0E+00	0.0E+00	0.0E+00 - 2.0E-04
Total Amphibole	0	0.0E+00	0.0E+00	0.0E+00 - 2.0E-04
actinolite (AC)	0	0.0E+00	0.0E+00	0.0E+00 - 2.0E-04
amosite (AM)	0	0.0E+00	0.0E+00	0.0E+00 - 2.0E-04
anthophyllite (AN)	0	0.0E+00	0.0E+00	0.0E+00 - 2.0E-04
crocidolite (CR)	0	0.0E+00	0.0E+00	0.0E+00 - 2.0E-04
tremolite (TR)	0	0.0E+00	0.0E+00	0.0E+00 - 2.0E-04
winchite/richterite/tremolite /actinolite (WRTA)	0	0.0E+00	0.0E+00	0.0E+00 - 2.0E-04
other amphibole (OA)	0	0.0E+00	0.0E+00	0.0E+00 - 2.0E-04
Solid Soln: Amosite	0	0.0E+00	0.0E+00	0.0E+00 - 2.0E-04
Solid Soln: Trem-Act	0	0.0E+00	0.0E+00	0.0E+00 - 2.0E-04
other mineral class (OM)	0	0.0E+00	0.0E+00	0.0E+00 - 2.0E-04
**PCM Equivalent Structures (PCME)**				
Total Asbestos	0	0.0E+00	0.0E+00	0.0E+00 - 2.0E-04
Total Chrysotile (CH)	0	0.0E+00	0.0E+00	0.0E+00 - 2.0E-04
Total Amphibole	0	0.0E+00	0.0E+00	0.0E+00 - 2.0E-04
actinolite (AC)	0	0.0E+00	0.0E+00	0.0E+00 - 2.0E-04
amosite (AM)	0	0.0E+00	0.0E+00	0.0E+00 - 2.0E-04
anthophyllite (AN)	0	0.0E+00	0.0E+00	0.0E+00 - 2.0E-04
crocidolite (CR)	0	0.0E+00	0.0E+00	0.0E+00 - 2.0E-04
tremolite (TR)	0	0.0E+00	0.0E+00	0.0E+00 - 2.0E-04
winchite/richterite/tremolite /actinolite (WRTA)	0	0.0E+00	0.0E+00	0.0E+00 - 2.0E-04
other amphibole (OA)	0	0.0E+00	0.0E+00	0.0E+00 - 2.0E-04
Solid Soln: Amosite	0	0.0E+00	0.0E+00	0.0E+00 - 2.0E-04
Solid Soln: Trem-Act	0	0.0E+00	0.0E+00	0.0E+00 - 2.0E-04
other mineral class (OM)	0	0.0E+00	0.0E+00	0.0E+00 - 2.0E-04

Binning Rule Description:

Apply to fibers (F) only:
L ≥ 0.5µm, AR ≥ 3

No restrictions for other structure types.

ChiSq test for even filter loading for Total TE (see Annex F2 in ISO 10312)

Filter loading is OK

Binning Rule Description:

Apply to all structures where Total column > 0

L > 5µm, W ≥ 0.25µm and W ≤ 3µm, AR ≥ 3

ChiSq test for even filter loading for PCMe (see Annex F2 in ISO 10312)

[redacted]

(a) Based on countable structures only.

(b) Loading on Filter (s/mm²) = N structures / (GOs Counted * GO Area). Results for indirect samples are based on the secondary filter.

(c) Air Concentration (s/cc) = (N structures * EFA) / (GOs Counted * GO Area * F-factor * Air Volume * 1000).

Dust Loading (s/cm2) = (N structures * EFA) / (GOs Counted * GO Area * F-factor * Dust Collection Area).

version 13-DRAFT

Sumas Mtn Asbestos Soil [SFP-078A]

National Asbestos Data Entry Spreadsheet (NADES) for Air & Dust Analysis by Superfund TEM

ANALYTICAL REPORT

FILE NAME: SFP-078A_LabCor Inc._14394180_01-20-15_141014_TEM_D.xls

SAMPLE/ANALYSIS INFORMATION

**Field Sample Number**	14394180	**Lab Sample Number**	141014-S27
**Media**	Air	**Preparation**	Direct
**Sample Type**	Field Sample	**Sample Status**	Analyzed
**Air Volume (L)**	2193.6	**Analysis Date**	1/20/2015
**QA Sample Type**	Not QC	**Method SOP**	0

Stopping Rule(s): Max Area = n/a, Structures = 100, Sensitivity = 3.00E-04

Recording Rule(s): Min Aspect Ratio = , Min Length = 0.5µm, Min Width = µm

* Chrysotile was not counted for all grid openings evaluated; this field is utilized only for the Libby site.

ANALYSIS PARAMETERS

**Effective filter area (mm2)**	385
**F-factor**	1.00E+00
**Grid opening area (mm2)**	0.0104
**# GOs counted High Magnification**	57
**# GOs counted Low Magnification**	0
**Sensitivity (1/cc)**	
**Total Asbestos**	0.0002955
**PCME**	0.0002955
**Maximum Area Examined**	
**High Magnification**	5.9E-01
**Low Magnification**	0.0E+00

Number of Structures with Fatal Data Entry Errors 0 *(Structures with fatal errors are excluded from calculations below)*

Desired Confidence Interval (%):

Mineral Class	Number of Structures (a)	Loading on Filter (b) (s/mm²)	Air Conc (c) (s/cc)	% Poisson Confidence Interval for this Sample
**Total TEM Structures**				
Total Asbestos	1	1.7E+00	3.0E-04	2.0E-04 - 5.0E-04
Total Chrysotile (CH)	1	1.7E+00	3.0E-04	2.0E-04 - 5.0E-04
Total Amphibole	0	0.0E+00	0.0E+00	0.0E+00 - 2.0E-04
actinolite (AC)	0	0.0E+00	0.0E+00	0.0E+00 - 2.0E-04
amosite (AM)	0	0.0E+00	0.0E+00	0.0E+00 - 2.0E-04
anthophyllite (AN)	0	0.0E+00	0.0E+00	0.0E+00 - 2.0E-04
crocidolite (CR)	0	0.0E+00	0.0E+00	0.0E+00 - 2.0E-04
tremolite (TR)	0	0.0E+00	0.0E+00	0.0E+00 - 2.0E-04
winchite/richterite/tremolite /actinolite (WRTA)	0	0.0E+00	0.0E+00	0.0E+00 - 2.0E-04
other amphibole (OA)	0	0.0E+00	0.0E+00	0.0E+00 - 2.0E-04
Solid Soln: Amosite	0	0.0E+00	0.0E+00	0.0E+00 - 2.0E-04
Solid Soln: Trem-Act	0	0.0E+00	0.0E+00	0.0E+00 - 2.0E-04
other mineral class (OM)	0	0.0E+00	0.0E+00	0.0E+00 - 2.0E-04
**PCM Equivalent Structures (PCME)**				
Total Asbestos	0	0.0E+00	0.0E+00	0.0E+00 - 2.0E-04
Total Chrysotile (CH)	0	0.0E+00	0.0E+00	0.0E+00 - 2.0E-04
Total Amphibole	0	0.0E+00	0.0E+00	0.0E+00 - 2.0E-04
actinolite (AC)	0	0.0E+00	0.0E+00	0.0E+00 - 2.0E-04
amosite (AM)	0	0.0E+00	0.0E+00	0.0E+00 - 2.0E-04
anthophyllite (AN)	0	0.0E+00	0.0E+00	0.0E+00 - 2.0E-04
crocidolite (CR)	0	0.0E+00	0.0E+00	0.0E+00 - 2.0E-04
tremolite (TR)	0	0.0E+00	0.0E+00	0.0E+00 - 2.0E-04
winchite/richterite/tremolite /actinolite (WRTA)	0	0.0E+00	0.0E+00	0.0E+00 - 2.0E-04
other amphibole (OA)	0	0.0E+00	0.0E+00	0.0E+00 - 2.0E-04
Solid Soln: Amosite	0	0.0E+00	0.0E+00	0.0E+00 - 2.0E-04
Solid Soln: Trem-Act	0	0.0E+00	0.0E+00	0.0E+00 - 2.0E-04
other mineral class (OM)	0	0.0E+00	0.0E+00	0.0E+00 - 2.0E-04

Binning Rule Description:

Apply to fibers (F) only:
L ≥ 0.5µm, AR ≥ 3

No restrictions for other structure types.

ChiSq test for even filter loading for Total TE (see Annex F2 in ISO 10312)

Filter loading is OK

Binning Rule Description:

Apply to all structures where Total column > 0

L > 5µm, W ≥ 0.25µm and W ≤ 3µm, AR ≥ 3

ChiSq test for even filter loading for PCMe (see Annex F2 in ISO 10312)

[redacted]

(a) Based on countable structures only.
(b) Loading on Filter (s/mm²) = N structures / (GOs Counted * GO Area). Results for indirect samples are based on the secondary filter.
(c) Air Concentration (s/cc) = (N structures * EFA) / (GOs Counted * GO Area * F-factor * Air Volume * 1000).
Dust Loading (s/cm2) = (N structures * EFA) / (GOs Counted * GO Area * F-factor * Dust Collection Area)

version 13-DRAFT

Sumas Mtn Asbestos Soil [SFP-078A]

National Asbestos Data Entry Spreadsheet (NADES) for Air & Dust Analysis by Superfund TEM

ANALYTICAL REPORT

FILE NAME: SFP-078A_LabCor Inc._14394181_01-19-15_141014_TEM_D.xls

SAMPLE/ANALYSIS INFORMATION				ANALYSIS PARAMETERS	
**Field Sample Number**	14394181	**Lab Sample Number**	141014-S28	**Effective filter area (mm2)**	385
				**F-factor**	1.00E+00
**Media**	Air	**Preparation**	Direct	**Grid opening area (mm2)**	0.0104
**Sample Type**	Field Sample	**Sample Status**	Analyzed	**# GOs counted High Magnification**	85
**Air Volume (L)**	145.5	**Analysis Date**	1/19/2015 - 1/20/2015	**# GOs counted Low Magnification**	0
**QA Sample Type**	Not QC	**Method SOP**	0	**Sensitivity (1/cc)**	
				**Total Asbestos**	0.0029875
**Stopping Rule(s):**	Max Area = n/a, Structures = 100, Sensitivity = 3.00E-03			**PCME**	0.0029875
**Recording Rule(s):**	Min Aspect Ratio = , Min Length = 0.5µm, Min Width = µm			**Maximum Area Examined**	
				**High Magnification**	8.9E-01
* Chrysotile was not counted for all grid openings evaluated; this field is utilized only for the Libby site.				**Low Magnification**	0.0E+00

Number of Structures with Fatal Data Entry Errors 0 *(Structures with fatal errors are excluded from calculations below)*

Mineral Class	Number of Structures (a)	Loading on Filter (b) (s/mm²)	Air Conc (c) (s/cc)	% Poisson Confidence Interval for this Sample
**Total TEM Structures**				
Total Asbestos	33	3.7E+01	9.9E-02	9.8E-02 - 1.0E-01
Total Chrysotile (CH)	33	3.7E+01	9.9E-02	9.8E-02 - 1.0E-01
Total Amphibole	0	0.0E+00	0.0E+00	0.0E+00 - 2.1E-03
actinolite (AC)	0	0.0E+00	0.0E+00	0.0E+00 - 2.1E-03
amosite (AM)	0	0.0E+00	0.0E+00	0.0E+00 - 2.1E-03
anthophyllite (AN)	0	0.0E+00	0.0E+00	0.0E+00 - 2.1E-03
crocidolite (CR)	0	0.0E+00	0.0E+00	0.0E+00 - 2.1E-03
tremolite (TR)	0	0.0E+00	0.0E+00	0.0E+00 - 2.1E-03
winchite/richterite/tremolite /actinolite (WRTA)	0	0.0E+00	0.0E+00	0.0E+00 - 2.1E-03
other amphibole (OA)	0	0.0E+00	0.0E+00	0.0E+00 - 2.1E-03
Solid Soln: Amosite	0	0.0E+00	0.0E+00	0.0E+00 - 2.1E-03
Solid Soln: Trem-Act	0	0.0E+00	0.0E+00	0.0E+00 - 2.1E-03
other mineral class (OM)	0	0.0E+00	0.0E+00	0.0E+00 - 2.1E-03
**PCM Equivalent Structures (PCME)**				
Total Asbestos	0	0.0E+00	0.0E+00	0.0E+00 - 2.1E-03
Total Chrysotile (CH)	0	0.0E+00	0.0E+00	0.0E+00 - 2.1E-03
Total Amphibole	0	0.0E+00	0.0E+00	0.0E+00 - 2.1E-03
actinolite (AC)	0	0.0E+00	0.0E+00	0.0E+00 - 2.1E-03
amosite (AM)	0	0.0E+00	0.0E+00	0.0E+00 - 2.1E-03
anthophyllite (AN)	0	0.0E+00	0.0E+00	0.0E+00 - 2.1E-03
crocidolite (CR)	0	0.0E+00	0.0E+00	0.0E+00 - 2.1E-03
tremolite (TR)	0	0.0E+00	0.0E+00	0.0E+00 - 2.1E-03
winchite/richterite/tremolite /actinolite (WRTA)	0	0.0E+00	0.0E+00	0.0E+00 - 2.1E-03
other amphibole (OA)	0	0.0E+00	0.0E+00	0.0E+00 - 2.1E-03
Solid Soln: Amosite	0	0.0E+00	0.0E+00	0.0E+00 - 2.1E-03
Solid Soln: Trem-Act	0	0.0E+00	0.0E+00	0.0E+00 - 2.1E-03
other mineral class (OM)	0	0.0E+00	0.0E+00	0.0E+00 - 2.1E-03

Desired Confidence Interval (%):

Binning Rule Description:

Apply to fibers (F) only:
L ≥ 0.5µm, AR ≥ 3

No restrictions for other structure types.

ChiSq test for even filter loading for Total TE (see Annex F2 in ISO 10312)

Filter loading appears uneven. Results may be uncertain.

Binning Rule Description:

Apply to all structures where Total column > 0

L > 5µm, W ≥ 0.25µm and W ≤ 3µm, AR ≥ 3

ChiSq test for even filter loading for PCMe (see Annex F2 in ISO 10312)

[redacted]

(a) Based on countable structures only.
(b) Loading on Filter (s/mm²) = N structures / (GOs Counted * GO Area). Results for indirect samples are based on the secondary filter
(c) Air Concentration (s/cc) = (N structures * EFA) / (GOs Counted * GO Area * F-factor * Air Volume * 1000)
Dust Loading (s/cm2) = (N structures * EFA) / (GOs Counted * GO Area * F-factor * Dust Collection Area).

version 13-DRAFT

Sumas Mtn Asbestos Soil [SFP-078A]

National Asbestos Data Entry Spreadsheet (NADES) for Air & Dust Analysis by Superfund TEM

ANALYTICAL REPORT

FILE NAME: SFP-078A_LabCor Inc._14394182_01-19-15_141014_TEM_D.xls

SAMPLE/ANALYSIS INFORMATION			
**Field Sample Number**	14394182	**Lab Sample Number**	141014-S29
**Media**	Air	**Preparation**	Direct
**Sample Type**	Field Sample	**Sample Status**	Analyzed
**Air Volume (L)**	242.99	**Analysis Date**	1/19/2015
**QA Sample Type**	Not QC	**Method SOP**	0
**Stopping Rule(s):**	Max Area = n/a, Structures = 100, Sensitivity = 3.00E-03		
**Recording Rule(s):**	Min Aspect Ratio = , Min Length = 0.5µm, Min Width = µm		

* Chrysotile was not counted for all grid openings evaluated; this field is utilized only for the Libby site.

ANALYSIS PARAMETERS	
**Effective filter area (mm2)**	385
**F-factor**	1.00E+00
**Grid opening area (mm2)**	0.0104
**# GOs counted High Magnification**	51
**# GOs counted Low Magnification**	0
**Sensitivity (1/cc)**	
**Total Asbestos**	0.0029815
**PCME**	0.0029815
**Maximum Area Examined**	
**High Magnification**	5.3E-01
**Low Magnification**	0.0E+00

Number of Structures with Fatal Data Entry Errors 0 *(Structures with fatal errors are excluded from calculations below)*

Desired Confidence Interval (%):

Mineral Class	Number of Structures (a)	Loading on Filter (b) (s/mm²)	Air Conc (c) (s/cc)	% Poisson Confidence Interval for this Sample
**Total TEM Structures**				
Total Asbestos	0	0.0E+00	0.0E+00	0.0E+00 - 2.1E-03
Total Chrysotile (CH)	0	0.0E+00	0.0E+00	0.0E+00 - 2.1E-03
Total Amphibole	0	0.0E+00	0.0E+00	0.0E+00 - 2.1E-03
actinolite (AC)	0	0.0E+00	0.0E+00	0.0E+00 - 2.1E-03
amosite (AM)	0	0.0E+00	0.0E+00	0.0E+00 - 2.1E-03
anthophyllite (AN)	0	0.0E+00	0.0E+00	0.0E+00 - 2.1E-03
crocidolite (CR)	0	0.0E+00	0.0E+00	0.0E+00 - 2.1E-03
tremolite (TR)	0	0.0E+00	0.0E+00	0.0E+00 - 2.1E-03
winchite/richterite/tremolite /actinolite (WRTA)	0	0.0E+00	0.0E+00	0.0E+00 - 2.1E-03
other amphibole (OA)	0	0.0E+00	0.0E+00	0.0E+00 - 2.1E-03
Solid Soln: Amosite	0	0.0E+00	0.0E+00	0.0E+00 - 2.1E-03
Solid Soln: Trem-Act	0	0.0E+00	0.0E+00	0.0E+00 - 2.1E-03
other mineral class (OM)	0	0.0E+00	0.0E+00	0.0E+00 - 2.1E-03
**PCM Equivalent Structures (PCME)**				
Total Asbestos	0	0.0E+00	0.0E+00	0.0E+00 - 2.1E-03
Total Chrysotile (CH)	0	0.0E+00	0.0E+00	0.0E+00 - 2.1E-03
Total Amphibole	0	0.0E+00	0.0E+00	0.0E+00 - 2.1E-03
actinolite (AC)	0	0.0E+00	0.0E+00	0.0E+00 - 2.1E-03
amosite (AM)	0	0.0E+00	0.0E+00	0.0E+00 - 2.1E-03
anthophyllite (AN)	0	0.0E+00	0.0E+00	0.0E+00 - 2.1E-03
crocidolite (CR)	0	0.0E+00	0.0E+00	0.0E+00 - 2.1E-03
tremolite (TR)	0	0.0E+00	0.0E+00	0.0E+00 - 2.1E-03
winchite/richterite/tremolite /actinolite (WRTA)	0	0.0E+00	0.0E+00	0.0E+00 - 2.1E-03
other amphibole (OA)	0	0.0E+00	0.0E+00	0.0E+00 - 2.1E-03
Solid Soln: Amosite	0	0.0E+00	0.0E+00	0.0E+00 - 2.1E-03
Solid Soln: Trem-Act	0	0.0E+00	0.0E+00	0.0E+00 - 2.1E-03
other mineral class (OM)	0	0.0E+00	0.0E+00	0.0E+00 - 2.1E-03

Binning Rule Description:

Apply to fibers (F) only:
L ≥ 0.5µm, AR ≥ 3

No restrictions for other structure types.

ChiSq test for even filter loading for Total TE (see Annex F2 in ISO 10312)

Filter loading is OK

Binning Rule Description:

Apply to all structures where Total column > 0

L > 5µm, W ≥ 0.25µm and W ≤ 3µm, AR ≥ 3

ChiSq test for even filter loading for PCMe (see Annex F2 in ISO 10312)

[redacted]

(a) Based on countable structures only.
(b) Loading on Filter (s/mm²) = N structures / (GOs Counted * GO Area). Results for indirect samples are based on the secondary filter.
(c) Air Concentration (s/cc) = (N structures * EFA) / (GOs Counted * GO Area * F-factor * Air Volume * 1000).
Dust Loading (s/cm2) = (N structures * EFA) / (GOs Counted * GO Area * F-factor * Dust Collection Area).

version 13-DRAFT

Sumas Mtn Asbestos Soil [SFP-078A]

National Asbestos Data Entry Spreadsheet (NADES) for Air & Dust Analysis by Superfund TEM

ANALYTICAL REPORT

FILE NAME: SFP-078A_LabCor Inc._14394183_01-19-15_141014_TEM_D.xls

SAMPLE/ANALYSIS INFORMATION			
**Field Sample Number**	14394183	**Lab Sample Number**	141014-S30
**Media**	Air	**Preparation**	Direct
**Sample Type**	Field Sample	**Sample Status**	Analyzed
**Air Volume (L)**	253.33	**Analysis Date**	1/19/2015
**QA Sample Type**	Not QC	**Method SOP**	0
**Stopping Rule(s):**	Max Area = n/a, Structures = 100, Sensitivity = 3.00E-03		
**Recording Rule(s):**	Min Aspect Ratio = , Min Length = 0.5µm, Min Width = µm		

* Chrysotile was not counted for all grid openings evaluated; this field is utilized only for the Libby site

ANALYSIS PARAMETERS	
**Effective filter area (mm2)**	385
**F-factor**	1.00E+00
**Grid opening area (mm2)**	0.0104
**# GOs counted High Magnification**	49
**# GOs counted Low Magnification**	0
**Sensitivity (1/cc)**	
**Total Asbestos**	0.0029765
**PCME**	0.0029765
**Maximum Area Examined**	
**High Magnification**	5.1E-01
**Low Magnification**	0.0E+00

Number of Structures with Fatal Data Entry Errors 0 *(Structures with fatal errors are excluded from calculations below)*

Desired Confidence Interval (%):

Mineral Class	Number of Structures (a)	Loading on Filter (b) (s/mm^2)	Air Conc (c) (s/cc)	% Poisson Confidence Interval for this Sample
**Total TEM Structures**				
Total Asbestos	0	0.0E+00	0.0E+00	0.0E+00 - 2.1E-03
Total Chrysotile (CH)	0	0.0E+00	0.0E+00	0.0E+00 - 2.1E-03
Total Amphibole	0	0.0E+00	0.0E+00	0.0E+00 - 2.1E-03
actinolite (AC)	0	0.0E+00	0.0E+00	0.0E+00 - 2.1E-03
amosite (AM)	0	0.0E+00	0.0E+00	0.0E+00 - 2.1E-03
anthophyllite (AN)	0	0.0E+00	0.0E+00	0.0E+00 - 2.1E-03
crocidolite (CR)	0	0.0E+00	0.0E+00	0.0E+00 - 2.1E-03
tremolite (TR)	0	0.0E+00	0.0E+00	0.0E+00 - 2.1E-03
winchite/richterite/tremolite /actinolite (WRTA)	0	0.0E+00	0.0E+00	0.0E+00 - 2.1E-03
other amphibole (OA)	0	0.0E+00	0.0E+00	0.0E+00 - 2.1E-03
Solid Soln: Amosite	0	0.0E+00	0.0E+00	0.0E+00 - 2.1E-03
Solid Soln: Trem-Act	0	0.0E+00	0.0E+00	0.0E+00 - 2.1E-03
other mineral class (OM)	0	0.0E+00	0.0E+00	0.0E+00 - 2.1E-03
**PCM Equivalent Structures (PCME)**				
Total Asbestos	0	0.0E+00	0.0E+00	0.0E+00 - 2.1E-03
Total Chrysotile (CH)	0	0.0E+00	0.0E+00	0.0E+00 - 2.1E-03
Total Amphibole	0	0.0E+00	0.0E+00	0.0E+00 - 2.1E-03
actinolite (AC)	0	0.0E+00	0.0E+00	0.0E+00 - 2.1E-03
amosite (AM)	0	0.0E+00	0.0E+00	0.0E+00 - 2.1E-03
anthophyllite (AN)	0	0.0E+00	0.0E+00	0.0E+00 - 2.1E-03
crocidolite (CR)	0	0.0E+00	0.0E+00	0.0E+00 - 2.1E-03
tremolite (TR)	0	0.0E+00	0.0E+00	0.0E+00 - 2.1E-03
winchite/richterite/tremolite /actinolite (WRTA)	0	0.0E+00	0.0E+00	0.0E+00 - 2.1E-03
other amphibole (OA)	0	0.0E+00	0.0E+00	0.0E+00 - 2.1E-03
Solid Soln: Amosite	0	0.0E+00	0.0E+00	0.0E+00 - 2.1E-03
Solid Soln: Trem-Act	0	0.0E+00	0.0E+00	0.0E+00 - 2.1E-03
other mineral class (OM)	0	0.0E+00	0.0E+00	0.0E+00 - 2.1E-03

Binning Rule Description:

Apply to fibers (F) only:
L ≥ 0.5µm, AR ≥ 3

No restrictions for other structure types.

ChiSq test for even filter loading for Total TE
(see Annex F2 in ISO 10312)

Filter loading is OK

Binning Rule Description:

Apply to all structures where Total column > 0

L > 5µm, W ≥ 0.25µm and W ≤ 3µm, AR ≥ 3

ChiSq test for even filter loading for PCMe
(see Annex F2 in ISO 10312)

[redacted]

(a) Based on countable structures only.

(b) Loading on Filter (s/mm^2) = N structures / (GOs Counted * GO Area). Results for indirect samples are based on the secondary filter.

(c) Air Concentration (s/cc) = (N structures * EFA) / (GOs Counted * GO Area * F-factor * Air Volume * 1000).
Dust Loading (s/cm2) = (N structures * EFA) / (GOs Counted * GO Area * F-factor * Dust Collection Area).

version 13-DRAFT

Sumas Mtn Asbestos Soil [SFP-078A]

National Asbestos Data Entry Spreadsheet (NADES) for Air & Dust Analysis by Superfund TEM

ANALYTICAL REPORT

FILE NAME: SFP-078A_LabCor Inc._14394186_01-20-15_141014_TEM_D.xls

SAMPLE/ANALYSIS INFORMATION				
**Field Sample Number**	14394186	**Lab Sample Number**	141014-S31	
**Media**	Air	**Preparation**	Direct	
**Sample Type**	Field Sample	**Sample Status**	Analyzed	
**Air Volume (L)**	843.3	**Analysis Date**	1/20/2015	1/21/2015
**QA Sample Type**	Not QC	**Method SOP**	0	
**Stopping Rule(s):**	Max Area = n/a, Structures = 100, Sensitivity = 3.00E-04			
**Recording Rule(s):**	Min Aspect Ratio = , Min Length = 0.5µm, Min Width = µm			

* Chrysotile was not counted for all grid openings evaluated; this field is utilized only for the Libby site.

ANALYSIS PARAMETERS	
**Effective filter area (mm2)**	385
**F-factor**	1.00E+00
**Grid opening area (mm2)**	0.0104
**# GOs counted High Magnification**	147
**# GOs counted Low Magnification**	0
**Sensitivity (1/cc)**	
**Total Asbestos**	0.0002981
**PCME**	0.0002981
**Maximum Area Examined**	
**High Magnification**	1.5E+00
**Low Magnification**	0.0E+00

Number of Structures with Fatal Data Entry Errors 0 *(Structures with fatal errors are excluded from calculations below)*

Desired Confidence Interval (%):

Mineral Class	Number of Structures (a)	Loading on Filter (b) (s/mm^2)	Air Conc (c) (s/cc)	% Poisson Confidence Interval for this Sample
**Total TEM Structures**				
Total Asbestos	7	4.6E+00	2.1E-03	2.0E-03 - 2.3E-03
Total Chrysotile (CH)	7	4.6E+00	2.1E-03	2.0E-03 - 2.3E-03
Total Amphibole	0	0.0E+00	0.0E+00	0.0E+00 - 2.1E-04
actinolite (AC)	0	0.0E+00	0.0E+00	0.0E+00 - 2.1E-04
amosite (AM)	0	0.0E+00	0.0E+00	0.0E+00 - 2.1E-04
anthophyllite (AN)	0	0.0E+00	0.0E+00	0.0E+00 - 2.1E-04
crocidolite (CR)	0	0.0E+00	0.0E+00	0.0E+00 - 2.1E-04
tremolite (TR)	0	0.0E+00	0.0E+00	0.0E+00 - 2.1E-04
winchite/richterite/tremolite /actinolite (WRTA)	0	0.0E+00	0.0E+00	0.0E+00 - 2.1E-04
other amphibole (OA)	0	0.0E+00	0.0E+00	0.0E+00 - 2.1E-04
Solid Soln: Amosite	0	0.0E+00	0.0E+00	0.0E+00 - 2.1E-04
Solid Soln: Trem-Act	0	0.0E+00	0.0E+00	0.0E+00 - 2.1E-04
other mineral class (OM)	0	0.0E+00	0.0E+00	0.0E+00 - 2.1E-04
**PCM Equivalent Structures (PCME)**				
Total Asbestos	1	6.5E-01	3.0E-04	2.1E-04 - 5.0E-04
Total Chrysotile (CH)	1	6.5E-01	3.0E-04	2.1E-04 - 5.0E-04
Total Amphibole	0	0.0E+00	0.0E+00	0.0E+00 - 2.1E-04
actinolite (AC)	0	0.0E+00	0.0E+00	0.0E+00 - 2.1E-04
amosite (AM)	0	0.0E+00	0.0E+00	0.0E+00 - 2.1E-04
anthophyllite (AN)	0	0.0E+00	0.0E+00	0.0E+00 - 2.1E-04
crocidolite (CR)	0	0.0E+00	0.0E+00	0.0E+00 - 2.1E-04
tremolite (TR)	0	0.0E+00	0.0E+00	0.0E+00 - 2.1E-04
winchite/richterite/tremolite /actinolite (WRTA)	0	0.0E+00	0.0E+00	0.0E+00 - 2.1E-04
other amphibole (OA)	0	0.0E+00	0.0E+00	0.0E+00 - 2.1E-04
Solid Soln: Amosite	0	0.0E+00	0.0E+00	0.0E+00 - 2.1E-04
Solid Soln: Trem-Act	0	0.0E+00	0.0E+00	0.0E+00 - 2.1E-04
other mineral class (OM)	0	0.0E+00	0.0E+00	0.0E+00 - 2.1E-04

Binning Rule Description:

Apply to fibers (F) only:
L ≥ 0.5µm, AR ≥ 3

No restrictions for other structure types.

ChiSq test for even filter loading for Total TE (see Annex F2 in ISO 10312)

Filter loading appears uneven. Results may be uncertain.

Binning Rule Description:

Apply to all structures where Total column > 0

L > 5µm, W ≥ 0.25µm and W ≤ 3µm, AR ≥ 3

ChiSq test for even filter loading for PCMe (see Annex F2 in ISO 10312)

Filter loading is OK

[redacted]

(a) Based on countable structures only.

(b) Loading on Filter (s/mm^2) = N structures / (GOs Counted * GO Area). Results for indirect samples are based on the secondary filter

(c) Air Concentration (s/cc) = (N structures * EFA) / (GOs Counted * GO Area * F-factor * Air Volume * 1000).

Dust Loading (s/cm2) = (N structures * EFA) / (GOs Counted * GO Area * F-factor * Dust Collection Area).

version 13-DRAFT

Sumas Mtn Asbestos Soil [SFP-078A]

National Asbestos Data Entry Spreadsheet (NADES) for Air & Dust Analysis by Superfund TEM

ANALYTICAL REPORT

FILE NAME: SFP-078A_LabCor Inc._14394187_01-21-15_141014_TEM_D.xls

SAMPLE/ANALYSIS INFORMATION			
**Field Sample Number**	14394187	**Lab Sample Number**	141014-S32
**Media**	Air	**Preparation**	Direct
**Sample Type**	Field Sample	**Sample Status**	Analyzed
**Air Volume (L)**	812.7	**Analysis Date**	1/21/2015
**QA Sample Type**	Not QC	**Method SOP**	0
**Stopping Rule(s):**	Max Area = n/a, Structures = 100, Sensitivity = 3.00E-04		
**Recording Rule(s):**	Min Aspect Ratio = , Min Length = 0.5µm, Min Width = µm		

* Chrysotile was not counted for all grid openings evaluated; this field is utilized only for the Libby site.

ANALYSIS PARAMETERS	
**Effective filter area (mm2)**	385
**F-factor**	1.00E+00
**Grid opening area (mm2)**	0.0104
**# GOs counted High Magnification**	157
**# GOs counted Low Magnification**	0
**Sensitivity (1/cc)**	
**Total Asbestos**	0.0002896
**PCME**	0.0002896
**Maximum Area Examined**	
**High Magnification**	1.6E+00
**Low Magnification**	0.0E+00

Number of Structures with Fatal Data Entry Errors 0 *(Structures with fatal errors are excluded from calculations below)*

Desired Confidence Interval (%):

Mineral Class	Number of Structures (a)	Loading on Filter (b) (s/mm^2)	Air Conc (c) (s/cc)	% Poisson Confidence Interval for this Sample
**Total TEM Structures**				
Total Asbestos	6	3.7E+00	1.7E-03	1.6E-03 - 1.9E-03
Total Chrysotile (CH)	6	3.7E+00	1.7E-03	1.6E-03 - 1.9E-03
Total Amphibole	0	0.0E+00	0.0E+00	0.0E+00 - 2.0E-04
actinolite (AC)	0	0.0E+00	0.0E+00	0.0E+00 - 2.0E-04
amosite (AM)	0	0.0E+00	0.0E+00	0.0E+00 - 2.0E-04
anthophyllite (AN)	0	0.0E+00	0.0E+00	0.0E+00 - 2.0E-04
crocidolite (CR)	0	0.0E+00	0.0E+00	0.0E+00 - 2.0E-04
tremolite (TR)	0	0.0E+00	0.0E+00	0.0E+00 - 2.0E-04
winchite/richterite/tremolite /actinolite (WRTA)	0	0.0E+00	0.0E+00	0.0E+00 - 2.0E-04
other amphibole (OA)	0	0.0E+00	0.0E+00	0.0E+00 - 2.0E-04
Solid Soln: Amosite	0	0.0E+00	0.0E+00	0.0E+00 - 2.0E-04
Solid Soln: Trem-Act	0	0.0E+00	0.0E+00	0.0E+00 - 2.0E-04
other mineral class (OM)	0	0.0E+00	0.0E+00	0.0E+00 - 2.0E-04
**PCM Equivalent Structures (PCME)**				
Total Asbestos	0	0.0E+00	0.0E+00	0.0E+00 - 2.0E-04
Total Chrysotile (CH)	0	0.0E+00	0.0E+00	0.0E+00 - 2.0E-04
Total Amphibole	0	0.0E+00	0.0E+00	0.0E+00 - 2.0E-04
actinolite (AC)	0	0.0E+00	0.0E+00	0.0E+00 - 2.0E-04
amosite (AM)	0	0.0E+00	0.0E+00	0.0E+00 - 2.0E-04
anthophyllite (AN)	0	0.0E+00	0.0E+00	0.0E+00 - 2.0E-04
crocidolite (CR)	0	0.0E+00	0.0E+00	0.0E+00 - 2.0E-04
tremolite (TR)	0	0.0E+00	0.0E+00	0.0E+00 - 2.0E-04
winchite/richterite/tremolite /actinolite (WRTA)	0	0.0E+00	0.0E+00	0.0E+00 - 2.0E-04
other amphibole (OA)	0	0.0E+00	0.0E+00	0.0E+00 - 2.0E-04
Solid Soln: Amosite	0	0.0E+00	0.0E+00	0.0E+00 - 2.0E-04
Solid Soln: Trem-Act	0	0.0E+00	0.0E+00	0.0E+00 - 2.0E-04
other mineral class (OM)	0	0.0E+00	0.0E+00	0.0E+00 - 2.0E-04

Binning Rule Description:

Apply to fibers (F) only:
L ≥ 0.5µm, AR ≥ 3

No restrictions for other structure types.

ChiSq test for even filter loading for Total TE
(see Annex F2 in ISO 10312)

Filter loading appears uneven. Results may be uncertain.

Binning Rule Description:

Apply to all structures where Total column > 0

L > 5µm, W ≥ 0.25µm and W ≤ 3µm, AR ≥ 3

ChiSq test for even filter loading for PCMe
(see Annex F2 in ISO 10312)

(a) Based on countable structures only.
(b) Loading on Filter (s/mm^2) = N structures / (GOs Counted * GO Area). Results for indirect samples are based on the secondary filter.
(c) Air Concentration (s/cc) = (N structures * EFA) / (GOs Counted * GO Area * F-factor * Air Volume * 1000).
Dust Loading (s/cm2) = (N structures * EFA) / (GOs Counted * GO Area * F-factor * Dust Collection Area).

version 13-DRAFT

Sumas Mtn Asbestos Soil [SFP-078A]

National Asbestos Data Entry Spreadsheet (NADES) for Air & Dust Analysis by Superfund TEM

ANALYTICAL REPORT

FILE NAME: SFP-078A_LabCor Inc._14394188_01-22-15_141014_TEM_D.xls

SAMPLE/ANALYSIS INFORMATION				
**Field Sample Number**	14394188	**Lab Sample Number**	141014-S33	
**Media**	Air	**Preparation**	Direct	
**Sample Type**	Field Sample	**Sample Status**	Analyzed	
**Air Volume (L)**	811.8	**Analysis Date**	1/22/2015 -	1/23/2015
**QA Sample Type**	Not QC	**Method SOP**	0	
**Stopping Rule(s):**	Max Area = n/a, Structures = 100, Sensitivity = 3.00E-04			
**Recording Rule(s):**	Min Aspect Ratio = , Min Length = 0.5µm, Min Width = µm			

* Chrysotile was not counted for all grid openings evaluated; this field is utilized only for the Libby site.

ANALYSIS PARAMETERS	
**Effective filter area (mm2)**	385
**F-factor**	1.00E+00
**Grid opening area (mm2)**	0.0104
**# GOs counted High Magnification**	152
**# GOs counted Low Magnification**	0
**Sensitivity (1/cc)**	
**Total Asbestos**	0.0002994
**PCME**	0.0002994
**Maximum Area Examined**	
**High Magnification**	1.6E+00
**Low Magnification**	0.0E+00

Number of Structures with Fatal Data Entry Errors 0 *(Structures with fatal errors are excluded from calculations below)*

Desired Confidence Interval (%):

Mineral Class	Number of Structures (a)	Loading on Filter (b) (s/mm^2)	Air Conc (c) (s/cc)	% Poisson Confidence Interval for this Sample
**Total TEM Structures**				
Total Asbestos	0	0.0E+00	0.0E+00	0.0E+00 - 2.1E-04
Total Chrysotile (CH)	0	0.0E+00	0.0E+00	0.0E+00 - 2.1E-04
Total Amphibole	0	0.0E+00	0.0E+00	0.0E+00 - 2.1E-04
actinolite (AC)	0	0.0E+00	0.0E+00	0.0E+00 - 2.1E-04
amosite (AM)	0	0.0E+00	0.0E+00	0.0E+00 - 2.1E-04
anthophyllite (AN)	0	0.0E+00	0.0E+00	0.0E+00 - 2.1E-04
crocidolite (CR)	0	0.0E+00	0.0E+00	0.0E+00 - 2.1E-04
tremolite (TR)	0	0.0E+00	0.0E+00	0.0E+00 - 2.1E-04
winchite/richterite/tremolite /actinolite (WRTA)	0	0.0E+00	0.0E+00	0.0E+00 - 2.1E-04
other amphibole (OA)	0	0.0E+00	0.0E+00	0.0E+00 - 2.1E-04
Solid Soln: Amosite	0	0.0E+00	0.0E+00	0.0E+00 - 2.1E-04
Solid Soln: Trem-Act	0	0.0E+00	0.0E+00	0.0E+00 - 2.1E-04
other mineral class (OM)	0	0.0E+00	0.0E+00	0.0E+00 - 2.1E-04
**PCM Equivalent Structures (PCME)**				
Total Asbestos	0	0.0E+00	0.0E+00	0.0E+00 - 2.1E-04
Total Chrysotile (CH)	0	0.0E+00	0.0E+00	0.0E+00 - 2.1E-04
Total Amphibole	0	0.0E+00	0.0E+00	0.0E+00 - 2.1E-04
actinolite (AC)	0	0.0E+00	0.0E+00	0.0E+00 - 2.1E-04
amosite (AM)	0	0.0E+00	0.0E+00	0.0E+00 - 2.1E-04
anthophyllite (AN)	0	0.0E+00	0 0E+00	0.0E+00 - 2.1E-04
crocidolite (CR)	0	0.0E+00	0.0E+00	0.0E+00 - 2.1E-04
tremolite (TR)	0	0.0E+00	0.0E+00	0.0E+00 - 2.1E-04
winchite/richterite/tremolite /actinolite (WRTA)	0	0.0E+00	0.0E+00	0.0E+00 - 2.1E-04
other amphibole (OA)	0	0.0E+00	0.0E+00	0.0E+00 - 2.1E-04
Solid Soln: Amosite	0	0.0E+00	0.0E+00	0.0E+00 - 2.1E-04
Solid Soln: Trem-Act	0	0.0E+00	0.0E+00	0.0E+00 - 2.1E-04
other mineral class (OM)	0	0.0E+00	0.0E+00	0.0E+00 - 2.1E-04

Binning Rule Description:

Apply to fibers (F) only:
L ≥ 0.5µm, AR ≥ 3

No restrictions for other structure types.

ChiSq test for even filter loading for Total TE
(see Annex F2 in ISO 10312)

Filter loading is OK

Binning Rule Description:

Apply to all structures where Total column > 0

L > 5µm, W ≥ 0.25µm and W ≤ 3µm, AR ≥ 3

ChiSq test for even filter loading for PCMe
(see Annex F2 in ISO 10312)

[redacted]

(a) Based on countable structures only.
(b) Loading on Filter (s/mm^2) = N structures / (GOs Counted * GO Area). Results for indirect samples are based on the secondary filter.
(c) Air Concentration (s/cc) = (N structures * EFA) / (GOs Counted * GO Area * F-factor * Air Volume * 1000).
Dust Loading (s/cm2) = (N structures * EFA) / (GOs Counted * GO Area * F-factor * Dust Collection Area).

version 13-DRAFT

Sumas Mtn Asbestos Soil [SFP-078A]

National Asbestos Data Entry Spreadsheet (NADES) for Air & Dust Analysis by Superfund TEM

ANALYTICAL REPORT

FILE NAME: SFP-078A_LabCor Inc._14394153_02-24-15_141014_TEM_D_RD.xls

SAMPLE/ANALYSIS INFORMATION			
**Field Sample Number**	14394153	**Lab Sample Number**	141014-S8
**Media**	Air	**Preparation**	Direct
**Sample Type**	Field Sample	**Sample Status**	Analyzed
**Air Volume (L)**	2277.6	**Analysis Date**	2/24/2015
**QA Sample Type**	RD	**Method SOP**	0
**Stopping Rule(s):**	Max Area = n/a, Structures = 100, Sensitivity = 3.00E-04		
**Recording Rule(s):**	Min Aspect Ratio = , Min Length = 0.5µm, Min Width = µm		

* Chrysotile was not counted for all grid openings evaluated; this field is utilized only for the Libby site.

ANALYSIS PARAMETERS	
**Effective filter area (mm2)**	385
**F-factor**	1.00E+00
**Grid opening area (mm2)**	0.0104
**# GOs counted High Magnification**	55
**# GOs counted Low Magnification**	0
**Sensitivity (1/cc)**	
**Total Asbestos**	0.000295
**PCME**	0.000295
**Maximum Area Examined**	
**High Magnification**	5.7E-01
**Low Magnification**	0.0E+00

Number of Structures with Fatal Data Entry Errors 0 *(Structures with fatal errors are excluded from calculations below)*

Desired Confidence Interval (%):

Mineral Class	Number of Structures (a)	Loading on Filter (b) (s/mm²)	Air Conc (c) (s/cc)	% Poisson Confidence Interval for this Sample
**Total TEM Structures**				
Total Asbestos	24	4.2E+01	7.1E-03	7.0E-03 - 7.3E-03
Total Chrysotile (CH)	24	4.2E+01	7.1E-03	7.0E-03 - 7.3E-03
Total Amphibole	0	0.0E+00	0.0E+00	0.0E+00 - 2.0E-04
actinolite (AC)	0	0.0E+00	0.0E+00	0.0E+00 - 2.0E-04
amosite (AM)	0	0.0E+00	0.0E+00	0.0E+00 - 2.0E-04
anthophyllite (AN)	0	0.0E+00	0.0E+00	0.0E+00 - 2.0E-04
crocidolite (CR)	0	0.0E+00	0.0E+00	0.0E+00 - 2.0E-04
tremolite (TR)	0	0.0E+00	0.0E+00	0.0E+00 - 2.0E-04
winchite/richterite/tremolite /actinolite (WRTA)	0	0.0E+00	0.0E+00	0.0E+00 - 2.0E-04
other amphibole (OA)	0	0.0E+00	0.0E+00	0.0E+00 - 2.0E-04
Solid Soln: Amosite	0	0.0E+00	0.0E+00	0.0E+00 - 2.0E-04
Solid Soln: Trem-Act	0	0.0E+00	0.0E+00	0.0E+00 - 2.0E-04
other mineral class (OM)	0	0.0E+00	0.0E+00	0.0E+00 - 2.0E-04
**PCM Equivalent Structures (PCME)**				
Total Asbestos	3	5.2E+00	8.8E-04	7.9E-04 - 1.1E-03
Total Chrysotile (CH)	3	5.2E+00	8.8E-04	7.9E-04 - 1.1E-03
Total Amphibole	0	0.0E+00	0.0E+00	0.0E+00 - 2.0E-04
actinolite (AC)	0	0.0E+00	0.0E+00	0.0E+00 - 2.0E-04
amosite (AM)	0	0.0E+00	0.0E+00	0.0E+00 - 2.0E-04
anthophyllite (AN)	0	0.0E+00	0.0E+00	0.0E+00 - 2.0E-04
crocidolite (CR)	0	0.0E+00	0.0E+00	0.0E+00 - 2.0E-04
tremolite (TR)	0	0.0E+00	0.0E+00	0.0E+00 - 2.0E-04
winchite/richterite/tremolite /actinolite (WRTA)	0	0.0E+00	0.0E+00	0.0E+00 - 2.0E-04
other amphibole (OA)	0	0.0E+00	0.0E+00	0.0E+00 - 2.0E-04
Solid Soln: Amosite	0	0.0E+00	0.0E+00	0.0E+00 - 2.0E-04
Solid Soln: Trem-Act	0	0.0E+00	0.0E+00	0.0E+00 - 2.0E-04
other mineral class (OM)	0	0.0E+00	0.0E+00	0.0E+00 - 2.0E-04

Binning Rule Description:

Apply to fibers (F) only:
L ≥ 0.5µm, AR ≥ 3

No restrictions for other structure types.

ChiSq test for even filter loading for Total TE (see Annex F2 in ISO 10312)

Filter loading appears uneven. Results may be uncertain.

Binning Rule Description:

Apply to all structures where Total column > 0

L > 5µm, W ≥ 0.25µm and W ≤ 3µm, AR ≥ 3

ChiSq test for even filter loading for PCMe (see Annex F2 in ISO 10312)

Filter loading is OK

[redacted]

(a) Based on countable structures only.

(b) Loading on Filter (s/mm²) = N structures / (GOs Counted * GO Area). Results for indirect samples are based on the secondary filter.

(c) Air Concentration (s/cc) = (N structures * EFA) / (GOs Counted * GO Area * F-factor * Air Volume * 1000).

Dust Loading (s/cm2) = (N structures * EFA) / (GOs Counted * GO Area * F-factor * Dust Collection Area).

version 13-DRAFT

Sumas Mtn Asbestos Soil [SFP-078A]

National Asbestos Data Entry Spreadsheet (NADES) for Air & Dust Analysis by Superfund TEM

ANALYTICAL REPORT

FILE NAME: SFP-078A_LabCor Inc._14394154_02-23-15_141014_TEM_D_RS.xls

SAMPLE/ANALYSIS INFORMATION			
**Field Sample Number**	14394154	**Lab Sample Number**	141014-S9
**Media**	Air	**Preparation**	Direct
**Sample Type**	Field Sample	**Sample Status**	Analyzed
**Air Volume (L)**	2172	**Analysis Date**	2/23/2015
**QA Sample Type**	RS	**Method SOP**	0
**Stopping Rule(s):**	Max Area = n/a, Structures = 100, Sensitivity = 3.00E-04		
**Recording Rule(s):**	Min Aspect Ratio = , Min Length = 0.5µm, Min Width = µm		

* Chrysotile was not counted for all grid openings evaluated; this field is utilized only for the Libby site.

ANALYSIS PARAMETERS	
**Effective filter area (mm2)**	385
**F-factor**	1.00E+00
**Grid opening area (mm2)**	0.0104
**# GOs counted High Magnification**	57
**# GOs counted Low Magnification**	0
**Sensitivity (1/cc)**	
**Total Asbestos**	0.0002984
**PCME**	0.0002984
**Maximum Area Examined**	
**High Magnification**	5.9E-01
**Low Magnification**	0.0E+00

Number of Structures with Fatal Data Entry Errors 0 *(Structures with fatal errors are excluded from calculations below)*

Desired Confidence Interval (%):

Mineral Class	Number of Structures (a)	Loading on Filter (b) (s/mm²)	Air Conc (c) (s/cc)	% Poisson Confidence Interval for this Sample
**Total TEM Structures**				
Total Asbestos	63	1.1E+02	1.9E-02	1.9E-02 - 1.9E-02
Total Chrysotile (CH)	62	1.0E+02	1.9E-02	1.8E-02 - 1.9E-02
Total Amphibole	1	1.7E+00	3.0E-04	2.1E-04 - 5.0E-04
actinolite (AC)	0	0.0E+00	0.0E+00	0.0E+00 - 2.1E-04
amosite (AM)	0	0.0E+00	0.0E+00	0.0E+00 - 2.1E-04
anthophyllite (AN)	0	0.0E+00	0.0E+00	0.0E+00 - 2.1E-04
crocidolite (CR)	0	0.0E+00	0.0E+00	0.0E+00 - 2.1E-04
tremolite (TR)	0	0.0E+00	0.0E+00	0.0E+00 - 2.1E-04
winchite/richterite/tremolite /actinolite (WRTA)	0	0.0E+00	0.0E+00	0.0E+00 - 2.1E-04
other amphibole (OA)	1	1.7E+00	3.0E-04	2.1E-04 - 5.0E-04
Solid Soln: Amosite	0	0.0E+00	0.0E+00	0.0E+00 - 2.1E-04
Solid Soln: Trem-Act	0	0.0E+00	0.0E+00	0.0E+00 - 2.1E-04
other mineral class (OM)	0	0.0E+00	0.0E+00	0.0E+00 - 2.1E-04
**PCM Equivalent Structures (PCME)**				
Total Asbestos	1	1.7E+00	3.0E-04	2.1E-04 - 5.0E-04
Total Chrysotile (CH)	1	1.7E+00	3.0E-04	2.1E-04 - 5.0E-04
Total Amphibole	0	0.0E+00	0.0E+00	0.0E+00 - 2.1E-04
actinolite (AC)	0	0.0E+00	0.0E+00	0.0E+00 - 2.1E-04
amosite (AM)	0	0.0E+00	0.0E+00	0.0E+00 - 2.1E-04
anthophyllite (AN)	0	0.0E+00	0.0E+00	0.0E+00 - 2.1E-04
crocidolite (CR)	0	0.0E+00	0.0E+00	0.0E+00 - 2.1E-04
tremolite (TR)	0	0.0E+00	0.0E+00	0.0E+00 - 2.1E-04
winchite/richterite/tremolite /actinolite (WRTA)	0	0.0E+00	0.0E+00	0.0E+00 - 2.1E-04
other amphibole (OA)	0	0.0E+00	0.0E+00	0.0E+00 - 2.1E-04
Solid Soln: Amosite	0	0.0E+00	0.0E+00	0.0E+00 - 2.1E-04
Solid Soln: Trem-Act	0	0.0E+00	0.0E+00	0.0E+00 - 2.1E-04
other mineral class (OM)	0	0.0E+00	0.0E+00	0.0E+00 - 2.1E-04

Binning Rule Description:

Apply to fibers (F) only:
L ≥ 0.5µm, AR ≥ 3

No restrictions for other structure types.

ChiSq test for even filter loading for Total TE (see Annex F2 in ISO 10312)

Filter loading appears uneven. Results may be uncertain.

Binning Rule Description:

Apply to all structures where Total column > 0

L > 5µm, W ≥ 0.25µm and W ≤ 3µm, AR ≥ 3

ChiSq test for even filter loading for PCMe (see Annex F2 in ISO 10312)

Filter loading is OK

[redacted]

(a) Based on countable structures only

(b) Loading on Filter (s/mm²) = N structures / (GOs Counted * GO Area). Results for indirect samples are based on the secondary filter.

(c) Air Concentration (s/cc) = (N structures * EFA) / (GOs Counted * GO Area * F-factor * Air Volume * 1000)
Dust Loading (s/cm2) = (N structures * EFA) / (GOs Counted * GO Area * F-factor * Dust Collection Area)

version 13-DRAFT

Sumas Mtn Asbestos Soil [SFP-078A]

National Asbestos Data Entry Spreadsheet (NADES) for Air & Dust Analysis by Superfund TEM

ANALYTICAL REPORT

FILE NAME: SFP-078A_LabCor Inc._14394181_02-24-15_141014_TEM_D_RD.xls

SAMPLE/ANALYSIS INFORMATION			
**Field Sample Number**	14394181	**Lab Sample Number**	141014-S28
**Media**	Air	**Preparation**	Direct
**Sample Type**	Field Sample	**Sample Status**	Analyzed
**Air Volume (L)**	145.5	**Analysis Date**	2/24/2015
**QA Sample Type**	RD	**Method SOP**	0
**Stopping Rule(s):**	Max Area = n/a, Structures = 100, Sensitivity = 3.00E-03		
**Recording Rule(s):**	Min Aspect Ratio = , Min Length = 0.5µm, Min Width = µm		

* Chrysotile was not counted for all grid openings evaluated; this field is utilized only for the Libby site.

ANALYSIS PARAMETERS	
**Effective filter area (mm2)**	385
**F-factor**	1.00E+00
**Grid opening area (mm2)**	0.0104
**# GOs counted High Magnification**	85
**# GOs counted Low Magnification**	0
**Sensitivity (1/cc)**	
**Total Asbestos**	0.0029875
**PCME**	0.0029875
**Maximum Area Examined**	
**High Magnification**	8.9E-01
**Low Magnification**	0.0E+00

Number of Structures with Fatal Data Entry Errors 0 *(Structures with fatal errors are excluded from calculations below)*

Desired Confidence Interval (%):

Mineral Class	Number of Structures (a)	Loading on Filter (b) (s/mm^2)	Air Conc (c) (s/cc)	% Poisson Confidence Interval for this Sample
**Total TEM Structures**				
Total Asbestos	7	7.9E+00	2.1E-02	2.0E-02 - 2.3E-02
Total Chrysotile (CH)	7	7.9E+00	2.1E-02	2.0E-02 - 2.3E-02
Total Amphibole	0	0.0E+00	0.0E+00	0.0E+00 - 2.1E-03
actinolite (AC)	0	0.0E+00	0.0E+00	0.0E+00 - 2.1E-03
amosite (AM)	0	0.0E+00	0.0E+00	0.0E+00 - 2.1E-03
anthophyllite (AN)	0	0.0E+00	0.0E+00	0.0E+00 - 2.1E-03
crocidolite (CR)	0	0.0E+00	0.0E+00	0.0E+00 - 2.1E-03
tremolite (TR)	0	0.0E+00	0.0E+00	0.0E+00 - 2.1E-03
winchite/richterite/tremolite /actinolite (WRTA)	0	0.0E+00	0.0E+00	0.0E+00 - 2.1E-03
other amphibole (OA)	0	0.0E+00	0.0E+00	0.0E+00 - 2.1E-03
Solid Soln: Amosite	0	0.0E+00	0.0E+00	0.0E+00 - 2.1E-03
Solid Soln: Trem-Act	0	0.0E+00	0.0E+00	0.0E+00 - 2.1E-03
other mineral class (OM)	0	0.0E+00	0.0E+00	0.0E+00 - 2.1E-03
**PCM Equivalent Structures (PCME)**				
Total Asbestos	1	1.1E+00	3.0E-03	2.1E-03 - 5.0E-03
Total Chrysotile (CH)	1	1.1E+00	3.0E-03	2.1E-03 - 5.0E-03
Total Amphibole	0	0.0E+00	0.0E+00	0.0E+00 - 2.1E-03
actinolite (AC)	0	0.0E+00	0.0E+00	0.0E+00 - 2.1E-03
amosite (AM)	0	0.0E+00	0.0E+00	0.0E+00 - 2.1E-03
anthophyllite (AN)	0	0.0E+00	0.0E+00	0.0E+00 - 2.1E-03
crocidolite (CR)	0	0.0E+00	0.0E+00	0.0E+00 - 2.1E-03
tremolite (TR)	0	0.0E+00	0.0E+00	0.0E+00 - 2.1E-03
winchite/richterite/tremolite /actinolite (WRTA)	0	0.0E+00	0.0E+00	0.0E+00 - 2.1E-03
other amphibole (OA)	0	0.0E+00	0.0E+00	0.0E+00 - 2.1E-03
Solid Soln: Amosite	0	0.0E+00	0.0E+00	0.0E+00 - 2.1E-03
Solid Soln: Trem-Act	0	0.0E+00	0.0E+00	0.0E+00 - 2.1E-03
other mineral class (OM)	0	0.0E+00	0.0E+00	0.0E+00 - 2.1E-03

RC J DR

Binning Rule Description:

Apply to fibers (F) only:
L ≥ 0.5µm, AR ≥ 3

No restrictions for other structure types.

ChiSq test for even filter loading for Total TE (see Annex F2 in ISO 10312)

Filter loading appears uneven. Results may be uncertain.

Binning Rule Description:

Apply to all structures where Total column > 0

L > 5µm, W ≥ 0.25µm and W ≤ 3µm, AR ≥ 3

ChiSq test for even filter loading for PCMe (see Annex F2 in ISO 10312)

Filter loading is OK

[redacted]

(a) Based on countable structures only.

(b) Loading on Filter (s/mm^2) = N structures / (GOs Counted * GO Area). Results for indirect samples are based on the secondary filter

(c) Air Concentration (s/cc) = (N structures * EFA) / (GOs Counted * GO Area * F-factor * Air Volume * 1000).
Dust Loading (s/cm2) = (N structures * EFA) / (GOs Counted * GO Area * F-factor * Dust Collection Area)

version 13-DRAFT

Sumas Mtn Asbestos Soil [SFP-078A]

National Asbestos Data Entry Spreadsheet (NADES) for Air & Dust Analysis by Superfund TEM

ANALYTICAL REPORT

FILE NAME: SFP-078A_LabCor Inc._NA_03-29-15_Q141014d_TEM_D_LB.xls

SAMPLE/ANALYSIS INFORMATION			
**Field Sample Number**	NA	**Lab Sample Number**	Q141014d-B1
**Media**	N/A	**Preparation**	Direct
**Sample Type**	Lab QC	**Sample Status**	Analyzed
		**Analysis Date**	3/29/2015
**QA Sample Type**	LB	**Method SOP**	0
**Stopping Rule(s):**	Max Area = , Structures = , Sensitivity = 0.00E+00		
**Recording Rule(s):**	Min Aspect Ratio = , Min Length = µm, Min Width = µm		

* Chrysotile was not counted for all grid openings evaluated; this field is utilized only for the Libby site.

ANALYSIS PARAMETERS	
**Effective filter area (mm2)**	385
**F-factor**	1.00E+00
**Grid opening area (mm2)**	0.0104
**# GOs counted High Magnification**	10
**# GOs counted Low Magnification**	0
**Sensitivity (1/cc)**	
**Total Asbestos**	blank
**PCME**	blank
**Maximum Area Examined**	
**High Magnification**	1.0E-01
**Low Magnification**	0.0E+00

Number of Structures with Fatal Data Entry Errors 0 *(Structures with fatal errors are excluded from calculations below)*

Desired Confidence Interval (%):

Mineral Class	Number of Structures (a)	Loading on Filter (b) (s/mm^2)		% Poisson Confidence Interval for this Sample
**Total TEM Structures**				
Total Asbestos	0	0.0E+00	blank	blank - blank
Total Chrysotile (CH)	0	0.0E+00	blank	blank - blank
Total Amphibole	0	0.0E+00	blank	blank - blank
actinolite (AC)	0	0.0E+00	blank	blank - blank
amosite (AM)	0	0.0E+00	blank	blank - blank
anthophyllite (AN)	0	0.0E+00	blank	blank - blank
crocidolite (CR)	0	0.0E+00	blank	blank - blank
tremolite (TR)	0	0.0E+00	blank	blank - blank
winchite/richterite/tremolite /actinolite (WRTA)	0	0.0E+00	blank	blank - blank
other amphibole (OA)	0	0.0E+00	blank	blank - blank
Solid Soln: Amosite	0	0.0E+00	blank	blank - blank
Solid Soln: Trem-Act	0	0.0E+00	blank	blank - blank
other mineral class (OM)	0	0.0E+00	blank	blank - blank
**PCM Equivalent Structures (PCME)**				
Total Asbestos	0	0.0E+00	blank	blank - blank
Total Chrysotile (CH)	0	0.0E+00	blank	blank - blank
Total Amphibole	0	0.0E+00	blank	blank - blank
actinolite (AC)	0	0.0E+00	blank	blank - blank
amosite (AM)	0	0.0E+00	blank	blank - blank
anthophyllite (AN)	0	0.0E+00	blank	blank - blank
crocidolite (CR)	0	0.0E+00	blank	blank - blank
tremolite (TR)	0	0.0E+00	blank	blank - blank
winchite/richterite/tremolite /actinolite (WRTA)	0	0.0E+00	blank	blank - blank
other amphibole (OA)	0	0.0E+00	blank	blank - blank
Solid Soln: Amosite	0	0.0E+00	blank	blank - blank
Solid Soln: Trem-Act	0	0.0E+00	blank	blank - blank
other mineral class (OM)	0	0.0E+00	blank	blank - blank

Binning Rule Description:

Apply to fibers (F) only:
L ≥ 0.5µm, AR ≥ 3

No restrictions for other structure types.

ChiSq test for even filter loading for Total TE
(see Annex F2 in ISO 10312)

Filter loading is OK

Binning Rule Description:

Apply to all structures where Total column > 0

L > 5µm, W ≥ 0.25µm and W ≤ 3µm, AR ≥ 3

ChiSq test for even filter loading for PCMe
(see Annex F2 in ISO 10312)

[redacted]

(a) Based on countable structures only

(b) Loading on Filter (s/mm^2) = N structures / (GOs Counted * GO Area). Results for indirect samples are based on the secondary filter

(c) Air Concentration (s/cc) = (N structures * EFA) / (GOs Counted * GO Area * F-factor * Air Volume * 1000)
Dust Loading (s/cm2) = (N structures * EFA) / (GOs Counted * GO Area * F-factor * Dust Collection Area)

version 13-DRAFT

Sumas Mtn Asbestos Soil [SFP-078A]

National Asbestos Data Entry Spreadsheet (NADES) for Air & Dust Analysis by Superfund TEM

ANALYTICAL REPORT

FILE NAME: SFP-078A_LabCor Inc._NA_03-29-15_Q141014e_TEM_D_LB.xls

SAMPLE/ANALYSIS INFORMATION			
**Field Sample Number**	NA	**Lab Sample Number**	Q141014e-B2
**Media**	N/A	**Preparation**	Direct
**Sample Type**	Lab QC	**Sample Status**	Analyzed
		**Analysis Date**	3/29/2015
**QA Sample Type**	LB	**Method SOP**	0
**Stopping Rule(s):**	Max Area = , Structures = , Sensitivity = 0.00E+00		
**Recording Rule(s):**	Min Aspect Ratio = , Min Length = µm, Min Width = µm		

* Chrysotile was not counted for all grid openings evaluated; this field is utilized only for the Libby site.

ANALYSIS PARAMETERS	
**Effective filter area (mm2)**	385
**F-factor**	1.00E+00
**Grid opening area (mm2)**	0.0104
**# GOs counted High Magnification**	10
**# GOs counted Low Magnification**	0
**Sensitivity (1/cc)**	
**Total Asbestos**	blank
**PCME**	blank
**Maximum Area Examined**	
**High Magnification**	1.0E-01
**Low Magnification**	0.0E+00

Number of Structures with Fatal Data Entry Errors 0 *(Structures with fatal errors are excluded from calculations below)*

Desired Confidence Interval (%):

Mineral Class	Number of Structures (a)	Loading on Filter (b) (s/mm^2)		% Poisson Confidence Interval for this Sample
**Total TEM Structures**				
Total Asbestos	0	0.0E+00	blank	blank - blank
Total Chrysotile (CH)	0	0.0E+00	blank	blank - blank
Total Amphibole	0	0.0E+00	blank	blank - blank
actinolite (AC)	0	0.0E+00	blank	blank - blank
amosite (AM)	0	0.0E+00	blank	blank - blank
anthophyllite (AN)	0	0.0E+00	blank	blank - blank
crocidolite (CR)	0	0.0E+00	blank	blank - blank
tremolite (TR)	0	0.0E+00	blank	blank - blank
winchite/richterite/tremolite /actinolite (WRTA)	0	0.0E+00	blank	blank - blank
other amphibole (OA)	0	0.0E+00	blank	blank - blank
Solid Soln: Amosite	0	0.0E+00	blank	blank - blank
Solid Soln: Trem-Act	0	0.0E+00	blank	blank - blank
other mineral class (OM)	0	0.0E+00	blank	blank - blank
**PCM Equivalent Structures (PCME)**				
Total Asbestos	0	0.0E+00	blank	blank - blank
Total Chrysotile (CH)	0	0.0E+00	blank	blank - blank
Total Amphibole	0	0.0E+00	blank	blank - blank
actinolite (AC)	0	0.0E+00	blank	blank - blank
amosite (AM)	0	0.0E+00	blank	blank - blank
anthophyllite (AN)	0	0.0E+00	blank	blank - blank
crocidolite (CR)	0	0.0E+00	blank	blank - blank
tremolite (TR)	0	0.0E+00	blank	blank - blank
winchite/richterite/tremolite /actinolite (WRTA)	0	0.0E+00	blank	blank - blank
other amphibole (OA)	0	0.0E+00	blank	blank - blank
Solid Soln: Amosite	0	0.0E+00	blank	blank - blank
Solid Soln: Trem-Act	0	0.0E+00	blank	blank - blank
other mineral class (OM)	0	0.0E+00	blank	blank - blank

Binning Rule Description:

Apply to fibers (F) only:
L ≥ 0.5µm, AR ≥ 3

No restrictions for other structure types.

ChiSq test for even filter loading for Total TE
(see Annex F2 in ISO 10312)

Filter loading is OK

Binning Rule Description:

Apply to all structures where Total column > 0

L > 5µm, W ≥ 0.25µm and W ≤ 3µm, AR ≥ 3

ChiSq test for even filter loading for PCMe
(see Annex F2 in ISO 10312)

[redacted]

(a) Based on countable structures only.
(b) Loading on Filter (s/mm^2) = N structures / (GOs Counted * GO Area). Results for indirect samples are based on the secondary filter.
(c) Air Concentration (s/cc) = (N structures * EFA) / (GOs Counted * GO Area * F-factor * Air Volume * 1000)
Dust Loading (s/cm2) = (N structures * EFA) / (GOs Counted * GO Area * F-factor * Dust Collection Area).

version 13-DRAFT

Sumas Mtn Asbestos Soil [SFP-078A]

National Asbestos Data Entry Spreadsheet (NADES) for Air & Dust Analysis by Superfund TEM

ANALYTICAL REPORT

FILE NAME: SFP-078A_LabCor Inc._NA_03-29-15_Q141014f_TEM_D_LB.xls

SAMPLE/ANALYSIS INFORMATION			
**Field Sample Number**	NA	**Lab Sample Number**	Q141014f-B3
**Media**	N/A	**Preparation**	Direct
**Sample Type**	Lab QC	**Sample Status**	Analyzed
		**Analysis Date**	3/29/2015
**QA Sample Type**	LB	**Method SOP**	0
**Stopping Rule(s):**	Max Area = , Structures = , Sensitivity = 0.00E+00		
**Recording Rule(s):**	Min Aspect Ratio = , Min Length = µm, Min Width = µm		

* Chrysotile was not counted for all grid openings evaluated, this field is utilized only for the Libby site.

ANALYSIS PARAMETERS	
**Effective filter area (mm2)**	385
**F-factor**	1.00E+00
**Grid opening area (mm2)**	0.0104
**# GOs counted High Magnification**	10
**# GOs counted Low Magnification**	0
**Sensitivity (1/cc)**	
**Total Asbestos**	blank
**PCME**	blank
**Maximum Area Examined**	
**High Magnification**	1.0E-01
**Low Magnification**	0.0E+00

Number of Structures with Fatal Data Entry Errors 0 *(Structures with fatal errors are excluded from calculations below)*

Desired Confidence Interval (%):

Mineral Class	Number of Structures (a)	Loading on Filter (b) (s/mm^2)		% Poisson Confidence Interval for this Sample
**Total TEM Structures**				
Total Asbestos	0	0.0E+00	blank	blank - blank
Total Chrysotile (CH)	0	0.0E+00	blank	blank - blank
Total Amphibole	0	0.0E+00	blank	blank - blank
actinolite (AC)	0	0.0E+00	blank	blank - blank
amosite (AM)	0	0.0E+00	blank	blank - blank
anthophyllite (AN)	0	0.0E+00	blank	blank - blank
crocidolite (CR)	0	0.0E+00	blank	blank - blank
tremolite (TR)	0	0.0E+00	blank	blank - blank
winchite/richterite/tremolite /actinolite (WRTA)	0	0.0E+00	blank	blank - blank
other amphibole (OA)	0	0.0E+00	blank	blank - blank
Solid Soln: Amosite	0	0.0E+00	blank	blank - blank
Solid Soln: Trem-Act	0	0.0E+00	blank	blank - blank
other mineral class (OM)	0	0.0E+00	blank	blank - blank
**PCM Equivalent Structures (PCME)**				
Total Asbestos	0	0.0E+00	blank	blank - blank
Total Chrysotile (CH)	0	0.0E+00	blank	blank - blank
Total Amphibole	0	0.0E+00	blank	blank - blank
actinolite (AC)	0	0.0E+00	blank	blank - blank
amosite (AM)	0	0.0E+00	blank	blank - blank
anthophyllite (AN)	0	0.0E+00	blank	blank - blank
crocidolite (CR)	0	0.0E+00	blank	blank - blank
tremolite (TR)	0	0.0E+00	blank	blank - blank
winchite/richterite/tremolite /actinolite (WRTA)	0	0.0E+00	blank	blank - blank
other amphibole (OA)	0	0.0E+00	blank	blank - blank
Solid Soln: Amosite	0	0.0E+00	blank	blank - blank
Solid Soln: Trem-Act	0	0.0E+00	blank	blank - blank
other mineral class (OM)	0	0.0E+00	blank	blank - blank

Binning Rule Description:

Apply to fibers (F) only:
L ≥ 0.5µm, AR ≥ 3

No restrictions for other structure types.

ChiSq test for even filter loading for Total TE
(see Annex F2 in ISO 10312)

Filter loading is OK

Binning Rule Description:

Apply to all structures where Total column > 0

L > 5µm, W ≥ 0.25µm and W ≤ 3µm, AR ≥ 3

ChiSq test for even filter loading for PCMe
(see Annex F2 in ISO 10312)

[redacted]

(a) Based on countable structures only.

(b) Loading on Filter (s/mm^2) = N structures / (GOs Counted * GO Area). Results for indirect samples are based on the secondary filter.

(c) Air Concentration (s/cc) = (N structures * EFA) / (GOs Counted * GO Area * F-factor * Air Volume * 1000).
Dust Loading (s/cm2) = (N structures * EFA) / (GOs Counted * GO Area * F-factor * Dust Collection Area).
