# Supplementary material for: Comparison of soil sampling and analytical methods for asbestos at the Sumas Mountain Asbestos Site—Working towards a toolbox for better assessment
Source: PLoS One. 2017 Jul 31;12(7):e0180210. doi: 10.1371/journal.pone.0180210 (PMC5536314; doi:10.1371/journal.pone.0180210)
Supplement: S6 Text — (PDF) [file pone.0180210.s007.pdf]

CB&I
2700 Chandler Avenue, Building C
Las Vegas, NV 89120
Tel: +1 702 795 0515
Fax: +1 702 795 8210
www.CBI.com

July 10, 2015

Julie Wroble
EPA Site Manager Region 10
EPA Region 10
1200 6th Ave, Suite 900
Seattle, WA 98101

Document ID #: 3015-07102015-6

Dear Ms. Wroble:

EPA CONTRACT NUMBER EP-W-10-033
TASK ORDER NUMBER 3015
ASBESTOS QA SUPPORT

Enclosed please find the Release of Validated Data Report for the validation of Polarized Light Microscopy-Visual Estimation (PLM-VE) and Transmission Electron Microscopy (TEM) soil sample data, Laboratory Job Number 142958. The twenty-four (24) soil samples associated with these data were analyzed by Lab/Cor Portland, Inc., Portland, Oregon for the Sumas Mountain Asbestos Soil Project. This report and accompanying appendices are deliverables under Task 10 of the subject Task Order.

If you have any questions, please feel free to contact me.

Sincerely,

Lyndsay Gensler
Task Leader, QATS Program
CB&I Federal Services LLC
Phone: 702.895.8730
E-Mail Address: lyndsay.gensler@cbifederalservices.com

cc: Shari Myer, EPA-ASB, QATS Task Order Project Officer
Administrative Contracting Officer (letter only)

RELEASE OF VALIDATED DATA

DATE:	July 10, 2015
SUBJECT:	Review of Data for Laboratory Job Number: 142958
LABORATORY:	Lab/Cor Portland, Inc., Portland, Oregon
FROM:	Quality Assurance Technical Support (QATS) Program, Las Vegas, NV<br>CB&I Federal Services LLC
TO:	Julie Wroble, Environmental Protection Agency

QATS reviewed the data for the following case:

Applicable SAP:	NA
Chain-of-Custody Number:	10-100614-121545-0001
Method:	Transmission Electron Microscopy (TEM) by EPA 600-R-93-116, (with preparation by ASTM D7521), and Polarized Light Microscopy (PLM) VE by CARB 435.
Applicable Laboratory Modification(s):	NA
Number and Type of Samples:	24 Soil Samples
EPA Sample Numbers:	14394100, 14394101, 14394102, 14394103, 14394104, 14394105, 14394106, 14394107, 14394108, 14394109, 14394110, 14394111, 14394112, 14394113, 14394114, 14394115, 14394116, 14394117, 14394118, 14394119, 14394120, 14394121, 14394122, 14394123.

Note that samples 14294100 through 14394122 were analyzed by TEM as indicated on the ASTM D7521 results sheet.

VALIDATION SUMMARY

Twenty-four (24) soil samples from Laboratory Job Number 142958, were collected on 09/30/2014 and 10/01/2014 and shipped to Lab/Cor Portland, Inc. in Portland, OR for PLM-VE analysis by CARB 435 (with preparation by ASTM D7521), and TEM by EPA 600-R-93-116. The samples were received at the laboratory intact on 12/15/2014, and were analyzed between 01/25/2015 and 01/30/2015 for PLM and between 02/03/2015 and 02/19/2015 for TEM.

Listed below are the Data Qualification Summary Table, EDD/Bench Sheet Discrepancy Table, Data Qualifier Table, and Reason Code Table.

DATA QUALIFICATION SUMMARY TABLE

Criteria Exceeded	EPA Sample ID	Validation Qualifier	Reason Code
PLM Daily Calibration was not documented for 01/26/2015.	14394108<br>14394109<br>14394110<br>14394111<br>14394112<br>14394113	UJ	MC
TEM k-factors not performed at required frequency.	14394109<br>14394109 (RD QC Sample)	J	IC
TEM Daily Calibration was not documented for 02/11/2015.	14394110 (RD QC Sample)	UJ	MC
	14394109 (RD QC Sample)	J	

EDD/BENCH SHEET DISCREPANCY TABLE

EPA Sample ID	C# *	Method/Matrix	Lab. Job No.	Analysis Date	Discrepancy
14394121	NA	ASTM D7521	142958	01/28/2015	The (W_M) value reported on the ASTM D7521 Total Asbestos % sheet for sample 14394121 (132.2 g) does not match the value recorded on the ASTM D7521 Prep Sheet (133.2 g).

'*' The EDD correction number in column 2. (i.e., C0, C1, C2, etc..)

DATA QUALIFIER TABLE

Qualifier	Definition
**J**	The result is estimated. The associated numerical value is an approximation.
**UJ**	The non-detect result may be inaccurate or imprecise due to the quality of the data generated because certain QC criteria were not met.
**R**	The sample results are rejected due to serious deficiencies.
**X**	Validator defined.

TEM REASON CODE TABLE

Reason Code	Definition
**MC**	Structure/fiber counts and recorded structure dimensions may be inaccurate due to improper or infrequent scope alignment and/or magnification calibrations.
**IC**	Identification by elemental composition or diffraction pattern may be inaccurate due to improper or infrequent EDXA or camera constant calibration.
**PA**	Structure/fiber counts and reported concentrations may be inaccurate due to improper or infrequent calibration of the plasma asher.
**SC**	The reported concentration may be inaccurate due to the condition of samples upon receipt at the laboratory.

Reason Code	Definition
**DL**	The area analyzed, structures counted, or AS do not meet the requirements specified in the applicable SAP Analytical Summary.
**ID**	The asbestos identification and concentrations may be inaccurate because the recorded structure types are not consistent with those described in the applicable TEM Method and/or laboratory modification(s).

PLM REASON CODE TABLE

Reason Code	Definition
**MC**	Reported concentrations or analyte identification may be inaccurate due to improper or infrequent scope alignment.
**IC**	Identification may be inaccurate due to improper or infrequent Refractive Index (RI) liquid calibrations.
**B**	The reported concentration may be inaccurate due to the presence of analyte structures/fibers in the associate contamination check or a contamination check was not performed daily.
**SC**	The reported concentration may be inaccurate due to the condition of samples upon receipt at the laboratory and/or improper storage prior to sample preparation and/or analysis.
**ID**	The asbestos identification and concentrations may be inaccurate because the recorded optical properties are not consistent with those described in the project-specific PLM SOPs.

VALIDATION PROCESS

The samples for Laboratory Job Number 142958 were collected from the subject site on 09/30/2014 and 10/01/2014. All samples were prepared and analyzed in accordance with CARB 435, ASTM D7521, and EPA 600-R-93-116. CB&I's Quality Assurance Technical Support (QATS) Program performed validation and a transcription check in accordance with method-specific data validation SOPs. QATS preparation of this report was performed under Technical Direction 03, Task 10, of Task Order 3015.

The sample results on bench sheets and other supporting documents provided in the hardcopy deliverables were compared to the entries in the associated laboratory method-specific EDDs (where applicable) to ensure that the reported results are complete, compliant with the specified methodology, and accurate. Additional support information provided in this data validation report include the QATS Data Review Checklist used to document the data validation process (see Appendix A); and the sample results as reported by the laboratory, with qualifiers as applicable (see Appendix B).

TEM VALIDATION SUMMARY

1. **DATA PACKAGE INVENTORY AND SAMPLE RECEIPT:** The data package included a narrative, Chain-of-Custody (COC) record, EDD files, raw data (bench sheets), and QC samples. The samples were properly packaged, sealed, undamaged, and labeled upon receipt at the laboratory. The COC record was reviewed and found to be acceptable.

2. **SAMPLE PREPARATION:** The appropriate preparation documents were provided.

3. **EQUIPMENT CALIBRATION AND PERFORMANCE CHECKS (i.e., daily microscope alignment, screen magnification, EDS calibration, and sensitivity checks):** The equipment alignment and calibration documentation provided shows that instrument alignment and calibration were performed at the correct frequency, indicating that the instruments were in proper working order during the time of sample analyses. Two exceptions include: The daily calibration was not recorded for 02/11/2015 on instrument H-7000, affecting QC (RD) samples 14394109 and 14394110. These samples are qualified "J" and "UJ", respectively. The TEM the k-factors provided by the laboratory were performed on 01/24/2014 for instrument (scope) H-7000, more than six months prior to the analyses of the samples in this SDG. The laboratory's QAPP states for k-factors "Calibration is performed on a biyearly basis, the first week of January and the first week of July." QATS requested the January 2015 k-factors from the laboratory; however, k factors calculated on 03/30/2015 were received. As a result of the described k-factor deficiency, one sample and the Recount Same (RS) of that sample, each with an amphibole result, are qualified "J".

4. **ANALYTICAL SENSITIVITY:** A sufficient number of grid openings have been analyzed to achieve the required analytical sensitivity and/or the appropriate stopping rule was invoked.

5. **STRUCTURE RECORDING AND ASBESTOS IDENTIFICATION:** The structure recording and asbestos identification were found to be acceptable.

6. **BLANK ANALYSIS:** No blanks were analyzed and reported with this sample set.

7. **ANALYTICAL VARIABILITY:** The laboratory performed two recount different (RD) analyses on EPA Sample Nos. 14394109 and 14394110 and two recount same (RS) analyses on EPA Sample Nos. 14394100 and 14394101. All QC samples passed the established QC criteria.

8. **OVERALL ASSESSMENT OF DATA:** With the exception of the k-factor and calibration discrepancies, the deliverable was found to be complete and accurate. Qualified samples are listed in the Data Qualification Summary Table.

REVIEWED BY: Shellee McGrath DATE: 07/09/2015

PLM VALIDATION SUMMARY

1. **DATA PACKAGE INVENTORY AND SAMPLE RECEIPT:** The data package included a narrative, Chain-of-Custody (COC) record, EDD files, raw data (bench sheets), and QC samples. The samples were properly packaged, sealed, undamaged, and labeled upon receipt at the laboratory. The COC record was reviewed and found to be acceptable.

2. **SAMPLE PREPARATION:** The appropriate preparation documents were provided.

3. **EQUIPMENT CALIBRATION AND PERFORMANCE CHECKS (i.e., daily microscope alignment, RI liquid calibration check):** The Scope ID was not documented in the NADES file; however, the analyst and dates were matched up to PLM #1. According to the NADES file the samples were analyzed on 01/25/2015, 01/26/2015, 01/27/2015, and 01/30/2015. The 142958R02_020150325 Final PLMVis.pdf file lists the analysis date for all 24 samples as 02/05/2015. For the calibration requirement, the analysis dates from the NADES file were used. The required daily microscope alignments and monthly calibration of the commonly used RI oils was performed and recorded for all dates with the exception of 01/26/2015; therefore, six samples, 14394108 through 14394113, were qualified "UJ" due to the lack of daily calibration documentation. The QC samples were analyzed on 03/16/2015, the calibration was provided for this date on both PLM instruments.

4. **MINERAL/FIBER IDENTIFICATION:** The fiber identification and quantification were found to be acceptable.

5. **CONTAMINATION CHECK:** The appropriate daily contamination checks were performed and recorded on the Equipment Maintenance Form for all dates except 01/26/2015 (see Comment #3 above).

6. **REFERENCE MATERIAL ANALYSIS (CALIBRATION STANDARDS):** The PLM Reference Material Comparison spreadsheet, PLM Accuracy QC and RTI Reference Material Comparison.xls, provided Reference Material results for analyst R. Brown. The analyst for the samples in this SDG is S. Golden; therefore, the PLM Reference Material analysis could not be evaluated.

7. **ANALYTICAL VARIABILITY:** The laboratory performed three Laboratory Duplicates (by the same analyst) on EPA Sample Nos. 14394106, 14394117, and 14394123 and two Laboratory Duplicates (by a different analyst) on EPA Sample Nos. 14394106 and 14394109. All QC samples passed the established QC criteria.

8. **LABORATORY MODIFICATIONS:** NA

9. **GRAVIMETRIC ANALYSIS:** Gravimetric analysis was performed and recorded on the ASTM D7521 Prep Sheets.

9. **OVERALL ASSESSMENT OF DATA:** With the exception of the daily calibration documentation for 01/26/2015, and the W_M value discrepancy in the EDD/Bench Sheet Discrepancy Table, the deliverable was found to be complete and accurate. Qualified samples are listed in the Data Qualification Summary Table.

REVIEWED BY: Shellee McGrath DATE: 06/08/2015

Appendix A

Data Review Checklist

Data Review Checklist for the Verification and Validation of Transmission Electron Microscopy (TEM) Data Deliverables

**Project Name: Sumas Mtn Asbestos Soil Project**	**Case or Sample Set ID: 142958**
**Number of Samples/Matrix: 23 Soil Samples**	**COC Number: 10-100614-121545-0001**
**TEM Analytical Method: EPA 600-R-93-116**	**Level of Validation (Circle one): 1 2 3 Other**

**1.0 Data Package Inventory**	**Yes**	**No**	**Comments**
1.1 Were the project-specific requirements (i.e. acceptance criteria & analytical sensitivities) provided by the client prior to the initiation of validation activities?	☐	☒	A request was made to the laboratory on 06/04/2015 for the laboratory's QAPP and/or SOPs documenting the frequency of the different TEM calibrations. The documents were received on 06/18/2015. k-factors were also requested from the laboratory.
1.2 Did the received hard copy deliverables contain all the necessary components:			
1.2.1 Case Narrative (Level 1, 2 & 3)?	☒	☐	
1.2.2 Chain-of-Custody (Level 1, 2 & 3)?	☒	☐	
1.2.3 Form I or equivalent (Level 1, 2 & 3)?	☒	☐	
1.2.4 Raw Data - Count Sheets (Level 1, 2 & 3)?	☒	☐	
1.2.5 QC Sample Data (Level 2 & 3):	☒	☐	
1.2.5.1 Blank(s)?	☐	☒	The laboratory performed two recount different (RD) analyses on EPA Sample Nos. 14394109 and 14394110 and two recount same (RS) analyses on EPA Sample Nos. 14394100 and 14394101.
1.2.5.2 Replicate(s)?	☒	☐	
1.2.5.3 Duplicate(s)?	☒	☐	
1.2.5.4 Verified Analysis?	☐	☒	
1.2.6 Calibration Data (Level 3)?	☒	☐	
1.2.7 Communication Records (Level 1, 2 & 3)?	☐	☒	
1.2.8 Miscellaneous?	☐	☐	NA
1.3 Were the necessary components received to perform the requested level of validation?	☒	☐	
**2.0 Chain-of-Custody Information Verification (Level 1, 2 & 3)**			
2.1 Were the following information recorded in the hard copy electronic deliverables (if applicable) consistent with the information recorded on the COC:			
2.1.1 COC Number?	☒	☐	
2.1.2 Case or Sample Set Number?	☒	☐	
2.1.3 EPA Sample ID?	☒	☐	
2.1.4 Date/Time Collected?	☒	☐	
2.1.5 Sample Volume?	☒	☐	
2.1.6 Sample Matrix?	☒	☐	
2.1.7 Analyses (Method)?	☒	☐	
2.1.8 Date/Time Received?	☒	☐	
2.1.9 Other (describe)?	☐	☐	
2.2 Were the COC records signed and dated upon receipt?	☒	☐	

Additional Comments:

Data Review Checklist for the Verification and Validation of Transmission Electron Microscopy (TEM) Data Deliverables

3.0 Sample Result Verification & Validation (Level 1, 2 & 3)	Yes	No	Comments
3.1 Is the sample preparation method documented and final sample volume recorded?	☒	☐	
3.2 Is the correct number of grid openings used to achieve the specified analytical sensitivity?	☒	☐	
3.3 Verify that the following information from the laboratory's bench sheets have been transcribed correctly:			
3.3.1.1 Grid identification?	☒	☐	
3.3.1.2 Grid opening?	☒	☐	
3.3.1.3 Structure type?	☒	☐	
3.3.1.4 Number of primary and secondary structures?	☒	☐	
3.3.1.5 Length and width dimensions?	☒	☐	
3.3.1.6 Structure identification?	☒	☐	
3.3.1.7 Mineral type?	☒	☐	
3.4 Are overloaded samples correctly reported to the specified percent obscuration (i.e. 10%, 25%)?	☐	☐	NA
3.5 If overloading occurs, are samples prepared by an alternate method (i.e. indirect preparation)?	☐	☐	NA
3.6 Verify that the following information is documented correctly:			
3.6.1 Magnification?	☒	☐	
3.6.2 Field or QC sample type?	☒	☐	
3.6.3 Number of grids prepared?	☒	☐	
3.6.4 Filter area in (mm^2)?	☒	☐	
3.6.5 Analysis date?	☒	☐	
3.7 Verify the totals reported on the count sheets for the various types of structures. These may include:			
3.7.1 Total EPA Structures	☒	☐	
3.7.2 PCMe Structures	☒	☐	
3.7.3 AHERA Structures	☐	☐	NA
3.7.4 Berman Crump Structures	☐	☐	NA
3.8 Are the required spectra included for all hits reported (i.e. ED, EDXA, SAED)?	☒	☐	
3.9 Recalculate the reported concentration on at least 10% of the results reported.			
3.9.1 Are the recalculated concentrations consistent with those reported?	☒	☐	

Additional Comments:

Data Review Checklist for the Verification and Validation of Transmission Electron Microscopy (TEM) Data Deliverables

4.0 Quality Control Verification & Validation (Level 2 and 3)	Yes	No	Comments
4.1 Blanks			
4.1.1 Are laboratory blanks (direct, indirect) prepared and analyzed at the required frequency?	☐	☒	
4.1.2 Are laboratory blank results within the specified criteria?	☐	☐	
4.1.2.1 If "no" then qualify the associated results in accordance with the Blank Result table in SOP QATS-70-091.			
4.2 Replicate Analyses			
4.2.1 Are replicate (second analyst on the same grids but different grid openings) sample analyses performed at the required frequency?	☒	☐	
4.2.2 Are replicate sample results within the specified acceptance limits?	☒	☐	The laboratory performed two recount different (RD) analyses on EPA Sample Nos. 14394109 and 14394110 and two recount same (RS) analyses on EPA Sample Nos. 14394100 and 14394101.
4.2.2.1 If "no" then qualify the associated results in accordance with the Analytical Variability Results table in SOP QATS-70-091.			
4.3 Duplicate Analyses			
4.3.1 Are duplicates (analysis of a second sample preparation obtained from the final filter) prepared and analyzed at the required frequency?	☒	☐	
4.3.2 Are duplicate sample results within the specified acceptance limits?	☒	☐	
4.3.2.1 If "no" then qualify the associated results in accordance with the Analytical Variability Results table in SOP QATS-70-091.			
4.4 Verified Analyses			
4.4.1 Are verified analyses (second analysis on same grids and grid openings) at the required frequency?	☐	☒	
4.4.2 Are sample verification results within the specified acceptance limits?	☐	☐	
4.4.2.1 If "no" then qualify the associated results in accordance with the Analytical Variability Results table in SOP QATS-70-091.			

Additional Comments:

Data Review Checklist for the Verification and Validation of Transmission Electron Microscopy (TEM) Data Deliverables

5.0 Calibration & Microscope Alignment Validation (Level 3)	Yes	No	Comments
5.1 Is evidence of the calibration of TEM Screen Magnification provided for all sample analyses?	☒	☐	Daily calibration was not performed on 02/11/2015 affecting two QC samples. The samples are qualified "J" or "UJ".
5.1.1 Camera Constant Calibration?	☒	☐	
5.1.2 Calibration of the EDXA System?	☒	☐	
5.1.3 k-Factors?	☒	☐	
5.2 Are the calibration checks listed above performed at the required frequencies?	☐	☒	The k-factors provided by the laboratory were performed on 01/24/2014, more than one year prior to the analysis of the samples in this SDG. The laboratory's QAPP states for k-factors "Calibration is performed on a biyearly basis, the first week of January and the first week of July." As a result, one sample and one QC sample are qualified "J" due to the k-factor calibration.
5.3 Are the calibration checks within the specified criteria?	☒	☐	
5.4 Are all calibration checks traceable to the associated samples analyses?	☒	☐	
5.5 If required, are the following additional system checks provided:			
5.5.1 Beam Dose Check?	☒	☐	
5.5.2 Spot Size Check?	☒	☐	
5.5.3 Detector Resolution Check?	☒	☐	
5.5.4 Resolvable Na, Mg, and Si Peaks?	☒	☐	
5.5.5 If "no" then qualify the associated results in accordance with the Calibration Results table in SOP QATS-70-091.			
**6.0 Case Narrative Validation (Levels 2 & 3)**			
6.1 Does the data package narrative include descriptions of the following:			
6.1.1 Samples received (matrix/method)?	☒	☐	
6.1.2 Method/project requirement deviations?	☒	☐	
6.1.3 Example sample calculation?	☒	☐	
6.1.4 Laboratory blank contamination?	☐	☐	NA
6.1.5 Quality control analyses outside specified criteria?	☐	☐	NA
6.1.6 Any problems encountered and subsequent corrective action?	☐	☐	NA

Additional Comments:

Validator's Signature Shellee McGrath **Date** 07/09/2015

QA Review Lyndsay Gensler **Date** 07/09/2015

Data Review Checklist for the Verification and Validation of Polarized Light Microscopy (PLM) Data Deliverables

**Project Name: Sumas Mtn Asbestos Soil Project**	**Case or Sample Set ID: 142958**
**Number of Samples/Matrix: 24 Soil Samples**	**COC Number: 10-100614-121545-0001**
**PLM Analytical Method: CARB 435; ASTM D7521**	**Level of Validation (Circle one): 1 2 [3] Other**

**1.0 Data Package Inventory**	**Yes**	**No**	**Comments**
1.1 Were the project-specific requirements (i.e. acceptance criteria & analytical sensitivities) provided by the client prior to the initiation of validation activities?	☒	☐	
1.2 Did the received hard copy deliverables contain all the necessary components:			
1.2.1 Case Narrative (Level 1, 2 & 3)?	☒	☐	
1.2.2 Chain-of-Custody (Level 1, 2 & 3)?	☒	☐	
1.2.3 Form I or equivalent (Level 1, 2 & 3)?	☒	☐	
1.2.4 Raw Data - Count Sheets (Level 1, 2 & 3)?	☒	☐	The laboratory performed three Laboratory Duplicates (by the same analyst) on EPA Sample Nos. 14394106, 14394117, and 14394123 and two Laboratory Duplicates (by a different analyst) on EPA Sample Nos. 14394106 and 14394109.
1.2.5 QC Sample Data (Level 2 & 3):	☒	☐	
1.2.5.1 Blank(s)?	☐	☒	
1.2.5.2 Replicate(s)?	☒	☐	
1.2.5.3 Duplicate(s)?	☒	☐	
1.2.6 Calibration Data (Level 3)?	☒	☐	
1.2.7 Communication Records (Level 1, 2 & 3)?	☐	☒	
1.2.8 Miscellaneous?	☐	☐	NA
1.3 Were the necessary components received to perform the requested level of validation?	☒	☐	
**2.0 Chain-of-Custody Information Verification (Level 1, 2 & 3)**			
2.1 Were the following information recorded in the hard copy electronic deliverables (if applicable) consistent with the information recorded on the COC:			
2.1.1 COC Number?	☒	☐	
2.1.2 Case or Sample Set Number?	☒	☐	
2.1.3 EPA Sample ID?	☒	☐	
2.1.4 Date/Time Collected?	☒	☐	
2.1.5 Sample Matrix?	☒	☐	
2.1.6 Analyses (Method)?	☒	☐	
2.1.7 Date/Time Received?	☒	☐	
2.1.8 Other (describe)?	☐	☐	
2.2 Were the COC records signed and dated upon receipt?	☒	☐	

Additional Comments:

Data Review Checklist for the Verification and Validation of Polarized Light Microscopy (PLM) Data Deliverables

3.0 Sample Result Verification & Validation (Level 1, 2 & 3)	Yes	No	Comments
3.1 Prior to analysis by PLM, are samples examined at low magnification using a stereoscope?	☒	☐	
3.1.1 Are the following observations recorded for each sample:			
3.1.1.1 Color?	☒	☐	
3.1.1.2 Texture?	☒	☐	
3.1.1.3 Percent (%) fibrous material?	☒	☐	
3.2 Is the technique used to prepare samples to slides recorded (i.e. particle size reduction, acid treatment, heating, melting or teasing)?	☒	☐	
3.3 Were gravimetric analysis performed?	☒	☐	ASTM D7521
3.3.1 If yes, were the necessary sample weights and tare weights recorded and provided?	☒	☐	
Using the recorded weights, recalculate a minimum of 10% of the samples for which gravimetric analysis was performed.			
3.3.1.1 Are the recalculated concentrations consistent with those reported?	☒	☐	
3.4 Is qualitative identification of fibrous materials made by examining fiber morphology and observance of optical properties?	☒	☐	.
3.4.1 Are the following recorded for all reported fibrous materials:			
3.4.1.1 Morphology?	☒	☐	
3.4.1.2 Refractive Indices?	☒	☐	
3.4.1.3 Sign of Elongation?	☒	☐	
3.4.1.4 Extinction Angle?	☒	☐	
3.4.1.5 Pleochroism?	☒	☐	
3.4.1.6 Birefringence?	☒	☐	
3.5 Do the recorded morphology and optical properties in the raw data agree with the type of fibrous material(s) reported?	☒	☐	
Note: Refer to Attachments A and B of SOP QATS-70-090 for the morphology and optical properties of various asbestos and non-asbestos fibers.			

Additional Comments:

Data Review Checklist for the Verification and Validation of Polarized Light Microscopy (PLM) Data Deliverables

3.0 Sample Result Verification & Validation (Level 1, 2 & 3)	Yes	No	Comments
3.6 Was quantitative analysis performed by point counting?	☐	☒	
3.6.1 Was the point counting performed as described in the project and/or method specified?	☐	☐	
3.6.2 Where the following recorded:			
3.6.2.1 Magnification?	☐	☐	
3.6.2.2 Graticule size/type?	☐	☐	
3.6.2.3 Number of slide mounts prepared?	☐	☐	
3.6.2.4 Empty and non-empty points counted?	☐	☐	
3.6.2.5 The observance of fibers in a field of view, but not directly under a point?	☐	☐	

4.0 Quality Control Verification & Validation (Level 2 and 3)	Yes	No	Comments
4.1 Blanks			
4.1.1 Are laboratory contamination blanks prepared and analyzed at the required frequency?	☐	☒	A daily calibration blank was not analyzed on 01/26/2015, affecting 6 samples.
4.1.2 Are laboratory blank results within the specified criteria?	☒	☐	
4.1.2.1 If "no" then qualify the associated results in accordance with the Blank Result table in SOP QATS-70-090.			
4.2 Replicate Analyses			
4.2.1 Are replicate (reanalyzed by the same or second analyst) sample analyses performed at the required frequency?	☒	☐	
4.2.2 Are replicate sample results within the specified acceptance limits?	☒	☐	The laboratory performed three Laboratory Duplicates (by the same analyst) on EPA Sample Nos. 14394106, 14394117, and 14394123 and two Laboratory Duplicates (by a different analyst) on EPA Sample Nos. 14394106 and 14394109.
4.3 Duplicate Analyses			
4.3.1 Are duplicate sample analyses performed at the required frequency?	☒	☐	
4.3.2 Are duplicate sample results within the specified acceptance limits?	☒	☐	
4.3.2.1 If "no" then qualify the associated results in accordance with the Analytical Variability Results table in SOP QATS-70-090.			

Additional Comments:

Data Review Checklist for the Verification and Validation of Polarized Light Microscopy (PLM) Data Deliverables

4.0 Quality Control Verification & Validation (Level 2 and 3)	Yes	No	Comments
4.4 Reference Slide Analysis (if applicable)			The PLM Reference Material Comparison spreadsheet, PLM Accuracy QC and RTI Reference Material Comparison.xls, provided Reference Material results for analyst R. Brown. The analyst for the samples in this SDG is S. Golden; therefore, the PLM Reference Material analysis could not be evaluated.
4.4.1 Are reference slide analyses performed at the required frequency?	☒	☐	
4.4.2 Are the reference slide analyses results within the specified acceptance criteria?	☒	☐	
4.4.2.1 If "no" then qualify the associated results in accordance with the Reference Slide Analysis table in SOP QATS-70-090.			
**5.0 Calibration & Microscope Alignment Validation (Level 3)**			
5.1 Are evidence of microscope alignment and Refractive Index (RI) liquid calibration provided for all sample analyses?	☒	☐	
5.1.1 Microscope-specific alignment checks?	☐	☒	The daily scope alignment and contamination blank check were performed for all dates except 01/26/2015, resulting in the qualification of 6 samples analyzed on that date.
5.1.2 Microscope-specific contamination checks?	☐	☒	
5.1.3 Calibration RI liquids?	☒	☐	
5.2 Are alignment and calibration checks listed above performed at the required frequencies?	☐	☒	
5.3 Are alignment and calibration checks within the specified criteria?	☒	☐	
5.4 Are all alignment and calibration checks traceable to the associated samples analyses?	☒	☐	
5.4.1 If "no" then qualify the associated results in accordance with the Calibration Results table in SOP QATS-70-090.			
**6.0 Case Narrative Validation (Levels 2 & 3)**			
6.1 Does the data package narrative include descriptions of the following:			
6.1.1 Samples received (matrix/method)?	☒	☐	
6.1.2 Method/project requirement deviations?	☒	☐	
6.1.3 Example sample calculation?	☒	☐	
6.1.4 Laboratory blank contamination?	☐	☐	NA
6.1.5 Quality control analyses outside specified criteria?	☐	☐	NA
6.1.6 Any problems encountered and subsequent corrective action?	☐	☐	NA

Additional Comments:

Validator's Signature **Shellee McGrath** **Date 06/08/2015**

QA Review **Lyndsay Gensler** **Date 06/19/2015**

Appendix B

Qualified Result Forms

National Asbestos Data Entry Spreadsl

Site or Project Name: Sumas Mtn Asbestos Soil | Site/Project Identifier Code: SFP-078A | Date received by lab: 12/15/14

Chain of Custody Number: 121545-0001

State/Federal Site No or Project Identifier: SFP-078A

WARNING: The current file w saved and Microsoft Excel wi as part of the export process

Lab Name: Lab/Cor Portland, Inc.
Lab Job No: 142958 | Method: SRC-Libby-03_VE

Data Entry by: S. Golden | QA by: K. March
Data Entry Date: 03/18/15 | QA Date: 03/29/15

Client Sample ID	Sample Type	Index Suffix Char.	Index Suffix No.	QA Type (a)	Lab Sample ID	Date Analyzed	Analyst Name	Sample Appearance	Ref Material	Base Mineral Type of Reference Material (b)	Actinolite (AC) Qual	Actinolite (AC) AC-AF (%)	Actinolite (AC) AC-MF (%)	Amosite (AM) Qual	Amosite (AM) AM-AF (%)	Amosite (AM) AM-MF (%)	Ant Qual
14394100	soil			Not QA	142958-S1	1/25/2015	S. Golden	Coarse, Medium, Fine Grain			ND	0	0	ND	0	0	ND
14394101	soil			Not QA	142958-S2	1/25/2015	S. Golden	Coarse, Medium, Fine Grain			ND	0	0	ND	0	0	ND
14394102	soil			Not QA	142958-S3	1/25/2015	S. Golden	Coarse, Medium, Fine Grain			ND	0	0	ND	0	0	ND
14394103	soil			Not QA	142958-S4	1/25/2015	S. Golden	Coarse, Medium, Fine Grain			ND	0	0	ND	0	0	ND
14394104	soil			Not QA	142958-S5	1/25/2015	S. Golden	Coarse, Medium, Fine Grain			ND	0	0	ND	0	0	ND
14394105	soil			Not QA	142958-S6	1/25/2015	S. Golden	Coarse, Medium, Fine Grain			ND	0	0	ND	0	0	ND
14394106	soil			Not QA	142958-S7	1/25/2015	S. Golden	Coarse, Medium, Fine Grain		RC*	ND	0	0	ND	0	0	ND
14394107	soil			Not QA	142958-S8	1/25/2015	S. Golden	Coarse, Medium, Fine Grain			ND	0	0	ND	0	0	ND
14394108	soil			Not QA	142958-S9	1/26/2015	S. Golden	Coarse, Medium, Fine Grain	UJ	MC	ND	0	0	ND	0	0	ND
14394109	soil			Not QA	142958-S10	1/26/2015	S. Golden	Coarse, Medium, Fine Grain	UJ	MC	ND	0	0	ND	0	0	ND
14394110	soil			Not QA	142958-S11	1/26/2015	S. Golden	Coarse, Medium, Fine Grain	UJ	MC	ND	0	0	ND	0	0	ND
14394111	soil			Not QA	142958-S12	1/26/2015	S. Golden	Coarse, Medium, Fine Grain	UJ	MC	ND	0	0	ND	0	0	ND
14394112	soil			Not QA	142958-S13	1/26/2015	S. Golden	Coarse, Medium, Fine Grain	UJ	MC	ND	0	0	ND	0	0	ND
14394113	soil			Not QA	142958-S14	1/26/2015	S. Golden	Coarse, Medium, Fine Grain	UJ	MC	ND	0	0	ND	0	0	ND
14394114	soil			Not QA	142958-S15	1/27/2015	S. Golden	Coarse, Medium, Fine Grain			ND	0	0	ND	0	0	ND
14394115	soil			Not QA	142958-S16	1/27/2015	S. Golden	Coarse, Medium, Fine Grain			ND	0	0	ND	0	0	ND
14394116	soil			Not QA	142958-S17	1/30/2015	S. Golden	Coarse, Medium, Fine Grain			ND	0	0	ND	0	0	ND
14394117	soil			Not QA	142958-S18	1/30/2015	S. Golden	Coarse, Medium, Fine Grain			ND	0	0	ND	0	0	ND
14394118	soil			Not QA	142958-S19	1/30/2015	S. Golden	Coarse, Medium, Fine Grain			ND	0	0	ND	0	0	ND
14394119	soil			Not QA	142958-S20	1/30/2015	S. Golden	Coarse, Medium, Fine Grain			ND	0	0	ND	0	0	ND
14394120	soil			Not QA	142958-S21	1/30/2015	S. Golden	Coarse, Medium, Fine Grain			ND	0	0	ND	0	0	ND
14394121	soil			Not QA	142958-S22	1/30/2015	S. Golden	Coarse, Medium, Fine Grain			ND	0	0	ND	0	0	ND
14394122	soil			Not QA	142958-S23	1/30/2015	S. Golden	Coarse, Medium, Fine Grain			ND	0	0	ND	0	0	ND
14394123	soil			Not QA	142958-S24	1/30/2015	S. Golden	Coarse, Medium, Fine Grain			ND	0	0	ND	0	0	ND

*RC= Reason Code

Sumas Mtn Asbestos Soil

heet (NADES) for Bulk & Soil Analysis by PLM Visual Estimation

ill be
ll close

thophyllite (AN)		Chrysotile (CH)			Crocidolite (CR)			Tremolite (TR)			winchite/richterite/ tremolite/actinolite (WRTA)			Other Amphibole (OA)			Non-asbestos Material (NAM)			Other Mineral Type (OM)			
AN-AF (%)	AN-MF (%)	Qual	CH-AF (%)	CH-MF (%)	Qual	CR-AF (%)	CR-MF (%)	Qual	TR-AF (%)	TR-MF (%)	Qual	WRTA-AF (%)	WRTA-MF (%)	Qual	OA-AF(%)	OA-MF(%)	Qual	NAM-AF (%)	NAM-MF (%)	Qual	OM-AF (%)	OM-MF (%)	OM Type
0	0	ND	0	0	ND	0	0	ND	0	0	ND	0	0	ND	0	0	ND	0	0	ND	0	0	
0	0	ND	0	0	ND	0	0	ND	0	0	ND	0	0	ND	0	0	ND	0	0	ND	0	0	
0	0	ND	0	0	ND	0	0	ND	0	0	ND	0	0	ND	0	0	ND	0	0	ND	0	0	
0	0	ND	0	0	ND	0	0	ND	0	0	ND	0	0	ND	0	0	ND	0	0	ND	0	0	
0	0	ND	0	0	ND	0	0	ND	0	0	ND	0	0	ND	0	0	ND	0	0	ND	0	0	
0	0	ND	0	0	ND	0	0	ND	0	0	ND	0	0	ND	0	0	ND	0	0	ND	0	0	
0	0	ND	0	0	ND	0	0	ND	0	0	ND	0	0	ND	0	0	ND	0	0	ND	0	0	
0	0	ND	0	0	ND	0	0	ND	0	0	ND	0	0	ND	0	0	ND	0	0	ND	0	0	
0	0	ND	0	0	ND	0	0	ND	0	0	ND	0	0	ND	0	0	ND	0	0	ND	0	0	
0	0	ND	0	0	ND	0	0	ND	0	0	ND	0	0	ND	0	0	ND	0	0	ND	0	0	
0	0	ND	0	0	ND	0	0	ND	0	0	ND	0	0	ND	0	0	ND	0	0	ND	0	0	
0	0	ND	0	0	ND	0	0	ND	0	0	ND	0	0	ND	0	0	ND	0	0	ND	0	0	
0	0	ND	0	0	ND	0	0	ND	0	0	ND	0	0	ND	0	0	ND	0	0	ND	0	0	
0	0	ND	0	0	ND	0	0	ND	0	0	ND	0	0	ND	0	0	ND	0	0	ND	0	0	
0	0	ND	0	0	ND	0	0	ND	0	0	ND	0	0	ND	0	0	ND	0	0	ND	0	0	
0	0	ND	0	0	ND	0	0	ND	0	0	ND	0	0	ND	0	0	ND	0	0	ND	0	0	
0	0	ND	0	0	ND	0	0	ND	0	0	ND	0	0	ND	0	0	ND	0	0	ND	0	0	
0	0	ND	0	0	ND	0	0	ND	0	0	ND	0	0	ND	0	0	ND	0	0	ND	0	0	
0	0	ND	0	0	ND	0	0	ND	0	0	ND	0	0	ND	0	0	ND	0	0	ND	0	0	
0	0	ND	0	0	ND	0	0	ND	0	0	ND	0	0	ND	0	0	ND	0	0	ND	0	0	
0	0	ND	0	0	ND	0	0	ND	0	0	ND	0	0	ND	0	0	ND	0	0	ND	0	0	
0	0	ND	0	0	ND	0	0	ND	0	0	ND	0	0	ND	0	0	ND	0	0	ND	0	0	
0	0	ND	0	0	ND	0	0	ND	0	0	ND	0	0	ND	0	0	ND	0	0	ND	0	0	
0	0		2.259		ND	0	0	ND	0	0	ND	0	0	ND	0	0	ND	0	0	ND	0	0	

Lab/Cor Portland, Inc.

Customer Name: Alion Science and Technology
Cust. Project Name: Sumas Mtn Asbestos Soil
Cust. Project Number: 10-100614-121545-0001
LCP Job Number: 142958

Total Asbestos (%)=

$$\frac{[\%_F \text{ PLM PC} * W_F]+[\%_M \text{PLM} * W_M]+[\%_C \text{PLM} * W_C]}{W_F + W_M + W_C}$$

		Fine Fraction			Medium Fraction			Coarse Fraction		Total %
Client Sample ID	Sample Number	W_F	PC		W_M	PLM		W_C	PLM	
14394100	S1	6.8	0	*	60.6	0		113.8	0	0
14394101	S2	9.2	0	*	86.6	0		133.4	0	0
14394102	S3	5.8	0	*	61	0		24.6	0	0
14394103	S4	9.8	0	*	137.4	0		65.8	0	0
14394104	S5	7.6	0	*	77	0		45.2	0	0
14394105	S6	7.8	0	*	78	0		45.8	0	0
14394106	S7	7.4	0	*	83.4	0		44.2	0	0
14394107	S8	7.2	0	*	88	0		64	0	0
14394108	S9	6.8	0	*	70.8	0		71.8	0	0
14394109	S10	6	0	*	61	0		54.6	0	0
14394110	S11	7	0	*	77.4	0		64	0	0
14394111	S12	11.4	0	*	108.2	0		60.6	0	0
14394112	S13	9.6	0	*	77.2	0		134.6	0	0
14394113	S14	8.8	0	*	104.6	0		82.4	0	0
14394114	S15	9.8	0	*	75	0		140	0	0
14394115	S16	10.8	0	*	109.4	0		78.4	0	0
14394116	S17	8	0	*	97	0		47.4	0	0
14394117	S18	8.6	0	*	89.2	0		48.8	0	0
14394118	S19	7.2	0	*	84.8	0		47.6	0	0
14394119	S20	12.2	0	*	108.8	0		109	0	0
14394120	S21	10.8	0	*	116.6	0		83.4	0	0
14394121	S22	9.1	0	*	132.2	0		81.4	0	0
14394122	S23	11.2	0	*	143.2	0	**	80.2	0	0
14394123	S24	12.2	2		221.4	2		81.8	3	2.2594

* Indicates samples that were sent to TEM Analysis
** Indicates trace (<1%) asbestos by PLM

LabCor Portland Inc

Lab/Cor Portland, Inc.
4321 SW Corbett Ave., Ste A
Portland, OR 97239

Phone: (503) 224-5055
Fax: (503) 228-8282
http://www.labcorpdx.net

Final Report

Asbestos and Environmental Analysis

EPA 600-R-93-116 - TEM - Bulk Quantitative

Job Number: 142958 **PDX**
Client: Alion Science and Technology
Project Name: Sumas Mtn Asbestos Soil
Project No.: 10-100614-121545-0001

Report Number: 142958R02
Date Received: 12/15/2014

Lab/Cor Sample No. : S1
Client Sample No. : 14394100 -
GRR : 0.359
Dilution : 0.0025
Dilution Factor : 1

As Received Weight (g) : 189.60
Lab Filter Area (mm2) : 193
Grid Openings Analyzed : 10
Average Grid Opening Area (mm2) : 0.01042
Area Analyzed (mm2) : 0.1042
Analytical Sens. (Weight Percent) : 1.33E-07
Analytical Sens. (struc/g) : 2.21E+06

Analyst(s)	Analysis Date	Microscope	Magnification
TH	2/3/2015	H-7000	20000
TH	2/8/2015	H-7000	20000

Structure Type	Weight Percent (%)	Concentration (struc/g)	Struct Count[1] Primary/Total		Wa
Total Asbestos Structures	NA*	< 2213154.996		0	0.00E+00
Total Asb & Libby-OtherAmph Structures	NA*	< 2213154.996		0	0.00E+00
Total Chrysotile Structures	NA*	< 2213154.996		0	0.00E+00

Lab/Cor Sample No. : S2
Client Sample No. : 14394101 -
GRR : 0.359
Dilution : 0.0025
Dilution Factor : 1

As Received Weight (g) : 229.80
Lab Filter Area (mm2) : 193
Grid Openings Analyzed : 10
Average Grid Opening Area (mm2) : 0.01042
Area Analyzed (mm2) : 0.1042
Analytical Sens. (Weight Percent) : 1.33E-07
Analytical Sens. (struc/g) : 2.21E+06

Analyst(s)	Analysis Date	Microscope	Magnification
TH	2/3/2015	H-7000	20000

Structure Type	Weight Percent (%)	Concentration (struc/g)	Struct Count[1] Primary/Total		Wa
Total Asbestos Structures	NA*	< 2213154.996		0	0.00E+00
Total Asb & Libby-OtherAmph Structures	NA*	< 2213154.996		0	0.00E+00
Total Chrysotile Structures	NA*	< 2213154.996		0	0.00E+00

* NA - weight percent is only calculated using total structures, counting categories that are counted using primary structures are not calculated.

LabCor Portland Inc
Lab/Cor Portland, Inc.
4321 SW Corbett Ave., Ste A
Portland, OR 97239

Final Report

Phone: (503) 224-5055
Fax: (503) 228-8282
http://www.labcorpdx.net

Asbestos and Environmental Analysis

EPA 600-R-93-116 - TEM - Bulk Quantitative

Job Number: 142958 PDX
Client: Alion Science and Technology
Project Name: Sumas Mtn Asbestos Soil
Project No.: 10-100614-121545-0001

Report Number: 142958R02
Date Received: 12/15/2014

Lab/Cor Sample No. : S3
Client Sample No. : 14394102 -
GRR : 0.235
Dilution : 0.0025
Dilution Factor : 1

As Received Weight (g) : 92.40
Lab Filter Area (mm2) : 193
Grid Openings Analyzed : 10
Average Grid Opening Area (mm2) : 0.01042
Area Analyzed (mm2) : 0.1042
Analytical Sens. (Weight Percent) : 9.18E-08
Analytical Sens. (struc/g) : 1.53E+06

Analyst(s)	Analysis Date	Microscope	Magnification
TH	2/3/2015	H-7000	20000
TH	2/4/2015	H-7000	20000
TH	2/8/2015	H-7000	20000

Structure Type	Weight Percent (%)	Concentration (struc/g)	Struct Count[1] Primary/Total		Wa
Total Asbestos Structures	NA*	< 1528197.012		0	0.00E+00
Total Asb & Libby-OtherAmph Structures	NA*	< 1528197.012		0	0.00E+00
Total Chrysotile Structures	NA*	< 1528197.012		0	0.00E+00

Lab/Cor Sample No. : S4
Client Sample No. : 14394103 -
GRR : 0.266
Dilution : 0.0025
Dilution Factor : 1

As Received Weight (g) : 215.80
Lab Filter Area (mm2) : 193
Grid Openings Analyzed : 10
Average Grid Opening Area (mm2) : 0.01042
Area Analyzed (mm2) : 0.1042
Analytical Sens. (Weight Percent) : 1.14E-07
Analytical Sens. (struc/g) : 1.90E+06

Analyst(s)	Analysis Date	Microscope	Magnification
TH	2/4/2015	H-7000	20000

Structure Type	Weight Percent (%)	Concentration (struc/g)	Struct Count[1] Primary/Total		Wa
Total Asbestos Structures	2.07E-02	1.14E+07		6	2.02E-07
Total Asb & Libby-OtherAmph Structures	2.07E-02	1.14E+07		6	2.02E-07
Total Chrysotile Structures	2.07E-02	1.14E+07		6	2.02E-07

* NA - weight percent is only calculated using total structures, counting categories that are counted using primary structures are not calculated.

LabCor Portland Inc

Lab/Cor Portland, Inc.

4321 SW Corbett Ave., Ste A
Portland, OR 97239

Phone: (503) 224-5055
Fax: (503) 228-8282
http://www.labcorpdx.net

Final Report

Asbestos and Environmental Analysis

EPA 600-R-93-116 - TEM - Bulk Quantitative

Job Number: 142958 **PDX**
Client: Alion Science and Technology
Project Name: Sumas Mtn Asbestos Soil
Project No.: 10-100614-121545-0001

Report Number: 142958R02
Date Received: 12/15/2014

Lab/Cor Sample No. : S5
Client Sample No. : 14394104 -
GRR : 0.252
Dilution : 0.0025
Dilution Factor : 1

As Received Weight (g) : 130.60
Lab Filter Area (mm2) : 193
Grid Openings Analyzed : 10
Average Grid Opening Area (mm2) : 0.01042
Area Analyzed (mm2) : 0.1042
Analytical Sens. (Weight Percent) : 1.00E-07
Analytical Sens. (struc/g) : 1.67E+06

Analyst(s)	Analysis Date	Microscope	Magnification
TH	2/4/2015	H-7000	20000

Structure Type	Weight Percent (%)	Concentration (struc/g)	Struct Count¹ Primary	Struct Count¹ Total	Wa
Total Asbestos Structures	NA*	< 1666837.74		0	0.00E+00
Total Asb & Libby-OtherAmph Structures	NA*	< 1666837.74		0	0.00E+00
Total Chrysotile Structures	NA*	< 1666837.74		0	0.00E+00

Lab/Cor Sample No. : S6
Client Sample No. : 14394105 -
GRR : 0.241
Dilution : 0.0025
Dilution Factor : 1

As Received Weight (g) : 133.20
Lab Filter Area (mm2) : 193
Grid Openings Analyzed : 10
Average Grid Opening Area (mm2) : 0.01042
Area Analyzed (mm2) : 0.1042
Analytical Sens. (Weight Percent) : 1.11E-07
Analytical Sens. (struc/g) : 1.84E+06

Analyst(s)	Analysis Date	Microscope	Magnification
TH	2/4/2015	H-7000	20000

Structure Type	Weight Percent (%)	Concentration (struc/g)	Struct Count¹ Primary	Struct Count¹ Total	Wa
Total Asbestos Structures	NA*	< 1839422.923		0	0.00E+00
Total Asb & Libby-OtherAmph Structures	NA*	< 1839422.923		0	0.00E+00
Total Chrysotile Structures	NA*	< 1839422.923		0	0.00E+00

Lab/Cor Sample No. : S7
Client Sample No. : 14394106 -
GRR : 0.333
Dilution : 0.0025
Dilution Factor : 1

As Received Weight (g) : 135.60
Lab Filter Area (mm2) : 193
Grid Openings Analyzed : 10
Average Grid Opening Area (mm2) : 0.01042
Area Analyzed (mm2) : 0.1042
Analytical Sens. (Weight Percent) : 1.28E-07
Analytical Sens. (struc/g) : 2.14E+06

Analyst(s)	Analysis Date	Microscope	Magnification
TH	2/4/2015	H-7000	20000

Structure Type	Weight Percent (%)	Concentration (struc/g)	Struct Count¹ Primary	Struct Count¹ Total	Wa
Total Asbestos Structures	NA*	< 2138273.631		0	0.00E+00
Total Asb & Libby-OtherAmph Structures	NA*	< 2138273.631		0	0.00E+00
Total Chrysotile Structures	NA*	< 2138273.631		0	0.00E+00

* NA - weight percent is only calculated using total structures, counting categories that are counted using primary structures are not calculated.

LabCor Portland Inc

Lab/Cor Portland, Inc.

4321 SW Corbett Ave., Ste A
Portland, OR 97239

Final Report

Asbestos and Environmental Analysis

Phone: (503) 224-5055
Fax: (503) 228-8282
http://www.labcorpdx.net

EPA 600-R-93-116 - TEM - Bulk Quantitative

Job Number: 142958 **PDX**
Client: **Alion Science and Technology**
Project Name: Sumas Mtn Asbestos Soil
Project No.: 10-100614-121545-0001

Report Number: 142958R02
Date Received: 12/15/2014

Lab/Cor Sample No. : S8
Client Sample No. : 14394107 -
GRR : 0.205
Dilution : 0.0025
Dilution Factor : 1

As Received Weight (g) : 160.00
Lab Filter Area (mm2) : 193
Grid Openings Analyzed : 10
Average Grid Opening Area (mm2) : 0.01042
Area Analyzed (mm2) : 0.1042
Analytical Sens. (Weight Percent) : 9.55E-08
Analytical Sens. (struc/g) : 1.59E+06

Analyst(s)	Analysis Date	Microscope	Magnification
TH	2/4/2015	H-7000	20000

Structure Type	Weight Percent (%)	Concentration (struc/g)	Struct Count[1] Primary/Total		Wa
Total Asbestos Structures	NA*	< 1589378.381		0	0.00E+00
Total Asb & Libby-OtherAmph Structures	NA*	< 1589378.381		0	0.00E+00
Total Chrysotile Structures	NA*	< 1589378.381		0	0.00E+00

Lab/Cor Sample No. : S9
Client Sample No. : 14394108 -
GRR : 0.212
Dilution : 0.0025
Dilution Factor : 1

As Received Weight (g) : 149.80
Lab Filter Area (mm2) : 193
Grid Openings Analyzed : 11
Average Grid Opening Area (mm2) : 0.01042
Area Analyzed (mm2) : 0.11462
Analytical Sens. (Weight Percent) : 7.41E-08
Analytical Sens. (struc/g) : 1.23E+06

Analyst(s)	Analysis Date	Microscope	Magnification
TH	2/4/2015	H-7000	20000

Structure Type	Weight Percent (%)	Concentration (struc/g)	Struct Count[1] Primary/Total		Wa
Total Asbestos Structures	NA*	< 1232527.787		0	0.00E+00
Total Asb & Libby-OtherAmph Structures	NA*	< 1232527.787		0	0.00E+00
Total Chrysotile Structures	NA*	< 1232527.787		0	0.00E+00

Lab/Cor Sample No. : S10
Client Sample No. : 14394109 -
GRR : 0.326
Dilution : 0.0025
Dilution Factor : 1

As Received Weight (g) : 121.20
Lab Filter Area (mm2) : 193
Grid Openings Analyzed : 10
Average Grid Opening Area (mm2) : 0.01042
Area Analyzed (mm2) : 0.1042
Analytical Sens. (Weight Percent) : 1.22E-07
Analytical Sens. (struc/g) : 2.03E+06

Analyst(s)	Analysis Date	Microscope	Magnification
TH	2/4/2015	H-7000	20000

RC

Structure Type	Weight Percent (%)	Concentration (struc/g)	Struct Count[1] Primary/Total		Wa
Total Asbestos Structures	2.81E-03	2.03E+06		1	2.56E-08
Total Asb & Libby-OtherAmph Structures	2.81E-03	2.03E+06		1	2.56E-08
Total Chrysotile Structures	NA*	< 2029475.095		0	0.00E+00

1C

* NA - weight percent is only calculated using total structures, counting categories that are counted using primary structures are not calculated.

LabCor Portland Inc
Lab/Cor Portland, Inc.
4321 SW Corbett Ave., Ste A
Portland, OR 97239

Final Report

Asbestos and Environmental Analysis

Phone: (503) 224-5055
Fax: (503) 228-8282
http://www.labcorpdx.net

EPA 600-R-93-116 - TEM - Bulk Quantitative

Job Number: 142958 **PDX**
Client: Alion Science and Technology
Project Name: Sumas Mtn Asbestos Soil
Project No.: 10-100614-121545-0001

Report Number: 142958R02
Date Received: 12/15/2014

Lab/Cor Sample No. : S11
Client Sample No. : 14394110 -
GRR : 0.205
Dilution : 0.0025
Dilution Factor : 1

As Received Weight (g) : 148.60
Lab Filter Area (mm2) : 193
Grid Openings Analyzed : 10
Average Grid Opening Area (mm2) : 0.01042
Area Analyzed (mm2) : 0.1042
Analytical Sens. (Weight Percent) : 5.50E-08
Analytical Sens. (struc/g) : 9.16E+05

Analyst(s)	Analysis Date	Microscope	Magnification
TH	2/4/2015	H-7000	20000

Structure Type	Weight Percent (%)	Concentration (struc/g)	Struct Count[1] Primary	Struct Count[1] Total	Wa
Total Asbestos Structures	1.10E-05	9.16E+05		1	2.22E-10
Total Asb & Libby-OtherAmph Structures	1.10E-05	9.16E+05		1	2.22E-10
Total Chrysotile Structures	1.10E-05	9.16E+05		1	2.22E-10

Lab/Cor Sample No. : S12
Client Sample No. : 14394111 -
GRR : 0.218
Dilution : 0.0025
Dilution Factor : 1

As Received Weight (g) : 179.80
Lab Filter Area (mm2) : 193
Grid Openings Analyzed : 10
Average Grid Opening Area (mm2) : 0.01042
Area Analyzed (mm2) : 0.1042
Analytical Sens. (Weight Percent) : 8.91E-08
Analytical Sens. (struc/g) : 1.48E+06

Analyst(s)	Analysis Date	Microscope	Magnification
TH	2/4/2015	H-7000	20000

Structure Type	Weight Percent (%)	Concentration (struc/g)	Struct Count[1] Primary	Struct Count[1] Total	Wa
Total Asbestos Structures	NA*	< 1482309.802		0	0.00E+00
Total Asb & Libby-OtherAmph Structures	NA*	< 1482309.802		0	0.00E+00
Total Chrysotile Structures	NA*	< 1482309.802		0	0.00E+00

Lab/Cor Sample No. : S13
Client Sample No. : 14394112 -
GRR : 0.222
Dilution : 0.0025
Dilution Factor : 1

As Received Weight (g) : 221.80
Lab Filter Area (mm2) : 193
Grid Openings Analyzed : 10
Average Grid Opening Area (mm2) : 0.01042
Area Analyzed (mm2) : 0.1042
Analytical Sens. (Weight Percent) : 9.00E-08
Analytical Sens. (struc/g) : 1.50E+06

Analyst(s)	Analysis Date	Microscope	Magnification
TH	2/4/2015	H-7000	20000

Structure Type	Weight Percent (%)	Concentration (struc/g)	Struct Count[1] Primary	Struct Count[1] Total	Wa
Total Asbestos Structures	3.54E-05	1.50E+06		1	4.37E-10
Total Asb & Libby-OtherAmph Structures	3.54E-05	1.50E+06		1	4.37E-10
Total Chrysotile Structures	3.54E-05	1.50E+06		1	4.37E-10

* NA - weight percent is only calculated using total structures, counting categories that are counted using primary structures are not calculated.

LabCor Portland Inc

Lab/Cor Portland, Inc.
4321 SW Corbett Ave., Ste A
Portland, OR 97239

Phone: (503) 224-5055
Fax: (503) 228-8282
http://www.labcorpdx.net

Final Report

Asbestos and Environmental Analysis

EPA 600-R-93-116 - TEM - Bulk Quantitative

Job Number: 142958 **PDX**
Client: Alion Science and Technology
Project Name: Sumas Mtn Asbestos Soil
Project No.: 10-100614-121545-0001

Report Number: 142958R02
Date Received: 12/15/2014

Lab/Cor Sample No. : S14
Client Sample No. : 14394113 -
GRR : 0.212
Dilution : 0.01
Dilution Factor : 1

As Received Weight (g) : 198.80
Lab Filter Area (mm2) : 193
Grid Openings Analyzed : 10
Average Grid Opening Area (mm2) : 0.01042
Area Analyzed (mm2) : 0.1042
Analytical Sens. (Weight Percent) : 4.54E-08
Analytical Sens. (struc/g) : 7.55E+05

Analyst(s)	Analysis Date	Microscope	Magnification
TH	2/4/2015	H-7000	20000

Structure Type	Weight Percent (%)	Concentration (struc/g)	Struct Count[1] Primary/Total		Wa
Total Asbestos Structures	NA*	< 755421.212		0	0.00E+00
Total Asb & Libby-OtherAmph Structures	NA*	< 755421.212		0	0.00E+00
Total Chrysotile Structures	NA*	< 755421.212		0	0.00E+00

Lab/Cor Sample No. : S15
Client Sample No. : 14394114 -
GRR : 0.238
Dilution : 0.0025
Dilution Factor : 1

As Received Weight (g) : 224.60
Lab Filter Area (mm2) : 193
Grid Openings Analyzed : 10
Average Grid Opening Area (mm2) : 0.01042
Area Analyzed (mm2) : 0.1042
Analytical Sens. (Weight Percent) : 1.02E-07
Analytical Sens. (struc/g) : 1.69E+06

Analyst(s)	Analysis Date	Microscope	Magnification
TH	2/4/2015	H-7000	20000

Structure Type	Weight Percent (%)	Concentration (struc/g)	Struct Count[1] Primary/Total		Wa
Total Asbestos Structures	NA*	< 1689471.442		0	0.00E+00
Total Asb & Libby-OtherAmph Structures	NA*	< 1689471.442		0	0.00E+00
Total Chrysotile Structures	NA*	< 1689471.442		0	0.00E+00

Lab/Cor Sample No. : S16
Client Sample No. : 14394115 -
GRR : 0.321
Dilution : 0.0025
Dilution Factor : 1

As Received Weight (g) : 199.20
Lab Filter Area (mm2) : 193
Grid Openings Analyzed : 10
Average Grid Opening Area (mm2) : 0.01042
Area Analyzed (mm2) : 0.1042
Analytical Sens. (Weight Percent) : 1.25E-07
Analytical Sens. (struc/g) : 2.09E+06

Analyst(s)	Analysis Date	Microscope	Magnification
TH	2/4/2015	H-7000	20000

Structure Type	Weight Percent (%)	Concentration (struc/g)	Struct Count[1] Primary/Total		Wa
Total Asbestos Structures	NA*	< 2086170.32		0	0.00E+00
Total Asb & Libby-OtherAmph Structures	NA*	< 2086170.32		0	0.00E+00
Total Chrysotile Structures	NA*	< 2086170.32		0	0.00E+00

* NA - weight percent is only calculated using total structures, counting categories that are counted using primary structures are not calculated.

LabCor Portland Inc **Lab/Cor Portland, Inc.**
4321 SW Corbett Ave., Ste A
Portland, OR 97239

Phone: (503) 224-5055
Fax: (503) 228-8282
http://www.labcorpdx.net

Final Report

Asbestos and Environmental Analysis

EPA 600-R-93-116 - TEM - Bulk Quantitative

Job Number: 142958 **PDX**
Client: Alion Science and Technology
Project Name: Sumas Mtn Asbestos Soil
Project No.: 10-100614-121545-0001

Report Number: 142958R02
Date Received: 12/15/2014

Lab/Cor Sample No. : S17
Client Sample No. : 14394116 -
GRR : 0.174
Dilution : 0.0025
Dilution Factor : 1

As Received Weight (g) : 152.00
Lab Filter Area (mm2) : 193
Grid Openings Analyzed : 10
Average Grid Opening Area (mm2) : 0.01042
Area Analyzed (mm2) : 0.1042
Analytical Sens. (Weight Percent) : 3.39E-08
Analytical Sens. (struc/g) : 5.63E+05

Analyst(s)	Analysis Date	Microscope	Magnification
TH	2/4/2015	H-7000	20000

Structure Type	Weight Percent (%)	Concentration (struc/g)	Struct Count[1] Primary	Total	Wa
Total Asbestos Structures	2.57E-05	1.13E+06		2	8.45E-10
Total Asb & Libby-OtherAmph Structures	2.57E-05	1.13E+06		2	8.45E-10
Total Chrysotile Structures	2.57E-05	1.13E+06		2	8.45E-10

Lab/Cor Sample No. : S18
Client Sample No. : 14394117 -
GRR : 0.182
Dilution : 0.0025
Dilution Factor : 1

As Received Weight (g) : 145.60
Lab Filter Area (mm2) : 193
Grid Openings Analyzed : 10
Average Grid Opening Area (mm2) : 0.01042
Area Analyzed (mm2) : 0.1042
Analytical Sens. (Weight Percent) : 4.70E-08
Analytical Sens. (struc/g) : 7.83E+05

Analyst(s)	Analysis Date	Microscope	Magnification
TH	2/4/2015	H-7000	20000

Structure Type	Weight Percent (%)	Concentration (struc/g)	Struct Count[1] Primary	Total	Wa
Total Asbestos Structures	1.60E-05	7.83E+05		1	3.78E-10
Total Asb & Libby-OtherAmph Structures	1.60E-05	7.83E+05		1	3.78E-10
Total Chrysotile Structures	1.60E-05	7.83E+05		1	3.78E-10

Lab/Cor Sample No. : S19
Client Sample No. : 14394118 -
GRR : 0.401
Dilution : 0.0025
Dilution Factor : 1

As Received Weight (g) : 130.00
Lab Filter Area (mm2) : 193
Grid Openings Analyzed : 10
Average Grid Opening Area (mm2) : 0.01042
Area Analyzed (mm2) : 0.1042
Analytical Sens. (Weight Percent) : 9.59E-08
Analytical Sens. (struc/g) : 1.60E+06

Analyst(s)	Analysis Date	Microscope	Magnification
TH	2/4/2015	H-7000	20000

Structure Type	Weight Percent (%)	Concentration (struc/g)	Struct Count[1] Primary	Total	Wa
Total Asbestos Structures	NA*	< 1596421.547		0	0.00E+00
Total Asb & Libby-OtherAmph Structures	NA*	< 1596421.547		0	0.00E+00
Total Chrysotile Structures	NA*	< 1596421.547		0	0.00E+00

* NA - weight percent is only calculated using total structures, counting categories that are counted using primary structures are not calculated.

LabCor Portland Inc

Lab/Cor Portland, Inc.
4321 SW Corbett Ave., Ste A
Portland, OR 97239

Phone: (503) 224-5055
Fax: (503) 228-8282
http://www.labcorpdx.net

Final Report

Asbestos and Environmental Analysis

EPA 600-R-93-116 - TEM - Bulk Quantitative

Job Number: 142958 **PDX**
Client: Alion Science and Technology
Project Name: Sumas Mtn Asbestos Soil
Project No.: 10-100614-121545-0001

Report Number: 142958R02
Date Received: 12/15/2014

Lab/Cor Sample No. : S20
Client Sample No. : 14394119 -
GRR : 0.344
Dilution : 0.0025
Dilution Factor : 1

As Received Weight (g) : 140.00
Lab Filter Area (mm2) : 193
Grid Openings Analyzed : 10
Average Grid Opening Area (mm2) : 0.01042
Area Analyzed (mm2) : 0.1042
Analytical Sens. (Weight Percent) : 1.15E-07
Analytical Sens. (struc/g) : 1.91E+06

Analyst(s)	Analysis Date	Microscope	Magnification
TH	2/4/2015	H-7000	20000

Structure Type	Weight Percent (%)	Concentration (struc/g)	Struct Count[1] Primary	Struct Count[1] Total	Wa
Total Asbestos Structures	NA*	< 1914828.878		0	0.00E+00
Total Asb & Libby-OtherAmph Structures	NA*	< 1914828.878		0	0.00E+00
Total Chrysotile Structures	NA*	< 1914828.878		0	0.00E+00

Lab/Cor Sample No. : S21
Client Sample No. : 14394120 -
GRR : 0.332
Dilution : 0.0025
Dilution Factor : 1

As Received Weight (g) : 211.40
Lab Filter Area (mm2) : 193
Grid Openings Analyzed : 10
Average Grid Opening Area (mm2) : 0.01042
Area Analyzed (mm2) : 0.1042
Analytical Sens. (Weight Percent) : 1.03E-07
Analytical Sens. (struc/g) : 1.72E+06

Analyst(s)	Analysis Date	Microscope	Magnification
TH	2/5/2015	H-7000	20000

Structure Type	Weight Percent (%)	Concentration (struc/g)	Struct Count[1] Primary	Struct Count[1] Total	Wa
Total Asbestos Structures	NA*	< 1717449.578		0	0.00E+00
Total Asb & Libby-OtherAmph Structures	NA*	< 1717449.578		0	0.00E+00
Total Chrysotile Structures	NA*	< 1717449.578		0	0.00E+00

Lab/Cor Sample No. : S22
Client Sample No. : 14394121 -
GRR : 0.193
Dilution : 0.01
Dilution Factor : 1

As Received Weight (g) : 224.20
Lab Filter Area (mm2) : 193
Grid Openings Analyzed : 10
Average Grid Opening Area (mm2) : 0.01042
Area Analyzed (mm2) : 0.1042
Analytical Sens. (Weight Percent) : 1.81E-08
Analytical Sens. (struc/g) : 3.02E+05

Analyst(s)	Analysis Date	Microscope	Magnification
TH	2/5/2015	H-7000	20000

Structure Type	Weight Percent (%)	Concentration (struc/g)	Struct Count[1] Primary	Struct Count[1] Total	Wa
Total Asbestos Structures	NA*	< 302049.859		0	0.00E+00
Total Asb & Libby-OtherAmph Structures	NA*	< 302049.859		0	0.00E+00
Total Chrysotile Structures	NA*	< 302049.859		0	0.00E+00

* NA - weight percent is only calculated using total structures, counting categories that are counted using primary structures are not calculated.

LabCor Portland Inc

Lab/Cor Portland, Inc.
4321 SW Corbett Ave., Ste A
Portland, OR 97239

Final Report

Phone: (503) 224-5055
Fax: (503) 228-8282
http://www.labcorpdx.net

Asbestos and Environmental Analysis

EPA 600-R-93-116 - TEM - Bulk Quantitative

Job Number: 142958 **PDX**
Client: **Alion Science and Technology**
Project Name: Sumas Mtn Asbestos Soil
Project No.: 10-100614-121545-0001

Report Number: 142958R02
Date Received: 12/15/2014

Lab/Cor Sample No. : S23
Client Sample No. : 14394122 -
GRR : 0.169
Dilution : 0.0025
Dilution Factor : 1

As Received Weight (g) : 230.80
Lab Filter Area (mm2) : 193
Grid Openings Analyzed : 10
Average Grid Opening Area (mm2) : 0.01042
Area Analyzed (mm2) : 0.1042
Analytical Sens. (Weight Percent) : 5.12E-08
Analytical Sens. (struc/g) : 8.52E+05

Analyst(s)	Analysis Date	Microscope	Magnification
TH	2/5/2015	H-7000	20000

Structure Type	Weight Percent (%)	Concentration (struc/g)	Struct Count[1] Primary	Struct Count[1] Total	Wa
**Total Asbestos Structures**	1.83E-04	8.52E+05		1	3.98E-09
**Total Asb & Libby-OtherAmph Structures**	1.83E-04	8.52E+05		1	3.98E-09
**Total Chrysotile Structures**	1.83E-04	8.52E+05		1	3.98E-09

Reviewed by:

x [signature]

Stephanie Golden
Technical Manager

* NA - weight percent is only calculated using total structures, counting categories that are counted using primary structures are not calculated.

LabCor Portland Inc

Lab/Cor Portland, Inc.
4321 SW Corbett Ave., Ste A
Portland, OR 97239

Final Report

Phone: (503) 224-5055
Fax: (503) 228-8282
http://www.labcorpdx.net

Asbestos and Environmental Analysis

EPA 600-R-93-116 - TEM - Bulk Quantitative

Job Number: Q142958a **PDX**
Client: Alion Science and Technology
Project Name: Sumas Mtn Asbestos Soil
Project No.: 10-100614-121545-0001

Report Number: Q142958aR01
Date Received: 12/15/2014

Lab/Cor Sample No. : S1
Client Sample No. : 14394100 -
GRR : 0.359
Dilution : 0.0025
Dilution Factor : 1

As Received Weight (g) : 189.60
Lab Filter Area (mm2) : 193
Grid Openings Analyzed : 10
Average Grid Opening Area (mm2) : 0.01042
Area Analyzed (mm2) : 0.1042
Analytical Sens. (Weight Percent) : 1.33E-07
Analytical Sens. (struc/g) : 2.21E+06

Analyst(s)	Analysis Date	Microscope	Magnification
TH	2/19/2015	H-7000	20000

Structure Type	Weight Percent (%)	Concentration (struc/g)	Struct Count[1] Primary	Struct Count[1] Total	Wa
Total Asbestos Structures	NA*	< 2213154.996		0	0.00E+00
Total Asb & Libby-OtherAmph Structures	NA*	< 2213154.996		0	0.00E+00
Total Chrysotile Structures	NA*	< 2213154.996		0	0.00E+00

Reviewed by:

x
Tracy Handrich
Analyst

* NA - weight percent is only calculated using total structures, counting categories that are counted using primary structures are not calculated.

LabCor Portland Inc

Lab/Cor Portland, Inc.
4321 SW Corbett Ave., Ste A
Portland, OR 97239

Final Report

Phone: (503) 224-5055
Fax: (503) 228-8282
http://www.labcorpdx.net

Asbestos and Environmental Analysis

EPA 600-R-93-116 - TEM - Bulk Quantitative

Job Number: Q142958b **PDX**
Client: Alion Science and Technology
Project Name: Sumas Mtn Asbestos Soil
Project No.: 10-100614-121545-0001

Report Number: Q142958bR01
Date Received: 12/15/2014

Lab/Cor Sample No. : S2
Client Sample No. : 14394101 -
GRR : 0.359
Dilution : 0.0025
Dilution Factor : 1

As Received Weight (g) : 229.80
Lab Filter Area (mm2) : 193
Grid Openings Analyzed : 10
Average Grid Opening Area (mm2) : 0.01042
Area Analyzed (mm2) : 0.1042
Analytical Sens. (Weight Percent) : 1.33E-07
Analytical Sens. (struc/g) : 2.21E+06

Analyst(s)	Analysis Date	Microscope	Magnification
TH	2/19/2015	H-7000	20000

Structure Type	Weight Percent (%)	Concentration (struc/g)	Struct Count[1] Primary	Struct Count[1] Total	Wa
Total Asbestos Structures	NA*	< 2213154.996		0	0.00E+00
Total Asb & Libby-OtherAmph Structures	NA*	< 2213154.996		0	0.00E+00
Total Chrysotile Structures	NA*	< 2213154.996		0	0.00E+00

Reviewed by:

x
Tracy Handrich
Analyst

* NA - weight percent is only calculated using total structures, counting categories that are counted using primary structures are not calculated.

LabCor Portland Inc

Lab/Cor Portland, Inc.
4321 SW Corbett Ave., Ste A
Portland, OR 97239

Final Report

Phone: (503) 224-5055
Fax: (503) 228-8282
http://www.labcorpdx.net

Asbestos and Environmental Analysis

EPA 600-R-93-116 - TEM - Bulk Quantitative

Job Number: Q142958d **PDX**
Client: Alion Science and Technology
Project Name: Sumas Mtn Asbestos Soil
Project No.: 10-100614-121545-0001

Report Number: Q142958dR01
Date Received: 12/15/2014

Lab/Cor Sample No. : S11
Client Sample No. : 14394110 -
GRR : 0.205
Dilution : 0.0025
Dilution Factor : 1

As Received Weight (g) : 148.60
Lab Filter Area (mm2) : 193
Grid Openings Analyzed : 10
Average Grid Opening Area (mm2) : 0.01042
Area Analyzed (mm2) : 0.1042
Analytical Sens. (Weight Percent) : 5.50E-08
Analytical Sens. (struc/g) : 9.16E+05

Analyst(s)	Analysis Date	Microscope	Magnification
PK	2/11/2015	H-7000	20000

RC

Structure Type	Weight Percent (%)	Concentration (struc/g)	Struct Count[1] Primary	Struct Count[1] Total	Wa
Total Asbestos Structures	NA*	< 916215.226		0	0.00E+00
Total Asb & Libby-OtherAmph Structures	NA*	< 916215.226		0	0.00E+00
Total Chrysotile Structures	NA*	< 916215.226		0	0.00E+00

UJ MC

Reviewed by:

x [signature]
Paulina Kho
Analyst

* NA - weight percent is only calculated using total structures, counting categories that are counted using primary structures are not calculated.

LabCor Portland Inc

Lab/Cor Portland, Inc.
4321 SW Corbett Ave., Ste A
Portland, OR 97239

Final Report

Phone: (503) 224-5055
Fax: (503) 228-8282
http://www.labcorpdx.net

Asbestos and Environmental Analysis

EPA 600-R-93-116 - TEM - Bulk Quantitative

Job Number: Q142958e **PDX**
Client: Alion Science and Technology
Project Name: Sumas Mtn Asbestos Soil
Project No.: 10-100614-121545-0001

Report Number: Q142958eR01
Date Received: 12/15/2014

Lab/Cor Sample No. : S10
Client Sample No. : 14394109 -
GRR : 0.326
Dilution : 0.0025
Dilution Factor : 1

As Received Weight (g) : 121.20
Lab Filter Area (mm2) : 193
Grid Openings Analyzed : 10
Average Grid Opening Area (mm2) : 0.01042
Area Analyzed (mm2) : 0.1042
Analytical Sens. (Weight Percent) : 1.22E-07
Analytical Sens. (struc/g) : 2.03E+06

Analyst(s)	Analysis Date	Microscope	Magnification
PK	2/11/2015	H-7000	20000

Structure Type	Weight Percent (%)	Concentration (struc/g)	Struct Count[1] Primary	Struct Count[1] Total	Wa
Total Asbestos Structures	1.54E-03	4.06E+06		2	1.41E-08
Total Asb & Libby-OtherAmph Structures	1.54E-03	4.06E+06		2	1.41E-08
Total Chrysotile Structures	7.65E-05	2.03E+06		1	6.98E-10

RC
IC
MC

Reviewed by:

x [signature]
Paulina Kho
Analyst

* NA - weight percent is only calculated using total structures, counting categories that are counted using primary structures are not calculated.
