# Supplementary material for: Comparison of soil sampling and analytical methods for asbestos at the Sumas Mountain Asbestos Site—Working towards a toolbox for better assessment
Source: PLoS One. 2017 Jul 31;12(7):e0180210. doi: 10.1371/journal.pone.0180210 (PMC5536314; doi:10.1371/journal.pone.0180210)
Supplement: S7 Text — (PDF) [file pone.0180210.s008.pdf]

CB&I
2700 Chandler Avenue, Building C
Las Vegas, NV 89120
Tel: +1 702 795 0515
Fax: +1 702 795 8210
www.CBI.com

July 10, 2015

Julie Wroble
EPA Site Manager Region 10
EPA Region 10
1200 6th Ave, Suite 900
Seattle, WA 98101

Document ID #: 3015-07102015-5

Dear Ms. Wroble:

EPA CONTRACT NUMBER EP-W-10-033
TASK ORDER NUMBER 3015
ASBESTOS QA SUPPORT

Enclosed please find the Release of Validated Data Report for the validation of Polarized Light Microscopy-Visual Estimation (PLM-VE) and Transmission Electron Microscopy (TEM) soil sample data, Laboratory Job Number 142959. The twenty-one (21) soil samples associated with these data were analyzed by Lab/Cor Portland, Inc., Portland, Oregon for the Sumas Mountain Asbestos Soil Project. This report and accompanying appendices are deliverables under Task 10 of the subject Task Order.

If you have any questions, please feel free to contact me.

Sincerely,

Lyndsay Gensler
Task Leader, QATS Program
CB&I Federal Services LLC
Phone: 702.895.8730
E-Mail Address: lyndsay.gensler@cbifederalservices.com

cc: Shari Myer, EPA-ASB, QATS Task Order Project Officer
Administrative Contracting Officer (letter only)

RELEASE OF VALIDATED DATA

DATE:	July 10, 2015
SUBJECT:	Review of Data for Laboratory Job Number: 142959
LABORATORY:	Lab/Cor Portland, Inc., Portland, Oregon
FROM:	Quality Assurance Technical Support (QATS) Program, Las Vegas, NV<br>CB&I Federal Services LLC
TO:	Julie Wroble, Environmental Protection Agency

QATS reviewed the data for the following case:

Applicable SAP:	NA
Chain-of-Custody Number:	10-100614-132555-0002
Method:	Transmission Electron Microscopy (TEM) by EPA 600-R-93-116 and Polarized Light Microscopy (PLM) VE by CARB 435 (with preparation by ASTM D7521).
Applicable Laboratory Modification(s):	NA
Number and Type of Samples:	21 Soil Samples
EPA Sample Numbers:	14394124, 14394125, 14394126, 14394127, 14394128, 14394129, 14394130, 14394131, 14394132, 14394133, 14394134, 14394135, 14394136, 14394137, 14394138, 14394139, 14394140, 14394141, 14394142, 14394143, 14394144.

Note that samples 14294124 through 14394127 were analyzed by TEM as indicated on the ASTM D7521 results sheet.

VALIDATION SUMMARY

Twenty-one (21) soil samples from Laboratory Job Number 142959 were collected between 09/30/2014 and 10/02/2014 and shipped to Lab/Cor Portland, Inc. in Portland, OR for PLM-VE analysis by CARB 435 (with preparation by ASTM D7521), and TEM by EPA 600-R-93-116. The samples were received at the laboratory intact on 12/15/2014, and were analyzed between 01/30/2015 and 02/03/2015 for PLM and between 02/05/2015 and 02/19/2015 for TEM.

Listed below are the Data Qualification Summary Table, EDD/Bench Sheet Discrepancy Table, Data Qualifier Table, and Reason Code Table.

DATA QUALIFICATION SUMMARY TABLE

Criteria Exceeded	EPA Sample ID	Validation Qualifier	Reason Code
TEM k-factors not performed at required frequency.	14394126	J	IC

EDD/BENCH SHEET DISCREPANCY TABLE

EPA Sample ID	C# *	Method/Matrix	Lab. Job No.	Analysis Date	Discrepancy
14394129	NA	ASTM D7521	142959	02/01/2015	The (W_M) value reported on the ASTM D7521 Total Asbestos % sheet for sample 14394129 (86.6 g) does not match the value recorded on the ASTM D7521 Prep Sheet (86.8 g).

'*' The EDD correction number in column 2. (i.e., C0, C1, C2, etc..)

DATA QUALIFIER TABLE

Qualifier	Definition
**J**	The result is estimated. The associated numerical value is an approximation.
**UJ**	The non-detect result may be inaccurate or imprecise due to the quality of the data generated because certain QC criteria were not met.
**R**	The sample results are rejected due to serious deficiencies.
**X**	Validator defined.

TEM REASON CODE TABLE

Reason Code	Definition
**MC**	Structure/fiber counts and recorded structure dimensions may be inaccurate due to improper or infrequent scope alignment and/or magnification calibrations.
**IC**	Identification by elemental composition or diffraction pattern may be inaccurate due to improper or infrequent EDXA or camera constant calibration.
**PA**	Structure/fiber counts and reported concentrations may be inaccurate due to improper or infrequent calibration of the plasma asher.
**SC**	The reported concentration may be inaccurate due to the condition of samples upon receipt at the laboratory.
**DL**	The area analyzed, structures counted, or AS do not meet the requirements specified in the applicable SAP Analytical Summary.
**ID**	The asbestos identification and concentrations may be inaccurate because the recorded structure types are not consistent with those described in the applicable TEM Method and/or laboratory modification(s).

PLM REASON CODE TABLE

Reason Code	Definition
**MC**	Reported concentrations or analyte identification may be inaccurate due to improper or infrequent scope alignment.
**IC**	Identification may be inaccurate due to improper or infrequent Refractive Index (RI) liquid calibrations.
**B**	The reported concentration may be inaccurate due to the presence of analyte structures/fibers in the associate contamination check or a contamination check was not performed daily.
**SC**	The reported concentration may be inaccurate due to the condition of samples upon receipt at the laboratory and/or improper storage prior to sample preparation and/or analysis.
**ID**	The asbestos identification and concentrations may be inaccurate because the recorded optical properties are not consistent with those described in the project-specific PLM SOPs.

VALIDATION PROCESS

The samples for Laboratory Job Number 142959 were collected from the subject site between 09/30/2014 and 10/02/2014. The samples were prepared and analyzed in accordance with CARB 435, ASTM D7521, and EPA 600-R-93-116. CB&I's Quality Assurance Technical Support (QATS) Program performed validation and a transcription check in accordance with method-specific data validation SOPs. QATS preparation of this report was performed under Technical Direction 03, Task 10, of Task Order 3015.

The sample results on bench sheets and other supporting documents provided in the hardcopy deliverables were compared to the entries in the associated laboratory method-specific EDDs (where applicable) to ensure that the reported results are complete, compliant with the specified methodology, and accurate. Additional support information provided in this data validation report include the QATS Data Review Checklist used to document the data validation process (see Appendix A); and the sample results as reported by the laboratory, with qualifiers as applicable (see Appendix B).

TEM VALIDATION SUMMARY

1. **DATA PACKAGE INVENTORY AND SAMPLE RECEIPT:** The data package included a narrative, Chain-of-Custody (COC) record, EDD files, raw data (bench sheets), and QC samples. The samples were properly packaged, sealed, undamaged, and labeled upon receipt at the laboratory. The COC record was reviewed and found to be acceptable.

2. **SAMPLE PREPARATION:** The appropriate preparation documents were provided.

3. **EQUIPMENT CALIBRATION AND PERFORMANCE CHECKS (i.e., daily microscope alignment, screen magnification, EDS calibration, and sensitivity checks):** The equipment alignment and calibration documentation provided shows that instrument alignment and calibration were performed at the correct frequency, indicating that the instruments were in proper working order during the time of sample analyses with the following exception: The TEM the k-factors provided by the laboratory were performed on 01/24/2014 for instrument (scope) H-7000, more than six months prior to the analyses of the samples in this SDG. The laboratory's QAPP states for k-factors "Calibration is performed on a biyearly basis, the first week of January and the first week of July." QATS requested the January 2015 k-factors from the laboratory; however, k factors calculated on 03/30/2015 were received. As a result of the described k-factor deficiency, one sample with an amphibole result reported in this SDG is qualified "J".

4. **ANALYTICAL SENSITIVITY:** A sufficient number of grid openings have been analyzed to achieve the required analytical sensitivity and/or the appropriate stopping rule was invoked.

5. **STRUCTURE RECORDING AND ASBESTOS IDENTIFICATION:** The structure recording and asbestos identification were found to be acceptable.

6. **BLANK ANALYSIS:** No blanks were analyzed and reported with this sample set.

7. **ANALYTICAL VARIABILITY:** The laboratory performed one recount different (RD) analysis on EPA Sample No. 14394125 and one recount same (RS) analysis on EPA Sample No. 14394124. All QC samples passed the established QC criteria.

8. **OVERALL ASSESSMENT OF DATA:** With the exception of the k-factor calibration discrepancy, the deliverable was found to be complete and accurate. The qualified sample is listed in the Data Qualification Summary Table.

REVIEWED BY: **Shellee McGrath** **DATE: 07/08/2015**

PLM VALIDATION SUMMARY

1. **DATA PACKAGE INVENTORY AND SAMPLE RECEIPT:** The data package included a narrative, Chain-of-Custody (COC) record, EDD files, raw data (bench sheets), and QC samples. The samples were properly packaged, sealed, undamaged, and labeled upon receipt at the laboratory. The COC record was reviewed and found to be acceptable.

2. **SAMPLE PREPARATION:** The appropriate preparation documents were provided.

3. **EQUIPMENT CALIBRATION AND PERFORMANCE CHECKS (i.e., daily microscope alignment, RI liquid calibration check):** The Scope ID was not documented in the NADES file; however, the analyst and dates were matched up to PLM #1. According to the NADES file the samples were analyzed on 01/30/2015, 02/01/2015, 02/02/2015, and 02/03/2015. The 142959R02_020150325 Final Carb 435.pdf file lists the analysis date for all 21 samples as 02/06/2015. For the calibration requirement, the analysis dates from the NADES file were used. The required daily microscope alignments and monthly calibration of the commonly used RI oils was performed and recorded for all dates. The QC samples were analyzed on 03/16/2015, the calibration was provided for this date on both PLM instruments.

4. **MINERAL/FIBER IDENTIFICATION:** The fiber identification and quantification were found to be acceptable.

5. **CONTAMINATION CHECK:** The appropriate daily contamination checks were performed and recorded on the Equipment Maintenance Form for all dates and were found to be acceptable.

6. **REFERENCE MATERIAL ANALYSIS (CALIBRATION STANDARDS):** The PLM Reference Material Comparison spreadsheet, PLM Accuracy QC and RTI Reference Material Comparison.xls, provided Reference Material results for analyst R. Brown. The analyst for the samples in this SDG is S. Golden; therefore, the PLM Reference Material analysis could not be evaluated.

7. **ANALYTICAL VARIABILITY:** The laboratory performed two Laboratory Duplicates (by the same analyst) on EPA Sample Nos. 14394130 and 14394143 and two Laboratory Duplicates (by a different analyst) on EPA Sample Nos. 14394126 and 14394132. All QC samples passed the established QC criteria.

8. **LABORATORY MODIFICATIONS:** NA

9. **GRAVIMETRIC ANALYSIS:** Gravimetric analysis was performed and recorded on the ASTM D7521 Prep Sheets.

9. **OVERALL ASSESSMENT OF DATA**: With the exception of the W_M value discrepancy listed in the EDD/Bench Sheet Discrepancy Table, the deliverable was found to be complete and accurate. No qualification of the data is necessary.

REVIEWED BY: Shellee McGrath DATE: 06/11/2015

Appendix A

Data Review Checklist

Data Review Checklist for the Verification and Validation of Transmission Electron Microscopy (TEM) Data Deliverables

**Project Name: Sumas Mtn Asbestos Soil Project**	**Case or Sample Set ID: 142959**
**Number of Samples/Matrix: 4 Soil Samples**	**COC Number: 10-100614-132555-0002**
**TEM Analytical Method: EPA 600-R-93-116**	**Level of Validation (Circle one): 1 2 [3] Other**

**1.0 Data Package Inventory**	**Yes**	**No**	**Comments**
1.1 Were the project-specific requirements (i.e. acceptance criteria & analytical sensitivities) provided by the client prior to the initiation of validation activities?	☐	☒	A request was made to the laboratory on 06/04/2015 for the laboratory's QAPP and/or SOPs documenting the frequency of the different TEM calibrations. The documents were received on 06/18/2015. k-factors were also requested from the laboratory.
1.2 Did the received hard copy deliverables contain all the necessary components:			
1.2.1 Case Narrative (Level 1, 2 & 3)?	☒	☐	
1.2.2 Chain-of-Custody (Level 1, 2 & 3)?	☒	☐	
1.2.3 Form I or equivalent (Level 1, 2 & 3)?	☒	☐	
1.2.4 Raw Data - Count Sheets (Level 1, 2 & 3)?	☒	☐	
1.2.5 QC Sample Data (Level 2 & 3):	☒	☐	
1.2.5.1 Blank(s)?	☐	☒	
1.2.5.2 Replicate(s)?	☒	☐	The laboratory performed one recount different (RD) analysis on EPA Sample No. 14394125 and one recount same (RS) analysis on EPA Sample No. 14394124.
1.2.5.3 Duplicate(s)?	☒	☐	
1.2.5.4 Verified Analysis?	☐	☒	
1.2.6 Calibration Data (Level 3)?	☒	☐	
1.2.7 Communication Records (Level 1, 2 & 3)?	☐	☒	
1.2.8 Miscellaneous?	☐	☐	NA
1.3 Were the necessary components received to perform the requested level of validation?	☒	☐	
**2.0 Chain-of-Custody Information Verification (Level 1, 2 & 3)**			
2.1 Were the following information recorded in the hard copy electronic deliverables (if applicable) consistent with the information recorded on the COC:			
2.1.1 COC Number?	☒	☐	
2.1.2 Case or Sample Set Number?	☒	☐	
2.1.3 EPA Sample ID?	☒	☐	
2.1.4 Date/Time Collected?	☒	☐	
2.1.5 Sample Volume?	☒	☐	
2.1.6 Sample Matrix?	☒	☐	
2.1.7 Analyses (Method)?	☒	☐	
2.1.8 Date/Time Received?	☒	☐	
2.1.9 Other (describe)?	☐	☐	
2.2 Were the COC records signed and dated upon receipt?	☒	☐	

Additional Comments:

Data Review Checklist for the Verification and Validation of Transmission Electron Microscopy (TEM) Data Deliverables

3.0 Sample Result Verification & Validation (Level 1, 2 & 3)	Yes	No	Comments
3.1 Is the sample preparation method documented and final sample volume recorded?	☒	☐	
3.2 Is the correct number of grid openings used to achieve the specified analytical sensitivity?	☒	☐	
3.3 Verify that the following information from the laboratory's bench sheets have been transcribed correctly:			
3.3.1.1 Grid identification?	☒	☐	
3.3.1.2 Grid opening?	☒	☐	
3.3.1.3 Structure type?	☒	☐	
3.3.1.4 Number of primary and secondary structures?	☒	☐	
3.3.1.5 Length and width dimensions?	☒	☐	
3.3.1.6 Structure identification?	☒	☐	
3.3.1.7 Mineral type?	☒	☐	
3.4 Are overloaded samples correctly reported to the specified percent obscuration (i.e. 10%, 25%)?	☐	☐	NA
3.5 If overloading occurs, are samples prepared by an alternate method (i.e. indirect preparation)?	☐	☐	NA
3.6 Verify that the following information is documented correctly:			
3.6.1 Magnification?	☒	☐	
3.6.2 Field or QC sample type?	☒	☐	
3.6.3 Number of grids prepared?	☒	☐	
3.6.4 Filter area in (mm^2)?	☒	☐	
3.6.5 Analysis date?	☒	☐	
3.7 Verify the totals reported on the count sheets for the various types of structures. These may include:			
3.7.1 Total EPA Structures	☒	☐	
3.7.2 PCMe Structures	☒	☐	
3.7.3 AHERA Structures	☐	☐	NA
3.7.4 Berman Crump Structures	☐	☐	NA
3.8 Are the required spectra included for all hits reported (i.e. ED, EDXA, SAED)?	☒	☐	
3.9 Recalculate the reported concentration on at least 10% of the results reported.			
3.9.1 Are the recalculated concentrations consistent with those reported?	☒	☐	

Additional Comments:

Data Review Checklist for the Verification and Validation of Transmission Electron Microscopy (TEM) Data Deliverables

4.0 Quality Control Verification & Validation (Level 2 and 3)	Yes	No	Comments
4.1 Blanks			
4.1.1 Are laboratory blanks (direct, indirect) prepared and analyzed at the required frequency?	☐	☒	
4.1.2 Are laboratory blank results within the specified criteria?	☐	☐	
4.1.2.1 If "no" then qualify the associated results in accordance with the Blank Result table in SOP QATS-70-091.			
4.2 Replicate Analyses			
4.2.1 Are replicate (second analyst on the same grids but different grid openings) sample analyses performed at the required frequency?	☒	☐	
4.2.2 Are replicate sample results within the specified acceptance limits?	☒	☐	
4.2.2.1 If "no" then qualify the associated results in accordance with the Analytical Variability Results table in SOP QATS-70-091.			The laboratory performed one recount different (RD) analysis on EPA Sample No. 14394125 and one recount same (RS) analysis on EPA Sample No. 14394124.
4.3 Duplicate Analyses			
4.3.1 Are duplicates (analysis of a second sample preparation obtained from the final filter) prepared and analyzed at the required frequency?	☒	☐	
4.3.2 Are duplicate sample results within the specified acceptance limits?	☒	☐	
4.3.2.1 If "no" then qualify the associated results in accordance with the Analytical Variability Results table in SOP QATS-70-091.			
4.4 Verified Analyses			
4.4.1 Are verified analyses (second analysis on same grids and grid openings) at the required frequency?	☐	☒	
4.4.2 Are sample verification results within the specified acceptance limits?	☐	☐	
4.4.2.1 If "no" then qualify the associated results in accordance with the Analytical Variability Results table in SOP QATS-70-091.			

Additional Comments:

Data Review Checklist for the Verification and Validation of Transmission Electron Microscopy (TEM) Data Deliverables

5.0 Calibration & Microscope Alignment Validation (Level 3)	Yes	No	Comments
5.1 Is evidence of the calibration of TEM Screen Magnification provided for all sample analyses?	☒	☐	
5.1.1 Camera Constant Calibration?	☒	☐	
5.1.2 Calibration of the EDXA System?	☒	☐	
5.1.3 k-Factors?	☒	☐	
5.2 Are the calibration checks listed above performed at the required frequencies?	☐	☒	The k-factors provided by the laboratory were performed on 01/24/2014, more than 6 months prior to the analysis of the samples in this SDG. The laboratory's QAPP states for k-factors "Calibration is performed on a biyearly basis, the first week of January and the first week of July." As a result, one TEM result in this SDG is qualified "J" due to the k-factor calibration.
5.3 Are the calibration checks within the specified criteria?	☒	☐	
5.4 Are all calibration checks traceable to the associated samples analyses?	☒	☐	
5.5 If required, are the following additional system checks provided:			
5.5.1 Beam Dose Check?	☒	☐	
5.5.2 Spot Size Check?	☒	☐	
5.5.3 Detector Resolution Check?	☒	☐	
5.5.4 Resolvable Na, Mg, and Si Peaks?	☒	☐	
5.5.5 If "no" then qualify the associated results in accordance with the Calibration Results table in SOP QATS-70-091.			
**6.0 Case Narrative Validation (Levels 2 & 3)**			
6.1 Does the data package narrative include descriptions of the following:			
6.1.1 Samples received (matrix/method)?	☒	☐	
6.1.2 Method/project requirement deviations?	☒	☐	
6.1.3 Example sample calculation?	☒	☐	
6.1.4 Laboratory blank contamination?	☐	☐	NA
6.1.5 Quality control analyses outside specified criteria?	☐	☐	NA
6.1.6 Any problems encountered and subsequent corrective action?	☐	☐	NA

Additional Comments:

Validator's Signature Shellee McGrath **Date** 07/08/2015

QA Review Lyndsay Gensler **Date** 07/08/2015

Data Review Checklist for the Verification and Validation of Polarized Light Microscopy (PLM) Data Deliverables

**Project Name: Sumas Mtn Asbestos Soil Project**	**Case or Sample Set ID: 142959**
**Number of Samples/Matrix: 21 Soil Samples**	**COC Number: 10-100614-132555-0002**
**PLM Analytical Method: CARB 435; ASTM D7521**	**Level of Validation (Circle one): 1 2 [3] Other**

**1.0 Data Package Inventory**	**Yes**	**No**	**Comments**
1.1 Were the project-specific requirements (i.e. acceptance criteria & analytical sensitivities) provided by the client prior to the initiation of validation activities?	☒	☐	
1.2 Did the received hard copy deliverables contain all the necessary components:			
1.2.1 Case Narrative (Level 1, 2 & 3)?	☒	☐	
1.2.2 Chain-of-Custody (Level 1, 2 & 3)?	☒	☐	
1.2.3 Form I or equivalent (Level 1, 2 & 3)?	☒	☐	
1.2.4 Raw Data - Count Sheets (Level 1, 2 & 3)?	☒	☐	The laboratory performed two Laboratory Duplicates (by the same analyst) on EPA Sample Nos. 14394130 and 14394143 and two Laboratory Duplicates (by a different analyst) on EPA Sample Nos. 14394126 and 14394132.
1.2.5 QC Sample Data (Level 2 & 3):	☒	☐	
1.2.5.1 Blank(s)?	☐	☒	
1.2.5.2 Replicate(s)?	☒	☐	
1.2.5.3 Duplicate(s)?	☒	☐	
1.2.6 Calibration Data (Level 3)?	☒	☐	
1.2.7 Communication Records (Level 1, 2 & 3)?	☐	☒	
1.2.8 Miscellaneous?	☐	☐	NA
1.3 Were the necessary components received to perform the requested level of validation?	☒	☐	
**2.0 Chain-of-Custody Information Verification (Level 1, 2 & 3)**			
2.1 Were the following information recorded in the hard copy electronic deliverables (if applicable) consistent with the information recorded on the COC:			
2.1.1 COC Number?	☒	☐	
2.1.2 Case or Sample Set Number?	☒	☐	
2.1.3 EPA Sample ID?	☒	☐	
2.1.4 Date/Time Collected?	☒	☐	
2.1.5 Sample Matrix?	☒	☐	
2.1.6 Analyses (Method)?	☒	☐	
2.1.7 Date/Time Received?	☒	☐	
2.1.8 Other (describe)?	☐	☐	
2.2 Were the COC records signed and dated upon receipt?	☒	☐	

Additional Comments:

Data Review Checklist for the Verification and Validation of Polarized Light Microscopy (PLM) Data Deliverables

**3.0 Sample Result Verification & Validation (Level 1, 2 & 3)**	**Yes**	**No**	**Comments**
3.1 Prior to analysis by PLM, are samples examined at low magnification using a stereoscope?	☒	☐	
3.1.1 Are the following observations recorded for each sample:			
3.1.1.1 Color?	☒	☐	
3.1.1.2 Texture?	☒	☐	
3.1.1.3 Percent (%) fibrous material?	☒	☐	
3.2 Is the technique used to prepare samples to slides recorded (i.e. particle size reduction, acid treatment, heating, melting or teasing)?	☒	☐	
3.3 Were gravimetric analysis performed?	☒	☐	ASTM D7521
3.3.1 If yes, were the necessary sample weights and tare weights recorded and provided?	☒	☐	
Using the recorded weights, recalculate a minimum of 10% of the samples for which gravimetric analysis was performed.			
3.3.1.1 Are the recalculated concentrations consistent with those reported?	☒	☐	
3.4 Is qualitative identification of fibrous materials made by examining fiber morphology and observance of optical properties?	☒	☐	.
3.4.1 Are the following recorded for all reported fibrous materials:			
3.4.1.1 Morphology?	☒	☐	
3.4.1.2 Refractive Indices?	☒	☐	
3.4.1.3 Sign of Elongation?	☒	☐	
3.4.1.4 Extinction Angle?	☒	☐	
3.4.1.5 Pleochroism?	☒	☐	
3.4.1.6 Birefringence?	☒	☐	
3.5 Do the recorded morphology and optical properties in the raw data agree with the type of fibrous material(s) reported?	☒	☐	
Note: Refer to Attachments A and B of SOP QATS-70-090 for the morphology and optical properties of various asbestos and non-asbestos fibers.			

Additional Comments:

Data Review Checklist for the Verification and Validation of Polarized Light Microscopy (PLM) Data Deliverables

3.0 Sample Result Verification & Validation (Level 1, 2 & 3)	Yes	No	Comments
3.6 Was quantitative analysis performed by point counting?	☐	☒	
3.6.1 Was the point counting performed as described in the project and/or method specified?	☐	☐	
3.6.2 Where the following recorded:			
3.6.2.1 Magnification?	☐	☐	
3.6.2.2 Graticule size/type?	☐	☐	
3.6.2.3 Number of slide mounts prepared?	☐	☐	
3.6.2.4 Empty and non-empty points counted?	☐	☐	
3.6.2.5 The observance of fibers in a field of view, but not directly under a point?	☐	☐	

4.0 Quality Control Verification & Validation (Level 2 and 3)	Yes	No	Comments
4.1 Blanks			
4.1.1 Are laboratory contamination blanks prepared and analyzed at the required frequency?	☐	☐	NA
4.1.2 Are laboratory blank results within the specified criteria?	☐	☐	
4.1.2.1 If "no" then qualify the associated results in accordance with the Blank Result table in SOP QATS-70-090.			
4.2 Replicate Analyses			
4.2.1 Are replicate (reanalyzed by the same or second analyst) sample analyses performed at the required frequency?	☒	☐	
4.2.2 Are replicate sample results within the specified acceptance limits?	☒	☐	The laboratory performed two Laboratory Duplicates (by the same analyst) on EPA Sample Nos. 14394130 and 14394143 and two Laboratory Duplicates (by a different analyst) on EPA Sample Nos. 14394126 and 14394132.
4.3 Duplicate Analyses			
4.3.1 Are duplicate sample analyses performed at the required frequency?	☒	☐	
4.3.2 Are duplicate sample results within the specified acceptance limits?	☒	☐	
4.3.2.1 If "no" then qualify the associated results in accordance with the Analytical Variability Results table in SOP QATS-70-090.			

Additional Comments:

Data Review Checklist for the Verification and Validation of Polarized Light Microscopy (PLM) Data Deliverables

4.0 Quality Control Verification & Validation (Level 2 and 3)	Yes	No	Comments
4.4 Reference Slide Analysis (if applicable)			The PLM Reference Material Comparison spreadsheet, PLM Accuracy QC and RTI Reference Material Comparison.xls, provided Reference Material results for analyst R. Brown. The analyst for the samples in this SDG is S. Golden; therefore, the PLM Reference Material analysis could not be evaluated.
4.4.1 Are reference slide analyses performed at the required frequency?	☒	☐	
4.4.2 Are the reference slide analyses results within the specified acceptance criteria?	☒	☐	
4.4.2.1 If "no" then qualify the associated results in accordance with the Reference Slide Analysis table in SOP QATS-70-090.			
**5.0 Calibration & Microscope Alignment Validation (Level 3)**			
5.1 Are evidence of microscope alignment and Refractive Index (RI) liquid calibration provided for all sample analyses?	☒	☐	
5.1.1 Microscope-specific alignment checks?	☒	☐	
5.1.2 Microscope-specific contamination checks?	☒	☐	
5.1.3 Calibration RI liquids?	☒	☐	
5.2 Are alignment and calibration checks listed above performed at the required frequencies?	☒	☐	
5.3 Are alignment and calibration checks within the specified criteria?	☒	☐	
5.4 Are all alignment and calibration checks traceable to the associated samples analyses?	☒	☐	
5.4.1 If "no" then qualify the associated results in accordance with the Calibration Results table in SOP QATS-70-090.			
**6.0 Case Narrative Validation (Levels 2 & 3)**			
6.1 Does the data package narrative include descriptions of the following:			
6.1.1 Samples received (matrix/method)?	☒	☐	
6.1.2 Method/project requirement deviations?	☒	☐	
6.1.3 Example sample calculation?	☒	☐	
6.1.4 Laboratory blank contamination?	☐	☐	NA
6.1.5 Quality control analyses outside specified criteria?	☐	☐	NA
6.1.6 Any problems encountered and subsequent corrective action?	☐	☐	NA

Additional Comments:

Validator's Signature Shellee McGrath **Date** 06/11/2015

QA Review Lyndsay Gensler **Date** 06/19/2015

Appendix B

Qualified Result Forms

Suma

National Asbestos Data Entry Spreadsheet (N

Site or Project Name: Sumas Mtn Asbestos Soil
Site/Project Identifier Code: SFP-078A
Date received by lab: 12/15/14
Chain of Custody Number: -132555-0002

State/Federal Site No or Project Identifier: SFP-078A

WARNING: The current file will be saved and Microsoft Excel will close as part of the export process.

Lab Name: Lab/Cor Portland, Inc.
Lab Job No: 142959
Method: SRC-Libby-03_VE

Data Entry by: S. Golden
Data Entry Date: 03/18/15
QA by: K. March
QA Date: 03/29/15

Client Sample ID	Sample Type	Index Suffix Char.	Index Suffix No.	QA Type (a)	Lab Sample ID	Date Analyzed	Analyst Name	Sample Appearance	Ref Material	Base Mineral Type of Reference Material (b)	Actinolite (AC)			Amosite (AM)			Anthophylli	
											Qual	AC-AF (%)	AC-MF (%)	Qual	AM-AF (%)	AM-MF (%)	Qual	AN-AF (%)
14394124	soil			Not QA	142959-S1	1/30/2015	S. Golden	Coarse, Medium, Fine Grain			ND	0	0	ND	0	0	ND	0
14394125	soil			Not QA	142959-S2	1/30/2015	S. Golden	Coarse, Medium, Fine Grain			ND	0	0	ND	0	0	ND	0
14394126	soil			Not QA	142959-S3	1/30/2015	S. Golden	Coarse, Medium, Fine Grain			ND	0	0	ND	0	0	ND	0
14394127	soil			Not QA	142959-S4	1/30/2015	S. Golden	Coarse, Medium, Fine Grain			ND	0	0	ND	0	0	ND	0
14394128	soil			Not QA	142959-S5	2/1/2015	S. Golden	Coarse, Medium, Fine Grain			ND	0	0	ND	0	0	ND	0
14394129	soil			Not QA	142959-S6	2/1/2015	S. Golden	Coarse, Medium, Fine Grain			ND	0	0	ND	0	0	ND	0
14394130	soil			Not QA	142959-S7	2/1/2015	S. Golden	Coarse, Medium, Fine Grain			ND	0	0	ND	0	0	ND	0
14394131	soil			Not QA	142959-S8	2/1/2015	S. Golden	Coarse, Medium, Fine Grain			ND	0	0	ND	0	0	ND	0
14394132	soil			Not QA	142959-S9	2/1/2015	S. Golden	Coarse, Medium, Fine Grain			ND	0	0	ND	0	0	ND	0
14394133	soil			Not QA	142959-S10	2/2/2015	S. Golden	Coarse, Medium, Fine Grain			ND	0	0	ND	0	0	ND	0
14394134	soil			Not QA	142959-S11	2/2/2015	S. Golden	Coarse, Medium, Fine Grain			ND	0	0	ND	0	0	ND	0
14394135	soil			Not QA	142959-S12	2/2/2015	S. Golden	Coarse, Medium, Fine Grain			ND	0	0	ND	0	0	ND	0
14394136	soil			Not QA	142959-S13	2/2/2015	S. Golden	Coarse, Medium, Fine Grain			ND	0	0	ND	0	0	ND	0
14394137	soil			Not QA	142959-S14	2/2/2015	S. Golden	Coarse, Medium, Fine Grain			ND	0	0	ND	0	0	ND	0
14394138	soil			Not QA	142959-S15	2/2/2015	S. Golden	Coarse, Medium, Fine Grain			ND	0	0	ND	0	0	ND	0
14394139	soil			Not QA	142959-S16	2/2/2015	S. Golden	Coarse, Medium, Fine Grain			ND	0	0	ND	0	0	ND	0
14394140	soil			Not QA	142959-S17	2/3/2015	S. Golden	Coarse, Medium, Fine Grain			ND	0	0	ND	0	0	ND	0
14394141	soil			Not QA	142959-S18	2/3/2015	S. Golden	Coarse, Medium, Fine Grain			ND	0	0	ND	0	0	ND	0
14394142	soil			Not QA	142959-S19	2/3/2015	S. Golden	Coarse, Medium, Fine Grain			ND	0	0	ND	0	0	ND	0
14394143	soil			Not QA	142959-S20	2/3/2015	S. Golden	Coarse, Medium, Fine Grain			ND	0	0	ND	0	0	ND	0
14394144	soil			Not QA	142959-S21	2/3/2015	S. Golden	Coarse, Medium, Fine Grain			ND	0	0	ND	0	0	ND	0

ıs Mtn Asbestos Soil
ɅADES) for Bulk & Soil Analysis by PLM Visual Estimation

te (AN)	Chrysotile (CH)			Crocidolite (CR)			Tremolite (TR)			winchite/richterite/ tremolite/actinolite (WRTA)			Other Amphibole (OA)			Non-asbestos Material (NAM)			Other Mineral Type (OM)				
AN-MF (%)	Qual	CH-AF (%)	CH-MF (%)	Qual	CR-AF (%)	CR-MF (%)	Qual	TR-AF (%)	TR-MF (%)	Qual	WRTA-AF (%)	WRTA-MF (%)	Qual	OA-AF(%)	OA-MF(%)	Qual	NAM-AF (%)	NAM-MF (%)	Qual	OM-AF (%)	OM-MF (%)	OM Type	Deviation?
0	ND	0	0	ND	0	0	ND	0	0	ND	0	0	ND	0	0	ND	0	0	ND	0	0		
0	ND	0	0	ND	0	0	ND	0	0	ND	0	0	ND	0	0	ND	0	0	ND	0	0		
0	ND	0	0	ND	0	0	ND	0	0	ND	0	0	ND	0	0	ND	0	0	ND	0	0		
0	ND	0	0	ND	0	0	ND	0	0	ND	0	0	ND	0	0	ND	0	0	ND	0	0		
0		5.242		ND	0	0	ND	0	0	ND	0	0	ND	0	0	ND	0	0	ND	0	0		Used [illegible]
0		5.4895		ND	0	0	ND	0	0	ND	0	0	ND	0	0	ND	0	0	ND	0	0		Used [illegible]
0		4.5176		ND	0	0	ND	0	0	ND	0	0	ND	0	0	ND	0	0	ND	0	0		Used [illegible]
0		6.4279		ND	0	0	ND	0	0	ND	0	0	ND	0	0	ND	0	0	ND	0	0		Used [illegible]
0		6.4391		ND	0	0	ND	0	0	ND	0	0	ND	0	0	ND	0	0	ND	0	0		Used [illegible]
0		3.9793		ND	0	0	ND	0	0	ND	0	0	ND	0	0	ND	0	0	ND	0	0		Used [illegible]
0		3		ND	0	0	ND	0	0	ND	0	0	ND	0	0	ND	0	0	ND	0	0		Used [illegible]
0		5.3776		ND	0	0	ND	0	0	ND	0	0	ND	0	0	ND	0	0	ND	0	0		Used [illegible]
0		4.3626		ND	0	0	ND	0	0	ND	0	0	ND	0	0	ND	0	0	ND	0	0		Used [illegible]
0		1.8321		ND	0	0	ND	0	0	ND	0	0	ND	0	0	ND	0	0	ND	0	0		Used [illegible]
0		2.6356		ND	0	0	ND	0	0	ND	0	0	ND	0	0	ND	0	0	ND	0	0		Used [illegible]
0		1.8116		ND	0	0	ND	0	0	ND	0	0	ND	0	0	ND	0	0	ND	0	0		Used [illegible]
0		2.5036		ND	0	0	ND	0	0	ND	0	0	ND	0	0	ND	0	0	ND	0	0		Used [illegible]
0		2.5531		ND	0	0	ND	0	0	ND	0	0	ND	0	0	ND	0	0	ND	0	0		Used [illegible]
0		2.8681		ND	0	0	ND	0	0	ND	0	0	ND	0	0	ND	0	0	ND	0	0		Used [illegible]
0		1.8011		ND	0	0	ND	0	0	ND	0	0	ND	0	0	ND	0	0	ND	0	0		Used [illegible]
0		2.6876		ND	0	0	ND	0	0	ND	0	0	ND	0	0	ND	0	0	ND	0	0		Used [illegible]

Lab/Cor Portland, Inc.

Customer Name: Alion Science and Technology
Cust. Project Name: Sumas Mtn Asbestos Soil
Cust. Project Number: 10-100614-132555-0002
LCP Job Number: 142959

Total Asbestos (%)=

$$\frac{[\%_F PLM\ PC * W_F]+[\%_M PLM * W_M]+[\%_C PLM * W_C]}{W_F + W_M + W_C}$$

		Fine Fraction		Medium Fraction		Coarse Fraction		Total %
Client Sample ID	Sample Numbe	W_F	PC	W_M	PLM	W_C	PLM	
14394124	S1	8	0	97.4	0	151.4	0	0.0000
14394125	S2	6.8	0	84.2	0	77.4	0	0.0000
14394126	S3	6	0	85.6	0	63	0	0.0000
14394127	S4	7	0	107.4	0	89.6	0	0.0000
14394128	S5	1.8	5	55.8	4	92	6	5.2420
14394129	S6	2.4	4	86.6	6	83	5	5.4895
14394130	S7	4.8	4	94	5	82.8	4	4.5176
14394131	S8	5.4	6	91.4	6	72.4	7	6.4279
14394132	S9	3	4	94.2	6	86.8	7	6.4391
14394133	S10	0.8	2	50.8	4	25.8	4	3.9793
14394134	S11	0.8	3	67.4	3	38	3	3.0000
14394135	S12	0.8	5	60.2	5	37	6	5.3776
14394136	S13	28	3	295.6	5	63.6	2	4.3626
14394137	S14	24.8	2	305.2	2	66.6	1	1.8321
14394138	S15	27.4	3	312.8	3	75.8	1	2.6356
14394139	S16	16.6	2	245.4	2	60.8	1	1.8116
14394140	S17	28	3	320	3	69	0	2.5036 **
14394141	S18	32.4	3	342.4	3	65.6	0	2.5531 **
14394142	S19	34.2	3	251.4	3	43.4	2	2.8681
14394143	S20	29.8	3	269.6	2	49.6	0	1.8011 **
14394144	S21	37	3	328.2	3	67.6	1	2.6876

* Indicates samples that were sent to TEM Analysis
** Indicates trace (<1%) asbestos by PLM

LabCor Portland Inc
Lab/Cor Portland, Inc.
4321 SW Corbett Ave., Ste A
Portland, OR 97239

Phone: (503) 224-5055
Fax: (503) 228-8282
http://www.labcorpdx.net

Final Report

Asbestos and Environmental Analysis

EPA 600-R-93-116 - TEM - Bulk Quantitative

Job Number: 142959 **PDX**
Client: Alion Science and Technology
Project Name: Sumas Mtn Asbestos Soil
Project No.: 10-100614-132555-0002

Report Number: 142959R02
Date Received: 12/15/2014

Lab/Cor Sample No. : S1
Client Sample No. : 14394124 -
GRR : 0.422
Dilution : 0.0025
Dilution Factor : 1

As Received Weight (g) : 256.80
Lab Filter Area (mm2) : 193
Grid Openings Analyzed : 10
Average Grid Opening Area (mm2) : 0.01042
Area Analyzed (mm2) : 0.1042
Analytical Sens. (Weight Percent) : 1.13E-07
Analytical Sens. (struc/g) : 1.88E+06

Analyst(s)	Analysis Date	Microscope	Magnification
TH	2/5/2015	H-7000	20000

Structure Type	Weight Percent (%)	Concentration (struc/g)	Struct Count[1] Primary	Total	Wa
Total Asbestos Structures	2.96E-03	1.88E+06		1	2.92E-08
Total Asb & Libby-OtherAmph Structures	2.96E-03	1.88E+06		1	2.92E-08
Total Chrysotile Structures	2.96E-03	1.88E+06		1	2.92E-08

Lab/Cor Sample No. : S2
Client Sample No. : 14394125 -
GRR : 0.288
Dilution : 0.0025
Dilution Factor : 1

As Received Weight (g) : 169.00
Lab Filter Area (mm2) : 193
Grid Openings Analyzed : 10
Average Grid Opening Area (mm2) : 0.01042
Area Analyzed (mm2) : 0.1042
Analytical Sens. (Weight Percent) : 7.56E-08
Analytical Sens. (struc/g) : 1.26E+06

Analyst(s)	Analysis Date	Microscope	Magnification
TH	2/5/2015	H-7000	20000

Structure Type	Weight Percent (%)	Concentration (struc/g)	Struct Count[1] Primary	Total	Wa
Total Asbestos Structures	7.79E-05	2.51E+06		2	1.15E-09
Total Asb & Libby-OtherAmph Structures	7.79E-05	2.51E+06		2	1.15E-09
Total Chrysotile Structures	7.79E-05	2.51E+06		2	1.15E-09

Lab/Cor Sample No. : S3
Client Sample No. : 14394126 -
GRR : 0.309
Dilution : 0.0025
Dilution Factor : 1

As Received Weight (g) : 154.40
Lab Filter Area (mm2) : 193
Grid Openings Analyzed : 10
Average Grid Opening Area (mm2) : 0.01042
Area Analyzed (mm2) : 0.1042
Analytical Sens. (Weight Percent) : 1.10E-07
Analytical Sens. (struc/g) : 1.83E+06

Analyst(s)	Analysis Date	Microscope	Magnification
TH	2/5/2015	H-7000	20000

Structure Type	Weight Percent (%)	Concentration (struc/g)	Struct Count[1] Primary	Total	Wa
Total Asbestos Structures	4.24E-02	3.66E+06		2	4.29E-07
Total Asb & Libby-OtherAmph Structures	4.24E-02	3.66E+06		2	4.29E-07
Total Chrysotile Structures	3.75E-02	1.83E+06		1	3.80E-07

* NA - weight percent is only calculated using total structures, counting categories that are counted using primary structures are not calculated.

LabCor Portland Inc.

Lab/Cor Portland, Inc.
4321 SW Corbett Ave., Ste A
Portland, OR 97239

Final Report

Phone: (503) 224-5055
Fax: (503) 228-8282
http://www.labcorpdx.net

Asbestos and Environmental Analysis

EPA 600-R-93-116 - TEM - Bulk Quantitative

Job Number: 142959 **PDX**
Client: Alion Science and Technology
Project Name: Sumas Mtn Asbestos Soil
Project No.: 10-100614-132555-0002

Report Number: 142959R02
Date Received: 12/15/2014

Lab/Cor Sample No. : S4
Client Sample No. : 14394127 -
GRR : 0.213
Dilution : 0.0025
Dilution Factor : 1

As Received Weight (g) : 204.80
Lab Filter Area (mm2) : 193
Grid Openings Analyzed : 10
Average Grid Opening Area (mm2) : 0.01042
Area Analyzed (mm2) : 0.1042
Analytical Sens. (Weight Percent) : 3.94E-08
Analytical Sens. (struc/g) : 6.55E+05

Analyst(s)	Analysis Date	Microscope	Magnification
TH	2/5/2015	H-7000	20000

Structure Type	Weight Percent (%)	Concentration (struc/g)	Struct Count[1] Primary	Struct Count[1] Total	Wa
Total Asbestos Structures	1.69E-03	6.55E+05		1	4.77E-08
Total Asb & Libby-OtherAmph Structures	1.69E-03	6.55E+05		1	4.77E-08
Total Chrysotile Structures	1.69E-03	6.55E+05		1	4.77E-08

Reviewed by:

x [signature]

Stephanie Golden
Technical Manager

* NA - weight percent is only calculated using total structures, counting categories that are counted using primary structures are not calculated.

LabCor Portland Inc
Lab/Cor Portland, Inc.
4321 SW Corbett Ave., Ste A
Portland, OR 97239

Final Report

Phone: (503) 224-5055
Fax: (503) 228-8282
http://www.labcorpdx.net

Asbestos and Environmental Analysis

EPA 600-R-93-116 - TEM - Bulk Quantitative

Job Number: Q142959a **PDX**
Client: Alion Science and Technology
Project Name: Sumas Mtn Asbestos Soil
Project No.: 10-100614-132555-0002

Report Number: Q142959aR01
Date Received: 12/15/2014

Lab/Cor Sample No. : S1
Client Sample No. : 14394124 -
GRR : 0.422
Dilution : 0.0025
Dilution Factor : 1

As Received Weight (g) : 256.80
Lab Filter Area (mm2) : 193
Grid Openings Analyzed : 10
Average Grid Opening Area (mm2) : 0.01042
Area Analyzed (mm2) : 0.1042
Analytical Sens. (Weight Percent) : 1.13E-07
Analytical Sens. (struc/g) : 1.88E+06

Analyst(s)	Analysis Date	Microscope	Magnification
TH	2/19/2015	H-7000	20000

Structure Type	Weight Percent (%)	Concentration (struc/g)	Struct Count[1] Primary	Total	Wa
Total Asbestos Structures	2.42E-03	1.88E+06		1	2.39E-08
Total Asb & Libby-OtherAmph Structures	2.42E-03	1.88E+06		1	2.39E-08
Total Chrysotile Structures	2.42E-03	1.88E+06		1	2.39E-08

Reviewed by:

x
Tracy Handrich
Analyst

* NA - weight percent is only calculated using total structures, counting categories that are counted using primary structures are not calculated.

LabCor Portland Inc

Lab/Cor Portland, Inc.
4321 SW Corbett Ave., Ste A
Portland, OR 97239

Final Report

Phone: (503) 224-5055
Fax: (503) 228-8282
http://www.labcorpdx.net

Asbestos and Environmental Analysis

EPA 600-R-93-116 - TEM - Bulk Quantitative

Job Number: Q142959b PDX
Client: Alion Science and Technology
Project Name: Sumas Mtn Asbestos Soil
Project No.: 10-100614-132555-0002

Report Number: Q142959bR01
Date Received: 12/15/2014

Lab/Cor Sample No. : S2
Client Sample No. : 14394125 -
GRR : 0.288
Dilution : 0.0025
Dilution Factor : 1

As Received Weight (g) : 169.00
Lab Filter Area (mm2) : 193
Grid Openings Analyzed : 10
Average Grid Opening Area (mm2) : 0.01042
Area Analyzed (mm2) : 0.1042
Analytical Sens. (Weight Percent) : 7.56E-08
Analytical Sens. (struc/g) : 1.26E+06

Analyst(s)	Analysis Date	Microscope	Magnification
PK	2/16/2015	H-7000	20000

Structure Type	Weight Percent (%)	Concentration (struc/g)	Struct Count¹ Primary	Struct Count¹ Total	Wa
Total Asbestos Structures	2.58E+00	6.29E+06		5	3.79E-05
Total Asb & Libby-OtherAmph Structures	2.58E+00	6.29E+06		5	3.79E-05
Total Chrysotile Structures	2.58E+00	6.29E+06		5	3.79E-05

Reviewed by:

x [signature]
Paulina Kho
Analyst

* NA - weight percent is only calculated using total structures, counting categories that are counted using primary structures are not calculated.
