# Supplementary material for: Comparison of soil sampling and analytical methods for asbestos at the Sumas Mountain Asbestos Site—Working towards a toolbox for better assessment
Source: PLoS One. 2017 Jul 31;12(7):e0180210. doi: 10.1371/journal.pone.0180210 (PMC5536314; doi:10.1371/journal.pone.0180210)
Supplement: S9 Text — (PDF) [file pone.0180210.s010.pdf]

CB&I
2700 Chandler Avenue, Building C
Las Vegas, NV 89120
Tel: +1 702 795 0515
Fax: +1 702 795 8210
www.CBI.com

July 10, 2015

Julie Wroble
EPA Site Manager Region 10
EPA Region 10
1200 6th Ave, Suite 900
Seattle, WA 98101

Document ID #: 3015-07102015-3

Dear Ms. Wroble:

EPA CONTRACT NUMBER EP-W-10-033
TASK ORDER NUMBER 3015
ASBESTOS QA SUPPORT

Enclosed please find the Release of Validated Data Report for the validation of Polarized Light Microscopy-Point Count (PLM-PC) soil sample data, Laboratory Job Number 142961. The twenty-one (21) soil samples associated with these data were analyzed by Lab/Cor Portland, Inc., Portland, Oregon for the Sumas Mountain Asbestos Soil Project. This report and accompanying appendices are deliverables under Task 10 of the subject Task Order.

If you have any questions, please feel free to contact me.

Sincerely,

Lyndsay Gensler
Task Leader, QATS Program
CB&I Federal Services LLC
Phone: 702.895.8730
E-Mail Address: lyndsay.gensler@cbifederalservices.com

cc: Shari Myer, EPA-ASB, QATS Task Order Project Officer
Administrative Contracting Officer (letter only)

RELEASE OF VALIDATED DATA

DATE: July 10, 2015

SUBJECT: Review of Data for Laboratory Job Number: 142961

LABORATORY: Lab/Cor Portland, Inc., Portland, Oregon

FROM: Quality Assurance Technical Support (QATS) Program, Las Vegas, NV
CB&I Federal Services LLC

TO: Julie Wroble, Environmental Protection Agency

QATS reviewed the data for the following case:

Applicable SAP: NA

Chain-of-Custody Number: 10-100614-133206-0004

Method: Polarized Light Microscopy (PLM) Point Count (PC) by CARB 435.

Applicable Laboratory Modification(s): NA

Number and Type of Samples: 21 Soil Samples

EPA Sample Numbers: 14394124, 14394125, 14394126, 14394127, 14394128, 14394129, 14394130, 14394131, 14394132, 14394133, 14394134, 14394135, 14394136, 14394137, 14394138, 14394139, 14394140, 14394141, 14394142, 14394143, 14394144.

VALIDATION SUMMARY

Twenty-one (21) soil samples from Laboratory Job Number 142961, were collected between 09/30/2014 and 10/02/2014 and shipped to Lab/Cor Portland, Inc. in Portland, OR for PLM-PC analysis by CARB 435. The samples were received at the laboratory intact on 12/15/2014, and were analyzed between 02/03/2015 and 03/25/2015.

Listed below are the Data Qualification Summary Table, EDD/Bench Sheet Discrepancy Table, Data Qualifier Table, and Reason Code Table.

DATA QUALIFICATION SUMMARY TABLE

Criteria Exceeded	EPA Sample ID	Validation Qualifier	Reason Code
None			

EDD/BENCH SHEET DISCREPANCY TABLE

EPA Sample ID	C# *	Method/Matrix	Lab. Job No.	Analysis Date	Discrepancy
14394128<br>14394129	R02	PLM PC/Soil	142961	02/04/2015	The date analyzed for the two samples was incorrectly entered into the NADES file as 02/07/2015. The date analyzed on the Point Counting Worksheet is 02/04/2015. Note that a daily calibration was not performed on 02/07/2015 and was performed on 02/04/2015.

'*' The EDD correction number in column 2. (i.e., C0, C1, C2, etc..)

DATA QUALIFIER TABLE

Qualifier	Definition
**J**	The result is estimated. The associated numerical value is an approximation.
**UJ**	The non-detect result may be inaccurate or imprecise due to the quality of the data generated because certain QC criteria were not met.
**R**	The sample results are rejected due to serious deficiencies.
**X**	Validator defined.

PLM REASON CODE TABLE

Reason Code	Definition
**MC**	Reported concentrations or analyte identification may be inaccurate due to improper or infrequent scope alignment.
**IC**	Identification may be inaccurate due to improper or infrequent Refractive Index (RI) liquid calibrations.
**B**	The reported concentration may be inaccurate due to the presence of analyte structures/fibers in the associate contamination check or a contamination check was not performed daily.
**SC**	The reported concentration may be inaccurate due to the condition of samples upon receipt at the laboratory and/or improper storage prior to sample preparation and/or analysis.
**ID**	The asbestos identification and concentrations may be inaccurate because the recorded optical properties are not consistent with those described in the project-specific PLM SOPs.

VALIDATION PROCESS

The samples for Laboratory Job Number 142961 were collected from the subject site between 09/30/2014 and 10/02/2014. All samples were prepared and analyzed in accordance with the CARB 435 Method. CB&I's Quality Assurance Technical Support (QATS) Program performed validation and a transcription check in accordance with method-specific data validation SOPs. QATS preparation of this report was performed under Technical Direction 03, Task 10, of Task Order 3015.

The sample results on bench sheets and other supporting documents provided in the hardcopy deliverables were compared to the entries in the associated laboratory method-specific EDDs (where applicable) to ensure that the reported results are complete, compliant with the specified methodology, and accurate. Additional support information provided in this data validation report include the QATS Data Review Checklist used to document the data validation process (see Appendix A); and the sample results as reported by the laboratory, with qualifiers as applicable (see Appendix B).

PLM VALIDATION SUMMARY

1. **DATA PACKAGE INVENTORY AND SAMPLE RECEIPT:** The data package included a narrative, Chain-of-Custody (COC) record, EDD files and QC samples. The raw data (bench sheets) were not provided with the original submission of the data. The point count worksheets were requested on 06/16/2015 and received on 06/19/2015. The samples were properly packaged, sealed, undamaged, and labeled upon receipt at the laboratory. The COC record was reviewed and found to be acceptable.

2. **SAMPLE PREPARATION:** The appropriate preparation documents were provided.

3. **EQUIPMENT CALIBRATION AND PERFORMANCE CHECKS (i.e., daily microscope alignment, RI liquid calibration check):** The Scope ID was not documented in the NADES file; however, the analyst and dates were matched up to PLM #1 for S. Golden and PLM #2 for R. Brown. According to the NADES file, the samples were analyzed on 02/03/2015, 02/04/2015, and 02/05/2015. The required daily microscope alignments and monthly calibration of the commonly used RI oils was performed and recorded for all dates. The QC samples were analyzed on 02/12/2015, 03/19/2015, and 03/25/2015, the calibration was provided for these dates on both PLM instruments.

4. **MINERAL/FIBER IDENTIFICATION:** The fiber identification and quantification were found to be acceptable.

5. **CONTAMINATION CHECK:** The appropriate daily contamination checks were performed and recorded on the Equipment Maintenance Form for all dates and were found to be acceptable.

6. **REFERENCE MATERIAL ANALYSIS (CALIBRATION STANDARDS):** The PLM Reference Material Comparison spreadsheet, PLM Accuracy QC and RTI Reference Material Comparison.xls, provided Reference Material results for analyst R. Brown. The analyst for some of the samples in this SDG is S. Golden; therefore, the PLM Reference Material analysis could not be evaluated for all analysts.

7. **ANALYTICAL VARIABILITY:** The laboratory performed two Laboratory Duplicates (by the same analyst) on EPA Sample Nos. 14394133 and 14394127 and four Laboratory Duplicates (by a different analyst) on EPA Sample Nos. 14394130, 14394134, 14394136, and 14394138. All QC samples passed the established QC criteria.

8. **LABORATORY MODIFICATIONS:** NA

9. **GRAVIMETRIC ANALYSIS:** NA

10. **OVERALL ASSESSMENT OF DATA:** With the exception of the discrepancy described in the EDD/Bench Sheet Discrepancy Table, the deliverable was found to be complete and accurate. No qualification of the data is necessary.

REVIEWED BY: Shellee McGrath DATE: 06/19/2015

Appendix A

Data Review Checklist

Data Review Checklist for the Verification and Validation of Polarized Light Microscopy (PLM) Data Deliverables

**Project Name: Sumas Mtn Asbestos Soil Project**	**Case or Sample Set ID: 142961**
**Number of Samples/Matrix: 21 Soil Samples**	**COC Number: 10-100614-133206-0004**
**PLM Analytical Method: CARB 435 Point Count**	**Level of Validation (Circle one): 1 2 3 Other**

**1.0 Data Package Inventory**	**Yes**	**No**	**Comments**
1.1 Were the project-specific requirements (i.e. acceptance criteria & analytical sensitivities) provided by the client prior to the initiation of validation activities?	☒	☐	The laboratory performed two Laboratory Duplicates (by the same analyst) on EPA Sample Nos. 14394133 and 14394127 and four Laboratory Duplicates (by a different analyst) on EPA Sample Nos. 14394130, 14394134, 14394136, and 14394138.
1.2 Did the received hard copy deliverables contain all the necessary components:			
1.2.1 Case Narrative (Level 1, 2 & 3)?	☒	☐	
1.2.2 Chain-of-Custody (Level 1, 2 & 3)?	☒	☐	
1.2.3 Form I or equivalent (Level 1, 2 & 3)?	☒	☐	
1.2.4 Raw Data - Count Sheets (Level 1, 2 & 3)?	☒	☐	
1.2.5 QC Sample Data (Level 2 & 3):	☒	☐	NA
1.2.5.1 Blank(s)?	☐	☒	
1.2.5.2 Replicate(s)?	☒	☐	
1.2.5.3 Duplicate(s)?	☒	☐	
1.2.6 Calibration Data (Level 3)?	☒	☐	The point count worksheets were not provided with the original deliverable. They were requested from the laboratory on 06/16/2015 and received on 06/19/2015.
1.2.7 Communication Records (Level 1, 2 & 3)?	☐	☒	
1.2.8 Miscellaneous?	☐	☐	
1.3 Were the necessary components received to perform the requested level of validation?	☒	☐	
**2.0 Chain-of-Custody Information Verification (Level 1, 2 & 3)**			
2.1 Were the following information recorded in the hard copy electronic deliverables (if applicable) consistent with the information recorded on the COC:			
2.1.1 COC Number?	☒	☐	
2.1.2 Case or Sample Set Number?	☒	☐	
2.1.3 EPA Sample ID?	☒	☐	
2.1.4 Date/Time Collected?	☒	☐	
2.1.5 Sample Matrix?	☒	☐	
2.1.6 Analyses (Method)?	☒	☐	
2.1.7 Date/Time Received?	☒	☐	
2.1.8 Other (describe)?	☐	☐	
2.2 Were the COC records signed and dated upon receipt?	☒	☐	

Additional Comments:

Data Review Checklist for the Verification and Validation of Polarized Light Microscopy (PLM) Data Deliverables

3.0 Sample Result Verification & Validation (Level 1, 2 & 3)	Yes	No	Comments
3.1 Prior to analysis by PLM, are samples examined at low magnification using a stereoscope?	☒	☐	
3.1.1 Are the following observations recorded for each sample:			
3.1.1.1 Color?	☒	☐	
3.1.1.2 Texture?	☒	☐	
3.1.1.3 Percent (%) fibrous material?	☒	☐	
3.2 Is the technique used to prepare samples to slides recorded (i.e. particle size reduction, acid treatment, heating, melting or teasing)?	☒	☐	
3.3 Were gravimetric analysis performed?	☐	☒	
3.3.1 If yes, were the necessary sample weights and tare weights recorded and provided?	☐	☐	
Using the recorded weights, recalculate a minimum of 10% of the samples for which gravimetric analysis was performed.			
3.3.1.1 Are the recalculated concentrations consistent with those reported?	☐	☐	
3.4 Is qualitative identification of fibrous materials made by examining fiber morphology and observance of optical properties?	☒	☐	.
3.4.1 Are the following recorded for all reported fibrous materials:			
3.4.1.1 Morphology?	☒	☐	
3.4.1.2 Refractive Indices?	☒	☐	
3.4.1.3 Sign of Elongation?	☒	☐	
3.4.1.4 Extinction Angle?	☒	☐	
3.4.1.5 Pleochroism?	☒	☐	
3.4.1.6 Birefringence?	☒	☐	
3.5 Do the recorded morphology and optical properties in the raw data agree with the type of fibrous material(s) reported?	☒	☐	
Note: Refer to Attachments A and B of SOP QATS-70-090 for the morphology and optical properties of various asbestos and non-asbestos fibers.			

Additional Comments:

Data Review Checklist for the Verification and Validation of Polarized Light Microscopy (PLM) Data Deliverables

3.0 Sample Result Verification & Validation (Level 1, 2 & 3)	Yes	No	Comments
3.6 Was quantitative analysis performed by point counting?	☒	☐	
3.6.1 Was the point counting performed as described in the project and/or method specified?	☒	☐	
3.6.2 Where the following recorded:			
3.6.2.1 Magnification?	☒	☐	
3.6.2.2 Graticule size/type?	☒	☐	
3.6.2.3 Number of slide mounts prepared?	☒	☐	
3.6.2.4 Empty and non-empty points counted?	☒	☐	
3.6.2.5 The observance of fibers in a field of view, but not directly under a point?	☒	☐	

4.0 Quality Control Verification & Validation (Level 2 and 3)	Yes	No	Comments
4.1 Blanks			
4.1.1 Are laboratory contamination blanks prepared and analyzed at the required frequency?	☐	☐	NA
4.1.2 Are laboratory blank results within the specified criteria?	☐	☐	
4.1.2.1 If "no" then qualify the associated results in accordance with the Blank Result table in SOP QATS-70-090.			
4.2 Replicate Analyses			
4.2.1 Are replicate (reanalyzed by the same or second analyst) sample analyses performed at the required frequency?	☒	☐	The laboratory performed two Laboratory Duplicates (by the same analyst) on EPA Sample Nos. 14394133 and 14394127 and four Laboratory Duplicates (by a different analyst) on EPA Sample Nos. 14394130, 14394134, 14394136, and 14394138.
4.2.2 Are replicate sample results within the specified acceptance limits?	☒	☐	
4.3 Duplicate Analyses			
4.3.1 Are duplicate sample analyses performed at the required frequency?	☒	☐	
4.3.2 Are duplicate sample results within the specified acceptance limits?	☒	☐	
4.3.2.1 If "no" then qualify the associated results in accordance with the Analytical Variability Results table in SOP QATS-70-090.			

Additional Comments:

Data Review Checklist for the Verification and Validation of Polarized Light Microscopy (PLM) Data Deliverables

4.0 Quality Control Verification & Validation (Level 2 and 3)	Yes	No	Comments
4.4 Reference Slide Analysis (if applicable)			The PLM Reference Material Comparison spreadsheet, PLM Accuracy QC and RTI Reference Material Comparison.xls, provided Reference Material results for analyst R. Brown. The analysts for the samples in this SDG are S. Golden and R. Brown; therefore, the PLM Reference Material analysis could not be evaluated for all analysts.
4.4.1 Are reference slide analyses performed at the required frequency?	☒	☐	
4.4.2 Are the reference slide analyses results within the specified acceptance criteria?	☒	☐	
4.4.2.1 If "no" then qualify the associated results in accordance with the Reference Slide Analysis table in SOP QATS-70-090.			
**5.0 Calibration & Microscope Alignment Validation (Level 3)**			
5.1 Are evidence of microscope alignment and Refractive Index (RI) liquid calibration provided for all sample analyses?	☒	☐	
5.1.1 Microscope-specific alignment checks?	☒	☐	
5.1.2 Microscope-specific contamination checks?	☒	☐	
5.1.3 Calibration RI liquids?	☒	☐	
5.2 Are alignment and calibration checks listed above performed at the required frequencies?	☒	☐	
5.3 Are alignment and calibration checks within the specified criteria?	☒	☐	
5.4 Are all alignment and calibration checks traceable to the associated samples analyses?	☒	☐	
5.4.1 If "no" then qualify the associated results in accordance with the Calibration Results table in SOP QATS-70-090.			
**6.0 Case Narrative Validation (Levels 2 & 3)**			
6.1 Does the data package narrative include descriptions of the following:			
6.1.1 Samples received (matrix/method)?	☒	☐	
6.1.2 Method/project requirement deviations?	☒	☐	
6.1.3 Example sample calculation?	☐	☒	
6.1.4 Laboratory blank contamination?	☐	☐	NA
6.1.5 Quality control analyses outside specified criteria?	☐	☐	NA
6.1.6 Any problems encountered and subsequent corrective action?	☐	☐	NA

Additional Comments:

Validator's Signature **Shellee McGrath** **Date 06/19/2015**

QA Review **Lyndsay Gensler** **Date 06/19/2015**

Appendix B

Qualified Result Forms

LabCor Portland Inc

Lab/Cor Portland, Inc.
4321 SW Corbett Ave., Ste A
Portland, OR 97239

Phone: (503) 224-5055
Fax: (503) 228-8282
http://www.labcorpdx.net

BULK SAMPLE ASBESTOS ANALYSIS

Asbestos and Environmental Analysis

Client: Alion Science and Technology
1000 Park Forty Plaza, Ste 200
Durham, NC 27713

Report Number: 142961R02
Report Date: 03/25/2015

P.O. No: SFP-78A

Job Number: 142961
Project Name: Sumas Mtn Asbestos Soil
Project Number: 10-100614-133206-0004
Project Notes:

Client Sample ID: 14394124 **Sample ID:** S1 **Date Analyzed:** 02/03/2015
Client Sample Description: **Analyst:** Stephanie Golden

Asbestos Mineral Fibers	Layer Percent:	Chrysotile	Amosite	Crocidolite	Point Count	Point Count Fields	Percent Asbestos:
**Homogeneous**					Point Count: 0	Point Count Fields: 400	
fine powder, brown	100 %	-	-	-			**NAD**

Other Fibers	Fibrous Glass	Cellulose	Mineral Wool	Synthetic	Other		Matrix
	-	1.25 %	-	-	-	-	98.75 %

Client Sample ID: 14394125 **Sample ID:** S2 **Date Analyzed:** 02/03/2015
Client Sample Description: **Analyst:** Stephanie Golden

Asbestos Mineral Fibers	Layer Percent:	Chrysotile	Amosite	Crocidolite	Point Count	Point Count Fields	Percent Asbestos:
**Homogeneous**					Point Count: 0	Point Count Fields: 400	
fine powder, brown	100 %	Trace	-	-			**< 0.25 %**

Other Fibers	Fibrous Glass	Cellulose	Mineral Wool	Synthetic	Other		Matrix
	-	1.25 %	-	-	-	-	98.75 %

Comments: 17 field of view counts for chrysotile in 400 fields.

Client Sample ID: 14394126 **Sample ID:** S3 **Date Analyzed:** 02/04/2015
Client Sample Description: **Analyst:** Ryan Brown

Asbestos Mineral Fibers	Layer Percent:	Chrysotile	Amosite	Crocidolite	Point Count	Point Count Fields	Percent Asbestos:
**Homogeneous**					Point Count: 0	Point Count Fields: 400	
fine powder, brown	100 %	Trace	-	-			**< 0.25 %**

Other Fibers	Fibrous Glass	Cellulose	Mineral Wool	Synthetic	Other		Matrix
	-	0.25 %	-	-	-	-	99.75 %

Comments: 10 field of view counts for chrysotile in 400 fields.

Client Sample ID: 14394127 **Sample ID:** S4 **Date Analyzed:** 02/04/2015
Client Sample Description: **Analyst:** Ryan Brown

Asbestos Mineral Fibers	Layer Percent:	Chrysotile	Amosite	Crocidolite	Point Count	Point Count Fields	Percent Asbestos:
**Homogeneous**					Point Count: 0	Point Count Fields: 400	
fine powder, brown	100 %	Trace	-	-			**< 0.25 %**

Other Fibers	Fibrous Glass	Cellulose	Mineral Wool	Synthetic	Other		Matrix
	-	0.5 %	-	-	-	-	99.5 %

Comments: 14 field of view counts for chrysotile in 400 fields.

NVLAP

LabCor Portland Inc

Lab/Cor Portland, Inc.
4321 SW Corbett Ave., Ste A
Portland, OR 97239

BULK SAMPLE ASBESTOS ANALYSIS

Asbestos and Environmental Analysis

Phone: (503) 224-5055
Fax: (503) 228-8282
http://www.labcorpdx.net

Job Number: 142961

Report Number: 142961R02
Report Date: 03/25/2015

Client Sample ID: 14394128 **Sample ID:** S5 **Date Analyzed:** 02/04/2015
Client Sample Description: **Analyst:** Ryan Brown

Asbestos Mineral Fibers	Layer Percent:	Chrysotile	Amosite	Crocidolite		Percent Asbestos:
**Homogeneous**					**Point Count:** 5 **Point Count Fields:** 400	
fine powder, brown	100 %	1.25 %	-	-		**1.25 %**

Other Fibers	Fibrous Glass	Cellulose	Mineral Wool	Synthetic	Other		Matrix
	-	0.25 %	-	-	-	-	98.5 %

Client Sample ID: 14394129 **Sample ID:** S6 **Date Analyzed:** 02/04/2015
Client Sample Description: **Analyst:** Ryan Brown

Asbestos Mineral Fibers	Layer Percent:	Chrysotile	Amosite	Crocidolite		Percent Asbestos:
**Homogeneous**					**Point Count:** 11 **Point Count Fields:** 400	
fine powder, brown	100 %	2.75 %	-	-		**2.75 %**

Other Fibers	Fibrous Glass	Cellulose	Mineral Wool	Synthetic	Other		Matrix
	-	Trace	-	-	-	-	97.25 %

Client Sample ID: 14394130 **Sample ID:** S7 **Date Analyzed:** 02/04/2015
Client Sample Description: **Analyst:** Ryan Brown

Asbestos Mineral Fibers	Layer Percent:	Chrysotile	Amosite	Crocidolite		Percent Asbestos:
**Homogeneous**					**Point Count:** 9 **Point Count Fields:** 400	
fine powder, brown	100 %	2.25 %	-	-		**2.25 %**

Other Fibers	Fibrous Glass	Cellulose	Mineral Wool	Synthetic	Other		Matrix
	-	0.25 %	-	-	-	-	97.5 %

Client Sample ID: 14394131 **Sample ID:** S8 **Date Analyzed:** 02/04/2015
Client Sample Description: **Analyst:** Ryan Brown

Asbestos Mineral Fibers	Layer Percent:	Chrysotile	Amosite	Crocidolite		Percent Asbestos:
**Homogeneous**					**Point Count:** 8 **Point Count Fields:** 400	
fine powder, brown	100 %	2 %	-	-		**2 %**

Other Fibers	Fibrous Glass	Cellulose	Mineral Wool	Synthetic	Other		Matrix
	-	-	-	-	-	-	98 %

Client Sample ID: 14394132 **Sample ID:** S9 **Date Analyzed:** 02/04/2015
Client Sample Description: **Analyst:** Ryan Brown

Asbestos Mineral Fibers	Layer Percent:	Chrysotile	Amosite	Crocidolite		Percent Asbestos:
**Homogeneous**					**Point Count:** 13 **Point Count Fields:** 400	
fine powder, brown	100 %	3.25 %	-	-		**3.25 %**

Other Fibers	Fibrous Glass	Cellulose	Mineral Wool	Synthetic	Other		Matrix
	-	-	-	-	-	-	96.75 %

NVLAP

LabCor Portland Inc

Lab/Cor Portland, Inc.
4321 SW Corbett Ave., Ste A
Portland, OR 97239

BULK SAMPLE ASBESTOS ANALYSIS

Asbestos and Environmental Analysis

Phone: (503) 224-5055
Fax: (503) 228-8282
http://www.labcorpdx.net

Job Number: 142961

Report Number: 142961R02
Report Date: 03/25/2015

Client Sample ID: 14394133 | **Sample ID:** S10 | **Date Analyzed:** 02/05/2015
Client Sample Description: | **Analyst:** Stephanie Golden

Asbestos Mineral Fibers	Layer Percent:	Chrysotile	Amosite	Crocidolite	Point Count	Point Count Fields	Percent Asbestos:
Homogeneous					45	400	
fine powder, brown	100 %	11.25 %	-	-			11.25 %

Other Fibers	Fibrous Glass	Cellulose	Mineral Wool	Synthetic	Other		Matrix
	-	-	-	-	-	-	88.75 %

Client Sample ID: 14394134 | **Sample ID:** S11 | **Date Analyzed:** 02/05/2015
Client Sample Description: | **Analyst:** Stephanie Golden

Asbestos Mineral Fibers	Layer Percent:	Chrysotile	Amosite	Crocidolite	Point Count	Point Count Fields	Percent Asbestos:
Homogeneous					46	400	
fine powder, brown	100 %	11.5 %	-	-			11.5 %

Other Fibers	Fibrous Glass	Cellulose	Mineral Wool	Synthetic	Other		Matrix
	-	-	-	-	-	-	88.5 %

Client Sample ID: 14394135 | **Sample ID:** S12 | **Date Analyzed:** 02/05/2015
Client Sample Description: | **Analyst:** Stephanie Golden

Asbestos Mineral Fibers	Layer Percent:	Chrysotile	Amosite	Crocidolite	Point Count	Point Count Fields	Percent Asbestos:
Homogeneous					34	400	
fine powder, brown	100 %	8.5 %	-	-			8.5 %

Other Fibers	Fibrous Glass	Cellulose	Mineral Wool	Synthetic	Other		Matrix
	-	-	-	-	-	-	91.5 %

Client Sample ID: 14394136 | **Sample ID:** S13 | **Date Analyzed:** 02/05/2015
Client Sample Description: | **Analyst:** Stephanie Golden

Asbestos Mineral Fibers	Layer Percent:	Chrysotile	Amosite	Crocidolite	Point Count	Point Count Fields	Percent Asbestos:
Homogeneous					44	400	
fine powder, brown	100 %	11 %	-	-			11 %

Other Fibers	Fibrous Glass	Cellulose	Mineral Wool	Synthetic	Other		Matrix
	-	-	-	-	-	-	89 %

Client Sample ID: 14394137 | **Sample ID:** S14 | **Date Analyzed:** 02/05/2015
Client Sample Description: | **Analyst:** Stephanie Golden

Asbestos Mineral Fibers	Layer Percent:	Chrysotile	Amosite	Crocidolite	Point Count	Point Count Fields	Percent Asbestos:
Homogeneous					45	400	
fine powder, brown	100 %	11.25 %	-	-			11.25 %

Other Fibers	Fibrous Glass	Cellulose	Mineral Wool	Synthetic	Other		Matrix
	-	-	-	-	-	-	88.75 %

NVLAP

LabCor Portland Inc
Lab/Cor Portland, Inc.
4321 SW Corbett Ave., Ste A
Portland, OR 97239

BULK SAMPLE ASBESTOS ANALYSIS

Asbestos and Environmental Analysis

Phone: (503) 224-5055
Fax: (503) 228-8282
http://www.labcorpdx.net

Job Number: 142961

Report Number: 142961R02
Report Date: 03/25/2015

Client Sample ID: 14394138 **Sample ID:** S15 **Date Analyzed:** 02/05/2015
Client Sample Description: **Analyst:** Stephanie Golden

Asbestos Mineral Fibers	Layer Percent:	Chrysotile	Amosite	Crocidolite		Percent Asbestos:
Homogeneous					Point Count: 41 Point Count Fields: 400	
fine powder, brown	100 %	10.25 %	-	-		10.25 %

Other Fibers	Fibrous Glass	Cellulose	Mineral Wool	Synthetic	Other		Matrix
	-	-	-	-	-	-	89.75 %

Client Sample ID: 14394139 **Sample ID:** S16 **Date Analyzed:** 02/05/2015
Client Sample Description: **Analyst:** Stephanie Golden

Asbestos Mineral Fibers	Layer Percent:	Chrysotile	Amosite	Crocidolite		Percent Asbestos:
Homogeneous					Point Count: 40 Point Count Fields: 400	
fine powder, brown	100 %	10 %	-	-		10 %

Other Fibers	Fibrous Glass	Cellulose	Mineral Wool	Synthetic	Other		Matrix
	-	-	-	-	-	-	90 %

Client Sample ID: 14394140 **Sample ID:** S17 **Date Analyzed:** 02/05/2015
Client Sample Description: **Analyst:** Stephanie Golden

Asbestos Mineral Fibers	Layer Percent:	Chrysotile	Amosite	Crocidolite		Percent Asbestos:
Homogeneous					Point Count: 41 Point Count Fields: 400	
fine powder, brown	100 %	10.25 %	-	-		10.25 %

Other Fibers	Fibrous Glass	Cellulose	Mineral Wool	Synthetic	Other		Matrix
	-	-	-	-	-	-	89.75 %

Client Sample ID: 14394141 **Sample ID:** S18 **Date Analyzed:** 02/05/2015
Client Sample Description: **Analyst:** Stephanie Golden

Asbestos Mineral Fibers	Layer Percent:	Chrysotile	Amosite	Crocidolite		Percent Asbestos:
Homogeneous					Point Count: 39 Point Count Fields: 400	
fine powder, brown	100 %	9.75 %	-	-		9.75 %

Other Fibers	Fibrous Glass	Cellulose	Mineral Wool	Synthetic	Other		Matrix
	-	-	-	-	-	-	90.25 %

Client Sample ID: 14394142 **Sample ID:** S19 **Date Analyzed:** 02/05/2015
Client Sample Description: **Analyst:** Stephanie Golden

Asbestos Mineral Fibers	Layer Percent:	Chrysotile	Amosite	Crocidolite		Percent Asbestos:
Homogeneous					Point Count: 40 Point Count Fields: 400	
fine powder, brown	100 %	10 %	-	-		10 %

Other Fibers	Fibrous Glass	Cellulose	Mineral Wool	Synthetic	Other		Matrix
	-	-	-	-	-	-	90 %

NVLAP

LabCor Portland Inc
Lab/Cor Portland, Inc.
4321 SW Corbett Ave., Ste A
Portland, OR 97239
Phone: (503) 224-5055
Fax: (503) 228-8282
http://www.labcorpdx.net

BULK SAMPLE ASBESTOS ANALYSIS

Asbestos and Environmental Analysis

Job Number: 142961

Report Number: 142961R02
Report Date: 03/25/2015

Client Sample ID: 14394143 **Sample ID:** S20 **Date Analyzed:** 02/05/2015
Client Sample Description: **Analyst:** Stephanie Golden

Asbestos Mineral Fibers	Layer Percent:	Chrysotile	Amosite	Crocidolite			Percent Asbestos:
**Homogeneous**					**Point Count:** 39	**Point Count Fields:** 400	
fine powder, brown	100 %	9.75 %	-	-			**9.75 %**

Other Fibers	Fibrous Glass	Cellulose	Mineral Wool	Synthetic	Other		Matrix
	-	-	-	-	-	-	90.25 %

Client Sample ID: 14394144 **Sample ID:** S21 **Date Analyzed:** 02/05/2015
Client Sample Description: **Analyst:** Stephanie Golden

Asbestos Mineral Fibers	Layer Percent:	Chrysotile	Amosite	Crocidolite			Percent Asbestos:
**Homogeneous**					**Point Count:** 40	**Point Count Fields:** 400	
fine powder, brown	100 %	10 %	-	-			**10 %**

Other Fibers	Fibrous Glass	Cellulose	Mineral Wool	Synthetic	Other		Matrix
	-	-	-	-	-	-	90 %

This laboratory participates in the National Voluntary Laboratory Accreditation Program (NVLAP).
Testing method is per 40 CFR 763 Subpart F, Appendix A, PLM.

Layered samples are considered non-homogeneous."Misc" is miscellaneous. "NAD" is No Asbestos Detected.
Asbestos consists of the following minerals: chrysotile, amosite, crocidolite, tremolite, actinolite, anthophyllite.
Small diameter fibers such as those found in vinyl floor tiles, may not be detected by PLM.
Asbestos detection interferences may result from material binders.
Qualitative and quantitative TEM analysis may be recommended for difficult samples.
Quantitative analysis by PLM point count or TEM is recommended for samples testing at < or = to 1% asbestos.
The following estimate of error for this method by visual estimation of asbestos percent are as follows:
1% asbestos: 0-3% error, 5% asbestos: 1-9% error, 10% asbestos: 5-15% error, 20% asbestos: 10-30% error.
This report pertains only to the samples listed on the report. Report considered valid only when signed by analyst.

Reviewed by:

x [signature]

Stephanie Golden
Technical Manager

NVLAP
