# Supplementary material for: Comparison of soil sampling and analytical methods for asbestos at the Sumas Mountain Asbestos Site—Working towards a toolbox for better assessment
Source: PLoS One. 2017 Jul 31;12(7):e0180210. doi: 10.1371/journal.pone.0180210 (PMC5536314; doi:10.1371/journal.pone.0180210)
Supplement: S10 Text — (PDF) [file pone.0180210.s011.pdf]

CB&I
2700 Chandler Avenue, Building C
Las Vegas, NV 89120
Tel: +1 702 795 0515
Fax: +1 702 795 8210
www.CBI.com

July 10, 2015

Julie Wroble
EPA Site Manager Region 10
EPA Region 10
1200 6th Ave, Suite 900
Seattle, WA 98101

Document ID #: 3015-07102015-7

Dear Ms. Wroble:

EPA CONTRACT NUMBER EP-W-10-033
TASK ORDER NUMBER 3015
ASBESTOS QA SUPPORT

Enclosed please find the Release of Validated Data Report for the validation of Transmission Electron Microscopy (TEM) soil sample data, Laboratory Job Number 150099. The forty-five (45) soil samples associated with these data were analyzed by Lab/Cor, Inc., Seattle, Washington for the Sumas Mountain Asbestos Soil Project. This report and accompanying appendices are deliverables under Task 10 of the subject Task Order.

If you have any questions, please feel free to contact me.

Sincerely,

Lyndsay Gensler
Task Leader, QATS Program
CB&I Federal Services LLC
Phone: 702.895.8730
E-Mail Address: lyndsay.gensler@cbifederalservices.com

cc: Shari Myer, EPA-ASB, QATS Task Order Project Officer
Administrative Contracting Officer (letter only)

RELEASE OF VALIDATED DATA

DATE: July 10, 2015

SUBJECT: Review of Data for Laboratory Job Number: 150099

LABORATORY: Lab/Cor, Inc., Seattle, Washington

FROM: Quality Assurance Technical Support (QATS) Program, Las Vegas, NV
CB&I Federal Services LLC

TO: Julie Wroble, Environmental Protection Agency

QATS reviewed the data for the following case:

Applicable SAP: NA

Chain-of-Custody Number: FBAS - 150209001

Method: Transmission Electron Microscopy (TEM) ISO 10312

Applicable Laboratory Modification(s): NA

Number and Type of Samples: 45 Soil Samples

EPA Sample Numbers: 14394100, 14394101, 14394102, 14394103, 14394104, 14394105, 14394106, 14394107, 14394108, 14394109, 14394110, 14394111, 14394112, 14394113, 14394114, 14394115, 14394116, 14394117, 14394118, 14394119, 14394120, 14394121, 14394122, 14394123, 14394124, 14394125, 14394126, 14394127, 14394128, 14394129, 14394130, 14394131, 14394132, 14394133, 14394134, 14394135, 14394136, 14394137, 14394138, 14394139, 14394140, 14394141, 14394142, 14394143, 14394144.

VALIDATION SUMMARY

Forty-five (45) soil samples from Laboratory Job Number 150099, were hand-delivered to Lab/Cor, Inc. in Seattle, WA for preparation by the Fluidized Bed Asbestos Segregator (FBAS) technique and analysis for asbestos by the TEM-ISO 10312 Method. The samples were received at the laboratory intact on 02/09/2015, and were analyzed between 02/17/2015 and 03/30/2015.

Listed below are the Data Qualification Summary Table, EDD/Bench Sheet Discrepancy Table, Data Qualifier Table, and Reason Code Table.

DATA QUALIFICATION SUMMARY TABLE

Criteria Exceeded	EPA Sample ID	Validation Qualifier	Reason Code
Daily Cu Al Alignment was not recorded for analysis date 02/26/2015 on the Hitachi H7000 Instrument.	14394126<br>14394127	UJ<br>J	MC
Daily Cu Al Alignment was not recorded for analysis date 03/15/2015 on the Hitachi H7000 Instrument.	14394123<br>14394124<br>14394125	J	MC
Daily Cu Al Alignment failed the Cu Peak Alignment with a value below the Lower Acceptance Value for analysis date 03/18/2015 on the Hitachi H7000 Instrument.	14394192 (Prep Lab Blank)	UJ	MC
Daily Cu Al Alignment was not recorded for analysis date 02/17/2015 on the JOEL Instrument.	14394100	J	MC
Daily Cu Al Alignment was not recorded for analysis date 03/15/2015 on the JOEL Instrument.	14394119	J	MC
Daily Cu Al Alignment was not recorded for analysis date 03/30/2015 on the JOEL Instrument.	Q150099g-B1 (Lab Blank)<br>Q150099h-B2 (Lab Blank)<br>Q150099I-B3 (Lab Blank)	UJ	MC
Daily Cu Al Alignment was not recorded for analysis date 02/27/2015 on the Hitachi H7000FA Instrument.	14394104	J	MC
Daily Cu Al Alignment was not recorded for analysis date 03/03/2015 on the Hitachi H7000FA Instrument.	14394109	J	MC
Daily Cu Al Alignment was not recorded for analysis date 03/08/2015 on the Hitachi H7000FA Instrument.	14394115<br>14394116	J	MC
Daily Cu Al Alignment was not recorded for analysis date 03/18/2015 on the Hitachi H7000FA Instrument.	14394122	J	MC
Daily Cu Al Alignment was not recorded for analysis date 03/22/2015 on the Hitachi H7000FA Instrument.	14394104 (Recount Different)	J	MC
k-Factors not performed at the required frequency (last performed on 01/24/2014 for instrument H-7000.)	14394127	J	IC
k-Factors not performed at the required frequency (last performed on 07/25/2014 for instrument JOEL 1200.)	14394100<br>14394106<br>14394107<br>14394111<br>14394119	J	IC
k-Factors not performed at the required frequency (last performed on 01/08/2014 for instrument H-7000FA.)	14394101<br>14394104<br>14394109<br>14394112<br>14394113<br>14394114<br>14394115<br>14394116<br>14394117<br>14394118<br>14394120<br>14394121<br>14394122<br>14394104 (Recount Different)	J	IC

EDD/BENCH SHEET DISCREPANCY TABLE

EPA Sample ID	C# *	Method/Matrix	Lab. Job No.	Analysis Date	Discrepancy
14394100	C0	TEM/Soil	150099	02/17/2015	The image ID for total structure #2 was incorrectly entered into the NADES file as J38721 (BF, DF, SP). The correct image ID is J38722 (BF, DF, SP).
14394100	C0	TEM/Soil	150099	02/17/2015	The structure type entered into the NADES file for primary structure #4 should be MC10. Only MC was entered.
14394101	C0	TEM/Soil	150099	02/21/2015	The structure type entered into the NADES file for primary structure #22 is incorrect. The MD11 should be MD10 because the length of total structure #22 is less than 5 µm.
14394106	C0	TEM/Soil	150099	02/27/2015	The image ID for total structure #21 was incorrectly entered into the NADES file as J38869 (BF, DF, SP). The correct image ID is J38880 (BF, DF, SP).
14394110	C0	TEM/Soil	150099	03/03/2015	The two-digit number assigned to the MR structure type was not recorded in the structure type column or the comments column for the MR in this sample.
14394115	C0	TEM/Soil	150099	03/07/2015	The image ID for total structure #2 was incorrectly entered into the NADES file as F39259 (BF, DF). The correct image ID is F39260 (BF, SP). Also there was no DF file as the diffraction pattern was too faint.
14394115	C0	TEM/Soil	150099	03/07/2015	The image ID for total structure #3 was omitted from the NADES file. The correct image ID for total structure #3 is F39261 (BF, DF).
14394116	C0	TEM/Soil	150099	03/08/2015	The structure type entered into the NADES file for primary structure #4 should be MD10. Only MD was entered.<br>The structure type entered into the NADES file for primary structures #41 and #50 should be MC10. Only MC was entered for each.<br>The brightfield file F39265BF was not provided with the 150099 images. The file is listed on the Direct Raw Data sheet, in the NADES file, and on the SEA150099 Final Image Log.
14394119	C0	TEM/Soil	150099	03/13/2015	The structure type entered into the NADES file for total structure #22 should be MR10. Only MR was entered.
14394123	C0	TEM/Soil	150099	03/14/2015	The structure type for primary structure #93 entered into NADES is MD1, the structure type on the ISO 10312 Direct Raw Data sheet is MD10; because the length of following total structure is less than 5 µm, a 0 should be entered after the 1.<br>The MF for total structure #94 does not meet recording criteria. The aspect ratio for length 15 and width 12.5 is less than 3:1. A "74-0" was entered into the comments field on the ISO 10312 Direct Raw Data sheet for this structure indicating that the MF maybe should have been reported as a MR+0 which would change the structure type of primary structure #59 to MD+1.
14394124	C0	TEM/Soil	150099	03/15/2015	The structure type for total structure #14 entered into NADES is MR with 13-0 in the comments field. The structure type should have been reported as a MR+0 which would change the structure type of primary structure #10 to MD+1.
14394125	C0	TEM/Soil	150099	03/16/2015	The structure types entered into the NADES file for primary structured #1 and #6 should be MD10. Only MD was entered.<br>The structure type entered into the NADES file for primary structure #7 should be MD20. Only MD was entered.
14394128	C0	TEM/Soil	150099	03/02/2015	The structure type entered into the NADES file for primary structure #25 should be MD20. MD10 was incorrectly entered.<br>The structure type entered into the NADES file for primary structure #29 should be MD10. Only 10 is entered.<br>The structure type entered into the NADES file for primary structure #41 is MD20; however, it is followed by five MFs not two.<br>In addition, the structure type entered into the NADES file for total structure #151 should be CR+0. Only CR was entered.<br>The two-digit number assigned to each of the MR structure types were not recorded in the structure type column for the 22 MR's in this sample.
14394129	C0	TEM/Soil	150099	03/03/2015	Image file, H39135BF provided is not a "Brightfield" file. It is actually a DF file.<br>The structure type entered into the NADES file for primary structure #61 should be MD30. Only MD was entered.<br>The F for total structure #110 does not meet recording criteria. The aspect ratio for length 1.06 and width 0.86 is less than 3:1.<br>The structure type entered into the NADES file for primary structure #90 is MD30; however, it is followed by four MFs not three.
14394130	C0	TEM/Soil	150099	03/03/2015	The 15-0 entered into the comments section for total structure #70 should be 28-0 according to the ISO 10312 Direct Raw Data sheet. The 15-0 is for total structure #71.<br>The MF for total structure #17 does not meet recording criteria. The aspect ratio for length 1.8 and width 0.8 is less than 3:1.<br>The structure type entered into the NADES file for primary structure #95 should be MD10. Only MD was entered.
14394131	C0	TEM/Soil	150099	03/04/2015	The image ID for total structure #7 was incorrectly entered into the NADES file

EPA Sample ID	C# *	Method/Matrix	Lab. Job No.	Analysis Date	Discrepancy
					as H39136 (BF, DF, SP). The correct image ID is H39139 (BF, DF, SP). The structure type entered into the NADES file for primary structure #2 should be MD10. MD20 was entered. The structure type entered into the NADES file for primary structure #3 should be MD50. MD60 was entered.
14394132	C0	TEM/Soil	150099	03/04/2015	The structure type entered into the NADES file for total structure #46 should be MC+0. Only 0 was entered. The structure type entered into the NADES file for total structure #96 is MR50. The number of component fibers in the comments section of the ISO 10312 Direct Raw Data Sheet is 32-0, therefore, the MR50 should have been reported as MR+0 which would make the structure type for primary structure #59 MD+0.
14394133	C0	TEM/Soil	150099	03/04/2015	The structure type entered into the NADES file for primary structure #92 should be MD10. 10MD was entered.
14394134	C0	TEM/Soil	150099	03/05/2015	The structure type entered into the NADES file for primary structure #48 should be MD+1. MD+D142 was entered.
14394135	C0	TEM/Soil	150099	03/17/2015	The brightfield and diffraction files H39368BF and DF were not provided with the 150099 images. It is listed on the Direct Raw Data sheet and on the SEA150099 Final Image Log. Note that the file names were not entered into the NADES file. Only the Spectra (SP) was provided. The structure type entered into the NADES file for primary structure #15 should be MD10. Only 10 is entered. The number of component fibers for the MR reported for total structure #123 is not recorded in the NADES file nor is it reported on the ISO 10312 Direct Raw Data sheet. The structure type entered into the NADES file for primary structure #74 should be MD10. Only MD was entered. The MF for total structure #124 is incorrectly reported as MF10. 149-0 was incorrectly entered into the comments column for total structure #131, 149-0 should have been entered for total structure #130. 10MD was entered as the structure type for primary structure #94. MD10 should have been entered. The structure type entered into the NADES file for total structure #173 is MR. The number of components is not recorded in the comments column nor is it recorded on the ISO 10312 Direct Raw Data sheet.
14394136	C0	TEM/Soil	150099	03/06/2015	The structure type entered into the NADES file for primary structure #83 is MD+1. The structure type that should have been entered is MD43. The reason for the incorrect structure type is that the MR+0 that was entered for total structure #120 should be reported as MR32.
14394137	C0	TEM/Soil	150099	03/06/2015	The structure type entered into the NADES file for primary structure #19 should be MD+0. MD+1 was entered; however, there were no structures > 5 um for the following MF and MR. The ISO 10312 Direct Raw Data file incorrectly lists that structure as MD2-1 when primary structure #19 was only followed by 1 MF that was < 5 um in length. The structure type entered into the NADES file for primary structure #68 is MD41; however, it is followed by five MFs not four.
14394138	C0	TEM/Soil	150099	03/06/2015	The brightfield and diffraction files H39369BF and DF were not provided with the 150099 images. It is listed on the Direct Raw Data sheet and on the SEA150099 Final Image Log. Note that the file names were not entered into the NADES file. Only the Spectra (SP) was provided. The length and width entered into the NADES file for primary structure #1 (3.87/2.25) do not match those on the ISO 10312 Direct Raw Data sheet (3.99/2.42). The lengths entered into the NADES file for total structure #1 and #2 (3.34 and 1.36, respectively) do not match those on the Direct Raw Data sheet (3.56 and 1.32). The structure type entered into the NADES file for primary structure #89 should be MD40. MD50 was entered.
14394139	C0	TEM/Soil	150099	03/07/2015	The structure type entered into the NADES file for primary structure #6 is MD20; however, it is followed by three MFs not two.
14394140	C0	TEM/Soil	150099	03/08/2015	The brightfield and diffraction files H39272BF and DF were not provided with the 150099 images. It is listed on the Direct Raw Data sheet and on the SEA150099 Final Image Log. Note that the file names were not entered into the NADES file. Only the Spectra (SP) was provided. The structure type entered into the NADES file for primary structure #61 should be MD+0. MD+D2050 was entered. The structure type entered for total structure #118 is MR+0; however, the number of components is not recorded in the comments column nor is it recorded on the ISO 10312 Direct Raw Data sheet. The brightfield file H39273BF was not provided with the 150099 images. It is listed on the Direct Raw Data sheet, NADES file, and on the SEA150099 Final

EPA Sample ID	C# *	Method/Matrix	Lab. Job No.	Analysis Date	Discrepancy
					Image Log.
14394141	C0	TEM/Soil	150099	03/09/2015	The structure type entered into the NADES file for total structure #95 should be MR+0. MR+0+D195 was entered.<br>The structure type entered into the NADES file for primary structure #85 should be MD60. MD+0 was entered.
14394142	C0	TEM/Soil	150099	03/10/2015	The structure type entered into the NADES file for primary structure #17 is MD+2. The structure type that should have been entered is MD+1. The reason for the incorrect structure type is that the MR+1 that was entered for total structure #26 should be reported as MR+0 according to the ISO 10312 Direct Raw Data sheet.<br>The structure type entered into the NADES file for primary structure #55 is MC20. The structure type for primary structure #55 reported on the ISO 10312 Direct Raw Data is MC50.<br>The MF for total structure #59 does not meet recording criteria. The aspect ratio for length 4 and width 2.35 is less than 3:1.
14394143	C0	TEM/Soil	150099	03/10/2015	The brightfield file H39297BF was not provided with the 150099 images. It is listed on the Direct Raw Data sheet, NADES file, and on the SEA150099 Final Image Log. Only the DF and SP files were provided.<br>The structure type entered into the NADES file for primary structure #81 is MD10 which is followed by 2 MF's; therefore, the structure type should be MD20.<br>The structure type entered into the NADES file for primary structure #92 is MD21 which is followed by 5 MF's; therefore, the structure type should be MD51.
14394143 Dup	C0	TEM/Soil	150099	03/11/2015	The structure type entered into the NADES file for primary structure #21 is MD50 which is followed by 1 MF and MR50; therefore, the structure type should be MD60.<br>The structure type entered into the NADES file for primary structure #29 is MD20 which is followed by 1 MF; therefore, the structure type should be MD10.<br>The structure type entered for total structure #48 is MR+0; however, the number of components is not recorded in the comments column nor is it recorded on the ISO 10312 Direct Raw Data sheet.<br>The structure type entered into the NADES file for primary structure #78 is MD51. The structure type that should have been entered is MD+1. The reason for the incorrect structure type is that the MR40 that was entered for total structure #114 should be reported as MR+0 according to the ISO 10312 Direct Raw Data sheet.
14394144	C0	TEM/Soil	150099	03/11/2015	The brightfield and diffraction files H39310BF and DF were not provided with the 150099 images. It is listed on the Direct Raw Data sheet and on the SEA150099 Final Image Log. Only the Spectra (SP) was provided.<br>The structure type entered into the NADES file for primary structure #10 is MD20 which is followed by 4 MF; therefore, the structure type should be MD40.<br>The structure type entered into the NADES file for primary structure #22 should be MD20. Only 20 was entered.<br>The MF for total structure #84 does not meet recording criteria. The aspect ratio for length 0.9 and width 2 is less than 3:1.
Q150099I-B3	C0	TEM/Soil	150099	03/30/2015	The NADES file lists S. Golden as the QA reviewer; however, a QA date is not provided.
14394104 RD	C0	TEM/Soil	150099	03/21/2015	The ISO 10312 Direct Raw Data sheets were not provided for the Recount Different and Repreparation QC samples; however, In the Data Entry 2 tab, the Grid was reported in the Grid Opening column, the Grid Opening was reported in the Low Mag? Column, and the Grid column was populated with S5 (which appears to be from the lab sample ID of 150099-S5)
13494129 RP	C0	TEM/Soil	150099	03/18/2015	The structure type entered into the NADES file for primary structure #8 should be MD20. Only MD was entered.<br>The MF for total structure #94 does not meet recording criteria. The aspect ratio for length 0.1 and width 0.1 is less than 3:1.
NA	NA	TEM/Soil	150099	NA	The Upper Acceptance Value for the Al Peak Alignment changes from 1.496 to 1.495 on 02/20/2015 on the Daily Cu Al Calibration sheets provided for the Hitachi H7000 instrument. The values of 1.496 found after that date are not highlighted as being out of criteria indicating that the change in criteria from 1.496 to 1.495 was not intentional.

'*' The EDD correction number in column 2. (i.e., C0, C1, C2, etc..)

DATA QUALIFIER TABLE

Qualifier	Definition
**J**	The result is estimated. The associated numerical value is an approximation.
**UJ**	The non-detect result may be inaccurate or imprecise due to the quality of the data generated because certain QC criteria were not met.
**R**	The sample results are rejected due to serious deficiencies.
**X**	Validator defined.

TEM REASON CODE TABLE

Reason Code	Definition
**MC**	Structure/fiber counts and recorded structure dimensions may be inaccurate due to improper or infrequent scope alignment and/or magnification calibrations.
**IC**	Identification by elemental composition or diffraction pattern may be inaccurate due to improper or infrequent EDXA or camera constant calibration.
**PA**	Structure/fiber counts and reported concentrations may be inaccurate due to improper or infrequent calibration of the plasma asher.
**SC**	The reported concentration may be inaccurate due to the condition of samples upon receipt at the laboratory.
**DL**	The area analyzed, structures counted, or AS do not meet the requirements specified in the applicable SAP Analytical Summary.
**ID**	The asbestos identification and concentrations may be inaccurate because the recorded structure types are not consistent with those described in the applicable TEM Method and/or laboratory modification(s).

VALIDATION PROCESS

The samples for Laboratory Job Number 150099 were received at the laboratory on 02/09/2015. The soil samples were prepared using the FBAS technique and analyzed in accordance with the TEM-ISO 10312 Method. CB&I's Quality Assurance Technical Support (QATS) Program performed validation in accordance with method-specific data validation SOPs. QATS preparation of this report and appendices was performed under Technical Direction 03, Task 10, of Task Order 3015.

The sample results on bench sheets and other supporting documents provided in the hardcopy deliverables were compared to the entries in the associated laboratory method-specific EDDs (where applicable) to ensure that the reported results are complete, compliant with the specified methodology, and accurate. Additional support information provided in this data validation report include the QATS Data Review Checklist used to document the data validation process (see Appendix A); and the sample results as reported by the laboratory, with qualifiers as applicable (see Appendix B).

TEM VALIDATION SUMMARY

1. **DATA PACKAGE INVENTORY AND SAMPLE RECEIPT:** The data package included a narrative, Chain-of-Custody (COC) record, EDD files, and QC samples. The samples were properly packaged, sealed, undamaged, and labeled upon receipt at the laboratory. The COC record was reviewed and found to be acceptable.

2. **SAMPLE PREPARATION:** The appropriate preparation documents were provided.

3. **EQUIPMENT CALIBRATION AND PERFORMANCE CHECKS (i.e., daily microscope alignment, screen magnification, EDS calibration, and sensitivity checks):** The Daily Cu Al alignment calibrations for 16 samples and blanks were not documented on the EDS Calibration (Al,Cu) sheets provided by the laboratory, and the Cu peak alignment failed for one preparation blank. The dates, instruments, and samples affected are listed in the Data Qualification Summary Table. Additionally, the k-factors provided by the laboratory were performed more than six months prior to the analyses of the samples in this SDG. These k-factors were performed on 01/24/2014 for instrument (scope) H-7000; on 07/25/2014 for instrument (scope) JOEL 1200; and on 01/08/2014 for instrument (scope) H7000FA. The laboratory's QAPP states for k-factors, "Calibration is performed on a biyearly basis, the first week of January and the first week of July." QATS requested the January 2015 k-factors from the laboratory; however, k-factors calculated on 03/30/2015 for Scope H-7000 and 05/21/2015 for Scopes JOEL 1200 and H7000FA were received. As a result of the described calibration deficiencies, twenty samples with TEM amphibole results reported in this SDG are qualified "J" due to the k-factor calibration. The equipment alignment and calibration for the remaining samples were performed at the correct frequency, indicating that the instruments were in proper working order during the time of sample analyses.

4. **ANALYTICAL SENSITIVITY:** A sufficient number of grid openings have been analyzed to achieve the required analytical sensitivity and/or the appropriate stopping rule was invoked.

5. **STRUCTURE RECORDING AND ASBESTOS IDENTIFICATION:** The structure recording and asbestos identification were found to be acceptable.

6. **BLANK ANALYSIS:** Five preparation laboratory blanks and two sand blanks were analyzed and reported with this sample set. There were no structures observed.

7. **ANALYTICAL VARIABILITY:** The laboratory performed one duplicate analysis on EPA Sample No. 14394143, five recount different (RD) analyses on EPA Sample Nos. 14394104, 14394125, 14394126, 14394131, and 14394192 (which is a lab prep blank) and one repreparation (RP)

analysis on EPA Sample No. 14394129. According to the laboratory's QC Summary Sheet, all QC samples passed the established QC criteria. Note that with the exception of the duplicate analysis performed on EPA Sample No. 14394143, the ISO 10312 Direct Raw Data sheets were not provided for the QC samples.

8. **LABORATORY MODIFICATIONS:** NA

9. **OVERALL ASSESSMENT OF DATA:** With the exception of the discrepancies described in the EDD/Bench Sheet Discrepancy Table and the qualifiers assigned to samples with undocumented daily Cu Al calibrations and the k-factor issues listed in the Data Qualification Summary Table, the deliverable was found to be complete. Note that the stopping rules were only entered into one of the NADES sample files (14394101) and the high magnification counting rules were not entered into NADES for samples 14394106 and 14394111.

   The validator also observed that when the number of component fibers or bundles in either the original matrix or matrix residual is outside of the range 1 – 9, the additional information of the number of component fibers and bundles was not always noted in the comments column of the NADES file. While this information was not consistently entered into the NADES files, it was consistently reported in the ISO 10312 Direct Raw Data.

   The ISO Analysis Narrative lists two samples where the total structure counts entered into the Data Entry 2 tab of the NADES file do not match the total structure count on the ISO Report tab. The QATS validator noted three additional samples where the total count reported does not match the total number entered:

   EPA Sample ID 14394100 reports 30 total structures when there were 29 entered.
   EPA Sample ID 14394101 reports 45 total structures when there were 38 entered.
   EPA Sample ID 14394115 reports 25 total structures when there were 23 entered.

   Samples reported in the narrative:
   EPA Sample ID 14394116 reports 68 total structures when there were 56 entered.
   EPA Sample ID 14394120 reports 93 total structures when there were 81 entered.

REVIEWED BY: Shellee McGrath DATE: 07/08/2015

Appendix A

Data Review Checklist

Appendix B

Qualified Result Forms

Data Review Checklist for the Verification and Validation of Transmission Electron Microscopy (TEM) Data Deliverables

**Project Name: Sumas Mtn Asbestos Soil Project**	**Case or Sample Set ID: 150099**
**Number of Samples/Matrix: 45 Soil Samples**	**COC Number: FBAS - 150209001**
**TEM Analytical Method: FBAS – TEM ISO 10312**	**Level of Validation (Circle one): 1 2 [3] Other**

**1.0 Data Package Inventory**	**Yes**	**No**	**Comments**
1.1 Were the project-specific requirements (i.e. acceptance criteria & analytical sensitivities) provided by the client prior to the initiation of validation activities?	☐	☒	A request was made to the laboratory for the laboratory's QAPP and/or SOPs documenting the frequency of the different TEM calibrations. The k-factor calibration was also requested.
1.2 Did the received hard copy deliverables contain all the necessary components:			
1.2.1 Case Narrative (Level 1, 2 & 3)?	☒	☐	
1.2.2 Chain-of-Custody (Level 1, 2 & 3)?	☒	☐	
1.2.3 Form I or equivalent (Level 1, 2 & 3)?	☒	☐	
1.2.4 Raw Data - Count Sheets (Level 1, 2 & 3)?	☒	☐	
1.2.5 QC Sample Data (Level 2 & 3):	☒	☐	Five preparation laboratory blanks and two sand blanks were reported.
1.2.5.1 Blank(s)?	☒	☐	
1.2.5.2 Replicate(s)?	☒	☐	One duplicate on EPA Sample No. 14394143, five recount different (RD) analyses on EPA Sample Nos. 14394104, 14394125, 14394126, 14394131, and 14394192 (which is a lab prep blank) and one repreparation (RP) analysis on EPA Sample No. 14394129 were performed.
1.2.5.3 Duplicate(s)?	☒	☐	
1.2.5.4 Verified Analysis?	☐	☒	
1.2.6 Calibration Data (Level 3)?	☒	☐	
1.2.7 Communication Records (Level 1, 2 & 3)?	☐	☒	
1.2.8 Miscellaneous?	☐	☐	
1.3 Were the necessary components received to perform the requested level of validation?	☒	☐	
**2.0 Chain-of-Custody Information Verification (Level 1, 2 & 3)**			
2.1 Were the following information recorded in the hard copy electronic deliverables (if applicable) consistent with the information recorded on the COC:			
2.1.1 COC Number?	☒	☐	
2.1.2 Case or Sample Set Number?	☒	☐	
2.1.3 EPA Sample ID?	☒	☐	
2.1.4 Date/Time Collected?	☒	☐	
2.1.5 Sample Volume?	☒	☐	
2.1.6 Sample Matrix?	☒	☐	
2.1.7 Analyses (Method)?	☒	☐	
2.1.8 Date/Time Received?	☒	☐	
2.1.9 Other (describe)?	☐	☐	
2.2 Were the COC records signed and dated upon receipt?	☒	☐	

Additional Comments:

Data Review Checklist for the Verification and Validation of Transmission Electron Microscopy (TEM) Data Deliverables

3.0 Sample Result Verification & Validation (Level 1, 2 & 3)	Yes	No	Comments
3.1 Is the sample preparation method documented and final sample volume recorded?	☒	☐	
3.2 Is the correct number of grid openings used to achieve the specified analytical sensitivity?	☒	☐	
3.3 Verify that the following information from the laboratory's bench sheets have been transcribed correctly:			
3.3.1.1 Grid identification?	☒	☐	
3.3.1.2 Grid opening?	☒	☐	Several discrepancies were observed and are listed in the EDD/Bench Sheet Discrepancy Table of the Asbestos Validation Narrative.
3.3.1.3 Structure type?	☐	☒	
3.3.1.4 Number of primary and secondary structures?	☐	☒	
3.3.1.5 Length and width dimensions?	☐	☒	
3.3.1.6 Structure identification?	☒	☐	
3.3.1.7 Mineral type?	☒	☐	
3.4 Are overloaded samples correctly reported to the specified percent obscuration (i.e. 10%, 25%)?	☐	☐	Not Recorded by Laboratory.
3.5 If overloading occurs, are samples prepared by an alternate method (i.e. indirect preparation)?	☐	☐	NA
3.6 Verify that the following information is documented correctly:			
3.6.1 Magnification?	☒	☐	
3.6.2 Field or QC sample type?	☒	☐	
3.6.3 Number of grids prepared?	☒	☐	
3.6.4 Filter area in (mm^2)?	☒	☐	
3.6.5 Analysis date?	☒	☐	
3.7 Verify the totals reported on the count sheets for the various types of structures. These may include:			A few discrepancies between the total structures reported and the number entered into NADES.
3.7.1 Total EPA Structures	☐	☒	See Asbestos Validation Narrative.
3.7.2 PCMe Structures	☒	☐	
3.7.3 AHERA Structures	☐	☐	NA
3.7.4 Berman Crump Structures	☐	☐	NA
3.8 Are the required spectra included for all hits reported (i.e. ED, EDXA, SAED)?	☐	☒	Not all of the files listed were included in the deliverable. See EDD/Bench Sheet Discrepancy Table in the Asbestos Validation Narrative.
3.9 Recalculate the reported concentration on at least 10% of the results reported.			
3.9.1 Are the recalculated concentrations consistent with those reported?	☒	☐	

Additional Comments:

Data Review Checklist for the Verification and Validation of Transmission Electron Microscopy (TEM) Data Deliverables

4.0 Quality Control Verification & Validation (Level 2 and 3)	Yes	No	Comments
4.1 Blanks			
4.1.1 Are laboratory blanks (direct, indirect) prepared and analyzed at the required frequency?	☒	☐	Five preparation laboratory blanks and two sand blanks were reported.
4.1.2 Are laboratory blank results within the specified criteria?	☒	☐	
4.1.2.1 If "no" then qualify the associated results in accordance with the Blank Result table in SOP QATS-70-091.			
4.2 Replicate Analyses			
4.2.1 Are replicate (second analyst on the same grids but different grid openings) sample analyses performed at the required frequency?	☒	☐	The laboratory performed one duplicate on EPA Sample No. 14394143, five recount different (RD) analyses on EPA Sample Nos. 14394104, 14394125, 14394126, 14394131, and 14394192 (which is a lab prep blank) and one repreparation (RP) analysis on EPA Sample No. 14394129.
4.2.2 Are replicate sample results within the specified acceptance limits?	☒	☐	
4.2.2.1 If "no" then qualify the associated results in accordance with the Analytical Variability Results table in SOP QATS-70-091.			
4.3 Duplicate Analyses			
4.3.1 Are duplicates (analysis of a second sample preparation obtained from the final filter) prepared and analyzed at the required frequency?	☒	☐	
4.3.2 Are duplicate sample results within the specified acceptance limits?	☒	☐	
4.3.2.1 If "no" then qualify the associated results in accordance with the Analytical Variability Results table in SOP QATS-70-091.			
4.4 Verified Analyses			
4.4.1 Are verified analyses (second analysis on same grids and grid openings) at the required frequency?	☐	☒	
4.4.2 Are sample verification results within the specified acceptance limits?	☐	☐	
4.4.2.1 If "no" then qualify the associated results in accordance with the Analytical Variability Results table in SOP QATS-70-091.			

Additional Comments:

Data Review Checklist for the Verification and Validation of Transmission Electron Microscopy (TEM) Data Deliverables

5.0 Calibration & Microscope Alignment Validation (Level 3)	Yes	No	Comments
5.1 Is evidence of the calibration of TEM Screen Magnification provided for all sample analyses?	☒	☐	Daily calibration was not recorded for 10 days between the three instruments affecting a total of 16 field samples and QC. One daily calibration failed the Cu Peak Alignment on instrument H7000 affecting one prep lab blank. All affected samples are qualified "J" or "UJ" with a reason code of MC. The k-Factors provided by the laboratory were performed on 01/24/2014) for instrument H-7000 and 07/25/2014 for instrument JOEL 1200, and 01/08/2014 for instrument H7000FA, more than six months prior to the analyses of the samples in this SDG. The laboratory's QAPP states for k-factors "Calibration is performed on a biyearly basis, the first week of January and the first week of July." Amphibole results for 20 samples were affected and qualified "J" with a reason code of "IC".
5.1.1 Camera Constant Calibration?	☒	☐	
5.1.2 Calibration of the EDXA System?	☒	☐	
5.1.3 k-Factors?	☐	☒	
5.2 Are the calibration checks listed above performed at the required frequencies?	☒	☐	
5.3 Are the calibration checks within the specified criteria?	☒	☐	
5.4 Are all calibration checks traceable to the associated samples analyses?	☒	☐	
5.5 If required, are the following additional system checks provided:			
5.5.1 Beam Dose Check?	☒	☐	
5.5.2 Spot Size Check?	☒	☐	
5.5.3 Detector Resolution Check?	☒	☐	
5.5.4 Resolvable Na, Mg, and Si Peaks?	☒	☐	
5.5.5 If "no" then qualify the associated results in accordance with the Calibration Results table in SOP QATS-70-091.			
**6.0 Case Narrative Validation (Levels 2 & 3)**			
6.1 Does the data package narrative include descriptions of the following:			
6.1.1 Samples received (matrix/method)?	☒	☐	
6.1.2 Method/project requirement deviations?	☒	☐	
6.1.3 Example sample calculation?	☒	☐	
6.1.4 Laboratory blank contamination?	☐	☐	NA
6.1.5 Quality control analyses outside specified criteria?	☐	☐	NA
6.1.6 Any problems encountered and subsequent corrective action?	☐	☐	NA

Additional Comments:

Validator's Signature Shellee McGrath **Date** 07/08/2015

QA Review Lyndsay Gensler **Date** 07/09/2015

Sumas Mtn Asbestos Methods - FBAS_Labcor Inc_14394100_SO_02-17-15_150099_TEM-EPAISO__D.xlsm
FluidizedBedEDD_v1

TEM Asbestos Structure Count for Fluidized Bed Material -- ISO
ANALYTICAL REPORT

FILE NAME:Sumas Mtn Asbestos Methods - FBAS_Labcor Inc_14394100_SO_02-17-15_150099_TEM-EPAISO__D.xlsm

SAMPLE INFORMATION		ANALYSIS PARAMETERS		
**Field Sample Number**	14394100	**EFA (mm^2)**	385	
		**GO area (mm^2) [Ago]**	0.0104	
**Matrix**	Soil	**# GOs counted - high mag**	100	
**Field QC Type**	Not QC	**# GOs counted - low mag only**	0	
**Sample Mass (g dry wt) [M]**	0.56	**F-factor [f]**	1.00E+00	
**Fluidized Bed Flow Ratio[Q_R]**	0.0122			
**Filter Status**	ANALYZED	Sensitivity - High mag	5.5E+04	1/g
JOEL		Sensitivity - PCME (high+low mag)	5.5E+04	1/g

ANALYSIS INFORMATION

Lab Name Labcor Inc
Lab Sample Number 150099-S1
Lab QC Type NOT QC
TEM Prep Method Direct
Est. particulate loading on filter NA

Recording Rules:

Initial (high mag)

Min. AR	Min length (um)	Min width (um)
3:1	0	0

PCME Supplemental (low mag)

Min. AR	Min length (um)	Min width (um)
3:1	0	0

Stopping Rules:

Target S (1/g)	Max Area (mm^2)	Max N

RESULTS SUMMARY

Structure Metric	Observed Structure Count (s)	Structure Filter Loading (s/mm^2)	Soil Conc. Structures per gram (s/g)
**High mag only**			
Total Asbestos	30	2.9E+01	1.6E+06
Total Chrysotile (CH)	17	1.6E+01	9.3E+05
Total Amphibole	13	1.3E+01	7.1E+05
actinolite (AC)	8	7.7E+00	4.4E+05
amosite (AM)	0	0.0E+00	0.0E+00
anthophyllite (AN)	0	0.0E+00	0.0E+00
crocidolite (CR)	0	0.0E+00	0.0E+00
tremolite (TR)	3	2.9E+00	1.6E+05
winchite/richterite/tremolite/actinolite (WRTA)	0	0.0E+00	0.0E+00
other amphibole (OA)	2	1.9E+00	1.1E+05
other mineral class (OM)	0	0.0E+00	0.0E+00
**PCME (high+low mag)**			
Total Asbestos	0	0.0E+00	0.0E+00
Total Chrysotile (CH)	0	0.0E+00	0.0E+00
Total Amphibole	0	0.0E+00	0.0E+00
actinolite (AC)	0	0.0E+00	0.0E+00
amosite (AM)	0	0.0E+00	0.0E+00
anthophyllite (AN)	0	0.0E+00	0.0E+00
crocidolite (CR)	0	0.0E+00	0.0E+00
tremolite (TR)	0	0.0E+00	0.0E+00
winchite/richterite/tremolite/actinolite (WRTA)	0	0.0E+00	0.0E+00
other amphibole (OA)	0	0.0E+00	0.0E+00
other mineral class (OM)	0	0.0E+00	0.0E+00

Σ Asbestos Structure Mass (g)		Mass fraction (as %)
**Total TEM**		
WRTA	0.00E+00	0.0000%
OA	2.98E-09	1.6349%
CH	2.76E-09	1.5150%
AC	5.07E-10	0.2783%
AM	0.00E+00	0.0000%
AN	0.00E+00	0.0000%
CR	0.00E+00	0.0000%
TR	2.50E-09	1.3723%
**PCME**		
WRTA	0.00E+00	0.0000%
OA	0.00E+00	0.0000%
CH	0.00E+00	0.0000%
AC	#N/A	#N/A
AM	0.00E+00	0.0000%
AN	0.00E+00	0.0000%
CR	0.00E+00	0.0000%
TR	0.00E+00	0.0000%

Asbestos structure mass = Length • Width^2 • Density • 1E-12

LA Density = 3.1 g/cm^3
OA Density = 3.0 g/cm^3
CH Density = 2.6 g/cm^3
AC Density = 3.1 g/cm^3
AM Density = 3.5 g/cm^3
AN Density = 3.0 g/cm^3
CR Density = 3.3 g/cm^3
TR Density = 3.0 g/cm^3

Csoil (mass fraction, as %) = Σ Asbestos structure mass • Sensitivity • 100

Equations:
Filter Loading (s/mm^2) = N / (GOx • Ago)
Sensitivity (1/g) = EFA / (GOx • Ago • M • f • Q_R)
Csoil (s/g) = N • Sensitivity

Asbestos Types:
AC - actinolite
AM - amosite
AN - anthophyllite
CH - chrysotile
CR - crocidolite
TR - tremolite
LA - Libby amphibole
OA - other amphibole
NAM - non-asbestos material
OM - other mineral type (specify in "other mineral description" field)

Structure Types:
High mag: Length ≥ 0.5 um, Aspect Ratio ≥ 3:1
PCME: Length > 5 um, Width ≥ 0.25 um, Aspect Ratio ≥ 3:1

Sumas Mtn Asbestos Methods - FBAS_LabCor Inc_14394101_SO_02-21-15_150099_TEM-EPAISO__D.xlsm
FluidizedBedEDD_v1

TEM Asbestos Structure Count for Fluidized Bed Material -- ISO ANALYTICAL REPORT

FILE NAME:Sumas Mtn Asbestos Methods - FBAS_LabCor Inc_14394101_SO_02-21-15_150099_TEM-EPAISO__D.xlsm

SAMPLE INFORMATION		ANALYSIS PARAMETERS		
**Field Sample Number**	14394101	**EFA (mm^2)**	385	
		**GO area (mm^2) [Ago]**	0.0104	
**Matrix**	Soil	**# GOs counted - high mag**	100	
**Field QC Type**	Not QC	**# GOs counted - low mag only**	0	
**Sample Mass (g dry wt) [M]**	0.50	**F-factor [f]**	1.00E+00	
**Fluidized Bed Flow Ratio[Q_R]**	0.0122			
**Filter Status**	ANALYZED	Sensitivity - High mag	6.1E+04	1/g
		Sensitivity - PCME (high+low mag)	6.1E+04	1/g

1700DFA

ANALYSIS INFORMATION

Lab Name LabCor Inc
Lab Sample Number 150099-S2
Lab QC Type NOT QC
TEM Prep Method Direct
Est. particulate loading on filter NA

Recording Rules:

Initial (high mag)

Min. AR	Min length (um)	Min width (um)
3:1	0	0

PCME Supplemental (low mag)

Min. AR	Min length (um)	Min width (um)
3:1	0	0

Stopping Rules:

Target S (1/g)	Max Area (mm^2)	Max N
	1.035	100

RESULTS SUMMARY

Structure Metric	Observed Structure Count (s)	Structure Filter Loading (s/mm²)	Soil Conc. Structures per gram (s/g)
**High mag only**			
Total Asbestos	45	4.3E+01	2.7E+06
Total Chrysotile (CH)	17	1.6E+01	1.0E+06
Total Amphibole	28	2.7E+01	1.7E+06
actinolite (AC)	5	4.8E+00	3.0E+05
amosite (AM)	5	4.8E+00	3.0E+05
anthophyllite (AN)	3	2.9E+00	1.8E+05
crocidolite (CR)	0	0.0E+00	0.0E+00
tremolite (TR)	10	9.7E+00	6.1E+05
winchite/richterite/tremolite/actinolite (WRTA)	0	0.0E+00	0.0E+00
other amphibole (OA)	5	4.8E+00	3.0E+05
other mineral class (OM)	0	0.0E+00	0.0E+00
**PCME (high+low mag)**			
Total Asbestos	6.000000132	5.8E+00	3.7E+05
Total Chrysotile (CH)	4	3.9E+00	2.4E+05
Total Amphibole	2.000000132	1.9E+00	1.2E+05
actinolite (AC)	1	9.7E-01	6.1E+04
amosite (AM)	1.31739E-07	1.3E-07	8.0E-03
anthophyllite (AN)	1	9.7E-01	6.1E+04
crocidolite (CR)	0	0.0E+00	0.0E+00
tremolite (TR)	0	0.0E+00	0.0E+00
winchite/richterite/tremolite/actinolite (WRTA)	0	0.0E+00	0.0E+00
other amphibole (OA)	3.5293E-13	3.4E-13	2.1E-08
other mineral class (OM)	0	0.0E+00	0.0E+00

RC

	Σ Asbestos Structure Mass (g)	Mass fraction (as %)
**Total TEM**		
WRTA	0.00E+00	0.0000%
OA	5.90E-10	0.3591%
CH	2.28E-08	13.8835%
AC	8.86E-11	0.0539%
AM	0.00E+00	0.0000%
AN	1.40E-08	8.5478%
CR	0.00E+00	0.0000%
TR	3.99E-07	########
**PCME**		
WRTA	0.00E+00	0.0000%
OA	5.89E-10	0.3583%
CH	4.17E-10	0.2538%
AC	#N/A	#N/A
AM	0.00E+00	0.0000%
AN	#N/A	#N/A
CR	0.00E+00	0.0000%
TR	#N/A	#N/A

Asbestos structure mass = Length • Width^2 • Density • 1E-12

LA Density = 3.1 g/cm^3
OA Density = 3.0 g/cm^3
CH Density = 2.6 g/cm^3
AC Density = 3.1 g/cm^3
AM Density = 3.5 g/cm^3
AN Density = 3.0 g/cm^3
CR Density = 3.3 g/cm^3
TR Density = 3.0 g/cm^3

Csoil (mass fraction, as %) = Σ Asbestos structure mass • Sensitivity • 100

Equations:

Filter Loading (s/mm^2) = N / (GOx • Ago)
Sensitivity (1/g) = EFA / (GOx • Ago • M • f • Q_R)
Csoil (s/g) = N • Sensitivity

Asbestos Types:

AC - actinolite
AM - amosite
AN - anthophyllite
CH - chrysotile
CR - crocidolite
TR - tremolite
LA - Libby amphibole
OA - other amphibole
NAM - non-asbestos material
OM - other mineral type (specify in "other mineral description" field)

Structure Types:

High mag: Length ≥ 0.5 um, Aspect Ratio ≥ 3:1
PCME: Length > 5 um, Width ≥ 0.25 um, Aspect Ratio ≥ 3:1

Sumas Mtn Asbestos Methods - FBAS_LabCor Inc_14394102_SO_02-21-15_150099_TEM-EPAISO__D.xlsm
FluidizedBedEDD_v1

TEM Asbestos Structure Count for Fluidized Bed Material -- ISO
ANALYTICAL REPORT

FILE NAME:Sumas Mtn Asbestos Methods - FBAS_LabCor Inc_14394102_SO_02-21-15_150099_TEM-EPAISO__D.xlsm

SAMPLE INFORMATION		ANALYSIS PARAMETERS		
**Field Sample Number**	14394102	**EFA (mm^2)**	385	
		**GO area (mm^2) [Ago]**	0.0104	
**Matrix**	Soil	**# GOs counted - high mag**	100	
**Field QC Type**	Not QC	**# GOs counted - low mag only**	0	
**Sample Mass (g dry wt) [M]**	0.50	**F-factor [f]**	1.00E+00	
**Fluidized Bed Flow Ratio[Q_R]**	0.0122			
**Filter Status**	ANALYZED	Sensitivity - High mag	6.1E+04	1/g
		Sensitivity - PCME (high+low mag)	6.1E+04	1/g

ANALYSIS INFORMATION

**Lab Name**	LabCor Inc
**Lab Sample Number**	150099-S3
**Lab QC Type**	NOT QC
**TEM Prep Method**	Direct
**Est. particulate loading on filter**	NA

Recording Rules:

Initial (high mag)

Min. AR	Min length (um)	Min width (um)
3:1	0	0

PCME Supplemental (low mag)

Min. AR	Min length (um)	Min width (um)
3:1	0	0

Stopping Rules:

Target S (1/g)	Max Area (mm^2)	Max N

RESULTS SUMMARY

Structure Metric	Observed Structure Count (s)	Structure Filter Loading (s/mm^2)	Soil Conc. Structures per gram (s/g)
**High mag only**			
Total Asbestos	2	1.9E+00	1.2E+05
Total Chrysotile (CH)	2	1.9E+00	1.2E+05
Total Amphibole	0	0.0E+00	0.0E+00
actinolite (AC)	0	0.0E+00	0.0E+00
amosite (AM)	0	0.0E+00	0.0E+00
anthophyllite (AN)	0	0.0E+00	0.0E+00
crocidolite (CR)	0	0.0E+00	0.0E+00
tremolite (TR)	0	0.0E+00	0.0E+00
winchite/richterite/tremolite/actinolite (WRTA)	0	0.0E+00	0.0E+00
other amphibole (OA)	0	0.0E+00	0.0E+00
other mineral class (OM)	0	0.0E+00	0.0E+00
**PCME (high+low mag)**			
Total Asbestos	0	0.0E+00	0.0E+00
Total Chrysotile (CH)	0	0.0E+00	0.0E+00
Total Amphibole	0	0.0E+00	0.0E+00
actinolite (AC)	0	0.0E+00	0.0E+00
amosite (AM)	0	0.0E+00	0.0E+00
anthophyllite (AN)	0	0.0E+00	0.0E+00
crocidolite (CR)	0	0.0E+00	0.0E+00
tremolite (TR)	0	0.0E+00	0.0E+00
winchite/richterite/tremolite/actinolite (WRTA)	0	0.0E+00	0.0E+00
other amphibole (OA)	0	0.0E+00	0.0E+00
other mineral class (OM)	0	0.0E+00	0.0E+00

Σ Asbestos Structure Mass (g)		Mass fraction (as %)
**Total TEM**		
WRTA	0.00E+00	0.0000%
OA	0.00E+00	0.0000%
CH	6.25E-11	0.0381%
AC	0.00E+00	0.0000%
AM	0.00E+00	0.0000%
AN	0.00E+00	0.0000%
CR	0.00E+00	0.0000%
TR	0.00E+00	0.0000%
**PCME**		
WRTA	0.00E+00	0.0000%
OA	0.00E+00	0.0000%
CH	8.95E-12	0.0055%
AC	0.00E+00	0.0000%
AM	0.00E+00	0.0000%
AN	0.00E+00	0.0000%
CR	0.00E+00	0.0000%
TR	0.00E+00	0.0000%

Asbestos structure mass = Length • Width^2 • Density • 1E-12

LA Density = 3.1 g/cm^3
OA Density = 3.0 g/cm^3
CH Density = 2.6 g/cm^3
AC Density = 3.1 g/cm^3
AM Density = 3.5 g/cm^3
AN Density = 3.0 g/cm^3
CR Density = 3.3 g/cm^3
TR Density = 3.0 g/cm^3

Csoil (mass fraction, as %) = Σ Asbestos structure mass • Sensitivity • 100

Equations:

Filter Loading (s/mm^2) = N / (GOx • Ago)
Sensitivity (1/g) = EFA / (GOx • Ago • M • f • Q_R)
Csoil (s/g) = N • Sensitivity

Asbestos Types:

AC - actinolite
AM - amosite
AN - anthophyllite
CH - chrysotile
CR - crocidolite
TR - tremolite
LA - Libby amphibole
OA - other amphibole
NAM - non-asbestos material
OM - other mineral type (specify in "other mineral description" field)

Structure Types:

High mag: Length ≥ 0.5 um, Aspect Ratio ≥ 3:1
PCME: Length > 5 um, Width ≥ 0.25 um, Aspect Ratio ≥ 3:1

Sumas Mtn Asbestos Methods - FBAS_LabCor Inc_14394103_SO_02-22-15_150099_TEM-EPAISO__D.xlsm
FluidizedBedEDD_v1

TEM Asbestos Structure Count for Fluidized Bed Material -- ISO
ANALYTICAL REPORT

FILE NAME:Sumas Mtn Asbestos Methods - FBAS_LabCor Inc_14394103_SO_02-22-15_150099_TEM-EPAISO__D.xlsm

SAMPLE INFORMATION		ANALYSIS PARAMETERS		
**Field Sample Number**	14394103	**EFA (mm^2)**	385	
		**GO area (mm^2) [Ago]**	0.0104	
**Matrix**	Soil	**# GOs counted - high mag**	100	
**Field QC Type**	Not QC	**# GOs counted - low mag only**	0	
**Sample Mass (g dry wt) [M]**	0 51	**F-factor [f]**	1 00E+00	
**Fluidized Bed Flow Ratio[Q_R]**	0.0122			
**Filter Status**	ANALYZED	Sensitivity - High mag	6.0E+04	1/g
		Sensitivity - PCME (high+low mag)	6 0E+04	1/g

ANALYSIS INFORMATION

**Lab Name**	LabCor Inc
**Lab Sample Number**	150099-S4
**Lab QC Type**	NOT QC
**TEM Prep Method**	Direct
**Est. particulate loading on filter**	NA

Recording Rules:

Initial (high mag)

Min. AR	Min length (um)	Min width (um)
3:1	0	0

PCME Supplemental (low mag)

Min. AR	Min length (um)	Min width (um)
3:1	0	0

Stopping Rules:

Target S (1/g)	Max Area (mm^2)	Max N

RESULTS SUMMARY

Structure Metric	Observed Structure Count (s)	Structure Filter Loading (s/mm^2)	Soil Conc. Structures per gram (s/g)
**High mag only**			
Total Asbestos	2	1.9E+00	1.2E+05
Total Chrysotile (CH)	2	1 9E+00	1 2E+05
Total Amphibole	0	0.0E+00	0.0E+00
actinolite (AC)	0	0.0E+00	0.0E+00
amosite (AM)	0	0 0E+00	0 0E+00
anthophyllite (AN)	0	0 0E+00	0 0E+00
crocidolite (CR)	0	0.0E+00	0.0E+00
tremolite (TR)	0	0.0E+00	0.0E+00
winchite/richterite/tremolite/actinolite (WRTA)	0	0.0E+00	0.0E+00
other amphibole (OA)	0	0.0E+00	0.0E+00
other mineral class (OM)	0	0.0E+00	0.0E+00
**PCME (high+low mag)**			
Total Asbestos	0	0.0E+00	0.0E+00
Total Chrysotile (CH)	0	0.0E+00	0.0E+00
Total Amphibole	0	0.0E+00	0.0E+00
actinolite (AC)	0	0.0E+00	0 0E+00
amosite (AM)	0	0.0E+00	0.0E+00
anthophyllite (AN)	0	0.0E+00	0.0E+00
crocidolite (CR)	0	0.0E+00	0 0E+00
tremolite (TR)	0	0.0E+00	0.0E+00
winchite/richterite/tremolite/actinolite (WRTA)	0	0.0E+00	0.0E+00
other amphibole (OA)	0	0.0E+00	0.0E+00
other mineral class (OM)	0	0.0E+00	0.0E+00

Σ Asbestos Structure Mass (g)		Mass fraction (as %)
**Total TEM**		
WRTA	0.00E+00	0.0000%
OA	0.00E+00	0.0000%
CH	1.67E-12	0.0010%
AC	0.00E+00	0.0000%
AM	0.00E+00	0.0000%
AN	0.00E+00	0.0000%
CR	0.00E+00	0.0000%
TR	0.00E+00	0.0000%
**PCME**		
WRTA	0.00E+00	0.0000%
OA	0 00E+00	0.0000%
CH	0.00E+00	0.0000%
AC	0.00E+00	0.0000%
AM	0 00E+00	0.0000%
AN	0.00E+00	0.0000%
CR	0.00E+00	0.0000%
TR	0 00E+00	0.0000%

Asbestos structure mass = Length • Width^2 • Density • 1E-12

LA Density = 3.1 g/cm^3
OA Density = 3.0 g/cm^3
CH Density = 2.6 g/cm^3
AC Density = 3.1 g/cm^3
AM Density = 3.5 g/cm^3
AN Density = 3.0 g/cm^3
CR Density = 3.3 g/cm^3
TR Density = 3.0 g/cm^3

Csoil (mass fraction, as %) = Σ Asbestos structure mass • Sensitivity • 100

Equations:

Filter Loading (s/mm^2) = N / (GOx • Ago)
Sensitivity (1/g) = EFA / (GOx • Ago • M • f • Q_R)
Csoil (s/g) = N • Sensitivity

Asbestos Types:

AC - actinolite
AM - amosite
AN - anthophyllite
CH - chrysotile
CR - crocidolite
TR - tremolite
LA - Libby amphibole
OA - other amphibole
NAM - non-asbestos material
OM - other mineral type (specify in "other mineral description" field)

Structure Types:

High mag: Length ≥ 0.5 um, Aspect Ratio ≥ 3:1
PCME: Length > 5 um, Width ≥ 0.25 um, Aspect Ratio ≥ 3:1

Sumas Mtn Asbestos Methods - FBAS_LabCor Inc_14394104_SO_02-25-15_150099_TEM-EPAISO__D.xlsm
FluidizedBedEDD_v1

TEM Asbestos Structure Count for Fluidized Bed Material -- ISO
ANALYTICAL REPORT

FILE NAME:Sumas Mtn Asbestos Methods - FBAS_LabCor Inc_14394104_SO_02-25-15_150099_TEM-EPAISO__D.xlsm

SAMPLE INFORMATION		ANALYSIS PARAMETERS		
**Field Sample Number**	14394104	**EFA (mm^2)**	385	
		**GO area (mm^2) [Ago]**	0.0104	
**Matrix**	Soil	**# GOs counted - high mag**	100	
**Field QC Type**	Not QC	**# GOs counted - low mag only**	0	
**Sample Mass (g dry wt) [M]**	0.51	**F-factor [f]**	1.00E+00	
**Fluidized Bed Flow Ratio[Q_R]**	0.0122			
**Filter Status**	ANALYZED	Sensitivity - High mag	6.0E+04	1/g
H7000FA		Sensitivity - PCME (high+low mag)	6.0E+04	1/g

ANALYSIS INFORMATION

**Lab Name**	LabCor Inc
**Lab Sample Number**	150099-S5
**Lab QC Type**	NOT QC
**TEM Prep Method**	Direct
**Est. particulate loading on filter**	NA

Recording Rules:

Initial (high mag)

Min. AR	Min length (um)	Min width (um)
3:1	0	0

PCME Supplemental (low mag)

Min. AR	Min length (um)	Min width (um)
3:1	0	0

Stopping Rules:

Target S (1/g)	Max Area (mm^2)	Max N

RESULTS SUMMARY

Structure Metric	Observed Structure Count (s)	Structure Filter Loading (s/mm^2)	Soil Conc. Structures per gram (s/g)
**High mag only**			
Total Asbestos	4	3.9E+00	2.4E+05
Total Chrysotile (CH)	3	2.9E+00	1.8E+05
Total Amphibole	1	9.7E-01	6.0E+04
actinolite (AC)	0	0.0E+00	0.0E+00
amosite (AM)	0	0.0E+00	0.0E+00
anthophyllite (AN)	0	0.0E+00	0.0E+00
crocidolite (CR)	0	0.0E+00	0.0E+00
tremolite (TR)	1	9.7E-01	6.0E+04
winchite/richterite/tremolite/actinolite (WRTA)	0	0.0E+00	0.0E+00
other amphibole (OA)	0	0.0E+00	0.0E+00
other mineral class (OM)	0	0.0E+00	0.0E+00
**PCME (high+low mag)**			
Total Asbestos	0	0.0E+00	0.0E+00
Total Chrysotile (CH)	0	0.0E+00	0.0E+00
Total Amphibole	0	0.0E+00	0.0E+00
actinolite (AC)	0	0.0E+00	0.0E+00
amosite (AM)	0	0.0E+00	0.0E+00
anthophyllite (AN)	0	0.0E+00	0.0E+00
crocidolite (CR)	0	0.0E+00	0.0E+00
tremolite (TR)	0	0.0E+00	0.0E+00
winchite/richterite/tremolite/actinolite (WRTA)	0	0.0E+00	0.0E+00
other amphibole (OA)	0	0.0E+00	0.0E+00
other mineral class (OM)	0	0.0E+00	0.0E+00

	Σ Asbestos Structure Mass (g)	Mass fraction (as %)
**Total TEM**		
WRTA	0.00E+00	0.0000%
OA	0.00E+00	0.0000%
CH	2.84E-10	0.1697%
AC	0.00E+00	0.0000%
AM	0.00E+00	0.0000%
AN	0.00E+00	0.0000%
CR	0.00E+00	0.0000%
TR	4.36E-14	0.0000%
**PCME**		
WRTA	0.00E+00	0.0000%
OA	0.00E+00	0.0000%
CH	0.00E+00	0.0000%
AC	0.00E+00	0.0000%
AM	0.00E+00	0.0000%
AN	0.00E+00	0.0000%
CR	0.00E+00	0.0000%
TR	0.00E+00	0.0000%

Asbestos structure mass = Length • Width^2 • Density • 1E-12

LA Density = 3.1 g/cm^3
OA Density = 3.0 g/cm^3
CH Density = 2.6 g/cm^3
AC Density = 3.1 g/cm^3
AM Density = 3.5 g/cm^3
AN Density = 3.0 g/cm^3
CR Density = 3.3 g/cm^3
TR Density = 3.0 g/cm^3

Csoil (mass fraction, as %) = Σ Asbestos structure mass • Sensitivity • 100

Equations:

Filter Loading (s/mm^2) = N / (GOx • Ago)
Sensitivity (1/g) = EFA / (GOx • Ago • M • f • Q_R)
Csoil (s/g) = N • Sensitivity

Asbestos Types:

AC - actinolite
AM - amosite
AN - anthophyllite
CH - chrysotile
CR - crocidolite
TR - tremolite
LA - Libby amphibole
OA - other amphibole
NAM - non-asbestos material
OM - other mineral type (specify in "other mineral description" field)

Structure Types:

High mag: Length ≥ 0.5 um, Aspect Ratio ≥ 3:1
PCME: Length > 5 um, Width ≥ 0.25 um, Aspect Ratio ≥ 3:1

Sumas Mtn Asbestos Methods - FBAS_LabCor Inc_14394105_SO_02-25-15_150099_TEM-EPAISO__D.xlsm
FluidizedBedEDD_v1

TEM Asbestos Structure Count for Fluidized Bed Material -- ISO
ANALYTICAL REPORT

FILE NAME:Sumas Mtn Asbestos Methods - FBAS_LabCor Inc_14394105_SO_02-25-15_150099_TEM-EPAISO__D.xlsm

SAMPLE INFORMATION		ANALYSIS PARAMETERS		
**Field Sample Number**	14394105	**EFA (mm^2)**	385	
		**GO area (mm^2) [Ago]**	0.0104	
**Matrix**	Soil	**# GOs counted - high mag**	100	
**Field QC Type**	Not QC	**# GOs counted - low mag only**	0	
**Sample Mass (g dry wt) [M]**	0.50	**F-factor [f]**	1.00E+00	
**Fluidized Bed Flow Ratio[Q_R]**	0.0122			
**Filter Status**	ANALYZED	Sensitivity - High mag	6.2E+04	1/g
		Sensitivity - PCME (high+low mag)	6.2E+04	1/g

ANALYSIS INFORMATION

**Lab Name**	LabCor Inc
**Lab Sample Number**	150099-S6
**Lab QC Type**	NOT QC
**TEM Prep Method**	Direct
**Est. particulate loading on filter**	NA

Recording Rules:

Initial (high mag)

Min. AR	Min length (um)	Min width (um)
3:1	0	0

PCME Supplemental (low mag)

Min. AR	Min length (um)	Min width (um)
3:1	0	0

Stopping Rules:

Target S (1/g)	Max Area (mm^2)	Max N

RESULTS SUMMARY

Structure Metric	Observed Structure Count (s)	Structure Filter Loading (s/mm^2)	Soil Conc. Structures per gram (s/g)
**High mag only**			
Total Asbestos	2	1.9E+00	1.2E+05
Total Chrysotile (CH)	2	1.9E+00	1.2E+05
Total Amphibole	0	0.0E+00	0.0E+00
actinolite (AC)	0	0.0E+00	0.0E+00
amosite (AM)	0	0.0E+00	0.0E+00
anthophyllite (AN)	0	0.0E+00	0.0E+00
crocidolite (CR)	0	0.0E+00	0.0E+00
tremolite (TR)	0	0.0E+00	0.0E+00
winchite/richterite/tremolite/actinolite (WRTA)	0	0.0E+00	0.0E+00
other amphibole (OA)	0	0.0E+00	0.0E+00
other mineral class (OM)	0	0.0E+00	0.0E+00
**PCME (high+low mag)**			
Total Asbestos	0	0.0E+00	0.0E+00
Total Chrysotile (CH)	0	0.0E+00	0.0E+00
Total Amphibole	0	0.0E+00	0.0E+00
actinolite (AC)	0	0.0E+00	0.0E+00
amosite (AM)	0	0.0E+00	0.0E+00
anthophyllite (AN)	0	0.0E+00	0.0E+00
crocidolite (CR)	0	0.0E+00	0.0E+00
tremolite (TR)	0	0.0E+00	0.0E+00
winchite/richterite/tremolite/actinolite (WRTA)	0	0.0E+00	0.0E+00
other amphibole (OA)	0	0.0E+00	0.0E+00
other mineral class (OM)	0	0.0E+00	0.0E+00

Σ Asbestos Structure Mass (g)		Mass fraction (as %)
**Total TEM**		
WRTA	0.00E+00	0.0000%
OA	0.00E+00	0.0000%
CH	2.21E-14	0.0000%
AC	0.00E+00	0.0000%
AM	0.00E+00	0.0000%
AN	0.00E+00	0.0000%
CR	0.00E+00	0.0000%
TR	0.00E+00	0.0000%
**PCME**		
WRTA	0.00E+00	0.0000%
OA	0.00E+00	0.0000%
CH	0.00E+00	0.0000%
AC	0.00E+00	0.0000%
AM	0.00E+00	0.0000%
AN	0.00E+00	0.0000%
CR	0.00E+00	0.0000%
TR	0.00E+00	0.0000%

Asbestos structure mass = Length • Width^2 • Density • 1E-12

LA Density = 3.1 g/cm^3
OA Density = 3.0 g/cm^3
CH Density = 2.6 g/cm^3
AC Density = 3.1 g/cm^3
AM Density = 3.5 g/cm^3
AN Density = 3.0 g/cm^3
CR Density = 3.3 g/cm^3
TR Density = 3.0 g/cm^3

Csoil (mass fraction, as %) = Σ Asbestos structure mass • Sensitivity • 100

Equations:

Filter Loading (s/mm^2) = N / (GOx • Ago)
Sensitivity (1/g) = EFA / (GOx • Ago • M • f • Q_R)
Csoil (s/g) = N • Sensitivity

Asbestos Types:

AC - actinolite
AM - amosite
AN - anthophyllite
CH - chrysotile
CR - crocidolite
TR - tremolite
LA - Libby amphibole
OA - other amphibole
NAM - non-asbestos material
OM - other mineral type (specify in "other mineral description" field)

Structure Types:

High mag: Length ≥ 0.5 um, Aspect Ratio ≥ 3:1
PCME: Length > 5 um, Width ≥ 0.25 um, Aspect Ratio ≥ 3:1

Sumas Mtn Asbestos Methods - FBAS_Labcor Inc_14394106_SO_02-25-15_150099_TEM-EPAISO__D.xlsm
FluidizedBedEDD_v1

TEM Asbestos Structure Count for Fluidized Bed Material -- ISO
ANALYTICAL REPORT

FILE NAME:Sumas Mtn Asbestos Methods - FBAS_Labcor Inc_14394106_SO_02-25-15_150099_TEM-EPAISO__D.xlsm

SAMPLE INFORMATION		ANALYSIS PARAMETERS		
**Field Sample Number**	14394106	**EFA (mm^2)**	385	
		**GO area (mm^2) [Ago]**	0.0104	
**Matrix**	Soil	**# GOs counted - high mag**	100	
**Field QC Type**	Not QC	**# GOs counted - low mag only**	0	
**Sample Mass (g dry wt) [M]**	0.50	**F-factor [f]**	1.00E+00	
**Fluidized Bed Flow Ratio[Q_R]**	0.0122			
**Filter Status**	ANALYZED	Sensitivity - High mag	6.1E+04	1/g
JOEL		Sensitivity - PCME (high+low mag)	6.1E+04	1/g

ANALYSIS INFORMATION

Lab Name Labcor Inc
Lab Sample Number 150099-S7
Lab QC Type NOT QC
TEM Prep Method Direct
Est. particulate loading on filter NA

Recording Rules:

Initial (high mag)

Min. AR	Min length (um)	Min width (um)
none		

PCME Supplemental (low mag)

Min. AR	Min length (um)	Min width (um)
none		

Stopping Rules:

Target S (1/g)	Max Area (mm^2)	Max N

RESULTS SUMMARY

Structure Metric	Observed Structure Count (s)	Structure Filter Loading (s/mm^2)	Soil Conc. Structures per gram (s/g)
**High mag only**			
Total Asbestos	22	2.1E+01	1.3E+06
Total Chrysotile (CH)	20	1.9E+01	1.2E+06
Total Amphibole	2	1.9E+00	1.2E+05
actinolite (AC)	0	0.0E+00	0.0E+00
amosite (AM)	0	0.0E+00	0.0E+00
anthophyllite (AN)	1	9.7E-01	6.1E+04
crocidolite (CR)	0	0.0E+00	0.0E+00
tremolite (TR)	1	9.7E-01	6.1E+04
winchite/richterite/tremolite/actinolite (WRTA)	0	0.0E+00	0.0E+00
other amphibole (OA)	0	0.0E+00	0.0E+00
other mineral class (OM)	0	0.0E+00	0.0E+00
**PCME (high+low mag)**			
Total Asbestos	0	0.0E+00	0.0E+00
Total Chrysotile (CH)	0	0.0E+00	0.0E+00
Total Amphibole	0	0.0E+00	0.0E+00
actinolite (AC)	0	0.0E+00	0.0E+00
amosite (AM)	0	0.0E+00	0.0E+00
anthophyllite (AN)	0	0.0E+00	0.0E+00
crocidolite (CR)	0	0.0E+00	0.0E+00
tremolite (TR)	0	0.0E+00	0.0E+00
winchite/richterite/tremolite/actinolite (WRTA)	0	0.0E+00	0.0E+00
other amphibole (OA)	0	0.0E+00	0.0E+00
other mineral class (OM)	0	0.0E+00	0.0E+00

Σ Asbestos Structure Mass (g)		Mass fraction (as %)
**Total TEM**		
WRTA	0.00E+00	0.0000%
OA	0.00E+00	0.0000%
CH	3.67E-09	2.2404%
AC	0.00E+00	0.0000%
AM	0.00E+00	0.0000%
AN	1.05E-11	0.0064%
CR	0.00E+00	0.0000%
TR	2.68E-09	1.6326%
**PCME**		
WRTA	0.00E+00	0.0000%
OA	0.00E+00	0.0000%
CH	6.63E-10	0.4041%
AC	0.00E+00	0.0000%
AM	0.00E+00	0.0000%
AN	#N/A	#N/A
CR	0.00E+00	0.0000%
TR	0.00E+00	0.0000%

Asbestos structure mass = Length • Width^2 • Density • 1E-12

LA Density = 3.1 g/cm^3
OA Density = 3.0 g/cm^3
CH Density = 2.6 g/cm^3
AC Density = 3.1 g/cm^3
AM Density = 3.5 g/cm^3
AN Density = 3.0 g/cm^3
CR Density = 3.3 g/cm^3
TR Density = 3.0 g/cm^3

Csoil (mass fraction, as %) = Σ Asbestos structure mass • Sensitivity • 100

Equations:
Filter Loading (s/mm^2) = N / (GOx • Ago)
Sensitivity (1/g) = EFA / (GOx • Ago • M • f • Q_R)
Csoil (s/g) = N • Sensitivity

Asbestos Types:
AC - actinolite
AM - amosite
AN - anthophyllite
CH - chrysotile
CR - crocidolite
TR - tremolite
LA - Libby amphibole
OA - other amphibole
NAM - non-asbestos material
OM - other mineral type (specify in "other mineral description" field)

Structure Types:
High mag: Length ≥ 0.5 um, Aspect Ratio ≥ 3:1
PCME: Length > 5 um, Width ≥ 0.25 um, Aspect Ratio ≥ 3:1

Sumas Mtn Asbestos Methods - FBAS_Labcor Inc_14394107_SO_02-27-15_150099_TEM-EPAISO__D.xlsm
FluidizedBedEDD_v1

TEM Asbestos Structure Count for Fluidized Bed Material -- ISO
ANALYTICAL REPORT

FILE NAME:Sumas Mtn Asbestos Methods - FBAS_Labcor Inc_14394107_SO_02-27-15_150099_TEM-EPAISO__D.xlsm

SAMPLE INFORMATION		ANALYSIS PARAMETERS		
**Field Sample Number**	14394107	**EFA (mm^2)**	385	
		**GO area (mm^2) [Ago]**	0.0104	
**Matrix**	Soil	**# GOs counted - high mag**	100	
**Field QC Type**	Not QC	**# GOs counted - low mag only**	0	
**Sample Mass (g dry wt) [M]**	0.51	**F-factor [f]**	1.00E+00	
**Fluidized Bed Flow Ratio[Q_R]**	0.0122			
**Filter Status**	ANALYZED	Sensitivity - High mag	6.0E+04	1/g
JOEL		Sensitivity - PCME (high+low mag)	6.0E+04	1/g

ANALYSIS INFORMATION

Lab Name Labcor Inc
Lab Sample Number 150099-S8
Lab QC Type NOT QC
TEM Prep Method Direct
Est. particulate loading on filter NA

Recording Rules:

Initial (high mag)

Min. AR	Min length (um)	Min width (um)
3:1	0	0

PCME Supplemental (low mag)

Min. AR	Min length (um)	Min width (um)
3:1	0	0

Stopping Rules:

Target S (1/g)	Max Area (mm^2)	Max N

RESULTS SUMMARY

Structure Metric	Observed Structure Count (s)	Structure Filter Loading (s/mm^2)	Soil Conc. Structures per gram (s/g)
**High mag only**			
Total Asbestos	24	2.3E+01	1.4E+06
Total Chrysotile (CH)	18	1.7E+01	1.1E+06
Total Amphibole	6	5.8E+00	3.6E+05
actinolite (AC)	5	4.8E+00	3.0E+05
amosite (AM)	1	9.7E-01	6.0E+04
anthophyllite (AN)	0	0.0E+00	0.0E+00
crocidolite (CR)	0	0.0E+00	0.0E+00
tremolite (TR)	0	0.0E+00	0.0E+00
winchite/richterite/tremolite/actinolite (WRTA)	0	0.0E+00	0.0E+00
other amphibole (OA)	0	0.0E+00	0.0E+00
other mineral class (OM)	0	0.0E+00	0.0E+00
**PCME (high+low mag)**			
Total Asbestos	1	9.7E-01	6.0E+04
Total Chrysotile (CH)	1	9.7E-01	6.0E+04
Total Amphibole	0	0.0E+00	0.0E+00
actinolite (AC)	0	0.0E+00	0.0E+00
amosite (AM)	0	0.0E+00	0.0E+00
anthophyllite (AN)	0	0.0E+00	0.0E+00
crocidolite (CR)	0	0.0E+00	0.0E+00
tremolite (TR)	0	0.0E+00	0.0E+00
winchite/richterite/tremolite/actinolite (WRTA)	0	0.0E+00	0.0E+00
other amphibole (OA)	0	0.0E+00	0.0E+00
other mineral class (OM)	0	0.0E+00	0.0E+00

JT CC

Σ Asbestos Structure Mass (g)		Mass fraction (as %)
**Total TEM**		
WRTA	0.00E+00	0.0000%
OA	0.00E+00	0.0000%
CH	1.38E-09	0.8301%
AC	1.60E-08	9.5978%
AM	1.94E-11	0.0116%
AN	0.00E+00	0.0000%
CR	0.00E+00	0.0000%
TR	0.00E+00	0.0000%
**PCME**		
WRTA	0.00E+00	0.0000%
OA	0.00E+00	0.0000%
CH	1.10E-11	0.0066%
AC	#N/A	#N/A
AM	0.00E+00	0.0000%
AN	0.00E+00	0.0000%
CR	0.00E+00	0.0000%
TR	0.00E+00	0.0000%

Asbestos structure mass = Length • Width^2 • Density • 1E-12

LA Density = 3.1 g/cm^3
OA Density = 3.0 g/cm^3
CH Density = 2.6 g/cm^3
AC Density = 3.1 g/cm^3
AM Density = 3.5 g/cm^3
AN Density = 3.0 g/cm^3
CR Density = 3.3 g/cm^3
TR Density = 3.0 g/cm^3

Csoil (mass fraction, as %) = Σ Asbestos structure mass • Sensitivity • 100

Equations:
Filter Loading (s/mm^2) = N / (GOx • Ago)
Sensitivity (1/g) = EFA / (GOx • Ago • M • f • Q_R)
Csoil (s/g) = N • Sensitivity

Asbestos Types:
AC - actinolite
AM - amosite
AN - anthophyllite
CH - chrysotile
CR - crocidolite
TR - tremolite
LA - Libby amphibole
OA - other amphibole
NAM - non-asbestos material
OM - other mineral type (specify in "other mineral description" field)

Structure Types:
High mag: Length ≥ 0.5 um, Aspect Ratio ≥ 3:1
PCME: Length > 5 um, Width ≥ 0.25 um, Aspect Ratio ≥ 3:1

Sumas Mtn Asbestos Methods - FBAS_LabCor Inc_14394108_SO_02-28-15_150099_TEM-EPAISO__D.xlsm
FluidizedBedEDD_v1

TEM Asbestos Structure Count for Fluidized Bed Material -- ISO
ANALYTICAL REPORT

FILE NAME:Sumas Mtn Asbestos Methods - FBAS_LabCor Inc_14394108_SO_02-28-15_150099_TEM-EPAISO__D.xlsm

SAMPLE INFORMATION		ANALYSIS PARAMETERS		
**Field Sample Number**	14394108	**EFA (mm^2)**	385	
		**GO area (mm^2) [Ago]**	0.0104	
**Matrix**	Soil	**# GOs counted - high mag**	100	
**Field QC Type**	Not QC	**# GOs counted - low mag only**	0	
**Sample Mass (g dry wt) [M]**	0.50	**F-factor [f]**	1.00E+00	
**Fluidized Bed Flow Ratio[Q_R]**	0.0122			
**Filter Status**	ANALYZED	Sensitivity - High mag	6.1E+04	1/g
		Sensitivity - PCME (high+low mag)	6.1E+04	1/g

ANALYSIS INFORMATION

**Lab Name**	LabCor Inc
**Lab Sample Number**	150099-S9
**Lab QC Type**	NOT QC
**TEM Prep Method**	Direct
**Est. particulate loading on filter**	NA

Recording Rules:

Initial (high mag)

Min. AR	Min length (um)	Min width (um)
3:1	0	0

PCME Supplemental (low mag)

Min. AR	Min length (um)	Min width (um)
3:1	0	0

Stopping Rules:

Target S (1/g)	Max Area (mm^2)	Max N

RESULTS SUMMARY

Structure Metric	Observed Structure Count (s)	Structure Filter Loading (s/mm^2)	Soil Conc. Structures per gram (s/g)
**High mag only**			
Total Asbestos	0	0.0E+00	0.0E+00
Total Chrysotile (CH)	0	0.0E+00	0.0E+00
Total Amphibole	0	0.0E+00	0.0E+00
actinolite (AC)	0	0.0E+00	0.0E+00
amosite (AM)	0	0.0E+00	0.0E+00
anthophyllite (AN)	0	0.0E+00	0.0E+00
crocidolite (CR)	0	0.0E+00	0.0E+00
tremolite (TR)	0	0.0E+00	0.0E+00
winchite/richterite/tremolite/actinolite (WRTA)	0	0.0E+00	0.0E+00
other amphibole (OA)	0	0.0E+00	0.0E+00
other mineral class (OM)	0	0.0E+00	0.0E+00
**PCME (high+low mag)**			
Total Asbestos	0	0.0E+00	0.0E+00
Total Chrysotile (CH)	0	0.0E+00	0.0E+00
Total Amphibole	0	0.0E+00	0.0E+00
actinolite (AC)	0	0.0E+00	0.0E+00
amosite (AM)	0	0.0E+00	0.0E+00
anthophyllite (AN)	0	0.0E+00	0.0E+00
crocidolite (CR)	0	0.0E+00	0.0E+00
tremolite (TR)	0	0.0E+00	0.0E+00
winchite/richterite/tremolite/actinolite (WRTA)	0	0.0E+00	0.0E+00
other amphibole (OA)	0	0.0E+00	0.0E+00
other mineral class (OM)	0	0.0E+00	0.0E+00

Σ Asbestos Structure Mass (g)		Mass fraction (as %)
**Total TEM**		
WRTA	0.00E+00	0.0000%
OA	0.00E+00	0.0000%
CH	0.00E+00	0.0000%
AC	0.00E+00	0.0000%
AM	0.00E+00	0.0000%
AN	0.00E+00	0.0000%
CR	0.00E+00	0.0000%
TR	0.00E+00	0.0000%
[illegible]		
WRTA	0.00E+00	0.0000%
OA	0.00E+00	0.0000%
CH	0.00E+00	0.0000%
AC	0.00E+00	0.0000%
AM	0.00E+00	0.0000%
AN	0.00E+00	0.0000%
CR	0.00E+00	0.0000%
TR	0.00E+00	0.0000%

Asbestos structure mass = Length • $Width^2$ • Density • 1E-12

LA Density = 3.1 g/cm^3
OA Density = 3.0 g/cm^3
CH Density = 2.6 g/cm^3
AC Density = 3.1 g/cm^3
AM Density = 3.5 g/cm^3
AN Density = 3.0 g/cm^3
CR Density = 3.3 g/cm^3
TR Density = 3.0 g/cm^3

Csoil (mass fraction, as %) = Σ Asbestos structure mass • Sensitivity • 100

Equations:

Filter Loading (s/mm^2) = N / (GOx • Ago)
Sensitivity (1/g) = EFA / (GOx • Ago • M • f • Q_R)
Csoil (s/g) = N • Sensitivity

Asbestos Types:

AC - actinolite
AM - amosite
AN - anthophyllite
CH - chrysotile
CR - crocidolite
TR - tremolite
LA - Libby amphibole
OA - other amphibole
NAM - non-asbestos material
OM - other mineral type (specify in "other mineral description" field)

Structure Types:

High mag: Length ≥ 0.5 um, Aspect Ratio ≥ 3:1
PCME: Length > 5 um, Width ≥ 0.25 um, Aspect Ratio ≥ 3:1

Sumas Mtn Asbestos Methods - FBAS_LabCor Inc_14394109_SO_03-03-15_150099_TEM-EPAISO__D.xlsm
FluidizedBedEDD_v1

TEM Asbestos Structure Count for Fluidized Bed Material -- ISO
ANALYTICAL REPORT

FILE NAME:Sumas Mtn Asbestos Methods - FBAS_LabCor Inc_14394109_SO_03-03-15_150099_TEM-EPAISO__D.xlsm

SAMPLE INFORMATION		ANALYSIS PARAMETERS		
**Field Sample Number**	14394109	**EFA (mm^2)**	385	
		**GO area (mm^2) [Ago]**	0.0104	
**Matrix**	Soil	**# GOs counted - high mag**	100	
**Field QC Type**	Not QC	**# GOs counted - low mag only**	0	
**Sample Mass (g dry wt) [M]**	0.50	**F-factor [f]**	1.00E+00	
**Fluidized Bed Flow Ratio[Q_R]**	0.0122			
**Filter Status**	ANALYZED	Sensitivity - High mag	6.1E+04	1/g
H7000FA		Sensitivity - PCME (high+low mag)	6.1E+04	1/g

ANALYSIS INFORMATION

**Lab Name**	LabCor Inc
**Lab Sample Number**	150099-S10
**Lab QC Type**	NOT QC
**TEM Prep Method**	Direct
**Est. particulate loading on filter**	NA

Recording Rules:

Initial (high mag)

Min. AR	Min length (um)	Min width (um)
3:1	0	0

PCME Supplemental (low mag)

Min. AR	Min length (um)	Min width (um)
3:1	0	0

Stopping Rules:

Target S (1/g)	Max Area (mm^2)	Max N

RESULTS SUMMARY

Structure Metric	Observed Structure Count (s)	Structure Filter Loading (s/mm^2)	Soil Conc. Structures per gram (s/g)
**High mag only**			
Total Asbestos	9	8.7E+00	5.5E+05
Total Chrysotile (CH)	7	6.8E+00	4.3E+05
Total Amphibole	2	1.9E+00	1.2E+05
actinolite (AC)	1	9.7E-01	6.1E+04
amosite (AM)	0	0.0E+00	0.0E+00
anthophyllite (AN)	0	0.0E+00	0.0E+00
crocidolite (CR)	0	0.0E+00	0.0E+00
tremolite (TR)	1	9.7E-01	6.1E+04
winchite/richterite/tremolite/actinolite (WRTA)	0	0.0E+00	0.0E+00
other amphibole (OA)	0	0.0E+00	0.0E+00
other mineral class (OM)	0	0.0E+00	0.0E+00
**PCME (high+low mag)**			
Total Asbestos	0	0.0E+00	0.0E+00
Total Chrysotile (CH)	0	0.0E+00	0.0E+00
Total Amphibole	0	0.0E+00	0.0E+00
actinolite (AC)	0	0.0E+00	0.0E+00
amosite (AM)	0	0.0E+00	0.0E+00
anthophyllite (AN)	0	0.0E+00	0.0E+00
crocidolite (CR)	0	0.0E+00	0.0E+00
tremolite (TR)	0	0.0E+00	0.0E+00
winchite/richterite/tremolite/actinolite (WRTA)	0	0.0E+00	0.0E+00
other amphibole (OA)	0	0.0E+00	0.0E+00
other mineral class (OM)	0	0.0E+00	0.0E+00

	Σ Asbestos Structure Mass (g)	Mass fraction (as %)
**Total TEM**		
WRTA	0.00E+00	0.0000%
OA	0.00E+00	0.0000%
CH	2.18E-08	13.2946%
AC	3.75E-11	0.0228%
AM	0.00E+00	0.0000%
AN	0.00E+00	0.0000%
CR	0.00E+00	0.0000%
TR	1.99E-09	1.2110%
**PCME**		
WRTA	0.00E+00	0.0000%
OA	0.00E+00	0.0000%
CH	0.00E+00	0.0000%
AC	#N/A	#N/A
AM	0.00E+00	0.0000%
AN	0.00E+00	0.0000%
CR	0.00E+00	0.0000%
TR	0.00E+00	0.0000%

Asbestos structure mass = Length • Width^2 • Density • 1E-12

LA Density = 3.1 g/cm^3
OA Density = 3.0 g/cm^3
CH Density = 2.6 g/cm^3
AC Density = 3.1 g/cm^3
AM Density = 3.5 g/cm^3
AN Density = 3.0 g/cm^3
CR Density = 3.3 g/cm^3
TR Density = 3.0 g/cm^3

Csoil (mass fraction, as %) = Σ Asbestos structure mass • Sensitivity • 100

Equations:

Filter Loading (s/mm^2) = N / (GOx • Ago)
Sensitivity (1/g) = EFA / (GOx • Ago • M • f • Q_R)
Csoil (s/g) = N • Sensitivity

Asbestos Types:

AC - actinolite
AM - amosite
AN - anthophyllite
CH - chrysotile
CR - crocidolite
TR - tremolite
LA - Libby amphibole
OA - other amphibole
NAM - non-asbestos material
OM - other mineral type (specify in "other mineral description" field)

Structure Types:

High mag: Length ≥ 0.5 um, Aspect Ratio ≥ 3:1
PCME: Length > 5 um, Width ≥ 0.25 um, Aspect Ratio ≥ 3:1

Sumas Mtn Asbestos Methods - FBAS_LabCor Inc_14394110_SO_03-03-15_150099_TEM-EPAISO__D.xlsm
FluidizedBedEDD_v1

TEM Asbestos Structure Count for Fluidized Bed Material -- ISO
ANALYTICAL REPORT

FILE NAME:Sumas Mtn Asbestos Methods - FBAS_LabCor Inc_14394110_SO_03-03-15_150099_TEM-EPAISO__D.xlsm

SAMPLE INFORMATION		ANALYSIS PARAMETERS		
**Field Sample Number**	14394110	**EFA (mm^2)**	385	
		**GO area (mm^2) [Ago]**	0.0104	
**Matrix**	Soil	**# GOs counted - high mag**	100	
**Field QC Type**	Not QC	**# GOs counted - low mag only**	0	
**Sample Mass (g dry wt) [M]**	0.51	**F-factor [f]**	1.00E+00	
**Fluidized Bed Flow Ratio[Q_R]**	0.0122			
**Filter Status**	ANALYZED	Sensitivity - High mag	6.0E+04	1/g
		Sensitivity - PCME (high+low mag)	6.0E+04	1/g

ANALYSIS INFORMATION

**Lab Name**	LabCor Inc
**Lab Sample Number**	15099-S11
**Lab QC Type**	NOT QC
**TEM Prep Method**	Direct
**Est. particulate loading on filter**	NA

Recording Rules:

Initial (high mag)

Min. AR	Min length (um)	Min width (um)
3:1	0	0

PCME Supplemental (low mag)

Min. AR	Min length (um)	Min width (um)
3:1	0	0

Stopping Rules:

Target S (1/g)	Max Area (mm^2)	Max N

RESULTS SUMMARY

Structure Metric	Observed Structure Count (s)	Structure Filter Loading (s/mm^2)	Soil Conc. Structures per gram (s/g)
**High mag only**			
Total Asbestos	3	2.9E+00	1.8E+05
Total Chrysotile (CH)	3	2.9E+00	1.8E+05
Total Amphibole	0	0.0E+00	0.0E+00
actinolite (AC)	0	0.0E+00	0.0E+00
amosite (AM)	0	0.0E+00	0.0E+00
anthophyllite (AN)	0	0.0E+00	0.0E+00
crocidolite (CR)	0	0.0E+00	0.0E+00
tremolite (TR)	0	0.0E+00	0.0E+00
winchite/richterite/tremolite/actinolite (WRTA)	0	0.0E+00	0.0E+00
other amphibole (OA)	0	0.0E+00	0.0E+00
other mineral class (OM)	0	0.0E+00	0.0E+00
**PCME (high+low mag)**			
Total Asbestos	0	0.0E+00	0.0E+00
Total Chrysotile (CH)	0	0.0E+00	0.0E+00
Total Amphibole	0	0.0E+00	0.0E+00
actinolite (AC)	0	0.0E+00	0.0E+00
amosite (AM)	0	0.0E+00	0.0E+00
anthophyllite (AN)	0	0.0E+00	0.0E+00
crocidolite (CR)	0	0.0E+00	0.0E+00
tremolite (TR)	0	0.0E+00	0.0E+00
winchite/richterite/tremolite/actinolite (WRTA)	0	0.0E+00	0.0E+00
other amphibole (OA)	0	0.0E+00	0.0E+00
other mineral class (OM)	0	0.0E+00	0.0E+00

Σ Asbestos Structure Mass (g)		Mass fraction (as %)
**Total TEM**		
WRTA	0.00E+00	0.0000%
OA	0.00E+00	0.0000%
CH	1.04E-12	0.0006%
AC	0.00E+00	0.0000%
AM	0.00E+00	0.0000%
AN	0.00E+00	0.0000%
CR	0.00E+00	0.0000%
TR	0.00E+00	0.0000%
**PCME**		
WRTA	0.00E+00	0.0000%
OA	0.00E+00	0.0000%
CH	0.00E+00	0.0000%
AC	0.00E+00	0.0000%
AM	0.00E+00	0.0000%
AN	0.00E+00	0.0000%
CR	0.00E+00	0.0000%
TR	0.00E+00	0.0000%

Asbestos structure mass = Length • $Width^2$ • Density • 1E-12

LA Density = 3.1 g/cm^3
OA Density = 3.0 g/cm^3
CH Density = 2.6 g/cm^3
AC Density = 3.1 g/cm^3
AM Density = 3.5 g/cm^3
AN Density = 3.0 g/cm^3
CR Density = 3.3 g/cm^3
TR Density = 3.0 g/cm^3

Csoil (mass fraction, as %) = Σ Asbestos structure mass • Sensitivity • 100

Equations:

Filter Loading (s/mm^2) = N / (GOx • Ago)
Sensitivity (1/g) = EFA / (GOx • Ago • M • f • Q_R)
Csoil (s/g) = N • Sensitivity

Asbestos Types:

AC - actinolite
AM - amosite
AN - anthophyllite
CH - chrysotile
CR - crocidolite
TR - tremolite
LA - Libby amphibole
OA - other amphibole
NAM - non-asbestos material
OM - other mineral type (specify in "other mineral description" field)

Structure Types:

High mag: Length ≥ 0.5 um, Aspect Ratio ≥ 3:1
PCME: Length > 5 um, Width ≥ 0.25 um, Aspect Ratio ≥ 3:1

Sumas Mtn Asbestos Methods - FBAS_LabCor Inc_14394111_SO_03-03-15_150099_TEM-EPAISO__D.xlsm
FluidizedBedEDD_v1

TEM Asbestos Structure Count for Fluidized Bed Material -- ISO
ANALYTICAL REPORT

FILE NAME:Sumas Mtn Asbestos Methods - FBAS_LabCor Inc_14394111_SO_03-03-15_150099_TEM-EPAISO__D.xlsm

SAMPLE INFORMATION		ANALYSIS PARAMETERS		
**Field Sample Number**	14394111	**EFA (mm^2)**	385	
		**GO area (mm^2) [Ago]**	0.0104	
**Matrix**	Soil	**# GOs counted - high mag**	100	
**Field QC Type**	Not QC	**# GOs counted - low mag only**	0	
**Sample Mass (g dry wt) [M]**	0.51	**F-factor [f]**	1.00E+00	
**Fluidized Bed Flow Ratio[Q_R]**	0.0122			
**Filter Status**	ANALYZED	Sensitivity - High mag	5.9E+04	1/g
		Sensitivity - PCME (high+low mag)	5.9E+04	1/g

ANALYSIS INFORMATION

**Lab Name**	LabCor Inc
**Lab Sample Number**	150099-S12
**Lab QC Type**	NOT QC
**TEM Prep Method**	Direct
**Est. particulate loading on filter**	NA

Recording Rules:

Initial (high mag)

Min. AR	Min length (um)	Min width (um)
none		

PCME Supplemental (low mag)

Min. AR	Min length (um)	Min width (um)
none		

Stopping Rules:

Target S (1/g)	Max Area (mm^2)	Max N

RESULTS SUMMARY

Structure Metric	Observed Structure Count (s)	Structure Filter Loading (s/mm^2)	Soil Conc. Structures per gram (s/g)
**High mag only**			
Total Asbestos	24	2.3E+01	1.4E+06
Total Chrysotile (CH)	19	1.8E+01	1.1E+06
Total Amphibole	5	4.8E+00	3.0E+05
actinolite (AC)	2	1.9E+00	1.2E+05
amosite (AM)	0	0.0E+00	0.0E+00
anthophyllite (AN)	0	0.0E+00	0.0E+00
crocidolite (CR)	0	0.0E+00	0.0E+00
tremolite (TR)	3	2.9E+00	1.8E+05
winchite/richterite/tremolite/actinolite (WRTA)	0	0.0E+00	0.0E+00
other amphibole (OA)	0	0.0E+00	0.0E+00
other mineral class (OM)	0	0.0E+00	0.0E+00
**PCME (high+low mag)**			
Total Asbestos	0	0.0E+00	0.0E+00
Total Chrysotile (CH)	0	0.0E+00	0.0E+00
Total Amphibole	0	0.0E+00	0.0E+00
actinolite (AC)	0	0.0E+00	0.0E+00
amosite (AM)	0	0.0E+00	0.0E+00
anthophyllite (AN)	0	0.0E+00	0.0E+00
crocidolite (CR)	0	0.0E+00	0.0E+00
tremolite (TR)	0	0.0E+00	0.0E+00
winchite/richterite/tremolite/actinolite (WRTA)	0	0.0E+00	0.0E+00
other amphibole (OA)	0	0.0E+00	0.0E+00
other mineral class (OM)	0	0.0E+00	0.0E+00

Σ Asbestos Structure Mass (g)		Mass fraction (as %)
**Total TEM**		
WRTA	0.00E+00	0.0000%
OA	0.00E+00	0.0000%
CH	4.12E-09	2.4469%
AC	2.30E-10	0.1363%
AM	0.00E+00	0.0000%
AN	0.00E+00	0.0000%
CR	0.00E+00	0.0000%
TR	3.67E-09	2.1772%
**PCME**		
WRTA	0.00E+00	0.0000%
OA	0.00E+00	0.0000%
CH	8.24E-12	0.0049%
AC	0.00E+00	0.0000%
AM	0.00E+00	0.0000%
AN	0.00E+00	0.0000%
CR	0.00E+00	0.0000%
TR	0.00E+00	0.0000%

Asbestos structure mass = Length • Width^2 • Density • 1E-12

LA Density = 3.1 g/cm^3
OA Density = 3.0 g/cm^3
CH Density = 2.6 g/cm^3
AC Density = 3.1 g/cm^3
AM Density = 3.5 g/cm^3
AN Density = 3.0 g/cm^3
CR Density = 3.3 g/cm^3
TR Density = 3.0 g/cm^3

Csoil (mass fraction, as %) = Σ Asbestos structure mass • Sensitivity • 100

Equations:

Filter Loading (s/mm^2) = N / (GOx • Ago)
Sensitivity (1/g) = EFA / (GOx • Ago • M • f • Q_R)
Csoil (s/g) = N • Sensitivity

Asbestos Types:

AC - actinolite
AM - amosite
AN - anthophyllite
CH - chrysotile
CR - crocidolite
TR - tremolite
LA - Libby amphibole
OA - other amphibole
NAM - non-asbestos material
OM - other mineral type (specify in "other mineral description" field)

Structure Types:

High mag: Length ≥ 0.5 um, Aspect Ratio ≥ 3:1
PCME: Length > 5 um, Width ≥ 0.25 um, Aspect Ratio ≥ 3:1

Sumas Mtn Asbestos Methods - FBAS_LabCor Inc_14394112_SO_03-04-15_150099_TEM-EPAISO__D.xlsm
FluidizedBedEDD_v1

TEM Asbestos Structure Count for Fluidized Bed Material -- ISO
ANALYTICAL REPORT

FILE NAME:Sumas Mtn Asbestos Methods - FBAS_LabCor Inc_14394112_SO_03-04-15_150099_TEM-EPAISO__D.xlsm

SAMPLE INFORMATION		ANALYSIS PARAMETERS		
**Field Sample Number**	14394112	**EFA (mm^2)**	385	
		**GO area (mm^2) [Ago]**	0.0104	
**Matrix**	Soil	**# GOs counted - high mag**	100	
**Field QC Type**	Not QC	**# GOs counted - low mag only**	0	
**Sample Mass (g dry wt) [M]**	0.51	**F-factor [f]**	1.00E+00	
**Fluidized Bed Flow Ratio[Q_R]**	0.0122			
**Filter Status**	ANALYZED	Sensitivity - High mag	6.0E+04	1/g
H7000FA		Sensitivity - PCME (high+low mag)	6.0E+04	1/g

ANALYSIS INFORMATION

**Lab Name**	LabCor Inc
**Lab Sample Number**	150099-S13
**Lab QC Type**	NOT QC
**TEM Prep Method**	Direct
**Est. particulate loading on filter**	NA

Recording Rules:

Initial (high mag)

Min. AR	Min length (um)	Min width (um)
3:1	0	0

PCME Supplemental (low mag)

Min. AR	Min length (um)	Min width (um)
3:1	0	0

Stopping Rules:

Target S (1/g)	Max Area (mm^2)	Max N

RESULTS SUMMARY

Structure Metric	Observed Structure Count (s)	Structure Filter Loading (s/mm^2)	Soil Conc. Structures per gram (s/g)
**High mag only**			
Total Asbestos	18	1.7E+01	1.1E+06
Total Chrysotile (CH)	13	1.3E+01	7.8E+05
Total Amphibole	5	4.8E+00	3.0E+05
actinolite (AC)	2	1.9E+00	1.2E+05
amosite (AM)	0	0.0E+00	0.0E+00
anthophyllite (AN)	0	0.0E+00	0.0E+00
crocidolite (CR)	0	0.0E+00	0.0E+00
tremolite (TR)	3	2.9E+00	1.8E+05
winchite/richterite/tremolite/actinolite (WRTA)	0	0.0E+00	0.0E+00
other amphibole (OA)	0	0.0E+00	0.0E+00
other mineral class (OM)	0	0.0E+00	0.0E+00
**PCME (high+low mag)**			
Total Asbestos	0	0.0E+00	0.0E+00
Total Chrysotile (CH)	0	0.0E+00	0.0E+00
Total Amphibole	0	0.0E+00	0.0E+00
actinolite (AC)	0	0.0E+00	0.0E+00
amosite (AM)	0	0.0E+00	0.0E+00
anthophyllite (AN)	0	0.0E+00	0.0E+00
crocidolite (CR)	0	0.0E+00	0.0E+00
tremolite (TR)	0	0.0E+00	0.0E+00
winchite/richterite/tremolite/actinolite (WRTA)	0	0.0E+00	0.0E+00
other amphibole (OA)	0	0.0E+00	0.0E+00
other mineral class (OM)	0	0.0E+00	0.0E+00

	Σ Asbestos Structure Mass (g)	Mass fraction (as %)
**Total TEM**		
WRTA	0.00E+00	0.0000%
OA	0.00E+00	0.0000%
CH	1.23E-09	0.7315%
AC	1.07E-09	0.6405%
AM	0.00E+00	0.0000%
AN	0.00E+00	0.0000%
CR	0.00E+00	0.0000%
TR	1.79E-11	0.0107%
**PCME**		
WRTA	0.00E+00	0.0000%
OA	0.00E+00	0.0000%
CH	0.00E+00	0.0000%
AC	0.00E+00	0.0000%
AM	0.00E+00	0.0000%
AN	0.00E+00	0.0000%
CR	0.00E+00	0.0000%
TR	#N/A	#N/A

Asbestos structure mass = Length • Width^2 • Density • 1E-12

LA Density = 3.1 g/cm^3
OA Density = 3.0 g/cm^3
CH Density = 2.6 g/cm^3
AC Density = 3.1 g/cm^3
AM Density = 3.5 g/cm^3
AN Density = 3.0 g/cm^3
CR Density = 3.3 g/cm^3
TR Density = 3.0 g/cm^3

Csoil (mass fraction, as %) = Σ Asbestos structure mass • Sensitivity • 100

Equations:

Filter Loading (s/mm^2) = N / (GOx • Ago)
Sensitivity (1/g) = EFA / (GOx • Ago • M • f • Q_R)
Csoil (s/g) = N • Sensitivity

Asbestos Types:

AC - actinolite
AM - amosite
AN - anthophyllite
CH - chrysotile
CR - crocidolite
TR - tremolite
LA - Libby amphibole
OA - other amphibole
NAM - non-asbestos material
OM - other mineral type (specify in "other mineral description" field)

Structure Types:

High mag: Length ≥ 0.5 um, Aspect Ratio ≥ 3:1
PCME: Length > 5 um, Width ≥ 0.25 um, Aspect Ratio ≥ 3:1

Sumas Mtn Asbestos Methods - FBAS_LabCor Inc_14394113_SO_03-04-15_150099_TEM-EPAISO__D xlsm
FluidizedBedEDD_v1

TEM Asbestos Structure Count for Fluidized Bed Material -- ISO
ANALYTICAL REPORT

FILE NAME:Sumas Mtn Asbestos Methods - FBAS_LabCor Inc_14394113_SO_03-04-15_150099_TEM-EPAISO__D.xlsm

SAMPLE INFORMATION		ANALYSIS PARAMETERS		
**Field Sample Number**	14394113	**EFA (mm^2)**	385	
		**GO area (mm^2) [Ago]**	0.0104	
**Matrix**	Soil	**# GOs counted - high mag**	100	
**Field QC Type**	Not QC	**# GOs counted - low mag only**	0	
**Sample Mass (g dry wt) [M]**	0 52	**F-factor [f]**	1.00E+00	
**Fluidized Bed Flow Ratio[Q_R]**	0.122			
**Filter Status**	ANALYZED	Sensitivity - High mag	5.9E+03	1/g
H7000FA		Sensitivity - PCME (high+low mag)	5.9E+03	1/g

ANALYSIS INFORMATION

Lab Name LabCor Inc
Lab Sample Number 150099-S14
Lab QC Type NOT QC
TEM Prep Method Direct
Est. particulate loading on filter NA

Recording Rules:

Initial (high mag)

Min. AR	Min length (um)	Min width (um)
3:1	0	0

PCME Supplemental (low mag)

Min AR	Min length (um)	Min width (um)
3:1	0	0

Stopping Rules:

Target S (1/g)	Max Area (mm^2)	Max N

RESULTS SUMMARY

Structure Metric	Observed Structure Count (s)	Structure Filter Loading (s/mm^2)	Soil Conc. Structures per gram (s/g)
**High mag only**			
Total Asbestos	16	1.5E+01	9.4E+04
Total Chrysotile (CH)	14	1.4E+01	8.2E+04
Total Amphibole	2	1.9E+00	1.2E+04
actinolite (AC)	0	0.0E+00	0.0E+00
amosite (AM)	0	0.0E+00	0.0E+00
anthophyllite (AN)	0	0.0E+00	0 0E+00
crocidolite (CR)	0	0.0E+00	0.0E+00
tremolite (TR)	1	9.7E-01	5.9E+03
winchite/richterite/tremolite/actinolite (WRTA)	0	0.0E+00	0.0E+00
other amphibole (OA)	1	9.7E-01	5.9E+03
other mineral class (OM)	0	0.0E+00	0.0E+00
**PCME (high+low mag)**			
Total Asbestos	4.225E-14	4.1E-14	2.5E-10
Total Chrysotile (CH)	0	0 0E+00	0.0E+00
Total Amphibole	4.225E-14	4.1E-14	2.5E-10
actinolite (AC)	0	0.0E+00	0 0E+00
amosite (AM)	0	0.0E+00	0.0E+00
anthophyllite (AN)	0	0.0E+00	0 0E+00
crocidolite (CR)	0	0 0E+00	0 0E+00
tremolite (TR)	0	0.0E+00	0.0E+00
winchite/richterite/tremolite/actinolite (WRTA)	0	0.0E+00	0.0E+00
other amphibole (OA)	4.225E-14	4.1E-14	2.5E-10
other mineral class (OM)	0	0.0E+00	0.0E+00

Σ Asbestos Structure Mass (g)		Mass fraction (as %)
**Total TEM**		
WRTA	0.00E+00	0.0000%
OA	8.54E-12	0.0005%
CH	1.23E-08	0.7223%
AC	0.00E+00	0.0000%
AM	0.00E+00	0.0000%
AN	0.00E+00	0.0000%
CR	0.00E+00	0.0000%
TR	3.23E-10	0.0189%
**PCM[illegible]**		
WRTA	0.00E+00	0.0000%
OA	0.00E+00	0.0000%
CH	4.19E-10	0.0245%
AC	0 00E+00	0.0000%
AM	0.00E+00	0.0000%
AN	0 00E+00	0.0000%
CR	0.00E+00	0.0000%
TR	0.00E+00	0.0000%

Asbestos structure mass = Length • Width^2 • Density • 1E-12

LA Density = 3 1 g/cm^3
OA Density = 3.0 g/cm^3
CH Density = 2.6 g/cm^3
AC Density = 3.1 g/cm^3
AM Density = 3.5 g/cm^3
AN Density = 3.0 g/cm^3
CR Density = 3.3 g/cm^3
TR Density = 3.0 g/cm^3

Csoil (mass fraction, as %) = Σ Asbestos structure mass • Sensitivity • 100

Equations:

Filter Loading (s/mm^2) = N / (GOx • Ago)
Sensitivity (1/g) = EFA / (GOx • Ago • M • f • Q_R)
Csoil (s/g) = N • Sensitivity

Asbestos Types:

AC - actinolite
AM - amosite
AN - anthophyllite
CH - chrysotile
CR - crocidolite
TR - tremolite
LA - Libby amphibole
OA - other amphibole
NAM - non-asbestos material
OM - other mineral type (specify in "other mineral description" field)

Structure Types:

High mag: Length ≥ 0.5 um, Aspect Ratio ≥ 3:1
PCME: Length > 5 um, Width ≥ 0.25 um, Aspect Ratio ≥ 3:1

Sumas Mtn Asbestos Methods - FBAS_LabCor Inc_14394114_SO_03-05-15_150099_TEM-EPAISO__D.xlsm
FluidizedBedEDD_v1

TEM Asbestos Structure Count for Fluidized Bed Material -- ISO
ANALYTICAL REPORT

FILE NAME:Sumas Mtn Asbestos Methods - FBAS_LabCor Inc_14394114_SO_03-05-15_150099_TEM-EPAISO__D.xlsm

SAMPLE INFORMATION		ANALYSIS PARAMETERS		
**Field Sample Number**	14394114	**EFA (mm^2)**	385	
		**GO area (mm^2) [Ago]**	0.0104	
**Matrix**	Soil	**# GOs counted - high mag**	100	
**Field QC Type**	Not QC	**# GOs counted - low mag only**	0	
**Sample Mass (g dry wt) [M]**	0.50	**F-factor [f]**	1.00E+00	
**Fluidized Bed Flow Ratio[Q_R]**	0.0122			
**Filter Status**	ANALYZED	Sensitivity - High mag	6.0E+04	1/g
		Sensitivity - PCME (high+low mag)	6.0E+04	1/g

H7000FA

ANALYSIS INFORMATION

**Lab Name**	LabCor Inc
**Lab Sample Number**	150099-S15
**Lab QC Type**	NOT QC
**TEM Prep Method**	Direct
**Est. particulate loading on filter**	NA

Recording Rules:

Initial (high mag)

Min. AR	Min length (um)	Min width (um)
3:1	0	0

PCME Supplemental (low mag)

Min. AR	Min length (um)	Min width (um)
3:1	0	0

Stopping Rules:

Target S (1/g)	Max Area (mm^2)	Max N

RESULTS SUMMARY

Structure Metric	Observed Structure Count (s)	Structure Filter Loading (s/mm^2)	Soil Conc. Structures per gram (s/g)
**High mag only**			
Total Asbestos	58	5.6E+01	3.5E+06
Total Chrysotile (CH)	51	4.9E+01	3.1E+06
Total Amphibole	7	6.8E+00	4.2E+05
actinolite (AC)	3	2.9E+00	1.8E+05
amosite (AM)	0	0.0E+00	0.0E+00
anthophyllite (AN)	0	0.0E+00	0.0E+00
crocidolite (CR)	0	0.0E+00	0.0E+00
tremolite (TR)	4	3.9E+00	2.4E+05
winchite/richterite/tremolite/actinolite (WRTA)	0	0.0E+00	0.0E+00
other amphibole (OA)	0	0.0E+00	0.0E+00
other mineral class (OM)	0	0.0E+00	0.0E+00
**PCME (high+low mag)**			
Total Asbestos	2	1.9E+00	1.2E+05
Total Chrysotile (CH)	2	1.9E+00	1.2E+05
Total Amphibole	0	0.0E+00	0.0E+00
actinolite (AC)	0	0.0E+00	0.0E+00
amosite (AM)	0	0.0E+00	0.0E+00
anthophyllite (AN)	0	0.0E+00	0.0E+00
crocidolite (CR)	0	0.0E+00	0.0E+00
tremolite (TR)	0	0.0E+00	0.0E+00
winchite/richterite/tremolite/actinolite (WRTA)	0	0.0E+00	0.0E+00
other amphibole (OA)	0	0.0E+00	0.0E+00
other mineral class (OM)	0	0.0E+00	0.0E+00

	Σ Asbestos Structure Mass (g)	Mass fraction (as %)
**Total TEM**		
WRTA	0.00E+00	0.0000%
OA	0.00E+00	0.0000%
CH	3.56E-08	21.5080%
AC	4.23E-10	0.2557%
AM	0.00E+00	0.0000%
AN	0.00E+00	0.0000%
CR	0.00E+00	0.0000%
TR	3.90E-09	2.3616%
**PCME**		
WRTA	0.00E+00	0.0000%
OA	0.00E+00	0.0000%
CH	5.05E-09	3.0579%
AC	#N/A	#N/A
AM	0.00E+00	0.0000%
AN	0.00E+00	0.0000%
CR	0.00E+00	0.0000%
TR	0.00E+00	0.0000%

Asbestos structure mass = Length • Width^2 • Density • 1E-12

LA Density = 3.1 g/cm^3
OA Density = 3.0 g/cm^3
CH Density = 2.6 g/cm^3
AC Density = 3.1 g/cm^3
AM Density = 3.5 g/cm^3
AN Density = 3.0 g/cm^3
CR Density = 3.3 g/cm^3
TR Density = 3.0 g/cm^3

Csoil (mass fraction, as %) = Σ Asbestos structure mass • Sensitivity • 100

Equations:

Filter Loading (s/mm^2) = N / (GOx • Ago)
Sensitivity (1/g) = EFA / (GOx • Ago • M • f • Q_R)
Csoil (s/g) = N • Sensitivity

Asbestos Types:

AC - actinolite
AM - amosite
AN - anthophyllite
CH - chrysotile
CR - crocidolite
TR - tremolite
LA - Libby amphibole
OA - other amphibole
NAM - non-asbestos material
OM - other mineral type (specify in "other mineral description" field)

Structure Types:

High mag: Length ≥ 0 5 um, Aspect Ratio ≥ 3:1
PCME: Length > 5 um, Width ≥ 0.25 um, Aspect Ratio ≥ 3:1

Sumas Mtn Asbestos Methods - FBAS_LabCor Inc_14394115_SO_03-07-15_150099_TEM-EPAISO__D.xlsm
FluidizedBedEDD_v1

TEM Asbestos Structure Count for Fluidized Bed Material -- ISO
ANALYTICAL REPORT

FILE NAME:Sumas Mtn Asbestos Methods - FBAS_LabCor Inc_14394115_SO_03-07-15_150099_TEM-EPAISO__D.xlsm

SAMPLE INFORMATION		ANALYSIS PARAMETERS		
**Field Sample Number**	14394115	**EFA (mm^2)**	385	
		**GO area (mm^2) [Ago]**	0.0104	
**Matrix**	Soil	**# GOs counted - high mag**	100	
**Field QC Type**	Not QC	**# GOs counted - low mag only**	0	
**Sample Mass (g dry wt) [M]**	1.00	**F-factor [f]**	1.00E+00	
**Fluidized Bed Flow Ratio[Q_R]**	0.0122			
**Filter Status**	ANALYZED	Sensitivity - High mag	3.0E+04	1/g
		Sensitivity - PCME (high+low mag)	3.0E+04	1/g

ANALYSIS INFORMATION

**Lab Name**	LabCor Inc
**Lab Sample Number**	150099-S16
**Lab QC Type**	NOT QC
**TEM Prep Method**	Direct
**Est. particulate loading on filter**	NA

Recording Rules:

Initial (high mag)

Min. AR	Min length (um)	Min width (um)
3:1	0	0

PCME Supplemental (low mag)

Min. AR	Min length (um)	Min width (um)
3:1	0	0

Stopping Rules:

Target S (1/g)	Max Area (mm^2)	Max N

RESULTS SUMMARY

Structure Metric	Observed Structure Count (s)	Structure Filter Loading (s/mm^2)	Soil Conc. Structures per gram (s/g)
**High mag only**			
Total Asbestos	25	2.4E+01	7.6E+05
Total Chrysotile (CH)	17	1.6E+01	5.2E+05
Total Amphibole	8	7.7E+00	2.4E+05
actinolite (AC)	2	1.9E+00	6.1E+04
amosite (AM)	2	1.9E+00	6.1E+04
anthophyllite (AN)	1	9.7E-01	3.0E+04
crocidolite (CR)	0	0.0E+00	0.0E+00
tremolite (TR)	3	2.9E+00	9.1E+04
winchite/richterite/tremolite/actinolite (WRTA)	0	0.0E+00	0.0E+00
other amphibole (OA)	0	0.0E+00	0.0E+00
other mineral class (OM)	0	0.0E+00	0.0E+00
**PCME (high+low mag)**			
Total Asbestos	1	9.7E-01	3.0E+04
Total Chrysotile (CH)	1	9.7E-01	3.0E+04
Total Amphibole	3.9375E-14	3.8E-14	1.2E-09
actinolite (AC)	0	0.0E+00	0.0E+00
amosite (AM)	3.9375E-14	3.8E-14	1.2E-09
anthophyllite (AN)	0	0.0E+00	0.0E+00
crocidolite (CR)	0	0.0E+00	0.0E+00
tremolite (TR)	0	0.0E+00	0.0E+00
winchite/richterite/tremolite/actinolite (WRTA)	0	0.0E+00	0.0E+00
other amphibole (OA)	0	0.0E+00	0.0E+00
other mineral class (OM)	0	0.0E+00	0.0E+00

Σ Asbestos Structure Mass (g)		Mass fraction (as %)
**Total TEM**		
WRTA	0.00E+00	0.0000%
OA	0.00E+00	0.0000%
CH	9.85E-09	3.0016%
AC	5.81E-10	0.1768%
AM	0.00E+00	0.0000%
AN	3.00E-09	0.9138%
CR	0.00E+00	0.0000%
TR	2.82E-10	0.0858%
**PCME**		
WRTA	0.00E+00	0.0000%
OA	0.00E+00	0.0000%
CH	1.64E-11	0.0050%
AC	#N/A	#N/A
AM	0.00E+00	0.0000%
AN	0.00E+00	0.0000%
CR	0.00E+00	0.0000%
TR	#N/A	#N/A

Asbestos structure mass = Length • Width^2 • Density • 1E-12

LA Density = 3.1 g/cm^3
OA Density = 3.0 g/cm^3
CH Density = 2.6 g/cm^3
AC Density = 3.1 g/cm^3
AM Density = 3.5 g/cm^3
AN Density = 3.0 g/cm^3
CR Density = 3.3 g/cm^3
TR Density = 3.0 g/cm^3

Csoil (mass fraction, as %) = Σ Asbestos structure mass • Sensitivity • 100

Equations:

Filter Loading (s/mm^2) = N / (GOx • Ago)
Sensitivity (1/g) = EFA / (GOx • Ago • M • f • Q_R)
Csoil (s/g) = N • Sensitivity

Asbestos Types:

AC - actinolite
AM - amosite
AN - anthophyllite
CH - chrysotile
CR - crocidolite
TR - tremolite
LA - Libby amphibole
OA - other amphibole
NAM - non-asbestos material
OM - other mineral type (specify in "other mineral description" field)

Structure Types:

High mag: Length ≥ 0.5 um, Aspect Ratio ≥ 3:1
PCME: Length > 5 um, Width ≥ 0.25 um, Aspect Ratio ≥ 3:1

Sumas Mtn Asbestos Methods - FBAS_LabCor Inc_14394116_SO_03-08-15_150099_TEM-EPAISO__D.xlsm
FluidizedBedEDD_v1

TEM Asbestos Structure Count for Fluidized Bed Material -- ISO
ANALYTICAL REPORT

FILE NAME:Sumas Mtn Asbestos Methods - FBAS_LabCor Inc_14394116_SO_03-08-15_150099_TEM-EPAISO__D.xlsm

SAMPLE INFORMATION		ANALYSIS PARAMETERS		
**Field Sample Number**	14394116	**EFA (mm^2)**	385	
		**GO area (mm^2) [Ago]**	0.0104	
**Matrix**	Soil	**# GOs counted - high mag**	100	
**Field QC Type**	Not QC	**# GOs counted - low mag only**	0	
**Sample Mass (g dry wt) [M]**	1.05	**F-factor [f]**	1.00E+00	
**Fluidized Bed Flow Ratio[Q_R]**	0.0122			
**Filter Status**	ANALYZED	Sensitivity - High mag	2.9E+04	1/g
H70 60FA		Sensitivity - PCME (high+low mag)	2.9E+04	1/g

ANALYSIS INFORMATION

**Lab Name**	LabCor Inc
**Lab Sample Number**	150099-S17
**Lab QC Type**	NOT QC
**TEM Prep Method**	Direct
**Est. particulate loading on filter**	NA

Recording Rules:

Initial (high mag)

Min. AR	Min length (um)	Min width (um)
3:1	0	0

PCME Supplemental (low mag)

Min. AR	Min length (um)	Min width (um)
3:1	0	0

Stopping Rules:

Target S (1/g)	Max Area (mm^2)	Max N

RESULTS SUMMARY

Structure Metric	Observed Structure Count (s)	Structure Filter Loading (s/mm^2)	Soil Conc. Structures per gram (s/g)
**High mag only**			
Total Asbestos	68	6.6E+01	2.0E+06
Total Chrysotile (CH)	36	3.5E+01	1.0E+06
Total Amphibole	32	3.1E+01	9.3E+05
actinolite (AC)	11	1.1E+01	3.2E+05
amosite (AM)	10	9.7E+00	2.9E+05
anthophyllite (AN)	0	0.0E+00	0.0E+00
crocidolite (CR)	0	0.0E+00	0.0E+00
tremolite (TR)	5	4.8E+00	1.5E+05
winchite/richterite/tremolite/actinolite (WRTA)	0	0.0E+00	0.0E+00
other amphibole (OA)	6	5.8E+00	1.7E+05
other mineral class (OM)	0	0.0E+00	0.0E+00
**PCME (high+low mag)**			
Total Asbestos	4	3.9E+00	1.2E+05
Total Chrysotile (CH)	1	9.7E-01	2.9E+04
Total Amphibole	3	2.9E+00	8.7E+04
actinolite (AC)	1	9.7E-01	2.9E+04
amosite (AM)	4.30064E-10	4.2E-10	1.3E-05
anthophyllite (AN)	0	0.0E+00	0.0E+00
crocidolite (CR)	0	0.0E+00	0.0E+00
tremolite (TR)	1	9.7E-01	2.9E+04
winchite/richterite/tremolite/actinolite (WRTA)	0	0.0E+00	0.0E+00
other amphibole (OA)	1	9.7E-01	2.9E+04
other mineral class (OM)	0	0.0E+00	0.0E+00

	Σ Asbestos Structure Mass (g)	Mass fraction (as %)
**Total TEM**		
WRTA	0.00E+00	0.0000%
OA	1.51E-10	0.0440%
CH	1.92E-07	55.7749%
AC	5.36E-08	15.5870%
AM	0.00E+00	0.0000%
AN	0.00E+00	0.0000%
CR	0.00E+00	0.0000%
TR	3.40E-08	9.8757%
**PCME**		
WRTA	0.00E+00	0.0000%
OA	1.19E-10	0.0347%
CH	4.58E-10	0.1332%
AC	#N/A	#N/A
AM	0.00E+00	0.0000%
AN	0.00E+00	0.0000%
CR	0.00E+00	0.0000%
TR	0.00E+00	0.0000%

Asbestos structure mass = Length • Width^2 • Density • 1E-12

LA Density = 3.1 g/cm^3
OA Density = 3.0 g/cm^3
CH Density = 2.6 g/cm^3
AC Density = 3.1 g/cm^3
AM Density = 3.5 g/cm^3
AN Density = 3.0 g/cm^3
CR Density = 3.3 g/cm^3
TR Density = 3.0 g/cm^3

Csoil (mass fraction, as %) = Σ Asbestos structure mass • Sensitivity • 100

Equations:
Filter Loading (s/mm^2) = N / (GOx • Ago)
Sensitivity (1/g) = EFA / (GOx • Ago • M • f • Q_R)
Csoil (s/g) = N • Sensitivity

Asbestos Types:
AC - actinolite
AM - amosite
AN - anthophyllite
CH - chrysotile
CR - crocidolite
TR - tremolite
LA - Libby amphibole
OA - other amphibole
NAM - non-asbestos material
OM - other mineral type (specify in "other mineral description" field)

Structure Types:
High mag: Length ≥ 0.5 um, Aspect Ratio ≥ 3:1
PCME: Length > 5 um, Width ≥ 0.25 um, Aspect Ratio ≥ 3:1

Sumas Mtn Asbestos Methods - FBAS_LabCor Inc_14394117_SO_03-12-15_150099_TEM-EPAISO__D.xlsm
FluidizedBedEDD_v1

TEM Asbestos Structure Count for Fluidized Bed Material -- ISO
ANALYTICAL REPORT

FILE NAME:Sumas Mtn Asbestos Methods - FBAS_LabCor Inc_14394117_SO_03-12-15_150099_TEM-EPAISO__D.xlsm

SAMPLE INFORMATION		ANALYSIS PARAMETERS		
**Field Sample Number**	14394117	**EFA (mm^2)**	385	
		**GO area (mm^2) [Ago]**	0 0104	
**Matrix**	Soil	**# GOs counted - high mag**	100	
**Field QC Type**	Not QC	**# GOs counted - low mag only**	0	
**Sample Mass (g dry wt) [M]**	1.00	**F-factor [f]**	1.00E+00	
**Fluidized Bed Flow Ratio[Q_R]**	0.0122			
**Filter Status**	ANALYZED	Sensitivity - High mag	3 0E+04	1/g
H7000FA		Sensitivity - PCME (high+low mag)	3.0E+04	1/g

ANALYSIS INFORMATION

Lab Name LabCor Inc
Lab Sample Number 150099-S18
Lab QC Type NOT QC
TEM Prep Method Direct
Est. particulate loading on filter NA

Recording Rules:

Initial (high mag)

Min. AR	Min length (um)	Min width (um)
3:1	0	0

PCME Supplemental (low mag)

Min. AR	Min length (um)	Min width (um)
3:1	0	0

Stopping Rules:

Target S (1/g)	Max Area (mm^2)	Max N

RESULTS SUMMARY

Structure Metric	Observed Structure Count (s)	Structure Filter Loading (s/mm²)	Soil Conc. Structures per gram (s/g)
**High mag only**			
Total Asbestos	25	2.4E+01	7.6E+05
Total Chrysotile (CH)	22	2.1E+01	6.7E+05
Total Amphibole	3	2.9E+00	9.1E+04
actinolite (AC)	0	0.0E+00	0 0E+00
amosite (AM)	0	0.0E+00	0.0E+00
anthophyllite (AN)	0	0.0E+00	0.0E+00
crocidolite (CR)	0	0.0E+00	0.0E+00
tremolite (TR)	3	2.9E+00	9.1E+04
winchite/richterite/tremolite/actinolite (WRTA)	0	0.0E+00	0.0E+00
other amphibole (OA)	0	0.0E+00	0 0E+00
other mineral class (OM)	0	0.0E+00	0.0E+00
**PCME (high+low mag)**			
Total Asbestos	0	0.0E+00	0.0E+00
Total Chrysotile (CH)	0	0.0E+00	0.0E+00
Total Amphibole	0	0.0E+00	0.0E+00
actinolite (AC)	0	0.0E+00	0.0E+00
amosite (AM)	0	0.0E+00	0.0E+00
anthophyllite (AN)	0	0.0E+00	0.0E+00
crocidolite (CR)	0	0.0E+00	0.0E+00
tremolite (TR)	0	0.0E+00	0.0E+00
winchite/richterite/tremolite/actinolite (WRTA)	0	0.0E+00	0.0E+00
other amphibole (OA)	0	0.0E+00	0.0E+00
other mineral class (OM)	0	0.0E+00	0.0E+00

	Σ Asbestos Structure Mass (g)	Mass fraction (as %)
**Total TEM**		
WRTA	0.00E+00	0.0000%
OA	0.00E+00	0.0000%
CH	3.95E-08	12.0117%
AC	0.00E+00	0.0000%
AM	0.00E+00	0.0000%
AN	0.00E+00	0.0000%
CR	0.00E+00	0.0000%
TR	9.64E-11	0.0293%
**PCME**		
WRTA	0.00E+00	0.0000%
OA	0.00E+00	0.0000%
CH	0.00E+00	0.0000%
AC	0.00E+00	0.0000%
AM	0.00E+00	0.0000%
AN	0.00E+00	0.0000%
CR	0.00E+00	0.0000%
TR	#N/A	#N/A

Asbestos structure mass = Length • Width² • Density • 1E-12

LA Densitv = 3.1 g/cm³
OA Density = 3.0 g/cm³
CH Density = 2.6 g/cm³
AC Density = 3.1 g/cm³
AM Density = 3.5 g/cm³
AN Density = 3.0 g/cm³
CR Density = 3 3 g/cm³
TR Density = 3.0 g/cm³

Csoil (mass fraction, as %) = Σ Asbestos structure mass • Sensitivity • 100

Equations:

Filter Loading (s/mm^2) = N / (GOx • Ago)
Sensitivity (1/g) = EFA / (GOx • Ago • M • f • Q_R)
Csoil (s/g) = N • Sensitivity

Asbestos Types:

AC - actinolite
AM - amosite
AN - anthophyllite
CH - chrysotile
CR - crocidolite
TR - tremolite
LA - Libby amphibole
OA - other amphibole
NAM - non-asbestos material
OM - other mineral type (specify in "other mineral description" field)

Structure Types:

High mag: Length ≥ 0.5 um, Aspect Ratio ≥ 3:1
PCME: Length > 5 um, Width ≥ 0.25 um, Aspect Ratio ≥ 3:1

Sumas Mtn Asbestos Methods - FBAS_LabCor Inc_14394118_SO_03-13-15_150099_TEM-EPAISO__D.xlsm
FluidizedBedEDD_v1

TEM Asbestos Structure Count for Fluidized Bed Material -- ISO
ANALYTICAL REPORT

FILE NAME:Sumas Mtn Asbestos Methods - FBAS_LabCor Inc_14394118_SO_03-13-15_150099_TEM-EPAISO__D.xlsm

SAMPLE INFORMATION		ANALYSIS PARAMETERS		
**Field Sample Number**	14394118	**EFA (mm^2)**	385	
		**GO area (mm^2) [Ago]**	0 0104	
**Matrix**	Soil	**# GOs counted - high mag**	100	
**Field QC Type**	Not QC	**# GOs counted - low mag only**	0	
**Sample Mass (g dry wt) [M]**	1.01	**F-factor [f]**	1.00E+00	
**Fluidized Bed Flow Ratio[Q_R]**	0.0122			
**Filter Status**	ANALYZED	Sensitivity - High mag	3.0E+04	1/g
H1000FA		Sensitivity - PCME (high+low mag)	3.0E+04	1/g

ANALYSIS INFORMATION

Lab Name LabCor Inc
Lab Sample Number 150099-S19
Lab QC Type NOT QC
TEM Prep Method Direct
Est. particulate loading on filter NA

Recording Rules:

Initial (high mag)

Min. AR	Min length (um)	Min width (um)
3:1	0	0

PCME Supplemental (low mag)

Min. AR	Min length (um)	Min width (um)
3:1	0	0

Stopping Rules:

Target S (1/g)	Max Area (mm^2)	Max N

RESULTS SUMMARY

Structure Metric	Observed Structure Count (s)	Structure Filter Loading (s/mm^2)	Soil Conc. Structures per gram (s/g)
**High mag only**			RC
Total Asbestos	15	1.4E+01	4.5E+05
Total Chrysotile (CH)	10	9.7E+00	3.0E+05
Total Amphibole	5 J	4.8E+00	1.5E+05
actinolite (AC)	2	1.9E+00 IC	6.1E+04
amosite (AM)	0	0.0E+00	0.0E+00
anthophyllite (AN)	0	0.0E+00	0.0E+00
crocidolite (CR)	0	0.0E+00	0.0E+00
tremolite (TR)	3 J	2.9E+00 IC	9.1E+04
winchite/richterite/tremolite/actinolite (WRTA)	0	0.0E+00	0.0E+00
other amphibole (OA)	0	0.0E+00	0.0E+00
other mineral class (OM)	0	0.0E+00	0.0E+00
**PCME (high+low mag)**			
Total Asbestos	0	0.0E+00	0.0E+00
Total Chrysotile (CH)	0	0.0E+00	0.0E+00
Total Amphibole	0	0.0E+00	0.0E+00
actinolite (AC)	0	0.0E+00	0.0E+00
amosite (AM)	0	0.0E+00	0.0E+00
anthophyllite (AN)	0	0.0E+00	0.0E+00
crocidolite (CR)	0	0.0E+00	0.0E+00
tremolite (TR)	0	0.0E+00	0.0E+00
winchite/richterite/tremolite/actinolite (WRTA)	0	0.0E+00	0.0E+00
other amphibole (OA)	0	0.0E+00	0.0E+00
other mineral class (OM)	0	0.0E+00	0.0E+00

Σ Asbestos Structure Mass (g)		Mass fraction (as %)
**Total TEM**		
WRTA	0.00E+00	0.0000%
OA	0.00E+00	0.0000%
CH	3.54E-09	1.0719%
AC	1.24E-11	0.0037%
AM	0.00E+00	0.0000%
AN	0.00E+00	0.0000%
CR	0.00E+00	0.0000%
TR	6.41E-11	0.0194%
**PCME**		
WRTA	0.00E+00	0.0000%
OA	0.00E+00	0.0000%
CH	0.00E+00	0.0000%
AC	#N/A	#N/A
AM	0.00E+00	0.0000%
AN	0.00E+00	0.0000%
CR	0.00E+00	0.0000%
TR	0.00E+00	0.0000%

Asbestos structure mass = Length • Width^2 • Density • 1E-12

LA Density = 3.1 g/cm^3
OA Density = 3.0 g/cm^3
CH Density = 2.6 g/cm^3
AC Density = 3.1 g/cm^3
AM Density = 3.5 g/cm^3
AN Density = 3.0 g/cm^3
CR Density = 3.3 g/cm^3
TR Density = 3.0 g/cm^3

Csoil (mass fraction, as %) = Σ Asbestos structure mass • Sensitivity • 100

Equations:
Filter Loading (s/mm^2) = N / (GOx • Ago)
Sensitivity (1/g) = EFA / (GOx • Ago • M • f • Q_R)
Csoil (s/g) = N • Sensitivity

Asbestos Types:
AC - actinolite
AM - amosite
AN - anthophyllite
CH - chrysotile
CR - crocidolite
TR - tremolite
LA - Libby amphibole
OA - other amphibole
NAM - non-asbestos material
OM - other mineral type (specify in "other mineral description" field)

Structure Types:
High mag: Length ≥ 0.5 um, Aspect Ratio ≥ 3:1
PCME: Length > 5 um, Width ≥ 0.25 um, Aspect Ratio ≥ 3:1

Sumas Mtn Asbestos Methods - FBAS_LabCor Inc_14394119_SO_03-13-15_150099_TEM-EPAISO__D.xlsm
FluidizedBedEDD_v1

TEM Asbestos Structure Count for Fluidized Bed Material -- ISO
ANALYTICAL REPORT

FILE NAME:Sumas Mtn Asbestos Methods - FBAS_LabCor Inc_14394119_SO_03-13-15_150099_TEM-EPAISO__D.xlsm

SAMPLE INFORMATION		ANALYSIS PARAMETERS		
**Field Sample Number**	14394119	**EFA (mm^2)**	385	
		**GO area (mm^2) [Ago]**	0.0104	
**Matrix**	Soil	**# GOs counted - high mag**	100	
**Field QC Type**	Not QC	**# GOs counted - low mag only**	0	
**Sample Mass (g dry wt) [M]**	1.01	**F-factor [f]**	1.00E+00	
**Fluidized Bed Flow Ratio[Q_R]**	0.0122			
**Filter Status**	ANALYZED	Sensitivity - High mag	3.0E+04	1/g
		Sensitivity - PCME (high+low mag)	3.0E+04	1/g

JCEL

ANALYSIS INFORMATION

**Lab Name**	LabCor Inc
**Lab Sample Number**	150099-S20
**Lab QC Type**	NOT QC
**TEM Prep Method**	Direct
**Est. particulate loading on filter**	NA

Recording Rules:

Initial (high mag)

Min. AR	Min length (um)	Min width (um)
3:1	0	0

PCME Supplemental (low mag)

Min. AR	Min length (um)	Min width (um)
3:1	0	0

Stopping Rules:

Target S (1/g)	Max Area (mm^2)	Max N

RESULTS SUMMARY

Structure Metric	Observed Structure Count (s)	Structure Filter Loading (s/mm^2)	Soil Conc. Structures per gram (s/g)
**High mag only**			
Total Asbestos	68	6.6E+01	2.1E+06
Total Chrysotile (CH)	52	5.0E+01	1.6E+06
Total Amphibole	16	1.5E+01	4.9E+05
actinolite (AC)	7	6.8E+00	2.1E+05
amosite (AM)	0	0.0E+00	0.0E+00
anthophyllite (AN)	0	0.0E+00	0.0E+00
crocidolite (CR)	0	0.0E+00	0.0E+00
tremolite (TR)	9	8.7E+00	2.7E+05
winchite/richterite/tremolite/actinolite (WRTA)	0	0.0E+00	0.0E+00
other amphibole (OA)	0	0.0E+00	0.0E+00
other mineral class (OM)	0	0.0E+00	0.0E+00
**PCME (high+low mag)**			
Total Asbestos	5	4.8E+00	1.5E+05
Total Chrysotile (CH)	4	3.9E+00	1.2E+05
Total Amphibole	1	9.7E-01	3.0E+04
actinolite (AC)	0	0.0E+00	0.0E+00
amosite (AM)	0	0.0E+00	0.0E+00
anthophyllite (AN)	0	0.0E+00	0.0E+00
crocidolite (CR)	0	0.0E+00	0.0E+00
tremolite (TR)	1	9.7E-01	3.0E+04
winchite/richterite/tremolite/actinolite (WRTA)	0	0.0E+00	0.0E+00
other amphibole (OA)	0	0.0E+00	0.0E+00
other mineral class (OM)	0	0.0E+00	0.0E+00

	Σ Asbestos Structure Mass (g)	Mass fraction (as %)
**Total TEM**		
WRTA	0.00E+00	0.0000%
OA	0.00E+00	0.0000%
CH	4.33E-09	1.3145%
AC	3.01E-11	0.0091%
AM	0.00E+00	0.0000%
AN	0.00E+00	0.0000%
CR	0.00E+00	0.0000%
TR	1.14E-08	3.4574%
**PCME**		
WRTA	0.00E+00	0.0000%
OA	0.00E+00	0.0000%
CH	2.04E-11	0.0062%
AC	#N/A	#N/A
AM	0.00E+00	0.0000%
AN	0.00E+00	0.0000%
CR	0.00E+00	0.0000%
TR	#N/A	#N/A

Asbestos structure mass = Length • Width^2 • Density • 1E-12

LA Density = 3.1 g/cm^3
OA Density = 3.0 g/cm^3
CH Density = 2.6 g/cm^3
AC Density = 3.1 g/cm^3
AM Density = 3.5 g/cm^3
AN Density = 3.0 g/cm^3
CR Density = 3.3 g/cm^3
TR Density = 3.0 g/cm^3

Csoil (mass fraction, as %) = Σ Asbestos structure mass • Sensitivity • 100

Equations:

Filter Loading (s/mm^2) = N / (GOx • Ago)
Sensitivity (1/g) = EFA / (GOx • Ago • M • f • Q_R)
Csoil (s/g) = N • Sensitivity

Asbestos Types:

AC - actinolite
AM - amosite
AN - anthophyllite
CH - chrysotile
CR - crocidolite
TR - tremolite
LA - Libby amphibole
OA - other amphibole
NAM - non-asbestos material
OM - other mineral type (specify in "other mineral description" field)

Structure Types:

High mag: Length ≥ 0.5 um, Aspect Ratio ≥ 3:1
PCME: Length > 5 um, Width ≥ 0.25 um, Aspect Ratio ≥ 3:1

Sumas Mtn Asbestos Methods - FBAS_Labcor Inc_14394120_SO_03-13-15_150099_TEM-EPAISO__D.xlsm
FluidizedBedEDD_v1

TEM Asbestos Structure Count for Fluidized Bed Material -- ISO
ANALYTICAL REPORT

FILE NAME:Sumas Mtn Asbestos Methods - FBAS_Labcor Inc_14394120_SO_03-13-15_150099_TEM-EPAISO__D.xlsm

SAMPLE INFORMATION		ANALYSIS PARAMETERS		
**Field Sample Number**	14394120	**EFA (mm^2)**	385	
		**GO area (mm^2) [Ago]**	0.0104	
**Matrix**	Soil	**# GOs counted - high mag**	100	
**Field QC Type**	Not QC	**# GOs counted - low mag only**	0	
**Sample Mass (g dry wt) [M]**	1.01	**F-factor [f]**	1.00E+00	
**Fluidized Bed Flow Ratio[Q_R]**	0.0122			
**Filter Status**	ANALYZED	Sensitivity - High mag	3.0E+04	1/g
41000 FA		Sensitivity - PCME (high+low mag)	3.0E+04	1/g

ANALYSIS INFORMATION

Lab Name Labcor Inc
Lab Sample Number 150099-S21
Lab QC Type NOT QC
TEM Prep Method Direct
Est. particulate loading on filter NA

Recording Rules:

Initial (high mag)

Min. AR	Min length (um)	Min width (um)
3:1	0	0

PCME Supplemental (low mag)

Min. AR	Min length (um)	Min width (um)
3:1	0	0

Stopping Rules:

Target S (1/g)	Max Area (mm^2)	Max N

RESULTS SUMMARY

Structure Metric	Observed Structure Count (s)	Structure Filter Loading (s/mm^2)	Soil Conc. Structures per gram (s/g)
**High mag only**			
Total Asbestos	93	9.0E+01	2.8E+06
Total Chrysotile (CH)	58	5.6E+01	1.8E+06
Total Amphibole	35	3.4E+01	1.1E+06
actinolite (AC)	13	1.3E+01	3.9E+05
amosite (AM)	12	1.2E+01	3.6E+05
anthophyllite (AN)	0	0.0E+00	0.0E+00
crocidolite (CR)	0	0.0E+00	0.0E+00
tremolite (TR)	8	7.7E+00	2.4E+05
winchite/richterite/tremolite/actinolite (WRTA)	0	0.0E+00	0.0E+00
other amphibole (OA)	2	1.9E+00	6.1E+04
other mineral class (OM)	0	0.0E+00	0.0E+00
**PCME (high+low mag)**			
Total Asbestos	5.000000001	4.8E+00	1.5E+05
Total Chrysotile (CH)	2	1.9E+00	6.1E+04
Total Amphibole	3.000000001	2.9E+00	9.1E+04
actinolite (AC)	2	1.9E+00	6.1E+04
amosite (AM)	1.45279E-09	1.4E-09	4.4E-05
anthophyllite (AN)	0	0.0E+00	0.0E+00
crocidolite (CR)	0	0.0E+00	0.0E+00
tremolite (TR)	1	9.7E-01	3.0E+04
winchite/richterite/tremolite/actinolite (WRTA)	0	0.0E+00	0.0E+00
other amphibole (OA)	0	0.0E+00	0.0E+00
other mineral class (OM)	0	0.0E+00	0.0E+00

	Σ Asbestos Structure Mass (g)	Mass fraction (as %)
**Total TEM**		
WRTA	0.00E+00	0.0000%
OA	2.27E-12	0.0007%
CH	7.50E-08	22.7629%
AC	5.58E-09	1.6921%
AM	0.00E+00	0.0000%
AN	0.00E+00	0.0000%
CR	0.00E+00	0.0000%
TR	1.08E-08	3.2849%
**PCME**		
WRTA	0.00E+00	0.0000%
OA	0.00E+00	0.0000%
CH	1.51E-10	0.0457%
AC	#N/A	#N/A
AM	0.00E+00	0.0000%
AN	0.00E+00	0.0000%
CR	0.00E+00	0.0000%
TR	#N/A	#N/A

Asbestos structure mass = Length • $Width^2$ • Density • 1E-12

LA Density = 3.1 g/cm^3
OA Density = 3.0 g/cm^3
CH Density = 2.6 g/cm^3
AC Density = 3.1 g/cm^3
AM Density = 3.5 g/cm^3
AN Density = 3.0 g/cm^3
CR Density = 3.3 g/cm^3
TR Density = 3.0 g/cm^3

Csoil (mass fraction, as %) = Σ Asbestos structure mass • Sensitivity • 100

Equations:

Filter Loading (s/mm^2) = N / (GOx • Ago)
Sensitivity (1/g) = EFA / (GOx • Ago • M • f • Q_R)
Csoil (s/g) = N • Sensitivity

Asbestos Types:

AC - actinolite
AM - amosite
AN - anthophyllite
CH - chrysotile
CR - crocidolite
TR - tremolite
LA - Libby amphibole
OA - other amphibole
NAM - non-asbestos material
OM - other mineral type (specify in "other mineral description" field)

Structure Types:

High mag: Length ≥ 0.5 um, Aspect Ratio ≥ 3:1
PCME: Length > 5 um, Width ≥ 0.25 um, Aspect Ratio ≥ 3:1

Sumas Mtn Asbestos Methods - FBAS_LabCor Inc_14394121_SO_03-14-15_150099_TEM-EPAISO__D.xlsm
FluidizedBedEDD_v1

TEM Asbestos Structure Count for Fluidized Bed Material -- ISO ANALYTICAL REPORT

FILE NAME:Sumas Mtn Asbestos Methods - FBAS_LabCor Inc_14394121_SO_03-14-15_150099_TEM-EPAISO__D.xlsm

SAMPLE INFORMATION		ANALYSIS PARAMETERS		
**Field Sample Number**	14394121	**EFA (mm^2)**	385	
		**GO area (mm^2) [Ago]**	0.0104	
**Matrix**	Soil	**# GOs counted - high mag**	100	
**Field QC Type**	Not QC	**# GOs counted - low mag only**	0	
**Sample Mass (g dry wt) [M]**	1.01	**F-factor [f]**	1.00E+00	
**Fluidized Bed Flow Ratio[Q_R]**	0.0122			
**Filter Status**	ANALYZED	Sensitivity - High mag	3.0E+04	1/g
47000 FA		Sensitivity - PCME (high+low mag)	3.0E+04	1/g

ANALYSIS INFORMATION

**Lab Name**	LabCor Inc
**Lab Sample Number**	150099-S22
**Lab QC Type**	NOT QC
**TEM Prep Method**	Direct
**Est. particulate loading on filter**	NA

Recording Rules:

Initial (high mag)

Min. AR	Min length (um)	Min width (um)
3:1	0	0

PCME Supplemental (low mag)

Min. AR	Min length (um)	Min width (um)
3:1	0	0

Stopping Rules:

Target S (1/g)	Max Area (mm^2)	Max N

RESULTS SUMMARY

Structure Metric	Observed Structure Count (s)	Structure Filter Loading (s/mm^2)	Soil Conc. Structures per gram (s/g)
**High mag only**			
Total Asbestos	110	1.1E+02	3.3E+06
Total Chrysotile (CH)	104	1.0E+02	3.1E+06
Total Amphibole	6	5.8E+00	1.8E+05
actinolite (AC)	1	9.7E-01	3.0E+04
amosite (AM)	0	0.0E+00	0.0E+00
anthophyllite (AN)	0	0.0E+00	0.0E+00
crocidolite (CR)	0	0.0E+00	0.0E+00
tremolite (TR)	5	4.8E+00	1.5E+05
winchite/richterite/tremolite/actinolite (WRTA)	0	0.0E+00	0.0E+00
other amphibole (OA)	0	0.0E+00	0.0E+00
other mineral class (OM)	0	0.0E+00	0.0E+00
**PCME (high+low mag)**			
Total Asbestos	5	4.8E+00	1.5E+05
Total Chrysotile (CH)	5	4.8E+00	1.5E+05
Total Amphibole	0	0.0E+00	0.0E+00
actinolite (AC)	0	0.0E+00	0.0E+00
amosite (AM)	0	0.0E+00	0.0E+00
anthophyllite (AN)	0	0.0E+00	0.0E+00
crocidolite (CR)	0	0.0E+00	0.0E+00
tremolite (TR)	0	0.0E+00	0.0E+00
winchite/richterite/tremolite/actinolite (WRTA)	0	0.0E+00	0.0E+00
other amphibole (OA)	0	0.0E+00	0.0E+00
other mineral class (OM)	0	0.0E+00	0.0E+00

	Σ Asbestos Structure Mass (g)	Mass fraction (as %)
**Total TEM**		
WRTA	0.00E+00	0.0000%
OA	0.00E+00	0.0000%
CH	1.71E-07	51.7061%
AC	1.55E-12	0.0005%
AM	0.00E+00	0.0000%
AN	0.00E+00	0.0000%
CR	0.00E+00	0.0000%
TR	4.24E-08	12.7975%
**PC[redacted]**		
WRTA	0.00E+00	0.0000%
OA	0.00E+00	0.0000%
CH	4.14E-10	0.1250%
AC	0.00E+00	0.0000%
AM	0.00E+00	0.0000%
AN	0.00E+00	0.0000%
CR	0.00E+00	0.0000%
TR	0.00E+00	0.0000%

Asbestos structure mass = Length • Width^2 • Density • 1E-12

LA Density = 3.1 g/cm^3
OA Density = 3.0 g/cm^3
CH Density = 2.6 g/cm^3
AC Density = 3.1 g/cm^3
AM Density = 3.5 g/cm^3
AN Density = 3.0 g/cm^3
CR Density = 3.3 g/cm^3
TR Density = 3.0 g/cm^3

Csoil (mass fraction, as %) = Σ Asbestos structure mass • Sensitivity • 100

Equations:

Filter Loading (s/mm^2) = N / (GOx • Ago)
Sensitivity (1/g) = EFA / (GOx • Ago • M • f • Q_R)
Csoil (s/g) = N • Sensitivity

Asbestos Types:

AC - actinolite
AM - amosite
AN - anthophyllite
CH - chrysotile
CR - crocidolite
TR - tremolite
LA - Libby amphibole
OA - other amphibole
NAM - non-asbestos material
OM - other mineral type (specify in "other mineral description" field)

Structure Types:

High mag: Length ≥ 0.5 um, Aspect Ratio ≥ 3:1
PCME: Length > 5 um, Width ≥ 0.25 um, Aspect Ratio ≥ 3:1

Report Printed: 7/1/2015, 1:16 PM

Sumas Mtn Asbestos Methods - FBAS_LabCor Inc_14394122_SO_03-17-15_150099_TEM-EPAISO__D.xlsm
FluidizedBedEDD_v1

TEM Asbestos Structure Count for Fluidized Bed Material -- ISO ANALYTICAL REPORT

FILE NAME:Sumas Mtn Asbestos Methods - FBAS_LabCor Inc_14394122_SO_03-17-15_150099_TEM-EPAISO__D.xlsm

SAMPLE INFORMATION		ANALYSIS PARAMETERS		
**Field Sample Number**	14394122	**EFA (mm^2)**	385	
		**GO area (mm^2) [Ago]**	0.0104	
**Matrix**	Soil	**# GOs counted - high mag**	39	
**Field QC Type**	Not QC	**# GOs counted - low mag only**	0	
**Sample Mass (g dry wt) [M]**	1.01	**F-factor [f]**	1.00E+00	
**Fluidized Bed Flow Ratio[Q_R]**	0.0122			
**Filter Status**	ANALYZED	Sensitivity - High mag	7.8E+04	1/g
H700FA		Sensitivity - PCME (high+low mag)	7.8E+04	1/g

ANALYSIS INFORMATION

Lab Name LabCor Inc
Lab Sample Number 150099-S23
Lab QC Type NOT QC
TEM Prep Method Direct
Est. particulate loading on filter NA

Recording Rules:

Initial (high mag)

Min. AR	Min length (um)	Min width (um)
3:1	0	0

PCME Supplemental (low mag)

Min. AR	Min length (um)	Min width (um)
3:1	0	0

Stopping Rules:

Target S (1/g)	Max Area (mm^2)	Max N

RESULTS SUMMARY

Structure Metric	Observed Structure Count (s)	Structure Filter Loading (s/mm^2)	Soil Conc. Structures per gram (s/g)
**High mag only**			
Total Asbestos	102 J	2.5E+02	7.9E+06
Total Chrysotile (CH)	100 J	2.5E+02 MC	7.8E+06
Total Amphibole	2 J	5.0E+00	1.6E+05
actinolite (AC)	1 J	2.5E+00 IC	7.8E+04
amosite (AM)	0	0.0E+00	0.0E+00
anthophyllite (AN)	0	0.0E+00	0.0E+00
crocidolite (CR)	0	0.0E+00	0.0E+00
tremolite (TR)	1 J	2.5E+00 IC	7.8E+04
winchite/richterite/tremolite/actinolite (WRTA)	0	0.0E+00	0.0E+00
other amphibole (OA)	0	0.0E+00	0.0E+00
other mineral class (OM)	0	0.0E+00	0.0E+00
**PCME (high+low mag)**			
Total Asbestos	2 J	5.0E+00	1.6E+05
Total Chrysotile (CH)	2 J	5.0E+00 MC	1.6E+05
Total Amphibole	0	0.0E+00	0.0E+00
actinolite (AC)	0	0.0E+00	0.0E+00
amosite (AM)	0	0.0E+00	0.0E+00
anthophyllite (AN)	0	0.0E+00	0.0E+00
crocidolite (CR)	0	0.0E+00	0.0E+00
tremolite (TR)	0	0.0E+00	0.0E+00
winchite/richterite/tremolite/actinolite (WRTA)	0	0.0E+00	0.0E+00
other amphibole (OA)	0	0.0E+00	0.0E+00
other mineral class (OM)	0	0.0E+00	0.0E+00

RC

Σ Asbestos Structure Mass (g)		Mass fraction (as %)
**Total TEM**		
WRTA	0.00E+00	0.0000%
OA	0.00E+00	0.0000%
CH	4.53E-07	########
AC	2.23E-12	0.0017%
AM	0.00E+00	0.0000%
AN	0.00E+00	0.0000%
CR	0.00E+00	0.0000%
TR	7.41E-08	57.4344%
**PCME**		
WRTA	0.00E+00	0.0000%
OA	0.00E+00	0.0000%
CH	3.16E-08	24.5011%
AC	0.00E+00	0.0000%
AM	0.00E+00	0.0000%
AN	0.00E+00	0.0000%
CR	0.00E+00	0.0000%
TR	0.00E+00	0.0000%

Asbestos structure mass = Length • Width^2 • Density • 1E-12

LA Density = 3.1 g/cm^3
OA Density = 3.0 g/cm^3
CH Density = 2.6 g/cm^3
AC Density = 3.1 g/cm^3
AM Density = 3.5 g/cm^3
AN Density = 3.0 g/cm^3
CR Density = 3.3 g/cm^3
TR Density = 3.0 g/cm^3

Csoil (mass fraction, as %) = Σ Asbestos structure mass • Sensitivity • 100

Equations:

Filter Loading (s/mm^2) = N / (GOx • Ago)
Sensitivity (1/g) = EFA / (GOx • Ago • M • f • Q_R)
Csoil (s/g) = N • Sensitivity

Asbestos Types:

AC - actinolite
AM - amosite
AN - anthophyllite
CH - chrysotile
CR - crocidolite
TR - tremolite
LA - Libby amphibole
OA - other amphibole
NAM - non-asbestos material
OM - other mineral type (specify in "other mineral description" field)

Structure Types:

High mag: Length ≥ 0.5 um, Aspect Ratio ≥ 3:1
PCME: Length > 5 um, Width ≥ 0.25 um, Aspect Ratio ≥ 3:1

Sumas Mtn Asbestos Methods - FBAS_LabCor Inc_14394123_SO_03-13-15_150099_TEM-EPAISO__D.xlsm
FluidizedBedEDD_v1

TEM Asbestos Structure Count for Fluidized Bed Material -- ISO
ANALYTICAL REPORT

FILE NAME:Sumas Mtn Asbestos Methods - FBAS_LabCor Inc_14394123_SO_03-13-15_150099_TEM-EPAISO__D.xlsm

SAMPLE INFORMATION		ANALYSIS PARAMETERS		
**Field Sample Number**	14394123	**EFA (mm^2)**	385	
		**GO area (mm^2) [Ago]**	0.0104	
**Matrix**	Soil	**# GOs counted - high mag**	37	
**Field QC Type**	Not QC	**# GOs counted - low mag only**	0	
**Sample Mass (g dry wt) [M]**	1.00	**F-factor [f]**	1.00E+00	
**Fluidized Bed Flow Ratio[Q_R]**	0.0122			
**Filter Status**	ANALYZED	Sensitivity - High mag	8.2E+04	1/g
		Sensitivity - PCME (high+low mag)	8.2E+04	1/g

ANALYSIS INFORMATION

**Lab Name**	LabCor Inc
**Lab Sample Number**	150099-S24
**Lab QC Type**	NOT QC
**TEM Prep Method**	Direct
**Est. particulate loading on filter**	NA

Recording Rules:

Initial (high mag)

Min. AR	Min length (um)	Min width (um)
3:1	0	0

PCME Supplemental (low mag)

Min. AR	Min length (um)	Min width (um)
3:1	0	0

Stopping Rules:

Target S (1/g)	Max Area (mm^2)	Max N

RESULTS SUMMARY

Structure Metric	Observed Structure Count (s)	Structure Filter Loading (s/mm^2)	Soil Conc. Structures per gram (s/g)
**High mag only**			
Total Asbestos	155	4.0E+02	1.3E+07
Total Chrysotile (CH)	155	4.0E+02	1.3E+07
Total Amphibole	0	0.0E+00	0.0E+00
actinolite (AC)	0	0.0E+00	0.0E+00
amosite (AM)	0	0.0E+00	0.0E+00
anthophyllite (AN)	0	0.0E+00	0.0E+00
crocidolite (CR)	0	0.0E+00	0.0E+00
tremolite (TR)	0	0.0E+00	0.0E+00
winchite/richterite/tremolite/actinolite (WRTA)	0	0.0E+00	0.0E+00
other amphibole (OA)	0	0.0E+00	0.0E+00
other mineral class (OM)	0	0.0E+00	0.0E+00
**PCME (high+low mag)**			
Total Asbestos	7	1.8E+01	5.8E+05
Total Chrysotile (CH)	7	1.8E+01	5.8E+05
Total Amphibole	0	0.0E+00	0.0E+00
actinolite (AC)	0	0.0E+00	0.0E+00
amosite (AM)	0	0.0E+00	0.0E+00
anthophyllite (AN)	0	0.0E+00	0.0E+00
crocidolite (CR)	0	0.0E+00	0.0E+00
tremolite (TR)	0	0.0E+00	0.0E+00
winchite/richterite/tremolite/actinolite (WRTA)	0	0.0E+00	0.0E+00
other amphibole (OA)	0	0.0E+00	0.0E+00
other mineral class (OM)	0	0.0E+00	0.0E+00

Σ Asbestos Structure Mass (g)		Mass fraction (as %)
**Total TEM**		
WRTA	0.00E+00	0.0000%
OA	0.00E+00	0.0000%
CH	4.80E-08	39.5547%
AC	0.00E+00	0.0000%
AM	0.00E+00	0.0000%
AN	0.00E+00	0.0000%
CR	0.00E+00	0.0000%
TR	0.00E+00	0.0000%
**PCME**		
WRTA	0.00E+00	0.0000%
OA	0.00E+00	0.0000%
CH	1.11E-09	0.9172%
AC	0.00E+00	0.0000%
AM	0.00E+00	0.0000%
AN	0.00E+00	0.0000%
CR	0.00E+00	0.0000%
TR	0.00E+00	0.0000%

Asbestos structure mass = Length • Width^2 • Density • 1E-12

LA Density = 3.1 g/cm^3
OA Density = 3.0 g/cm^3
CH Density = 2.6 g/cm^3
AC Density = 3.1 g/cm^3
AM Density = 3.5 g/cm^3
AN Density = 3.0 g/cm^3
CR Density = 3.3 g/cm^3
TR Density = 3.0 g/cm^3

Csoil (mass fraction, as %) = Σ Asbestos structure mass • Sensitivity • 100

Equations:

Filter Loading (s/mm^2) = N / (GOx • Ago)
Sensitivity (1/g) = EFA / (GOx • Ago • M • f • Q_R)
Csoil (s/g) = N • Sensitivity

Asbestos Types:

AC - actinolite
AM - amosite
AN - anthophyllite
CH - chrysotile
CR - crocidolite
TR - tremolite
LA - Libby amphibole
OA - other amphibole
NAM - non-asbestos material
OM - other mineral type (specify in "other mineral description" field)

Structure Types:

High mag: Length ≥ 0.5 um, Aspect Ratio ≥ 3:1
PCME: Length > 5 um, Width ≥ 0.25 um, Aspect Ratio ≥ 3:1

Sumas Mtn Asbestos Methods - FBAS_LabCor Inc_14394124_SO_03-15-15_150099_TEM-EPAISO__D.xlsm
FluidizedBedEDD_v1

TEM Asbestos Structure Count for Fluidized Bed Material -- ISO
ANALYTICAL REPORT

FILE NAME:Sumas Mtn Asbestos Methods - FBAS_LabCor Inc_14394124_SO_03-15-15_150099_TEM-EPAISO__D.xlsm

SAMPLE INFORMATION		ANALYSIS PARAMETERS		
**Field Sample Number**	14394124	**EFA (mm^2)**	385	
		**GO area (mm^2) [Ago]**	0.0104	
**Matrix**	Soil	**# GOs counted - high mag**	100	
**Field QC Type**	Not QC	**# GOs counted - low mag only**	0	
**Sample Mass (g dry wt) [M]**	1.01	**F-factor [f]**	1.00E+00	
**Fluidized Bed Flow Ratio[Q_R]**	0.0122			
**Filter Status**	ANALYZED	Sensitivity - High mag	3.0E+04	1/g
		Sensitivity - PCME (high+low mag)	3.0E+04	1/g

ANALYSIS INFORMATION

**Lab Name**	LabCor Inc
**Lab Sample Number**	150099-S25
**Lab QC Type**	NOT QC
**TEM Prep Method**	Direct
**Est. particulate loading on filter**	NA

Recording Rules:

Initial (high mag)

Min. AR	Min length (um)	Min width (um)
3:1	0	0

PCME Supplemental (low mag)

Min. AR	Min length (um)	Min width (um)
3:1	0	0

Stopping Rules:

Target S (1/g)	Max Area (mm^2)	Max N

RESULTS SUMMARY

Structure Metric	Observed Structure Count (s)	Structure Filter Loading (s/mm²)	Soil Conc. Structures per gram (s/g)
**High mag only**			
Total Asbestos	57	5.5E+01	1.7E+06
Total Chrysotile (CH)	57	5.5E+01	1.7E+06
Total Amphibole	0	0.0E+00	0.0E+00
actinolite (AC)	0	0.0E+00	0.0E+00
amosite (AM)	0	0.0E+00	0.0E+00
anthophyllite (AN)	0	0.0E+00	0.0E+00
crocidolite (CR)	0	0.0E+00	0.0E+00
tremolite (TR)	0	0.0E+00	0.0E+00
winchite/richterite/tremolite/actinolite (WRTA)	0	0.0E+00	0.0E+00
other amphibole (OA)	0	0.0E+00	0.0E+00
other mineral class (OM)	0	0.0E+00	0.0E+00
**PCME (high+low mag)**			
Total Asbestos	2	1.9E+00	6.0E+04
Total Chrysotile (CH)	2	1.9E+00	6.0E+04
Total Amphibole	0	0.0E+00	0.0E+00
actinolite (AC)	0	0.0E+00	0.0E+00
amosite (AM)	0	0.0E+00	0.0E+00
anthophyllite (AN)	0	0.0E+00	0.0E+00
crocidolite (CR)	0	0.0E+00	0.0E+00
tremolite (TR)	0	0.0E+00	0.0E+00
winchite/richterite/tremolite/actinolite (WRTA)	0	0.0E+00	0.0E+00
other amphibole (OA)	0	0.0E+00	0.0E+00
other mineral class (OM)	0	0.0E+00	0.0E+00

Σ Asbestos Structure Mass (g)		Mass fraction (as %)
**Total TEM**		
WRTA	0.00E+00	0.0000%
OA	0.00E+00	0.0000%
CH	2.85E-09	0.8598%
AC	0.00E+00	0.0000%
AM	0.00E+00	0.0000%
AN	0.00E+00	0.0000%
CR	0.00E+00	0.0000%
TR	0.00E+00	0.0000%
**PCME**		
WRTA	0.00E+00	0.0000%
OA	0.00E+00	0.0000%
CH	5.30E-11	0.0160%
AC	0.00E+00	0.0000%
AM	0.00E+00	0.0000%
AN	0.00E+00	0.0000%
CR	0.00E+00	0.0000%
TR	0.00E+00	0.0000%

Asbestos structure mass = Length • Width^2 • Density • 1E-12

LA Density = 3.1 g/cm^3
OA Density = 3.0 g/cm^3
CH Density = 2.6 g/cm^3
AC Density = 3.1 g/cm^3
AM Density = 3.5 g/cm^3
AN Density = 3.0 g/cm^3
CR Density = 3.3 g/cm^3
TR Density = 3.0 g/cm^3

Csoil (mass fraction, as %) = Σ Asbestos structure mass • Sensitivity • 100

Equations:

Filter Loading (s/mm^2) = N / (GOx • Ago)
Sensitivity (1/g) = EFA / (GOx • Ago • M • f • Q_R)
Csoil (s/g) = N • Sensitivity

Asbestos Types:

AC - actinolite
AM - amosite
AN - anthophyllite
CH - chrysotile
CR - crocidolite
TR - tremolite
LA - Libby amphibole
OA - other amphibole
NAM - non-asbestos material
OM - other mineral type (specify in "other mineral description" field)

Structure Types:

High mag: Length ≥ 0.5 um, Aspect Ratio ≥ 3:1
PCME: Length > 5 um, Width ≥ 0.25 um, Aspect Ratio ≥ 3:1

Report Printed: 7/1/2015, 1:16 PM

Sumas Mtn Asbestos Methods - FBAS_LabCor Inc_14394125_SO_03-15-15_150099_TEM-EPAISO__D.xlsm
FluidizedBedEDD_v1

TEM Asbestos Structure Count for Fluidized Bed Material -- ISO
ANALYTICAL REPORT

FILE NAME:Sumas Mtn Asbestos Methods - FBAS_LabCor Inc_14394125_SO_03-15-15_150099_TEM-EPAISO__D.xlsm

SAMPLE INFORMATION		ANALYSIS PARAMETERS		
**Field Sample Number**	14394125	**EFA (mm^2)**	385	
		**GO area (mm^2) [Ago]**	0.0104	
**Matrix**	Soil	**# GOs counted - high mag**	100	
**Field QC Type**	Not QC	**# GOs counted - low mag only**	0	
**Sample Mass (g dry wt) [M]**	1.01	**F-factor [f]**	1.00E+00	
**Fluidized Bed Flow Ratio[Q_R]**	0.0122			
**Filter Status**	ANALYZED	Sensitivity - High mag	3.0E+04	1/g
		Sensitivity - PCME (high+low mag)	3.0E+04	1/g

ANALYSIS INFORMATION

**Lab Name**	LabCor Inc
**Lab Sample Number**	150099-S26
**Lab QC Type**	NOT QC
**TEM Prep Method**	Direct
**Est. particulate loading on filter**	NA

Recording Rules:

Initial (high mag)

Min. AR	Min length (um)	Min width (um)
3:1	0	0

PCME Supplemental (low mag)

Min. AR	Min length (um)	Min width (um)
3:1	0	0

Stopping Rules:

Target S (1/g)	Max Area (mm^2)	Max N

RESULTS SUMMARY

Structure Metric	Observed Structure Count (s)	Structure Filter Loading (s/mm^2)	Soil Conc. Structures per gram (s/g)
**High mag only**			
Total Asbestos	8	7.7E+00	2.4E+05
Total Chrysotile (CH)	8	7.7E+00	2.4E+05
Total Amphibole	0	0.0E+00	0.0E+00
actinolite (AC)	0	0.0E+00	0.0E+00
amosite (AM)	0	0.0E+00	0.0E+00
anthophyllite (AN)	0	0.0E+00	0.0E+00
crocidolite (CR)	0	0.0E+00	0.0E+00
tremolite (TR)	0	0.0E+00	0.0E+00
winchite/richterite/tremolite/actinolite (WRTA)	0	0.0E+00	0.0E+00
other amphibole (OA)	0	0.0E+00	0.0E+00
other mineral class (OM)	0	0.0E+00	0.0E+00
**PCME (high+low mag)**			
Total Asbestos	0	0.0E+00	0.0E+00
Total Chrysotile (CH)	0	0.0E+00	0.0E+00
Total Amphibole	0	0.0E+00	0.0E+00
actinolite (AC)	0	0.0E+00	0.0E+00
amosite (AM)	0	0.0E+00	0.0E+00
anthophyllite (AN)	0	0.0E+00	0.0E+00
crocidolite (CR)	0	0.0E+00	0.0E+00
tremolite (TR)	0	0.0E+00	0.0E+00
winchite/richterite/tremolite/actinolite (WRTA)	0	0.0E+00	0.0E+00
other amphibole (OA)	0	0.0E+00	0.0E+00
other mineral class (OM)	0	0.0E+00	0.0E+00

Σ Asbestos Structure Mass (g)		Mass fraction (as %)
**Total TEM**		
WRTA	0.00E+00	0.0000%
OA	0.00E+00	0.0000%
CH	1.13E-12	0.0003%
AC	0.00E+00	0.0000%
AM	0.00E+00	0.0000%
AN	0.00E+00	0.0000%
CR	0.00E+00	0.0000%
TR	0.00E+00	0.0000%
**PCME**		
WRTA	0.00E+00	0.0000%
OA	0.00E+00	0.0000%
CH	0.00E+00	0.0000%
AC	0.00E+00	0.0000%
AM	0.00E+00	0.0000%
AN	0.00E+00	0.0000%
CR	0.00E+00	0.0000%
TR	0.00E+00	0.0000%

Asbestos structure mass = Length • Width^2 • Density • 1E-12

LA Density = 3.1 g/cm^3
OA Density = 3.0 g/cm^3
CH Density = 2.6 g/cm^3
AC Density = 3.1 g/cm^3
AM Density = 3.5 g/cm^3
AN Density = 3.0 g/cm^3
CR Density = 3.3 g/cm^3
TR Density = 3.0 g/cm^3

Csoil (mass fraction, as %) = Σ Asbestos structure mass • Sensitivity • 100

Equations:

Filter Loading (s/mm^2) = N / (GOx • Ago)
Sensitivity (1/g) = EFA / (GOx • Ago • M • f • Q_R)
Csoil (s/g) = N • Sensitivity

Asbestos Types:

AC - actinolite
AM - amosite
AN - anthophyllite
CH - chrysotile
CR - crocidolite
TR - tremolite
LA - Libby amphibole
OA - other amphibole
NAM - non-asbestos material
OM - other mineral type (specify in "other mineral description" field)

Structure Types:

High mag: Length ≥ 0.5 um, Aspect Ratio ≥ 3:1
PCME: Length > 5 um, Width ≥ 0 25 um, Aspect Ratio ≥ 3:1

Sumas Mtn Asbestos Methods - FBAS_LabCor Inc_14394126_SO_02-26-15_150099_TEM-EPAISO__D.xlsm
FluidizedBedEDD_v1

TEM Asbestos Structure Count for Fluidized Bed Material -- ISO
ANALYTICAL REPORT

FILE NAME:Sumas Mtn Asbestos Methods - FBAS_LabCor Inc_14394126_SO_02-26-15_150099_TEM-EPAISO__D.xlsm

SAMPLE INFORMATION		ANALYSIS PARAMETERS		
**Field Sample Number**	14394126	**EFA (mm^2)**	385	
		**GO area (mm^2) [Ago]**	0.0104	
**Matrix**	Soil	**# GOs counted - high mag**	100	
**Field QC Type**	Not QC	**# GOs counted - low mag only**	0	
**Sample Mass (g dry wt) [M]**	1.00	**F-factor [f]**	1.00E+00	
**Fluidized Bed Flow Ratio[Q_R]**	0.0122			
**Filter Status**	ANALYZED	Sensitivity - High mag	3.0E+04	1/g
		Sensitivity - PCME (high+low mag)	3.0E+04	1/g

ANALYSIS INFORMATION

**Lab Name**	LabCor Inc
**Lab Sample Number**	150099-S27
**Lab QC Type**	NOT QC
**TEM Prep Method**	Direct
**Est. particulate loading on filter**	NA

Recording Rules:

Initial (high mag)

Min. AR	Min length (um)	Min width (um)
3:1	0	0

PCME Supplemental (low mag)

Min. AR	Min length (um)	Min width (um)
3:1	0	0

Stopping Rules:

Target S (1/g)	Max Area (mm^2)	Max N

RESULTS SUMMARY

Structure Metric	Observed Structure Count (s)	Structure Filter Loading (s/mm^2)	Soil Conc. Structures per gram (s/g)
**High mag only**			
Total Asbestos	0	0.0E+00	0.0E+00
Total Chrysotile (CH)	0	0.0E+00	0.0E+00
Total Amphibole	0	0.0E+00	0.0E+00
actinolite (AC)	0	0.0E+00	0.0E+00
amosite (AM)	0	0.0E+00	0.0E+00
anthophyllite (AN)	0	0.0E+00	0.0E+00
crocidolite (CR)	0	0.0E+00	0.0E+00
tremolite (TR)	0	0.0E+00	0.0E+00
winchite/richterite/tremolite/actinolite (WRTA)	0	0.0E+00	0.0E+00
other amphibole (OA)	0	0.0E+00	0.0E+00
other mineral class (OM)	0	0.0E+00	0.0E+00
**PCME (high+low mag)**			
Total Asbestos	0	0.0E+00	0.0E+00
Total Chrysotile (CH)	0	0.0E+00	0.0E+00
Total Amphibole	0	0.0E+00	0.0E+00
actinolite (AC)	0	0.0E+00	0.0E+00
amosite (AM)	0	0.0E+00	0.0E+00
anthophyllite (AN)	0	0.0E+00	0.0E+00
crocidolite (CR)	0	0.0E+00	0.0E+00
tremolite (TR)	0	0.0E+00	0.0E+00
winchite/richterite/tremolite/actinolite (WRTA)	0	0.0E+00	0.0E+00
other amphibole (OA)	0	0.0E+00	0.0E+00
other mineral class (OM)	0	0.0E+00	0.0E+00

WJ PC MC

	Σ Asbestos Structure Mass (g)	Mass fraction (as %)
**Total TEM**		
WRTA	0.00E+00	0.0000%
OA	0.00E+00	0.0000%
CH	0.00E+00	0.0000%
AC	0.00E+00	0.0000%
AM	0.00E+00	0.0000%
AN	0.00E+00	0.0000%
CR	0.00E+00	0.0000%
TR	0.00E+00	0.0000%
**PCME**		
WRTA	0.00E+00	0.0000%
OA	0.00E+00	0.0000%
CH	0.00E+00	0.0000%
AC	0.00E+00	0.0000%
AM	0.00E+00	0.0000%
AN	0.00E+00	0.0000%
CR	0.00E+00	0.0000%
TR	0.00E+00	0.0000%

Asbestos structure mass = Length • Width^2 • Density • 1E-12

LA Density = 3.1 g/cm^3
OA Density = 3.0 g/cm^3
CH Density = 2.6 g/cm^3
AC Density = 3.1 g/cm^3
AM Density = 3.5 g/cm^3
AN Density = 3.0 g/cm^3
CR Density = 3.3 g/cm^3
TR Density = 3.0 g/cm^3

Csoil (mass fraction, as %) = Σ Asbestos structure mass • Sensitivity • 100

Equations:

Filter Loading (s/mm^2) = N / (GOx • Ago)
Sensitivity (1/g) = EFA / (GOx • Ago • M • f • Q_R)
Csoil (s/g) = N • Sensitivity

Asbestos Types:

AC - actinolite
AM - amosite
AN - anthophyllite
CH - chrysotile
CR - crocidolite
TR - tremolite
LA - Libby amphibole
OA - other amphibole
NAM - non-asbestos material
OM - other mineral type (specify in "other mineral description" field)

Structure Types:

High mag: Length ≥ 0.5 um, Aspect Ratio ≥ 3:1
PCME: Length > 5 um, Width ≥ 0.25 um, Aspect Ratio ≥ 3:1

Report Printed: 7/1/2015, 1:17 PM

Sumas Mtn Asbestos Methods - FBAS_LabCor Inc_14394127_SO_02-26-15_150099_TEM-EPAISO__D.xlsm
FluidizedBedEDD_v1

TEM Asbestos Structure Count for Fluidized Bed Material -- ISO
ANALYTICAL REPORT

FILE NAME:Sumas Mtn Asbestos Methods - FBAS_LabCor Inc_14394127_SO_02-26-15_150099_TEM-EPAISO__D.xlsm

SAMPLE INFORMATION		ANALYSIS PARAMETERS		
**Field Sample Number**	14394127	**EFA (mm^2)**	385	
		**GO area (mm^2) [Ago]**	0.0104	
**Matrix**	Soil	**# GOs counted - high mag**	60	
**Field QC Type**	Not QC	**# GOs counted - low mag only**	0	
**Sample Mass (g dry wt) [M]**	1.00	**F-factor [f]**	1.00E+00	
**Fluidized Bed Flow Ratio[Q_R]**	0.0122			
**Filter Status**	ANALYZED	Sensitivity - High mag	5.1E+04	1/g
		Sensitivity - PCME (high+low mag)	5.1E+04	1/g

1/1000

ANALYSIS INFORMATION

Lab Name LabCor Inc
Lab Sample Number 150099-S28
Lab QC Type NOT QC
TEM Prep Method Direct
Est. particulate loading on filter NA

Recording Rules:

Initial (high mag)

Min. AR	Min length (um)	Min width (um)
3:1	0	0

PCME Supplemental (low mag)

Min. AR	Min length (um)	Min width (um)
3:1	0	0

Stopping Rules:

Target S (1/g)	Max Area (mm^2)	Max N

RESULTS SUMMARY

Structure Metric	Observed Structure Count (s)	Structure Filter Loading (s/mm^2)	Soil Conc. Structures per gram (s/g)
**High mag only**			
Total Asbestos	130	2.1E+02	6.6E+06
Total Chrysotile (CH)	128	2.1E+02	6.5E+06
Total Amphibole	2	3.2E+00	1.0E+05
actinolite (AC)	2	3.2E+00	1.0E+05
amosite (AM)	0	0.0E+00	0.0E+00
anthophyllite (AN)	0	0.0E+00	0.0E+00
crocidolite (CR)	0	0.0E+00	0.0E+00
tremolite (TR)	0	0.0E+00	0.0E+00
winchite/richterite/tremolite/actinolite (WRTA)	0	0.0E+00	0.0E+00
other amphibole (OA)	0	0.0E+00	0.0E+00
other mineral class (OM)	0	0.0E+00	0.0E+00
**PCME (high+low mag)**			
Total Asbestos	5	8.1E+00	2.5E+05
Total Chrysotile (CH)	5	8.1E+00	2.5E+05
Total Amphibole	0	0.0E+00	0.0E+00
actinolite (AC)	0	0.0E+00	0.0E+00
amosite (AM)	0	0.0E+00	0.0E+00
anthophyllite (AN)	0	0.0E+00	0.0E+00
crocidolite (CR)	0	0.0E+00	0.0E+00
tremolite (TR)	0	0.0E+00	0.0E+00
winchite/richterite/tremolite/actinolite (WRTA)	0	0.0E+00	0.0E+00
other amphibole (OA)	0	0.0E+00	0.0E+00
other mineral class (OM)	0	0.0E+00	0.0E+00

	Σ Asbestos Structure Mass (g)	Mass fraction (as %)
**Total TEM**		
WRTA	0.00E+00	0.0000%
OA	0.00E+00	0.0000%
CH	7.12E-09	3.6087%
AC	1.45E-12	0.0007%
AM	0.00E+00	0.0000%
AN	0.00E+00	0.0000%
CR	0.00E+00	0.0000%
TR	0.00E+00	0.0000%
**PCM[illegible]**		
WRTA	0.00E+00	0.0000%
OA	0.00E+00	0.0000%
CH	1.31E-09	0.6658%
AC	0.00E+00	0.0000%
AM	0.00E+00	0.0000%
AN	0.00E+00	0.0000%
CR	0.00E+00	0.0000%
TR	0.00E+00	0.0000%

Asbestos structure mass = Length • $Width^2$ • Density • 1E-12

LA Density = 3.1 g/cm^3
OA Density = 3.0 g/cm^3
CH Density = 2.6 g/cm^3
AC Density = 3.1 g/cm^3
AM Density = 3.5 g/cm^3
AN Density = 3.0 g/cm^3
CR Density = 3.3 g/cm^3
TR Density = 3.0 g/cm^3

Csoil (mass fraction, as %) = Σ Asbestos structure mass • Sensitivity • 100

Equations:

Filter Loading (s/mm^2) = N / (GOx • Ago)
Sensitivity (1/g) = EFA / (GOx • Ago • M • f • Q_R)
Csoil (s/g) = N • Sensitivity

Asbestos Types:

AC - actinolite
AM - amosite
AN - anthophyllite
CH - chrysotile
CR - crocidolite
TR - tremolite
LA - Libby amphibole
OA - other amphibole
NAM - non-asbestos material
OM - other mineral type (specify in "other mineral description" field)

Structure Types:

High mag: Length ≥ 0.5 um, Aspect Ratio ≥ 3:1
PCME: Length > 5 um, Width ≥ 0.25 um, Aspect Ratio ≥ 3:1

Sumas Mtn Asbestos Methods - FBAS_LabCor Inc_14394128_SO_03-02-15_150099_TEM-EPAISO__D.xlsm
FluidizedBedEDD_v1

TEM Asbestos Structure Count for Fluidized Bed Material -- ISO
ANALYTICAL REPORT

FILE NAME:Sumas Mtn Asbestos Methods - FBAS_LabCor Inc_14394128_SO_03-02-15_150099_TEM-EPAISO__D.xlsm

SAMPLE INFORMATION		ANALYSIS PARAMETERS		
**Field Sample Number**	14394128	**EFA (mm²)**	385	
		**GO area (mm²) [Ago]**	0.0104	
**Matrix**	Soil	**# GOs counted - high mag**	6	
**Field QC Type**	Not QC	**# GOs counted - low mag only**	0	
**Sample Mass (g dry wt) [M]**	1.00	**F-factor [f]**	1.00E+00	
**Fluidized Bed Flow Ratio[Q_R]**	0.0122			
**Filter Status**	ANALYZED	Sensitivity - High mag	5.1E+05	1/g
		Sensitivity - PCME (high+low mag)	5.1E+05	1/g

ANALYSIS INFORMATION

Lab Name LabCor Inc
Lab Sample Number 150099-S29
Lab QC Type NOT QC
TEM Prep Method Direct
Est. particulate loading on filter NA

Recording Rules:

Initial (high mag)

Min. AR	Min length (um)	Min width (um)
3:1	0	0

PCME Supplemental (low mag)

Min. AR	Min length (um)	Min width (um)
3:1	0	0

Stopping Rules:

Target S (1/g)	Max Area (mm²)	Max N

RESULTS SUMMARY

Structure Metric	Observed Structure Count (s)	Structure Filter Loading (s/mm²)	Soil Conc. Structures per gram (s/g)
**High mag only**			
Total Asbestos	206	3.3E+03	1.0E+08
Total Chrysotile (CH)	206	3.3E+03	1.0E+08
Total Amphibole	0	0.0E+00	0.0E+00
actinolite (AC)	0	0.0E+00	0.0E+00
amosite (AM)	0	0.0E+00	0.0E+00
anthophyllite (AN)	0	0.0E+00	0.0E+00
crocidolite (CR)	0	0.0E+00	0.0E+00
tremolite (TR)	0	0.0E+00	0.0E+00
winchite/richterite/tremolite/actinolite (WRTA)	0	0.0E+00	0.0E+00
other amphibole (OA)	0	0.0E+00	0.0E+00
other mineral class (OM)	0	0.0E+00	0.0E+00
**PCME (high+low mag)**			
Total Asbestos	4	6.4E+01	2.0E+06
Total Chrysotile (CH)	4	6.4E+01	2.0E+06
Total Amphibole	0	0.0E+00	0.0E+00
actinolite (AC)	0	0.0E+00	0.0E+00
amosite (AM)	0	0.0E+00	0.0E+00
anthophyllite (AN)	0	0.0E+00	0.0E+00
crocidolite (CR)	0	0.0E+00	0.0E+00
tremolite (TR)	0	0.0E+00	0.0E+00
winchite/richterite/tremolite/actinolite (WRTA)	0	0.0E+00	0.0E+00
other amphibole (OA)	0	0.0E+00	0.0E+00
other mineral class (OM)	0	0.0E+00	0.0E+00

Σ Asbestos Structure Mass (g)		Mass fraction (as %)
**Total TEM**		
WRTA	0.00E+00	0.0000%
OA	0.00E+00	0.0000%
CH	1.57E-08	79.9573%
AC	0.00E+00	0.0000%
AM	0.00E+00	0.0000%
AN	0.00E+00	0.0000%
CR	0.00E+00	0.0000%
TR	0.00E+00	0.0000%
**PCME**		
WRTA	0.00E+00	0.0000%
OA	0.00E+00	0.0000%
CH	6.46E-11	0.3284%
AC	0.00E+00	0.0000%
AM	0.00E+00	0.0000%
AN	0.00E+00	0.0000%
CR	0.00E+00	0.0000%
TR	0.00E+00	0.0000%

Asbestos structure mass = Length • Width² • Density • 1E-12

LA Density = 3.1 g/cm³
OA Density = 3.0 g/cm³
CH Density = 2.6 g/cm³
AC Density = 3.1 g/cm³
AM Density = 3.5 g/cm³
AN Density = 3.0 g/cm³
CR Density = 3.3 g/cm³
TR Density = 3.0 g/cm³

Csoil (mass fraction, as %) = Σ Asbestos structure mass • Sensitivity • 100

Equations:

Filter Loading (s/mm²) = N / (GOx • Ago)
Sensitivity (1/g) = EFA / (GOx • Ago • M • f • Q_R)
Csoil (s/g) = N • Sensitivity

Asbestos Types:

AC - actinolite
AM - amosite
AN - anthophyllite
CH - chrysotile
CR - crocidolite
TR - tremolite
LA - Libby amphibole
OA - other amphibole
NAM - non-asbestos material
OM - other mineral type (specify in "other mineral description" field)

Structure Types:

High mag: Length ≥ 0.5 um, Aspect Ratio ≥ 3:1
PCME: Length > 5 um, Width ≥ 0.25 um, Aspect Ratio ≥ 3:1

Sumas Mtn Asbestos Methods - FBAS_LabCor Inc_14394129_SO_03-02-15_150099_TEM-EPAISO__D.xlsm
FluidizedBedEDD_v1

TEM Asbestos Structure Count for Fluidized Bed Material -- ISO
ANALYTICAL REPORT

FILE NAME:Sumas Mtn Asbestos Methods - FBAS_LabCor Inc_14394129_SO_03-02-15_150099_TEM-EPAISO__D.xlsm

SAMPLE INFORMATION		ANALYSIS PARAMETERS		
**Field Sample Number**	14394129	**EFA (mm^2)**	385	
		**GO area (mm^2) [Ago]**	0.0104	
**Matrix**	Soil	**# GOs counted - high mag**	2	
**Field QC Type**	Not QC	**# GOs counted - low mag only**	0	
**Sample Mass (g dry wt) [M]**	1 00	**F-factor [f]**	1.00E+00	
**Fluidized Bed Flow Ratio[Q_R]**	0.0122			
**Filter Status**	ANALYZED	Sensitivity - High mag	1.5E+06	1/g
		Sensitivity - PCME (high+low mag)	1.5E+06	1/g

ANALYSIS INFORMATION

Lab Name LabCor Inc
Lab Sample Number 150099-S30
Lab QC Type NOT QC
TEM Prep Method Direct
Est. particulate loading on filter NA

Recording Rules:

Initial (high mag)

Min. AR	Min length (um)	Min width (um)
3:1	0	0

PCME Supplemental (low mag)

Min. AR	Min length (um)	Min width (um)
3:1	0	0

Stopping Rules:

Target S (1/g)	Max Area (mm^2)	Max N

RESULTS SUMMARY

Structure Metric	Observed Structure Count (s)	Structure Filter Loading (s/mm^2)	Soil Conc. Structures per gram (s/g)
**High mag only**			
Total Asbestos	217	1.0E+04	3.3E+08
Total Chrysotile (CH)	217	1.0E+04	3.3E+08
Total Amphibole	0	0.0E+00	0.0E+00
actinolite (AC)	0	0.0E+00	0 0E+00
amosite (AM)	0	0.0E+00	0 0E+00
anthophyllite (AN)	0	0.0E+00	0.0E+00
crocidolite (CR)	0	0.0E+00	0.0E+00
tremolite (TR)	0	0.0E+00	0.0E+00
winchite/richterite/tremolite/actinolite (WRTA)	0	0.0E+00	0.0E+00
other amphibole (OA)	0	0.0E+00	0 0E+00
other mineral class (OM)	0	0.0E+00	0.0E+00
**PCME (high+low mag)**			
Total Asbestos	2	9.7E+01	3.0E+06
Total Chrysotile (CH)	2	9.7E+01	3.0E+06
Total Amphibole	0	0.0E+00	0 0E+00
actinolite (AC)	0	0.0E+00	0.0E+00
amosite (AM)	0	0.0E+00	0.0E+00
anthophyllite (AN)	0	0.0E+00	0.0E+00
crocidolite (CR)	0	0.0E+00	0.0E+00
tremolite (TR)	0	0.0E+00	0.0E+00
winchite/richterite/tremolite/actinolite (WRTA)	0	0.0E+00	0.0E+00
other amphibole (OA)	0	0.0E+00	0.0E+00
other mineral class (OM)	0	0.0E+00	0 0E+00

Σ Asbestos Structure Mass (g)		Mass fraction (as %)
**Total TEM**		
WRTA	0.00E+00	0.0000%
OA	0.00E+00	0.0000%
CH	1.41E-08	########
AC	0.00E+00	0.0000%
AM	0.00E+00	0.0000%
AN	0.00E+00	0.0000%
CR	0.00E+00	0.0000%
TR	0.00E+00	0.0000%
**PCME**		
WRTA	0.00E+00	0.0000%
OA	0.00E+00	0.0000%
CH	1.85E-10	2.8168%
AC	0.00E+00	0.0000%
AM	0.00E+00	0.0000%
AN	0.00E+00	0.0000%
CR	0.00E+00	0.0000%
TR	0.00E+00	0.0000%

Asbestos structure mass = Length • Width^2 • Density • 1E-12

LA Density = 3.1 g/cm^3
OA Density = 3.0 g/cm^3
CH Density = 2.6 g/cm^3
AC Density = 3.1 g/cm^3
AM Density = 3.5 g/cm^3
AN Density = 3.0 g/cm^3
CR Density = 3.3 g/cm^3
TR Density = 3.0 g/cm^3

Csoil (mass fraction, as %) = Σ Asbestos structure mass • Sensitivity • 100

Equations:

Filter Loading (s/mm^2) = N / (GOx • Ago)
Sensitivity (1/g) = EFA / (GOx • Ago • M • f • Q_R)
Csoil (s/g) = N • Sensitivity

Asbestos Types:

AC - actinolite
AM - amosite
AN - anthophyllite
CH - chrysotile
CR - crocidolite
TR - tremolite
LA - Libby amphibole
OA - other amphibole
NAM - non-asbestos material
OM - other mineral type (specify in "other mineral description" field)

Structure Types:

High mag: Length ≥ 0 5 um, Aspect Ratio ≥ 3:1
PCME: Length > 5 um, Width ≥ 0.25 um, Aspect Ratio ≥ 3:1

Sumas Mtn Asbestos Methods - FBAS_LabCor Inc_14394130_SO_03-02-15_150099_TEM-EPAISO__D.xlsm
FluidizedBedEDD_v1

TEM Asbestos Structure Count for Fluidized Bed Material -- ISO
ANALYTICAL REPORT

FILE NAME:Sumas Mtn Asbestos Methods - FBAS_LabCor Inc_14394130_SO_03-02-15_150099_TEM-EPAISO__D.xlsm

SAMPLE INFORMATION		ANALYSIS PARAMETERS		
**Field Sample Number**	14394130	**EFA (mm^2)**	385	
		**GO area (mm^2) [Ago]**	0.0104	
**Matrix**	Soil	**# GOs counted - high mag**	4	
**Field QC Type**	Not QC	**# GOs counted - low mag only**	0	
**Sample Mass (g dry wt) [M]**	1.00	**F-factor [f]**	1.00E+00	
**Fluidized Bed Flow Ratio[Q_R]**	0.0122			
**Filter Status**	ANALYZED	Sensitivity - High mag	7.6E+05	1/g
		Sensitivity - PCME (high+low mag)	7.6E+05	1/g

ANALYSIS INFORMATION

**Lab Name**	LabCor Inc
**Lab Sample Number**	150099-S31
**Lab QC Type**	NOT QC
**TEM Prep Method**	Direct
**Est. particulate loading on filter**	NA

Recording Rules:

Initial (high mag)

Min. AR	Min length (um)	Min width (um)
3:1	0	0

PCME Supplemental (low mag)

Min. AR	Min length (um)	Min width (um)
3:1	0	0

Stopping Rules:

Target S (1/g)	Max Area (mm^2)	Max N

RESULTS SUMMARY

Structure Metric	Observed Structure Count (s)	Structure Filter Loading (s/mm^2)	Soil Conc. Structures per gram (s/g)
**High mag only**			
Total Asbestos	165	4.0E+03	1.3E+08
Total Chrysotile (CH)	165	4.0E+03	1.3E+08
Total Amphibole	0	0.0E+00	0.0E+00
actinolite (AC)	0	0.0E+00	0.0E+00
amosite (AM)	0	0.0E+00	0.0E+00
anthophyllite (AN)	0	0.0E+00	0.0E+00
crocidolite (CR)	0	0.0E+00	0.0E+00
tremolite (TR)	0	0.0E+00	0.0E+00
winchite/richterite/tremolite/actinolite (WRTA)	0	0.0E+00	0.0E+00
other amphibole (OA)	0	0.0E+00	0.0E+00
other mineral class (OM)	0	0.0E+00	0.0E+00
**PCME (high+low mag)**			
Total Asbestos	5	1.2E+02	3.8E+06
Total Chrysotile (CH)	5	1.2E+02	3.8E+06
Total Amphibole	0	0.0E+00	0.0E+00
actinolite (AC)	0	0.0E+00	0.0E+00
amosite (AM)	0	0.0E+00	0.0E+00
anthophyllite (AN)	0	0.0E+00	0.0E+00
crocidolite (CR)	0	0.0E+00	0.0E+00
tremolite (TR)	0	0.0E+00	0.0E+00
winchite/richterite/tremolite/actinolite (WRTA)	0	0.0E+00	0.0E+00
other amphibole (OA)	0	0.0E+00	0.0E+00
other mineral class (OM)	0	0.0E+00	0.0E+00

Σ Asbestos Structure Mass (g)		Mass fraction (as %)
**Total TEM**		
WRTA	0.00E+00	0.0000%
OA	0.00E+00	0.0000%
CH	5.10E-08	########
AC	0.00E+00	0.0000%
AM	0.00E+00	0.0000%
AN	0.00E+00	0.0000%
CR	0.00E+00	0.0000%
TR	0.00E+00	0.0000%
**PCME**		
WRTA	0.00E+00	0.0000%
OA	0.00E+00	0.0000%
CH	2.16E-08	########
AC	0.00E+00	0.0000%
AM	0.00E+00	0.0000%
AN	0.00E+00	0.0000%
CR	0.00E+00	0.0000%
TR	0.00E+00	0.0000%

Asbestos structure mass = Length • Width^2 • Density • 1E-12

LA Density = 3.1 g/cm^3
OA Density = 3 0 g/cm^3
CH Density = 2.6 g/cm^3
AC Density = 3.1 g/cm^3
AM Density = 3.5 g/cm^3
AN Density = 3.0 g/cm^3
CR Density = 3 3 g/cm^3
TR Density = 3.0 g/cm^3

Csoil (mass fraction, as %) = Σ Asbestos structure mass • Sensitivity • 100

Equations:

Filter Loading (s/mm^2) = N / (GOx • Ago)
Sensitivity (1/g) = EFA / (GOx • Ago • M • f • Q_R)
Csoil (s/g) = N • Sensitivity

Asbestos Types:

AC - actinolite
AM - amosite
AN - anthophyllite
CH - chrysotile
CR - crocidolite
TR - tremolite
LA - Libby amphibole
OA - other amphibole
NAM - non-asbestos material
OM - other mineral type (specify in "other mineral description" field)

Structure Types:

High mag: Length ≥ 0.5 um, Aspect Ratio ≥ 3:1
PCME: Length > 5 um, Width ≥ 0.25 um, Aspect Ratio ≥ 3:1

Sumas Mtn Asbestos Methods - FBAS_LabCor Inc_14394131_SO_03-04-15_150099_TEM-EPAISO__D.xlsm
FluidizedBedEDD_v1

TEM Asbestos Structure Count for Fluidized Bed Material -- ISO
ANALYTICAL REPORT

FILE NAME:Sumas Mtn Asbestos Methods - FBAS_LabCor Inc_14394131_SO_03-04-15_150099_TEM-EPAISO__D.xlsm

SAMPLE INFORMATION		ANALYSIS PARAMETERS		
**Field Sample Number**	14394131	**EFA (mm^2)**	385	
		**GO area (mm^2) [Ago]**	0 0104	
**Matrix**	Soil	**# GOs counted - high mag**	2	
**Field QC Type**	Not QC	**# GOs counted - low mag only**	0	
**Sample Mass (g dry wt) [M]**	1.01	**F-factor [f]**	1.00E+00	
**Fluidized Bed Flow Ratio[Q_R]**	0.0122			
**Filter Status**	ANALYZED	Sensitivity - High mag	1.5E+06	1/g
		Sensitivity - PCME (high+low mag)	1.5E+06	1/g

ANALYSIS INFORMATION

Lab Name LabCor Inc
Lab Sample Number 150099-S32
Lab QC Type NOT QC
TEM Prep Method Direct
Est. particulate loading on filter NA

Recording Rules:

Initial (high mag)

Min. AR	Min length (um)	Min width (um)
3:1	0	0

PCME Supplemental (low mag)

Min. AR	Min length (um)	Min width (um)
3:1	0	0

Stopping Rules:

Target S (1/g)	Max Area (mm^2)	Max N

RESULTS SUMMARY

Structure Metric	Observed Structure Count (s)	Structure Filter Loading (s/mm^2)	Soil Conc. Structures per gram (s/g)
**High mag only**			
Total Asbestos	157	7.6E+03	2.4E+08
Total Chrysotile (CH)	157	7.6E+03	2.4E+08
Total Amphibole	0	0.0E+00	0.0E+00
actinolite (AC)	0	0.0E+00	0.0E+00
amosite (AM)	0	0.0E+00	0.0E+00
anthophyllite (AN)	0	0.0E+00	0.0E+00
crocidolite (CR)	0	0.0E+00	0.0E+00
tremolite (TR)	0	0.0E+00	0.0E+00
winchite/richterite/tremolite/actinolite (WRTA)	0	0.0E+00	0.0E+00
other amphibole (OA)	0	0.0E+00	0.0E+00
other mineral class (OM)	0	0.0E+00	0.0E+00
**PCME (high+low mag)**			
Total Asbestos	6	2.9E+02	9.1E+06
Total Chrysotile (CH)	6	2.9E+02	9.1E+06
Total Amphibole	0	0.0E+00	0.0E+00
actinolite (AC)	0	0.0E+00	0.0E+00
amosite (AM)	0	0.0E+00	0.0E+00
anthophyllite (AN)	0	0.0E+00	0.0E+00
crocidolite (CR)	0	0.0E+00	0.0E+00
tremolite (TR)	0	0.0E+00	0.0E+00
winchite/richterite/tremolite/actinolite (WRTA)	0	0.0E+00	0.0E+00
other amphibole (OA)	0	0.0E+00	0.0E+00
other mineral class (OM)	0	0.0E+00	0.0E+00

Σ Asbestos Structure Mass (g)		Mass fraction (as %)
**Total TEM**		
WRTA	0.00E+00	0.0000%
OA	0.00E+00	0.0000%
CH	1.65E-07	########
AC	0.00E+00	0.0000%
AM	0.00E+00	0.0000%
AN	0.00E+00	0.0000%
CR	0.00E+00	0.0000%
TR	0.00E+00	0.0000%
**PCME**		
WRTA	0.00E+00	0.0000%
OA	0.00E+00	0.0000%
CH	3.50E-11	0.5298%
AC	0.00E+00	0.0000%
AM	0.00E+00	0.0000%
AN	0.00E+00	0.0000%
CR	0.00E+00	0.0000%
TR	0.00E+00	0.0000%

Asbestos structure mass = Length • Width^2 • Density • 1E-12

LA Density = 3.1 g/cm^3
OA Density = 3.0 g/cm^3
CH Density = 2.6 g/cm^3
AC Density = 3.1 g/cm^3
AM Density = 3.5 g/cm^3
AN Density = 3.0 g/cm^3
CR Density = 3.3 g/cm^3
TR Density = 3.0 g/cm^3

Csoil (mass fraction, as %) = Σ Asbestos structure mass • Sensitivity • 100

Equations:

Filter Loading (s/mm^2) = N / (GOx • Ago)
Sensitivity (1/g) = EFA / (GOx • Ago • M • f • Q_R)
Csoil (s/g) = N • Sensitivity

Asbestos Types:

AC - actinolite
AM - amosite
AN - anthophyllite
CH - chrysotile
CR - crocidolite
TR - tremolite
LA - Libby amphibole
OA - other amphibole
NAM - non-asbestos material
OM - other mineral type (specify in "other mineral description" field)

Structure Types:

High mag: Length ≥ 0.5 um, Aspect Ratio ≥ 3:1
PCME: Length > 5 um, Width ≥ 0.25 um, Aspect Ratio ≥ 3:1

Sumas Mtn Asbestos Methods - FBAS_LabCor Inc_14394132_SO_03-04-15_150099_TEM-EPAISO__D.xlsm
FluidizedBedEDD_v1

TEM Asbestos Structure Count for Fluidized Bed Material -- ISO
ANALYTICAL REPORT

FILE NAME:Sumas Mtn Asbestos Methods - FBAS_LabCor Inc_14394132_SO_03-04-15_150099_TEM-EPAISO__D.xlsm

SAMPLE INFORMATION		ANALYSIS PARAMETERS		
**Field Sample Number**	14394132	**EFA (mm^2)**	385	
		**GO area (mm^2) [Ago]**	0.0104	
**Matrix**	Soil	**# GOs counted - high mag**	13	
**Field QC Type**	Not QC	**# GOs counted - low mag only**	0	
**Sample Mass (g dry wt) [M]**	1.00	**F-factor [f]**	1.00E+00	
**Fluidized Bed Flow Ratio[Q_R]**	0.0122			
**Filter Status**	ANALYZED	Sensitivity - High mag	2.4E+05	1/g
		Sensitivity - PCME (high+low mag)	2.4E+05	1/g

ANALYSIS INFORMATION

**Lab Name**	LabCor Inc
**Lab Sample Number**	150099-S33
**Lab QC Type**	NOT QC
**TEM Prep Method**	Direct
**Est. particulate loading on filter**	NA

Recording Rules:

Initial (high mag)

Min. AR	Min length (um)	Min width (um)
3:1	0	0

PCME Supplemental (low mag)

Min. AR	Min length (um)	Min width (um)
3:1	0	0

Stopping Rules:

Target S (1/g)	Max Area (mm^2)	Max N

RESULTS SUMMARY

Structure Metric	Observed Structure Count (s)	Structure Filter Loading (s/mm^2)	Soil Conc. Structures per gram (s/g)
**High mag only**			
Total Asbestos	171	1.3E+03	4.0E+07
Total Chrysotile (CH)	171	1.3E+03	4.0E+07
Total Amphibole	0	0.0E+00	0.0E+00
actinolite (AC)	0	0.0E+00	0.0E+00
amosite (AM)	0	0.0E+00	0.0E+00
anthophyllite (AN)	0	0.0E+00	0.0E+00
crocidolite (CR)	0	0.0E+00	0.0E+00
tremolite (TR)	0	0.0E+00	0.0E+00
winchite/richterite/tremolite/actinolite (WRTA)	0	0.0E+00	0.0E+00
other amphibole (OA)	0	0.0E+00	0.0E+00
other mineral class (OM)	0	0.0E+00	0.0E+00
**PCME (high+low mag)**			
Total Asbestos	3	2.2E+01	7.1E+05
Total Chrysotile (CH)	3	2.2E+01	7.1E+05
Total Amphibole	0	0.0E+00	0.0E+00
actinolite (AC)	0	0.0E+00	0.0E+00
amosite (AM)	0	0.0E+00	0.0E+00
anthophyllite (AN)	0	0.0E+00	0.0E+00
crocidolite (CR)	0	0.0E+00	0.0E+00
tremolite (TR)	0	0.0E+00	0.0E+00
winchite/richterite/tremolite/actinolite (WRTA)	0	0.0E+00	0.0E+00
other amphibole (OA)	0	0.0E+00	0.0E+00
other mineral class (OM)	0	0.0E+00	0.0E+00

Σ Asbestos Structure Mass (g)		Mass fraction (as %)
**Total TEM**		
WRTA	0.00E+00	0.0000%
OA	0.00E+00	0.0000%
CH	3.11E-09	7.3187%
AC	0.00E+00	0.0000%
AM	0.00E+00	0.0000%
AN	0.00E+00	0.0000%
CR	0.00E+00	0.0000%
TR	0.00E+00	0.0000%
**PCME**		
WRTA	0.00E+00	0.0000%
OA	0.00E+00	0.0000%
CH	4.38E-11	0.1030%
AC	0.00E+00	0.0000%
AM	0.00E+00	0.0000%
AN	0.00E+00	0.0000%
CR	0.00E+00	0.0000%
TR	0.00E+00	0.0000%

Asbestos structure mass = Length • Width^2 • Density • 1E-12

LA Density = 3.1 g/cm^3
OA Density = 3.0 g/cm^3
CH Density = 2.6 g/cm^3
AC Density = 3.1 g/cm^3
AM Density = 3.5 g/cm^3
AN Density = 3.0 g/cm^3
CR Density = 3.3 g/cm^3
TR Density = 3.0 g/cm^3

Csoil (mass fraction, as %) = Σ Asbestos structure mass • Sensitivity • 100

Equations:

Filter Loading (s/mm^2) = N / (GOx • Ago)
Sensitivity (1/g) = EFA / (GOx • Ago • M • f • Q_R)
Csoil (s/g) = N • Sensitivity

Asbestos Types:

AC - actinolite
AM - amosite
AN - anthophyllite
CH - chrysotile
CR - crocidolite
TR - tremolite
LA - Libby amphibole
OA - other amphibole
NAM - non-asbestos material
OM - other mineral type (specify in "other mineral description" field)

Structure Types:

High mag: Length ≥ 0.5 um, Aspect Ratio ≥ 3:1
PCME: Length > 5 um, Width ≥ 0.25 um, Aspect Ratio ≥ 3:1

Sumas Mtn Asbestos Methods - FBAS_LabCor Inc_14394133_SO_03-04-15_150099_TEM-EPAISO__D.xlsm
FluidizedBedEDD_v1

TEM Asbestos Structure Count for Fluidized Bed Material -- ISO
ANALYTICAL REPORT

FILE NAME:Sumas Mtn Asbestos Methods - FBAS_LabCor Inc_14394133_SO_03-04-15_150099_TEM-EPAISO__D.xlsm

SAMPLE INFORMATION		ANALYSIS PARAMETERS		
**Field Sample Number**	14394133	**EFA (mm^2)**	385	
		**GO area (mm^2) [Ago]**	0 0104	
**Matrix**	Soil	**# GOs counted - high mag**	6	
**Field QC Type**	Not QC	**# GOs counted - low mag only**	0	
**Sample Mass (g dry wt) [M]**	1.00	**F-factor [f]**	1.00E+00	
**Fluidized Bed Flow Ratio[Q_R]**	0.0122			
**Filter Status**	ANALYZED	Sensitivity - High mag	5.1E+05	1/g
		Sensitivity - PCME (high+low mag)	5.1E+05	1/g

ANALYSIS INFORMATION

**Lab Name**	LabCor Inc
**Lab Sample Number**	150099-S34
**Lab QC Type**	NOT QC
**TEM Prep Method**	Direct
**Est. particulate loading on filter**	NA

Recording Rules:

Initial (high mag)

Min. AR	Min length (um)	Min width (um)
3:1	0	0

PCME Supplemental (low mag)

Min. AR	Min length (um)	Min width (um)
3:1	0	0

Stopping Rules:

Target S (1/g)	Max Area (mm^2)	Max N

RESULTS SUMMARY

Structure Metric	Observed Structure Count (s)	Structure Filter Loading (s/mm^2)	Soil Conc. Structures per gram (s/g)
**High mag only**			
Total Asbestos	161	2.6E+03	8.2E+07
Total Chrysotile (CH)	161	2.6E+03	8.2E+07
Total Amphibole	0	0.0E+00	0.0E+00
actinolite (AC)	0	0.0E+00	0.0E+00
amosite (AM)	0	0.0E+00	0.0E+00
anthophyllite (AN)	0	0.0E+00	0.0E+00
crocidolite (CR)	0	0.0E+00	0.0E+00
tremolite (TR)	0	0.0E+00	0.0E+00
winchite/richterite/tremolite/actinolite (WRTA)	0	0.0E+00	0.0E+00
other amphibole (OA)	0	0.0E+00	0.0E+00
other mineral class (OM)	0	0.0E+00	0.0E+00
**PCME (high+low mag)**			
Total Asbestos	5	8.1E+01	2.5E+06
Total Chrysotile (CH)	5	8.1E+01	2.5E+06
Total Amphibole	0	0.0E+00	0.0E+00
actinolite (AC)	0	0.0E+00	0.0E+00
amosite (AM)	0	0.0E+00	0.0E+00
anthophyllite (AN)	0	0.0E+00	0.0E+00
crocidolite (CR)	0	0.0E+00	0.0E+00
tremolite (TR)	0	0.0E+00	0.0E+00
winchite/richterite/tremolite/actinolite (WRTA)	0	0.0E+00	0.0E+00
other amphibole (OA)	0	0.0E+00	0.0E+00
other mineral class (OM)	0	0.0E+00	0.0E+00

Σ Asbestos Structure Mass (g)		Mass fraction (as %)
**Total TEM**		
WRTA	0.00E+00	0.0000%
OA	0.00E+00	0.0000%
CH	7.36E-09	37.2760%
AC	0.00E+00	0.0000%
AM	0.00E+00	0.0000%
AN	0.00E+00	0.0000%
CR	0.00E+00	0.0000%
TR	0.00E+00	0.0000%
**PCME**		
WRTA	0.00E+00	0.0000%
OA	0.00E+00	0.0000%
CH	1.22E-09	6.1945%
AC	0.00E+00	0.0000%
AM	0.00E+00	0.0000%
AN	0.00E+00	0.0000%
CR	0.00E+00	0.0000%
TR	0.00E+00	0.0000%

Asbestos structure mass = Length • Width^2 • Density • 1E-12

LA Density = 3.1 g/cm^3
OA Density = 3.0 g/cm^3
CH Density = 2.6 g/cm^3
AC Density = 3.1 g/cm^3
AM Density = 3.5 g/cm^3
AN Density = 3.0 g/cm^3
CR Density = 3.3 g/cm^3
TR Density = 3.0 g/cm^3

Csoil (mass fraction, as %) = Σ Asbestos structure mass • Sensitivity • 100

Equations:

Filter Loading (s/mm^2) = N / (GOx • Ago)
Sensitivity (1/g) = EFA / (GOx • Ago • M • f • Q_R)
Csoil (s/g) = N • Sensitivity

Asbestos Types:

AC - actinolite
AM - amosite
AN - anthophyllite
CH - chrysotile
CR - crocidolite
TR - tremolite
LA - Libby amphibole
OA - other amphibole
NAM - non-asbestos material
OM - other mineral type (specify in "other mineral description" field)

Structure Types:

High mag: Length ≥ 0.5 um, Aspect Ratio ≥ 3:1
PCME: Length > 5 um, Width ≥ 0.25 um, Aspect Ratio ≥ 3:1

Sumas Mtn Asbestos Methods - FBAS_LabCor Inc_14394134_SO_03-05-15_150099_TEM-EPAISO__D.xlsm
FluidizedBedEDD_v1

TEM Asbestos Structure Count for Fluidized Bed Material -- ISO
ANALYTICAL REPORT

FILE NAME:Sumas Mtn Asbestos Methods - FBAS_LabCor Inc_14394134_SO_03-05-15_150099_TEM-EPAISO__D.xlsm

SAMPLE INFORMATION		ANALYSIS PARAMETERS		
**Field Sample Number**	14394134	**EFA (mm^2)**	385	
		**GO area (mm^2) [Ago]**	0.0104	
**Matrix**	Soil	**# GOs counted - high mag**	9	
**Field QC Type**	Not QC	**# GOs counted - low mag only**	0	
**Sample Mass (g dry wt) [M]**	1.01	**F-factor [f]**	1.00E+00	
**Fluidized Bed Flow Ratio[Q_R]**	0.0122			
**Filter Status**	ANALYZED	Sensitivity - High mag	3.4E+05	1/g
		Sensitivity - PCME (high+low mag)	3.4E+05	1/g

ANALYSIS INFORMATION

**Lab Name**	LabCor Inc
**Lab Sample Number**	150099-S35
**Lab QC Type**	NOT QC
**TEM Prep Method**	Direct
**Est. particulate loading on filter**	NA

Recording Rules:

Initial (high mag)

Min. AR	Min length (um)	Min width (um)
3:1	0	0

PCME Supplemental (low mag)

Min. AR	Min length (um)	Min width (um)
3:1	0	0

Stopping Rules:

Target S (1/g)	Max Area (mm^2)	Max N

RESULTS SUMMARY

Structure Metric	Observed Structure Count (s)	Structure Filter Loading (s/mm^2)	Soil Conc. Structures per gram (s/g)
**High mag only**			
Total Asbestos	166	1.8E+03	5.6E+07
Total Chrysotile (CH)	166	1.8E+03	5.6E+07
Total Amphibole	0	0.0E+00	0.0E+00
actinolite (AC)	0	0.0E+00	0.0E+00
amosite (AM)	0	0.0E+00	0.0E+00
anthophyllite (AN)	0	0.0E+00	0.0E+00
crocidolite (CR)	0	0.0E+00	0.0E+00
tremolite (TR)	0	0.0E+00	0.0E+00
winchite/richterite/tremolite/actinolite (WRTA)	0	0.0E+00	0.0E+00
other amphibole (OA)	0	0.0E+00	0.0E+00
other mineral class (OM)	0	0.0E+00	0.0E+00
**PCME (high+low mag)**			
Total Asbestos	6	6.4E+01	2.0E+06
Total Chrysotile (CH)	6	6.4E+01	2.0E+06
Total Amphibole	0	0.0E+00	0.0E+00
actinolite (AC)	0	0.0E+00	0.0E+00
amosite (AM)	0	0.0E+00	0.0E+00
anthophyllite (AN)	0	0.0E+00	0.0E+00
crocidolite (CR)	0	0.0E+00	0.0E+00
tremolite (TR)	0	0.0E+00	0.0E+00
winchite/richterite/tremolite/actinolite (WRTA)	0	0.0E+00	0.0E+00
other amphibole (OA)	0	0.0E+00	0.0E+00
other mineral class (OM)	0	0.0E+00	0.0E+00

Σ Asbestos Structure Mass (g)		Mass fraction (as %)
**Total TEM**		
WRTA	0.00E+00	0.0000%
OA	0.00E+00	0.0000%
CH	2.52E-08	84.6076%
AC	0.00E+00	0.0000%
AM	0.00E+00	0.0000%
AN	0.00E+00	0.0000%
CR	0.00E+00	0.0000%
TR	0.00E+00	0.0000%
**PCME**		
WRTA	0.00E+00	0.0000%
OA	0.00E+00	0.0000%
CH	2.21E-11	0.0742%
AC	0.00E+00	0.0000%
AM	0.00E+00	0.0000%
AN	0.00E+00	0.0000%
CR	0.00E+00	0.0000%
TR	0.00E+00	0.0000%

Asbestos structure mass = Length • Width^2 • Density • 1E-12

LA Density = 3.1 g/cm^3
OA Density = 3.0 g/cm^3
CH Density = 2.6 g/cm^3
AC Density = 3.1 g/cm^3
AM Density = 3.5 g/cm^3
AN Density = 3.0 g/cm^3
CR Density = 3.3 g/cm^3
TR Density = 3.0 g/cm^3

Csoil (mass fraction, as %) = Σ Asbestos structure mass • Sensitivity • 100

Equations:

Filter Loading (s/mm^2) = N / (GOx • Ago)
Sensitivity (1/g) = EFA / (GOx • Ago • M • f • Q_R)
Csoil (s/g) = N • Sensitivity

Asbestos Types:

AC - actinolite
AM - amosite
AN - anthophyllite
CH - chrysotile
CR - crocidolite
TR - tremolite
LA - Libby amphibole
OA - other amphibole
NAM - non-asbestos material
OM - other mineral type (specify in "other mineral description" field)

Structure Types:

High mag: Length ≥ 0.5 um, Aspect Ratio ≥ 3:1
PCME: Length > 5 um, Width ≥ 0.25 um, Aspect Ratio ≥ 3:1

Sumas Mtn Asbestos Methods - FBAS_LabCor Inc_14394135_SO_03-05-15_150099_TEM-EPAISO__D.xlsm
FluidizedBedEDD_v1

TEM Asbestos Structure Count for Fluidized Bed Material -- ISO
ANALYTICAL REPORT

FILE NAME:Sumas Mtn Asbestos Methods - FBAS_LabCor Inc_14394135_SO_03-05-15_150099_TEM-EPAISO__D.xlsm

SAMPLE INFORMATION		ANALYSIS PARAMETERS		
**Field Sample Number**	14394135	**EFA (mm^2)**	385	
		**GO area (mm^2) [Ago]**	0.0104	
**Matrix**	Soil	**# GOs counted - high mag**	4	
**Field QC Type**	Not QC	**# GOs counted - low mag only**	0	
**Sample Mass (g dry wt) [M]**	1.00	**F-factor [f]**	1.00E+00	
**Fluidized Bed Flow Ratio[Q_R]**	0.0122			
**Filter Status**	ANALYZED	Sensitivity - High mag	7.6E+05	1/g
		Sensitivity - PCME (high+low mag)	7.6E+05	1/g

ANALYSIS INFORMATION

**Lab Name**	LabCor Inc
**Lab Sample Number**	150099-S36
**Lab QC Type**	NOT QC
**TEM Prep Method**	Direct
**Est. particulate loading on filter**	NA

Recording Rules:

Initial (high mag)

Min. AR	Min length (um)	Min width (um)
3:1	0	0

PCME Supplemental (low mag)

Min. AR	Min length (um)	Min width (um)
3:1	0	0

Stopping Rules:

Target S (1/g)	Max Area (mm^2)	Max N

RESULTS SUMMARY

Structure Metric	Observed Structure Count (s)	Structure Filter Loading (s/mm^2)	Soil Conc. Structures per gram (s/g)
**High mag only**			
Total Asbestos	235	5.7E+03	1.8E+08
Total Chrysotile (CH)	235	5.7E+03	1.8E+08
Total Amphibole	0	0.0E+00	0.0E+00
actinolite (AC)	0	0.0E+00	0.0E+00
amosite (AM)	0	0.0E+00	0.0E+00
anthophyllite (AN)	0	0.0E+00	0.0E+00
crocidolite (CR)	0	0.0E+00	0.0E+00
tremolite (TR)	0	0.0E+00	0.0E+00
winchite/richterite/tremolite/actinolite (WRTA)	0	0.0E+00	0.0E+00
other amphibole (OA)	0	0.0E+00	0.0E+00
other mineral class (OM)	0	0.0E+00	0.0E+00
**PCME (high+low mag)**			
Total Asbestos	7	1.7E+02	5.3E+06
Total Chrysotile (CH)	7	1.7E+02	5.3E+06
Total Amphibole	0	0.0E+00	0.0E+00
actinolite (AC)	0	0.0E+00	0.0E+00
amosite (AM)	0	0.0E+00	0.0E+00
anthophyllite (AN)	0	0.0E+00	0.0E+00
crocidolite (CR)	0	0.0E+00	0.0E+00
tremolite (TR)	0	0.0E+00	0.0E+00
winchite/richterite/tremolite/actinolite (WRTA)	0	0.0E+00	0.0E+00
other amphibole (OA)	0	0.0E+00	0.0E+00
other mineral class (OM)	0	0.0E+00	0.0E+00

Σ Asbestos Structure Mass (g)		Mass fraction (as %)
**Total TEM**		
WRTA	0.00E+00	0.0000%
OA	0.00E+00	0.0000%
CH	2.49E-08	########
AC	0.00E+00	0.0000%
AM	0.00E+00	0.0000%
AN	0.00E+00	0.0000%
CR	0.00E+00	0.0000%
TR	0.00E+00	0.0000%
**PCME**		
WRTA	0.00E+00	0.0000%
OA	0.00E+00	0.0000%
CH	2.19E-09	16.6680%
AC	0.00E+00	0.0000%
AM	0.00E+00	0.0000%
AN	0.00E+00	0.0000%
CR	0.00E+00	0.0000%
TR	0.00E+00	0.0000%

Asbestos structure mass = Length • $Width^2$ • Density • 1E-12

LA Density = 3.1 g/cm^3
OA Density = 3.0 g/cm^3
CH Density = 2.6 g/cm^3
AC Density = 3.1 g/cm^3
AM Density = 3.5 g/cm^3
AN Density = 3.0 g/cm^3
CR Density = 3.3 g/cm^3
TR Density = 3.0 g/cm^3

Csoil (mass fraction, as %) = Σ Asbestos structure mass • Sensitivity • 100

Equations:

Filter Loading (s/mm^2) = N / (GOx • Ago)
Sensitivity (1/g) = EFA / (GOx • Ago • M • f • Q_R)
Csoil (s/g) = N • Sensitivity

Asbestos Types:

AC - actinolite
AM - amosite
AN - anthophyllite
CH - chrysotile
CR - crocidolite
TR - tremolite
LA - Libby amphibole
OA - other amphibole
NAM - non-asbestos material
OM - other mineral type (specify in "other mineral description" field)

Structure Types:

High mag: Length ≥ 0.5 um, Aspect Ratio ≥ 3:1
PCME: Length > 5 um, Width ≥ 0.25 um, Aspect Ratio ≥ 3:1

Sumas Mtn Asbestos Methods - FBAS_LabCor Inc_14394136_SO_03-06-15_150099_TEM-EPAISO__D.xlsm
FluidizedBedEDD_v1

TEM Asbestos Structure Count for Fluidized Bed Material -- ISO
ANALYTICAL REPORT

FILE NAME:Sumas Mtn Asbestos Methods - FBAS_LabCor Inc_14394136_SO_03-06-15_150099_TEM-EPAISO__D.xlsm

SAMPLE INFORMATION		ANALYSIS PARAMETERS		
**Field Sample Number**	14394136	**EFA (mm^2)**	385	
		**GO area (mm^2) [Ago]**	0.0104	
**Matrix**	Soil	**# GOs counted - high mag**	11	
**Field QC Type**	Not QC	**# GOs counted - low mag only**	0	
**Sample Mass (g dry wt) [M]**	1.00	**F-factor [f]**	1.00E+00	
**Fluidized Bed Flow Ratio[Q_R]**	0.0122			
**Filter Status**	ANALYZED	Sensitivity - High mag	2.8E+05	1/g
		Sensitivity - PCME (high+low mag)	2.8E+05	1/g

ANALYSIS INFORMATION

**Lab Name**	LabCor Inc
**Lab Sample Number**	150099-S37
**Lab QC Type**	NOT QC
**TEM Prep Method**	Direct
**Est. particulate loading on filter**	NA

Recording Rules:

Initial (high mag)

Min. AR	Min length (um)	Min width (um)
3:1	0	0

PCME Supplemental (low mag)

Min. AR	Min length (um)	Min width (um)
3:1	0	0

Stopping Rules:

Target S (1/g)	Max Area (mm^2)	Max N

RESULTS SUMMARY

Structure Metric	Observed Structure Count (s)	Structure Filter Loading (s/mm^2)	Soil Conc. Structures per gram (s/g)
**High mag only**			
Total Asbestos	144	1.3E+03	4.0E+07
Total Chrysotile (CH)	144	1.3E+03	4.0E+07
Total Amphibole	0	0.0E+00	0.0E+00
actinolite (AC)	0	0.0E+00	0.0E+00
amosite (AM)	0	0.0E+00	0.0E+00
anthophyllite (AN)	0	0.0E+00	0.0E+00
crocidolite (CR)	0	0.0E+00	0.0E+00
tremolite (TR)	0	0.0E+00	0.0E+00
winchite/richterite/tremolite/actinolite (WRTA)	0	0.0E+00	0.0E+00
other amphibole (OA)	0	0.0E+00	0.0E+00
other mineral class (OM)	0	0.0E+00	0.0E+00
**PCME (high+low mag)**			
Total Asbestos	5	4.4E+01	1.4E+06
Total Chrysotile (CH)	5	4.4E+01	1.4E+06
Total Amphibole	0	0.0E+00	0.0E+00
actinolite (AC)	0	0.0E+00	0.0E+00
amosite (AM)	0	0.0E+00	0.0E+00
anthophyllite (AN)	0	0.0E+00	0.0E+00
crocidolite (CR)	0	0.0E+00	0.0E+00
tremolite (TR)	0	0.0E+00	0.0E+00
winchite/richterite/tremolite/actinolite (WRTA)	0	0.0E+00	0.0E+00
other amphibole (OA)	0	0.0E+00	0.0E+00
other mineral class (OM)	0	0.0E+00	0.0E+00

Σ Asbestos Structure Mass (g)		Mass fraction (as %)
**Total TEM**		
WRTA	0.00E+00	0.0000%
OA	0.00E+00	0.0000%
CH	2.09E-08	57.8936%
AC	0.00E+00	0.0000%
AM	0.00E+00	0.0000%
AN	0.00E+00	0.0000%
CR	0.00E+00	0.0000%
TR	0.00E+00	0.0000%
**PCME**		
WRTA	0.00E+00	0.0000%
OA	0.00E+00	0.0000%
CH	2.19E-10	0.6087%
AC	0.00E+00	0.0000%
AM	0.00E+00	0.0000%
AN	0.00E+00	0.0000%
CR	0.00E+00	0.0000%
TR	0.00E+00	0.0000%

Asbestos structure mass = Length • Width^2 • Density • 1E-12

LA Density = 3.1 g/cm^3
OA Density = 3.0 g/cm^3
CH Density = 2.6 g/cm^3
AC Density = 3.1 g/cm^3
AM Density = 3.5 g/cm^3
AN Density = 3.0 g/cm^3
CR Density = 3.3 g/cm^3
TR Density = 3.0 g/cm^3

Csoil (mass fraction, as %) = Σ Asbestos structure mass • Sensitivity • 100

Equations:

Filter Loading (s/mm^2) = N / (GOx • Ago)
Sensitivity (1/g) = EFA / (GOx • Ago • M • f • Q_R)
Csoil (s/g) = N • Sensitivity

Asbestos Types:

AC - actinolite
AM - amosite
AN - anthophyllite
CH - chrysotile
CR - crocidolite
TR - tremolite
LA - Libby amphibole
OA - other amphibole
NAM - non-asbestos material
OM - other mineral type (specify in "other mineral description" field)

Structure Types:

High mag: Length ≥ 0.5 um, Aspect Ratio ≥ 3:1
PCME: Length > 5 um, Width ≥ 0.25 um, Aspect Ratio ≥ 3:1

Sumas Mtn Asbestos Methods - FBAS_LabCor Inc_14394137_SO_03-06-15_150099_TEM-EPAISO__D.xlsm
FluidizedBedEDD_v1

TEM Asbestos Structure Count for Fluidized Bed Material -- ISO
ANALYTICAL REPORT

FILE NAME:Sumas Mtn Asbestos Methods - FBAS_LabCor Inc_14394137_SO_03-06-15_150099_TEM-EPAISO__D.xlsm

SAMPLE INFORMATION		ANALYSIS PARAMETERS		
**Field Sample Number**	14394137	**EFA (mm^2)**	385	
		**GO area (mm^2) [Ago]**	0 0104	
**Matrix**	Soil	**# GOs counted - high mag**	5	
**Field QC Type**	Not QC	**# GOs counted - low mag only**	0	
**Sample Mass (g dry wt) [M]**	1.00	**F-factor [f]**	1.00E+00	
**Fluidized Bed Flow Ratio[Q_R]**	0.0122			
**Filter Status**	ANALYZED	Sensitivity - High mag	6.1E+05	1/g
		Sensitivity - PCME (high+low mag)	6.1E+05	1/g

ANALYSIS INFORMATION

**Lab Name**	LabCor Inc
**Lab Sample Number**	150099-S38
**Lab QC Type**	NOT QC
**TEM Prep Method**	Direct
**Est. particulate loading on filter**	NA

Recording Rules:

Initial (high mag)

Min. AR	Min length (um)	Min width (um)
3:1	0	0

PCME Supplemental (low mag)

Min. AR	Min length (um)	Min width (um)
3:1	0	0

Stopping Rules:

Target S (1/g)	Max Area (mm^2)	Max N

RESULTS SUMMARY

Structure Metric	Observed Structure Count (s)	Structure Filter Loading (s/mm^2)	Soil Conc. Structures per gram (s/g)
**High mag only**			
Total Asbestos	148	2.9E+03	9.0E+07
Total Chrysotile (CH)	148	2.9E+03	9.0E+07
Total Amphibole	0	0.0E+00	0.0E+00
actinolite (AC)	0	0.0E+00	0.0E+00
amosite (AM)	0	0.0E+00	0.0E+00
anthophyllite (AN)	0	0.0E+00	0.0E+00
crocidolite (CR)	0	0.0E+00	0.0E+00
tremolite (TR)	0	0.0E+00	0.0E+00
winchite/richterite/tremolite/actinolite (WRTA)	0	0.0E+00	0.0E+00
other amphibole (OA)	0	0.0E+00	0.0E+00
other mineral class (OM)	0	0.0E+00	0.0E+00
**PCME (high+low mag)**			
Total Asbestos	2	3.9E+01	1.2E+06
Total Chrysotile (CH)	2	3.9E+01	1.2E+06
Total Amphibole	0	0.0E+00	0.0E+00
actinolite (AC)	0	0.0E+00	0.0E+00
amosite (AM)	0	0.0E+00	0.0E+00
anthophyllite (AN)	0	0.0E+00	0.0E+00
crocidolite (CR)	0	0.0E+00	0.0E+00
tremolite (TR)	0	0.0E+00	0.0E+00
winchite/richterite/tremolite/actinolite (WRTA)	0	0.0E+00	0.0E+00
other amphibole (OA)	0	0.0E+00	0.0E+00
other mineral class (OM)	0	0.0E+00	0.0E+00

Σ Asbestos Structure Mass (g)		Mass fraction (as %)
**Total TEM**		
WRTA	0.00E+00	0.0000%
OA	0.00E+00	0.0000%
CH	4.70E-09	28.5381%
AC	0.00E+00	0.0000%
AM	0.00E+00	0.0000%
AN	0.00E+00	0.0000%
CR	0.00E+00	0.0000%
TR	0.00E+00	0.0000%
**PCME**		
WRTA	0.00E+00	0.0000%
OA	0.00E+00	0.0000%
CH	1.11E-11	0.0673%
AC	0.00E+00	0.0000%
AM	0.00E+00	0.0000%
AN	0.00E+00	0.0000%
CR	0.00E+00	0.0000%
TR	0.00E+00	0.0000%

Asbestos structure mass = Length • Width^2 • Density • 1E-12

LA Density = 3.1 g/cm^3
OA Density = 3.0 g/cm^3
CH Density = 2.6 g/cm^3
AC Density = 3.1 g/cm^3
AM Density = 3.5 g/cm^3
AN Density = 3.0 g/cm^3
CR Density = 3.3 g/cm^3
TR Density = 3.0 g/cm^3

Csoil (mass fraction, as %) = Σ Asbestos structure mass • Sensitivity • 100

Equations:

Filter Loading (s/mm^2) = N / (GOx • Ago)
Sensitivity (1/g) = EFA / (GOx • Ago • M • f • Q_R)
Csoil (s/g) = N • Sensitivity

Asbestos Types:

AC - actinolite
AM - amosite
AN - anthophyllite
CH - chrysotile
CR - crocidolite
TR - tremolite
LA - Libby amphibole
OA - other amphibole
NAM - non-asbestos material
OM - other mineral type (specify in "other mineral description" field)

Structure Types:

High mag: Length ≥ 0.5 um, Aspect Ratio ≥ 3:1
PCME: Length > 5 um, Width ≥ 0.25 um, Aspect Ratio ≥ 3:1

Sumas Mtn Asbestos Methods - FBAS_LabCor Inc_14394138_SO_03-06-15_150099_TEM-EPAISO__D.xlsm
FluidizedBedEDD_v1

TEM Asbestos Structure Count for Fluidized Bed Material -- ISO
ANALYTICAL REPORT

FILE NAME:Sumas Mtn Asbestos Methods - FBAS_LabCor Inc_14394138_SO_03-06-15_150099_TEM-EPAISO__D.xlsm

SAMPLE INFORMATION		ANALYSIS PARAMETERS		
**Field Sample Number**	14394138	**EFA (mm^2)**	385	
		**GO area (mm^2) [Ago]**	0.0104	
**Matrix**	Soil	**# GOs counted - high mag**	8	
**Field QC Type**	Not QC	**# GOs counted - low mag only**	0	
**Sample Mass (g dry wt) [M]**	1.00	**F-factor [f]**	1.00E+00	
**Fluidized Bed Flow Ratio[Q_R]**	0.0122			
**Filter Status**	ANALYZED	Sensitivity - High mag	3.8E+05	1/g
		Sensitivity - PCME (high+low mag)	3.8E+05	1/g

ANALYSIS INFORMATION

**Lab Name**	LabCor Inc
**Lab Sample Number**	150099-S39
**Lab QC Type**	NOT QC
**TEM Prep Method**	Direct
**Est. particulate loading on filter**	NA

Recording Rules:

Initial (high mag)

Min. AR	Min length (um)	Min width (um)
3:1	0	0

PCME Supplemental (low mag)

Min. AR	Min length (um)	Min width (um)
3:1	0	0

Stopping Rules:

Target S (1/g)	Max Area (mm^2)	Max N

RESULTS SUMMARY

Structure Metric	Observed Structure Count (s)	Structure Filter Loading (s/mm^2)	Soil Conc. Structures per gram (s/g)
**High mag only**			
Total Asbestos	154	1.9E+03	5.9E+07
Total Chrysotile (CH)	154	1.9E+03	5.9E+07
Total Amphibole	0	0.0E+00	0.0E+00
actinolite (AC)	0	0.0E+00	0.0E+00
amosite (AM)	0	0.0E+00	0.0E+00
anthophyllite (AN)	0	0.0E+00	0.0E+00
crocidolite (CR)	0	0.0E+00	0.0E+00
tremolite (TR)	0	0.0E+00	0.0E+00
winchite/richterite/tremolite/actinolite (WRTA)	0	0.0E+00	0.0E+00
other amphibole (OA)	0	0.0E+00	0.0E+00
other mineral class (OM)	0	0.0E+00	0.0E+00
**PCME (high+low mag)**			
Total Asbestos	4	4.8E+01	1.5E+06
Total Chrysotile (CH)	4	4.8E+01	1.5E+06
Total Amphibole	0	0.0E+00	0.0E+00
actinolite (AC)	0	0.0E+00	0.0E+00
amosite (AM)	0	0.0E+00	0.0E+00
anthophyllite (AN)	0	0.0E+00	0.0E+00
crocidolite (CR)	0	0.0E+00	0.0E+00
tremolite (TR)	0	0.0E+00	0.0E+00
winchite/richterite/tremolite/actinolite (WRTA)	0	0.0E+00	0.0E+00
other amphibole (OA)	0	0.0E+00	0.0E+00
other mineral class (OM)	0	0.0E+00	0.0E+00

	Σ Asbestos Structure Mass (g)	Mass fraction (as %)
**Total TEM**		
WRTA	0.00E+00	0.0000%
OA	0.00E+00	0.0000%
CH	3.71E-08	########
AC	0.00E+00	0.0000%
AM	0.00E+00	0.0000%
AN	0.00E+00	0.0000%
CR	0.00E+00	0.0000%
TR	0.00E+00	0.0000%
**PCME**		
WRTA	0.00E+00	0.0000%
OA	0.00E+00	0.0000%
CH	1.45E-10	0.5525%
AC	0.00E+00	0.0000%
AM	0.00E+00	0.0000%
AN	0.00E+00	0.0000%
CR	0.00E+00	0.0000%
TR	0.00E+00	0.0000%

Asbestos structure mass = Length • Width^2 • Density • 1E-12

LA Density = 3.1 g/cm^3
OA Density = 3.0 g/cm^3
CH Density = 2.6 g/cm^3
AC Density = 3.1 g/cm^3
AM Density = 3.5 g/cm^3
AN Density = 3.0 g/cm^3
CR Density = 3.3 g/cm^3
TR Density = 3.0 g/cm^3

Csoil (mass fraction, as %) = Σ Asbestos structure mass • Sensitivity • 100

Equations:

Filter Loading (s/mm^2) = N / (GOx • Ago)
Sensitivity (1/g) = EFA / (GOx • Ago • M • f • Q_R)
Csoil (s/g) = N • Sensitivity

Asbestos Types:

AC - actinolite
AM - amosite
AN - anthophyllite
CH - chrysotile
CR - crocidolite
TR - tremolite
LA - Libby amphibole
OA - other amphibole
NAM - non-asbestos material
OM - other mineral type (specify in "other mineral description" field)

Structure Types:

High mag: Length ≥ 0.5 um, Aspect Ratio ≥ 3:1
PCME: Length > 5 um, Width ≥ 0.25 um, Aspect Ratio ≥ 3:1

Sumas Mtn Asbestos Methods - FBAS_LabCor Inc_14394139_SO_03-07-15_150099_TEM-EPAISO__D.xlsm
FluidizedBedEDD_v1

TEM Asbestos Structure Count for Fluidized Bed Material -- ISO
ANALYTICAL REPORT

FILE NAME:Sumas Mtn Asbestos Methods - FBAS_LabCor Inc_14394139_SO_03-07-15_150099_TEM-EPAISO__D.xlsm

SAMPLE INFORMATION		ANALYSIS PARAMETERS		
**Field Sample Number**	14394139	**EFA (mm^2)**	385	
		**GO area (mm^2) [Ago]**	0.0104	
**Matrix**	Soil	**# GOs counted - high mag**	19	
**Field QC Type**	Not QC	**# GOs counted - low mag only**	0	
**Sample Mass (g dry wt) [M]**	1.00	**F-factor [f]**	1.00E+00	
**Fluidized Bed Flow Ratio[Q_R]**	0 0122			
**Filter Status**	ANALYZED	Sensitivity - High mag	1.6E+05	1/g
		Sensitivity - PCME (high+low mag)	1 6E+05	1/g

ANALYSIS INFORMATION

**Lab Name**	LabCor Inc
**Lab Sample Number**	150099-S40
**Lab QC Type**	NOT QC
**TEM Prep Method**	Direct
**Est. particulate loading on filter**	NA

Recording Rules:

Initial (high mag)

Min. AR	Min length (um)	Min width (um)
3:1	0	0

PCME Supplemental (low mag)

Min. AR	Min length (um)	Min width (um)
3:1	0	0

Stopping Rules:

Target S (1/g)	Max Area (mm^2)	Max N

RESULTS SUMMARY

Structure Metric	Observed Structure Count (s)	Structure Filter Loading (s/mm^2)	Soil Conc Structures per gram (s/g)
**High mag only**			
Total Asbestos	134	6.8E+02	2.1E+07
Total Chrysotile (CH)	134	6 8E+02	2 1E+07
Total Amphibole	0	0.0E+00	0.0E+00
actinolite (AC)	0	0.0E+00	0.0E+00
amosite (AM)	0	0.0E+00	0.0E+00
anthophyllite (AN)	0	0.0E+00	0 0E+00
crocidolite (CR)	0	0.0E+00	0.0E+00
tremolite (TR)	0	0.0E+00	0.0E+00
winchite/richterite/tremolite/actinolite (WRTA)	0	0.0E+00	0.0E+00
other amphibole (OA)	0	0.0E+00	0.0E+00
other mineral class (OM)	0	0.0E+00	0.0E+00
**PCME (high+low mag)**			
Total Asbestos	4	2.0E+01	6.4E+05
Total Chrysotile (CH)	4	2.0E+01	6 4E+05
Total Amphibole	0	0.0E+00	0.0E+00
actinolite (AC)	0	0.0E+00	0 0E+00
amosite (AM)	0	0.0E+00	0.0E+00
anthophyllite (AN)	0	0.0E+00	0.0E+00
crocidolite (CR)	0	0.0E+00	0 0E+00
tremolite (TR)	0	0.0E+00	0.0E+00
winchite/richterite/tremolite/actinolite (WRTA)	0	0.0E+00	0.0E+00
other amphibole (OA)	0	0.0E+00	0.0E+00
other mineral class (OM)	0	0.0E+00	0.0E+00

Σ Asbestos Structure Mass (g)		Mass fraction (as %)
**Total TEM**		
WRTA	0.00E+00	0.0000%
OA	0.00E+00	0.0000%
CH	5.08E-09	8.1226%
AC	0.00E+00	0.0000%
AM	0.00E+00	0.0000%
AN	0.00E+00	0.0000%
CR	0.00E+00	0.0000%
TR	0.00E+00	0.0000%
**[illegible]ME**		
WRTA	0.00E+00	0.0000%
OA	0.00E+00	0.0000%
CH	8.60E-11	0.1375%
AC	0.00E+00	0.0000%
AM	0.00E+00	0.0000%
AN	0 00E+00	0.0000%
CR	0.00E+00	0.0000%
TR	0.00E+00	0.0000%

Asbestos structure mass = Length • Width^2 • Density • 1E-12

LA Density = 3.1 g/cm^3
OA Density = 3.0 g/cm^3
CH Density = 2.6 g/cm^3
AC Density = 3.1 g/cm^3
AM Density = 3.5 g/cm^3
AN Density = 3.0 g/cm^3
CR Density = 3.3 g/cm^3
TR Density = 3.0 g/cm^3

Csoil (mass fraction, as %) = Σ Asbestos structure mass • Sensitivity • 100

Equations:

Filter Loading (s/mm^2) = N / (GOx • Ago)
Sensitivity (1/g) = EFA / (GOx • Ago • M • f • Q_R)
Csoil (s/g) = N • Sensitivity

Asbestos Types:

AC - actinolite
AM - amosite
AN - anthophyllite
CH - chrysotile
CR - crocidolite
TR - tremolite
LA - Libby amphibole
OA - other amphibole
NAM - non-asbestos material
OM - other mineral type (specify in "other mineral description" field)

Structure Types:

High mag: Length ≥ 0.5 um, Aspect Ratio ≥ 3:1
PCME: Length > 5 um, Width ≥ 0 25 um, Aspect Ratio ≥ 3:1

Sumas Mtn Asbestos Methods - FBAS_LabCor Inc_14394140_SO_03-08-15_150099_TEM-EPAISO__D.xlsm
FluidizedBedEDD_v1

TEM Asbestos Structure Count for Fluidized Bed Material -- ISO
ANALYTICAL REPORT

FILE NAME:Sumas Mtn Asbestos Methods - FBAS_LabCor Inc_14394140_SO_03-08-15_150099_TEM-EPAISO__D.xlsm

SAMPLE INFORMATION		ANALYSIS PARAMETERS		
**Field Sample Number**	14394140	**EFA (mm^2)**	385	
		**GO area (mm^2) [Ago]**	0.0104	
**Matrix**	Soil	**# GOs counted - high mag**	61	
**Field QC Type**	Not QC	**# GOs counted - low mag only**	0	
**Sample Mass (g dry wt) [M]**	1.00	**F-factor [f]**	1.00E+00	
**Fluidized Bed Flow Ratio[Q_R]**	0.0122			
**Filter Status**	ANALYZED	Sensitivity - High mag	5.0E+04	1/g
		Sensitivity - PCME (high+low mag)	5.0E+04	1/g

ANALYSIS INFORMATION

**Lab Name**	LabCor Inc
**Lab Sample Number**	150099-S41
**Lab QC Type**	NOT QC
**TEM Prep Method**	Direct
**Est. particulate loading on filter**	NA

Recording Rules:

Initial (high mag)

Min. AR	Min length (um)	Min width (um)
3:1	0	0

PCME Supplemental (low mag)

Min. AR	Min length (um)	Min width (um)
3:1	0	0

Stopping Rules:

Target S (1/g)	Max Area (mm^2)	Max N

RESULTS SUMMARY

Structure Metric	Observed Structure Count (s)	Structure Filter Loading (s/mm^2)	Soil Conc. Structures per gram (s/g)
**High mag only**			
Total Asbestos	163	2.6E+02	8.1E+06
Total Chrysotile (CH)	163	2.6E+02	8.1E+06
Total Amphibole	0	0.0E+00	0.0E+00
actinolite (AC)	0	0.0E+00	0.0E+00
amosite (AM)	0	0.0E+00	0.0E+00
anthophyllite (AN)	0	0.0E+00	0.0E+00
crocidolite (CR)	0	0.0E+00	0.0E+00
tremolite (TR)	0	0.0E+00	0.0E+00
winchite/richterite/tremolite/actinolite (WRTA)	0	0.0E+00	0.0E+00
other amphibole (OA)	0	0.0E+00	0.0E+00
other mineral class (OM)	0	0.0E+00	0.0E+00
**PCME (high+low mag)**			
Total Asbestos	9	1.4E+01	4.5E+05
Total Chrysotile (CH)	9	1.4E+01	4.5E+05
Total Amphibole	0	0.0E+00	0.0E+00
actinolite (AC)	0	0.0E+00	0.0E+00
amosite (AM)	0	0.0E+00	0.0E+00
anthophyllite (AN)	0	0.0E+00	0.0E+00
crocidolite (CR)	0	0.0E+00	0.0E+00
tremolite (TR)	0	0.0E+00	0.0E+00
winchite/richterite/tremolite/actinolite (WRTA)	0	0.0E+00	0.0E+00
other amphibole (OA)	0	0.0E+00	0.0E+00
other mineral class (OM)	0	0.0E+00	0.0E+00

Σ Asbestos Structure Mass (g)		Mass fraction (as %)
**Total TEM**		
WRTA	0.00E+00	0.0000%
OA	0.00E+00	0.0000%
CH	3.05E-08	15.2242%
AC	0.00E+00	0.0000%
AM	0.00E+00	0.0000%
AN	0.00E+00	0.0000%
CR	0.00E+00	0.0000%
TR	0.00E+00	0.0000%
**PCME**		
WRTA	0.00E+00	0.0000%
OA	0.00E+00	0.0000%
CH	1.18E-08	5.8797%
AC	0.00E+00	0.0000%
AM	0.00E+00	0.0000%
AN	0.00E+00	0.0000%
CR	0.00E+00	0.0000%
TR	0.00E+00	0.0000%

Asbestos structure mass = Length • Width^2 • Density • 1E-12

LA Density = 3.1 g/cm^3
OA Density = 3.0 g/cm^3
CH Density = 2.6 g/cm^3
AC Density = 3.1 g/cm^3
AM Density = 3.5 g/cm^3
AN Density = 3.0 g/cm^3
CR Density = 3.3 g/cm^3
TR Density = 3.0 g/cm^3

Csoil (mass fraction, as %) = Σ Asbestos structure mass • Sensitivity • 100

Equations:

Filter Loading (s/mm^2) = N / (GOx • Ago)
Sensitivity (1/g) = EFA / (GOx • Ago • M • f • Q_R)
Csoil (s/g) = N • Sensitivity

Asbestos Types:

AC - actinolite
AM - amosite
AN - anthophyllite
CH - chrysotile
CR - crocidolite
TR - tremolite
LA - Libby amphibole
OA - other amphibole
NAM - non-asbestos material
OM - other mineral type (specify in "other mineral description" field)

Structure Types:

High mag: Length ≥ 0.5 um, Aspect Ratio ≥ 3:1
PCME: Length > 5 um, Width ≥ 0.25 um, Aspect Ratio ≥ 3:1

Sumas Mtn Asbestos Methods - FBAS_LabCor Inc_14394141_SO_03-09-15_150099_TEM-EPAISO__D.xlsm
FluidizedBedEDD_v1

TEM Asbestos Structure Count for Fluidized Bed Material -- ISO
ANALYTICAL REPORT

FILE NAME:Sumas Mtn Asbestos Methods - FBAS_LabCor Inc_14394141_SO_03-09-15_150099_TEM-EPAISO__D.xlsm

SAMPLE INFORMATION		ANALYSIS PARAMETERS		
**Field Sample Number**	14394141	**EFA (mm^2)**	385	
		**GO area (mm^2) [Ago]**	0.0104	
**Matrix**	Soil	**# GOs counted - high mag**	12	
**Field QC Type**	Not QC	**# GOs counted - low mag only**	0	
**Sample Mass (g dry wt) [M]**	1.01	**F-factor [f]**	1.00E+00	
**Fluidized Bed Flow Ratio[Q_R]**	0.0122			
**Filter Status**	ANALYZED	Sensitivity - High mag	2.5E+05	1/g
		Sensitivity - PCME (high+low mag)	2.5E+05	1/g

ANALYSIS INFORMATION

**Lab Name**	LabCor Inc
**Lab Sample Number**	150099-S42
**Lab QC Type**	NOT QC
**TEM Prep Method**	Direct
**Est. particulate loading on filter**	NA

Recording Rules:

Initial (high mag)

Min. AR	Min length (um)	Min width (um)
3:1	0	0

PCME Supplemental (low mag)

Min. AR	Min length (um)	Min width (um)
3:1	0	0

Stopping Rules:

Target S (1/g)	Max Area (mm^2)	Max N

RESULTS SUMMARY

Structure Metric	Observed Structure Count (s)	Structure Filter Loading (s/mm^2)	Soil Conc. Structures per gram (s/g)
**High mag only**			
Total Asbestos	149	1.2E+03	3.8E+07
Total Chrysotile (CH)	149	1.2E+03	3.8E+07
Total Amphibole	0	0.0E+00	0.0E+00
actinolite (AC)	0	0.0E+00	0.0E+00
amosite (AM)	0	0.0E+00	0.0E+00
anthophyllite (AN)	0	0.0E+00	0.0E+00
crocidolite (CR)	0	0.0E+00	0.0E+00
tremolite (TR)	0	0.0E+00	0.0E+00
winchite/richterite/tremolite/actinolite (WRTA)	0	0.0E+00	0.0E+00
other amphibole (OA)	0	0.0E+00	0.0E+00
other mineral class (OM)	0	0.0E+00	0.0E+00
**PCME (high+low mag)**			
Total Asbestos	6	4.8E+01	1.5E+06
Total Chrysotile (CH)	6	4.8E+01	1.5E+06
Total Amphibole	0	0.0E+00	0.0E+00
actinolite (AC)	0	0.0E+00	0.0E+00
amosite (AM)	0	0.0E+00	0.0E+00
anthophyllite (AN)	0	0.0E+00	0.0E+00
crocidolite (CR)	0	0.0E+00	0.0E+00
tremolite (TR)	0	0.0E+00	0.0E+00
winchite/richterite/tremolite/actinolite (WRTA)	0	0.0E+00	0.0E+00
other amphibole (OA)	0	0.0E+00	0.0E+00
other mineral class (OM)	0	0.0E+00	0.0E+00

Σ Asbestos Structure Mass (g)		Mass fraction (as %)
**Total TEM**		
WRTA	0.00E+00	0.0000%
OA	0.00E+00	0.0000%
CH	3.01E-09	7.6192%
AC	0.00E+00	0.0000%
AM	0.00E+00	0.0000%
AN	0.00E+00	0.0000%
CR	0.00E+00	0.0000%
TR	0.00E+00	0.0000%
**PCME**		
WRTA	0.00E+00	0.0000%
OA	0.00E+00	0.0000%
CH	2.87E-10	0.7251%
AC	0.00E+00	0.0000%
AM	0.00E+00	0.0000%
AN	0.00E+00	0.0000%
CR	0.00E+00	0.0000%
TR	0.00E+00	0.0000%

Asbestos structure mass = Length • Width^2 • Density • 1E-12

LA Density = 3.1 g/cm^3
OA Density = 3.0 g/cm^3
CH Density = 2.6 g/cm^3
AC Density = 3.1 g/cm^3
AM Density = 3.5 g/cm^3
AN Density = 3.0 g/cm^3
CR Density = 3.3 g/cm^3
TR Density = 3.0 g/cm^3

Csoil (mass fraction, as %) = Σ Asbestos structure mass • Sensitivity • 100

Equations:

Filter Loading (s/mm^2) = N / (GOx • Ago)
Sensitivity (1/g) = EFA / (GOx • Ago • M • f • Q_R)
Csoil (s/g) = N • Sensitivity

Asbestos Types:

AC - actinolite
AM - amosite
AN - anthophyllite
CH - chrysotile
CR - crocidolite
TR - tremolite
LA - Libby amphibole
OA - other amphibole
NAM - non-asbestos material
OM - other mineral type (specify in "other mineral description" field)

Structure Types:

High mag: Length ≥ 0.5 um, Aspect Ratio ≥ 3:1
PCME: Length > 5 um, Width ≥ 0.25 um, Aspect Ratio ≥ 3:1

Sumas Mtn Asbestos Methods - FBAS_LabCor Inc_14394142_SO_03-10-15_150099_TEM-EPAISO__D.xlsm
FluidizedBedEDD_v1

TEM Asbestos Structure Count for Fluidized Bed Material -- ISO
ANALYTICAL REPORT

FILE NAME:Sumas Mtn Asbestos Methods - FBAS_LabCor Inc_14394142_SO_03-10-15_150099_TEM-EPAISO__D.xlsm

SAMPLE INFORMATION		ANALYSIS PARAMETERS		
**Field Sample Number**	14394142	**EFA (mm^2)**	385	
		**GO area (mm^2) [Ago]**	0 0104	
**Matrix**	Soil	**# GOs counted - high mag**	38	
**Field QC Type**	Not QC	**# GOs counted - low mag only**	0	
**Sample Mass (g dry wt) [M]**	1.00	**F-factor [f]**	1.00E+00	
**Fluidized Bed Flow Ratio[Q_R]**	0.0122			
**Filter Status**	ANALYZED	Sensitivity - High mag	8.0E+04	1/g
		Sensitivity - PCME (high+low mag)	8 0E+04	1/g

ANALYSIS INFORMATION

**Lab Name**	LabCor Inc
**Lab Sample Number**	150099-S43
**Lab QC Type**	NOT QC
**TEM Prep Method**	Direct
**Est. particulate loading on filter**	NA

Recording Rules:

Initial (high mag)

Min. AR	Min length (um)	Min width (um)
3:1	0	0

PCME Supplemental (low mag)

Min. AR	Min length (um)	Min width (um)
3:1	0	0

Stopping Rules:

Target S (1/g)	Max Area (mm^2)	Max N

RESULTS SUMMARY

Structure Metric	Observed Structure Count (s)	Structure Filter Loading (s/mm^2)	Soil Conc. Structures per gram (s/g)
**High mag only**			
Total Asbestos	185	4.7E+02	1.5E+07
Total Chrysotile (CH)	185	4.7E+02	1.5E+07
Total Amphibole	0	0.0E+00	0.0E+00
actinolite (AC)	0	0.0E+00	0 0E+00
amosite (AM)	0	0.0E+00	0.0E+00
anthophyllite (AN)	0	0.0E+00	0.0E+00
crocidolite (CR)	0	0.0E+00	0.0E+00
tremolite (TR)	0	0.0E+00	0.0E+00
winchite/richterite/tremolite/actinolite (WRTA)	0	0.0E+00	0.0E+00
other amphibole (OA)	0	0.0E+00	0.0E+00
other mineral class (OM)	0	0.0E+00	0.0E+00
**PCME (high+low mag)**			
Total Asbestos	11	2.8E+01	8.8E+05
Total Chrysotile (CH)	11	2.8E+01	8.8E+05
Total Amphibole	0	0.0E+00	0.0E+00
actinolite (AC)	0	0.0E+00	0.0E+00
amosite (AM)	0	0.0E+00	0.0E+00
anthophyllite (AN)	0	0.0E+00	0.0E+00
crocidolite (CR)	0	0.0E+00	0.0E+00
tremolite (TR)	0	0.0E+00	0.0E+00
winchite/richterite/tremolite/actinolite (WRTA)	0	0.0E+00	0.0E+00
other amphibole (OA)	0	0.0E+00	0.0E+00
other mineral class (OM)	0	0.0E+00	0.0E+00

Σ Asbestos Structure Mass (g)		Mass fraction (as %)
**Total TEM**		
WRTA	0.00E+00	0.0000%
OA	0.00E+00	0.0000%
CH	1.06E-08	8.4901%
AC	0.00E+00	0.0000%
AM	0.00E+00	0.0000%
AN	0.00E+00	0.0000%
CR	0.00E+00	0.0000%
TR	0.00E+00	0.0000%
**PCME**		
WRTA	0.00E+00	0.0000%
OA	0.00E+00	0.0000%
CH	2.61E-09	2.0910%
AC	0.00E+00	0.0000%
AM	0.00E+00	0.0000%
AN	0.00E+00	0.0000%
CR	0.00E+00	0.0000%
TR	0.00E+00	0.0000%

Asbestos structure mass = Length • Width^2 • Density • 1E-12

LA Density = 3.1 g/cm^3
OA Density = 3.0 g/cm^3
CH Density = 2.6 g/cm^3
AC Density = 3.1 g/cm^3
AM Density = 3.5 g/cm^3
AN Density = 3.0 g/cm^3
CR Density = 3 3 g/cm^3
TR Density = 3.0 g/cm^3

Csoil (mass fraction, as %) = Σ Asbestos structure mass • Sensitivity • 100

Equations:
Filter Loading (s/mm^2) = N / (GOx • Ago)
Sensitivity (1/g) = EFA / (GOx • Ago • M • f • Q_R)
Csoil (s/g) = N • Sensitivity

Asbestos Types:
AC - actinolite
AM - amosite
AN - anthophyllite
CH - chrysotile
CR - crocidolite
TR - tremolite
LA - Libby amphibole
OA - other amphibole
NAM - non-asbestos material
OM - other mineral type (specify in "other mineral description" field)

Structure Types:
High mag: Length ≥ 0.5 um, Aspect Ratio ≥ 3:1
PCME: Length > 5 um, Width ≥ 0.25 um, Aspect Ratio ≥ 3:1

Sumas Mtn Asbestos Methods - FBAS_LabCor Inc_14394143_SO_03-10-15_150099_TEM-EPAISO__D.xlsm
FluidizedBedEDD_v1

TEM Asbestos Structure Count for Fluidized Bed Material -- ISO
ANALYTICAL REPORT

FILE NAME:Sumas Mtn Asbestos Methods - FBAS_LabCor Inc_14394143_SO_03-10-15_150099_TEM-EPAISO__D.xlsm

SAMPLE INFORMATION		ANALYSIS PARAMETERS		
**Field Sample Number**	14394143	**EFA (mm^2)**	385	
		**GO area (mm^2) [Ago]**	0.0104	
**Matrix**	Soil	**# GOs counted - high mag**	16	
**Field QC Type**	Not QC	**# GOs counted - low mag only**	0	
**Sample Mass (g dry wt) [M]**	1.00	**F-factor [f]**	1.00E+00	
**Fluidized Bed Flow Ratio[Q_R]**	0.0122			
**Filter Status**	ANALYZED	Sensitivity - High mag	1.9E+05	1/g
		Sensitivity - PCME (high+low mag)	1.9E+05	1/g

ANALYSIS INFORMATION

**Lab Name**	LabCor Inc
**Lab Sample Number**	150099-S44
**Lab QC Type**	NOT QC
**TEM Prep Method**	Direct
**Est. particulate loading on filter**	NA

Recording Rules:

Initial (high mag)

Min. AR	Min length (um)	Min width (um)
3:1	0	0

PCME Supplemental (low mag)

Min. AR	Min length (um)	Min width (um)
3:1	0	0

Stopping Rules:

Target S (1/g)	Max Area (mm^2)	Max N

RESULTS SUMMARY

Structure Metric	Observed Structure Count (s)	Structure Filter Loading (s/mm^2)	Soil Conc. Structures per gram (s/g)
**High mag only**			
Total Asbestos	160	9.7E+02	3.1E+07
Total Chrysotile (CH)	160	9.7E+02	3.1E+07
Total Amphibole	0	0.0E+00	0.0E+00
actinolite (AC)	0	0.0E+00	0.0E+00
amosite (AM)	0	0.0E+00	0.0E+00
anthophyllite (AN)	0	0.0E+00	0.0E+00
crocidolite (CR)	0	0.0E+00	0.0E+00
tremolite (TR)	0	0.0E+00	0.0E+00
winchite/richterite/tremolite/actinolite (WRTA)	0	0.0E+00	0.0E+00
other amphibole (OA)	0	0.0E+00	0.0E+00
other mineral class (OM)	0	0.0E+00	0.0E+00
**PCME (high+low mag)**			
Total Asbestos	6	3.6E+01	1.1E+06
Total Chrysotile (CH)	6	3.6E+01	1.1E+06
Total Amphibole	0	0.0E+00	0.0E+00
actinolite (AC)	0	0.0E+00	0.0E+00
amosite (AM)	0	0.0E+00	0.0E+00
anthophyllite (AN)	0	0.0E+00	0.0E+00
crocidolite (CR)	0	0.0E+00	0.0E+00
tremolite (TR)	0	0.0E+00	0.0E+00
winchite/richterite/tremolite/actinolite (WRTA)	0	0.0E+00	0.0E+00
other amphibole (OA)	0	0.0E+00	0.0E+00
other mineral class (OM)	0	0.0E+00	0.0E+00

Σ Asbestos Structure Mass (g)		Mass fraction (as %)
**Total TEM**		
WRTA	0.00E+00	0.0000%
OA	0.00E+00	0.0000%
CH	8.80E-09	16.7788%
AC	0.00E+00	0.0000%
AM	0.00E+00	0.0000%
AN	0.00E+00	0.0000%
CR	0.00E+00	0.0000%
TR	0.00E+00	0.0000%
**PCME**		
WRTA	0.00E+00	0.0000%
OA	0.00E+00	0.0000%
CH	2.80E-09	5.3432%
AC	0.00E+00	0.0000%
AM	0.00E+00	0.0000%
AN	0.00E+00	0.0000%
CR	0.00E+00	0.0000%
TR	0.00E+00	0.0000%

Asbestos structure mass = Length • Width^2 • Density • 1E-12

LA Density = 3.1 g/cm^3
OA Density = 3.0 g/cm^3
CH Density = 2.6 g/cm^3
AC Density = 3.1 g/cm^3
AM Density = 3.5 g/cm^3
AN Density = 3.0 g/cm^3
CR Density = 3.3 g/cm^3
TR Density = 3.0 g/cm^3

Csoil (mass fraction, as %) = Σ Asbestos structure mass • Sensitivity • 100

Equations:

Filter Loading (s/mm^2) = N / (GOx • Ago)
Sensitivity (1/g) = EFA / (GOx • Ago • M • f • Q_R)
Csoil (s/g) = N • Sensitivity

Asbestos Types:

AC - actinolite
AM - amosite
AN - anthophyllite
CH - chrysotile
CR - crocidolite
TR - tremolite
LA - Libby amphibole
OA - other amphibole
NAM - non-asbestos material
OM - other mineral type (specify in "other mineral description" field)

Structure Types:

High mag: Length ≥ 0.5 um, Aspect Ratio ≥ 3:1
PCME: Length > 5 um, Width ≥ 0.25 um, Aspect Ratio ≥ 3:1

Sumas Mtn Asbestos Methods - FBAS_LabCor Inc_14394143-DUP_SO_03-11-15_150099_TEM-EPAISO__D.xlsm
FluidizedBedEDD_v1

TEM Asbestos Structure Count for Fluidized Bed Material -- ISO
ANALYTICAL REPORT

FILE NAME:Sumas Mtn Asbestos Methods - FBAS_LabCor Inc_14394143-DUP_SO_03-11-15_150099_TEM-EPAISO__D.xls

SAMPLE INFORMATION		ANALYSIS PARAMETERS		
**Field Sample Number**	14394143-DUP	**EFA (mm^2)**	385	
		**GO area (mm^2) [Ago]**	0.0104	
**Matrix**	Soil	**# GOs counted - high mag**	7	
**Field QC Type**	Not QC	**# GOs counted - low mag only**	0	
**Sample Mass (g dry wt) [M]**	1.00	**F-factor [f]**	1.00E+00	
**Fluidized Bed Flow Ratio[Q_R]**	0.0122			
**Filter Status**	ANALYZED	Sensitivity - High mag	4.4E+05	1/g
		Sensitivity - PCME (high+low mag)	4.4E+05	1/g

ANALYSIS INFORMATION

**Lab Name**	LabCor Inc
**Lab Sample Number**	150099-S45
**Lab QC Type**	NOT QC
**TEM Prep Method**	Direct
**Est. particulate loading on filter**	NA

Recording Rules:

Initial (high mag)

Min. AR	Min length (um)	Min width (um)
3:1	0	0

PCME Supplemental (low mag)

Min. AR	Min length (um)	Min width (um)
3:1	0	0

Stopping Rules:

Target S (1/g)	Max Area (mm^2)	Max N

RESULTS SUMMARY

Structure Metric	Observed Structure Count (s)	Structure Filter Loading (s/mm^2)	Soil Conc. Structures per gram (s/g)
**High mag only**			
Total Asbestos	153	2.1E+03	6.7E+07
Total Chrysotile (CH)	153	2.1E+03	6.7E+07
Total Amphibole	0	0.0E+00	0.0E+00
actinolite (AC)	0	0.0E+00	0.0E+00
amosite (AM)	0	0.0E+00	0.0E+00
anthophyllite (AN)	0	0.0E+00	0.0E+00
crocidolite (CR)	0	0.0E+00	0.0E+00
tremolite (TR)	0	0.0E+00	0.0E+00
winchite/richterite/tremolite/actinolite (WRTA)	0	0.0E+00	0.0E+00
other amphibole (OA)	0	0.0E+00	0.0E+00
other mineral class (OM)	0	0.0E+00	0.0E+00
**PCME (high+low mag)**			
Total Asbestos	7	9.7E+01	3.0E+06
Total Chrysotile (CH)	7	9.7E+01	3.0E+06
Total Amphibole	0	0.0E+00	0.0E+00
actinolite (AC)	0	0.0E+00	0.0E+00
amosite (AM)	0	0.0E+00	0.0E+00
anthophyllite (AN)	0	0.0E+00	0.0E+00
crocidolite (CR)	0	0.0E+00	0.0E+00
tremolite (TR)	0	0.0E+00	0.0E+00
winchite/richterite/tremolite/actinolite (WRTA)	0	0.0E+00	0.0E+00
other amphibole (OA)	0	0.0E+00	0.0E+00
other mineral class (OM)	0	0.0E+00	0.0E+00

Σ Asbestos Structure Mass (g)		Mass fraction (as %)
**Total TEM**		
WRTA	0.00E+00	0.0000%
OA	0.00E+00	0.0000%
CH	4.48E-08	########
AC	0.00E+00	0.0000%
AM	0.00E+00	0.0000%
AN	0.00E+00	0.0000%
CR	0.00E+00	0.0000%
TR	0.00E+00	0.0000%
**PCME**		
WRTA	0.00E+00	0.0000%
OA	0.00E+00	0.0000%
CH	3.84E-10	1.6716%
AC	0.00E+00	0.0000%
AM	0.00E+00	0.0000%
AN	0.00E+00	0.0000%
CR	0.00E+00	0.0000%
TR	0.00E+00	0.0000%

Asbestos structure mass = Length • Width^2 • Density • 1E-12

LA Density = 3.1 g/cm^3
OA Density = 3.0 g/cm^3
CH Density = 2.6 g/cm^3
AC Density = 3.1 g/cm^3
AM Density = 3.5 g/cm^3
AN Density = 3.0 g/cm^3
CR Density = 3.3 g/cm^3
TR Density = 3.0 g/cm^3

Csoil (mass fraction, as %) = Σ Asbestos structure mass • Sensitivity • 100

Equations:

Filter Loading (s/mm^2) = N / (GOx • Ago)
Sensitivity (1/g) = EFA / (GOx • Ago • M • f • Q_R)
Csoil (s/g) = N • Sensitivity

Asbestos Types:

AC - actinolite
AM - amosite
AN - anthophyllite
CH - chrysotile
CR - crocidolite
TR - tremolite
LA - Libby amphibole
OA - other amphibole
NAM - non-asbestos material
OM - other mineral type (specify in "other mineral description" field)

Structure Types:

High mag: Length ≥ 0.5 um, Aspect Ratio ≥ 3:1
PCME: Length > 5 um, Width ≥ 0.25 um, Aspect Ratio ≥ 3:1

Sumas Mtn Asbestos Methods - FBAS_LabCor Inc_14394144_SO_03-11-15_150099_TEM-EPAISO__D.xlsm
FluidizedBedEDD_v1

TEM Asbestos Structure Count for Fluidized Bed Material -- ISO
ANALYTICAL REPORT

FILE NAME:Sumas Mtn Asbestos Methods - FBAS_LabCor Inc_14394144_SO_03-11-15_150099_TEM-EPAISO__D.xlsm

SAMPLE INFORMATION		ANALYSIS PARAMETERS		
**Field Sample Number**	14394144	**EFA (mm^2)**	385	
		**GO area (mm^2) [Ago]**	0.0104	
**Matrix**	Soil	**# GOs counted - high mag**	4	
**Field QC Type**	Not QC	**# GOs counted - low mag only**	0	
**Sample Mass (g dry wt) [M]**	1.00	**F-factor [f]**	1.00E+00	
**Fluidized Bed Flow Ratio[Q_R]**	0 0122			
**Filter Status**	ANALYZED	Sensitivity - High mag	7.6E+05	1/g
		Sensitivity - PCME (high+low mag)	7.6E+05	1/g

ANALYSIS INFORMATION

**Lab Name**	LabCor Inc
**Lab Sample Number**	150099-S46
**Lab QC Type**	NOT QC
**TEM Prep Method**	Direct
**Est. particulate loading on filter**	NA

Recording Rules:

Initial (high mag)

Min. AR	Min length (um)	Min width (um)
3:1	0	0

PCME Supplemental (low mag)

Min. AR	Min length (um)	Min width (um)
3:1	0	0

Stopping Rules:

Target S (1/g)	Max Area (mm^2)	Max N

RESULTS SUMMARY

Structure Metric	Observed Structure Count (s)	Structure Filter Loading (s/mm^2)	Soil Conc. Structures per gram (s/g)
**High mag only**			
Total Asbestos	153	3.7E+03	1.2E+08
Total Chrysotile (CH)	153	3.7E+03	1.2E+08
Total Amphibole	0	0.0E+00	0.0E+00
actinolite (AC)	0	0.0E+00	0.0E+00
amosite (AM)	0	0.0E+00	0.0E+00
anthophyllite (AN)	0	0.0E+00	0.0E+00
crocidolite (CR)	0	0.0E+00	0.0E+00
tremolite (TR)	0	0.0E+00	0.0E+00
winchite/richterite/tremolite/actinolite (WRTA)	0	0.0E+00	0.0E+00
other amphibole (OA)	0	0.0E+00	0.0E+00
other mineral class (OM)	0	0.0E+00	0.0E+00
**PCME (high+low mag)**			
Total Asbestos	7	1.7E+02	5.3E+06
Total Chrysotile (CH)	7	1.7E+02	5.3E+06
Total Amphibole	0	0.0E+00	0.0E+00
actinolite (AC)	0	0.0E+00	0.0E+00
amosite (AM)	0	0.0E+00	0.0E+00
anthophyllite (AN)	0	0.0E+00	0.0E+00
crocidolite (CR)	0	0.0E+00	0.0E+00
tremolite (TR)	0	0.0E+00	0.0E+00
winchite/richterite/tremolite/actinolite (WRTA)	0	0.0E+00	0.0E+00
other amphibole (OA)	0	0.0E+00	0.0E+00
other mineral class (OM)	0	0.0E+00	0.0E+00

Σ Asbestos Structure Mass (g)		Mass fraction (as %)
**Total TEM**		
WRTA	0.00E+00	0.0000%
OA	0.00E+00	0.0000%
CH	3.67E-08	########
AC	0.00E+00	0.0000%
AM	0.00E+00	0.0000%
AN	0.00E+00	0.0000%
CR	0.00E+00	0.0000%
TR	0.00E+00	0.0000%
**PCME**		
WRTA	0.00E+00	0.0000%
OA	0.00E+00	0.0000%
CH	3.06E-09	23.2377%
AC	0.00E+00	0.0000%
AM	0.00E+00	0.0000%
AN	0.00E+00	0.0000%
CR	0.00E+00	0.0000%
TR	0.00E+00	0.0000%

Asbestos structure mass = Length • Width^2 • Density • 1E-12

LA Density = 3.1 g/cm^3
OA Density = 3.0 g/cm^3
CH Density = 2.6 g/cm^3
AC Density = 3.1 g/cm^3
AM Density = 3.5 g/cm^3
AN Density = 3.0 g/cm^3
CR Density = 3 3 g/cm^3
TR Density = 3.0 g/cm^3

Csoil (mass fraction, as %) = Σ Asbestos structure mass • Sensitivity • 100

Equations:

Filter Loading (s/mm^2) = N / (GOx • Ago)
Sensitivity (1/g) = EFA / (GOx • Ago • M • f • Q_R)
Csoil (s/g) = N • Sensitivity

Asbestos Types:

AC - actinolite
AM - amosite
AN - anthophyllite
CH - chrysotile
CR - crocidolite
TR - tremolite
LA - Libby amphibole
OA - other amphibole
NAM - non-asbestos material
OM - other mineral type (specify in "other mineral description" field)

Structure Types:

High mag: Length ≥ 0.5 um, Aspect Ratio ≥ 3:1
PCME: Length > 5 um, Width ≥ 0.25 um, Aspect Ratio ≥ 3:1

Sumas Mtn Asbestos Methods - FBAS_LabCor Inc_14394192_SO_03-12-15_150099_TEM-EPAISO__D.xlsm
FluidizedBedEDD_v1

TEM Asbestos Structure Count for Fluidized Bed Material -- ISO
ANALYTICAL REPORT

FILE NAME:Sumas Mtn Asbestos Methods - FBAS_LabCor Inc_14394192_SO_03-12-15_150099_TEM-EPAISO__D.xlsm

SAMPLE INFORMATION		ANALYSIS PARAMETERS		
**Field Sample Number**	14394192	**EFA (mm^2)**	385	
		**GO area (mm^2) [Ago]**	0.0104	
**Matrix**	Soil	**# GOs counted - high mag**	100	
**Field QC Type**	PB	**# GOs counted - low mag only**	0	
**Sample Mass (g dry wt) [M]**	0.00	**F-factor [f]**	1.00E+00	
**Fluidized Bed Flow Ratio[Q_R]**	0			
**Filter Status**	ANALYZED	Sensitivity - High mag	---	1/g
		Sensitivity - PCME (high+low mag)	---	1/g

ANALYSIS INFORMATION

Lab Name LabCor Inc
Lab Sample Number 150099-S47
Lab QC Type NOT QC
TEM Prep Method Direct
Est. particulate loading on filter NA

Recording Rules:

Initial (high mag)

Min. AR	Min length (um)	Min width (um)
3:1	0	0

PCME Supplemental (low mag)

Min. AR	Min length (um)	Min width (um)
3:1	0	0

Stopping Rules:

Target S (1/g)	Max Area (mm^2)	Max N

RESULTS SUMMARY

Structure Metric	Observed Structure Count (s)	Structure Filter Loading (s/mm^2)	Soil Conc. Structures per gram (s/g)
**High mag only**			
Total Asbestos	0	0.0E+00	---
Total Chrysotile (CH)	0	0.0E+00	---
Total Amphibole	0	0.0E+00	---
actinolite (AC)	0	0.0E+00	---
amosite (AM)	0	0.0E+00	---
anthophyllite (AN)	0	0.0E+00	---
crocidolite (CR)	0	0.0E+00	---
tremolite (TR)	0	0.0E+00	---
winchite/richterite/tremolite/actinolite (WRTA)	0	0.0E+00	---
other amphibole (OA)	0	0.0E+00	---
other mineral class (OM)	0	0.0E+00	---
**PCME (high+low mag)**			
Total Asbestos	0	0.0E+00	---
Total Chrysotile (CH)	0	0.0E+00	---
Total Amphibole	0	0.0E+00	---
actinolite (AC)	0	0.0E+00	---
amosite (AM)	0	0.0E+00	---
anthophyllite (AN)	0	0.0E+00	---
crocidolite (CR)	0	0.0E+00	---
tremolite (TR)	0	0.0E+00	---
winchite/richterite/tremolite/actinolite (WRTA)	0	0.0E+00	---
other amphibole (OA)	0	0.0E+00	---
other mineral class (OM)	0	0.0E+00	---

Σ Asbestos Structure Mass (g)		Mass fraction (as %)
**Total TEM**		
WRTA	0.00E+00	---
OA	0.00E+00	---
CH	0.00E+00	---
AC	0.00E+00	---
AM	0.00E+00	---
AN	0.00E+00	---
CR	0.00E+00	---
TR	0.00E+00	---
**PCME**		
WRTA	0.00E+00	---
OA	0.00E+00	---
CH	0.00E+00	---
AC	0.00E+00	---
AM	0.00E+00	---
AN	0.00E+00	---
CR	0.00E+00	---
TR	0.00E+00	---

Asbestos structure mass = Length • Width^2 • Density • 1E-12

LA Density = 3.1 g/cm^3
OA Density = 3.0 g/cm^3
CH Density = 2.6 g/cm^3
AC Density = 3.1 g/cm^3
AM Density = 3.5 g/cm^3
AN Density = 3.0 g/cm^3
CR Density = 3.3 g/cm^3
TR Density = 3.0 g/cm^3

Csoil (mass fraction, as %) = Σ Asbestos structure mass • Sensitivity • 100

Equations:

Filter Loading (s/mm^2) = N / (GOx • Ago)
Sensitivity (1/g) = EFA / (GOx • Ago • M • f • Q_R)
Csoil (s/g) = N • Sensitivity

Asbestos Types:

AC - actinolite
AM - amosite
AN - anthophyllite
CH - chrysotile
CR - crocidolite
TR - tremolite
LA - Libby amphibole
OA - other amphibole
NAM - non-asbestos material
OM - other mineral type (specify in "other mineral description" field)

Structure Types:

High mag: Length ≥ 0.5 um, Aspect Ratio ≥ 3:1
PCME: Length > 5 um, Width ≥ 0.25 um, Aspect Ratio ≥ 3:1

Report Printed: 7/1/2015, 1:21 PM

Sumas Mtn Asbestos Methods - FBAS_LabCor Inc_14394193_SO_03-12-15_150099_TEM-EPAISO__D.xlsm
FluidizedBedEDD_v1

TEM Asbestos Structure Count for Fluidized Bed Material -- ISO
ANALYTICAL REPORT

FILE NAME:Sumas Mtn Asbestos Methods - FBAS_LabCor Inc_14394193_SO_03-12-15_150099_TEM-EPAISO__D.xlsm

SAMPLE INFORMATION		ANALYSIS PARAMETERS		
**Field Sample Number**	14394193	**EFA (mm^2)**	385	
		**GO area (mm^2) [Ago]**	0.0104	
**Matrix**	Soil	**# GOs counted - high mag**	100	
**Field QC Type**	SB	**# GOs counted - low mag only**	0	
**Sample Mass (g dry wt) [M]**	0.00	**F-factor [f]**	1.00E+00	
**Fluidized Bed Flow Ratio[Q_R]**	0.0122			
**Filter Status**	ANALYZED	Sensitivity - High mag	---	1/g
		Sensitivity - PCME (high+low mag)	---	1/g

ANALYSIS INFORMATION

Lab Name LabCor Inc
Lab Sample Number 150099-S48
Lab QC Type NOT QC
TEM Prep Method Direct
Est. particulate loading on filter NA

Recording Rules:

Initial (high mag)

Min. AR	Min length (um)	Min width (um)
3:1	0	0

PCME Supplemental (low mag)

Min. AR	Min length (um)	Min width (um)
3:1	0	0

Stopping Rules:

Target S (1/g)	Max Area (mm^2)	Max N

RESULTS SUMMARY

Structure Metric	Observed Structure Count (s)	Structure Filter Loading (s/mm^2)	Soil Conc. Structures per gram (s/g)
**High mag only**			
Total Asbestos	0	0.0E+00	---
Total Chrysotile (CH)	0	0.0E+00	---
Total Amphibole	0	0.0E+00	---
actinolite (AC)	0	0.0E+00	---
amosite (AM)	0	0.0E+00	---
anthophyllite (AN)	0	0.0E+00	---
crocidolite (CR)	0	0.0E+00	---
tremolite (TR)	0	0.0E+00	---
winchite/richterite/tremolite/actinolite (WRTA)	0	0.0E+00	---
other amphibole (OA)	0	0.0E+00	---
other mineral class (OM)	0	0.0E+00	---
**PCME (high+low mag)**			
Total Asbestos	0	0.0E+00	---
Total Chrysotile (CH)	0	0.0E+00	---
Total Amphibole	0	0.0E+00	---
actinolite (AC)	0	0.0E+00	---
amosite (AM)	0	0.0E+00	---
anthophyllite (AN)	0	0.0E+00	---
crocidolite (CR)	0	0.0E+00	---
tremolite (TR)	0	0.0E+00	---
winchite/richterite/tremolite/actinolite (WRTA)	0	0.0E+00	---
other amphibole (OA)	0	0.0E+00	---
other mineral class (OM)	0	0.0E+00	---

Σ Asbestos Structure Mass (g)		Mass fraction (as %)
**Total TEM**		
WRTA	0.00E+00	---
OA	0.00E+00	---
CH	0.00E+00	---
AC	0.00E+00	---
AM	0.00E+00	---
AN	0.00E+00	---
CR	0.00E+00	---
TR	0.00E+00	---
**PCME**		
WRTA	0.00E+00	---
OA	0.00E+00	---
CH	0.00E+00	---
AC	0.00E+00	---
AM	0.00E+00	---
AN	0.00E+00	---
CR	0.00E+00	---
TR	0.00E+00	---

Asbestos structure mass = Length • Width^2 • Density • 1E-12

LA Density = 3.1 g/cm^3
OA Density = 3.0 g/cm^3
CH Density = 2.6 g/cm^3
AC Density = 3.1 g/cm^3
AM Density = 3.5 g/cm^3
AN Density = 3.0 g/cm^3
CR Density = 3.3 g/cm^3
TR Density = 3.0 g/cm^3

Csoil (mass fraction, as %) = Σ Asbestos structure mass • Sensitivity • 100

Equations:

Filter Loading (s/mm^2) = N / (GOx • Ago)
Sensitivity (1/g) = EFA / (GOx • Ago • M • f • Q_R)
Csoil (s/g) = N • Sensitivity

Asbestos Types:

AC - actinolite
AM - amosite
AN - anthophyllite
CH - chrysotile
CR - crocidolite
TR - tremolite
LA - Libby amphibole
OA - other amphibole
NAM - non-asbestos material
OM - other mineral type (specify in "other mineral description" field)

Structure Types:

High mag: Length ≥ 0.5 um, Aspect Ratio ≥ 3:1
PCME: Length > 5 um, Width ≥ 0.25 um, Aspect Ratio ≥ 3:1

Sumas Mtn Asbestos Methods - FBAS_LabCor Inc_14394194_SO_03-13-15_150099_TEM-EPAISO__D.xlsm
FluidizedBedEDD_v1

TEM Asbestos Structure Count for Fluidized Bed Material -- ISO
ANALYTICAL REPORT

FILE NAME:Sumas Mtn Asbestos Methods - FBAS_LabCor Inc_14394194_SO_03-13-15_150099_TEM-EPAISO__D.xlsm

SAMPLE INFORMATION		ANALYSIS PARAMETERS		
**Field Sample Number**	14394194	**EFA (mm^2)**	385	
		**GO area (mm^2) [Ago]**	0.0104	
**Matrix**	Soil	**# GOs counted - high mag**	100	
**Field QC Type**	PB	**# GOs counted - low mag only**	0	
**Sample Mass (g dry wt) [M]**	0.00	**F-factor [f]**	1.00E+00	
**Fluidized Bed Flow Ratio[Q_R]**	0			
**Filter Status**	ANALYZED	Sensitivity - High mag	---	1/g
		Sensitivity - PCME (high+low mag)	---	1/g

ANALYSIS INFORMATION

**Lab Name**	LabCor Inc
**Lab Sample Number**	150099-S49
**Lab QC Type**	NOT QC
**TEM Prep Method**	Direct
**Est. particulate loading on filter**	NA

Recording Rules:

Initial (high mag)

Min. AR	Min length (um)	Min width (um)
3:1	0	0

PCME Supplemental (low mag)

Min. AR	Min length (um)	Min width (um)
3:1	0	0

Stopping Rules:

Target S (1/g)	Max Area (mm^2)	Max N

RESULTS SUMMARY

Structure Metric	Observed Structure Count (s)	Structure Filter Loading (s/mm^2)	Soil Conc. Structures per gram (s/g)
**High mag only**			
Total Asbestos	0	0.0E+00	---
Total Chrysotile (CH)	0	0.0E+00	---
Total Amphibole	0	0.0E+00	---
actinolite (AC)	0	0.0E+00	---
amosite (AM)	0	0.0E+00	---
anthophyllite (AN)	0	0.0E+00	---
crocidolite (CR)	0	0.0E+00	---
tremolite (TR)	0	0.0E+00	---
winchite/richterite/tremolite/actinolite (WRTA)	0	0.0E+00	---
other amphibole (OA)	0	0.0E+00	---
other mineral class (OM)	0	0.0E+00	---
**PCME (high+low mag)**			
Total Asbestos	0	0.0E+00	---
Total Chrysotile (CH)	0	0.0E+00	---
Total Amphibole	0	0.0E+00	---
actinolite (AC)	0	0.0E+00	---
amosite (AM)	0	0.0E+00	---
anthophyllite (AN)	0	0.0E+00	---
crocidolite (CR)	0	0.0E+00	---
tremolite (TR)	0	0.0E+00	---
winchite/richterite/tremolite/actinolite (WRTA)	0	0.0E+00	---
other amphibole (OA)	0	0.0E+00	---
other mineral class (OM)	0	0.0E+00	---

Σ Asbestos Structure Mass (g)		Mass fraction (as %)
**Total TEM**		
WRTA	0.00E+00	---
OA	0.00E+00	---
CH	0.00E+00	---
AC	0.00E+00	---
AM	0.00E+00	---
AN	0.00E+00	---
CR	0.00E+00	---
TR	0.00E+00	---
**PCME**		
WRTA	0.00E+00	---
OA	0.00E+00	---
CH	0.00E+00	---
AC	0.00E+00	---
AM	0.00E+00	---
AN	0.00E+00	---
CR	0.00E+00	---
TR	0.00E+00	---

Asbestos structure mass = Length • Width^2 • Density • 1E-12

LA Density = 3.1 g/cm^3
OA Density = 3.0 g/cm^3
CH Density = 2.6 g/cm^3
AC Density = 3.1 g/cm^3
AM Density = 3.5 g/cm^3
AN Density = 3.0 g/cm^3
CR Density = 3.3 g/cm^3
TR Density = 3.0 g/cm^3

Csoil (mass fraction, as %) = Σ Asbestos structure mass • Sensitivity • 100

Equations:

Filter Loading (s/mm^2) = N / (GOx • Ago)
Sensitivity (1/g) = EFA / (GOx • Ago • M • f • Q_R)
Csoil (s/g) = N • Sensitivity

Asbestos Types:

AC - actinolite
AM - amosite
AN - anthophyllite
CH - chrysotile
CR - crocidolite
TR - tremolite
LA - Libby amphibole
OA - other amphibole
NAM - non-asbestos material
OM - other mineral type (specify in "other mineral description" field)

Structure Types:

High mag: Length ≥ 0.5 um, Aspect Ratio ≥ 3:1
PCME: Length > 5 um, Width ≥ 0.25 um, Aspect Ratio ≥ 3:1

Sumas Mtn Asbestos Methods - FBAS_LabCor Inc_14394195_SO_03-13-15_150099_TEM-EPAISO__D.xlsm
FluidizedBedEDD_v1

TEM Asbestos Structure Count for Fluidized Bed Material -- ISO
ANALYTICAL REPORT

FILE NAME:Sumas Mtn Asbestos Methods - FBAS_LabCor Inc_14394195_SO_03-13-15_150099_TEM-EPAISO__D.xlsm

SAMPLE INFORMATION		ANALYSIS PARAMETERS		
**Field Sample Number**	14394195	**EFA (mm^2)**	385	
		**GO area (mm^2) [Ago]**	0.0104	
**Matrix**	Soil	**# GOs counted - high mag**	100	
**Field QC Type**	SB	**# GOs counted - low mag only**	0	
**Sample Mass (g dry wt) [M]**	0.00	**F-factor [f]**	1.00E+00	
**Fluidized Bed Flow Ratio[Q_R]**	0.0122			
**Filter Status**	ANALYZED	Sensitivity - High mag	---	1/g
		Sensitivity - PCME (high+low mag)	---	1/g

ANALYSIS INFORMATION

Lab Name LabCor Inc
Lab Sample Number 150099-S50
Lab QC Type NOT QC
TEM Prep Method Direct
Est. particulate loading on filter NA

Recording Rules:

Initial (high mag)

Min. AR	Min length (um)	Min width (um)
3:1	0	0

PCME Supplemental (low mag)

Min. AR	Min length (um)	Min width (um)
3:1	0	0

Stopping Rules:

Target S (1/g)	Max Area (mm^2)	Max N

RESULTS SUMMARY

Structure Metric	Observed Structure Count (s)	Structure Filter Loading (s/mm^2)	Soil Conc. Structures per gram (s/g)
**High mag only**			
Total Asbestos	0	0.0E+00	---
Total Chrysotile (CH)	0	0.0E+00	---
Total Amphibole	0	0.0E+00	---
actinolite (AC)	0	0.0E+00	---
amosite (AM)	0	0.0E+00	---
anthophyllite (AN)	0	0.0E+00	---
crocidolite (CR)	0	0.0E+00	---
tremolite (TR)	0	0.0E+00	---
winchite/richterite/tremolite/actinolite (WRTA)	0	0.0E+00	---
other amphibole (OA)	0	0.0E+00	---
other mineral class (OM)	0	0.0E+00	---
**PCME (high+low mag)**			
Total Asbestos	0	0.0E+00	---
Total Chrysotile (CH)	0	0.0E+00	---
Total Amphibole	0	0.0E+00	---
actinolite (AC)	0	0.0E+00	---
amosite (AM)	0	0.0E+00	---
anthophyllite (AN)	0	0.0E+00	---
crocidolite (CR)	0	0.0E+00	---
tremolite (TR)	0	0.0E+00	---
winchite/richterite/tremolite/actinolite (WRTA)	0	0.0E+00	---
other amphibole (OA)	0	0.0E+00	---
other mineral class (OM)	0	0.0E+00	---

Σ Asbestos Structure Mass (g)		Mass fraction (as %)
**Total TEM**		
WRTA	0.00E+00	---
OA	0.00E+00	---
CH	0.00E+00	---
AC	0.00E+00	---
AM	0.00E+00	---
AN	0.00E+00	---
CR	0.00E+00	---
TR	0.00E+00	---
**PCME**		
WRTA	0.00E+00	---
OA	0.00E+00	---
CH	0.00E+00	---
AC	0.00E+00	---
AM	0.00E+00	---
AN	0.00E+00	---
CR	0.00E+00	---
TR	0.00E+00	---

Asbestos structure mass = Length • Width^2 • Density • 1E-12

LA Density = 3.1 g/cm^3
OA Density = 3.0 g/cm^3
CH Density = 2.6 g/cm^3
AC Density = 3.1 g/cm^3
AM Density = 3.5 g/cm^3
AN Density = 3.0 g/cm^3
CR Density = 3.3 g/cm^3
TR Density = 3.0 g/cm^3

Csoil (mass fraction, as %) = Σ Asbestos structure mass • Sensitivity • 100

Equations:

Filter Loading (s/mm^2) = N / (GOx • Ago)
Sensitivity (1/g) = EFA / (GOx • Ago • M • f • Q_R)
Csoil (s/g) = N • Sensitivity

Asbestos Types:

AC - actinolite
AM - amosite
AN - anthophyllite
CH - chrysotile
CR - crocidolite
TR - tremolite
LA - Libby amphibole
OA - other amphibole
NAM - non-asbestos material
OM - other mineral type (specify in "other mineral description" field)

Structure Types:

High mag: Length ≥ 0.5 um, Aspect Ratio ≥ 3:1
PCME: Length > 5 um, Width ≥ 0.25 um, Aspect Ratio ≥ 3:1

Sumas Mtn Asbestos Methods - FBAS_LabCor Inc_14394104_SO_03-21-15_150099_TEM-EPAISO__D_Recount Different.xlsm
FluidizedBedEDD_v1

TEM Asbestos Structure Count for Fluidized Bed Material -- ISO
ANALYTICAL REPORT

FILE NAME:Sumas Mtn Asbestos Methods - FBAS_LabCor Inc_14394104_SO_03-21-15_150099_TEM-EPAISO__D_Recour

SAMPLE INFORMATION		ANALYSIS PARAMETERS		
**Field Sample Number**	14394104	**EFA (mm^2)**	385	
		**GO area (mm^2) [Ago]**	0.0104	
**Matrix**	Soil	**# GOs counted - high mag**	2	
**Field QC Type**	Not QC	**# GOs counted - low mag only**	0	
**Sample Mass (g dry wt) [M]**	0.51	**F-factor [f]**	1.00E+00	
**Fluidized Bed Flow Ratio[Q_R]**	0.0122			
**Filter Status**	ANALYZED	Sensitivity - High mag	3.0E+06	1/g
		Sensitivity - PCME (high+low mag)	3.0E+06	1/g

ANALYSIS INFORMATION

**Lab Name**	LabCor Inc
**Lab Sample Number**	150099-S5
**Lab QC Type**	Recount Different
**TEM Prep Method**	Direct
**Est. particulate loading on filter**	NA

Recording Rules:

Initial (high mag)

Min. AR	Min length (um)	Min width (um)
3:1	0	0

PCME Supplemental (low mag)

Min. AR	Min length (um)	Min width (um)
3:1	0	0

Stopping Rules:

Target S (1/g)	Max Area (mm^2)	Max N

RESULTS SUMMARY

Structure Metric	Observed Structure Count (s)	Structure Filter Loading (s/mm^2)	Soil Conc. Structures per gram (s/g)
**High mag only**			
Total Asbestos	5	2.4E+02	1.5E+07
Total Chrysotile (CH)	4	1.9E+02	1.2E+07
Total Amphibole	1	4.8E+01	3.0E+06
actinolite (AC)	0	0.0E+00	0.0E+00
amosite (AM)	0	0.0E+00	0.0E+00
anthophyllite (AN)	0	0.0E+00	0.0E+00
crocidolite (CR)	0	0.0E+00	0.0E+00
tremolite (TR)	1	4.8E+01	3.0E+06
winchite/richterite/tremolite/actinolite (WRTA)	0	0.0E+00	0.0E+00
other amphibole (OA)	0	0.0E+00	0.0E+00
other mineral class (OM)	0	0.0E+00	0.0E+00
**PCME (high+low mag)**			
Total Asbestos	0	0.0E+00	0.0E+00
Total Chrysotile (CH)	0	0.0E+00	0.0E+00
Total Amphibole	0	0.0E+00	0.0E+00
actinolite (AC)	0	0.0E+00	0.0E+00
amosite (AM)	0	0.0E+00	0.0E+00
anthophyllite (AN)	0	0.0E+00	0.0E+00
crocidolite (CR)	0	0.0E+00	0.0E+00
tremolite (TR)	0	0.0E+00	0.0E+00
winchite/richterite/tremolite/actinolite (WRTA)	0	0.0E+00	0.0E+00
other amphibole (OA)	0	0.0E+00	0.0E+00
other mineral class (OM)	0	0.0E+00	0.0E+00

Σ Asbestos Structure Mass (g)		Mass fraction (as %)
**Total TEM**		
WRTA	0.00E+00	0.0000%
OA	0.00E+00	0.0000%
CH	1.05E-09	31.2617%
AC	0.00E+00	0.0000%
AM	0.00E+00	0.0000%
AN	0.00E+00	0.0000%
CR	0.00E+00	0.0000%
TR	5.74E-14	0.0017%
**PCME**		
WRTA	0.00E+00	0.0000%
OA	0.00E+00	0.0000%
CH	0.00E+00	0.0000%
AC	0.00E+00	0.0000%
AM	0.00E+00	0.0000%
AN	0.00E+00	0.0000%
CR	0.00E+00	0.0000%
TR	0.00E+00	0.0000%

Asbestos structure mass = Length • Width^2 • Density • 1E-12

LA Density = 3.1 g/cm^3
OA Density = 3.0 g/cm^3
CH Density = 2.6 g/cm^3
AC Density = 3.1 g/cm^3
AM Density = 3.5 g/cm^3
AN Density = 3.0 g/cm^3
CR Density = 3.3 g/cm^3
TR Density = 3.0 g/cm^3

Csoil (mass fraction, as %) = Σ Asbestos structure mass • Sensitivity • 100

Equations:

Filter Loading (s/mm^2) = N / (GOx • Ago)
Sensitivity (1/g) = EFA / (GOx • Ago • M • f • Q_R)
Csoil (s/g) = N • Sensitivity

Asbestos Types:

AC - actinolite
AM - amosite
AN - anthophyllite
CH - chrysotile
CR - crocidolite
TR - tremolite
LA - Libby amphibole
OA - other amphibole
NAM - non-asbestos material
OM - other mineral type (specify in "other mineral description" field)

Structure Types:

High mag: Length ≥ 0.5 um, Aspect Ratio ≥ 3:1
PCME: Length > 5 um, Width ≥ 0.25 um, Aspect Ratio ≥ 3:1

Report Printed: 7/1/2015, 1:22 PM

Sumas Mtn Asbestos Methods - FBAS_LabCor Inc_14394125_SO_03-20-15_150099_TEM-EPAISO__D_Recount Different.xlsm
FluidizedBedEDD_v1

TEM Asbestos Structure Count for Fluidized Bed Material -- ISO
ANALYTICAL REPORT

FILE NAME:Sumas Mtn Asbestos Methods - FBAS_LabCor Inc_14394125_SO_03-20-15_150099_TEM-EPAISO__D_Recour

SAMPLE INFORMATION		ANALYSIS PARAMETERS		
**Field Sample Number**	14394125	**EFA (mm^2)**	385	
		**GO area (mm^2) [Ago]**	0.0104	
**Matrix**	Soil	**# GOs counted - high mag**	100	
**Field QC Type**	Not QC	**# GOs counted - low mag only**	0	
**Sample Mass (g dry wt) [M]**	1.01	**F-factor [f]**	1.00E+00	
**Fluidized Bed Flow Ratio[Q_R]**	0.0122			
**Filter Status**	ANALYZED	Sensitivity - High mag	3.0E+04	1/g
		Sensitivity - PCME (high+low mag)	3.0E+04	1/g

ANALYSIS INFORMATION

Lab Name LabCor Inc
Lab Sample Number 150099-S26
Lab QC Type Recount Different
TEM Prep Method Direct
Est. particulate loading on filter NA

Recording Rules:

Initial (high mag)

Min. AR	Min length (um)	Min width (um)
3:1	0	0

PCME Supplemental (low mag)

Min. AR	Min length (um)	Min width (um)
3:1	0	0

Stopping Rules:

Target S (1/g)	Max Area (mm^2)	Max N

RESULTS SUMMARY

Structure Metric	Observed Structure Count (s)	Structure Filter Loading (s/mm^2)	Soil Conc Structures per gram (s/g)
**High mag only**			
Total Asbestos	16	1.5E+01	4.8E+05
Total Chrysotile (CH)	16	1.5E+01	4.8E+05
Total Amphibole	0	0.0E+00	0.0E+00
actinolite (AC)	0	0.0E+00	0.0E+00
amosite (AM)	0	0.0E+00	0.0E+00
anthophyllite (AN)	0	0.0E+00	0.0E+00
crocidolite (CR)	0	0.0E+00	0.0E+00
tremolite (TR)	0	0.0E+00	0.0E+00
winchite/richterite/tremolite/actinolite (WRTA)	0	0.0E+00	0.0E+00
other amphibole (OA)	0	0.0E+00	0.0E+00
other mineral class (OM)	0	0.0E+00	0.0E+00
**PCME (high+low mag)**			
Total Asbestos	0	0.0E+00	0.0E+00
Total Chrysotile (CH)	0	0.0E+00	0.0E+00
Total Amphibole	0	0.0E+00	0.0E+00
actinolite (AC)	0	0.0E+00	0.0E+00
amosite (AM)	0	0.0E+00	0.0E+00
anthophyllite (AN)	0	0.0E+00	0.0E+00
crocidolite (CR)	0	0.0E+00	0.0E+00
tremolite (TR)	0	0.0E+00	0.0E+00
winchite/richterite/tremolite/actinolite (WRTA)	0	0.0E+00	0.0E+00
other amphibole (OA)	0	0.0E+00	0.0E+00
other mineral class (OM)	0	0.0E+00	0.0E+00

Σ Asbestos Structure Mass (g)		Mass fraction (as %)
**Total TEM**		
WRTA	0.00E+00	0.0000%
OA	0.00E+00	0.0000%
CH	1.90E-11	0.0058%
AC	0.00E+00	0.0000%
AM	0.00E+00	0.0000%
AN	0.00E+00	0.0000%
CR	0.00E+00	0.0000%
TR	0.00E+00	0.0000%
**PCME**		
WRTA	0.00E+00	0.0000%
OA	0.00E+00	0.0000%
CH	0.00E+00	0.0000%
AC	0.00E+00	0.0000%
AM	0.00E+00	0.0000%
AN	0.00E+00	0.0000%
CR	0.00E+00	0.0000%
TR	0.00E+00	0.0000%

Asbestos structure mass = Length • Width^2 • Density • 1E-12

LA Density = 3.1 g/cm^3
OA Density = 3.0 g/cm^3
CH Density = 2.6 g/cm^3
AC Density = 3.1 g/cm^3
AM Density = 3.5 g/cm^3
AN Density = 3.0 g/cm^3
CR Density = 3.3 g/cm^3
TR Density = 3.0 g/cm^3

Csoil (mass fraction, as %) = Σ Asbestos structure mass • Sensitivity • 100

Equations:

Filter Loading (s/mm^2) = N / (GOx • Ago)
Sensitivity (1/g) = EFA / (GOx • Ago • M • f • Q_R)
Csoil (s/g) = N • Sensitivity

Asbestos Types:

AC - actinolite
AM - amosite
AN - anthophyllite
CH - chrysotile
CR - crocidolite
TR - tremolite
LA - Libby amphibole
OA - other amphibole
NAM - non-asbestos material
OM - other mineral type (specify in "other mineral description" field)

Structure Types:

High mag: Length ≥ 0.5 um, Aspect Ratio ≥ 3:1
PCME: Length > 5 um, Width ≥ 0.25 um, Aspect Ratio ≥ 3:1

Sumas Mtn Asbestos Methods - FBAS_LabCor Inc_14394126_SO_03-19-15_150099_TEM-EPAISO__D_Recount Different xlsm
FluidizedBedEDD_v1

TEM Asbestos Structure Count for Fluidized Bed Material -- ISO
ANALYTICAL REPORT

FILE NAME:Sumas Mtn Asbestos Methods - FBAS_LabCor Inc_14394126_SO_03-19-15_150099_TEM-EPAISO__D_Recour

SAMPLE INFORMATION		ANALYSIS PARAMETERS		
**Field Sample Number**	14394126	**EFA (mm^2)**	385	
		**GO area (mm^2) [Ago]**	0.0104	
**Matrix**	Soil	**# GOs counted - high mag**	100	
**Field QC Type**	Not QC	**# GOs counted - low mag only**	0	
**Sample Mass (g dry wt) [M]**	1.00	**F-factor [f]**	1.00E+00	
**Fluidized Bed Flow Ratio[Q_R]**	0.0122			
**Filter Status**	ANALYZED	Sensitivity - High mag	3.0E+04	1/g
		Sensitivity - PCME (high+low mag)	3.0E+04	1/g

ANALYSIS INFORMATION

**Lab Name**	LabCor Inc
**Lab Sample Number**	150099-S27
**Lab QC Type**	Recount Different
**TEM Prep Method**	Direct
**Est. particulate loading on filter**	NA

Recording Rules:

Initial (high mag)

Min. AR	Min length (um)	Min width (um)
3:1	0	0

PCME Supplemental (low mag)

Min. AR	Min length (um)	Min width (um)
3:1	0	0

Stopping Rules:

Target S (1/g)	Max Area (mm^2)	Max N

RESULTS SUMMARY

Structure Metric	Observed Structure Count (s)	Structure Filter Loading (s/mm^2)	Soil Conc. Structures per gram (s/g)
**High mag only**			
Total Asbestos	0	0.0E+00	0.0E+00
Total Chrysotile (CH)	0	0.0E+00	0.0E+00
Total Amphibole	0	0.0E+00	0.0E+00
actinolite (AC)	0	0.0E+00	0.0E+00
amosite (AM)	0	0.0E+00	0.0E+00
anthophyllite (AN)	0	0.0E+00	0.0E+00
crocidolite (CR)	0	0.0E+00	0.0E+00
tremolite (TR)	0	0.0E+00	0.0E+00
winchite/richterite/tremolite/actinolite (WRTA)	0	0.0E+00	0.0E+00
other amphibole (OA)	0	0.0E+00	0.0E+00
other mineral class (OM)	0	0.0E+00	0.0E+00
**PCME (high+low mag)**			
Total Asbestos	0	0.0E+00	0.0E+00
Total Chrysotile (CH)	0	0.0E+00	0.0E+00
Total Amphibole	0	0.0E+00	0.0E+00
actinolite (AC)	0	0.0E+00	0.0E+00
amosite (AM)	0	0.0E+00	0.0E+00
anthophyllite (AN)	0	0.0E+00	0.0E+00
crocidolite (CR)	0	0.0E+00	0.0E+00
tremolite (TR)	0	0.0E+00	0.0E+00
winchite/richterite/tremolite/actinolite (WRTA)	0	0.0E+00	0.0E+00
other amphibole (OA)	0	0.0E+00	0.0E+00
other mineral class (OM)	0	0.0E+00	0.0E+00

Σ Asbestos Structure Mass (g)		Mass fraction (as %)
**Total TEM**		
WRTA	0.00E+00	0.0000%
OA	0.00E+00	0.0000%
CH	0.00E+00	0.0000%
AC	0.00E+00	0.0000%
AM	0.00E+00	0.0000%
AN	0.00E+00	0.0000%
CR	0.00E+00	0.0000%
TR	0.00E+00	0.0000%
**PCME**		
WRTA	0.00E+00	0.0000%
OA	0.00E+00	0.0000%
CH	0.00E+00	0.0000%
AC	0.00E+00	0.0000%
AM	0.00E+00	0.0000%
AN	0.00E+00	0.0000%
CR	0.00E+00	0.0000%
TR	0.00E+00	0.0000%

Asbestos structure mass = Length • Width^2 • Density • 1E-12

LA Density = 3.1 g/cm^3
OA Density = 3.0 g/cm^3
CH Density = 2.6 g/cm^3
AC Density = 3.1 g/cm^3
AM Density = 3.5 g/cm^3
AN Density = 3.0 g/cm^3
CR Density = 3.3 g/cm^3
TR Density = 3.0 g/cm^3

Csoil (mass fraction, as %) = Σ Asbestos structure mass • Sensitivity • 100

Equations:

Filter Loading (s/mm^2) = N / (GOx • Ago)
Sensitivity (1/g) = EFA / (GOx • Ago • M • f • Q_R)
Csoil (s/g) = N • Sensitivity

Asbestos Types:

AC - actinolite
AM - amosite
AN - anthophyllite
CH - chrysotile
CR - crocidolite
TR - tremolite
LA - Libby amphibole
OA - other amphibole
NAM - non-asbestos material
OM - other mineral type (specify in "other mineral description" field)

Structure Types:

High mag: Length ≥ 0.5 um, Aspect Ratio ≥ 3:1
PCME: Length > 5 um, Width ≥ 0.25 um, Aspect Ratio ≥ 3:1

Sumas Mtn Asbestos Methods - FBAS_LabCor Inc_14394129_SO_03-18-15_150099_TEM-EPAISO__D_Repreparation.xlsm
FluidizedBedEDD_v1

TEM Asbestos Structure Count for Fluidized Bed Material -- ISO
ANALYTICAL REPORT

FILE NAME:Sumas Mtn Asbestos Methods - FBAS_LabCor Inc_14394129_SO_03-18-15_150099_TEM-EPAISO__D_Reprep

SAMPLE INFORMATION		ANALYSIS PARAMETERS		
**Field Sample Number**	14394129	**EFA (mm^2)**	385	
		**GO area (mm^2) [Ago]**	0.0104	
**Matrix**	Soil	**# GOs counted - high mag**	2	
**Field QC Type**	Not QC	**# GOs counted - low mag only**	0	
**Sample Mass (g dry wt) [M]**	1 00	**F-factor [f]**	1 00E+00	
**Fluidized Bed Flow Ratio[Q_R]**	0 0122			
**Filter Status**	ANALYZED	Sensitivity - High mag	1.5E+06	1/g
		Sensitivity - PCME (high+low mag)	1.5E+06	1/g

ANALYSIS INFORMATION

**Lab Name**	LabCor Inc
**Lab Sample Number**	150099-S30
**Lab QC Type**	Repreparation
**TEM Prep Method**	Direct
**Est. particulate loading on filter**	NA

Recording Rules:

Initial (high mag)

Min. AR	Min length (um)	Min width (um)
3:1	0	0

PCME Supplemental (low mag)

Min. AR	Min length (um)	Min width (um)
3:1	0	0

Stopping Rules:

Target S (1/g)	Max Area (mm^2)	Max N

RESULTS SUMMARY

Structure Metric	Observed Structure Count (s)	Structure Filter Loading (s/mm^2)	Soil Conc. Structures per gram (s/g)
**High mag only**			
Total Asbestos	175	8.5E+03	2.7E+08
Total Chrysotile (CH)	175	8.5E+03	2.7E+08
Total Amphibole	0	0.0E+00	0.0E+00
actinolite (AC)	0	0.0E+00	0.0E+00
amosite (AM)	0	0.0E+00	0.0E+00
anthophyllite (AN)	0	0.0E+00	0.0E+00
crocidolite (CR)	0	0.0E+00	0.0E+00
tremolite (TR)	0	0.0E+00	0.0E+00
winchite/richterite/tremolite/actinolite (WRTA)	0	0.0E+00	0.0E+00
other amphibole (OA)	0	0.0E+00	0.0E+00
other mineral class (OM)	0	0.0E+00	0.0E+00
**PCME (high+low mag)**			
Total Asbestos	3	1.4E+02	4.6E+06
Total Chrysotile (CH)	3	1.4E+02	4.6E+06
Total Amphibole	0	0.0E+00	0.0E+00
actinolite (AC)	0	0.0E+00	0.0E+00
amosite (AM)	0	0.0E+00	0.0E+00
anthophyllite (AN)	0	0.0E+00	0.0E+00
crocidolite (CR)	0	0.0E+00	0.0E+00
tremolite (TR)	0	0.0E+00	0.0E+00
winchite/richterite/tremolite/actinolite (WRTA)	0	0.0E+00	0.0E+00
other amphibole (OA)	0	0.0E+00	0.0E+00
other mineral class (OM)	0	0.0E+00	0.0E+00

Σ Asbestos Structure Mass (g)		Mass fraction (as %)
**Total TEM**		
WRTA	0.00E+00	0.0000%
OA	0.00E+00	0.0000%
CH	5.90E-08	########
AC	0.00E+00	0.0000%
AM	0.00E+00	0.0000%
AN	0.00E+00	0.0000%
CR	0.00E+00	0.0000%
TR	0.00E+00	0.0000%
**PCME**		
WRTA	0.00E+00	0.0000%
OA	0.00E+00	0.0000%
CH	6.19E-11	0.9434%
AC	0.00E+00	0.0000%
AM	0.00E+00	0.0000%
AN	0.00E+00	0.0000%
CR	0.00E+00	0.0000%
TR	0.00E+00	0.0000%

Asbestos structure mass = Length • Width^2 • Density • 1E-12

LA Density = 3.1 g/cm^3
OA Density = 3.0 g/cm^3
CH Density = 2.6 g/cm^3
AC Density = 3.1 g/cm^3
AM Density = 3.5 g/cm^3
AN Density = 3.0 g/cm^3
CR Density = 3.3 g/cm^3
TR Density = 3.0 g/cm^3

Csoil (mass fraction, as %) = Σ Asbestos structure mass • Sensitivity • 100

Equations:

Filter Loading (s/mm^2) = N / (GOx • Ago)
Sensitivity (1/g) = EFA / (GOx • Ago • M • f • Q_R)
Csoil (s/g) = N • Sensitivity

Asbestos Types:

AC - actinolite
AM - amosite
AN - anthophyllite
CH - chrysotile
CR - crocidolite
TR - tremolite
LA - Libby amphibole
OA - other amphibole
NAM - non-asbestos material
OM - other mineral type (specify in "other mineral description" field)

Structure Types:

High mag: Length ≥ 0 5 um, Aspect Ratio ≥ 3:1
PCME: Length > 5 um, Width ≥ 0 25 um, Aspect Ratio ≥ 3:1

Sumas Mtn Asbestos Methods - FBAS_LabCor Inc_14394131_SO_03-17-15_150099_TEM-EPAISO__D_Recount Different.xlsm
FluidizedBedEDD_v1

TEM Asbestos Structure Count for Fluidized Bed Material -- ISO
ANALYTICAL REPORT

FILE NAME:Sumas Mtn Asbestos Methods - FBAS_LabCor Inc_14394131_SO_03-17-15_150099_TEM-EPAISO__D_Recour

SAMPLE INFORMATION		ANALYSIS PARAMETERS		
**Field Sample Number**	14394131	**EFA (mm^2)**	385	
		**GO area (mm^2) [Ago]**	0.0104	
**Matrix**	Soil	**# GOs counted - high mag**	2	
**Field QC Type**	Not QC	**# GOs counted - low mag only**	0	
**Sample Mass (g dry wt) [M]**	1.01	**F-factor [f]**	1.00E+00	
**Fluidized Bed Flow Ratio[Q_R]**	0.0122			
**Filter Status**	ANALYZED	Sensitivity - High mag	1.5E+06	1/g
		Sensitivity - PCME (high+low mag)	1.5E+06	1/g

ANALYSIS INFORMATION

**Lab Name**	LabCor Inc
**Lab Sample Number**	150099-S32
**Lab QC Type**	Recount Different
**TEM Prep Method**	Direct
**Est. particulate loading on filter**	NA

Recording Rules:

Initial (high mag)

Min. AR	Min length (um)	Min width (um)
3:1	0	0

PCME Supplemental (low mag)

Min. AR	Min length (um)	Min width (um)
3:1	0	0

Stopping Rules:

Target S (1/g)	Max Area (mm^2)	Max N

RESULTS SUMMARY

Structure Metric	Observed Structure Count (s)	Structure Filter Loading (s/mm^2)	Soil Conc. Structures per gram (s/g)
**High mag only**			
Total Asbestos	161	7.8E+03	2.4E+08
Total Chrysotile (CH)	161	7.8E+03	2.4E+08
Total Amphibole	0	0.0E+00	0.0E+00
actinolite (AC)	0	0.0E+00	0.0E+00
amosite (AM)	0	0.0E+00	0.0E+00
anthophyllite (AN)	0	0.0E+00	0.0E+00
crocidolite (CR)	0	0.0E+00	0.0E+00
tremolite (TR)	0	0.0E+00	0.0E+00
winchite/richterite/tremolite/actinolite (WRTA)	0	0.0E+00	0.0E+00
other amphibole (OA)	0	0.0E+00	0.0E+00
other mineral class (OM)	0	0.0E+00	0.0E+00
**PCME (high+low mag)**			
Total Asbestos	2	9.7E+01	3.0E+06
Total Chrysotile (CH)	2	9.7E+01	3.0E+06
Total Amphibole	0	0.0E+00	0.0E+00
actinolite (AC)	0	0.0E+00	0.0E+00
amosite (AM)	0	0.0E+00	0.0E+00
anthophyllite (AN)	0	0.0E+00	0.0E+00
crocidolite (CR)	0	0.0E+00	0.0E+00
tremolite (TR)	0	0.0E+00	0.0E+00
winchite/richterite/tremolite/actinolite (WRTA)	0	0.0E+00	0.0E+00
other amphibole (OA)	0	0.0E+00	0.0E+00
other mineral class (OM)	0	0.0E+00	0.0E+00

Σ Asbestos Structure Mass (g)		Mass fraction (as %)
**Total TEM**		
WRTA	0.00E+00	0.0000%
OA	0.00E+00	0.0000%
CH	2.36E-07	########
AC	0.00E+00	0.0000%
AM	0.00E+00	0.0000%
AN	0.00E+00	0.0000%
CR	0.00E+00	0.0000%
TR	0.00E+00	0.0000%
**PCME**		
WRTA	0.00E+00	0.0000%
OA	0.00E+00	0.0000%
CH	1.46E-10	2.2041%
AC	0.00E+00	0.0000%
AM	0.00E+00	0.0000%
AN	0.00E+00	0.0000%
CR	0.00E+00	0.0000%
TR	0.00E+00	0.0000%

Asbestos structure mass = Length • Width^2 • Density • 1E-12

LA Density = 3.1 g/cm^3
OA Density = 3.0 g/cm^3
CH Density = 2.6 g/cm^3
AC Density = 3.1 g/cm^3
AM Density = 3.5 g/cm^3
AN Density = 3.0 g/cm^3
CR Density = 3 3 g/cm^3
TR Density = 3.0 g/cm^3

Csoil (mass fraction, as %) = Σ Asbestos structure mass • Sensitivity • 100

Equations:

Filter Loading (s/mm^2) = N / (GOx • Ago)
Sensitivity (1/g) = EFA / (GOx • Ago • M • f • Q_R)
Csoil (s/g) = N • Sensitivity

Asbestos Types:

AC - actinolite
AM - amosite
AN - anthophyllite
CH - chrysotile
CR - crocidolite
TR - tremolite
LA - Libby amphibole
OA - other amphibole
NAM - non-asbestos material
OM - other mineral type (specify in "other mineral description" field)

Structure Types:

High mag: Length ≥ 0.5 um, Aspect Ratio ≥ 3:1
PCME: Length > 5 um, Width ≥ 0.25 um, Aspect Ratio ≥ 3:1

Sumas Mtn Asbestos Methods - FBAS_Labcor Inc_NA_SO_03-30-15_Q150099g_TEM-EPAISO__D_Lab Blank.xlsm
FluidizedBedEDD_v1

TEM Asbestos Structure Count for Fluidized Bed Material -- ISO
ANALYTICAL REPORT

FILE NAME:Sumas Mtn Asbestos Methods - FBAS_Labcor Inc_NA_SO_03-30-15_Q150099g_TEM-EPAISO__D_Lab Blank.x

SAMPLE INFORMATION		ANALYSIS PARAMETERS		
**Field Sample Number**	NA	**EFA (mm^2)**	385	
		**GO area (mm^2) [Ago]**	0.0104	
**Matrix**	Soil	**# GOs counted - high mag**	10	
**Field QC Type**	PB	**# GOs counted - low mag only**	0	
**Sample Mass (g dry wt) [M]**	0.00	**F-factor [f]**	1.00E+00	
**Fluidized Bed Flow Ratio[Q_R]**	0			
**Filter Status**	ANALYZED	Sensitivity - High mag	---	1/g
		Sensitivity - PCME (high+low mag)	---	1/g

ANALYSIS INFORMATION

**Lab Name**	Labcor Inc
**Lab Sample Number**	Q150099g-B1
**Lab QC Type**	Lab Blank
**TEM Prep Method**	Direct
**Est. particulate loading on filter**	NA

Recording Rules:

Initial (high mag)

Min AR	Min length (um)	Min width (um)
3:1	0	0

PCME Supplemental (low mag)

Min. AR	Min length (um)	Min width (um)
3:1	0	0

Stopping Rules:

Target S (1/g)	Max Area (mm^2)	Max N

RESULTS SUMMARY

Structure Metric	Observed Structure Count (s)	Structure Filter Loading (s/mm^2)	Soil Conc. Structures per gram (s/g)
**High mag only**			
Total Asbestos	0	0.0E+00	---
Total Chrysotile (CH)	0	0.0E+00	---
Total Amphibole	0	0.0E+00	---
actinolite (AC)	0	0.0E+00	---
amosite (AM)	0	0.0E+00	---
anthophyllite (AN)	0	0.0E+00	---
crocidolite (CR)	0	0.0E+00	---
tremolite (TR)	0	0.0E+00	---
winchite/richterite/tremolite/actinolite (WRTA)	0	0.0E+00	---
other amphibole (OA)	0	0.0E+00	---
other mineral class (OM)	0	0.0E+00	---
**PCME (high+low mag)**			
Total Asbestos	0	0.0E+00	---
Total Chrysotile (CH)	0	0.0E+00	---
Total Amphibole	0	0.0E+00	---
actinolite (AC)	0	0.0E+00	---
amosite (AM)	0	0.0E+00	---
anthophyllite (AN)	0	0.0E+00	---
crocidolite (CR)	0	0.0E+00	---
tremolite (TR)	0	0.0E+00	---
winchite/richterite/tremolite/actinolite (WRTA)	0	0.0E+00	---
other amphibole (OA)	0	0.0E+00	---
other mineral class (OM)	0	0.0E+00	---

Σ Asbestos Structure Mass (g)		Mass fraction (as %)
**Total TEM**		
WRTA	0.00E+00	---
OA	0.00E+00	---
CH	0.00E+00	---
AC	0.00E+00	---
AM	0.00E+00	---
AN	0.00E+00	---
CR	0.00E+00	---
TR	0.00E+00	---
**PCME**		
WRTA	0.00E+00	---
OA	0.00E+00	---
CH	0.00E+00	---
AC	0.00E+00	---
AM	0.00E+00	---
AN	0.00E+00	---
CR	0.00E+00	---
TR	0.00E+00	---

Asbestos structure mass = Length • Width^2 • Density • 1E-12

LA Density = 3.1 g/cm^3
OA Density = 3.0 g/cm^3
CH Density = 2.6 g/cm^3
AC Density = 3.1 g/cm^3
AM Density = 3.5 g/cm^3
AN Density = 3.0 g/cm^3
CR Density = 3.3 g/cm^3
TR Density = 3.0 g/cm^3

Csoil (mass fraction, as %) = Σ Asbestos structure mass • Sensitivity • 100

Equations:

Filter Loading (s/mm^2) = N / (GOx • Ago)
Sensitivity (1/g) = EFA / (GOx • Ago • M • f • Q_R)
Csoil (s/g) = N • Sensitivity

Asbestos Types:

AC - actinolite
AM - amosite
AN - anthophyllite
CH - chrysotile
CR - crocidolite
TR - tremolite
LA - Libby amphibole
OA - other amphibole
NAM - non-asbestos material
OM - other mineral type (specify in "other mineral description" field)

Structure Types:

High mag: Length ≥ 0.5 um, Aspect Ratio ≥ 3:1
PCME: Length > 5 um, Width ≥ 0.25 um, Aspect Ratio ≥ 3:1

Sumas Mtn Asbestos Methods - FBAS_Labcor Inc_NA_SO_03-30-15_Q150099h_TEM-EPAISO__D_Lab Blank.xlsm
FluidizedBedEDD_v1

TEM Asbestos Structure Count for Fluidized Bed Material -- ISO
ANALYTICAL REPORT

FILE NAME:Sumas Mtn Asbestos Methods - FBAS_Labcor Inc_NA_SO_03-30-15_Q150099h_TEM-EPAISO__D_Lab Blank.x

SAMPLE INFORMATION		ANALYSIS PARAMETERS	
Field Sample Number	NA	EFA (mm^2)	385
		GO area (mm^2) [Ago]	0.0104
Matrix	Soil	# GOs counted - high mag	10
Field QC Type	PB	# GOs counted - low mag only	0
Sample Mass (g dry wt) [M]	0.00	F-factor [f]	1.00E+00
Fluidized Bed Flow Ratio[Q_R]	0		
Filter Status	ANALYZED	Sensitivity - High mag	— 1/g
		Sensitivity - PCME (high+low mag)	— 1/g

ANALYSIS INFORMATION

Lab Name	Labcor Inc
Lab Sample Number	Q150099H-B2
Lab QC Type	Lab Blank
TEM Prep Method	Direct
Est. particulate loading on filter	NA

Recording Rules:

Initial (high mag)

Min. AR	Min length (um)	Min width (um)
3:1	0	0

PCME Supplemental (low mag)

Min. AR	Min length (um)	Min width (um)
3:1	0	0

Stopping Rules:

Target S (1/g)	Max Area (mm^2)	Max N

RESULTS SUMMARY

Structure Metric	Observed Structure Count (s)	Structure Filter Loading (s/mm^2)	Soil Conc. Structures per gram (s/g)
**High mag only**			
Total Asbestos	0	0.0E+00	---
Total Chrysotile (CH)	0	0.0E+00	---
Total Amphibole	0	0.0E+00	---
actinolite (AC)	0	0.0E+00	---
amosite (AM)	0	0.0E+00	---
anthophyllite (AN)	0	0.0E+00	---
crocidolite (CR)	0	0.0E+00	---
tremolite (TR)	0	0.0E+00	---
winchite/richterite/tremolite/actinolite (WRTA)	0	0.0E+00	---
other amphibole (OA)	0	0.0E+00	---
other mineral class (OM)	0	0.0E+00	---
**PCME (high+low mag)**			
Total Asbestos	0	0.0E+00	---
Total Chrysotile (CH)	0	0.0E+00	---
Total Amphibole	0	0.0E+00	---
actinolite (AC)	0	0.0E+00	---
amosite (AM)	0	0.0E+00	---
anthophyllite (AN)	0	0.0E+00	---
crocidolite (CR)	0	0.0E+00	---
tremolite (TR)	0	0.0E+00	---
winchite/richterite/tremolite/actinolite (WRTA)	0	0.0E+00	---
other amphibole (OA)	0	0.0E+00	---
other mineral class (OM)	0	0.0E+00	---

	Σ Asbestos Structure Mass (g)	Mass fraction (as %)
**Total TEM**		
WRTA	0.00E+00	---
OA	0.00E+00	---
CH	0.00E+00	---
AC	0.00E+00	---
AM	0.00E+00	---
AN	0.00E+00	---
CR	0.00E+00	---
TR	0.00E+00	---
**PCME**		
WRTA	0.00E+00	---
OA	0.00E+00	---
CH	0.00E+00	---
AC	0.00E+00	---
AM	0.00E+00	---
AN	0.00E+00	---
CR	0.00E+00	---
TR	0.00E+00	---

Asbestos structure mass = Length • Width^2 • Density • 1E-12

LA Density = 3.1 g/cm^3
OA Density = 3.0 g/cm^3
CH Density = 2.6 g/cm^3
AC Density = 3.1 g/cm^3
AM Density = 3.5 g/cm^3
AN Density = 3 0 g/cm^3
CR Density = 3 3 g/cm^3
TR Density = 3 0 g/cm^3

Csoil (mass fraction, as %) = Σ Asbestos structure mass • Sensitivity • 100

Equations:

Filter Loading (s/mm^2) = N / (GOx • Ago)
Sensitivity (1/g) = EFA / (GOx • Ago • M • f • Q_R)
Csoil (s/g) = N • Sensitivity

Asbestos Types:

AC - actinolite
AM - amosite
AN - anthophyllite
CH - chrysotile
CR - crocidolite
TR - tremolite
LA - Libby amphibole
OA - other amphibole
NAM - non-asbestos material
OM - other mineral type (specify in "other mineral description" field)

Structure Types:

High mag: Length ≥ 0 5 um, Aspect Ratio ≥ 3:1
PCME: Length > 5 um, Width ≥ 0 25 um, Aspect Ratio ≥ 3:1

Report Printed: 7/1/2015, 1:24 PM

Sumas Mtn Asbestos Methods - FBAS_Labcor Inc_NA_SO_03-30-15_Q150099I_TEM-EPAISO__D_Lab Blank.xlsm
FluidizedBedEDD_v1

TEM Asbestos Structure Count for Fluidized Bed Material -- ISO
ANALYTICAL REPORT

FILE NAME:Sumas Mtn Asbestos Methods - FBAS_Labcor Inc_NA_SO_03-30-15_Q150099I_TEM-EPAISO__D_Lab Blank.xl

SAMPLE INFORMATION		ANALYSIS PARAMETERS		
**Field Sample Number**	NA	**EFA (mm^2)**	385	
		**GO area (mm^2) [Ago]**	0.0104	
**Matrix**	Soil	**# GOs counted - high mag**	10	
**Field QC Type**	PB	**# GOs counted - low mag only**	0	
**Sample Mass (g dry wt) [M]**	0.00	**F-factor [f]**	1.00E+00	
**Fluidized Bed Flow Ratio[Q_R]**	0			
**Filter Status**	ANALYZED	Sensitivity - High mag	---	1/g
		Sensitivity - PCME (high+low mag)	---	1/g

ANALYSIS INFORMATION

**Lab Name**	Labcor Inc
**Lab Sample Number**	Q150099I-B3
**Lab QC Type**	Lab Blank
**TEM Prep Method**	Direct
**Est. particulate loading on filter**	NA

Recording Rules:

Initial (high mag)

Min. AR	Min length (um)	Min width (um)
3:1	0	0

PCME Supplemental (low mag)

Min. AR	Min length (um)	Min width (um)
3:1	0	0

Stopping Rules:

Target S (1/g)	Max Area (mm^2)	Max N

RESULTS SUMMARY

Structure Metric	Observed Structure Count (s)	Structure Filter Loading (s/mm^2)	Soil Conc. Structures per gram (s/g)
**High mag only**			
Total Asbestos	0	0.0E+00	---
Total Chrysotile (CH)	0	0.0E+00	---
Total Amphibole	0	0.0E+00	---
actinolite (AC)	0	0.0E+00	---
amosite (AM)	0	0.0E+00	---
anthophyllite (AN)	0	0.0E+00	---
crocidolite (CR)	0	0.0E+00	---
tremolite (TR)	0	0.0E+00	---
winchite/richterite/tremolite/actinolite (WRTA)	0	0.0E+00	---
other amphibole (OA)	0	0.0E+00	---
other mineral class (OM)	0	0.0E+00	---
**PCME (high+low mag)**			
Total Asbestos	0	0.0E+00	---
Total Chrysotile (CH)	0	0.0E+00	---
Total Amphibole	0	0.0E+00	---
actinolite (AC)	0	0.0E+00	---
amosite (AM)	0	0.0E+00	---
anthophyllite (AN)	0	0.0E+00	---
crocidolite (CR)	0	0.0E+00	---
tremolite (TR)	0	0.0E+00	---
winchite/richterite/tremolite/actinolite (WRTA)	0	0.0E+00	---
other amphibole (OA)	0	0.0E+00	---
other mineral class (OM)	0	0.0E+00	---

	Σ Asbestos Structure Mass (g)	Mass fraction (as %)
**Total TEM**		
WRTA	0.00E+00	---
OA	0.00E+00	---
CH	0.00E+00	---
AC	0.00E+00	---
AM	0.00E+00	---
AN	0.00E+00	---
CR	0.00E+00	---
TR	0.00E+00	---
**PCME**		
WRTA	0.00E+00	---
OA	0.00E+00	---
CH	0.00E+00	---
AC	0.00E+00	---
AM	0.00E+00	---
AN	0.00E+00	---
CR	0.00E+00	---
TR	0.00E+00	---

Asbestos structure mass = Length • Width^2 • Density • 1E-12

LA Density = 3.1 g/cm^3
OA Density = 3.0 g/cm^3
CH Density = 2.6 g/cm^3
AC Density = 3.1 g/cm^3
AM Density = 3.5 g/cm^3
AN Density = 3.0 g/cm^3
CR Density = 3.3 g/cm^3
TR Density = 3.0 g/cm^3

Csoil (mass fraction, as %) = Σ Asbestos structure mass • Sensitivity • 100

Equations:

Filter Loading (s/mm^2) = N / (GOx • Ago)
Sensitivity (1/g) = EFA / (GOx • Ago • M • f • Q_R)
Csoil (s/g) = N • Sensitivity

Asbestos Types:

AC - actinolite
AM - amosite
AN - anthophyllite
CH - chrysotile
CR - crocidolite
TR - tremolite
LA - Libby amphibole
OA - other amphibole
NAM - non-asbestos material
OM - other mineral type (specify in "other mineral description" field)

Structure Types:

High mag: Length ≥ 0.5 um, Aspect Ratio ≥ 3:1
PCME: Length > 5 um, Width ≥ 0.25 um, Aspect Ratio ≥ 3:1
