# Supplementary material for: Comparison of soil sampling and analytical methods for asbestos at the Sumas Mountain Asbestos Site—Working towards a toolbox for better assessment
Source: PLoS One. 2017 Jul 31;12(7):e0180210. doi: 10.1371/journal.pone.0180210 (PMC5536314; doi:10.1371/journal.pone.0180210)
Supplement: S11 Text — Quality Assurance Review of the Sumas Mountain Asbestos Site Regional Methods Study for Metals. (PDF) [file pone.0180210.s012.pdf]

UNITED STATES ENVIRONMENTAL PROTECTION AGENCY
REGION 10 LABORATORY
7411 Beach Dr. East
Port Orchard, Washington 98366

QUALITY ASSURANCE MEMORANDUM FOR INORGANIC CHEMICAL ANALYSES

DATE: May 6, 2015

TO: Julie Wroble, Project Manager
Office of Environmental Assessment, Risk Evaluation Unit, US EPA Region 10

FROM: Stephanie Le, Chemist
Office of Environmental Assessment, US EPA Region 10 Laboratory

SUBJECT: Quality Assurance Review of the Sumas Mountain Asbestos Site Regional Methods Study For Metals

Project Code: SFP-078A
Account Code: 2015T10P303DD210EGLA00

CC: Tim Frederick, US EPA Region 4

The following is a quality assurance review of the results of the analysis of 17 Incremental Sampling Methodology (ISM) soil samples and 28 discrete soil samples for metals. These samples were submitted for the Sumas Mountain Asbestos Site Regional Methods Study project. The analysis was performed by ESAT chemists at the US EPA Region 10 Laboratory in Port Orchard, WA, following US EPA and Laboratory guidelines.

This review was conducted for the following samples:

Incremental Sampling Methodology (ISM) samples

14394106	14394107	14394108	14394109	14394110	14394116	14394117
14394118	14394125	14394126	14394127	14394133	14394134	14394135
14394142	14394143	14394144				

Soil samples

14394100	14394101	14394102	14394103	14394104	14394105	14394111
14394112	14394113	14394114	14394115	14394119	14394120	14394121
14394122	14394123	14394124	14394128	14394129	14394130	14394131
14394132	14394136	14394137	14394138	14394139	14394140	14394141

Data Qualifications

Comments below refer to the quality control specifications outlined in the Laboratory's current Quality Assurance Manual, Standard Operating Procedures (SOPs) and the Quality Assurance Project Plan (QAPP). No excursions were required from the method Standard Operating Procedure.

The quality control measures which did not meet Laboratory/QAPP criteria are annotated in the title of each affected subsection with **"Laboratory/QAPP Criteria Not Met."**

For those tests for which the USEPA Region 10 Laboratory has been accredited by The NELAC Institute (TNI), all requirements of the current TNI Standard have been met. The Region 10 Laboratory's Quality System has also been accredited to the Standards of The NELAC Institute (TNI).

1. Sample Transport and Receipt

Upon sample receipt, all conditions met Laboratory/QAPP requirements for this project.

2. Sample Holding Times

The concentration of an analyte in a sample or sample extract may increase or decrease over time depending on the nature of the analyte. For this reason, holding time limits are recommended for samples. The samples covered by this review met method holding time recommendations, where applicable.

3. Sample Preparation

Samples were prepared according to the method outlined in the SOP for these analytes for this type of matrix. No qualification of the data was required based on sample preparation.

4. Initial Calibration and Calibration Verification

The calibration factors generated for the initial calibration met method criteria. The low point of the calibration curve is usually the Minimum Reporting Level (MRL) of the method. All calibration verification checks met the frequency and recovery criteria on the day of analysis. No qualification was required based on calibration or calibration verification.

5. Laboratory Control Samples

All laboratory control sample results met the recovery acceptance criteria for the methods. No qualification was required based on laboratory control sample analysis.

6. Blank Analysis

The procedural blanks did not contain levels of analytes which would require data qualification. No qualification was required based on blank analysis.

7. Duplicate Analysis - Laboratory/QAPP Criteria Not Met

Duplicate analyses were performed on samples 14394120 and 14394137. Sample results which were above the Low Range Standard levels were within the ±20% RPD with the exception of barium (62.5% RPD) for sample 14394137. Associated barium results were qualified (J), estimated. No other qualification was required based on duplicate analysis.

8. Matrix Spike/Matrix Spike Duplicate Analysis – Laboratory/QAPP Criteria Not Met

Matrix spike analyses were performed on samples 14394108, 14394120 and 14394137. Sample results were within the 75-125% recovery requirements, with the following exceptions:

The antimony spike recoveries for all spikes were low (28%/29%, 38%/41%, 16%/20%). All antimony results were qualified (J), estimated.

The calcium spike recoveries for sample 14394108 were high at 137%/ 140%. Calcium results for all (ISM) samples were qualified (J), estimated.

The nickel spike recovery for matrix spike for sample 14394108 was high at 131%. Nickel results for all (ISM) samples were qualified (J), estimated.

No other qualification was required based on matrix spike analyses.

9. Reference Materials - Laboratory/QAPP Criteria Not Met

A reference material was prepared and analyzed with these samples. Analytical values for this sample were within the range of acceptable results with the following exceptions:

The control associated with the ISM samples had high recoveries for of cobalt, sodium and thallium. Cobalt and sodium results were qualified (J), estimated, on this basis. Thallium results were not qualified because all results were non-detects and thus demonstrate no high bias.

The control associated with the discrete samples 14394100-14394121 had high recoveries for beryllium, cadmium, cobalt, copper, nickel, selenium and thallium. All associated results for cobalt, copper, and nickel were qualified (J), estimated. Beryllium, cadmium, selenium, and thallium results were not qualified because all results were non-detects and thus demonstrate no high bias.

No other qualification was necessary based on analysis of the reference material.

10. Instrument Peak Integrations

No manual integrations were performed for these methods.

11. Reporting Limits

Results are reported on a dry weight basis.

All sample results that fall below the MRL are assigned the value of the MRL and the 'U' qualifier is attached. Sample results above the MRL but below the LRS are reported to two significant figures; results above the LRS level are reported to three significant figures.

12. Data Qualifiers

The U qualifier was attached to results below the reporting limit.

The antimony results for all samples were qualified (J), estimated, because of low matrix spikes recoveries.

The barium results for samples 14394122, 14394123, 14394124, 14394128, 14394129, 14394130, 14394131, 14394132, 14394136, 14394137, 14394137DU, 14394138, 14394139, 14394140 and 14394141 were qualified (J), estimated because of poor duplication.

The calcium and nickel results for samples 14394106, 14394107, 14394108, 14394109, 14394110, 14394116, 14394117, 14394118, 14394125, 14394126, 14394127, 14394133, 14394134, 14394135, 14394142, 14394143 and 14394144 were qualified (J), estimated because of high matrix spikes recoveries.

The cobalt and sodium results for samples 14394106, 14394107, 14394108, 14394109, 14394110, 14394116, 14394117, 14394118, 14394125, 14394126, 14394127, 14394133, 14394134, 14394135, 14394142, 14394143 and 14394144 were qualified (J), estimated because a reference material sample was out of acceptance limits.

The cobalt, copper, and nickel results for samples 14394100, 14394101, 14394102, 14394103, 14394104, 14394105, 14394111, 14394112, 14394113, 14394114, 14394115, 14394119, 14394120, 14394120DU and 14394121 were qualified (J), estimated because a reference material sample was out of acceptance limits.

No other data qualification was required for this analysis.

Below are the definitions for the codes used for qualifying data from these analyses. When more than one quality issue

was involved, the most restrictive qualifier has been attached to the data.

U - The analyte was not detected at or above the reported value.

J - The identification of the analyte is acceptable; however the reported value is an estimate.

UJ - The analyte was not detected at or above the reported value. The reported value is an estimate.

The usefulness of qualified data should be treated according to the severity of the qualifier in light of the project's data quality objectives. Should questions arise regarding the data, contact Stephanie Le at the Region 10 Laboratory, phone number (360) 871- 8715.

13. Definitions

Accuracy - the degree of conformity of a measured or calculated quantity to its actual value.

Duplicate Analysis – when a duplicate of a sample (DS), a matrix spike (MSD), or a laboratory control sample (LCSD) is analyzed, it is possible to use the comparison of the results in terms of relative percent difference (RPD) to calculate precision.

Internal standards - Compounds used to help evaluate instrument analytical performance for individual samples. Internal standards provide an instrument response for reference to accurately quantify the analytes for all associated instrumental analyses.

Laboratory Control Sample (LCS) - a clean matrix spiked with known quantities of analytes. The LCS is processed with samples through every step of preparation and analysis. Measuring percent recovery of each analyte in the LCS provides a measurement of accuracy for the analyte in the project samples. A laboratory control sample is prepared and analyzed at a frequency no less than one for every 20 project samples.

Matrix Spike/Matrix Spike Duplicate (MS/MSD) - Sample analyses performed to provide information about the effect of the sample matrix on analyte recovery and measurement within the project samples. To create the MS/MSD, a project sample is spiked with known quantities of analytes and the percent recoveries of the analytes are determined.

Method Blank- An analytical control that is carried through the entire analytical procedure. The method blank is used to define the level of laboratory background and reagent contamination. A method blank is prepared and analyzed for every batch of samples at a minimum frequency of one per every 20 samples. To produce unqualified data, the result of the method blank analysis is required to be less than the MRL and less than 10 times the amount of analyte found in any project sample.

Minimum Reporting Level (MRL) - the smallest measured concentration of a substance that can be reliably measured using a given analytical method.

Low Range Standard (LRS) Level – A level where it has been demonstrated that the instrument achieves defined levels of accuracy and precision, as checked with the Low Range Standard during analysis.

Precision – the degree of mutual agreement or repeatability among a series of individual results.

Reference materials – Samples with analyte values that are homogeneous and well established. This allows the reference material to be used to assess the accuracy of the measurement method.

Relative Percent Difference – The difference between two sample results divided by their mean and expressed as a percentage.
