# Supplementary material for: Comparison of soil sampling and analytical methods for asbestos at the Sumas Mountain Asbestos Site—Working towards a toolbox for better assessment
Source: PLoS One. 2017 Jul 31;12(7):e0180210. doi: 10.1371/journal.pone.0180210 (PMC5536314; doi:10.1371/journal.pone.0180210)
Supplement: S12 Text — Trip report and X-ray diffraction of samples collected April 6, 2006. (PDF) [file pone.0180210.s013.pdf]

UNITED STATES ENVIRONMENTAL PROTECTION AGENCY
REGION 10
1200 Sixth Avenue
Seattle, WA 98101

July 24, 2006

Reply to
Attn Of: OEA-095

MEMORANDUM

TO: Denise Baker, Site Assessment Manager
Office of Environmental Cleanup

FROM: Jed Januch, David Frank, and Lorraine Edmond
Office of Environmental Assessment

SUBJECT: Trip report and X-ray diffraction analysis of samples collected April 6, 2006
Project Code ESD-122A, Swift Creek Asbestos project

Twenty-four samples of rock, water, sediment, and dredge-pile material (EPA numbers 06144000-06144023) were collected April 6, 2006, from four areas along the Swift Creek channel near Nooksack, Whatcom County, Washington (Figure 1). The uppermost sample location was just downstream from the toe of an active deep-seated landslide on the west slope of Sumas Mountain. The landslide occurs in a geologic formation of serpentinite (rock rich in serpentine minerals). The other three sample areas were located in and adjacent to downstream reaches of the channel where it cuts across a composite fan described in the Whatcom County Management Plan for Swift Creek (Whatcom County, 2005) as having formed by flood deposits and debris flows. Sample areas are listed below with corresponding sample numbers:

Toe - just below toe of the Swift Creek landslide	R2 - middle of Reach 2 below wooden bridge	R3-U - along upper part of Reach 3 near Goodwin Road	R3-L - along lower part of Reach 3 near Oat Coles Road
06144000 *	06144007 *	06144010 *	06144020
06144001 *	06144008	06144011	06144021 *
06144002	06144009	06144012	06144022 *
06144003 *		06144013 *	06144023 *
06144004		06144014	
06144005 *		06144015	
06144006 *		06144016	
		06144017	
		06144018 *	
		06144019 *	

The purpose of the sample collection was to identify the mineralogy and morphology of fine-grained materials eroded from the landslide and reported to contain naturally occurring asbestos. The focus of sampling was on fine-grained material. Samples included fines in water, sediment, dredge pile material, soft clay-rich rocks, and rocks with fine-grained coatings and veins. A subset of 13 samples (asterisks) was analyzed by X-ray diffraction (XRD) with results reported here. Previous analyses by polarized light microscopy (PLM) for a subset of 8 of these

Printed on Recycled Paper

samples were reported in an April 27, 2006, memorandum by Jed Januch. Samples are archived and additional material from the group of 24 may be analyzed at a later date.

Sample descriptions are listed in Table 1 and include source material of rock, water with suspended sediment, and bottom sediment from just below the toe of the landslide; and water with suspended sediment, bottom sediment and dredge pile material from downstream reaches. Attachment 1 contains photographs documenting field characteristics

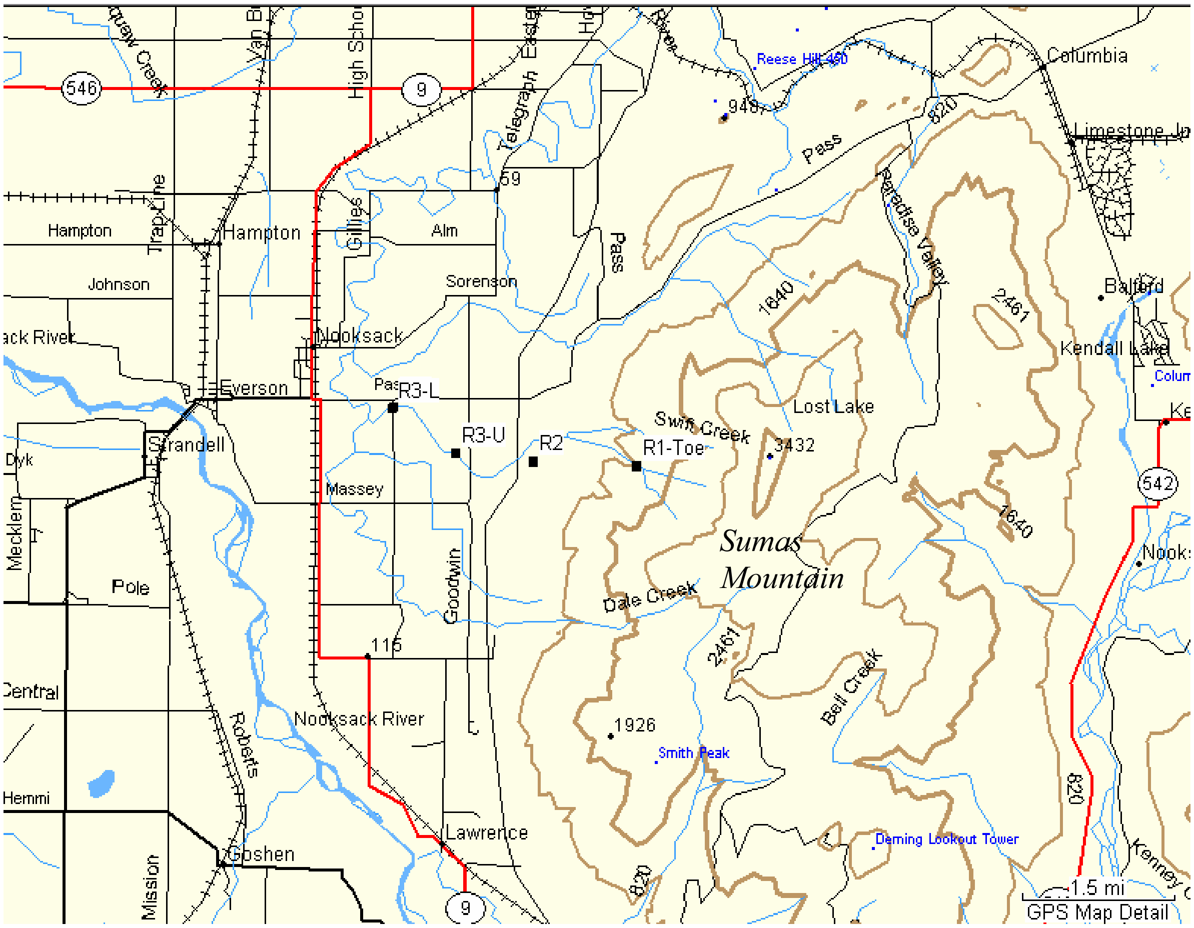

Figure 1. Location of four reaches of Swift Creek from which samples were collected April 6, 2006. Reaches are designated as follows:

R1-Toe - toe of the Swift Creek landslide on west slope of Sumas Mountain.
R2 - Swift Creek channel near forest land at wooden bridge.
R3-U - upper reach of Swift Creek in farmland near Goodwin Road.
R3-L - lower reach of Swift Creek in farmland near Oat Coles Road.

Table 1. Samples collected April 6, 2006. Project ESD-122A, Swift Creek.

SAMPLE	SAMPLE TYPE	DESCRIPTION	XRD [1]	PLM [2]
Toe	Just below toe of Swift Creek landslide on Sumas Mountain.		T40N/R4E-35L[3]	
061440000	bottom sediment, wet	from rivulet discharging from toe	*	
061440001	water, suspended sediment	from stream discharging from toe	*	*
061440002	rocks, hard green	assorted serpentinite w/ veins		
061440003	rock, soft green	breccia boulder, landslide gouge	*	
061440004	rocks, hard white	assorted vein material		
061440005	rocks, soft pale green	assorted vein material	*	*
061440006	rock, hard green	serpentinite with pale green coating	*	*
R2	Just downstream of wooden bridge in the middle of Reach 2.		T40N/R4E-34G	
061440007	bottom sediment, wet	pale greenish grey from channel margin	*	
061440008	rocks, hard green	serpentinite w/ coatings, no visible fibers		
061440009	rocks, hard green	serpentinite w/ veins, felty fibrous		
R3-U	Along the upper part of Reach 3 near Goodwin Road		T40N/R4E-33J-33K	
061440010	bottom sediment, dry	mudcracked channel	*	*
061440011	bottom sediment, damp	light tan surface layer, 2 mm		
061440012	bottom sediment, damp	brown/tan laminated mid-layer, 10 mm		
061440013	bottom sediment, damp	pale green/grey, bottom layer, 6 mm	*	
061440014	bottom sediment, wet	grey to white		
061440015	water, suspended sediment	water, ambient		
061440016	water, suspended sediment	water, turbid plume		
061440017	bottom sediment, dry	mudcracked channel, near fence		
061440018	dredge pile, top	light brown laminated crust	*	*
061440019	dredge pile, interior	brown silt	*	*
R3-L	Along the lower part Reach 3 near Oat Coles Road.		T40N/R4E-33C-33D	
061440020	dredge pile, top	crust, ne of bridge		
061440021	dredge pile, top	large laminated clast, soft silt/clay	*	*
061440022	dredge pile, interior	brown medium sand w/clayey clumps	*	*
061440023	bottom sediment, wet	pale greenish grey fines, sw of bridge	*	

[1] Samples analyzed by XRD for this report.

[2] Samples analyzed by PLM, reported by Jed Januch memorandum April 27, 2006.

[3] Sample areas are designated by township, range, section, and 40-acre tract.

METHODS

X-ray diffraction followed Method XRD-QL for Compound Identification by X-ray Diffraction Analysis (USEPA Manchester Laboratory). The objective of the analysis was to identify minerals present in the samples, with an emphasis on the serpentine group of minerals and XRD verification of the presence of chrysotile. Optical evaluation and XRD measurements were made during April 7 to May 9, 2006, by Jed Januch. XRD pattern interpretation was by David Frank.

With reference to terminology, serpentine refers to a group of several silicate minerals of which the most common are lizardite, antigorite, and chrysotile, all magnesium silicate hydroxides. Each of these minerals occurs in a variety of crystalline structures and morphological habit. Chrysotile, the serpentine mineral of prime environmental concern, commonly has a distinctive fibrous habit that is asbestiform but also occurs in non-asbestiform massive, banded or splintery habits (Wicks and O'Hanley, 1988, p. 102). The XRD method used here provides verification of chrysotile identification based on internal crystalline structure, but does not distinguish the habit or outward form of the mineral. Therefore, in analysis of asbestos-containing material, XRD is complementary to optical studies in that XRD verifies mineral identity whereas optical methods describe mineral habit. In other words, not all chrysotile identified by XRD in this report is necessarily asbestos. Qualitative microscopic observations of XRD specimens are reported here along with the XRD results. Quantitative estimates of asbestos content are presented in the April 27 memorandum by Jed Januch.

Chrysotile, in its various outward forms, occurs in at least four types of internal crystalline structures referred to as clinochrysotile, orthochrysotile, parachrysotile, and polygonal serpentine (Wicks and O'Hanley, 1988). In order to improve the discrimination of chrysotile from other serpentine minerals, particularly lizardite, the XRD patterns were evaluated for clinochrysotile and orthochrysotile, but the presence of other members of the chrysotile subgroup cannot be ruled out. Samples were also evaluated for two lizardite types (single layer lizardite-1T and multilayer lizardite (Wicks, 2000). Relative amounts of chrysotile and lizardite were estimated by a modeling approach that compared diagnostic peak intensities in measured XRD patterns with calculated patterns of clinochrysotile/orthochrysotile mixtures published by Whittaker and Zussman (1956), and with calculated patterns of clinochrysotile/lizardite-1T mixtures based on chrysotile and lizardite reference materials. The modeling procedure and abundance estimates of the various structural types of chrysotile and lizardite are described in the data documentation. The XRD results are consolidated for this memorandum, and chrysotile and lizardite are more simply reported here as subgroups that are undifferentiated for the various types of each mineral.

The subset of samples was examined with a Wild M5 stereomicroscope and a Carl Zeiss Axioskop 40 polarized light microscope (PLM) to aid in preparation of XRD specimen mounts. Sediments were examined in bulk form and then dried, disaggregated and lightly ground with isopropanol in a mortar and pestle. Vein material, coatings and inclusions were hand-picked from rocks and examined as concentrates. Material from two samples (06144001-suspended sediment and 06144006-rock coating) were examined before and after treatment with dilute HCl to remove acid-soluble minerals in order to concentrate the serpentine phases.

A Scintag X1 X-ray diffractometer was used to acquire diffraction data with Co$K\alpha$ radiation at a wavelength of 1.78897 angstroms (Å), generated at 36 mA and 45 kV. Patterns were recorded at scan speeds of 0.5-15 degrees of two-theta ($^{o}2\theta$) units per minute over a 2-76 degree range. Mineral identification was made by comparison with reference samples, the Powder Diffraction File (PDF) maintained by the International Centre for Diffraction Data (ICDD, 2002), and criteria described by Whittaker and Zussman (1956), Bailey (1980), Wicks and O'Hanley (1988) and Wicks (2000).

The abundance of each phase is qualitatively reported as major, minor, or trace amounts based on the intensity of diagnostic diffraction peaks and consideration of X-ray absorption characteristics. Corresponding numerical values are approximately greater than 20% by weight for major, 5-20% for minor, and less than 5% for trace amounts. The detection limit is approximately 1-5%. Some, but not all, phases less than 5% of the bulk sample are included among the minerals present in trace amounts. All major and minor phases have been identified.

Quality control checks for the XRD analysis include the following:

1. At the beginning of the project (April 7, 2006) the diffractometer's goniometer alignment was verified with NIST Standard Reference Material SRM 1976, a flat plate of sintered alumina (corundum) provided by the National Institute of Standards and Technology (NIST).

2. At the beginning of each day of data collection, the alignment of the goniometer and stability of the X-ray intensity was checked by measuring the position and peak height of the 3.34 Å (101) peak of a novaculite (fine-grained quartz) reference plate.

3. The 3.34 Å peak of quartz was present in most of the samples and provided an internal reference for evaluation of sample displacement error.

4. NBS SRM 1866 chrysotile asbestos, and Ward's #96143 Cornwall lizardite and Eden Mills antigorite were used as reference minerals. XRD patterns for the reference minerals contained internal standards of NIST SRM 640c silicon as an alignment check.

RESULTS

Table 2 lists the consolidated results of the XRD analysis. All of the samples examined in the subset of thirteen contain major amounts of serpentine, either chrysotile or lizardite or both, accompanied by a variety of other minerals.

Bottom Sediment

Five samples of fine-grained bottom sediment (Table 1) include light grey mud from a rivulet discharging from the toe of the landslide (sample 06144000); pale greenish grey mud from the margin of Swift Creek near the wooden bridge (06144007), between Goodwin and Oat Coles Roads (06144013)and just below the Oat Coles Road bridge (06144023); and dry, light grey mud-cracked sediment in the channel between Goodwin and Oat Coles Road (06144010). In hand-specimen, the bottom sediments appear to range from sandy silt to silty clay.

XRD results for the bottom sediment yield a similar mineral suite from all four areas (Table 2, Figure 2 - bottom five XRD patterns). All bottom sediment samples contain major amounts of chrysotile and lizardite and lesser amounts of coalingite, brucite, chlorite, quartz and magnetite. In addition, pyroaurite and feldspar were identified in three sediment samples and amphibole in two samples. See Table 2 for the chemical formulae for the minerals.

Table 2. Minerals identified by X-ray diffraction for a subset of samples 06144000-06144023. Project ESD-122A, Swift Creek Asbestos.

MINERAL	IDEAL FORMULA	ABUNDANCE [1]										
		Source Material		Downstream Material								
		Rock	Suspended Sediment	Bottom Sediment					Dredge Piles			
		Just Below Toe dry coatings, veins altered matrix	Just Below Toe wet silty clay	Below Toe wet silty clay	Reach 2 Wood Bridge wet silty clay	Reach 3-U Goodwin dry sandy silt	Reach 3-U Goodwin wet sandy silt	Reach 3-L Oat Coles wet silty clay	Reach 3-U Goodwin surface sandy silt	Reach 3-U Goodwin interior sandy silt	Reach 3-L Oat Coles surface sandy silt	Reach 3-L Oat Coles interior sandy silt
		0614403, 05, 06	06144001	06144000	06144007	06144010	06144013	06144023	06144018	06144019	06144021	06144022
SHEET SILICATES												
Serpentine [2]	$Mg_3Si_2O_5(OH)_4$											
Chrysotile [2]	$Mg_3Si_2O_5(OH)_4$	Major	Major	Major	Major	Major	Major	Major	Major	Major	Major	Major
Lizardite [2]	$(Mg,Al)_3(Si,Fe)_2O_5(OH)_4$	Major	Minor	Major	Major	Major	Major	Major	Major	Major	Major	Major
Chlorite	$(Mg,Fe)_6AlSi_3O_{10}(OH)_8$	Trace	Trace	Trace	Trace	Trace	Trace	Trace	Trace	Trace	Minor	Trace
Mica	$(K,Na)(Mg,Fe,Al)_{2\text{-}3}(Al,Si)_4O_{10}(F,OH)_2$	A	A	A	A	A	A	A	A	A	Trace	Trace
FRAMEWORK SILICATES												
Quartz	SiO_2	Trace	Trace	Trace	Trace	Trace	Trace	Trace	Trace	Trace	Minor	Major
Feldspar	$(K,Na,Ca)Al(Al,Si)_3O_8$	A	A	A	Trace	Trace	Trace	A	Trace	Trace	Minor	Trace
Zeolite												
Heulandite-Ca	$Ca(Si_7Al_2)O_{18}.6H_2O$	A	A	A	A	A	A	A	A	A	Trace	A
CHAIN SILICATES												
Pyroxene	$(Ca,Mg,Fe)_2(Si,Al)_2O_6$	A	A	A	A	A	A	A	A	A	Trace	A
Amphibole	$(Ca,Na)_2(Mg,Fe,Al)_5(Si,Al)_8O_{22}(OH)_2$	A	A	Trace	A	Trace	A	A	A	Trace	Trace	Trace
CARBONATES												
Calcite	$CaCO_3$	Trace	A	A	A	A	A	A	A		A	A
Coalingite [3,4]	$Mg_{10}Fe_3(OH)_{24}CO_3.2H_2O$	Minor	Minor	Trace	Minor	Minor	Minor	Trace	Minor	Trace	A	A
Pyroaurite [3,4]	$Mg_6Fe_2(OH)_{16}CO_3.(H_2O)_4$	Trace	Trace	A	Trace	Trace	Trace	Trace	Trace	Minor	A	Trace
HYDROXIDES												
Brucite [4]	$Mg(OH)_2$	Minor	Trace	Minor	Trace	Trace	Trace	Trace	A	Trace	A	A
OXIDES												
Magnetite [5]	Fe_3O_4	Trace	Trace	Trace	Trace	Trace	Trace	Trace	Trace	Trace	Trace	Trace

MINERAL NOTES

[1] Qualitative abundance designated by major, minor, and trace amounts. Other unidentified phases were present in trace amounts. Phases that were not present or were undetected are designated by A.

[2] Relative abundance of different serpentine minerals estimated by comparison with mixing models. Chrysotile identification includes undifferentiated asbestiform and non-asbestiform habits.

[3] Coalingite is poorly crystalline and may include other poorly crystalline phases; pyroaurite may also include sjogrenite ($Mg_{10}Fe_3(OH)_{24}CO_3.(H_2O)_4)0.25$).

[4] The magnesium hydroxide (brucite) and hydroxy carbonates (coalingite, pyroaurite, sjogrenite) appear to have element substitution.

[5] Magnetite is the predominant oxide phase, but also includes chromite ($FeCr_2O_4$).

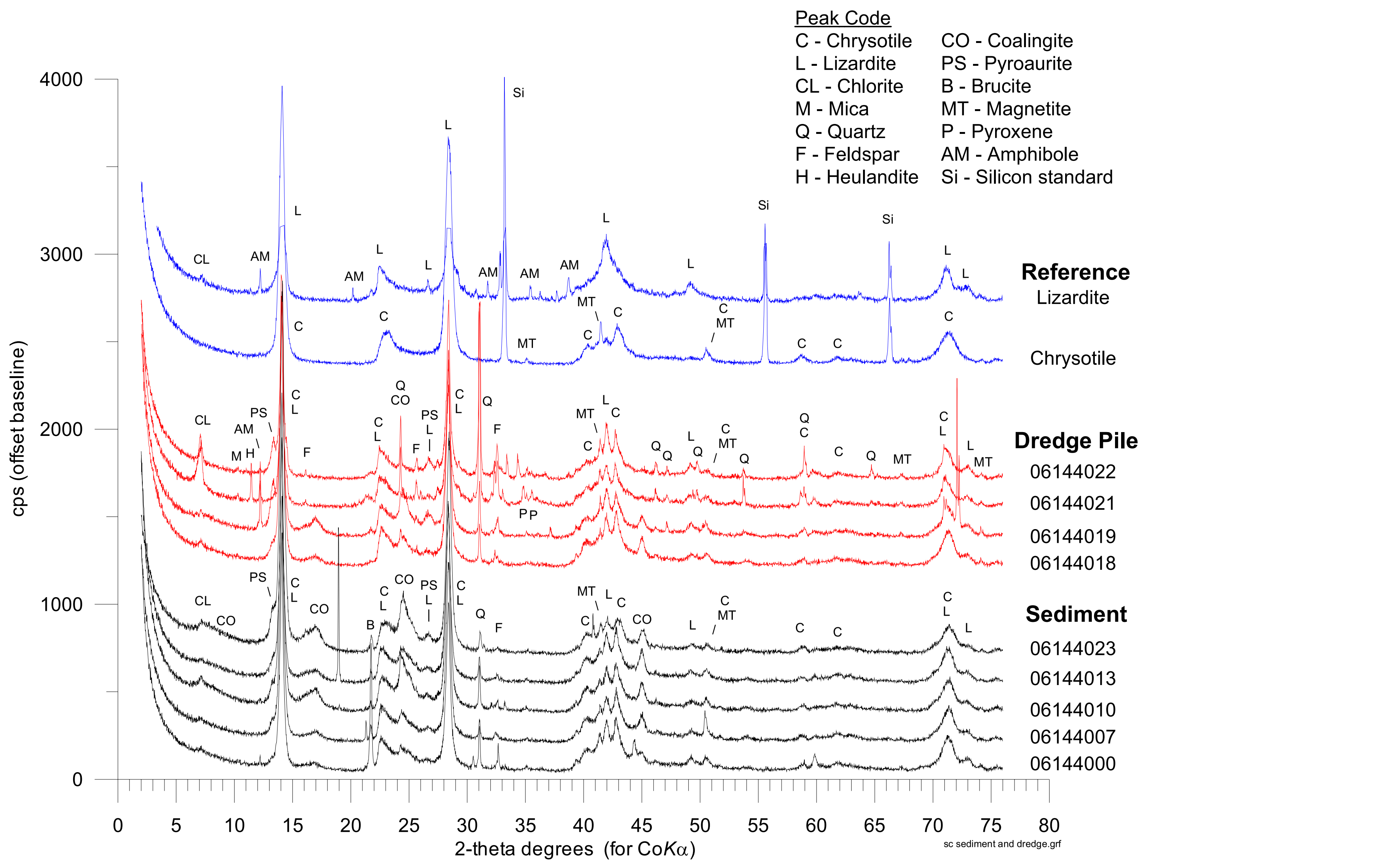

Figure 2. XRD patterns of chrysotile and lizardite reference materials, and bottom sediment and dredge pile material from Swift Creek.

The diffraction characteristics for chrysotile and lizardite indicate that most of the chrysotile in the bottom sediment samples occurs as clinochrysotile with a smaller component of orthochrysotile, and most of the lizardite occurs as lizardite-1T. Other structural types of chrysotile and lizardite may be present. Coalingite (a magnesium iron hydroxy carbonate) has broad diffraction peaks indicating it is a poorly crystallized phase. The hydroxy minerals, coalingite, pyroaurite (another magnesium iron hydroxy carbonate) and brucite (magnesium hydroxide) have peak positions that indicate element substitutions for the metal content in their ideal formulae. The phase identified as pyroaurite may also include sjoegrenite (a third magnesium iron hydroxy carbonate). The phase identified as magnetite (iron oxide) by XRD also includes chromite (iron chromium oxide).

Microscopic observation of XRD specimens indicate the chrysotile occurs in more than one habit. Fibrous (including asbestiform) and splintery forms of chrysotile were observed. Lizardite occurs mainly as very fine-grained non-fibrous aggregates. Chrysotile may also occur in the very fine-grained aggregates, but in this form could not be differentiated from lizardite. Coalingite occurs as brown to reddish brown flakes. Magnetite is medium to very fine-grained and occurs both as free grains and as inclusions in other minerals. Chromite was observed by microscopy but was not differentiated from magnetite by XRD. The habits of pyroaurite and brucite were not determined, although both minerals commonly occur in fibrous as well as platy forms. The habits of the other minerals were not determined.

Dredge Piles

Four samples of dredge-pile material (Table 1) include dry, fine-grained clumps of sandy silts collected as pairs from the surface and interior of the piles at two localities along the south side of Swift Creek between Goodwin and Oat Coles Roads (samples 06144018, 06144019, 06144021, 06144022). Three of these samples (all but 06144019) were from chunks of distinctly laminated material, commonly observed to be strewn about the surface of the dredge piles, indicating they represented chunks of laminated bottom sediments that were picked up from the channel and deposited in the piles by the dredging operations.

XRD results for the dredge-pile samples yield a similar mineral suite to that found in the bottom sediments, but with the occurrence of some additional minerals in small amounts (Table 2, Figure 2 - middle four XRD patterns). XRD patterns for chrysotile and lizardite reference materials are also shown for comparison in Figure 2 (top two patterns). All dredge-pile samples contain major amounts of chrysotile and lizardite and lesser amounts of chlorite, quartz, feldspar and magnetite. In addition, pyroaurite and amphibole were identified in three dredge-pile samples, coalingite and mica in two samples, and brucite, calcite, heulandite (a zeolite), and pyroxene in one sample. See Table 2 for the chemical formulae for the minerals.

The diffraction characteristics for the minerals in the dredge-pile samples are similar to those described for the bottom sediments. Most of the chrysotile occurs as clinochrysotile with a smaller component of orthochrysotile, and most of the lizardite occurs as lizardite-1T. Other structural types of chrysotile and lizardite may be present. Coalingite has broad diffraction peaks indicating it is a poorly crystallized phase. The hydroxy minerals, coalingite, pyroaurite and brucite have peak positions that indicate element substitutions for the metal content in their ideal

formulae. The phase identified as pyroaurite may also include sjoegrenite. The phase identified as magnetite by XRD also includes chromite.

Microscopic observations of XRD specimens of dredge-pile samples are similar to those for bottom sediments. Chrysotile occurs in more than one habit. Fibrous (including asbestiform) and splintery forms were observed. Lizardite occurs mainly as very fine grained non-fibrous aggregates. Chrysotile may also occur in the very fine grained aggregates, but in this form could not be differentiated from lizardite. Coalingite occurs as brown to reddish brown lamellar flakes. Magnetite is medium to very fine-grained and occurs both as free grains and as inclusions in other minerals. Chromite was observed by microscopy but was not differentiated from magnetite by XRD. The habits of pyroaurite, brucite, and heulandite were not determined, although these minerals commonly occur in fibrous as well as platy forms. The habits of the other minerals were not determined.

Source Material and Suspended Sediment

Samples of rock and suspended sediment from the toe of the landslide (Table 1) represent source material subject to erosion and subsequent transport and deposition downstream. These samples included water with pale grey suspended sediment (sample 06144001) collected from one of several streams that discharged from the toe of the landslide into Swift Creek; an altered, breccia boulder of serpentinite (06144003) with a soft green, fragmental clayey matrix and hard, greenish black pebble-sized clasts; light green vein material (06144005) picked out of disaggregated rocks; and a serpentinite cobble (06144006) with a pale green coating of exposed vein material. The altered breccia boulder appeared to be a fragmental chunk of landslide gouge, analogous to fault gouge, derived from cataclastic deformation as would occur during landslide movement.

XRD results for suspended sediment and other source materials yield a similar mineral suite to that found in the downstream bottom sediments and dredge piles, but with concentrated occurrences of particular minerals in the different types of materials (Table 2). Figure 3 shows patterns of some of the source materials. A light fraction of the suspended sediment (Figure 3 - top XRD pattern) contains major chrysotile with lesser amounts of coalingite, pyroaurite, brucite, and chlorite. Vein material (Figure 3 - middle two patterns) also contains major chrysotile with lesser lizardite and pyroaurite. Chrysotile-rich vein material in the coating on sample 06144006 is well mixed with major brucite and coalingite. In contrast, the clayey boulder breccia (Figure 3 - bottom pattern) contains primarily lizardite in both the clayey matrix and the greenish black pebble clasts, and very little chrysotile.

The diffraction characteristics and microscopic observations for the minerals in the source materials are generally similar to those described for the bottom sediments and dredge-pile materials. Two samples of source material (06144001, 06144006) were treated with hydrochloric acid to concentrate the serpentine phases to aid identification of types in the chrysotile and lizardite mineral subgroups. Most of the chrysotile in the limited set of source materials examined occurs as clinochrysotile, and most of the lizardite as lizardite-1T. However, one sample of vein material (06144005) was particularly abundant in other members of these mineral subgroups, including orthochrysotile and multilayer lizardite (Figure 3). The acid treatment, in addition, indicated all of the hydroxy minerals are acid soluble.

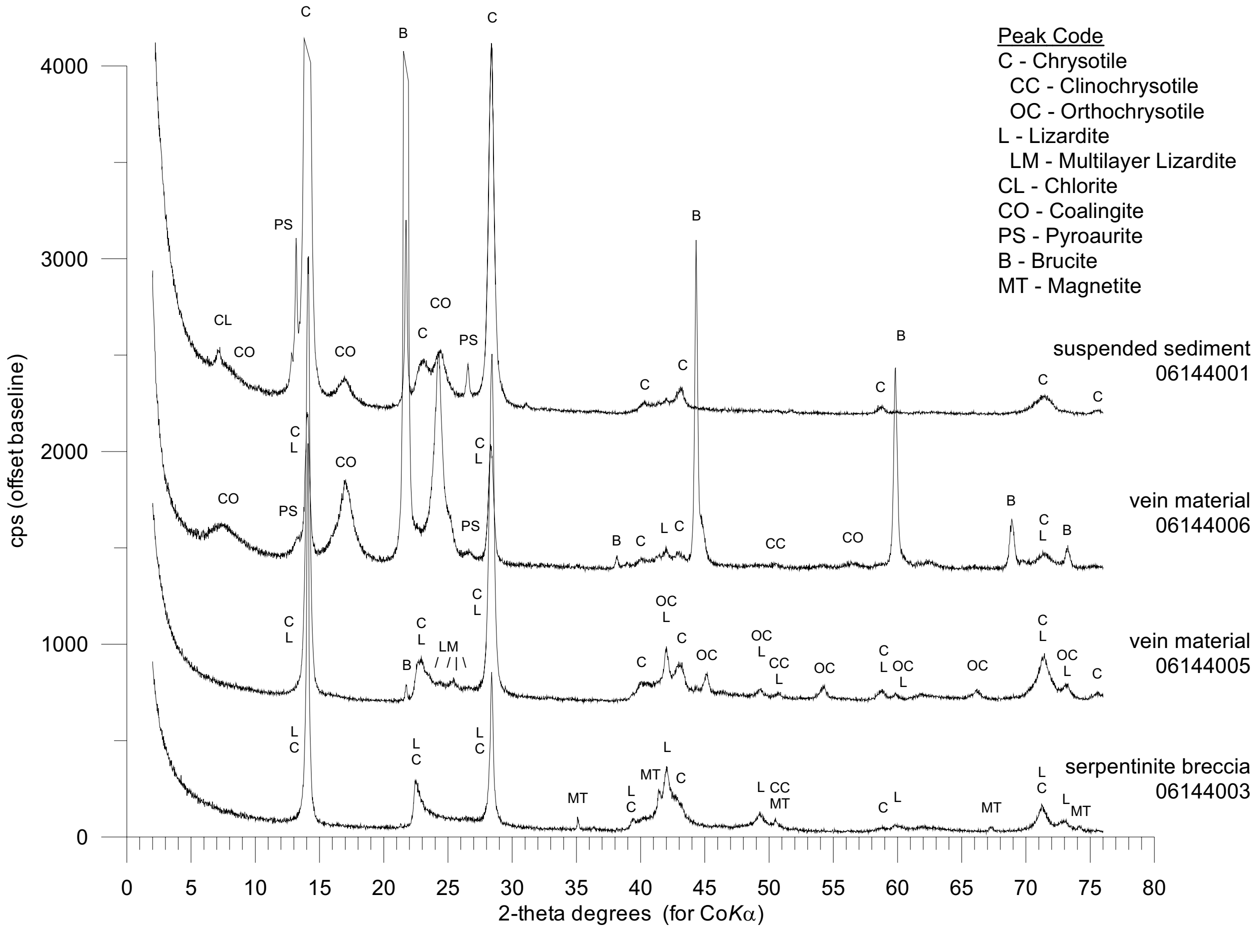

Figure 3. XRD patterns for source materials for sediment in Swift Creek. Collected from the toe of the Swift Creek landslide on Sumas Mountain.

DISCUSSION

A comparison of the source materials with bottom sediment and dredge pile materials indicates that altered serpentinite in the Swift Creek landslide contributes discrete sources of chrysotile, lizardite, the hydroxy minerals (brucite, coalingite and pyroaurite), and chlorite and magnetite to the Swift Creek drainage. Increasing amounts of quartz, feldspar and mica downgradient indicate that the serpentinite components of the fine fractions are increasingly mixed with erodible minerals from other geologic formations as sediment is transported downstream.

The hydroxy minerals associated with the serpentine minerals are acid soluble. The hydroxy minerals are also relatively more soluble in water than other minerals found in these samples, and are likely contributors to elevated magnesium and iron in Swift Creek water. Depending on metal substitution in the hydroxy minerals, they may be contributors for other metals as well.

CONCLUSIONS

The results may be summarized as follows:

1. Twenty-four samples were collected along the Swift Creek channel and bank on April 6, 2006, for the purpose of identifying the mineralogy and morphology of fine-grained materials reported to contain asbestos. Results for a subset of thirteen samples are reported herein.

2. Serpentine minerals were identified in all samples of fines examined from four areas. Samples consisted of source material of suspended sediment, bottom sediment, soft clayey rock, veins and coatings from the toe of the Swift Creek landslide; bottom sediment at the wooden bridge on Swift Creek; bottom sediment and dredge pile material near Goodwin Road; and bottom sediment and dredge pile material near Oat Coles Road.

3. The serpentine consists of a mix of chrysotile and lizardite in major amounts (greater than approximately 20%) derived from the landslide of altered serpentinite. Lizardite and chrysotile (magnesium silicate hydroxides) comprise two of the three more common members of the serpentine group. Note that the XRD method used here provides verification of chrysotile identification based on internal crystalline structure, but does not distinguish various habits or outward forms of the mineral. An asbestiform component of chrysotile occurs in these samples based on microscopic observation, but other chrysotile habits are also present. Consequently, not all chrysotile identified by XRD in this memorandum is asbestos.

4. The serpentine is well-mixed with hydroxy minerals, including brucite (magnesium hydroxide) and coalingite and pyroaurite (magnesium iron hydroxy carbonates).

5. Magnetite (iron oxide) occurs throughout the materials in trace amounts.

6. Other minerals also occur in variable amounts, including chlorite, mica, quartz, feldspar, pyroxene, amphibole, calcite, heulandite zeolite, and chromite.

7. The magnesium hydroxide and magnesium iron hydroxy carbonate minerals are somewhat soluble and are likely sources of dissolved magnesium, iron, and potentially other metals.

8. The fines in the dredge piles are similar in mineral composition to the fines in the bottom sediment.

9. Chrysotile is more concentrated in suspended sediment just below the toe of the landslide than in fines in either the bottom sediment or the dredge piles.

10. Samples have been archived to allow the opportunity for further analysis of suspended sediment, sediment variability along the course of Swift Creek, the mineralogical distribution of soluble metals, and additional detail on the characteristics of chrysotile and amphibole occurrence.

REFERENCES

Bailey, S.W., 1980, Structures of layer silicates, Chapter 1 In Brindley, G.W. and Brown, G., eds., Crystal structures of clay minerals and their X-ray identification: London, Mineralogical Society, Monograph N. 5, p.1-123.

ICDD, 2002, Powder diffraction file, release 2002: Newtown Square, Pennsylvania, International Centre for Diffraction Data, cd-rom.

Whatcom County, 2005, Swift Creek Management Plan: Whatcom County Flood Control Zone District, prepared by Kerr Wood Leidel Associates, Ltd., March 2005, KWL File No. 2039.003.

Whittaker, E.J.W. and Zussman, J, 1956, The characterization of serpentine minerals by X-ray diffraction: Mineralogical Magazine, v. 31, no. 233, p. 107-126.

Wicks, Fred J., 2000, Status of the reference X-ray powder -diffraction patterns for the serpentine minerals in the PDF database - 1997: Powder Diffraction, v. 15, no. 1, p. 42-50.

Wicks, F.J. and O'Hanley, D.S., 1988, Serpentine minerals: structures and petrology In Bailey, S.W., Hydrous phyllosilicates (exclusive of micas), Chapter 5: Mineralogical Society of America, Reviews in Mineralogy, v. 19, 91-167.

ATTACHMENT 1

Photographic Documentation.

Digital images acquired April 6, 2006
Swift Creek Asbestos site
near Sumas, Washington.

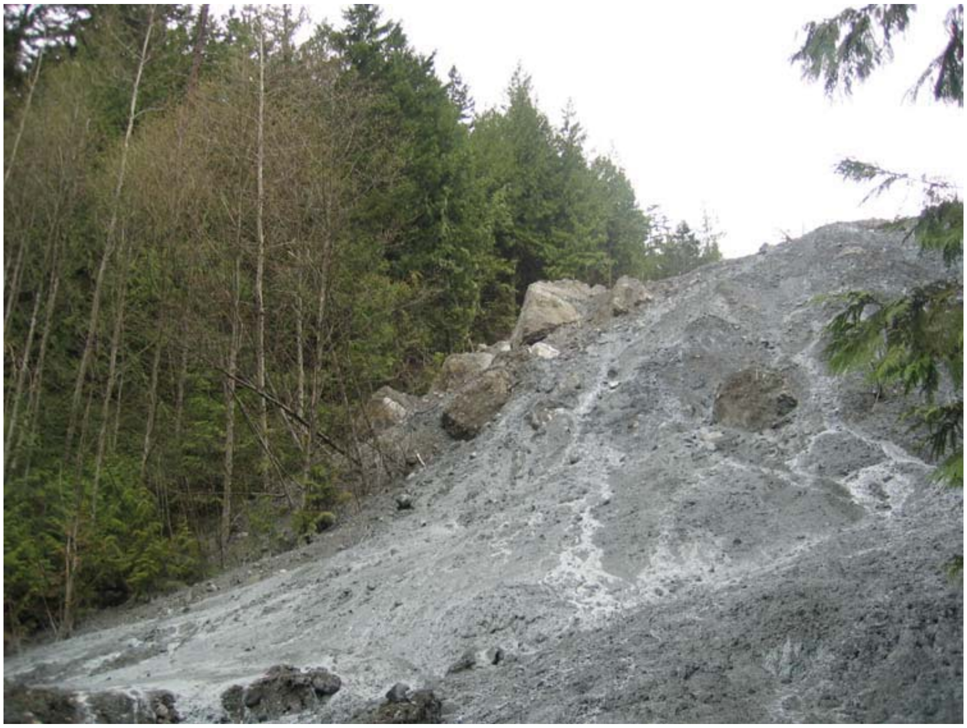

IMG_1301 - Toe of Swift Creek landslide in altered serpentinite, north edge.

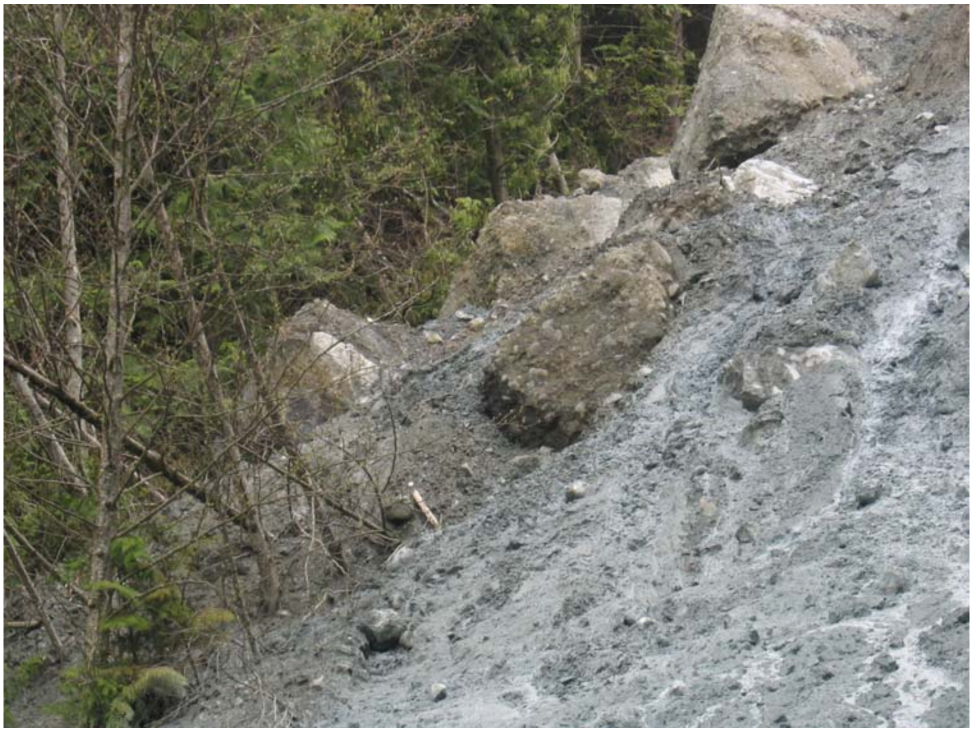

IMG_1302 - Toe of Swift Creek landslide in altered serpentinite, north edge close-up.

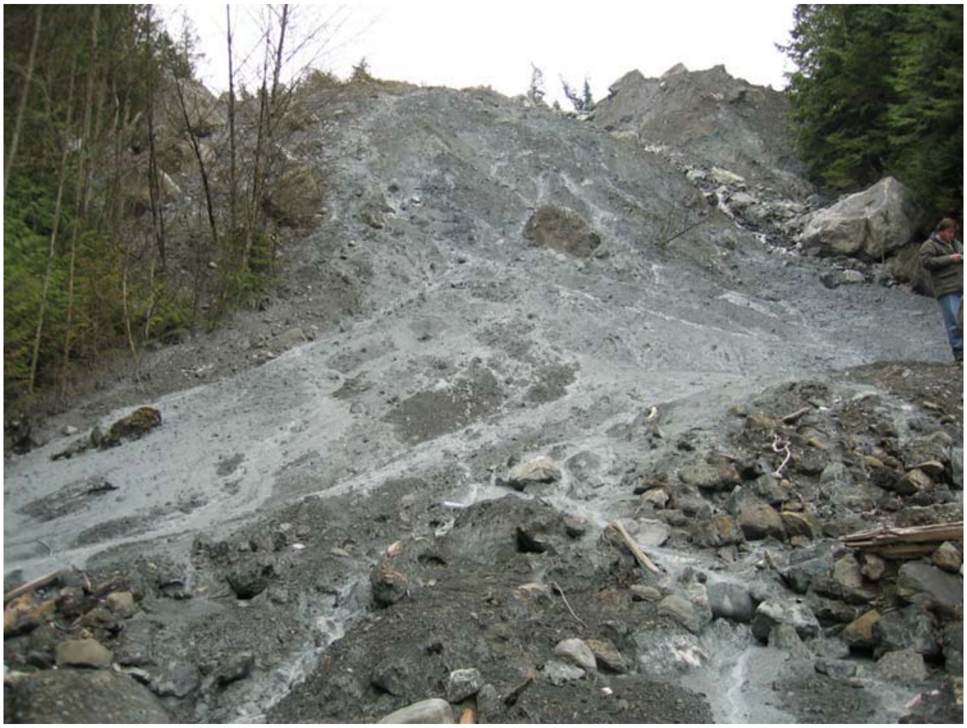

IMG_1305 - Toe of Swift Creek landslide in altered serpentinite, general locality for samples 06144000-06144006.

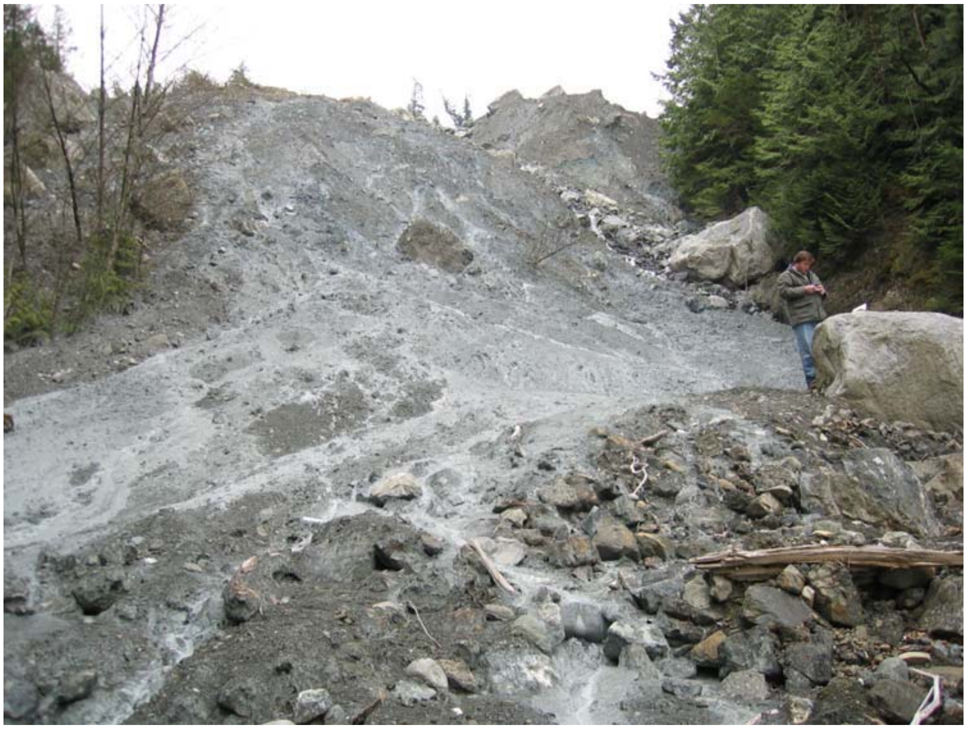

IMG_1306 - Toe of Swift Creek landslide in altered serpentinite, general locality for samples 06144000-06144006.

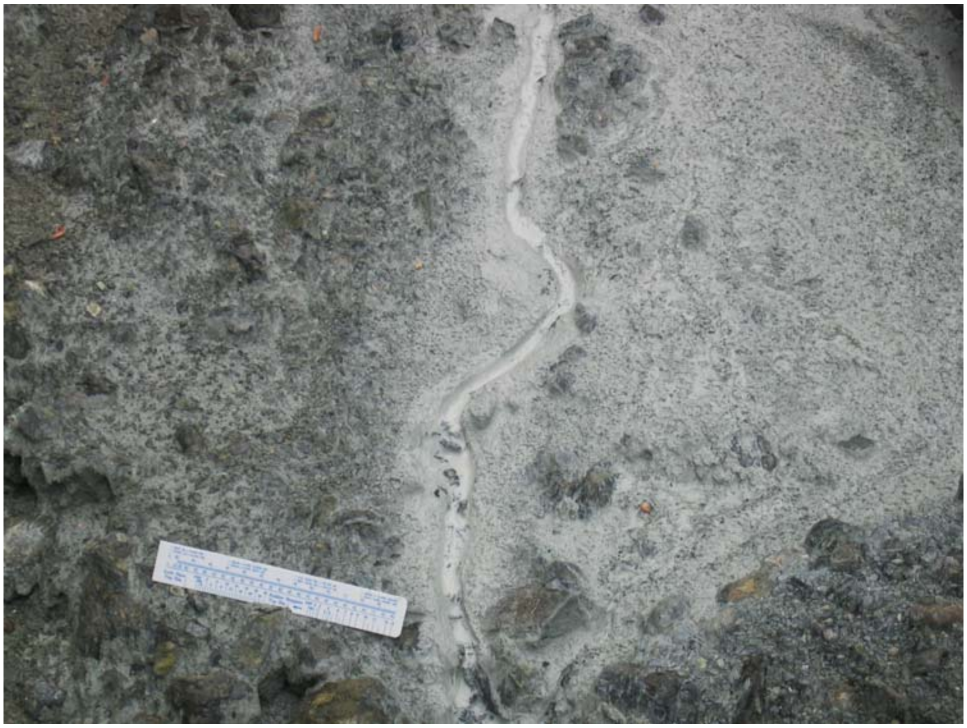

IMG_1310 - Rivulet of chrysotile-laden water from toe of Swift Creek landslide, site for sediment sample 06144000. 16-cm ruler for scale.

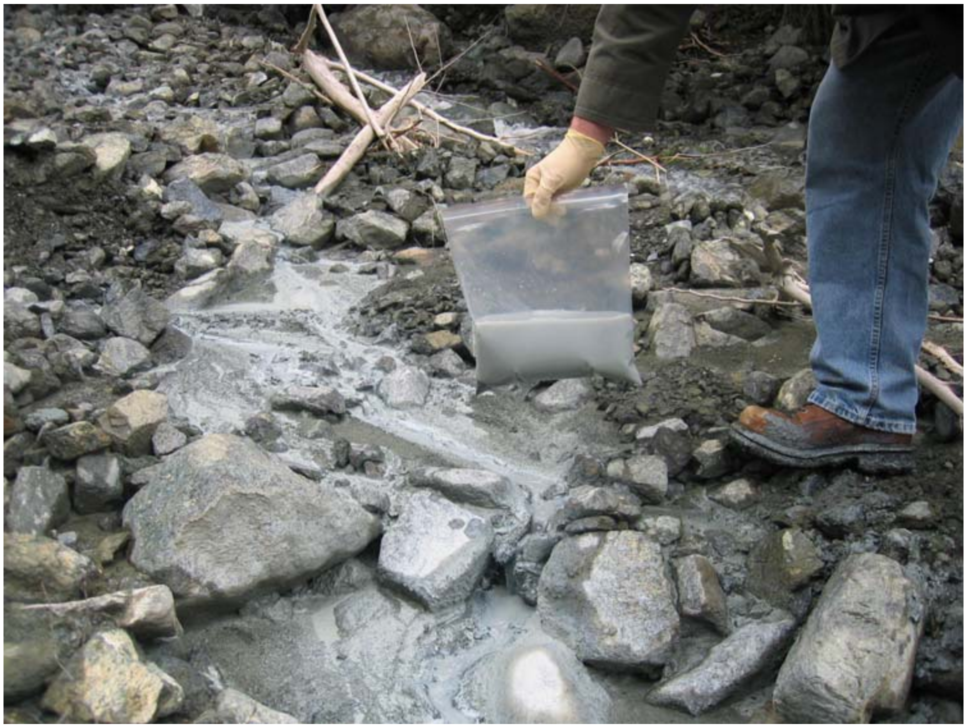

IMG_1311 - Stream of chrysotile-laden water from toe of Swift Creek landslide, water sample 06144001.

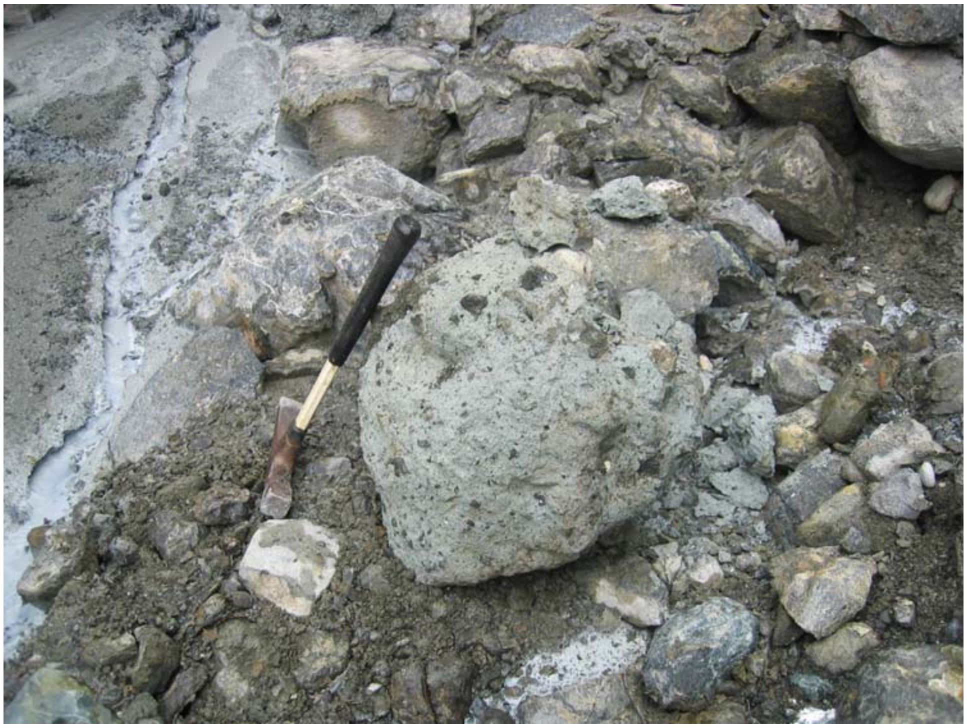

IMG_1312 - Block of serpentinitic landslide gouge at toe of Swift Creek landslide, source of rock sample 06144003. Rock hammer for scale.

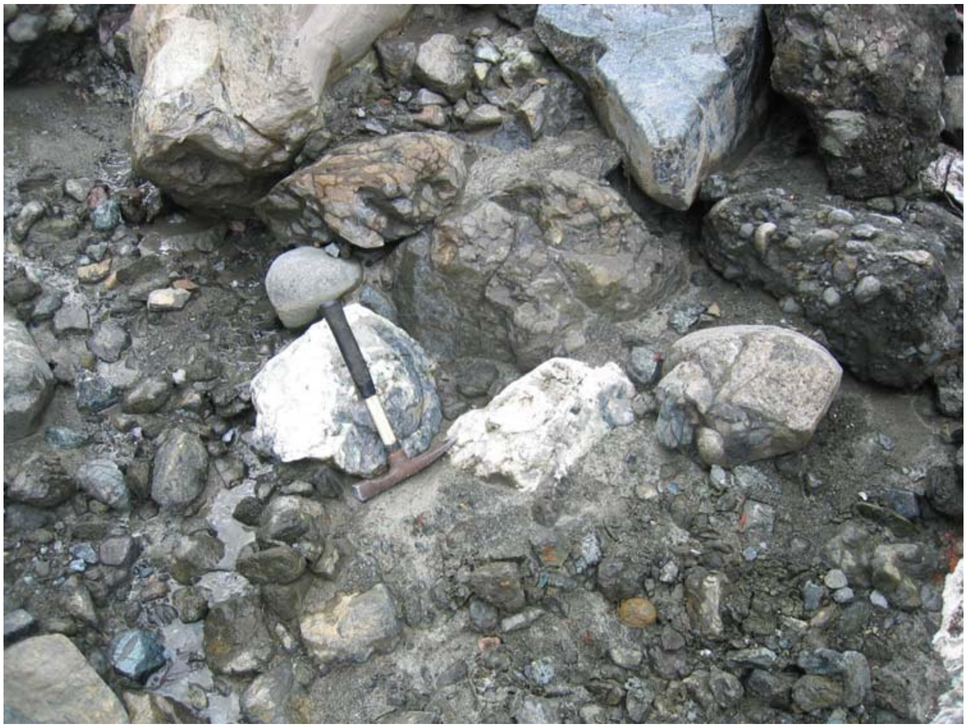

IMG_1313 - Serpentinite blocks with pale bluish green fibrous coatings, at toe of Swift Creek landslide. Rock hammer for scale.

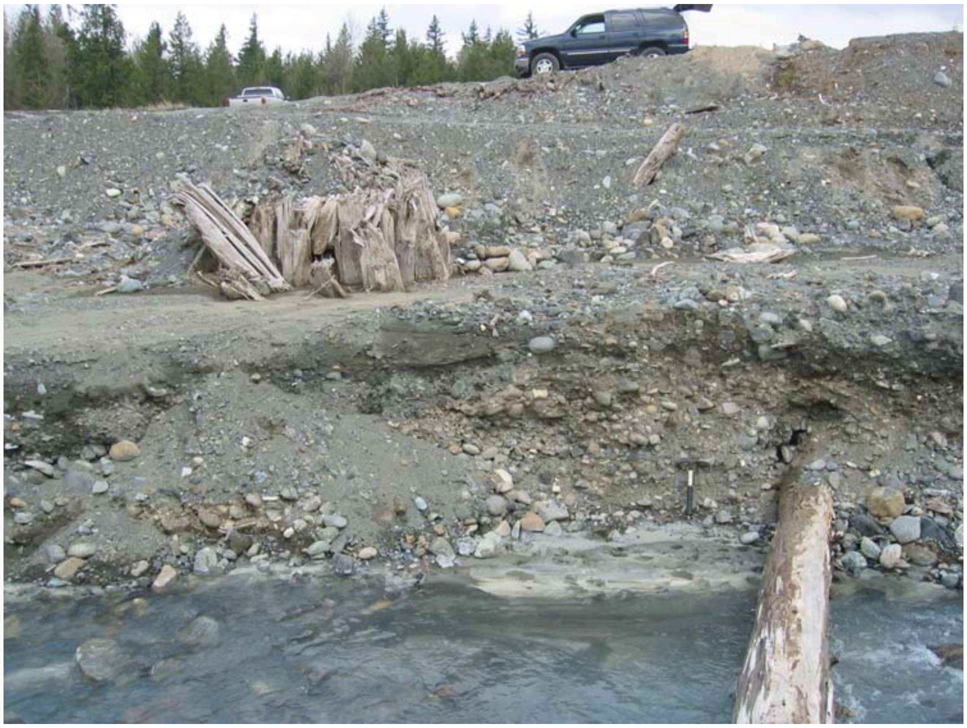

IMG_1314 – Northwest bank of Swift Creek near bridge at Great Western Lumber property. Brown serpentinite-poor sandy gravel occurs in lower part of bank. Medium-grey serpentinite-rich gravelly sand occurs in upper part of bank and in debris cones that have sloughed into the creek (lower left of image). Serpentinite-rich material also makes up the roadbed. Recent deposits of pale grey serpentine-rich mud occur along the water line and side bars of Swift Creek (below hammer). Near locality for samples 06144007-06144009.

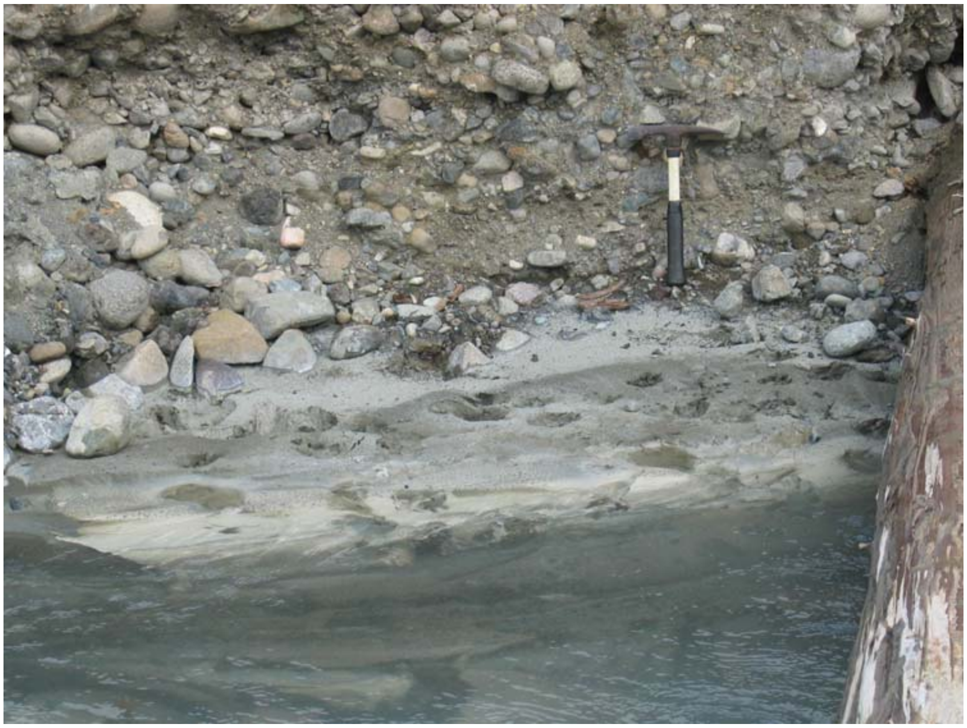

IMG_1315 - Northwest bank of Swift Creek near bridge at Great Western Lumber property. Close-up of recent deposits of pale grey serpentine-rich mud along the water line and side bar of Swift Creek (below hammer). Near locality for samples 06144007-06144009.

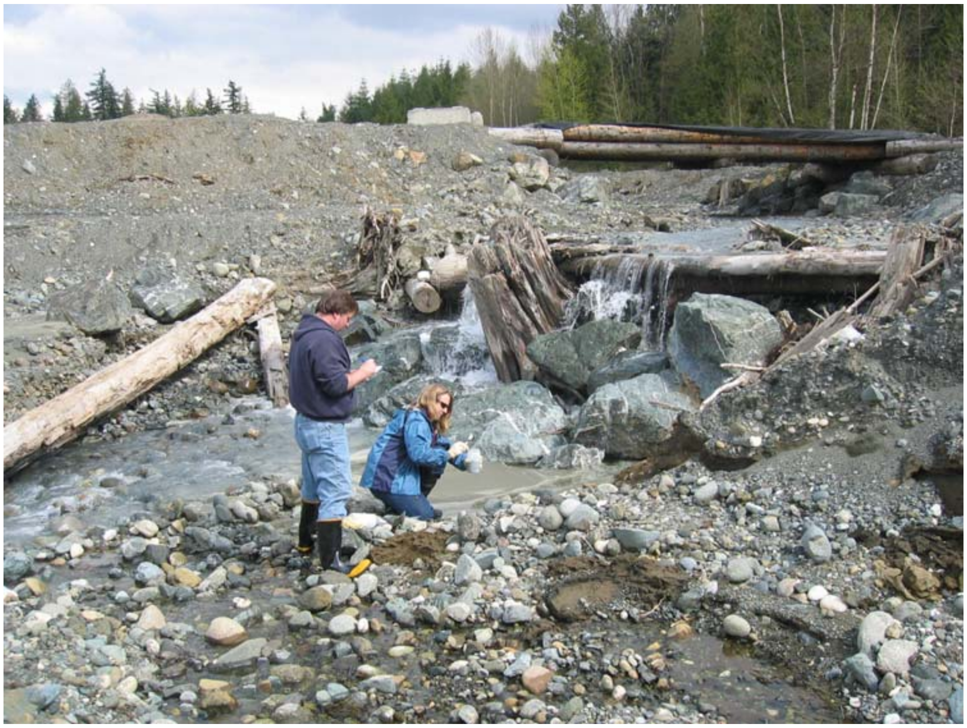

IMG_1316 - Collecting pale grey sediment sample 06144007 from channel of Swift Creek near bridge at Great Western Lumber property.

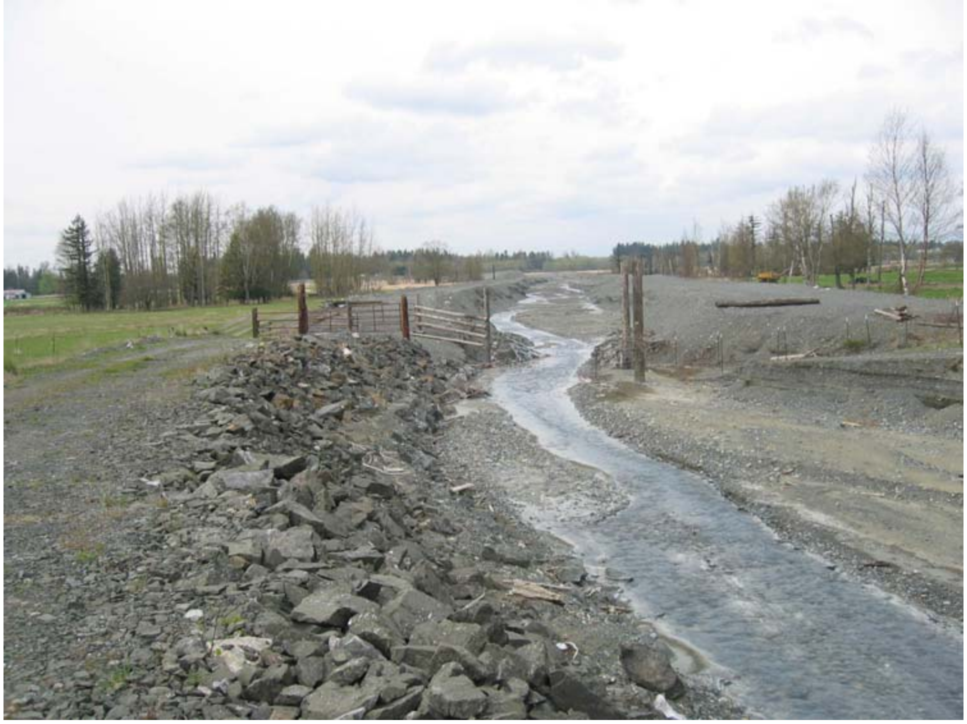

IMG_1317 - Swift Creek and adjacent dredge piles near fence at west edge of Gimmaka property near Goodwin Road, looking downstream to the northwest. Locality of pale brownish grey, mud-cracked sediment sample 06144017 on far side of creek.

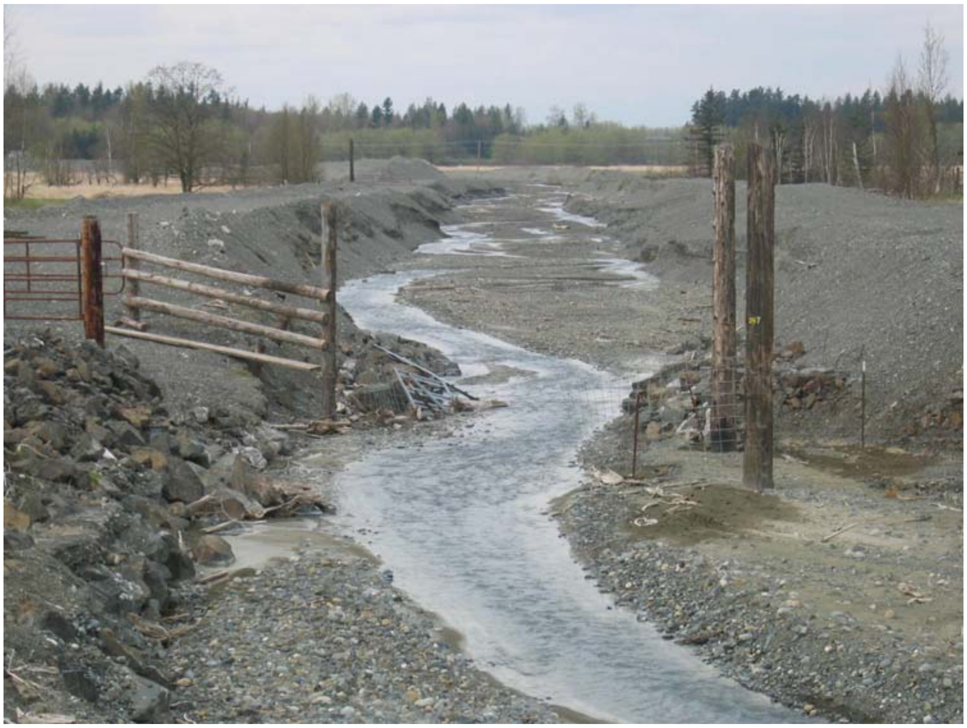

IMG_1318 - Swift Creek and adjacent dredge piles near fence at west edge of Gimmaka property near Goodwin Road, looking downstream to the northwest. Close-up of locality of pale brownish grey, mud-cracked sediment sample 06144017 on far side of creek.

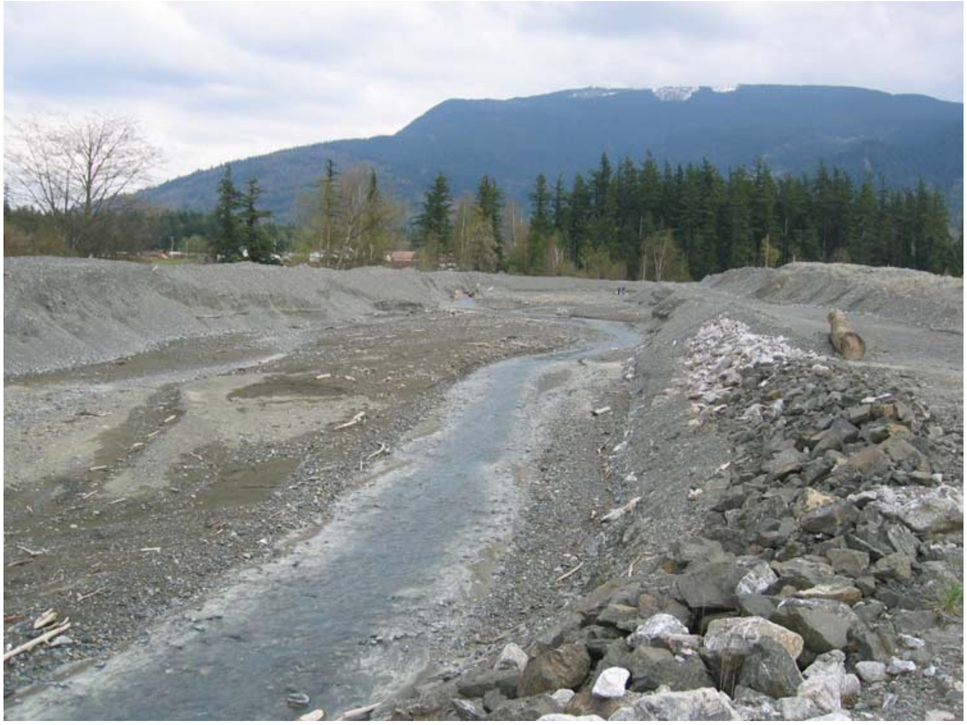

IMG_1319 - Swift Creek and adjacent dredge piles at Gimmaka property near Goodwin Road, looking upstream to the east toward Sumas Mountain. General locality of samples 06144010-06144019.

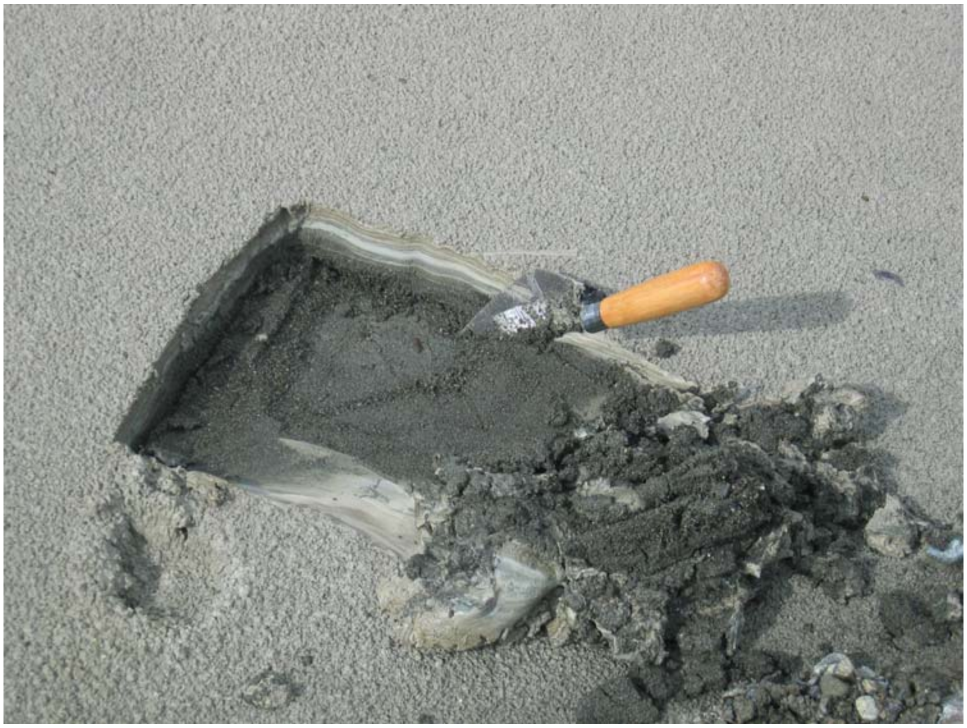

IMG_1321 - Excavation in damp laminated sediment in channel of Swift Creek at Gimmaka property near Goodwin Road. Samples 06144011-06144013 taken from lamination sequence, respectively, from top to bottom.

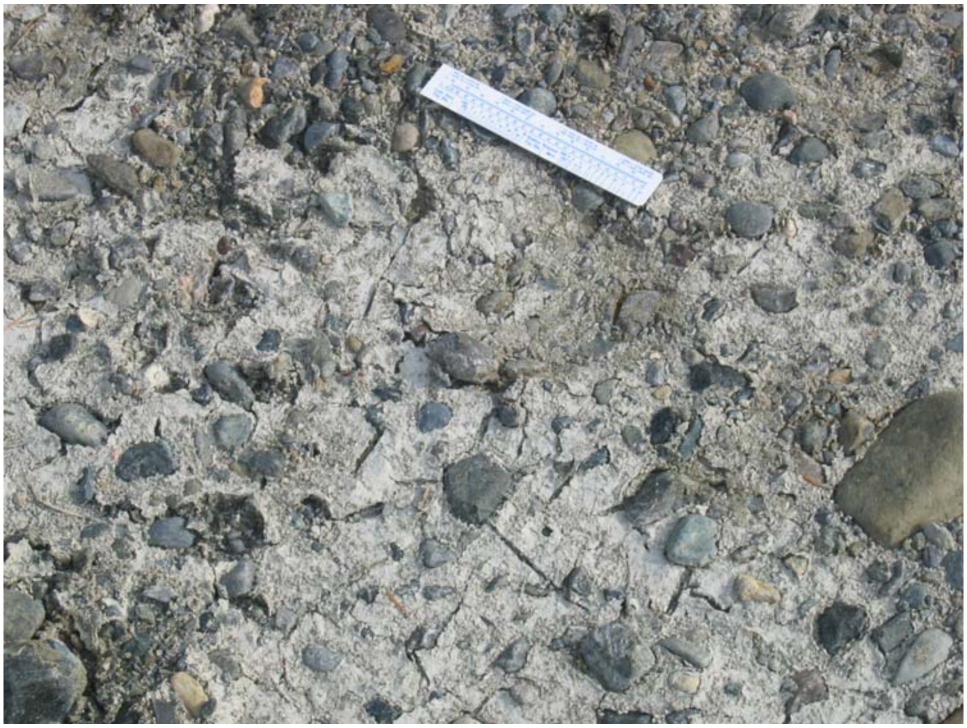

IMG_1327 - Dry, mud-cracked sediment in channel of Swift Creek at Gimmaka property near Goodwin Road. Caked fines collected as sediment sample 06144010.

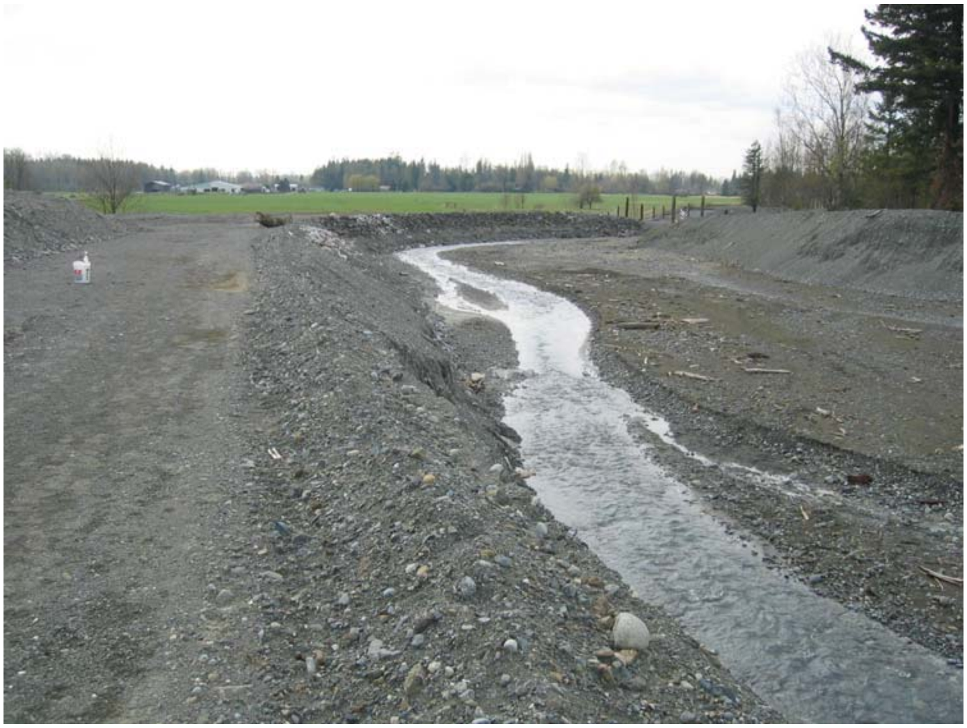

IMG_1328 - Swift Creek and adjacent dredge piles at Gimmaka property near Goodwin Road, looking downstream to the west. Water samples 06144015-06144016 collected in the rippled reach (middle of image).

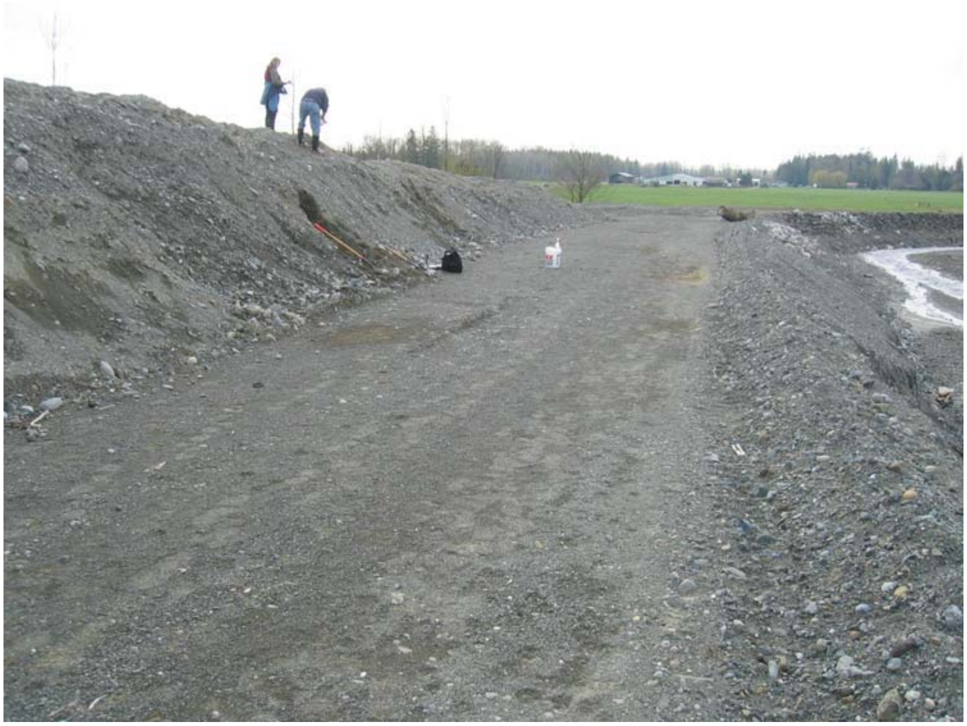

IMG_1329 - Sampling of dredge pile on the south side of Swift Creek at Gimmaka property near Goodwin Road, looking downstream to the west. Surface sample 06144018 taken from the top of the pile. Interior sample 06144019 taken from the interior of the pile at the excavation.

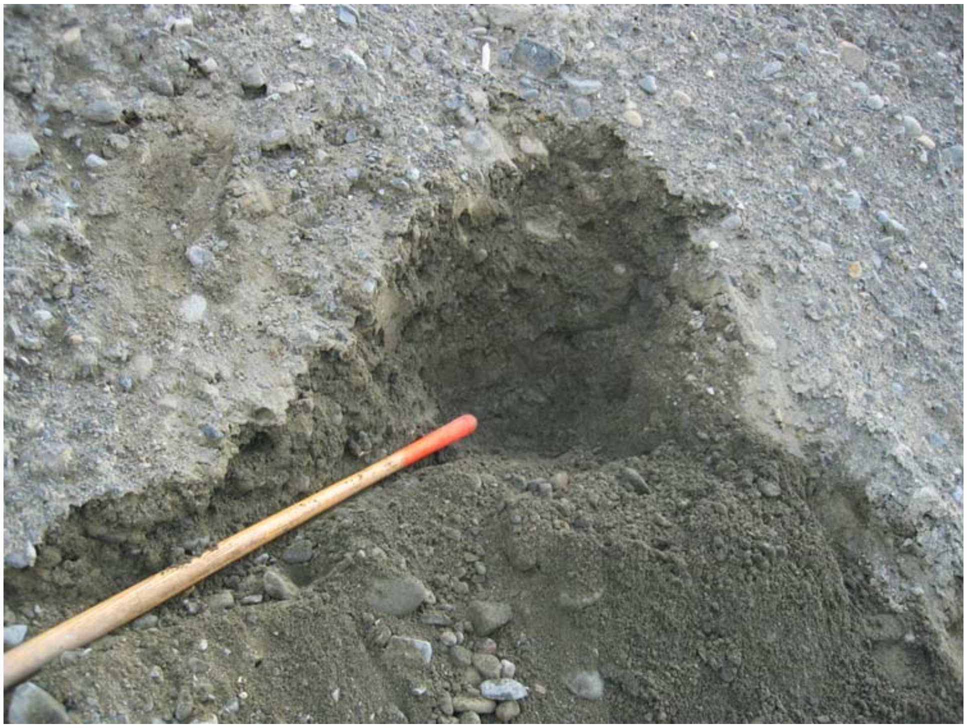

IMG_1330 - Excavation for sample 06144019 taken from the interior of the dredge pile, Gimmaka property near Goodwin Road. Shovel handle points to brown, fine-grained clump used for the sample.

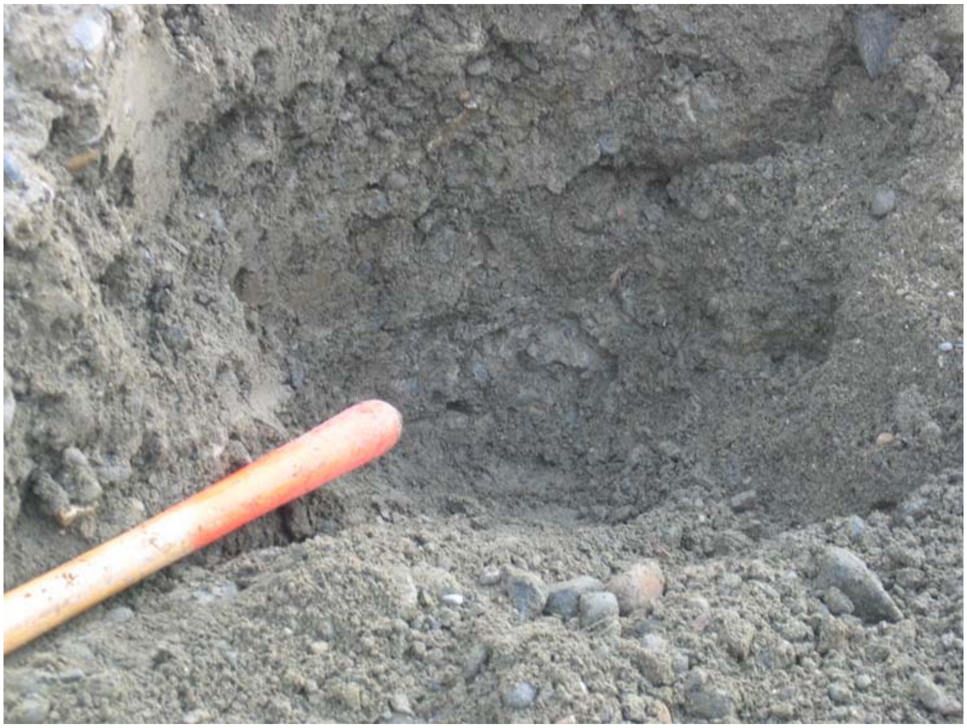

IMG_1331 - Close-up of excavation for sample 06144019 taken from the interior of the dredge pile, Gimmaka property near Goodwin Road. Shovel handle points to brown, fine-grained clump used for the sample.

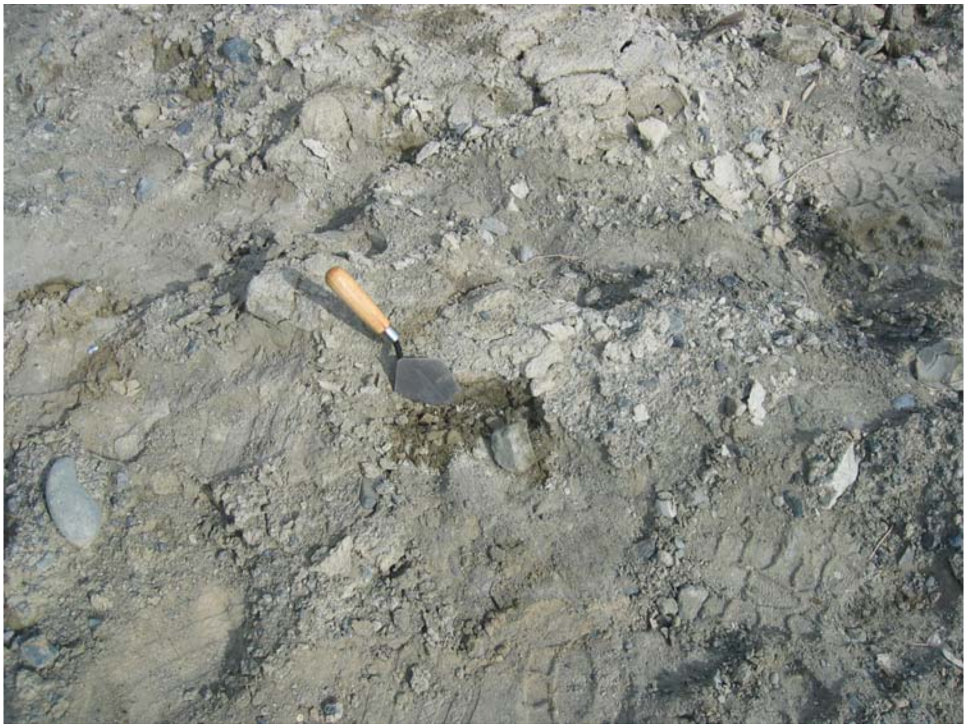

IMG_1332 - Close-up of fine-grained clumps similar to sample 06144018 taken from the surface of the top of the dredge pile, Gimmaka property near Goodwin Road.

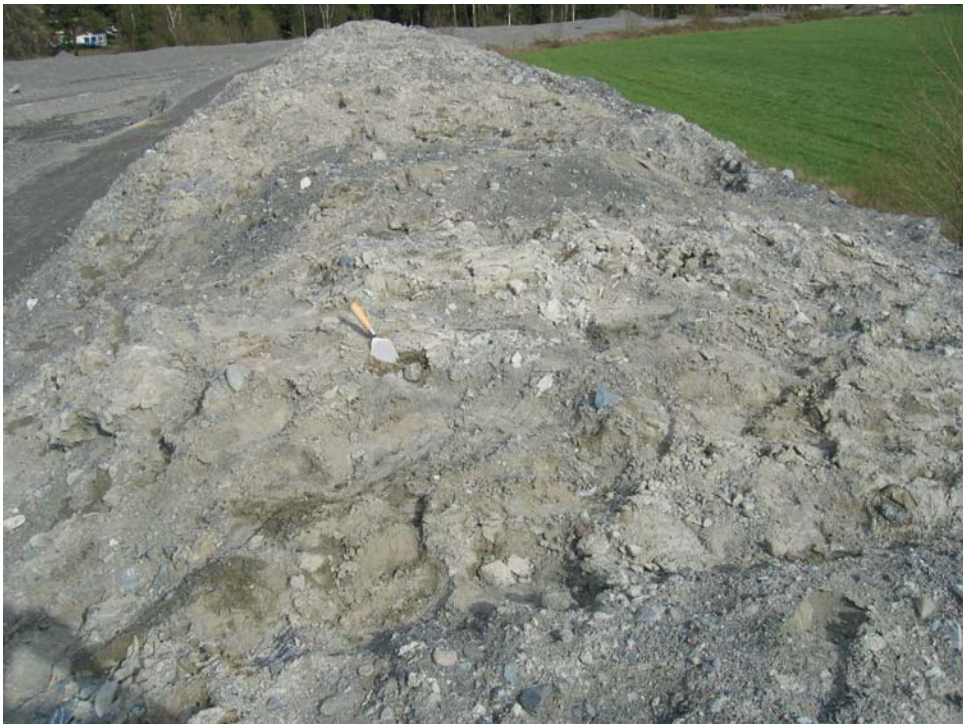

IMG_1333 – Fine-grained clumps, in area of sample 06144018, scattered across the top of the dredge pile, Gimmaka property near Goodwin Road, looking upstream to the east.

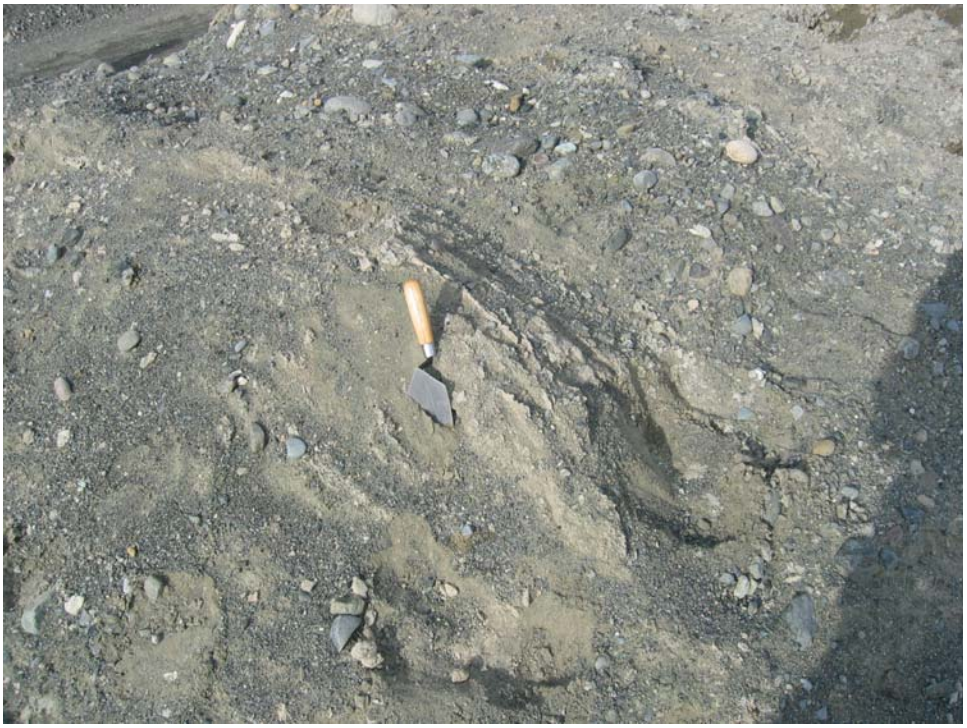

IMG_1334 - Close-up of an upturned block of clumpy, laminated fined-grained bottom sediment (to the right of the trowel) exposed on the top of the dredge pile, Gimmaka property near Goodwin Road. Near locality of dredge-pile sample 06144018.

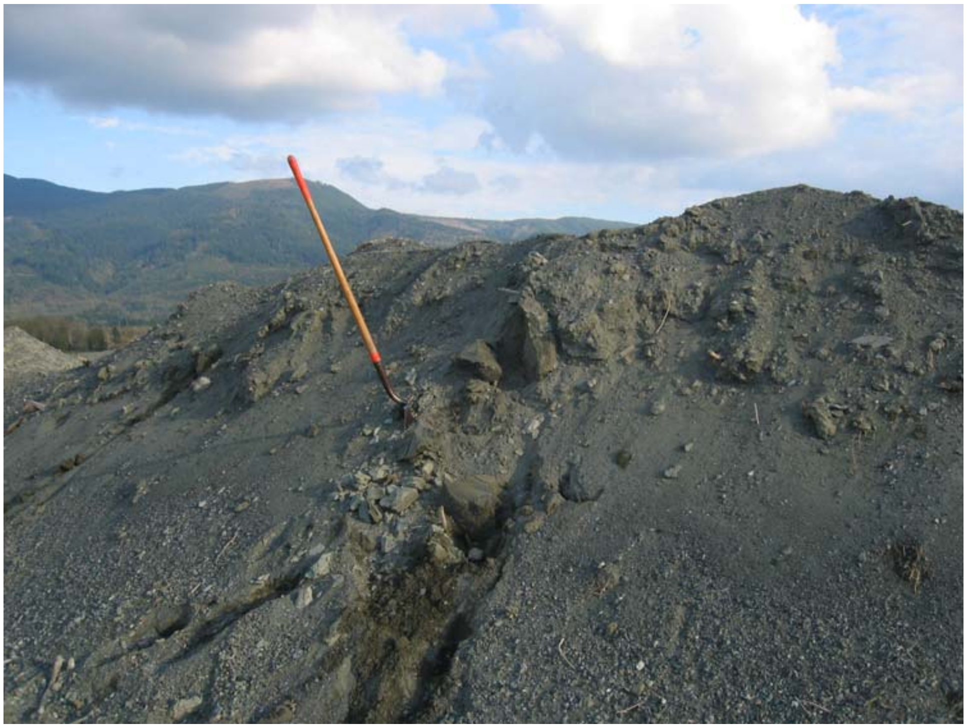

IMG_1335 - Excavation for sample 06144020 taken from the side of one of several blocks of laminated fine-grained bottom sediment exposed near the top of the dredge pile, Parker property near Oat Coles Road, looking southeast toward Sumas Mountain.

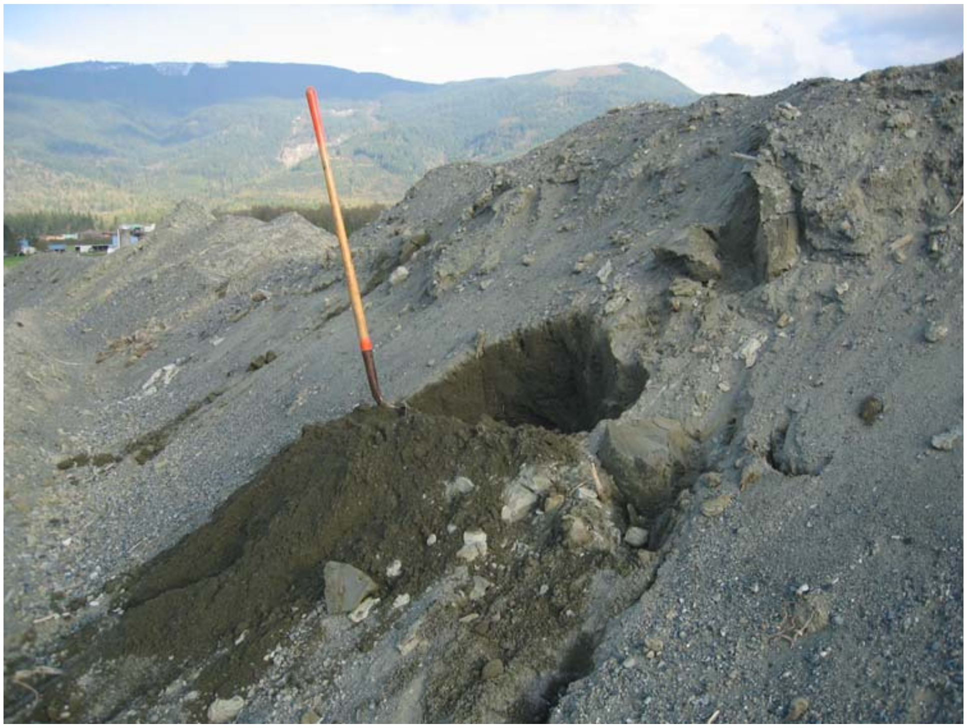

IMG_1336 - Excavation for sample 06144021 taken from a fine-grained clump in the interior of the dredge pile, Parker property near Oat Coles Road, looking east toward Sumas Mountain, with Swift Creek landslide appearing behind the shovel handle.

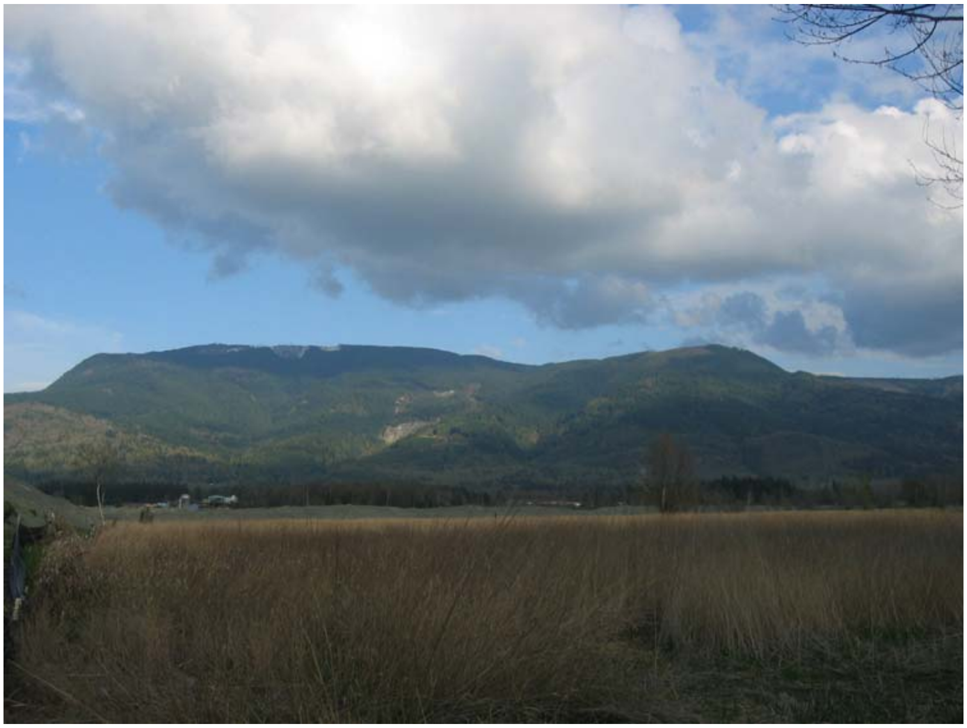

IMG_1337 - Eastward view across pastureland from Oat Coles Road toward Sumas Mountain and the Swift Creek landslide. Dredge piles along Swift Creek cross the lower middle of the image.

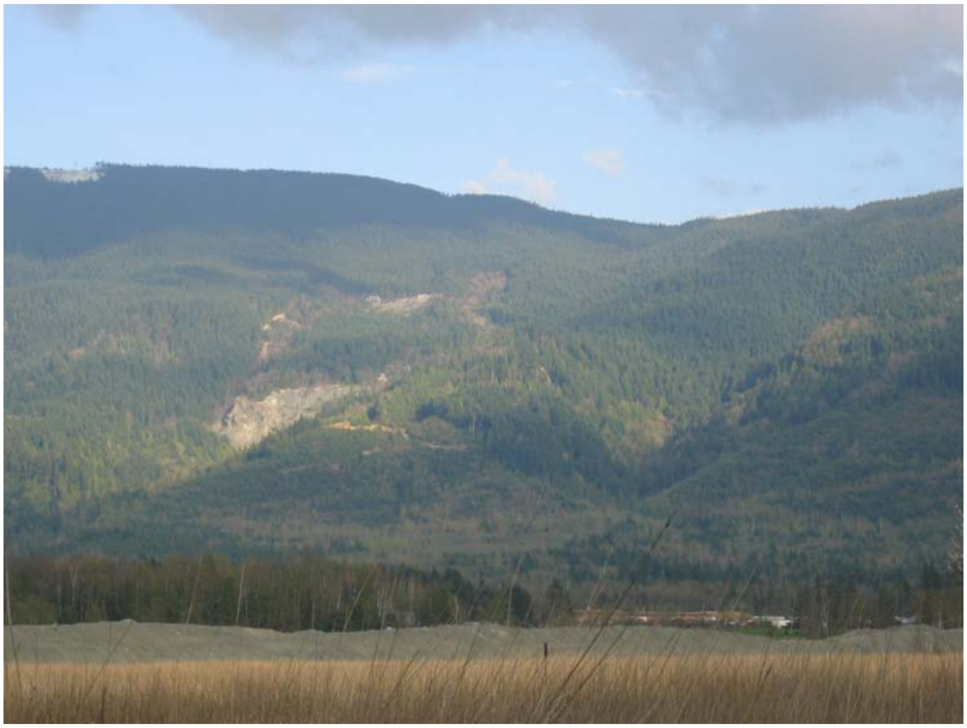

IMG_1338 - Close-up of eastward view from Oat Coles Road toward Sumas Mountain and the Swift Creek landslide. The toe of the landslide is the light grey barren slope in the left of middle in the image. Newly constructed logging roads crisscross the ridge adjacent to the landslide toe. Dredge piles along Swift Creek cross the lower middle of the image.

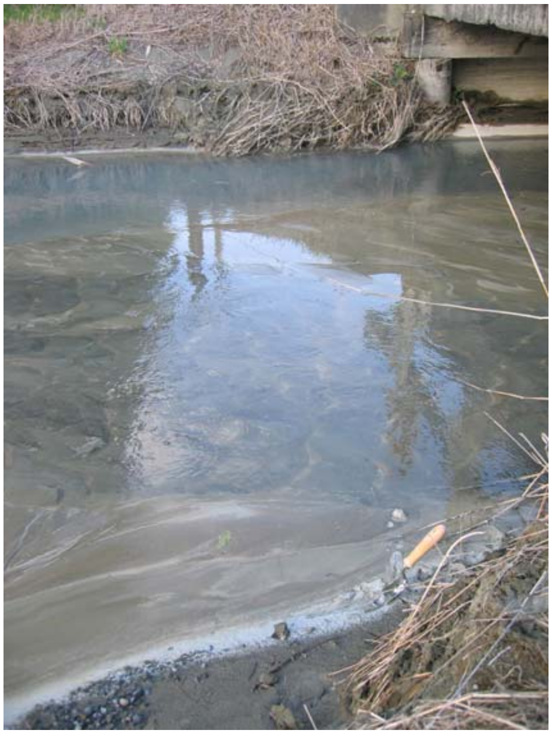

IMG_1339 - Brown to light-grey, fine-grained bottom sediment in Swift Creek, west of Oat Coles Road bridge. Sample 06144023 collected at trowel. Bridge abutment at upper right.

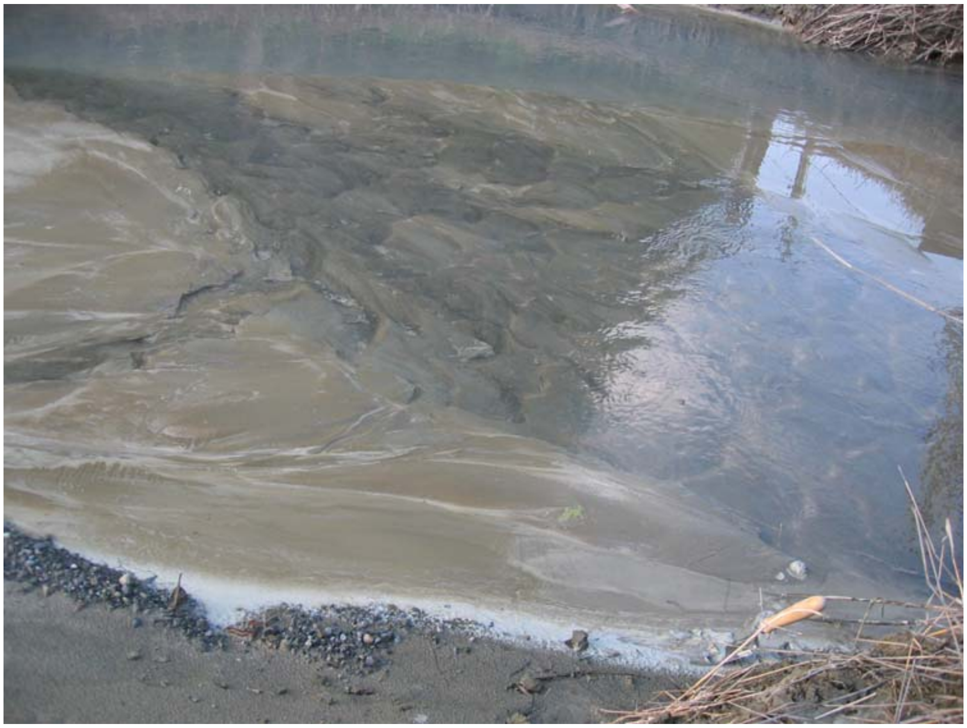

IMG_1340 - Brown to light-grey, fine-grained bottom sediment in Swift Creek, west of Oat Coles Road bridge. Sample 06144023 collected at trowel.

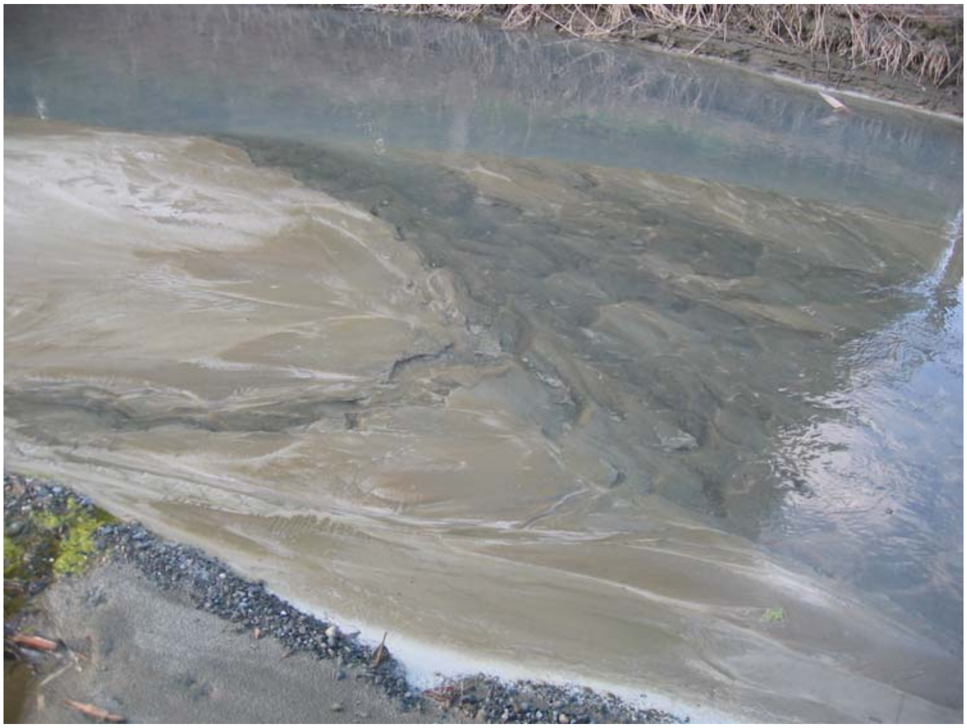

IMG_1341 - Brown to light-grey, fine-grained bottom sediment in Swift Creek, west of Oat Coles Road bridge. Near site for sample 06144023, downstream extension of image 1340.

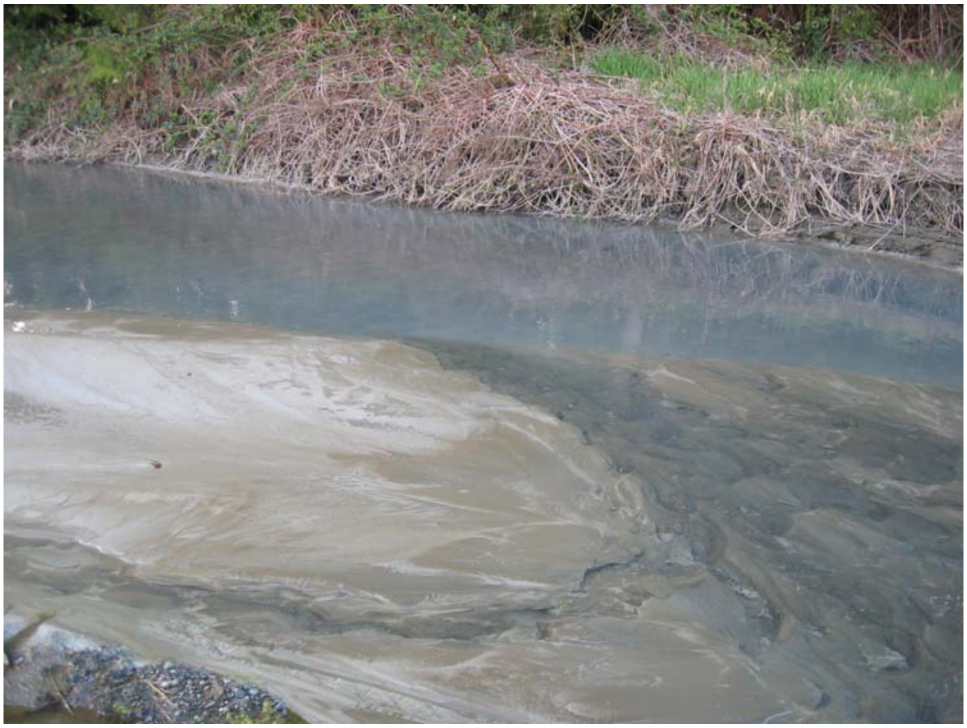

IMG_1342 - Brown to light-grey, fine-grained bottom sediment in Swift Creek, west of Oat Coles Road bridge. Near site for sample 06144023, downstream extension of images 1340-1341.

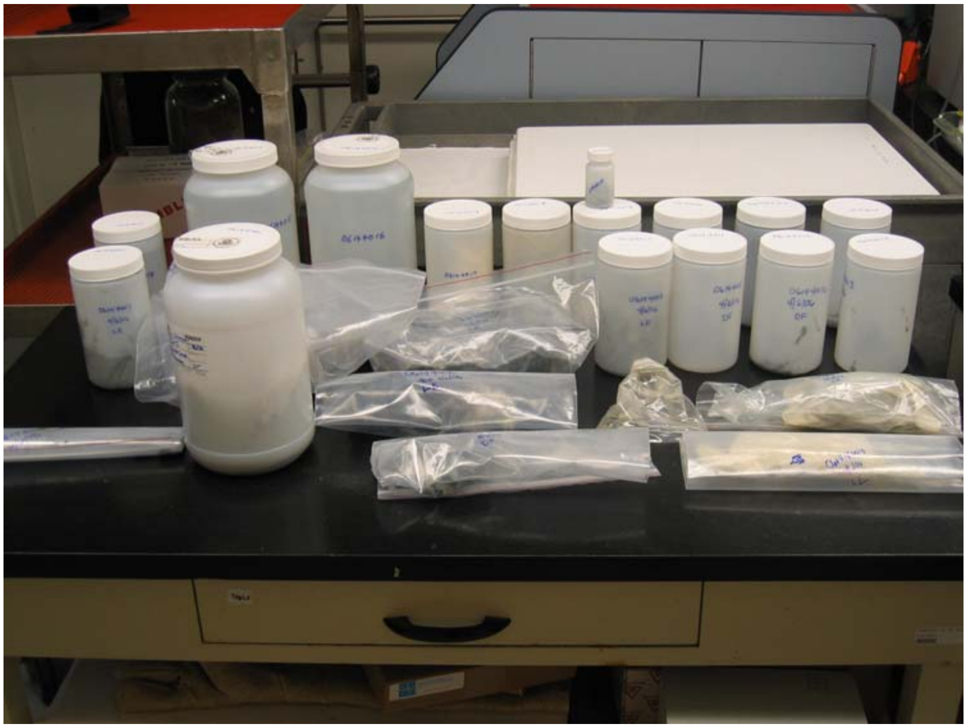

IMG_1344 - Samples 06144000-06144023 collected April 6, 2006 at Swift Creek Asbestos site, near Sumas, Washington. April 10, USEPA Region 10 Manchester Environmental Laboratory.
