# Supplementary material for: Comparison of soil sampling and analytical methods for asbestos at the Sumas Mountain Asbestos Site—Working towards a toolbox for better assessment
Source: PLoS One. 2017 Jul 31;12(7):e0180210. doi: 10.1371/journal.pone.0180210 (PMC5536314; doi:10.1371/journal.pone.0180210)
Supplement: S13 Text — El Dorado Hills Naturally Occurring Asbestos Multimedia Exposure Study. (PDF) [file pone.0180210.s014.pdf]

US EPA ARCHIVE DOCUMENT

US EPA ARCHIVE DOCUMENT

El Dorado Hills
Naturally Occurring Asbestos
Multimedia Exposure Assessment
El Dorado Hills, California

Preliminary Assessment and Site Inspection Report
Interim Final

Contract No.: 68-W-01-012
TDD No.: 09-04-01-0011
Job No.: 001275.0440.01CP

May 2005

Prepared for:

United States Environmental Protection Agency
Region IX

Prepared by:
Karen Ladd, START
Ecology and Environment, Inc.

Table of Contents

Section **Page**

1 Introduction 1-1

2 Apparent Problem 2-1

3 Site Description and History 3-1
3.1 Site Location 3-1
3.2 Site Description 3-3
3.3 Site History 3-4

4 Regulatory Involvement 4-1

5 Summary of Investigative Efforts 5-1
5.1 Objectives 5-1
5.2 Scope of Work 5-2
5.3 Schedule 5-3
5.4 Air Sampling 5-3
5.4.1 Ambient Outdoor Air Sampling 5-3
5.4.1.1 Fixed Ambient Air Sampling at the Community Park 5-5
5.4.1.2 Fixed Ambient Air Sampling for Activity-Based Reference Level Determinations 5-7
5.4.1.3 Perimeter Monitoring on the New York Creek Nature Trail 5-16
5.4.2 Activity-Based Air Sampling 5-20
5.4.2.1 Silva Valley Elementary School 5-26
5.4.2.2 Rolling Hills Middle School 5-36
5.4.2.3 Children's Playground at the Community Park 5-45
5.4.2.4 Playing Fields at the Community Park 5-53
5.4.2.5 New York Creek Nature Trail 5-82
5.4.2.6 Jackson Elementary School 5-90
5.5 Soil Sampling 5-107

6 Hazard Ranking System Factors 6-1
6.1 Sources of Contamination 6-2
6.1.1 Areas of Concern 6-2
6.1.2 Contaminant Types and Quantities 6-2
6.2 Groundwater Migration Pathway 6-4
6.3 Surface Water Migration Pathway 6-4
6.4 Soil Exposure Pathway 6-6
6.4.1 Contamination 6-6
6.4.2 Population 6-6
6.4.3 Sensitive Environments 6-7

US EPA ARCHIVE DOCUMENT

Table of Contents (Cont'd.)

Section **Page**

6.5 Air Migration Pathway . . . 6-7
6.5.1 Release of Asbestos to Air . . . 6-8
6.5.2 Population . . . 6-11
6.5.3 Sensitive Environments . . . 6-11

7 Emergency Response Considerations . . . 7-1

8 Summary . . . 8-1

Appendix

A Meteorological Data
B Dust Monitoring Results
C References

US EPA ARCHIVE DOCUMENT

List of Tables

Table		Page
5-1	Ambient Air Monitoring Station Air Sample Summary Results	5-8
5-2	Southern Reference Area Air Sample Summary Results	5-11
5-3	Northern Reference Area Air Sample Summary Results	5-17
5-4	New York Creek Nature Trail Perimeter Monitoring Air Sample Summary Results	5-21
5-5	Final Schedule of Field Work	5-25
5-6	Silva Valley Elementary School Baseball Playing Field Maintenance Scenario Air Sample Summary Results	5-29
5-7	Moisture Levels in Activity Area Soils	5-32
5-8	Silva Valley Elementary School Baseball Scenario A Air Sample Summary Results	5-35
5-9	Silva Valley Elementary School Baseball Scenario B Air Sample Summary Results	5-38
5-10	Rolling Hills Middle School Soccer Scenario Air Sample Summary Results	5-42
5-11	Rolling Hills Middle School Basketball Scenario Air Sample Summary Results	5-47
5-12	Children's Playground Scenario Activities	5-50
5-13	Status of Leaf Blower and Fans During Aggressive Activity Scenario	5-52
5-14	Community Park Children's Playground Typical and Aggressive Activity Scenarios Air Sample Summary Results	5-55
5-15	Community Park North Field Baseball Scenario Air Sample Summary Results	5-59
5-16	Community Park South Field Baseball Scenario A Air Sample Summary Results	5-63
5-17	Community Park South Field Baseball Scenario B Air Sample Summary Results	5-67
5-18	Community Park South Field Baseball Scenario C (Without Field Maintenance) Air Sample Summary Results	5-71
5-19	Community Park New York Creek Field Baseball Scenario Air Sample Summary Results	5-75
5-20	Community Park Lower Soccer Field Soccer Scenario Air Sample Summary Results	5-80
5-21	Biking Scenario Positions	5-83
5-22	New York Creek Nature Trail Biking Scenario Air Sample Summary Results	5-85
5-23	Positions for Jogging/Walking Scenario A	5-87
5-24	New York Creek Nature Trail Jogging/Walking Scenario A Air Sample Summary Results	5-89

US EPA ARCHIVE DOCUMENT

List of Tables (Cont.)

Table **Page**

5-25 Positions for Jogging/Walking Scenario B 5-90
5-26 New York Creek Nature Trail Jogging/Walking Scenario B Air Sample Summary Results 5-91
5-27 Jackson Elementary School Garden/Outdoor Classroom Scenario Air Sample Summary Results 5-95
5-28 Jackson Elementary School Soccer Scenario Air Sample Summary Results 5-99
5-29 Jackson Elementary School Basketball and Kindergarten Playground Scenario Air Sample Summary Results 5-104
6-1 Summary of Air Sample Results 6-9

US EPA ARCHIVE DOCUMENT

List of Illustrations

Figure **Page**

Figure		Page
**3-1**	Sites Location Map	3-2
**5-1**	Fixed Ambient Outdoor Air Sampling Locations	5-6
**5-2**	New York Creek Nature Trail Perimeter Monitoring Locations	5-19
**5-3**	Scenarios Location Map	5-23
**5-4**	Silva Valley Elementary School Baseball Playing Field Activity-Based Outdoor Air Sampling Locations–Maintenance Scenario	5-28
**5-5**	Silva Valley Elementary School Baseball Playing Field Activity-Based Outdoor Air Sampling Locations–Baseball Scenario A	5-34
**5-6**	Silva Valley Elementary School Baseball Playing Field Activity-Based Outdoor Air Sampling Locations–Baseball Scenario B	5-37
**5-7**	Rolling Hills Middle School Soccer Field Activity-Based Outdoor Air Sampling Locations–Soccer Scenario	5-41
**5-8**	Rolling Hills Middle School Activity-Based Outdoor Air Sampling Locations–Basketball Scenario	5-46
**5-9**	Community Park Children's Playground Activity-Based Outdoor Air Sampling Area–Typical and Aggressive Activity Scenarios	5-54
**5-10**	Community Park North Field Activity-Based Outdoor Air Sampling Locations–Baseball Scenario	5-58
**5-11**	Community Park South Field Activity-Based Outdoor Air Sampling Locations–Baseball Scenario A	5-62
**5-12**	Community Park South Field Activity-Based Outdoor Air Sampling Locations–Baseball Scenario B	5-66
**5-13**	Community Park South Field Activity-Based Outdoor Air Sampling Locations–Baseball Scenario C (Without Field Maintenance)	5-70
**5-14**	Community Park New York Creek Field Activity-Based Outdoor Air Sampling Locations–Baseball Scenario	5-74
**5-15**	Community Park Lower Soccer Field Activity-Based Outdoor Air Sampling Locations–Soccer Scenario	5-79
**5-16**	New York Creek Nature Trail Activity-Based Outdoor Air Sampling Locations–Biking Scenario	5-84
**5-17**	New York Creek Nature Trail Activity-Based Outdoor Air Sampling Locations–Jogging/Walking Scenarios	5-88
**5-18**	Jackson Elementary School Garden and Outdoor Classroom Activity-Based Outdoor Air Sampling Locations–Garden/Outdoor Classroom Scenario	5-94
**5-19**	Jackson Elementary School Grass-Covered Playing Field Activity-Based Outdoor Air Sampling Locations–Soccer Scenario	5-98
**5-20**	Jackson Elementary School Basketball Court and Kindergarten Playground Activity-Based Outdoor Air Sampling Locations–Basketball and Kindergarten Playground Scenario	5-103

US EPA ARCHIVE DOCUMENT

List of Illustrations (Cont'd.)

Figure **Page**

5-21 Silva Valley Elementary School Baseball Playing Field Soil Sample Locations 5-108
5-22 Rolling Hills Middle School Soccer Field Soil Sample Locations 5-109
5-23 Community Park North Field Soil Sample Locations 5-110
5-24 Community Park South Field Soil Sample Locations 5-111
5-25 Community Park New York Creek Field Soil Sample Locations 5-112
5-26 Community Park Lower Soccer Field Soil Sample Locations 5-113
5-27 New York Creek Nature Trail Soil Sample Locations 5-114
5-28 Jackson Elementary School Garden and Outdoor Classroom Soil Sample Locations 5-115
5-29 Jackson Elementary School Grass-Covered Playing Field Soil Sample Locations 5-116
5-30 Dirt Embankment Soil Sample Locations 5-117
5-31 Dirt Parking Area Soil Sample Locations 5-118

US EPA ARCHIVE DOCUMENT

List of Acronyms and Abbreviations

AAMS	Ambient Air Monitoring Station
AHERA	Asbestos Hazard Emergency Response Act
cc	cubic centimeter
CARB	California Air Resources Board
CERCLA	Comprehensive Environmental Response, Compensation, and Liability Act
CERCLIS	Comprehensive Environmental Response, Compensation, and Liability Information System
CoEMD	El Dorado County Environmental Management Department
CSD	El Dorado Hills Community Services District
DQO	Data Quality Objective
DTSC	California Department of Toxic Substances Control
E & E	Ecology and Environment, Inc.
ERT	Emergency Response Team
f/cc	fibers per cubic centimeter
FSP	Field Sampling Plan
HRS	Hazard Ranking System
mg/m^3	milligrams per cubic meter
µm	micrometer or micron
NPL	National Priorities List
NRA	Northern Reference Area
PA	preliminary assessment
PA/SI	preliminary assessment/site inspection
PE	performance evaluation
PCM	phase contrast microscopy
PCME	phase contrast microscopy equivalents
PLM	polarized light microscopy
PPE	personal protective equipment
QA	quality assurance
QAO	Quality Assurance Office

US EPA ARCHIVE DOCUMENT

List of Acronyms and Abbreviations (Cont.)

QAPP	Quality Assurance Project Plan
QC	quality control
SARA	Superfund Amendments and Reauthorization Act
s/cc	structures per cubic centimeter
SI	site inspection
SOP	standard operating procedure
SOW	statement of work
SRA	Southern Reference Area
START	Superfund Technical Assessment and Response Team
STEL	short-term exposure limit
TEM	transmission electron microscopy
TWA	time-weighted average
U.S. EPA	U.S. Environmental Protection Agency

US EPA ARCHIVE DOCUMENT

Definitions

activity-based air sampling: Collecting air samples while engaging in dust generation activities (e.g., those that could disturb asbestos fibers and release them into the air).

ambient air: Generally, the surrounding air present throughout a vicinity. For the El Dorado Hills Naturally Occurring Asbestos Multimedia Exposure Assessment, ambient air is specifically defined as outdoor air (as opposed to indoor air) collected from the general vicinity of the various subject sites, and which may be used for reference samples. These samples may variably be upwind, downwind, or crosswind from locations that activity-based sampling scenarios are conducted, and they may or may not be influenced by sampling activities. In addition, some of the ambient air samples were collected under normal conditions (i.e., while no activity-based sampling was conducted).

amphibole: One of the two groups of minerals (serpentine and amphibole) that can crystallize as asbestos. The regulated asbestiform minerals of this group are crocidolite, amosite, anthophyllite asbestos, tremolite asbestos, and actinolite asbestos.

analytical sensitivity: The sample-specific lowest concentration of asbestos the laboratory can reliably detect.

asbestos: Asbestos is the generic name used for a group of naturally occurring mineral silicate fibers of the serpentine and amphibole series. Asbestos is composed of fiber bundles that are made up of extremely long and thin fibers that are easily separated from one another. For the purposes of this project, asbestos encompasses not only the six regulated varieties, but also the non-regulated asbestiform minerals.

asbestiform: Fibrous or tending to break into fibers.

aspect ratio: Length to width ratio.

US EPA ARCHIVE DOCUMENT

Definitions (Cont.)

breathing height: A height representing a typical height of a person's nose/mouth area.

chrysotile: A regulated mineral in the serpentine group of minerals that can crystallize as asbestos. Chrysotile is also known as serpentine asbestos.

fixed sample pump: An air sample pump whose position is constant throughout the entire duration of the sampling effort. A fixed sample pump remains in its fixed location on a long-term basis over a period longer than 1 day. Typically a high-flow sample pump is used where a fixed sample pump is needed.

high-flow sample pump: Also known as a high-volume sample pump, this is an air sample pump that is capable of drawing up to about 30 liters per minute of air. This type of sample pump is not generally portable and is typically used for sampling from fixed and stationary positions.

hi-vol: Shorthand for high-volume or high-flow (sample pump).

infield skin: The non-grass infield area of a baseball or softball field; also commonly referred to as infield "dirt" or "base pad."

levels of personal protection: When sampling is conducted where contamination may exist, personal protective equipment (PPE) must be worn to prevent or reduce skin and eye contact, inhalation, and ingestion of the substance. Protective equipment to protect the body against contact with known or anticipated chemical hazards has been divided into four categories known as Levels A, B, C, and D:

- **Level D** is primarily a work uniform and is used for nuisance contamination only. Level D generally includes basic work clothing with steel-toed and steel-shanked boots, and may include coveralls, a hard hat, gloves, ear plugs, and safety goggles.
- **Level C** protection is worn when the type of airborne substance is known, concentration measured, criteria for using air-purifying respirators (APR) met, and skin and eye exposure is unlikely. Level C generally includes everything used for Level D, with the addition of an APR or powered APR for inhalation protection.
- **Level B** protection is worn when the highest level of respiratory protection is needed, but a lesser level of skin and eye protection. Level B also generally includes everything

US EPA ARCHIVE DOCUMENT

used for Level D; and in addition includes appropriate chemical-resistant coveralls and gloves for dermal protection, and a full-faced mask and self-contained breathing apparatus (SCBA) or supplied air for eye protection and complete respiratory protection.

- **Level A** protection is worn when the highest level of respiratory, skin, eye and mucous membrane protection is needed. Level A protection includes a fully encapsulated suit for total skin, eye and mucous membrane protection and an SCBA for complete respiratory protection.

naturally occurring asbestos: Asbestos minerals that occur in rock and soil as the result of natural geologic processes, often in veins near earthquake faults in the coast ranges and the foothills of the Sierra Nevada mountains and other areas of California.

personal sample pump: Also known as a low-flow or low-volume sample pump, this is an air sample pump that is portable so that it can be worn by a member of the sampling team during activity-based sample collection. The air flow for a personal sample pump is typically 1 to 5 liters per minute.

phase contrast microscopy (PCM): A light-enhancing microscope technology that employs an optical mechanism to translate small variations in phase into corresponding changes in amplitude, resulting in high-contrast images. This method was used traditionally to measure airborne fibers in occupational environments; however, it cannot distinguish between asbestos fibers and other fibers.

phase contrast microscopy equivalent (PCME): This refers to asbestiform structures identified through transmission electron microscopy (TEM) analysis that are equivalent to those that would be identified in the same sample through phase contrast microscopy analysis, with the main difference being that TEM additionally permits the specific identification of asbestos fibers. PCME structures are asbestiform structures greater than 5 microns in length having at least a 3 to 1 length to width (aspect) ratio.

polarized light microscopy (PLM): A microscope technology that uses the polarity (or orientation) of light waves to provide better images than a standard optical microscope.

US EPA ARCHIVE DOCUMENT

Definitions (Cont.)

reference sample: An ambient air sample from outside the specific area of concern collected concurrently with the activity-based samples; it is used as a reference for comparison with the activity-based air samples.

stationary sample pump: An air sample pump that is placed in a single location and is not moved during a sampling event. A stationary sample pump remains in its stationary location during one or more sample events. Typically a high-flow sample pump will be used where a stationary sample pump is needed.

transmission electron microscopy (TEM): A microscope technology that uses the properties of electrons to provide more detailed images than even polarized light microscopy.

ultramafic rock: An igneous rock containing mainly dark, ferromagnesian minerals (i.e., greater than 90% of olivine, pyroxene, or hornblende). Commercial deposits of asbestos have been associated with ultramafic rocks.

US EPA ARCHIVE DOCUMENT

US EPA ARCHIVE DOCUMENT

1 Introduction

U.S. EPA
U.S. Environmental Protection Agency

CERCLA
Comprehensive Environmental Response, Compensation, and Liability Act of 1980

SARA
Superfund Amendments and Reauthorization Act of 1986

E & E
Ecology and Environment, Inc.

START
Superfund Technical Assessment and Response Team

PA/SI
preliminary assessment/site inspection

HRS
U.S. EPA's Hazard Ranking System

NPL
National Priorities List

The **U.S. Environmental Protection Agency** (U.S. EPA), Region IX, under the authority of the **Comprehensive Environmental Response, Compensation, and Liability Act of 1980** (CERCLA) and the **Superfund Amendments and Reauthorization Act of 1986** (SARA), tasked the **Ecology and Environment, Inc.,** (E & E) **Superfund Technical Assessment and Response Team** (START) to conduct a multimedia assessment of community areas and schools in El Dorado Hills in El Dorado County, California, to assess the potential for exposure from naturally occurring asbestos present in soils that have been disturbed. This **preliminary assessment** and **site inspection** (PA/SI) report identifies the subject area, describes the data-gathering activities that have been conducted to date, and summarizes the results of those activities.

As part of the PA/SI, the site is evaluated using U.S. EPA's **Hazard Ranking System** (HRS) criteria. The HRS assesses the relative threat associated with actual or potential releases of hazardous substances to the environment and has been adopted by the U.S. EPA to assist in setting priorities for further evaluation and eventual remedial action. The HRS is the primary method for determining a site's eligibility for placement on the **National Priorities List** (NPL). The NPL identifies sites where the U.S. EPA may conduct remedial response actions.

US EPA ARCHIVE DOCUMENT

2 Apparent Problem

ultramafic
A type of igneous rock containing mainly dark, ferromagnesian minerals

ambient air
The surrounding air present throughout a vicinity

DTSC
California Department of Toxic Substances Control

CARB
California Air Resources Board

Exposure risk from naturally occurring asbestos, particularly an exposure occurring as a result of construction activities, has been a concern in El Dorado County for some time. (See *Findings and Recommendations on Naturally-Occurring Asbestos to El Dorado County*, State of California Asbestos Task Force, March 11, 1999.) Naturally occurring asbestos is found in **ultramafic** rock formations in many locations in El Dorado County, California. In El Dorado Hills, the location of this assessment, asbestos is found in association with the West Bear Mountains Fault Zone, which runs north to south across El Dorado County.

In El Dorado Hills the presence of asbestos in exposed soil and **ambient air** has already been documented through previous investigations as well as visual inspection conducted by the U.S. EPA, the **California Department of Toxic Substances Control** (DTSC), the **California Air Resources Board** (CARB), and the El Dorado Union High School District. These previous investigations were conducted in a residential area on Woedee Drive and at Oak Ridge High School, located at 1120 Harvard Way. Mitigation activities to address asbestos contamination in disturbed soils on the campus of Oak Ridge High School have been conducted by El Dorado Union High School District, with oversight by El Dorado County and the state and by U.S. EPA.

PA
preliminary assessment

CERCLIS
Comprehensive Environmental Response, Compensation, and Liability Information System

In September 2003, a citizen petitioned U.S. EPA to conduct a **preliminary assessment** at the El Dorado Hills Community Park, Silva Valley Elementary School, Rolling Hills Middle School, and other locations in the community where the suspected presence of naturally occurring asbestos in exposed and disturbed soil may be causing releases to air. After review of the petition and discussions with the petitioner, the U.S. EPA defined the study area (called El Dorado Hills Naturally Occurring Asbestos) to include the El Dorado Hills Community Park, Silva Valley Elementary School, Rolling Hills Middle School, Jackson Elementary School, and the New York Creek Nature Trail. The U.S. EPA entered the El Dorado Hills Naturally Occurring Asbestos site into the **Comprehensive Environmental Response, Compensation, and Liability Information System** (CERCLIS) with U.S. EPA Identification Number CAN000906083 on April 7, 2004.

US EPA ARCHIVE DOCUMENT

US EPA ARCHIVE DOCUMENT

3 Site Description and History

3.1 SITE LOCATION

El Dorado Hills is approximately 20 miles east of Sacramento, California. The community is within an unincorporated area of El Dorado County that is commonly referred to as the Western County Region. With a current population of about 31,000, El Dorado Hills has become a "bedroom" community, with a growing number of residents commuting to work in Sacramento.

A number of areas throughout El Dorado Hills are the subject of the El Dorado Hills Naturally Occurring Asbestos Multimedia Exposure Assessment. These areas, addressed in this PA/SI report and shown on Figure 3-1 (Sites Location Map), include the following:

- The El Dorado Hills Community Park, including several play areas and the New York Creek Nature Trail;
- Silva Valley Elementary School;
- Jackson Elementary School;
- Rolling Hills Middle School, including the dirt embankment inside the school's eastern boundary (Dirt Embankment); and
- An unpaved lot used for parking on public property adjacent to and in front of Rolling Hills Middle School (Dirt Parking Area).

Figure 3-1

US EPA ARCHIVE DOCUMENT

3.2 SITE DESCRIPTION

El Dorado Hills Community Park and New York Creek Nature Trail. The El Dorado Hills Community Park at 1021 Harvard Way is situated on about 40 acres of property along El Dorado Hills Boulevard between Harvard Way and St. Andrews Drive (Latitude 38E 40' 59" North, Longitude 121E04' 28" West). The Community Park property, which is transected by New York Creek, contains three baseball diamonds, soccer playing fields, a children's playground, a swimming pool, community center structures, the southern end of the New York Creek Nature Trail, other picnic and recreational areas, and parking areas. The New York Creek Nature Trail is an unpaved trail adjacent to New York Creek. From Harvard Way, the trail runs north almost 2 miles through the Community Park property and residential neighborhoods to Art Weisberg Park, which is opposite Jackson Elementary School on Francisco Drive.

Silva Valley Elementary School. Silva Valley Elementary School is located at 3001 Golden Eagle Lane (Latitude 38E 40' 40" North, Longitude 121E04' 11" West). With about 650 students, it is a year-round K-5 elementary school in the Buckeye Union School District. Some of the school's facilities include six classroom buildings, a multipurpose room, an administration building, a library, a computer laboratory, a grass-covered playing field, a grass-infield baseball diamond, a "Life Lab" garden area, and play structures.

Jackson Elementary School. Jackson Elementary School is located at 2561 Francisco Drive (Latitude 38E 42' 14" North, Longitude 121E04' 51" West). With almost 500 students, it is a traditional K-5 elementary school in the Rescue Union School District. Some of the school's facilities include approximately 26

US EPA ARCHIVE DOCUMENT

classrooms (all carpeted), an administration building, a library, a computer laboratory, a grass-covered playing field with grass-infield baseball diamonds, an outdoor classroom and garden, two paved play areas with basketball courts and tetherball, and play structures. The outdoor classroom and garden area is a place where students participate in gardening activities as part of the educational program.

Rolling Hills Middle School and the Dirt Embankment. Rolling Hills Middle School is located at 7141 Silva Valley Parkway (Latitude 38E 40' 54" North, Longitude 121E04' 07" West). Built in its current location in 1998, the school is a year-round middle school (6th to 8th grades) with about 800 students. The school is part of the Buckeye Union School District. Some of the school's facilities include classrooms, an administration building, a library, a grass-covered soccer field, and a paved basketball play area. The Dirt Embankment is a dirt embankment/hillside behind Rolling Hills Middle School and inside its eastern boundary.

The Dirt Parking Area. The Dirt Parking Area is an unpaved lot used for parking on public property in front of Rolling Hills Middle School, outside its western boundary. The property is said to be used regularly as a parking lot primarily by high school students who attend the nearby Oak Ridge High School.

3.3 SITE HISTORY

CSD
El Dorado Hills Community Services District

On May 21, 1962, the El Dorado County Board of Supervisors adopted Resolution #98-62 creating the **El Dorado Hills Community Services District** (CSD). The CSD is governed by a five-member elected Board of Directors and is the primary provider of parks and recreation services to residents of El Dorado Hills, in addition to a variety of other community services.

US EPA ARCHIVE DOCUMENT

The population of El Dorado Hills has grown rapidly in the last two decades. For example, the population jumped 81 percent in ten years, from an estimated 12,105 people in 1991 to 21,917 people in 2001. According to the State Department of Finance, by August 2001 the population was 23,013, and in January 2005 the population was about 31,000. Prior to this expansive growth, much of the land in the community was undeveloped open space.

The El Dorado Hills Community Park is one of almost two dozen neighborhood and community parks provided by the El Dorado Hills CSD. The CSD main office is at the Community Park, and there are about 26 full-time and 8 part-time workers on staff there year-round.

The southern end of the New York Creek Nature Trail begins in the Community Park, and the northern end is in Art Weisberg Park near Jackson Elementary School. The trail, which is considered an educational opportunity for local schools, provides botanical settings and views of native wildlife within the approximately 28-acre New York Creek Nature Area.

El Dorado Hills CSD received part of the area that is now the New York Creek Nature Trail in 1972 as a gift deed. In 1986 the area was enlarged when an 11-acre parcel along New York Creek was conveyed to the CSD for use as an open space natural area. The New York Creek Stewardship Committee was formed to encourage local community involvement in the protection, conservation, care and use of New York Creek.

The Community Park grounds are maintained by both in-house and contracted maintenance workers who perform such duties as mowing lawns using a riding mower, grooming the baseball dia-

US EPA ARCHIVE DOCUMENT

mond infields using an electric maintenance cart (i.e., golf cart) and drag chains, and using a leaf blower to clear walkways and parking areas and to clean playground structures.

Silva Valley Elementary School and Rolling Hills Middle School are within the Buckeye Union School District. Built in 1992, Silva Valley Elementary School was once surrounded by empty fields, but new homes now flank the property. There are about 650 students currently enrolled at the year-round school, with about 29 teachers and 20 staff members, including maintenance staff. At Rolling Hills Middle School, which moved to its current permanent facilities in August 1998, there are about 800 students, 30 teachers, and 20 to 25 staff members, including maintenance staff. School district staff maintain the grounds at both schools, with the exception of mowing the playing fields, which is done by a contractor to the district.

The Dirt Parking Area, which sits adjacent to Rolling Hills Middle School, is apparently maintained by and under the jurisdiction of the El Dorado County Department of Transportation.

Jackson Elementary School, part of the Rescue Union School District, is a traditional elementary school. In the fall, there will be 22 teachers, about nine staff members, and about 470 students. The school grounds (along with the grounds of other schools in the district) are maintained by two district groundsmen; this number is expected to increase to three in June.

While Oak Ridge High School is not part of the El Dorado Hills Naturally Occurring Multimedia Assessment, it is centrally located between the majority of the locations that are included in the assessment, and it is in a geologically similar region. In February

US EPA ARCHIVE DOCUMENT

2002, construction began of two soccer fields along the southwest border of Oak Ridge High School. During construction, veins of asbestos-bearing minerals were disturbed.

CoEMD
El Dorado County Environmental Management Department

The El Dorado Union High School District reportedly encountered difficulties in acquiring reclaimed irrigation water for the project, so the soccer fields were left without landscaping for more than a year while a solution was sought. Subsequent erosion of disturbed, potentially asbestos-bearing soils from the unfinished fields caused by winter rains in 2002/2003 impacted classrooms and locker rooms downslope. In addition, the El Dorado Union High School District, in coordination with the **El Dorado County Environmental Management Department** (CoEMD) and DTSC, identified other areas of concern on the campus. This led the school district to undertake mitigation activities at Oak Ridge High School in the summer of 2003. See Section 4, Regulatory Involvement, for a brief discussion of activities subsequent to this mitigation effort.

US EPA ARCHIVE DOCUMENT

4 Regulatory Involvement

f/cc
fibers per cubic centimeter

The CARB has conducted air monitoring in several locations in California to determine levels of asbestos in air at selected sites. In April 1999, the CARB measured ambient asbestos concentrations in air at seven monitoring locations at and near Silva Valley Elementary School. Of the 20 samples collected at the school, four of the samples contained detectable levels of asbestos; the highest level detected (in two of the samples) was 0.0019 **fibers per cubic centimeter** (f/cc).

While there has been no other regulatory involvement to date related to the locations that are the subject of the El Dorado Hills Naturally Occurring Exposure Multimedia Assessment, the El Dorado Union High School District has been working with several agencies at the Federal, State and local levels to address the exposed asbestos at the nearby Oak Ridge High School. Mitigation actions undertaken by the school district are described in the *El Dorado Union High School District Oak Ridge High School Naturally Occurring Asbestos (NOA) Operations and Maintenance (O&M) Plan*, dated December 2003, prepared by MACTEC Federal Programs.

The CARB conducted air sampling in June and July 2003 to assess the type and quantity of asbestos fibers released to ambient air during mitigation activities at the soccer fields. The CARB sam-

US EPA ARCHIVE DOCUMENT

s/cc
structures per cubic centimeter

pling documented the presence of asbestos in ambient air samples collected during mitigation activities, with a maximum asbestos level recorded in air of 0.0039 **structures per cubic centimeter** (s/cc) and an average concentration at the mitigation fence line of 0.001 s/cc. A complete description of the CARB sampling locations, methodology and findings is available in the November 6, 2003, CARB report *Sampling for Airborne Naturally Occurring Asbestos at Oak Ridge High School June 2003*.

Due to citizens' concerns about asbestos on the Oak Ridge High School campus, the U.S. EPA and START conducted an assessment of surficial soil at the high school in November 2003 to determine whether additional mitigation efforts were required in areas other than those related to the soccer fields. During the assessment exposed soils throughout the campus were sampled, particularly those areas where observed or expected student or public traffic could disturb asbestos-containing soil or rock. Sampling documented that asbestos was present in exposed soils throughout the campus ranging from less than 0.0001 to 8.8 percent by weight. U.S. EPA subsequently performed remediation at Oak Ridge High School by covering exposed soil with landscaping, concrete, or pavement.

US EPA ARCHIVE DOCUMENT

activity-based air sampling
Collecting air samples while engaging in dust generation activities (e.g., those that could disturb asbestos fibers and release them into the air.)

personal air sampler
Also known as a low-flow or low-volume sample pump, this is an air sample pump that is portable so that it can be worn by a member of the sampling team during activity-based sample collection. The air flow for a personal sample pump is typically 1 to 5 liters per minute.

stationary air sampler
An air sample pump that is placed in a single location and is not moved during a sampling event. A stationary sample pump remains in its stationary location during one or more sample events. Typically a high-flow sample pump will be used where a stationary sample pump is needed.

fixed air sampler
An air sample pump whose position is constant throughout the entire duration of the sampling effort. A fixed sample pump remains in its fixed location on a long-term basis over a period longer than 1 day. Typically a high-flow sample pump is used where a fixed sample pump is needed.

ambient air
Generally, the surrounding air present throughout a vicinity.

reference sample
An ambient air sample from outside the specific area of concern collected concurrently with the activity-based samples; it is used as a reference for comparison with the activity-based air samples.

Summary of Investigative Efforts

5.1 OBJECTIVES

As part of the El Dorado Hills Naturally Occurring Asbestos Multimedia Exposure Assessment, the START collected outdoor air and soil samples to assist the U.S. EPA in identifying and estimating associated exposure levels for locations in El Dorado Hills where there is a potential for exposure to asbestos from disturbed areas of naturally occurring asbestos.

- The START collected **activity-based** outdoor air samples from the Community Park, the New York Creek Nature Trail, and three schools in El Dorado Hills. This was to document whether and at what concentrations asbestos fibers were present in outdoor air during activities conducted at sampled locations on the days of sampling. Activity-based outdoor air samples were collected under conditions ranging from minimal activity to dust generation activities while members of the sampling team wore **personal sample pumps**. In addition, **stationary air samplers** were set up in and around the activity areas during most of the activity-based sampling.
- The START also set up **fixed air samplers** at the Community Park and schools to collect **ambient** outdoor air samples collected from outside activity areas to serve as **reference** samples.

US EPA ARCHIVE DOCUMENT

infield skin
The non-grass infield area of a baseball or softball field; also commonly referred to as infield "dirt" or "base pad"

QAPP
Quality Assurance Project Plan

FSP
Field Sampling Plan

DQO
Data Quality Objective

SOW
Statement of Work

- The START collected surface soil samples at the Community Park, the Dirt Embankment, the Dirt Parking Area and the schools to document whether and at what concentrations asbestos fibers were present in soil at sampled locations. At the baseball playing fields at the Community Park, where the **infield skin** is imported material, the START also collected shallow subsurface soil samples from at and below the interface of the infield fill and the soil beneath (down to about 1½ to 2 feet below ground surface).

5.2 SCOPE OF WORK

The U.S. EPA directed the START to develop a **Quality Assurance Project Plan** (QAPP) and **Field Sampling Plans** (FSPs), conduct ambient outdoor air sampling, activity-based outdoor air sampling, dust monitoring, soil sampling, meteorological data collection, video monitoring of fugitive dust present during outdoor air sampling, and limited video documentation of the soil sampling. The START assisted the U.S. EPA with the project objectives planning, including the development of data quality related objectives using the U.S. EPA's **Data Quality Objective** (DQO) planning process. The START developed the *El Dorado Hills Naturally Occurring Asbestos, Multimedia Exposure Assessment, El Dorado Hills, California, Quality Assurance Project Plan* based upon the DQO planning process and developed supporting FSP and analytical **Statement of Work** (SOW) documents.

The START conducted ambient and activity-based air sampling for asbestos fibers in outdoor air, real-time air monitoring to measure total dust, soil sampling for asbestos fibers, meteorological data collection, and video monitoring to document dust generation and sampling. For logistical reasons, the START did not conduct video monitoring of the soil sampling effort, but photographed

US EPA ARCHIVE DOCUMENT

many of the locations of soil samples collected for asbestos analysis. The START procured subcontractors as needed to aid in the collection of the air samples, preparation and analysis of samples, and validation of generated data. The U.S. EPA **Quality Assurance Office** (QAO) is over-seeing the data validation of the analytical results.

QAO
Quality Assurance Office

ERT
U.S. EPA's Environmental Response Team

The START is using the Scribe data management system to manage asbestos and meteorological data generated as part of the project. Scribe is a software tool developed by the U.S. EPA's **Environmental Response Team** (ERT) to assist in the process of managing environmental data.

5.3 SCHEDULE

Outdoor air sampling activities began in late September 2004. The START conducted ambient air reference sampling between September 27 and October 12, 2004. The activity-based air sampling took place between October 2 and October 10, 2004. The START collected soil samples for asbestos analysis between October 8 and October 11, 2004.

5.4 AIR SAMPLING

5.4.1 Ambient Outdoor Air Sampling

The START conducted ambient outdoor air sampling according to the *El Dorado Hills Naturally Occurring Asbestos Multimedia Exposure Assessment, El Dorado Hills, California, Fixed Ambient Outdoor Reference Air Sampling Field Sampling Plan* and the *El Dorado Hills Naturally Occurring Asbestos Multimedia Exposure Assessment, El Dorado Hills, California, Activity-Based Outdoor Air Sampling of Community Park and Schools Field Sampling Plan*.

US EPA ARCHIVE DOCUMENT

The START collected fixed ambient outdoor reference air samples from locations that were selected so as to collect from areas nearby but primarily outside of the influence of activity-based outdoor air sampling activities. These fixed ambient outdoor reference air sampling locations were in the following areas:

- Five locations, referred to as the Southern Reference Area, at the El Dorado Hills Community Park, Silva Valley Elementary School, and Rolling Hills Middle School, and
- Five locations, referred to as the Northern Reference Area, around Jackson Elementary School.

In addition to these reference air sampling locations, the START placed one fixed ambient outdoor air sampler co-located with a temporary meteorological station positioned in a secure location west of the children's playground at the El Dorado Hills Community Park. This location is referred to as the Ambient Air Monitoring Station.

Finally, along the New York Creek Nature Trail, the START placed stationary air samplers to conduct ambient outdoor air monitoring on two days when there was public activity on the trail but no activity-based sampling conducted there.

Except for the Ambient Air Monitoring Station (which was enclosed by a cyclone fence), the air sample pumps used for ambient outdoor air sampling generally were in locations that had public access. To ensure the integrity of the samples, either a member of the sampling team or a security guard watched the air sample pumps while they were operating. The START also conducted regular checks throughout the day to make sure the pumps were operating and the power source was adequate and continuous.

US EPA ARCHIVE DOCUMENT

5.4.1.1 Fixed Ambient Air Sampling at the Community Park

Beginning on September 27, several days prior to conducting activity-based outdoor air sampling, the START positioned and began operating a temporary meteorological station, a high-volume (i.e., high-flow, approximately 10 liters/minute) air sample pump, and a dust monitor within an existing small fenced area west of the children's playground at the El Dorado Hills Community Park. The location of this area is shown as the Ambient Air Meteorological Station (AAMS) on Figure 5-1 (Fixed Ambient Outdoor Air Sampling Locations). The START operated the meteorological station, the air sample pump, and the dust monitor at this location daily through October 12.

The meteorological station was set up as a base station for the project to monitor wind speed, wind direction, humidity, temperature, and other meteorological conditions. Due to equipment malfunction, the electronic set of data collected from the meteorological base station was lost for the period from October 5 through October 8, 2004; however the START did collect some of the meteorological data for this period through manual periodic logging. In addition, a second mobile meteorological station was used to collect meteorological data during some of the activity-based air sampling scenarios that were conducted during this period. The weather during the week leading up to the sampling was generally dry, and it was dry during most of the first week of sampling, but it drizzled on October 9, 2004. See Appendix A for a summary of meteorological data collected from the base station and from the mobile station.

The START collected air samples on air filters from the Ambient Air Meteorological Station fixed air sampler each day over an approximately 8-hour time interval. The daily time interval gener-

US EPA ARCHIVE DOCUMENT

Figure 5-1

US EPA ARCHIVE DOCUMENT

breathing height
A height representing a typical height of a person's nose/mouth area

ally coincided with activity-based scenario sampling time periods. The primary focus of the investigative efforts is the potential for exposure of asbestos to children, so the intake of the air collection filter cassette at this location was positioned at a height of about 3 feet above the ground surface to represent the **breathing height** of a child. A summary of results for the ambient air samples from this monitoring station is shown in Table 5-1 (Ambient Air Monitoring Station Air Sample Summary Results).

The dust monitor, used to measure total dust, was positioned at the same height as the air collection filter cassette. See Appendix B for a summary of the results from the dust monitor from this location and from other dust monitors used during sampling.

5.4.1.2 Fixed Ambient Air Sampling for Activity-Based Reference Level Determinations

<u>Southern Reference Area at the El Dorado Hills Community Park, Silva Valley Elementary School, and Rolling Hills Middle School</u>
One day prior to starting the activity-based outdoor air sampling at the El Dorado Hills Community Park, Silva Valley Elementary School, and Rolling Hills Middle School, the START positioned and began operating five high-flow air sample pumps at the park and the two schools. The five fixed ambient outdoor reference air sample locations, which are shown as the Southern Reference Area on Figure 5-1 (Fixed Ambient Outdoor Air Sampling Locations), are the following:

SRA-R01	Community Park, west of main office
SRA-R02	Community Park courtyard between main office and pool area
SRA-R03	Rolling Hills Middle School at the southern end of the campus

US EPA ARCHIVE DOCUMENT

Table 5-1
Ambient Air Monitoring Station Air Sample Summary Results

Sample ID	Date	PCME Structures (s/cc)	AHERA-like Total Structures (s/cc)	Sensitivity (s/cc)
AAMS-D01-092704	9/27/04	0.00115	0.00172	0.000286
AAMS-D02-092804	9/28/04	<0.000872	0.00117	0.000292
AAMS-D03-092904	9/29/04	0.000570	0.000570	0.000285
AAMS-D04-093005	9/30/04	0.000860	0.00229	0.000287
AAMS-D05-100104	10/01/04	0.00197	0.00282	0.000282
AAMS-D06-100204	10/02/04	0.000582	0.00146	0.000291
AAMS-D07-100304	10/03/04	0.000306	0.000611	0.000306
AAMS-D107-100304[1]	10/03/04	0.000613	0.00245	0.000307
AAMS-D08-100404	10/04/04	<0.000871	<0.000871	0.000291
AAMS-D09-100504	10/05/04	results pending	results pending	results pending
AAMS-D10-100604	10/06/04	results pending	results pending	results pending
AAMS-D11-100704	10/07/04	0.000580	0.00174	0.000290
AAMS-D12-100804	10/08/04	0.000290	0.00232	0.000290
AAMS-D13-100904	10/09/04	<0.000851	<0.000851	0.000285
AAMS-D14-101004	10/10/04	0.000583	0.00612	0.000292
AAMS-D15-101104	10/11/04	<0.000894	0.00239	0.000299
AAMS-D16-101204	10/12/04	results pending	results pending	results pending
AAMS-1ZB-092904[2]	9/29/04	<0.000871	<0.000871	0.000291
AAMS-1ZB-100204[2]	10/02/04	<0.000871	<0.000871	0.000291
AAMS-2ZB-100204[2]	10/02/04	<0.000869	<0.000869	0.000291
AAMS-FB-093004[3]	9/30/04	<0.000974	<0.000974	0.000326

PCME fibers = fibers longer than 5 microns with a width between 0.25 and 3 microns, and an aspect ratio (length to width) greater than 3:1
AHERA-like total structures = structures longer than 0.5 microns with an aspect ratio greater than 3:1 (Note this differs somewhat from the strict AHERA structure definition)
Sensitivity = the sample-specific lowest concentration of asbestos the laboratory can reliably detect
s/cc = structures per cubic centimeter
[1] AAMS-D107-100304 is a duplicate sample for (i.e., co-located with) AAMS-D07-100304
[2] field blank sample
[3] filter blank sample

US EPA ARCHIVE DOCUMENT

SRA-R04	Silva Valley Elementary School at the southwestern corner of the campus
SRA-R05	On Community Park property in open space west of the North Field baseball playing field

Location SRA-R01 was originally sited close to El Dorado Hills Boulevard under the flag pole (SRA-R01a). The location was later changed (October 4, 2004) to a spot closer to the main office (SRA-R01b) due to problems obtaining electrical power to operate the air sample pump at that location and because park personnel wanted to remove the turf covering the driveway that passes through that area since it had become muddy and posed a hazard to drivers.

The START collected air samples on air filters from these fixed locations in the Southern Reference Area daily over an approximately 8-hour time interval one day prior to, each day during, and the day after the activity-based outdoor air sampling was conducted at the Community Park (including along the New York Creek Nature Trail) and the southern two schools. The daily time interval generally coincided with the activity-based scenario sampling time periods at the park and schools. The intakes of the air collection filter cassettes were positioned at a height of about 3 feet above the ground surface. This height was selected so as to be the same as that of the air collection filter cassette intakes that were used during activity-based sampling to represent the breathing height of a child. A dust monitor was co-located with the air sample pump at Rolling Hills Middle School at the southern end of the campus (SRA-R03), and total dust concentrations were monitored. The dust monitor was positioned at the same height as the air collection filter cassette. A summary of results for the Southern Reference Area samples is shown in Table 5-2 (Southern Reference Area Air

US EPA ARCHIVE DOCUMENT

Sample Summary Results). See Appendix B for a summary of the results from the dust monitor from these locations.

<u>Northern Reference Area Around Jackson Elementary School</u>

One day prior to conducting activity-based outdoor air sampling at Jackson Elementary School, the START positioned five high-flow air sample pumps at five locations in the vicinity of the school. The five fixed ambient outdoor reference air sample locations, which are shown as the Northern Reference Area on Figure 5-1 (Fixed Ambient Outdoor Air Sampling Locations), are the following:

NRA-R01	St. Andrews Park
NRA-R02	Art Weisberg Park, on the east side of the park in a grassy area among the trees
NRA-R03	Art Weisberg Park, on the west side of the park in an open grassy area next to Pendleton Drive
NRA-R04	Adjacent to the walkway that joins the nearby residential neighborhood to the northern end of the New York Creek Nature Trail
NRA-R05	On the median of El Dorado Hills Boulevard just north of Campbell Ranch Drive

The START collected air samples on air filters from these fixed locations daily over an approximately 8-hour time interval one day prior to, the day of, and the day after the activity-based outdoor air sampling was conducted at Jackson Elementary School. The daily time interval generally coincided with the activity-based scenario sampling time periods at the school. The intakes of the air collection filter cassettes were positioned at a height of about 3 feet above the ground surface. This height was selected so as to be the same as that of the air collection filter cassette intakes that were used during activity-based sampling to represent the breathing

US EPA ARCHIVE DOCUMENT

US EPA ARCHIVE DOCUMENT

Table 5-2
Southern Reference Area Air Sample Summary Results

Sample ID	Location	Location Description	Date	PCME Structures (s/cc)	AHERA-like Total Structures (s/cc)	Sensitivity (s/cc)
SRA-R01-100104	SRA-R01	Community Park, west of main office	10/01/04	0.000859	0.00229	0.000286
SRA-R02-100104	SRA-R02	Community Park courtyard between main office and pool area	10/01/04	0.00198	0.00368	0.000283
SRA-R03-100104	SRA-R03	Rolling Hills Middle School at the southern end of the campus	10/01/04	0.000269	0.00188	0.000269
SRA-R04-100104	SRA-R04	Silva Valley Elementary School at the southwestern corner of the campus	10/01/04	0.000870	0.00377	0.000290
SRA-R05-100104	SRA-R05	On Community Park property in open space west of the North Field baseball playing field	10/01/04	0.00113	0.00113	0.000283
SRA-R01-100204	SRA-R01	Community Park, west of main office	10/02/04	0.000289	0.00375	0.000289
SRA-R101-100204	SRA-R01	Community Park, west of main office	10/02/04	<0.000853	0.000856	0.000285
SRA-R02-100204	SRA-R02	Community Park courtyard between main office and pool area	10/02/04	0.000570	0.00256	0.000285
SRA-R03-100204	SRA-R03	Rolling Hills Middle School at the southern end of the campus	10/02/04	0.000287	0.00115	0.000287
SRA-R04-100204	SRA-R04	Silva Valley Elementary School at the southwestern corner of the campus	10/02/04	0.000583	0.00204	0.000292
SRA-R05-100204	SRA-R05	On Community Park property in open space west of the North Field baseball playing field	10/02/04	0.000582	0.00233	0.000291
SRA-R01-100304	SRA-R01	Community Park, west of main office	10/03/04	<0.000895	0.00180	0.000299

US EPA ARCHIVE DOCUMENT

Table 5-2
Southern Reference Area Air Sample Summary Results

Sample ID	Location	Location Description	Date	PCME Structures (s/cc)	AHERA-like Total Structures (s/cc)	Sensitivity (s/cc)
SRA-R02-100304	SRA-R02	Community Park courtyard between main office and pool area	10/03/04	<0.000894	0.000897	0.000299
SRA-R03-100304	SRA-R03	Rolling Hills Middle School at the southern end of the campus	10/03/04	<0.000840	0.000281	0.000281
SRA-R04-100304	SRA-R04	Silva Valley Elementary School at the southwestern corner of the campus	10/03/04	0.000584	0.00234	0.000292
SRA-R05-100304	SRA-R05	On Community Park property in open space west of the North Field baseball playing field	10/03/04	<0.000891	0.000894	0.000298
SRA-R01-100404	SRA-R01	Community Park, west of main office	10/04/04	<0.000882	0.000885	0.000295
SRA-R02-100404	SRA-R02	Community Park courtyard between main office and pool area	10/04/04	0.000589	0.00118	0.000294
SRA-R03-100404	SRA-R03	Rolling Hills Middle School at the southern end of the campus	10/04/04	<0.000894	0.000897	0.000299
SRA-R04-100404	SRA-R04	Silva Valley Elementary School at the southwestern corner of the campus	10/04/04	0.000290	0.00290	0.000290
SRA-R05-100404	SRA-R05	On Community Park property in open space west of the North Field baseball playing field	10/04/04	<0.000888	0.000891	0.000297
SRA-R01-100504	SRA-R01	Community Park, west of main office	10/05/04	0.000889	0.00356	0.000296
SRA-R02-100504	SRA-R02	Community Park courtyard between main office and pool area	10/05/04	0.000585	0.00205	0.000292

Table 5-2
Southern Reference Area Air Sample Summary Results

Sample ID	Location	Location Description	Date	PCME Structures (s/cc)	AHERA-like Total Structures (s/cc)	Sensitivity (s/cc)
SRA-R103-100504	SRA-R03	Rolling Hills Middle School at the southern end of the campus	10/05/04	0.000863	0.00259	0.000288
SRA-R03-100504	SRA-R03	Rolling Hills Middle School at the southern end of the campus	10/05/04	0.000289	0.00173	0.000289
SRA-R04-100504	SRA-R04	Silva Valley Elementary School at the southwestern corner of the campus	10/05/04	<0.000888	0.00267	0.000297
SRA-R05-100504	SRA-R05	On Community Park property in open space west of the North Field baseball playing field	10/05/04	0.000291	0.00204	0.000291
SRA-R01-100604	SRA-R01	Community Park, west of main office	10/06/04	0.00440	0.00586	0.000293
SRA-R02-100604	SRA-R02	Community Park courtyard between main office and pool area	10/06/04	<0.000851	0.00285	0.000285
SRA-R102-100604	SRA-R02	Community Park courtyard between main office and pool area	10/06/04	0.00115	0.00288	0.000288
SRA-R03-100604	SRA-R03	Rolling Hills Middle School at the southern end of the campus	10/06/04	0.00145	0.00347	0.000290
SRA-R04-100604	SRA-R04	Silva Valley Elementary School at the southwestern corner of the campus	10/06/04	0.000907	0.00574	0.000302
SRA-R05-100604	SRA-R05	On Community Park property in open space west of the North Field baseball playing field	10/06/04	0.00177	0.00325	0.000296

US EPA ARCHIVE DOCUMENT

US EPA ARCHIVE DOCUMENT

Table 5-2
Southern Reference Area Air Sample Summary Results

Sample ID	Location	Location Description	Date	PCME Structures (s/cc)	AHERA-like Total Structures (s/cc)	Sensitivity (s/cc)
SRA-R01-100704	SRA-R01	Community Park, west of main office	10/07/04	results pending	results pending	results pending
SRA-R02-100704	SRA-R02	Community Park courtyard between main office and pool area	10/07/04	0.000575	0.00402	0.000287
SRA-R03-100704	SRA-R03	Rolling Hills Middle School at the southern end of the campus	10/07/04	<0.000844	0.000282	0.000282
SRA-R04-100704	SRA-R04	Silva Valley Elementary School at the southwestern corner of the campus	10/07/04	0.000866	0.00115	0.000289
SRA-R05-100704	SRA-R05	On Community Park property in open space west of the North Field baseball playing field	10/07/04	0.000285	0.000856	0.000285
SRA-R01-100804	SRA-R01	Community Park, west of main office	10/08/04	<0.000883	0.00118	0.000295
SRA-R02-100804	SRA-R02	Community Park courtyard between main office and pool area	10/08/04	0.000577	0.00289	0.000289
SRA-R03-100804	SRA-R03	Rolling Hills Middle School at the southern end of the campus	10/08/04	<0.000883	0.000590	0.000295
SRA-R103-100804	SRA-R03	Rolling Hills Middle School at the southern end of the campus	10/08/04	0.000295	0.00412	0.000295
SRA-R04-100804	SRA-R04	Silva Valley Elementary School at the southwestern corner of the campus	10/08/04	0.000564	0.00169	0.000282

US EPA ARCHIVE DOCUMENT

Table 5-2
Southern Reference Area Air Sample Summary Results

Sample ID	Location	Location Description	Date	PCME Structures (s/cc)	AHERA-like Total Structures (s/cc)	Sensitivity (s/cc)
SRA-R05-100804	SRA-R05	On Community Park property in open space west of the North Field baseball playing field	10/08/04	0.000877	0.00205	0.000292
SRA-1ZB-100804	field blank	field blank	10/08/04	<0.000853	<0.000853	0.000285
SRA-2ZB-100804	field blank	field blank	10/08/04	<0.000846	<0.000846	0.000283

PCME fibers = fibers longer than 5 microns with a width between 0.25 and 3 microns, and an aspect ratio (length to width) greater than 3:1
AHERA-like total structures = structures longer than 0.5 microns with an aspect ratio greater than 3:1 (Note this differs somewhat from the strict AHERA structure definition)
Sensitivity = the sample-specific lowest concentration of asbestos the laboratory can reliably detect
s/cc = structures per cubic centimeter

height of a child. A dust monitor was co-located with the air sample pump at Art Weisberg Park, on the west side of the park in an open grassy area next to Pendleton Drive (NRA-R03), and total dust concentrations were monitored. The dust monitor was positioned at the same height as the air collection filter cassette. A summary of results for the Northern Reference Area samples is shown in Table 5-3 (Northern Reference Area Air Sample Summary Results). See Appendix B for a summary of the results from the dust monitor from these locations.

5.4.1.3 Perimeter Monitoring on the New York Creek Nature Trail

On October 3 and October 9, 2004, the START placed five high-flow air sample pumps along the New York Creek Nature Trail to collect ambient outdoor air samples during those two days. The START was not conducting activity-based air sampling in the immediate vicinity on those two days, but members of the public were active in the park and on the trail. On October 3, a dog-walk event was held in the park, and on October 9 the trail was open to normal activity for a Saturday.

The locations of the five stationary sample pumps that were operated on the trail on October 3 are shown as CC2-1CT, CC2-2CT, CC2-3CT, CC2-4CT, and CC2-5CT, and the locations of the five sample pumps that operated on October 9 are shown as TRA-1TR/TRA11TR, TRA-2TR, TRA-3TR, TRA-4TR, and TRA-5TR on Figure 5-2 (New York Creek Nature Trail Perimeter Monitoring Locations). The START collected air samples on air filters from these stationary locations over an approximately 8-hour time interval on these two days. The intakes of the air collection filter cassettes in stationary locations along the trail were

US EPA ARCHIVE DOCUMENT

US EPA ARCHIVE DOCUMENT

Table 5-3
Northern Reference Area Air Sample Summary Results

Sample ID	Location	Location Description	Date	PCME Structures (s/cc)	AHERA-like Total Structures (s/cc)	Sensitivity (s/cc)
NRA-R01-100904	NRA-R01	St. Andrews Park	10/09/04	<0.000854	<0.000854	0.000286
NRA-R02-100904	NRA-R02	Art Weisberg Park, on the east side of the park in a grassy area among the trees	10/09/04	<0.000879	0.00118	0.000294
NRA-R03-100904	NRA-R03	Art Weisberg Park, on the west side of the park in an open grassy area next to Pendleton Drive	10/09/04	<0.000880	<0.000880	0.000294
NRA-R04-100904	NRA-R04	Adjacent to the walkway that joins the nearby residential neighborhood to the northern end of the New York Creek Nature Trail	10/09/04	<0.000866	0.00145	0.000289
NRA-R05-100904	NRA-R05	On the median of El Dorado Hills Boulevard just north of Campbell Ranch Drive	10/09/04	0.000582	0.00204	0.000291
NRA-R01-101004	NRA-R01	St. Andrews Park	10/10/04	0.00114	0.00142	0.000285
NRA-R101-101004	NRA-R01	St. Andrews Park	10/10/04	0.000593	0.00119	0.000297
NRA-R02-101004	NRA-R02	Art Weisberg Park, on the east side of the park in a grassy area among the trees	10/10/04	0.00146	0.00205	0.000292
NRA-R03-101004	NRA-R03	Art Weisberg Park, on the west side of the park in an open grassy area next to Pendleton Drive	10/10/04	0.000294	0.00529	0.000294
NRA-R04-101004	NRA-R04	Adjacent to the walkway that joins the nearby residential neighborhood to the northern end of the New York Creek Nature Trail	10/10/04	0.000284	0.00114	0.000284
NRA-R05-101004	NRA-R05	On the median of El Dorado Hills Boulevard just north of Campbell Ranch Drive	10/10/04	0.000876	0.00233	0.000292

US EPA ARCHIVE DOCUMENT

Table 5-3
Northern Reference Area Air Sample Summary Results

Sample ID	Location	Location Description	Date	PCME Structures (s/cc)	AHERA-like Total Structures (s/cc)	Sensitivity (s/cc)
NRA-R01-101104	NRA-R01	St. Andrews Park	10/11/04	0.000578	0.00116	0.000289
NRA-R02-101104	NRA-R02	Art Weisberg Park, on the east side of the park in a grassy area among the trees	10/11/04	0.000855	0.00257	0.000285
NRA-R03-101104	NRA-R03	Art Weisberg Park, on the west side of the park in an open grassy area next to Pendleton Drive	10/11/04	0.000891	0.00624	0.000297
NRA-R04-101104	NRA-R04	Adjacent to the walkway that joins the nearby residential neighborhood to the northern end of the New York Creek Nature Trail	10/11/04	0.00177	0.00354	0.000295
NRA-R05-101104	NRA-R05	On the median of El Dorado Hills Boulevard just north of Campbell Ranch Drive	10/11/04	<0.000860	0.00115	0.000288
NRA-1ZB-101104	field blank	field blank	10/11/04	<0.000905	<0.000905	0.000303
NRA-2ZB-101104	field blank	field blank	10/11/04	<0.000929	<0.000929	0.000311
NRA-1ZB-101204	field blank	field blank	10/12/04	<0.000905	<0.000905	0.000303

PCME fibers = fibers longer than 5 microns with a width between 0.25 and 3 microns, and an aspect ratio (length to width) greater than 3:1
AHERA-like total structures = structures longer than 0.5 microns with an aspect ratio greater than 3:1 (Note this differs somewhat from the strict AHERA structure definition)
Sensitivity = the sample-specific lowest concentration of asbestos the laboratory can reliably detect
s/cc = structures per cubic centimeter

Figure 5-2

US EPA ARCHIVE DOCUMENT

positioned at a height of about 5 feet above the ground surface to represent the breathing height of an adult. A summary of results for the trail perimeter samples is shown in Table 5-4 (New York Creek Nature Trail Perimeter Monitoring Air Sample Summary Results).

Dust monitors were co-located with two of the air sample pumps (CC2-1CT and CC2-3CT) on October 3 and with all five air sample pumps on October 9. These dust monitors measured total dust concentrations along the trail. The dust monitors were positioned at the same height as the air collection filter cassettes. See Appendix B for a summary of the results from the dust monitor from these locations.

5.4.2 Activity-Based Air Sampling

The START conducted activity-based air sampling according to the *El Dorado Hills Naturally Occurring Asbestos Multimedia Exposure Assessment, El Dorado Hills, California, Activity-Based Outdoor Air Sampling of Community Park and Schools Field Sampling Plan* and the *El Dorado Hills Naturally Occurring Asbestos Multimedia Exposure Assessment, El Dorado Hills, California, Activity-Based Outdoor Air Sampling of Community Park Children's Playground Field Sampling Plan*. The types of activities the START conducted during activity-based outdoor air sampling included simulating work and play on baseball fields, soccer fields, basketball courts, playgrounds, and a hiking trail. Changes to these plans, if any, are noted in the discussion below.

The START conducted activity-based outdoor air sample collection at the following locations during 19 different activity-based sampling events or scenarios. These areas are illustrated on Figure 5-3 (Scenarios Location Map):

US EPA ARCHIVE DOCUMENT

Table 5-4
New York Creek Nature Trail Perimeter Monitoring Air Sample Summary Results

Sample ID	Location	Location Description	Date	PCME Structures (s/cc)	AHERA-like Total Structures (s/cc)	Sensitivity (s/cc)
CC2-H8-1CT-100304	CC2-1CT	trail position #1CT hi-vol	10/03/04	0.000883	0.00353	0.000294
CC2-H8-2CT-100304	CC2-2CT	trail position #2CT hi-vol	10/03/04	0.000583	0.00321	0.000291
CC2-H8-3CT-100304	CC2-3CT	trail position #3CT hi-vol	10/03/04	<0.000892	0.00328	0.000298
CC2-H8-4CT-100304	CC2-4CT	trail position #4CT hi-vol	10/03/04	0.000830	0.00277	0.000277
CC2-H8-5CT-100304	CC2-5CT	trail position #5CT hi-vol	10/03/04	0.000955	0.00573	0.000955
TRA-H8-1TR-100904	TRA-1TRA	trail position #TRA1 hi-vol	10/09/04	0.000294	0.00118	0.000294
TRA-H8-11TR-100904	TRA-1TRA	duplicate of trail position #TRA1 hi-vol	10/09/04	<0.000867	<0.000867	0.000290
TRA-H8-2TR-100904	TRA-2TRA	trail position #TRA2 hi-vol	10/09/04	0.000286	0.000286	0.000286
TRA-H8-3TR-100904	TRA-3TRA	trail position #TRA3 hi-vol	10/09/04	<0.000881	<0.000881	0.000295
TRA-H8-4TR-100904	TRA-4TRA	trail position #TRA4 hi-vol	10/09/04	<0.000879	0.000879	0.000294
TRA-H8-5TR-100904	TRA-5TRA	trail position #TRA5 hi-vol	10/09/04	<0.000875	<0.000875	0.000293
TRA-H2-1ZP-100904	performance evaluation	performance evaluation	10/09/04	0.0380	0.313	0.00316
TRA-L2-1ZP-100904	performance evaluation	performance evaluation	10/09/04	0.252	1.21	0.0126

PCME fibers = fibers longer than 5 microns with a width between 0.25 and 3 microns, and an aspect ratio (length to width) greater than 3:1
AHERA-like total structures = structures longer than 0.5 microns with an aspect ratio greater than 3:1 (Note this differs somewhat from the strict AHERA structure definition)
Sensitivity = the sample-specific lowest concentration of asbestos the laboratory can reliably detect
s/cc = structures per cubic centimeter

US EPA ARCHIVE DOCUMENT

- The New York Creek Nature Trail;
- The New York Creek baseball playing field at the Community Park;
- The north baseball playing field at the Community Park;
- The south baseball playing field at the Community Park;
- The (lower) soccer playing field between the north and south baseball playing fields at the Community Park;
- The children's playground at the Community Park;
- The baseball playing field at Silva Valley Elementary School;
- The basketball court area at Rolling Hills Middle School;
- The soccer playing field at Rolling Hills Middle School;
- Playing fields and paved play areas at Jackson Elementary School; and
- The garden and outdoor classroom at Jackson Elementary School.

Level C personal protective equipment
Protective equipment to protect the body against contact with known or anticipated chemical hazards has been divided into four categories known as Levels A, B, C, and D. Level C generally includes the use of an air purifying respirator for inhalation protection.

Members of the sampling team wore personal air monitoring pumps and **Level C personal protective equipment** while conducting activities within scripted scenarios to collect samples on air collection filter cassettes. The majority of the scenarios were designed to simulate primarily the activities of children, with simulation of adult activities as a small component. Some of the scenarios included simulation of only child activities, and some scenarios included simulation of only adult activities. Air collection filter cassettes were placed at a height of about 3 feet above ground surface to represent the breathing height of a child and about 5 feet above ground surface to represent the breathing height of an adult.

At each of the areas of concern the sampling team conducted the scenarios for the duration of the event, engaging in dust generation

US EPA ARCHIVE DOCUMENT

Figure 5-3

US EPA ARCHIVE DOCUMENT

activities that could disturb asbestos fibers and release them into the air. The level of activity ranged from minimal to aggressive dust generation. Except as noted, the sampling team conducted each scenario for 2 hours. For all baseball and soccer scenarios, members of the sampling team who participated as players wore baseball cleats.

Activities within each scenario area were conducted by a specific number of people (either five, six, or seven, depending on the activity). When a member of the sampling team took a break from the activity, another member of the sampling team took his or her place in the scenario so that the number of people conducting scenario activities remained constant. The sample pumps and dust monitors were carried in removable work belts to facilitate the exchange of sampling team members as needed.

In addition to the personal air sample pumps worn by members of the sampling team, and except as noted, the START positioned several stationary air sample pumps within each scenario activity and upwind and downwind of scenario activity. For activity-based sampling scenarios conducted at the baseball playing fields at the Community Park, the START also positioned stationary sampling pumps at the Children's Playground at the Community Park. The intakes for the air collection filter cassettes of the stationary sample pumps were set at a height of about 3 feet, except as noted.

Field conditions required the START to make several changes to the FSPs, most of which are related to the sampling schedule. Table 5-5 (Final Schedule of Field Work) shows the final sampling schedule as conducted.

US EPA ARCHIVE DOCUMENT

US EPA ARCHIVE DOCUMENT

Table 5-5 Final Schedule of Field Work

Date	Scenario 1	Scenario 2	Scenario 3	Reference Sample Areas
9/27/2005				Ambient Air Monitoring Station (AAMS)
9/28/2005				AAMS
9/29/2005				AAMS
9/30/2005				AAMS
10/1/2005	Rehearsal All Day and Press Availability 1:00 pm to 3:00 pm			AAMS, Southern Reference Area
10/2/2005	Silva Valley Baseball Field Maintenance	Silva Valley Baseball (A)		AAMS, Southern Reference Area
10/3/2005	Silva Valley Baseball (B)	Rolling Hills Soccer	Rolling Hills Basketball	AAMS, Southern Reference Area
	New York Creek Nature Trail 8-Hour Perimeter Sampling (CC2-#CT)			
10/4/2005	Community Park Children's Playground - Typical Scenario	Community Park Children's Playground - Aggressive Scenario		AAMS, Southern Reference Area
10/5/2005	New York Creek Nature Trail Biking	Community Park Baseball North Field	Community Park Baseball South Field (A)	AAMS, Southern Reference Area
10/6/2005	New York Creek Nature Trail Jogging (A)	Community Park Baseball South Field (B)	Community Park Baseball South Field–without maintenance (C)	AAMS, Southern Reference Area
10/7/2005	New York Creek Nature Trail Jogging (B)	Community Park Baseball New York Creek Field	Community Park Soccer Lower Field	AAMS, Southern Reference Area
10/8/2005	Soil Sampling - Community Park, New York Creek Nature Trail			AAMS, Southern Reference Area
10/9/2005	Soil Sampling - Silva Valley Elementary School, Rolling Hills Middle School			AAMS, Northern Reference Area
	New York Creek Nature Trail 8-Hour Perimeter Sampling (TRA-#TR)			
10/10/2005	Jackson School Garden and Outdoor Classroom	Jackson School Soccer	Jackson School Basketball and Kindergarten Play Area	AAMS, Northern Reference Area
10/11/2005	Soil Sampling - Dirt Parking Area			AAMS, Northern Reference Area
10/12/2005				AAMS

5.4.2.1 Silva Valley Elementary School

Field Maintenance Scenario. The baseball playing field at Silva Valley Elementary School is ordinarily maintained by parent volunteers during the playing season. The school district closed the field to Little League play during 2004. The field was mowed routinely, but grass had been allowed to grow over the basepaths and pitcher's mound areas. To conduct the baseball scenarios, the pitcher's mound and basepaths had to be cleared to bare soil, so the START added a new "field maintenance" scenario to the schedule. In doing so, the START was able to collect samples representing activities of parent volunteers while restoring the field to a condition suitable for conducting baseball play scenarios.

The START conducted the field maintenance scenario on October 2, 2004. The field maintenance scenario was conducted with six members of the sampling team simulating adult/parent volunteers who used rakes, hoes, and other garden tools to clear the basepaths and the pitcher's mound area. Sampling team members removed the grass and weeds from the areas cleared, shaking loose the dirt from the roots as needed, then placed the material that was removed in plastic garbage bags, which were later taken to the local dump in accordance with appropriate disposal procedures.

During the field maintenance scenario each member of the sampling team wore a personal air monitoring pump whose intake was set at a height of about 5 feet above ground surface to approximate the breathing height of parent volunteers. The intakes were also set at a height of about 5 feet for the air collection filter cassettes of the stationary sample pumps for the field maintenance scenario.

The outline of the field and the positions of the stationary air sample pumps operating during the field maintenance scenario are

US EPA ARCHIVE DOCUMENT

shown as SVM-1FD, SVM-2FD, SVM-3FD, SVM-4FD, and SVM-5FD on Figure 5-4 (Silva Valley Elementary School Baseball Playing Field Activity-Based Outdoor Air Sampling Locations–Maintenance Scenario). The position of the mobile meteorological station is shown as SVM-MS. A summary of results for the field maintenance scenario samples is shown in Table 5-6 (Silva Valley Elementary School Baseball Playing Field Maintenance Scenario Air Sample Summary Results). See Appendix B for a summary of the results from dust monitoring during this scenario.

Baseball Scenario A. The playing field at Silva Valley Elementary School was initially wet when the START arrived, and after clearing the basepaths and pitcher's mound the soil was still fairly moist. The purpose of the sampling during the baseball scenarios was to simulate baseball play on the field during the dry season, so instead of conducting two more baseball play scenarios immediately after the maintenance scenario, the START revised the planned sampling schedule. The START conducted one baseball play scenario on the field on the afternoon following the maintenance scenario (October 2, 2004), but then waited until the next morning to conduct the second baseball play scenario to allow the soil to dry out somewhat. This change impacted the schedule for scenarios that had been planned for the following day, so one of the three jogging/walking scenarios on the New York Creek Nature Trail was dropped from the schedule.

Prior to conducting the baseball play scenarios at Silva Valley Elementary School, to test the moisture level of the field the START collected three soil samples from dispersed locations on

US EPA ARCHIVE DOCUMENT

Figure 5-4

US EPA ARCHIVE DOCUMENT

US EPA ARCHIVE DOCUMENT

Table 5-6
Silva Valley Elementary School Baseball Playing Field Maintenance Scenario Air Sample Summary Results

Sample ID	Location	Location Description	Date	PCME Structures (s/cc)	AHERA-like Total Structures (s/cc)	Sensitivity (s/cc)
SVM-H2-1FD-100204	SVM-1FD	pitcher's mound hi-vol	10/02/04	0.000998	0.00200	0.000998
SVM-H2-2FD-100204	SVM-2FD	downwind hi-vol	10/02/04	0.00194	0.00291	0.000968
SVM-H2-3FD-100204	SVM-3FD	offset downwind hi-vol	10/02/04	0.000994	0.000994	0.000994
SVM-H2-4FD-100204	SVM-4FD	upwind hi-vol	10/02/04	<0.00289	<0.00289	0.000967
SVM-H2-5FD-100204	SVM-5FD	far downwind hi-vol	10/02/04	0.00387	0.00580	0.000967
SVM-L2-1AD-100204	SVM-1AD	adult #1	10/02/04	<0.00299	0.00500	0.000999
SVM-L2-2AD-100204	SVM-2AD	adult #2	10/02/04	0.00299	0.00498	0.000995
SVM-L2-3AD-100204	SVM-3AD	adult #3	10/02/04	0.00499	0.00699	0.000998
SVM-L2-4AD-100204	SVM-4AD	adult #4	10/02/04	0.000992	0.00298	0.000992
SVM-L2-5AD-100204	SVM-5AD	adult #5	10/02/04	0.00299	0.00498	0.000997
SVM-L2-15AD-100204	SVM-5AD	duplicate of adult #5	10/02/04	<0.00300	0.00100	0.00100
SVM-L2-6AD-100204	SVM-6AD	adult #6	10/02/04	0.000991	0.00297	0.00297
CC1-L6-1CA-100204	adult #1	composite sample collected during the 100204 scenarios	10/02/04	0.000992	0.00595	0.000992
CC1-L6-1CB-100204	adult/child #1	composite sample collected during the 100204 scenarios	10/02/04	0.000972	0.00389	0.000972
CC1-L6-2CB-100204	adult/child #2	composite sample collected during the 100204 scenarios	10/02/04	0.00202	0.00808	0.00101

US EPA ARCHIVE DOCUMENT

Table 5-6
Silva Valley Elementary School Baseball Playing Field Maintenance Scenario Air Sample Summary Results

Sample ID	Location	Location Description	Date	PCME Structures (s/cc)	AHERA-like Total Structures (s/cc)	Sensitivit y (s/cc)
CC1-L6-3CB-100204	adult/child #3	composite sample collected during the 100204 scenarios	10/02/04	results pending	results pending	results pending

PCME fibers = fibers longer than 5 microns with a width between 0.25 and 3 microns, and an aspect ratio (length to width) greater than 3:1
AHERA-like total structures = structures longer than 0.5 microns with an aspect ratio greater than 3:1 (Note this differs somewhat from the strict AHERA structure definition)
Sensitivity = the sample-specific lowest concentration of asbestos the laboratory can reliably detect
s/cc = structures per cubic centimeter

the field just below the top ¼ to ½ inch of soil. The START also collected soil moisture samples similarly from other playing fields and areas where activity-based sampling was conducted throughout the week. Many of these soil moisture samples were collected just prior to conducting activity-based sampling; in a few cases they were collected after activity-based sampling had already been conducted. Table 5-7 (Moisture Levels in Activity Area Soils) shows the results of the soil moisture tests conducted for the Silva Valley Elementary School baseball playing field, as well as for the other playing fields.

The START conducted activity-based outdoor air sampling during this scenario according to the following scripted schedule:

- For the entire 2-hour scenario, one member of the sampling team simulated the activities of an adult/parent spectator walking and standing behind the backstop and sitting in the dugouts. The intake for the air collection filter cassette worn by this sampling team member was set at a height of about 5 feet.
- For the first 30 minutes, five other members of the sampling team used rakes and brooms to perform types of field maintenance activities that might be conducted prior to a game. One team member swept in the dugouts, three used rakes on the dirt areas of the infield, and one observed the maintenance activities at close range to the others who were performing the work. The intakes for the air collection filter cassettes worn by these sampling team members were set at a height of about 3 feet.
- For the remaining 90 minutes, the five other members of the sampling team alternately sat in the dugouts for 10 minutes then played baseball (infield practice) for 20 minutes; this pattern was repeated three times in the 90 minutes.

US EPA ARCHIVE DOCUMENT

Table 5-7
Moisture Levels in Activity Area Soils

Sample Location	sample #	Collection Date	Collection Time	Scenario Date	Scenario Start Time	Moisture Concentration	RL
Silva Valley Elementary School Baseball Playing Field (Baseball Scenario A)	1	10/02/04	2:00 PM	10/02/04	4:24 PM	19.2	0.1
	2	10/02/04	2:04 PM			10.8	0.1
	3	10/02/04	2:08 PM			20.0	0.1
Silva Valley Elementary School Baseball Playing Field (Baseball Scenario B)	1	10/03/04	9:45 AM	10/03/04	10:06 AM	17.7	0.1
	2	10/03/04	9:48 AM			15.0	0.1
	3	10/03/04	9:50 AM			17.7	0.1
Rolling Hills Middle School Soccer Field	1	10/03/04	2:20 PM	10/03/04	2:50 PM	70.5	0.1
	2	10/03/04	2:23 PM			37.0	0.1
	3	10/03/04	2:25 PM			9.1	0.1
Community Park North Field Baseball Playing Field	1	10/07/04	3:19 PM	10/05/04	2:36 PM	1.3	0.1
	2	10/07/04	3:21 PM			1.9	0.1
	3	10/07/04	3:22 PM			1.8	0.1
Community Park South Field Baseball Playing Field (Baseball Scenario A)	1	10/07/04	3:10 PM	10/05/04	5:23 PM	2.2	0.1
	2	10/07/04	3:12 PM			3.0	0.1
	3	10/07/04	3:14 PM			1.8	0.1
Community Park South Field Baseball Playing Field (Baseball Scenario B)	*			10/06/04	1:05 PM	*	
	*					*	
	*					*	
Community Park South Field Baseball Playing Field (Baseball Scenario C)	*			10/06/04	3:58 PM	*	
	*					*	
	*					*	
New York Creek Baseball Playing Field	1	10/07/04	12:09 PM	10/07/04	12:39 PM	1.2	0.1
	2	10/07/04	12:11 PM			1.2	0.1
	3	10/07/04	12:13 PM			1.3	0.1
Community Park Lower Soccer Field	1	10/07/04	1:57 PM	10/07/04	3:45 PM	18.2	0.1
	2	10/07/04	1:59 PM			22.3	0.1
	3	10/07/04	2:02 PM			7.3	0.1
Jackson Elementary School Garden and Outdoor Classroom	1	10/11/04	8:06 AM	10/10/04	9:14 AM	3.2	0.1
	2	10/11/04	8:10 AM			2.4	0.1
	3	10/11/04	8:13 AM			6.1	0.1
Jackson Elementary School Grass-Covered Playing Field	1	10/11/04	11:39 AM	10/10/04	12:06 PM	6.6	0.1
	2	10/11/04	11:26 AM			6.3	0.1
	3	10/11/04	11:48 AM			14.0	0.1

Method: ASTM D 2216-90

Matrix: soil

Analysis: moisture

units: % by weight

RL = reporting limit

* = samples from South Field collected only one time on 10/07/04 (see results for South Field Baseball Scenario A)

US EPA ARCHIVE DOCUMENT

- During the last 30 minutes of infield practice play, a seventh member of the sampling team who was not wearing a sample pump entered the scenario to run bases and slide toward bases. This member of the sampling team was allowed to engage in more vigorous physical activity than the other members of the sampling team without having to worry about damaging the equipment or having the pumps fall out of the belts.

The outline of the field and the positions of the stationary air sample pumps operating during the first baseball scenario are shown as SVBA-1FD, SVBA-2FD, SVBA-3FD, SVBA-4FD, and SVBA-5FD on Figure 5-5 (Silva Valley Elementary School Baseball Playing Field Activity-Based Outdoor Air Sampling Locations–Baseball Scenario A). The position of the mobile meteorological station is shown as SVBA-MS. A summary of results for *Baseball Scenario A* samples is shown in Table 5-8 (Silva Valley Elementary School Baseball Scenario A Air Sample Summary Results). See Appendix B for a summary of the results from dust monitoring during this scenario.

Baseball Scenario B. The START collected soil samples to test the moisture level again immediately prior to conducting the second baseball play scenario at Silva Valley Elementary School (see Table 5-7: Moisture Levels in Activity Area Soils).

The START conducted *Baseball Scenario B* on October 3, 2004. The START conducted activity-based outdoor air sampling during the second baseball play scenario according to the same scripted schedule that was used for *Baseball Scenario A*.

The outline of the field and the positions of the stationary air sample pumps operating during the second baseball scenario are

US EPA ARCHIVE DOCUMENT

Figure 5-5

US EPA ARCHIVE DOCUMENT

US EPA ARCHIVE DOCUMENT

Table 5-8
Silva Valley Elementary School Baseball Scenario A Air Sample Summary Results

Sample ID	Location	Location Description	Date	PCME Structures (s/cc)	AHERA-like Total Structures (s/cc)	Sensitivity (s/cc)
SVBA-H2-1FD-100204	SVBA-1FD	pitcher's mound hi-vol	10/02/04	0.000994	0.0169	0.000994
SVBA-H2-2FD-100204	SVBA-2FD	downwind hi-vol	10/02/04	0.00300	0.00900	0.00100
SVBA-H2-3FD-100204	SVBA-3FD	offset downwind hi-vol	10/02/04	<0.00294	0.00295	0.000984
SVBA-H2-4FD-100204	SVBA-4FD	upwind hi-vol	10/02/04	0.000967	0.00193	0.000967
SVBA-H2-5FD-100204	SVBA-5FD	far downwind hi-vol	10/02/04	0.000964	0.00289	0.000964
SVBA-L2-1CH-100204	SVBA-1CH	child #1	10/02/04	0.0101	0.0202	0.00101
SVBA-L2-11CH-100204	SVBA-1CH	duplicate of child #1	10/02/04	0.00498	0.0189	0.000997
SVBA-L2-2CH-100204	SVBA-2CH	child #2	10/02/04	<0.00296	0.0139	0.000992
SVBA-L2-3CH-100204	SVBA-3CH	child #3	10/02/04	0.00199	0.00897	0.000997
SVBA-L2-4CH-100204	SVBA-4CH	child #4	10/02/04	0.00697	0.0110	0.000996
SVBA-L2-5CH-100204	SVBA-5CH	child #5	10/02/04	0.00393	0.0137	0.000981
SVBA-L2-1NA-100204	SVBA-1NA	non-active adult	10/02/04	<0.00298	<0.00298	0.000995
CC1-L6-1CA-100204	adult #1	composite sample collected during the 100204 scenarios	10/02/04	0.000992	0.00595	0.000992
CC1-L6-1CB-100204	adult/child #1	composite sample collected during the 100204 scenarios	10/02/04	0.000972	0.00389	0.000972
CC1-L6-2CB-100204	adult/child #2	composite sample collected during the 100204 scenarios	10/02/04	0.00202	0.00808	0.00101
CC1-L6-3CB-100204	adult/child #3	composite sample collected during the 100204 scenarios	10/02/04	results pending	results pending	results pending
SVBA-L2-1ZB-100204	field blank	field blank	10/02/04	<0.00296	<0.00296	0.000991

PCME fibers = fibers longer than 5 microns with a width between 0.25 and 3 microns, and an aspect ratio (length to width) greater than 3:1
AHERA-like total structures = structures longer than 0.5 microns with an aspect ratio greater than 3:1 (Note this differs somewhat from the strict AHERA structure definition)
Sensitivity = the sample-specific lowest concentration of asbestos the laboratory can reliably detect
s/cc = structures per cubic centimeter

shown as SVBB-1FD, SVBB-2FD, SVBB-3FD, SVBB-4FD, and SVBB-5FD on Figure 5-6 (Silva Valley Elementary School Baseball Playing Field Activity-Based Outdoor Air Sampling Locations–Baseball Scenario B). The position of the mobile meteorological station is shown as SVBB-MS. A summary of results for the field maintenance scenario samples is shown in Table 5-9 (Silva Valley Elementary School Baseball Scenario B Air Sample Summary Results). See Appendix B for a summary of the results from dust monitoring during this scenario.

5.4.2.2 Rolling Hills Middle School

The logistics of conducting one scenario at Silva Valley Elementary School in the morning, and then moving all the equipment and personnel to Rolling Hills Middle School to conduct two more scenarios took more time than was anticipated. In order to complete scenarios at both the soccer field and the basketball play area at Rolling Hills Middle School before dark, the START cut the length of time the soccer scenario was performed; instead of 2 hours the soccer scenario was stopped after about 1.5 hours of activity had been completed.

Soccer Scenario. The START collected soil samples to test the moisture level immediately prior to conducting the soccer scenario at Rolling Hills Middle School (see Table 5-7: Moisture Levels in Activity Area Soils). The soccer scenario activity was conducted on a rectangular area toward the southern end of the field where the START observed bare areas in the grass.

The START conducted activity-based outdoor air sampling during the soccer scenario on October 3, 2004, according to the following scripted schedule:

US EPA ARCHIVE DOCUMENT

Figure 5-6

US EPA ARCHIVE DOCUMENT

Table 5-9
Silva Valley Elementary School Baseball Scenario B Air Sample Summary Results

Sample ID	Location	Location Description	Date	PCME Structures (s/cc)	AHERA-like Total Structures (s/cc)	Sensitivity (s/cc)
SVBB-H2-1FD-100304	SVBB-1FD	pitcher's mound hi-vol	10/03/04	<0.00302	0.00202	0.00101
SVBB-H2-2FD-100304	SVBB-2FD	downwind hi-vol	10/03/04	<0.00294	<0.00294	0.000983
SVBB-H2-12FD-100304	SVBB-2FD	duplicate of downwind hi-vol	10/03/04	<0.00297	0.00199	0.000994
SVBB-H2-3FD-100304	SVBB-3FD	offset downwind hi-vol	10/03/04	<0.00297	<0.00297	0.000994
SVBB-H2-4FD-100304	SVBB-4FD	upwind hi-vol	10/03/04	0.000958	0.00192	0.000958
SVBB-H2-5FD-100304	SVBB-5FD	far downwind hi-vol	10/03/04	0.000998	<0.00298	0.000998
SVBB-L2-1CH-100304	SVBB-1CH	child #1	10/03/04	0.00299	0.00699	0.000998
SVBB-L2-2CH-100304	SVBB-2CH	child #2	10/03/04	0.000999	0.00999	0.000999
SVBB-L2-12CH-100304	SVBB-2CH	duplicate of child #2	10/03/04	<0.00296	0.00396	0.000989
SVBB-L2-3CH-100304	SVBB-3CH	child #3	10/03/04	0.00293	0.00586	0.000977
SVBB-L2-4CH-100304	SVBB-4CH	child #4	10/03/04	0.00296	0.00888	0.00148
SVBB-L2-5CH-100304	SVBB-5CH	child #5	10/03/04	0.000997	0.00399	0.000997
SVBB-L2-1NA-100304	SVBB-1NA	non-active adult	10/03/04	<0.00294	<0.00294	0.000984
CC2A-L6-1CA-100304	adult #1	composite sample collected during the 100304 scenarios	10/03/04	0.000995	0.0109	0.000995
CC2-L6-1CC-100304	child #1	composite sample collected during the 100304 scenarios	10/03/04	<0.00292	0.00195	0.000976

US EPA ARCHIVE DOCUMENT

US EPA ARCHIVE DOCUMENT

Table 5-9
Silva Valley Elementary School Baseball Scenario B Air Sample Summary Results

Sample ID	Location	Location Description	Date	PCME Structures (s/cc)	AHERA-like Total Structures (s/cc)	Sensitivity (s/cc)
CC2-L6-11CC-100304	duplicate of child #1	composite sample collected during the 100304 scenarios	10/03/04	0.000982	0.00295	0.000982
CC2-L6-2CC-100304	child #2	composite sample collected during the 100304 scenarios	10/03/04	<0.00298	<0.00298	0.000996
CC2-L6-3CC-100304	child #3	composite sample collected during the 100304 scenarios	10/03/04	0.00491	0.0108	0.000982
CC2-L6-4CC-100304	child #4	composite sample collected during the 100304 scenarios	10/03/04	0.00300	0.0150	0.000999
SVBB-L2-1ZB-100304	field blank	field blank	10/03/04	<0.00298	<0.00298	0.000998

PCME fibers = fibers longer than 5 microns with a width between 0.25 and 3 microns, and an aspect ratio (length to width) greater than 3:1
AHERA-like total structures = structures longer than 0.5 microns with an aspect ratio greater than 3:1 (Note this differs somewhat from the strict AHERA structure definition)
Sensitivity = the sample-specific lowest concentration of asbestos the laboratory can reliably detect
s/cc = structures per cubic centimeter

- For the entire 1.5-hour scenario, one member of the sampling team simulated the activities of an adult/parent spectator sitting near the edge or walking around and occasionally within the area of play. This member of the sampling team would sometimes retrieve a ball kicked out of the area of play. The intake for the air collection filter cassette worn by this sampling team member was set at a height of about 5 feet.
- For the entire 1.5-hour scenario, five other members of the sampling team passed soccer balls back and forth to each other. They sometimes formed a small circle to practice passing; other times they dribbled the ball across the field with other sampling team members close by. The intakes for the air collection filter cassettes worn by these sampling team members were set at a height of about 3 feet.

The area within which the START conducted the activity is outlined and the positions of the stationary air sample pumps operating during the soccer scenario are shown as RHS-1FD, RHS-2FD, RHS-3FD, RHS-4FD, and RHS-5FD on Figure 5-7 (Rolling Hills Middle School Soccer Field Activity-Based Outdoor Air Sampling Locations–Soccer Scenario). The position of the mobile meteorological station is shown as RHS-MS. A summary of results for the soccer scenario samples is shown in Table 5-10 (Rolling Hills Middle School Soccer Scenario Air Sample Summary Results). See Appendix B for a summary of the results from dust monitoring during this scenario.

Basketball Scenario. The START conducted the basketball scenario on a half court of one of the basketball courts at Rolling Hills Middle School on October 3, 2004.

US EPA ARCHIVE DOCUMENT

Figure 5-7

US EPA ARCHIVE DOCUMENT

US EPA ARCHIVE DOCUMENT

Table 5-10
Rolling Hills Middle School Soccer Scenario Air Sample Summary Results

Sample ID	Location	Location Description	Date	PCME Structures (s/cc)	AHERA-like Total Structures (s/cc)	Sensitivity (s/cc)
RHS-H2-1FD-100304	RHS-1FD	on-field hi-vol	10/03/04	0.000988	0.00198	0.000988
RHS-H2-2FD-100304	RHS-2FD	on-field hi-vol	10/03/04	<0.00295	0.00197	0.000986
RHS-H2-3FD-100304	RHS-3FD	on-field hi-vol	10/03/04	<0.00295	<0.00295	0.000987
RHS-H2-4FD-100304	RHS-4FD	upwind hi-vol	10/03/04	<0.00284	<0.00284	0.000951
RHS-H2-5FD-100304	RHS-5FD	far downwind hi-vol	10/03/04	<0.00283	0.00189	0.000947
RHS-L2-1CH-100304	RHS-1CH	child #1	10/03/04	<0.00298	<0.00298	0.000998
RHS-L2-2CH-100304	RHS-2CH	child #2	10/03/04	0.000994	0.00398	0.000994
RHS-L2-3CH-100304	RHS-3CH	child #3	10/03/04	0.000999	0.000999	0.000999
RHS-L2-4CH-100304	RHS-4CH	child #4	10/03/04	0.000996	0.000996	0.000996
RHS-L2-14CH-100304	RHS-4CH	duplicate of child #4	10/03/04	0.000990	0.000990	0.000990
RHS-L2-5CH-100304	RHS-5CH	child #5	10/03/04	<0.00296	0.00198	0.000991
RHS-L2-1NA-100304	RHS-1NA	non-active adult	10/03/04	<0.00298	<0.00298	0.000998
CC2A-L6-1CA-100304	adult #1	composite sample collected during the 100304 scenarios	10/03/04	0.000995	0.0109	0.000995
CC2-L6-1CC-100304	child #1	composite sample collected during the 100304 scenarios	10/03/04	<0.00292	0.00195	0.000976
CC2-L6-11CC-100304	duplicate of child #1	composite sample collected during the 100304 scenarios	10/03/04	0.000982	0.00295	0.000982

US EPA ARCHIVE DOCUMENT

Table 5-10
Rolling Hills Middle School Soccer Scenario Air Sample Summary Results

Sample ID	Location	Location Description	Date	PCME Structures (s/cc)	AHERA-like Total Structures (s/cc)	Sensitivity (s/cc)
CC2-L6-2CC-100304	child #2	composite sample collected during the 100304 scenarios	10/03/04	<0.00298	<0.00298	0.000996
CC2-L6-3CC-100304	child #3	composite sample collected during the 100304 scenarios	10/03/04	0.00393	0.0108	0.000982
CC2-L6-4CC-100304	child #4	composite sample collected during the 100304 scenarios	10/03/04	0.00300	0.0150	0.000999

PCME fibers = fibers longer than 5 microns with a width between 0.25 and 3 microns, and an aspect ratio (length to width) greater than 3:1
AHERA-like total structures = structures longer than 0.5 microns with an aspect ratio greater than 3:1 (Note this differs somewhat from the strict AHERA structure definition)
Sensitivity = the sample-specific lowest concentration of asbestos the laboratory can reliably detect
s/cc = structures per cubic centimeter

The START conducted activity-based outdoor air sampling during the basketball scenario according to the following scripted schedule:

- For the entire 2-hour scenario, one member of the sampling team simulated the activities of an adult/parent spectator sitting near the edge or walking around and occasionally within the area of play. This member of the sampling team would sometimes retrieve a ball that bounced out of the area of play. The intake for the air collection filter cassette worn by this sampling team member was set at a height of about 5 feet.
- For the first 10 minutes, two other members of the sampling team used brooms to sweep the half-court, two dribbled basketballs around the half-court, and one walked close by those sweeping or dribbling. The intakes for the air collection filter cassettes worn by these sampling team members were set at a height of about 3 feet.
- For the remaining 110 minutes, these five other members of the sampling team played basketball and conducted practice drills according to the following 10-minute activity modules:
  - Layups
  - Top of the Key
  - Half-Court Game
  - Foul Line Shots
  - Layups
  - Top of the Key
  - Half-Court Game
  - Foul Line Shots
  - Layups
  - Top of the Key
  - Free Shots

US EPA ARCHIVE DOCUMENT

The area within which the START conducted the activity is outlined and the positions of the stationary air sample pumps operating during the basketball scenario are shown as RHB-1FD, RHB-2FD, RHB-3FD, RHB-4FD, and RHB-5FD on Figure 5-8 (Rolling Hills Middle School Activity-Based Outdoor Air Sampling Locations–Basketball Scenario). The position of the mobile meteorological station is shown as RHB-MS. A summary of results for the basketball scenario samples is shown in Table 5-11 (Rolling Hills Middle School Basketball Scenario Air Sample Summary Results). See Appendix B for a summary of the results from dust monitoring during this scenario.

5.4.2.3 Children's Playground at the Community Park

The START conducted two scenarios at the Children's Playground at the Community Park on October 4, 2004: a *Typical Activity Scenario* and an *Aggressive Activity Scenario*. The plan for these scenarios, as described in the *El Dorado Hills Naturally Occurring Asbestos Multimedia Exposure Assessment, El Dorado Hills, California, Activity-Based Outdoor Air Sampling of Community Park Children's Playground Field Sampling Plan*, had called for members of the sampling team to simulate typical levels of children's play activity during the *Typical Activity Scenario* and a slightly more vigorous level of activity during the *Aggressive Activity Scenario*. To limit the variables between the two scenarios, however, U.S. EPA directed members of the sampling team to simulate children's play at about the same level of activity for both scenarios. The primary differences between the two scenarios were the addition of the following for the *Aggressive Activity Scenario*:

- A leaf blower was used just prior to conducting scenario activity on each side of the playground.

US EPA ARCHIVE DOCUMENT

Figure 5-8

US EPA ARCHIVE DOCUMENT

US EPA ARCHIVE DOCUMENT

Table 5-11
Rolling Hills Middle School Basketball Scenario Air Sample Summary Results

Sample ID	Location	Location Description	Date	PCME Structures (s/cc)	AHERA-like Total Structures (s/cc)	Sensitivity (s/cc)
RHB-H2-1FD-100304	RHB-1FD	on basketball court hi-vol	10/03/04	0.000974	0.000974	0.000974
RHB-H2-2FD-100304	RHB-2FD	on basketball court hi-vol	10/03/04	0.00297	0.00594	0.000990
RHB-H2-3FD-100304	RHB-3FD	on basketball court hi-vol	10/03/04	<0.00303	0.00203	0.00101
RHB-H2-4FD-100304	RHB-4FD	upwind hi-vol	10/03/04	<0.00286	0.00478	0.000957
RHB-H2-5FD-100304	RHB-5FD	far downwind hi-vol	10/03/04	<0.00293	0.000981	0.000981
RHB-L2-1CH-100304	RHB-1CH	child #1	10/03/04	0.00300	0.00900	0.000999
RHB-L2-2CH-100304	RHB-2CH	child #2	10/03/04	0.000995	0.00299	0.000995
RHB-L2-3CH-100304	RHB-3CH	child #3	10/03/04	0.000996	0.000996	0.000996
RHB-L2-4CH-100304	RHB-4CH	child #4	10/03/04	0.000989	0.00395	0.000989
RHB-L2-14CH-100304	RHB-4CH	duplicate of child #4	10/03/04	0.000991	0.00495	0.000991
RHB-L2-5CH-100304	RHB-5CH	child #5	10/03/04	0.000999	0.00400	0.000999
RHB-L2-1NA-100304	RHB-1NA	non-active adult	10/03/04	0.00100	0.00502	0.00100
CC2A-L6-1CA-100304	adult #1	composite sample collected during the 100304 scenarios	10/03/04	0.000995	0.0109	0.000995
CC2-L6-1CC-100304	child #1	composite sample collected during the 100304 scenarios	10/03/04	<0.00292	0.00195	0.000976
CC2-L6-11CC-100304	duplicate of child #1	composite sample collected during the 100304 scenarios	10/03/04	0.000982	0.00295	0.000982

US EPA ARCHIVE DOCUMENT

Table 5-11
Rolling Hills Middle School Basketball Scenario Air Sample Summary Results

Sample ID	Location	Location Description	Date	PCME Structures (s/cc)	AHERA-like Total Structures (s/cc)	Sensitivity (s/cc)
CC2-L6-2CC-100304	child #2	composite sample collected during the 100304 scenarios	10/03/04	<0.00298	<0.00298	0.000996
CC2-L6-3CC-100304	child #3	composite sample collected during the 100304 scenarios	10/03/04	0.00393	0.0108	0.000982
CC2-L6-4CC-100304	child #4	composite sample collected during the 100304 scenarios	10/03/04	0.00300	0.0150	0.000999
RHB-L2-1ZB-100304	field blank	field blank	10/03/04	<0.00277	<0.00277	0.000925

PCME fibers = fibers longer than 5 microns with a width between 0.25 and 3 microns, and an aspect ratio (length to width) greater than 3:1
AHERA-like total structures = structures longer than 0.5 microns with an aspect ratio greater than 3:1 (Note this differs somewhat from the strict AHERA structure definition)
Sensitivity = the sample-specific lowest concentration of asbestos the laboratory can reliably detect
s/cc = structures per cubic centimeter

- Several box fans were set up in rings around each side of the playground to blow air toward the center of the playground during the scenario.

For both scenarios, the intakes for the air collection filter cassettes worn by the sampling team members were set at a height of about 3 feet. No stationary air sample pumps were operated during the either of the Children's Playground scenarios.

<u>*Typical Activity Scenario*</u>. The START performed the *Typical Activity Scenario* first. The START conducted activity-based outdoor air sampling during the scenario according to the following scripted schedule (see Table 5-12: Children's Playground Scenario Activities):

- For the first hour of the scenario five members of the sampling team simulated the activities of children playing in the western portion of the Children's Playground, which has a wood chip ground cover. One member of the sampling team conducted solitary play activities during the scenario, while the other four sampling team members conducted activities in two pairs. They alternated activities every 10 minutes, as shown in Table 5-12 (Children's Playground Scenario Activities).
- For the second hour of the scenario five members of the sampling team simulated the activities of children playing in the eastern portion of the Children's Playground, which has rubber-like playground surface in most of the area and a sand box in one corner. One member of the sampling team continued to conduct solitary play activities during the scenario, while the other four sampling team members conducted activities in two pairs. They alternated activities every 10 minutes, as shown in Table 5-12 (Children's Playground Scenario Activities).

US EPA ARCHIVE DOCUMENT

Table 5-12
Children's Playground Scenario Activities

**Western Portion of Playground (with wood chip ground cover)**						
	**0 to 10 minutes**	**10 to 20 minutes**	**20 to 30 minutes**	**30 to 40 minutes**	**40 to 50 minutes**	**50 to 60 minutes**
Pair A	swings	spinners	ball	play structure	ball	walk/run
Pair B	ball	play structure	walking/ running (some foot-dragging)	swings	spinners	ball and climbing
Solitary Player	play structure	climbing	swings	ball/foot-dragging	play structure (also sliding with wood chips on slide)	mix of activities
**Eastern Portion of Playground (with rubber-like ground surface and sand box)**						
	**60 to 70 minutes**	**70 to 80 minutes**	**80 to 90 minutes**	**90 to 100 minutes**	**100 to 110 minutes**	**110 to 120 minutes**
Pair A	play structure	sand box	ball	play structure	sand box	ball
Pair B	sand box	ball	play structure	sand box	ball	play structure
Solitary Player	ball	play structure	sand box	ball	play structure	sand box

US EPA ARCHIVE DOCUMENT

<u>*Aggressive Activity Scenario*</u>. The START performed the *Aggressive Activity Scenario* second, with a break of more than an hour between the *Typical Activity Scenario* and the *Aggressive Activity Scenario*. The START conducted activity-based outdoor air sampling during the scenario according to the following scripted schedule:

- For about 20 minutes prior to starting the scenario, one member of the sampling team used a leaf blower in the western portion of the Children's Playground, which has a wood chip ground cover. The START placed several box fans in a ring around this portion of the playground facing the center of the area. The team member using the leaf blower turned on each of the fans as he walked around in that portion of the playground blowing toward the ground, at the play structures, and up toward the shade canopy. See Table 5-13: Status of Leaf Blower and Fans During Aggressive Activity Scenario.
- The leaf blower was turned off but the fans remained turned on, and for the first hour of the scenario five members of the sampling team simulated the activities of children playing in the western portion of the Children's Playground. As was done during the *Typical Activity Scenario*, one member of the sampling team conducted solitary play activities during the scenario, while the other four sampling team members conducted activities in two pairs. They alternated activities every 10 minutes, as shown in Table 5-12 (Children's Playground Scenario Activities).

US EPA ARCHIVE DOCUMENT

Table 5-13
Status of Leaf Blower and Fans During Aggressive Activity Scenario

20 minutes before start of scenario	0 to 40 minutes	40 to 60 minutes	60 to 120 minutes
use leaf blower in western portion; turn on fans in western portion	leaf blower off; fans remain on in western portion	use leaf blower in eastern portion; turn on fans in eastern portion; fans still on in western portion or in transit to eastern portion	leaf blower off; fans on in eastern portion; fans off in western portion
no activity-based sampling	activity-based sampling in western portion	activity-based sampling in western portion	activity-based sampling in eastern portion

- For about the last 20 minutes of the first hour, while the five members of the sampling team were still conducting scenario activities in the western portion of the Children's Playground, another member of the sampling team used a leaf blower in the eastern portion of the playground. This side of the playground has a rubber-like ground surface and a sand box. The START placed several other box fans in a circle around this portion of the playground, and the team member using the leaf blower turned on each of the fans as he walked around in that portion of the playground blowing toward the ground and at the play structures.
- The leaf blower was then turned off while the fans remained turned on, and for the second hour of the scenario five members of the sampling team simulated the activities of children playing in the eastern portion of the Children's Playground. One member of the sampling team continued to conduct solitary play activities during the scenario, while the other four sampling team members conducted activities in two pairs.

US EPA ARCHIVE DOCUMENT

They alternated activities every 10 minutes, as shown in Table 5-12 (Children's Playground Scenario Activities).

The outline of the Children's Playground is shown on Figure 5-9 (Community Park Children's Playground Activity-Based Outdoor Air Sampling Area–Typical and Aggressive Activity Scenarios). The position of the mobile meteorological station (which was used only during the Typical Activity Scenario) and the Ambient Air Monitoring Station are shown as TPG-MS and AAMS, respectively. The position of the mobile meteorological station is shown as RHB-MS. A summary of results for the playground scenario samples is shown in Table 5-14 (Community Park Children's Playground Typical and Aggressive Activity Scenarios Air Sample Summary Results). See Appendix B for a summary of the results from dust monitoring during these scenarios.

5.4.2.4 Playing Fields at the Community Park

During the baseball and soccer scenarios conducted on the playing fields at the Community Park, the START operated additional stationary high-flow air sample pumps at the Children's Playground.

North Field Baseball Playing Field. The START conducted activity-based outdoor air sampling at the North Field on October 5. The START intended to collect soil samples to test the moisture level of the field prior to conducting the scenario, but this was inadvertently overlooked. The soil moisture samples were collected instead on October 7, 2004 (see Table 5-7: Moisture Levels in Activity Area Soils).

The START conducted activity-based outdoor air sampling at the North Field, according to the following scripted schedule:

US EPA ARCHIVE DOCUMENT

Figure 5-9

US EPA ARCHIVE DOCUMENT

US EPA ARCHIVE DOCUMENT

Table 5-14
Community Park Children's Playground Typical and Aggressive Activity Scenarios Air Sample Summary Results

Sample ID	Location	Location Description	Date	PCME Structures (s/cc)	AHERA-like Total Structures (s/cc)	Sensitivity (s/cc)
TPG-L2-1CH-100404	TPG-1CH	child #1	10/04/04	0.00701	0.0260	0.00100
TPG-L2-11CH-100404	TPG-1CH	duplicate of child #1	10/04/04	0.00400	0.0140	0.00100
TPG-L2-2CH-100404	TPG-2CH	child #2	10/04/04	0.00624	0.108	0.00208
TPG-L2-3CH-100404	TPG-3CH	child #3	10/04/04	0.000998	0.0220	0.000998
TPG-L2-4CH-100404	TPG-4CH	child #4	10/04/04	<0.0161	0.264	0.00539
TPG-L2-5CH-100404	TPG-5CH	child #5	10/04/04	0.00389	0.0557	0.00130
TPG-L2-1ZB-100404	field blank	field blank	10/04/04	<0.00297	<0.00297	0.000995
APG-L2-1CH-100404	APG-1CH	child #1	10/04/04	results pending	results pending	results pending
APG-L2-2CH-100404	APG-2CH	child #2	10/04/04	0.00999	0.0190	0.000999
APG-L2-3CH-100404	APG-3CH	child #3	10/04/04	0.000998	0.00889	0.000988
APG-L2-13CH-100404	APG-3CH	duplicate of child #3	10/04/04	results pending	results pending	results pending
APG-L2-4CH-100404	APG-4CH	child #4	10/04/04	0.00399	0.00997	0.000997
APG-L2-5CH-100404	APG-5CH	child #5	10/04/04	0.00100	0.00603	0.00100
APG-L2-1ZB-100404	field blank	field blank	10/04/04	<0.00319	<0.00319	0.00107

PCME fibers = fibers longer than 5 microns with a width between 0.25 and 3 microns, and an aspect ratio (length to width) greater than 3:1
AHERA-like total structures = structures longer than 0.5 microns with an aspect ratio greater than 3:1 (Note this differs somewhat from the strict AHERA structure definition)
Sensitivity = the sample-specific lowest concentration of asbestos the laboratory can reliably detect
s/cc = structures per cubic centimeter

- For the entire 2-hour scenario, one member of the sampling team simulated the activities of an adult/parent spectator walking and standing behind the backstop and sitting on the bleachers and in the dugouts. The intake for the air collection filter cassette worn by this sampling team member was set at a height of about 5 feet.
- For the first 10 minutes, another member of the sampling team drove an electric maintenance cart towing a steel drag mat around to groom the infield. The cart and drag mat are the same equipment the CSD maintenance staff use to groom the infields prior to games. Use of the cart and drag mat mimicked CSD infield maintenance procedures, except that the START did not wet the infield down, as would normally be done prior to dragging, in an attempt to capture the upper end of exposure levels. During the infield dragging, two other members of the sampling team sat in the dugouts, and another two members of the sampling team walked around the infield, occasionally within or near the cloud of dust created by the drag mat. The intakes for the air collection filter cassettes worn by these five sampling team members were set at a height of about 3 feet.
- For the next 20 minutes, the electric cart and drag mat were set aside, and these five members of the sampling team walked around in the infield and spectator areas and used hand tools to perform field grooming tasks. One member of the sampling team swept the dugouts, one swept behind the backstops near the spectator bleachers, one used a dirt rake on the infield dirt, one walked around the bases, and one used a dirt tamp to tamp around the pitcher's mound and home plate.
- For the remaining 90 minutes, the five members of the sampling team simulating the activities of children alternately sat in the dugouts for 10 minutes then played baseball (infield prac-

US EPA ARCHIVE DOCUMENT

tice) for 20 minutes; this pattern was repeated three times in the 90 minutes.

- During the last 30 minutes of infield practice play, a seventh member of the sampling team who was not wearing a sample pump entered the scenario to run bases and slide toward bases. This member of the sampling team was allowed to engage in more vigorous physical activity than the other members of the sampling team without having to worry about damaging the equipment or having the pumps fall out of the belts.

The outline of the field and the positions of the stationary air sample pumps operating during the North Field baseball scenario are shown as NFB-1FD, NFB-2FD, NFB-3FD, NFB-4FD, NFB-5FD, NFB-1PG, NFB-2PG, NFB-3PG, NFB-4PG, and NFB-5PG on Figure 5-10 (Community Park North Field Activity-Based Outdoor Air Sampling Locations–Baseball Scenario). Two composite samples collected during both baseball scenarios conducted on October 5 are shown as CC2-05-1CP and CC2-05-2CP. The position of the mobile meteorological station is shown as NFB-MS. A summary of results for the baseball scenario samples is shown in Table 5-15 (Community Park North Field Baseball Scenario Air Sample Summary Results). See Appendix B for a summary of the results from dust monitoring during this scenario.

South Field Baseball Playing Field. The START conducted three baseball scenarios on the South Field at the Community Park. One scenario was conducted on October 5 (*Baseball Scenario A*), and two were conducted on October 6, 2004 (*Baseball Scenario B* and *Baseball Scenario C*). The START intended to collect soil samples to test the moisture level of the field prior to conducting each scenario, but this was inadvertently overlooked. Instead, a single set of soil moisture samples was collected for the South Field on

US EPA ARCHIVE DOCUMENT

Figure 5-10

US EPA ARCHIVE DOCUMENT

US EPA ARCHIVE DOCUMENT

Table 5-15
Community Park North Field Baseball Scenario Air Sample Summary Results

Sample ID	Location	Location Description	Date	PCME Structures (s/cc)	AHERA-like Total Structures (s/cc)	Sensitivity (s/cc)
NFB-H2-1FD-100504	NFB-1FD	pitcher's mound hi-vol	10/05/04	<0.00299	0.00899	0.000998
NFB-H2-2FD-100504	NFB-2FD	downwind hi-vol (somewhat crosswind)	10/05/04	0.00692	0.0198	0.000988
NFB-H2-3FD-100504	NFB-3FD	offset downwind hi-vol (somewhat crosswind)	10/05/04	results pending	results pending	results pending
NFB-H2-4FD-100504	NFB-4FD	upwind hi-vol (somewhat crosswind)	10/05/04	0.00490	0.00979	0.000979
NFB-H2-5FD-100504	NFB-5FD	far downwind hi-vol (somewhat crosswind)	10/05/04	0.00293	0.00489	0.000977
NFB-H2-1PG-100504	NFB-1PG	children's playground hi-vol	10/05/04	0.000958	0.00383	0.000958
NFB-H2-2PG-100504	NFB-2PG	children's playground hi-vol	10/05/04	<0.00294	0.000983	0.000983
NFB-H2-3PG-100504	NFB-3PG	children's playground hi-vol	10/05/04	0.00196	0.00490	0.000981
NFB-H2-4PG-100504	NFB-4PG	children's playground hi-vol	10/05/04	0.00196	0.00393	0.000982
NFB-H2-5PG-100504	NFB-5PG	children's playground hi-vol	10/05/04	0.00297	0.00396	0.000991
NFB-L2-1CH-100504	NFB-1CH	child #1	10/05/04	results pending	results pending	results pending
NFB-L2-2CH-100504	NFB-2CH	child #2	10/05/04	0.00761	0.0399	0.000951
NFB-L2-3CH-100504	NFB-3CH	child #3	10/05/04	0.0169	0.0627	0.000995
NFB-L2-4CH-100504	NFB-4CH	child #4	10/05/04	results pending	results pending	results pending
NFB-L2-5CH-100504	NFB-5CH	child #5	10/05/04	results pending	results pending	results pending

US EPA ARCHIVE DOCUMENT

Table 5-15
Community Park North Field Baseball Scenario Air Sample Summary Results

Sample ID	Location	Location Description	Date	PCME Structures (s/cc)	AHERA-like Total Structures (s/cc)	Sensitivity (s/cc)
NFB-L2-15CH-100504	NFB-5CH	duplicate of child #5	10/05/04	results pending	results pending	results pending
NFB-L2-1NA-100504	NFB-1NA	non-active adult	10/05/04	0.00697	0.0159	0.000996
CC2-H6-1CP-100504	CC2-05-1CP	children's playground hi-vol composite sample collected during the 100504 scenarios	10/05/04	0.000991	0.0129	0.000991
CC2-H6-2CP-100504	CC2-05-2CP	children's playground hi-vol composite sample collected during the 100504 scenarios	10/05/04	results pending	results pending	results pending
CC2-L6-1CA-100504	adult #1	composite sample collected during the 100504 scenarios	10/05/04	0.000995	0.0229	0.000995
CC2-L6-1CC-100504	child #1	composite sample collected during the 100504 scenarios	10/05/04	results pending	results pending	results pending
CC2-L6-11CC-100504	child #1	duplicate of child #1 composite sample collected during the 100504 scenarios	10/05/04	results pending	results pending	results pending
CC2-L6-2CC-100504	child #2	composite sample collected during the 100504 scenarios	10/05/04	results pending	results pending	results pending
CC2-L6-3CC-100504	child #3	composite sample collected during the 100504 scenarios	10/05/04	results pending	results pending	results pending
NFB-L2-1ZB-100504	field blank	field blank	10/05/04	<0.00296	<0.00296	0.000991

PCME fibers = fibers longer than 5 microns with a width between 0.25 and 3 microns, and an aspect ratio (length to width) greater than 3:1
AHERA-like total structures = structures longer than 0.5 microns with an aspect ratio greater than 3:1 (Note this differs somewhat from the strict AHERA structure definition)
Sensitivity = the sample-specific lowest concentration of asbestos the laboratory can reliably detect
s/cc = structures per cubic centimeter

October 7, 2004 (see Table 5-7: Soil Moisture Levels in Activity Area Soils).

The START conducted *Baseball Scenario A* and *Baseball Scenario B* according to the same scripted schedule used for the North Field. For *Baseball Scenario C*, however, the START conducted it entirely as a baseball infield practice scenario. The first 30 minutes of field maintenance activity (including the use of the steel drag mat) was replaced with infield practice play, so the scenario was still 2 hours in length.

South Field *Baseball Scenario A*. The START conducted activity-based outdoor air sampling during *Baseball Scenario A* at the South Field according to the same scripted schedule that was used for the North Field.

The outline of the field and the positions of the stationary air sample pumps operating during the South Field *Baseball Scenario A* are shown as SFBA-1FD, SFBA-2FD, SFBA-3FD, SFBA-4FD, SFBA-5FD, SFBA-1PG, SFBA-2PG, SFBA-3PG, SFBA-4PG, and SFBA-5PG on Figure 5-11 (Community Park South Field Activity-Based Outdoor Air Sampling Locations–Baseball Scenario A). Two composite samples collected during both baseball scenarios conducted on October 5 are shown as CC2-05-1CP and CC2-05-2CP. The position of the mobile meteorological station is shown as SFBA-MS. A summary of results for *Baseball Scenario A* samples is shown in Table 5-16 (Community Park South Field Baseball Scenario A Air Sample Summary Results).

US EPA ARCHIVE DOCUMENT

Figure 5-11

US EPA ARCHIVE DOCUMENT

US EPA ARCHIVE DOCUMENT

Table 5-16
Community Park South Field Baseball Scenario A Air Sample Summary Results

Sample ID	Location	Location Description	Date	PCME Structures (s/cc)	AHERA-like Total Structures (s/cc)	Sensitivity (s/cc)
SFBA-H2-1FD-100504	SFBA-1FD	pitcher's mound hi-vol	10/05/04	results pending	results pending	results pending
SFBA-H2-2FD-100504	SFBA-2FD	downwind hi-vol	10/05/04	0.00481	0.0786	0.00160
SFBA-H2-3FD-100504	SFBA-3FD	offset downwind hi-vol	10/05/04	0.0195	0.195	0.00390
SFBA-H2-4FD-100504	SFBA-4FD	upwind hi-vol	10/05/04	0.000982	0.0177	0.000982
SFBA-H2-5FD-100504	SFBA-5FD	far downwind hi-vol	10/05/04	0.00493	0.00691	0.000987
SFBA-H2-1PG-100504	SFBA-1PG	children's playground hi-vol	10/05/04	0.00369	0.0194	0.000923
SFBA-H2-2PG-100504	SFBA-2PG	children's playground hi-vol	10/05/04	results pending	results pending	results pending
SFBA-H2-3PG-100504	SFBA-3PG	children's playground hi-vol	10/05/04	0.000964	0.00675	0.000964
SFBA-H2-4PG-100504	SFBA-4PG	children's playground hi-vol	10/05/04	results pending	results pending	results pending
SFBA-H2-5PG-100504	SFBA-5PG	children's playground hi-vol	10/05/04	0.00958	0.0249	0.000958
SFBA-L2-1CH-100504	SFBA-1CH	child #1	10/05/04	results pending	results pending	results pending
SFBA-L2-2CH-100504	SFBA-2CH	child #2	10/05/04	results pending	results pending	results pending
SFBA-L2-3CH-100504	SFBA-3CH	child #3	10/05/04	<0.0430	0.762	0.0144
SFBA-L2-4CH-100504	SFBA-4CH	child #4	10/05/04	0.0109	0.177	0.00362
SFBA-L2-5CH-100504	SFBA-5CH	child #5	10/05/04	0.0251	0.653	0.0126

US EPA ARCHIVE DOCUMENT

Table 5-16
Community Park South Field Baseball Scenario A Air Sample Summary Results

Sample ID	Location	Location Description	Date	PCME Structures (s/cc)	AHERA-like Total Structures (s/cc)	Sensitivity (s/cc)
SFBA-L2-1NA-100504	SFBA-1NA	non-active adult	10/05/04	0.00999	0.0799	0.000999
SFBA-L2-11NA-100504	SFBA-1NA	duplicate of non-active adult	10/05/04	0.00881	0.0509	0.000979
CC2-H6-1CP-100504	CC2-05-1CP	children's playground hi-vol composite sample collected during the 100504 scenarios	10/05/04	0.000991	0.0129	0.000991
CC2-H6-2CP-100504	CC2-05-2CP	children's playground hi-vol composite sample collected during the 100504 scenarios	10/05/04	results pending	results pending	results pending
CC2-L6-1CA-100504	adult #1	composite sample collected during the 100504 scenarios	10/05/04	0.000995	0.0229	0.000995
CC2-L6-1CC-100504	child #1	composite sample collected during the 100504 scenarios	10/05/04	results pending	results pending	results pending
CC2-L6-11CC-100504	child #1	duplicate of child #1 composite sample collected during the 100504 scenarios	10/05/04	results pending	results pending	results pending
CC2-L6-2CC-100504	child #2	composite sample collected during the 100504 scenarios	10/05/04	results pending	results pending	results pending
CC2-L6-3CC-100504	child #3	composite sample collected during the 100504 scenarios	10/05/04	results pending	results pending	results pending
SFBA-L2-1ZB-100504	field blank	field blank	10/05/04	<0.00290	<0.00290	0.000970

PCME fibers = fibers longer than 5 microns with a width between 0.25 and 3 microns, and an aspect ratio (length to width) greater than 3:1
AHERA-like total structures = structures longer than 0.5 microns with an aspect ratio greater than 3:1 (Note this differs somewhat from the strict AHERA structure definition)
Sensitivity = the sample-specific lowest concentration of asbestos the laboratory can reliably detect
s/cc = structures per cubic centimeter

South Field *Baseball Scenario B*. The START conducted activity-based outdoor air sampling during *Baseball Scenario B* at the South Field according to the same scripted schedule that was used for the North Field.

The outline of the field and the positions of the stationary air sample pumps operating during the South Field *Baseball Scenario B* are shown as SFBB-1FD/SFBB-11FD/SFBB-21FD, SFBB-2FD, SFBB-3FD, SFBB-4FD, SFBB-5FD, SFBB-1PG/SFBB-11PG, SFBB-2PG, SFBB-3PG, SFBB-4PG, and SFBB-5PG on Figure 5-12 (Community Park South Field Activity-Based Outdoor Air Sampling Locations–Baseball Scenario B). Two composite samples collected during both baseball scenarios conducted on October 6 are shown as CC5-1CP and CC5-2CP. The position of the mobile meteorological station is shown as SFBB-MS. A summary of results for *Baseball Scenario B* samples is shown in Table 5-17 (Community Park South Field Baseball Scenario B Air Sample Summary Results). See Appendix B for a summary of the results from dust monitoring during this scenario.

South Field *Baseball Scenario C* (Without Field Maintenance). The START conducted activity-based outdoor air sampling during *Baseball Scenario C* at the South Field according to the same scripted schedule that was used for the North Field, except that the maintenance component of the scenario was eliminated. Instead, during the first 30 minutes the five other sampling team members (i.e., all but the sampling team member simulating a parent/adult spectator) conducted infield practice. For the remaining 90 minutes of the scenario, activities were conducted in the same manner as for the North Field.

US EPA ARCHIVE DOCUMENT

Figure 5-12

US EPA ARCHIVE DOCUMENT

US EPA ARCHIVE DOCUMENT

Table 5-17<br>Community Park South Field Baseball Scenario B Air Sample Summary Results						
**Sample ID**	**Location**	**Location Description**	**Date**	**PCME Structures (s/cc)**	**AHERA-like Total Structures (s/cc)**	**Sensitivity (s/cc)**
SFBB-H2-1FD-100604	SFBB-1FD	pitcher's mound hi-vol	10/06/04	results pending	results pending	results pending
SFBB-H2-11FD-100604	SFBB-1FD	duplicate of pitcher's mound hi-vol	10/06/04	0.00488	0.229	0.00488
SFBB-H2-21FD-100604	SFBB-1FD	duplicate of pitcher's mound hi-vol, but with 0.45 µm filter	10/06/04	<0.0202	0.330	0.00674
SFBB-H2-2FD-100604	SFBB-2FD	downwind hi-vol	10/06/04	results pending	results pending	results pending
SFBB-H2-3FD-100604	SFBB-3FD	offset downwind hi-vol	10/06/04	0.00393	0.0167	0.000981
SFBB-H2-4FD-100604	SFBB-4FD	upwind hi-vol	10/06/04	0.00390	0.00586	0.000976
SFBB-H2-5FD-100604	SFBB-5FD	far downwind hi-vol	10/06/04	<0.00314	0.00944	0.00105
SFBB-H2-1PG-100604	SFBB-1PG	children's playground hi-vol	10/06/04	0.00294	0.0128	0.000981
SFBB-H2-11PG-100604	SFBB-1PG	duplicate of children's playground hi-vol	10/06/04	0.00399	0.0270	0.000998
SFBB-H2-2PG-100604	SFBB-2PG	children's playground hi-vol	10/06/04	results pending	results pending	results pending
SFBB-H2-3PG-100604	SFBB-3PG	children's playground hi-vol	10/06/04	<0.00298	0.00798	0.000998
SFBB-H2-4PG-100604	SFBB-4PG	children's playground hi-vol	10/06/04	results pending	results pending	results pending
SFBB-H2-5PG-100604	SFBB-5PG	children's playground hi-vol	10/06/04	results pending	results pending	results pending
SFBB-L2-1CH-100604	SFBB-1CH	child #1	10/06/04	0.00510	0.0510	0.00102
SFBB-L2-2CH-100604	SFBB-2CH	child #2	10/06/04	results pending	results pending	results pending

Table 5-17
Community Park South Field Baseball Scenario B Air Sample Summary Results

Sample ID	Location	Location Description	Date	PCME Structures (s/cc)	AHERA-like Total Structures (s/cc)	Sensitivity (s/cc)
SFBB-L2-3CH-100604	SFBB-3CH	child #3	10/06/04	0.00772	0.0965	0.00193
SFBB-L2-13CH-100604	SFBB-3CH	duplicate of child #3	10/06/04	0.0114	0.190	0.00380
SFBB-L2-4CH-100604	SFBB-4CH	child #4	10/06/04	0.00336	0.168	0.00336
SFBB-L2-5CH-100604	SFBB-5CH	child #5	10/06/04	0.00967	0.161	0.00322
SFBB-L2-1NA-100604	SFBB-1NA	non-active adult	10/06/04	0.0123	0.123	0.00123
CC5-H6-1CP-100604	CC5-1CP	children's playground hi-vol composite sample collected during the 100604 scenarios	10/06/04	results pending	results pending	results pending
CC5-H6-2CP-100604	CC5-2CP	children's playground hi-vol composite sample collected during the 100604 scenarios	10/06/04	results pending	results pending	results pending
CC5-L6-1CA-100604	adult #1	composite sample collected during the 100604 scenarios	10/06/04	0.000980	0.0235	0.000980
CC5-L6-1CB-100604	adult/child #1	composite sample collected during the 100604 scenarios	10/06/04	0.00698	0.0605	0.00116
CC5-L6-2CB-100604	adult/child #2	composite sample collected during the 100604 scenarios	10/06/04	results pending	results pending	results pending
SFBB-L2-1ZB-100604	field blank	field blank	10/06/04	<0.00296	<0.00296	0.000989

PCME fibers = fibers longer than 5 microns with a width between 0.25 and 3 microns, and an aspect ratio (length to width) greater than 3:1
AHERA-like total structures = structures longer than 0.5 microns with an aspect ratio greater than 3:1 (Note this differs somewhat from the strict AHERA structure definition)
Sensitivity = the sample-specific lowest concentration of asbestos the laboratory can reliably detect
s/cc = structures per cubic centimeter

US EPA ARCHIVE DOCUMENT

The outline of the field and the positions of the stationary air sample pumps operating during the South Field *Baseball Scenario C* are shown as SFBC-1FD/SFBC-11FD, SFBC-2FD, SFBC-3FD, SFBC-4FD, SFBC-5FD, SFBC-1PG, SFBC-2PG, SFBC-3PG, SFBC-4FD, and SFBC-5FD on Figure 5-13 (Community Park South Field Activity-Based Outdoor Air Sampling Locations–Baseball Scenario C [Without Field Maintenance]). Two composite samples collected during both baseball scenarios conducted on October 6 are shown as CC5-1CP and CC5-2CP. The position of the mobile meteorological station is shown as SFBC-MS. A summary of results for *Baseball Scenario C* samples is shown in Table 5-18 (Community Park South Field Baseball Scenario C [Without Field Maintenance] Air Sample Summary Results). See Appendix B for a summary of the results from dust monitoring during this scenario.

New York Creek Field Baseball Playing Field. The START collected soil samples to test the moisture level of the field just prior to conducting the scenario at the New York Creek Field (see Table 5-7: Soil Moisture Levels in Activity Area Soils).

The START conducted activity-based outdoor air sampling during the baseball scenario at the New York Creek Field according to the same scripted schedule that was used for the North Field.

The outline of the field and the positions of the stationary air sample pumps operating during the New York Creek Field baseball scenario are shown as NYB-1FD, NYB-2FD/NYB-12FD/NYB-22FD, NYB-3FD, NYB-4FD, NYB-1PG, NYB-2PG, NYB-3PG/NYB-13PG, NYB-4PG, and NYB-5PG on Figure 5-14 (Community Park New York Creek Field Activity-Based Outdoor Air

US EPA ARCHIVE DOCUMENT

Figure 5-13

US EPA ARCHIVE DOCUMENT

US EPA ARCHIVE DOCUMENT

Table 5-18
Community Park South Field Baseball Scenario C (Without Field Maintenance) Air Sample Summary Results

Sample ID	Location	Location Description	Date	PCME Structures (s/cc)	AHERA-like Total Structures (s/cc)	Sensitivity (s/cc)
SFBC-H2-1FD-100604	SFBC-1FD	pitcher's mound hi-vol	10/06/04	0.00194	0.031	0.00101
SFBC-H2-11FD-100604	SFBC-1FD	duplicate of pitcher's mound hi-vol	10/06/04	<0.00296	0.00396	0.000990
SFBC-H2-2FD-100604	SFBC-2FD	downwind hi-vol	10/06/04	results pending	results pending	results pending
SFBC-H2-3FD-100604	SFBC-3FD	offset downwind hi-vol	10/06/04	0.00402	0.0381	0.00100
SFBC-H2-4FD-100604	SFBC-4FD	upwind hi-vol	10/06/04	0.00194	0.00388	0.000969
SFBC-H2-5FD-100604	SFBC-5FD	far downwind hi-vol	10/06/04	0.00675	0.0125	0.000965
SFBC-H2-1PG-100604	SFBC-1PG	children's playground hi-vol	10/06/04	<0.00293	0.0648	0.000981
SFBC-H2-2PG-100604	SFBC-2PG	children's playground hi-vol	10/06/04	0.000971	0.0495	0.000971
SFBC-H2-3PG-100604	SFBC-3PG	children's playground hi-vol	10/06/04	<0.00300	0.0692	0.00100
SFBC-H2-4PG-10-06-04	SFBC-4PG	children’s playground hi-vol	10/06/04	results pending	results pending	results pending
SFBC-H2-5PG-100604	SFBC-5PG	children's playground hi-vol	10/06/04	0.00784	0.0343	0.000981
SFBC-L2-1CH-100604	SFBC-1CH	child #1	10/06/04	results pending	results pending	results pending
SFBC-L2-2CH-100604	SFBC-2CH	child #2	10/06/04	0.0242	0.175	0.00173
SFBC-L2-3CH-100604	SFBC-3CH	child #3	10/06/04	results pending	results pending	results pending

US EPA ARCHIVE DOCUMENT

Table 5-18
Community Park South Field Baseball Scenario C (Without Field Maintenance) Air Sample Summary Results

Sample ID	Location	Location Description	Date	PCME Structures (s/cc)	AHERA-like Total Structures (s/cc)	Sensitivity (s/cc)
SFBC-L2-4CH-100604	SFBC-4CH	child #4	10/06/04	results pending	results pending	results pending
SFBC-L2-5CH-100604	SFBC-5CH	child #5	10/06/04	results pending	results pending	results pending
SFBC-L2-1NA-100604	SFBC-1NA	non-active adult	10/06/04	0.00993	0.0546	0.000993
CC5-H6-1CP-100604	CC5-1CP	children's playground hi-vol composite sample collected during the 100604 scenarios	10/06/04	results pending	results pending	results pending
CC5-H6-2CP-100604	CC5-2CP	children's playground hi-vol composite sample collected during the 100604 scenarios	10/06/04	results pending	results pending	results pending
CC5-L6-1CA-100604	adult #1	composite sample collected during the 100604 scenarios	10/06/04	0.000980	0.0235	0.000980
CC5-L6-1CB-100604	adult/child #1	composite sample collected during the 100604 scenarios	10/06/04	0.00698	0.0605	0.00116
CC5-L6-2CB-100604	adult/child #2	composite sample collected during the 100604 scenarios	10/06/04	results pending	results pending	results pending
SFBC-L2-1ZB-100604	field blank	field blank	10/06/04	<0.00296	<0.00296	0.000991

PCME fibers = fibers longer than 5 microns with a width between 0.25 and 3 microns, and an aspect ratio (length to width) greater than 3:1
AHERA-like total structures = structures longer than 0.5 microns with an aspect ratio greater than 3:1 (Note this differs somewhat from the strict AHERA structure definition)
Sensitivity = the sample-specific lowest concentration of asbestos the laboratory can reliably detect
s/cc = structures per cubic centimeter

Sampling Locations–Baseball Scenario). Two composite samples collected during both baseball scenarios conducted on October 7 are shown as CC6-1CP and CC6-2CP. The position of the mobile meteorological station is shown as NYB-MS. A summary of results for the baseball scenario samples is shown in Table 5-19 (Community Park New York Creek Field Baseball Scenario Air Sample Summary Results). See Appendix B for a summary of the results from dust monitoring during this scenario.

Lower Soccer Field at the Community Park (Between North Field and South Field. The START collected soil samples to test the moisture level of the field just prior to conducting the scenario at the Lower Soccer Field (see Table 5-7: Soil Moisture Levels in Activity Area Soils).

The soccer scenario activity was conducted in three 40-minute sequential sessions on three separate rectangular areas where the grass was noted to be slightly barer than on other parts of the field. The START conducted activity-based outdoor air sampling during the soccer scenario at the lower soccer field on October 7, 2004, according to the following scripted schedule:

- For the entire 2-hour scenario, one member of the sampling team simulated the activities of an adult/parent spectator sitting near the edge or walking around and occasionally within the area of play. This member of the sampling team would sometimes retrieve a ball kicked out of the area of play. The intake for the air collection filter cassette worn by this sampling team member was set at a height of about 5 feet.

US EPA ARCHIVE DOCUMENT

Figure 5-14

US EPA ARCHIVE DOCUMENT

US EPA ARCHIVE DOCUMENT

Table 5-19
Community Park New York Creek Field Baseball Scenario Air Sample Summary Results

Sample ID	Location	Location Description	Date	PCME Structures (s/cc)	AHERA-like Total Structures (s/cc)	Sensitivity (s/cc)
NYB-H2-1FD-100704	NYB-1FD	pitcher's mound hi-vol	10/07/04	results pending	results pending	results pending
NYB-H2-2FD-100704	NYB-2FD	downwind hi-vol (somewhat crosswind)	10/07/04	results pending	results pending	results pending
NYB-H2-12FD-100704	NYB-2FD	duplicate of downwind hi-vol (somewhat crosswind)	10/07/04	results pending	results pending	results pending
NYB-H2-22FD-100704	NYB-2FD	duplicate of downwind hi-vol (somewhat crosswind), but with 0.45 μm filter	10/07/04	0.000983	0.00786	0.000983
NYB-H2-3FD-100704	NYB-3FD	offset downwind hi-vol (somewhat crosswind)	10/07/04	results pending	results pending	results pending
NYB-H2-4FD-100704	NYB-4FD	upwind hi-vol (somewhat crosswind)	10/07/04	<0.00297	<0.00297	0.000992
NYB-H2-5FD-100704	NYB-5FD	far downwind hi-vol (somewhat crosswind)	10/07/04	0.00194	0.00291	0.000971
NYB-H2-1PG-100704	NYB-1PG	children's playground hi-vol	10/07/04	<0.00290	<0.00290	0.000969
NYB-H2-2PG-100704	NYB-2PG	children's playground hi-vol	10/07/04	<0.00291	0.000973	0.000973
NYB-H2-3PG-100704	NYB-3PG	children's playground hi-vol	10/07/04	<0.00294	0.000983	0.000983
NYB-H2-13PG-100704	NYB-3PG	duplicate of children's playground hi-vol	10/07/04	<0.00289	0.000968	0.000968
NYB-H2-4PG-100704	NYB-4PG	children's playground hi-vol	10/07/04	<0.00287	<0.00287	0.000960
NYB-H2-5PG-100704	NYB-5PG	children's playground hi-vol	10/07/04	0.000982	0.000982	0.000982

US EPA ARCHIVE DOCUMENT

Table 5-19
Community Park New York Creek Field Baseball Scenario Air Sample Summary Results

Sample ID	Location	Location Description	Date	PCME Structures (s/cc)	AHERA-like Total Structures (s/cc)	Sensitivity (s/cc)
NYB-L2-1CH-100704	NYB-1CH	child #1	10/07/04	results pending	results pending	results pending
NYB-L2-11CH-100704	NYB-1CH	duplicate of child #1	10/07/04	results pending	results pending	results pending
NYB-L2-2CH-100704	NYB-2CH	child #2	10/07/04	0.04	0.032	0.00101
NYB-L2-3CH-100704	NYB-3CH	child #3	10/07/04	results pending	results pending	results pending
NYB-L2-4CH-100704	NYB-4CH	child #4	10/07/04	results pending	results pending	results pending
NYB-L2-5CH-100704	NYB-5CH	child #5	10/07/04	results pending	results pending	results pending
NYB-L2-1NA-100704	NYB-1NA	non-active adult	10/07/04	0.000995	0.00597	0.000995
CC6-H6-1CP-100704	children's playground hi-vol	composite sample collected during the 100704 scenarios	10/07/04	<0.00296	<0.00296	0.000992
CC6-H6-2CP-100704	children's playground hi-vol	composite sample collected during the 100704 scenarios	10/07/04	0.000985	0.00197	0.000985
CC6-L6-1CA-100704	adult #1	composite sample collected during the 100704 scenarios	10/07/04	0.000965	0.00579	0.000965

US EPA ARCHIVE DOCUMENT

Table 5-19
Community Park New York Creek Field Baseball Scenario Air Sample Summary Results

Sample ID	Location	Location Description	Date	PCME Structures (s/cc)	AHERA-like Total Structures (s/cc)	Sensitivity (s/cc)
CC6-L6-1CB-100704	adult/child #1	composite sample collected during the 100704 scenarios	10/07/04	results pending	results pending	results pending
CC6-L6-2CB-100704	adult/child #2	composite sample collected during the 100704 scenarios	10/07/04	results pending	results pending	results pending
CC6-L6-3CB-100704	adult/child #3	composite sample collected during the 100704 scenarios	10/07/04	results pending	results pending	results pending
NYB-H2-1ZP-100704	performance evaluation	performance evaluation	10/07/04	0.0375	0.174	0.00170
NYB-L2-1ZP-100704	performance evaluation	performance evaluation	10/07/04	0.110	1.09	0.0110
NYB-L2-1ZB-100704	field blank	field blank	10/07/04	<0.00299	<0.00299	0.00100

PCME fibers = fibers longer than 5 microns with a width between 0.25 and 3 microns, and an aspect ratio (length to width) greater than 3:1
AHERA-like total structures = structures longer than 0.5 microns with an aspect ratio greater than 3:1 (Note this differs somewhat from the strict AHERA structure definition)
Sensitivity = the sample-specific lowest concentration of asbestos the laboratory can reliably detect
s/cc = structures per cubic centimeter

- For the entire 2-hour scenario, five other members of the sampling team passed soccer balls back and forth to each other. They sometimes formed a small circle to practice passing; other times they dribbled the ball across the field with other sampling team members close by. They spent 40 minutes on each of three areas of the field. The intakes for the air collection filter cassettes worn by these sampling team members were set at a height of about 3 feet.

The three areas within which the START conducted the activity are outlined and the positions of the stationary air sample pumps operating during the soccer scenario are shown as CPS-1FD, CPS-2FD, CPS-3FD, CPS-4FD, CPS-5FD, CPS-1PG, CPS-2PG, CPS-3PG, CPS-4PG, and CPS-5PG on Figure 5-15 (Community Park Lower Soccer Field Activity-Based Outdoor Air Sampling Locations–Soccer Scenario). Two composite samples collected during both baseball scenarios conducted on October 7 are shown as CC6-1CP and CC6-2CP. The position of the mobile meteorological station is shown as CPS-MS. A summary of results for the baseball scenario samples is shown in Table 5-20 (Community Park Lower Soccer Field Soccer Scenario Air Sample Summary Results). See Appendix B for a summary of the results from dust monitoring during this scenario.

US EPA ARCHIVE DOCUMENT

Figure 5-15

US EPA ARCHIVE DOCUMENT

US EPA ARCHIVE DOCUMENT

Table 5-20
Community Park Lower Soccer Field Soccer Scenario Air Sample Summary Results

Sample ID	Location	Location Description	Date	PCME Structures (s/cc)	AHERA-like Total Structures (s/cc)	Sensitivity (s/cc)
CPS-H2-1FD-100704	CPS-1FD	on-field hi-vol	10/07/04	0.000933	0.000933	0.000933
CPS-H2-2FD-100704	CPS-2FD	on-field hi-vol	10/07/04	0.00199	0.000994	0.000994
CPS-H2-3FD-100704	CPS-3FD	on-field hi-vol	10/07/04	<0.00294	0.00491	0.000982
CPS-H2-4FD-100704	CPS-4FD	upwind hi-vol	10/07/04	0.00193	0.00675	0.000965
CPS-H2-14FD-100704	CPS-4FD	duplicate of upwind hi-vol	10/07/04	0.000974	0.00292	0.000974
CPS-H2-5FD-100704	CPS-5FD	far downwind hi-vol	10/07/04	0.000977	0.00391	0.000977
CPS-H2-1PG-100704	CPS-1PG	children's playground hi-vol	10/07/04	<0.00319	0.00531	0.000971
CPS-H2-2PG-100704	CPS-2PG	children's playground hi-vol	10/07/04	<0.00299	0.00300	0.000999
CPS-H2-3PG-100704	CPS-3PG	children's playground hi-vol	10/07/04	<0.00292	0.00195	0.000977
CPS-H2-4PG-100704	CPS-4PG	children's playground hi-vol	10/07/04	0.00192	0.00383	0.000959
CPS-H2-5PG-100704	CPS-5PG	children's playground hi-vol	10/07/04	0.00193	0.00770	0.000963
CPS-L2-1CH-100704	CPS-1CH	child #1	10/07/04	0.00683	0.0234	0.000988
CPS-L2-2CH-100704	CPS-2CH	child #2	10/07/04	0.00300	0.00699	0.000999
CPS-L2-3CH-100704	CPS-3CH	child #3	10/07/04	0.00501	0.0240	0.00100
CPS-L2-4CH-100704	CPS-4CH	child #4	10/07/04	0.00400	0.0110	0.000999
CPS-L2-5CH-100704	CPS-5CH	child #5	10/07/04	0.00695	0.0149	0.000993
CPS-L2-15CH-100704	CPS-5CH	duplicate of child #5	10/07/04	0.00996	0.0249	0.000996

US EPA ARCHIVE DOCUMENT

Table 5-20
Community Park Lower Soccer Field Soccer Scenario Air Sample Summary Results

Sample ID	Location	Location Description	Date	PCME Structures (s/cc)	AHERA-like Total Structures (s/cc)	Sensitivity (s/cc)
CPS-L2-1NA-100704	non-active adult	non-active adult	10/07/04	0.000994	0.00298	0.000994
CC6-H6-1CP-100704	children's playground hi-vol	composite sample collected during the 100704 scenarios	10/07/04	<0.00296	<0.00296	0.000992
CC6-H6-2CP-100704	children's playground hi-vol	composite sample collected during the 100704 scenarios	10/07/04	0.000985	0.00197	0.000985
CC6-L6-1CA-100704	adult #1	composite sample collected during the 100704 scenarios	10/07/04	0.000965	0.00579	0.000965
CC6-L6-1CB-100704	adult/child #1	composite sample collected during the 100704 scenarios	10/07/04	results pending	results pending	results pending
CC6-L6-2CB-100704	adult/child #2	composite sample collected during the 100704 scenarios	10/07/04	results pending	results pending	results pending
CC6-L6-3CB-100704	adult/child #3	composite sample collected during the 100704 scenarios	10/07/04	results pending	results pending	results pending
CPS-H2-1ZB-100704	field blank	field blank	10/07/04	<0.00288	<0.00288	0.000962
CPS-L2-FB-100704	filter blank	filter blank	10/07/04	<0.00297	<0.00297	0.000994

PCME fibers = fibers longer than 5 microns with a width between 0.25 and 3 microns, and an aspect ratio (length to width) greater than 3:1
AHERA-like total structures = structures longer than 0.5 microns with an aspect ratio greater than 3:1 (Note this differs somewhat from the strict AHERA structure definition)
Sensitivity = the sample-specific lowest concentration of asbestos the laboratory can reliably detect
s/cc = structures per cubic centimeter

US EPA ARCHIVE DOCUMENT

5.4.2.5 New York Creek Nature Trail

The START conducted activity-based outdoor air sampling during one biking and two jogging/walking scenarios along the New York Creek Nature Trail. The trail was officially closed to public use during the activity-based sampling, but a few members of the public did enter the trail during sampling in spite of its closure.

The plan for the trail scenarios, as described in the *El Dorado Hills Naturally Occurring Asbestos Multimedia Exposure Assessment, El Dorado Hills, California, Activity-Based Outdoor Air Sampling of Community Park and Schools Field Sampling Plan*, had called for the START to conduct three jogging/walking scenarios. Changes to the schedule for sampling at Silva Valley Elementary School impacted the schedule for scenarios that had been planned for the following day, necessitating that one of the three jogging/walking scenarios on the New York Creek Nature Trail be dropped.

Biking Scenario. During the biking scenario on the New York Creek Nature Trail, five members of the sampling team rode bicycles along the southern end of the trail from Harvard Way through the Community Park and slightly beyond its northern border at St. Andrews Drive. The START placed five high-flow stationary air sample pumps along the trail where the bicyclists rode. The START also placed two other high-flow stationary air sample pumps along the northern end of the trail where there was no activity from the bicyclists. The intakes for the high-flow stationary air sample pumps were positioned at a height of about 5 feet.

The START conducted the biking scenario on October 5, 2004, according to the following scripted schedule:

- For the entire 2-hour scenario, one member of the sampling team simulated the activities of an adult/parent walking and standing along the trail. The intake for the air collection filter cassette worn by this sampling team member was set at a height of about 5 feet.
- For the entire 2-hour scenario, five other members of the sampling team rode bicycles along the trail. The intakes for the air collection filter cassettes worn by these sampling team members were set at a height of about 3 feet. Four sampling team members rode in two separate pairs, and one rode alone, although the sets of riders passed one another periodically during the scenario. For the bicyclists who rode in pairs, the relative position (i.e., leader and follower) remained constant throughout the entire scenario. Table 5-21 (Biking Scenario Positions) shows the configuration of the bicyclists during the scenario.

Table 5-21
Biking Scenario Positions

Pair A	Leader<br>Follower	BIK-2CH<br>BIK-1CH
Pair B	Leader<br>Follower	BIK-3CH<br>BIK-5CH
Solitary Rider		BIK-4CH

The portion of the trail along which the START conducted the biking activity is shown and the positions of the stationary air sample pumps operating during the biking scenario are shown as BIK-1TR/BIK-11TR, BIK-2TR, BIK-3TR, BIK-4TR, BIK-5TR, BIK-6TR, and BIK-7TR on Figure 5-16 (New York Creek Nature Trail Activity-Based Outdoor Air Sampling Locations–Biking Scenario). A summary of results for the biking scenario samples is

US EPA ARCHIVE DOCUMENT

Figure 5-16

US EPA ARCHIVE DOCUMENT

Table 5-22
New York Creek Nature Trail Biking Scenario Air Sample Summary Results

Sample ID	Location	Location Description	Date	PCME Structures (s/cc)	AHERA-like Total Structures (s/cc)	Sensitivity (s/cc)
BIK-H2-1TR-100504	BIK-1TR	trail position #BIK1 hi-vol	10/05/04	0.000986	0.00197	0.00986
BIK-H2-11TR-100504	BIK-1TR	duplicate of trail position #BIK1 hi-vol	10/05/04	0.00192	0.00192	0.000960
BIK-H2-2TR-100504	BIK-2TR	trail position #BIK2 hi-vol	10/05/04	0.00487	0.0107	0.000974
BIK-H2-3TR-100504	BIK-3TR	trail position #BIK3 hi-vol	10/05/04	0.00200	0.00200	0.00100
BIK-H2-4TR-100504	BIK-4TR	trail position #BIK4 hi-vol	10/05/04	0.00576	0.00864	0.000960
BIK-H2-5TR-100504	BIK-5TR	trail position #BIK5 hi-vol	10/05/04	0.00482	0.00675	0.000965
BIK-H2-6TR-100504	BIK-6TR	trail position #BIK6 hi-vol	10/05/04	<0.00292	0.00293	0.000977
BIK-H2-7TR-100504	BIK-7TR	trail position #BIK7 hi-vol	10/05/04	0.00100	0.0110	0.00100
BIK-L2-1CH-100504	BIK-1CH	child #1	10/05/04	0.0668	0.145	0.00145
BIK-L2-2CH-100504	BIK-2CH	child #2	10/05/04	0.00694	0.0228	0.000991
BIK-L2-3CH-100504	BIK-3CH	child #3	10/05/04	0.000998	0.0170	0.000998
BIK-L2-13CH-100504	BIK-3CH	duplicate of child #3	10/05/04	0.0140	0.0470	0.000999
BIK-L2-4CH-100504	BIK-4CH	child #4	10/05/04	0.0178	0.0426	0.000991
BIK-L2-5CH-100504	BIK-5CH	child #5	10/05/04	0.0309	0.0639	0.00101
BIK-L2-1NA-100504	BIK-1NA	non-active adult	10/05/04	0.00198	0.00694	0.000992

PCME fibers = fibers longer than 5 microns with a width between 0.25 and 3 microns, and an aspect ratio (length to width) greater than 3:1
AHERA-like total structures = structures longer than 0.5 microns with an aspect ratio greater than 3:1 (Note this differs somewhat from the strict AHERA structure definition)
Sensitivity = the sample-specific lowest concentration of asbestos the laboratory can reliably detect
s/cc = structures per cubic centimeter

US EPA ARCHIVE DOCUMENT

shown in Table 5-22 (New York Creek Nature Trail Biking Scenario Air Sample Summary Results). See Appendix B for a summary of the results from dust monitoring during this scenario.

Jogging/Walking Scenario A. During the first jogging/walking scenario on the New York Creek Nature Trail, five members of the sampling team jogged and walked along the entire length of the trail from Harvard Way through the Community Park and up to the end of the trail near Jackson Elementary School. The START placed five high-flow stationary air sample pumps along the length of the trail. The intakes for the high-flow stationary air sample pumps were positioned at a height of about 5 feet.

The START conducted the *Jogging/Walking Scenario A* on October 6, 2004, according to the following scripted schedule:

- For the entire 2-hour scenario, one member of the sampling team simulated the activities of an adult/parent walking and standing along the trail. The intake for the air collection filter cassette worn by this sampling team member was set at a height of about 5 feet.
- For the entire 2-hour scenario, five members of the sampling team simulated the activities of an adult/parent jogging and walking along the trail. The intakes for the air collection filter cassettes worn by these sampling team members were set at a height of about 5 feet. Three sampling team members jogged and walked in a group with one jogger in the lead position and two others following far behind (about 20 to 30 feet) but staying fairly close to one another. Two other sampling team members jogged as a pair close to each other.
- For each jogger/walker group of two or three sampling team members, the relative positions of the joggers remained the same throughout the scenario. That is, for the trio, the far

US EPA ARCHIVE DOCUMENT

leader, second leader, and follower remained in those positions throughout the entire scenario. For the pair, the leader and the follower remained in those positions. Table 5-23 (Positions for Jogging/Walking Scenario A) shows the configuration of the jogger/walkers during the first jogging/walking scenario.

Table 5-23
Positions for Jogging/Walking Scenario A

Trio	Far Leader<br>Second Leader<br>Follower	JOGA-1AD<br>JOGA-3AD<br>JOGA-2AD
Pair	Leader<br>Follower	JOGA-4AD<br>JOGA-5AD

The entire length of the trail along which the START conducted the jogging/walking activity is shown and the positions of the stationary air sample pumps operating during *Jogging/Walking Scenario A* are shown as JOGA-1TR, JOGA-2TR, JOGA-3TR, JOGA-4TR, and JOGA-5TR on Figure 5-17 (New York Creek Nature Trail Activity-Based Outdoor Air Sampling Locations–Jogging/Walking Scenarios). A summary of results for *Jogging/Walking Scenario A* samples is shown in Table 5-24 (New York Creek Nature Trail Jogging/Walking Scenario A Air Sample Summary Results). See Appendix B for a summary of the results from dust monitoring during this scenario.

Jogging/Walking Scenario B. The START conducted the *Jogging/Walking Scenario B* on October 7, 2004, according to the same scripted schedule that was used for *Jogging/Walking Scenario A*. Table 5-25 (Positions for Jogging/Walking Scenario B) shows the configuration of the jogger/walkers during the second jogging/walking scenario.

US EPA ARCHIVE DOCUMENT

Figure 5-17

US EPA ARCHIVE DOCUMENT

US EPA ARCHIVE DOCUMENT

Table 5-24
New York Creek Nature Trail Jogging/Walking Scenario A Air Sample Summary Results

Sample ID	Location	Location Description	Date	PCME Structures (s/cc)	AHERA-like Total Structures (s/cc)	Sensitivity (s/cc)
JOGA-H2-1TR-100604	JOGA-1TR	trail position #JOG1 hi-vol	10/06/04	0.00198	0.0109	0.000992
JOGA-H2-2TR-100604	JOGA-2TR	trail position #JOG2 hi-vol	10/06/04	0.00395	0.0128	0.000986
JOGA-H2-3TR-100604	JOGA-3TR	trail position #JOG3 hi-vol	10/06/04	0.0126	0.0416	0.000967
JOGA-H2-4TR-100604	JOGA-4TR	trail position #JOG4 hi-vol	10/06/04	0.00198	0.00990	0.000990
JOGA-H2-5TR-100604	JOGA-5TR	trail position #JOG5 hi-vol	10/06/04	<0.00295	0.00296	0.000986
JOGA-L2-1AD-100604	JOGA-1AD	adult #1	10/06/04	0.00299	0.0180	0.000998
JOGA-L2-2AD-100604	JOGA-2AD	adult #2	10/06/04	0.0249	0.0588	0.000996
JOGA-L2-3AD-100604	JOGA-3AD	adult #3	10/06/04	0.0208	0.0367	0.000992
JOGA-L2-4AD-100604	JOGA-4AD	adult #4	10/06/04	0.0110	0.0280	0.000998
JOGA-L2-5AD-100604	JOGA-5AD	adult #5	10/06/04	0.0180	0.0319	0.000998
JOGA-L2-1NA-100604	JOGA-1NA	non-active adult	10/06/04	0.0120	0.0240	0.00100

PCME fibers = fibers longer than 5 microns with a width between 0.25 and 3 microns, and an aspect ratio (length to width) greater than 3:1
AHERA-like total structures = structures longer than 0.5 microns with an aspect ratio greater than 3:1 (Note this differs somewhat from the strict AHERA structure definition)
Sensitivity = the sample-specific lowest concentration of asbestos the laboratory can reliably detect
s/cc = structures per cubic centimeter

Table 5-25<br>Positions for Jogging/Walking Scenario B		
Trio	Far Leader<br>Second Leader<br>Follower	JOGB-1AD<br>JOGB-2AD<br>JOGB-3AD
Pair	Leader<br>Follower	JOGB-4AD<br>JOGB-5AD

The entire length of the trail along which the START conducted the jogging/walking activity is shown and the positions of the stationary air sample pumps operating during *Jogging/Walking Scenario B* are shown as JOGB-1TR, JOGB-2TR/JOGB-12TR, JOGB-3TR, JOGB-4TR, and JOGB-5TR on Figure 5-17 (New York Creek Nature Trail Activity-Based Outdoor Air Sampling Locations–Jogging/Walking Scenarios). A summary of results for *Jogging/Walking Scenario B* samples is shown in Table 5-26 (New York Creek Nature Trail Jogging/Walking Scenario B Air Sample Summary Results). See Appendix B for a summary of the results from dust monitoring during this scenario.

5.4.2.6 Jackson Elementary School

The START conducted activity-based air sampling during three scenarios at the garden and outdoor classroom, the grass-covered playing field, and two paved play areas. Because the paved play areas selected for the activity were physically separate from one another, the START placed additional high-flow air sample pumps in stationary positions in each area, but only operated them within each area for the 1 hour of the scenario when activity was conducted there.

US EPA ARCHIVE DOCUMENT

US EPA ARCHIVE DOCUMENT

Table 5-26
New York Creek Nature Trail Jogging/Walking Scenario B Air Sample Summary Results

Sample ID	Location	Location Description	Date	PCME Structures (s/cc)	AHERA-like Total Structures (s/cc)	Sensitivity (s/cc)
JOGB-H2-1TR-100704	JOGB-1TR	trail position #JOG1 hi-vol	10/07/04	0.00290	0.00484	0.000968
JOGB-H2-2TR-100704	JOGB-2TR	trail position #JOG2 hi-vol	10/07/04	0.00288	0.00288	0.000959
JOGB-H2-12TR-100704	JOGB-2TR	duplicate of trail position #JOG2 hi-vol	10/07/04	<0.00291	0.00292	0.000974
JOGB-H2-3TR-100704	JOGB-3TR	trail position #JOG3 hi-vol	10/07/04	0.000975	0.000975	0.000975
JOGB-H2-4TR-100704	JOGB-4TR	trail position #JOG4 hi-vol	10/07/04	<0.00293	0.00393	0.000982
JOGB-H2-5TR-100704	JOGB-5TR	trail position #JOG5 hi-vol	10/07/04	0.000980	0.00490	0.000980
JOGB-L2-1AD-100704	JOGB-1AD	adult #1	10/07/04	0.00701	0.0280	0.00100
JOGB-L2-2AD-100704	JOGB-2AD	adult #2	10/07/04	0.0159	0.0458	0.000995
JOGB-L2-12AD-100704	JOGB-2AD	duplicate of adult #2	10/07/04	0.0110	0.0330	0.00100
JOGB-L2-3AD-100704	JOGB-3AD	adult #3	10/07/04	0.0441	0.123	0.00227
JOGB-L2-4AD-100704	JOGB-4AD	adult #4	10/07/04	0.00792	0.0158	0.000990
JOGB-L2-5AD-100704	JOGB-5AD	adult #5	10/07/04	0.00598	0.0179	0.000997
JOGB-L2-1NA-100704	JOGB-1NA	non-active adult	10/07/04	<0.00295	0.00591	0.000985

PCME fibers = fibers longer than 5 microns with a width between 0.25 and 3 microns, and an aspect ratio (length to width) greater than 3:1
AHERA-like total structures = structures longer than 0.5 microns with an aspect ratio greater than 3:1 (Note this differs somewhat from the strict AHERA structure definition)
Sensitivity = the sample-specific lowest concentration of asbestos the laboratory can reliably detect
s/cc = structures per cubic centimeter

Garden and Outdoor Classroom. The START collected samples to test the moisture level of the soil just prior to conducting the scenario at the Garden and Outdoor Classroom (see Table 5-7: Moisture Levels in Activity Area Soils).

The START conducted activity-based outdoor air sampling during the garden and outdoor classroom scenario on October 10, 2004, according to the following scripted schedule:

- For the entire 2-hour scenario, one member of the sampling team simulated the activities of an adult/teacher sitting, standing, walking, and gardening. The intake for the air collection filter cassette worn by this sampling team member was set at a height of about 5 feet.
- For the entire 2-hour scenario, five other members of the sampling team simulated the activities of a students sitting, standing, walking, and gardening. The intakes for the air collection filter cassettes worn by these sampling team members were set at a height of about 3 feet.
- Members of the sampling team conducted the activity in pairs, each pair spending 20 minutes twice during the 2 hours at each of three areas within the garden and outdoor classroom. The three areas included the garden plots and area in front of the garden shed in the northwest corner, the benches in front of the chalkboard, and the tables and area in front of the garden shed in the southwest corner.
- At the garden plots and area in front of the garden shed in the northwest corner, activity included using rakes and hoes and scooping dirt into a bucket and dumping it out. At the benches in front of the chalkboard, activity included sitting and shuffling feet and walking around. At the tables and area in front of the garden shed in the southwest corner, activity also included

US EPA ARCHIVE DOCUMENT

using rakes and hoes and scooping dirt into a bucket and dumping it out.

The outline of the garden and outdoor classroom area and the positions of the stationary air sample pumps operating during the scenario are shown as JEG-1FD, JEG-2FD, JEG-3FD, JEG-4FD, and JEG-5FD on Figure 5-18 (Jackson Elementary School Garden and Outdoor Classroom Activity-Based Outdoor Air Sampling Locations–Garden/Outdoor Classroom Scenario). The position of the mobile meteorological station is shown as JEG-MS. A summary of results for the garden/outdoor classroom scenario samples is shown in Table 5-27 (Jackson Elementary School Garden/Outdoor Classroom Scenario Air Sample Summary Results). See Appendix B for a summary of the results from dust monitoring during this scenario.

Grass-Covered Playing Field–Soccer Scenario. The START collected soil samples to test the moisture level just prior to conducting the soccer scenario at the grass-covered playing field at Jackson Elementary School (see Table 5-7: Moisture Levels in Activity Area Soils).

The soccer scenario activity was conducted in twelve 10-minute sequential sessions on three separate rectangular areas where the grass was noted to be slightly barer than on other parts of the field. (The sampling team members conducted four 10-minute sessions for a total of 40 minutes in each of the three areas.) The START conducted activity-based outdoor air sampling during the soccer scenario at the grass-covered playing field on October 10, 2004, according to the following scripted schedule:

US EPA ARCHIVE DOCUMENT

Figure 5-18

US EPA ARCHIVE DOCUMENT

Table 5-27
Jackson Elementary School Garden/Outdoor Classroom Scenario Air Sample Summary Results

Sample ID	Location	Location Description	Date	PCME Structures (s/cc)	AHERA-like Total Structures (s/cc)	Sensitivity (s/cc)
JEG-H2-1FD-101004	JEG-1FD	garden area hi-vol	10/10/04	results pending	results pending	results pending
JEG-H2-2FD-101004	JEG-2FD	garden area hi-vol	10/10/04	results pending	results pending	results pending
JEG-H2-3FD-101004	JEG-3FD	garden area hi-vol	10/10/04	<0.00299	0.00100	0.00100
JEG-H2-4FD-101004	JEG-4FD	upwind hi-vol	10/10/04	0.000973	0.00292	0.000973
JEG-H2-5FD-101004	JEG-5FD	far downwind hi-vol	10/10/04	results pending	results pending	results pending
JEG-L2-1AD-101004	JEG-1AD	adult #1	10/10/04	results pending	results pending	results pending
JEG-L2-1CH-101004	JEG-1CH	child #1	10/10/04	results pending	results pending	results pending
JEG-L2-2CH-101004	JEG-2CH	child #2	10/10/04	results pending	results pending	results pending
JEG-L2-3CH-101004	JEG-3CH	child #3	10/10/04	results pending	results pending	results pending
JEG-L2-4CH-101004	JEG-4CH	child #4	10/10/04	results pending	results pending	results pending
JEG-L2-5CH-101004	JEG-5CH	child #5	10/10/04	results pending	results pending	results pending

US EPA ARCHIVE DOCUMENT

US EPA ARCHIVE DOCUMENT

Table 5-27
Jackson Elementary School Garden/Outdoor Classroom Scenario Air Sample Summary Results

Sample ID	Location	Location Description	Date	PCME Structures (s/cc)	AHERA-like Total Structures (s/cc)	Sensitivity (s/cc)
JEG-L2-15CH-101004	JEG-5CH	duplicate of child #5	10/10/04	results pending	results pending	results pending
CC9-L6-1CA-101004	adult #1	composite sample collected during the 101004 scenarios	10/10/04	results pending	results pending	results pending
CC9-L6-11CA-101004	adult #1	duplicate of adult #1 composite sample collected during the 101004 scenarios	10/10/04	results pending	results pending	results pending
CC9-L6-1CC-101004	child #1	composite sample collected during the 101004 scenarios	10/10/04	<0.00297	0.000992	0.000992
CC9-L6-2CC-101004	child #2	composite sample collected during the 101004 scenarios	10/10/04	results pending	results pending	results pending
CC9-L6-3CC-101004	child #3	composite sample collected during the 101004 scenarios	10/10/04	results pending	results pending	results pending
JEG-L2-1ZB-101004	field blank	field blank	10/10/04	<0.00298	<0.00298	0.000997
JEG-L2-FB-101004	filter blank	filter blank	10/10/04	<0.00298	<0.00298	0.000995

PCME fibers = fibers longer than 5 microns with a width between 0.25 and 3 microns, and an aspect ratio (length to width) greater than 3:1
AHERA-like total structures = structures longer than 0.5 microns with an aspect ratio greater than 3:1 (Note this differs somewhat from the strict AHERA structure definition)
Sensitivity = the sample-specific lowest concentration of asbestos the laboratory can reliably detect
s/cc = structures per cubic centimeter

- For the entire 2-hour scenario, one member of the sampling team simulated the activities of an adult/parent spectator sitting near the edge or walking around and occasionally within the area of play. This member of the sampling team would sometimes retrieve a ball kicked out of the area of play. The intake for the air collection filter cassette worn by this sampling team member was set at a height of about 5 feet.
- For the entire 2-hour scenario, five other members of the sampling team passed soccer balls back and forth to each other. They sometimes formed a small circle to practice passing; other times they dribbled the ball across the field with other sampling team members close by. They spent 40 minutes on each of three areas of the field. The intakes for the air collection filter cassettes worn by these sampling team members were set at a height of about 3 feet.

The three areas within which the START conducted the activity are outlined and the positions of the stationary air sample pumps operating during the soccer scenario are shown as JEP-1FD, JEP-2FD, JEP-3FD, JEP-4FD, and JEP-5FD/JEP-15FD on Figure 5-19 (Jackson Elementary School Grass-Covered Playing Field Activity-Based Outdoor Air Sampling Locations–Soccer Scenario). The position of the mobile meteorological station is shown as JEP-MS. A summary of results for the soccer scenario samples is shown in Table 5-28 (Jackson Elementary School Soccer Scenario Air Sample Summary Results). See Appendix B for a summary of the results from dust monitoring during this scenario.

US EPA ARCHIVE DOCUMENT

Figure 5-19

US EPA ARCHIVE DOCUMENT

US EPA ARCHIVE DOCUMENT

Table 5-28
Jackson Elementary School Soccer Scenario B Air Sample Summary Results

Sample ID	Location	Location Description	Date	PCME Structures (s/cc)	AHERA-like Total Structures (s/cc)	Sensitivity (s/cc)
JEP-H2-1FD-101004	JEP-1FD	on-field hi-vol	10/10/04	<0.00271	<0.00271	0.000905
JEP-H2-2FD-101004	JEP-2FD	on-field hi-vol	10/10/04	0.00281	0.00281	0.000938
JEP-H2-3FD-101004	JEP-3FD	on-field hi-vol	10/10/04	0.00196	0.00294	0.000980
JEP-H2-4FD-101004	JEP-4FD	upwind hi-vol	10/10/04	<0.00297	<0.00297	0.000993
JEP-H2-5FD-101004	JEP-5FD	far downwind hi-vol	10/10/04	<0.00271	0.000907	0.000907
JEP-H2-15FD-101004	JEP-5FD	duplicate of far downwind hi-vol	10/10/04	<0.00294	0.000982	0.000982
JEP-L2-1CH-101004	JEP-1CH	child #1	10/10/04	<0.00298	0.00796	0.000995
JEP-L2-2CH-101004	JEP-2CH	child #2	10/10/04	0.000980	0.00588	0.000980
JEP-L2-3CH-101004	JEP-3CH	child #3	10/10/04	0.00299	0.00998	0.000998
JEP-L2-4CH-101004	JEP-4CH	child #4	10/10/04	0.00200	0.00399	0.000999
JEP-L2-5CH-101004	JEP-5CH	child #5	10/10/04	<0.00299	0.00499	0.000998
JEP-L2-1NA-101004	JEP-1NA	non-active adult	10/10/04	<0.00297	0.000993	0.000993
CC9-L6-1CA-101004	adult #1	composite sample collected during the 101004 scenarios	10/10/04	results pending	results pending	results pending
CC9-L6-11CA-101004	adult #1	duplicate of adult #1 composite sample collected during the 101004 scenarios	10/10/04	results pending	results pending	results pending
CC9-L6-1CC-101004	child #1	composite sample collected during the 101004 scenarios	10/10/04	<0.00297	0.000992	0.000992

Table 5-28
Jackson Elementary School Soccer Scenario B Air Sample Summary Results

Sample ID	Location	Location Description	Date	PCME Structures (s/cc)	AHERA-like Total Structures (s/cc)	Sensitivity (s/cc)
CC9-L6-2CC-101004	child #2	composite sample collected during the 101004 scenarios	10/10/04	results pending	results pending	results pending
CC9-L6-3CC-101004	child #3	composite sample collected during the 101004 scenarios	10/10/04	results pending	results pending	results pending
JEP-L2-1ZB-101004	field blank	field blank	10/10/04	<0.00296	<0.00296	0.000989

PCME fibers = fibers longer than 5 microns with a width between 0.25 and 3 microns, and an aspect ratio (length to width) greater than 3:1
AHERA-like total structures = structures longer than 0.5 microns with an aspect ratio greater than 3:1 (Note this differs somewhat from the strict AHERA structure definition)
Sensitivity = the sample-specific lowest concentration of asbestos the laboratory can reliably detect
s/cc = structures per cubic centimeter

US EPA ARCHIVE DOCUMENT

Basketball and Kindergarten Playground Scenario. For the basketball and kindergarten playground scenario on October 10, 2004, the START conducted activity-based outdoor air sampling for 1 hour on a half court of one of the basketball courts at Jackson Elementary School and 1 hour in the paved portion of the kindergarten playground. The START did not perform a maintenance component of the basketball portion of the scenario. The upwind high-flow air sample pump (JEB-4FD) was operated for the entire 2 hours and serves as the upwind sample location for both halves of the scenario. The other high-flow air sample pumps were operated for 1 hour each (i.e., during each half of the scenario). The personal air sample pumps worn by sampling team members were operated for the entire 2-hour scenario.

The START conducted activity-based outdoor air sampling during the basketball portion of the scenario according to the following scripted schedule:

- For the entire hour of this half-scenario, one member of the sampling team simulated the activities of an adult/parent spectator sitting near the edge or walking around and occasionally within the area of play. This member of the sampling team would sometimes retrieve a ball that bounced out of the area of play. The intake for the air collection filter cassette worn by this sampling team member was set at a height of about 5 feet.
- For the entire hour of this half-scenario, five other members of the sampling team played basketball and conducted practice drills according to the following 10-minute activity modules:
    - Half-Court Game
    - Layups
    - Top of the Key

US EPA ARCHIVE DOCUMENT

- Foul Line Shots
- Layups
- Half-Court Game

The intakes for the air collection filter cassettes worn by these sampling team members were set at a height of about 3 feet.

The area within which the START conducted the activity is outlined and the positions of the stationary air sample pumps operating during the basketball portion of the scenario are shown as JEB-1FD, JEB-2FD/JEB-12FD, JEB-3FD, JEB-4FD, and JEB-5EFD on Figure 5-20 (Jackson Elementary School Basketball Court Activity-Based Outdoor Air Sampling Locations–Basketball and Kindergarten Playground Scenario). The position of the mobile meteorological station is shown as JEB-MS. A summary of results for the basketball and kindergarten playground scenario samples is shown in Table 5-29 (Jackson Elementary School Basketball and Kindergarten Playground Scenario Air Sample Summary Results). See Appendix B for a summary of the results from dust monitoring during this scenario.

For the kindergarten playground portion of the scenario, the START conducted activity-based outdoor air sampling according to the following scripted schedule:

- For the entire hour of this half-scenario, one member of the sampling team simulated the activities of an adult/teacher standing and walking near the other members of the sampling team. The intake for the air collection filter cassette worn by this sampling team member was set at a height of about 5 feet.

US EPA ARCHIVE DOCUMENT

Figure 5-20

US EPA ARCHIVE DOCUMENT

US EPA ARCHIVE DOCUMENT

Table 5-29
Jackson Elementary School Basketball and Kindergarten Playground Scenario B Air Sample Summary Results

Sample ID	Location	Location Description	Date	PCME Structures (s/cc)	AHERA-like Total Structures (s/cc)	Sensitivity (s/cc)
JEB-H1-1FD-101004	JEB-1FD	on basketball court (1-hour) hi-vol	10/10/04	<0.00289	<0.00289	0.000968
JEB-H1-2FD-101004	JEB-2FD	on basketball court (1-hour) hi-vol	10/10/04	<0.00295	0.00198	0.000988
JEB-H1-12FD-101004	JEB-2FD	duplicate on basketball court (1-hour) hi-vol	10/10/04	results pending	results pending	results pending
JEB-H1-3FD-101004	JEB-3FD	on basketball court (1-hour) hi-vol	10/10/04	results pending	results pending	results pending
JEB-H2-4FD-101004	JEB-4FD	upwind (of basketball court and kindergarten playground) hi-vol	10/10/04	<0.00291	0.000974	0.000974
JEB-H2-5EFD-101004	JEB-5EFD	far downwind (of basketball court) hi-vol	10/10/04	0.00196	0.00196	0.000982
JEB-H2-5WFD-101004	JEB-5WFD	far downwind (of kindergarten playground) hi-vol	10/10/04	0.000971	0.00194	0.000971
JEB-H1-6FD-101004	JEB-6FD	kindergarten playground hi-vol (1-hour)	10/10/04	0.00596	0.00993	0.000993
JEB-H1-7FD-101004	JEB-7FD	kindergarten playground hi-vol (1-hour)	10/10/04	<0.00290	0.00291	0.000970
JEB-L2-1CH-101004	JEB-1CH	child #1	10/10/04	0.00399	0.00599	0.000998
JEB-L2-2CH-101004	JEB-2CH	child #2	10/10/04	0.000992	0.00793	0.000992
JEB-L2-3CH-101004	JEB-3CH	child #3	10/10/04	0.000993	0.00695	0.000993
JEB-L2-4CH-101004	JEB-4CH	child #4	10/10/04	0.000998	0.00698	0.000998
JEB-L2-5CH-101004	JEB-5CH	child #5	10/10/04	0.00575	0.00958	0.000958

US EPA ARCHIVE DOCUMENT

Table 5-29
Jackson Elementary School Basketball and Kindergarten Playground Scenario B Air Sample Summary Results

Sample ID	Location	Location Description	Date	PCME Structures (s/cc)	AHERA-like Total Structures (s/cc)	Sensitivity (s/cc)
JEB-L2-1NA-101004	JEB-1NA	non-active adult	10/10/04	<0.00299	<0.00299	0.000999
CC9-L6-1CA-101004	adult #1	composite sample collected during the 101004 scenarios	10/10/04	results pending	results pending	results pending
CC9-L6-11CA-101004	adult #1	duplicate of adult #1 composite sample collected during the 101004 scenarios	10/10/04	results pending	results pending	results pending
CC9-L6-1CC-101004	child #1	composite sample collected during the 101004 scenarios	10/10/04	<0.00297	0.000992	0.000992
CC9-L6-2CC-101004	child #2	composite sample collected during the 101004 scenarios	10/10/04	results pending	results pending	results pending
CC9-L6-3CC-101004	child #3	composite sample collected during the 101004 scenarios	10/10/04	results pending	results pending	results pending
JEB-L2-1ZP-101004	performance evaluation	performance evaluation	10/10/04	0.0775	1.21	0.0111

PCME fibers = fibers longer than 5 microns with a width between 0.25 and 3 microns, and an aspect ratio (length to width) greater than 3:1
AHERA-like total structures = structures longer than 0.5 microns with an aspect ratio greater than 3:1 (Note this differs somewhat from the strict AHERA structure definition)
Sensitivity = the sample-specific lowest concentration of asbestos the laboratory can reliably detect
s/cc = structures per cubic centimeter

- For the entire hour of this half-scenario, five other members of the sampling team played foursquare, dodgeball, and hopscotch in groups of two and three, alternating at 10-minute intervals from one side of the playground to the other. The intakes for the air collection filter cassettes worn by these sampling team members were set at a height of about 3 feet.

The area within which the START conducted the activity is outlined and the positions of the stationary air sample pumps operating during the kindergarten playground portion of the scenario are shown as JEB-4FD, JEB-5WFD, JEB-6FD, and JEB-7FD on Figure 5-21 (Jackson Elementary School Kindergarten Playground Activity-Based Outdoor Air Sampling Locations–Basketball and Kindergarten Playground Scenario). The position of the mobile meteorological station is shown as JEB-MS. A summary of results for the basketball and kindergarten playground scenario samples is shown in Table 5-29 (Jackson Elementary School Basketball and Kindergarten Playground Scenario Air Sample Summary Results). See Appendix B for a summary of the results from dust monitoring during this scenario.

US EPA ARCHIVE DOCUMENT

5.5 SOIL SAMPLING

The START conducted soil sampling according to the *El Dorado Hills Naturally Occurring Asbestos Multimedia Exposure Assessment, El Dorado Hills, California, Soil Sampling of Community Park, Schools, and Public Areas Field Sampling Plan*. At all of the locations where the START conducted soil sampling, surface soil samples were collected. In addition, the START collected subsurface soil samples at the same surface soil sample locations at all three baseball playing fields at the Community Park (i.e., North Field, South Field, and New York Creek Field). Soil sample locations are shown on Figures 5-21 through 5-31. The soil samples are still being analyzed by a laboratory, and U.S. EPA is currently evaluating preliminary data. No results are available for release at the time of this report.

US EPA ARCHIVE DOCUMENT

Figure 5-21

US EPA ARCHIVE DOCUMENT

Figure 5-22

US EPA ARCHIVE DOCUMENT

Figure 5-23

US EPA ARCHIVE DOCUMENT

Figure 5-24

US EPA ARCHIVE DOCUMENT

Figure 5-25

US EPA ARCHIVE DOCUMENT

Figure 5-26

US EPA ARCHIVE DOCUMENT

Figure 5-27

US EPA ARCHIVE DOCUMENT

Figure 5-28

US EPA ARCHIVE DOCUMENT

Figure 5-29

US EPA ARCHIVE DOCUMENT

Figure 5-30

US EPA ARCHIVE DOCUMENT

Figure 5-31

US EPA ARCHIVE DOCUMENT

US EPA ARCHIVE DOCUMENT

6 Hazard Ranking System Factors

HRS
U.S. EPA's Hazard Ranking System

NPL
National Priorities List (also called Superfund List)

The **Hazard Ranking System** (HRS) is the principal mechanism EPA uses to place sites on the **National Priorities List** (NPL). It is a numerically-based screening system that uses information from initial, limited investigations to assess the relative potential of sites to pose a threat to human health or the environment.

The HRS uses a structured analysis approach to determining site scores. This approach assigns numerical values to factors that relate to risk based on conditions at the site. The factors are grouped into three categories:

- Likelihood that sources of contamination at a site have released or have the potential to release hazardous substances into the environment;
- Characteristics of the waste (e.g., toxicity and waste quantity); and
- People or sensitive environments (targets) affected by the release.

Four pathways can be scored under the HRS:

- Groundwater migration (drinking water);
- Surface water migration (drinking water, human food chain, sensitive environments);
- Soil exposure (resident population, nearby population, sensitive environments); and
- Air migration (population, sensitive environments).

6.1 SOURCES OF CONTAMINATION

6.1.1 Areas of Concern

In all the locations that are the subject of the El Dorado Hills Naturally Occurring Asbestos Multimedia Assessment, areas of concern are those areas where the presence of asbestos in exposed and disturbed soil may be causing releases to air. The original expectation at the outset of the project was that asbestos present in soils would be from naturally occurring sources and would pose a potential threat of exposure because of activities that disturbed it from its natural state. Several of the playing fields where START found asbestos during activity-based air sampling (e.g., baseball playing fields at the Community Park) have imported infield mix, however, so the possibility that not all of the asbestos is endemic to El Dorado Hills must be considered.

chrysotile
A regulated mineral in the serpentine group of minerals that can crystallize as asbestos. Chrysotile is also known as serpentine asbestos.

amphibole
One of the two groups of minerals (serpentine and amphibole) that can crystallize as asbestos. The regulated asbestiform minerals of this group are crocidolite, amosite, anthophyllite asbestos, tremolite asbestos, and actinolite asbestos.

6.1.2 Contaminant Types and Quantities

Types

In samples collected during activity-based air sampling, the laboratory identified **chrysotile** and **amphibole** asbestos. The following regulated (indicated with an asterisk) and non-regulated asbestiform minerals were in air samples above the laboratory levels of detection:

- actinolite*
- amosite*
- anthophyllite*
- chrysotile*
- edenite
- ferro-edenite
- richterite (one sample)
- tremolite*
- winchite

US EPA ARCHIVE DOCUMENT

Quantities

The New York Creek Nature Trail runs almost 2 miles starting from Harvard Way, traversing through the El Dorado Hills Community Park, and ending near Jackson Elementary School.

The three baseball playing fields at the Community Park have "skinned" infields, which means that there is no grass in their infields. There is grass covering the outfield areas, which are also used as soccer playing fields. The baseball bases are set at 60 feet apart. Imported "infield mix," which is composed of 50% topsoil and 50% crushed lava rock, is present within the infield.

The Dirt Embankment is about 1,200 feet along the downslope edge. The width of the Dirt Embankment varies, but is estimated from aerial photographs to be about 75 feet wide at its widest point.

The Silva Valley Elementary School baseball playing field has a grass infield. When the field is maintained, the outfield and the area inside the baselines is grass (except for the pitcher's mound), and the rest of the infield is exposed dirt (e.g., see Figure 5-4: Silva Valley Elementary School Baseball Playing Field Activity-Based Outdoor Air Sampling Locations–Maintenance Scenario). According to school district personnel, imported "infield mix" would typically be used in the dirt areas of the infield including on the basepaths and pitcher's mound. The baseball bases are set at 60 feet apart.

US EPA ARCHIVE DOCUMENT

6.2 GROUNDWATER MIGRATION PATHWAY

In determining a score for the groundwater migration pathway, the HRS evaluates: 1) the likelihood that sources at a site actually have released, or potentially could release, hazardous substances to groundwater; 2) the characteristics of the hazardous substances that are available for a release (i.e., toxicity, mobility, and quantity); and 3) the people (targets) who actually have been, or potentially could be, impacted by the release. For the targets component of the evaluation, the HRS focuses on the number of people who regularly obtain their drinking water from wells that are located within 4 miles of the site. The HRS emphasizes drinking water usage over other uses of groundwater (e.g., food crop irrigation and livestock watering), because, as a screening tool, it is designed to give the greatest weight to the most direct and extensively studied exposure routes.

According to the *Draft Water Resources Development and Management Plan* for El Dorado County, usable groundwater is limited in the western slope of the county. Groundwater quality in this region is said to be satisfactory but marginal. There are some wells, typically in the range of 100 to 200 feet below ground surface, but the amount of water they provide to serve the community's drinking water needs compared to surface water sources is minimal.

6.3 SURFACE WATER MIGRATION PATHWAY

In determining the score for the surface water pathway, the HRS evaluates: 1) the likelihood that sources at a site actually have released, or potentially could release, hazardous substances to surface water (e.g., streams, rivers, lakes, and oceans); 2) the characteristics of the hazardous substances that are available for a release (i.e., toxicity, persistence, bioaccumulation potential, and

US EPA ARCHIVE DOCUMENT

quantity); and 3) the people or sensitive environments (targets) who actually have been, or potentially could be, impacted by the release. For the targets component of the evaluation, the HRS focuses on drinking water intakes, fisheries, and sensitive environments associated with surface water bodies within 15 miles downstream of the site.

The El Dorado Irrigation District is the primary water purveyor in El Dorado Hills. The water supply comes from a variety of sources, essentially all from surface water sources. The largest water supply source for El Dorado Hills Irrigation District is Jenkinson Lake (Sly Park Reservoir and Dam). In addition, Folsom Lake, the South Fork of the American River (at Kyburz), the North Fork of the Cosumnes River, and Clear Creek (Crawford Ditch) provide significant supply to the system. Of these sources, all except Folsom Lake are geographically upslope of the area of concern. The New York Creek, which is an ephemeral stream, flows through the Community Park along the New York Creek Nature Trail, past Jackson Elementary School to Folsom Lake, with the point of entry just over 3 miles from the northern boundary of the Community Park.

Folsom Lake State Recreation Area is an 18,000-acre lake and recreation area offering opportunities for angling, hiking, biking, running, camping, picnicking, horseback riding, water-skiing and boating. The lake has trout, catfish, big and small mouth bass and perch.

US EPA ARCHIVE DOCUMENT

6.4 SOIL EXPOSURE PATHWAY

In determining the score for the soil exposure pathway, the HRS evaluates: 1) the likelihood that there is surficial contamination associated with the site (e.g., contaminated soil that is not covered by pavement or at least 2 feet of clean soil); 2) the characteristics of the hazardous substances in the surficial contamination (i.e., toxicity and quantity); and 3) the people or sensitive environments (targets) who actually have been, or potentially could be, exposed to the contamination. For the targets component of the evaluation, the HRS focuses on populations that are regularly and currently present on or within 200 feet of surficial contamination. The four populations that receive the most weight are residents, students, daycare attendees, and terrestrial sensitive environments.

6.4.1 Contamination

The contaminant of concern is asbestos. The START collected soil samples from areas of concern within 2 feet of ground surface. The soil samples are still being analyzed by a laboratory, and U.S. EPA is currently evaluating preliminary data. The results are not available for release at the time of this report.

6.4.2 Population

According to the State Department of Finance, as of January 2005 there were about 31,000 residents in El Dorado Hills. The population of El Dorado Hills has grown rapidly in the last few decades. The residential area of El Dorado Hills is about 28 square miles.

There are about 800 students and 50 to 55 teachers and staff members at Rolling Hills Middle School. At Silva Valley Elementary School there are about 650 students and 49 teachers and staff members. Jackson Elementary School is reducing its size, and

US EPA ARCHIVE DOCUMENT

next fall there are expected to be about 470 students and 35 teachers and staff members. According to the California Department of Education, at the nearby Oak Ridge High School, there were reportedly 1,829 students and 87 staff members for the 2003-2004 school year.

There are 26 full-time and about 8 part-time year-round staff members at the CSD offices at the Community Park. There are seasonal changes in part-time staff (e.g., life guards) at the Community Park. About 35 to 40 children attend day camp at the Community Park during the school year, and about 150 attend during the summer. The Community Park and the schools have staff who maintain the play areas and the Dirt Embankment. The CSD is responsible for maintaining the New York Creek Nature Trail as well.

6.4.3 Sensitive Environments

Based on review of biological resources maps included in the draft *Environmental Impact Report for the El Dorado County General Plan* as well as the draft *Water Resources Development and Management Plan* for El Dorado County, there do not appear to be terrestrial or aquatic sensitive environments or special status species documented within 4 miles of the areas that are the subject of the El Dorado Hills Naturally Occurring Asbestos Multimedia Assessment.

6.5 AIR MIGRATION PATHWAY

In determining the score for the air migration pathway, the HRS evaluates: 1) the likelihood that sources at a site actually have released, or potentially could release, hazardous substances to ambient outdoor air; 2) the characteristics of the hazardous substances that are available for a release (i.e., toxicity, mobility, and

US EPA ARCHIVE DOCUMENT

quantity); and 3) the people or sensitive environments (targets) who actually have been, or potentially could be, impacted by the release. For the targets component of the evaluation, the HRS focuses on regularly occupied residences, schools, and workplaces within 4 miles of the site. Transient populations, such as customers and travelers passing through the area, are not counted.

6.5.1 Release of Asbestos to Air

The START collected activity-based air samples containing elevated levels of asbestos (i.e., relative to ambient reference air samples that were collected during the same time periods). Table 6-1 (Summary of Sample Results) shows a comparison of the results from the personal air monitors with results from ambient reference air samples collected simultaneously. The table shows 1) the ratio of the average personal asbestos exposure measurement to the average ambient air asbestos concentration measured simultaneously in the same general area, 2) the average personal asbestos exposure concentration as measured by the personal samplers during the simulated activity, and 3) the average concentration of asbestos measured in nearby ambient air at the same time the simulated activity was taking place. For most scenarios this value is the average asbestos concentration from five fixed air sample pumps collecting nearby ambient air on the same day each activity was occurring. Numeric ratios are presented only for those scenarios where the elevated exposure was determined to be significant by the Z-test statistical procedure specified in the **Asbestos Hazardous Emergency Response Act** (AHERA) regulations (40 CFR Part 763; October 30, 1987) covering asbestos in schools. The results showed that personal exposure levels of asbestos were significantly higher during most sports and play activities as compared to nearby asbestos air samples taken outside the areas of activity.

AHERA
Asbestos Hazardous Emergency Response Act

US EPA ARCHIVE DOCUMENT

US EPA ARCHIVE DOCUMENT

Table 6-1

Summary of Air Sample Results

U.S. EPA Activity-Based Asbestos Exposure Sampling - Community Park, Silva Valley School, Rolling Hills School, & Jackson School, El Dorado Hills, October 2004

Asbestos concentration used for cancer risk assessment. (→ Long Fibers (PCME))

Location & Activity Scenario	Long Fibers (PCME) [1, 4]			Total Structures (AHERA) [2, 4]			Comments
	Ratio: Personal Exposure to Reference [3]	Average of Personal Exposure (f/cc) [6]	Reference Concentration (f/cc) [6]	Ratio: Personal Exposure to Reference [3]	Average of Personal Exposure (s/cc) [7]	Reference Concentration (s/cc) [7]	
**New York Trail**, ► *Child Biking Scenario*	43	0.0336	0.0008	23	0.0564	0.0024	PCME & short fibers ~ all amphiboles.
**New York Trail**, ► *Adult Jogging Scenario-B*	39	0.0212	0.0005	28	0.0439	0.0016	PCME & short fibers ~ all amphiboles.
**North Field Baseball Diamond, Community Park** ► *Child Baseball Game*	22	0.0171	0.0008	21	0.0513	0.0024	PCME mostly amphiboles, including actinolite, amosite & anthrophylite.
**South Field Baseball Diamond, Community Park** ► *Child Baseball Game A*	22	0.0168	0.0008	217	0.5307	0.0024	PCME mostly amphiboles; short fibers mostly chrysotile.
**North/South Soccer Field, Community Park** ► *Child Soccer Game*	16	0.0087	0.0005	11	0.0175	0.0016	PCME ~ all amphiboles; short fibers mostly amphiboles.
**New York Trail**, ► *Adult Jogging Scenario-A*	12	0.0197	0.0017	10	0.0347	0.0036	PCME & short fibers ~ all amphiboles.
**Community Park** Baseball * ***Adult Observer Exposure***	11	0.0114	0.0010	21	0.0550	0.0026	PCME mostly amphiboles; short fibers mostly chrysotile.
**South Field Baseball Diamond, Community Park** ► *Pooled Child Baseball Games*	10	0.0118	0.0012	95	0.2823	0.0030	PCME mostly amphiboles; short fibers mostly chrysotile.
**Toddler Playground, Community Park,** ► *Typical Child Play Scenario*	10	0.0067	0.0007	60	0.0816	0.0014	PCME mostly amphiboles, some chrysotile, edenite & amosite.
**Silva Valley Baseball Diamond**, **Silva Valley Elem School** ► *Child Baseball Game A*	9	0.0062	0.0006	7	0.0144	0.0021	Wet conditions. PCME & short fibers ~ all amphiboles.
**Silva Valley Baseball Diamond**, **Silva Valley Elem School** ► *Child Baseball Game B*	7	0.0032	0.0005	5	0.0066	0.0012	Wet conditions. PCME & short fibers ~ all amphiboles.
**South Field Baseball Diamond, Community Park** (Game A) ► *Impact on Toddler Playground*	6	0.0047	0.0008	7	0.0170	0.0024	PCME ~ all amphiboles; short fibers mixed amphibole & chrysotile
**Toddler Playground, Community Park,** ► *Aggressive Child Play Scenario*	6	0.0040	0.0007	8	0.0110	0.0014	PCME & short fibers mostly amphiboles; some edenite & amosite observed.
**New York Trail** ► *Adult Observers*	5	0.0053	0.0010	5	0.0123	0.0026	PCME & short fibers ~ all amphiboles.
**South Field Diamond, Community Park** ► *Child Baseball Game B*	5	0.0089	0.0017	37	0.1333	0.0036	PCME mostly amphiboles; short fibers mostly chrysotile; wincherite observed.
**Silva Valley Baseball Diamond**, **Silva Valley Elem School** ► *Baseball & Maintenance*	4	0.0024	0.0006	NS	0.0041	0.0021	Wet conditions. PCME & short fibers ~ all amphiboles.
**Rolling Hills Basketball Court, Rolling Hills Middle School** ► *Child Basketball Game*	4	0.0017	0.0005	3	0.0043	0.0012	PCME & short fibers mostly amphiboles.
**South Field Baseball Diamond, Community Park** (Game C) ► *Impact on Toddler Playground*	3	0.0056	0.0017	15	0.0542	0.0036	PCME ~ all amphiboles; short fibers mostly chrysotile

US EPA ARCHIVE DOCUMENT

Table 6-1

Summary of Air Sample Results

U.S. EPA Activity-Based Asbestos Exposure Sampling - Community Park, Silva Valley School, Rolling Hills School, & Jackson School, El Dorado Hills, October 2004

Asbestos concentration used for cancer risk assessment. → Long Fibers (PCME)

Location & Activity Scenario	*Long Fibers (PCME)* [1, 4]			Total Structures (AHERA) [2, 4]			Comments
	Ratio: Personal Exposure to Reference [3]	*Average of Personal Exposure* (f/cc) [6]	*Reference Concentration* (f/cc) [6]	*Ratio: Personal Exposure to Reference* [3]	*Average of Personal Exposure* (s/cc) [7]	*Reference Concentration* (s/cc) [7]	
**North/South Soccer Field, Community Park** ► *Impact on Toddler Playground*	***3***	***0.0016***	***0.0005***	***3***	***0.0044***	***0.0016***	PCME mostly amphiboles; short fibers mixed amphibole & chrysotile.
**North Field Diamond, Community Park** ► *Impact on Tot Lot*	***3***	***0.0021***	***0.0008***	***NS***	***0.0035***	***0.0024***	PCME & short fibers ~ all amphiboles.
**Rolling Hills Soccer Field, Rolling Hills Middle School** ► *Child Soccer Game*	***3***	***0.0013***	***0.0005***	***NS***	***0.0017***	***0.0012***	PCME & short fibers mostly amphiboles
**Jackson Elem School** Basketball Game	***3***	***0.0026***	***0.0010***	***3***	***0.0075***	***0.0022***	PCME ~ all amphiboles; short fibers mostly amphiboles.
**New York Baseball Diamond, Community Park** ► *Impact on Toddler Playground*	***2***	***0.0013***	***0.0005***	***NS***	***0.0011***	***0.0016***	PCME ~ all amphiboles; short fibers mixed amphibole & chrysotile
**South Field Baseball Diamond, Community Park** (Game B) ► *Impact on Toddler Playground*	***NS***	***0.0028***	***0.0017***	***4***	***0.0159***	***0.0036***	PCME ~ all amphiboles; short fibers mixed amphibole & chrysotile
**New York Trail**, * ***South Perimeter Sampling***	***NS***	***0.0009***	***0.0005***	***3***	***0.0037***	***0.0012***	PCME ~ all amphiboles; short fibers mostly amphiboles.
**New York Trail**, * ***North Perimeter Sampling***	***NS***	***0.0004***	***0.0005***	***NS***	***0.0006***	***0.0011***	PCME & short fibers ~ all amphiboles.
**Rolling Hills School** Basketball & Soccer * ***Adult Observer Exposure***	***NS***	***0.0012***	***0.0005***	***NS***	***0.0033***	***0.0012***	PCME mostly amphiboles; short fibers mixed amphibole & chrysotile.
**Overall mean - Northern reference samples.**			***0.0009***			***0.0021***	
**Overall mean - Southern reference samples.**			***0.0008***			***0.0021***	
			(f/cc)			***(s/cc)***	

Notes:
Statistical significance of elevated exposure determined by Z-test (AHERA) - "NS" = not significant
[1] PCME fibers = fibers longer than 5 microns with a width between 0.25 and 3 microns, and an aspect ratio (length to width) greater than 3:1
[2] "AHERA structures" = structures longer than 0.5 microns with an aspect ratio greater than 3:1 (Note this differs somewhat from the strict AHERA fiber definition.)
[3] Ratio = average asbestos concentration from personal samples collected during simulated activity divided by the average asbestos concentration from "reference" samples collected
[4] Fiber counts are from direct analysis of PCM filters using ISO 10312 procedure.
[5] Reference Concentration refers to the average asbestos concentration measured on the same day by 5 stationary monitoring stations.
These reference stations were located in the general study area, but outside of the zone of influence by the activity.
[6] f/cc = fibers per cubic centimeter
[7] s/cc = structures per cubic centimeter

6.5.2 Population

See discussion of population for the soil exposure pathway in Section 6.4.2.

6.5.3 Sensitive Environments

See discussion of sensitive environments for the soil exposure pathway in Section 6.4.3.

US EPA ARCHIVE DOCUMENT

7 Emergency Response Considerations

The National Contingency Plan (40 CFR 300.415 (b) (2)), authorizes the U.S. EPA to consider emergency response actions at sites that pose an imminent threat to human health or the environment. U.S. EPA is continuing to evaluate whether asbestos in disturbed soils at the locations that are the subject of this PA/SI poses an imminent threat requiring mitigative measures.

The U.S. EPA has met with local, state, and federal agencies; the El Dorado Hills Community Services District; and the schools to discuss preliminary results from the sampling. The U.S. EPA has initiated efforts to convene a science advisory panel of health and asbestos experts to evaluate the data and answer a series of questions about the significance of the exposures.

US EPA ARCHIVE DOCUMENT

8 Summary

To assess the potential for exposure from naturally occurring asbestos present in soils that have been disturbed, the U.S. Environmental Protection Agency (U.S. EPA), Region IX, tasked the Ecology and Environment, Inc., (E & E) Superfund Technical Assessment and Response Team (START) to conduct a multimedia assessment of community areas and schools in El Dorado Hills in El Dorado County, California.

The START collected activity-based air samples, reference air samples, and soil samples at and around the following areas in El Dorado Hills:

- The El Dorado Hills Community Park, including several play areas and the New York Creek Nature Trail;
- Silva Valley Elementary School;
- Jackson Elementary School;
- Rolling Hills Middle School, including the dirt embankment inside the school's eastern boundary (Dirt Embankment); and
- An unpaved lot used for parking on public property adjacent to and in front of Rolling Hills Middle School (Dirt Parking Area).

US EPA ARCHIVE DOCUMENT

The most significant HRS factors associated with the El Dorado Hills Naturally Occurring Asbestos Multimedia Assessment site are as follows:

- Asbestos is known to be naturally occurring in soils in El Dorado Hills.
- Activity-based sampling through disturbing soils at schools and recreation areas within El Dorado Hills shows the presence of asbestos at elevated levels in air at breathing heights for children and adults.
- In addition to asbestos that occurs naturally in soil, asbestos may be present in non-native soils used as "infield mix" on some or all of the playing fields that were studied.

US EPA ARCHIVE DOCUMENT

A

Meteorological Data

US EPA ARCHIVE DOCUMENT

B

Dust Monitoring Results

US EPA ARCHIVE DOCUMENT

US EPA ARCHIVE DOCUMENT

C References

Appendix C
References

Boike, Ray, Buckeye Union School District, and Karen Ladd, Ecology and Environment, Inc. START, telephone conversation, April 28, 2005.

Buckeye Union School District website, buckeye.k12.ca.us.

California Air Resources Board, *Sampling for Airborne Naturally Occurring Asbestos at Oak Ridge High School June 2003*, November 6, 2003.

California Air Resources Board website, http://www.arb.ca.gov/toxics/asbestos/silvavalley.htm *Measured Ambient Asbestos Concentrations in El Dorado County, Asbestos Monitoring in Silva Valley*, sampling period April 21, 1999, through April 29, 1999, updated September 15, 1999.

Ecology & Environment, Inc. START, *El Dorado Hills Naturally Occurring Asbestos Multimedia Exposure Assessment, El Dorado Hills, California, Activity-Based Outdoor Air Sampling of Community Park and Schools Field Sampling Plan*, September 2004.

Ecology & Environment, Inc. START, *El Dorado Hills Naturally Occurring Asbestos Multimedia Exposure Assessment, El Dorado Hills, California, Activity-Based Outdoor Air Sampling of Community Park Children's Playground Field Sampling Plan*, September 2004.

Ecology & Environment, Inc. START, *El Dorado Hills Naturally Occurring Asbestos Multimedia Exposure Assessment, El Dorado Hills, California, Fixed Ambient Outdoor Reference Air Sampling Field Sampling Plan*, September 2004.

Ecology & Environment, Inc. START, *El Dorado Hills Naturally Occurring Asbestos, Multimedia Exposure Assessment, El Dorado Hills, California, Quality Assurance Project Plan*, September 2004.

US EPA ARCHIVE DOCUMENT

Appendix C, References (Cont.)

Ecology & Environment, Inc. START, *El Dorado Hills Naturally Occurring Asbestos Multimedia Exposure Assessment, El Dorado Hills, California, Soil Sampling of Community Park, Schools, and Public Areas Field Sampling Plan*, September 2004.

EDAW, *El Dorado County General Plan Draft Environmental Impact Report*, State Clearinghouse No. 2001082030, May 2003.

El Dorado County Water Agency, *Draft Water Resources Development and Management Plan*, June 2003.

El Dorado Hills Chamber of Commerce website, http://www.eldoradohillschamber.com.

El Dorado Hills Community Services District website, http://www.edhcsd.org.

El Dorado Union High School District website, www.eduhsd.k12.ca.us.

Lowery, Wayne, El Dorado Hills Community Services District, and Karen Ladd, Ecology and Environment, Inc. START, telephone conversation, April 28, 2005.

MACTEC Federal Programs, *El Dorado Union High School District Oak Ridge High School Naturally Occurring Asbestos (NOA) Operations and Maintenance (O&M) Plan*, December 2003.

Rescue Union School District website, rescue.k12.ca.us.

State of California Asbestos Task Force, *Findings and Recommendations on Naturally-Occurring Asbestos to El Dorado County*, March 11, 1999.

State of California Department of Conservation, Division of Mines and Geology, *Areas More Likely to Contain Natural Occurrences of Asbestos in Western El Dorado County, California*, Open-File Report 2000-002, 2000.

U.S. Census Bureau website, factfinder.census.gov.

US EPA ARCHIVE DOCUMENT

Appendix C, References (Cont.)

U.S. Department of Health and Human Services, Agency for Toxic Substances and Disease Registry, *Chemical-Specific Health Consultation: Tremolite Asbestos and Other Related Types of Asbestos*, September 2001.

Wolcott, Doug, Rescue Union School District, and Karen Ladd, Ecology and Environment, Inc. START, telephone conversation, April 27, 2005.

US EPA ARCHIVE DOCUMENT
